# Radiomic feature stability across 4D respiratory phases and its impact on lung tumor prognosis prediction

**DOI:** 10.1371/journal.pone.0216480

**Published:** 2019-05-07

**Authors:** Qian Du, Michael Baine, Kyle Bavitz, Josiah McAllister, Xiaoying Liang, Hongfeng Yu, Jeffrey Ryckman, Lina Yu, Hengle Jiang, Sumin Zhou, Chi Zhang, Dandan Zheng

**Affiliations:** 1 Biological Sciences, University of Nebraska Lincoln, Lincoln, NE, United States of America; 2 Radiation Oncology, University of Nebraska Medical Center, Omaha, NE, United States of America; 3 University of Florida Proton Therapy Institute, Jacksonville, FL, United States of America; 4 Computer Science, University of Nebraska Lincoln, Lincoln, NE, United States of America; Taichung Veterans General Hospital, TAIWAN

## Abstract

Radiomic analysis has recently demonstrated versatile uses in improving diagnostic and prognostic prediction accuracy for lung cancer. However, since lung tumors are subject to substantial motion due to respiration, the stability of radiomic features over the respiratory cycle of the patient needs to be investigated to better evaluate the robustness of the inter-patient feature variability for clinical applications, and its impact in such applications needs to be assessed. A full panel of 841 radiomic features, including tumor intensity, shape, texture, and wavelet features, were extracted from individual phases of a four-dimensional (4D) computed tomography on 20 early-stage non-small-cell lung cancer (NSCLC) patients. The stability of each radiomic feature was assessed across different phase images of the same patient using the coefficient of variation (COV). The relationship between individual COVs and tumor motion magnitude was inspected. Population COVs, the mean COVs of all 20 patients, were used to evaluate feature motion stability and categorize the radiomic features into 4 different groups. The two extremes, the Very Small group (COV≤5%) and the Large group (COV>20%), each accounted for about a quarter of the features. Shape features were the most stable, with COV≤10% for all features. A clinical study was subsequently conducted using 140 early-stage NSCLC patients. Radiomic features were employed to predict the overall survival with a 500-round bootstrapping. Identical multiple regression model development process was applied, and the model performance was compared between models with and without a feature pre-selection step based on 4D COV to pre-exclude unstable features. Among the systematically tested cutoff values, feature pre-selection with 4D COV≤5% achieved the optimal model performance. The resulting 3-feature radiomic model significantly outperformed its counterpart with no 4D COV pre-selection, with P = 2.16x10^-27^ in the one-tailed t-test comparing the prediction performances of the two models.

## Introduction

A current frontier of medical imaging research and clinical utilization is radiomics[1–4]. Using high-throughput quantitative radiomic features calculated from medical images obtained in routine patient care, predictive models are made in combination with conventional clinical data to increase diagnostic, prognostic, and predictive power. The greatest success of this new approach has been reported for lung cancer[3,5–10]. In a large body of literature, radiomic features have been found to be prognostic for various clinical outcomes and tumor biology, such as distant metastasis[5], pathological response[6], local recurrence[7], responsiveness to chemoradiotherapy[8], disease-free survival[9], and radiation pneumonitis[9]. Through this data, radiomic signatures prove to be capable of providing more accurate prognostication than the traditional staging system and hence more personalized care for the lung cancer patients.

At the same time, respiratory motion also poses a significant challenge to the imaging of lung tumors[11]. Motion can change the intensity within and distort the shape of the tumor in a regular free-breathing CT[12]. To mitigate motion artifacts, four-dimensional computed tomography (4D CT) has been developed to provide images of individual phases of a breathing cycle[13,14]. In a 4D CT, a long CT scan spanning an entire breathing cycle at each imaged anatomical position is temporally correlated to individual phases (usually 8 or 10) of the breathing cycle to “freeze” the motion.

However, among the existing lung cancer radiomic research, most studies did not take the lung tumor motion into consideration. In these studies, radiomic features were extracted from the free-breathing CT and this could obviously introduce uncertainty into the clinical studies. This important aspect only recently began to be evaluated by the research community. One study compared radiomic features between the free-breathing CT and the average-intensity-projection (AIP) CT in their prognostic performance for distant metastasis and locoregional recurrence[15]. Their results suggested that the AIP CT performed better than its free-breathing counterpart. Another study used different phases of the 4D CT to study the robustness of radiomic features for lung and esophagus cancers[16]. They found that this method could select robust features somewhat different from using the conventional selection method with the test-retest CT scans. Despite these efforts, the literature on the stability of radiomic features over respiration is still scarce. The relationship between the feature stability and the respiratory motion magnitude has not been studied, and the impact of such stability on clinical prediction has not been explicitly explored.

The goal of this study, therefore, is to investigate the stability of different radiomic features over individual breathing phases of the 4D CT for early-stage non-small cell lung cancer (NSCLC) patients, study its relationship with the tumor motion magnitude, and explore its impact on prognosis prediction.

## Methods and materials

### Patient selection, image acquisition, and tumor segmentation

Under the approval of the University of Nebraska Medical Center institutional review board, this retrospective study was conducted on consecutive early stage NSCLC ≤5 cm that received stereotactic body radiotherapy (SBRT) between 2006 and 2015 at the University of Nebraska Medical Center. The 4D feature stability analysis was conducted on 20 patients, and the clinical study to assess its impact was conducted on 140 patients including the 20 patients used for the 4D study. The IRB has approved a waiver of the informed consent for this retrospective medical record study. After all relevant data were retrieved from the medical records, the patients were anonymized before analysis.

As part of the radiotherapy treatment planning simulation, each patient had undergone a 4D CT scan and a free-breathing CT scan of the thorax. The scans were acquired using the same protocol for all patients in 2 mm axial slice thickness with a Sensation Open CT simulator (Siemens, Erlangen, Germany). Either the Anzai belt system (Anzai Medical Systems, Tokyo, Japan) or the Real-time Position Management system (Varian Medical Systems, Palo Alto, CA) was used as the respiratory surrogate for the 4D CT scans. Each 4D CT was consisted of 8 phases, corresponding to 0% inhalation, 25% inhalation, 50% inhalation, 75% inhalation, 100% inhalation, 75% exhalation, 50% exhalation, and 25% exhalation, respectively. For the 4D stability study, the lung tumor was manually segmented on each phase of the 4D CT by a single investigator using the iPlan software (Brainlab AG, Feldkirchen, Germany) and applying a consistent lung window/level setting. After being reviewed by a radiation oncologist, the tumor segmentation was exported together with the corresponding image set from which the tumor was segmented. For the clinical study of 140 patients, the tumor was segmented similarly, but only on the free-breathing CT, and the segmentation was done by the attending radiation oncologists of the patient, following a departmental protocol.

### Radiomic feature extraction

From each segmented tumor, 841 radiomic features were extracted using the radiomics module on 3D Slicer 4.9[17] and visualized using an interactive visualization platform[18]. A resampled 3x3x3 mm^3^ voxel size and a bin width of 25 were used for feature extraction. The features are defined in compliance with feature definitions as described by the Imaging Biomarker Standardization Initiative (IBSI)[19] and can be divided into original features (105 features) and wavelet features (736 features). The original features can be subdivided into 7 classes, including 13 Shape features, 18 First Order statistical features, 23 Gray Level Co-occurrence Matrix (GLCM) features, 14 Gray Level Dependence Matrix (GLDM) features, 16 Gray Level Run Length Matrix (GLRLM) features, 16 Gray Level Size Zone Matrix (GLSZM) features, and 5 Neighboring Gray Tone Difference Matrix (NGTDM) features. The wavelet features included all except Shape features calculated on the filtered images with all 8 combinations of applying either a High or a Low pass filter in each of the three dimensions.

### 4D stability and motion analysis

To analyze the 4D stability of radiomic features across respiratory phases, the inter-phase variability was characterized for each patient using the coefficient of variation (COV) as defined in Eq (1):

$$COV=ABS\left(\frac{SD}{mean}\right)*100\%$$ (1)

where SD and mean are the standard deviation and the mean value of a feature over the different respiratory phases of a patient, respectively, and ABS denotes getting the absolute value. This COV calculation followed existing studies in the literature investigating feature stability against different image reconstructions[20], and in our study, they were calculated across all 8 respiratory phases of the 4D CT. As the COV was calculated for individual patients, the variability consisted solely of the variation due to the change in the intra-patient respiration (and possible noise from repeated scans) without the interference of the inter-patient variability.

The population mean 4D COV for each feature was then calculated over all 20 patients, based on which the features were grouped into the following four 4D stability categories similar to the reference[20]: Very Small (COV≤5%), Small (5%<COV≤10%), Intermediate (10%<COV≤20%), and Large (COV>20%).

For each patient, the tumor centroid on each phase of the 4D CT was extracted to calculate the tumor motion magnitude, defined as the centroid maximum three-dimension excursion among the 8 breathing phases. Individual patient’s feature variability, or individual COV, was studied against the tumor motion magnitude of the patient.

### The clinical study

To investigate the impact of 4D stability on prognostic prediction using radiomic features, we conducted a clinical study to assess whether applying the feature 4D stability information learned from the 4D analysis as an additional feature selection step would improve prediction model performance on free-breathing CT. Free-breathing CT was chosen as it is most prevalently used in lung radiomic studies due to the ubiquitous utilization of free-breathing CT scans in routine lung cancer patient care. Clinical data were retrospectively collected for 140 consecutive patients treated with SBRT as the sole therapy for primary NSCLC ≤5 cm between 2006 and 2015 at the University of Nebraska Medical Center. The patient, treatment and tumor characteristics are reported in Table 1. Collected patient data included radiomic features from the free-breathing CT, patient, disease, and treatment characteristics, along with parameters related to overall survival or last follow-up. Data analysis, described with more details in the following sections, was performed by using R (version 3.3.2)[21].

**Table 1:** Patient, tumor, and treatment characteristics and clinical outcomes.

	Total (n = 140)	Median (range) or Number of patients (percentage)
**Patient characteristics**		
Age		73 (40–94)
Gender	Female	72 (51%)
	Male	68 (49%)
Ethnicity	African-American	15 (11%)
	Caucasian	124 (89%)
	Others	1 (1%)
Pack-years		40 (0–180)
ECOG performance status	0	40 (29%)
	1	67 (48%)
	2	28 (20%)
	3	5 (4%)
Histology	Adenocarcinoma	61 (44%)
	Squamous cell carcinoma	46 (33%)
	NSCLC not otherwise specified	33 (24%)
Overall stage	Stage 1	140 (100%)
Tumor stage	T1	122 (87%)
	T2	18 (13%)
Tumor location	Right upper lobe	45 (32%)
	Right middle lobe	5 (4%)
	Right lower lobe	31 (22%)
	Left upper lobe	37 (26%)
	Left lower lobe	21 (15%)
	Chestwall	1 (1%)
**Treatment characteristics**		
SBRT prescription dose and fractionation	60 Gy in 5 fractions	2 (1%)
	50 Gy in 5 fractions	120 (86%)
	48 Gy in 4 fractions	18 (13%)
**Clinical outcomes**		
Follow-up time (months)		22.1 (0.5–121.3)
Overall survival at last follow-up	No	57 (41%)
	Yes	83 (59%)
Time to death (months)		15.6 (0.6–67.1)

#### Unsupervised clustering

A heat map was plotted to investigate the radiomic feature patterns as well as their relationship with clinical parameters including Eastern Cooperative Oncology Group (ECOG) performance status, gender, race, pack-year, histology, and tumor location. All radiomic features were classified into the Stable group (COV≤5%) or the Unstable group (COV>5%). For Stable features, a hierarchical clustering was conducted with average-linkage clustering based on the Euclidean distance for both features and patients. For Unstable features, the same clustering method was performed for features only and the order of patients was kept the same as the patient clustering based on the Stable features. The associations between patient clusters and the clinical parameters were tested using a non-parametric test “Kruskal-Wallis rank sum test” for pack-year, and using a χ^2^ test of independence for the other parameters.

#### Feature dimension reduction and multiple regression modeling for overall survival

The overall survival time from SBRT radiotherapy simulation (i.e. CT acquisition) was used as the clinical endpoint in this clinical study to evaluate the impact of radiomic feature stability across 4D respiratory phases. A univariate Cox proportional hazard model was first conducted on each feature for overall survival. The robustness of the fit was determined by the P-value and false discovery rate (FDR) adjusted Q-value of the regression. To improve the robustness of feature ranking, a 500-round bootstrapping procedure was performed from which the predictive value of each feature was determined by the median χ^2^ of the logrank test between the predicted low- and high-risk groups based on the median prediction score (A risk score was calculated for each patient based on the feature, and the patient was assigned into the low- or high-risk group depending on whether his or her risk score was lower or higher than the median score of the whole cohort). Subsequently, to study the impact of 4D stability, multiple regression modeling was performed, also with a 500-round bootstrapping procedure, that compared models with 4D stability feature pre-selection (using systematically varying 4D COV cutoffs from 5% to 25%) and those without. Because the purpose of the clinical study was to assess the impact of 4D stability of the radiomic features on the prediction performance, the multiple regression models considered only the radiomic features and not the clinical variables. For each feature pool with or without 4D pre-selection, a radiomic multiple Cox regression model was developed using feature selection based on the univariate predictive value χ^2^ ranks with applying a clustering step to exclude the redundant features having R>0.75 correlations with the selected features. A 500-round bootstrap sampling was also performed for the multiple Cox regression. To investigate the impact of feature pre-selection, the pre-selected feature pools applied systematically changing 4D COV cutoffs from 5% to 25% and univariate regression P-value cutoffs from 0.005 to 0.05 and FDR adjusted Q-value cutoffs from 0.04 to 0.09. Model performance with and without 4D stability pre-selection was compared by performing a one-tailed t-test between the set of the logarithm of χ^2^ values in the bootstrapping rounds obtained from the pool of features with the 4D stability pre-selection and its counterpart without. As in the univariate regression evaluation, a risk score was calculated for each patient based on the multiple regression model and the patient was assigned into the low- or high-risk group. A logrank test was conducted between the predicted low- and high-risk groups from which the χ^2^ value was calculated.

## Results

### Stability analysis based on population mean COV

**Fig 1:**
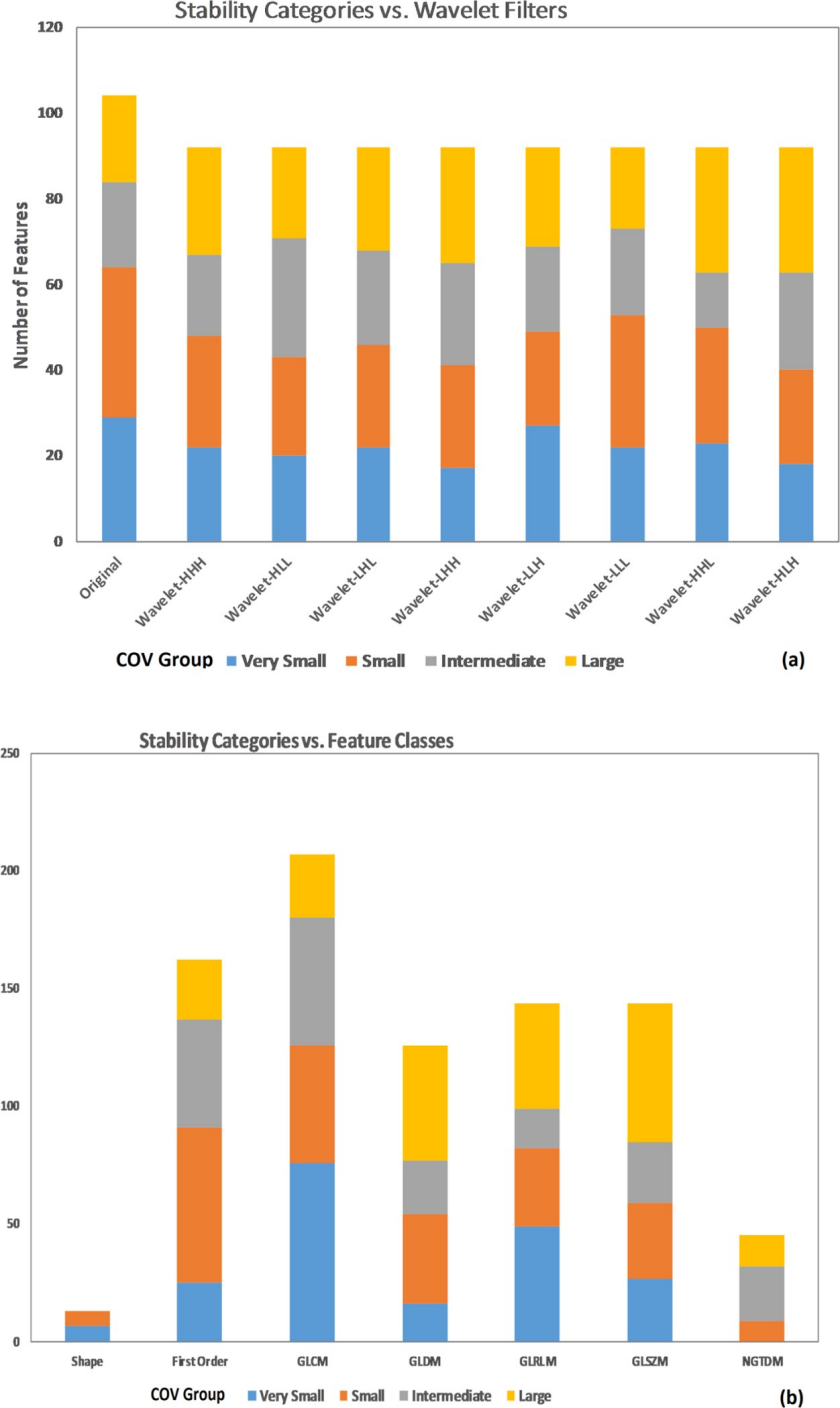
Radiomic feaure 4D stability COV grouping. (a) The COV categories comparing the original features vs. derived features applying different wavelet filters. (b) The COV categories comparing different feature classes.

**Fig 2:**
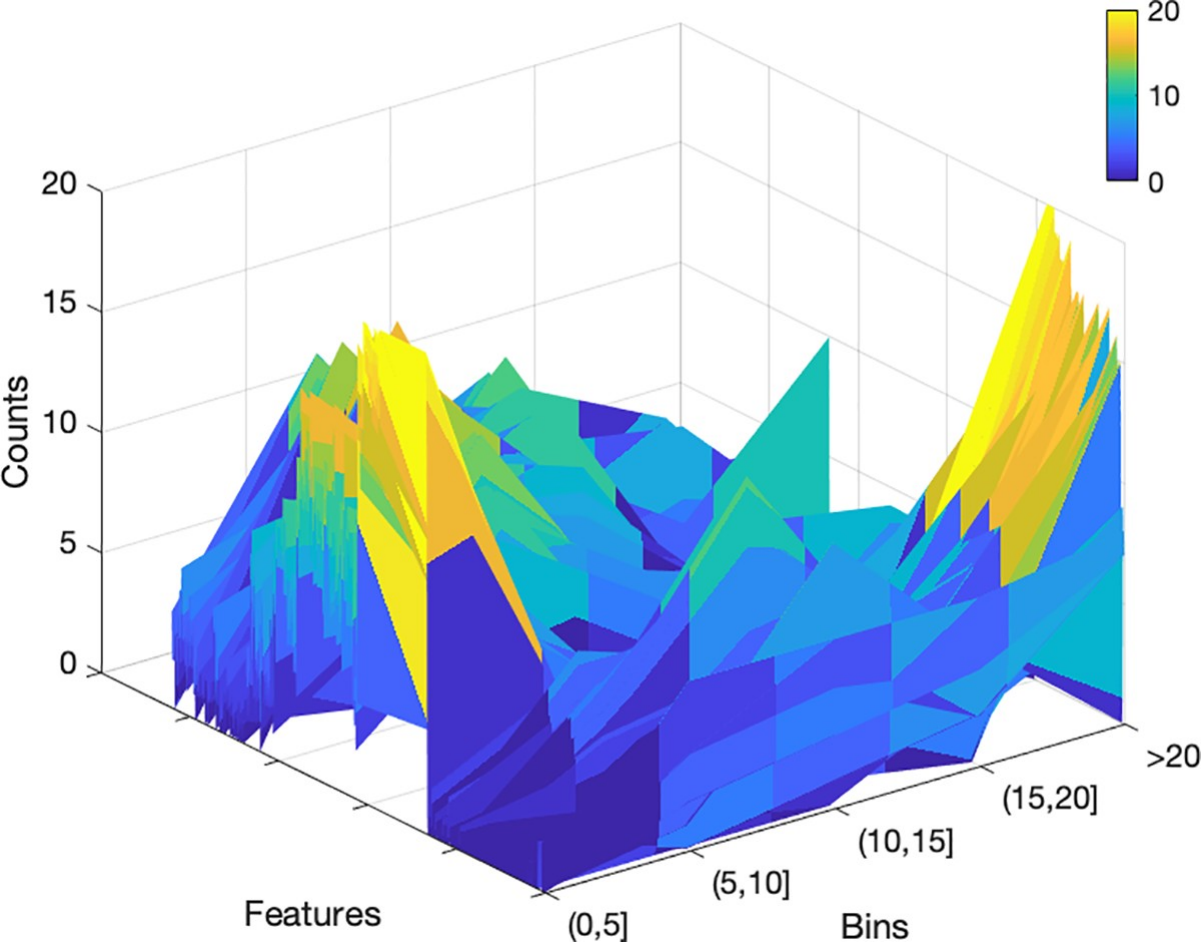
The distributions of COV for the population (total count = 20) among different stability bins (COV≤5%, 5%<COV≤10%, 10%<COV≤15%, 15%<COV≤20%, and COV>20%) for all features.

The radiomic features were grouped into 4 stability categories based on the population mean 4D COV. Of the total 841 radiomic features, 200 features (24%) were in the Very Small group (COV≤5%), 234 (28%) were Small (5%<COV≤10%), 189 (22%) were Intermediate (10%<COV≤20%), and 218 (26%) were Large (COV>20%). The distribution of radiomic feature grouping can be visualized in Fig 1. Comparing original vs. wavelet features, the original features had a lower percentage with COV>10% (i.e., more stable) than the wavelet-filtered features (38% vs. 50%), and the wavelet-LLL features had the lowest percentage (42%) among all wavelet features (Fig 1A). Comparing different feature classes, the Shape features were the most stable (Fig 1B), having 0% with COV>10% (i.e., all Shape features were in either the Very Small or Small group), and the NGTDM features were the least stable, having none in the Very Small group. Table 2 describes the detailed grouping information for the original features divided by feature class and stability grouping. Fig 2 plots the COV histogram (distribution) in 5 bins of each feature for the population.

**Table 2:** Radiomic feature 4D variability grouping based on population mean COV calculated over individual 4D breathing phases.

Feature Class	Very Small (COV≤5%)	Small (5%<COV≤10%)	Intermediate (10%<COV≤20%)	Large (COV>20%)
Shape	*M3DD*, *M2DDS*, *Sphericity*, *MinorAxis*, *SVR*, *MajorAxis*, *LA*	*Elongation*, *Volume*, *SA*, *Flatness*, *M2DDC*, *M2DDR*	* *	* *
First Order	*Minimum*, *Entropy*, *10Percentile*	*IR*, *Uniformity*, *RMAD*, *MAD*, *RMS*, *Range*, *Variance*, *Kurtosis*, *Mean*	*TotalEnergy*, *Energy*	*Skewness*, *Median*, *Maximum*, *90Percentile*
GLCM	*JE*, *IDMN*, *DE*, *IDN*, *SE*, *IMC2*, *ID*	*JA*, *SA*, *Contrast*, *IV*, *DV*, *IDM*, *Correlation*, *IMC1*, *DA*	*JointEnergy*, *Autocorrelation*, *SumSquares*, *CP*, *CT*	*CS*, *MP*
GLRLM	LRE, SRE, RP, RunEntropy, RLNUN	GLV, GLNUN, GLNU, RLNU	RV, SRHGLE, LRHGLE, HGLRE	SRLGLE, LGLRE, LRLGLE
GLSZM	GLNUN, SZNUN, ZP, SAE, ZE	GLV, SZNU, GLNU	SAHGLE, HGLZE	ZV, LAE, LALGLE, LAHGLE, LGLZE, SALGLE
GLDM	SDE, DE	GLV, DNU, DNUN	HGLE, GLNU, SDHGLE, LDE	LDLGLE, Emphasis, DV, LDHGLE, SDLGLE, LGLE
NGTDM		Coarseness	Complexity, Strength, Contrast, Busyness	

### Feature stability and tumor motion magnitude

**Fig 3:**
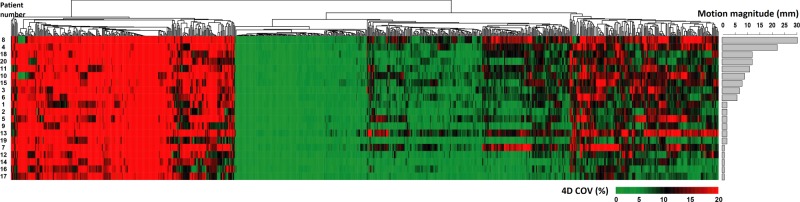
Heat map of the individual COV_4D_ plotted against the patients’ tumor motion magnitude. Rows of the heatmap represent different patients (ordered by the patient’s tumor motion magnitude) and columns describe features. The cell color, changing gradually from green to red, indicates increasing 4D COV from 0% to≥20%. Features were grouped with the complete-linkage hierarchical clustering method, based on the Euclidean distance.

The tumor motion magnitude of the 20 studied patients ranged from 1 mm to 30 mm. In Fig 3 we plotted a heat map of each patient’s individual COVs for all features along with a bar plot of the tumor motion magnitude of that patient. Clearly, some features are unstable for all patients as shown in the red cluster, and others are stable for all patients as shown in the green cluster. There are also features that are more stable for some patients than for others, as shown in those clusters with varying shadings. For these features, no obvious trend could be observed between the feature stability and the tumor motion magnitude. The detailed 4D feature stability clustering information can be found in the supplementary file.

### The impact of 4D stability on prognostic prediction

#### Radiomics heat map

**Fig 4:**
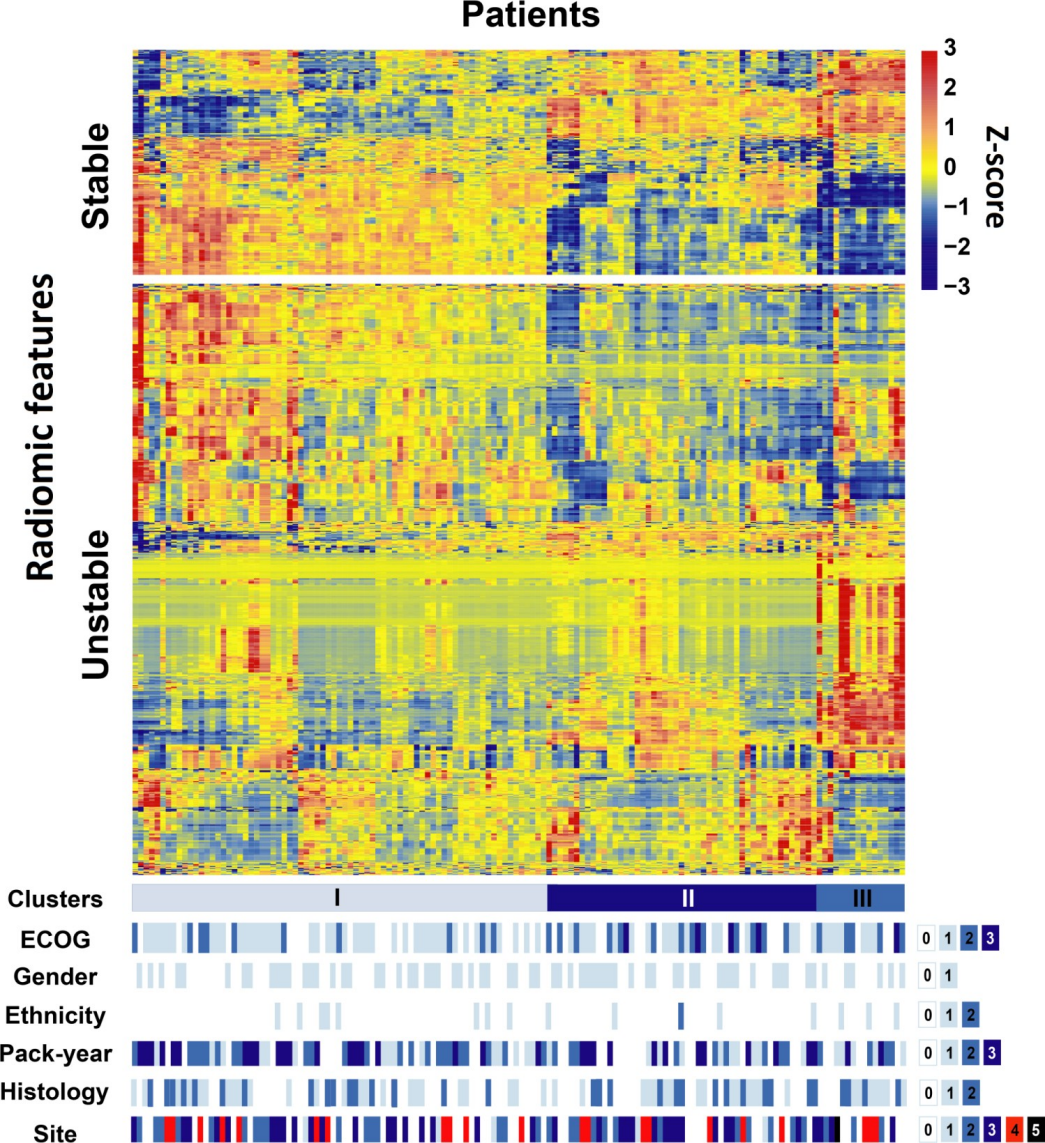
Radiomic heat map. Unsupervised clustering of patients based on the Stable features (COV≤5%) on the y axis (n = 140) and the radiomic feature expression patterns separately within the Stable (n = 200) and Unstable groups (n = 641) on the x axis. Clinical parameters for the patients are also illustrated at the bottom. For gender, 0 = male and 1 = female; for race (ethnicity), 0 = Caucasian, 1 = African-American, and 2 = others; for pack-year, the patients were divided into 4 quartiles and 0–3 represent the quartiles from low to high; for histology, 0 = adenocarcinoma, 1 = squamous cell carcinoma, and 2 = non-small cell not otherwise classified pathologically; for tumor location (site), 0 = right upper lobe, 1 = right middle lobe, 2 = right lower lobe, 3 = left upper lobe, 4 = left lower lobe, and 5 = chest wall.

The 140 patients were clustered by heat map into 3 clusters with similar Stable (COV≤5%) radiomic expression patterns and compared against patient clinical parameters (Fig 4). Among the clinical parameters, only ECOG performance status was significantly associated with patient clusters (p = 0.036) while the other included clinical parameters including gender (p = 0.421), race (ethnicity) (p = 0.520), histology (p = 0.827), tumor location (site) (p = 0.416) and pack-year smoking history, (p = 0.294) were not.

#### The impact of 4D stability on radiomic prediction for overall survival

Multiple Cox regression predictive models were developed from different feature pools with varying or no 4D stability pre-selection with a 500-round bootstrapping. For each round, the patients in the test dataset were grouped into low- and high-risk groups based on their risk scores calculated from the prediction model. The logrank χ^2^ was calculated between the predicted low- and high-risk groups for each bootstrap sampling round for each model with a higher χ^2^ indicating better model prediction performance.

**Fig 5:**
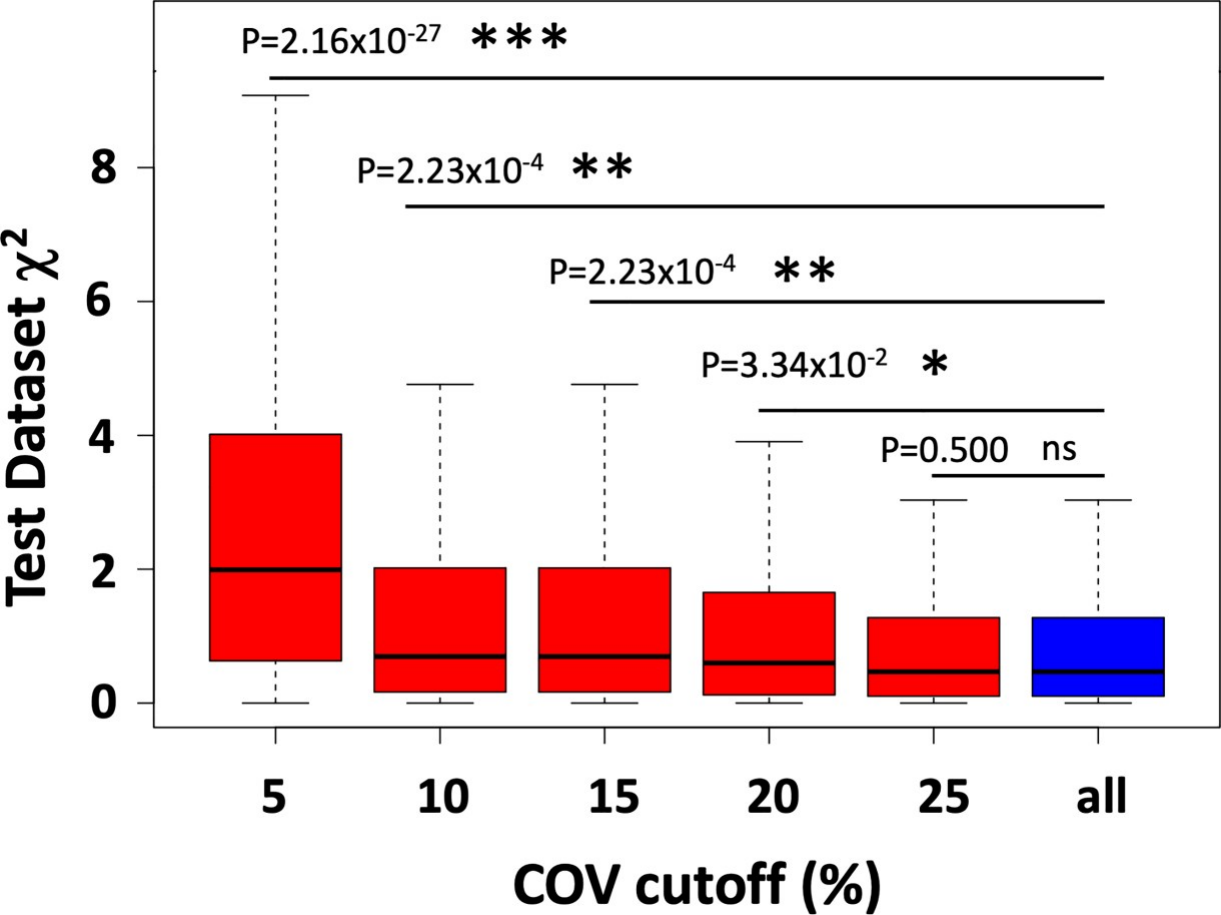
A box plot of the χ^2^ values of data in the test datasets during the 500 bootstrapping round of multiple regression for overall survival prediction with different COV cutoffs and all features. For each model, a higher χ^2^ indicates a superior prediction performance, i.e. a large difference between high risk cases and low risk cases. Except COV cutoffs, all models had the same setting, i.e. a univariate regression P-value cutoff of 0.01 and Q-value cutoff of 0.05 were used by all models. The box plot shows χ^2^ statistics among the 500 bootstrapping rounds, including the median (middle horizontal line), the first and third quartile (the lower and upper bound of the box), and the minimum and maximum (the dash line limits). A comparison of prediction performance between using a given COV cutoff and not using any COV cutoff (i.e. modeling with all features) was conducted. The P-value of the one-tailed t-test comparing the logarithm of χ^2^ values between the model applying a given 4D COV cutoff and its corresponding model without any 4D COV pre-selection (i.e. all) is shown for each 4D pre-selection model with a given COV cutoff. *** indicates P-value < 10^−5^, ** indicates <10^−3^, * indicates P-value < 0.05, and “ns” indicates not significant (p-value > 0.05).

**Fig 6:**
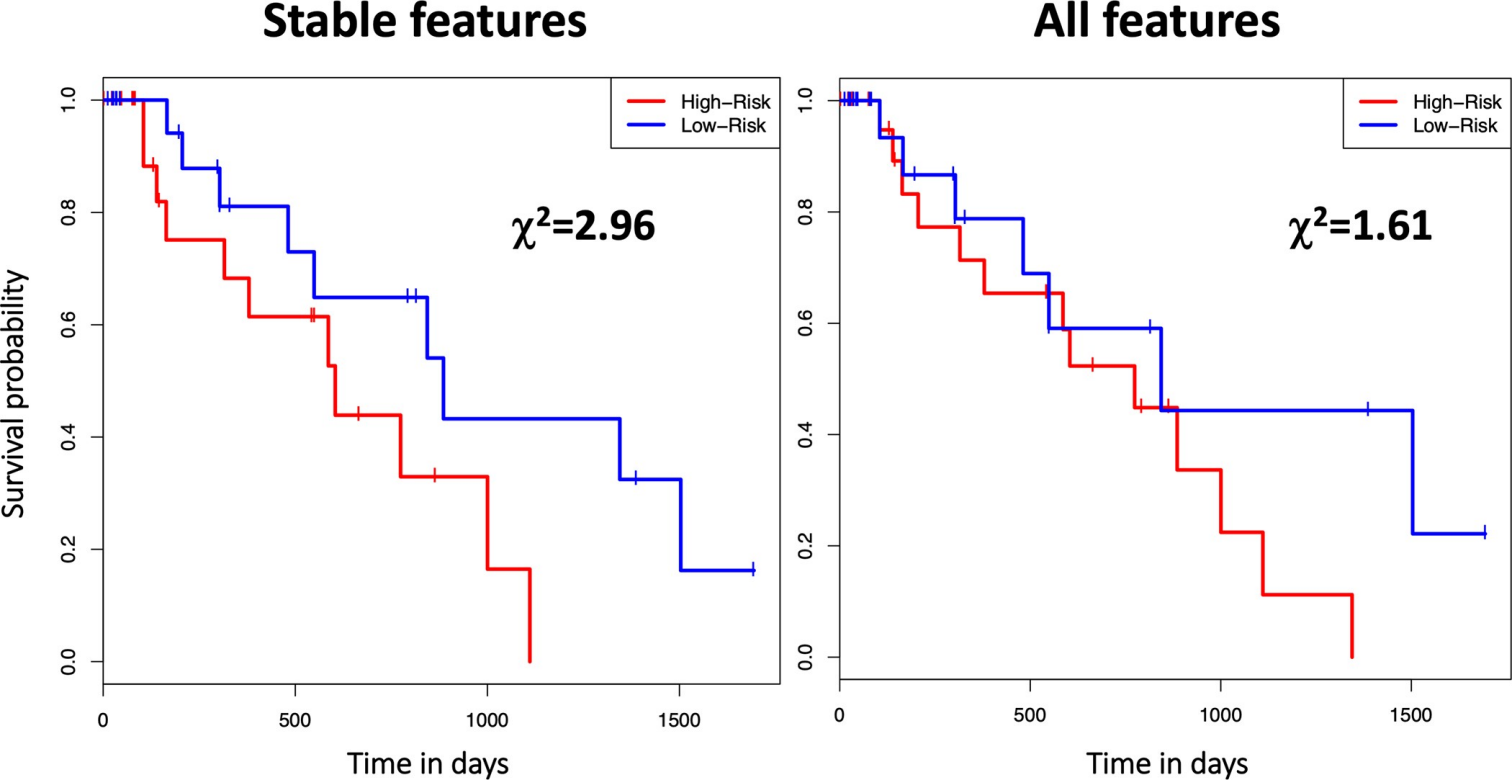
Kaplan-Meier survival curves of an example bootstrapping round to compare the 4D pre-selection model with the optimal cutoff (4D COV≤5%) and the corresponding model without 4D stability pre-selection. For each case, the Kaplan-Meier survival curves are shown to compare the overall survival of the model predicted low- and high-risk groups based on the median risk score. The χ^2^ value from the logrank test comparing the two risk groups is also shown.

Models were developed from the feature pools applying 4D stability pre-selection with varying 4D COV cutoffs and the all-feature pool without 4D pre-selection. Prior to 4D pre-selection, an additional feature pre-selection based on a univariate regression P-value cutoff of 0.01 and Q-value cutoff of 0.05 was applied to all pools. In the univariate regression pre-selection, the P-value cutoff was used to control the false positive rate and the false discovery rate (FDR) adjusted Q-value cutoff was used to control the FDR. Pre-selecting 4D stable features by applying increasingly strict 4D COV cutoffs yielded models with superior performances than the model developed without 4D stability pre-selection (Fig 5). Optimal model performance was achieved at feature pre-selection with a 4D COV cutoff of 5% (i.e., keeping only the features in the Very Small group in the 4D stability study described in III.1). This optimal model consisted of 3 wavelet texture features: wavelet_HHL_glcm_Id, wavelet_HHH_glcm_DifferenceAverage, and wavelet_HLL_gldm_DependenceNonUniformityNormalized. Kaplan-Meier survival curves also revealed that bootstrapping of the optimal 4D pre-selection model (with 4D COV≤5%) was superior to the model without 4D pre-selection in predicting overall survival between low- and high-risk patients (Fig 6).

Further, one-tailed t-test comparing the logarithm of the test-dataset χ^2^ values from the entire 500 bootstrapping rounds between each 4D pre-selection model and the corresponding model without 4D pre-selection while systematically varying the cutoffs for 4D stability as well as the cutoffs for the univariate regression P-values and Q-values revealed that the 4D stability pre-selection model significantly outperforms the corresponding model without 4D stability pre-selection (Table 3). In this analysis, feature pre-selection using 4D COV≤5% and univariate regression P-value<0.01, Q-value<0.05 achieved the greatest model performance improvement, at P = 2.16x10^-27^ for the one-tailed t-test compared with its corresponding model without 4D stability pre-selection.

**Table 3:** The P-values of the one-tailed t-test comparing the logarithm of χ^2^ values between the model meeting a given pre-selection criteria (applying both the 4D COV and the combined univariate regression P-value and FDR adjusted Q-value cutoffs) and its corresponding model without 4D COV pre-selection (applying the combined univariate regression P-value and FDR adjusted Q-value cutoffs alone). A 3-feature radiomic model developed from the stable features with 4D COV≤5% and univariate regression P-value<0.01 and Q-value<0.05 achieved the most significantly improved performance over its counterpart without 4D COV pre-selection (i.e. developed from all features with univariate regression P-value<0.01 and Q-value<0.05 regardless of their 4D COV values), with a P-value of 2.16x10^-27^.

Pre-selection criteria	Univariate P<0.005, Q<0.04	Univariate P<0.007, Q<0.045	Univariate P<0.01, Q<0.05	Univariate P<0.03, Q<0.07	Univariate P<0.05, Q<0.09
4D COV≤5%	6.07 x10^-8^	1.28 x10^-16^	2.16 x10^-27^	1.66 x10^-7^	0.037
4D COV≤10%	7.93 x10^-3^	9.84 x10^-4^	2.23 x10^-4^	1.94 x10^-2^	0.463
4D COV≤15%	7.93 x10^-3^	9.84 x10^-4^	2.23 x10^-4^	1.974 x10^-2^	0.302
4D COV≤20%	0.258	0.137	3.34 x10^-2^	0.154	0.396
4D COV≤25%	0.500	0.500	0.500	0.087	0.016

## Discussion

As an emerging frontier, radiomics has become increasingly important in cancer research. With its high-throughput mining of rich, quantitative information from standard-of-care medical images to facilitate clinical decision making, radiomics is recognized as the bridge between medical imaging and personalized medicine[4].While we are excited and galvanized by the great potentials this new approach opens for personalized medicine, it needs to be cautioned that the data and process quality assurance is an essential step for the success of radiomics. To correctly correlate radiomic signatures with meaningful clinical endpoints, the quantitative differences of radiomic features need to reflect the real inter-patient variability instead of reflecting other underlining fluctuations or noise of the features. On this important aspect, the body of literature is also growing, albeit at a much slower rate than that of the literature on clinical studies using radiomics. Recently, studies have been reported to compare the inter-scan, inter-reader, inter-scanner, inter-acquisition parameter, and inter-reconstruction-algorithm variability of radiomic features[22–28]. However, there is a paucity of studies that considered the inter-respiratory-phase variability of radiomic features. Investigations on this new aspect of feature stability are critically needed due to the rapidly-outpouring reports on radiomic investigations of lung cancer and the clear susceptibility of lung tumors to respiratory motions.

Regarding the respiratory motion, the existing radiomic studies on lung cancer usually do not explicitly consider it. The radiomic data are most often extracted from regular free-breathing CTs[3,5–7], although one study suggested that the AIP image generated from the 4D CT contained more information than the regular free-breathing CT and could be more optimal for radiomic studies[15]. In another recent study[16], Larue *et*. *al*. used 4D CT scans to analyze feature robustness for 20 lung cancer patients and 20 esophageal cancer patients, and they concluded that feature 4D stability was tumor-site specific and independent of prognostic value. In our study, we first analyzed the feature 4D stability on 20 NSCLC patients. In this analysis we adopted the COV classification used by the previous stability studies investigating feature stability over varying image reconstruction settings[20], and about a quarter of all features fell into the most stable (with Very Small COV) or the least stable (with Large COV) categories. Larue *et*. *al*. observed that a higher percentage of original features are stable than the wavelet features[16] and our study made a similar observation (62% vs. 50% with COV≤10%). This seems to indicate that applying wavelet filters makes the features less stable against the respiratory motion. However, with closer inspection, the difference was actually due to the fact that the Shape feature class was the most stable across 4D phases (100% of Shape features with COV≤10%). Because Shape features do not change with wavelet filters, they are not included in the wavelet features. If Shape features were excluded, exactly the same fraction of original features (50%) had COV≤10% as the wavelet features. Therefore, from the 4D analysis, our study found that Shape features were the most stable among different feature classes, and applying wavelet filters did not undermine feature stability over 4D respiratory phases. In addition, our study also analyzed the individual 4D COV against the tumor motion magnitude. From the results, it was clear that some features were stable for all patients and others were unstable for all patients, regardless of the motion magnitude. But for features that had varying stability among patients, no obvious trend was observed between the feature stability and the motion magnitude.

Our study then applied the 4D stability results from the first part of the investigation to a clinical study of 140 patients to investigate the impact of feature 4D stability on prognostic prediction. This is, to our knowledge, the first study to explicitly apply 4D stability as a feature selection step and compare the model performance with and without it. Indeed our results unequivocally demonstrated the usefulness of applying the 4D stability feature pre-selection.

Two interesting future research directions based on the current study are to apply the 4D feature selection on clinical predictive studies based on average-intensity-projection images and to compare the feature selection as well as prediction difference between 4D feature selection and the conventional test-retest selection. For the former, the 4D stability characteristics may be more translatable onto the average-intensity-projection images as they all come from the 4D CT, and such a study will become more relevant when more clinical investigations start to use average-intensity-projection instead of free-breathing CT as recommended by Huynh et al^15^. For the latter, Larue et al. found that the feature selection showed some similarity and some difference between the 4D method and the test-retest method^16^. This is not surprising because although the two methods have their different focuses, they also have some inherent overlaps. Respiration-induced feature changes may be randomly included in the test-retest image samples, and the feature variations captured by the 4D method will also contain a noise component. It will also be interesting to further look at the two methods’ comparison in prediction tasks.

There are some limitations to our study. First of all, our stability analysis patient size was limited at 20 patients and the statistics might be strengthened if more patients were studied. On the other hand, such a sample size is typical in reproducibility/stability studies[16,20,22–24,27]. This is because, unlike clinical radiomic studies that need very large sample sizes to suppress many confounding factors and correlate the inter-patient feature variability with clinical endpoints, in a reproducibility/stability study the investigated perturbations are singled out and each patient acts as its own control. In this part of study, for each patient the investigated image sets of individual breathing phases acted as each other’s control.

In the clinical analysis of our study, 140 patients were included which also might be strengthened with an even larger sample size and an external validation set but nevertheless represented a sample size on par with the literature. In this analysis, we performed a 500-round bootstrapping, as bootstrapping can provide nearly unbiased estimates of model performance without sacrificing sample size[29]. For each round of bootstrapping, a same-size of samples were randomly selected with replacement from all original samples as the training dataset, while those out-of-bag samples not selected for model fitting were used as the test dataset. With our sample size of 140 patients, the bootstrapping had a training/test split of about 0.632/0.368. It is worth noting that we applied bootstrapping in our study as it is arguably the most efficient validation procedure that makes the most use of all available samples due to no holdout, and it was sufficient in our study focused on assessing the prediction performance differences with and without 4D stability pre-selection. For clinical studies that aim at developing the optimal predictive model, other validation approached such as cross-validation may also be considered and compared for performance. Also, we did not exclude the 20 patients used for the 4D stability study from the 140 patients for the clinical study, for the larger sample size of the clinical study. This could have introduced some bias, such that the quantitative results might be somewhat different if these patients were excluded. Yet the focus of the study was to show the impact of 4D stability feature selection in the clinical radiomic prediction using one specific example. The chosen clinical endpoint and the exact quantitative results related to the detailed experiment design were of less importance.

Another limitation of our study is that our patient population is specifically limited to early-stage NSCLC treated with SBRT and therefore may not be generalized to patients receiving other types of treatments or other lung tumor stages or histology. It should be noted, however, that we selected early-stage NSCLC both because of its prevalence in clinical radiomic research[6,8,9] and because these small lung tumors tend to have larger motion magnitudes than larger tumors[30], therefore representing a worse-case scenario better justified for the respiratory motion radiomic feature stability investigation. Also a limitation is the clinical endpoint we chose to model in our clinical study: Cause-specific survival may be more clinically meaningful than overall survival which we used in our study, because of the low cause-specific motality rate of early-stage NSCLC and the advanced ages for these patients. However, the cause-specific survival data in our cohort is unattainable as a number of these patients are lost to followup or are followed up elsewhere with their primary care physicians outside of our institution, making it difficult to assess for cause of death. As such, clinical studies on early-stage NSCLC with SBRT often assess overall surivival instead of cause-specific survival. Also even if the cause-specific survival data is available, it would require a prohibitively large number of patients to conduct a meaningful analysis due to the very low event rate. Therefore most of the existing radiomic studies investigated overall survival. More importantly, the focus of our work is not to develop a new clinical radiomic model but to investigate and demonstrate the effects of 4D-based feature pre-selection on radiomic analyses. A clinical analysis based on the overall survival endpoint was only chosen based on the data available and because of the widely reported similar investigations. Similarly, a longer followup time than what our study cohort had available would also greatly help clinical studies focused on developing new predictive models. Stratifying the patients based on histology and conducting histology-specific radiomic analyses would also be of future clinical interest.

Last but not least, although 4D CT largely mitigates the respiratory motion artifacts compared with free-breathing CT, it is still subject to various residual artifacts[31]. These artifacts, along with other factors such as the tumor segmentation uncertainty[32], also impact the uncertainty of our study results. In addition, 4D CT imposes an additional radiation dose of a few cGys to the patient, although 4D CT is routinely used for lung cancer patients receiving radiotherapy and as shown in our example, for patients on whom a radiomics-based predictive model is being developed, 4D CTs are not required to apply the 4D stability feature selection.

## Conclusions

The respiration-related stability of radiomic features was investigated across individual respiratory phases for non-small-cell lung cancer. Respiration does lead to considerable variability for some radiomic features, while other features are more robust against it. Features in the “Shape” class appear to be the most robust with regard to respiration, and applying wavelet filters does not change the 4D stability of the features. Radiomic feature 4D stability was used as a feature selection step to assess its impact on prognostic prediction for overall survival on a cohort of early-stage non-small-cell lung cancer patients that received stereotactic body radiotherapy, and it was found to improve the prediction performance.

## Supporting information

S1 File List of features in each cluster. (PDF)

S2 File List of features used with cutoffs of P-values, Q-values, and COV. (PDF)

S3 File List of all feature data. (PDF)

## Abbreviations

COV: coefficient of variation
CT: computed tomography
ECOG: Eastern Cooperative Oncology Group
4D: four-dimensional
GLCM: Gray Level Co-occurrence Matrix
GLDM: Gray Level Dependence Matrix
GLRLM: Gray Level Run Length Matrix
GLSZM: Gray Level Size Zone Matrix
NGTDM: Neighboring Gray Tone Difference Matrix
NSCLC: non-small-cell lung cancer
