# Supplementary material for: Radiomic feature stability across 4D respiratory phases and its impact on lung tumor prognosis prediction
Source: PLoS One. 2019 May 7;14(5):e0216480. doi: 10.1371/journal.pone.0216480 (PMC6504105; doi:10.1371/journal.pone.0216480)
Supplement: S1 File — (PDF) [file pone.0216480.s001.pdf]

Supplementary File 1: List of features in each cluster

Cluster (Left Red)

wavelet_LLL_glszm_LargeAreaEmphasis
original_glcm_MaximumProbability
original_glcm_JointEnergy
wavelet_LLL_glcm_MaximumProbability
wavelet_HHL_firstorder_Maximum
wavelet_HHH_firstorder_Maximum
wavelet_HLL_gldm_DependenceVariance
wavelet_HLL_glcm_MaximumProbability
wavelet_HHH_glrlm_ShortRunLowGrayLevelEmphasis
wavelet_HHH_glrlm_LongRunLowGrayLevelEmphasis
wavelet_HHH_gldm_LowGrayLevelEmphasis
wavelet_HHH_glrlm_LowGrayLevelRunEmphasis
wavelet_HHH_glrlm_LongRunHighGrayLevelEmphasis
wavelet_HHH_glrlm_ShortRunHighGrayLevelEmphasis
wavelet_HHH_glcm_Autocorrelation
wavelet_HHH_gldm_HighGrayLevelEmphasis
wavelet_HHH_glrlm_HighGrayLevelRunEmphasis
wavelet_HHH_gldm_LargeDependenceLowGrayLevelEmphasis
wavelet_HHH_gldm_LargeDependenceHighGrayLevelEmphasis
wavelet_HHH_glszm_HighGrayLevelZoneEmphasis
wavelet_LLL_glszm_LargeAreaLowGrayLevelEmphasis
wavelet_LLL_gldm_LargeDependenceLowGrayLevelEmphasis
wavelet_LLL_glszm_LowGrayLevelZoneEmphasis
wavelet_LLL_glrlm_LongRunLowGrayLevelEmphasis
wavelet_LLL_gldm_LowGrayLevelEmphasis
wavelet_LLL_glrlm_ShortRunLowGrayLevelEmphasis
wavelet_LLL_glrlm_LowGrayLevelRunEmphasis
wavelet_LLL_gldm_SmallDependenceLowGrayLevelEmphasis
wavelet_LLL_glszm_SmallAreaLowGrayLevelEmphasis
wavelet_LHH_ngtdm_Contrast
wavelet_HLH_glcm_ClusterProminence
wavelet_HHH_glcm_ClusterProminence
wavelet_HHL_ngtdm_Complexity
wavelet_HHL_ngtdm_Strength
wavelet_HHH_ngtdm_Strength
wavelet_HHH_ngtdm_Contrast
wavelet_HHH_ngtdm_Complexity
wavelet_HHH_ngtdm_Busyness
wavelet_LHL_glcm_ClusterProminence
wavelet_LHH_glcm_ClusterProminence
wavelet_LLH_glszm_LargeAreaLowGrayLevelEmphasis
wavelet_HLH_gldm_SmallDependenceHighGrayLevelEmphasis
wavelet_HLH_gldm_LargeDependenceLowGrayLevelEmphasis

wavelet_HLH_glszm_SmallAreaHighGrayLevelEmphasis
wavelet_HLH_glszm_HighGrayLevelZoneEmphasis
wavelet_HLH_glrlm_LongRunHighGrayLevelEmphasis
wavelet_HLH_glcm_Autocorrelation
wavelet_HLH_glrlm_ShortRunHighGrayLevelEmphasis
wavelet_HLH_gldm_HighGrayLevelEmphasis
wavelet_HLH_glrlm_HighGrayLevelRunEmphasis
wavelet_HLL_gldm_LargeDependenceHighGrayLevelEmphasis
wavelet_HLL_glszm_SmallAreaLowGrayLevelEmphasis
wavelet_HLL_gldm_SmallDependenceLowGrayLevelEmphasis
wavelet_HLL_glszm_LowGrayLevelZoneEmphasis
wavelet_HLL_glrlm_LongRunLowGrayLevelEmphasis
wavelet_HLL_glrlm_ShortRunLowGrayLevelEmphasis
wavelet_HLL_gldm_LowGrayLevelEmphasis
wavelet_HLL_glrlm_LowGrayLevelRunEmphasis
wavelet_LHH_glcm_Correlation
wavelet_LHL_glrlm_ShortRunHighGrayLevelEmphasis
wavelet_LHL_gldm_HighGrayLevelEmphasis
wavelet_LHL_glrlm_HighGrayLevelRunEmphasis
wavelet_LHL_glcm_Autocorrelation
wavelet_LHL_glrlm_LongRunHighGrayLevelEmphasis
wavelet_LHL_glszm_SmallAreaHighGrayLevelEmphasis
wavelet_LHL_gldm_SmallDependenceHighGrayLevelEmphasis
wavelet_LHL_glszm_HighGrayLevelZoneEmphasis
original_gldm_SmallDependenceLowGrayLevelEmphasis
original_glszm_LowGrayLevelZoneEmphasis
original_glrlm_LongRunLowGrayLevelEmphasis
original_glrlm_ShortRunLowGrayLevelEmphasis
original_gldm_LowGrayLevelEmphasis
original_glrlm_LowGrayLevelRunEmphasis
original_glszm_ZoneVariance
original_glszm_LargeAreaLowGrayLevelEmphasis
original_glszm_LargeAreaEmphasis
original_glszm_LargeAreaHighGrayLevelEmphasis
wavelet_HLL_glszm_ZoneVariance
wavelet_LHL_glszm_ZoneVariance
wavelet_LHL_glszm_LargeAreaEmphasis
wavelet_HHL_gldm_SmallDependenceHighGrayLevelEmphasis
wavelet_HHL_glszm_LargeAreaHighGrayLevelEmphasis
wavelet_HHL_ngtdm_Busyness
wavelet_HHL_glcm_Autocorrelation
wavelet_HHL_glrlm_ShortRunHighGrayLevelEmphasis
wavelet_HHL_gldm_HighGrayLevelEmphasis
wavelet_HHL_glrlm_HighGrayLevelRunEmphasis
wavelet_HHL_gldm_LargeDependenceHighGrayLevelEmphasis

wavelet_HHL_glrlm_LongRunHighGrayLevelEmphasis
wavelet_HHL_glszm_SmallAreaHighGrayLevelEmphasis
wavelet_HHL_glszm_HighGrayLevelZoneEmphasis
wavelet_LHH_gldm_LargeDependenceHighGrayLevelEmphasis
wavelet_LHH_gldm_SmallDependenceHighGrayLevelEmphasis
wavelet_LHH_ngtdm_Busyness
wavelet_LHH_glszm_HighGrayLevelZoneEmphasis
wavelet_LHH_glszm_SmallAreaHighGrayLevelEmphasis
wavelet_LHH_glrlm_LongRunHighGrayLevelEmphasis
wavelet_LHH_glcm_Autocorrelation
wavelet_LHH_glrlm_ShortRunHighGrayLevelEmphasis
wavelet_LHH_gldm_HighGrayLevelEmphasis
wavelet_LHH_glrlm_HighGrayLevelRunEmphasis
wavelet_LLL_glszm_ZoneVariance
wavelet_LHH_ngtdm_Complexity
wavelet_LHH_gldm_LargeDependenceLowGrayLevelEmphasis
wavelet_LHH_glrlm_LongRunLowGrayLevelEmphasis
wavelet_LHH_glrlm_ShortRunLowGrayLevelEmphasis
wavelet_LHH_gldm_LowGrayLevelEmphasis
wavelet_LHH_glrlm_LowGrayLevelRunEmphasis
wavelet_LHH_ngtdm_Strength
wavelet_HLH_gldm_LargeDependenceHighGrayLevelEmphasis
wavelet_LLH_gldm_LargeDependenceLowGrayLevelEmphasis
wavelet_LLH_glszm_LargeAreaHighGrayLevelEmphasis
wavelet_LLH_glrlm_LongRunLowGrayLevelEmphasis
wavelet_LLH_gldm_LowGrayLevelEmphasis
wavelet_LLH_glrlm_ShortRunLowGrayLevelEmphasis
wavelet_LLH_glrlm_LowGrayLevelRunEmphasis
wavelet_LLH_glszm_LowGrayLevelZoneEmphasis
wavelet_LLH_gldm_SmallDependenceLowGrayLevelEmphasis
wavelet_LLH_glszm_SmallAreaLowGrayLevelEmphasis
wavelet_LHH_glszm_LargeAreaHighGrayLevelEmphasis
wavelet_LHH_glszm_LowGrayLevelZoneEmphasis
wavelet_LHH_gldm_SmallDependenceLowGrayLevelEmphasis
wavelet_LHH_glszm_SmallAreaLowGrayLevelEmphasis
wavelet_LHL_gldm_LargeDependenceHighGrayLevelEmphasis
wavelet_LHL_glrlm_LongRunLowGrayLevelEmphasis
wavelet_LHL_gldm_LowGrayLevelEmphasis
wavelet_LHL_glrlm_ShortRunLowGrayLevelEmphasis
wavelet_LHL_glrlm_LowGrayLevelRunEmphasis
wavelet_LHL_gldm_SmallDependenceLowGrayLevelEmphasis
wavelet_LHL_glszm_LowGrayLevelZoneEmphasis
original_glszm_SmallAreaLowGrayLevelEmphasis
wavelet_HLL_glszm_LargeAreaHighGrayLevelEmphasis
wavelet_LHL_glszm_LargeAreaHighGrayLevelEmphasis

wavelet_HLH_glszm_LargeAreaHighGrayLevelEmphasis
wavelet_HHL_gldm_LargeDependenceLowGrayLevelEmphasis
wavelet_HHL_glszm_LargeAreaLowGrayLevelEmphasis
wavelet_LHL_gldm_LargeDependenceLowGrayLevelEmphasis
wavelet_LHL_glszm_LargeAreaLowGrayLevelEmphasis
wavelet_LLH_firstorder_Skewness
wavelet_HLH_glszm_LargeAreaLowGrayLevelEmphasis
wavelet_HLL_firstorder_Skewness
wavelet_LHL_firstorder_Skewness
wavelet_HLH_glrlm_LongRunLowGrayLevelEmphasis
wavelet_HLH_glrlm_ShortRunLowGrayLevelEmphasis
wavelet_HLH_gldm_LowGrayLevelEmphasis
wavelet_HLH_glrlm_LowGrayLevelRunEmphasis
wavelet_HLH_glszm_SmallAreaLowGrayLevelEmphasis
wavelet_HHL_firstorder_Skewness
wavelet_LHL_glszm_SmallAreaLowGrayLevelEmphasis
wavelet_HHL_glszm_LowGrayLevelZoneEmphasis
wavelet_HHL_gldm_SmallDependenceLowGrayLevelEmphasis
wavelet_HHL_glszm_SmallAreaLowGrayLevelEmphasis
wavelet_LHH_glszm_LargeAreaLowGrayLevelEmphasis
wavelet_HHL_glcm_ClusterShade
wavelet_HLH_gldm_SmallDependenceLowGrayLevelEmphasis
wavelet_HLH_glszm_LowGrayLevelZoneEmphasis
wavelet_HLH_glcm_Correlation
wavelet_HHH_glszm_SmallAreaLowGrayLevelEmphasis
wavelet_HLH_firstorder_Mean
wavelet_HLH_firstorder_Median
wavelet_HLH_firstorder_Skewness
wavelet_HLH_glcm_ClusterShade
wavelet_HHL_firstorder_Mean
wavelet_HHL_firstorder_Median
wavelet_HHL_glcm_Correlation
wavelet_LLH_glcm_ClusterShade
wavelet_LHH_firstorder_Mean
wavelet_LHH_firstorder_Median
wavelet_LHH_firstorder_Skewness
wavelet_LHH_glcm_ClusterShade
wavelet_LHL_glcm_ClusterShade
wavelet_HHH_firstorder_Mean
wavelet_HHH_firstorder_Median
wavelet_HHH_glcm_ClusterShade
wavelet_HHH_firstorder_Skewness
wavelet_HHH_gldm_SmallDependenceHighGrayLevelEmphasis
wavelet_HHH_glszm_SmallAreaHighGrayLevelEmphasis
wavelet_HHL_glrlm_LongRunLowGrayLevelEmphasis

wavelet_HHL_gldm_LowGrayLevelEmphasis
wavelet_HHL_glrlm_ShortRunLowGrayLevelEmphasis
wavelet_HHL_glrlm_LowGrayLevelRunEmphasis
wavelet_HHH_glszm_LargeAreaLowGrayLevelEmphasis
wavelet_HHH_glszm_LargeAreaHighGrayLevelEmphasis
wavelet_HHH_gldm_SmallDependenceLowGrayLevelEmphasis
wavelet_HHH_glszm_LowGrayLevelZoneEmphasis
wavelet_HLL_glcm_ClusterShade
wavelet_HLL_gldm_LargeDependenceLowGrayLevelEmphasis
wavelet_HLL_glszm_LargeAreaLowGrayLevelEmphasis
wavelet_HLH_ngtdm_Busyness
wavelet_HLH_ngtdm_Complexity
wavelet_HLH_ngtdm_Strength
original_firstorder_Maximum
wavelet_LLL_firstorder_Maximum
wavelet_HLL_glszm_LargeAreaEmphasis
wavelet_LHL_ngtdm_Complexity
wavelet_LHL_ngtdm_Strength
wavelet_LHL_ngtdm_Busyness
wavelet_HLL_ngtdm_Strength
wavelet_LLH_firstorder_Maximum
wavelet_HLL_ngtdm_Complexity
wavelet_LHL_firstorder_Median
wavelet_LHH_firstorder_Minimum
wavelet_LHH_glcm_JointAverage
wavelet_LHH_glcm_SumAverage
wavelet_HLL_gldm_SmallDependenceHighGrayLevelEmphasis
wavelet_HLL_glrlm_LongRunHighGrayLevelEmphasis
wavelet_HLL_glrlm_ShortRunHighGrayLevelEmphasis
wavelet_HLL_gldm_HighGrayLevelEmphasis
wavelet_HLL_glrlm_HighGrayLevelRunEmphasis
wavelet_HLL_glszm_SmallAreaHighGrayLevelEmphasis
wavelet_HLL_glszm_HighGrayLevelZoneEmphasis
wavelet_HLL_glcm_Autocorrelation
wavelet_HLL_ngtdm_Busyness
wavelet_LLL_glszm_LargeAreaHighGrayLevelEmphasis
wavelet_LLL_gldm_DependenceVariance
wavelet_LLL_gldm_LargeDependenceHighGrayLevelEmphasis
original_firstorder_Skewness
wavelet_LLL_firstorder_Skewness
wavelet_HLH_glszm_ZoneVariance
wavelet_HLH_glszm_LargeAreaEmphasis
wavelet_LLH_glszm_ZoneVariance
wavelet_LLH_glszm_LargeAreaEmphasis
original_gldm_DependenceVariance

original_gldm_LargeDependenceHighGrayLevelEmphasis
wavelet_HHL_glcm_ClusterProminence
wavelet_HHL_glszm_ZoneVariance
wavelet_HHL_glszm_LargeAreaEmphasis
original_glcm_ClusterShade
wavelet_LLL_glcm_ClusterShade
original_gldm_LargeDependenceLowGrayLevelEmphasis
original_firstorder_90Percentile
wavelet_LLL_firstorder_90Percentile
wavelet_LLH_ngtdm_Strength
wavelet_LLH_ngtdm_Complexity
wavelet_LLH_ngtdm_Busyness
wavelet_LLH_glcm_ClusterProminence
wavelet_LLH_gldm_LargeDependenceHighGrayLevelEmphasis
wavelet_LLH_gldm_SmallDependenceHighGrayLevelEmphasis
wavelet_LLH_glszm_SmallAreaHighGrayLevelEmphasis
wavelet_LLH_glszm_HighGrayLevelZoneEmphasis
wavelet_LLH_glrlm_LongRunHighGrayLevelEmphasis
wavelet_LLH_glcm_Autocorrelation
wavelet_LLH_glrlm_ShortRunHighGrayLevelEmphasis
wavelet_LLH_gldm_HighGrayLevelEmphasis
wavelet_LLH_glrlm_HighGrayLevelRunEmphasis
original_firstorder_Energy
original_firstorder_TotalEnergy
wavelet_LLL_firstorder_Energy
wavelet_LLL_firstorder_TotalEnergy
original_glcm_ClusterProminence
wavelet_LLL_glcm_ClusterProminence
wavelet_HLL_glcm_ClusterProminence
wavelet_HLL_firstorder_Median
wavelet_HLL_firstorder_Mean
wavelet_HHH_firstorder_Energy
wavelet_HHH_firstorder_TotalEnergy
wavelet_HHH_glszm_ZoneVariance
wavelet_HHH_glszm_LargeAreaEmphasis
wavelet_HLH_ngtdm_Contrast
wavelet_LHH_glszm_SizeZoneNonUniformity
wavelet_LHH_glszm_ZoneVariance
wavelet_LHH_glszm_LargeAreaEmphasis
wavelet_LHH_firstorder_Maximum
wavelet_HHL_ngtdm_Contrast
wavelet_HHH_glszm_SizeZoneNonUniformity
wavelet_HHH_glszm_GrayLevelNonUniformity

Cluster (Middle Green)

wavelet_HHH_gldm_DependenceNonUniformityNormalized
original_glrlm_LongRunEmphasis
original_glcm_Idmn
wavelet_LLL_glcm_Idmn
wavelet_LHL_glcm_Idmn
wavelet_LLH_glcm_Idmn
wavelet_HHH_glcm_Idmn
wavelet_HLH_glcm_Idmn
wavelet_LHH_glcm_Idmn
wavelet_HHL_glcm_Idmn
wavelet_LLL_glcm_Imc2
wavelet_LHL_glrlm_ShortRunEmphasis
original_glrlm_ShortRunEmphasis
wavelet_LLH_glrlm_ShortRunEmphasis
wavelet_HLL_glrlm_RunPercentage
wavelet_LLL_glrlm_RunPercentage
wavelet_HLL_glrlm_ShortRunEmphasis
wavelet_LLL_glrlm_ShortRunEmphasis
original_glrlm_RunPercentage
wavelet_LHL_glrlm_RunPercentage
wavelet_LLH_glrlm_RunPercentage
wavelet_LLL_glrlm_RunLengthNonUniformityNormalized
wavelet_HLL_glrlm_RunLengthNonUniformityNormalized
wavelet_HHL_glrlm_ShortRunEmphasis
wavelet_HHL_glrlm_RunPercentage
wavelet_HLL_glrlm_RunEntropy
wavelet_HLL_glcm_SumEntropy
wavelet_HLL_firstorder_Entropy
wavelet_HLL_glcm_JointEntropy
wavelet_HHL_glrlm_RunLengthNonUniformityNormalized
original_glrlm_RunEntropy
original_glszm_ZoneEntropy
wavelet_LLL_glcm_JointEntropy
wavelet_LLL_glcm_SumEntropy
wavelet_LLL_firstorder_Entropy
original_glcm_DifferenceEntropy
wavelet_LLL_glcm_DifferenceEntropy
wavelet_LLL_glrlm_LongRunEmphasis
original_glrlm_RunLengthNonUniformityNormalized
wavelet_LLH_glrlm_RunLengthNonUniformityNormalized
wavelet_HHH_glrlm_ShortRunEmphasis
wavelet_HHH_glrlm_RunPercentage
wavelet_LHH_glrlm_RunPercentage
wavelet_LHL_glrlm_RunLengthNonUniformityNormalized

wavelet_LHH_glrlm_ShortRunEmphasis
wavelet_LLL_glszm_SmallAreaEmphasis
wavelet_HHH_glrlm_RunEntropy
wavelet_HHL_glrlm_RunEntropy
wavelet_HHH_gldm_DependenceEntropy
wavelet_HHH_glcm_InverseVariance
wavelet_HLH_glrlm_ShortRunEmphasis
wavelet_HLH_glrlm_RunPercentage
wavelet_HHH_glcm_Idn
wavelet_LHH_glcm_Idn
wavelet_HHL_glcm_Idn
wavelet_LHL_gldm_DependenceEntropy
wavelet_LHH_gldm_DependenceEntropy
wavelet_LLL_glrlm_RunEntropy
wavelet_LLL_gldm_DependenceEntropy
wavelet_LLL_glszm_ZoneEntropy
original_gldm_DependenceEntropy
wavelet_HLL_gldm_DependenceEntropy
wavelet_HLL_glszm_ZoneEntropy
wavelet_HLH_gldm_DependenceEntropy
wavelet_HLL_glcm_Idn
wavelet_LLH_gldm_DependenceEntropy
wavelet_HHL_gldm_DependenceEntropy
wavelet_HLL_glcm_Idmn
wavelet_LHL_glcm_Idn
wavelet_LLH_glcm_Idn
wavelet_HLH_glcm_Idn
original_glcm_Idn
wavelet_LLL_glcm_Idn
original_glcm_Imc2
wavelet_HLL_glcm_Imc2
wavelet_HLL_glrlm_LongRunEmphasis
wavelet_LHL_glcm_DifferenceEntropy
wavelet_LHL_glcm_SumEntropy
wavelet_LHL_glcm_JointEntropy
wavelet_LHL_firstorder_Entropy
wavelet_LHL_glrlm_LongRunEmphasis
wavelet_LHH_glrlm_RunLengthNonUniformityNormalized
wavelet_LLH_glrlm_LongRunEmphasis
original_glcm_JointEntropy
original_glcm_SumEntropy
original_firstorder_Entropy
wavelet_HHL_glcm_SumEntropy
wavelet_HHL_glcm_DifferenceEntropy
wavelet_HHL_glcm_JointEntropy

wavelet_HHL_firstorder_Entropy
wavelet_HLL_glszm_SmallAreaEmphasis
wavelet_HHL_glszm_ZoneEntropy
wavelet_HLH_glszm_ZoneEntropy
wavelet_HLH_glrlm_RunEntropy
wavelet_HLL_glcm_DifferenceEntropy
wavelet_LLH_glcm_Id
wavelet_HLH_glrlm_RunLengthNonUniformityNormalized
wavelet_LLH_glrlm_RunEntropy
wavelet_LLH_glcm_DifferenceEntropy
wavelet_LLH_firstorder_Entropy
wavelet_LLH_glcm_JointEntropy
wavelet_LLH_glcm_SumEntropy
wavelet_LHH_glrlm_RunEntropy
wavelet_LLH_glszm_ZoneEntropy
wavelet_LHL_glrlm_RunEntropy
wavelet_LHL_glszm_ZoneEntropy
wavelet_LHL_glcm_Imc2
wavelet_LLH_glcm_Imc2
wavelet_LHL_glszm_SmallAreaEmphasis
wavelet_HHL_glszm_SmallAreaEmphasis
wavelet_HLH_glcm_SumEntropy
wavelet_HLH_glcm_JointEntropy
wavelet_HLH_glcm_DifferenceEntropy
wavelet_HLH_firstorder_Entropy
wavelet_HLH_glcm_InverseVariance
wavelet_HLH_glcm_Id
original_shape_Sphericity
wavelet_HHL_glcm_Id
wavelet_HHL_glrlm_LongRunEmphasis
original_glszm_SmallAreaEmphasis
wavelet_LLL_glszm_SizeZoneNonUniformityNormalized
wavelet_LLH_glszm_SmallAreaEmphasis
wavelet_LLH_glcm_InverseVariance
wavelet_LLH_glcm_Idm
wavelet_HHH_glrlm_LongRunEmphasis
wavelet_LHH_glrlm_LongRunEmphasis
wavelet_LHH_glszm_ZoneEntropy
wavelet_LHH_glcm_Idm
wavelet_LHH_glcm_SumEntropy
wavelet_LHH_glcm_DifferenceEntropy
wavelet_LHH_glcm_JointEntropy
wavelet_LHH_firstorder_Entropy
wavelet_LLL_gldm_SmallDependenceEmphasis
wavelet_LLL_glszm_ZonePercentage

wavelet_HHH_glcm_SumEntropy
wavelet_HHH_glcm_DifferenceEntropy
wavelet_HHH_glcm_JointEntropy
wavelet_HHH_firstorder_Entropy
wavelet_LHL_glcm_Id
wavelet_LHH_glcm_InverseVariance
wavelet_LHH_glcm_Id
wavelet_HHH_glrlm_RunLengthNonUniformityNormalized
wavelet_HHH_glcm_Idm
wavelet_HHH_glcm_Id
wavelet_HHH_glszm_ZoneEntropy
wavelet_LHL_gldm_DependenceNonUniformityNormalized
original_shape_MinorAxis
wavelet_HHL_glcm_InverseVariance
wavelet_HHL_glcm_Idm
original_shape_SurfaceVolumeRatio
wavelet_HLL_gldm_SmallDependenceEmphasis
wavelet_HLL_glszm_ZonePercentage
wavelet_LLL_glcm_Imc1
wavelet_LHL_glcm_InverseVariance
wavelet_LHL_glcm_Idm
wavelet_HLH_glcm_Idm
wavelet_HLH_glrlm_LongRunEmphasis
wavelet_LHL_firstorder_Kurtosis
wavelet_HHH_gldm_DependenceVariance
wavelet_LHL_glcm_Correlation
wavelet_HHH_glszm_ZonePercentage
wavelet_HHH_gldm_SmallDependenceEmphasis
wavelet_HLH_glcm_Imc1
wavelet_HLL_firstorder_90Percentile
original_firstorder_Mean
wavelet_LLL_firstorder_Mean
wavelet_HLH_glszm_GrayLevelNonUniformityNormalized
wavelet_HLH_gldm_DependenceNonUniformityNormalized
wavelet_HLH_gldm_SmallDependenceEmphasis
wavelet_HLH_firstorder_10Percentile
original_glcm_InverseVariance
original_glcm_Idm
wavelet_HLH_firstorder_InterquartileRange
wavelet_HLH_firstorder_90Percentile
wavelet_HLH_firstorder_RootMeanSquared
wavelet_HLH_glcm_DifferenceAverage
wavelet_HLH_firstorder_MeanAbsoluteDeviation
wavelet_HLH_firstorder_RobustMeanAbsoluteDeviation
wavelet_HLH_firstorder_Uniformity

wavelet_HLH_glrlm_GrayLevelNonUniformityNormalized
wavelet_HLL_firstorder_10Percentile
original_glcm_Correlation
wavelet_LLL_glcm_Correlation
wavelet_LLL_firstorder_Kurtosis
original_shape_Flatness
wavelet_HLL_firstorder_RootMeanSquared
wavelet_HLL_glszm_GrayLevelNonUniformityNormalized
original_shape_Elongation
wavelet_HLL_glcm_Imc1
original_shape_Maximum2DDiameterRow
original_shape_Maximum3DDiameter
original_shape_MajorAxis
original_glszm_GrayLevelVariance
wavelet_HLL_gldm_DependenceNonUniformityNormalized
wavelet_HLL_glcm_Id
wavelet_HLL_glcm_InverseVariance
wavelet_HLL_glcm_Idm
wavelet_HLL_firstorder_Uniformity
wavelet_HLL_glrlm_GrayLevelNonUniformityNormalized
wavelet_HLL_glcm_DifferenceAverage
wavelet_HLL_firstorder_MeanAbsoluteDeviation
wavelet_LLH_glcm_Correlation
wavelet_LLH_glcm_Imc1
wavelet_LLL_glcm_JointAverage
wavelet_LLL_glcm_SumAverage
wavelet_LHL_glszm_SizeZoneNonUniformityNormalized
wavelet_HHH_glszm_SmallAreaEmphasis
wavelet_LHH_gldm_DependenceNonUniformityNormalized
original_shape_LeastAxis
original_glcm_Imc1
wavelet_HHH_glcm_MaximumProbability
wavelet_HHH_firstorder_Uniformity
wavelet_HHH_glrlm_GrayLevelNonUniformityNormalized
wavelet_HHH_glcm_DifferenceAverage
wavelet_HHH_firstorder_MeanAbsoluteDeviation
wavelet_HHH_firstorder_RootMeanSquared
wavelet_HHH_firstorder_90Percentile
wavelet_HHH_firstorder_10Percentile
wavelet_HHH_firstorder_InterquartileRange
wavelet_HHH_firstorder_RobustMeanAbsoluteDeviation
wavelet_LLL_gldm_DependenceNonUniformityNormalized
wavelet_LHL_gldm_SmallDependenceEmphasis
wavelet_LHL_glszm_ZonePercentage
wavelet_LHL_glszm_GrayLevelNonUniformityNormalized

wavelet_LHL_firstorder_InterquartileRange
wavelet_LHL_glcm_DifferenceAverage
wavelet_LHL_firstorder_10Percentile
wavelet_LHL_firstorder_MeanAbsoluteDeviation
wavelet_LHL_firstorder_RootMeanSquared
wavelet_LHL_firstorder_RobustMeanAbsoluteDeviation
wavelet_LHL_firstorder_Uniformity
wavelet_LHL_glrlm_GrayLevelNonUniformityNormalized
wavelet_LHH_firstorder_10Percentile
wavelet_LHH_firstorder_90Percentile
wavelet_LHH_firstorder_InterquartileRange
wavelet_LHH_firstorder_RobustMeanAbsoluteDeviation
wavelet_LHH_firstorder_Uniformity
wavelet_LHH_glrlm_GrayLevelNonUniformityNormalized
wavelet_LHH_firstorder_RootMeanSquared
wavelet_LHH_glcm_DifferenceAverage
wavelet_LHH_firstorder_MeanAbsoluteDeviation
original_firstorder_Range
wavelet_LLL_firstorder_Range
original_shape_Maximum2DDiameterColumn
wavelet_HHL_gldm_DependenceNonUniformityNormalized
wavelet_HHL_firstorder_InterquartileRange
wavelet_HHL_firstorder_RobustMeanAbsoluteDeviation
wavelet_HHL_firstorder_90Percentile
wavelet_HHL_firstorder_10Percentile
wavelet_HHL_firstorder_RootMeanSquared
wavelet_HHL_firstorder_Uniformity
wavelet_HHL_glrlm_GrayLevelNonUniformityNormalized
wavelet_HHL_glcm_DifferenceAverage
wavelet_HHL_firstorder_MeanAbsoluteDeviation
wavelet_HHL_glcm_Imc2
wavelet_HHL_glszm_GrayLevelNonUniformityNormalized
original_gldm_DependenceNonUniformityNormalized
original_glszm_GrayLevelNonUniformityNormalized
wavelet_LLL_glszm_GrayLevelNonUniformityNormalized
wavelet_LLL_glcm_DifferenceAverage
original_glcm_DifferenceAverage
original_firstorder_MeanAbsoluteDeviation
wavelet_LLL_firstorder_MeanAbsoluteDeviation
original_glszm_SizeZoneNonUniformityNormalized
wavelet_LLH_glszm_SizeZoneNonUniformityNormalized
wavelet_HLH_glcm_Imc2
wavelet_LLH_gldm_SmallDependenceEmphasis
wavelet_LLH_glszm_ZonePercentage
original_glcm_Id

wavelet_LLH_glcm_DifferenceAverage
wavelet_LLH_gldm_DependenceNonUniformityNormalized
original_gldm_SmallDependenceEmphasis
original_glszm_ZonePercentage
wavelet_LLL_glcm_Id
wavelet_LLH_firstorder_Uniformity
wavelet_LLH_glrlm_GrayLevelNonUniformityNormalized
wavelet_LLH_firstorder_InterquartileRange
wavelet_LLH_firstorder_RobustMeanAbsoluteDeviation
wavelet_LLH_glszm_GrayLevelNonUniformityNormalized
wavelet_LLH_firstorder_10Percentile
wavelet_LLH_firstorder_MeanAbsoluteDeviation
wavelet_LLH_firstorder_RootMeanSquared
original_shape_Maximum2DDiameterSlice
wavelet_HLH_glszm_SmallAreaEmphasis
wavelet_HLL_glszm_SizeZoneNonUniformityNormalized
wavelet_LHH_glszm_SmallAreaEmphasis
wavelet_LLL_firstorder_Minimum
original_firstorder_Minimum
original_glcm_JointAverage
original_glcm_SumAverage
original_firstorder_RootMeanSquared
wavelet_LLL_firstorder_RootMeanSquared
original_firstorder_10Percentile
wavelet_LLL_firstorder_10Percentile
original_ngtdm_Coarseness
wavelet_LLL_ngtdm_Coarseness
original_gldm_DependenceNonUniformity
original_glszm_SizeZoneNonUniformity
wavelet_HLH_glszm_GrayLevelNonUniformity
wavelet_HLL_gldm_DependenceNonUniformity
wavelet_HLL_glszm_SizeZoneNonUniformity
wavelet_HHL_glszm_GrayLevelNonUniformity
wavelet_HHL_gldm_DependenceNonUniformity
wavelet_LLH_glszm_GrayLevelNonUniformity
wavelet_HLH_gldm_DependenceNonUniformity
wavelet_HHL_gldm_GrayLevelNonUniformity
wavelet_HHL_glrlm_GrayLevelNonUniformity
wavelet_LLH_gldm_DependenceNonUniformity
wavelet_LLL_gldm_DependenceNonUniformity
wavelet_LLL_glszm_SizeZoneNonUniformity
wavelet_HLL_glszm_GrayLevelNonUniformity
wavelet_LHL_gldm_GrayLevelNonUniformity
wavelet_LHL_glrlm_GrayLevelNonUniformity
wavelet_LLH_gldm_GrayLevelNonUniformity

wavelet_LLH_glrlm_GrayLevelNonUniformity
wavelet_LHH_gldm_GrayLevelNonUniformity
wavelet_LHH_glrlm_GrayLevelNonUniformity
original_glszm_GrayLevelNonUniformity
original_shape_SurfaceArea
wavelet_LLL_glszm_GrayLevelNonUniformity
wavelet_LHL_gldm_DependenceNonUniformity
wavelet_LHH_glrlm_RunLengthNonUniformity
wavelet_HHH_gldm_DependenceNonUniformity
wavelet_HHH_glrlm_RunLengthNonUniformity
wavelet_HHH_ngtdm_Coarseness
wavelet_LHL_ngtdm_Coarseness
wavelet_HLH_glrlm_RunLengthNonUniformity
wavelet_HHH_glrlm_GrayLevelNonUniformity
wavelet_HHL_glrlm_RunLengthNonUniformity
wavelet_LHL_glrlm_RunLengthNonUniformity
original_shape_Volume
wavelet_HLL_glrlm_RunLengthNonUniformity
wavelet_LLL_glrlm_RunLengthNonUniformity
original_glrlm_RunLengthNonUniformity
wavelet_LLH_glrlm_RunLengthNonUniformity
wavelet_LLH_ngtdm_Coarseness
wavelet_LHH_ngtdm_Coarseness
wavelet_HHL_ngtdm_Coarseness
wavelet_HHH_gldm_GrayLevelNonUniformity
wavelet_HLH_ngtdm_Coarseness
wavelet_LHH_gldm_DependenceNonUniformity
wavelet_HLL_ngtdm_Coarseness
wavelet_HLH_gldm_GrayLevelNonUniformity
wavelet_HLH_glrlm_GrayLevelNonUniformity
wavelet_LLL_gldm_GrayLevelNonUniformity
wavelet_LLL_glrlm_GrayLevelNonUniformity
original_gldm_GrayLevelNonUniformity
original_glrlm_GrayLevelNonUniformity
wavelet_HLL_gldm_GrayLevelNonUniformity
wavelet_HLL_glrlm_GrayLevelNonUniformity
wavelet_HLL_firstorder_Kurtosis
wavelet_HLL_firstorder_InterquartileRange
wavelet_HLL_firstorder_RobustMeanAbsoluteDeviation
wavelet_LHH_glszm_GrayLevelNonUniformityNormalized
wavelet_LHH_gldm_SmallDependenceEmphasis
wavelet_LHH_glszm_ZonePercentage
wavelet_HHH_glcm_Imc2
wavelet_HHH_glszm_GrayLevelVariance
wavelet_HHH_firstorder_Variance

wavelet_HHH_glcm_JointEnergy
wavelet_HHH_glcm_DifferenceVariance
wavelet_HHH_glcm_ClusterTendency
wavelet_HHH_glcm_Contrast
wavelet_HHH_glcm_SumSquares
wavelet_HHH_gldm_GrayLevelVariance
wavelet_HHH_glrlm_GrayLevelVariance
wavelet_LHL_glcm_JointEnergy
wavelet_LHL_gldm_LargeDependenceEmphasis
wavelet_LHL_glrlm_RunVariance
wavelet_HHL_glszm_SizeZoneNonUniformityNormalized
wavelet_HHL_glcm_Imc1
wavelet_HHL_gldm_SmallDependenceEmphasis
wavelet_HHL_glszm_ZonePercentage
wavelet_HLH_glszm_SizeZoneNonUniformityNormalized
wavelet_LHH_glszm_SizeZoneNonUniformityNormalized
wavelet_LHH_glszm_GrayLevelNonUniformity
original_firstorder_Uniformity
original_glrlm_GrayLevelNonUniformityNormalized
wavelet_LLH_firstorder_90Percentile
wavelet_LLH_glcm_JointEnergy
wavelet_LLL_glcm_InverseVariance
wavelet_LLL_glcm_Idm
wavelet_LLH_gldm_LargeDependenceEmphasis
wavelet_LLH_glrlm_RunVariance
original_firstorder_Kurtosis
original_firstorder_InterquartileRange
original_firstorder_RobustMeanAbsoluteDeviation
wavelet_LLL_firstorder_InterquartileRange
wavelet_LLL_firstorder_RobustMeanAbsoluteDeviation
wavelet_HLL_firstorder_Range
wavelet_LHL_firstorder_90Percentile
wavelet_LHL_glcm_Imc1
wavelet_LHH_glcm_Imc2
wavelet_LLL_firstorder_Uniformity
wavelet_LLL_glrlm_GrayLevelNonUniformityNormalized
wavelet_LHL_glszm_GrayLevelNonUniformity
wavelet_HHH_gldm_LargeDependenceEmphasis
wavelet_HHH_glrlm_RunVariance

Cluster (Right Mixed)

original_firstorder_Median
wavelet_LLL_glcm_JointEnergy
wavelet_LLL_firstorder_Median

wavelet_HHH_firstorder_Kurtosis
wavelet_HHH_firstorder_Minimum
wavelet_HHH_firstorder_Range
wavelet_HHH_glcm_JointAverage
wavelet_HHH_glcm_SumAverage
wavelet_LLH_firstorder_Kurtosis
wavelet_LLH_firstorder_Minimum
wavelet_LLH_glcm_JointAverage
wavelet_LLH_glcm_SumAverage
wavelet_HHH_glcm_Correlation
original_gldm_LargeDependenceEmphasis
original_glrlm_RunVariance
wavelet_HLL_ngtdm_Contrast
wavelet_HLL_glcm_Correlation
wavelet_HLH_glszm_SizeZoneNonUniformity
wavelet_HLL_glcm_Contrast
wavelet_HLL_glcm_DifferenceVariance
wavelet_LLL_gldm_LargeDependenceEmphasis
wavelet_LLL_glrlm_RunVariance
wavelet_LLH_gldm_DependenceVariance
wavelet_LLH_glcm_MaximumProbability
wavelet_LLH_ngtdm_Contrast
wavelet_LHL_gldm_DependenceVariance
wavelet_LHL_glcm_MaximumProbability
wavelet_HHH_glcm_Imc1
wavelet_HHL_glszm_SizeZoneNonUniformity
wavelet_HHL_firstorder_Energy
wavelet_HHL_firstorder_TotalEnergy
wavelet_LHH_glszm_GrayLevelVariance
wavelet_LHH_firstorder_Energy
wavelet_LHH_firstorder_TotalEnergy
wavelet_LHH_glcm_DifferenceVariance
wavelet_LHH_glcm_Contrast
wavelet_LHH_glcm_SumSquares
wavelet_LHH_glcm_ClusterTendency
wavelet_LHH_firstorder_Variance
wavelet_LHH_gldm_GrayLevelVariance
wavelet_LHH_glrlm_GrayLevelVariance
wavelet_LHL_firstorder_Energy
wavelet_LHL_firstorder_TotalEnergy
wavelet_LHL_firstorder_Maximum
wavelet_LHL_glszm_SizeZoneNonUniformity
wavelet_LHL_glszm_GrayLevelVariance
wavelet_LHL_glrlm_GrayLevelVariance
wavelet_LHL_gldm_GrayLevelVariance

wavelet_LHL_firstorder_Variance
wavelet_LHL_glcm_SumSquares
wavelet_LHL_glcm_ClusterTendency
wavelet_LHL_glcm_Contrast
wavelet_LHL_glcm_DifferenceVariance
wavelet_HLL_glcm_JointEnergy
wavelet_HLL_gldm_LargeDependenceEmphasis
wavelet_HLL_glrlm_RunVariance
wavelet_LHL_firstorder_Minimum
wavelet_LHL_glcm_JointAverage
wavelet_LHL_glcm_SumAverage
wavelet_HLL_firstorder_Maximum
wavelet_LHL_firstorder_Range
wavelet_LHL_ngtdm_Contrast
wavelet_HHL_firstorder_Minimum
wavelet_HHL_glcm_JointAverage
wavelet_HHL_glcm_SumAverage
wavelet_HLH_firstorder_Maximum
wavelet_HLH_firstorder_Minimum
wavelet_HLH_glcm_JointAverage
wavelet_HLH_glcm_SumAverage
wavelet_HLH_firstorder_Range
wavelet_HLH_firstorder_Kurtosis
wavelet_HLL_firstorder_Minimum
wavelet_HLL_glcm_JointAverage
wavelet_HLL_glcm_SumAverage
wavelet_HHH_glszm_SizeZoneNonUniformityNormalized
wavelet_LHH_firstorder_Kurtosis
wavelet_LHH_glcm_Imc1
wavelet_LHH_firstorder_Range
wavelet_LLH_firstorder_Variance
wavelet_LLH_gldm_GrayLevelVariance
wavelet_LLH_glrlm_GrayLevelVariance
wavelet_LLH_glcm_SumSquares
wavelet_LLH_glcm_ClusterTendency
wavelet_LLH_glcm_Contrast
wavelet_LLH_glcm_DifferenceVariance
wavelet_LLH_glszm_SizeZoneNonUniformity
wavelet_LLH_firstorder_Energy
wavelet_LLH_firstorder_TotalEnergy
wavelet_LLH_firstorder_Median
original_ngtdm_Complexity
wavelet_LLL_ngtdm_Busyness
wavelet_LHL_firstorder_Mean
wavelet_LLL_ngtdm_Strength

original_gldm_SmallDependenceHighGrayLevelEmphasis
original_glszm_SmallAreaHighGrayLevelEmphasis
original_glszm_HighGrayLevelZoneEmphasis
original_glrlm_LongRunHighGrayLevelEmphasis
original_glrlm_ShortRunHighGrayLevelEmphasis
original_glcm_Autocorrelation
original_gldm_HighGrayLevelEmphasis
original_glrlm_HighGrayLevelRunEmphasis
original_ngtdm_Strength
original_ngtdm_Busyness
wavelet_LLH_firstorder_Mean
wavelet_LLH_firstorder_Range
wavelet_LLH_glszm_GrayLevelVariance
wavelet_LLL_gldm_SmallDependenceHighGrayLevelEmphasis
wavelet_LLL_glszm_SmallAreaHighGrayLevelEmphasis
wavelet_LLL_glszm_HighGrayLevelZoneEmphasis
wavelet_LLL_glrlm_LongRunHighGrayLevelEmphasis
wavelet_LLL_glcm_Autocorrelation
wavelet_LLL_glrlm_ShortRunHighGrayLevelEmphasis
wavelet_LLL_gldm_HighGrayLevelEmphasis
wavelet_LLL_glrlm_HighGrayLevelRunEmphasis
original_ngtdm_Contrast
wavelet_LLL_ngtdm_Contrast
wavelet_HLH_firstorder_Energy
wavelet_HLH_firstorder_TotalEnergy
wavelet_HLH_glszm_GrayLevelVariance
wavelet_HLH_glszm_ZonePercentage
wavelet_HLH_glcm_Contrast
wavelet_HLH_glcm_SumSquares
wavelet_HLH_glcm_ClusterTendency
wavelet_HLH_glcm_DifferenceVariance
wavelet_HLH_glrlm_GrayLevelVariance
wavelet_HLH_gldm_GrayLevelVariance
wavelet_HLH_firstorder_Variance
wavelet_HLH_gldm_DependenceVariance
wavelet_HLH_glcm_JointEnergy
wavelet_HLH_glcm_MaximumProbability
wavelet_HLH_gldm_LargeDependenceEmphasis
wavelet_HLH_glrlm_RunVariance
wavelet_HLL_glszm_GrayLevelVariance
wavelet_HLL_firstorder_Energy
wavelet_HLL_firstorder_TotalEnergy
wavelet_HLL_glrlm_GrayLevelVariance
wavelet_HLL_gldm_GrayLevelVariance
wavelet_HLL_firstorder_Variance

wavelet_HLL_glcm_SumSquares
wavelet_HLL_glcm_ClusterTendency
wavelet_LLL_glcm_ClusterTendency
original_glcm_SumSquares
original_glcm_ClusterTendency
wavelet_LLL_ngtdm_Complexity
wavelet_LLL_glszm_GrayLevelVariance
original_glrlm_GrayLevelVariance
original_gldm_GrayLevelVariance
original_firstorder_Variance
wavelet_LLL_glcm_SumSquares
wavelet_LLL_glrlm_GrayLevelVariance
wavelet_LLL_gldm_GrayLevelVariance
wavelet_LLL_firstorder_Variance
original_glcm_Contrast
original_glcm_DifferenceVariance
wavelet_LLL_glcm_Contrast
wavelet_LLL_glcm_DifferenceVariance
wavelet_HHL_gldm_DependenceVariance
wavelet_HHL_glcm_MaximumProbability
wavelet_HHL_glcm_JointEnergy
wavelet_HHL_gldm_LargeDependenceEmphasis
wavelet_HHL_glrlm_RunVariance
wavelet_HHL_glszm_GrayLevelVariance
wavelet_HHL_glcm_DifferenceVariance
wavelet_HHL_glcm_Contrast
wavelet_HHL_glcm_SumSquares
wavelet_HHL_glcm_ClusterTendency
wavelet_HHL_firstorder_Variance
wavelet_HHL_gldm_GrayLevelVariance
wavelet_HHL_glrlm_GrayLevelVariance
wavelet_HHL_firstorder_Range
wavelet_HHL_firstorder_Kurtosis
wavelet_LHH_glcm_JointEnergy
wavelet_LHH_gldm_DependenceVariance
wavelet_HHH_glszm_GrayLevelNonUniformityNormalized
wavelet_LHH_glrlm_RunVariance
wavelet_LHH_gldm_LargeDependenceEmphasis
wavelet_LHH_glcm_MaximumProbability
