# Supplementary material for: Radiomic feature stability across 4D respiratory phases and its impact on lung tumor prognosis prediction
Source: PLoS One. 2019 May 7;14(5):e0216480. doi: 10.1371/journal.pone.0216480 (PMC6504105; doi:10.1371/journal.pone.0216480)
Supplement: S3 File — (PDF) [file pone.0216480.s003.pdf]

Supplementary File 3: List of all feature data

Image type	Feature Class	Feature Name	11: RTSTRUCT: C99-DNU_segment_ITV-LUL
general	info	BoundingBox	(0, 1, 1, 7, 6, 5)
general	info	EnabledImageTypes	{u'Original': {}, u'Wavelet': {}}
general	info	GeneralSettings	{'distances': [1], 'additionalInfo': True, 'force2D': False, 'interpolator': 'sitkBSpline', 'resampledPixelSpacing': [3.0], 'label': 1, 'normalizeScale': 1, 'normalize': False, 'force2Ddimension': 0, 'removeOutliers': None, 'minimumROISize': None, u'binWidth': 25.0, 'minimumROIDimensions': 1, u'correctMask': True, 'preCrop': False, u'symmetricalGLCM': True, 'resegmentRange': None, 'padDistance': 5}
general	info	ImageHash	13441ddb8b7f5355e5cf424418097e0e8466ade8
general	info	ImageSpacing	(0.9765625000000002, 0.9765625000000002, 2.0)
general	info	MaskHash	510484d399fbebe2145cd5dfa62bb28cf8603e15
general	info	NumpyVersion	1.13.1
general	info	PyWaveletVersion	0.5.2
general	info	SimpleITKVersion	1.1.0
general	info	Version	1.3.0.post102+g1b0eb74
general	info	VolumeNum	1
general	info	VoxelNum	97
original	shape	Maximum3DDiameter	20.34698995
original	shape	Maximum2DDiameterSlice	18.97366596
original	shape	Sphericity	0.846749749
original	shape	MinorAxis	16.17708417
original	shape	Elongation	0.839009922
original	shape	SurfaceVolumeRatio	0.414333977
original	shape	Volume	2619
original	shape	MajorAxis	19.28115955
original	shape	SurfaceArea	1085.140686
original	shape	Flatness	0.662809184
original	shape	LeastAxis	12.77972962
original	shape	Maximum2DDiameterColumn	19.20937271
original	shape	Maximum2DDiameterRow	17.49285568
original	gldm	GrayLevelVariance	100.4349028
original	gldm	HighGrayLevelEmphasis	519.628866
original	gldm	DependenceEntropy	5.496618512
original	gldm	DependenceNonUniformity	42.15463918
original	gldm	GrayLevelNonUniformity	3.865979381
original	gldm	SmallDependenceEmphasis	0.682701802
original	gldm	SmallDependenceHighGrayLevelEmphasis	253.4345178
original	gldm	DependenceNonUniformityNormalized	0.434583909
original	gldm	LargeDependenceEmphasis	4.525773196
original	gldm	LargeDependenceLowGrayLevelEmphasis	0.032447205
original	gldm	DependenceVariance	1.490275268
original	gldm	LargeDependenceHighGrayLevelEmphasis	4152.082474
original	gldm	SmallDependenceLowGrayLevelEmphasis	0.027197922
original	gldm	LowGrayLevelEmphasis	0.027810052
original	glcm	JointAverage	23.26080762
original	glcm	SumAverage	46.52161523

original	glcm	JointEntropy	6.602887119
original	glcm	ClusterShade	-1026.389778
original	glcm	MaximumProbability	0.027240005
original	glcm	Idmn	0.905618795
original	glcm	JointEnergy	0.011127664
original	glcm	Contrast	158.9469443
original	glcm	DifferenceEntropy	4.237238526
original	glcm	InverseVariance	0.129852614
original	glcm	DifferenceVariance	59.87710115
original	glcm	Idn	0.806996059
original	glcm	Idm	0.124776724
original	glcm	Correlation	0.203276077
original	glcm	Autocorrelation	561.2335003
original	glcm	SumEntropy	5.013575556
original	glcm	SumSquares	99.40605072
original	glcm	ClusterProminence	125526.7419
original	glcm	Imc2	0.997697742
original	glcm	Imc1	-0.580989124
original	glcm	DifferenceAverage	9.85318638
original	glcm	Id	0.201413977
original	glcm	ClusterTendency	238.6772586
original	firstorder	InterquartileRange	437
original	firstorder	Skewness	-0.044193001
original	firstorder	Uniformity	0.039855458
original	firstorder	Median	-361
original	firstorder	Energy	16385078
original	firstorder	RobustMeanAbsoluteDeviation	173.8684432
original	firstorder	MeanAbsoluteDeviation	216.1511319
original	firstorder	TotalEnergy	442397106
original	firstorder	Maximum	56
original	firstorder	RootMeanSquared	410.9967517
original	firstorder	90Percentile	20.2
original	firstorder	Minimum	-815
original	firstorder	Entropy	4.837091002
original	firstorder	Range	871
original	firstorder	Variance	62467.49516
original	firstorder	10Percentile	-652.6
original	firstorder	Kurtosis	1.8165423
original	firstorder	Mean	-326.2680412
original	glrlm	ShortRunLowGrayLevelEmphasis	0.028564966
original	glrlm	GrayLevelVariance	98.9942949
original	glrlm	LowGrayLevelRunEmphasis	0.028596092
original	glrlm	GrayLevelNonUniformityNormalized	0.039012984
original	glrlm	RunVariance	0.031854639
original	glrlm	GrayLevelNonUniformity	3.67677289
original	glrlm	LongRunEmphasis	1.093190844
original	glrlm	ShortRunHighGrayLevelEmphasis	485.545775
original	glrlm	RunLengthNonUniformity	89.21100424
original	glrlm	ShortRunEmphasis	0.978592573
original	glrlm	LongRunHighGrayLevelEmphasis	594.8858686
original	glrlm	RunPercentage	0.971451229
original	glrlm	LongRunLowGrayLevelEmphasis	0.028746765
original	glrlm	RunEntropy	4.936245325
original	glrlm	HighGrayLevelRunEmphasis	506.4325587
original	glrlm	RunLengthNonUniformityNormalized	0.94586137

original	glszm	GrayLevelVariance	83.41184414
original	glszm	ZoneVariance	1.11554784
original	glszm	GrayLevelNonUniformityNormalized	0.038580247
original	glszm	SizeZoneNonUniformityNormalized	0.705246914
original	glszm	SizeZoneNonUniformity	50.77777778
original	glszm	GrayLevelNonUniformity	2.777777778
original	glszm	LargeAreaEmphasis	2.930555556
original	glszm	SmallAreaHighGrayLevelEmphasis	299.3736497
original	glszm	ZonePercentage	0.742268041
original	glszm	LargeAreaLowGrayLevelEmphasis	0.039650779
original	glszm	LargeAreaHighGrayLevelEmphasis	2301.666667
original	glszm	HighGrayLevelZoneEmphasis	396.0138889
original	glszm	SmallAreaEmphasis	0.862365934
original	glszm	LowGrayLevelZoneEmphasis	0.036832719
original	glszm	ZoneEntropy	5.110693578
original	glszm	SmallAreaLowGrayLevelEmphasis	0.036508883
original	ngtdm	Coarseness	0.036433476
original	ngtdm	Complexity	3536.953427
original	ngtdm	Strength	20.30119245
original	ngtdm	Contrast	1.288494025
original	ngtdm	Busyness	0.041594592
wavelet-HHH	gldm	GrayLevelVariance	5.858220852
wavelet-HHH	gldm	HighGrayLevelEmphasis	36.16494845
wavelet-HHH	gldm	DependenceEntropy	5.041843936
wavelet-HHH	gldm	DependenceNonUniformity	20.3814433
wavelet-HHH	gldm	GrayLevelNonUniformity	11.45360825
wavelet-HHH	gldm	SmallDependenceEmphasis	0.338708126
wavelet-HHH	gldm	SmallDependenceHighGrayLevelEmphasis	16.46649116
wavelet-HHH	gldm	DependenceNonUniformityNormalized	0.210117972
wavelet-HHH	gldm	LargeDependenceEmphasis	10.07216495
wavelet-HHH	gldm	LargeDependenceLowGrayLevelEmphasis	0.671372686
wavelet-HHH	gldm	DependenceVariance	2.38154958
wavelet-HHH	gldm	LargeDependenceHighGrayLevelEmphasis	288.6597938
wavelet-HHH	gldm	SmallDependenceLowGrayLevelEmphasis	0.045176147
wavelet-HHH	gldm	LowGrayLevelEmphasis	0.11084716
wavelet-HHH	glcm	JointAverage	5.463783872
wavelet-HHH	glcm	SumAverage	10.92756774
wavelet-HHH	glcm	JointEntropy	5.723762733
wavelet-HHH	glcm	ClusterShade	6.107989548
wavelet-HHH	glcm	MaximumProbability	0.047347194
wavelet-HHH	glcm	Idmn	0.92699797
wavelet-HHH	glcm	JointEnergy	0.022166676
wavelet-HHH	glcm	Contrast	12.94841198
wavelet-HHH	glcm	DifferenceEntropy	2.831868009
wavelet-HHH	glcm	InverseVariance	0.294573541
wavelet-HHH	glcm	DifferenceVariance	4.584006088
wavelet-HHH	glcm	Idn	0.82475134
wavelet-HHH	glcm	Idm	0.289680156
wavelet-HHH	glcm	Correlation	-0.027223539
wavelet-HHH	glcm	Autocorrelation	29.68691258
wavelet-HHH	glcm	SumEntropy	3.582645787
wavelet-HHH	glcm	SumSquares	6.302391826
wavelet-HHH	glcm	ClusterProminence	444.4833422
wavelet-HHH	glcm	Imc2	0.886394285
wavelet-HHH	glcm	Imc1	-0.241390898

wavelet-HHH	glcm	DifferenceAverage	2.847931369
wavelet-HHH	glcm	Id	0.377698619
wavelet-HHH	glcm	ClusterTendency	12.26115533
wavelet-HHH	firstorder	InterquartileRange	82.2214901
wavelet-HHH	firstorder	Skewness	0.079710856
wavelet-HHH	firstorder	Uniformity	0.118078436
wavelet-HHH	firstorder	Median	-2.494598284
wavelet-HHH	firstorder	Energy	361974.1236
wavelet-HHH	firstorder	RobustMeanAbsoluteDeviation	34.26159143
wavelet-HHH	firstorder	MeanAbsoluteDeviation	49.02167823
wavelet-HHH	firstorder	TotalEnergy	9773301.337
wavelet-HHH	firstorder	Maximum	156.070323
wavelet-HHH	firstorder	RootMeanSquared	61.08757644
wavelet-HHH	firstorder	90Percentile	72.18066914
wavelet-HHH	firstorder	Minimum	-122.9512672
wavelet-HHH	firstorder	Entropy	3.239957914
wavelet-HHH	firstorder	Range	279.0215903
wavelet-HHH	firstorder	Variance	3731.594561
wavelet-HHH	firstorder	10Percentile	-83.31122652
wavelet-HHH	firstorder	Kurtosis	2.767126685
wavelet-HHH	firstorder	Mean	0.312145255
wavelet-HHH	glrlm	ShortRunLowGrayLevelEmphasis	0.109758916
wavelet-HHH	glrlm	GrayLevelVariance	6.079350711
wavelet-HHH	glrlm	LowGrayLevelRunEmphasis	0.113651266
wavelet-HHH	glrlm	GrayLevelNonUniformityNormalized	0.115991848
wavelet-HHH	glrlm	RunVariance	0.074520833
wavelet-HHH	glrlm	GrayLevelNonUniformity	10.48414332
wavelet-HHH	glrlm	LongRunEmphasis	1.228393749
wavelet-HHH	glrlm	ShortRunHighGrayLevelEmphasis	35.13897476
wavelet-HHH	glrlm	RunLengthNonUniformity	78.75845136
wavelet-HHH	glrlm	ShortRunEmphasis	0.946691197
wavelet-HHH	glrlm	LongRunHighGrayLevelEmphasis	43.2122206
wavelet-HHH	glrlm	RunPercentage	0.931800159
wavelet-HHH	glrlm	LongRunLowGrayLevelEmphasis	0.130273315
wavelet-HHH	glrlm	RunEntropy	3.544362028
wavelet-HHH	glrlm	HighGrayLevelRunEmphasis	36.70386665
wavelet-HHH	glrlm	RunLengthNonUniformityNormalized	0.870210513
wavelet-HHH	glszm	GrayLevelVariance	9.204470743
wavelet-HHH	glszm	ZoneVariance	8.813938199
wavelet-HHH	glszm	GrayLevelNonUniformityNormalized	0.111111111
wavelet-HHH	glszm	SizeZoneNonUniformityNormalized	0.392504931
wavelet-HHH	glszm	SizeZoneNonUniformity	15.30769231
wavelet-HHH	glszm	GrayLevelNonUniformity	4.333333333
wavelet-HHH	glszm	LargeAreaEmphasis	15
wavelet-HHH	glszm	SmallAreaHighGrayLevelEmphasis	34.98857128
wavelet-HHH	glszm	ZonePercentage	0.402061856
wavelet-HHH	glszm	LargeAreaLowGrayLevelEmphasis	0.902259891
wavelet-HHH	glszm	LargeAreaHighGrayLevelEmphasis	483.1282051
wavelet-HHH	glszm	HighGrayLevelZoneEmphasis	44.8974359
wavelet-HHH	glszm	SmallAreaEmphasis	0.646895324
wavelet-HHH	glszm	LowGrayLevelZoneEmphasis	0.14854193
wavelet-HHH	glszm	ZoneEntropy	4.404260476
wavelet-HHH	glszm	SmallAreaLowGrayLevelEmphasis	0.088106412
wavelet-HHH	ngtdm	Coarseness	0.054302131
wavelet-HHH	ngtdm	Complexity	112.600775

wavelet-HHH	ngtdm	Strength	2.230602818
wavelet-HHH	ngtdm	Contrast	0.184747465
wavelet-HHH	ngtdm	Busyness	0.333514174
wavelet-HLL	gldm	GrayLevelVariance	77.72536933
wavelet-HLL	gldm	HighGrayLevelEmphasis	372.3608247
wavelet-HLL	gldm	DependenceEntropy	5.586875084
wavelet-HLL	gldm	DependenceNonUniformity	36.31958763
wavelet-HLL	gldm	GrayLevelNonUniformity	3.804123711
wavelet-HLL	gldm	SmallDependenceEmphasis	0.594716495
wavelet-HLL	gldm	SmallDependenceHighGrayLevelEmphasis	261.2484966
wavelet-HLL	gldm	DependenceNonUniformityNormalized	0.374428738
wavelet-HLL	gldm	LargeDependenceEmphasis	3.845360825
wavelet-HLL	gldm	LargeDependenceLowGrayLevelEmphasis	0.063393053
wavelet-HLL	gldm	DependenceVariance	0.809862897
wavelet-HLL	gldm	LargeDependenceHighGrayLevelEmphasis	932.5876289
wavelet-HLL	gldm	SmallDependenceLowGrayLevelEmphasis	0.019008563
wavelet-HLL	gldm	LowGrayLevelEmphasis	0.02381652
wavelet-HLL	glcm	JointAverage	15.89084836
wavelet-HLL	glcm	SumAverage	31.78169673
wavelet-HLL	glcm	JointEntropy	6.611299698
wavelet-HLL	glcm	ClusterShade	997.2611855
wavelet-HLL	glcm	MaximumProbability	0.021623228
wavelet-HLL	glcm	Idmn	0.932790545
wavelet-HLL	glcm	JointEnergy	0.010848768
wavelet-HLL	glcm	Contrast	97.62609071
wavelet-HLL	glcm	DifferenceEntropy	3.895785224
wavelet-HLL	glcm	InverseVariance	0.144282216
wavelet-HLL	glcm	DifferenceVariance	37.1758853
wavelet-HLL	glcm	Idn	0.836092669
wavelet-HLL	glcm	Idm	0.136060521
wavelet-HLL	glcm	Correlation	0.371779182
wavelet-HLL	glcm	Autocorrelation	282.8399359
wavelet-HLL	glcm	SumEntropy	4.964577356
wavelet-HLL	glcm	SumSquares	78.94409583
wavelet-HLL	glcm	ClusterProminence	107412.6049
wavelet-HLL	glcm	Imc2	0.998054898
wavelet-HLL	glcm	Imc1	-0.594099053
wavelet-HLL	glcm	DifferenceAverage	7.527066116
wavelet-HLL	glcm	Id	0.223933772
wavelet-HLL	glcm	ClusterTendency	218.1502926
wavelet-HLL	firstorder	InterquartileRange	322.6237991
wavelet-HLL	firstorder	Skewness	0.18539487
wavelet-HLL	firstorder	Uniformity	0.03921777
wavelet-HLL	firstorder	Median	-101.1848328
wavelet-HLL	firstorder	Energy	5299933.742
wavelet-HLL	firstorder	RobustMeanAbsoluteDeviation	142.5581464
wavelet-HLL	firstorder	MeanAbsoluteDeviation	185.1078684
wavelet-HLL	firstorder	TotalEnergy	143098211
wavelet-HLL	firstorder	Maximum	338.5350391
wavelet-HLL	firstorder	RootMeanSquared	233.7487801
wavelet-HLL	firstorder	90Percentile	221.4411504
wavelet-HLL	firstorder	Minimum	-475.9200324
wavelet-HLL	firstorder	Entropy	4.836687909
wavelet-HLL	firstorder	Range	814.4550715
wavelet-HLL	firstorder	Variance	47704.93587

wavelet-HLL	firstorder	10Percentile	-363.7216081
wavelet-HLL	firstorder	Kurtosis	1.979360103
wavelet-HLL	firstorder	Mean	-83.26797894
wavelet-HLL	glrlm	ShortRunLowGrayLevelEmphasis	0.023844508
wavelet-HLL	glrlm	GrayLevelVariance	77.90380351
wavelet-HLL	glrlm	LowGrayLevelRunEmphasis	0.024136669
wavelet-HLL	glrlm	GrayLevelNonUniformityNormalized	0.0389489
wavelet-HLL	glrlm	RunVariance	0.030187404
wavelet-HLL	glrlm	GrayLevelNonUniformity	3.670399865
wavelet-HLL	glrlm	LongRunEmphasis	1.091649145
wavelet-HLL	glrlm	ShortRunHighGrayLevelEmphasis	371.7931055
wavelet-HLL	glrlm	RunLengthNonUniformity	89.08625287
wavelet-HLL	glrlm	ShortRunEmphasis	0.978048052
wavelet-HLL	glrlm	LongRunHighGrayLevelEmphasis	397.3201433
wavelet-HLL	glrlm	RunPercentage	0.971451229
wavelet-HLL	glrlm	LongRunLowGrayLevelEmphasis	0.025328043
wavelet-HLL	glrlm	RunEntropy	4.925423626
wavelet-HLL	glrlm	HighGrayLevelRunEmphasis	376.7686754
wavelet-HLL	glrlm	RunLengthNonUniformityNormalized	0.944403144
wavelet-HLL	glszm	GrayLevelVariance	80.81577189
wavelet-HLL	glszm	ZoneVariance	0.724883047
wavelet-HLL	glszm	GrayLevelNonUniformityNormalized	0.036310982
wavelet-HLL	glszm	SizeZoneNonUniformityNormalized	0.550011138
wavelet-HLL	glszm	SizeZoneNonUniformity	36.85074627
wavelet-HLL	glszm	GrayLevelNonUniformity	2.432835821
wavelet-HLL	glszm	LargeAreaEmphasis	2.820895522
wavelet-HLL	glszm	SmallAreaHighGrayLevelEmphasis	351.3198549
wavelet-HLL	glszm	ZonePercentage	0.690721649
wavelet-HLL	glszm	LargeAreaLowGrayLevelEmphasis	0.050860767
wavelet-HLL	glszm	LargeAreaHighGrayLevelEmphasis	825.2686567
wavelet-HLL	glszm	HighGrayLevelZoneEmphasis	424.4477612
wavelet-HLL	glszm	SmallAreaEmphasis	0.770290216
wavelet-HLL	glszm	LowGrayLevelZoneEmphasis	0.029211656
wavelet-HLL	glszm	ZoneEntropy	5.386838817
wavelet-HLL	glszm	SmallAreaLowGrayLevelEmphasis	0.026582452
wavelet-HLL	ngtdm	Coarseness	0.061382561
wavelet-HLL	ngtdm	Complexity	1973.642819
wavelet-HLL	ngtdm	Strength	24.58097021
wavelet-HLL	ngtdm	Contrast	0.696488662
wavelet-HLL	ngtdm	Busyness	0.037610753
wavelet-LHL	gldm	GrayLevelVariance	71.41226485
wavelet-LHL	gldm	HighGrayLevelEmphasis	555.8659794
wavelet-LHL	gldm	DependenceEntropy	5.411415806
wavelet-LHL	gldm	DependenceNonUniformity	38.15463918
wavelet-LHL	gldm	GrayLevelNonUniformity	3.742268041
wavelet-LHL	gldm	SmallDependenceEmphasis	0.620418099
wavelet-LHL	gldm	SmallDependenceHighGrayLevelEmphasis	320.1204181
wavelet-LHL	gldm	DependenceNonUniformityNormalized	0.393346796
wavelet-LHL	gldm	LargeDependenceEmphasis	3.391752577
wavelet-LHL	gldm	LargeDependenceLowGrayLevelEmphasis	0.02042573
wavelet-LHL	gldm	DependenceVariance	0.636837071
wavelet-LHL	gldm	LargeDependenceHighGrayLevelEmphasis	2114.051546
wavelet-LHL	gldm	SmallDependenceLowGrayLevelEmphasis	0.013674908
wavelet-LHL	gldm	LowGrayLevelEmphasis	0.014670156
wavelet-LHL	glcm	JointAverage	21.3931924

wavelet-LHL	glcm	SumAverage	42.7863848
wavelet-LHL	glcm	JointEntropy	6.664870476
wavelet-LHL	glcm	ClusterShade	221.2365245
wavelet-LHL	glcm	MaximumProbability	0.018301398
wavelet-LHL	glcm	Idmn	0.941386161
wavelet-LHL	glcm	JointEnergy	0.010283709
wavelet-LHL	glcm	Contrast	92.44765563
wavelet-LHL	glcm	DifferenceEntropy	3.899226982
wavelet-LHL	glcm	InverseVariance	0.166546566
wavelet-LHL	glcm	DifferenceVariance	33.80178355
wavelet-LHL	glcm	Idn	0.844913203
wavelet-LHL	glcm	Idm	0.141146183
wavelet-LHL	glcm	Correlation	0.315401655
wavelet-LHL	glcm	Autocorrelation	479.757582
wavelet-LHL	glcm	SumEntropy	4.920342969
wavelet-LHL	glcm	SumSquares	68.08493802
wavelet-LHL	glcm	ClusterProminence	73712.45355
wavelet-LHL	glcm	Imc2	0.997911568
wavelet-LHL	glcm	Imc1	-0.585634639
wavelet-LHL	glcm	DifferenceAverage	7.381000396
wavelet-LHL	glcm	Id	0.225723411
wavelet-LHL	glcm	ClusterTendency	179.8920964
wavelet-LHL	firstorder	InterquartileRange	352.0220645
wavelet-LHL	firstorder	Skewness	-0.305313326
wavelet-LHL	firstorder	Uniformity	0.038580083
wavelet-LHL	firstorder	Median	-31.02497234
wavelet-LHL	firstorder	Energy	4447889.484
wavelet-LHL	firstorder	RobustMeanAbsoluteDeviation	138.7350689
wavelet-LHL	firstorder	MeanAbsoluteDeviation	179.0619812
wavelet-LHL	firstorder	TotalEnergy	120093016.1
wavelet-LHL	firstorder	Maximum	324.401999
wavelet-LHL	firstorder	RootMeanSquared	214.1367105
wavelet-LHL	firstorder	90Percentile	236.4596262
wavelet-LHL	firstorder	Minimum	-561.7843263
wavelet-LHL	firstorder	Entropy	4.800410823
wavelet-LHL	firstorder	Range	886.1863253
wavelet-LHL	firstorder	Variance	44523.47141
wavelet-LHL	firstorder	10Percentile	-316.8495364
wavelet-LHL	firstorder	Kurtosis	2.171075155
wavelet-LHL	firstorder	Mean	-36.4836861
wavelet-LHL	glrlm	ShortRunLowGrayLevelEmphasis	0.014942401
wavelet-LHL	glrlm	GrayLevelVariance	71.78175494
wavelet-LHL	glrlm	LowGrayLevelRunEmphasis	0.014991548
wavelet-LHL	glrlm	GrayLevelNonUniformityNormalized	0.038454533
wavelet-LHL	glrlm	RunVariance	0.025323982
wavelet-LHL	glrlm	GrayLevelNonUniformity	3.636099834
wavelet-LHL	glrlm	LongRunEmphasis	1.079123915
wavelet-LHL	glrlm	ShortRunHighGrayLevelEmphasis	540.9516665
wavelet-LHL	glrlm	RunLengthNonUniformity	89.80898753
wavelet-LHL	glrlm	ShortRunEmphasis	0.980219021
wavelet-LHL	glrlm	LongRunHighGrayLevelEmphasis	604.3268316
wavelet-LHL	glrlm	RunPercentage	0.974623315
wavelet-LHL	glrlm	LongRunLowGrayLevelEmphasis	0.015188133
wavelet-LHL	glrlm	RunEntropy	4.878806774
wavelet-LHL	glrlm	HighGrayLevelRunEmphasis	553.6266995

wavelet-LHL	glrlm	RunLengthNonUniformityNormalized	0.949352035
wavelet-LHL	glszm	GrayLevelVariance	76.74984247
wavelet-LHL	glszm	ZoneVariance	0.646922915
wavelet-LHL	glszm	GrayLevelNonUniformityNormalized	0.038857383
wavelet-LHL	glszm	SizeZoneNonUniformityNormalized	0.577399706
wavelet-LHL	glszm	SizeZoneNonUniformity	39.84057971
wavelet-LHL	glszm	GrayLevelNonUniformity	2.68115942
wavelet-LHL	glszm	LargeAreaEmphasis	2.623188406
wavelet-LHL	glszm	SmallAreaHighGrayLevelEmphasis	399.9695692
wavelet-LHL	glszm	ZonePercentage	0.711340206
wavelet-LHL	glszm	LargeAreaLowGrayLevelEmphasis	0.023547995
wavelet-LHL	glszm	LargeAreaHighGrayLevelEmphasis	1594.565217
wavelet-LHL	glszm	HighGrayLevelZoneEmphasis	526.9275362
wavelet-LHL	glszm	SmallAreaEmphasis	0.788522544
wavelet-LHL	glszm	LowGrayLevelZoneEmphasis	0.019569523
wavelet-LHL	glszm	ZoneEntropy	5.195197645
wavelet-LHL	glszm	SmallAreaLowGrayLevelEmphasis	0.018990169
wavelet-LHL	ngtdm	Coarseness	0.052196871
wavelet-LHL	ngtdm	Complexity	2020.176824
wavelet-LHL	ngtdm	Strength	19.64004188
wavelet-LHL	ngtdm	Contrast	0.822680953
wavelet-LHL	ngtdm	Busyness	0.037768249
wavelet-LHH	gldm	GrayLevelVariance	11.996599
wavelet-LHH	gldm	HighGrayLevelEmphasis	85.56701031
wavelet-LHH	gldm	DependenceEntropy	4.963127729
wavelet-LHH	gldm	DependenceNonUniformity	25.24742268
wavelet-LHH	gldm	GrayLevelNonUniformity	10.19587629
wavelet-LHH	gldm	SmallDependenceEmphasis	0.505084391
wavelet-LHH	gldm	SmallDependenceHighGrayLevelEmphasis	46.21777784
wavelet-LHH	gldm	DependenceNonUniformityNormalized	0.260282708
wavelet-LHH	gldm	LargeDependenceEmphasis	9
wavelet-LHH	gldm	LargeDependenceLowGrayLevelEmphasis	0.242734754
wavelet-LHH	gldm	DependenceVariance	2.929110426
wavelet-LHH	gldm	LargeDependenceHighGrayLevelEmphasis	684.9278351
wavelet-LHH	gldm	SmallDependenceLowGrayLevelEmphasis	0.021558721
wavelet-LHH	gldm	LowGrayLevelEmphasis	0.047943047
wavelet-LHH	glcm	JointAverage	8.603135519
wavelet-LHH	glcm	SumAverage	17.20627104
wavelet-LHH	glcm	JointEntropy	6.010741723
wavelet-LHH	glcm	ClusterShade	-7.149271747
wavelet-LHH	glcm	MaximumProbability	0.054770664
wavelet-LHH	glcm	Idmn	0.939849602
wavelet-LHH	glcm	JointEnergy	0.019009613
wavelet-LHH	glcm	Contrast	26.09521328
wavelet-LHH	glcm	DifferenceEntropy	3.235039516
wavelet-LHH	glcm	InverseVariance	0.214727735
wavelet-LHH	glcm	DifferenceVariance	9.532771445
wavelet-LHH	glcm	Idn	0.84067278
wavelet-LHH	glcm	Idm	0.226589489
wavelet-LHH	glcm	Correlation	-0.013441422
wavelet-LHH	glcm	Autocorrelation	73.8354825
wavelet-LHH	glcm	SumEntropy	3.957585766
wavelet-LHH	glcm	SumSquares	12.85173229
wavelet-LHH	glcm	ClusterProminence	1981.729421
wavelet-LHH	glcm	Imc2	0.956321074

wavelet-LHH	glcm	Imc1	-0.344169636
wavelet-LHH	glcm	DifferenceAverage	3.998009773
wavelet-LHH	glcm	Id	0.320403452
wavelet-LHH	glcm	ClusterTendency	25.3117159
wavelet-LHH	firstorder	InterquartileRange	95.40852768
wavelet-LHH	firstorder	Skewness	0.11079303
wavelet-LHH	firstorder	Uniformity	0.105112127
wavelet-LHH	firstorder	Median	5.988001591
wavelet-LHH	firstorder	Energy	727748.636
wavelet-LHH	firstorder	RobustMeanAbsoluteDeviation	41.50687293
wavelet-LHH	firstorder	MeanAbsoluteDeviation	65.39695608
wavelet-LHH	firstorder	TotalEnergy	19649213.17
wavelet-LHH	firstorder	Maximum	252.2683445
wavelet-LHH	firstorder	RootMeanSquared	86.61733809
wavelet-LHH	firstorder	90Percentile	115.7678078
wavelet-LHH	firstorder	Minimum	-189.6512649
wavelet-LHH	firstorder	Entropy	3.611650886
wavelet-LHH	firstorder	Range	441.9196094
wavelet-LHH	firstorder	Variance	7496.998197
wavelet-LHH	firstorder	10Percentile	-117.3452557
wavelet-LHH	firstorder	Kurtosis	3.122361321
wavelet-LHH	firstorder	Mean	2.359038141
wavelet-LHH	glrlm	ShortRunLowGrayLevelEmphasis	0.047522607
wavelet-LHH	glrlm	GrayLevelVariance	12.45764179
wavelet-LHH	glrlm	LowGrayLevelRunEmphasis	0.048937551
wavelet-LHH	glrlm	GrayLevelNonUniformityNormalized	0.098809591
wavelet-LHH	glrlm	RunVariance	0.061083166
wavelet-LHH	glrlm	GrayLevelNonUniformity	9.055822076
wavelet-LHH	glrlm	LongRunEmphasis	1.187483294
wavelet-LHH	glrlm	ShortRunHighGrayLevelEmphasis	82.7276808
wavelet-LHH	glrlm	RunLengthNonUniformity	81.83416666
wavelet-LHH	glrlm	ShortRunEmphasis	0.956025141
wavelet-LHH	glrlm	LongRunHighGrayLevelEmphasis	100.4488918
wavelet-LHH	glrlm	RunPercentage	0.94369548
wavelet-LHH	glrlm	LongRunLowGrayLevelEmphasis	0.054770629
wavelet-LHH	glrlm	RunEntropy	3.882841343
wavelet-LHH	glrlm	HighGrayLevelRunEmphasis	86.10830884
wavelet-LHH	glrlm	RunLengthNonUniformityNormalized	0.892241069
wavelet-LHH	glszm	GrayLevelVariance	16.05994898
wavelet-LHH	glszm	ZoneVariance	3.517538265
wavelet-LHH	glszm	GrayLevelNonUniformityNormalized	0.075255102
wavelet-LHH	glszm	SizeZoneNonUniformityNormalized	0.581632653
wavelet-LHH	glszm	SizeZoneNonUniformity	32.57142857
wavelet-LHH	glszm	GrayLevelNonUniformity	4.214285714
wavelet-LHH	glszm	LargeAreaEmphasis	6.517857143
wavelet-LHH	glszm	SmallAreaHighGrayLevelEmphasis	72.67879588
wavelet-LHH	glszm	ZonePercentage	0.577319588
wavelet-LHH	glszm	LargeAreaLowGrayLevelEmphasis	0.19918563
wavelet-LHH	glszm	LargeAreaHighGrayLevelEmphasis	502.1428571
wavelet-LHH	glszm	HighGrayLevelZoneEmphasis	90.14285714
wavelet-LHH	glszm	SmallAreaEmphasis	0.788128445
wavelet-LHH	glszm	LowGrayLevelZoneEmphasis	0.05029515
wavelet-LHH	glszm	ZoneEntropy	4.496924958
wavelet-LHH	glszm	SmallAreaLowGrayLevelEmphasis	0.030591783
wavelet-LHH	ngtdm	Coarseness	0.058023003

wavelet-LHH	ngtdm	Complexity	299.0286784
wavelet-LHH	ngtdm	Strength	4.840620481
wavelet-LHH	ngtdm	Contrast	0.24522807
wavelet-LHH	ngtdm	Busyness	0.119855949
wavelet-LLH	gldm	GrayLevelVariance	69.77404613
wavelet-LLH	gldm	HighGrayLevelEmphasis	538.4742268
wavelet-LLH	gldm	DependenceEntropy	5.34450627
wavelet-LLH	gldm	DependenceNonUniformity	37.6185567
wavelet-LLH	gldm	GrayLevelNonUniformity	4.154639175
wavelet-LLH	gldm	SmallDependenceEmphasis	0.624982818
wavelet-LLH	gldm	SmallDependenceHighGrayLevelEmphasis	363.4297652
wavelet-LLH	gldm	DependenceNonUniformityNormalized	0.387820172
wavelet-LLH	gldm	LargeDependenceEmphasis	3.721649485
wavelet-LLH	gldm	LargeDependenceLowGrayLevelEmphasis	0.027258122
wavelet-LLH	gldm	DependenceVariance	0.828143267
wavelet-LLH	gldm	LargeDependenceHighGrayLevelEmphasis	1667.443299
wavelet-LLH	gldm	SmallDependenceLowGrayLevelEmphasis	0.014355637
wavelet-LLH	gldm	LowGrayLevelEmphasis	0.016002964
wavelet-LLH	glcm	JointAverage	21.19070485
wavelet-LLH	glcm	SumAverage	42.38140971
wavelet-LLH	glcm	JointEntropy	6.639111129
wavelet-LLH	glcm	ClusterShade	241.4688331
wavelet-LLH	glcm	MaximumProbability	0.020664583
wavelet-LLH	glcm	Idmn	0.949691313
wavelet-LLH	glcm	JointEnergy	0.010571525
wavelet-LLH	glcm	Contrast	94.41956971
wavelet-LLH	glcm	DifferenceEntropy	3.937016398
wavelet-LLH	glcm	InverseVariance	0.138375923
wavelet-LLH	glcm	DifferenceVariance	34.14316062
wavelet-LLH	glcm	Idn	0.853224536
wavelet-LLH	glcm	Idm	0.131277204
wavelet-LLH	glcm	Correlation	0.276248142
wavelet-LLH	glcm	Autocorrelation	468.0096058
wavelet-LLH	glcm	SumEntropy	4.911159241
wavelet-LLH	glcm	SumSquares	66.11101345
wavelet-LLH	glcm	ClusterProminence	76569.10418
wavelet-LLH	glcm	Imc2	0.997191794
wavelet-LLH	glcm	Imc1	-0.564165981
wavelet-LLH	glcm	DifferenceAverage	7.579615533
wavelet-LLH	glcm	Id	0.218067474
wavelet-LLH	glcm	ClusterTendency	170.0244841
wavelet-LLH	firstorder	InterquartileRange	301.0678901
wavelet-LLH	firstorder	Skewness	-0.347586077
wavelet-LLH	firstorder	Uniformity	0.042831332
wavelet-LLH	firstorder	Median	-24.99054137
wavelet-LLH	firstorder	Energy	4493064.386
wavelet-LLH	firstorder	RobustMeanAbsoluteDeviation	115.7761731
wavelet-LLH	firstorder	MeanAbsoluteDeviation	169.1285682
wavelet-LLH	firstorder	TotalEnergy	121312738.4
wavelet-LLH	firstorder	Maximum	402.650138
wavelet-LLH	firstorder	RootMeanSquared	215.2214009
wavelet-LLH	firstorder	90Percentile	190.8638033
wavelet-LLH	firstorder	Minimum	-573.0955312
wavelet-LLH	firstorder	Entropy	4.707757627
wavelet-LLH	firstorder	Range	975.7456692

wavelet-LLH	firstorder	Variance	44159.67464
wavelet-LLH	firstorder	10Percentile	-337.2903284
wavelet-LLH	firstorder	Kurtosis	2.699168096
wavelet-LLH	firstorder	Mean	-46.48200466
wavelet-LLH	glrlm	ShortRunLowGrayLevelEmphasis	0.016245519
wavelet-LLH	glrlm	GrayLevelVariance	70.45112559
wavelet-LLH	glrlm	LowGrayLevelRunEmphasis	0.016333421
wavelet-LLH	glrlm	GrayLevelNonUniformityNormalized	0.042748393
wavelet-LLH	glrlm	RunVariance	0.028583622
wavelet-LLH	glrlm	GrayLevelNonUniformity	4.035087961
wavelet-LLH	glrlm	LongRunEmphasis	1.085797105
wavelet-LLH	glrlm	ShortRunHighGrayLevelEmphasis	532.5286661
wavelet-LLH	glrlm	RunLengthNonUniformity	89.49693076
wavelet-LLH	glrlm	ShortRunEmphasis	0.979479746
wavelet-LLH	glrlm	LongRunHighGrayLevelEmphasis	579.0357565
wavelet-LLH	glrlm	RunPercentage	0.973037272
wavelet-LLH	glrlm	LongRunLowGrayLevelEmphasis	0.016690973
wavelet-LLH	glrlm	RunEntropy	4.787005748
wavelet-LLH	glrlm	HighGrayLevelRunEmphasis	541.3655729
wavelet-LLH	glrlm	RunLengthNonUniformityNormalized	0.947631359
wavelet-LLH	glszm	GrayLevelVariance	79.5442134
wavelet-LLH	glszm	ZoneVariance	0.820835959
wavelet-LLH	glszm	GrayLevelNonUniformityNormalized	0.04515858
wavelet-LLH	glszm	SizeZoneNonUniformityNormalized	0.597143457
wavelet-LLH	glszm	SizeZoneNonUniformity	41.20289855
wavelet-LLH	glszm	GrayLevelNonUniformity	3.115942029
wavelet-LLH	glszm	LargeAreaEmphasis	2.797101449
wavelet-LLH	glszm	SmallAreaHighGrayLevelEmphasis	473.5802093
wavelet-LLH	glszm	ZonePercentage	0.711340206
wavelet-LLH	glszm	LargeAreaLowGrayLevelEmphasis	0.027770571
wavelet-LLH	glszm	LargeAreaHighGrayLevelEmphasis	1373.855072
wavelet-LLH	glszm	HighGrayLevelZoneEmphasis	571.8695652
wavelet-LLH	glszm	SmallAreaEmphasis	0.800901771
wavelet-LLH	glszm	LowGrayLevelZoneEmphasis	0.020772183
wavelet-LLH	glszm	ZoneEntropy	5.079255616
wavelet-LLH	glszm	SmallAreaLowGrayLevelEmphasis	0.019838442
wavelet-LLH	ngtdm	Coarseness	0.050553699
wavelet-LLH	ngtdm	Complexity	2241.828731
wavelet-LLH	ngtdm	Strength	21.31606404
wavelet-LLH	ngtdm	Contrast	0.801949779
wavelet-LLH	ngtdm	Busyness	0.037274687
wavelet-LLL	gldm	GrayLevelVariance	571.7376979
wavelet-LLL	gldm	HighGrayLevelEmphasis	2151.185567
wavelet-LLL	gldm	DependenceEntropy	5.952955239
wavelet-LLL	gldm	DependenceNonUniformity	59.96907216
wavelet-LLL	gldm	GrayLevelNonUniformity	1.865979381
wavelet-LLL	gldm	SmallDependenceEmphasis	0.811569301
wavelet-LLL	gldm	SmallDependenceHighGrayLevelEmphasis	1727.485682
wavelet-LLL	gldm	DependenceNonUniformityNormalized	0.618237857
wavelet-LLL	gldm	LargeDependenceEmphasis	1.845360825
wavelet-LLL	gldm	LargeDependenceLowGrayLevelEmphasis	0.021112738
wavelet-LLL	gldm	DependenceVariance	0.237432246
wavelet-LLL	gldm	LargeDependenceHighGrayLevelEmphasis	4266.237113
wavelet-LLL	gldm	SmallDependenceLowGrayLevelEmphasis	0.013629961
wavelet-LLL	gldm	LowGrayLevelEmphasis	0.015120887

wavelet-LLL	glcm	JointAverage	47.45218233
wavelet-LLL	glcm	SumAverage	94.90436466
wavelet-LLL	glcm	JointEntropy	6.799087597
wavelet-LLL	glcm	ClusterShade	4539.030939
wavelet-LLL	glcm	MaximumProbability	0.014892405
wavelet-LLL	glcm	Idmn	0.92187971
wavelet-LLL	glcm	JointEnergy	0.009148933
wavelet-LLL	glcm	Contrast	857.7849235
wavelet-LLL	glcm	DifferenceEntropy	4.954306345
wavelet-LLL	glcm	InverseVariance	0.032019161
wavelet-LLL	glcm	DifferenceVariance	262.4292058
wavelet-LLL	glcm	Idn	0.813085521
wavelet-LLL	glcm	Idm	0.038530664
wavelet-LLL	glcm	Correlation	0.301008513
wavelet-LLL	glcm	Autocorrelation	2431.139963
wavelet-LLL	glcm	SumEntropy	5.45705745
wavelet-LLL	glcm	SumSquares	606.0056309
wavelet-LLL	glcm	ClusterProminence	5525903.682
wavelet-LLL	glcm	Imc2	0.999948162
wavelet-LLL	glcm	Imc1	-0.807625835
wavelet-LLL	glcm	DifferenceAverage	24.03901275
wavelet-LLL	glcm	Id	0.091558108
wavelet-LLL	glcm	ClusterTendency	1566.2376
wavelet-LLL	firstorder	InterquartileRange	906.1623342
wavelet-LLL	firstorder	Skewness	0.426073379
wavelet-LLL	firstorder	Uniformity	0.019236901
wavelet-LLL	firstorder	Median	-1099.526443
wavelet-LLL	firstorder	Energy	135440714
wavelet-LLL	firstorder	RobustMeanAbsoluteDeviation	371.9773088
wavelet-LLL	firstorder	MeanAbsoluteDeviation	498.6845269
wavelet-LLL	firstorder	TotalEnergy	3656899279
wavelet-LLL	firstorder	Maximum	359.3122504
wavelet-LLL	firstorder	RootMeanSquared	1181.649703
wavelet-LLL	firstorder	90Percentile	-169.2985768
wavelet-LLL	firstorder	Minimum	-1982.001143
wavelet-LLL	firstorder	Entropy	5.867752533
wavelet-LLL	firstorder	Range	2341.313393
wavelet-LLL	firstorder	Variance	355490.3431
wavelet-LLL	firstorder	10Percentile	-1758.604321
wavelet-LLL	firstorder	Kurtosis	2.257652837
wavelet-LLL	firstorder	Mean	-1020.198842
wavelet-LLL	glrlm	ShortRunLowGrayLevelEmphasis	0.015142178
wavelet-LLL	glrlm	GrayLevelVariance	569.8576184
wavelet-LLL	glrlm	LowGrayLevelRunEmphasis	0.01520133
wavelet-LLL	glrlm	GrayLevelNonUniformityNormalized	0.019161291
wavelet-LLL	glrlm	RunVariance	0.010303299
wavelet-LLL	glrlm	GrayLevelNonUniformity	1.839506168
wavelet-LLL	glrlm	LongRunEmphasis	1.031607481
wavelet-LLL	glrlm	ShortRunHighGrayLevelEmphasis	2129.715804
wavelet-LLL	glrlm	RunLengthNonUniformity	94.04395046
wavelet-LLL	glrlm	ShortRunEmphasis	0.99209813
wavelet-LLL	glrlm	LongRunHighGrayLevelEmphasis	2224.289266
wavelet-LLL	glrlm	RunPercentage	0.989690722
wavelet-LLL	glrlm	LongRunLowGrayLevelEmphasis	0.015437936
wavelet-LLL	glrlm	RunEntropy	5.883663491

wavelet-LLL	glrlm	HighGrayLevelRunEmphasis	2148.630496
wavelet-LLL	glrlm	RunLengthNonUniformityNormalized	0.979393403
wavelet-LLL	glszm	GrayLevelVariance	544.7147109
wavelet-LLL	glszm	ZoneVariance	0.178429705
wavelet-LLL	glszm	GrayLevelNonUniformityNormalized	0.018424036
wavelet-LLL	glszm	SizeZoneNonUniformityNormalized	0.767290249
wavelet-LLL	glszm	SizeZoneNonUniformity	64.45238095
wavelet-LLL	glszm	GrayLevelNonUniformity	1.547619048
wavelet-LLL	glszm	LargeAreaEmphasis	1.511904762
wavelet-LLL	glszm	SmallAreaHighGrayLevelEmphasis	1902.167989
wavelet-LLL	glszm	ZonePercentage	0.865979381
wavelet-LLL	glszm	LargeAreaLowGrayLevelEmphasis	0.019777163
wavelet-LLL	glszm	LargeAreaHighGrayLevelEmphasis	3443.821429
wavelet-LLL	glszm	HighGrayLevelZoneEmphasis	2113.440476
wavelet-LLL	glszm	SmallAreaEmphasis	0.898478836
wavelet-LLL	glszm	LowGrayLevelZoneEmphasis	0.016310269
wavelet-LLL	glszm	ZoneEntropy	5.930949714
wavelet-LLL	glszm	SmallAreaLowGrayLevelEmphasis	0.015451671
wavelet-LLL	ngtdm	Coarseness	0.035666164
wavelet-LLL	ngtdm	Complexity	33400.42581
wavelet-LLL	ngtdm	Strength	105.0977232
wavelet-LLL	ngtdm	Contrast	4.21759204
wavelet-LLL	ngtdm	Busyness	0.013920157
wavelet-HHL	gldm	GrayLevelVariance	9.058135827
wavelet-HHL	gldm	HighGrayLevelEmphasis	53
wavelet-HHL	gldm	DependenceEntropy	5.169313296
wavelet-HHL	gldm	DependenceNonUniformity	23.37113402
wavelet-HHL	gldm	GrayLevelNonUniformity	10.15463918
wavelet-HHL	gldm	SmallDependenceEmphasis	0.417393868
wavelet-HHL	gldm	SmallDependenceHighGrayLevelEmphasis	24.63687355
wavelet-HHL	gldm	DependenceNonUniformityNormalized	0.240939526
wavelet-HHL	gldm	LargeDependenceEmphasis	8.154639175
wavelet-HHL	gldm	LargeDependenceLowGrayLevelEmphasis	0.351498261
wavelet-HHL	gldm	DependenceVariance	2.184929323
wavelet-HHL	gldm	LargeDependenceHighGrayLevelEmphasis	361.7835052
wavelet-HHL	gldm	SmallDependenceLowGrayLevelEmphasis	0.049817075
wavelet-HHL	gldm	LowGrayLevelEmphasis	0.072912515
wavelet-HHL	glcm	JointAverage	6.721500282
wavelet-HHL	glcm	SumAverage	13.44300056
wavelet-HHL	glcm	JointEntropy	5.937086068
wavelet-HHL	glcm	ClusterShade	26.14663427
wavelet-HHL	glcm	MaximumProbability	0.050109558
wavelet-HHL	glcm	Idmn	0.921731068
wavelet-HHL	glcm	JointEnergy	0.019651851
wavelet-HHL	glcm	Contrast	18.94649624
wavelet-HHL	glcm	DifferenceEntropy	3.046402801
wavelet-HHL	glcm	InverseVariance	0.233155031
wavelet-HHL	glcm	DifferenceVariance	6.00398522
wavelet-HHL	glcm	Idn	0.816098813
wavelet-HHL	glcm	Idm	0.236490668
wavelet-HHL	glcm	Correlation	0.029433918
wavelet-HHL	glcm	Autocorrelation	45.50730742
wavelet-HHL	glcm	SumEntropy	3.882511556
wavelet-HHL	glcm	SumSquares	9.792493757
wavelet-HHL	glcm	ClusterProminence	1100.80218

wavelet-HHL	glcm	Imc2	0.927351521
wavelet-HHL	glcm	Imc1	-0.290426033
wavelet-HHL	glcm	DifferenceAverage	3.506036896
wavelet-HHL	glcm	Id	0.33229093
wavelet-HHL	glcm	ClusterTendency	20.22347879
wavelet-HHL	firstorder	InterquartileRange	103.3129412
wavelet-HHL	firstorder	Skewness	0.248454321
wavelet-HHL	firstorder	Uniformity	0.104687002
wavelet-HHL	firstorder	Median	3.088045405
wavelet-HHL	firstorder	Energy	546370.1703
wavelet-HHL	firstorder	RobustMeanAbsoluteDeviation	45.17729176
wavelet-HHL	firstorder	MeanAbsoluteDeviation	62.03939103
wavelet-HHL	firstorder	TotalEnergy	14751994.6
wavelet-HHL	firstorder	Maximum	177.9927838
wavelet-HHL	firstorder	RootMeanSquared	75.05119698
wavelet-HHL	firstorder	90Percentile	107.2439139
wavelet-HHL	firstorder	Minimum	-148.6919385
wavelet-HHL	firstorder	Entropy	3.48004055
wavelet-HHL	firstorder	Range	326.6847223
wavelet-HHL	firstorder	Variance	5617.123009
wavelet-HHL	firstorder	10Percentile	-84.31567414
wavelet-HHL	firstorder	Kurtosis	2.44995876
wavelet-HHL	firstorder	Mean	3.944509994
wavelet-HHL	glrlm	ShortRunLowGrayLevelEmphasis	0.073299859
wavelet-HHL	glrlm	GrayLevelVariance	9.236064257
wavelet-HHL	glrlm	LowGrayLevelRunEmphasis	0.074978175
wavelet-HHL	glrlm	GrayLevelNonUniformityNormalized	0.103092999
wavelet-HHL	glrlm	RunVariance	0.062120568
wavelet-HHL	glrlm	GrayLevelNonUniformity	9.445552724
wavelet-HHL	glrlm	LongRunEmphasis	1.185719034
wavelet-HHL	glrlm	ShortRunHighGrayLevelEmphasis	51.48756722
wavelet-HHL	glrlm	RunLengthNonUniformity	81.99379981
wavelet-HHL	glrlm	ShortRunEmphasis	0.956983512
wavelet-HHL	glrlm	LongRunHighGrayLevelEmphasis	62.34002421
wavelet-HHL	glrlm	RunPercentage	0.944488501
wavelet-HHL	glrlm	LongRunLowGrayLevelEmphasis	0.081928983
wavelet-HHL	glrlm	RunEntropy	3.71709191
wavelet-HHL	glrlm	HighGrayLevelRunEmphasis	53.4031035
wavelet-HHL	glrlm	RunLengthNonUniformityNormalized	0.893703029
wavelet-HHL	glszm	GrayLevelVariance	12.38026256
wavelet-HHL	glszm	ZoneVariance	5.251244907
wavelet-HHL	glszm	GrayLevelNonUniformityNormalized	0.089180625
wavelet-HHL	glszm	SizeZoneNonUniformityNormalized	0.451335446
wavelet-HHL	glszm	SizeZoneNonUniformity	21.21276596
wavelet-HHL	glszm	GrayLevelNonUniformity	4.191489362
wavelet-HHL	glszm	LargeAreaEmphasis	9.510638298
wavelet-HHL	glszm	SmallAreaHighGrayLevelEmphasis	42.71768643
wavelet-HHL	glszm	ZonePercentage	0.484536082
wavelet-HHL	glszm	LargeAreaLowGrayLevelEmphasis	0.425116715
wavelet-HHL	glszm	LargeAreaHighGrayLevelEmphasis	412.8297872
wavelet-HHL	glszm	HighGrayLevelZoneEmphasis	58.44680851
wavelet-HHL	glszm	SmallAreaEmphasis	0.695337005
wavelet-HHL	glszm	LowGrayLevelZoneEmphasis	0.110139932
wavelet-HHL	glszm	ZoneEntropy	4.572908735
wavelet-HHL	glszm	SmallAreaLowGrayLevelEmphasis	0.096099115

wavelet-HHL	ngtdm	Coarseness	0.049141821
wavelet-HHL	ngtdm	Complexity	177.2476032
wavelet-HHL	ngtdm	Strength	3.019545908
wavelet-HHL	ngtdm	Contrast	0.248046624
wavelet-HHL	ngtdm	Busyness	0.27777636
wavelet-HLH	gldm	GrayLevelVariance	15.91072378
wavelet-HLH	gldm	HighGrayLevelEmphasis	114.2680412
wavelet-HLH	gldm	DependenceEntropy	5.23689223
wavelet-HLH	gldm	DependenceNonUniformity	25.6185567
wavelet-HLH	gldm	GrayLevelNonUniformity	8.422680412
wavelet-HLH	gldm	SmallDependenceEmphasis	0.4639748
wavelet-HLH	gldm	SmallDependenceHighGrayLevelEmphasis	59.91408648
wavelet-HLH	gldm	DependenceNonUniformityNormalized	0.264108832
wavelet-HLH	gldm	LargeDependenceEmphasis	7.041237113
wavelet-HLH	gldm	LargeDependenceLowGrayLevelEmphasis	0.110805392
wavelet-HLH	gldm	DependenceVariance	1.850356042
wavelet-HLH	gldm	LargeDependenceHighGrayLevelEmphasis	686.4123711
wavelet-HLH	gldm	SmallDependenceLowGrayLevelEmphasis	0.021132471
wavelet-HLH	gldm	LowGrayLevelEmphasis	0.029306725
wavelet-HLH	glcm	JointAverage	9.992700843
wavelet-HLH	glcm	SumAverage	19.98540169
wavelet-HLH	glcm	JointEntropy	6.174111585
wavelet-HLH	glcm	ClusterShade	48.49915774
wavelet-HLH	glcm	MaximumProbability	0.03768224
wavelet-HLH	glcm	Idmn	0.950595619
wavelet-HLH	glcm	JointEnergy	0.016032005
wavelet-HLH	glcm	Contrast	30.73308286
wavelet-HLH	glcm	DifferenceEntropy	3.385670253
wavelet-HLH	glcm	InverseVariance	0.220186322
wavelet-HLH	glcm	DifferenceVariance	11.7846826
wavelet-HLH	glcm	Idn	0.855194212
wavelet-HLH	glcm	Idm	0.215303179
wavelet-HLH	glcm	Correlation	-0.023447396
wavelet-HLH	glcm	Autocorrelation	99.56252294
wavelet-HLH	glcm	SumEntropy	4.038414071
wavelet-HLH	glcm	SumSquares	15.00427881
wavelet-HLH	glcm	ClusterProminence	2987.346062
wavelet-HLH	glcm	Imc2	0.964197948
wavelet-HLH	glcm	Imc1	-0.357037043
wavelet-HLH	glcm	DifferenceAverage	4.296444079
wavelet-HLH	glcm	Id	0.307546337
wavelet-HLH	glcm	ClusterTendency	29.28403237
wavelet-HLH	firstorder	InterquartileRange	110.3706016
wavelet-HLH	firstorder	Skewness	0.600216032
wavelet-HLH	firstorder	Uniformity	0.086831757
wavelet-HLH	firstorder	Median	-9.404327624
wavelet-HLH	firstorder	Energy	954851.9076
wavelet-HLH	firstorder	RobustMeanAbsoluteDeviation	52.38039858
wavelet-HLH	firstorder	MeanAbsoluteDeviation	77.80995944
wavelet-HLH	firstorder	TotalEnergy	25781001.5
wavelet-HLH	firstorder	Maximum	341.6425766
wavelet-HLH	firstorder	RootMeanSquared	99.21609798
wavelet-HLH	firstorder	90Percentile	143.8983466
wavelet-HLH	firstorder	Minimum	-202.9168611
wavelet-HLH	firstorder	Entropy	3.819739676

wavelet-HLH	firstorder	Range	544.5594378
wavelet-HLH	firstorder	Variance	9729.674109
wavelet-HLH	firstorder	10Percentile	-98.53546179
wavelet-HLH	firstorder	Kurtosis	3.659831693
wavelet-HLH	firstorder	Mean	10.68456784
wavelet-HLH	glrlm	ShortRunLowGrayLevelEmphasis	0.029574747
wavelet-HLH	glrlm	GrayLevelVariance	16.44046652
wavelet-HLH	glrlm	LowGrayLevelRunEmphasis	0.030108758
wavelet-HLH	glrlm	GrayLevelNonUniformityNormalized	0.084224557
wavelet-HLH	glrlm	RunVariance	0.052426061
wavelet-HLH	glrlm	GrayLevelNonUniformity	7.769628631
wavelet-HLH	glrlm	LongRunEmphasis	1.159755473
wavelet-HLH	glrlm	ShortRunHighGrayLevelEmphasis	111.4093578
wavelet-HLH	glrlm	RunLengthNonUniformity	83.4960702
wavelet-HLH	glrlm	ShortRunEmphasis	0.96198205
wavelet-HLH	glrlm	LongRunHighGrayLevelEmphasis	130.8775831
wavelet-HLH	glrlm	RunPercentage	0.950832672
wavelet-HLH	glrlm	LongRunLowGrayLevelEmphasis	0.032314685
wavelet-HLH	glrlm	RunEntropy	4.040898142
wavelet-HLH	glrlm	HighGrayLevelRunEmphasis	115.1101512
wavelet-HLH	glrlm	RunLengthNonUniformityNormalized	0.904702161
wavelet-HLH	glszm	GrayLevelVariance	24.51980008
wavelet-HLH	glszm	ZoneVariance	3.774702038
wavelet-HLH	glszm	GrayLevelNonUniformityNormalized	0.065743945
wavelet-HLH	glszm	SizeZoneNonUniformityNormalized	0.500961169
wavelet-HLH	glszm	SizeZoneNonUniformity	25.54901961
wavelet-HLH	glszm	GrayLevelNonUniformity	3.352941176
wavelet-HLH	glszm	LargeAreaEmphasis	7.392156863
wavelet-HLH	glszm	SmallAreaHighGrayLevelEmphasis	98.71464839
wavelet-HLH	glszm	ZonePercentage	0.525773196
wavelet-HLH	glszm	LargeAreaLowGrayLevelEmphasis	0.125443362
wavelet-HLH	glszm	LargeAreaHighGrayLevelEmphasis	747.8039216
wavelet-HLH	glszm	HighGrayLevelZoneEmphasis	126.4901961
wavelet-HLH	glszm	SmallAreaEmphasis	0.733432129
wavelet-HLH	glszm	LowGrayLevelZoneEmphasis	0.042799529
wavelet-HLH	glszm	ZoneEntropy	4.880964009
wavelet-HLH	glszm	SmallAreaLowGrayLevelEmphasis	0.037859433
wavelet-HLH	ngtdm	Coarseness	0.048274309
wavelet-HLH	ngtdm	Complexity	465.3319554
wavelet-HLH	ngtdm	Strength	6.224588977
wavelet-HLH	ngtdm	Contrast	0.30741188
wavelet-HLH	ngtdm	Busyness	0.123303287

Image type	Feature Class	Feature Name	29: RTSTRUCT: C99-DNU_segment_ITV
general	info	BoundingBox	(0, 3, 1, 7, 10, 4)
general	info	EnabledImageTypes	{u'Original': {}, u'Wavelet': {}}
general	info	GeneralSettings	{'distances': [1], 'additionalInfo': True, 'force2D': False, 'interpolator': 'sitkBSpline', 'resampledPixelSpacing': [3.0], 'label': 1, 'normalizeScale': 1, 'normalize': False, 'force2Ddimension': 0, 'removeOutliers': None, 'minimumROISize': None, u'binWidth': 25.0, 'minimumROIDimensions': 1, u'correctMask': True, 'preCrop': False, u'symmetricalGLCM': True, 'resegmentRange': None, 'padDistance': 5}
general	info	ImageHash	e7637080b00ff9d669859e77dad4297181c53f72
general	info	ImageSpacing	(0.9765625000000002, 0.9765625000000002, 2.0)
general	info	MaskHash	e1153147483252c3f75ab462c6ae369a76ba6da9
general	info	NumpyVersion	1.13.1
general	info	PyWaveletVersion	0.5.2
general	info	SimpleITKVersion	1.1.0
general	info	Version	1.3.0.post102+g1b0eb74
general	info	VolumeNum	1
general	info	VoxelNum	108
original	shape	Maximum3DDiameter	27.82085549
original	shape	Maximum2DDiameterSlice	24.73863375
original	shape	Sphericity	0.825062707
original	shape	MinorAxis	15.6109778
original	shape	Elongation	0.635107016
original	shape	SurfaceVolumeRatio	0.41026833
original	shape	Volume	2916
original	shape	MajorAxis	24.58007454
original	shape	SurfaceArea	1196.342452
original	shape	Flatness	0.469128985
original	shape	LeastAxis	11.53122542
original	shape	Maximum2DDiameterColumn	18
original	shape	Maximum2DDiameterRow	27.65863337
original	gldm	GrayLevelVariance	104.4451303
original	gldm	HighGrayLevelEmphasis	373.6481481
original	gldm	DependenceEntropy	5.473372428
original	gldm	DependenceNonUniformity	51.77777778
original	gldm	GrayLevelNonUniformity	3.907407407
original	gldm	SmallDependenceEmphasis	0.708461934
original	gldm	SmallDependenceHighGrayLevelEmphasis	235.9691358
original	gldm	DependenceNonUniformityNormalized	0.479423868
original	gldm	LargeDependenceEmphasis	2.703703704
original	gldm	LargeDependenceLowGrayLevelEmphasis	0.057599223
original	gldm	DependenceVariance	0.508916324
original	gldm	LargeDependenceHighGrayLevelEmphasis	1163.87037
original	gldm	SmallDependenceLowGrayLevelEmphasis	0.032766931

original	gldm	LowGrayLevelEmphasis	0.036656296
original	glcm	JointAverage	19.02193139
original	glcm	SumAverage	38.04386278
original	glcm	JointEntropy	6.847560626
original	glcm	ClusterShade	1044.516773
original	glcm	MaximumProbability	0.018681754
original	glcm	Idmn	0.915318328
original	glcm	JointEnergy	0.009092243
original	glcm	Contrast	171.5303328
original	glcm	DifferenceEntropy	4.362871633
original	glcm	InverseVariance	0.095318572
original	glcm	DifferenceVariance	58.65535591
original	glcm	Idn	0.809275954
original	glcm	Idm	0.089957972
original	glcm	Correlation	0.245782237
original	glcm	Autocorrelation	389.4932219
original	glcm	SumEntropy	5.154410129
original	glcm	SumSquares	113.0422628
original	glcm	ClusterProminence	180085.7205
original	glcm	Imc2	0.998829246
original	glcm	Imc1	-0.6180506
original	glcm	DifferenceAverage	10.52193429
original	glcm	Id	0.168763174
original	glcm	ClusterTendency	280.6387185
original	firstorder	InterquartileRange	402.25
original	firstorder	Skewness	0.46127002
original	firstorder	Uniformity	0.036179698
original	firstorder	Median	-574.5
original	firstorder	Energy	37219210
original	firstorder	RobustMeanAbsoluteDeviation	169.3904813
original	firstorder	MeanAbsoluteDeviation	218.5576132
original	firstorder	TotalEnergy	1004918670
original	firstorder	Maximum	51
original	firstorder	RootMeanSquared	587.0454112
original	firstorder	90Percentile	-161.8
original	firstorder	Minimum	-923
original	firstorder	Entropy	4.98815365
original	firstorder	Range	974
original	firstorder	Variance	65329.63855
original	firstorder	10Percentile	-837.3
original	firstorder	Kurtosis	2.18064826
original	firstorder	Mean	-528.4814815
original	glrlm	ShortRunLowGrayLevelEmphasis	0.036945711
original	glrlm	GrayLevelVariance	103.7746701
original	glrlm	LowGrayLevelRunEmphasis	0.037114837
original	glrlm	GrayLevelNonUniformityNormalized	0.035906855
original	glrlm	RunVariance	0.02148847
original	glrlm	GrayLevelNonUniformity	3.806705402
original	glrlm	LongRunEmphasis	1.060549698
original	glrlm	ShortRunHighGrayLevelEmphasis	365.0547215
original	glrlm	RunLengthNonUniformity	102.4103575
original	glrlm	ShortRunEmphasis	0.986530642
original	glrlm	LongRunHighGrayLevelEmphasis	399.0873273

original	glrlm	RunPercentage	0.981481481
original	glrlm	LongRunLowGrayLevelEmphasis	0.037895595
original	glrlm	RunEntropy	5.038376154
original	glrlm	HighGrayLevelRunEmphasis	371.7758376
original	glrlm	RunLengthNonUniformityNormalized	0.965631135
original	glszm	GrayLevelVariance	93.13078821
original	glszm	ZoneVariance	0.764697344
original	glszm	GrayLevelNonUniformityNormalized	0.035563943
original	glszm	SizeZoneNonUniformityNormalized	0.689650167
original	glszm	SizeZoneNonUniformity	57.24096386
original	glszm	GrayLevelNonUniformity	2.951807229
original	glszm	LargeAreaEmphasis	2.457831325
original	glszm	SmallAreaHighGrayLevelEmphasis	273.1234473
original	glszm	ZonePercentage	0.768518519
original	glszm	LargeAreaLowGrayLevelEmphasis	0.061076376
original	glszm	LargeAreaHighGrayLevelEmphasis	1000.578313
original	glszm	HighGrayLevelZoneEmphasis	339.2048193
original	glszm	SmallAreaEmphasis	0.855814824
original	glszm	LowGrayLevelZoneEmphasis	0.043928161
original	glszm	ZoneEntropy	5.231739998
original	glszm	SmallAreaLowGrayLevelEmphasis	0.041802595
original	ngtdm	Coarseness	0.035198864
original	ngtdm	Complexity	4156.088884
original	ngtdm	Strength	22.529406
original	ngtdm	Contrast	1.107331548
original	ngtdm	Busyness	0.065910827
wavelet-HHH	gldm	GrayLevelVariance	12.58256173
wavelet-HHH	gldm	HighGrayLevelEmphasis	280.2685185
wavelet-HHH	gldm	DependenceEntropy	5.10171534
wavelet-HHH	gldm	DependenceNonUniformity	29.61111111
wavelet-HHH	gldm	GrayLevelNonUniformity	11.07407407
wavelet-HHH	gldm	SmallDependenceEmphasis	0.380156736
wavelet-HHH	gldm	SmallDependenceHighGrayLevelEmphasis	108.9177964
wavelet-HHH	gldm	DependenceNonUniformityNormalized	0.274176955
wavelet-HHH	gldm	LargeDependenceEmphasis	7.796296296
wavelet-HHH	gldm	LargeDependenceLowGrayLevelEmphasis	0.04022582
wavelet-HHH	gldm	DependenceVariance	1.911179698
wavelet-HHH	gldm	LargeDependenceHighGrayLevelEmphasis	2086.046296
wavelet-HHH	gldm	SmallDependenceLowGrayLevelEmphasis	0.010939643
wavelet-HHH	gldm	LowGrayLevelEmphasis	0.013390039
wavelet-HHH	glcm	JointAverage	16.41922894
wavelet-HHH	glcm	SumAverage	32.83845788
wavelet-HHH	glcm	JointEntropy	6.102563414
wavelet-HHH	glcm	ClusterShade	-18.00052019
wavelet-HHH	glcm	MaximumProbability	0.045865987
wavelet-HHH	glcm	Idmn	0.970058836
wavelet-HHH	glcm	JointEnergy	0.017450322
wavelet-HHH	glcm	Contrast	27.17433497
wavelet-HHH	glcm	DifferenceEntropy	3.227193987
wavelet-HHH	glcm	InverseVariance	0.256847116
wavelet-HHH	glcm	DifferenceVariance	12.00081093
wavelet-HHH	glcm	Idn	0.8891221
wavelet-HHH	glcm	Idm	0.246999249

wavelet-HHH	glcm	Correlation	-0.043153558
wavelet-HHH	glcm	Autocorrelation	269.0513173
wavelet-HHH	glcm	SumEntropy	3.96727229
wavelet-HHH	glcm	SumSquares	13.04004476
wavelet-HHH	glcm	ClusterProminence	3262.407293
wavelet-HHH	glcm	Imc2	0.944765309
wavelet-HHH	glcm	Imc1	-0.311330564
wavelet-HHH	glcm	DifferenceAverage	3.826691507
wavelet-HHH	glcm	Id	0.336300651
wavelet-HHH	glcm	ClusterTendency	24.98584409
wavelet-HHH	firstorder	InterquartileRange	83.4153275
wavelet-HHH	firstorder	Skewness	-0.282551059
wavelet-HHH	firstorder	Uniformity	0.102537723
wavelet-HHH	firstorder	Median	-3.806025117
wavelet-HHH	firstorder	Energy	832739.1986
wavelet-HHH	firstorder	RobustMeanAbsoluteDeviation	38.4946778
wavelet-HHH	firstorder	MeanAbsoluteDeviation	62.82762315
wavelet-HHH	firstorder	TotalEnergy	22483958.36
wavelet-HHH	firstorder	Maximum	285.4428839
wavelet-HHH	firstorder	RootMeanSquared	87.80972688
wavelet-HHH	firstorder	90Percentile	91.21558434
wavelet-HHH	firstorder	Minimum	-377.0098483
wavelet-HHH	firstorder	Entropy	3.594080099
wavelet-HHH	firstorder	Range	662.4527322
wavelet-HHH	firstorder	Variance	7699.354942
wavelet-HHH	firstorder	10Percentile	-104.3134182
wavelet-HHH	firstorder	Kurtosis	6.180240589
wavelet-HHH	firstorder	Mean	-3.345623102
wavelet-HHH	glrlm	ShortRunLowGrayLevelEmphasis	0.013783247
wavelet-HHH	glrlm	GrayLevelVariance	13.07102611
wavelet-HHH	glrlm	LowGrayLevelRunEmphasis	0.013945095
wavelet-HHH	glrlm	GrayLevelNonUniformityNormalized	0.100097441
wavelet-HHH	glrlm	RunVariance	0.065404875
wavelet-HHH	glrlm	GrayLevelNonUniformity	10.21887198
wavelet-HHH	glrlm	LongRunEmphasis	1.187032321
wavelet-HHH	glrlm	ShortRunHighGrayLevelEmphasis	269.6738042
wavelet-HHH	glrlm	RunLengthNonUniformity	91.81243698
wavelet-HHH	glrlm	ShortRunEmphasis	0.958867414
wavelet-HHH	glrlm	LongRunHighGrayLevelEmphasis	331.241124
wavelet-HHH	glrlm	RunPercentage	0.945156695
wavelet-HHH	glrlm	LongRunLowGrayLevelEmphasis	0.014682623
wavelet-HHH	glrlm	RunEntropy	3.844875806
wavelet-HHH	glrlm	HighGrayLevelRunEmphasis	280.8414114
wavelet-HHH	glrlm	RunLengthNonUniformityNormalized	0.898400392
wavelet-HHH	glszm	GrayLevelVariance	21.6001666
wavelet-HHH	glszm	ZoneVariance	7.264473136
wavelet-HHH	glszm	GrayLevelNonUniformityNormalized	0.082882132
wavelet-HHH	glszm	SizeZoneNonUniformityNormalized	0.402748855
wavelet-HHH	glszm	SizeZoneNonUniformity	19.73469388
wavelet-HHH	glszm	GrayLevelNonUniformity	4.06122449
wavelet-HHH	glszm	LargeAreaEmphasis	12.12244898
wavelet-HHH	glszm	SmallAreaHighGrayLevelEmphasis	182.9740244
wavelet-HHH	glszm	ZonePercentage	0.453703704

wavelet-HHH	glszm	LargeAreaLowGrayLevelEmphasis	0.068576277
wavelet-HHH	glszm	LargeAreaHighGrayLevelEmphasis	3217.081633
wavelet-HHH	glszm	HighGrayLevelZoneEmphasis	287.4897959
wavelet-HHH	glszm	SmallAreaEmphasis	0.635041393
wavelet-HHH	glszm	LowGrayLevelZoneEmphasis	0.02477612
wavelet-HHH	glszm	ZoneEntropy	4.792982038
wavelet-HHH	glszm	SmallAreaLowGrayLevelEmphasis	0.023311587
wavelet-HHH	ngtdm	Coarseness	0.053453657
wavelet-HHH	ngtdm	Complexity	379.7808232
wavelet-HHH	ngtdm	Strength	6.701656986
wavelet-HHH	ngtdm	Contrast	0.223600238
wavelet-HHH	ngtdm	Busyness	0.0634521
wavelet-HLL	gldm	GrayLevelVariance	126.5729595
wavelet-HLL	gldm	HighGrayLevelEmphasis	807.8796296
wavelet-HLL	gldm	DependenceEntropy	5.593923817
wavelet-HLL	gldm	DependenceNonUniformity	47.88888889
wavelet-HLL	gldm	GrayLevelNonUniformity	3.685185185
wavelet-HLL	gldm	SmallDependenceEmphasis	0.660300926
wavelet-HLL	gldm	SmallDependenceHighGrayLevelEmphasis	495.7431199
wavelet-HLL	gldm	DependenceNonUniformityNormalized	0.443415638
wavelet-HLL	gldm	LargeDependenceEmphasis	2.833333333
wavelet-HLL	gldm	LargeDependenceLowGrayLevelEmphasis	0.02162383
wavelet-HLL	gldm	DependenceVariance	0.47085048
wavelet-HLL	gldm	LargeDependenceHighGrayLevelEmphasis	2648.175926
wavelet-HLL	gldm	SmallDependenceLowGrayLevelEmphasis	0.015902536
wavelet-HLL	gldm	LowGrayLevelEmphasis	0.016915286
wavelet-HLL	glcm	JointAverage	25.81025168
wavelet-HLL	glcm	SumAverage	51.62050335
wavelet-HLL	glcm	JointEntropy	6.836170689
wavelet-HLL	glcm	ClusterShade	-916.740353
wavelet-HLL	glcm	MaximumProbability	0.021855784
wavelet-HLL	glcm	Idmn	0.930688725
wavelet-HLL	glcm	JointEnergy	0.009223398
wavelet-HLL	glcm	Contrast	205.1735981
wavelet-HLL	glcm	DifferenceEntropy	4.327113897
wavelet-HLL	glcm	InverseVariance	0.104953687
wavelet-HLL	glcm	DifferenceVariance	76.42250153
wavelet-HLL	glcm	Idn	0.833632553
wavelet-HLL	glcm	Idm	0.098085832
wavelet-HLL	glcm	Correlation	0.234478388
wavelet-HLL	glcm	Autocorrelation	699.0387242
wavelet-HLL	glcm	SumEntropy	5.1664442
wavelet-HLL	glcm	SumSquares	135.0380994
wavelet-HLL	glcm	ClusterProminence	294049.6145
wavelet-HLL	glcm	Imc2	0.999032337
wavelet-HLL	glcm	Imc1	-0.630047371
wavelet-HLL	glcm	DifferenceAverage	10.85088253
wavelet-HLL	glcm	Id	0.177159927
wavelet-HLL	glcm	ClusterTendency	334.9787994
wavelet-HLL	firstorder	InterquartileRange	367.0300998
wavelet-HLL	firstorder	Skewness	-0.298502044
wavelet-HLL	firstorder	Uniformity	0.034122085
wavelet-HLL	firstorder	Median	-34.01615026

wavelet-HLL	firstorder	Energy	8874418.183
wavelet-HLL	firstorder	RobustMeanAbsoluteDeviation	163.963716
wavelet-HLL	firstorder	MeanAbsoluteDeviation	229.3385992
wavelet-HLL	firstorder	TotalEnergy	239609290.9
wavelet-HLL	firstorder	Maximum	495.7614681
wavelet-HLL	firstorder	RootMeanSquared	286.6540401
wavelet-HLL	firstorder	90Percentile	272.2054434
wavelet-HLL	firstorder	Minimum	-690.8684633
wavelet-HLL	firstorder	Entropy	5.035555646
wavelet-HLL	firstorder	Range	1186.629931
wavelet-HLL	firstorder	Variance	78664.40464
wavelet-HLL	firstorder	10Percentile	-470.1358239
wavelet-HLL	firstorder	Kurtosis	2.45362445
wavelet-HLL	firstorder	Mean	-59.21261763
wavelet-HLL	glrlm	ShortRunLowGrayLevelEmphasis	0.017176477
wavelet-HLL	glrlm	GrayLevelVariance	126.2050356
wavelet-HLL	glrlm	LowGrayLevelRunEmphasis	0.01722039
wavelet-HLL	glrlm	GrayLevelNonUniformityNormalized	0.034108676
wavelet-HLL	glrlm	RunVariance	0.02060686
wavelet-HLL	glrlm	GrayLevelNonUniformity	3.607112704
wavelet-HLL	glrlm	LongRunEmphasis	1.064495721
wavelet-HLL	glrlm	ShortRunHighGrayLevelEmphasis	788.4797606
wavelet-HLL	glrlm	RunLengthNonUniformity	101.4898457
wavelet-HLL	glrlm	ShortRunEmphasis	0.98387607
wavelet-HLL	glrlm	LongRunHighGrayLevelEmphasis	867.2686078
wavelet-HLL	glrlm	RunPercentage	0.979344729
wavelet-HLL	glrlm	LongRunLowGrayLevelEmphasis	0.017396041
wavelet-HLL	glrlm	RunEntropy	5.081441254
wavelet-HLL	glrlm	HighGrayLevelRunEmphasis	804.2375301
wavelet-HLL	glrlm	RunLengthNonUniformityNormalized	0.95878628
wavelet-HLL	glszm	GrayLevelVariance	124.5910684
wavelet-HLL	glszm	ZoneVariance	0.444444444
wavelet-HLL	glszm	GrayLevelNonUniformityNormalized	0.03368389
wavelet-HLL	glszm	SizeZoneNonUniformityNormalized	0.603414114
wavelet-HLL	glszm	SizeZoneNonUniformity	48.87654321
wavelet-HLL	glszm	GrayLevelNonUniformity	2.728395062
wavelet-HLL	glszm	LargeAreaEmphasis	2.222222222
wavelet-HLL	glszm	SmallAreaHighGrayLevelEmphasis	597.3203875
wavelet-HLL	glszm	ZonePercentage	0.75
wavelet-HLL	glszm	LargeAreaLowGrayLevelEmphasis	0.024840447
wavelet-HLL	glszm	LargeAreaHighGrayLevelEmphasis	1914.135802
wavelet-HLL	glszm	HighGrayLevelZoneEmphasis	771.382716
wavelet-HLL	glszm	SmallAreaEmphasis	0.805041152
wavelet-HLL	glszm	LowGrayLevelZoneEmphasis	0.021591592
wavelet-HLL	glszm	ZoneEntropy	5.427821571
wavelet-HLL	glszm	SmallAreaLowGrayLevelEmphasis	0.020968577
wavelet-HLL	ngtdm	Coarseness	0.042862834
wavelet-HLL	ngtdm	Complexity	4657.547406
wavelet-HLL	ngtdm	Strength	30.04661728
wavelet-HLL	ngtdm	Contrast	1.269333927
wavelet-HLL	ngtdm	Busyness	0.027604301
wavelet-LHL	gldm	GrayLevelVariance	45.96879287
wavelet-LHL	gldm	HighGrayLevelEmphasis	281.6481481

wavelet-LHL	gldm	DependenceEntropy	5.424806572
wavelet-LHL	gldm	DependenceNonUniformity	40.7962963
wavelet-LHL	gldm	GrayLevelNonUniformity	5.148148148
wavelet-LHL	gldm	SmallDependenceEmphasis	0.599215535
wavelet-LHL	gldm	SmallDependenceHighGrayLevelEmphasis	164.7602238
wavelet-LHL	gldm	DependenceNonUniformityNormalized	0.377743484
wavelet-LHL	gldm	LargeDependenceEmphasis	3.574074074
wavelet-LHL	gldm	LargeDependenceLowGrayLevelEmphasis	0.045915292
wavelet-LHL	gldm	DependenceVariance	0.671467764
wavelet-LHL	gldm	LargeDependenceHighGrayLevelEmphasis	1008.648148
wavelet-LHL	gldm	SmallDependenceLowGrayLevelEmphasis	0.017557971
wavelet-LHL	gldm	LowGrayLevelEmphasis	0.021892166
wavelet-LHL	glcm	JointAverage	14.96545504
wavelet-LHL	glcm	SumAverage	29.93091009
wavelet-LHL	glcm	JointEntropy	6.703252034
wavelet-LHL	glcm	ClusterShade	-17.14985743
wavelet-LHL	glcm	MaximumProbability	0.023782045
wavelet-LHL	glcm	Idmn	0.932631432
wavelet-LHL	glcm	JointEnergy	0.010375342
wavelet-LHL	glcm	Contrast	73.682525
wavelet-LHL	glcm	DifferenceEntropy	3.840177254
wavelet-LHL	glcm	InverseVariance	0.13739056
wavelet-LHL	glcm	DifferenceVariance	24.81650802
wavelet-LHL	glcm	Idn	0.829039291
wavelet-LHL	glcm	Idm	0.132762179
wavelet-LHL	glcm	Correlation	0.220777905
wavelet-LHL	glcm	Autocorrelation	234.7049289
wavelet-LHL	glcm	SumEntropy	4.86131902
wavelet-LHL	glcm	SumSquares	47.46044808
wavelet-LHL	glcm	ClusterProminence	29362.00657
wavelet-LHL	glcm	Imc2	0.995544915
wavelet-LHL	glcm	Imc1	-0.525825421
wavelet-LHL	glcm	DifferenceAverage	6.846453997
wavelet-LHL	glcm	Id	0.223658537
wavelet-LHL	glcm	ClusterTendency	116.1592673
wavelet-LHL	firstorder	InterquartileRange	272.0384764
wavelet-LHL	firstorder	Skewness	-0.134160837
wavelet-LHL	firstorder	Uniformity	0.047668038
wavelet-LHL	firstorder	Median	-8.003181937
wavelet-LHL	firstorder	Energy	3217046.391
wavelet-LHL	firstorder	RobustMeanAbsoluteDeviation	106.6379084
wavelet-LHL	firstorder	MeanAbsoluteDeviation	141.9556894
wavelet-LHL	firstorder	TotalEnergy	86860252.55
wavelet-LHL	firstorder	Maximum	333.7555564
wavelet-LHL	firstorder	RootMeanSquared	172.5904591
wavelet-LHL	firstorder	90Percentile	176.6378578
wavelet-LHL	firstorder	Minimum	-383.9696248
wavelet-LHL	firstorder	Entropy	4.586876773
wavelet-LHL	firstorder	Range	717.7251811
wavelet-LHL	firstorder	Variance	28918.85587
wavelet-LHL	firstorder	10Percentile	-272.8367273
wavelet-LHL	firstorder	Kurtosis	2.17272586
wavelet-LHL	firstorder	Mean	-29.4722024

wavelet-LHL	glrlm	ShortRunLowGrayLevelEmphasis	0.022018366
wavelet-LHL	glrlm	GrayLevelVariance	46.24518915
wavelet-LHL	glrlm	LowGrayLevelRunEmphasis	0.022227366
wavelet-LHL	glrlm	GrayLevelNonUniformityNormalized	0.047280511
wavelet-LHL	glrlm	RunVariance	0.027025358
wavelet-LHL	glrlm	GrayLevelNonUniformity	4.968136262
wavelet-LHL	glrlm	LongRunEmphasis	1.084121287
wavelet-LHL	glrlm	ShortRunHighGrayLevelEmphasis	275.5151714
wavelet-LHL	glrlm	RunLengthNonUniformity	99.44134806
wavelet-LHL	glrlm	ShortRunEmphasis	0.978969678
wavelet-LHL	glrlm	LongRunHighGrayLevelEmphasis	305.5805536
wavelet-LHL	glrlm	RunPercentage	0.972934473
wavelet-LHL	glrlm	LongRunLowGrayLevelEmphasis	0.023063365
wavelet-LHL	glrlm	RunEntropy	4.684598403
wavelet-LHL	glrlm	HighGrayLevelRunEmphasis	281.5282478
wavelet-LHL	glrlm	RunLengthNonUniformityNormalized	0.945949284
wavelet-LHL	glszm	GrayLevelVariance	49.91837107
wavelet-LHL	glszm	ZoneVariance	0.842951059
wavelet-LHL	glszm	GrayLevelNonUniformityNormalized	0.044558072
wavelet-LHL	glszm	SizeZoneNonUniformityNormalized	0.562089116
wavelet-LHL	glszm	SizeZoneNonUniformity	41.59459459
wavelet-LHL	glszm	GrayLevelNonUniformity	3.297297297
wavelet-LHL	glszm	LargeAreaEmphasis	2.972972973
wavelet-LHL	glszm	SmallAreaHighGrayLevelEmphasis	213.3564189
wavelet-LHL	glszm	ZonePercentage	0.685185185
wavelet-LHL	glszm	LargeAreaLowGrayLevelEmphasis	0.046058255
wavelet-LHL	glszm	LargeAreaHighGrayLevelEmphasis	814.1081081
wavelet-LHL	glszm	HighGrayLevelZoneEmphasis	281.0405405
wavelet-LHL	glszm	SmallAreaEmphasis	0.777965465
wavelet-LHL	glszm	LowGrayLevelZoneEmphasis	0.027091032
wavelet-LHL	glszm	ZoneEntropy	5.165072115
wavelet-LHL	glszm	SmallAreaLowGrayLevelEmphasis	0.024487736
wavelet-LHL	ngtdm	Coarseness	0.046777368
wavelet-LHL	ngtdm	Complexity	1506.405643
wavelet-LHL	ngtdm	Strength	13.19277143
wavelet-LHL	ngtdm	Contrast	0.543745411
wavelet-LHL	ngtdm	Busyness	0.055789891
wavelet-LHH	gldm	GrayLevelVariance	14.41529492
wavelet-LHH	gldm	HighGrayLevelEmphasis	71.22222222
wavelet-LHH	gldm	DependenceEntropy	5.033467104
wavelet-LHH	gldm	DependenceNonUniformity	31.51851852
wavelet-LHH	gldm	GrayLevelNonUniformity	9.87037037
wavelet-LHH	gldm	SmallDependenceEmphasis	0.444125514
wavelet-LHH	gldm	SmallDependenceHighGrayLevelEmphasis	42.95283951
wavelet-LHH	gldm	DependenceNonUniformityNormalized	0.291838134
wavelet-LHH	gldm	LargeDependenceEmphasis	5.703703704
wavelet-LHH	gldm	LargeDependenceLowGrayLevelEmphasis	0.346602111
wavelet-LHH	gldm	DependenceVariance	1.168381344
wavelet-LHH	gldm	LargeDependenceHighGrayLevelEmphasis	330.1111111
wavelet-LHH	gldm	SmallDependenceLowGrayLevelEmphasis	0.025372902
wavelet-LHH	gldm	LowGrayLevelEmphasis	0.076810774
wavelet-LHH	glcm	JointAverage	7.542238654
wavelet-LHH	glcm	SumAverage	15.08447731

wavelet-LHH	glcm	JointEntropy	6.17052066
wavelet-LHH	glcm	ClusterShade	53.17291812
wavelet-LHH	glcm	MaximumProbability	0.043871497
wavelet-LHH	glcm	Idmn	0.94360941
wavelet-LHH	glcm	JointEnergy	0.016629034
wavelet-LHH	glcm	Contrast	29.72039214
wavelet-LHH	glcm	DifferenceEntropy	3.320033665
wavelet-LHH	glcm	InverseVariance	0.214704938
wavelet-LHH	glcm	DifferenceVariance	10.61962772
wavelet-LHH	glcm	Idn	0.843561379
wavelet-LHH	glcm	Idm	0.204808551
wavelet-LHH	glcm	Correlation	-0.009653727
wavelet-LHH	glcm	Autocorrelation	56.70952628
wavelet-LHH	glcm	SumEntropy	4.072087822
wavelet-LHH	glcm	SumSquares	14.6745395
wavelet-LHH	glcm	ClusterProminence	2664.501712
wavelet-LHH	glcm	Imc2	0.957407263
wavelet-LHH	glcm	Imc1	-0.339972134
wavelet-LHH	glcm	DifferenceAverage	4.293801244
wavelet-LHH	glcm	Id	0.299300938
wavelet-LHH	glcm	ClusterTendency	28.97776587
wavelet-LHH	firstorder	InterquartileRange	127.0437763
wavelet-LHH	firstorder	Skewness	0.472863263
wavelet-LHH	firstorder	Uniformity	0.091392318
wavelet-LHH	firstorder	Median	5.398601485
wavelet-LHH	firstorder	Energy	967510.6341
wavelet-LHH	firstorder	RobustMeanAbsoluteDeviation	52.33717381
wavelet-LHH	firstorder	MeanAbsoluteDeviation	74.48998562
wavelet-LHH	firstorder	TotalEnergy	26122787.12
wavelet-LHH	firstorder	Maximum	330.4606659
wavelet-LHH	firstorder	RootMeanSquared	94.64899259
wavelet-LHH	firstorder	90Percentile	124.7901377
wavelet-LHH	firstorder	Minimum	-170.8416239
wavelet-LHH	firstorder	Entropy	3.724401479
wavelet-LHH	firstorder	Range	501.3022897
wavelet-LHH	firstorder	Variance	8958.427906
wavelet-LHH	firstorder	10Percentile	-124.3436195
wavelet-LHH	firstorder	Kurtosis	3.35500083
wavelet-LHH	firstorder	Mean	0.062382871
wavelet-LHH	glrlm	ShortRunLowGrayLevelEmphasis	0.074788637
wavelet-LHH	glrlm	GrayLevelVariance	14.73922563
wavelet-LHH	glrlm	LowGrayLevelRunEmphasis	0.077143757
wavelet-LHH	glrlm	GrayLevelNonUniformityNormalized	0.089033776
wavelet-LHH	glrlm	RunVariance	0.049367133
wavelet-LHH	glrlm	GrayLevelNonUniformity	9.200675994
wavelet-LHH	glrlm	LongRunEmphasis	1.143515654
wavelet-LHH	glrlm	ShortRunHighGrayLevelEmphasis	70.29499106
wavelet-LHH	glrlm	RunLengthNonUniformity	94.95034854
wavelet-LHH	glrlm	ShortRunEmphasis	0.967489447
wavelet-LHH	glrlm	LongRunHighGrayLevelEmphasis	79.74053882
wavelet-LHH	glrlm	RunPercentage	0.956552707
wavelet-LHH	glrlm	LongRunLowGrayLevelEmphasis	0.086815896
wavelet-LHH	glrlm	RunEntropy	3.924206581

wavelet-LHH	glrlm	HighGrayLevelRunEmphasis	72.03059627
wavelet-LHH	glrlm	RunLengthNonUniformityNormalized	0.918468334
wavelet-LHH	glszm	GrayLevelVariance	19.62468112
wavelet-LHH	glszm	ZoneVariance	5.280612245
wavelet-LHH	glszm	GrayLevelNonUniformityNormalized	0.070153061
wavelet-LHH	glszm	SizeZoneNonUniformityNormalized	0.439413265
wavelet-LHH	glszm	SizeZoneNonUniformity	24.60714286
wavelet-LHH	glszm	GrayLevelNonUniformity	3.928571429
wavelet-LHH	glszm	LargeAreaEmphasis	9
wavelet-LHH	glszm	SmallAreaHighGrayLevelEmphasis	74.11587913
wavelet-LHH	glszm	ZonePercentage	0.518518519
wavelet-LHH	glszm	LargeAreaLowGrayLevelEmphasis	0.400203488
wavelet-LHH	glszm	LargeAreaHighGrayLevelEmphasis	560.25
wavelet-LHH	glszm	HighGrayLevelZoneEmphasis	87.98214286
wavelet-LHH	glszm	SmallAreaEmphasis	0.685919924
wavelet-LHH	glszm	LowGrayLevelZoneEmphasis	0.075504181
wavelet-LHH	glszm	ZoneEntropy	4.76691452
wavelet-LHH	glszm	SmallAreaLowGrayLevelEmphasis	0.0321318
wavelet-LHH	ngtdm	Coarseness	0.044576322
wavelet-LHH	ngtdm	Complexity	373.7504438
wavelet-LHH	ngtdm	Strength	4.652602817
wavelet-LHH	ngtdm	Contrast	0.337117987
wavelet-LHH	ngtdm	Busyness	0.212975643
wavelet-LLH	gldm	GrayLevelVariance	62.31987311
wavelet-LLH	gldm	HighGrayLevelEmphasis	521.787037
wavelet-LLH	gldm	DependenceEntropy	5.582394998
wavelet-LLH	gldm	DependenceNonUniformity	40.27777778
wavelet-LLH	gldm	GrayLevelNonUniformity	4.796296296
wavelet-LLH	gldm	SmallDependenceEmphasis	0.573109568
wavelet-LLH	gldm	SmallDependenceHighGrayLevelEmphasis	287.3881173
wavelet-LLH	gldm	DependenceNonUniformityNormalized	0.372942387
wavelet-LLH	gldm	LargeDependenceEmphasis	3.722222222
wavelet-LLH	gldm	LargeDependenceLowGrayLevelEmphasis	0.022627291
wavelet-LLH	gldm	DependenceVariance	0.692043896
wavelet-LLH	gldm	LargeDependenceHighGrayLevelEmphasis	2170.787037
wavelet-LLH	gldm	SmallDependenceLowGrayLevelEmphasis	0.012988306
wavelet-LLH	gldm	LowGrayLevelEmphasis	0.014280006
wavelet-LLH	glcm	JointAverage	21.44302385
wavelet-LLH	glcm	SumAverage	42.88604769
wavelet-LLH	glcm	JointEntropy	6.687605363
wavelet-LLH	glcm	ClusterShade	-983.1814593
wavelet-LLH	glcm	MaximumProbability	0.023753883
wavelet-LLH	glcm	Idmn	0.947764326
wavelet-LLH	glcm	JointEnergy	0.010503567
wavelet-LLH	glcm	Contrast	66.01664424
wavelet-LLH	glcm	DifferenceEntropy	3.727948179
wavelet-LLH	glcm	InverseVariance	0.149894421
wavelet-LLH	glcm	DifferenceVariance	20.91748502
wavelet-LLH	glcm	Idn	0.847924257
wavelet-LLH	glcm	Idm	0.140100969
wavelet-LLH	glcm	Correlation	0.483415877
wavelet-LLH	glcm	Autocorrelation	491.9291044
wavelet-LLH	glcm	SumEntropy	4.961477382

wavelet-LLH	glcm	SumSquares	64.63966726
wavelet-LLH	glcm	ClusterProminence	83452.79539
wavelet-LLH	glcm	Imc2	0.99684638
wavelet-LLH	glcm	Imc1	-0.552911242
wavelet-LLH	glcm	DifferenceAverage	6.478238378
wavelet-LLH	glcm	Id	0.231642299
wavelet-LLH	glcm	ClusterTendency	192.5420248
wavelet-LLH	firstorder	InterquartileRange	323.7861948
wavelet-LLH	firstorder	Skewness	-0.410600163
wavelet-LLH	firstorder	Uniformity	0.044410151
wavelet-LLH	firstorder	Median	-29.7811619
wavelet-LLH	firstorder	Energy	4459132.027
wavelet-LLH	firstorder	RobustMeanAbsoluteDeviation	130.3166746
wavelet-LLH	firstorder	MeanAbsoluteDeviation	167.3448749
wavelet-LLH	firstorder	TotalEnergy	120396564.7
wavelet-LLH	firstorder	Maximum	234.5304497
wavelet-LLH	firstorder	RootMeanSquared	203.1951267
wavelet-LLH	firstorder	90Percentile	191.7497355
wavelet-LLH	firstorder	Minimum	-571.0494603
wavelet-LLH	firstorder	Entropy	4.647358893
wavelet-LLH	firstorder	Range	805.5799101
wavelet-LLH	firstorder	Variance	38582.45544
wavelet-LLH	firstorder	10Percentile	-301.5212687
wavelet-LLH	firstorder	Kurtosis	2.328909314
wavelet-LLH	firstorder	Mean	-52.01734396
wavelet-LLH	glrlm	ShortRunLowGrayLevelEmphasis	0.014560136
wavelet-LLH	glrlm	GrayLevelVariance	62.23303871
wavelet-LLH	glrlm	LowGrayLevelRunEmphasis	0.014621549
wavelet-LLH	glrlm	GrayLevelNonUniformityNormalized	0.044293419
wavelet-LLH	glrlm	RunVariance	0.033147296
wavelet-LLH	glrlm	GrayLevelNonUniformity	4.64768821
wavelet-LLH	glrlm	LongRunEmphasis	1.096048552
wavelet-LLH	glrlm	ShortRunHighGrayLevelEmphasis	507.1236295
wavelet-LLH	glrlm	RunLengthNonUniformity	99.49687223
wavelet-LLH	glrlm	ShortRunEmphasis	0.978686917
wavelet-LLH	glrlm	LongRunHighGrayLevelEmphasis	575.490704
wavelet-LLH	glrlm	RunPercentage	0.971509972
wavelet-LLH	glrlm	LongRunLowGrayLevelEmphasis	0.014925236
wavelet-LLH	glrlm	RunEntropy	4.73756378
wavelet-LLH	glrlm	HighGrayLevelRunEmphasis	519.8225055
wavelet-LLH	glrlm	RunLengthNonUniformityNormalized	0.946746108
wavelet-LLH	glszm	GrayLevelVariance	63.82022894
wavelet-LLH	glszm	ZoneVariance	0.742728467
wavelet-LLH	glszm	GrayLevelNonUniformityNormalized	0.042972415
wavelet-LLH	glszm	SizeZoneNonUniformityNormalized	0.520735598
wavelet-LLH	glszm	SizeZoneNonUniformity	38.01369863
wavelet-LLH	glszm	GrayLevelNonUniformity	3.136986301
wavelet-LLH	glszm	LargeAreaEmphasis	2.931506849
wavelet-LLH	glszm	SmallAreaHighGrayLevelEmphasis	374.0973135
wavelet-LLH	glszm	ZonePercentage	0.675925926
wavelet-LLH	glszm	LargeAreaLowGrayLevelEmphasis	0.026032015
wavelet-LLH	glszm	LargeAreaHighGrayLevelEmphasis	1595.767123
wavelet-LLH	glszm	HighGrayLevelZoneEmphasis	506.5479452

wavelet-LLH	glszm	SmallAreaEmphasis	0.747408676
wavelet-LLH	glszm	LowGrayLevelZoneEmphasis	0.01963339
wavelet-LLH	glszm	ZoneEntropy	5.381337572
wavelet-LLH	glszm	SmallAreaLowGrayLevelEmphasis	0.018892633
wavelet-LLH	ngtdm	Coarseness	0.053727115
wavelet-LLH	ngtdm	Complexity	1562.303775
wavelet-LLH	ngtdm	Strength	22.18229702
wavelet-LLH	ngtdm	Contrast	0.569782072
wavelet-LLH	ngtdm	Busyness	0.032108076
wavelet-LLL	gldm	GrayLevelVariance	482.861797
wavelet-LLL	gldm	HighGrayLevelEmphasis	1785.537037
wavelet-LLL	gldm	DependenceEntropy	6.03266528
wavelet-LLL	gldm	DependenceNonUniformity	70.66666667
wavelet-LLL	gldm	GrayLevelNonUniformity	2.166666667
wavelet-LLL	gldm	SmallDependenceEmphasis	0.833333333
wavelet-LLL	gldm	SmallDependenceHighGrayLevelEmphasis	1455.759259
wavelet-LLL	gldm	DependenceNonUniformityNormalized	0.654320988
wavelet-LLL	gldm	LargeDependenceEmphasis	1.666666667
wavelet-LLL	gldm	LargeDependenceLowGrayLevelEmphasis	0.014495496
wavelet-LLL	gldm	DependenceVariance	0.172839506
wavelet-LLL	gldm	LargeDependenceHighGrayLevelEmphasis	3104.648148
wavelet-LLL	gldm	SmallDependenceLowGrayLevelEmphasis	0.013191401
wavelet-LLL	gldm	LowGrayLevelEmphasis	0.01345222
wavelet-LLL	glcm	JointAverage	42.4479866
wavelet-LLL	glcm	SumAverage	84.89597321
wavelet-LLL	glcm	JointEntropy	6.95128779
wavelet-LLL	glcm	ClusterShade	19204.74165
wavelet-LLL	glcm	MaximumProbability	0.01471801
wavelet-LLL	glcm	Idmn	0.941742577
wavelet-LLL	glcm	JointEnergy	0.008273869
wavelet-LLL	glcm	Contrast	681.6614667
wavelet-LLL	glcm	DifferenceEntropy	4.972806369
wavelet-LLL	glcm	InverseVariance	0.05837112
wavelet-LLL	glcm	DifferenceVariance	222.1847079
wavelet-LLL	glcm	Idn	0.838258213
wavelet-LLL	glcm	Idm	0.048658858
wavelet-LLL	glcm	Correlation	0.36805102
wavelet-LLL	glcm	Autocorrelation	1996.188394
wavelet-LLL	glcm	SumEntropy	5.595781637
wavelet-LLL	glcm	SumSquares	532.9896738
wavelet-LLL	glcm	ClusterProminence	5133450.626
wavelet-LLL	glcm	Imc2	0.999932755
wavelet-LLL	glcm	Imc1	-0.78407939
wavelet-LLL	glcm	DifferenceAverage	21.10231643
wavelet-LLL	glcm	Id	0.104177524
wavelet-LLL	glcm	ClusterTendency	1450.297229
wavelet-LLL	firstorder	InterquartileRange	788.1868138
wavelet-LLL	firstorder	Skewness	0.696255992
wavelet-LLL	firstorder	Uniformity	0.020061728
wavelet-LLL	firstorder	Median	-1663.14799
wavelet-LLL	firstorder	Energy	287250391.1
wavelet-LLL	firstorder	RobustMeanAbsoluteDeviation	324.199099
wavelet-LLL	firstorder	MeanAbsoluteDeviation	449.9604674

wavelet-LLL	firstorder	TotalEnergy	7755760559
wavelet-LLL	firstorder	Maximum	57.28774842
wavelet-LLL	firstorder	RootMeanSquared	1630.866593
wavelet-LLL	firstorder	90Percentile	-791.9379591
wavelet-LLL	firstorder	Minimum	-2414.402377
wavelet-LLL	firstorder	Entropy	5.791562502
wavelet-LLL	firstorder	Range	2471.690125
wavelet-LLL	firstorder	Variance	301349.8514
wavelet-LLL	firstorder	10Percentile	-2140.170695
wavelet-LLL	firstorder	Kurtosis	2.821541444
wavelet-LLL	firstorder	Mean	-1535.700489
wavelet-LLL	glrlm	ShortRunLowGrayLevelEmphasis	0.013545713
wavelet-LLL	glrlm	GrayLevelVariance	483.5219246
wavelet-LLL	glrlm	LowGrayLevelRunEmphasis	0.013555933
wavelet-LLL	glrlm	GrayLevelNonUniformityNormalized	0.020030042
wavelet-LLL	glrlm	RunVariance	0.008542764
wavelet-LLL	glrlm	GrayLevelNonUniformity	2.144828228
wavelet-LLL	glrlm	LongRunEmphasis	1.026167837
wavelet-LLL	glrlm	ShortRunHighGrayLevelEmphasis	1770.944573
wavelet-LLL	glrlm	RunLengthNonUniformity	105.2686996
wavelet-LLL	glrlm	ShortRunEmphasis	0.993458041
wavelet-LLL	glrlm	LongRunHighGrayLevelEmphasis	1835.588169
wavelet-LLL	glrlm	RunPercentage	0.991452991
wavelet-LLL	glrlm	LongRunLowGrayLevelEmphasis	0.013596811
wavelet-LLL	glrlm	RunEntropy	5.807111361
wavelet-LLL	glrlm	HighGrayLevelRunEmphasis	1783.873292
wavelet-LLL	glrlm	RunLengthNonUniformityNormalized	0.982914472
wavelet-LLL	glszm	GrayLevelVariance	492.2552083
wavelet-LLL	glszm	ZoneVariance	0.109375
wavelet-LLL	glszm	GrayLevelNonUniformityNormalized	0.019314236
wavelet-LLL	glszm	SizeZoneNonUniformityNormalized	0.78125
wavelet-LLL	glszm	SizeZoneNonUniformity	75
wavelet-LLL	glszm	GrayLevelNonUniformity	1.854166667
wavelet-LLL	glszm	LargeAreaEmphasis	1.375
wavelet-LLL	glszm	SmallAreaHighGrayLevelEmphasis	1575.895833
wavelet-LLL	glszm	ZonePercentage	0.888888889
wavelet-LLL	glszm	LargeAreaLowGrayLevelEmphasis	0.015524976
wavelet-LLL	glszm	LargeAreaHighGrayLevelEmphasis	2503.395833
wavelet-LLL	glszm	HighGrayLevelZoneEmphasis	1761.395833
wavelet-LLL	glszm	SmallAreaEmphasis	0.90625
wavelet-LLL	glszm	LowGrayLevelZoneEmphasis	0.014938134
wavelet-LLL	glszm	ZoneEntropy	6.022462501
wavelet-LLL	glszm	SmallAreaLowGrayLevelEmphasis	0.014791423
wavelet-LLL	ngtdm	Coarseness	0.03681095
wavelet-LLL	ngtdm	Complexity	24693.18768
wavelet-LLL	ngtdm	Strength	97.87014333
wavelet-LLL	ngtdm	Contrast	3.199564287
wavelet-LLL	ngtdm	Busyness	0.017100564
wavelet-HHL	gldm	GrayLevelVariance	28.33950617
wavelet-HHL	gldm	HighGrayLevelEmphasis	212.0925926
wavelet-HHL	gldm	DependenceEntropy	5.488515688
wavelet-HHL	gldm	DependenceNonUniformity	31.2962963
wavelet-HHL	gldm	GrayLevelNonUniformity	7.722222222

wavelet-HHL	gldm	SmallDependenceEmphasis	0.459025206
wavelet-HHL	gldm	SmallDependenceHighGrayLevelEmphasis	105.4984851
wavelet-HHL	gldm	DependenceNonUniformityNormalized	0.289780521
wavelet-HHL	gldm	LargeDependenceEmphasis	5.481481481
wavelet-HHL	gldm	LargeDependenceLowGrayLevelEmphasis	0.05665539
wavelet-HHL	gldm	DependenceVariance	1.102537723
wavelet-HHL	gldm	LargeDependenceHighGrayLevelEmphasis	1069.407407
wavelet-HHL	gldm	SmallDependenceLowGrayLevelEmphasis	0.016044449
wavelet-HHL	gldm	LowGrayLevelEmphasis	0.020919293
wavelet-HHL	glcm	JointAverage	13.60507307
wavelet-HHL	glcm	SumAverage	27.21014613
wavelet-HHL	glcm	JointEntropy	6.486705974
wavelet-HHL	glcm	ClusterShade	65.03373038
wavelet-HHL	glcm	MaximumProbability	0.029703124
wavelet-HHL	glcm	Idmn	0.948536571
wavelet-HHL	glcm	JointEnergy	0.012548713
wavelet-HHL	glcm	Contrast	59.95376392
wavelet-HHL	glcm	DifferenceEntropy	3.701927864
wavelet-HHL	glcm	InverseVariance	0.193162264
wavelet-HHL	glcm	DifferenceVariance	24.88491607
wavelet-HHL	glcm	Idn	0.857410864
wavelet-HHL	glcm	Idm	0.185184588
wavelet-HHL	glcm	Correlation	-0.023700836
wavelet-HHL	glcm	Autocorrelation	184.3889617
wavelet-HHL	glcm	SumEntropy	4.377179879
wavelet-HHL	glcm	SumSquares	29.24392752
wavelet-HHL	glcm	ClusterProminence	12347.3019
wavelet-HHL	glcm	Imc2	0.982132404
wavelet-HHL	glcm	Imc1	-0.413884359
wavelet-HHL	glcm	DifferenceAverage	5.781603506
wavelet-HHL	glcm	Id	0.272875038
wavelet-HHL	glcm	ClusterTendency	57.02194614
wavelet-HHL	firstorder	InterquartileRange	122.9245352
wavelet-HHL	firstorder	Skewness	0.411222613
wavelet-HHL	firstorder	Uniformity	0.071502058
wavelet-HHL	firstorder	Median	-2.348332645
wavelet-HHL	firstorder	Energy	1872086.242
wavelet-HHL	firstorder	RobustMeanAbsoluteDeviation	59.05781934
wavelet-HHL	firstorder	MeanAbsoluteDeviation	96.33114901
wavelet-HHL	firstorder	TotalEnergy	50546328.53
wavelet-HHL	firstorder	Maximum	430.3419204
wavelet-HHL	firstorder	RootMeanSquared	131.6591503
wavelet-HHL	firstorder	90Percentile	154.7691241
wavelet-HHL	firstorder	Minimum	-308.6657658
wavelet-HHL	firstorder	Entropy	4.067205196
wavelet-HHL	firstorder	Range	739.0076863
wavelet-HHL	firstorder	Variance	17333.5689
wavelet-HHL	firstorder	10Percentile	-176.1473972
wavelet-HHL	firstorder	Kurtosis	3.942480521
wavelet-HHL	firstorder	Mean	0.750312563
wavelet-HHL	glrlm	ShortRunLowGrayLevelEmphasis	0.021227856
wavelet-HHL	glrlm	GrayLevelVariance	28.99143456
wavelet-HHL	glrlm	LowGrayLevelRunEmphasis	0.021477405

wavelet-HHL	glrlm	GrayLevelNonUniformityNormalized	0.070530834
wavelet-HHL	glrlm	RunVariance	0.049619005
wavelet-HHL	glrlm	GrayLevelNonUniformity	7.297509175
wavelet-HHL	glrlm	LongRunEmphasis	1.143438914
wavelet-HHL	glrlm	ShortRunHighGrayLevelEmphasis	206.8876602
wavelet-HHL	glrlm	RunLengthNonUniformity	95.61761119
wavelet-HHL	glrlm	ShortRunEmphasis	0.968556984
wavelet-HHL	glrlm	LongRunHighGrayLevelEmphasis	241.0068474
wavelet-HHL	glrlm	RunPercentage	0.957977208
wavelet-HHL	glrlm	LongRunLowGrayLevelEmphasis	0.02276978
wavelet-HHL	glrlm	RunEntropy	4.241907777
wavelet-HHL	glrlm	HighGrayLevelRunEmphasis	212.9077594
wavelet-HHL	glrlm	RunLengthNonUniformityNormalized	0.922343128
wavelet-HHL	glszm	GrayLevelVariance	38.26083333
wavelet-HHL	glszm	ZoneVariance	1.593333333
wavelet-HHL	glszm	GrayLevelNonUniformityNormalized	0.058888889
wavelet-HHL	glszm	SizeZoneNonUniformityNormalized	0.430555556
wavelet-HHL	glszm	SizeZoneNonUniformity	25.83333333
wavelet-HHL	glszm	GrayLevelNonUniformity	3.533333333
wavelet-HHL	glszm	LargeAreaEmphasis	4.833333333
wavelet-HHL	glszm	SmallAreaHighGrayLevelEmphasis	159.6652376
wavelet-HHL	glszm	ZonePercentage	0.555555556
wavelet-HHL	glszm	LargeAreaLowGrayLevelEmphasis	0.062648109
wavelet-HHL	glszm	LargeAreaHighGrayLevelEmphasis	966.1666667
wavelet-HHL	glszm	HighGrayLevelZoneEmphasis	224.5833333
wavelet-HHL	glszm	SmallAreaEmphasis	0.67441421
wavelet-HHL	glszm	LowGrayLevelZoneEmphasis	0.030453383
wavelet-HHL	glszm	ZoneEntropy	5.177316637
wavelet-HHL	glszm	SmallAreaLowGrayLevelEmphasis	0.027348182
wavelet-HHL	ngtdm	Coarseness	0.050981016
wavelet-HHL	ngtdm	Complexity	956.056885
wavelet-HHL	ngtdm	Strength	10.04637765
wavelet-HHL	ngtdm	Contrast	0.456245823
wavelet-HHL	ngtdm	Busyness	0.068187061
wavelet-HLH	gldm	GrayLevelVariance	16.97085048
wavelet-HLH	gldm	HighGrayLevelEmphasis	128
wavelet-HLH	gldm	DependenceEntropy	5.397772456
wavelet-HLH	gldm	DependenceNonUniformity	28.11111111
wavelet-HLH	gldm	GrayLevelNonUniformity	8.685185185
wavelet-HLH	gldm	SmallDependenceEmphasis	0.496651077
wavelet-HLH	gldm	SmallDependenceHighGrayLevelEmphasis	76.16073549
wavelet-HLH	gldm	DependenceNonUniformityNormalized	0.260288066
wavelet-HLH	gldm	LargeDependenceEmphasis	7.944444444
wavelet-HLH	gldm	LargeDependenceLowGrayLevelEmphasis	0.111976385
wavelet-HLH	gldm	DependenceVariance	2.413237311
wavelet-HLH	gldm	LargeDependenceHighGrayLevelEmphasis	798.9259259
wavelet-HLH	gldm	SmallDependenceLowGrayLevelEmphasis	0.018077742
wavelet-HLH	gldm	LowGrayLevelEmphasis	0.025719993
wavelet-HLH	glcm	JointAverage	10.62814983
wavelet-HLH	glcm	SumAverage	21.25629966
wavelet-HLH	glcm	JointEntropy	6.296226284
wavelet-HLH	glcm	ClusterShade	63.70673086
wavelet-HLH	glcm	MaximumProbability	0.039646531

wavelet-HLH	glcm	Idmn	0.939514809
wavelet-HLH	glcm	JointEnergy	0.015087221
wavelet-HLH	glcm	Contrast	32.11331061
wavelet-HLH	glcm	DifferenceEntropy	3.411744962
wavelet-HLH	glcm	InverseVariance	0.215321824
wavelet-HLH	glcm	DifferenceVariance	11.83031879
wavelet-HLH	glcm	Idn	0.840594843
wavelet-HLH	glcm	Idm	0.216073762
wavelet-HLH	glcm	Correlation	0.008173649
wavelet-HLH	glcm	Autocorrelation	113.4538303
wavelet-HLH	glcm	SumEntropy	4.123768464
wavelet-HLH	glcm	SumSquares	16.42318532
wavelet-HLH	glcm	ClusterProminence	3677.633371
wavelet-HLH	glcm	Imc2	0.972461301
wavelet-HLH	glcm	Imc1	-0.384129442
wavelet-HLH	glcm	DifferenceAverage	4.42706054
wavelet-HLH	glcm	Id	0.306332088
wavelet-HLH	glcm	ClusterTendency	33.57943065
wavelet-HLH	firstorder	InterquartileRange	114.906577
wavelet-HLH	firstorder	Skewness	0.318929834
wavelet-HLH	firstorder	Uniformity	0.080418381
wavelet-HLH	firstorder	Median	-10.26725591
wavelet-HLH	firstorder	Energy	1158221.847
wavelet-HLH	firstorder	RobustMeanAbsoluteDeviation	52.07263325
wavelet-HLH	firstorder	MeanAbsoluteDeviation	79.82321949
wavelet-HLH	firstorder	TotalEnergy	31271989.87
wavelet-HLH	firstorder	Maximum	265.1796799
wavelet-HLH	firstorder	RootMeanSquared	103.5580821
wavelet-HLH	firstorder	90Percentile	143.4986008
wavelet-HLH	firstorder	Minimum	-227.9130539
wavelet-HLH	firstorder	Entropy	3.908958413
wavelet-HLH	firstorder	Range	493.0927338
wavelet-HLH	firstorder	Variance	10723.85109
wavelet-HLH	firstorder	10Percentile	-130.4699855
wavelet-HLH	firstorder	Kurtosis	3.023030923
wavelet-HLH	firstorder	Mean	0.652127229
wavelet-HLH	glrlm	ShortRunLowGrayLevelEmphasis	0.025900971
wavelet-HLH	glrlm	GrayLevelVariance	17.64000013
wavelet-HLH	glrlm	LowGrayLevelRunEmphasis	0.026417261
wavelet-HLH	glrlm	GrayLevelNonUniformityNormalized	0.078028622
wavelet-HLH	glrlm	RunVariance	0.061107032
wavelet-HLH	glrlm	GrayLevelNonUniformity	7.991451711
wavelet-HLH	glrlm	LongRunEmphasis	1.176113292
wavelet-HLH	glrlm	ShortRunHighGrayLevelEmphasis	125.6500356
wavelet-HLH	glrlm	RunLengthNonUniformity	92.52714892
wavelet-HLH	glrlm	ShortRunEmphasis	0.960781094
wavelet-HLH	glrlm	LongRunHighGrayLevelEmphasis	146.854503
wavelet-HLH	glrlm	RunPercentage	0.948005698
wavelet-HLH	glrlm	LongRunLowGrayLevelEmphasis	0.028741383
wavelet-HLH	glrlm	RunEntropy	4.143226086
wavelet-HLH	glrlm	HighGrayLevelRunEmphasis	129.5968052
wavelet-HLH	glrlm	RunLengthNonUniformityNormalized	0.902693909
wavelet-HLH	glszm	GrayLevelVariance	24.24993701

wavelet-HLH	glszm	ZoneVariance	2.712018141
wavelet-HLH	glszm	GrayLevelNonUniformityNormalized	0.06122449
wavelet-HLH	glszm	SizeZoneNonUniformityNormalized	0.536407155
wavelet-HLH	glszm	SizeZoneNonUniformity	33.79365079
wavelet-HLH	glszm	GrayLevelNonUniformity	3.857142857
wavelet-HLH	glszm	LargeAreaEmphasis	5.650793651
wavelet-HLH	glszm	SmallAreaHighGrayLevelEmphasis	120.6645237
wavelet-HLH	glszm	ZonePercentage	0.583333333
wavelet-HLH	glszm	LargeAreaLowGrayLevelEmphasis	0.094321746
wavelet-HLH	glszm	LargeAreaHighGrayLevelEmphasis	589.8095238
wavelet-HLH	glszm	HighGrayLevelZoneEmphasis	146.6507937
wavelet-HLH	glszm	SmallAreaEmphasis	0.759488266
wavelet-HLH	glszm	LowGrayLevelZoneEmphasis	0.033800633
wavelet-HLH	glszm	ZoneEntropy	4.972768614
wavelet-HLH	glszm	SmallAreaLowGrayLevelEmphasis	0.029400548
wavelet-HLH	ngtdm	Coarseness	0.048781303
wavelet-HLH	ngtdm	Complexity	487.1054275
wavelet-HLH	ngtdm	Strength	5.88121162
wavelet-HLH	ngtdm	Contrast	0.292789583
wavelet-HLH	ngtdm	Busyness	0.114004275

Image type	Feature Class	Feature Name	4: RTSTRUCT: C99-DNU_segment_GTV
general	info	BoundingBox	(1, 0, 0, 9, 13, 24)
general	info	EnabledImageTypes	{u'Original': {}, u'Wavelet': {}}
general	info	GeneralSettings	{'distances': [1], 'additionalInfo': True, 'force2D': False, 'interpolator': 'sitkBSpline', 'resampledPixelSpacing': [3.0], 'label': 1, 'normalizeScale': 1, 'normalize': False, 'force2Ddimension': 0, 'removeOutliers': None, 'minimumROISize': None, u'binWidth': 25.0, 'minimumROIDimensions': 1, u'correctMask': True, 'preCrop': False, u'symmetricalGLCM': True, 'resegmentRange': None, 'padDistance': 5}
general	info	ImageHash	ea31af20167ef08dc6aa5b7621a600b8085ca9ba
general	info	ImageSpacing	(0.9765624999999998, 0.9765624999999998, 1.9999999999999996)
general	info	MaskHash	daef1add07c8c8d9d1b302b7789fa22c00595b3f
general	info	NumpyVersion	1.13.1
general	info	PyWaveletVersion	0.5.2
general	info	SimpleITKVersion	1.1.0
general	info	Version	1.3.0.post102+g1b0eb74
general	info	VolumeNum	1
general	info	VoxelNum	876
original	shape	Maximum3DDiameter	71.81225522
original	shape	Maximum2DDiameterSlice	30
original	shape	Sphericity	0.632903127
original	shape	MinorAxis	27.79280797
original	shape	Elongation	0.307012037
original	shape	SurfaceVolumeRatio	0.266189504
original	shape	Volume	23652
original	shape	MajorAxis	90.52676974
original	shape	SurfaceArea	6295.914137
original	shape	Flatness	0.22402172
original	shape	LeastAxis	20.27996262
original	shape	Maximum2DDiameterColumn	70.61161378
original	shape	Maximum2DDiameterRow	71.30918594
original	gldm	GrayLevelVariance	99.23273467
original	gldm	HighGrayLevelEmphasis	1193.28653
original	gldm	DependenceEntropy	6.687263549
original	gldm	DependenceNonUniformity	253.3196347
original	gldm	GrayLevelNonUniformity	33.69634703
original	gldm	SmallDependenceEmphasis	0.528369116
original	gldm	SmallDependenceHighGrayLevelEmphasis	516.1245179
original	gldm	DependenceNonUniformityNormalized	0.289177665
original	gldm	LargeDependenceEmphasis	11.29680365
original	gldm	LargeDependenceLowGrayLevelEmphasis	0.010642565
original	gldm	DependenceVariance	5.183039344
original	gldm	LargeDependenceHighGrayLevelEmphasis	20048.77511

original	gldm	SmallDependenceLowGrayLevelEmphasis	0.003594954
original	gldm	LowGrayLevelEmphasis	0.004081027
original	glcm	JointAverage	34.04221583
original	glcm	SumAverage	68.08443167
original	glcm	JointEntropy	8.803216877
original	glcm	ClusterShade	-2853.112573
original	glcm	MaximumProbability	0.027408169
original	glcm	Idmn	0.956155577
original	glcm	JointEnergy	0.00442688
original	glcm	Contrast	125.8088994
original	glcm	DifferenceEntropy	4.45585701
original	glcm	InverseVariance	0.173184769
original	glcm	DifferenceVariance	59.58821044
original	glcm	Idn	0.872170695
original	glcm	Idm	0.177568227
original	glcm	Correlation	0.390989246
original	glcm	Autocorrelation	1199.038048
original	glcm	SumEntropy	5.770555478
original	glcm	SumSquares	103.0455625
original	glcm	ClusterProminence	214677.4012
original	glcm	Imc2	0.937080214
original	glcm	Imc1	-0.214088549
original	glcm	DifferenceAverage	8.095364426
original	glcm	Id	0.253437221
original	glcm	ClusterTendency	286.3733505
original	firstorder	InterquartileRange	399.25
original	firstorder	Skewness	-0.644158423
original	firstorder	Uniformity	0.03846615
original	firstorder	Median	-231
original	firstorder	Energy	114151711
original	firstorder	RobustMeanAbsoluteDeviation	163.7305298
original	firstorder	MeanAbsoluteDeviation	210.5360449
original	firstorder	TotalEnergy	3082096197
original	firstorder	Maximum	133
original	firstorder	RootMeanSquared	360.985003
original	firstorder	90Percentile	23
original	firstorder	Minimum	-1056
original	firstorder	Entropy	5.007005577
original	firstorder	Range	1189
original	firstorder	Variance	62010.88464
original	firstorder	10Percentile	-590
original	firstorder	Kurtosis	2.685328607
original	firstorder	Mean	-261.3413242
original	glrlm	ShortRunLowGrayLevelEmphasis	0.004248173
original	glrlm	GrayLevelVariance	98.44449935
original	glrlm	LowGrayLevelRunEmphasis	0.00428014
original	glrlm	GrayLevelNonUniformityNormalized	0.03574623
original	glrlm	RunVariance	0.075706231
original	glrlm	GrayLevelNonUniformity	29.54479843
original	glrlm	LongRunEmphasis	1.199869817
original	glrlm	ShortRunHighGrayLevelEmphasis	1097.042554
original	glrlm	RunLengthNonUniformity	744.6016305
original	glrlm	ShortRunEmphasis	0.959957985

original	glrlm	LongRunHighGrayLevelEmphasis	1505.108354
original	glrlm	RunPercentage	0.943361433
original	glrlm	LongRunLowGrayLevelEmphasis	0.004430599
original	glrlm	RunEntropy	5.300771644
original	glrlm	HighGrayLevelRunEmphasis	1163.544187
original	glrlm	RunLengthNonUniformityNormalized	0.900842424
original	glszm	GrayLevelVariance	91.43629002
original	glszm	ZoneVariance	12.09809867
original	glszm	GrayLevelNonUniformityNormalized	0.031133838
original	glszm	SizeZoneNonUniformityNormalized	0.577270935
original	glszm	SizeZoneNonUniformity	300.7581574
original	glszm	GrayLevelNonUniformity	16.22072937
original	glszm	LargeAreaEmphasis	14.92514395
original	glszm	SmallAreaHighGrayLevelEmphasis	741.6554604
original	glszm	ZonePercentage	0.594748858
original	glszm	LargeAreaLowGrayLevelEmphasis	0.014508132
original	glszm	LargeAreaHighGrayLevelEmphasis	27050.61228
original	glszm	HighGrayLevelZoneEmphasis	989.2495202
original	glszm	SmallAreaEmphasis	0.78813641
original	glszm	LowGrayLevelZoneEmphasis	0.006205858
original	glszm	ZoneEntropy	6.193424511
original	glszm	SmallAreaLowGrayLevelEmphasis	0.005923459
original	ngtdm	Coarseness	0.006087012
original	ngtdm	Complexity	5652.519617
original	ngtdm	Strength	6.637933123
original	ngtdm	Contrast	0.508141404
original	ngtdm	Busyness	0.088947289
wavelet-HHH	gldm	GrayLevelVariance	21.75450366
wavelet-HHH	gldm	HighGrayLevelEmphasis	569.5068493
wavelet-HHH	gldm	DependenceEntropy	6.20019861
wavelet-HHH	gldm	DependenceNonUniformity	180.3424658
wavelet-HHH	gldm	GrayLevelNonUniformity	66.27853881
wavelet-HHH	gldm	SmallDependenceEmphasis	0.402143859
wavelet-HHH	gldm	SmallDependenceHighGrayLevelEmphasis	237.9667989
wavelet-HHH	gldm	DependenceNonUniformityNormalized	0.205870395
wavelet-HHH	gldm	LargeDependenceEmphasis	11.55251142
wavelet-HHH	gldm	LargeDependenceLowGrayLevelEmphasis	0.023010057
wavelet-HHH	gldm	DependenceVariance	3.806837847
wavelet-HHH	gldm	LargeDependenceHighGrayLevelEmphasis	6396.203196
wavelet-HHH	gldm	SmallDependenceLowGrayLevelEmphasis	0.002145359
wavelet-HHH	gldm	LowGrayLevelEmphasis	0.003305758
wavelet-HHH	glcm	JointAverage	23.45859771
wavelet-HHH	glcm	SumAverage	46.91719542
wavelet-HHH	glcm	JointEntropy	7.651004698
wavelet-HHH	glcm	ClusterShade	-8.325939425
wavelet-HHH	glcm	MaximumProbability	0.027403984
wavelet-HHH	glcm	Idmn	0.981045818
wavelet-HHH	glcm	JointEnergy	0.008090852
wavelet-HHH	glcm	Contrast	40.3493523
wavelet-HHH	glcm	DifferenceEntropy	3.677773571
wavelet-HHH	glcm	InverseVariance	0.232215655
wavelet-HHH	glcm	DifferenceVariance	19.21127807
wavelet-HHH	glcm	Idn	0.91270578

wavelet-HHH	glcm	Idm	0.231491652
wavelet-HHH	glcm	Correlation	-0.027818313
wavelet-HHH	glcm	Autocorrelation	549.7872766
wavelet-HHH	glcm	SumEntropy	4.51089184
wavelet-HHH	glcm	SumSquares	19.65277391
wavelet-HHH	glcm	ClusterProminence	8032.02363
wavelet-HHH	glcm	Imc2	0.783088874
wavelet-HHH	glcm	Imc1	-0.117767004
wavelet-HHH	glcm	DifferenceAverage	4.560449464
wavelet-HHH	glcm	Id	0.318849129
wavelet-HHH	glcm	ClusterTendency	38.26174334
wavelet-HHH	firstorder	InterquartileRange	117.8266487
wavelet-HHH	firstorder	Skewness	-0.035107484
wavelet-HHH	firstorder	Uniformity	0.075660432
wavelet-HHH	firstorder	Median	-3.831928935
wavelet-HHH	firstorder	Energy	11943457.98
wavelet-HHH	firstorder	RobustMeanAbsoluteDeviation	50.33821806
wavelet-HHH	firstorder	MeanAbsoluteDeviation	83.59011825
wavelet-HHH	firstorder	TotalEnergy	322473365.5
wavelet-HHH	firstorder	Maximum	505.3836829
wavelet-HHH	firstorder	RootMeanSquared	116.7650823
wavelet-HHH	firstorder	90Percentile	131.3405986
wavelet-HHH	firstorder	Minimum	-569.3292807
wavelet-HHH	firstorder	Entropy	4.131771249
wavelet-HHH	firstorder	Range	1074.712964
wavelet-HHH	firstorder	Variance	13627.20905
wavelet-HHH	firstorder	10Percentile	-127.5522523
wavelet-HHH	firstorder	Kurtosis	5.315167188
wavelet-HHH	firstorder	Mean	-2.622098434
wavelet-HHH	glrlm	ShortRunLowGrayLevelEmphasis	0.003311954
wavelet-HHH	glrlm	GrayLevelVariance	22.93558516
wavelet-HHH	glrlm	LowGrayLevelRunEmphasis	0.003410047
wavelet-HHH	glrlm	GrayLevelNonUniformityNormalized	0.072002233
wavelet-HHH	glrlm	RunVariance	0.080841701
wavelet-HHH	glrlm	GrayLevelNonUniformity	58.74977687
wavelet-HHH	glrlm	LongRunEmphasis	1.234111237
wavelet-HHH	glrlm	ShortRunHighGrayLevelEmphasis	542.2166314
wavelet-HHH	glrlm	RunLengthNonUniformity	712.7396325
wavelet-HHH	glrlm	ShortRunEmphasis	0.948364574
wavelet-HHH	glrlm	LongRunHighGrayLevelEmphasis	700.2396511
wavelet-HHH	glrlm	RunPercentage	0.931419038
wavelet-HHH	glrlm	LongRunLowGrayLevelEmphasis	0.003852067
wavelet-HHH	glrlm	RunEntropy	4.517312091
wavelet-HHH	glrlm	HighGrayLevelRunEmphasis	570.7356169
wavelet-HHH	glrlm	RunLengthNonUniformityNormalized	0.873250913
wavelet-HHH	glszm	GrayLevelVariance	39.27826296
wavelet-HHH	glszm	ZoneVariance	23.37859225
wavelet-HHH	glszm	GrayLevelNonUniformityNormalized	0.050317576
wavelet-HHH	glszm	SizeZoneNonUniformityNormalized	0.506689921
wavelet-HHH	glszm	SizeZoneNonUniformity	198.622449
wavelet-HHH	glszm	GrayLevelNonUniformity	19.7244898
wavelet-HHH	glszm	LargeAreaEmphasis	28.37244898
wavelet-HHH	glszm	SmallAreaHighGrayLevelEmphasis	442.8459296

wavelet-HHH	glszm	ZonePercentage	0.447488584
wavelet-HHH	glszm	LargeAreaLowGrayLevelEmphasis	0.055717354
wavelet-HHH	glszm	LargeAreaHighGrayLevelEmphasis	15611.78061
wavelet-HHH	glszm	HighGrayLevelZoneEmphasis	589.252551
wavelet-HHH	glszm	SmallAreaEmphasis	0.739012509
wavelet-HHH	glszm	LowGrayLevelZoneEmphasis	0.005014503
wavelet-HHH	glszm	ZoneEntropy	5.845210321
wavelet-HHH	glszm	SmallAreaLowGrayLevelEmphasis	0.004478878
wavelet-HHH	ngtdm	Coarseness	0.008087421
wavelet-HHH	ngtdm	Complexity	1770.714759
wavelet-HHH	ngtdm	Strength	3.227133502
wavelet-HHH	ngtdm	Contrast	0.127014752
wavelet-HHH	ngtdm	Busyness	0.119175642
wavelet-HLL	gldm	GrayLevelVariance	46.28760868
wavelet-HLL	gldm	HighGrayLevelEmphasis	403.652968
wavelet-HLL	gldm	DependenceEntropy	6.594503651
wavelet-HLL	gldm	DependenceNonUniformity	238.9360731
wavelet-HLL	gldm	GrayLevelNonUniformity	39.55251142
wavelet-HLL	gldm	SmallDependenceEmphasis	0.465686886
wavelet-HLL	gldm	SmallDependenceHighGrayLevelEmphasis	215.9901827
wavelet-HLL	gldm	DependenceNonUniformityNormalized	0.272758074
wavelet-HLL	gldm	LargeDependenceEmphasis	6.785388128
wavelet-HLL	gldm	LargeDependenceLowGrayLevelEmphasis	0.032266925
wavelet-HLL	gldm	DependenceVariance	1.840349451
wavelet-HLL	gldm	LargeDependenceHighGrayLevelEmphasis	2332.650685
wavelet-HLL	gldm	SmallDependenceLowGrayLevelEmphasis	0.00656672
wavelet-HLL	gldm	LowGrayLevelEmphasis	0.008839851
wavelet-HLL	glcm	JointAverage	18.4697743
wavelet-HLL	glcm	SumAverage	36.93954859
wavelet-HLL	glcm	JointEntropy	8.712599119
wavelet-HLL	glcm	ClusterShade	506.6117926
wavelet-HLL	glcm	MaximumProbability	0.010694084
wavelet-HLL	glcm	Idmn	0.966466921
wavelet-HLL	glcm	JointEnergy	0.003168914
wavelet-HLL	glcm	Contrast	82.28352506
wavelet-HLL	glcm	DifferenceEntropy	4.137163745
wavelet-HLL	glcm	InverseVariance	0.176128987
wavelet-HLL	glcm	DifferenceVariance	36.8467738
wavelet-HLL	glcm	Idn	0.885314336
wavelet-HLL	glcm	Idm	0.17017231
wavelet-HLL	glcm	Correlation	0.139931019
wavelet-HLL	glcm	Autocorrelation	348.1753594
wavelet-HLL	glcm	SumEntropy	5.300088903
wavelet-HLL	glcm	SumSquares	48.1702146
wavelet-HLL	glcm	ClusterProminence	45527.44577
wavelet-HLL	glcm	Imc2	0.894713889
wavelet-HLL	glcm	Imc1	-0.172212612
wavelet-HLL	glcm	DifferenceAverage	6.59362281
wavelet-HLL	glcm	Id	0.257050613
wavelet-HLL	glcm	ClusterTendency	110.3973333
wavelet-HLL	firstorder	InterquartileRange	202.4119929
wavelet-HLL	firstorder	Skewness	0.390972232
wavelet-HLL	firstorder	Uniformity	0.045151269

wavelet-HLL	firstorder	Median	-21.66921058
wavelet-HLL	firstorder	Energy	25467535.1
wavelet-HLL	firstorder	RobustMeanAbsoluteDeviation	85.11384008
wavelet-HLL	firstorder	MeanAbsoluteDeviation	129.8176891
wavelet-HLL	firstorder	TotalEnergy	687623447.8
wavelet-HLL	firstorder	Maximum	663.9303276
wavelet-HLL	firstorder	RootMeanSquared	170.5066821
wavelet-HLL	firstorder	90Percentile	215.7031568
wavelet-HLL	firstorder	Minimum	-469.0298626
wavelet-HLL	firstorder	Entropy	4.752025642
wavelet-HLL	firstorder	Range	1132.96019
wavelet-HLL	firstorder	Variance	28852.90382
wavelet-HLL	firstorder	10Percentile	-226.1142688
wavelet-HLL	firstorder	Kurtosis	3.620836612
wavelet-HLL	firstorder	Mean	-14.81974479
wavelet-HLL	glrlm	ShortRunLowGrayLevelEmphasis	0.008926685
wavelet-HLL	glrlm	GrayLevelVariance	47.4610618
wavelet-HLL	glrlm	LowGrayLevelRunEmphasis	0.00907349
wavelet-HLL	glrlm	GrayLevelNonUniformityNormalized	0.044402839
wavelet-HLL	glrlm	RunVariance	0.053985686
wavelet-HLL	glrlm	GrayLevelNonUniformity	37.06547805
wavelet-HLL	glrlm	LongRunEmphasis	1.155916364
wavelet-HLL	glrlm	ShortRunHighGrayLevelEmphasis	394.9676923
wavelet-HLL	glrlm	RunLengthNonUniformity	761.4247634
wavelet-HLL	glrlm	ShortRunEmphasis	0.96485344
wavelet-HLL	glrlm	LongRunHighGrayLevelEmphasis	459.7766513
wavelet-HLL	glrlm	RunPercentage	0.952932912
wavelet-HLL	glrlm	LongRunLowGrayLevelEmphasis	0.009711733
wavelet-HLL	glrlm	RunEntropy	5.018347742
wavelet-HLL	glrlm	HighGrayLevelRunEmphasis	406.8645748
wavelet-HLL	glrlm	RunLengthNonUniformityNormalized	0.91179644
wavelet-HLL	glszm	GrayLevelVariance	64.2382247
wavelet-HLL	glszm	ZoneVariance	5.566944425
wavelet-HLL	glszm	GrayLevelNonUniformityNormalized	0.036146812
wavelet-HLL	glszm	SizeZoneNonUniformityNormalized	0.474834051
wavelet-HLL	glszm	SizeZoneNonUniformity	226.0210084
wavelet-HLL	glszm	GrayLevelNonUniformity	17.20588235
wavelet-HLL	glszm	LargeAreaEmphasis	8.953781513
wavelet-HLL	glszm	SmallAreaHighGrayLevelEmphasis	347.0163288
wavelet-HLL	glszm	ZonePercentage	0.543378995
wavelet-HLL	glszm	LargeAreaLowGrayLevelEmphasis	0.041748414
wavelet-HLL	glszm	LargeAreaHighGrayLevelEmphasis	3146.781513
wavelet-HLL	glszm	HighGrayLevelZoneEmphasis	453.2226891
wavelet-HLL	glszm	SmallAreaEmphasis	0.715925841
wavelet-HLL	glszm	LowGrayLevelZoneEmphasis	0.012758522
wavelet-HLL	glszm	ZoneEntropy	6.296563482
wavelet-HLL	glszm	SmallAreaLowGrayLevelEmphasis	0.011477769
wavelet-HLL	ngtdm	Coarseness	0.008423331
wavelet-HLL	ngtdm	Complexity	3104.923386
wavelet-HLL	ngtdm	Strength	4.449008237
wavelet-HLL	ngtdm	Contrast	0.243723738
wavelet-HLL	ngtdm	Busyness	0.144322229
wavelet-LHL	gldm	GrayLevelVariance	74.77189148

wavelet-LHL	gldm	HighGrayLevelEmphasis	1486.592466
wavelet-LHL	gldm	DependenceEntropy	6.717611436
wavelet-LHL	gldm	DependenceNonUniformity	275.4063927
wavelet-LHL	gldm	GrayLevelNonUniformity	32.84703196
wavelet-LHL	gldm	SmallDependenceEmphasis	0.544952516
wavelet-LHL	gldm	SmallDependenceHighGrayLevelEmphasis	854.4513436
wavelet-LHL	gldm	DependenceNonUniformityNormalized	0.314390859
wavelet-LHL	gldm	LargeDependenceEmphasis	5.716894977
wavelet-LHL	gldm	LargeDependenceLowGrayLevelEmphasis	0.005751011
wavelet-LHL	gldm	DependenceVariance	1.652712621
wavelet-LHL	gldm	LargeDependenceHighGrayLevelEmphasis	7809.603881
wavelet-LHL	gldm	SmallDependenceLowGrayLevelEmphasis	0.001615886
wavelet-LHL	gldm	LowGrayLevelEmphasis	0.001985036
wavelet-LHL	glcm	JointAverage	37.50462767
wavelet-LHL	glcm	SumAverage	75.00925535
wavelet-LHL	glcm	JointEntropy	9.050147309
wavelet-LHL	glcm	ClusterShade	1052.439269
wavelet-LHL	glcm	MaximumProbability	0.009713465
wavelet-LHL	glcm	Idmn	0.97601451
wavelet-LHL	glcm	JointEnergy	0.002494314
wavelet-LHL	glcm	Contrast	134.3119158
wavelet-LHL	glcm	DifferenceEntropy	4.471531409
wavelet-LHL	glcm	InverseVariance	0.142344456
wavelet-LHL	glcm	DifferenceVariance	59.47572906
wavelet-LHL	glcm	Idn	0.901377124
wavelet-LHL	glcm	Idm	0.140062703
wavelet-LHL	glcm	Correlation	0.108228114
wavelet-LHL	glcm	Autocorrelation	1414.641758
wavelet-LHL	glcm	SumEntropy	5.580052881
wavelet-LHL	glcm	SumSquares	75.19258053
wavelet-LHL	glcm	ClusterProminence	105951.0866
wavelet-LHL	glcm	Imc2	0.928509458
wavelet-LHL	glcm	Imc1	-0.198646134
wavelet-LHL	glcm	DifferenceAverage	8.462650563
wavelet-LHL	glcm	Id	0.223148022
wavelet-LHL	glcm	ClusterTendency	166.4584064
wavelet-LHL	firstorder	InterquartileRange	246.8323232
wavelet-LHL	firstorder	Skewness	0.449333395
wavelet-LHL	firstorder	Uniformity	0.037496612
wavelet-LHL	firstorder	Median	-18.99723911
wavelet-LHL	firstorder	Energy	41091545.2
wavelet-LHL	firstorder	RobustMeanAbsoluteDeviation	105.918825
wavelet-LHL	firstorder	MeanAbsoluteDeviation	163.6567421
wavelet-LHL	firstorder	TotalEnergy	1109471720
wavelet-LHL	firstorder	Maximum	845.1718589
wavelet-LHL	firstorder	RootMeanSquared	216.5829093
wavelet-LHL	firstorder	90Percentile	288.3651158
wavelet-LHL	firstorder	Minimum	-912.4936941
wavelet-LHL	firstorder	Entropy	5.041056236
wavelet-LHL	firstorder	Range	1757.665553
wavelet-LHL	firstorder	Variance	46905.17812
wavelet-LHL	firstorder	10Percentile	-242.1933471
wavelet-LHL	firstorder	Kurtosis	4.096083145

wavelet-LHL	firstorder	Mean	1.725831957
wavelet-LHL	glrlm	ShortRunLowGrayLevelEmphasis	0.002009829
wavelet-LHL	glrlm	GrayLevelVariance	76.55579386
wavelet-LHL	glrlm	LowGrayLevelRunEmphasis	0.002033291
wavelet-LHL	glrlm	GrayLevelNonUniformityNormalized	0.036688991
wavelet-LHL	glrlm	RunVariance	0.043608314
wavelet-LHL	glrlm	GrayLevelNonUniformity	30.88363257
wavelet-LHL	glrlm	LongRunEmphasis	1.127182949
wavelet-LHL	glrlm	ShortRunHighGrayLevelEmphasis	1451.7266
wavelet-LHL	glrlm	RunLengthNonUniformity	779.4678883
wavelet-LHL	glrlm	ShortRunEmphasis	0.970675649
wavelet-LHL	glrlm	LongRunHighGrayLevelEmphasis	1663.940154
wavelet-LHL	glrlm	RunPercentage	0.960923779
wavelet-LHL	glrlm	LongRunLowGrayLevelEmphasis	0.002135998
wavelet-LHL	glrlm	RunEntropy	5.265471591
wavelet-LHL	glrlm	HighGrayLevelRunEmphasis	1491.780445
wavelet-LHL	glrlm	RunLengthNonUniformityNormalized	0.925686752
wavelet-LHL	glszm	GrayLevelVariance	97.92770918
wavelet-LHL	glszm	ZoneVariance	3.592387469
wavelet-LHL	glszm	GrayLevelNonUniformityNormalized	0.030431535
wavelet-LHL	glszm	SizeZoneNonUniformityNormalized	0.563343097
wavelet-LHL	glszm	SizeZoneNonUniformity	303.6419295
wavelet-LHL	glszm	GrayLevelNonUniformity	16.4025974
wavelet-LHL	glszm	LargeAreaEmphasis	6.233766234
wavelet-LHL	glszm	SmallAreaHighGrayLevelEmphasis	1238.773938
wavelet-LHL	glszm	ZonePercentage	0.615296804
wavelet-LHL	glszm	LargeAreaLowGrayLevelEmphasis	0.006909597
wavelet-LHL	glszm	LargeAreaHighGrayLevelEmphasis	8470.907236
wavelet-LHL	glszm	HighGrayLevelZoneEmphasis	1558.330241
wavelet-LHL	glszm	SmallAreaEmphasis	0.778785939
wavelet-LHL	glszm	LowGrayLevelZoneEmphasis	0.002718746
wavelet-LHL	glszm	ZoneEntropy	6.335183114
wavelet-LHL	glszm	SmallAreaLowGrayLevelEmphasis	0.002538839
wavelet-LHL	ngtdm	Coarseness	0.007350171
wavelet-LHL	ngtdm	Complexity	7404.755388
wavelet-LHL	ngtdm	Strength	7.349429067
wavelet-LHL	ngtdm	Contrast	0.320383429
wavelet-LHL	ngtdm	Busyness	0.06351471
wavelet-LHH	gldm	GrayLevelVariance	68.08354705
wavelet-LHH	gldm	HighGrayLevelEmphasis	1725.205479
wavelet-LHH	gldm	DependenceEntropy	6.576177185
wavelet-LHH	gldm	DependenceNonUniformity	263.3493151
wavelet-LHH	gldm	GrayLevelNonUniformity	39.7260274
wavelet-LHH	gldm	SmallDependenceEmphasis	0.532935001
wavelet-LHH	gldm	SmallDependenceHighGrayLevelEmphasis	940.2581918
wavelet-LHH	gldm	DependenceNonUniformityNormalized	0.300627072
wavelet-LHH	gldm	LargeDependenceEmphasis	7.198630137
wavelet-LHH	gldm	LargeDependenceLowGrayLevelEmphasis	0.005737669
wavelet-LHH	gldm	DependenceVariance	2.533819145
wavelet-LHH	gldm	LargeDependenceHighGrayLevelEmphasis	11882.5411
wavelet-LHH	gldm	SmallDependenceLowGrayLevelEmphasis	0.001570676
wavelet-LHH	gldm	LowGrayLevelEmphasis	0.001864733
wavelet-LHH	glcm	JointAverage	40.58969055

wavelet-LHH	glcm	SumAverage	81.17938109
wavelet-LHH	glcm	JointEntropy	8.747997952
wavelet-LHH	glcm	ClusterShade	-17.23252095
wavelet-LHH	glcm	MaximumProbability	0.015736003
wavelet-LHH	glcm	Idmn	0.98143316
wavelet-LHH	glcm	JointEnergy	0.00338455
wavelet-LHH	glcm	Contrast	132.4548262
wavelet-LHH	glcm	DifferenceEntropy	4.435849692
wavelet-LHH	glcm	InverseVariance	0.152570816
wavelet-LHH	glcm	DifferenceVariance	65.03787845
wavelet-LHH	glcm	Idn	0.91493284
wavelet-LHH	glcm	Idm	0.152896634
wavelet-LHH	glcm	Correlation	-0.028081848
wavelet-LHH	glcm	Autocorrelation	1646.061065
wavelet-LHH	glcm	SumEntropy	5.297222364
wavelet-LHH	glcm	SumSquares	64.75793394
wavelet-LHH	glcm	ClusterProminence	106086.6731
wavelet-LHH	glcm	Imc2	0.923172204
wavelet-LHH	glcm	Imc1	-0.198043353
wavelet-LHH	glcm	DifferenceAverage	8.089258271
wavelet-LHH	glcm	Id	0.236150617
wavelet-LHH	glcm	ClusterTendency	126.5769096
wavelet-LHH	firstorder	InterquartileRange	194.1213569
wavelet-LHH	firstorder	Skewness	0.07209414
wavelet-LHH	firstorder	Uniformity	0.045349346
wavelet-LHH	firstorder	Median	2.585738275
wavelet-LHH	firstorder	Energy	37172246.29
wavelet-LHH	firstorder	RobustMeanAbsoluteDeviation	83.0584458
wavelet-LHH	firstorder	MeanAbsoluteDeviation	142.3690117
wavelet-LHH	firstorder	TotalEnergy	1003650650
wavelet-LHH	firstorder	Maximum	979.6825592
wavelet-LHH	firstorder	RootMeanSquared	205.9953182
wavelet-LHH	firstorder	90Percentile	227.8008624
wavelet-LHH	firstorder	Minimum	-990.7494776
wavelet-LHH	firstorder	Entropy	4.893100632
wavelet-LHH	firstorder	Range	1970.432037
wavelet-LHH	firstorder	Variance	42406.48839
wavelet-LHH	firstorder	10Percentile	-227.7694955
wavelet-LHH	firstorder	Kurtosis	6.510462397
wavelet-LHH	firstorder	Mean	5.251924632
wavelet-LHH	glrlm	ShortRunLowGrayLevelEmphasis	0.001902572
wavelet-LHH	glrlm	GrayLevelVariance	70.65374459
wavelet-LHH	glrlm	LowGrayLevelRunEmphasis	0.001922896
wavelet-LHH	glrlm	GrayLevelNonUniformityNormalized	0.043625427
wavelet-LHH	glrlm	RunVariance	0.054867904
wavelet-LHH	glrlm	GrayLevelNonUniformity	36.51641575
wavelet-LHH	glrlm	LongRunEmphasis	1.151260122
wavelet-LHH	glrlm	ShortRunHighGrayLevelEmphasis	1674.494676
wavelet-LHH	glrlm	RunLengthNonUniformity	769.0408076
wavelet-LHH	glrlm	ShortRunEmphasis	0.967516606
wavelet-LHH	glrlm	LongRunHighGrayLevelEmphasis	1978.42256
wavelet-LHH	glrlm	RunPercentage	0.95539164
wavelet-LHH	glrlm	LongRunLowGrayLevelEmphasis	0.002017618

wavelet-LHH	glrlm	RunEntropy	5.153063631
wavelet-LHH	glrlm	HighGrayLevelRunEmphasis	1728.438829
wavelet-LHH	glrlm	RunLengthNonUniformityNormalized	0.918499304
wavelet-LHH	glszm	GrayLevelVariance	101.7990502
wavelet-LHH	glszm	ZoneVariance	6.67088165
wavelet-LHH	glszm	GrayLevelNonUniformityNormalized	0.032333489
wavelet-LHH	glszm	SizeZoneNonUniformityNormalized	0.569059204
wavelet-LHH	glszm	SizeZoneNonUniformity	298.1870229
wavelet-LHH	glszm	GrayLevelNonUniformity	16.94274809
wavelet-LHH	glszm	LargeAreaEmphasis	9.465648855
wavelet-LHH	glszm	SmallAreaHighGrayLevelEmphasis	1388.778722
wavelet-LHH	glszm	ZonePercentage	0.598173516
wavelet-LHH	glszm	LargeAreaLowGrayLevelEmphasis	0.007942299
wavelet-LHH	glszm	LargeAreaHighGrayLevelEmphasis	15648.00382
wavelet-LHH	glszm	HighGrayLevelZoneEmphasis	1761.583969
wavelet-LHH	glszm	SmallAreaEmphasis	0.782547395
wavelet-LHH	glszm	LowGrayLevelZoneEmphasis	0.002695201
wavelet-LHH	glszm	ZoneEntropy	6.265352378
wavelet-LHH	glszm	SmallAreaLowGrayLevelEmphasis	0.002557039
wavelet-LHH	ngtdm	Coarseness	0.008253425
wavelet-LHH	ngtdm	Complexity	9031.017994
wavelet-LHH	ngtdm	Strength	10.11170397
wavelet-LHH	ngtdm	Contrast	0.22820271
wavelet-LHH	ngtdm	Busyness	0.038639891
wavelet-LLH	gldm	GrayLevelVariance	128.2015492
wavelet-LLH	gldm	HighGrayLevelEmphasis	1257.038813
wavelet-LLH	gldm	DependenceEntropy	6.725805491
wavelet-LLH	gldm	DependenceNonUniformity	359.326484
wavelet-LLH	gldm	GrayLevelNonUniformity	26.41780822
wavelet-LLH	gldm	SmallDependenceEmphasis	0.653388935
wavelet-LLH	gldm	SmallDependenceHighGrayLevelEmphasis	874.239722
wavelet-LLH	gldm	DependenceNonUniformityNormalized	0.41019005
wavelet-LLH	gldm	LargeDependenceEmphasis	4.075342466
wavelet-LLH	gldm	LargeDependenceLowGrayLevelEmphasis	0.007038958
wavelet-LLH	gldm	DependenceVariance	1.158889514
wavelet-LLH	gldm	LargeDependenceHighGrayLevelEmphasis	4527.175799
wavelet-LLH	gldm	SmallDependenceLowGrayLevelEmphasis	0.003347062
wavelet-LLH	gldm	LowGrayLevelEmphasis	0.003738834
wavelet-LLH	glcm	JointAverage	33.70702212
wavelet-LLH	glcm	SumAverage	67.41404425
wavelet-LLH	glcm	JointEntropy	9.353392944
wavelet-LLH	glcm	ClusterShade	2176.668513
wavelet-LLH	glcm	MaximumProbability	0.008220403
wavelet-LLH	glcm	Idmn	0.963898164
wavelet-LLH	glcm	JointEnergy	0.001984027
wavelet-LLH	glcm	Contrast	237.6910212
wavelet-LLH	glcm	DifferenceEntropy	4.837179054
wavelet-LLH	glcm	InverseVariance	0.118337886
wavelet-LLH	glcm	DifferenceVariance	104.5521132
wavelet-LLH	glcm	Idn	0.881434709
wavelet-LLH	glcm	Idm	0.109376849
wavelet-LLH	glcm	Correlation	0.054449808
wavelet-LLH	glcm	Autocorrelation	1143.563537

wavelet-LLH	glcm	SumEntropy	5.849101323
wavelet-LLH	glcm	SumSquares	126.2100227
wavelet-LLH	glcm	ClusterProminence	302383.1648
wavelet-LLH	glcm	Imc2	0.969183747
wavelet-LLH	glcm	Imc1	-0.261748167
wavelet-LLH	glcm	DifferenceAverage	11.2053866
wavelet-LLH	glcm	Id	0.187083581
wavelet-LLH	glcm	ClusterTendency	267.1490697
wavelet-LLH	firstorder	InterquartileRange	311.4311062
wavelet-LLH	firstorder	Skewness	0.335164319
wavelet-LLH	firstorder	Uniformity	0.030157315
wavelet-LLH	firstorder	Median	-26.52394102
wavelet-LLH	firstorder	Energy	70122585.52
wavelet-LLH	firstorder	RobustMeanAbsoluteDeviation	134.021259
wavelet-LLH	firstorder	MeanAbsoluteDeviation	211.7892907
wavelet-LLH	firstorder	TotalEnergy	1893309809
wavelet-LLH	firstorder	Maximum	1041.886988
wavelet-LLH	firstorder	RootMeanSquared	282.928637
wavelet-LLH	firstorder	90Percentile	386.0867543
wavelet-LLH	firstorder	Minimum	-819.3409705
wavelet-LLH	firstorder	Entropy	5.415572301
wavelet-LLH	firstorder	Range	1861.227958
wavelet-LLH	firstorder	Variance	80043.42703
wavelet-LLH	firstorder	10Percentile	-317.3446829
wavelet-LLH	firstorder	Kurtosis	3.786360448
wavelet-LLH	firstorder	Mean	2.277407171
wavelet-LLH	glrlm	ShortRunLowGrayLevelEmphasis	0.003790894
wavelet-LLH	glrlm	GrayLevelVariance	130.6818139
wavelet-LLH	glrlm	LowGrayLevelRunEmphasis	0.003813038
wavelet-LLH	glrlm	GrayLevelNonUniformityNormalized	0.029472802
wavelet-LLH	glrlm	RunVariance	0.030286739
wavelet-LLH	glrlm	GrayLevelNonUniformity	25.11646772
wavelet-LLH	glrlm	LongRunEmphasis	1.087425043
wavelet-LLH	glrlm	ShortRunHighGrayLevelEmphasis	1240.227031
wavelet-LLH	glrlm	RunLengthNonUniformity	808.2353338
wavelet-LLH	glrlm	ShortRunEmphasis	0.979816848
wavelet-LLH	glrlm	LongRunHighGrayLevelEmphasis	1356.030442
wavelet-LLH	glrlm	RunPercentage	0.972778363
wavelet-LLH	glrlm	LongRunLowGrayLevelEmphasis	0.003909271
wavelet-LLH	glrlm	RunEntropy	5.576269109
wavelet-LLH	glrlm	HighGrayLevelRunEmphasis	1262.064328
wavelet-LLH	glrlm	RunLengthNonUniformityNormalized	0.948248714
wavelet-LLH	glszm	GrayLevelVariance	160.3831872
wavelet-LLH	glszm	ZoneVariance	1.74180994
wavelet-LLH	glszm	GrayLevelNonUniformityNormalized	0.024152377
wavelet-LLH	glszm	SizeZoneNonUniformityNormalized	0.660233664
wavelet-LLH	glszm	SizeZoneNonUniformity	413.9665072
wavelet-LLH	glszm	GrayLevelNonUniformity	15.14354067
wavelet-LLH	glszm	LargeAreaEmphasis	3.693779904
wavelet-LLH	glszm	SmallAreaHighGrayLevelEmphasis	1138.963714
wavelet-LLH	glszm	ZonePercentage	0.715753425
wavelet-LLH	glszm	LargeAreaLowGrayLevelEmphasis	0.007620134
wavelet-LLH	glszm	LargeAreaHighGrayLevelEmphasis	4182.421053

wavelet-LLH	glszm	HighGrayLevelZoneEmphasis	1322.899522
wavelet-LLH	glszm	SmallAreaEmphasis	0.838880977
wavelet-LLH	glszm	LowGrayLevelZoneEmphasis	0.004778575
wavelet-LLH	glszm	ZoneEntropy	6.423065063
wavelet-LLH	glszm	SmallAreaLowGrayLevelEmphasis	0.004590648
wavelet-LLH	ngtdm	Coarseness	0.00717891
wavelet-LLH	ngtdm	Complexity	15084.51357
wavelet-LLH	ngtdm	Strength	10.47575974
wavelet-LLH	ngtdm	Contrast	0.452389928
wavelet-LLH	ngtdm	Busyness	0.05598401
wavelet-LLL	gldm	GrayLevelVariance	485.5824524
wavelet-LLL	gldm	HighGrayLevelEmphasis	4264.182648
wavelet-LLL	gldm	DependenceEntropy	7.332105024
wavelet-LLL	gldm	DependenceNonUniformity	401.2283105
wavelet-LLL	gldm	GrayLevelNonUniformity	12.01598174
wavelet-LLL	gldm	SmallDependenceEmphasis	0.689954021
wavelet-LLL	gldm	SmallDependenceHighGrayLevelEmphasis	2725.130439
wavelet-LLL	gldm	DependenceNonUniformityNormalized	0.458023186
wavelet-LLL	gldm	LargeDependenceEmphasis	2.997716895
wavelet-LLL	gldm	LargeDependenceLowGrayLevelEmphasis	0.002615649
wavelet-LLL	gldm	DependenceVariance	0.636793436
wavelet-LLL	gldm	LargeDependenceHighGrayLevelEmphasis	15547.11416
wavelet-LLL	gldm	SmallDependenceLowGrayLevelEmphasis	0.001754815
wavelet-LLL	gldm	LowGrayLevelEmphasis	0.001887254
wavelet-LLL	glcm	JointAverage	64.03482455
wavelet-LLL	glcm	SumAverage	128.0696491
wavelet-LLL	glcm	JointEntropy	9.979380566
wavelet-LLL	glcm	ClusterShade	-17676.58363
wavelet-LLL	glcm	MaximumProbability	0.004541633
wavelet-LLL	glcm	Idmn	0.972350578
wavelet-LLL	glcm	JointEnergy	0.001097183
wavelet-LLL	glcm	Contrast	333.2467984
wavelet-LLL	glcm	DifferenceEntropy	5.154290222
wavelet-LLL	glcm	InverseVariance	0.082155856
wavelet-LLL	glcm	DifferenceVariance	126.13175
wavelet-LLL	glcm	Idn	0.888388897
wavelet-LLL	glcm	Idm	0.078916985
wavelet-LLL	glcm	Correlation	0.663116496
wavelet-LLL	glcm	Autocorrelation	4427.066373
wavelet-LLL	glcm	SumEntropy	7.047046398
wavelet-LLL	glcm	SumSquares	492.9683313
wavelet-LLL	glcm	ClusterProminence	6024843.721
wavelet-LLL	glcm	Imc2	0.99708191
wavelet-LLL	glcm	Imc1	-0.410152853
wavelet-LLL	glcm	DifferenceAverage	14.2042021
wavelet-LLL	glcm	Id	0.148795677
wavelet-LLL	glcm	ClusterTendency	1638.626527
wavelet-LLL	firstorder	InterquartileRange	841.0213178
wavelet-LLL	firstorder	Skewness	-0.16336332
wavelet-LLL	firstorder	Uniformity	0.013716874
wavelet-LLL	firstorder	Median	-728.7527612
wavelet-LLL	firstorder	Energy	759404142.8
wavelet-LLL	firstorder	RobustMeanAbsoluteDeviation	353.4396909

wavelet-LLL	firstorder	MeanAbsoluteDeviation	463.9243279
wavelet-LLL	firstorder	TotalEnergy	20503911857
wavelet-LLL	firstorder	Maximum	327.933613
wavelet-LLL	firstorder	RootMeanSquared	931.0744903
wavelet-LLL	firstorder	90Percentile	22.70194935
wavelet-LLL	firstorder	Minimum	-2262.407394
wavelet-LLL	firstorder	Entropy	6.315918865
wavelet-LLL	firstorder	Range	2590.341007
wavelet-LLL	firstorder	Variance	303215.2737
wavelet-LLL	firstorder	10Percentile	-1485.97678
wavelet-LLL	firstorder	Kurtosis	2.196091261
wavelet-LLL	firstorder	Mean	-750.7892066
wavelet-LLL	glrlm	ShortRunLowGrayLevelEmphasis	0.00191293
wavelet-LLL	glrlm	GrayLevelVariance	482.6367394
wavelet-LLL	glrlm	LowGrayLevelRunEmphasis	0.001918857
wavelet-LLL	glrlm	GrayLevelNonUniformityNormalized	0.013690834
wavelet-LLL	glrlm	RunVariance	0.023149059
wavelet-LLL	glrlm	GrayLevelNonUniformity	11.74573579
wavelet-LLL	glrlm	LongRunEmphasis	1.065884867
wavelet-LLL	glrlm	ShortRunHighGrayLevelEmphasis	4160.752141
wavelet-LLL	glrlm	RunLengthNonUniformity	824.4710043
wavelet-LLL	glrlm	ShortRunEmphasis	0.9848853
wavelet-LLL	glrlm	LongRunHighGrayLevelEmphasis	4592.369962
wavelet-LLL	glrlm	RunPercentage	0.979364243
wavelet-LLL	glrlm	LongRunLowGrayLevelEmphasis	0.001944557
wavelet-LLL	glrlm	RunEntropy	6.409537878
wavelet-LLL	glrlm	HighGrayLevelRunEmphasis	4241.519553
wavelet-LLL	glrlm	RunLengthNonUniformityNormalized	0.960928451
wavelet-LLL	glszm	GrayLevelVariance	446.5923423
wavelet-LLL	glszm	ZoneVariance	0.789465141
wavelet-LLL	glszm	GrayLevelNonUniformityNormalized	0.013806599
wavelet-LLL	glszm	SizeZoneNonUniformityNormalized	0.662739316
wavelet-LLL	glszm	SizeZoneNonUniformity	441.3843844
wavelet-LLL	glszm	GrayLevelNonUniformity	9.195195195
wavelet-LLL	glszm	LargeAreaEmphasis	2.51951952
wavelet-LLL	glszm	SmallAreaHighGrayLevelEmphasis	3262.081211
wavelet-LLL	glszm	ZonePercentage	0.760273973
wavelet-LLL	glszm	LargeAreaLowGrayLevelEmphasis	0.002863922
wavelet-LLL	glszm	LargeAreaHighGrayLevelEmphasis	13144.16216
wavelet-LLL	glszm	HighGrayLevelZoneEmphasis	3986.842342
wavelet-LLL	glszm	SmallAreaEmphasis	0.840925924
wavelet-LLL	glszm	LowGrayLevelZoneEmphasis	0.002353752
wavelet-LLL	glszm	ZoneEntropy	7.020440241
wavelet-LLL	glszm	SmallAreaLowGrayLevelEmphasis	0.002279177
wavelet-LLL	ngtdm	Coarseness	0.010830181
wavelet-LLL	ngtdm	Complexity	29718.94011
wavelet-LLL	ngtdm	Strength	36.59177326
wavelet-LLL	ngtdm	Contrast	0.855591457
wavelet-LLL	ngtdm	Busyness	0.018218064
wavelet-HHL	gldm	GrayLevelVariance	24.02508288
wavelet-HHL	gldm	HighGrayLevelEmphasis	405.6118721
wavelet-HHL	gldm	DependenceEntropy	6.300281092
wavelet-HHL	gldm	DependenceNonUniformity	187.6872146

wavelet-HHL	gldm	GrayLevelNonUniformity	63.456621
wavelet-HHL	gldm	SmallDependenceEmphasis	0.407314587
wavelet-HHL	gldm	SmallDependenceHighGrayLevelEmphasis	172.9811067
wavelet-HHL	gldm	DependenceNonUniformityNormalized	0.214254811
wavelet-HHL	gldm	LargeDependenceEmphasis	10.41780822
wavelet-HHL	gldm	LargeDependenceLowGrayLevelEmphasis	0.039811313
wavelet-HHL	gldm	DependenceVariance	3.233450095
wavelet-HHL	gldm	LargeDependenceHighGrayLevelEmphasis	4050.639269
wavelet-HHL	gldm	SmallDependenceLowGrayLevelEmphasis	0.003039312
wavelet-HHL	gldm	LowGrayLevelEmphasis	0.006611982
wavelet-HHL	glcm	JointAverage	19.55173925
wavelet-HHL	glcm	SumAverage	39.10347849
wavelet-HHL	glcm	JointEntropy	7.771102012
wavelet-HHL	glcm	ClusterShade	-20.05361016
wavelet-HHL	glcm	MaximumProbability	0.021807036
wavelet-HHL	glcm	Idmn	0.978440318
wavelet-HHL	glcm	JointEnergy	0.007088232
wavelet-HHL	glcm	Contrast	47.51544743
wavelet-HHL	glcm	DifferenceEntropy	3.750542385
wavelet-HHL	glcm	InverseVariance	0.224629404
wavelet-HHL	glcm	DifferenceVariance	23.49776569
wavelet-HHL	glcm	Idn	0.908485756
wavelet-HHL	glcm	Idm	0.22132688
wavelet-HHL	glcm	Correlation	-0.027804498
wavelet-HHL	glcm	Autocorrelation	381.6219219
wavelet-HHL	glcm	SumEntropy	4.599816305
wavelet-HHL	glcm	SumSquares	23.10491671
wavelet-HHL	glcm	ClusterProminence	12921.5913
wavelet-HHL	glcm	Imc2	0.80478286
wavelet-HHL	glcm	Imc1	-0.126803612
wavelet-HHL	glcm	DifferenceAverage	4.846230982
wavelet-HHL	glcm	Id	0.309130444
wavelet-HHL	glcm	ClusterTendency	44.90421941
wavelet-HHL	firstorder	InterquartileRange	120.0976795
wavelet-HHL	firstorder	Skewness	0.047666068
wavelet-HHL	firstorder	Uniformity	0.072439065
wavelet-HHL	firstorder	Median	2.472511186
wavelet-HHL	firstorder	Energy	13096075.56
wavelet-HHL	firstorder	RobustMeanAbsoluteDeviation	52.12521327
wavelet-HHL	firstorder	MeanAbsoluteDeviation	86.34836936
wavelet-HHL	firstorder	TotalEnergy	353594040.2
wavelet-HHL	firstorder	Maximum	620.2050653
wavelet-HHL	firstorder	RootMeanSquared	122.2696117
wavelet-HHL	firstorder	90Percentile	129.8771317
wavelet-HHL	firstorder	Minimum	-471.7458808
wavelet-HHL	firstorder	Entropy	4.180254559
wavelet-HHL	firstorder	Range	1091.950946
wavelet-HHL	firstorder	Variance	14949.35589
wavelet-HHL	firstorder	10Percentile	-142.0442605
wavelet-HHL	firstorder	Kurtosis	6.049230594
wavelet-HHL	firstorder	Mean	0.70855878
wavelet-HHL	glrlm	ShortRunLowGrayLevelEmphasis	0.006556493
wavelet-HHL	glrlm	GrayLevelVariance	25.18171834

wavelet-HHL	glrlm	LowGrayLevelRunEmphasis	0.006770693
wavelet-HHL	glrlm	GrayLevelNonUniformityNormalized	0.069579367
wavelet-HHL	glrlm	RunVariance	0.07885369
wavelet-HHL	glrlm	GrayLevelNonUniformity	57.01396411
wavelet-HHL	glrlm	LongRunEmphasis	1.222941856
wavelet-HHL	glrlm	ShortRunHighGrayLevelEmphasis	388.0375608
wavelet-HHL	glrlm	RunLengthNonUniformity	723.1310564
wavelet-HHL	glrlm	ShortRunEmphasis	0.952029732
wavelet-HHL	glrlm	LongRunHighGrayLevelEmphasis	493.4157677
wavelet-HHL	glrlm	RunPercentage	0.935370566
wavelet-HHL	glrlm	LongRunLowGrayLevelEmphasis	0.007711345
wavelet-HHL	glrlm	RunEntropy	4.546289011
wavelet-HHL	glrlm	HighGrayLevelRunEmphasis	406.7481917
wavelet-HHL	glrlm	RunLengthNonUniformityNormalized	0.882042477
wavelet-HHL	glszm	GrayLevelVariance	41.10629469
wavelet-HHL	glszm	ZoneVariance	19.05053645
wavelet-HHL	glszm	GrayLevelNonUniformityNormalized	0.048912181
wavelet-HHL	glszm	SizeZoneNonUniformityNormalized	0.498455037
wavelet-HHL	glszm	SizeZoneNonUniformity	197.8866499
wavelet-HHL	glszm	GrayLevelNonUniformity	19.41813602
wavelet-HHL	glszm	LargeAreaEmphasis	23.91939547
wavelet-HHL	glszm	SmallAreaHighGrayLevelEmphasis	316.0350394
wavelet-HHL	glszm	ZonePercentage	0.453196347
wavelet-HHL	glszm	LargeAreaLowGrayLevelEmphasis	0.07725252
wavelet-HHL	glszm	LargeAreaHighGrayLevelEmphasis	9403.853904
wavelet-HHL	glszm	HighGrayLevelZoneEmphasis	421.7984887
wavelet-HHL	glszm	SmallAreaEmphasis	0.733101649
wavelet-HHL	glszm	LowGrayLevelZoneEmphasis	0.008424199
wavelet-HHL	glszm	ZoneEntropy	5.969889087
wavelet-HHL	glszm	SmallAreaLowGrayLevelEmphasis	0.005532045
wavelet-HHL	ngtdm	Coarseness	0.007980539
wavelet-HHL	ngtdm	Complexity	2291.252988
wavelet-HHL	ngtdm	Strength	3.985128646
wavelet-HHL	ngtdm	Contrast	0.117072752
wavelet-HHL	ngtdm	Busyness	0.122652389
wavelet-HLH	gldm	GrayLevelVariance	25.65222785
wavelet-HLH	gldm	HighGrayLevelEmphasis	489.7694064
wavelet-HLH	gldm	DependenceEntropy	6.264727154
wavelet-HLH	gldm	DependenceNonUniformity	208.7853881
wavelet-HLH	gldm	GrayLevelNonUniformity	58.73287671
wavelet-HLH	gldm	SmallDependenceEmphasis	0.425293383
wavelet-HLH	gldm	SmallDependenceHighGrayLevelEmphasis	216.9041068
wavelet-HLH	gldm	DependenceNonUniformityNormalized	0.238339484
wavelet-HLH	gldm	LargeDependenceEmphasis	8.751141553
wavelet-HLH	gldm	LargeDependenceLowGrayLevelEmphasis	0.021890252
wavelet-HLH	gldm	DependenceVariance	2.614775547
wavelet-HLH	gldm	LargeDependenceHighGrayLevelEmphasis	4113.584475
wavelet-HLH	gldm	SmallDependenceLowGrayLevelEmphasis	0.002999823
wavelet-HLH	gldm	LowGrayLevelEmphasis	0.004363618
wavelet-HLH	glcm	JointAverage	21.57286059
wavelet-HLH	glcm	SumAverage	43.14572119
wavelet-HLH	glcm	JointEntropy	7.973109557
wavelet-HLH	glcm	ClusterShade	-64.43770689

wavelet-HLH	glcm	MaximumProbability	0.019538405
wavelet-HLH	glcm	Idmn	0.971413914
wavelet-HLH	glcm	JointEnergy	0.005904643
wavelet-HLH	glcm	Contrast	52.93160111
wavelet-HLH	glcm	DifferenceEntropy	3.84378695
wavelet-HLH	glcm	InverseVariance	0.2065759
wavelet-HLH	glcm	DifferenceVariance	24.67960716
wavelet-HLH	glcm	Idn	0.893444192
wavelet-HLH	glcm	Idm	0.201052935
wavelet-HLH	glcm	Correlation	-0.02538763
wavelet-HLH	glcm	Autocorrelation	464.7137081
wavelet-HLH	glcm	SumEntropy	4.704517525
wavelet-HLH	glcm	SumSquares	25.78904743
wavelet-HLH	glcm	ClusterProminence	13572.19035
wavelet-HLH	glcm	Imc2	0.826800834
wavelet-HLH	glcm	Imc1	-0.135850488
wavelet-HLH	glcm	DifferenceAverage	5.245753494
wavelet-HLH	glcm	Id	0.290014263
wavelet-HLH	glcm	ClusterTendency	50.22458859
wavelet-HLH	firstorder	InterquartileRange	132.8613369
wavelet-HLH	firstorder	Skewness	-0.173431859
wavelet-HLH	firstorder	Uniformity	0.067046663
wavelet-HLH	firstorder	Median	3.564328762
wavelet-HLH	firstorder	Energy	14058563.4
wavelet-HLH	firstorder	RobustMeanAbsoluteDeviation	56.47920428
wavelet-HLH	firstorder	MeanAbsoluteDeviation	91.83424897
wavelet-HLH	firstorder	TotalEnergy	379581211.9
wavelet-HLH	firstorder	Maximum	462.4455252
wavelet-HLH	firstorder	RootMeanSquared	126.6830232
wavelet-HLH	firstorder	90Percentile	141.5500075
wavelet-HLH	firstorder	Minimum	-510.1406382
wavelet-HLH	firstorder	Entropy	4.280095054
wavelet-HLH	firstorder	Range	972.5861634
wavelet-HLH	firstorder	Variance	16047.11154
wavelet-HLH	firstorder	10Percentile	-142.9821592
wavelet-HLH	firstorder	Kurtosis	4.856250178
wavelet-HLH	firstorder	Mean	1.215243514
wavelet-HLH	glrlm	ShortRunLowGrayLevelEmphasis	0.004391001
wavelet-HLH	glrlm	GrayLevelVariance	26.73968824
wavelet-HLH	glrlm	LowGrayLevelRunEmphasis	0.004488593
wavelet-HLH	glrlm	GrayLevelNonUniformityNormalized	0.064685832
wavelet-HLH	glrlm	RunVariance	0.066314069
wavelet-HLH	glrlm	GrayLevelNonUniformity	53.44656262
wavelet-HLH	glrlm	LongRunEmphasis	1.191151747
wavelet-HLH	glrlm	ShortRunHighGrayLevelEmphasis	470.7673488
wavelet-HLH	glrlm	RunLengthNonUniformity	739.2684791
wavelet-HLH	glrlm	ShortRunEmphasis	0.957483114
wavelet-HLH	glrlm	LongRunHighGrayLevelEmphasis	580.6969541
wavelet-HLH	glrlm	RunPercentage	0.94318581
wavelet-HLH	glrlm	LongRunLowGrayLevelEmphasis	0.004927805
wavelet-HLH	glrlm	RunEntropy	4.607832708
wavelet-HLH	glrlm	HighGrayLevelRunEmphasis	490.8862533
wavelet-HLH	glrlm	RunLengthNonUniformityNormalized	0.894398045

wavelet-HLH	glszm	GrayLevelVariance	42.63990188
wavelet-HLH	glszm	ZoneVariance	12.12771449
wavelet-HLH	glszm	GrayLevelNonUniformityNormalized	0.046168565
wavelet-HLH	glszm	SizeZoneNonUniformityNormalized	0.472220986
wavelet-HLH	glszm	SizeZoneNonUniformity	200.2216981
wavelet-HLH	glszm	GrayLevelNonUniformity	19.5754717
wavelet-HLH	glszm	LargeAreaEmphasis	16.39622642
wavelet-HLH	glszm	SmallAreaHighGrayLevelEmphasis	369.6419966
wavelet-HLH	glszm	ZonePercentage	0.484018265
wavelet-HLH	glszm	LargeAreaLowGrayLevelEmphasis	0.041069961
wavelet-HLH	glszm	LargeAreaHighGrayLevelEmphasis	7599.589623
wavelet-HLH	glszm	HighGrayLevelZoneEmphasis	506.615566
wavelet-HLH	glszm	SmallAreaEmphasis	0.714422898
wavelet-HLH	glszm	LowGrayLevelZoneEmphasis	0.0065256
wavelet-HLH	glszm	ZoneEntropy	6.073769808
wavelet-HLH	glszm	SmallAreaLowGrayLevelEmphasis	0.005801525
wavelet-HLH	ngtdm	Coarseness	0.007854449
wavelet-HLH	ngtdm	Complexity	2261.734885
wavelet-HLH	ngtdm	Strength	3.542109404
wavelet-HLH	ngtdm	Contrast	0.140077826
wavelet-HLH	ngtdm	Busyness	0.122173917

Image type	Feature Class	Feature Name	4: RTSTRUCT: C99-DNU_segment_RLL ITV
general	info	BoundingBox	(0, 0, 1, 8, 12, 6)
general	info	EnabledImageTypes	{u'Original': {}, u'Wavelet': {}}
general	info	GeneralSettings	{'distances': [1], 'additionalInfo': True, 'force2D': False, 'interpolator': 'sitkBSpline', 'resampledPixelSpacing': [3.0], 'label': 1, 'normalizeScale': 1, 'normalize': False, 'force2Ddimension': 0, 'removeOutliers': None, 'minimumROISize': None, u'binWidth': 25.0, 'minimumROIDimensions': 1, u'correctMask': True, 'preCrop': False, u'symmetricalGLCM': True, 'resegmentRange': None, 'padDistance': 5}
general	info	ImageHash	d1a7603fbbe1e90d89e6f947f5bc075365f4aa8d
general	info	ImageSpacing	(0.9765625000000002, 0.9765625000000002, 2.0)
general	info	MaskHash	1a7cb3ffca3a0646ef1907b65cf8b31c4b8d4e63
general	info	NumpyVersion	1.13.1
general	info	PyWaveletVersion	0.5.2
general	info	SimpleITKVersion	1.1.0
general	info	Version	1.3.0.post102+g1b0eb74
general	info	VolumeNum	1
general	info	VoxelNum	276
original	shape	Maximum3DDiameter	36.74234614
original	shape	Maximum2DDiameterSlice	30.88689042
original	shape	Sphericity	0.814666979
original	shape	MinorAxis	20.46006431
original	shape	Elongation	0.645145694
original	shape	SurfaceVolumeRatio	0.303910983
original	shape	Volume	7452
original	shape	MajorAxis	31.71386637
original	shape	SurfaceArea	2264.744647
original	shape	Flatness	0.53391955
original	shape	LeastAxis	16.93265327
original	shape	Maximum2DDiameterColumn	21.84032967
original	shape	Maximum2DDiameterRow	36.24913792
original	gldm	GrayLevelVariance	98.62522317
original	gldm	HighGrayLevelEmphasis	889.7427536
original	gldm	DependenceEntropy	5.984623567
original	gldm	DependenceNonUniformity	70.53623188
original	gldm	GrayLevelNonUniformity	23.80434783
original	gldm	SmallDependenceEmphasis	0.520025946
original	gldm	SmallDependenceHighGrayLevelEmphasis	315.7264647
original	gldm	DependenceNonUniformityNormalized	0.255566058
original	gldm	LargeDependenceEmphasis	28.33333333
original	gldm	LargeDependenceLowGrayLevelEmphasis	0.03373579
original	gldm	DependenceVariance	15.04783659
original	gldm	LargeDependenceHighGrayLevelEmphasis	36110.97464
original	gldm	SmallDependenceLowGrayLevelEmphasis	0.011069406
original	gldm	LowGrayLevelEmphasis	0.011678418

original	glcm	JointAverage	30.34127901
original	glcm	SumAverage	60.68255802
original	glcm	JointEntropy	6.653905093
original	glcm	ClusterShade	-2838.329952
original	glcm	MaximumProbability	0.10820817
original	glcm	Idmn	0.936378037
original	glcm	JointEnergy	0.025826327
original	glcm	Contrast	126.6996251
original	glcm	DifferenceEntropy	4.075743382
original	glcm	InverseVariance	0.26419504
original	glcm	DifferenceVariance	70.00287943
original	glcm	Idn	0.863548784
original	glcm	Idm	0.287694802
original	glcm	Correlation	0.187715803
original	glcm	Autocorrelation	936.3928498
original	glcm	SumEntropy	4.789347543
original	glcm	SumSquares	78.88926467
original	glcm	ClusterProminence	124032.4597
original	glcm	Imc2	0.954834522
original	glcm	Imc1	-0.314254228
original	glcm	DifferenceAverage	7.452341657
original	glcm	Id	0.342969957
original	glcm	ClusterTendency	188.8574336
original	firstorder	InterquartileRange	378.25
original	firstorder	Skewness	-0.965443874
original	firstorder	Uniformity	0.086247637
original	firstorder	Median	-40.5
original	firstorder	Energy	26360391
original	firstorder	RobustMeanAbsoluteDeviation	163.3995868
original	firstorder	MeanAbsoluteDeviation	212.4017801
original	firstorder	TotalEnergy	711730557
original	firstorder	Maximum	77
original	firstorder	RootMeanSquared	309.044759
original	firstorder	90Percentile	31
original	firstorder	Minimum	-866
original	firstorder	Entropy	4.39816226
original	firstorder	Range	943
original	firstorder	Variance	61557.9302
original	firstorder	10Percentile	-597
original	firstorder	Kurtosis	2.732030101
original	firstorder	Mean	-184.2572464
original	glrlm	ShortRunLowGrayLevelEmphasis	0.01284683
original	glrlm	GrayLevelVariance	101.7277544
original	glrlm	LowGrayLevelRunEmphasis	0.012909989
original	glrlm	GrayLevelNonUniformityNormalized	0.065017016
original	glrlm	RunVariance	0.172396498
original	glrlm	GrayLevelNonUniformity	16.14634676
original	glrlm	LongRunEmphasis	1.414989436
original	glrlm	ShortRunHighGrayLevelEmphasis	760.1232397
original	glrlm	RunLengthNonUniformity	208.4022106
original	glrlm	ShortRunEmphasis	0.931922194
original	glrlm	LongRunHighGrayLevelEmphasis	1375.462215
original	glrlm	RunPercentage	0.898272018
original	glrlm	LongRunLowGrayLevelEmphasis	0.013272741

original	glrlm	RunEntropy	4.935737956
original	glrlm	HighGrayLevelRunEmphasis	846.2017112
original	glrlm	RunLengthNonUniformityNormalized	0.839745512
original	glszm	GrayLevelVariance	93.64466471
original	glszm	ZoneVariance	29.70545152
original	glszm	GrayLevelNonUniformityNormalized	0.033200298
original	glszm	SizeZoneNonUniformityNormalized	0.74913381
original	glszm	SizeZoneNonUniformity	113.1192053
original	glszm	GrayLevelNonUniformity	5.013245033
original	glszm	LargeAreaEmphasis	33.04635762
original	glszm	SmallAreaHighGrayLevelEmphasis	506.7242525
original	glszm	ZonePercentage	0.547101449
original	glszm	LargeAreaLowGrayLevelEmphasis	0.046085753
original	glszm	LargeAreaHighGrayLevelEmphasis	41867.54305
original	glszm	HighGrayLevelZoneEmphasis	612.3377483
original	glszm	SmallAreaEmphasis	0.884887407
original	glszm	LowGrayLevelZoneEmphasis	0.02041591
original	glszm	ZoneEntropy	5.451181712
original	glszm	SmallAreaLowGrayLevelEmphasis	0.020109487
original	ngtdm	Coarseness	0.009801992
original	ngtdm	Complexity	4084.504674
original	ngtdm	Strength	12.1239673
original	ngtdm	Contrast	0.903405207
original	ngtdm	Busyness	0.070309837
wavelet-HHH	gldm	GrayLevelVariance	11.3227657
wavelet-HHH	gldm	HighGrayLevelEmphasis	169.6630435
wavelet-HHH	gldm	DependenceEntropy	5.7261501
wavelet-HHH	gldm	DependenceNonUniformity	49.74637681
wavelet-HHH	gldm	GrayLevelNonUniformity	27.60144928
wavelet-HHH	gldm	SmallDependenceEmphasis	0.345463916
wavelet-HHH	gldm	SmallDependenceHighGrayLevelEmphasis	61.34347688
wavelet-HHH	gldm	DependenceNonUniformityNormalized	0.180240496
wavelet-HHH	gldm	LargeDependenceEmphasis	14.37681159
wavelet-HHH	gldm	LargeDependenceLowGrayLevelEmphasis	0.097660976
wavelet-HHH	gldm	DependenceVariance	4.667716866
wavelet-HHH	gldm	LargeDependenceHighGrayLevelEmphasis	2369.612319
wavelet-HHH	gldm	SmallDependenceLowGrayLevelEmphasis	0.007634413
wavelet-HHH	gldm	LowGrayLevelEmphasis	0.012164787
wavelet-HHH	glcm	JointAverage	12.54982311
wavelet-HHH	glcm	SumAverage	25.09964622
wavelet-HHH	glcm	JointEntropy	6.674583725
wavelet-HHH	glcm	ClusterShade	5.703925931
wavelet-HHH	glcm	MaximumProbability	0.048547429
wavelet-HHH	glcm	Idmn	0.976212538
wavelet-HHH	glcm	JointEnergy	0.014055243
wavelet-HHH	glcm	Contrast	22.12845827
wavelet-HHH	glcm	DifferenceEntropy	3.279666797
wavelet-HHH	glcm	InverseVariance	0.270845556
wavelet-HHH	glcm	DifferenceVariance	9.76240675
wavelet-HHH	glcm	Idn	0.899880863
wavelet-HHH	glcm	Idm	0.274079585
wavelet-HHH	glcm	Correlation	-0.029686622
wavelet-HHH	glcm	Autocorrelation	157.1792955
wavelet-HHH	glcm	SumEntropy	4.059798971

wavelet-HHH	glcm	SumSquares	10.74275915
wavelet-HHH	glcm	ClusterProminence	1853.711748
wavelet-HHH	glcm	Imc2	0.829754597
wavelet-HHH	glcm	Imc1	-0.16116876
wavelet-HHH	glcm	DifferenceAverage	3.496952767
wavelet-HHH	glcm	Id	0.359471947
wavelet-HHH	glcm	ClusterTendency	20.84257833
wavelet-HHH	firstorder	InterquartileRange	83.49754242
wavelet-HHH	firstorder	Skewness	0.239204953
wavelet-HHH	firstorder	Uniformity	0.100005251
wavelet-HHH	firstorder	Median	2.433736287
wavelet-HHH	firstorder	Energy	1918061.674
wavelet-HHH	firstorder	RobustMeanAbsoluteDeviation	37.07237812
wavelet-HHH	firstorder	MeanAbsoluteDeviation	60.09272045
wavelet-HHH	firstorder	TotalEnergy	51787665.19
wavelet-HHH	firstorder	Maximum	416.4757545
wavelet-HHH	firstorder	RootMeanSquared	83.36365406
wavelet-HHH	firstorder	90Percentile	96.07202932
wavelet-HHH	firstorder	Minimum	-280.6320514
wavelet-HHH	firstorder	Entropy	3.670680449
wavelet-HHH	firstorder	Range	697.1078059
wavelet-HHH	firstorder	Variance	6945.437676
wavelet-HHH	firstorder	10Percentile	-101.1409659
wavelet-HHH	firstorder	Kurtosis	5.41819349
wavelet-HHH	firstorder	Mean	2.015227441
wavelet-HHH	glrlm	ShortRunLowGrayLevelEmphasis	0.012256866
wavelet-HHH	glrlm	GrayLevelVariance	12.07192465
wavelet-HHH	glrlm	LowGrayLevelRunEmphasis	0.012661985
wavelet-HHH	glrlm	GrayLevelNonUniformityNormalized	0.093780617
wavelet-HHH	glrlm	RunVariance	0.097822848
wavelet-HHH	glrlm	GrayLevelNonUniformity	23.78205318
wavelet-HHH	glrlm	LongRunEmphasis	1.283935796
wavelet-HHH	glrlm	ShortRunHighGrayLevelEmphasis	159.882206
wavelet-HHH	glrlm	RunLengthNonUniformity	215.7489166
wavelet-HHH	glrlm	ShortRunEmphasis	0.938233818
wavelet-HHH	glrlm	LongRunHighGrayLevelEmphasis	216.6494233
wavelet-HHH	glrlm	RunPercentage	0.918617614
wavelet-HHH	glrlm	LongRunLowGrayLevelEmphasis	0.014531207
wavelet-HHH	glrlm	RunEntropy	4.088413733
wavelet-HHH	glrlm	HighGrayLevelRunEmphasis	170.110737
wavelet-HHH	glrlm	RunLengthNonUniformityNormalized	0.85052202
wavelet-HHH	glszm	GrayLevelVariance	22.23111189
wavelet-HHH	glszm	ZoneVariance	30.82312866
wavelet-HHH	glszm	GrayLevelNonUniformityNormalized	0.067866189
wavelet-HHH	glszm	SizeZoneNonUniformityNormalized	0.451480479
wavelet-HHH	glszm	SizeZoneNonUniformity	48.30841121
wavelet-HHH	glszm	GrayLevelNonUniformity	7.261682243
wavelet-HHH	glszm	LargeAreaEmphasis	37.47663551
wavelet-HHH	glszm	SmallAreaHighGrayLevelEmphasis	126.4490356
wavelet-HHH	glszm	ZonePercentage	0.387681159
wavelet-HHH	glszm	LargeAreaLowGrayLevelEmphasis	0.253353045
wavelet-HHH	glszm	LargeAreaHighGrayLevelEmphasis	6086.64486
wavelet-HHH	glszm	HighGrayLevelZoneEmphasis	178.8317757
wavelet-HHH	glszm	SmallAreaEmphasis	0.697590291

wavelet-HHH	glszm	LowGrayLevelZoneEmphasis	0.020500439
wavelet-HHH	glszm	ZoneEntropy	5.171791781
wavelet-HHH	glszm	SmallAreaLowGrayLevelEmphasis	0.018251101
wavelet-HHH	ngtdm	Coarseness	0.025378764
wavelet-HHH	ngtdm	Complexity	462.0678378
wavelet-HHH	ngtdm	Strength	3.423651841
wavelet-HHH	ngtdm	Contrast	0.138990368
wavelet-HHH	ngtdm	Busyness	0.13290358
wavelet-HLL	gldm	GrayLevelVariance	108.4972038
wavelet-HLL	gldm	HighGrayLevelEmphasis	748.2572464
wavelet-HLL	gldm	DependenceEntropy	6.265278244
wavelet-HLL	gldm	DependenceNonUniformity	99.53623188
wavelet-HLL	gldm	GrayLevelNonUniformity	8.514492754
wavelet-HLL	gldm	SmallDependenceEmphasis	0.593486312
wavelet-HLL	gldm	SmallDependenceHighGrayLevelEmphasis	473.8104891
wavelet-HLL	gldm	DependenceNonUniformityNormalized	0.360638521
wavelet-HLL	gldm	LargeDependenceEmphasis	4.036231884
wavelet-HLL	gldm	LargeDependenceLowGrayLevelEmphasis	0.01686213
wavelet-HLL	gldm	DependenceVariance	0.884320521
wavelet-HLL	gldm	LargeDependenceHighGrayLevelEmphasis	2667.713768
wavelet-HLL	gldm	SmallDependenceLowGrayLevelEmphasis	0.007008366
wavelet-HLL	gldm	LowGrayLevelEmphasis	0.008107426
wavelet-HLL	glcm	JointAverage	24.53366835
wavelet-HLL	glcm	SumAverage	49.06733669
wavelet-HLL	glcm	JointEntropy	8.123621353
wavelet-HLL	glcm	ClusterShade	1446.132121
wavelet-HLL	glcm	MaximumProbability	0.012713209
wavelet-HLL	glcm	Idmn	0.950436488
wavelet-HLL	glcm	JointEnergy	0.004040327
wavelet-HLL	glcm	Contrast	140.1509302
wavelet-HLL	glcm	DifferenceEntropy	4.383677522
wavelet-HLL	glcm	InverseVariance	0.136731386
wavelet-HLL	glcm	DifferenceVariance	49.81812645
wavelet-HLL	glcm	Idn	0.857176821
wavelet-HLL	glcm	Idm	0.124062732
wavelet-HLL	glcm	Correlation	0.235172987
wavelet-HLL	glcm	Autocorrelation	626.8791969
wavelet-HLL	glcm	SumEntropy	5.574038504
wavelet-HLL	glcm	SumSquares	94.96383895
wavelet-HLL	glcm	ClusterProminence	175111.4018
wavelet-HLL	glcm	Imc2	0.991974545
wavelet-HLL	glcm	Imc1	-0.408724335
wavelet-HLL	glcm	DifferenceAverage	9.059661826
wavelet-HLL	glcm	Id	0.204838863
wavelet-HLL	glcm	ClusterTendency	239.7044255
wavelet-HLL	firstorder	InterquartileRange	380.4718462
wavelet-HLL	firstorder	Skewness	0.104299105
wavelet-HLL	firstorder	Uniformity	0.030849611
wavelet-HLL	firstorder	Median	-78.13532009
wavelet-HLL	firstorder	Energy	20416860.71
wavelet-HLL	firstorder	RobustMeanAbsoluteDeviation	153.221645
wavelet-HLL	firstorder	MeanAbsoluteDeviation	211.6276495
wavelet-HLL	firstorder	TotalEnergy	551255239.2
wavelet-HLL	firstorder	Maximum	511.9212296

wavelet-HLL	firstorder	RootMeanSquared	271.9818615
wavelet-HLL	firstorder	90Percentile	284.508592
wavelet-HLL	firstorder	Minimum	-697.0171925
wavelet-HLL	firstorder	Entropy	5.219176947
wavelet-HLL	firstorder	Range	1208.938422
wavelet-HLL	firstorder	Variance	67575.77236
wavelet-HLL	firstorder	10Percentile	-393.9549378
wavelet-HLL	firstorder	Kurtosis	2.351618505
wavelet-HLL	firstorder	Mean	-79.98975341
wavelet-HLL	glrlm	ShortRunLowGrayLevelEmphasis	0.008210251
wavelet-HLL	glrlm	GrayLevelVariance	109.7456813
wavelet-HLL	glrlm	LowGrayLevelRunEmphasis	0.008272393
wavelet-HLL	glrlm	GrayLevelNonUniformityNormalized	0.03039591
wavelet-HLL	glrlm	RunVariance	0.031498833
wavelet-HLL	glrlm	GrayLevelNonUniformity	8.140601348
wavelet-HLL	glrlm	LongRunEmphasis	1.095596113
wavelet-HLL	glrlm	ShortRunHighGrayLevelEmphasis	736.2810759
wavelet-HLL	glrlm	RunLengthNonUniformity	252.4969753
wavelet-HLL	glrlm	ShortRunEmphasis	0.977083802
wavelet-HLL	glrlm	LongRunHighGrayLevelEmphasis	812.5331826
wavelet-HLL	glrlm	RunPercentage	0.970178372
wavelet-HLL	glrlm	LongRunLowGrayLevelEmphasis	0.008552283
wavelet-HLL	glrlm	RunEntropy	5.355670235
wavelet-HLL	glrlm	HighGrayLevelRunEmphasis	751.386158
wavelet-HLL	glrlm	RunLengthNonUniformityNormalized	0.942020189
wavelet-HLL	glszm	GrayLevelVariance	125.1923922
wavelet-HLL	glszm	ZoneVariance	1.389524801
wavelet-HLL	glszm	GrayLevelNonUniformityNormalized	0.0267661
wavelet-HLL	glszm	SizeZoneNonUniformityNormalized	0.577870274
wavelet-HLL	glszm	SizeZoneNonUniformity	107.483871
wavelet-HLL	glszm	GrayLevelNonUniformity	4.978494624
wavelet-HLL	glszm	LargeAreaEmphasis	3.591397849
wavelet-HLL	glszm	SmallAreaHighGrayLevelEmphasis	638.7250932
wavelet-HLL	glszm	ZonePercentage	0.673913043
wavelet-HLL	glszm	LargeAreaLowGrayLevelEmphasis	0.019166604
wavelet-HLL	glszm	LargeAreaHighGrayLevelEmphasis	2272.607527
wavelet-HLL	glszm	HighGrayLevelZoneEmphasis	791.7204301
wavelet-HLL	glszm	SmallAreaEmphasis	0.788575272
wavelet-HLL	glszm	LowGrayLevelZoneEmphasis	0.010660122
wavelet-HLL	glszm	ZoneEntropy	6.054785178
wavelet-HLL	glszm	SmallAreaLowGrayLevelEmphasis	0.010153539
wavelet-HLL	ngtdm	Coarseness	0.020016092
wavelet-HLL	ngtdm	Complexity	5348.178593
wavelet-HLL	ngtdm	Strength	15.10906887
wavelet-HLL	ngtdm	Contrast	0.687686423
wavelet-HLL	ngtdm	Busyness	0.054667985
wavelet-LHL	gldm	GrayLevelVariance	41.97840527
wavelet-LHL	gldm	HighGrayLevelEmphasis	376.8949275
wavelet-LHL	gldm	DependenceEntropy	6.184667791
wavelet-LHL	gldm	DependenceNonUniformity	82.03623188
wavelet-LHL	gldm	GrayLevelNonUniformity	13.42753623
wavelet-LHL	gldm	SmallDependenceEmphasis	0.521342671
wavelet-LHL	gldm	SmallDependenceHighGrayLevelEmphasis	199.2039403
wavelet-LHL	gldm	DependenceNonUniformityNormalized	0.297232724

wavelet-LHL	gldm	LargeDependenceEmphasis	6.760869565
wavelet-LHL	gldm	LargeDependenceLowGrayLevelEmphasis	0.027733929
wavelet-LHL	gldm	DependenceVariance	2.191188826
wavelet-LHL	gldm	LargeDependenceHighGrayLevelEmphasis	2544.307971
wavelet-LHL	gldm	SmallDependenceLowGrayLevelEmphasis	0.007079217
wavelet-LHL	gldm	LowGrayLevelEmphasis	0.009067358
wavelet-LHL	glcm	JointAverage	18.20231247
wavelet-LHL	glcm	SumAverage	36.40462495
wavelet-LHL	glcm	JointEntropy	7.741225443
wavelet-LHL	glcm	ClusterShade	191.2071479
wavelet-LHL	glcm	MaximumProbability	0.019338097
wavelet-LHL	glcm	Idmn	0.966034802
wavelet-LHL	glcm	JointEnergy	0.005734651
wavelet-LHL	glcm	Contrast	56.21952468
wavelet-LHL	glcm	DifferenceEntropy	3.840862396
wavelet-LHL	glcm	InverseVariance	0.19400198
wavelet-LHL	glcm	DifferenceVariance	23.34975744
wavelet-LHL	glcm	Idn	0.881046638
wavelet-LHL	glcm	Idm	0.179401631
wavelet-LHL	glcm	Correlation	0.249118431
wavelet-LHL	glcm	Autocorrelation	341.0223739
wavelet-LHL	glcm	SumEntropy	5.087232416
wavelet-LHL	glcm	SumSquares	37.79903277
wavelet-LHL	glcm	ClusterProminence	30753.63067
wavelet-LHL	glcm	Imc2	0.960341431
wavelet-LHL	glcm	Imc1	-0.284284912
wavelet-LHL	glcm	DifferenceAverage	5.623471352
wavelet-LHL	glcm	Id	0.269058341
wavelet-LHL	glcm	ClusterTendency	94.97660639
wavelet-LHL	firstorder	InterquartileRange	193.6401981
wavelet-LHL	firstorder	Skewness	0.145735854
wavelet-LHL	firstorder	Uniformity	0.048650494
wavelet-LHL	firstorder	Median	-21.62405289
wavelet-LHL	firstorder	Energy	7513719.566
wavelet-LHL	firstorder	RobustMeanAbsoluteDeviation	83.86452871
wavelet-LHL	firstorder	MeanAbsoluteDeviation	125.5988447
wavelet-LHL	firstorder	TotalEnergy	202870428.3
wavelet-LHL	firstorder	Maximum	454.00866
wavelet-LHL	firstorder	RootMeanSquared	164.995823
wavelet-LHL	firstorder	90Percentile	195.5498497
wavelet-LHL	firstorder	Minimum	-453.7160984
wavelet-LHL	firstorder	Entropy	4.616280715
wavelet-LHL	firstorder	Range	907.7247584
wavelet-LHL	firstorder	Variance	26313.61389
wavelet-LHL	firstorder	10Percentile	-246.2302765
wavelet-LHL	firstorder	Kurtosis	3.074117997
wavelet-LHL	firstorder	Mean	-30.16633426
wavelet-LHL	glrlm	ShortRunLowGrayLevelEmphasis	0.009205597
wavelet-LHL	glrlm	GrayLevelVariance	43.21012546
wavelet-LHL	glrlm	LowGrayLevelRunEmphasis	0.009318957
wavelet-LHL	glrlm	GrayLevelNonUniformityNormalized	0.047357745
wavelet-LHL	glrlm	RunVariance	0.05364389
wavelet-LHL	glrlm	GrayLevelNonUniformity	12.49967627
wavelet-LHL	glrlm	LongRunEmphasis	1.147837464

wavelet-LHL	glrlm	ShortRunHighGrayLevelEmphasis	365.2251551
wavelet-LHL	glrlm	RunLengthNonUniformity	243.0976731
wavelet-LHL	glrlm	ShortRunEmphasis	0.968444669
wavelet-LHL	glrlm	LongRunHighGrayLevelEmphasis	431.8071794
wavelet-LHL	glrlm	RunPercentage	0.956243032
wavelet-LHL	glrlm	LongRunLowGrayLevelEmphasis	0.0098492
wavelet-LHL	glrlm	RunEntropy	4.833988569
wavelet-LHL	glrlm	HighGrayLevelRunEmphasis	377.0954192
wavelet-LHL	glrlm	RunLengthNonUniformityNormalized	0.920828112
wavelet-LHL	glszm	GrayLevelVariance	59.55397785
wavelet-LHL	glszm	ZoneVariance	5.712118139
wavelet-LHL	glszm	GrayLevelNonUniformityNormalized	0.038906244
wavelet-LHL	glszm	SizeZoneNonUniformityNormalized	0.577832772
wavelet-LHL	glszm	SizeZoneNonUniformity	90.71974522
wavelet-LHL	glszm	GrayLevelNonUniformity	6.108280255
wavelet-LHL	glszm	LargeAreaEmphasis	8.802547771
wavelet-LHL	glszm	SmallAreaHighGrayLevelEmphasis	302.6646626
wavelet-LHL	glszm	ZonePercentage	0.56884058
wavelet-LHL	glszm	LargeAreaLowGrayLevelEmphasis	0.037499252
wavelet-LHL	glszm	LargeAreaHighGrayLevelEmphasis	3242.859873
wavelet-LHL	glszm	HighGrayLevelZoneEmphasis	383.0955414
wavelet-LHL	glszm	SmallAreaEmphasis	0.787338759
wavelet-LHL	glszm	LowGrayLevelZoneEmphasis	0.012998253
wavelet-LHL	glszm	ZoneEntropy	5.658332199
wavelet-LHL	glszm	SmallAreaLowGrayLevelEmphasis	0.011878786
wavelet-LHL	ngtdm	Coarseness	0.027352877
wavelet-LHL	ngtdm	Complexity	1603.820968
wavelet-LHL	ngtdm	Strength	8.922167412
wavelet-LHL	ngtdm	Contrast	0.307760426
wavelet-LHL	ngtdm	Busyness	0.067486732
wavelet-LHH	gldm	GrayLevelVariance	25.35868252
wavelet-LHH	gldm	HighGrayLevelEmphasis	577.4384058
wavelet-LHH	gldm	DependenceEntropy	5.93606932
wavelet-LHH	gldm	DependenceNonUniformity	64.23913043
wavelet-LHH	gldm	GrayLevelNonUniformity	20.5
wavelet-LHH	gldm	SmallDependenceEmphasis	0.454678704
wavelet-LHH	gldm	SmallDependenceHighGrayLevelEmphasis	278.8231285
wavelet-LHH	gldm	DependenceNonUniformityNormalized	0.232750473
wavelet-LHH	gldm	LargeDependenceEmphasis	9.905797101
wavelet-LHH	gldm	LargeDependenceLowGrayLevelEmphasis	0.022728586
wavelet-LHH	gldm	DependenceVariance	3.325456837
wavelet-LHH	gldm	LargeDependenceHighGrayLevelEmphasis	5414.068841
wavelet-LHH	gldm	SmallDependenceLowGrayLevelEmphasis	0.004689329
wavelet-LHH	gldm	LowGrayLevelEmphasis	0.005776234
wavelet-LHH	glcm	JointAverage	23.60301533
wavelet-LHH	glcm	SumAverage	47.20603066
wavelet-LHH	glcm	JointEntropy	7.108754098
wavelet-LHH	glcm	ClusterShade	-20.70598489
wavelet-LHH	glcm	MaximumProbability	0.033521325
wavelet-LHH	glcm	Idmn	0.975169655
wavelet-LHH	glcm	JointEnergy	0.010341981
wavelet-LHH	glcm	Contrast	47.60319744
wavelet-LHH	glcm	DifferenceEntropy	3.671372382
wavelet-LHH	glcm	InverseVariance	0.246857457

wavelet-LHH	glcm	DifferenceVariance	23.34294025
wavelet-LHH	glcm	Idn	0.902680051
wavelet-LHH	glcm	Idm	0.229747866
wavelet-LHH	glcm	Correlation	-0.043328011
wavelet-LHH	glcm	Autocorrelation	556.0805774
wavelet-LHH	glcm	SumEntropy	4.489216559
wavelet-LHH	glcm	SumSquares	22.75983523
wavelet-LHH	glcm	ClusterProminence	9368.747608
wavelet-LHH	glcm	Imc2	0.913198672
wavelet-LHH	glcm	Imc1	-0.225751793
wavelet-LHH	glcm	DifferenceAverage	4.864946614
wavelet-LHH	glcm	Id	0.313358416
wavelet-LHH	glcm	ClusterTendency	43.43614348
wavelet-LHH	firstorder	InterquartileRange	134.7391721
wavelet-LHH	firstorder	Skewness	-0.138129732
wavelet-LHH	firstorder	Uniformity	0.074275362
wavelet-LHH	firstorder	Median	-10.15202455
wavelet-LHH	firstorder	Energy	4371201.895
wavelet-LHH	firstorder	RobustMeanAbsoluteDeviation	54.47446686
wavelet-LHH	firstorder	MeanAbsoluteDeviation	89.73047038
wavelet-LHH	firstorder	TotalEnergy	118022451.2
wavelet-LHH	firstorder	Maximum	434.1902517
wavelet-LHH	firstorder	RootMeanSquared	125.8478765
wavelet-LHH	firstorder	90Percentile	156.3340693
wavelet-LHH	firstorder	Minimum	-567.1296398
wavelet-LHH	firstorder	Entropy	4.122084875
wavelet-LHH	firstorder	Range	1001.319891
wavelet-LHH	firstorder	Variance	15837.4755
wavelet-LHH	firstorder	10Percentile	-123.4786262
wavelet-LHH	firstorder	Kurtosis	5.46853984
wavelet-LHH	firstorder	Mean	-0.461005451
wavelet-LHH	glrlm	ShortRunLowGrayLevelEmphasis	0.005939425
wavelet-LHH	glrlm	GrayLevelVariance	26.59206195
wavelet-LHH	glrlm	LowGrayLevelRunEmphasis	0.006024592
wavelet-LHH	glrlm	GrayLevelNonUniformityNormalized	0.070791589
wavelet-LHH	glrlm	RunVariance	0.075468012
wavelet-LHH	glrlm	GrayLevelNonUniformity	18.36653522
wavelet-LHH	glrlm	LongRunEmphasis	1.209524453
wavelet-LHH	glrlm	ShortRunHighGrayLevelEmphasis	555.5202715
wavelet-LHH	glrlm	RunLengthNonUniformity	231.5918954
wavelet-LHH	glrlm	ShortRunEmphasis	0.955983935
wavelet-LHH	glrlm	LongRunHighGrayLevelEmphasis	693.4002571
wavelet-LHH	glrlm	RunPercentage	0.939799331
wavelet-LHH	glrlm	LongRunLowGrayLevelEmphasis	0.006429792
wavelet-LHH	glrlm	RunEntropy	4.434900473
wavelet-LHH	glrlm	HighGrayLevelRunEmphasis	579.5550254
wavelet-LHH	glrlm	RunLengthNonUniformityNormalized	0.891973148
wavelet-LHH	glszm	GrayLevelVariance	42.70134083
wavelet-LHH	glszm	ZoneVariance	13.46972318
wavelet-LHH	glszm	GrayLevelNonUniformityNormalized	0.051362457
wavelet-LHH	glszm	SizeZoneNonUniformityNormalized	0.573529412
wavelet-LHH	glszm	SizeZoneNonUniformity	78
wavelet-LHH	glszm	GrayLevelNonUniformity	6.985294118
wavelet-LHH	glszm	LargeAreaEmphasis	17.58823529

wavelet-LHH	glszm	SmallAreaHighGrayLevelEmphasis	489.0673866
wavelet-LHH	glszm	ZonePercentage	0.492753623
wavelet-LHH	glszm	LargeAreaLowGrayLevelEmphasis	0.041393224
wavelet-LHH	glszm	LargeAreaHighGrayLevelEmphasis	9481.066176
wavelet-LHH	glszm	HighGrayLevelZoneEmphasis	608.1617647
wavelet-LHH	glszm	SmallAreaEmphasis	0.783749664
wavelet-LHH	glszm	LowGrayLevelZoneEmphasis	0.009703522
wavelet-LHH	glszm	ZoneEntropy	5.383501173
wavelet-LHH	glszm	SmallAreaLowGrayLevelEmphasis	0.009235118
wavelet-LHH	ngtdm	Coarseness	0.022531579
wavelet-LHH	ngtdm	Complexity	1372.702261
wavelet-LHH	ngtdm	Strength	6.681861082
wavelet-LHH	ngtdm	Contrast	0.238199345
wavelet-LHH	ngtdm	Busyness	0.058618901
wavelet-LLH	gldm	GrayLevelVariance	112.7217496
wavelet-LLH	gldm	HighGrayLevelEmphasis	994.1231884
wavelet-LLH	gldm	DependenceEntropy	6.354919195
wavelet-LLH	gldm	DependenceNonUniformity	85.98550725
wavelet-LLH	gldm	GrayLevelNonUniformity	10.60144928
wavelet-LLH	gldm	SmallDependenceEmphasis	0.549564566
wavelet-LLH	gldm	SmallDependenceHighGrayLevelEmphasis	566.8155111
wavelet-LLH	gldm	DependenceNonUniformityNormalized	0.311541693
wavelet-LLH	gldm	LargeDependenceEmphasis	6.115942029
wavelet-LLH	gldm	LargeDependenceLowGrayLevelEmphasis	0.011644283
wavelet-LLH	gldm	DependenceVariance	1.910470489
wavelet-LLH	gldm	LargeDependenceHighGrayLevelEmphasis	5927.297101
wavelet-LLH	gldm	SmallDependenceLowGrayLevelEmphasis	0.004756583
wavelet-LLH	gldm	LowGrayLevelEmphasis	0.005415483
wavelet-LLH	glcm	JointAverage	29.18014842
wavelet-LLH	glcm	SumAverage	58.36029684
wavelet-LLH	glcm	JointEntropy	7.944002531
wavelet-LLH	glcm	ClusterShade	3602.713967
wavelet-LLH	glcm	MaximumProbability	0.019578253
wavelet-LLH	glcm	Idmn	0.975535992
wavelet-LLH	glcm	JointEnergy	0.004966615
wavelet-LLH	glcm	Contrast	172.4076913
wavelet-LLH	glcm	DifferenceEntropy	4.403702382
wavelet-LLH	glcm	InverseVariance	0.141923798
wavelet-LLH	glcm	DifferenceVariance	88.70473726
wavelet-LLH	glcm	Idn	0.905708027
wavelet-LLH	glcm	Idm	0.144952642
wavelet-LLH	glcm	Correlation	0.098368023
wavelet-LLH	glcm	Autocorrelation	862.5500628
wavelet-LLH	glcm	SumEntropy	5.403996069
wavelet-LLH	glcm	SumSquares	97.23707383
wavelet-LLH	glcm	ClusterProminence	351041.4526
wavelet-LLH	glcm	Imc2	0.989118493
wavelet-LLH	glcm	Imc1	-0.390320792
wavelet-LLH	glcm	DifferenceAverage	8.893616966
wavelet-LLH	glcm	Id	0.227366757
wavelet-LLH	glcm	ClusterTendency	216.540604
wavelet-LLH	firstorder	InterquartileRange	250.314113
wavelet-LLH	firstorder	Skewness	0.972471663
wavelet-LLH	firstorder	Uniformity	0.038411048

wavelet-LLH	firstorder	Median	-56.77217172
wavelet-LLH	firstorder	Energy	19984689.06
wavelet-LLH	firstorder	RobustMeanAbsoluteDeviation	111.9811085
wavelet-LLH	firstorder	MeanAbsoluteDeviation	188.0762838
wavelet-LLH	firstorder	TotalEnergy	539586604.7
wavelet-LLH	firstorder	Maximum	1148.25271
wavelet-LLH	firstorder	RootMeanSquared	269.0878922
wavelet-LLH	firstorder	90Percentile	228.4255616
wavelet-LLH	firstorder	Minimum	-774.968352
wavelet-LLH	firstorder	Entropy	5.084449673
wavelet-LLH	firstorder	Range	1923.221062
wavelet-LLH	firstorder	Variance	70311.80476
wavelet-LLH	firstorder	10Percentile	-349.2798442
wavelet-LLH	firstorder	Kurtosis	5.903297781
wavelet-LLH	firstorder	Mean	-45.7874322
wavelet-LLH	glrlm	ShortRunLowGrayLevelEmphasis	0.005548593
wavelet-LLH	glrlm	GrayLevelVariance	115.9585773
wavelet-LLH	glrlm	LowGrayLevelRunEmphasis	0.005589017
wavelet-LLH	glrlm	GrayLevelNonUniformityNormalized	0.037083921
wavelet-LLH	glrlm	RunVariance	0.043850583
wavelet-LLH	glrlm	GrayLevelNonUniformity	9.823099198
wavelet-LLH	glrlm	LongRunEmphasis	1.130317463
wavelet-LLH	glrlm	ShortRunHighGrayLevelEmphasis	966.3960191
wavelet-LLH	glrlm	RunLengthNonUniformity	244.3427495
wavelet-LLH	glrlm	ShortRunEmphasis	0.969382047
wavelet-LLH	glrlm	LongRunHighGrayLevelEmphasis	1120.621773
wavelet-LLH	glrlm	RunPercentage	0.959587514
wavelet-LLH	glrlm	LongRunLowGrayLevelEmphasis	0.005758558
wavelet-LLH	glrlm	RunEntropy	5.284948236
wavelet-LLH	glrlm	HighGrayLevelRunEmphasis	995.6664096
wavelet-LLH	glrlm	RunLengthNonUniformityNormalized	0.92235368
wavelet-LLH	glszm	GrayLevelVariance	157.1020623
wavelet-LLH	glszm	ZoneVariance	3.332866496
wavelet-LLH	glszm	GrayLevelNonUniformityNormalized	0.028745492
wavelet-LLH	glszm	SizeZoneNonUniformityNormalized	0.584258254
wavelet-LLH	glszm	SizeZoneNonUniformity	98.73964497
wavelet-LLH	glszm	GrayLevelNonUniformity	4.857988166
wavelet-LLH	glszm	LargeAreaEmphasis	6
wavelet-LLH	glszm	SmallAreaHighGrayLevelEmphasis	827.8388925
wavelet-LLH	glszm	ZonePercentage	0.612318841
wavelet-LLH	glszm	LargeAreaLowGrayLevelEmphasis	0.013965378
wavelet-LLH	glszm	LargeAreaHighGrayLevelEmphasis	5770.994083
wavelet-LLH	glszm	HighGrayLevelZoneEmphasis	1027.526627
wavelet-LLH	glszm	SmallAreaEmphasis	0.791167953
wavelet-LLH	glszm	LowGrayLevelZoneEmphasis	0.007960798
wavelet-LLH	glszm	ZoneEntropy	6.068998188
wavelet-LLH	glszm	SmallAreaLowGrayLevelEmphasis	0.00760382
wavelet-LLH	ngtdm	Coarseness	0.023715848
wavelet-LLH	ngtdm	Complexity	7379.284744
wavelet-LLH	ngtdm	Strength	25.20071277
wavelet-LLH	ngtdm	Contrast	0.579554062
wavelet-LLH	ngtdm	Busyness	0.028196139
wavelet-LLL	gldm	GrayLevelVariance	582.4531611
wavelet-LLL	gldm	HighGrayLevelEmphasis	3711.963768

wavelet-LLL	gldm	DependenceEntropy	6.579756558
wavelet-LLL	gldm	DependenceNonUniformity	165.5724638
wavelet-LLL	gldm	GrayLevelNonUniformity	4.449275362
wavelet-LLL	gldm	SmallDependenceEmphasis	0.802614139
wavelet-LLL	gldm	SmallDependenceHighGrayLevelEmphasis	2545.513434
wavelet-LLL	gldm	DependenceNonUniformityNormalized	0.599900231
wavelet-LLL	gldm	LargeDependenceEmphasis	3.094202899
wavelet-LLL	gldm	LargeDependenceLowGrayLevelEmphasis	0.009524652
wavelet-LLL	gldm	DependenceVariance	1.035601764
wavelet-LLL	gldm	LargeDependenceHighGrayLevelEmphasis	18006.1087
wavelet-LLL	gldm	SmallDependenceLowGrayLevelEmphasis	0.009040617
wavelet-LLL	gldm	LowGrayLevelEmphasis	0.009102044
wavelet-LLL	glcm	JointAverage	62.70881017
wavelet-LLL	glcm	SumAverage	125.4176203
wavelet-LLL	glcm	JointEntropy	8.424694714
wavelet-LLL	glcm	ClusterShade	-23848.39102
wavelet-LLL	glcm	MaximumProbability	0.010777064
wavelet-LLL	glcm	Idmn	0.936291318
wavelet-LLL	glcm	JointEnergy	0.003104392
wavelet-LLL	glcm	Contrast	679.0281951
wavelet-LLL	glcm	DifferenceEntropy	5.403467588
wavelet-LLL	glcm	InverseVariance	0.06378017
wavelet-LLL	glcm	DifferenceVariance	250.5481221
wavelet-LLL	glcm	Idn	0.838596901
wavelet-LLL	glcm	Idm	0.063394442
wavelet-LLL	glcm	Correlation	0.332931988
wavelet-LLL	glcm	Autocorrelation	4108.965318
wavelet-LLL	glcm	SumEntropy	6.429612961
wavelet-LLL	glcm	SumSquares	513.7060197
wavelet-LLL	glcm	ClusterProminence	4394958.524
wavelet-LLL	glcm	Imc2	0.99959122
wavelet-LLL	glcm	Imc1	-0.594198357
wavelet-LLL	glcm	DifferenceAverage	20.24212717
wavelet-LLL	glcm	Id	0.124168695
wavelet-LLL	glcm	ClusterTendency	1375.795883
wavelet-LLL	firstorder	InterquartileRange	1071.238393
wavelet-LLL	firstorder	Skewness	-0.269906982
wavelet-LLL	firstorder	Uniformity	0.016120563
wavelet-LLL	firstorder	Median	-564.7385892
wavelet-LLL	firstorder	Energy	205005648.6
wavelet-LLL	firstorder	RobustMeanAbsoluteDeviation	419.2245947
wavelet-LLL	firstorder	MeanAbsoluteDeviation	523.7218606
wavelet-LLL	firstorder	TotalEnergy	5535152513
wavelet-LLL	firstorder	Maximum	340.5577511
wavelet-LLL	firstorder	RootMeanSquared	861.843425
wavelet-LLL	firstorder	90Percentile	139.0205545
wavelet-LLL	firstorder	Minimum	-1992.514165
wavelet-LLL	firstorder	Entropy	6.159806095
wavelet-LLL	firstorder	Range	2333.071916
wavelet-LLL	firstorder	Variance	363977.0026
wavelet-LLL	firstorder	10Percentile	-1415.104832
wavelet-LLL	firstorder	Kurtosis	1.934643812
wavelet-LLL	firstorder	Mean	-615.4649353
wavelet-LLL	glrlm	ShortRunLowGrayLevelEmphasis	0.009251119

wavelet-LLL	glrlm	GrayLevelVariance	579.4298988
wavelet-LLL	glrlm	LowGrayLevelRunEmphasis	0.009254145
wavelet-LLL	glrlm	GrayLevelNonUniformityNormalized	0.015806003
wavelet-LLL	glrlm	RunVariance	0.018436821
wavelet-LLL	glrlm	GrayLevelNonUniformity	4.290116895
wavelet-LLL	glrlm	LongRunEmphasis	1.053221876
wavelet-LLL	glrlm	ShortRunHighGrayLevelEmphasis	3584.67473
wavelet-LLL	glrlm	RunLengthNonUniformity	262.8177764
wavelet-LLL	glrlm	ShortRunEmphasis	0.987654932
wavelet-LLL	glrlm	LongRunHighGrayLevelEmphasis	4011.216485
wavelet-LLL	glrlm	RunPercentage	0.983277592
wavelet-LLL	glrlm	LongRunLowGrayLevelEmphasis	0.009266768
wavelet-LLL	glrlm	RunEntropy	6.221696336
wavelet-LLL	glrlm	HighGrayLevelRunEmphasis	3664.300583
wavelet-LLL	glrlm	RunLengthNonUniformityNormalized	0.968156436
wavelet-LLL	glszm	GrayLevelVariance	551.2697239
wavelet-LLL	glszm	ZoneVariance	0.523339908
wavelet-LLL	glszm	GrayLevelNonUniformityNormalized	0.015121631
wavelet-LLL	glszm	SizeZoneNonUniformityNormalized	0.804551099
wavelet-LLL	glszm	SizeZoneNonUniformity	188.2649573
wavelet-LLL	glszm	GrayLevelNonUniformity	3.538461538
wavelet-LLL	glszm	LargeAreaEmphasis	1.914529915
wavelet-LLL	glszm	SmallAreaHighGrayLevelEmphasis	2824.818521
wavelet-LLL	glszm	ZonePercentage	0.847826087
wavelet-LLL	glszm	LargeAreaLowGrayLevelEmphasis	0.010889689
wavelet-LLL	glszm	LargeAreaHighGrayLevelEmphasis	9359.666667
wavelet-LLL	glszm	HighGrayLevelZoneEmphasis	3280.662393
wavelet-LLL	glszm	SmallAreaEmphasis	0.914961354
wavelet-LLL	glszm	LowGrayLevelZoneEmphasis	0.01068417
wavelet-LLL	glszm	ZoneEntropy	6.401666953
wavelet-LLL	glszm	SmallAreaLowGrayLevelEmphasis	0.010652189
wavelet-LLL	ngtdm	Coarseness	0.017358041
wavelet-LLL	ngtdm	Complexity	41168.11347
wavelet-LLL	ngtdm	Strength	54.77709829
wavelet-LLL	ngtdm	Contrast	2.289823952
wavelet-LLL	ngtdm	Busyness	0.013166778
wavelet-HHL	gldm	GrayLevelVariance	15.31005829
wavelet-HHL	gldm	HighGrayLevelEmphasis	202.5833333
wavelet-HHL	gldm	DependenceEntropy	5.983739948
wavelet-HHL	gldm	DependenceNonUniformity	57.39130435
wavelet-HHL	gldm	GrayLevelNonUniformity	21.20289855
wavelet-HHL	gldm	SmallDependenceEmphasis	0.373178548
wavelet-HHL	gldm	SmallDependenceHighGrayLevelEmphasis	80.54650981
wavelet-HHL	gldm	DependenceNonUniformityNormalized	0.207939509
wavelet-HHL	gldm	LargeDependenceEmphasis	10.52898551
wavelet-HHL	gldm	LargeDependenceLowGrayLevelEmphasis	0.072262997
wavelet-HHL	gldm	DependenceVariance	3.026149968
wavelet-HHL	gldm	LargeDependenceHighGrayLevelEmphasis	1919.663043
wavelet-HHL	gldm	SmallDependenceLowGrayLevelEmphasis	0.008760481
wavelet-HHL	gldm	LowGrayLevelEmphasis	0.012764241
wavelet-HHL	glcm	JointAverage	13.65741544
wavelet-HHL	glcm	SumAverage	27.31483087
wavelet-HHL	glcm	JointEntropy	7.110571807
wavelet-HHL	glcm	ClusterShade	-26.79505191

wavelet-HHL	glcm	MaximumProbability	0.026012242
wavelet-HHL	glcm	Idmn	0.954116783
wavelet-HHL	glcm	JointEnergy	0.009107267
wavelet-HHL	glcm	Contrast	30.9859095
wavelet-HHL	glcm	DifferenceEntropy	3.486125798
wavelet-HHL	glcm	InverseVariance	0.237887375
wavelet-HHL	glcm	DifferenceVariance	12.63602748
wavelet-HHL	glcm	Idn	0.863189321
wavelet-HHL	glcm	Idm	0.235817713
wavelet-HHL	glcm	Correlation	-0.001791544
wavelet-HHL	glcm	Autocorrelation	186.5127407
wavelet-HHL	glcm	SumEntropy	4.343607727
wavelet-HHL	glcm	SumSquares	15.46747081
wavelet-HHL	glcm	ClusterProminence	3491.570578
wavelet-HHL	glcm	Imc2	0.882656611
wavelet-HHL	glcm	Imc1	-0.193548286
wavelet-HHL	glcm	DifferenceAverage	4.219482238
wavelet-HHL	glcm	Id	0.323310498
wavelet-HHL	glcm	ClusterTendency	30.88397375
wavelet-HHL	firstorder	InterquartileRange	124.2504275
wavelet-HHL	firstorder	Skewness	-0.099056839
wavelet-HHL	firstorder	Uniformity	0.076822096
wavelet-HHL	firstorder	Median	-2.056747312
wavelet-HHL	firstorder	Energy	2681799.835
wavelet-HHL	firstorder	RobustMeanAbsoluteDeviation	52.53611048
wavelet-HHL	firstorder	MeanAbsoluteDeviation	77.24796377
wavelet-HHL	firstorder	TotalEnergy	72408595.54
wavelet-HHL	firstorder	Maximum	274.4623182
wavelet-HHL	firstorder	RootMeanSquared	98.57315085
wavelet-HHL	firstorder	90Percentile	136.5075518
wavelet-HHL	firstorder	Minimum	-312.6139999
wavelet-HHL	firstorder	Entropy	3.945016953
wavelet-HHL	firstorder	Range	587.0763181
wavelet-HHL	firstorder	Variance	9693.001069
wavelet-HHL	firstorder	10Percentile	-109.3418555
wavelet-HHL	firstorder	Kurtosis	3.401616257
wavelet-HHL	firstorder	Mean	4.864668381
wavelet-HHL	glrlm	ShortRunLowGrayLevelEmphasis	0.012926515
wavelet-HHL	glrlm	GrayLevelVariance	15.92912515
wavelet-HHL	glrlm	LowGrayLevelRunEmphasis	0.013235672
wavelet-HHL	glrlm	GrayLevelNonUniformityNormalized	0.074854714
wavelet-HHL	glrlm	RunVariance	0.084657769
wavelet-HHL	glrlm	GrayLevelNonUniformity	19.27963823
wavelet-HHL	glrlm	LongRunEmphasis	1.235339102
wavelet-HHL	glrlm	ShortRunHighGrayLevelEmphasis	194.4758057
wavelet-HHL	glrlm	RunLengthNonUniformity	226.6176018
wavelet-HHL	glrlm	ShortRunEmphasis	0.950528471
wavelet-HHL	glrlm	LongRunHighGrayLevelEmphasis	247.9914968
wavelet-HHL	glrlm	RunPercentage	0.933110368
wavelet-HHL	glrlm	LongRunLowGrayLevelEmphasis	0.014703443
wavelet-HHL	glrlm	RunEntropy	4.27747409
wavelet-HHL	glrlm	HighGrayLevelRunEmphasis	203.6934224
wavelet-HHL	glrlm	RunLengthNonUniformityNormalized	0.879047562
wavelet-HHL	glszm	GrayLevelVariance	24.51083963

wavelet-HHL	glszm	ZoneVariance	9.64592896
wavelet-HHL	glszm	GrayLevelNonUniformityNormalized	0.060377092
wavelet-HHL	glszm	SizeZoneNonUniformityNormalized	0.426170468
wavelet-HHL	glszm	SizeZoneNonUniformity	50.71428571
wavelet-HHL	glszm	GrayLevelNonUniformity	7.18487395
wavelet-HHL	glszm	LargeAreaEmphasis	15.02521008
wavelet-HHL	glszm	SmallAreaHighGrayLevelEmphasis	149.991862
wavelet-HHL	glszm	ZonePercentage	0.43115942
wavelet-HHL	glszm	LargeAreaLowGrayLevelEmphasis	0.112138938
wavelet-HHL	glszm	LargeAreaHighGrayLevelEmphasis	2612.159664
wavelet-HHL	glszm	HighGrayLevelZoneEmphasis	217.697479
wavelet-HHL	glszm	SmallAreaEmphasis	0.677753055
wavelet-HHL	glszm	LowGrayLevelZoneEmphasis	0.021183653
wavelet-HHL	glszm	ZoneEntropy	5.494654979
wavelet-HHL	glszm	SmallAreaLowGrayLevelEmphasis	0.019063547
wavelet-HHL	ngtdm	Coarseness	0.020905419
wavelet-HHL	ngtdm	Complexity	652.45611
wavelet-HHL	ngtdm	Strength	3.358619188
wavelet-HHL	ngtdm	Contrast	0.190671941
wavelet-HHL	ngtdm	Busyness	0.153944947
wavelet-HLH	gldm	GrayLevelVariance	18.99657372
wavelet-HLH	gldm	HighGrayLevelEmphasis	262.9673913
wavelet-HLH	gldm	DependenceEntropy	5.737733107
wavelet-HLH	gldm	DependenceNonUniformity	71.89130435
wavelet-HLH	gldm	GrayLevelNonUniformity	18.66666667
wavelet-HLH	gldm	SmallDependenceEmphasis	0.400312922
wavelet-HLH	gldm	SmallDependenceHighGrayLevelEmphasis	106.3783387
wavelet-HLH	gldm	DependenceNonUniformityNormalized	0.26047574
wavelet-HLH	gldm	LargeDependenceEmphasis	8.282608696
wavelet-HLH	gldm	LargeDependenceLowGrayLevelEmphasis	0.044369377
wavelet-HLH	gldm	DependenceVariance	2.31910313
wavelet-HLH	gldm	LargeDependenceHighGrayLevelEmphasis	1977.684783
wavelet-HLH	gldm	SmallDependenceLowGrayLevelEmphasis	0.006177006
wavelet-HLH	gldm	LowGrayLevelEmphasis	0.00897881
wavelet-HLH	glcm	JointAverage	15.67149807
wavelet-HLH	glcm	SumAverage	31.34299613
wavelet-HLH	glcm	JointEntropy	7.353658034
wavelet-HLH	glcm	ClusterShade	12.91992475
wavelet-HLH	glcm	MaximumProbability	0.025149179
wavelet-HLH	glcm	Idmn	0.959786138
wavelet-HLH	glcm	JointEnergy	0.007610158
wavelet-HLH	glcm	Contrast	35.98625259
wavelet-HLH	glcm	DifferenceEntropy	3.601404841
wavelet-HLH	glcm	InverseVariance	0.201649058
wavelet-HLH	glcm	DifferenceVariance	13.69120572
wavelet-HLH	glcm	Idn	0.867238131
wavelet-HLH	glcm	Idm	0.19938999
wavelet-HLH	glcm	Correlation	0.018474584
wavelet-HLH	glcm	Autocorrelation	245.9117364
wavelet-HLH	glcm	SumEntropy	4.505876566
wavelet-HLH	glcm	SumSquares	18.28717409
wavelet-HLH	glcm	ClusterProminence	4193.529058
wavelet-HLH	glcm	Imc2	0.886852037
wavelet-HLH	glcm	Imc1	-0.191458736

wavelet-HLH	glcm	DifferenceAverage	4.676231389
wavelet-HLH	glcm	Id	0.292027254
wavelet-HLH	glcm	ClusterTendency	37.16244377
wavelet-HLH	firstorder	InterquartileRange	150.937609
wavelet-HLH	firstorder	Skewness	0.0356386
wavelet-HLH	firstorder	Uniformity	0.06763285
wavelet-HLH	firstorder	Median	-3.939378094
wavelet-HLH	firstorder	Energy	3250248.571
wavelet-HLH	firstorder	RobustMeanAbsoluteDeviation	61.78797518
wavelet-HLH	firstorder	MeanAbsoluteDeviation	87.09781338
wavelet-HLH	firstorder	TotalEnergy	87756711.43
wavelet-HLH	firstorder	Maximum	301.8129473
wavelet-HLH	firstorder	RootMeanSquared	108.5184912
wavelet-HLH	firstorder	90Percentile	155.374027
wavelet-HLH	firstorder	Minimum	-357.6645861
wavelet-HLH	firstorder	Entropy	4.087685345
wavelet-HLH	firstorder	Range	659.4775334
wavelet-HLH	firstorder	Variance	11766.12998
wavelet-HLH	firstorder	10Percentile	-131.1153367
wavelet-HLH	firstorder	Kurtosis	2.856767375
wavelet-HLH	firstorder	Mean	3.183230515
wavelet-HLH	glrlm	ShortRunLowGrayLevelEmphasis	0.009033511
wavelet-HLH	glrlm	GrayLevelVariance	19.53288615
wavelet-HLH	glrlm	LowGrayLevelRunEmphasis	0.009230941
wavelet-HLH	glrlm	GrayLevelNonUniformityNormalized	0.066108274
wavelet-HLH	glrlm	RunVariance	0.066123998
wavelet-HLH	glrlm	GrayLevelNonUniformity	17.23382347
wavelet-HLH	glrlm	LongRunEmphasis	1.190835333
wavelet-HLH	glrlm	ShortRunHighGrayLevelEmphasis	253.5326249
wavelet-HLH	glrlm	RunLengthNonUniformity	234.4220552
wavelet-HLH	glrlm	ShortRunEmphasis	0.958324663
wavelet-HLH	glrlm	LongRunHighGrayLevelEmphasis	310.8491578
wavelet-HLH	glrlm	RunPercentage	0.944537347
wavelet-HLH	glrlm	LongRunLowGrayLevelEmphasis	0.010145124
wavelet-HLH	glrlm	RunEntropy	4.3560502
wavelet-HLH	glrlm	HighGrayLevelRunEmphasis	263.9081713
wavelet-HLH	glrlm	RunLengthNonUniformityNormalized	0.897653305
wavelet-HLH	glszm	GrayLevelVariance	26.83994959
wavelet-HLH	glszm	ZoneVariance	5.84057971
wavelet-HLH	glszm	GrayLevelNonUniformityNormalized	0.059126234
wavelet-HLH	glszm	SizeZoneNonUniformityNormalized	0.392039488
wavelet-HLH	glszm	SizeZoneNonUniformity	54.10144928
wavelet-HLH	glszm	GrayLevelNonUniformity	8.15942029
wavelet-HLH	glszm	LargeAreaEmphasis	9.84057971
wavelet-HLH	glszm	SmallAreaHighGrayLevelEmphasis	169.2443309
wavelet-HLH	glszm	ZonePercentage	0.5
wavelet-HLH	glszm	LargeAreaLowGrayLevelEmphasis	0.054951616
wavelet-HLH	glszm	LargeAreaHighGrayLevelEmphasis	2331.615942
wavelet-HLH	glszm	HighGrayLevelZoneEmphasis	269.115942
wavelet-HLH	glszm	SmallAreaEmphasis	0.637696747
wavelet-HLH	glszm	LowGrayLevelZoneEmphasis	0.01328075
wavelet-HLH	glszm	ZoneEntropy	5.435754965
wavelet-HLH	glszm	SmallAreaLowGrayLevelEmphasis	0.011594305
wavelet-HLH	ngtdm	Coarseness	0.021213251

wavelet-HLH	ngtdm	Complexity	706.8984962
wavelet-HLH	ngtdm	Strength	3.29952043
wavelet-HLH	ngtdm	Contrast	0.260044433
wavelet-HLH	ngtdm	Busyness	0.154002364

Image type	Feature Class	Feature Name	5: RTSTRUCT: C99-DNU_segment_ITV
general	info	BoundingBox	(0, 3, 0, 12, 10, 10)
general	info	EnabledImageTypes	{u'Original': {}, u'Wavelet': {}}
general	info	GeneralSettings	{'distances': [1], 'additionalInfo': True, 'force2D': False, 'interpolator': 'sitkBSpline', 'resampledPixelSpacing': [3.0], 'label': 1, 'normalizeScale': 1, 'normalize': False, 'force2Ddimension': 0, 'removeOutliers': None, 'minimumROISize': None, u'binWidth': 25.0, 'minimumROIDimensions': 1, u'correctMask': True, 'preCrop': False, u'symmetricalGLCM': True, 'resegmentRange': None, 'padDistance': 5}
general	info	ImageHash	55fd05158524f46486093103894daba5afe4df18
general	info	ImageSpacing	(0.9765624999999998, 0.9765624999999998, 1.9999999999999996)
general	info	MaskHash	633e3f8839b7db403f68de34ba35f444602f6c91
general	info	NumpyVersion	1.13.1
general	info	PyWaveletVersion	0.5.2
general	info	SimpleITKVersion	1.1.0
general	info	Version	1.3.0.post102+g1b0eb74
general	info	VolumeNum	1
general	info	VoxelNum	145
original	shape	Maximum3DDiameter	40.36087214
original	shape	Maximum2DDiameterSlice	27.16615541
original	shape	Sphericity	0.558727255
original	shape	MinorAxis	21.10596198
original	shape	Elongation	0.462367615
original	shape	SurfaceVolumeRatio	0.549170262
original	shape	Volume	3915
original	shape	MajorAxis	45.6475784
original	shape	SurfaceArea	2150.001577
original	shape	Flatness	0.261062003
original	shape	LeastAxis	11.91684824
original	shape	Maximum2DDiameterColumn	27
original	shape	Maximum2DDiameterRow	30
original	gldm	GrayLevelVariance	61.75524376
original	gldm	HighGrayLevelEmphasis	412.6068966
original	gldm	DependenceEntropy	5.577593315
original	gldm	DependenceNonUniformity	58.77931034
original	gldm	GrayLevelNonUniformity	5.910344828
original	gldm	SmallDependenceEmphasis	0.647821839
original	gldm	SmallDependenceHighGrayLevelEmphasis	233.2907969
original	gldm	DependenceNonUniformityNormalized	0.405374554
original	gldm	LargeDependenceEmphasis	3.689655172
original	gldm	LargeDependenceLowGrayLevelEmphasis	0.031144276
original	gldm	DependenceVariance	0.881141498

original	gldm	LargeDependenceHighGrayLevelEmphasis	1956.331034
original	gldm	SmallDependenceLowGrayLevelEmphasis	0.016384939
original	gldm	LowGrayLevelEmphasis	0.018645394
original	glcm	JointAverage	19.74611128
original	glcm	SumAverage	39.49222257
original	glcm	JointEntropy	6.842587721
original	glcm	ClusterShade	-317.8832222
original	glcm	MaximumProbability	0.021410558
original	glcm	Idmn	0.926249486
original	glcm	JointEnergy	0.009412349
original	glcm	Contrast	106.091459
original	glcm	DifferenceEntropy	4.116305144
original	glcm	InverseVariance	0.121505819
original	glcm	DifferenceVariance	40.46681568
original	glcm	Idn	0.826000352
original	glcm	Idm	0.131381587
original	glcm	Correlation	0.165570372
original	glcm	Autocorrelation	400.5325496
original	glcm	SumEntropy	4.983051851
original	glcm	SumSquares	63.1759854
original	glcm	ClusterProminence	57417.09116
original	glcm	Imc2	0.995484794
original	glcm	Imc1	-0.519394939
original	glcm	DifferenceAverage	8.050042376
original	glcm	Id	0.217200449
original	glcm	ClusterTendency	146.6124826
original	firstorder	InterquartileRange	293
original	firstorder	Skewness	-0.254208599
original	firstorder	Uniformity	0.040760999
original	firstorder	Median	-291
original	firstorder	Energy	18181282
original	firstorder	RobustMeanAbsoluteDeviation	119.0564839
original	firstorder	MeanAbsoluteDeviation	161.762283
original	firstorder	TotalEnergy	490894614
original	firstorder	Maximum	90
original	firstorder	RootMeanSquared	354.1018946
original	firstorder	90Percentile	-40.4
original	firstorder	Minimum	-738
original	firstorder	Entropy	4.749343386
original	firstorder	Range	828
original	firstorder	Variance	38651.80851
original	firstorder	10Percentile	-568.8
original	firstorder	Kurtosis	2.261924305
original	firstorder	Mean	-294.5103448
original	glrlm	ShortRunLowGrayLevelEmphasis	0.018895116
original	glrlm	GrayLevelVariance	61.60422152
original	glrlm	LowGrayLevelRunEmphasis	0.019001862
original	glrlm	GrayLevelNonUniformityNormalized	0.040622653
original	glrlm	RunVariance	0.027041273
original	glrlm	GrayLevelNonUniformity	5.736963023
original	glrlm	LongRunEmphasis	1.081526237
original	glrlm	ShortRunHighGrayLevelEmphasis	398.0088154

original	glrlm	RunLengthNonUniformity	134.0813358
original	glrlm	ShortRunEmphasis	0.980216134
original	glrlm	LongRunHighGrayLevelEmphasis	453.5246993
original	glrlm	RunPercentage	0.974005305
original	glrlm	LongRunLowGrayLevelEmphasis	0.019435469
original	glrlm	RunEntropy	4.838692345
original	glrlm	HighGrayLevelRunEmphasis	408.9393784
original	glrlm	RunLengthNonUniformityNormalized	0.949122426
original	glszm	GrayLevelVariance	60.31800454
original	glszm	ZoneVariance	1.016780045
original	glszm	GrayLevelNonUniformityNormalized	0.041814059
original	glszm	SizeZoneNonUniformityNormalized	0.634829932
original	glszm	SizeZoneNonUniformity	66.65714286
original	glszm	GrayLevelNonUniformity	4.39047619
original	glszm	LargeAreaEmphasis	2.923809524
original	glszm	SmallAreaHighGrayLevelEmphasis	288.5687831
original	glszm	ZonePercentage	0.724137931
original	glszm	LargeAreaLowGrayLevelEmphasis	0.031776809
original	glszm	LargeAreaHighGrayLevelEmphasis	1487.752381
original	glszm	HighGrayLevelZoneEmphasis	369.7428571
original	glszm	SmallAreaEmphasis	0.824074735
original	glszm	LowGrayLevelZoneEmphasis	0.023532093
original	glszm	ZoneEntropy	5.273138495
original	glszm	SmallAreaLowGrayLevelEmphasis	0.022148234
original	ngtdm	Coarseness	0.032089326
original	ngtdm	Complexity	2220.785037
original	ngtdm	Strength	10.7995569
original	ngtdm	Contrast	0.801138444
original	ngtdm	Busyness	0.069058512
wavelet-HHH	gldm	GrayLevelVariance	16.01284185
wavelet-HHH	gldm	HighGrayLevelEmphasis	173.5586207
wavelet-HHH	gldm	DependenceEntropy	5.358728918
wavelet-HHH	gldm	DependenceNonUniformity	46.46206897
wavelet-HHH	gldm	GrayLevelNonUniformity	10.95862069
wavelet-HHH	gldm	SmallDependenceEmphasis	0.553436782
wavelet-HHH	gldm	SmallDependenceHighGrayLevelEmphasis	96.02611303
wavelet-HHH	gldm	DependenceNonUniformityNormalized	0.320428062
wavelet-HHH	gldm	LargeDependenceEmphasis	4.931034483
wavelet-HHH	gldm	LargeDependenceLowGrayLevelEmphasis	0.044074252
wavelet-HHH	gldm	DependenceVariance	1.175457788
wavelet-HHH	gldm	LargeDependenceHighGrayLevelEmphasis	903.3517241
wavelet-HHH	gldm	SmallDependenceLowGrayLevelEmphasis	0.012867244
wavelet-HHH	gldm	LowGrayLevelEmphasis	0.016367526
wavelet-HHH	glcm	JointAverage	12.62209082
wavelet-HHH	glcm	SumAverage	25.24418165
wavelet-HHH	glcm	JointEntropy	6.440122357
wavelet-HHH	glcm	ClusterShade	-14.91753664
wavelet-HHH	glcm	MaximumProbability	0.031778283
wavelet-HHH	glcm	Idmn	0.936323686
wavelet-HHH	glcm	JointEnergy	0.013246346
wavelet-HHH	glcm	Contrast	36.92881621
wavelet-HHH	glcm	DifferenceEntropy	3.498465746

wavelet-HHH	glcm	InverseVariance	0.173803405
wavelet-HHH	glcm	DifferenceVariance	12.28741772
wavelet-HHH	glcm	Idn	0.832788241
wavelet-HHH	glcm	Idm	0.186217225
wavelet-HHH	glcm	Correlation	-0.036896114
wavelet-HHH	glcm	Autocorrelation	158.6859846
wavelet-HHH	glcm	SumEntropy	4.221028855
wavelet-HHH	glcm	SumSquares	17.78810189
wavelet-HHH	glcm	ClusterProminence	3442.943156
wavelet-HHH	glcm	Imc2	0.967531175
wavelet-HHH	glcm	Imc1	-0.359123197
wavelet-HHH	glcm	DifferenceAverage	4.868273163
wavelet-HHH	glcm	Id	0.280844165
wavelet-HHH	glcm	ClusterTendency	34.22359136
wavelet-HHH	firstorder	InterquartileRange	140.2079432
wavelet-HHH	firstorder	Skewness	-0.175854657
wavelet-HHH	firstorder	Uniformity	0.075576694
wavelet-HHH	firstorder	Median	3.459345795
wavelet-HHH	firstorder	Energy	1434310.016
wavelet-HHH	firstorder	RobustMeanAbsoluteDeviation	59.19295245
wavelet-HHH	firstorder	MeanAbsoluteDeviation	81.67223495
wavelet-HHH	firstorder	TotalEnergy	38726370.43
wavelet-HHH	firstorder	Maximum	235.4673792
wavelet-HHH	firstorder	RootMeanSquared	99.45749451
wavelet-HHH	firstorder	90Percentile	129.7294817
wavelet-HHH	firstorder	Minimum	-278.1971252
wavelet-HHH	firstorder	Entropy	3.920905375
wavelet-HHH	firstorder	Range	513.6645044
wavelet-HHH	firstorder	Variance	9891.397871
wavelet-HHH	firstorder	10Percentile	-132.862918
wavelet-HHH	firstorder	Kurtosis	2.639157632
wavelet-HHH	firstorder	Mean	0.628763589
wavelet-HHH	glrlm	ShortRunLowGrayLevelEmphasis	0.016503974
wavelet-HHH	glrlm	GrayLevelVariance	16.21197017
wavelet-HHH	glrlm	LowGrayLevelRunEmphasis	0.016706115
wavelet-HHH	glrlm	GrayLevelNonUniformityNormalized	0.074872865
wavelet-HHH	glrlm	RunVariance	0.039329546
wavelet-HHH	glrlm	GrayLevelNonUniformity	10.46509043
wavelet-HHH	glrlm	LongRunEmphasis	1.116196961
wavelet-HHH	glrlm	ShortRunHighGrayLevelEmphasis	168.3939112
wavelet-HHH	glrlm	RunLengthNonUniformity	130.1646774
wavelet-HHH	glrlm	ShortRunEmphasis	0.97280028
wavelet-HHH	glrlm	LongRunHighGrayLevelEmphasis	194.3690196
wavelet-HHH	glrlm	RunPercentage	0.963925729
wavelet-HHH	glrlm	LongRunLowGrayLevelEmphasis	0.017556566
wavelet-HHH	glrlm	RunEntropy	4.092291053
wavelet-HHH	glrlm	HighGrayLevelRunEmphasis	173.2873237
wavelet-HHH	glrlm	RunLengthNonUniformityNormalized	0.930939604
wavelet-HHH	glszm	GrayLevelVariance	18.86530235
wavelet-HHH	glszm	ZoneVariance	1.343276679
wavelet-HHH	glszm	GrayLevelNonUniformityNormalized	0.067869118
wavelet-HHH	glszm	SizeZoneNonUniformityNormalized	0.546074691

wavelet-HHH	glszm	SizeZoneNonUniformity	50.78494624
wavelet-HHH	glszm	GrayLevelNonUniformity	6.311827957
wavelet-HHH	glszm	LargeAreaEmphasis	3.774193548
wavelet-HHH	glszm	SmallAreaHighGrayLevelEmphasis	133.7735474
wavelet-HHH	glszm	ZonePercentage	0.64137931
wavelet-HHH	glszm	LargeAreaLowGrayLevelEmphasis	0.041497135
wavelet-HHH	glszm	LargeAreaHighGrayLevelEmphasis	684.4301075
wavelet-HHH	glszm	HighGrayLevelZoneEmphasis	172.8387097
wavelet-HHH	glszm	SmallAreaEmphasis	0.766343903
wavelet-HHH	glszm	LowGrayLevelZoneEmphasis	0.021168996
wavelet-HHH	glszm	ZoneEntropy	4.989729172
wavelet-HHH	glszm	SmallAreaLowGrayLevelEmphasis	0.019250897
wavelet-HHH	ngtdm	Coarseness	0.031064392
wavelet-HHH	ngtdm	Complexity	552.5860114
wavelet-HHH	ngtdm	Strength	4.020447455
wavelet-HHH	ngtdm	Contrast	0.302326756
wavelet-HHH	ngtdm	Busyness	0.140155051
wavelet-HLL	gldm	GrayLevelVariance	96.44870392
wavelet-HLL	gldm	HighGrayLevelEmphasis	733.9241379
wavelet-HLL	gldm	DependenceEntropy	5.62880657
wavelet-HLL	gldm	DependenceNonUniformity	81.49655172
wavelet-HLL	gldm	GrayLevelNonUniformity	4.875862069
wavelet-HLL	gldm	SmallDependenceEmphasis	0.768582375
wavelet-HLL	gldm	SmallDependenceHighGrayLevelEmphasis	555.3766284
wavelet-HLL	gldm	DependenceNonUniformityNormalized	0.562045184
wavelet-HLL	gldm	LargeDependenceEmphasis	2.048275862
wavelet-HLL	gldm	LargeDependenceLowGrayLevelEmphasis	0.013841682
wavelet-HLL	gldm	DependenceVariance	0.276623068
wavelet-HLL	gldm	LargeDependenceHighGrayLevelEmphasis	1578.903448
wavelet-HLL	gldm	SmallDependenceLowGrayLevelEmphasis	0.011352376
wavelet-HLL	gldm	LowGrayLevelEmphasis	0.011820457
wavelet-HLL	glcm	JointAverage	24.77337991
wavelet-HLL	glcm	SumAverage	49.54675981
wavelet-HLL	glcm	JointEntropy	6.899461471
wavelet-HLL	glcm	ClusterShade	-22.72948354
wavelet-HLL	glcm	MaximumProbability	0.016614721
wavelet-HLL	glcm	Idmn	0.921826217
wavelet-HLL	glcm	JointEnergy	0.008827238
wavelet-HLL	glcm	Contrast	211.5128076
wavelet-HLL	glcm	DifferenceEntropy	4.355624027
wavelet-HLL	glcm	InverseVariance	0.101507794
wavelet-HLL	glcm	DifferenceVariance	72.46498738
wavelet-HLL	glcm	Idn	0.820436671
wavelet-HLL	glcm	Idm	0.088271302
wavelet-HLL	glcm	Correlation	-0.045479401
wavelet-HLL	glcm	Autocorrelation	607.7784362
wavelet-HLL	glcm	SumEntropy	5.033248737
wavelet-HLL	glcm	SumSquares	99.70508472
wavelet-HLL	glcm	ClusterProminence	109442.0367
wavelet-HLL	glcm	Imc2	0.998848785
wavelet-HLL	glcm	Imc1	-0.618732961
wavelet-HLL	glcm	DifferenceAverage	11.34443044

wavelet-HLL	glcm	Id	0.165783091
wavelet-HLL	glcm	ClusterTendency	187.3075313
wavelet-HLL	firstorder	InterquartileRange	321.6747492
wavelet-HLL	firstorder	Skewness	-0.16896623
wavelet-HLL	firstorder	Uniformity	0.033626635
wavelet-HLL	firstorder	Median	-70.71805401
wavelet-HLL	firstorder	Energy	9736399.248
wavelet-HLL	firstorder	RobustMeanAbsoluteDeviation	136.0154485
wavelet-HLL	firstorder	MeanAbsoluteDeviation	197.0353723
wavelet-HLL	firstorder	TotalEnergy	262882779.7
wavelet-HLL	firstorder	Maximum	436.7500587
wavelet-HLL	firstorder	RootMeanSquared	259.1285029
wavelet-HLL	firstorder	90Percentile	251.9479007
wavelet-HLL	firstorder	Minimum	-694.2592204
wavelet-HLL	firstorder	Entropy	5.068312436
wavelet-HLL	firstorder	Range	1131.009279
wavelet-HLL	firstorder	Variance	60513.90651
wavelet-HLL	firstorder	10Percentile	-423.4184031
wavelet-HLL	firstorder	Kurtosis	2.617212216
wavelet-HLL	firstorder	Mean	-81.44737264
wavelet-HLL	glrlm	ShortRunLowGrayLevelEmphasis	0.011929391
wavelet-HLL	glrlm	GrayLevelVariance	96.78589632
wavelet-HLL	glrlm	LowGrayLevelRunEmphasis	0.011948653
wavelet-HLL	glrlm	GrayLevelNonUniformityNormalized	0.033565376
wavelet-HLL	glrlm	RunVariance	0.012724032
wavelet-HLL	glrlm	GrayLevelNonUniformity	4.80495999
wavelet-HLL	glrlm	LongRunEmphasis	1.039053895
wavelet-HLL	glrlm	ShortRunHighGrayLevelEmphasis	725.4332577
wavelet-HLL	glrlm	RunLengthNonUniformity	139.5444406
wavelet-HLL	glrlm	ShortRunEmphasis	0.990236526
wavelet-HLL	glrlm	LongRunHighGrayLevelEmphasis	764.101284
wavelet-HLL	glrlm	RunPercentage	0.987267905
wavelet-HLL	glrlm	LongRunLowGrayLevelEmphasis	0.012025699
wavelet-HLL	glrlm	RunEntropy	5.111385339
wavelet-HLL	glrlm	HighGrayLevelRunEmphasis	733.166863
wavelet-HLL	glrlm	RunLengthNonUniformityNormalized	0.974551935
wavelet-HLL	glszm	GrayLevelVariance	101.6130046
wavelet-HLL	glszm	ZoneVariance	0.241650161
wavelet-HLL	glszm	GrayLevelNonUniformityNormalized	0.032716345
wavelet-HLL	glszm	SizeZoneNonUniformityNormalized	0.716822621
wavelet-HLL	glszm	SizeZoneNonUniformity	86.73553719
wavelet-HLL	glszm	GrayLevelNonUniformity	3.958677686
wavelet-HLL	glszm	LargeAreaEmphasis	1.67768595
wavelet-HLL	glszm	SmallAreaHighGrayLevelEmphasis	627.4167241
wavelet-HLL	glszm	ZonePercentage	0.834482759
wavelet-HLL	glszm	LargeAreaLowGrayLevelEmphasis	0.015036785
wavelet-HLL	glszm	LargeAreaHighGrayLevelEmphasis	1289.181818
wavelet-HLL	glszm	HighGrayLevelZoneEmphasis	722.4876033
wavelet-HLL	glszm	SmallAreaEmphasis	0.872187787
wavelet-HLL	glszm	LowGrayLevelZoneEmphasis	0.013774705
wavelet-HLL	glszm	ZoneEntropy	5.522913936
wavelet-HLL	glszm	SmallAreaLowGrayLevelEmphasis	0.013507001

wavelet-HLL	ngtdm	Coarseness	0.032233967
wavelet-HLL	ngtdm	Complexity	4984.649944
wavelet-HLL	ngtdm	Strength	17.66149047
wavelet-HLL	ngtdm	Contrast	0.971118936
wavelet-HLL	ngtdm	Busyness	0.036369196
wavelet-LHL	gldm	GrayLevelVariance	173.6349108
wavelet-LHL	gldm	HighGrayLevelEmphasis	1487.572414
wavelet-LHL	gldm	DependenceEntropy	5.946577452
wavelet-LHL	gldm	DependenceNonUniformity	72.90344828
wavelet-LHL	gldm	GrayLevelNonUniformity	4.117241379
wavelet-LHL	gldm	SmallDependenceEmphasis	0.732136015
wavelet-LHL	gldm	SmallDependenceHighGrayLevelEmphasis	1134.593439
wavelet-LHL	gldm	DependenceNonUniformityNormalized	0.502782402
wavelet-LHL	gldm	LargeDependenceEmphasis	2.64137931
wavelet-LHL	gldm	LargeDependenceLowGrayLevelEmphasis	0.010582155
wavelet-LHL	gldm	DependenceVariance	0.523852556
wavelet-LHL	gldm	LargeDependenceHighGrayLevelEmphasis	3388.924138
wavelet-LHL	gldm	SmallDependenceLowGrayLevelEmphasis	0.00813659
wavelet-LHL	gldm	LowGrayLevelEmphasis	0.008450251
wavelet-LHL	glcm	JointAverage	35.40344061
wavelet-LHL	glcm	SumAverage	70.80688122
wavelet-LHL	glcm	JointEntropy	6.910907029
wavelet-LHL	glcm	ClusterShade	4982.974521
wavelet-LHL	glcm	MaximumProbability	0.018868447
wavelet-LHL	glcm	Idmn	0.966948861
wavelet-LHL	glcm	JointEnergy	0.008831048
wavelet-LHL	glcm	Contrast	234.6265473
wavelet-LHL	glcm	DifferenceEntropy	4.390475742
wavelet-LHL	glcm	InverseVariance	0.08687137
wavelet-LHL	glcm	DifferenceVariance	88.1892837
wavelet-LHL	glcm	Idn	0.880625697
wavelet-LHL	glcm	Idm	0.089544175
wavelet-LHL	glcm	Correlation	0.231114876
wavelet-LHL	glcm	Autocorrelation	1288.802272
wavelet-LHL	glcm	SumEntropy	5.241860161
wavelet-LHL	glcm	SumSquares	152.251367
wavelet-LHL	glcm	ClusterProminence	543889.9911
wavelet-LHL	glcm	Imc2	0.999377795
wavelet-LHL	glcm	Imc1	-0.654731451
wavelet-LHL	glcm	DifferenceAverage	11.66454009
wavelet-LHL	glcm	Id	0.167251946
wavelet-LHL	glcm	ClusterTendency	374.3789207
wavelet-LHL	firstorder	InterquartileRange	387.8741365
wavelet-LHL	firstorder	Skewness	0.338543543
wavelet-LHL	firstorder	Uniformity	0.028394768
wavelet-LHL	firstorder	Median	49.01239093
wavelet-LHL	firstorder	Energy	15986914.42
wavelet-LHL	firstorder	RobustMeanAbsoluteDeviation	173.3659783
wavelet-LHL	firstorder	MeanAbsoluteDeviation	256.642492
wavelet-LHL	firstorder	TotalEnergy	431646689.2
wavelet-LHL	firstorder	Maximum	1100.022994
wavelet-LHL	firstorder	RootMeanSquared	332.0460543

wavelet-LHL	firstorder	90Percentile	439.8656887
wavelet-LHL	firstorder	Minimum	-837.6407153
wavelet-LHL	firstorder	Entropy	5.370605257
wavelet-LHL	firstorder	Range	1937.663709
wavelet-LHL	firstorder	Variance	108327.6816
wavelet-LHL	firstorder	10Percentile	-334.5729625
wavelet-LHL	firstorder	Kurtosis	3.512925562
wavelet-LHL	firstorder	Mean	43.89647518
wavelet-LHL	glrlm	ShortRunLowGrayLevelEmphasis	0.008566435
wavelet-LHL	glrlm	GrayLevelVariance	174.4733447
wavelet-LHL	glrlm	LowGrayLevelRunEmphasis	0.008582642
wavelet-LHL	glrlm	GrayLevelNonUniformityNormalized	0.028147962
wavelet-LHL	glrlm	RunVariance	0.018513026
wavelet-LHL	glrlm	GrayLevelNonUniformity	4.011087553
wavelet-LHL	glrlm	LongRunEmphasis	1.056101897
wavelet-LHL	glrlm	ShortRunHighGrayLevelEmphasis	1475.289879
wavelet-LHL	glrlm	RunLengthNonUniformity	137.7774958
wavelet-LHL	glrlm	ShortRunEmphasis	0.986572219
wavelet-LHL	glrlm	LongRunHighGrayLevelEmphasis	1560.717749
wavelet-LHL	glrlm	RunPercentage	0.982493369
wavelet-LHL	glrlm	LongRunLowGrayLevelEmphasis	0.008652893
wavelet-LHL	glrlm	RunEntropy	5.429617976
wavelet-LHL	glrlm	HighGrayLevelRunEmphasis	1492.164587
wavelet-LHL	glrlm	RunLengthNonUniformityNormalized	0.966178919
wavelet-LHL	glszm	GrayLevelVariance	185.1361474
wavelet-LHL	glszm	ZoneVariance	0.42887931
wavelet-LHL	glszm	GrayLevelNonUniformityNormalized	0.026010702
wavelet-LHL	glszm	SizeZoneNonUniformityNormalized	0.702288942
wavelet-LHL	glszm	SizeZoneNonUniformity	81.46551724
wavelet-LHL	glszm	GrayLevelNonUniformity	3.017241379
wavelet-LHL	glszm	LargeAreaEmphasis	1.99137931
wavelet-LHL	glszm	SmallAreaHighGrayLevelEmphasis	1349.575081
wavelet-LHL	glszm	ZonePercentage	0.8
wavelet-LHL	glszm	LargeAreaLowGrayLevelEmphasis	0.011547686
wavelet-LHL	glszm	LargeAreaHighGrayLevelEmphasis	2673.775862
wavelet-LHL	glszm	HighGrayLevelZoneEmphasis	1543.948276
wavelet-LHL	glszm	SmallAreaEmphasis	0.863251916
wavelet-LHL	glszm	LowGrayLevelZoneEmphasis	0.010261748
wavelet-LHL	glszm	ZoneEntropy	5.8358394
wavelet-LHL	glszm	SmallAreaLowGrayLevelEmphasis	0.010110421
wavelet-LHL	ngtdm	Coarseness	0.037252996
wavelet-LHL	ngtdm	Complexity	9091.868889
wavelet-LHL	ngtdm	Strength	41.51133657
wavelet-LHL	ngtdm	Contrast	1.120887879
wavelet-LHL	ngtdm	Busyness	0.018982036
wavelet-LHH	gldm	GrayLevelVariance	34.28156956
wavelet-LHH	gldm	HighGrayLevelEmphasis	473.8344828
wavelet-LHH	gldm	DependenceEntropy	5.296591705
wavelet-LHH	gldm	DependenceNonUniformity	54.1862069
wavelet-LHH	gldm	GrayLevelNonUniformity	8.682758621
wavelet-LHH	gldm	SmallDependenceEmphasis	0.586781609
wavelet-LHH	gldm	SmallDependenceHighGrayLevelEmphasis	289.9703065

wavelet-LHH	gldm	DependenceNonUniformityNormalized	0.373697979
wavelet-LHH	gldm	LargeDependenceEmphasis	3.606896552
wavelet-LHH	gldm	LargeDependenceLowGrayLevelEmphasis	0.017101408
wavelet-LHH	gldm	DependenceVariance	0.657978597
wavelet-LHH	gldm	LargeDependenceHighGrayLevelEmphasis	1639.793103
wavelet-LHH	gldm	SmallDependenceLowGrayLevelEmphasis	0.008646759
wavelet-LHH	gldm	LowGrayLevelEmphasis	0.009870771
wavelet-LHH	glcm	JointAverage	20.79399508
wavelet-LHH	glcm	SumAverage	41.58799015
wavelet-LHH	glcm	JointEntropy	6.685683261
wavelet-LHH	glcm	ClusterShade	-98.75442499
wavelet-LHH	glcm	MaximumProbability	0.023853653
wavelet-LHH	glcm	Idmn	0.969925747
wavelet-LHH	glcm	JointEnergy	0.010608429
wavelet-LHH	glcm	Contrast	74.96543939
wavelet-LHH	glcm	DifferenceEntropy	3.887503155
wavelet-LHH	glcm	InverseVariance	0.144594696
wavelet-LHH	glcm	DifferenceVariance	30.01371485
wavelet-LHH	glcm	Idn	0.884932761
wavelet-LHH	glcm	Idm	0.147885552
wavelet-LHH	glcm	Correlation	-0.031414662
wavelet-LHH	glcm	Autocorrelation	431.0533405
wavelet-LHH	glcm	SumEntropy	4.532903582
wavelet-LHH	glcm	SumSquares	36.06294763
wavelet-LHH	glcm	ClusterProminence	17182.84336
wavelet-LHH	glcm	Imc2	0.988544181
wavelet-LHH	glcm	Imc1	-0.448582313
wavelet-LHH	glcm	DifferenceAverage	6.621928524
wavelet-LHH	glcm	Id	0.238518277
wavelet-LHH	glcm	ClusterTendency	69.28635112
wavelet-LHH	firstorder	InterquartileRange	177.0315602
wavelet-LHH	firstorder	Skewness	0.158867481
wavelet-LHH	firstorder	Uniformity	0.059881094
wavelet-LHH	firstorder	Median	2.665958062
wavelet-LHH	firstorder	Energy	3150982.428
wavelet-LHH	firstorder	RobustMeanAbsoluteDeviation	71.26281244
wavelet-LHH	firstorder	MeanAbsoluteDeviation	110.176472
wavelet-LHH	firstorder	TotalEnergy	85076525.55
wavelet-LHH	firstorder	Maximum	659.6603001
wavelet-LHH	firstorder	RootMeanSquared	147.4140878
wavelet-LHH	firstorder	90Percentile	193.8764488
wavelet-LHH	firstorder	Minimum	-494.1974659
wavelet-LHH	firstorder	Entropy	4.305410707
wavelet-LHH	firstorder	Range	1153.857766
wavelet-LHH	firstorder	Variance	21602.89534
wavelet-LHH	firstorder	10Percentile	-141.2205059
wavelet-LHH	firstorder	Kurtosis	5.422326263
wavelet-LHH	firstorder	Mean	11.31450198
wavelet-LHH	glrlm	ShortRunLowGrayLevelEmphasis	0.010010913
wavelet-LHH	glrlm	GrayLevelVariance	34.72708083
wavelet-LHH	glrlm	LowGrayLevelRunEmphasis	0.010072157
wavelet-LHH	glrlm	GrayLevelNonUniformityNormalized	0.059252424

wavelet-LHH	glrlm	RunVariance	0.027533887
wavelet-LHH	glrlm	GrayLevelNonUniformity	8.355283054
wavelet-LHH	glrlm	LongRunEmphasis	1.086024834
wavelet-LHH	glrlm	ShortRunHighGrayLevelEmphasis	465.0240774
wavelet-LHH	glrlm	RunLengthNonUniformity	133.315734
wavelet-LHH	glrlm	ShortRunEmphasis	0.978493791
wavelet-LHH	glrlm	LongRunHighGrayLevelEmphasis	513.018898
wavelet-LHH	glrlm	RunPercentage	0.972413793
wavelet-LHH	glrlm	LongRunLowGrayLevelEmphasis	0.010317131
wavelet-LHH	glrlm	RunEntropy	4.434877102
wavelet-LHH	glrlm	HighGrayLevelRunEmphasis	474.6230415
wavelet-LHH	glrlm	RunLengthNonUniformityNormalized	0.944932226
wavelet-LHH	glszm	GrayLevelVariance	41.01765126
wavelet-LHH	glszm	ZoneVariance	0.915416794
wavelet-LHH	glszm	GrayLevelNonUniformityNormalized	0.053361902
wavelet-LHH	glszm	SizeZoneNonUniformityNormalized	0.546985002
wavelet-LHH	glszm	SizeZoneNonUniformity	54.15151515
wavelet-LHH	glszm	GrayLevelNonUniformity	5.282828283
wavelet-LHH	glszm	LargeAreaEmphasis	3.060606061
wavelet-LHH	glszm	SmallAreaHighGrayLevelEmphasis	382.1835908
wavelet-LHH	glszm	ZonePercentage	0.682758621
wavelet-LHH	glszm	LargeAreaLowGrayLevelEmphasis	0.018629838
wavelet-LHH	glszm	LargeAreaHighGrayLevelEmphasis	1429.636364
wavelet-LHH	glszm	HighGrayLevelZoneEmphasis	484.1414141
wavelet-LHH	glszm	SmallAreaEmphasis	0.765972136
wavelet-LHH	glszm	LowGrayLevelZoneEmphasis	0.013127295
wavelet-LHH	glszm	ZoneEntropy	5.147766793
wavelet-LHH	glszm	SmallAreaLowGrayLevelEmphasis	0.012389225
wavelet-LHH	ngtdm	Coarseness	0.035497117
wavelet-LHH	ngtdm	Complexity	1348.487124
wavelet-LHH	ngtdm	Strength	9.693055514
wavelet-LHH	ngtdm	Contrast	0.489702548
wavelet-LHH	ngtdm	Busyness	0.05927616
wavelet-LLH	gldm	GrayLevelVariance	84.98758621
wavelet-LLH	gldm	HighGrayLevelEmphasis	824.8275862
wavelet-LLH	gldm	DependenceEntropy	5.760683532
wavelet-LLH	gldm	DependenceNonUniformity	74.35172414
wavelet-LLH	gldm	GrayLevelNonUniformity	5.055172414
wavelet-LLH	gldm	SmallDependenceEmphasis	0.736015326
wavelet-LLH	gldm	SmallDependenceHighGrayLevelEmphasis	599.6302682
wavelet-LLH	gldm	DependenceNonUniformityNormalized	0.512770511
wavelet-LLH	gldm	LargeDependenceEmphasis	2.393103448
wavelet-LLH	gldm	LargeDependenceLowGrayLevelEmphasis	0.011743879
wavelet-LLH	gldm	DependenceVariance	0.394292509
wavelet-LLH	gldm	LargeDependenceHighGrayLevelEmphasis	1980.206897
wavelet-LLH	gldm	SmallDependenceLowGrayLevelEmphasis	0.009403453
wavelet-LLH	gldm	LowGrayLevelEmphasis	0.009775931
wavelet-LLH	glcm	JointAverage	27.03164581
wavelet-LLH	glcm	SumAverage	54.06329162
wavelet-LLH	glcm	JointEntropy	6.925769504
wavelet-LLH	glcm	ClusterShade	-216.3520541
wavelet-LLH	glcm	MaximumProbability	0.015979263

wavelet-LLH	glcm	Idmn	0.942771565
wavelet-LLH	glcm	JointEnergy	0.008674149
wavelet-LLH	glcm	Contrast	144.7148953
wavelet-LLH	glcm	DifferenceEntropy	4.176572163
wavelet-LLH	glcm	InverseVariance	0.126344037
wavelet-LLH	glcm	DifferenceVariance	58.96543744
wavelet-LLH	glcm	Idn	0.847408687
wavelet-LLH	glcm	Idm	0.110499719
wavelet-LLH	glcm	Correlation	0.151761789
wavelet-LLH	glcm	Autocorrelation	743.5408128
wavelet-LLH	glcm	SumEntropy	5.079187725
wavelet-LLH	glcm	SumSquares	85.1237514
wavelet-LLH	glcm	ClusterProminence	123048.6371
wavelet-LLH	glcm	Imc2	0.998600722
wavelet-LLH	glcm	Imc1	-0.604439485
wavelet-LLH	glcm	DifferenceAverage	9.081517823
wavelet-LLH	glcm	Id	0.194715514
wavelet-LLH	glcm	ClusterTendency	195.7801103
wavelet-LLH	firstorder	InterquartileRange	305.456777
wavelet-LLH	firstorder	Skewness	-0.316569281
wavelet-LLH	firstorder	Uniformity	0.034863258
wavelet-LLH	firstorder	Median	-38.20983734
wavelet-LLH	firstorder	Energy	7756215.983
wavelet-LLH	firstorder	RobustMeanAbsoluteDeviation	128.0892161
wavelet-LLH	firstorder	MeanAbsoluteDeviation	183.2182529
wavelet-LLH	firstorder	TotalEnergy	209417831.5
wavelet-LLH	firstorder	Maximum	408.0503535
wavelet-LLH	firstorder	RootMeanSquared	231.281527
wavelet-LLH	firstorder	90Percentile	277.1021457
wavelet-LLH	firstorder	Minimum	-676.4207981
wavelet-LLH	firstorder	Entropy	5.046250799
wavelet-LLH	firstorder	Range	1084.471152
wavelet-LLH	firstorder	Variance	52421.58641
wavelet-LLH	firstorder	10Percentile	-309.9824697
wavelet-LLH	firstorder	Kurtosis	2.889307606
wavelet-LLH	firstorder	Mean	-32.70410222
wavelet-LLH	glrlm	ShortRunLowGrayLevelEmphasis	0.009895176
wavelet-LLH	glrlm	GrayLevelVariance	85.73638686
wavelet-LLH	glrlm	LowGrayLevelRunEmphasis	0.00991249
wavelet-LLH	glrlm	GrayLevelNonUniformityNormalized	0.03463104
wavelet-LLH	glrlm	RunVariance	0.015901813
wavelet-LLH	glrlm	GrayLevelNonUniformity	4.941841906
wavelet-LLH	glrlm	LongRunEmphasis	1.048991268
wavelet-LLH	glrlm	ShortRunHighGrayLevelEmphasis	814.3599783
wavelet-LLH	glrlm	RunLengthNonUniformity	138.1973611
wavelet-LLH	glrlm	ShortRunEmphasis	0.987752183
wavelet-LLH	glrlm	LongRunHighGrayLevelEmphasis	865.6543878
wavelet-LLH	glrlm	RunPercentage	0.984084881
wavelet-LLH	glrlm	LongRunLowGrayLevelEmphasis	0.009981749
wavelet-LLH	glrlm	RunEntropy	5.108771323
wavelet-LLH	glrlm	HighGrayLevelRunEmphasis	824.6188602
wavelet-LLH	glrlm	RunLengthNonUniformityNormalized	0.968196374

wavelet-LLH	glszm	GrayLevelVariance	95.4503616
wavelet-LLH	glszm	ZoneVariance	0.318796114
wavelet-LLH	glszm	GrayLevelNonUniformityNormalized	0.031485134
wavelet-LLH	glszm	SizeZoneNonUniformityNormalized	0.691577179
wavelet-LLH	glszm	SizeZoneNonUniformity	80.91452991
wavelet-LLH	glszm	GrayLevelNonUniformity	3.683760684
wavelet-LLH	glszm	LargeAreaEmphasis	1.854700855
wavelet-LLH	glszm	SmallAreaHighGrayLevelEmphasis	697.4537037
wavelet-LLH	glszm	ZonePercentage	0.806896552
wavelet-LLH	glszm	LargeAreaLowGrayLevelEmphasis	0.013012138
wavelet-LLH	glszm	LargeAreaHighGrayLevelEmphasis	1517.350427
wavelet-LLH	glszm	HighGrayLevelZoneEmphasis	821.6837607
wavelet-LLH	glszm	SmallAreaEmphasis	0.857846629
wavelet-LLH	glszm	LowGrayLevelZoneEmphasis	0.011774036
wavelet-LLH	glszm	ZoneEntropy	5.654314027
wavelet-LLH	glszm	SmallAreaLowGrayLevelEmphasis	0.01157556
wavelet-LLH	ngtdm	Coarseness	0.037484051
wavelet-LLH	ngtdm	Complexity	4038.943467
wavelet-LLH	ngtdm	Strength	19.82610227
wavelet-LLH	ngtdm	Contrast	0.730413798
wavelet-LLH	ngtdm	Busyness	0.03040552
wavelet-LLL	gldm	GrayLevelVariance	242.6851843
wavelet-LLL	gldm	HighGrayLevelEmphasis	1181.57931
wavelet-LLL	gldm	DependenceEntropy	5.865981463
wavelet-LLL	gldm	DependenceNonUniformity	90.88965517
wavelet-LLL	gldm	GrayLevelNonUniformity	3.965517241
wavelet-LLL	gldm	SmallDependenceEmphasis	0.81901341
wavelet-LLL	gldm	SmallDependenceHighGrayLevelEmphasis	892.4569444
wavelet-LLL	gldm	DependenceNonUniformityNormalized	0.626825208
wavelet-LLL	gldm	LargeDependenceEmphasis	1.924137931
wavelet-LLL	gldm	LargeDependenceLowGrayLevelEmphasis	0.012243105
wavelet-LLL	gldm	DependenceVariance	0.296313912
wavelet-LLL	gldm	LargeDependenceHighGrayLevelEmphasis	2812.682759
wavelet-LLL	gldm	SmallDependenceLowGrayLevelEmphasis	0.010632601
wavelet-LLL	gldm	LowGrayLevelEmphasis	0.010935679
wavelet-LLL	glcm	JointAverage	34.23375701
wavelet-LLL	glcm	SumAverage	68.46751402
wavelet-LLL	glcm	JointEntropy	6.964888781
wavelet-LLL	glcm	ClusterShade	11496.94461
wavelet-LLL	glcm	MaximumProbability	0.015982539
wavelet-LLL	glcm	Idmn	0.951707268
wavelet-LLL	glcm	JointEnergy	0.008405233
wavelet-LLL	glcm	Contrast	356.1757388
wavelet-LLL	glcm	DifferenceEntropy	4.707806659
wavelet-LLL	glcm	InverseVariance	0.065473662
wavelet-LLL	glcm	DifferenceVariance	124.318144
wavelet-LLL	glcm	Idn	0.853410001
wavelet-LLL	glcm	Idm	0.064066325
wavelet-LLL	glcm	Correlation	0.3748889
wavelet-LLL	glcm	Autocorrelation	1277.48527
wavelet-LLL	glcm	SumEntropy	5.457055444
wavelet-LLL	glcm	SumSquares	280.9586189

wavelet-LLL	glcm	ClusterProminence	1722026.306
wavelet-LLL	glcm	Imc2	0.999632875
wavelet-LLL	glcm	Imc1	-0.690189908
wavelet-LLL	glcm	DifferenceAverage	15.00227588
wavelet-LLL	glcm	Id	0.133377853
wavelet-LLL	glcm	ClusterTendency	767.6587367
wavelet-LLL	firstorder	InterquartileRange	513.5187111
wavelet-LLL	firstorder	Skewness	0.565117713
wavelet-LLL	firstorder	Uniformity	0.027348395
wavelet-LLL	firstorder	Median	-1048.29685
wavelet-LLL	firstorder	Energy	173099562.8
wavelet-LLL	firstorder	RobustMeanAbsoluteDeviation	207.0829269
wavelet-LLL	firstorder	MeanAbsoluteDeviation	305.2067021
wavelet-LLL	firstorder	TotalEnergy	4673688195
wavelet-LLL	firstorder	Maximum	216.5948308
wavelet-LLL	firstorder	RootMeanSquared	1092.607014
wavelet-LLL	firstorder	90Percentile	-497.1082802
wavelet-LLL	firstorder	Minimum	-1760.872359
wavelet-LLL	firstorder	Entropy	5.458998878
wavelet-LLL	firstorder	Range	1977.46719
wavelet-LLL	firstorder	Variance	151921.2061
wavelet-LLL	firstorder	10Percentile	-1521.141496
wavelet-LLL	firstorder	Kurtosis	3.354252145
wavelet-LLL	firstorder	Mean	-1020.719786
wavelet-LLL	glrlm	ShortRunLowGrayLevelEmphasis	0.011024924
wavelet-LLL	glrlm	GrayLevelVariance	242.6137803
wavelet-LLL	glrlm	LowGrayLevelRunEmphasis	0.011037242
wavelet-LLL	glrlm	GrayLevelNonUniformityNormalized	0.027270254
wavelet-LLL	glrlm	RunVariance	0.010606547
wavelet-LLL	glrlm	GrayLevelNonUniformity	3.912290558
wavelet-LLL	glrlm	LongRunEmphasis	1.032367452
wavelet-LLL	glrlm	ShortRunHighGrayLevelEmphasis	1162.356725
wavelet-LLL	glrlm	RunLengthNonUniformity	140.436546
wavelet-LLL	glrlm	ShortRunEmphasis	0.991908137
wavelet-LLL	glrlm	LongRunHighGrayLevelEmphasis	1230.86563
wavelet-LLL	glrlm	RunPercentage	0.98938992
wavelet-LLL	glrlm	LongRunLowGrayLevelEmphasis	0.011086513
wavelet-LLL	glrlm	RunEntropy	5.492490612
wavelet-LLL	glrlm	HighGrayLevelRunEmphasis	1176.058506
wavelet-LLL	glrlm	RunLengthNonUniformityNormalized	0.978786906
wavelet-LLL	glszm	GrayLevelVariance	242.9324767
wavelet-LLL	glszm	ZoneVariance	0.207420005
wavelet-LLL	glszm	GrayLevelNonUniformityNormalized	0.026581003
wavelet-LLL	glszm	SizeZoneNonUniformityNormalized	0.785462333
wavelet-LLL	glszm	SizeZoneNonUniformity	98.96825397
wavelet-LLL	glszm	GrayLevelNonUniformity	3.349206349
wavelet-LLL	glszm	LargeAreaEmphasis	1.531746032
wavelet-LLL	glszm	SmallAreaHighGrayLevelEmphasis	971.7006724
wavelet-LLL	glszm	ZonePercentage	0.868965517
wavelet-LLL	glszm	LargeAreaLowGrayLevelEmphasis	0.013103887
wavelet-LLL	glszm	LargeAreaHighGrayLevelEmphasis	2029.468254
wavelet-LLL	glszm	HighGrayLevelZoneEmphasis	1113.650794

wavelet-LLL	glszm	SmallAreaEmphasis	0.907021605
wavelet-LLL	glszm	LowGrayLevelZoneEmphasis	0.012345423
wavelet-LLL	glszm	ZoneEntropy	5.750934639
wavelet-LLL	glszm	SmallAreaLowGrayLevelEmphasis	0.012176893
wavelet-LLL	ngtdm	Coarseness	0.027873071
wavelet-LLL	ngtdm	Complexity	14022.15921
wavelet-LLL	ngtdm	Strength	47.80915168
wavelet-LLL	ngtdm	Contrast	1.640815817
wavelet-LLL	ngtdm	Busyness	0.024080032
wavelet-HHL	gldm	GrayLevelVariance	45.16166468
wavelet-HHL	gldm	HighGrayLevelEmphasis	442.6827586
wavelet-HHL	gldm	DependenceEntropy	5.484999611
wavelet-HHL	gldm	DependenceNonUniformity	63.20689655
wavelet-HHL	gldm	GrayLevelNonUniformity	7.220689655
wavelet-HHL	gldm	SmallDependenceEmphasis	0.662643678
wavelet-HHL	gldm	SmallDependenceHighGrayLevelEmphasis	284.4114943
wavelet-HHL	gldm	DependenceNonUniformityNormalized	0.435909631
wavelet-HHL	gldm	LargeDependenceEmphasis	3.027586207
wavelet-HHL	gldm	LargeDependenceLowGrayLevelEmphasis	0.022475402
wavelet-HHL	gldm	DependenceVariance	0.576741974
wavelet-HHL	gldm	LargeDependenceHighGrayLevelEmphasis	1354.434483
wavelet-HHL	gldm	SmallDependenceLowGrayLevelEmphasis	0.016567241
wavelet-HHL	gldm	LowGrayLevelEmphasis	0.017399376
wavelet-HHL	glcm	JointAverage	20.1458765
wavelet-HHL	glcm	SumAverage	40.29175301
wavelet-HHL	glcm	JointEntropy	6.696964799
wavelet-HHL	glcm	ClusterShade	240.7429087
wavelet-HHL	glcm	MaximumProbability	0.026416009
wavelet-HHL	glcm	Idmn	0.96180427
wavelet-HHL	glcm	JointEnergy	0.010736147
wavelet-HHL	glcm	Contrast	74.53989803
wavelet-HHL	glcm	DifferenceEntropy	3.825749484
wavelet-HHL	glcm	InverseVariance	0.15457295
wavelet-HHL	glcm	DifferenceVariance	28.58501621
wavelet-HHL	glcm	Idn	0.87164744
wavelet-HHL	glcm	Idm	0.141356971
wavelet-HHL	glcm	Correlation	0.040445316
wavelet-HHL	glcm	Autocorrelation	408.4534248
wavelet-HHL	glcm	SumEntropy	4.600951488
wavelet-HHL	glcm	SumSquares	39.43049265
wavelet-HHL	glcm	ClusterProminence	32341.95027
wavelet-HHL	glcm	Imc2	0.99230534
wavelet-HHL	glcm	Imc1	-0.480160887
wavelet-HHL	glcm	DifferenceAverage	6.602773871
wavelet-HHL	glcm	Id	0.232878619
wavelet-HHL	glcm	ClusterTendency	83.18207255
wavelet-HHL	firstorder	InterquartileRange	195.3359022
wavelet-HHL	firstorder	Skewness	0.123010332
wavelet-HHL	firstorder	Uniformity	0.04979786
wavelet-HHL	firstorder	Median	-16.05199026
wavelet-HHL	firstorder	Energy	4100119.982
wavelet-HHL	firstorder	RobustMeanAbsoluteDeviation	82.02090235

wavelet-HHL	firstorder	MeanAbsoluteDeviation	127.5005559
wavelet-HHL	firstorder	TotalEnergy	110703239.5
wavelet-HHL	firstorder	Maximum	509.3378098
wavelet-HHL	firstorder	RootMeanSquared	168.156741
wavelet-HHL	firstorder	90Percentile	193.3638753
wavelet-HHL	firstorder	Minimum	-495.41691
wavelet-HHL	firstorder	Entropy	4.588774383
wavelet-HHL	firstorder	Range	1004.75472
wavelet-HHL	firstorder	Variance	28089.25597
wavelet-HHL	firstorder	10Percentile	-207.8862975
wavelet-HHL	firstorder	Kurtosis	3.751702425
wavelet-HHL	firstorder	Mean	-13.69063774
wavelet-HHL	glrlm	ShortRunLowGrayLevelEmphasis	0.017692922
wavelet-HHL	glrlm	GrayLevelVariance	45.78186312
wavelet-HHL	glrlm	LowGrayLevelRunEmphasis	0.017733277
wavelet-HHL	glrlm	GrayLevelNonUniformityNormalized	0.049198481
wavelet-HHL	glrlm	RunVariance	0.022841901
wavelet-HHL	glrlm	GrayLevelNonUniformity	6.979535269
wavelet-HHL	glrlm	LongRunEmphasis	1.068311691
wavelet-HHL	glrlm	ShortRunHighGrayLevelEmphasis	434.7806341
wavelet-HHL	glrlm	RunLengthNonUniformity	135.8602454
wavelet-HHL	glrlm	ShortRunEmphasis	0.983541426
wavelet-HHL	glrlm	LongRunHighGrayLevelEmphasis	473.2999687
wavelet-HHL	glrlm	RunPercentage	0.978249337
wavelet-HHL	glrlm	LongRunLowGrayLevelEmphasis	0.01790571
wavelet-HHL	glrlm	RunEntropy	4.685961612
wavelet-HHL	glrlm	HighGrayLevelRunEmphasis	442.3730183
wavelet-HHL	glrlm	RunLengthNonUniformityNormalized	0.957515267
wavelet-HHL	glszm	GrayLevelVariance	54.92844429
wavelet-HHL	glszm	ZoneVariance	1.062744749
wavelet-HHL	glszm	GrayLevelNonUniformityNormalized	0.042719829
wavelet-HHL	glszm	SizeZoneNonUniformityNormalized	0.64257743
wavelet-HHL	glszm	SizeZoneNonUniformity	68.11320755
wavelet-HHL	glszm	GrayLevelNonUniformity	4.528301887
wavelet-HHL	glszm	LargeAreaEmphasis	2.933962264
wavelet-HHL	glszm	SmallAreaHighGrayLevelEmphasis	352.4608495
wavelet-HHL	glszm	ZonePercentage	0.731034483
wavelet-HHL	glszm	LargeAreaLowGrayLevelEmphasis	0.027777726
wavelet-HHL	glszm	LargeAreaHighGrayLevelEmphasis	1281.915094
wavelet-HHL	glszm	HighGrayLevelZoneEmphasis	436.2830189
wavelet-HHL	glszm	SmallAreaEmphasis	0.828285428
wavelet-HHL	glszm	LowGrayLevelZoneEmphasis	0.022886844
wavelet-HHL	glszm	ZoneEntropy	5.317569399
wavelet-HHL	glszm	SmallAreaLowGrayLevelEmphasis	0.022469101
wavelet-HHL	ngtdm	Coarseness	0.036575433
wavelet-HHL	ngtdm	Complexity	2100.63649
wavelet-HHL	ngtdm	Strength	12.47572605
wavelet-HHL	ngtdm	Contrast	0.474466312
wavelet-HHL	ngtdm	Busyness	0.048713593
wavelet-HLH	gldm	GrayLevelVariance	33.14311534
wavelet-HLH	gldm	HighGrayLevelEmphasis	275.2137931
wavelet-HLH	gldm	DependenceEntropy	5.373518252

wavelet-HLH	gldm	DependenceNonUniformity	57.02758621
wavelet-HLH	gldm	GrayLevelNonUniformity	8.131034483
wavelet-HLH	gldm	SmallDependenceEmphasis	0.633273946
wavelet-HLH	gldm	SmallDependenceHighGrayLevelEmphasis	181.8031341
wavelet-HLH	gldm	DependenceNonUniformityNormalized	0.393293698
wavelet-HLH	gldm	LargeDependenceEmphasis	3.882758621
wavelet-HLH	gldm	LargeDependenceLowGrayLevelEmphasis	0.035748539
wavelet-HLH	gldm	DependenceVariance	0.88627824
wavelet-HLH	gldm	LargeDependenceHighGrayLevelEmphasis	987.6551724
wavelet-HLH	gldm	SmallDependenceLowGrayLevelEmphasis	0.018178213
wavelet-HLH	gldm	LowGrayLevelEmphasis	0.02024569
wavelet-HLH	glcm	JointAverage	15.26113906
wavelet-HLH	glcm	SumAverage	30.52227812
wavelet-HLH	glcm	JointEntropy	6.703834211
wavelet-HLH	glcm	ClusterShade	66.4537865
wavelet-HLH	glcm	MaximumProbability	0.025984901
wavelet-HLH	glcm	Idmn	0.946907266
wavelet-HLH	glcm	JointEnergy	0.010574666
wavelet-HLH	glcm	Contrast	67.95156666
wavelet-HLH	glcm	DifferenceEntropy	3.859210212
wavelet-HLH	glcm	InverseVariance	0.147136001
wavelet-HLH	glcm	DifferenceVariance	24.42679091
wavelet-HLH	glcm	Idn	0.847789639
wavelet-HLH	glcm	Idm	0.149867234
wavelet-HLH	glcm	Correlation	-0.001007833
wavelet-HLH	glcm	Autocorrelation	233.3977095
wavelet-HLH	glcm	SumEntropy	4.591446599
wavelet-HLH	glcm	SumSquares	33.98339686
wavelet-HLH	glcm	ClusterProminence	13741.6015
wavelet-HLH	glcm	Imc2	0.988043708
wavelet-HLH	glcm	Imc1	-0.442585951
wavelet-HLH	glcm	DifferenceAverage	6.511186885
wavelet-HLH	glcm	Id	0.239313005
wavelet-HLH	glcm	ClusterTendency	67.98202077
wavelet-HLH	firstorder	InterquartileRange	185.3480772
wavelet-HLH	firstorder	Skewness	0.16720247
wavelet-HLH	firstorder	Uniformity	0.0560761
wavelet-HLH	firstorder	Median	-15.88689199
wavelet-HLH	firstorder	Energy	2951491.661
wavelet-HLH	firstorder	RobustMeanAbsoluteDeviation	81.98149766
wavelet-HLH	firstorder	MeanAbsoluteDeviation	115.4062531
wavelet-HLH	firstorder	TotalEnergy	79690274.84
wavelet-HLH	firstorder	Maximum	432.2376218
wavelet-HLH	firstorder	RootMeanSquared	142.6713528
wavelet-HLH	firstorder	90Percentile	189.5907699
wavelet-HLH	firstorder	Minimum	-370.1938885
wavelet-HLH	firstorder	Entropy	4.378297139
wavelet-HLH	firstorder	Range	802.4315103
wavelet-HLH	firstorder	Variance	20351.21291
wavelet-HLH	firstorder	10Percentile	-163.1766986
wavelet-HLH	firstorder	Kurtosis	2.914282594
wavelet-HLH	firstorder	Mean	1.975344871

wavelet-HLH	glrlm	ShortRunLowGrayLevelEmphasis	0.020565205
wavelet-HLH	glrlm	GrayLevelVariance	33.57880327
wavelet-HLH	glrlm	LowGrayLevelRunEmphasis	0.02067942
wavelet-HLH	glrlm	GrayLevelNonUniformityNormalized	0.055374408
wavelet-HLH	glrlm	RunVariance	0.030297321
wavelet-HLH	glrlm	GrayLevelNonUniformity	7.803928541
wavelet-HLH	glrlm	LongRunEmphasis	1.090012675
wavelet-HLH	glrlm	ShortRunHighGrayLevelEmphasis	270.4802159
wavelet-HLH	glrlm	RunLengthNonUniformity	133.3639576
wavelet-HLH	glrlm	ShortRunEmphasis	0.978740444
wavelet-HLH	glrlm	LongRunHighGrayLevelEmphasis	298.3132639
wavelet-HLH	glrlm	RunPercentage	0.971883289
wavelet-HLH	glrlm	LongRunLowGrayLevelEmphasis	0.021170823
wavelet-HLH	glrlm	RunEntropy	4.506730488
wavelet-HLH	glrlm	HighGrayLevelRunEmphasis	275.9035614
wavelet-HLH	glrlm	RunLengthNonUniformityNormalized	0.945798754
wavelet-HLH	glszm	GrayLevelVariance	38.51239514
wavelet-HLH	glszm	ZoneVariance	1.057027053
wavelet-HLH	glszm	GrayLevelNonUniformityNormalized	0.048920728
wavelet-HLH	glszm	SizeZoneNonUniformityNormalized	0.636157979
wavelet-HLH	glszm	SizeZoneNonUniformity	65.52427184
wavelet-HLH	glszm	GrayLevelNonUniformity	5.038834951
wavelet-HLH	glszm	LargeAreaEmphasis	3.038834951
wavelet-HLH	glszm	SmallAreaHighGrayLevelEmphasis	238.5275755
wavelet-HLH	glszm	ZonePercentage	0.710344828
wavelet-HLH	glszm	LargeAreaLowGrayLevelEmphasis	0.037461066
wavelet-HLH	glszm	LargeAreaHighGrayLevelEmphasis	791.3980583
wavelet-HLH	glszm	HighGrayLevelZoneEmphasis	284.0582524
wavelet-HLH	glszm	SmallAreaEmphasis	0.822764968
wavelet-HLH	glszm	LowGrayLevelZoneEmphasis	0.026226019
wavelet-HLH	glszm	ZoneEntropy	5.114776533
wavelet-HLH	glszm	SmallAreaLowGrayLevelEmphasis	0.025180655
wavelet-HLH	ngtdm	Coarseness	0.033192344
wavelet-HLH	ngtdm	Complexity	1313.431243
wavelet-HLH	ngtdm	Strength	8.011540058
wavelet-HLH	ngtdm	Contrast	0.445721516
wavelet-HLH	ngtdm	Busyness	0.084099746

Image type	Feature Class	Feature Name	16: RTSTRUCT: C99-DNU_segment_RUL ITV
general	info	BoundingBox	(0, 1, 1, 13, 12, 8)
general	info	EnabledImageTypes	{u'Original': {}, u'Wavelet': {}}
general	info	GeneralSettings	{'distances': [1], 'additionalInfo': True, 'force2D': False, 'interpolator': 'sitkBSpline', 'resampledPixelSpacing': [3.0], 'label': 1, 'normalizeScale': 1, 'normalize': False, 'force2Ddimension': 0, 'removeOutliers': None, 'minimumROISize': None, u'binWidth': 25.0, 'minimumROIDimensions': 1, u'correctMask': True, 'preCrop': False, u'symmetricalGLCM': True, 'resegmentRange': None, 'padDistance': 5}
general	info	ImageHash	fd3d409fa12697949ef9a04d708a8555202436da
general	info	ImageSpacing	(0.9765625000000002, 0.9765625000000002, 2.0)
general	info	MaskHash	6a784867dbfd5c52e4d2181b82e0b113c27c893a
general	info	NumpyVersion	1.13.1
general	info	PyWaveletVersion	0.5.2
general	info	SimpleITKVersion	1.1.0
general	info	Version	1.3.0.post102+g1b0eb74
general	info	VolumeNum	1
general	info	VoxelNum	550
original	shape	Maximum3DDiameter	37.58989226
original	shape	Maximum2DDiameterSlice	35.11409973
original	shape	Sphericity	0.857953351
original	shape	MinorAxis	29.98654485
original	shape	Elongation	0.986368653
original	shape	SurfaceVolumeRatio	0.229321601
original	shape	Volume	14850
original	shape	MajorAxis	30.40095075
original	shape	SurfaceArea	3405.425774
original	shape	Flatness	0.760512986
original	shape	LeastAxis	23.12031783
original	shape	Maximum2DDiameterColumn	34.20526275
original	shape	Maximum2DDiameterRow	33.54101966
original	gldm	GrayLevelVariance	59.96653223
original	gldm	HighGrayLevelEmphasis	1157.141818
original	gldm	DependenceEntropy	6.121182914
original	gldm	DependenceNonUniformity	75.85090909
original	gldm	GrayLevelNonUniformity	79.20363636
original	gldm	SmallDependenceEmphasis	0.360895956
original	gldm	SmallDependenceHighGrayLevelEmphasis	284.989895
original	gldm	DependenceNonUniformityNormalized	0.137910744
original	gldm	LargeDependenceEmphasis	54.97454545
original	gldm	LargeDependenceLowGrayLevelEmphasis	0.041696145
original	gldm	DependenceVariance	23.85843306
original	gldm	LargeDependenceHighGrayLevelEmphasis	78266.25818
original	gldm	SmallDependenceLowGrayLevelEmphasis	0.00314918
original	gldm	LowGrayLevelEmphasis	0.003662095
original	glcm	JointAverage	34.67595149
original	glcm	SumAverage	69.35190298

original	glcm	JointEntropy	6.016367816
original	glcm	ClusterShade	-2015.873591
original	glcm	MaximumProbability	0.158518603
original	glcm	Idmn	0.968251151
original	glcm	JointEnergy	0.050402386
original	glcm	Contrast	62.50186233
original	glcm	DifferenceEntropy	3.532230656
original	glcm	InverseVariance	0.320592095
original	glcm	DifferenceVariance	40.96528054
original	glcm	Idn	0.911373708
original	glcm	Idm	0.398613867
original	glcm	Correlation	0.240590128
original	glcm	Autocorrelation	1212.319857
original	glcm	SumEntropy	4.241858738
original	glcm	SumSquares	41.13047053
original	glcm	ClusterProminence	72429.79814
original	glcm	Imc2	0.829647747
original	glcm	Imc1	-0.178465287
original	glcm	DifferenceAverage	4.630005116
original	glcm	Id	0.447583476
original	glcm	ClusterTendency	102.0200198
original	firstorder	InterquartileRange	179
original	firstorder	Skewness	-1.737800022
original	firstorder	Uniformity	0.144006612
original	firstorder	Median	14.5
original	firstorder	Energy	24547079
original	firstorder	RobustMeanAbsoluteDeviation	92.07481405
original	firstorder	MeanAbsoluteDeviation	148.7771835
original	firstorder	TotalEnergy	662771133
original	firstorder	Maximum	96
original	firstorder	RootMeanSquared	211.2606275
original	firstorder	90Percentile	49.1
original	firstorder	Minimum	-880
original	firstorder	Entropy	3.777124094
original	firstorder	Range	976
original	firstorder	Variance	37349.99897
original	firstorder	10Percentile	-373.9
original	firstorder	Kurtosis	5.342134308
original	firstorder	Mean	-85.32909091
original	glrlm	ShortRunLowGrayLevelEmphasis	0.004208462
original	glrlm	GrayLevelVariance	66.93384043
original	glrlm	LowGrayLevelRunEmphasis	0.004293235
original	glrlm	GrayLevelNonUniformityNormalized	0.101218213
original	glrlm	RunVariance	0.368388019
original	glrlm	GrayLevelNonUniformity	45.92174215
original	glrlm	LongRunEmphasis	1.844904604
original	glrlm	ShortRunHighGrayLevelEmphasis	938.1275973
original	glrlm	RunLengthNonUniformity	336.7204206
original	glrlm	ShortRunEmphasis	0.884039151
original	glrlm	LongRunHighGrayLevelEmphasis	2301.817685
original	glrlm	RunPercentage	0.823916084
original	glrlm	LongRunLowGrayLevelEmphasis	0.004898115
original	glrlm	RunEntropy	4.701129228
original	glrlm	HighGrayLevelRunEmphasis	1101.550809
original	glrlm	RunLengthNonUniformityNormalized	0.742554805

original	glszm	GrayLevelVariance	77.05725624
original	glszm	ZoneVariance	176.8453515
original	glszm	GrayLevelNonUniformityNormalized	0.040907029
original	glszm	SizeZoneNonUniformityNormalized	0.684988662
original	glszm	SizeZoneNonUniformity	143.847619
original	glszm	GrayLevelNonUniformity	8.59047619
original	glszm	LargeAreaEmphasis	183.7047619
original	glszm	SmallAreaHighGrayLevelEmphasis	631.6627436
original	glszm	ZonePercentage	0.381818182
original	glszm	LargeAreaLowGrayLevelEmphasis	0.136738258
original	glszm	LargeAreaHighGrayLevelEmphasis	261567.3143
original	glszm	HighGrayLevelZoneEmphasis	799.6428571
original	glszm	SmallAreaEmphasis	0.851701954
original	glszm	LowGrayLevelZoneEmphasis	0.008326262
original	glszm	ZoneEntropy	5.464540277
original	glszm	SmallAreaLowGrayLevelEmphasis	0.008150936
original	ngtdm	Coarseness	0.005213922
original	ngtdm	Complexity	2824.608655
original	ngtdm	Strength	9.622847096
original	ngtdm	Contrast	0.387084003
original	ngtdm	Busyness	0.102490797
wavelet-HHH	gldm	GrayLevelVariance	10.85567273
wavelet-HHH	gldm	HighGrayLevelEmphasis	222.2672727
wavelet-HHH	gldm	DependenceEntropy	6.12925928
wavelet-HHH	gldm	DependenceNonUniformity	75.42181818
wavelet-HHH	gldm	GrayLevelNonUniformity	62.79272727
wavelet-HHH	gldm	SmallDependenceEmphasis	0.302581327
wavelet-HHH	gldm	SmallDependenceHighGrayLevelEmphasis	70.54104811
wavelet-HHH	gldm	DependenceNonUniformityNormalized	0.137130579
wavelet-HHH	gldm	LargeDependenceEmphasis	32.14909091
wavelet-HHH	gldm	LargeDependenceLowGrayLevelEmphasis	0.161432756
wavelet-HHH	gldm	DependenceVariance	13.48669091
wavelet-HHH	gldm	LargeDependenceHighGrayLevelEmphasis	6694.634545
wavelet-HHH	gldm	SmallDependenceLowGrayLevelEmphasis	0.004090416
wavelet-HHH	gldm	LowGrayLevelEmphasis	0.007787708
wavelet-HHH	glcm	JointAverage	14.50833414
wavelet-HHH	glcm	SumAverage	29.01666829
wavelet-HHH	glcm	JointEntropy	6.597742237
wavelet-HHH	glcm	ClusterShade	-2.662307562
wavelet-HHH	glcm	MaximumProbability	0.078417646
wavelet-HHH	glcm	Idmn	0.974961033
wavelet-HHH	glcm	JointEnergy	0.023122878
wavelet-HHH	glcm	Contrast	20.64225335
wavelet-HHH	glcm	DifferenceEntropy	3.221423577
wavelet-HHH	glcm	InverseVariance	0.309740208
wavelet-HHH	glcm	DifferenceVariance	10.49181858
wavelet-HHH	glcm	Idn	0.904532011
wavelet-HHH	glcm	Idm	0.340425381
wavelet-HHH	glcm	Correlation	-0.034308339
wavelet-HHH	glcm	Autocorrelation	210.1406685
wavelet-HHH	glcm	SumEntropy	3.974428951
wavelet-HHH	glcm	SumSquares	9.96702133
wavelet-HHH	glcm	ClusterProminence	1920.477381
wavelet-HHH	glcm	Imc2	0.767557795
wavelet-HHH	glcm	Imc1	-0.127247304

wavelet-HHH	glcm	DifferenceAverage	3.156432948
wavelet-HHH	glcm	Id	0.412659953
wavelet-HHH	glcm	ClusterTendency	19.22583198
wavelet-HHH	firstorder	InterquartileRange	74.44516151
wavelet-HHH	firstorder	Skewness	-0.137033356
wavelet-HHH	firstorder	Uniformity	0.114168595
wavelet-HHH	firstorder	Median	1.057296503
wavelet-HHH	firstorder	Energy	3722941.218
wavelet-HHH	firstorder	RobustMeanAbsoluteDeviation	33.6088838
wavelet-HHH	firstorder	MeanAbsoluteDeviation	57.84768519
wavelet-HHH	firstorder	TotalEnergy	100519412.9
wavelet-HHH	firstorder	Maximum	301.9543249
wavelet-HHH	firstorder	RootMeanSquared	82.27383565
wavelet-HHH	firstorder	90Percentile	103.9889499
wavelet-HHH	firstorder	Minimum	-348.2743503
wavelet-HHH	firstorder	Entropy	3.624556983
wavelet-HHH	firstorder	Range	650.2286752
wavelet-HHH	firstorder	Variance	6767.587915
wavelet-HHH	firstorder	10Percentile	-96.0239452
wavelet-HHH	firstorder	Kurtosis	4.843279613
wavelet-HHH	firstorder	Mean	1.181574308
wavelet-HHH	glrlm	ShortRunLowGrayLevelEmphasis	0.007738161
wavelet-HHH	glrlm	GrayLevelVariance	12.11697627
wavelet-HHH	glrlm	LowGrayLevelRunEmphasis	0.008196063
wavelet-HHH	glrlm	GrayLevelNonUniformityNormalized	0.098980913
wavelet-HHH	glrlm	RunVariance	0.1987421
wavelet-HHH	glrlm	GrayLevelNonUniformity	47.50907656
wavelet-HHH	glrlm	LongRunEmphasis	1.515413706
wavelet-HHH	glrlm	ShortRunHighGrayLevelEmphasis	204.623417
wavelet-HHH	glrlm	RunLengthNonUniformity	379.5701135
wavelet-HHH	glrlm	ShortRunEmphasis	0.909062776
wavelet-HHH	glrlm	LongRunHighGrayLevelEmphasis	332.3276005
wavelet-HHH	glrlm	RunPercentage	0.872307692
wavelet-HHH	glrlm	LongRunLowGrayLevelEmphasis	0.010771048
wavelet-HHH	glrlm	RunEntropy	4.266236999
wavelet-HHH	glrlm	HighGrayLevelRunEmphasis	223.912829
wavelet-HHH	glrlm	RunLengthNonUniformityNormalized	0.790399945
wavelet-HHH	glszm	GrayLevelVariance	22.89760817
wavelet-HHH	glszm	ZoneVariance	110.2458718
wavelet-HHH	glszm	GrayLevelNonUniformityNormalized	0.062139807
wavelet-HHH	glszm	SizeZoneNonUniformityNormalized	0.476425095
wavelet-HHH	glszm	SizeZoneNonUniformity	87.18579235
wavelet-HHH	glszm	GrayLevelNonUniformity	11.3715847
wavelet-HHH	glszm	LargeAreaEmphasis	119.2786885
wavelet-HHH	glszm	SmallAreaHighGrayLevelEmphasis	168.9614184
wavelet-HHH	glszm	ZonePercentage	0.332727273
wavelet-HHH	glszm	LargeAreaLowGrayLevelEmphasis	0.585104568
wavelet-HHH	glszm	LargeAreaHighGrayLevelEmphasis	25081.84153
wavelet-HHH	glszm	HighGrayLevelZoneEmphasis	235.7704918
wavelet-HHH	glszm	SmallAreaEmphasis	0.716942071
wavelet-HHH	glszm	LowGrayLevelZoneEmphasis	0.012900371
wavelet-HHH	glszm	ZoneEntropy	5.407756491
wavelet-HHH	glszm	SmallAreaLowGrayLevelEmphasis	0.011176829
wavelet-HHH	ngtdm	Coarseness	0.013786286
wavelet-HHH	ngtdm	Complexity	658.7191539

wavelet-HHH	ngtdm	Strength	2.620709521
wavelet-HHH	ngtdm	Contrast	0.082074502
wavelet-HHH	ngtdm	Busyness	0.14657369
wavelet-HLL	gldm	GrayLevelVariance	140.7715702
wavelet-HLL	gldm	HighGrayLevelEmphasis	793.3418182
wavelet-HLL	gldm	DependenceEntropy	6.803535972
wavelet-HLL	gldm	DependenceNonUniformity	150.64
wavelet-HLL	gldm	GrayLevelNonUniformity	20.18909091
wavelet-HLL	gldm	SmallDependenceEmphasis	0.513303474
wavelet-HLL	gldm	SmallDependenceHighGrayLevelEmphasis	500.3889302
wavelet-HLL	gldm	DependenceNonUniformityNormalized	0.273890909
wavelet-HLL	gldm	LargeDependenceEmphasis	9.967272727
wavelet-HLL	gldm	LargeDependenceLowGrayLevelEmphasis	0.024983217
wavelet-HLL	gldm	DependenceVariance	4.154790083
wavelet-HLL	gldm	LargeDependenceHighGrayLevelEmphasis	7017.254545
wavelet-HLL	gldm	SmallDependenceLowGrayLevelEmphasis	0.004686004
wavelet-HLL	gldm	LowGrayLevelEmphasis	0.006612439
wavelet-HLL	glcm	JointAverage	24.52358412
wavelet-HLL	glcm	SumAverage	49.04716823
wavelet-HLL	glcm	JointEntropy	8.473216787
wavelet-HLL	glcm	ClusterShade	6252.00564
wavelet-HLL	glcm	MaximumProbability	0.019678272
wavelet-HLL	glcm	Idmn	0.971966859
wavelet-HLL	glcm	JointEnergy	0.00430978
wavelet-HLL	glcm	Contrast	187.6734163
wavelet-HLL	glcm	DifferenceEntropy	4.429598198
wavelet-HLL	glcm	InverseVariance	0.172692804
wavelet-HLL	glcm	DifferenceVariance	105.561789
wavelet-HLL	glcm	Idn	0.905868281
wavelet-HLL	glcm	Idm	0.172080542
wavelet-HLL	glcm	Correlation	0.146076398
wavelet-HLL	glcm	Autocorrelation	621.8168957
wavelet-HLL	glcm	SumEntropy	5.564874471
wavelet-HLL	glcm	SumSquares	114.1019428
wavelet-HLL	glcm	ClusterProminence	557514.8772
wavelet-HLL	glcm	Imc2	0.976543455
wavelet-HLL	glcm	Imc1	-0.310907115
wavelet-HLL	glcm	DifferenceAverage	8.754447792
wavelet-HLL	glcm	Id	0.251772952
wavelet-HLL	glcm	ClusterTendency	268.7343548
wavelet-HLL	firstorder	InterquartileRange	242.3924984
wavelet-HLL	firstorder	Skewness	1.303679196
wavelet-HLL	firstorder	Uniformity	0.036707438
wavelet-HLL	firstorder	Median	-68.51053154
wavelet-HLL	firstorder	Energy	49687196.97
wavelet-HLL	firstorder	RobustMeanAbsoluteDeviation	116.0253931
wavelet-HLL	firstorder	MeanAbsoluteDeviation	204.6728723
wavelet-HLL	firstorder	TotalEnergy	1341554318
wavelet-HLL	firstorder	Maximum	1171.059976
wavelet-HLL	firstorder	RootMeanSquared	300.5667282
wavelet-HLL	firstorder	90Percentile	347.5114772
wavelet-HLL	firstorder	Minimum	-656.8967649
wavelet-HLL	firstorder	Entropy	5.199572584
wavelet-HLL	firstorder	Range	1827.956741
wavelet-HLL	firstorder	Variance	87958.57689

wavelet-HLL	firstorder	10Percentile	-374.6369815
wavelet-HLL	firstorder	Kurtosis	5.450977461
wavelet-HLL	firstorder	Mean	-48.80349609
wavelet-HLL	glrlm	ShortRunLowGrayLevelEmphasis	0.006727042
wavelet-HLL	glrlm	GrayLevelVariance	147.2390104
wavelet-HLL	glrlm	LowGrayLevelRunEmphasis	0.006839188
wavelet-HLL	glrlm	GrayLevelNonUniformityNormalized	0.034970173
wavelet-HLL	glrlm	RunVariance	0.077916809
wavelet-HLL	glrlm	GrayLevelNonUniformity	18.20506815
wavelet-HLL	glrlm	LongRunEmphasis	1.200002946
wavelet-HLL	glrlm	ShortRunHighGrayLevelEmphasis	777.1774369
wavelet-HLL	glrlm	RunLengthNonUniformity	472.5456418
wavelet-HLL	glrlm	ShortRunEmphasis	0.961967555
wavelet-HLL	glrlm	LongRunHighGrayLevelEmphasis	933.726951
wavelet-HLL	glrlm	RunPercentage	0.945734266
wavelet-HLL	glrlm	LongRunLowGrayLevelEmphasis	0.007356922
wavelet-HLL	glrlm	RunEntropy	5.474919878
wavelet-HLL	glrlm	HighGrayLevelRunEmphasis	801.5910448
wavelet-HLL	glrlm	RunLengthNonUniformityNormalized	0.906878014
wavelet-HLL	glszm	GrayLevelVariance	211.3442858
wavelet-HLL	glszm	ZoneVariance	9.52127063
wavelet-HLL	glszm	GrayLevelNonUniformityNormalized	0.026317898
wavelet-HLL	glszm	SizeZoneNonUniformityNormalized	0.581216952
wavelet-HLL	glszm	SizeZoneNonUniformity	183.664557
wavelet-HLL	glszm	GrayLevelNonUniformity	8.316455696
wavelet-HLL	glszm	LargeAreaEmphasis	12.55063291
wavelet-HLL	glszm	SmallAreaHighGrayLevelEmphasis	812.391709
wavelet-HLL	glszm	ZonePercentage	0.574545455
wavelet-HLL	glszm	LargeAreaLowGrayLevelEmphasis	0.030508327
wavelet-HLL	glszm	LargeAreaHighGrayLevelEmphasis	8896.050633
wavelet-HLL	glszm	HighGrayLevelZoneEmphasis	928.9398734
wavelet-HLL	glszm	SmallAreaEmphasis	0.79012605
wavelet-HLL	glszm	LowGrayLevelZoneEmphasis	0.00893348
wavelet-HLL	glszm	ZoneEntropy	6.412794989
wavelet-HLL	glszm	SmallAreaLowGrayLevelEmphasis	0.007658505
wavelet-HLL	ngtdm	Coarseness	0.013217831
wavelet-HLL	ngtdm	Complexity	11477.91472
wavelet-HLL	ngtdm	Strength	20.81626155
wavelet-HLL	ngtdm	Contrast	0.491002903
wavelet-HLL	ngtdm	Busyness	0.045161016
wavelet-LHL	gldm	GrayLevelVariance	48.24935207
wavelet-LHL	gldm	HighGrayLevelEmphasis	603.58
wavelet-LHL	gldm	DependenceEntropy	6.542545806
wavelet-LHL	gldm	DependenceNonUniformity	116.9236364
wavelet-LHL	gldm	GrayLevelNonUniformity	32.85454545
wavelet-LHL	gldm	SmallDependenceEmphasis	0.440685393
wavelet-LHL	gldm	SmallDependenceHighGrayLevelEmphasis	263.1124639
wavelet-LHL	gldm	DependenceNonUniformityNormalized	0.21258843
wavelet-LHL	gldm	LargeDependenceEmphasis	19.32727273
wavelet-LHL	gldm	LargeDependenceLowGrayLevelEmphasis	0.034590773
wavelet-LHL	gldm	DependenceVariance	8.947160331
wavelet-LHL	gldm	LargeDependenceHighGrayLevelEmphasis	12483.76909
wavelet-LHL	gldm	SmallDependenceLowGrayLevelEmphasis	0.003882743
wavelet-LHL	gldm	LowGrayLevelEmphasis	0.004980502
wavelet-LHL	glcm	JointAverage	23.39799916

wavelet-LHL	glcm	SumAverage	46.79599833
wavelet-LHL	glcm	JointEntropy	7.765673412
wavelet-LHL	glcm	ClusterShade	216.0654403
wavelet-LHL	glcm	MaximumProbability	0.044200122
wavelet-LHL	glcm	Idmn	0.974753336
wavelet-LHL	glcm	JointEnergy	0.00922839
wavelet-LHL	glcm	Contrast	67.79531665
wavelet-LHL	glcm	DifferenceEntropy	3.932617751
wavelet-LHL	glcm	InverseVariance	0.224016164
wavelet-LHL	glcm	DifferenceVariance	35.60277619
wavelet-LHL	glcm	Idn	0.905769853
wavelet-LHL	glcm	Idm	0.239802276
wavelet-LHL	glcm	Correlation	0.160356316
wavelet-LHL	glcm	Autocorrelation	554.3575711
wavelet-LHL	glcm	SumEntropy	4.983080029
wavelet-LHL	glcm	SumSquares	40.7819226
wavelet-LHL	glcm	ClusterProminence	48644.67599
wavelet-LHL	glcm	Imc2	0.932823318
wavelet-LHL	glcm	Imc1	-0.234450361
wavelet-LHL	glcm	DifferenceAverage	5.566093244
wavelet-LHL	glcm	Id	0.318451432
wavelet-LHL	glcm	ClusterTendency	95.33237376
wavelet-LHL	firstorder	InterquartileRange	161.4488449
wavelet-LHL	firstorder	Skewness	0.16450686
wavelet-LHL	firstorder	Uniformity	0.059735537
wavelet-LHL	firstorder	Median	-31.27530288
wavelet-LHL	firstorder	Energy	17796889.19
wavelet-LHL	firstorder	RobustMeanAbsoluteDeviation	76.09538935
wavelet-LHL	firstorder	MeanAbsoluteDeviation	126.0889157
wavelet-LHL	firstorder	TotalEnergy	480516008.2
wavelet-LHL	firstorder	Maximum	568.4533895
wavelet-LHL	firstorder	RootMeanSquared	179.8832409
wavelet-LHL	firstorder	90Percentile	164.1172504
wavelet-LHL	firstorder	Minimum	-603.6526991
wavelet-LHL	firstorder	Entropy	4.598546329
wavelet-LHL	firstorder	Range	1172.106089
wavelet-LHL	firstorder	Variance	30044.91269
wavelet-LHL	firstorder	10Percentile	-265.7140314
wavelet-LHL	firstorder	Kurtosis	4.153795626
wavelet-LHL	firstorder	Mean	-48.09436209
wavelet-LHL	glrlm	ShortRunLowGrayLevelEmphasis	0.005181583
wavelet-LHL	glrlm	GrayLevelVariance	51.44645859
wavelet-LHL	glrlm	LowGrayLevelRunEmphasis	0.005287908
wavelet-LHL	glrlm	GrayLevelNonUniformityNormalized	0.053156278
wavelet-LHL	glrlm	RunVariance	0.122240194
wavelet-LHL	glrlm	GrayLevelNonUniformity	26.75147546
wavelet-LHL	glrlm	LongRunEmphasis	1.321265119
wavelet-LHL	glrlm	ShortRunHighGrayLevelEmphasis	562.0066783
wavelet-LHL	glrlm	RunLengthNonUniformity	430.9069258
wavelet-LHL	glrlm	ShortRunEmphasis	0.939309708
wavelet-LHL	glrlm	LongRunHighGrayLevelEmphasis	805.4767782
wavelet-LHL	glrlm	RunPercentage	0.914545455
wavelet-LHL	glrlm	LongRunLowGrayLevelEmphasis	0.005836556
wavelet-LHL	glrlm	RunEntropy	5.037373453
wavelet-LHL	glrlm	HighGrayLevelRunEmphasis	600.4801881

wavelet-LHL	glrlm	RunLengthNonUniformityNormalized	0.855309617
wavelet-LHL	glszm	GrayLevelVariance	75.32889339
wavelet-LHL	glszm	ZoneVariance	24.47439928
wavelet-LHL	glszm	GrayLevelNonUniformityNormalized	0.034658213
wavelet-LHL	glszm	SizeZoneNonUniformityNormalized	0.544914487
wavelet-LHL	glszm	SizeZoneNonUniformity	149.3065693
wavelet-LHL	glszm	GrayLevelNonUniformity	9.496350365
wavelet-LHL	glszm	LargeAreaEmphasis	28.50364964
wavelet-LHL	glszm	SmallAreaHighGrayLevelEmphasis	458.7541136
wavelet-LHL	glszm	ZonePercentage	0.498181818
wavelet-LHL	glszm	LargeAreaLowGrayLevelEmphasis	0.051611104
wavelet-LHL	glszm	LargeAreaHighGrayLevelEmphasis	18478.62409
wavelet-LHL	glszm	HighGrayLevelZoneEmphasis	592.6423358
wavelet-LHL	glszm	SmallAreaEmphasis	0.766021779
wavelet-LHL	glszm	LowGrayLevelZoneEmphasis	0.008089356
wavelet-LHL	glszm	ZoneEntropy	6.038535317
wavelet-LHL	glszm	SmallAreaLowGrayLevelEmphasis	0.007541208
wavelet-LHL	ngtdm	Coarseness	0.012131508
wavelet-LHL	ngtdm	Complexity	3393.066163
wavelet-LHL	ngtdm	Strength	8.286414046
wavelet-LHL	ngtdm	Contrast	0.212798101
wavelet-LHL	ngtdm	Busyness	0.064342125
wavelet-LHH	gldm	GrayLevelVariance	18.08415207
wavelet-LHH	gldm	HighGrayLevelEmphasis	364.5854545
wavelet-LHH	gldm	DependenceEntropy	6.280457383
wavelet-LHH	gldm	DependenceNonUniformity	81.25090909
wavelet-LHH	gldm	GrayLevelNonUniformity	53.88363636
wavelet-LHH	gldm	SmallDependenceEmphasis	0.334298781
wavelet-LHH	gldm	SmallDependenceHighGrayLevelEmphasis	132.6241056
wavelet-LHH	gldm	DependenceNonUniformityNormalized	0.147728926
wavelet-LHH	gldm	LargeDependenceEmphasis	25.98545455
wavelet-LHH	gldm	LargeDependenceLowGrayLevelEmphasis	0.08219732
wavelet-LHH	gldm	DependenceVariance	10.56198347
wavelet-LHH	gldm	LargeDependenceHighGrayLevelEmphasis	8681.48
wavelet-LHH	gldm	SmallDependenceLowGrayLevelEmphasis	0.003678005
wavelet-LHH	gldm	LowGrayLevelEmphasis	0.005774078
wavelet-LHH	glcm	JointAverage	18.6105414
wavelet-LHH	glcm	SumAverage	37.22108281
wavelet-LHH	glcm	JointEntropy	6.877849495
wavelet-LHH	glcm	ClusterShade	50.91275556
wavelet-LHH	glcm	MaximumProbability	0.064653307
wavelet-LHH	glcm	Idmn	0.979771167
wavelet-LHH	glcm	JointEnergy	0.018099511
wavelet-LHH	glcm	Contrast	31.29737511
wavelet-LHH	glcm	DifferenceEntropy	3.420308749
wavelet-LHH	glcm	InverseVariance	0.301189873
wavelet-LHH	glcm	DifferenceVariance	17.14865542
wavelet-LHH	glcm	Idn	0.916582909
wavelet-LHH	glcm	Idm	0.316036726
wavelet-LHH	glcm	Correlation	-0.008011165
wavelet-LHH	glcm	Autocorrelation	346.2135618
wavelet-LHH	glcm	SumEntropy	4.232343127
wavelet-LHH	glcm	SumSquares	15.50408864
wavelet-LHH	glcm	ClusterProminence	6237.570638
wavelet-LHH	glcm	Imc2	0.832935636

wavelet-LHH	glcm	Imc1	-0.159336255
wavelet-LHH	glcm	DifferenceAverage	3.729370753
wavelet-LHH	glcm	Id	0.389036616
wavelet-LHH	glcm	ClusterTendency	30.71897947
wavelet-LHH	firstorder	InterquartileRange	92.04877094
wavelet-LHH	firstorder	Skewness	0.280579985
wavelet-LHH	firstorder	Uniformity	0.097970248
wavelet-LHH	firstorder	Median	-5.250998945
wavelet-LHH	firstorder	Energy	6175497.328
wavelet-LHH	firstorder	RobustMeanAbsoluteDeviation	39.68481054
wavelet-LHH	firstorder	MeanAbsoluteDeviation	71.82722819
wavelet-LHH	firstorder	TotalEnergy	166738427.8
wavelet-LHH	firstorder	Maximum	470.4010881
wavelet-LHH	firstorder	RootMeanSquared	105.9630924
wavelet-LHH	firstorder	90Percentile	122.2088531
wavelet-LHH	firstorder	Minimum	-438.1052473
wavelet-LHH	firstorder	Entropy	3.877702235
wavelet-LHH	firstorder	Range	908.5063354
wavelet-LHH	firstorder	Variance	11220.55644
wavelet-LHH	firstorder	10Percentile	-101.0408845
wavelet-LHH	firstorder	Kurtosis	6.222909117
wavelet-LHH	firstorder	Mean	2.760529129
wavelet-LHH	glrlm	ShortRunLowGrayLevelEmphasis	0.00587562
wavelet-LHH	glrlm	GrayLevelVariance	19.98512938
wavelet-LHH	glrlm	LowGrayLevelRunEmphasis	0.00611766
wavelet-LHH	glrlm	GrayLevelNonUniformityNormalized	0.08654951
wavelet-LHH	glrlm	RunVariance	0.181004347
wavelet-LHH	glrlm	GrayLevelNonUniformity	42.26400043
wavelet-LHH	glrlm	LongRunEmphasis	1.453682233
wavelet-LHH	glrlm	ShortRunHighGrayLevelEmphasis	341.6137667
wavelet-LHH	glrlm	RunLengthNonUniformity	399.2755918
wavelet-LHH	glrlm	ShortRunEmphasis	0.921638169
wavelet-LHH	glrlm	LongRunHighGrayLevelEmphasis	520.3955509
wavelet-LHH	glrlm	RunPercentage	0.887412587
wavelet-LHH	glrlm	LongRunLowGrayLevelEmphasis	0.007515531
wavelet-LHH	glrlm	RunEntropy	4.455460549
wavelet-LHH	glrlm	HighGrayLevelRunEmphasis	368.1274474
wavelet-LHH	glrlm	RunLengthNonUniformityNormalized	0.817080867
wavelet-LHH	glszm	GrayLevelVariance	38.82474757
wavelet-LHH	glszm	ZoneVariance	78.57661014
wavelet-LHH	glszm	GrayLevelNonUniformityNormalized	0.04950495
wavelet-LHH	glszm	SizeZoneNonUniformityNormalized	0.514263308
wavelet-LHH	glszm	SizeZoneNonUniformity	103.8811881
wavelet-LHH	glszm	GrayLevelNonUniformity	10
wavelet-LHH	glszm	LargeAreaEmphasis	85.99009901
wavelet-LHH	glszm	SmallAreaHighGrayLevelEmphasis	299.8271748
wavelet-LHH	glszm	ZonePercentage	0.367272727
wavelet-LHH	glszm	LargeAreaLowGrayLevelEmphasis	0.273605234
wavelet-LHH	glszm	LargeAreaHighGrayLevelEmphasis	28208.59406
wavelet-LHH	glszm	HighGrayLevelZoneEmphasis	398.1336634
wavelet-LHH	glszm	SmallAreaEmphasis	0.743897734
wavelet-LHH	glszm	LowGrayLevelZoneEmphasis	0.010343842
wavelet-LHH	glszm	ZoneEntropy	5.661392561
wavelet-LHH	glszm	SmallAreaLowGrayLevelEmphasis	0.009486245
wavelet-LHH	ngtdm	Coarseness	0.012979979

wavelet-LHH	ngtdm	Complexity	1200.560862
wavelet-LHH	ngtdm	Strength	4.304887491
wavelet-LHH	ngtdm	Contrast	0.117376714
wavelet-LHH	ngtdm	Busyness	0.103007976
wavelet-LLH	gldm	GrayLevelVariance	72.54479669
wavelet-LLH	gldm	HighGrayLevelEmphasis	852.1727273
wavelet-LLH	gldm	DependenceEntropy	6.69451228
wavelet-LLH	gldm	DependenceNonUniformity	127.5854545
wavelet-LLH	gldm	GrayLevelNonUniformity	28.45454545
wavelet-LLH	gldm	SmallDependenceEmphasis	0.462362433
wavelet-LLH	gldm	SmallDependenceHighGrayLevelEmphasis	414.2270313
wavelet-LLH	gldm	DependenceNonUniformityNormalized	0.231973554
wavelet-LLH	gldm	LargeDependenceEmphasis	17.05818182
wavelet-LLH	gldm	LargeDependenceLowGrayLevelEmphasis	0.023396706
wavelet-LLH	gldm	DependenceVariance	8.101765289
wavelet-LLH	gldm	LargeDependenceHighGrayLevelEmphasis	14690.11091
wavelet-LLH	gldm	SmallDependenceLowGrayLevelEmphasis	0.003083366
wavelet-LLH	gldm	LowGrayLevelEmphasis	0.003906443
wavelet-LLH	glcm	JointAverage	27.67867733
wavelet-LLH	glcm	SumAverage	55.35735466
wavelet-LLH	glcm	JointEntropy	7.998697703
wavelet-LLH	glcm	ClusterShade	722.1286884
wavelet-LLH	glcm	MaximumProbability	0.040718868
wavelet-LLH	glcm	Idmn	0.971616842
wavelet-LLH	glcm	JointEnergy	0.008267634
wavelet-LLH	glcm	Contrast	101.3210745
wavelet-LLH	glcm	DifferenceEntropy	4.146541331
wavelet-LLH	glcm	InverseVariance	0.217641461
wavelet-LLH	glcm	DifferenceVariance	55.26488581
wavelet-LLH	glcm	Idn	0.903264139
wavelet-LLH	glcm	Idm	0.225006578
wavelet-LLH	glcm	Correlation	0.172101131
wavelet-LLH	glcm	Autocorrelation	777.1907619
wavelet-LLH	glcm	SumEntropy	5.229614614
wavelet-LLH	glcm	SumSquares	61.72179309
wavelet-LLH	glcm	ClusterProminence	105744.7822
wavelet-LLH	glcm	Imc2	0.960978731
wavelet-LLH	glcm	Imc1	-0.278400756
wavelet-LLH	glcm	DifferenceAverage	6.657097112
wavelet-LLH	glcm	Id	0.301068681
wavelet-LLH	glcm	ClusterTendency	145.5660978
wavelet-LLH	firstorder	InterquartileRange	183.9506586
wavelet-LLH	firstorder	Skewness	0.2716589
wavelet-LLH	firstorder	Uniformity	0.051735537
wavelet-LLH	firstorder	Median	-32.86100055
wavelet-LLH	firstorder	Energy	25672005.95
wavelet-LLH	firstorder	RobustMeanAbsoluteDeviation	86.59574007
wavelet-LLH	firstorder	MeanAbsoluteDeviation	150.3953399
wavelet-LLH	firstorder	TotalEnergy	693144160.6
wavelet-LLH	firstorder	Maximum	634.8972956
wavelet-LLH	firstorder	RootMeanSquared	216.0471579
wavelet-LLH	firstorder	90Percentile	249.9259084
wavelet-LLH	firstorder	Minimum	-706.9116888
wavelet-LLH	firstorder	Entropy	4.837121685
wavelet-LLH	firstorder	Range	1341.808984

wavelet-LLH	firstorder	Variance	45124.73133
wavelet-LLH	firstorder	10Percentile	-287.1802088
wavelet-LLH	firstorder	Kurtosis	3.965355437
wavelet-LLH	firstorder	Mean	-39.3909015
wavelet-LLH	glrlm	ShortRunLowGrayLevelEmphasis	0.004050526
wavelet-LLH	glrlm	GrayLevelVariance	77.28529443
wavelet-LLH	glrlm	LowGrayLevelRunEmphasis	0.004122664
wavelet-LLH	glrlm	GrayLevelNonUniformityNormalized	0.045999587
wavelet-LLH	glrlm	RunVariance	0.10952484
wavelet-LLH	glrlm	GrayLevelNonUniformity	23.36885808
wavelet-LLH	glrlm	LongRunEmphasis	1.283673425
wavelet-LLH	glrlm	ShortRunHighGrayLevelEmphasis	807.7200021
wavelet-LLH	glrlm	RunLengthNonUniformity	441.6162525
wavelet-LLH	glrlm	ShortRunEmphasis	0.946056849
wavelet-LLH	glrlm	LongRunHighGrayLevelEmphasis	1092.773624
wavelet-LLH	glrlm	RunPercentage	0.923356643
wavelet-LLH	glrlm	LongRunLowGrayLevelEmphasis	0.004490311
wavelet-LLH	glrlm	RunEntropy	5.237082326
wavelet-LLH	glrlm	HighGrayLevelRunEmphasis	852.9677549
wavelet-LLH	glrlm	RunLengthNonUniformityNormalized	0.869194005
wavelet-LLH	glszm	GrayLevelVariance	113.4121473
wavelet-LLH	glszm	ZoneVariance	23.49757934
wavelet-LLH	glszm	GrayLevelNonUniformityNormalized	0.029952565
wavelet-LLH	glszm	SizeZoneNonUniformityNormalized	0.557949044
wavelet-LLH	glszm	SizeZoneNonUniformity	159.5734266
wavelet-LLH	glszm	GrayLevelNonUniformity	8.566433566
wavelet-LLH	glszm	LargeAreaEmphasis	27.1958042
wavelet-LLH	glszm	SmallAreaHighGrayLevelEmphasis	705.0984113
wavelet-LLH	glszm	ZonePercentage	0.52
wavelet-LLH	glszm	LargeAreaLowGrayLevelEmphasis	0.037619609
wavelet-LLH	glszm	LargeAreaHighGrayLevelEmphasis	23337.12937
wavelet-LLH	glszm	HighGrayLevelZoneEmphasis	883.3776224
wavelet-LLH	glszm	SmallAreaEmphasis	0.774843881
wavelet-LLH	glszm	LowGrayLevelZoneEmphasis	0.006156074
wavelet-LLH	glszm	ZoneEntropy	6.211977146
wavelet-LLH	glszm	SmallAreaLowGrayLevelEmphasis	0.005733319
wavelet-LLH	ngtdm	Coarseness	0.014103624
wavelet-LLH	ngtdm	Complexity	4692.886929
wavelet-LLH	ngtdm	Strength	10.34421842
wavelet-LLH	ngtdm	Contrast	0.299883539
wavelet-LLH	ngtdm	Busyness	0.044418729
wavelet-LLL	gldm	GrayLevelVariance	442.7011868
wavelet-LLL	gldm	HighGrayLevelEmphasis	4092.838182
wavelet-LLL	gldm	DependenceEntropy	7.014264903
wavelet-LLL	gldm	DependenceNonUniformity	207.6472727
wavelet-LLL	gldm	GrayLevelNonUniformity	14.81090909
wavelet-LLL	gldm	SmallDependenceEmphasis	0.637309986
wavelet-LLL	gldm	SmallDependenceHighGrayLevelEmphasis	2106.563548
wavelet-LLL	gldm	DependenceNonUniformityNormalized	0.377540496
wavelet-LLL	gldm	LargeDependenceEmphasis	8.72
wavelet-LLL	gldm	LargeDependenceLowGrayLevelEmphasis	0.004916751
wavelet-LLL	gldm	DependenceVariance	4.007153719
wavelet-LLL	gldm	LargeDependenceHighGrayLevelEmphasis	49518.05636
wavelet-LLL	gldm	SmallDependenceLowGrayLevelEmphasis	0.003426342
wavelet-LLL	gldm	LowGrayLevelEmphasis	0.00351426

wavelet-LLL	glcm	JointAverage	65.66194734
wavelet-LLL	glcm	SumAverage	131.3238947
wavelet-LLL	glcm	JointEntropy	8.74904446
wavelet-LLL	glcm	ClusterShade	-31235.19922
wavelet-LLL	glcm	MaximumProbability	0.018296021
wavelet-LLL	glcm	Idmn	0.951719597
wavelet-LLL	glcm	JointEnergy	0.003739167
wavelet-LLL	glcm	Contrast	433.1977476
wavelet-LLL	glcm	DifferenceEntropy	5.113834374
wavelet-LLL	glcm	InverseVariance	0.146262127
wavelet-LLL	glcm	DifferenceVariance	221.1370395
wavelet-LLL	glcm	Idn	0.872864357
wavelet-LLL	glcm	Idm	0.144060657
wavelet-LLL	glcm	Correlation	0.368680217
wavelet-LLL	glcm	Autocorrelation	4438.825168
wavelet-LLL	glcm	SumEntropy	6.169209307
wavelet-LLL	glcm	SumSquares	343.5532231
wavelet-LLL	glcm	ClusterProminence	3078480.23
wavelet-LLL	glcm	Imc2	0.992074788
wavelet-LLL	glcm	Imc1	-0.384563127
wavelet-LLL	glcm	DifferenceAverage	14.45891661
wavelet-LLL	glcm	Id	0.209849944
wavelet-LLL	glcm	ClusterTendency	941.015145
wavelet-LLL	firstorder	InterquartileRange	844.9296499
wavelet-LLL	firstorder	Skewness	-0.824917382
wavelet-LLL	firstorder	Uniformity	0.026928926
wavelet-LLL	firstorder	Median	-125.4401126
wavelet-LLL	firstorder	Energy	211148555.4
wavelet-LLL	firstorder	RobustMeanAbsoluteDeviation	351.0729739
wavelet-LLL	firstorder	MeanAbsoluteDeviation	450.9795143
wavelet-LLL	firstorder	TotalEnergy	5701010995
wavelet-LLL	firstorder	Maximum	316.9473062
wavelet-LLL	firstorder	RootMeanSquared	619.6018595
wavelet-LLL	firstorder	90Percentile	180.1489175
wavelet-LLL	firstorder	Minimum	-1815.879043
wavelet-LLL	firstorder	Entropy	5.741571894
wavelet-LLL	firstorder	Range	2132.826349
wavelet-LLL	firstorder	Variance	276878.7977
wavelet-LLL	firstorder	10Percentile	-1091.612519
wavelet-LLL	firstorder	Kurtosis	2.599752075
wavelet-LLL	firstorder	Mean	-327.1508316
wavelet-LLL	glrlm	ShortRunLowGrayLevelEmphasis	0.00366502
wavelet-LLL	glrlm	GrayLevelVariance	448.3220387
wavelet-LLL	glrlm	LowGrayLevelRunEmphasis	0.00367148
wavelet-LLL	glrlm	GrayLevelNonUniformityNormalized	0.024692017
wavelet-LLL	glrlm	RunVariance	0.057996762
wavelet-LLL	glrlm	GrayLevelNonUniformity	12.97417374
wavelet-LLL	glrlm	LongRunEmphasis	1.155336887
wavelet-LLL	glrlm	ShortRunHighGrayLevelEmphasis	3827.657061
wavelet-LLL	glrlm	RunLengthNonUniformity	483.3126999
wavelet-LLL	glrlm	ShortRunEmphasis	0.967973505
wavelet-LLL	glrlm	LongRunHighGrayLevelEmphasis	4914.480624
wavelet-LLL	glrlm	RunPercentage	0.954965035
wavelet-LLL	glrlm	LongRunLowGrayLevelEmphasis	0.003701867
wavelet-LLL	glrlm	RunEntropy	5.988122837

wavelet-LLL	glrlm	HighGrayLevelRunEmphasis	4012.353693
wavelet-LLL	glrlm	RunLengthNonUniformityNormalized	0.919872402
wavelet-LLL	glszm	GrayLevelVariance	452.1771916
wavelet-LLL	glszm	ZoneVariance	7.766274811
wavelet-LLL	glszm	GrayLevelNonUniformityNormalized	0.016141895
wavelet-LLL	glszm	SizeZoneNonUniformityNormalized	0.754031782
wavelet-LLL	glszm	SizeZoneNonUniformity	277.4836957
wavelet-LLL	glszm	GrayLevelNonUniformity	5.940217391
wavelet-LLL	glszm	LargeAreaEmphasis	10
wavelet-LLL	glszm	SmallAreaHighGrayLevelEmphasis	2809.751962
wavelet-LLL	glszm	ZonePercentage	0.669090909
wavelet-LLL	glszm	LargeAreaLowGrayLevelEmphasis	0.006716755
wavelet-LLL	glszm	LargeAreaHighGrayLevelEmphasis	57129.8125
wavelet-LLL	glszm	HighGrayLevelZoneEmphasis	3326.652174
wavelet-LLL	glszm	SmallAreaEmphasis	0.887889615
wavelet-LLL	glszm	LowGrayLevelZoneEmphasis	0.005142667
wavelet-LLL	glszm	ZoneEntropy	6.592582952
wavelet-LLL	glszm	SmallAreaLowGrayLevelEmphasis	0.005103209
wavelet-LLL	ngtdm	Coarseness	0.008719326
wavelet-LLL	ngtdm	Complexity	30126.75559
wavelet-LLL	ngtdm	Strength	31.66998309
wavelet-LLL	ngtdm	Contrast	1.589610097
wavelet-LLL	ngtdm	Busyness	0.019328067
wavelet-HHL	gldm	GrayLevelVariance	17.66355372
wavelet-HHL	gldm	HighGrayLevelEmphasis	261.3072727
wavelet-HHL	gldm	DependenceEntropy	6.341748113
wavelet-HHL	gldm	DependenceNonUniformity	82.43636364
wavelet-HHL	gldm	GrayLevelNonUniformity	53.51272727
wavelet-HHL	gldm	SmallDependenceEmphasis	0.329307926
wavelet-HHL	gldm	SmallDependenceHighGrayLevelEmphasis	100.0855063
wavelet-HHL	gldm	DependenceNonUniformityNormalized	0.149884298
wavelet-HHL	gldm	LargeDependenceEmphasis	27.20727273
wavelet-HHL	gldm	LargeDependenceLowGrayLevelEmphasis	0.124751562
wavelet-HHL	gldm	DependenceVariance	11.38134215
wavelet-HHL	gldm	LargeDependenceHighGrayLevelEmphasis	6375.881818
wavelet-HHL	gldm	SmallDependenceLowGrayLevelEmphasis	0.004853186
wavelet-HHL	gldm	LowGrayLevelEmphasis	0.00828574
wavelet-HHL	glcm	JointAverage	15.61572477
wavelet-HHL	glcm	SumAverage	31.23144954
wavelet-HHL	glcm	JointEntropy	6.949035413
wavelet-HHL	glcm	ClusterShade	67.10425053
wavelet-HHL	glcm	MaximumProbability	0.06657914
wavelet-HHL	glcm	Idmn	0.970832177
wavelet-HHL	glcm	JointEnergy	0.017254375
wavelet-HHL	glcm	Contrast	30.84309904
wavelet-HHL	glcm	DifferenceEntropy	3.432384857
wavelet-HHL	glcm	InverseVariance	0.296960143
wavelet-HHL	glcm	DifferenceVariance	16.60977681
wavelet-HHL	glcm	Idn	0.900371279
wavelet-HHL	glcm	Idm	0.316080743
wavelet-HHL	glcm	Correlation	-0.000969946
wavelet-HHL	glcm	Autocorrelation	243.8890147
wavelet-HHL	glcm	SumEntropy	4.275015737
wavelet-HHL	glcm	SumSquares	15.44660496
wavelet-HHL	glcm	ClusterProminence	5173.997426

wavelet-HHL	glcm	Imc2	0.823007044
wavelet-HHL	glcm	Imc1	-0.152021626
wavelet-HHL	glcm	DifferenceAverage	3.738177363
wavelet-HHL	glcm	Id	0.389109537
wavelet-HHL	glcm	ClusterTendency	30.94332079
wavelet-HHL	firstorder	InterquartileRange	83.48978338
wavelet-HHL	firstorder	Skewness	0.256580487
wavelet-HHL	firstorder	Uniformity	0.097295868
wavelet-HHL	firstorder	Median	-7.295755812
wavelet-HHL	firstorder	Energy	6056381.257
wavelet-HHL	firstorder	RobustMeanAbsoluteDeviation	39.96260696
wavelet-HHL	firstorder	MeanAbsoluteDeviation	72.3565676
wavelet-HHL	firstorder	TotalEnergy	163522293.9
wavelet-HHL	firstorder	Maximum	351.1356037
wavelet-HHL	firstorder	RootMeanSquared	104.936182
wavelet-HHL	firstorder	90Percentile	140.4710148
wavelet-HHL	firstorder	Minimum	-373.6126143
wavelet-HHL	firstorder	Entropy	3.89918335
wavelet-HHL	firstorder	Range	724.748218
wavelet-HHL	firstorder	Variance	11003.95264
wavelet-HHL	firstorder	10Percentile	-105.3091044
wavelet-HHL	firstorder	Kurtosis	4.760164937
wavelet-HHL	firstorder	Mean	2.76579915
wavelet-HHL	glrlm	ShortRunLowGrayLevelEmphasis	0.008387301
wavelet-HHL	glrlm	GrayLevelVariance	19.4476008
wavelet-HHL	glrlm	LowGrayLevelRunEmphasis	0.008761405
wavelet-HHL	glrlm	GrayLevelNonUniformityNormalized	0.085392893
wavelet-HHL	glrlm	RunVariance	0.17998448
wavelet-HHL	glrlm	GrayLevelNonUniformity	41.5960573
wavelet-HHL	glrlm	LongRunEmphasis	1.457269095
wavelet-HHL	glrlm	ShortRunHighGrayLevelEmphasis	245.8802265
wavelet-HHL	glrlm	RunLengthNonUniformity	396.3272984
wavelet-HHL	glrlm	ShortRunEmphasis	0.919984794
wavelet-HHL	glrlm	LongRunHighGrayLevelEmphasis	371.8125238
wavelet-HHL	glrlm	RunPercentage	0.885454545
wavelet-HHL	glrlm	LongRunLowGrayLevelEmphasis	0.01085973
wavelet-HHL	glrlm	RunEntropy	4.488803199
wavelet-HHL	glrlm	HighGrayLevelRunEmphasis	264.7473704
wavelet-HHL	glrlm	RunLengthNonUniformityNormalized	0.813248848
wavelet-HHL	glszm	GrayLevelVariance	34.849375
wavelet-HHL	glszm	ZoneVariance	89.2675
wavelet-HHL	glszm	GrayLevelNonUniformityNormalized	0.0515
wavelet-HHL	glszm	SizeZoneNonUniformityNormalized	0.4938
wavelet-HHL	glszm	SizeZoneNonUniformity	98.76
wavelet-HHL	glszm	GrayLevelNonUniformity	10.3
wavelet-HHL	glszm	LargeAreaEmphasis	96.83
wavelet-HHL	glszm	SmallAreaHighGrayLevelEmphasis	231.3302198
wavelet-HHL	glszm	ZonePercentage	0.363636364
wavelet-HHL	glszm	LargeAreaLowGrayLevelEmphasis	0.444773958
wavelet-HHL	glszm	LargeAreaHighGrayLevelEmphasis	22022.935
wavelet-HHL	glszm	HighGrayLevelZoneEmphasis	301.355
wavelet-HHL	glszm	SmallAreaEmphasis	0.729755139
wavelet-HHL	glszm	LowGrayLevelZoneEmphasis	0.014133947
wavelet-HHL	glszm	ZoneEntropy	5.61460242
wavelet-HHL	glszm	SmallAreaLowGrayLevelEmphasis	0.012353188

wavelet-HHL	ngtdm	Coarseness	0.013995246
wavelet-HHL	ngtdm	Complexity	931.0884747
wavelet-HHL	ngtdm	Strength	3.228647735
wavelet-HHL	ngtdm	Contrast	0.128641899
wavelet-HHL	ngtdm	Busyness	0.128763253
wavelet-HLH	gldm	GrayLevelVariance	26.04793388
wavelet-HLH	gldm	HighGrayLevelEmphasis	452.6581818
wavelet-HLH	gldm	DependenceEntropy	6.475899878
wavelet-HLH	gldm	DependenceNonUniformity	97.59272727
wavelet-HLH	gldm	GrayLevelNonUniformity	43.30909091
wavelet-HLH	gldm	SmallDependenceEmphasis	0.389819953
wavelet-HLH	gldm	SmallDependenceHighGrayLevelEmphasis	186.7302872
wavelet-HLH	gldm	DependenceNonUniformityNormalized	0.177441322
wavelet-HLH	gldm	LargeDependenceEmphasis	22.6
wavelet-HLH	gldm	LargeDependenceLowGrayLevelEmphasis	0.059016826
wavelet-HLH	gldm	DependenceVariance	9.978128926
wavelet-HLH	gldm	LargeDependenceHighGrayLevelEmphasis	9312.138182
wavelet-HLH	gldm	SmallDependenceLowGrayLevelEmphasis	0.003355982
wavelet-HLH	gldm	LowGrayLevelEmphasis	0.004929491
wavelet-HLH	glcm	JointAverage	20.72405111
wavelet-HLH	glcm	SumAverage	41.44810223
wavelet-HLH	glcm	JointEntropy	7.375358362
wavelet-HLH	glcm	ClusterShade	68.79287427
wavelet-HLH	glcm	MaximumProbability	0.061637768
wavelet-HLH	glcm	Idmn	0.971398673
wavelet-HLH	glcm	JointEnergy	0.01310791
wavelet-HLH	glcm	Contrast	45.32394351
wavelet-HLH	glcm	DifferenceEntropy	3.67784319
wavelet-HLH	glcm	InverseVariance	0.266398385
wavelet-HLH	glcm	DifferenceVariance	23.98619555
wavelet-HLH	glcm	Idn	0.900718007
wavelet-HLH	glcm	Idm	0.279112369
wavelet-HLH	glcm	Correlation	0.011940761
wavelet-HLH	glcm	Autocorrelation	429.8480255
wavelet-HLH	glcm	SumEntropy	4.58250195
wavelet-HLH	glcm	SumSquares	22.99881486
wavelet-HLH	glcm	ClusterProminence	10732.92093
wavelet-HLH	glcm	Imc2	0.87236569
wavelet-HLH	glcm	Imc1	-0.178000961
wavelet-HLH	glcm	DifferenceAverage	4.576895922
wavelet-HLH	glcm	Id	0.354411604
wavelet-HLH	glcm	ClusterTendency	46.67131591
wavelet-HLH	firstorder	InterquartileRange	116.0845775
wavelet-HLH	firstorder	Skewness	0.147205169
wavelet-HLH	firstorder	Uniformity	0.078743802
wavelet-HLH	firstorder	Median	-8.238608914
wavelet-HLH	firstorder	Energy	8952835.361
wavelet-HLH	firstorder	RobustMeanAbsoluteDeviation	52.15437674
wavelet-HLH	firstorder	MeanAbsoluteDeviation	90.5591782
wavelet-HLH	firstorder	TotalEnergy	241726554.8
wavelet-HLH	firstorder	Maximum	404.3703006
wavelet-HLH	firstorder	RootMeanSquared	127.584805
wavelet-HLH	firstorder	90Percentile	172.0863266
wavelet-HLH	firstorder	Minimum	-487.8138831
wavelet-HLH	firstorder	Entropy	4.190856343

wavelet-HLH	firstorder	Range	892.1841837
wavelet-HLH	firstorder	Variance	16264.66438
wavelet-HLH	firstorder	10Percentile	-132.8257165
wavelet-HLH	firstorder	Kurtosis	4.367820797
wavelet-HLH	firstorder	Mean	3.635669758
wavelet-HLH	glrlm	ShortRunLowGrayLevelEmphasis	0.005021473
wavelet-HLH	glrlm	GrayLevelVariance	28.2446391
wavelet-HLH	glrlm	LowGrayLevelRunEmphasis	0.005192743
wavelet-HLH	glrlm	GrayLevelNonUniformityNormalized	0.068845223
wavelet-HLH	glrlm	RunVariance	0.153084792
wavelet-HLH	glrlm	GrayLevelNonUniformity	34.16387501
wavelet-HLH	glrlm	LongRunEmphasis	1.385317561
wavelet-HLH	glrlm	ShortRunHighGrayLevelEmphasis	428.0451791
wavelet-HLH	glrlm	RunLengthNonUniformity	416.8830028
wavelet-HLH	glrlm	ShortRunEmphasis	0.932361635
wavelet-HLH	glrlm	LongRunHighGrayLevelEmphasis	617.0973088
wavelet-HLH	glrlm	RunPercentage	0.901818182
wavelet-HLH	glrlm	LongRunLowGrayLevelEmphasis	0.006165773
wavelet-HLH	glrlm	RunEntropy	4.693690733
wavelet-HLH	glrlm	HighGrayLevelRunEmphasis	456.4400578
wavelet-HLH	glrlm	RunLengthNonUniformityNormalized	0.839838977
wavelet-HLH	glszm	GrayLevelVariance	46.62372522
wavelet-HLH	glszm	ZoneVariance	52.04330652
wavelet-HLH	glszm	GrayLevelNonUniformityNormalized	0.04567653
wavelet-HLH	glszm	SizeZoneNonUniformityNormalized	0.548226084
wavelet-HLH	glszm	SizeZoneNonUniformity	129.3813559
wavelet-HLH	glszm	GrayLevelNonUniformity	10.77966102
wavelet-HLH	glszm	LargeAreaEmphasis	57.47457627
wavelet-HLH	glszm	SmallAreaHighGrayLevelEmphasis	370.2131249
wavelet-HLH	glszm	ZonePercentage	0.429090909
wavelet-HLH	glszm	LargeAreaLowGrayLevelEmphasis	0.148986625
wavelet-HLH	glszm	LargeAreaHighGrayLevelEmphasis	23434.07627
wavelet-HLH	glszm	HighGrayLevelZoneEmphasis	481.0635593
wavelet-HLH	glszm	SmallAreaEmphasis	0.767396417
wavelet-HLH	glszm	LowGrayLevelZoneEmphasis	0.008075469
wavelet-HLH	glszm	ZoneEntropy	5.70584196
wavelet-HLH	glszm	SmallAreaLowGrayLevelEmphasis	0.007454084
wavelet-HLH	ngtdm	Coarseness	0.013632404
wavelet-HLH	ngtdm	Complexity	1608.410379
wavelet-HLH	ngtdm	Strength	4.42200622
wavelet-HLH	ngtdm	Contrast	0.167431982
wavelet-HLH	ngtdm	Busyness	0.087263481

Image type	Feature Class	Feature Name	4: RTSTRUCT: C99-DNU_segment_ITV
general	info	BoundingBox	(1, 1, 1, 6, 5, 6)
general	info	EnabledImageTypes	{u'Original': {}, u'Wavelet': {}}
general	info	GeneralSettings	{'distances': [1], 'additionalInfo': True, 'force2D': False, 'interpolator': 'sitkBSpline', 'resampledPixelSpacing': [3.0], 'label': 1, 'normalizeScale': 1, 'normalize': False, 'force2Ddimension': 0, 'removeOutliers': None, 'minimumROISize': None, u'binWidth': 25.0, 'minimumROIDimensions': 1, u'correctMask': True, 'preCrop': False, u'symmetricalGLCM': True, 'resegmentRange': None, 'padDistance': 5}
general	info	ImageHash	70aeba6c3849b93060ce5b4fbb5f55b0c1b25656
general	info	ImageSpacing	(0.9765625000000002, 0.9765625000000002, 2.0)
general	info	MaskHash	cbe7e1c709dc3ebdcb8a8f49f7a40fde700cee88
general	info	NumpyVersion	1.13.1
general	info	PyWaveletVersion	0.5.2
general	info	SimpleITKVersion	1.1.0
general	info	Version	1.3.0.post102+g1b0eb74
general	info	VolumeNum	1
general	info	VoxelNum	91
original	shape	Maximum3DDiameter	19.20937271
original	shape	Maximum2DDiameterSlice	17.49285568
original	shape	Sphericity	0.88996661
original	shape	MinorAxis	15.0332627
original	shape	Elongation	0.849763562
original	shape	SurfaceVolumeRatio	0.402694186
original	shape	Volume	2457
original	shape	MajorAxis	17.69111241
original	shape	SurfaceArea	989.4196152
original	shape	Flatness	0.766313213
original	shape	LeastAxis	13.5569332
original	shape	Maximum2DDiameterColumn	16.97056275
original	shape	Maximum2DDiameterRow	17.49285568
original	gldm	GrayLevelVariance	58.92935636
original	gldm	HighGrayLevelEmphasis	447.5824176
original	gldm	DependenceEntropy	5.504671426
original	gldm	DependenceNonUniformity	27.57142857
original	gldm	GrayLevelNonUniformity	4.824175824
original	gldm	SmallDependenceEmphasis	0.540034823
original	gldm	SmallDependenceHighGrayLevelEmphasis	187.9645803
original	gldm	DependenceNonUniformityNormalized	0.302982732
original	gldm	LargeDependenceEmphasis	7.285714286
original	gldm	LargeDependenceLowGrayLevelEmphasis	0.032876907
original	gldm	DependenceVariance	2.503562372
original	gldm	LargeDependenceHighGrayLevelEmphasis	4973.120879
original	gldm	SmallDependenceLowGrayLevelEmphasis	0.014365511

original	gldm	LowGrayLevelEmphasis	0.016903203
original	glcm	JointAverage	21.51113495
original	glcm	SumAverage	43.02226991
original	glcm	JointEntropy	6.394346338
original	glcm	ClusterShade	-1211.663906
original	glcm	MaximumProbability	0.038760813
original	glcm	Idmn	0.938819624
original	glcm	JointEnergy	0.013617969
original	glcm	Contrast	71.3690936
original	glcm	DifferenceEntropy	3.825625789
original	glcm	InverseVariance	0.169549167
original	glcm	DifferenceVariance	28.42752913
original	glcm	Idn	0.843519108
original	glcm	Idm	0.178304272
original	glcm	Correlation	0.314497269
original	glcm	Autocorrelation	479.8677349
original	glcm	SumEntropy	4.710577784
original	glcm	SumSquares	52.73385968
original	glcm	ClusterProminence	56071.34055
original	glcm	Imc2	0.992757245
original	glcm	Imc1	-0.499940328
original	glcm	DifferenceAverage	6.461330458
original	glcm	Id	0.262339303
original	glcm	ClusterTendency	139.5663451
original	firstorder	InterquartileRange	308
original	firstorder	Skewness	-0.536502694
original	firstorder	Uniformity	0.053012921
original	firstorder	Median	-177
original	firstorder	Energy	6744175
original	firstorder	RobustMeanAbsoluteDeviation	125.7770689
original	firstorder	MeanAbsoluteDeviation	161.1373023
original	firstorder	TotalEnergy	182092725
original	firstorder	Maximum	97
original	firstorder	RootMeanSquared	272.2348493
original	firstorder	90Percentile	22
original	firstorder	Minimum	-659
original	firstorder	Entropy	4.457539105
original	firstorder	Range	756
original	firstorder	Variance	36599.35998
original	firstorder	10Percentile	-490
original	firstorder	Kurtosis	2.206320929
original	firstorder	Mean	-193.6813187
original	glrlm	ShortRunLowGrayLevelEmphasis	0.017424542
original	glrlm	GrayLevelVariance	58.53519479
original	glrlm	LowGrayLevelRunEmphasis	0.017549527
original	glrlm	GrayLevelNonUniformityNormalized	0.050757528
original	glrlm	RunVariance	0.047281001
original	glrlm	GrayLevelNonUniformity	4.409321664
original	glrlm	LongRunEmphasis	1.146241019
original	glrlm	ShortRunHighGrayLevelEmphasis	413.4922309
original	glrlm	RunLengthNonUniformity	79.12657651
original	glrlm	ShortRunEmphasis	0.964433583
original	glrlm	LongRunHighGrayLevelEmphasis	534.616488

original	glrlm	RunPercentage	0.954353339
original	glrlm	LongRunLowGrayLevelEmphasis	0.018054539
original	glrlm	RunEntropy	4.635308215
original	glrlm	HighGrayLevelRunEmphasis	437.093747
original	glrlm	RunLengthNonUniformityNormalized	0.910555108
original	glszm	GrayLevelVariance	52.90446281
original	glszm	ZoneVariance	2.95338843
original	glszm	GrayLevelNonUniformityNormalized	0.04661157
original	glszm	SizeZoneNonUniformityNormalized	0.579504132
original	glszm	SizeZoneNonUniformity	31.87272727
original	glszm	GrayLevelNonUniformity	2.563636364
original	glszm	LargeAreaEmphasis	5.690909091
original	glszm	SmallAreaHighGrayLevelEmphasis	259.0274665
original	glszm	ZonePercentage	0.604395604
original	glszm	LargeAreaLowGrayLevelEmphasis	0.037941586
original	glszm	LargeAreaHighGrayLevelEmphasis	3526.909091
original	glszm	HighGrayLevelZoneEmphasis	352.5090909
original	glszm	SmallAreaEmphasis	0.78802132
original	glszm	LowGrayLevelZoneEmphasis	0.024937796
original	glszm	ZoneEntropy	4.962822441
original	glszm	SmallAreaLowGrayLevelEmphasis	0.023088912
original	ngtdm	Coarseness	0.04945409
original	ngtdm	Complexity	1341.077954
original	ngtdm	Strength	16.64477736
original	ngtdm	Contrast	0.852298099
original	ngtdm	Busyness	0.041191137
wavelet-HHH	gldm	GrayLevelVariance	7.942035986
wavelet-HHH	gldm	HighGrayLevelEmphasis	97.87912088
wavelet-HHH	gldm	DependenceEntropy	4.793930845
wavelet-HHH	gldm	DependenceNonUniformity	24.03296703
wavelet-HHH	gldm	GrayLevelNonUniformity	9.901098901
wavelet-HHH	gldm	SmallDependenceEmphasis	0.481480464
wavelet-HHH	gldm	SmallDependenceHighGrayLevelEmphasis	47.06506105
wavelet-HHH	gldm	DependenceNonUniformityNormalized	0.264098539
wavelet-HHH	gldm	LargeDependenceEmphasis	6.714285714
wavelet-HHH	gldm	LargeDependenceLowGrayLevelEmphasis	0.088102218
wavelet-HHH	gldm	DependenceVariance	1.63941553
wavelet-HHH	gldm	LargeDependenceHighGrayLevelEmphasis	673.5274725
wavelet-HHH	gldm	SmallDependenceLowGrayLevelEmphasis	0.019622721
wavelet-HHH	gldm	LowGrayLevelEmphasis	0.025688126
wavelet-HHH	glcm	JointAverage	9.519672672
wavelet-HHH	glcm	SumAverage	19.03934534
wavelet-HHH	glcm	JointEntropy	5.875625741
wavelet-HHH	glcm	ClusterShade	-10.46488268
wavelet-HHH	glcm	MaximumProbability	0.048410768
wavelet-HHH	glcm	Idmn	0.949220267
wavelet-HHH	glcm	JointEnergy	0.02005871
wavelet-HHH	glcm	Contrast	17.23781179
wavelet-HHH	glcm	DifferenceEntropy	3.000597291
wavelet-HHH	glcm	InverseVariance	0.280171202
wavelet-HHH	glcm	DifferenceVariance	6.290720925
wavelet-HHH	glcm	Idn	0.850349299
wavelet-HHH	glcm	Idm	0.247319706

wavelet-HHH	glcm	Correlation	-0.030688813
wavelet-HHH	glcm	Autocorrelation	90.40278051
wavelet-HHH	glcm	SumEntropy	3.726016298
wavelet-HHH	glcm	SumSquares	8.387315708
wavelet-HHH	glcm	ClusterProminence	919.7547474
wavelet-HHH	glcm	Imc2	0.928545154
wavelet-HHH	glcm	Imc1	-0.292658697
wavelet-HHH	glcm	DifferenceAverage	3.278641816
wavelet-HHH	glcm	Id	0.34020465
wavelet-HHH	glcm	ClusterTendency	16.31145104
wavelet-HHH	firstorder	InterquartileRange	92.13036794
wavelet-HHH	firstorder	Skewness	-0.23490678
wavelet-HHH	firstorder	Uniformity	0.108803285
wavelet-HHH	firstorder	Median	4.065513537
wavelet-HHH	firstorder	Energy	464484.0546
wavelet-HHH	firstorder	RobustMeanAbsoluteDeviation	40.22332373
wavelet-HHH	firstorder	MeanAbsoluteDeviation	56.99948216
wavelet-HHH	firstorder	TotalEnergy	12541069.47
wavelet-HHH	firstorder	Maximum	181.7941692
wavelet-HHH	firstorder	RootMeanSquared	71.44382675
wavelet-HHH	firstorder	90Percentile	81.9859238
wavelet-HHH	firstorder	Minimum	-205.7543774
wavelet-HHH	firstorder	Entropy	3.428686183
wavelet-HHH	firstorder	Range	387.5485466
wavelet-HHH	firstorder	Variance	5102.672982
wavelet-HHH	firstorder	10Percentile	-99.24059903
wavelet-HHH	firstorder	Kurtosis	2.946025461
wavelet-HHH	firstorder	Mean	-1.243944398
wavelet-HHH	glrlm	ShortRunLowGrayLevelEmphasis	0.026008257
wavelet-HHH	glrlm	GrayLevelVariance	8.223017294
wavelet-HHH	glrlm	LowGrayLevelRunEmphasis	0.026430592
wavelet-HHH	glrlm	GrayLevelNonUniformityNormalized	0.106007164
wavelet-HHH	glrlm	RunVariance	0.0498229
wavelet-HHH	glrlm	GrayLevelNonUniformity	9.183117096
wavelet-HHH	glrlm	LongRunEmphasis	1.155024495
wavelet-HHH	glrlm	ShortRunHighGrayLevelEmphasis	93.99295109
wavelet-HHH	glrlm	RunLengthNonUniformity	78.49151042
wavelet-HHH	glrlm	ShortRunEmphasis	0.962261377
wavelet-HHH	glrlm	LongRunHighGrayLevelEmphasis	113.3297402
wavelet-HHH	glrlm	RunPercentage	0.951817413
wavelet-HHH	glrlm	LongRunLowGrayLevelEmphasis	0.028153571
wavelet-HHH	glrlm	RunEntropy	3.655976579
wavelet-HHH	glrlm	HighGrayLevelRunEmphasis	97.76181481
wavelet-HHH	glrlm	RunLengthNonUniformityNormalized	0.905456241
wavelet-HHH	glszm	GrayLevelVariance	12.62200091
wavelet-HHH	glszm	ZoneVariance	5.251244907
wavelet-HHH	glszm	GrayLevelNonUniformityNormalized	0.083748302
wavelet-HHH	glszm	SizeZoneNonUniformityNormalized	0.596197374
wavelet-HHH	glszm	SizeZoneNonUniformity	28.0212766
wavelet-HHH	glszm	GrayLevelNonUniformity	3.936170213
wavelet-HHH	glszm	LargeAreaEmphasis	9
wavelet-HHH	glszm	SmallAreaHighGrayLevelEmphasis	77.73788416
wavelet-HHH	glszm	ZonePercentage	0.516483516

wavelet-HHH	glszm	LargeAreaLowGrayLevelEmphasis	0.121222399
wavelet-HHH	glszm	LargeAreaHighGrayLevelEmphasis	951.5957447
wavelet-HHH	glszm	HighGrayLevelZoneEmphasis	95.93617021
wavelet-HHH	glszm	SmallAreaEmphasis	0.790636492
wavelet-HHH	glszm	LowGrayLevelZoneEmphasis	0.039044864
wavelet-HHH	glszm	ZoneEntropy	4.35801798
wavelet-HHH	glszm	SmallAreaLowGrayLevelEmphasis	0.036210889
wavelet-HHH	ngtdm	Coarseness	0.061992864
wavelet-HHH	ngtdm	Complexity	173.6321889
wavelet-HHH	ngtdm	Strength	3.564501526
wavelet-HHH	ngtdm	Contrast	0.205917177
wavelet-HHH	ngtdm	Busyness	0.139138474
wavelet-HLL	gldm	GrayLevelVariance	52.38497766
wavelet-HLL	gldm	HighGrayLevelEmphasis	212.0879121
wavelet-HLL	gldm	DependenceEntropy	5.393683893
wavelet-HLL	gldm	DependenceNonUniformity	27.98901099
wavelet-HLL	gldm	GrayLevelNonUniformity	4.736263736
wavelet-HLL	gldm	SmallDependenceEmphasis	0.521083639
wavelet-HLL	gldm	SmallDependenceHighGrayLevelEmphasis	113.7966178
wavelet-HLL	gldm	DependenceNonUniformityNormalized	0.307571549
wavelet-HLL	gldm	LargeDependenceEmphasis	5.373626374
wavelet-HLL	gldm	LargeDependenceLowGrayLevelEmphasis	0.093316199
wavelet-HLL	gldm	DependenceVariance	1.329549571
wavelet-HLL	gldm	LargeDependenceHighGrayLevelEmphasis	1149.516484
wavelet-HLL	gldm	SmallDependenceLowGrayLevelEmphasis	0.022524902
wavelet-HLL	gldm	LowGrayLevelEmphasis	0.030716226
wavelet-HLL	glcm	JointAverage	12.1847551
wavelet-HLL	glcm	SumAverage	24.3695102
wavelet-HLL	glcm	JointEntropy	6.439603788
wavelet-HLL	glcm	ClusterShade	1464.389328
wavelet-HLL	glcm	MaximumProbability	0.028967206
wavelet-HLL	glcm	Idmn	0.942043531
wavelet-HLL	glcm	JointEnergy	0.012563327
wavelet-HLL	glcm	Contrast	61.32907854
wavelet-HLL	glcm	DifferenceEntropy	3.651219372
wavelet-HLL	glcm	InverseVariance	0.17877494
wavelet-HLL	glcm	DifferenceVariance	25.45505028
wavelet-HLL	glcm	Idn	0.848404676
wavelet-HLL	glcm	Idm	0.173995493
wavelet-HLL	glcm	Correlation	0.411241032
wavelet-HLL	glcm	Autocorrelation	170.2964787
wavelet-HLL	glcm	SumEntropy	4.683919534
wavelet-HLL	glcm	SumSquares	52.34869755
wavelet-HLL	glcm	ClusterProminence	61982.73889
wavelet-HLL	glcm	Imc2	0.994395753
wavelet-HLL	glcm	Imc1	-0.521324924
wavelet-HLL	glcm	DifferenceAverage	5.842725117
wavelet-HLL	glcm	Id	0.264883211
wavelet-HLL	glcm	ClusterTendency	148.0657117
wavelet-HLL	firstorder	InterquartileRange	253.013789
wavelet-HLL	firstorder	Skewness	0.642621641
wavelet-HLL	firstorder	Uniformity	0.052046854
wavelet-HLL	firstorder	Median	-121.1223076

wavelet-HLL	firstorder	Energy	3408401.353
wavelet-HLL	firstorder	RobustMeanAbsoluteDeviation	110.4985988
wavelet-HLL	firstorder	MeanAbsoluteDeviation	149.199557
wavelet-HLL	firstorder	TotalEnergy	92026836.54
wavelet-HLL	firstorder	Maximum	345.6994752
wavelet-HLL	firstorder	RootMeanSquared	193.5328394
wavelet-HLL	firstorder	90Percentile	217.0418214
wavelet-HLL	firstorder	Minimum	-354.1762696
wavelet-HLL	firstorder	Entropy	4.471812295
wavelet-HLL	firstorder	Range	699.8757448
wavelet-HLL	firstorder	Variance	32499.30999
wavelet-HLL	firstorder	10Percentile	-278.3128697
wavelet-HLL	firstorder	Kurtosis	2.425556052
wavelet-HLL	firstorder	Mean	-70.39637733
wavelet-HLL	glrlm	ShortRunLowGrayLevelEmphasis	0.030877872
wavelet-HLL	glrlm	GrayLevelVariance	52.28410655
wavelet-HLL	glrlm	LowGrayLevelRunEmphasis	0.031346715
wavelet-HLL	glrlm	GrayLevelNonUniformityNormalized	0.051267514
wavelet-HLL	glrlm	RunVariance	0.040595193
wavelet-HLL	glrlm	GrayLevelNonUniformity	4.484838494
wavelet-HLL	glrlm	LongRunEmphasis	1.124923505
wavelet-HLL	glrlm	ShortRunHighGrayLevelEmphasis	205.6453573
wavelet-HLL	glrlm	RunLengthNonUniformity	80.8888564
wavelet-HLL	glrlm	ShortRunEmphasis	0.969798884
wavelet-HLL	glrlm	LongRunHighGrayLevelEmphasis	238.5175076
wavelet-HLL	glrlm	RunPercentage	0.961115807
wavelet-HLL	glrlm	LongRunLowGrayLevelEmphasis	0.03324646
wavelet-HLL	glrlm	RunEntropy	4.603973901
wavelet-HLL	glrlm	HighGrayLevelRunEmphasis	212.0805638
wavelet-HLL	glrlm	RunLengthNonUniformityNormalized	0.923901342
wavelet-HLL	glszm	GrayLevelVariance	53.62446281
wavelet-HLL	glszm	ZoneVariance	1.680661157
wavelet-HLL	glszm	GrayLevelNonUniformityNormalized	0.045289256
wavelet-HLL	glszm	SizeZoneNonUniformityNormalized	0.509421488
wavelet-HLL	glszm	SizeZoneNonUniformity	28.01818182
wavelet-HLL	glszm	GrayLevelNonUniformity	2.490909091
wavelet-HLL	glszm	LargeAreaEmphasis	4.418181818
wavelet-HLL	glszm	SmallAreaHighGrayLevelEmphasis	166.318788
wavelet-HLL	glszm	ZonePercentage	0.604395604
wavelet-HLL	glszm	LargeAreaLowGrayLevelEmphasis	0.089078547
wavelet-HLL	glszm	LargeAreaHighGrayLevelEmphasis	888.8363636
wavelet-HLL	glszm	HighGrayLevelZoneEmphasis	219.8
wavelet-HLL	glszm	SmallAreaEmphasis	0.741754896
wavelet-HLL	glszm	LowGrayLevelZoneEmphasis	0.040144272
wavelet-HLL	glszm	ZoneEntropy	5.07191335
wavelet-HLL	glszm	SmallAreaLowGrayLevelEmphasis	0.035104693
wavelet-HLL	ngtdm	Coarseness	0.062181214
wavelet-HLL	ngtdm	Complexity	1172.421503
wavelet-HLL	ngtdm	Strength	18.42912927
wavelet-HLL	ngtdm	Contrast	0.599058434
wavelet-HLL	ngtdm	Busyness	0.065368256
wavelet-LHL	gldm	GrayLevelVariance	59.61188262
wavelet-LHL	gldm	HighGrayLevelEmphasis	945.1648352

wavelet-LHL	gldm	DependenceEntropy	5.526649448
wavelet-LHL	gldm	DependenceNonUniformity	29.50549451
wavelet-LHL	gldm	GrayLevelNonUniformity	4.384615385
wavelet-LHL	gldm	SmallDependenceEmphasis	0.500973748
wavelet-LHL	gldm	SmallDependenceHighGrayLevelEmphasis	504.6479518
wavelet-LHL	gldm	DependenceNonUniformityNormalized	0.324236203
wavelet-LHL	gldm	LargeDependenceEmphasis	4.516483516
wavelet-LHL	gldm	LargeDependenceLowGrayLevelEmphasis	0.017158498
wavelet-LHL	gldm	DependenceVariance	0.818258664
wavelet-LHL	gldm	LargeDependenceHighGrayLevelEmphasis	3892.769231
wavelet-LHL	gldm	SmallDependenceLowGrayLevelEmphasis	0.011590341
wavelet-LHL	gldm	LowGrayLevelEmphasis	0.012266632
wavelet-LHL	glcm	JointAverage	29.21864813
wavelet-LHL	glcm	SumAverage	58.43729626
wavelet-LHL	glcm	JointEntropy	6.465803121
wavelet-LHL	glcm	ClusterShade	744.1221502
wavelet-LHL	glcm	MaximumProbability	0.028315252
wavelet-LHL	glcm	Idmn	0.972796419
wavelet-LHL	glcm	JointEnergy	0.012301172
wavelet-LHL	glcm	Contrast	69.06474757
wavelet-LHL	glcm	DifferenceEntropy	3.785568046
wavelet-LHL	glcm	InverseVariance	0.142777529
wavelet-LHL	glcm	DifferenceVariance	25.70050284
wavelet-LHL	glcm	Idn	0.888641014
wavelet-LHL	glcm	Idm	0.146712414
wavelet-LHL	glcm	Correlation	0.289329878
wavelet-LHL	glcm	Autocorrelation	868.6401266
wavelet-LHL	glcm	SumEntropy	4.679892433
wavelet-LHL	glcm	SumSquares	49.26069414
wavelet-LHL	glcm	ClusterProminence	49931.35296
wavelet-LHL	glcm	Imc2	0.995851041
wavelet-LHL	glcm	Imc1	-0.543503384
wavelet-LHL	glcm	DifferenceAverage	6.466478722
wavelet-LHL	glcm	Id	0.238385925
wavelet-LHL	glcm	ClusterTendency	127.978029
wavelet-LHL	firstorder	InterquartileRange	238.3683414
wavelet-LHL	firstorder	Skewness	0.063470891
wavelet-LHL	firstorder	Uniformity	0.048182587
wavelet-LHL	firstorder	Median	-45.08504377
wavelet-LHL	firstorder	Energy	3444686.606
wavelet-LHL	firstorder	RobustMeanAbsoluteDeviation	106.3364201
wavelet-LHL	firstorder	MeanAbsoluteDeviation	152.0150034
wavelet-LHL	firstorder	TotalEnergy	93006538.36
wavelet-LHL	firstorder	Maximum	431.1899488
wavelet-LHL	firstorder	RootMeanSquared	194.5602708
wavelet-LHL	firstorder	90Percentile	273.1741792
wavelet-LHL	firstorder	Minimum	-731.6675433
wavelet-LHL	firstorder	Entropy	4.580780943
wavelet-LHL	firstorder	Range	1162.857492
wavelet-LHL	firstorder	Variance	37529.53927
wavelet-LHL	firstorder	10Percentile	-213.8815396
wavelet-LHL	firstorder	Kurtosis	4.061136915
wavelet-LHL	firstorder	Mean	-18.00443564

wavelet-LHL	glrlm	ShortRunLowGrayLevelEmphasis	0.012643795
wavelet-LHL	glrlm	GrayLevelVariance	60.34529422
wavelet-LHL	glrlm	LowGrayLevelRunEmphasis	0.012680924
wavelet-LHL	glrlm	GrayLevelNonUniformityNormalized	0.047708519
wavelet-LHL	glrlm	RunVariance	0.040783852
wavelet-LHL	glrlm	GrayLevelNonUniformity	4.188088059
wavelet-LHL	glrlm	LongRunEmphasis	1.11969078
wavelet-LHL	glrlm	ShortRunHighGrayLevelEmphasis	925.5963364
wavelet-LHL	glrlm	RunLengthNonUniformity	82.14035168
wavelet-LHL	glrlm	ShortRunEmphasis	0.973142356
wavelet-LHL	glrlm	LongRunHighGrayLevelEmphasis	1050.536286
wavelet-LHL	glrlm	RunPercentage	0.964497041
wavelet-LHL	glrlm	LongRunLowGrayLevelEmphasis	0.01284605
wavelet-LHL	glrlm	RunEntropy	4.694937027
wavelet-LHL	glrlm	HighGrayLevelRunEmphasis	948.6319788
wavelet-LHL	glrlm	RunLengthNonUniformityNormalized	0.933860207
wavelet-LHL	glszm	GrayLevelVariance	71.97959184
wavelet-LHL	glszm	ZoneVariance	1.091517857
wavelet-LHL	glszm	GrayLevelNonUniformityNormalized	0.041454082
wavelet-LHL	glszm	SizeZoneNonUniformityNormalized	0.453443878
wavelet-LHL	glszm	SizeZoneNonUniformity	25.39285714
wavelet-LHL	glszm	GrayLevelNonUniformity	2.321428571
wavelet-LHL	glszm	LargeAreaEmphasis	3.732142857
wavelet-LHL	glszm	SmallAreaHighGrayLevelEmphasis	714.3973264
wavelet-LHL	glszm	ZonePercentage	0.615384615
wavelet-LHL	glszm	LargeAreaLowGrayLevelEmphasis	0.022844648
wavelet-LHL	glszm	LargeAreaHighGrayLevelEmphasis	3264.892857
wavelet-LHL	glszm	HighGrayLevelZoneEmphasis	993.5357143
wavelet-LHL	glszm	SmallAreaEmphasis	0.695282738
wavelet-LHL	glszm	LowGrayLevelZoneEmphasis	0.019082804
wavelet-LHL	glszm	ZoneEntropy	5.436731931
wavelet-LHL	glszm	SmallAreaLowGrayLevelEmphasis	0.018675844
wavelet-LHL	ngtdm	Coarseness	0.067097517
wavelet-LHL	ngtdm	Complexity	1622.30336
wavelet-LHL	ngtdm	Strength	24.72207906
wavelet-LHL	ngtdm	Contrast	0.653378791
wavelet-LHL	ngtdm	Busyness	0.027743945
wavelet-LHH	gldm	GrayLevelVariance	29.80340539
wavelet-LHH	gldm	HighGrayLevelEmphasis	235.1428571
wavelet-LHH	gldm	DependenceEntropy	5.32884725
wavelet-LHH	gldm	DependenceNonUniformity	26.05494505
wavelet-LHH	gldm	GrayLevelNonUniformity	6.67032967
wavelet-LHH	gldm	SmallDependenceEmphasis	0.511736874
wavelet-LHH	gldm	SmallDependenceHighGrayLevelEmphasis	125.8463004
wavelet-LHH	gldm	DependenceNonUniformityNormalized	0.286318078
wavelet-LHH	gldm	LargeDependenceEmphasis	5.857142857
wavelet-LHH	gldm	LargeDependenceLowGrayLevelEmphasis	0.049915697
wavelet-LHH	gldm	DependenceVariance	1.451757034
wavelet-LHH	gldm	LargeDependenceHighGrayLevelEmphasis	1278.087912
wavelet-LHH	gldm	SmallDependenceLowGrayLevelEmphasis	0.018657226
wavelet-LHH	gldm	LowGrayLevelEmphasis	0.022048373
wavelet-LHH	glcm	JointAverage	14.26540654
wavelet-LHH	glcm	SumAverage	28.53081308

wavelet-LHH	glcm	JointEntropy	6.200714418
wavelet-LHH	glcm	ClusterShade	-25.26864322
wavelet-LHH	glcm	MaximumProbability	0.038883958
wavelet-LHH	glcm	Idmn	0.951369737
wavelet-LHH	glcm	JointEnergy	0.015667635
wavelet-LHH	glcm	Contrast	51.49864847
wavelet-LHH	glcm	DifferenceEntropy	3.469979644
wavelet-LHH	glcm	InverseVariance	0.238526692
wavelet-LHH	glcm	DifferenceVariance	23.7327617
wavelet-LHH	glcm	Idn	0.865425757
wavelet-LHH	glcm	Idm	0.212562262
wavelet-LHH	glcm	Correlation	0.030110959
wavelet-LHH	glcm	Autocorrelation	204.4306733
wavelet-LHH	glcm	SumEntropy	4.210895949
wavelet-LHH	glcm	SumSquares	26.60368404
wavelet-LHH	glcm	ClusterProminence	13885.81866
wavelet-LHH	glcm	Imc2	0.98719327
wavelet-LHH	glcm	Imc1	-0.459636012
wavelet-LHH	glcm	DifferenceAverage	5.120425034
wavelet-LHH	glcm	Id	0.298173478
wavelet-LHH	glcm	ClusterTendency	54.91608769
wavelet-LHH	firstorder	InterquartileRange	123.124496
wavelet-LHH	firstorder	Skewness	0.182219991
wavelet-LHH	firstorder	Uniformity	0.073300326
wavelet-LHH	firstorder	Median	-3.612598096
wavelet-LHH	firstorder	Energy	1709632.547
wavelet-LHH	firstorder	RobustMeanAbsoluteDeviation	56.02278478
wavelet-LHH	firstorder	MeanAbsoluteDeviation	97.09431068
wavelet-LHH	firstorder	TotalEnergy	46160078.78
wavelet-LHH	firstorder	Maximum	372.6351217
wavelet-LHH	firstorder	RootMeanSquared	137.0663009
wavelet-LHH	firstorder	90Percentile	166.7271248
wavelet-LHH	firstorder	Minimum	-340.8016736
wavelet-LHH	firstorder	Entropy	4.129380873
wavelet-LHH	firstorder	Range	713.4367953
wavelet-LHH	firstorder	Variance	18775.77497
wavelet-LHH	firstorder	10Percentile	-190.8116824
wavelet-LHH	firstorder	Kurtosis	3.851958426
wavelet-LHH	firstorder	Mean	-3.375779075
wavelet-LHH	glrlm	ShortRunLowGrayLevelEmphasis	0.022557693
wavelet-LHH	glrlm	GrayLevelVariance	30.73058493
wavelet-LHH	glrlm	LowGrayLevelRunEmphasis	0.022756942
wavelet-LHH	glrlm	GrayLevelNonUniformityNormalized	0.070779816
wavelet-LHH	glrlm	RunVariance	0.045670011
wavelet-LHH	glrlm	GrayLevelNonUniformity	6.170184545
wavelet-LHH	glrlm	LongRunEmphasis	1.137061076
wavelet-LHH	glrlm	ShortRunHighGrayLevelEmphasis	228.9026577
wavelet-LHH	glrlm	RunLengthNonUniformity	80.14541754
wavelet-LHH	glrlm	ShortRunEmphasis	0.96769982
wavelet-LHH	glrlm	LongRunHighGrayLevelEmphasis	265.7128217
wavelet-LHH	glrlm	RunPercentage	0.957734573
wavelet-LHH	glrlm	LongRunLowGrayLevelEmphasis	0.023592635
wavelet-LHH	glrlm	RunEntropy	4.326874969

wavelet-LHH	glrlm	HighGrayLevelRunEmphasis	235.9314114
wavelet-LHH	glrlm	RunLengthNonUniformityNormalized	0.918902646
wavelet-LHH	glszm	GrayLevelVariance	44.6212995
wavelet-LHH	glszm	ZoneVariance	4.051518647
wavelet-LHH	glszm	GrayLevelNonUniformityNormalized	0.048058439
wavelet-LHH	glszm	SizeZoneNonUniformityNormalized	0.574778931
wavelet-LHH	glszm	SizeZoneNonUniformity	29.31372549
wavelet-LHH	glszm	GrayLevelNonUniformity	2.450980392
wavelet-LHH	glszm	LargeAreaEmphasis	7.235294118
wavelet-LHH	glszm	SmallAreaHighGrayLevelEmphasis	195.2579316
wavelet-LHH	glszm	ZonePercentage	0.56043956
wavelet-LHH	glszm	LargeAreaLowGrayLevelEmphasis	0.068803045
wavelet-LHH	glszm	LargeAreaHighGrayLevelEmphasis	1558.666667
wavelet-LHH	glszm	HighGrayLevelZoneEmphasis	247.8235294
wavelet-LHH	glszm	SmallAreaEmphasis	0.782577614
wavelet-LHH	glszm	LowGrayLevelZoneEmphasis	0.034268816
wavelet-LHH	glszm	ZoneEntropy	4.99095696
wavelet-LHH	glszm	SmallAreaLowGrayLevelEmphasis	0.032341778
wavelet-LHH	ngtdm	Coarseness	0.067857196
wavelet-LHH	ngtdm	Complexity	899.5451471
wavelet-LHH	ngtdm	Strength	11.87785996
wavelet-LHH	ngtdm	Contrast	0.413829908
wavelet-LHH	ngtdm	Busyness	0.043048651
wavelet-LLH	gldm	GrayLevelVariance	94.74846033
wavelet-LLH	gldm	HighGrayLevelEmphasis	988.8241758
wavelet-LLH	gldm	DependenceEntropy	5.485601783
wavelet-LLH	gldm	DependenceNonUniformity	31.87912088
wavelet-LLH	gldm	GrayLevelNonUniformity	3.879120879
wavelet-LLH	gldm	SmallDependenceEmphasis	0.592014652
wavelet-LLH	gldm	SmallDependenceHighGrayLevelEmphasis	666.6298657
wavelet-LLH	gldm	DependenceNonUniformityNormalized	0.35032001
wavelet-LLH	gldm	LargeDependenceEmphasis	4.428571429
wavelet-LLH	gldm	LargeDependenceLowGrayLevelEmphasis	0.017920229
wavelet-LLH	gldm	DependenceVariance	1.060741456
wavelet-LLH	gldm	LargeDependenceHighGrayLevelEmphasis	3392.472527
wavelet-LLH	gldm	SmallDependenceLowGrayLevelEmphasis	0.011789933
wavelet-LLH	gldm	LowGrayLevelEmphasis	0.012430314
wavelet-LLH	glcm	JointAverage	30.0303913
wavelet-LLH	glcm	SumAverage	60.06078261
wavelet-LLH	glcm	JointEntropy	6.581252005
wavelet-LLH	glcm	ClusterShade	1601.847528
wavelet-LLH	glcm	MaximumProbability	0.025540807
wavelet-LLH	glcm	Idmn	0.959255414
wavelet-LLH	glcm	JointEnergy	0.011074081
wavelet-LLH	glcm	Contrast	131.375612
wavelet-LLH	glcm	DifferenceEntropy	4.09221996
wavelet-LLH	glcm	InverseVariance	0.133255058
wavelet-LLH	glcm	DifferenceVariance	54.12585665
wavelet-LLH	glcm	Idn	0.871803476
wavelet-LLH	glcm	Idm	0.136015721
wavelet-LLH	glcm	Correlation	0.241953724
wavelet-LLH	glcm	Autocorrelation	923.1592197
wavelet-LLH	glcm	SumEntropy	4.943200678

wavelet-LLH	glcm	SumSquares	86.93803856
wavelet-LLH	glcm	ClusterProminence	124874.9009
wavelet-LLH	glcm	Imc2	0.998215021
wavelet-LLH	glcm	Imc1	-0.601974805
wavelet-LLH	glcm	DifferenceAverage	8.485076792
wavelet-LLH	glcm	Id	0.219503196
wavelet-LLH	glcm	ClusterTendency	216.3765423
wavelet-LLH	firstorder	InterquartileRange	343.6472918
wavelet-LLH	firstorder	Skewness	0.239930673
wavelet-LLH	firstorder	Uniformity	0.042627702
wavelet-LLH	firstorder	Median	-47.32414491
wavelet-LLH	firstorder	Energy	5323763.658
wavelet-LLH	firstorder	RobustMeanAbsoluteDeviation	144.7405651
wavelet-LLH	firstorder	MeanAbsoluteDeviation	198.7552207
wavelet-LLH	firstorder	TotalEnergy	143741618.8
wavelet-LLH	firstorder	Maximum	552.3308945
wavelet-LLH	firstorder	RootMeanSquared	241.8737219
wavelet-LLH	firstorder	90Percentile	371.4121283
wavelet-LLH	firstorder	Minimum	-710.0906371
wavelet-LLH	firstorder	Entropy	4.828310162
wavelet-LLH	firstorder	Range	1262.421532
wavelet-LLH	firstorder	Variance	58397.64218
wavelet-LLH	firstorder	10Percentile	-269.313995
wavelet-LLH	firstorder	Kurtosis	2.852808536
wavelet-LLH	firstorder	Mean	10.25939387
wavelet-LLH	glrlm	ShortRunLowGrayLevelEmphasis	0.01275379
wavelet-LLH	glrlm	GrayLevelVariance	96.40813556
wavelet-LLH	glrlm	LowGrayLevelRunEmphasis	0.012792878
wavelet-LLH	glrlm	GrayLevelNonUniformityNormalized	0.041355099
wavelet-LLH	glrlm	RunVariance	0.033806596
wavelet-LLH	glrlm	GrayLevelNonUniformity	3.643035543
wavelet-LLH	glrlm	LongRunEmphasis	1.102083935
wavelet-LLH	glrlm	ShortRunHighGrayLevelEmphasis	979.4201938
wavelet-LLH	glrlm	RunLengthNonUniformity	82.6150035
wavelet-LLH	glrlm	ShortRunEmphasis	0.975450267
wavelet-LLH	glrlm	LongRunHighGrayLevelEmphasis	1072.033493
wavelet-LLH	glrlm	RunPercentage	0.967878276
wavelet-LLH	glrlm	LongRunLowGrayLevelEmphasis	0.012954977
wavelet-LLH	glrlm	RunEntropy	4.949811899
wavelet-LLH	glrlm	HighGrayLevelRunEmphasis	997.4176011
wavelet-LLH	glrlm	RunLengthNonUniformityNormalized	0.937512364
wavelet-LLH	glszm	GrayLevelVariance	116.5163889
wavelet-LLH	glszm	ZoneVariance	1.849722222
wavelet-LLH	glszm	GrayLevelNonUniformityNormalized	0.033333333
wavelet-LLH	glszm	SizeZoneNonUniformityNormalized	0.611666667
wavelet-LLH	glszm	SizeZoneNonUniformity	36.7
wavelet-LLH	glszm	GrayLevelNonUniformity	2
wavelet-LLH	glszm	LargeAreaEmphasis	4.15
wavelet-LLH	glszm	SmallAreaHighGrayLevelEmphasis	937.9510231
wavelet-LLH	glszm	ZonePercentage	0.659340659
wavelet-LLH	glszm	LargeAreaLowGrayLevelEmphasis	0.02308672
wavelet-LLH	glszm	LargeAreaHighGrayLevelEmphasis	3280.65
wavelet-LLH	glszm	HighGrayLevelZoneEmphasis	1107.716667

wavelet-LLH	glszm	SmallAreaEmphasis	0.808395576
wavelet-LLH	glszm	LowGrayLevelZoneEmphasis	0.018037683
wavelet-LLH	glszm	ZoneEntropy	5.315061012
wavelet-LLH	glszm	SmallAreaLowGrayLevelEmphasis	0.017741444
wavelet-LLH	ngtdm	Coarseness	0.054198699
wavelet-LLH	ngtdm	Complexity	3323.396314
wavelet-LLH	ngtdm	Strength	29.89244793
wavelet-LLH	ngtdm	Contrast	0.99522155
wavelet-LLH	ngtdm	Busyness	0.026852084
wavelet-LLL	gldm	GrayLevelVariance	370.8619732
wavelet-LLL	gldm	HighGrayLevelEmphasis	2163.593407
wavelet-LLL	gldm	DependenceEntropy	5.784998624
wavelet-LLL	gldm	DependenceNonUniformity	51.43956044
wavelet-LLL	gldm	GrayLevelNonUniformity	2.054945055
wavelet-LLL	gldm	SmallDependenceEmphasis	0.772893773
wavelet-LLL	gldm	SmallDependenceHighGrayLevelEmphasis	1638.313187
wavelet-LLL	gldm	DependenceNonUniformityNormalized	0.565269895
wavelet-LLL	gldm	LargeDependenceEmphasis	2.054945055
wavelet-LLL	gldm	LargeDependenceLowGrayLevelEmphasis	0.015300442
wavelet-LLL	gldm	DependenceVariance	0.286921869
wavelet-LLL	gldm	LargeDependenceHighGrayLevelEmphasis	5156.142857
wavelet-LLL	gldm	SmallDependenceLowGrayLevelEmphasis	0.014059915
wavelet-LLL	gldm	LowGrayLevelEmphasis	0.014303204
wavelet-LLL	glcm	JointAverage	47.59604772
wavelet-LLL	glcm	SumAverage	95.19209543
wavelet-LLL	glcm	JointEntropy	6.730238185
wavelet-LLL	glcm	ClusterShade	-7382.254355
wavelet-LLL	glcm	MaximumProbability	0.014755929
wavelet-LLL	glcm	Idmn	0.934815985
wavelet-LLL	glcm	JointEnergy	0.009593411
wavelet-LLL	glcm	Contrast	479.264849
wavelet-LLL	glcm	DifferenceEntropy	4.696801323
wavelet-LLL	glcm	InverseVariance	0.04937718
wavelet-LLL	glcm	DifferenceVariance	151.9507282
wavelet-LLL	glcm	Idn	0.829643554
wavelet-LLL	glcm	Idm	0.05362505
wavelet-LLL	glcm	Correlation	0.350173721
wavelet-LLL	glcm	Autocorrelation	2399.181169
wavelet-LLL	glcm	SumEntropy	5.299291947
wavelet-LLL	glcm	SumSquares	372.6511325
wavelet-LLL	glcm	ClusterProminence	2624132.502
wavelet-LLL	glcm	Imc2	0.999902071
wavelet-LLL	glcm	Imc1	-0.777367691
wavelet-LLL	glcm	DifferenceAverage	17.80131804
wavelet-LLL	glcm	Id	0.117474592
wavelet-LLL	glcm	ClusterTendency	1011.339681
wavelet-LLL	firstorder	InterquartileRange	602.5832511
wavelet-LLL	firstorder	Skewness	-0.008024673
wavelet-LLL	firstorder	Uniformity	0.022581814
wavelet-LLL	firstorder	Median	-701.8469864
wavelet-LLL	firstorder	Energy	60048569.06
wavelet-LLL	firstorder	RobustMeanAbsoluteDeviation	277.3768239
wavelet-LLL	firstorder	MeanAbsoluteDeviation	388.4106668

wavelet-LLL	firstorder	TotalEnergy	1621311364
wavelet-LLL	firstorder	Maximum	252.68055
wavelet-LLL	firstorder	RootMeanSquared	812.3265262
wavelet-LLL	firstorder	90Percentile	58.85361775
wavelet-LLL	firstorder	Minimum	-1676.801628
wavelet-LLL	firstorder	Entropy	5.633631178
wavelet-LLL	firstorder	Range	1929.482178
wavelet-LLL	firstorder	Variance	231049.2366
wavelet-LLL	firstorder	10Percentile	-1278.753356
wavelet-LLL	firstorder	Kurtosis	2.424889772
wavelet-LLL	firstorder	Mean	-654.847424
wavelet-LLL	glrlm	ShortRunLowGrayLevelEmphasis	0.014467875
wavelet-LLL	glrlm	GrayLevelVariance	369.8892396
wavelet-LLL	glrlm	LowGrayLevelRunEmphasis	0.014477702
wavelet-LLL	glrlm	GrayLevelNonUniformityNormalized	0.022577077
wavelet-LLL	glrlm	RunVariance	0.012660374
wavelet-LLL	glrlm	GrayLevelNonUniformity	2.028519895
wavelet-LLL	glrlm	LongRunEmphasis	1.039347907
wavelet-LLL	glrlm	ShortRunHighGrayLevelEmphasis	2131.983801
wavelet-LLL	glrlm	RunLengthNonUniformity	87.6178756
wavelet-LLL	glrlm	ShortRunEmphasis	0.990163023
wavelet-LLL	glrlm	LongRunHighGrayLevelEmphasis	2260.527865
wavelet-LLL	glrlm	RunPercentage	0.987320372
wavelet-LLL	glrlm	LongRunLowGrayLevelEmphasis	0.014517009
wavelet-LLL	glrlm	RunEntropy	5.649119869
wavelet-LLL	glrlm	HighGrayLevelRunEmphasis	2157.692614
wavelet-LLL	glrlm	RunLengthNonUniformityNormalized	0.974679253
wavelet-LLL	glszm	GrayLevelVariance	368.0978243
wavelet-LLL	glszm	ZoneVariance	0.174734357
wavelet-LLL	glszm	GrayLevelNonUniformityNormalized	0.022432113
wavelet-LLL	glszm	SizeZoneNonUniformityNormalized	0.715297689
wavelet-LLL	glszm	SizeZoneNonUniformity	55.07792208
wavelet-LLL	glszm	GrayLevelNonUniformity	1.727272727
wavelet-LLL	glszm	LargeAreaEmphasis	1.571428571
wavelet-LLL	glszm	SmallAreaHighGrayLevelEmphasis	1850.282468
wavelet-LLL	glszm	ZonePercentage	0.846153846
wavelet-LLL	glszm	LargeAreaLowGrayLevelEmphasis	0.017292369
wavelet-LLL	glszm	LargeAreaHighGrayLevelEmphasis	3577.831169
wavelet-LLL	glszm	HighGrayLevelZoneEmphasis	2125.558442
wavelet-LLL	glszm	SmallAreaEmphasis	0.871572872
wavelet-LLL	glszm	LowGrayLevelZoneEmphasis	0.01671163
wavelet-LLL	glszm	ZoneEntropy	5.786013164
wavelet-LLL	glszm	SmallAreaLowGrayLevelEmphasis	0.016568817
wavelet-LLL	ngtdm	Coarseness	0.048279664
wavelet-LLL	ngtdm	Complexity	17673.38356
wavelet-LLL	ngtdm	Strength	90.53037348
wavelet-LLL	ngtdm	Contrast	2.785263213
wavelet-LLL	ngtdm	Busyness	0.01127371
wavelet-HHL	gldm	GrayLevelVariance	17.12884917
wavelet-HHL	gldm	HighGrayLevelEmphasis	127.7252747
wavelet-HHL	gldm	DependenceEntropy	5.069684514
wavelet-HHL	gldm	DependenceNonUniformity	27.35164835
wavelet-HHL	gldm	GrayLevelNonUniformity	7.285714286

wavelet-HHL	gldm	SmallDependenceEmphasis	0.425518926
wavelet-HHL	gldm	SmallDependenceHighGrayLevelEmphasis	61.93017399
wavelet-HHL	gldm	DependenceNonUniformityNormalized	0.300567564
wavelet-HHL	gldm	LargeDependenceEmphasis	5.263736264
wavelet-HHL	gldm	LargeDependenceLowGrayLevelEmphasis	0.078926832
wavelet-HHL	gldm	DependenceVariance	0.858350441
wavelet-HHL	gldm	LargeDependenceHighGrayLevelEmphasis	598.4285714
wavelet-HHL	gldm	SmallDependenceLowGrayLevelEmphasis	0.016935166
wavelet-HHL	gldm	LowGrayLevelEmphasis	0.024386243
wavelet-HHL	glcm	JointAverage	10.18638536
wavelet-HHL	glcm	SumAverage	20.37277072
wavelet-HHL	glcm	JointEntropy	6.127922774
wavelet-HHL	glcm	ClusterShade	99.57371016
wavelet-HHL	glcm	MaximumProbability	0.036778585
wavelet-HHL	glcm	Idmn	0.959152594
wavelet-HHL	glcm	JointEnergy	0.016325296
wavelet-HHL	glcm	Contrast	29.40555924
wavelet-HHL	glcm	DifferenceEntropy	3.295668461
wavelet-HHL	glcm	InverseVariance	0.212923306
wavelet-HHL	glcm	DifferenceVariance	10.73824076
wavelet-HHL	glcm	Idn	0.865129435
wavelet-HHL	glcm	Idm	0.199993309
wavelet-HHL	glcm	Correlation	-0.012940496
wavelet-HHL	glcm	Autocorrelation	103.7824767
wavelet-HHL	glcm	SumEntropy	3.977060869
wavelet-HHL	glcm	SumSquares	14.68120265
wavelet-HHL	glcm	ClusterProminence	3596.372237
wavelet-HHL	glcm	Imc2	0.968772168
wavelet-HHL	glcm	Imc1	-0.376449311
wavelet-HHL	glcm	DifferenceAverage	4.243560593
wavelet-HHL	glcm	Id	0.296960883
wavelet-HHL	glcm	ClusterTendency	29.31925138
wavelet-HHL	firstorder	InterquartileRange	129.6160897
wavelet-HHL	firstorder	Skewness	0.862236508
wavelet-HHL	firstorder	Uniformity	0.080062794
wavelet-HHL	firstorder	Median	14.92774315
wavelet-HHL	firstorder	Energy	1009093.658
wavelet-HHL	firstorder	RobustMeanAbsoluteDeviation	53.67534023
wavelet-HHL	firstorder	MeanAbsoluteDeviation	78.91276145
wavelet-HHL	firstorder	TotalEnergy	27245528.77
wavelet-HHL	firstorder	Maximum	384.6854079
wavelet-HHL	firstorder	RootMeanSquared	105.3040422
wavelet-HHL	firstorder	90Percentile	168.5060036
wavelet-HHL	firstorder	Minimum	-212.8680804
wavelet-HHL	firstorder	Entropy	3.858599616
wavelet-HHL	firstorder	Range	597.5534883
wavelet-HHL	firstorder	Variance	10451.2583
wavelet-HHL	firstorder	10Percentile	-78.4321026
wavelet-HHL	firstorder	Kurtosis	4.353870259
wavelet-HHL	firstorder	Mean	25.25238596
wavelet-HHL	glrlm	ShortRunLowGrayLevelEmphasis	0.024473104
wavelet-HHL	glrlm	GrayLevelVariance	17.50391264
wavelet-HHL	glrlm	LowGrayLevelRunEmphasis	0.024904612

wavelet-HHL	glrlm	GrayLevelNonUniformityNormalized	0.079352751
wavelet-HHL	glrlm	RunVariance	0.043825129
wavelet-HHL	glrlm	GrayLevelNonUniformity	6.916105245
wavelet-HHL	glrlm	LongRunEmphasis	1.13696021
wavelet-HHL	glrlm	ShortRunHighGrayLevelEmphasis	124.7231782
wavelet-HHL	glrlm	RunLengthNonUniformity	80.10200227
wavelet-HHL	glrlm	ShortRunEmphasis	0.966753786
wavelet-HHL	glrlm	LongRunHighGrayLevelEmphasis	143.8357956
wavelet-HHL	glrlm	RunPercentage	0.957734573
wavelet-HHL	glrlm	LongRunLowGrayLevelEmphasis	0.026658249
wavelet-HHL	glrlm	RunEntropy	4.032316009
wavelet-HHL	glrlm	HighGrayLevelRunEmphasis	128.4312115
wavelet-HHL	glrlm	RunLengthNonUniformityNormalized	0.917466853
wavelet-HHL	glszm	GrayLevelVariance	25.00226347
wavelet-HHL	glszm	ZoneVariance	2.400181077
wavelet-HHL	glszm	GrayLevelNonUniformityNormalized	0.06926211
wavelet-HHL	glszm	SizeZoneNonUniformityNormalized	0.387958352
wavelet-HHL	glszm	SizeZoneNonUniformity	18.23404255
wavelet-HHL	glszm	GrayLevelNonUniformity	3.255319149
wavelet-HHL	glszm	LargeAreaEmphasis	6.14893617
wavelet-HHL	glszm	SmallAreaHighGrayLevelEmphasis	97.57545656
wavelet-HHL	glszm	ZonePercentage	0.516483516
wavelet-HHL	glszm	LargeAreaLowGrayLevelEmphasis	0.096339964
wavelet-HHL	glszm	LargeAreaHighGrayLevelEmphasis	750.7446809
wavelet-HHL	glszm	HighGrayLevelZoneEmphasis	137.7234043
wavelet-HHL	glszm	SmallAreaEmphasis	0.642722928
wavelet-HHL	glszm	LowGrayLevelZoneEmphasis	0.035574054
wavelet-HHL	glszm	ZoneEntropy	4.93502682
wavelet-HHL	glszm	SmallAreaLowGrayLevelEmphasis	0.030592069
wavelet-HHL	ngtdm	Coarseness	0.055738402
wavelet-HHL	ngtdm	Complexity	489.5513819
wavelet-HHL	ngtdm	Strength	7.940726922
wavelet-HHL	ngtdm	Contrast	0.325769535
wavelet-HHL	ngtdm	Busyness	0.119309167
wavelet-HLH	gldm	GrayLevelVariance	17.3674677
wavelet-HLH	gldm	HighGrayLevelEmphasis	203.9450549
wavelet-HLH	gldm	DependenceEntropy	5.205558448
wavelet-HLH	gldm	DependenceNonUniformity	23.83516484
wavelet-HLH	gldm	GrayLevelNonUniformity	7.285714286
wavelet-HLH	gldm	SmallDependenceEmphasis	0.414151404
wavelet-HLH	gldm	SmallDependenceHighGrayLevelEmphasis	85.69677656
wavelet-HLH	gldm	DependenceNonUniformityNormalized	0.261924888
wavelet-HLH	gldm	LargeDependenceEmphasis	6.494505495
wavelet-HLH	gldm	LargeDependenceLowGrayLevelEmphasis	0.05275008
wavelet-HLH	gldm	DependenceVariance	1.320130419
wavelet-HLH	gldm	LargeDependenceHighGrayLevelEmphasis	1324.164835
wavelet-HLH	gldm	SmallDependenceLowGrayLevelEmphasis	0.015202399
wavelet-HLH	gldm	LowGrayLevelEmphasis	0.019231579
wavelet-HLH	glcm	JointAverage	13.5791124
wavelet-HLH	glcm	SumAverage	27.1582248
wavelet-HLH	glcm	JointEntropy	6.260051114
wavelet-HLH	glcm	ClusterShade	-99.07517196
wavelet-HLH	glcm	MaximumProbability	0.035668371

wavelet-HLH	glcm	Idmn	0.934637666
wavelet-HLH	glcm	JointEnergy	0.014819338
wavelet-HLH	glcm	Contrast	39.49628068
wavelet-HLH	glcm	DifferenceEntropy	3.435703218
wavelet-HLH	glcm	InverseVariance	0.206629531
wavelet-HLH	glcm	DifferenceVariance	15.09561928
wavelet-HLH	glcm	Idn	0.837227292
wavelet-HLH	glcm	Idm	0.203755671
wavelet-HLH	glcm	Correlation	-0.029431356
wavelet-HLH	glcm	Autocorrelation	184.0229254
wavelet-HLH	glcm	SumEntropy	4.189144636
wavelet-HLH	glcm	SumSquares	19.28073914
wavelet-HLH	glcm	ClusterProminence	4954.065734
wavelet-HLH	glcm	Imc2	0.978918285
wavelet-HLH	glcm	Imc1	-0.408402824
wavelet-HLH	glcm	DifferenceAverage	4.811376194
wavelet-HLH	glcm	Id	0.294335275
wavelet-HLH	glcm	ClusterTendency	37.62667588
wavelet-HLH	firstorder	InterquartileRange	135.5256315
wavelet-HLH	firstorder	Skewness	-0.539840664
wavelet-HLH	firstorder	Uniformity	0.080062794
wavelet-HLH	firstorder	Median	4.315141353
wavelet-HLH	firstorder	Energy	972830.279
wavelet-HLH	firstorder	RobustMeanAbsoluteDeviation	60.05035508
wavelet-HLH	firstorder	MeanAbsoluteDeviation	83.33924582
wavelet-HLH	firstorder	TotalEnergy	26266417.53
wavelet-HLH	firstorder	Maximum	202.3423312
wavelet-HLH	firstorder	RootMeanSquared	103.3945967
wavelet-HLH	firstorder	90Percentile	129.6317447
wavelet-HLH	firstorder	Minimum	-314.5413561
wavelet-HLH	firstorder	Entropy	3.917004811
wavelet-HLH	firstorder	Range	516.8836873
wavelet-HLH	firstorder	Variance	10675.28896
wavelet-HLH	firstorder	10Percentile	-130.4818242
wavelet-HLH	firstorder	Kurtosis	3.149964336
wavelet-HLH	firstorder	Mean	3.892771478
wavelet-HLH	glrlm	ShortRunLowGrayLevelEmphasis	0.019661871
wavelet-HLH	glrlm	GrayLevelVariance	17.79821466
wavelet-HLH	glrlm	LowGrayLevelRunEmphasis	0.019905673
wavelet-HLH	glrlm	GrayLevelNonUniformityNormalized	0.078093786
wavelet-HLH	glrlm	RunVariance	0.050617496
wavelet-HLH	glrlm	GrayLevelNonUniformity	6.758697116
wavelet-HLH	glrlm	LongRunEmphasis	1.158502584
wavelet-HLH	glrlm	ShortRunHighGrayLevelEmphasis	196.0629693
wavelet-HLH	glrlm	RunLengthNonUniformity	78.31789914
wavelet-HLH	glrlm	ShortRunEmphasis	0.961391855
wavelet-HLH	glrlm	LongRunHighGrayLevelEmphasis	236.1320536
wavelet-HLH	glrlm	RunPercentage	0.950972105
wavelet-HLH	glrlm	LongRunLowGrayLevelEmphasis	0.020909143
wavelet-HLH	glrlm	RunEntropy	4.130652123
wavelet-HLH	glrlm	HighGrayLevelRunEmphasis	203.95957
wavelet-HLH	glrlm	RunLengthNonUniformityNormalized	0.90386705
wavelet-HLH	glszm	GrayLevelVariance	24.66162571

wavelet-HLH	glszm	ZoneVariance	3.325614367
wavelet-HLH	glszm	GrayLevelNonUniformityNormalized	0.063327032
wavelet-HLH	glszm	SizeZoneNonUniformityNormalized	0.402646503
wavelet-HLH	glszm	SizeZoneNonUniformity	18.52173913
wavelet-HLH	glszm	GrayLevelNonUniformity	2.913043478
wavelet-HLH	glszm	LargeAreaEmphasis	7.239130435
wavelet-HLH	glszm	SmallAreaHighGrayLevelEmphasis	135.8857126
wavelet-HLH	glszm	ZonePercentage	0.505494505
wavelet-HLH	glszm	LargeAreaLowGrayLevelEmphasis	0.06872353
wavelet-HLH	glszm	LargeAreaHighGrayLevelEmphasis	1502.586957
wavelet-HLH	glszm	HighGrayLevelZoneEmphasis	202.826087
wavelet-HLH	glszm	SmallAreaEmphasis	0.654621075
wavelet-HLH	glszm	LowGrayLevelZoneEmphasis	0.031658559
wavelet-HLH	glszm	ZoneEntropy	4.882045108
wavelet-HLH	glszm	SmallAreaLowGrayLevelEmphasis	0.028916857
wavelet-HLH	ngtdm	Coarseness	0.050924059
wavelet-HLH	ngtdm	Complexity	546.0622701
wavelet-HLH	ngtdm	Strength	6.807111556
wavelet-HLH	ngtdm	Contrast	0.326465363
wavelet-HLH	ngtdm	Busyness	0.075546402

Image type	Feature Class	Feature Name	22: RTSTRUCT: C99-DNU_segment_PTVfinal
general	info	BoundingBox	(0, 0, 1, 6, 6, 9)
general	info	EnabledImageTypes	{u'Original': {}, u'Wavelet': {}}
general	info	GeneralSettings	{'distances': [1], 'additionalInfo': True, 'force2D': False, 'interpolator': 'sitkBSpline', 'resampledPixelSpacing': [3.0], 'label': 1, 'normalizeScale': 1, 'normalize': False, 'force2Ddimension': 0, 'removeOutliers': None, 'minimumROISize': None, u'binWidth': 25.0, 'minimumROIDimensions': 1, u'correctMask': True, 'preCrop': False, u'symmetricalGLCM': True, 'resegmentRange': None, 'padDistance': 5}
general	info	ImageHash	555703461e58463dd16fd03b3d4ce9c3dd0a688e
general	info	ImageSpacing	(0.9765624999999998, 0.9765624999999998, 1.999999999999999)
general	info	MaskHash	e3f27d17a56970fa75baf44bd5b89045dc242dcf
general	info	NumpyVersion	1.13.1
general	info	PyWaveletVersion	0.5.2
general	info	SimpleITKVersion	1.1.0
general	info	Version	1.3.0.post102+g1b0eb74
general	info	VolumeNum	1
general	info	VoxelNum	136
original	shape	Maximum3DDiameter	29.69848481
original	shape	Maximum2DDiameterSlice	15.29705854
original	shape	Sphericity	0.787650513
original	shape	MinorAxis	15.01009793
original	shape	Elongation	0.486190879
original	shape	SurfaceVolumeRatio	0.397969394
original	shape	Volume	3672
original	shape	MajorAxis	30.87284968
original	shape	SurfaceArea	1461.343613
original	shape	Flatness	0.399954236
original	shape	LeastAxis	12.34772699
original	shape	Maximum2DDiameterColumn	25.63201124
original	shape	Maximum2DDiameterRow	28.3019434
original	gldm	GrayLevelVariance	23.316609
original	gldm	HighGrayLevelEmphasis	120.3970588
original	gldm	DependenceEntropy	5.588289522
original	gldm	DependenceNonUniformity	37.01470588
original	gldm	GrayLevelNonUniformity	8.485294118
original	gldm	SmallDependenceEmphasis	0.412826797
original	gldm	SmallDependenceHighGrayLevelEmphasis	42.06948121
original	gldm	DependenceNonUniformityNormalized	0.272166955
original	gldm	LargeDependenceEmphasis	6.352941176
original	gldm	LargeDependenceLowGrayLevelEmphasis	0.223356544
original	gldm	DependenceVariance	1.290441176
original	gldm	LargeDependenceHighGrayLevelEmphasis	916.9558824
original	gldm	SmallDependenceLowGrayLevelEmphasis	0.031272021
original	gldm	LowGrayLevelEmphasis	0.057003501
original	glcm	JointAverage	10.95267373
original	glcm	SumAverage	21.90534747

original	glcm	JointEntropy	6.733525365
original	glcm	ClusterShade	-65.70042926
original	glcm	MaximumProbability	0.027808468
original	glcm	Idmn	0.94747867
original	glcm	JointEnergy	0.010783607
original	glcm	Contrast	24.66081445
original	glcm	DifferenceEntropy	3.25135542
original	glcm	InverseVariance	0.239312872
original	glcm	DifferenceVariance	8.621505773
original	glcm	Idn	0.848207362
original	glcm	Idm	0.220505212
original	glcm	Correlation	0.433559116
original	glcm	Autocorrelation	129.5107022
original	glcm	SumEntropy	4.634078347
original	glcm	SumSquares	21.79280423
original	glcm	ClusterProminence	8659.663224
original	glcm	Imc2	0.969545786
original	glcm	Imc1	-0.350221683
original	glcm	DifferenceAverage	3.928473542
original	glcm	Id	0.313607581
original	glcm	ClusterTendency	62.51040246
original	firstorder	InterquartileRange	210.75
original	firstorder	Skewness	-0.000404194
original	firstorder	Uniformity	0.062391869
original	firstorder	Median	-514.5
original	firstorder	Energy	38340302
original	firstorder	RobustMeanAbsoluteDeviation	79.81504132
original	firstorder	MeanAbsoluteDeviation	101.9878893
original	firstorder	TotalEnergy	1035188154
original	firstorder	Maximum	-256
original	firstorder	RootMeanSquared	530.9557282
original	firstorder	90Percentile	-368
original	firstorder	Minimum	-742
original	firstorder	Entropy	4.127973134
original	firstorder	Range	486
original	firstorder	Variance	14533.74221
original	firstorder	10Percentile	-681
original	firstorder	Kurtosis	2.056247162
original	firstorder	Mean	-517.0882353
original	glrlm	ShortRunLowGrayLevelEmphasis	0.056894258
original	glrlm	GrayLevelVariance	23.31887217
original	glrlm	LowGrayLevelRunEmphasis	0.058076052
original	glrlm	GrayLevelNonUniformityNormalized	0.061976597
original	glrlm	RunVariance	0.053974647
original	glrlm	GrayLevelNonUniformity	8.02166953
original	glrlm	LongRunEmphasis	1.159970385
original	glrlm	ShortRunHighGrayLevelEmphasis	113.8391707
original	glrlm	RunLengthNonUniformity	117.8518299
original	glrlm	ShortRunEmphasis	0.963427779
original	glrlm	LongRunHighGrayLevelEmphasis	141.4981246
original	glrlm	RunPercentage	0.951923077
original	glrlm	LongRunLowGrayLevelEmphasis	0.064299755
original	glrlm	RunEntropy	4.315108409
original	glrlm	HighGrayLevelRunEmphasis	119.2462196
original	glrlm	RunLengthNonUniformityNormalized	0.909144964

original	glszm	GrayLevelVariance	24.13213668
original	glszm	ZoneVariance	3.588235294
original	glszm	GrayLevelNonUniformityNormalized	0.061851211
original	glszm	SizeZoneNonUniformityNormalized	0.39532872
original	glszm	SizeZoneNonUniformity	26.88235294
original	glszm	GrayLevelNonUniformity	4.205882353
original	glszm	LargeAreaEmphasis	7.588235294
original	glszm	SmallAreaHighGrayLevelEmphasis	62.81799897
original	glszm	ZonePercentage	0.5
original	glszm	LargeAreaLowGrayLevelEmphasis	0.247457245
original	glszm	LargeAreaHighGrayLevelEmphasis	1093.058824
original	glszm	HighGrayLevelZoneEmphasis	105.3970588
original	glszm	SmallAreaEmphasis	0.650263692
original	glszm	LowGrayLevelZoneEmphasis	0.074332334
original	glszm	ZoneEntropy	5.107621805
original	glszm	SmallAreaLowGrayLevelEmphasis	0.053946096
original	ngtdm	Coarseness	0.047351197
original	ngtdm	Complexity	409.5425215
original	ngtdm	Strength	6.129198708
original	ngtdm	Contrast	0.335576828
original	ngtdm	Busyness	0.134187791
wavelet-HHH	gldm	GrayLevelVariance	1.470588235
wavelet-HHH	gldm	HighGrayLevelEmphasis	13.72058824
wavelet-HHH	gldm	DependenceEntropy	4.758220722
wavelet-HHH	gldm	DependenceNonUniformity	14.76470588
wavelet-HHH	gldm	GrayLevelNonUniformity	34.73529412
wavelet-HHH	gldm	SmallDependenceEmphasis	0.114419169
wavelet-HHH	gldm	SmallDependenceHighGrayLevelEmphasis	2.107779953
wavelet-HHH	gldm	DependenceNonUniformityNormalized	0.108564014
wavelet-HHH	gldm	LargeDependenceEmphasis	35.05882353
wavelet-HHH	gldm	LargeDependenceLowGrayLevelEmphasis	3.52875817
wavelet-HHH	gldm	DependenceVariance	7.031141869
wavelet-HHH	gldm	LargeDependenceHighGrayLevelEmphasis	420.3970588
wavelet-HHH	gldm	SmallDependenceLowGrayLevelEmphasis	0.037335573
wavelet-HHH	gldm	LowGrayLevelEmphasis	0.130890523
wavelet-HHH	glcm	JointAverage	3.489098105
wavelet-HHH	glcm	SumAverage	6.978196209
wavelet-HHH	glcm	JointEntropy	4.119880885
wavelet-HHH	glcm	ClusterShade	2.403950408
wavelet-HHH	glcm	MaximumProbability	0.132516865
wavelet-HHH	glcm	Idmn	0.975139609
wavelet-HHH	glcm	JointEnergy	0.073548478
wavelet-HHH	glcm	Contrast	2.779179153
wavelet-HHH	glcm	DifferenceEntropy	1.930224241
wavelet-HHH	glcm	InverseVariance	0.458151714
wavelet-HHH	glcm	DifferenceVariance	1.227060363
wavelet-HHH	glcm	Idn	0.898993306
wavelet-HHH	glcm	Idm	0.524692219
wavelet-HHH	glcm	Correlation	-0.044744162
wavelet-HHH	glcm	Autocorrelation	12.10683015
wavelet-HHH	glcm	SumEntropy	2.566906889
wavelet-HHH	glcm	SumSquares	1.321908309
wavelet-HHH	glcm	ClusterProminence	28.50239518
wavelet-HHH	glcm	Imc2	0.55729692
wavelet-HHH	glcm	Imc1	-0.092653508

wavelet-HHH	glcm	DifferenceAverage	1.225091583
wavelet-HHH	glcm	Id	0.570402814
wavelet-HHH	glcm	ClusterTendency	2.508454084
wavelet-HHH	firstorder	InterquartileRange	35.30531263
wavelet-HHH	firstorder	Skewness	0.84206504
wavelet-HHH	firstorder	Uniformity	0.255406574
wavelet-HHH	firstorder	Median	-0.255835577
wavelet-HHH	firstorder	Energy	122602.3332
wavelet-HHH	firstorder	RobustMeanAbsoluteDeviation	15.1790229
wavelet-HHH	firstorder	MeanAbsoluteDeviation	22.56454685
wavelet-HHH	firstorder	TotalEnergy	3310262.996
wavelet-HHH	firstorder	Maximum	158.173551
wavelet-HHH	firstorder	RootMeanSquared	30.02478549
wavelet-HHH	firstorder	90Percentile	37.14336319
wavelet-HHH	firstorder	Minimum	-74.47608972
wavelet-HHH	firstorder	Entropy	2.197213468
wavelet-HHH	firstorder	Range	232.6496407
wavelet-HHH	firstorder	Variance	901.4547199
wavelet-HHH	firstorder	10Percentile	-38.45793144
wavelet-HHH	firstorder	Kurtosis	7.467397776
wavelet-HHH	firstorder	Mean	0.181725316
wavelet-HHH	glrlm	ShortRunLowGrayLevelEmphasis	0.122485743
wavelet-HHH	glrlm	GrayLevelVariance	1.641151245
wavelet-HHH	glrlm	LowGrayLevelRunEmphasis	0.136444691
wavelet-HHH	glrlm	GrayLevelNonUniformityNormalized	0.240805614
wavelet-HHH	glrlm	RunVariance	0.211297289
wavelet-HHH	glrlm	GrayLevelNonUniformity	27.33718441
wavelet-HHH	glrlm	LongRunEmphasis	1.653855104
wavelet-HHH	glrlm	ShortRunHighGrayLevelEmphasis	12.38296576
wavelet-HHH	glrlm	RunLengthNonUniformity	80.46829085
wavelet-HHH	glrlm	ShortRunEmphasis	0.865055222
wavelet-HHH	glrlm	LongRunHighGrayLevelEmphasis	21.97806657
wavelet-HHH	glrlm	RunPercentage	0.834841629
wavelet-HHH	glrlm	LongRunLowGrayLevelEmphasis	0.203704824
wavelet-HHH	glrlm	RunEntropy	2.929730243
wavelet-HHH	glrlm	HighGrayLevelRunEmphasis	14.02879369
wavelet-HHH	glrlm	RunLengthNonUniformityNormalized	0.706424255
wavelet-HHH	glszm	GrayLevelVariance	5.96484375
wavelet-HHH	glszm	ZoneVariance	198.25
wavelet-HHH	glszm	GrayLevelNonUniformityNormalized	0.1875
wavelet-HHH	glszm	SizeZoneNonUniformityNormalized	0.25
wavelet-HHH	glszm	SizeZoneNonUniformity	4
wavelet-HHH	glszm	GrayLevelNonUniformity	3
wavelet-HHH	glszm	LargeAreaEmphasis	270.5
wavelet-HHH	glszm	SmallAreaHighGrayLevelEmphasis	9.950420511
wavelet-HHH	glszm	ZonePercentage	0.117647059
wavelet-HHH	glszm	LargeAreaLowGrayLevelEmphasis	25.83263889
wavelet-HHH	glszm	LargeAreaHighGrayLevelEmphasis	3346.875
wavelet-HHH	glszm	HighGrayLevelZoneEmphasis	19.5625
wavelet-HHH	glszm	SmallAreaEmphasis	0.491024386
wavelet-HHH	glszm	LowGrayLevelZoneEmphasis	0.321822917
wavelet-HHH	glszm	ZoneEntropy	3.375
wavelet-HHH	glszm	SmallAreaLowGrayLevelEmphasis	0.2709435
wavelet-HHH	ngtdm	Coarseness	0.041549989
wavelet-HHH	ngtdm	Complexity	23.7983302

wavelet-HHH	ngtdm	Strength	1.03853459
wavelet-HHH	ngtdm	Contrast	0.06945661
wavelet-HHH	ngtdm	Busyness	0.989469693
wavelet-HLL	gldm	GrayLevelVariance	4.850291955
wavelet-HLL	gldm	HighGrayLevelEmphasis	47.77205882
wavelet-HLL	gldm	DependenceEntropy	5.440260916
wavelet-HLL	gldm	DependenceNonUniformity	19.41176471
wavelet-HLL	gldm	GrayLevelNonUniformity	19.16176471
wavelet-HLL	gldm	SmallDependenceEmphasis	0.130373085
wavelet-HLL	gldm	SmallDependenceHighGrayLevelEmphasis	7.229727367
wavelet-HLL	gldm	DependenceNonUniformityNormalized	0.142733564
wavelet-HLL	gldm	LargeDependenceEmphasis	23.92647059
wavelet-HLL	gldm	LargeDependenceLowGrayLevelEmphasis	0.809825016
wavelet-HLL	gldm	DependenceVariance	4.462802768
wavelet-HLL	gldm	LargeDependenceHighGrayLevelEmphasis	1019.580882
wavelet-HLL	gldm	SmallDependenceLowGrayLevelEmphasis	0.007687931
wavelet-HLL	gldm	LowGrayLevelEmphasis	0.047846076
wavelet-HLL	glcm	JointAverage	6.380431295
wavelet-HLL	glcm	SumAverage	12.76086259
wavelet-HLL	glcm	JointEntropy	5.465376872
wavelet-HLL	glcm	ClusterShade	18.19613123
wavelet-HLL	glcm	MaximumProbability	0.067293813
wavelet-HLL	glcm	Idmn	0.970834902
wavelet-HLL	glcm	JointEnergy	0.029767617
wavelet-HLL	glcm	Contrast	5.461194633
wavelet-HLL	glcm	DifferenceEntropy	2.335113613
wavelet-HLL	glcm	InverseVariance	0.40471693
wavelet-HLL	glcm	DifferenceVariance	2.279982278
wavelet-HLL	glcm	Idn	0.891340949
wavelet-HLL	glcm	Idm	0.439381871
wavelet-HLL	glcm	Correlation	0.440376871
wavelet-HLL	glcm	Autocorrelation	42.92865642
wavelet-HLL	glcm	SumEntropy	3.750211636
wavelet-HLL	glcm	SumSquares	4.942378686
wavelet-HLL	glcm	ClusterProminence	656.9009793
wavelet-HLL	glcm	Imc2	0.84495373
wavelet-HLL	glcm	Imc1	-0.221139929
wavelet-HLL	glcm	DifferenceAverage	1.735851498
wavelet-HLL	glcm	Id	0.500960478
wavelet-HLL	glcm	ClusterTendency	14.30832011
wavelet-HLL	firstorder	InterquartileRange	62.2875002
wavelet-HLL	firstorder	Skewness	0.298948926
wavelet-HLL	firstorder	Uniformity	0.140895329
wavelet-HLL	firstorder	Median	-27.64358743
wavelet-HLL	firstorder	Energy	479643.6694
wavelet-HLL	firstorder	RobustMeanAbsoluteDeviation	26.99332394
wavelet-HLL	firstorder	MeanAbsoluteDeviation	41.7948173
wavelet-HLL	firstorder	TotalEnergy	12950379.07
wavelet-HLL	firstorder	Maximum	149.4192476
wavelet-HLL	firstorder	RootMeanSquared	59.38679724
wavelet-HLL	firstorder	90Percentile	60.03457732
wavelet-HLL	firstorder	Minimum	-171.6938212
wavelet-HLL	firstorder	Entropy	3.071718526
wavelet-HLL	firstorder	Range	321.1130688
wavelet-HLL	firstorder	Variance	2994.392002

wavelet-HLL	firstorder	10Percentile	-84.69465826
wavelet-HLL	firstorder	Kurtosis	3.471093106
wavelet-HLL	firstorder	Mean	-23.07378782
wavelet-HLL	glrlm	ShortRunLowGrayLevelEmphasis	0.045466644
wavelet-HLL	glrlm	GrayLevelVariance	5.047350438
wavelet-HLL	glrlm	LowGrayLevelRunEmphasis	0.049505665
wavelet-HLL	glrlm	GrayLevelNonUniformityNormalized	0.136743747
wavelet-HLL	glrlm	RunVariance	0.163995111
wavelet-HLL	glrlm	GrayLevelNonUniformity	16.16268405
wavelet-HLL	glrlm	LongRunEmphasis	1.505725356
wavelet-HLL	glrlm	ShortRunHighGrayLevelEmphasis	43.5975139
wavelet-HLL	glrlm	RunLengthNonUniformity	90.88419306
wavelet-HLL	glrlm	ShortRunEmphasis	0.893418137
wavelet-HLL	glrlm	LongRunHighGrayLevelEmphasis	70.54596058
wavelet-HLL	glrlm	RunPercentage	0.868778281
wavelet-HLL	glrlm	LongRunLowGrayLevelEmphasis	0.067986807
wavelet-HLL	glrlm	RunEntropy	3.637835284
wavelet-HLL	glrlm	HighGrayLevelRunEmphasis	48.34056918
wavelet-HLL	glrlm	RunLengthNonUniformityNormalized	0.763795362
wavelet-HLL	glszm	GrayLevelVariance	8.222222222
wavelet-HLL	glszm	ZoneVariance	47.63888889
wavelet-HLL	glszm	GrayLevelNonUniformityNormalized	0.125
wavelet-HLL	glszm	SizeZoneNonUniformityNormalized	0.163194444
wavelet-HLL	glszm	SizeZoneNonUniformity	3.916666667
wavelet-HLL	glszm	GrayLevelNonUniformity	3
wavelet-HLL	glszm	LargeAreaEmphasis	79.75
wavelet-HLL	glszm	SmallAreaHighGrayLevelEmphasis	22.11092866
wavelet-HLL	glszm	ZonePercentage	0.176470588
wavelet-HLL	glszm	LargeAreaLowGrayLevelEmphasis	2.421817015
wavelet-HLL	glszm	LargeAreaHighGrayLevelEmphasis	3476.375
wavelet-HLL	glszm	HighGrayLevelZoneEmphasis	52.66666667
wavelet-HLL	glszm	SmallAreaEmphasis	0.357585328
wavelet-HLL	glszm	LowGrayLevelZoneEmphasis	0.079128034
wavelet-HLL	glszm	ZoneEntropy	4.334962501
wavelet-HLL	glszm	SmallAreaLowGrayLevelEmphasis	0.020560269
wavelet-HLL	ngtdm	Coarseness	0.054316891
wavelet-HLL	ngtdm	Complexity	65.24798925
wavelet-HLL	ngtdm	Strength	2.676735456
wavelet-HLL	ngtdm	Contrast	0.086051602
wavelet-HLL	ngtdm	Busyness	0.252758447
wavelet-LHL	gldm	GrayLevelVariance	19.39570718
wavelet-LHL	gldm	HighGrayLevelEmphasis	130.4191176
wavelet-LHL	gldm	DependenceEntropy	5.477117349
wavelet-LHL	gldm	DependenceNonUniformity	37.48529412
wavelet-LHL	gldm	GrayLevelNonUniformity	9.544117647
wavelet-LHL	gldm	SmallDependenceEmphasis	0.448415033
wavelet-LHL	gldm	SmallDependenceHighGrayLevelEmphasis	67.1358844
wavelet-LHL	gldm	DependenceNonUniformityNormalized	0.275627163
wavelet-LHL	gldm	LargeDependenceEmphasis	5.691176471
wavelet-LHL	gldm	LargeDependenceLowGrayLevelEmphasis	0.114662077
wavelet-LHL	gldm	DependenceVariance	1.081314879
wavelet-LHL	gldm	LargeDependenceHighGrayLevelEmphasis	652.0808824
wavelet-LHL	gldm	SmallDependenceLowGrayLevelEmphasis	0.014996522
wavelet-LHL	gldm	LowGrayLevelEmphasis	0.024690019
wavelet-LHL	glcm	JointAverage	10.28882869

wavelet-LHL	glcm	SumAverage	20.57765739
wavelet-LHL	glcm	JointEntropy	6.580483138
wavelet-LHL	glcm	ClusterShade	167.4049421
wavelet-LHL	glcm	MaximumProbability	0.028425932
wavelet-LHL	glcm	Idmn	0.945485056
wavelet-LHL	glcm	JointEnergy	0.012144993
wavelet-LHL	glcm	Contrast	34.93534311
wavelet-LHL	glcm	DifferenceEntropy	3.350814861
wavelet-LHL	glcm	InverseVariance	0.197034622
wavelet-LHL	glcm	DifferenceVariance	11.59800059
wavelet-LHL	glcm	Idn	0.845947415
wavelet-LHL	glcm	Idm	0.188849509
wavelet-LHL	glcm	Correlation	0.130316477
wavelet-LHL	glcm	Autocorrelation	108.5496134
wavelet-LHL	glcm	SumEntropy	4.36897933
wavelet-LHL	glcm	SumSquares	20.14661835
wavelet-LHL	glcm	ClusterProminence	7146.44292
wavelet-LHL	glcm	Imc2	0.972933619
wavelet-LHL	glcm	Imc1	-0.367197392
wavelet-LHL	glcm	DifferenceAverage	4.617815794
wavelet-LHL	glcm	Id	0.284870471
wavelet-LHL	glcm	ClusterTendency	45.65113029
wavelet-LHL	firstorder	InterquartileRange	145.4929546
wavelet-LHL	firstorder	Skewness	0.43221909
wavelet-LHL	firstorder	Uniformity	0.070177336
wavelet-LHL	firstorder	Median	-22.91829763
wavelet-LHL	firstorder	Energy	1704760.811
wavelet-LHL	firstorder	RobustMeanAbsoluteDeviation	62.91853355
wavelet-LHL	firstorder	MeanAbsoluteDeviation	88.80715745
wavelet-LHL	firstorder	TotalEnergy	46028541.9
wavelet-LHL	firstorder	Maximum	299.9122676
wavelet-LHL	firstorder	RootMeanSquared	111.9598409
wavelet-LHL	firstorder	90Percentile	124.3923666
wavelet-LHL	firstorder	Minimum	-262.7463048
wavelet-LHL	firstorder	Entropy	4.042295159
wavelet-LHL	firstorder	Range	562.6585724
wavelet-LHL	firstorder	Variance	12008.28524
wavelet-LHL	firstorder	10Percentile	-156.3011392
wavelet-LHL	firstorder	Kurtosis	2.900709244
wavelet-LHL	firstorder	Mean	-22.95039699
wavelet-LHL	glrlm	ShortRunLowGrayLevelEmphasis	0.024351115
wavelet-LHL	glrlm	GrayLevelVariance	19.57461226
wavelet-LHL	glrlm	LowGrayLevelRunEmphasis	0.024996521
wavelet-LHL	glrlm	GrayLevelNonUniformityNormalized	0.069791757
wavelet-LHL	glrlm	RunVariance	0.05123344
wavelet-LHL	glrlm	GrayLevelNonUniformity	9.077125652
wavelet-LHL	glrlm	LongRunEmphasis	1.153127085
wavelet-LHL	glrlm	ShortRunHighGrayLevelEmphasis	127.3901711
wavelet-LHL	glrlm	RunLengthNonUniformity	119.7088882
wavelet-LHL	glrlm	ShortRunEmphasis	0.965670702
wavelet-LHL	glrlm	LongRunHighGrayLevelEmphasis	148.6568504
wavelet-LHL	glrlm	RunPercentage	0.955882353
wavelet-LHL	glrlm	LongRunLowGrayLevelEmphasis	0.027746685
wavelet-LHL	glrlm	RunEntropy	4.22331592
wavelet-LHL	glrlm	HighGrayLevelRunEmphasis	131.2587819

wavelet-LHL	glrlm	RunLengthNonUniformityNormalized	0.917338309
wavelet-LHL	glszm	GrayLevelVariance	22.21805217
wavelet-LHL	glszm	ZoneVariance	2.227810096
wavelet-LHL	glszm	GrayLevelNonUniformityNormalized	0.066241321
wavelet-LHL	glszm	SizeZoneNonUniformityNormalized	0.433289548
wavelet-LHL	glszm	SizeZoneNonUniformity	31.63013699
wavelet-LHL	glszm	GrayLevelNonUniformity	4.835616438
wavelet-LHL	glszm	LargeAreaEmphasis	5.698630137
wavelet-LHL	glszm	SmallAreaHighGrayLevelEmphasis	108.7309928
wavelet-LHL	glszm	ZonePercentage	0.536764706
wavelet-LHL	glszm	LargeAreaLowGrayLevelEmphasis	0.136268546
wavelet-LHL	glszm	LargeAreaHighGrayLevelEmphasis	610.5890411
wavelet-LHL	glszm	HighGrayLevelZoneEmphasis	147.7808219
wavelet-LHL	glszm	SmallAreaEmphasis	0.68385738
wavelet-LHL	glszm	LowGrayLevelZoneEmphasis	0.029907783
wavelet-LHL	glszm	ZoneEntropy	5.239357737
wavelet-LHL	glszm	SmallAreaLowGrayLevelEmphasis	0.02518587
wavelet-LHL	ngtdm	Coarseness	0.037018937
wavelet-LHL	ngtdm	Complexity	609.7566662
wavelet-LHL	ngtdm	Strength	6.150875799
wavelet-LHL	ngtdm	Contrast	0.27672301
wavelet-LHL	ngtdm	Busyness	0.139274977
wavelet-LHH	gldm	GrayLevelVariance	19.92733564
wavelet-LHH	gldm	HighGrayLevelEmphasis	185.8823529
wavelet-LHH	gldm	DependenceEntropy	5.312194852
wavelet-LHH	gldm	DependenceNonUniformity	36.88235294
wavelet-LHH	gldm	GrayLevelNonUniformity	10.47058824
wavelet-LHH	gldm	SmallDependenceEmphasis	0.409726307
wavelet-LHH	gldm	SmallDependenceHighGrayLevelEmphasis	93.19719567
wavelet-LHH	gldm	DependenceNonUniformityNormalized	0.271193772
wavelet-LHH	gldm	LargeDependenceEmphasis	6.485294118
wavelet-LHH	gldm	LargeDependenceLowGrayLevelEmphasis	0.110600292
wavelet-LHH	gldm	DependenceVariance	1.356401384
wavelet-LHH	gldm	LargeDependenceHighGrayLevelEmphasis	1020.647059
wavelet-LHH	gldm	SmallDependenceLowGrayLevelEmphasis	0.007468055
wavelet-LHH	gldm	LowGrayLevelEmphasis	0.023530868
wavelet-LHH	glcm	JointAverage	12.71937207
wavelet-LHH	glcm	SumAverage	25.43874414
wavelet-LHH	glcm	JointEntropy	6.462350692
wavelet-LHH	glcm	ClusterShade	65.30361632
wavelet-LHH	glcm	MaximumProbability	0.033308797
wavelet-LHH	glcm	Idmn	0.950964804
wavelet-LHH	glcm	JointEnergy	0.013511071
wavelet-LHH	glcm	Contrast	40.05773849
wavelet-LHH	glcm	DifferenceEntropy	3.448969387
wavelet-LHH	glcm	InverseVariance	0.215149947
wavelet-LHH	glcm	DifferenceVariance	15.73205854
wavelet-LHH	glcm	Idn	0.857537969
wavelet-LHH	glcm	Idm	0.203121236
wavelet-LHH	glcm	Correlation	-0.099586261
wavelet-LHH	glcm	Autocorrelation	160.3764412
wavelet-LHH	glcm	SumEntropy	4.077971291
wavelet-LHH	glcm	SumSquares	18.58438428
wavelet-LHH	glcm	ClusterProminence	5750.453212
wavelet-LHH	glcm	Imc2	0.974313092

wavelet-LHH	glcm	Imc1	-0.379665567
wavelet-LHH	glcm	DifferenceAverage	4.797080267
wavelet-LHH	glcm	Id	0.29327871
wavelet-LHH	glcm	ClusterTendency	34.27979864
wavelet-LHH	firstorder	InterquartileRange	127.4340182
wavelet-LHH	firstorder	Skewness	0.302159969
wavelet-LHH	firstorder	Uniformity	0.076989619
wavelet-LHH	firstorder	Median	-3.99087698
wavelet-LHH	firstorder	Energy	1712800.163
wavelet-LHH	firstorder	RobustMeanAbsoluteDeviation	52.36108331
wavelet-LHH	firstorder	MeanAbsoluteDeviation	83.60326245
wavelet-LHH	firstorder	TotalEnergy	46245604.4
wavelet-LHH	firstorder	Maximum	336.001785
wavelet-LHH	firstorder	RootMeanSquared	112.2235218
wavelet-LHH	firstorder	90Percentile	146.4652703
wavelet-LHH	firstorder	Minimum	-278.2552978
wavelet-LHH	firstorder	Entropy	4.023432798
wavelet-LHH	firstorder	Range	614.2570828
wavelet-LHH	firstorder	Variance	12509.33597
wavelet-LHH	firstorder	10Percentile	-118.1250094
wavelet-LHH	firstorder	Kurtosis	3.68957601
wavelet-LHH	firstorder	Mean	9.207761866
wavelet-LHH	glrlm	ShortRunLowGrayLevelEmphasis	0.022807249
wavelet-LHH	glrlm	GrayLevelVariance	20.42454606
wavelet-LHH	glrlm	LowGrayLevelRunEmphasis	0.02365904
wavelet-LHH	glrlm	GrayLevelNonUniformityNormalized	0.075143984
wavelet-LHH	glrlm	RunVariance	0.053051742
wavelet-LHH	glrlm	GrayLevelNonUniformity	9.726975122
wavelet-LHH	glrlm	LongRunEmphasis	1.167621217
wavelet-LHH	glrlm	ShortRunHighGrayLevelEmphasis	181.6376951
wavelet-LHH	glrlm	RunLengthNonUniformity	117.8834846
wavelet-LHH	glrlm	ShortRunEmphasis	0.960747258
wavelet-LHH	glrlm	LongRunHighGrayLevelEmphasis	213.8556884
wavelet-LHH	glrlm	RunPercentage	0.951357466
wavelet-LHH	glrlm	LongRunLowGrayLevelEmphasis	0.027115765
wavelet-LHH	glrlm	RunEntropy	4.242807068
wavelet-LHH	glrlm	HighGrayLevelRunEmphasis	187.5978781
wavelet-LHH	glrlm	RunLengthNonUniformityNormalized	0.906939093
wavelet-LHH	glszm	GrayLevelVariance	27.37660155
wavelet-LHH	glszm	ZoneVariance	3.883217811
wavelet-LHH	glszm	GrayLevelNonUniformityNormalized	0.056920815
wavelet-LHH	glszm	SizeZoneNonUniformityNormalized	0.397185465
wavelet-LHH	glszm	SizeZoneNonUniformity	27.4057971
wavelet-LHH	glszm	GrayLevelNonUniformity	3.927536232
wavelet-LHH	glszm	LargeAreaEmphasis	7.768115942
wavelet-LHH	glszm	SmallAreaHighGrayLevelEmphasis	156.8235829
wavelet-LHH	glszm	ZonePercentage	0.507352941
wavelet-LHH	glszm	LargeAreaLowGrayLevelEmphasis	0.118080969
wavelet-LHH	glszm	LargeAreaHighGrayLevelEmphasis	1242.26087
wavelet-LHH	glszm	HighGrayLevelZoneEmphasis	214.5507246
wavelet-LHH	glszm	SmallAreaEmphasis	0.640716586
wavelet-LHH	glszm	LowGrayLevelZoneEmphasis	0.023922888
wavelet-LHH	glszm	ZoneEntropy	5.231287862
wavelet-LHH	glszm	SmallAreaLowGrayLevelEmphasis	0.009662772
wavelet-LHH	ngtdm	Coarseness	0.042899231

wavelet-LHH	ngtdm	Complexity	713.9043061
wavelet-LHH	ngtdm	Strength	6.448511274
wavelet-LHH	ngtdm	Contrast	0.284986684
wavelet-LHH	ngtdm	Busyness	0.085607583
wavelet-LLH	gldm	GrayLevelVariance	160.2499459
wavelet-LLH	gldm	HighGrayLevelEmphasis	1965.875
wavelet-LLH	gldm	DependenceEntropy	5.869169896
wavelet-LLH	gldm	DependenceNonUniformity	48.38235294
wavelet-LLH	gldm	GrayLevelNonUniformity	5.485294118
wavelet-LLH	gldm	SmallDependenceEmphasis	0.600324755
wavelet-LLH	gldm	SmallDependenceHighGrayLevelEmphasis	1145.344457
wavelet-LLH	gldm	DependenceNonUniformityNormalized	0.355752595
wavelet-LLH	gldm	LargeDependenceEmphasis	4.441176471
wavelet-LLH	gldm	LargeDependenceLowGrayLevelEmphasis	0.011281626
wavelet-LLH	gldm	DependenceVariance	1.062067474
wavelet-LLH	gldm	LargeDependenceHighGrayLevelEmphasis	9058.330882
wavelet-LLH	gldm	SmallDependenceLowGrayLevelEmphasis	0.008548349
wavelet-LLH	gldm	LowGrayLevelEmphasis	0.008911003
wavelet-LLH	glcm	JointAverage	43.17479837
wavelet-LLH	glcm	SumAverage	86.34959674
wavelet-LLH	glcm	JointEntropy	7.018646035
wavelet-LLH	glcm	ClusterShade	-6632.449589
wavelet-LLH	glcm	MaximumProbability	0.022052435
wavelet-LLH	glcm	Idmn	0.945738959
wavelet-LLH	glcm	JointEnergy	0.008525454
wavelet-LLH	glcm	Contrast	285.2937358
wavelet-LLH	glcm	DifferenceEntropy	4.292493353
wavelet-LLH	glcm	InverseVariance	0.109572509
wavelet-LLH	glcm	DifferenceVariance	134.8556657
wavelet-LLH	glcm	Idn	0.863540789
wavelet-LLH	glcm	Idm	0.11641402
wavelet-LLH	glcm	Correlation	-0.003923726
wavelet-LLH	glcm	Autocorrelation	1869.28359
wavelet-LLH	glcm	SumEntropy	5.145477089
wavelet-LLH	glcm	SumSquares	147.3679405
wavelet-LLH	glcm	ClusterProminence	503185.018
wavelet-LLH	glcm	Imc2	0.997644495
wavelet-LLH	glcm	Imc1	-0.557187823
wavelet-LLH	glcm	DifferenceAverage	11.57131111
wavelet-LLH	glcm	Id	0.195011396
wavelet-LLH	glcm	ClusterTendency	304.1780262
wavelet-LLH	firstorder	InterquartileRange	279.9219711
wavelet-LLH	firstorder	Skewness	-1.401462759
wavelet-LLH	firstorder	Uniformity	0.040333045
wavelet-LLH	firstorder	Median	1.798618373
wavelet-LLH	firstorder	Energy	14422825.73
wavelet-LLH	firstorder	RobustMeanAbsoluteDeviation	121.8964964
wavelet-LLH	firstorder	MeanAbsoluteDeviation	226.8640768
wavelet-LLH	firstorder	TotalEnergy	389416294.7
wavelet-LLH	firstorder	Maximum	431.8769066
wavelet-LLH	firstorder	RootMeanSquared	325.6534802
wavelet-LLH	firstorder	90Percentile	222.8784441
wavelet-LLH	firstorder	Minimum	-1117.992834
wavelet-LLH	firstorder	Entropy	4.955326075
wavelet-LLH	firstorder	Range	1549.869741

wavelet-LLH	firstorder	Variance	100452.7116
wavelet-LLH	firstorder	10Percentile	-603.2711562
wavelet-LLH	firstorder	Kurtosis	4.717796536
wavelet-LLH	firstorder	Mean	-74.81629247
wavelet-LLH	glrlm	ShortRunLowGrayLevelEmphasis	0.009175703
wavelet-LLH	glrlm	GrayLevelVariance	163.1749225
wavelet-LLH	glrlm	LowGrayLevelRunEmphasis	0.009195321
wavelet-LLH	glrlm	GrayLevelNonUniformityNormalized	0.039479915
wavelet-LLH	glrlm	RunVariance	0.037069878
wavelet-LLH	glrlm	GrayLevelNonUniformity	5.199584982
wavelet-LLH	glrlm	LongRunEmphasis	1.110048476
wavelet-LLH	glrlm	ShortRunHighGrayLevelEmphasis	1912.227897
wavelet-LLH	glrlm	RunLengthNonUniformity	123.9745148
wavelet-LLH	glrlm	ShortRunEmphasis	0.975363182
wavelet-LLH	glrlm	LongRunHighGrayLevelEmphasis	2188.940164
wavelet-LLH	glrlm	RunPercentage	0.967760181
wavelet-LLH	glrlm	LongRunLowGrayLevelEmphasis	0.00927942
wavelet-LLH	glrlm	RunEntropy	5.08118718
wavelet-LLH	glrlm	HighGrayLevelRunEmphasis	1962.796518
wavelet-LLH	glrlm	RunLengthNonUniformityNormalized	0.939473118
wavelet-LLH	glszm	GrayLevelVariance	191.9209157
wavelet-LLH	glszm	ZoneVariance	1.065787952
wavelet-LLH	glszm	GrayLevelNonUniformityNormalized	0.032951786
wavelet-LLH	glszm	SizeZoneNonUniformityNormalized	0.603884842
wavelet-LLH	glszm	SizeZoneNonUniformity	56.16129032
wavelet-LLH	glszm	GrayLevelNonUniformity	3.064516129
wavelet-LLH	glszm	LargeAreaEmphasis	3.204301075
wavelet-LLH	glszm	SmallAreaHighGrayLevelEmphasis	1520.287485
wavelet-LLH	glszm	ZonePercentage	0.683823529
wavelet-LLH	glszm	LargeAreaLowGrayLevelEmphasis	0.014202753
wavelet-LLH	glszm	LargeAreaHighGrayLevelEmphasis	6520.053763
wavelet-LLH	glszm	HighGrayLevelZoneEmphasis	1920.870968
wavelet-LLH	glszm	SmallAreaEmphasis	0.803240741
wavelet-LLH	glszm	LowGrayLevelZoneEmphasis	0.012642217
wavelet-LLH	glszm	ZoneEntropy	5.639709989
wavelet-LLH	glszm	SmallAreaLowGrayLevelEmphasis	0.012424227
wavelet-LLH	ngtdm	Coarseness	0.03476393
wavelet-LLH	ngtdm	Complexity	7789.250034
wavelet-LLH	ngtdm	Strength	35.903998
wavelet-LLH	ngtdm	Contrast	1.579699125
wavelet-LLH	ngtdm	Busyness	0.015662813
wavelet-LLL	gldm	GrayLevelVariance	182.4299308
wavelet-LLL	gldm	HighGrayLevelEmphasis	981.3235294
wavelet-LLL	gldm	DependenceEntropy	5.96684793
wavelet-LLL	gldm	DependenceNonUniformity	69.82352941
wavelet-LLL	gldm	GrayLevelNonUniformity	3.529411765
wavelet-LLL	gldm	SmallDependenceEmphasis	0.736111111
wavelet-LLL	gldm	SmallDependenceHighGrayLevelEmphasis	666.1768791
wavelet-LLL	gldm	DependenceNonUniformityNormalized	0.513408304
wavelet-LLL	gldm	LargeDependenceEmphasis	2.382352941
wavelet-LLL	gldm	LargeDependenceLowGrayLevelEmphasis	0.013277715
wavelet-LLL	gldm	DependenceVariance	0.389273356
wavelet-LLL	gldm	LargeDependenceHighGrayLevelEmphasis	2736.485294
wavelet-LLL	gldm	SmallDependenceLowGrayLevelEmphasis	0.011158479
wavelet-LLL	gldm	LowGrayLevelEmphasis	0.011512302

wavelet-LLL	glcm	JointAverage	30.8822397
wavelet-LLL	glcm	SumAverage	61.76447941
wavelet-LLL	glcm	JointEntropy	7.218692114
wavelet-LLL	glcm	ClusterShade	461.8360543
wavelet-LLL	glcm	MaximumProbability	0.013554145
wavelet-LLL	glcm	Idmn	0.953475252
wavelet-LLL	glcm	JointEnergy	0.007005549
wavelet-LLL	glcm	Contrast	178.2053168
wavelet-LLL	glcm	DifferenceEntropy	4.382969903
wavelet-LLL	glcm	InverseVariance	0.097276529
wavelet-LLL	glcm	DifferenceVariance	56.61363685
wavelet-LLL	glcm	Idn	0.853718399
wavelet-LLL	glcm	Idm	0.086708627
wavelet-LLL	glcm	Correlation	0.490967409
wavelet-LLL	glcm	Autocorrelation	1040.021125
wavelet-LLL	glcm	SumEntropy	5.533097966
wavelet-LLL	glcm	SumSquares	174.6277752
wavelet-LLL	glcm	ClusterProminence	594194.6766
wavelet-LLL	glcm	Imc2	0.999504314
wavelet-LLL	glcm	Imc1	-0.651460311
wavelet-LLL	glcm	DifferenceAverage	10.773446
wavelet-LLL	glcm	Id	0.16406213
wavelet-LLL	glcm	ClusterTendency	520.3057841
wavelet-LLL	firstorder	InterquartileRange	488.7304208
wavelet-LLL	firstorder	Skewness	0.208686989
wavelet-LLL	firstorder	Uniformity	0.025951557
wavelet-LLL	firstorder	Median	-1464.509022
wavelet-LLL	firstorder	Energy	304226026.9
wavelet-LLL	firstorder	RobustMeanAbsoluteDeviation	201.4172484
wavelet-LLL	firstorder	MeanAbsoluteDeviation	276.6476506
wavelet-LLL	firstorder	TotalEnergy	8214102725
wavelet-LLL	firstorder	Maximum	-720.8855309
wavelet-LLL	firstorder	RootMeanSquared	1495.645707
wavelet-LLL	firstorder	90Percentile	-998.9388563
wavelet-LLL	firstorder	Minimum	-2149.346628
wavelet-LLL	firstorder	Entropy	5.457050761
wavelet-LLL	firstorder	Range	1428.461097
wavelet-LLL	firstorder	Variance	115268.6322
wavelet-LLL	firstorder	10Percentile	-1897.885492
wavelet-LLL	firstorder	Kurtosis	2.274369923
wavelet-LLL	firstorder	Mean	-1456.601335
wavelet-LLL	glrlm	ShortRunLowGrayLevelEmphasis	0.011661576
wavelet-LLL	glrlm	GrayLevelVariance	183.0965542
wavelet-LLL	glrlm	LowGrayLevelRunEmphasis	0.011677377
wavelet-LLL	glrlm	GrayLevelNonUniformityNormalized	0.02581992
wavelet-LLL	glrlm	RunVariance	0.015829258
wavelet-LLL	glrlm	GrayLevelNonUniformity	3.455978112
wavelet-LLL	glrlm	LongRunEmphasis	1.04850745
wavelet-LLL	glrlm	ShortRunHighGrayLevelEmphasis	962.115056
wavelet-LLL	glrlm	RunLengthNonUniformity	129.628778
wavelet-LLL	glrlm	ShortRunEmphasis	0.987873138
wavelet-LLL	glrlm	LongRunHighGrayLevelEmphasis	1037.277143
wavelet-LLL	glrlm	RunPercentage	0.984162896
wavelet-LLL	glrlm	LongRunLowGrayLevelEmphasis	0.011740583
wavelet-LLL	glrlm	RunEntropy	5.507846236

wavelet-LLL	glrlm	HighGrayLevelRunEmphasis	977.1474734
wavelet-LLL	glrlm	RunLengthNonUniformityNormalized	0.968341484
wavelet-LLL	glszm	GrayLevelVariance	195.0999074
wavelet-LLL	glszm	ZoneVariance	0.333136941
wavelet-LLL	glszm	GrayLevelNonUniformityNormalized	0.024324552
wavelet-LLL	glszm	SizeZoneNonUniformityNormalized	0.697331874
wavelet-LLL	glszm	SizeZoneNonUniformity	76.00917431
wavelet-LLL	glszm	GrayLevelNonUniformity	2.651376147
wavelet-LLL	glszm	LargeAreaEmphasis	1.889908257
wavelet-LLL	glszm	SmallAreaHighGrayLevelEmphasis	764.1106014
wavelet-LLL	glszm	ZonePercentage	0.801470588
wavelet-LLL	glszm	LargeAreaLowGrayLevelEmphasis	0.015208104
wavelet-LLL	glszm	LargeAreaHighGrayLevelEmphasis	1996.330275
wavelet-LLL	glszm	HighGrayLevelZoneEmphasis	929.559633
wavelet-LLL	glszm	SmallAreaEmphasis	0.859072375
wavelet-LLL	glszm	LowGrayLevelZoneEmphasis	0.014033144
wavelet-LLL	glszm	ZoneEntropy	5.835627073
wavelet-LLL	glszm	SmallAreaLowGrayLevelEmphasis	0.013840779
wavelet-LLL	ngtdm	Coarseness	0.043889998
wavelet-LLL	ngtdm	Complexity	7445.182962
wavelet-LLL	ngtdm	Strength	48.25760272
wavelet-LLL	ngtdm	Contrast	0.959856448
wavelet-LLL	ngtdm	Busyness	0.01948203
wavelet-HHL	gldm	GrayLevelVariance	1.534115484
wavelet-HHL	gldm	HighGrayLevelEmphasis	23.61029412
wavelet-HHL	gldm	DependenceEntropy	4.967418957
wavelet-HHL	gldm	DependenceNonUniformity	16.45588235
wavelet-HHL	gldm	GrayLevelNonUniformity	30.80882353
wavelet-HHL	gldm	SmallDependenceEmphasis	0.15978292
wavelet-HHL	gldm	SmallDependenceHighGrayLevelEmphasis	3.937709162
wavelet-HHL	gldm	DependenceNonUniformityNormalized	0.120999135
wavelet-HHL	gldm	LargeDependenceEmphasis	31.10294118
wavelet-HHL	gldm	LargeDependenceLowGrayLevelEmphasis	1.460688119
wavelet-HHL	gldm	DependenceVariance	7.551903114
wavelet-HHL	gldm	LargeDependenceHighGrayLevelEmphasis	750.375
wavelet-HHL	gldm	SmallDependenceLowGrayLevelEmphasis	0.018022934
wavelet-HHL	gldm	LowGrayLevelEmphasis	0.063276154
wavelet-HHL	glcm	JointAverage	4.695501481
wavelet-HHL	glcm	SumAverage	9.391002963
wavelet-HHL	glcm	JointEntropy	4.368159922
wavelet-HHL	glcm	ClusterShade	0.277802417
wavelet-HHL	glcm	MaximumProbability	0.125980103
wavelet-HHL	glcm	Idmn	0.955872314
wavelet-HHL	glcm	JointEnergy	0.060016437
wavelet-HHL	glcm	Contrast	3.220408304
wavelet-HHL	glcm	DifferenceEntropy	2.049641596
wavelet-HHL	glcm	InverseVariance	0.424849405
wavelet-HHL	glcm	DifferenceVariance	1.243980804
wavelet-HHL	glcm	Idn	0.864757093
wavelet-HHL	glcm	Idm	0.481396597
wavelet-HHL	glcm	Correlation	-0.06774166
wavelet-HHL	glcm	Autocorrelation	21.94251834
wavelet-HHL	glcm	SumEntropy	2.669619094
wavelet-HHL	glcm	SumSquares	1.503770901
wavelet-HHL	glcm	ClusterProminence	22.60949895

wavelet-HHL	glcm	Imc2	0.601989334
wavelet-HHL	glcm	Imc1	-0.106264645
wavelet-HHL	glcm	DifferenceAverage	1.381676652
wavelet-HHL	glcm	Id	0.537996426
wavelet-HHL	glcm	ClusterTendency	2.7946753
wavelet-HHL	firstorder	InterquartileRange	44.12241125
wavelet-HHL	firstorder	Skewness	-0.151216698
wavelet-HHL	firstorder	Uniformity	0.226535467
wavelet-HHL	firstorder	Median	5.237027513
wavelet-HHL	firstorder	Energy	125870.0918
wavelet-HHL	firstorder	RobustMeanAbsoluteDeviation	17.0539314
wavelet-HHL	firstorder	MeanAbsoluteDeviation	24.25291631
wavelet-HHL	firstorder	TotalEnergy	3398492.479
wavelet-HHL	firstorder	Maximum	76.12770474
wavelet-HHL	firstorder	RootMeanSquared	30.42228428
wavelet-HHL	firstorder	90Percentile	40.43192236
wavelet-HHL	firstorder	Minimum	-89.20619165
wavelet-HHL	firstorder	Entropy	2.333536963
wavelet-HHL	firstorder	Range	165.3338964
wavelet-HHL	firstorder	Variance	910.7977148
wavelet-HHL	firstorder	10Percentile	-35.23581893
wavelet-HHL	firstorder	Kurtosis	2.793963865
wavelet-HHL	firstorder	Mean	3.83636105
wavelet-HHL	glrlm	ShortRunLowGrayLevelEmphasis	0.059740826
wavelet-HHL	glrlm	GrayLevelVariance	1.659988987
wavelet-HHL	glrlm	LowGrayLevelRunEmphasis	0.065698917
wavelet-HHL	glrlm	GrayLevelNonUniformityNormalized	0.216243416
wavelet-HHL	glrlm	RunVariance	0.191614615
wavelet-HHL	glrlm	GrayLevelNonUniformity	25.03580761
wavelet-HHL	glrlm	LongRunEmphasis	1.580729322
wavelet-HHL	glrlm	ShortRunHighGrayLevelEmphasis	20.79188773
wavelet-HHL	glrlm	RunLengthNonUniformity	85.72049414
wavelet-HHL	glrlm	ShortRunEmphasis	0.881340777
wavelet-HHL	glrlm	LongRunHighGrayLevelEmphasis	37.44519093
wavelet-HHL	glrlm	RunPercentage	0.851809955
wavelet-HHL	glrlm	LongRunLowGrayLevelEmphasis	0.09422064
wavelet-HHL	glrlm	RunEntropy	2.990778436
wavelet-HHL	glrlm	HighGrayLevelRunEmphasis	23.587799
wavelet-HHL	glrlm	RunLengthNonUniformityNormalized	0.736775701
wavelet-HHL	glszm	GrayLevelVariance	3.623263889
wavelet-HHL	glszm	ZoneVariance	105.8888889
wavelet-HHL	glszm	GrayLevelNonUniformityNormalized	0.173611111
wavelet-HHL	glszm	SizeZoneNonUniformityNormalized	0.336805556
wavelet-HHL	glszm	SizeZoneNonUniformity	8.083333333
wavelet-HHL	glszm	GrayLevelNonUniformity	4.166666667
wavelet-HHL	glszm	LargeAreaEmphasis	138
wavelet-HHL	glszm	SmallAreaHighGrayLevelEmphasis	14.9765507
wavelet-HHL	glszm	ZonePercentage	0.176470588
wavelet-HHL	glszm	LargeAreaLowGrayLevelEmphasis	6.565540497
wavelet-HHL	glszm	LargeAreaHighGrayLevelEmphasis	3231.125
wavelet-HHL	glszm	HighGrayLevelZoneEmphasis	25.79166667
wavelet-HHL	glszm	SmallAreaEmphasis	0.582876816
wavelet-HHL	glszm	LowGrayLevelZoneEmphasis	0.108728328
wavelet-HHL	glszm	ZoneEntropy	3.803508855
wavelet-HHL	glszm	SmallAreaLowGrayLevelEmphasis	0.082943121

wavelet-HHL	ngtdm	Coarseness	0.039819256
wavelet-HHL	ngtdm	Complexity	27.39501416
wavelet-HHL	ngtdm	Strength	0.741092397
wavelet-HHL	ngtdm	Contrast	0.059349601
wavelet-HHL	ngtdm	Busyness	0.641275444
wavelet-HLH	gldm	GrayLevelVariance	2.837316176
wavelet-HLH	gldm	HighGrayLevelEmphasis	110.4779412
wavelet-HLH	gldm	DependenceEntropy	5.072116243
wavelet-HLH	gldm	DependenceNonUniformity	15.02941176
wavelet-HLH	gldm	GrayLevelNonUniformity	29.20588235
wavelet-HLH	gldm	SmallDependenceEmphasis	0.218720765
wavelet-HLH	gldm	SmallDependenceHighGrayLevelEmphasis	22.62560818
wavelet-HLH	gldm	DependenceNonUniformityNormalized	0.110510381
wavelet-HLH	gldm	LargeDependenceEmphasis	33.73529412
wavelet-HLH	gldm	LargeDependenceLowGrayLevelEmphasis	0.301947423
wavelet-HLH	gldm	DependenceVariance	10.7517301
wavelet-HLH	gldm	LargeDependenceHighGrayLevelEmphasis	3932.375
wavelet-HLH	gldm	SmallDependenceLowGrayLevelEmphasis	0.010297215
wavelet-HLH	gldm	LowGrayLevelEmphasis	0.017609635
wavelet-HLH	glcm	JointAverage	10.46221675
wavelet-HLH	glcm	SumAverage	20.92443349
wavelet-HLH	glcm	JointEntropy	4.490602772
wavelet-HLH	glcm	ClusterShade	-13.01738173
wavelet-HLH	glcm	MaximumProbability	0.153236264
wavelet-HLH	glcm	Idmn	0.981201989
wavelet-HLH	glcm	JointEnergy	0.065102281
wavelet-HLH	glcm	Contrast	4.154248709
wavelet-HLH	glcm	DifferenceEntropy	2.131619687
wavelet-HLH	glcm	InverseVariance	0.458484648
wavelet-HLH	glcm	DifferenceVariance	2.0452979
wavelet-HLH	glcm	Idn	0.913448665
wavelet-HLH	glcm	Idm	0.488957607
wavelet-HLH	glcm	Correlation	0.018622882
wavelet-HLH	glcm	Autocorrelation	109.5253957
wavelet-HLH	glcm	SumEntropy	2.825910411
wavelet-HLH	glcm	SumSquares	2.140526642
wavelet-HLH	glcm	ClusterProminence	158.5355716
wavelet-HLH	glcm	Imc2	0.636939301
wavelet-HLH	glcm	Imc1	-0.111657842
wavelet-HLH	glcm	DifferenceAverage	1.441018097
wavelet-HLH	glcm	Id	0.539211912
wavelet-HLH	glcm	ClusterTendency	4.407857859
wavelet-HLH	firstorder	InterquartileRange	44.49085717
wavelet-HLH	firstorder	Skewness	-2.03201421
wavelet-HLH	firstorder	Uniformity	0.214749135
wavelet-HLH	firstorder	Median	2.742828105
wavelet-HLH	firstorder	Energy	223686.6648
wavelet-HLH	firstorder	RobustMeanAbsoluteDeviation	17.02351417
wavelet-HLH	firstorder	MeanAbsoluteDeviation	28.01451092
wavelet-HLH	firstorder	TotalEnergy	6039539.948
wavelet-HLH	firstorder	Maximum	81.21203967
wavelet-HLH	firstorder	RootMeanSquared	40.55557777
wavelet-HLH	firstorder	90Percentile	36.7788006
wavelet-HLH	firstorder	Minimum	-227.4901752
wavelet-HLH	firstorder	Entropy	2.531852901

wavelet-HLH	firstorder	Range	308.7022148
wavelet-HLH	firstorder	Variance	1638.377211
wavelet-HLH	firstorder	10Percentile	-37.79810484
wavelet-HLH	firstorder	Kurtosis	11.5988944
wavelet-HLH	firstorder	Mean	-2.525406272
wavelet-HLH	glrlm	ShortRunLowGrayLevelEmphasis	0.018126089
wavelet-HLH	glrlm	GrayLevelVariance	3.198950725
wavelet-HLH	glrlm	LowGrayLevelRunEmphasis	0.019122761
wavelet-HLH	glrlm	GrayLevelNonUniformityNormalized	0.19436988
wavelet-HLH	glrlm	RunVariance	0.215609476
wavelet-HLH	glrlm	GrayLevelNonUniformity	22.59368039
wavelet-HLH	glrlm	LongRunEmphasis	1.598148189
wavelet-HLH	glrlm	ShortRunHighGrayLevelEmphasis	97.01390892
wavelet-HLH	glrlm	RunLengthNonUniformity	87.50034538
wavelet-HLH	glrlm	ShortRunEmphasis	0.889139294
wavelet-HLH	glrlm	LongRunHighGrayLevelEmphasis	178.7483426
wavelet-HLH	glrlm	RunPercentage	0.854072398
wavelet-HLH	glrlm	LongRunLowGrayLevelEmphasis	0.024424449
wavelet-HLH	glrlm	RunEntropy	3.202547229
wavelet-HLH	glrlm	HighGrayLevelRunEmphasis	109.6949351
wavelet-HLH	glrlm	RunLengthNonUniformityNormalized	0.750684972
wavelet-HLH	glszm	GrayLevelVariance	8.126951093
wavelet-HLH	glszm	ZoneVariance	87.91467222
wavelet-HLH	glszm	GrayLevelNonUniformityNormalized	0.152965661
wavelet-HLH	glszm	SizeZoneNonUniformityNormalized	0.448491155
wavelet-HLH	glszm	SizeZoneNonUniformity	13.90322581
wavelet-HLH	glszm	GrayLevelNonUniformity	4.741935484
wavelet-HLH	glszm	LargeAreaEmphasis	107.1612903
wavelet-HLH	glszm	SmallAreaHighGrayLevelEmphasis	69.49641378
wavelet-HLH	glszm	ZonePercentage	0.227941176
wavelet-HLH	glszm	LargeAreaLowGrayLevelEmphasis	0.987351057
wavelet-HLH	glszm	LargeAreaHighGrayLevelEmphasis	12262.35484
wavelet-HLH	glszm	HighGrayLevelZoneEmphasis	103.0322581
wavelet-HLH	glszm	SmallAreaEmphasis	0.690604421
wavelet-HLH	glszm	LowGrayLevelZoneEmphasis	0.045834432
wavelet-HLH	glszm	ZoneEntropy	3.953565665
wavelet-HLH	glszm	SmallAreaLowGrayLevelEmphasis	0.042506419
wavelet-HLH	ngtdm	Coarseness	0.046796245
wavelet-HLH	ngtdm	Complexity	59.50660522
wavelet-HLH	ngtdm	Strength	2.691369584
wavelet-HLH	ngtdm	Contrast	0.073306515
wavelet-HLH	ngtdm	Busyness	0.174673402

Image type	Feature Class	Feature Name	502: RTSTRUCT: C99-DNU_segment_ITV
general	info	BoundingBox	(1, 0, 1, 2, 4, 3)
general	info	EnabledImageTypes	{u'Original': {}, u'Wavelet': {}}
general	info	GeneralSettings	{'distances': [1], 'additionalInfo': True, 'force2D': False, 'interpolator': 'sitkBSpline', 'resampledPixelSpacing': [3.0], 'label': 1, 'normalizeScale': 1, 'normalize': False, 'force2Ddimension': 0, 'removeOutliers': None, 'minimumROISize': None, u'binWidth': 25.0, 'minimumROIDimensions': 1, u'correctMask': True, 'preCrop': False, u'symmetricalGLCM': True, 'resegmentRange': None, 'padDistance': 5}
general	info	ImageHash	d17f0cc2f36e31c3c4a6b75adf86b46ae70033e8
general	info	ImageSpacing	(0.9765625000000002, 0.9765625000000002, 2.0)
general	info	MaskHash	32f349f3554876128e26aa60830c8eba3eec9528
general	info	NumpyVersion	1.13.1
general	info	PyWaveletVersion	0.5.2
general	info	SimpleITKVersion	1.1.0
general	info	Version	1.3.0.post102+g1b0eb74
general	info	VolumeNum	1
general	info	VoxelNum	14
original	shape	Maximum3DDiameter	9.486832981
original	shape	Maximum2DDiameterSlice	6.708203932
original	shape	Sphericity	1.021993642
original	shape	MinorAxis	8.22013146
original	shape	Elongation	0.814762667
original	shape	SurfaceVolumeRatio	0.654443257
original	shape	Volume	378
original	shape	MajorAxis	10.08898885
original	shape	SurfaceArea	247.3795511
original	shape	Flatness	0.585439383
original	shape	LeastAxis	5.906491407
original	shape	Maximum2DDiameterColumn	6.708203932
original	shape	Maximum2DDiameterRow	9.486832981
original	gldm	GrayLevelVariance	82.45408163
original	gldm	HighGrayLevelEmphasis	398.7857143
original	gldm	DependenceEntropy	3.521640636
original	gldm	DependenceNonUniformity	10.57142857
original	gldm	GrayLevelNonUniformity	1.285714286
original	gldm	SmallDependenceEmphasis	0.892857143
original	gldm	SmallDependenceHighGrayLevelEmphasis	346.9285714
original	gldm	DependenceNonUniformityNormalized	0.755102041
original	gldm	LargeDependenceEmphasis	1.428571429
original	gldm	LargeDependenceLowGrayLevelEmphasis	0.077152378
original	gldm	DependenceVariance	0.12244898
original	gldm	LargeDependenceHighGrayLevelEmphasis	606.2142857
original	gldm	SmallDependenceLowGrayLevelEmphasis	0.076045531

original	gldm	LowGrayLevelEmphasis	0.0762669
original	glcm	JointAverage	19.63814103
original	glcm	SumAverage	39.27628205
original	glcm	JointEntropy	3.070262097
original	glcm	ClusterShade	224.866705
original	glcm	MaximumProbability	0.139102564
original	glcm	Idmn	0.871814377
original	glcm	JointEnergy	0.130555556
original	glcm	Contrast	175.2455128
original	glcm	DifferenceEntropy	1.942056969
original	glcm	InverseVariance	0.115967093
original	glcm	DifferenceVariance	44.83899038
original	glcm	Idn	0.76264734
original	glcm	Idm	0.091001994
original	glcm	Correlation	-0.199994719
original	glcm	Autocorrelation	372.4064103
original	glcm	SumEntropy	2.039492867
original	glcm	SumSquares	72.15069311
original	glcm	ClusterProminence	29921.0159
original	glcm	Imc2	0.99713051
original	glcm	Imc1	-0.941722085
original	glcm	DifferenceAverage	11.12115385
original	glcm	Id	0.160007549
original	glcm	ClusterTendency	113.3572596
original	firstorder	InterquartileRange	305
original	firstorder	Skewness	0.022957734
original	firstorder	Uniformity	0.091836735
original	firstorder	Median	-351
original	firstorder	Energy	2357716
original	firstorder	RobustMeanAbsoluteDeviation	130.92
original	firstorder	MeanAbsoluteDeviation	191.2040816
original	firstorder	TotalEnergy	63658332
original	firstorder	Maximum	6
original	firstorder	RootMeanSquared	410.375786
original	firstorder	90Percentile	-15.1
original	firstorder	Minimum	-755
original	firstorder	Entropy	3.521640636
original	firstorder	Range	761
original	firstorder	Variance	51737.2449
original	firstorder	10Percentile	-594.4
original	firstorder	Kurtosis	2.016056657
original	firstorder	Mean	-341.5714286
original	glrlm	ShortRunLowGrayLevelEmphasis	0.076696789
original	glrlm	GrayLevelVariance	82.8288018
original	glrlm	LowGrayLevelRunEmphasis	0.076705958
original	glrlm	GrayLevelNonUniformityNormalized	0.091599863
original	glrlm	RunVariance	0.005461994
original	glrlm	GrayLevelNonUniformity	1.275570583
original	glrlm	LongRunEmphasis	1.017751479
original	glrlm	ShortRunHighGrayLevelEmphasis	396.1335587
original	glrlm	RunLengthNonUniformity	13.78106509
original	glrlm	ShortRunEmphasis	0.99556213
original	glrlm	LongRunHighGrayLevelEmphasis	406.8732037

original	glrlm	RunPercentage	0.994505495
original	glrlm	LongRunLowGrayLevelEmphasis	0.076742635
original	glrlm	RunEntropy	3.523560092
original	glrlm	HighGrayLevelRunEmphasis	398.2814877
original	glrlm	RunLengthNonUniformityNormalized	0.989076013
original	glszm	GrayLevelVariance	87.32544379
original	glszm	ZoneVariance	0.071005917
original	glszm	GrayLevelNonUniformityNormalized	0.088757396
original	glszm	SizeZoneNonUniformityNormalized	0.857988166
original	glszm	SizeZoneNonUniformity	11.15384615
original	glszm	GrayLevelNonUniformity	1.153846154
original	glszm	LargeAreaEmphasis	1.230769231
original	glszm	SmallAreaHighGrayLevelEmphasis	364.3076923
original	glszm	ZonePercentage	0.928571429
original	glszm	LargeAreaLowGrayLevelEmphasis	0.082451449
original	glszm	LargeAreaHighGrayLevelEmphasis	503.9230769
original	glszm	HighGrayLevelZoneEmphasis	392.2307692
original	glszm	SmallAreaEmphasis	0.942307692
original	glszm	LowGrayLevelZoneEmphasis	0.081974653
original	glszm	ZoneEntropy	3.546593564
original	glszm	SmallAreaLowGrayLevelEmphasis	0.081855454
original	ngtdm	Coarseness	0.094800484
original	ngtdm	Complexity	1290.650227
original	ngtdm	Strength	33.37535817
original	ngtdm	Contrast	11.12263238
original	ngtdm	Busyness	0.077971791
wavelet-HHH	gldm	GrayLevelVariance	6.37244898
wavelet-HHH	gldm	HighGrayLevelEmphasis	46.78571429
wavelet-HHH	gldm	DependenceEntropy	3.093069208
wavelet-HHH	gldm	DependenceNonUniformity	8.285714286
wavelet-HHH	gldm	GrayLevelNonUniformity	1.714285714
wavelet-HHH	gldm	SmallDependenceEmphasis	0.785714286
wavelet-HHH	gldm	SmallDependenceHighGrayLevelEmphasis	37.25
wavelet-HHH	gldm	DependenceNonUniformityNormalized	0.591836735
wavelet-HHH	gldm	LargeDependenceEmphasis	1.857142857
wavelet-HHH	gldm	LargeDependenceLowGrayLevelEmphasis	0.124809191
wavelet-HHH	gldm	DependenceVariance	0.204081633
wavelet-HHH	gldm	LargeDependenceHighGrayLevelEmphasis	84.92857143
wavelet-HHH	gldm	SmallDependenceLowGrayLevelEmphasis	0.095010084
wavelet-HHH	gldm	LowGrayLevelEmphasis	0.100969905
wavelet-HHH	glcm	JointAverage	6.186217949
wavelet-HHH	glcm	SumAverage	12.3724359
wavelet-HHH	glcm	JointEntropy	3.067056969
wavelet-HHH	glcm	ClusterShade	0.157442219
wavelet-HHH	glcm	MaximumProbability	0.140705128
wavelet-HHH	glcm	Idmn	0.899696826
wavelet-HHH	glcm	JointEnergy	0.129286859
wavelet-HHH	glcm	Contrast	13.24423077
wavelet-HHH	glcm	DifferenceEntropy	1.520570551
wavelet-HHH	glcm	InverseVariance	0.345113409
wavelet-HHH	glcm	DifferenceVariance	3.484887821
wavelet-HHH	glcm	Idn	0.788082888
wavelet-HHH	glcm	Idm	0.23197847

wavelet-HHH	glcm	Correlation	-0.1939022
wavelet-HHH	glcm	Autocorrelation	37.60128205
wavelet-HHH	glcm	SumEntropy	1.875409573
wavelet-HHH	glcm	SumSquares	5.65018563
wavelet-HHH	glcm	ClusterProminence	234.8358254
wavelet-HHH	glcm	Imc2	0.986745554
wavelet-HHH	glcm	Imc1	-0.813551315
wavelet-HHH	glcm	DifferenceAverage	2.98525641
wavelet-HHH	glcm	Id	0.326441036
wavelet-HHH	glcm	ClusterTendency	9.356511752
wavelet-HHH	firstorder	InterquartileRange	82.74569186
wavelet-HHH	firstorder	Skewness	-0.487554289
wavelet-HHH	firstorder	Uniformity	0.12244898
wavelet-HHH	firstorder	Median	22.65594672
wavelet-HHH	firstorder	Energy	56125.57429
wavelet-HHH	firstorder	RobustMeanAbsoluteDeviation	28.87040243
wavelet-HHH	firstorder	MeanAbsoluteDeviation	47.75543442
wavelet-HHH	firstorder	TotalEnergy	1515390.506
wavelet-HHH	firstorder	Maximum	109.2329345
wavelet-HHH	firstorder	RootMeanSquared	63.31642435
wavelet-HHH	firstorder	90Percentile	93.56470856
wavelet-HHH	firstorder	Minimum	-114.9660454
wavelet-HHH	firstorder	Entropy	3.093069208
wavelet-HHH	firstorder	Range	224.1989799
wavelet-HHH	firstorder	Variance	3610.440196
wavelet-HHH	firstorder	10Percentile	-47.70262492
wavelet-HHH	firstorder	Kurtosis	2.726623692
wavelet-HHH	firstorder	Mean	19.96320105
wavelet-HHH	glrlm	ShortRunLowGrayLevelEmphasis	0.101588817
wavelet-HHH	glrlm	GrayLevelVariance	6.418927016
wavelet-HHH	glrlm	LowGrayLevelRunEmphasis	0.101835673
wavelet-HHH	glrlm	GrayLevelNonUniformityNormalized	0.122727653
wavelet-HHH	glrlm	RunVariance	0.010923987
wavelet-HHH	glrlm	GrayLevelNonUniformity	1.699070161
wavelet-HHH	glrlm	LongRunEmphasis	1.035502959
wavelet-HHH	glrlm	ShortRunHighGrayLevelEmphasis	46.41779374
wavelet-HHH	glrlm	RunLengthNonUniformity	13.56213018
wavelet-HHH	glrlm	ShortRunEmphasis	0.99112426
wavelet-HHH	glrlm	LongRunHighGrayLevelEmphasis	48.39264582
wavelet-HHH	glrlm	RunPercentage	0.989010989
wavelet-HHH	glrlm	LongRunLowGrayLevelEmphasis	0.102823099
wavelet-HHH	glrlm	RunEntropy	3.091836269
wavelet-HHH	glrlm	HighGrayLevelRunEmphasis	46.81276416
wavelet-HHH	glrlm	RunLengthNonUniformityNormalized	0.978152025
wavelet-HHH	glszm	GrayLevelVariance	7.055555556
wavelet-HHH	glszm	ZoneVariance	0.138888889
wavelet-HHH	glszm	GrayLevelNonUniformityNormalized	0.125
wavelet-HHH	glszm	SizeZoneNonUniformityNormalized	0.722222222
wavelet-HHH	glszm	SizeZoneNonUniformity	8.666666667
wavelet-HHH	glszm	GrayLevelNonUniformity	1.5
wavelet-HHH	glszm	LargeAreaEmphasis	1.5
wavelet-HHH	glszm	SmallAreaHighGrayLevelEmphasis	41.60416667
wavelet-HHH	glszm	ZonePercentage	0.857142857

wavelet-HHH	glszm	LargeAreaLowGrayLevelEmphasis	0.127069056
wavelet-HHH	glszm	LargeAreaHighGrayLevelEmphasis	69.41666667
wavelet-HHH	glszm	HighGrayLevelZoneEmphasis	47.16666667
wavelet-HHH	glszm	SmallAreaEmphasis	0.875
wavelet-HHH	glszm	LowGrayLevelZoneEmphasis	0.113162806
wavelet-HHH	glszm	ZoneEntropy	3.084962501
wavelet-HHH	glszm	SmallAreaLowGrayLevelEmphasis	0.109686244
wavelet-HHH	ngtdm	Coarseness	0.265073819
wavelet-HHH	ngtdm	Complexity	77.65008503
wavelet-HHH	ngtdm	Strength	7.567187774
wavelet-HHH	ngtdm	Contrast	0.428457483
wavelet-HHH	ngtdm	Busyness	0.09568021
wavelet-HLL	gldm	GrayLevelVariance	130.6938776
wavelet-HLL	gldm	HighGrayLevelEmphasis	449.5714286
wavelet-HLL	gldm	DependenceEntropy	3.235926351
wavelet-HLL	gldm	DependenceNonUniformity	7.142857143
wavelet-HLL	gldm	GrayLevelNonUniformity	1.571428571
wavelet-HLL	gldm	SmallDependenceEmphasis	0.571428571
wavelet-HLL	gldm	SmallDependenceHighGrayLevelEmphasis	343.8214286
wavelet-HLL	gldm	DependenceNonUniformityNormalized	0.510204082
wavelet-HLL	gldm	LargeDependenceEmphasis	2.714285714
wavelet-HLL	gldm	LargeDependenceLowGrayLevelEmphasis	0.611421141
wavelet-HLL	gldm	DependenceVariance	0.244897959
wavelet-HLL	gldm	LargeDependenceHighGrayLevelEmphasis	872.5714286
wavelet-HLL	gldm	SmallDependenceLowGrayLevelEmphasis	0.039997249
wavelet-HLL	gldm	LowGrayLevelEmphasis	0.154282027
wavelet-HLL	glcm	JointAverage	19.06346154
wavelet-HLL	glcm	SumAverage	38.12692308
wavelet-HLL	glcm	JointEntropy	3.022185174
wavelet-HLL	glcm	ClusterShade	-836.8709551
wavelet-HLL	glcm	MaximumProbability	0.147115385
wavelet-HLL	glcm	Idmn	0.883773013
wavelet-HLL	glcm	JointEnergy	0.132625534
wavelet-HLL	glcm	Contrast	193.4487179
wavelet-HLL	glcm	DifferenceEntropy	1.915567666
wavelet-HLL	glcm	InverseVariance	0.086015042
wavelet-HLL	glcm	DifferenceVariance	52.24596154
wavelet-HLL	glcm	Idn	0.788180017
wavelet-HLL	glcm	Idm	0.112177546
wavelet-HLL	glcm	Correlation	0.100551485
wavelet-HLL	glcm	Autocorrelation	387.1371795
wavelet-HLL	glcm	SumEntropy	2.031800559
wavelet-HLL	glcm	SumSquares	117.3534669
wavelet-HLL	glcm	ClusterProminence	154422.3307
wavelet-HLL	glcm	Imc2	0.995051549
wavelet-HLL	glcm	Imc1	-0.895169795
wavelet-HLL	glcm	DifferenceAverage	10.91923077
wavelet-HLL	glcm	Id	0.19146362
wavelet-HLL	glcm	ClusterTendency	275.9651496
wavelet-HLL	firstorder	InterquartileRange	544.1159694
wavelet-HLL	firstorder	Skewness	-0.330878524
wavelet-HLL	firstorder	Uniformity	0.112244898
wavelet-HLL	firstorder	Median	108.4339559

wavelet-HLL	firstorder	Energy	1159433.589
wavelet-HLL	firstorder	RobustMeanAbsoluteDeviation	201.8387503
wavelet-HLL	firstorder	MeanAbsoluteDeviation	260.0460068
wavelet-HLL	firstorder	TotalEnergy	31304706.9
wavelet-HLL	firstorder	Maximum	432.0162078
wavelet-HLL	firstorder	RootMeanSquared	287.778882
wavelet-HLL	firstorder	90Percentile	299.3422795
wavelet-HLL	firstorder	Minimum	-423.4255926
wavelet-HLL	firstorder	Entropy	3.235926351
wavelet-HLL	firstorder	Range	855.4418003
wavelet-HLL	firstorder	Variance	82779.53547
wavelet-HLL	firstorder	10Percentile	-394.2369183
wavelet-HLL	firstorder	Kurtosis	1.591561384
wavelet-HLL	firstorder	Mean	6.095034119
wavelet-HLL	glrlm	ShortRunLowGrayLevelEmphasis	0.146123699
wavelet-HLL	glrlm	GrayLevelVariance	130.1282337
wavelet-HLL	glrlm	LowGrayLevelRunEmphasis	0.151250324
wavelet-HLL	glrlm	GrayLevelNonUniformityNormalized	0.112185551
wavelet-HLL	glrlm	RunVariance	0.021607748
wavelet-HLL	glrlm	GrayLevelNonUniformity	1.536207382
wavelet-HLL	glrlm	LongRunEmphasis	1.073964497
wavelet-HLL	glrlm	ShortRunHighGrayLevelEmphasis	450.4204529
wavelet-HLL	glrlm	RunLengthNonUniformity	13.15187377
wavelet-HLL	glrlm	ShortRunEmphasis	0.981508876
wavelet-HLL	glrlm	LongRunHighGrayLevelEmphasis	472.3527754
wavelet-HLL	glrlm	RunPercentage	0.978021978
wavelet-HLL	glrlm	LongRunLowGrayLevelEmphasis	0.171756826
wavelet-HLL	glrlm	RunEntropy	3.237591937
wavelet-HLL	glrlm	HighGrayLevelRunEmphasis	454.8069174
wavelet-HLL	glrlm	RunLengthNonUniformityNormalized	0.956784504
wavelet-HLL	glszm	GrayLevelVariance	126.69
wavelet-HLL	glszm	ZoneVariance	0.24
wavelet-HLL	glszm	GrayLevelNonUniformityNormalized	0.1
wavelet-HLL	glszm	SizeZoneNonUniformityNormalized	0.52
wavelet-HLL	glszm	SizeZoneNonUniformity	5.2
wavelet-HLL	glszm	GrayLevelNonUniformity	1
wavelet-HLL	glszm	LargeAreaEmphasis	2.2
wavelet-HLL	glszm	SmallAreaHighGrayLevelEmphasis	456.675
wavelet-HLL	glszm	ZonePercentage	0.714285714
wavelet-HLL	glszm	LargeAreaLowGrayLevelEmphasis	0.429326425
wavelet-HLL	glszm	LargeAreaHighGrayLevelEmphasis	826.8
wavelet-HLL	glszm	HighGrayLevelZoneEmphasis	530.7
wavelet-HLL	glszm	SmallAreaEmphasis	0.7
wavelet-HLL	glszm	LowGrayLevelZoneEmphasis	0.109329045
wavelet-HLL	glszm	ZoneEntropy	3.321928095
wavelet-HLL	glszm	SmallAreaLowGrayLevelEmphasis	0.0293297
wavelet-HLL	ngtdm	Coarseness	0.08362964
wavelet-HLL	ngtdm	Complexity	1194.437925
wavelet-HLL	ngtdm	Strength	50.55108359
wavelet-HLL	ngtdm	Contrast	21.53781758
wavelet-HLL	ngtdm	Busyness	0.105153745
wavelet-LHL	gldm	GrayLevelVariance	187.6938776
wavelet-LHL	gldm	HighGrayLevelEmphasis	622.7142857

wavelet-LHL	gldm	DependenceEntropy	3.521640636
wavelet-LHL	gldm	DependenceNonUniformity	8.285714286
wavelet-LHL	gldm	GrayLevelNonUniformity	1.571428571
wavelet-LHL	gldm	SmallDependenceEmphasis	0.785714286
wavelet-LHL	gldm	SmallDependenceHighGrayLevelEmphasis	334.5
wavelet-LHL	gldm	DependenceNonUniformityNormalized	0.591836735
wavelet-LHL	gldm	LargeDependenceEmphasis	1.857142857
wavelet-LHL	gldm	LargeDependenceLowGrayLevelEmphasis	0.079516873
wavelet-LHL	gldm	DependenceVariance	0.204081633
wavelet-LHL	gldm	LargeDependenceHighGrayLevelEmphasis	1775.571429
wavelet-LHL	gldm	SmallDependenceLowGrayLevelEmphasis	0.078590145
wavelet-LHL	gldm	LowGrayLevelEmphasis	0.078775491
wavelet-LHL	glcm	JointAverage	20.71955128
wavelet-LHL	glcm	SumAverage	41.43910256
wavelet-LHL	glcm	JointEntropy	3.0478262
wavelet-LHL	glcm	ClusterShade	-456.2263733
wavelet-LHL	glcm	MaximumProbability	0.140705128
wavelet-LHL	glcm	Idmn	0.844633315
wavelet-LHL	glcm	JointEnergy	0.130488782
wavelet-LHL	glcm	Contrast	408.3762821
wavelet-LHL	glcm	DifferenceEntropy	1.974542025
wavelet-LHL	glcm	InverseVariance	0.052574649
wavelet-LHL	glcm	DifferenceVariance	69.94369124
wavelet-LHL	glcm	Idn	0.7451247
wavelet-LHL	glcm	Idm	0.065098001
wavelet-LHL	glcm	Correlation	-0.109626766
wavelet-LHL	glcm	Autocorrelation	424.7153846
wavelet-LHL	glcm	SumEntropy	2.018980046
wavelet-LHL	glcm	SumSquares	185.8259014
wavelet-LHL	glcm	ClusterProminence	254629.8492
wavelet-LHL	glcm	Imc2	0.996155555
wavelet-LHL	glcm	Imc1	-0.917713221
wavelet-LHL	glcm	DifferenceAverage	16.79423077
wavelet-LHL	glcm	Id	0.129345871
wavelet-LHL	glcm	ClusterTendency	334.9273237
wavelet-LHL	firstorder	InterquartileRange	510.3712911
wavelet-LHL	firstorder	Skewness	0.191305817
wavelet-LHL	firstorder	Uniformity	0.112244898
wavelet-LHL	firstorder	Median	22.66556479
wavelet-LHL	firstorder	Energy	1675786.218
wavelet-LHL	firstorder	RobustMeanAbsoluteDeviation	247.9485672
wavelet-LHL	firstorder	MeanAbsoluteDeviation	320.7307846
wavelet-LHL	firstorder	TotalEnergy	45246227.88
wavelet-LHL	firstorder	Maximum	574.0651467
wavelet-LHL	firstorder	RootMeanSquared	345.9754551
wavelet-LHL	firstorder	90Percentile	485.7899297
wavelet-LHL	firstorder	Minimum	-493.0221614
wavelet-LHL	firstorder	Entropy	3.324862958
wavelet-LHL	firstorder	Range	1067.087308
wavelet-LHL	firstorder	Variance	119589.6115
wavelet-LHL	firstorder	10Percentile	-369.549198
wavelet-LHL	firstorder	Kurtosis	1.66088208
wavelet-LHL	firstorder	Mean	10.45964136

wavelet-LHL	glrlm	ShortRunLowGrayLevelEmphasis	0.079689832
wavelet-LHL	glrlm	GrayLevelVariance	186.3681922
wavelet-LHL	glrlm	LowGrayLevelRunEmphasis	0.079697509
wavelet-LHL	glrlm	GrayLevelNonUniformityNormalized	0.111362433
wavelet-LHL	glrlm	RunVariance	0.010923987
wavelet-LHL	glrlm	GrayLevelNonUniformity	1.542688081
wavelet-LHL	glrlm	LongRunEmphasis	1.035502959
wavelet-LHL	glrlm	ShortRunHighGrayLevelEmphasis	602.228656
wavelet-LHL	glrlm	RunLengthNonUniformity	13.56213018
wavelet-LHL	glrlm	ShortRunEmphasis	0.99112426
wavelet-LHL	glrlm	LongRunHighGrayLevelEmphasis	661.9180051
wavelet-LHL	glrlm	RunPercentage	0.989010989
wavelet-LHL	glrlm	LongRunLowGrayLevelEmphasis	0.079728217
wavelet-LHL	glrlm	RunEntropy	3.342674249
wavelet-LHL	glrlm	HighGrayLevelRunEmphasis	614.1665258
wavelet-LHL	glrlm	RunLengthNonUniformityNormalized	0.978152025
wavelet-LHL	glszm	GrayLevelVariance	166.2222222
wavelet-LHL	glszm	ZoneVariance	0.138888889
wavelet-LHL	glszm	GrayLevelNonUniformityNormalized	0.097222222
wavelet-LHL	glszm	SizeZoneNonUniformityNormalized	0.722222222
wavelet-LHL	glszm	SizeZoneNonUniformity	8.666666667
wavelet-LHL	glszm	GrayLevelNonUniformity	1.166666667
wavelet-LHL	glszm	LargeAreaEmphasis	1.5
wavelet-LHL	glszm	SmallAreaHighGrayLevelEmphasis	334.2083333
wavelet-LHL	glszm	ZonePercentage	0.857142857
wavelet-LHL	glszm	LargeAreaLowGrayLevelEmphasis	0.092193055
wavelet-LHL	glszm	LargeAreaHighGrayLevelEmphasis	1174.833333
wavelet-LHL	glszm	HighGrayLevelZoneEmphasis	502.3333333
wavelet-LHL	glszm	SmallAreaEmphasis	0.875
wavelet-LHL	glszm	LowGrayLevelZoneEmphasis	0.091760581
wavelet-LHL	glszm	ZoneEntropy	3.584962501
wavelet-LHL	glszm	SmallAreaLowGrayLevelEmphasis	0.091652463
wavelet-LHL	ngtdm	Coarseness	0.054474708
wavelet-LHL	ngtdm	Complexity	2437.327338
wavelet-LHL	ngtdm	Strength	49.10673754
wavelet-LHL	ngtdm	Contrast	40.40003345
wavelet-LHL	ngtdm	Busyness	0.073428571
wavelet-LHH	gldm	GrayLevelVariance	61.26530612
wavelet-LHH	gldm	HighGrayLevelEmphasis	185.4285714
wavelet-LHH	gldm	DependenceEntropy	3.664497779
wavelet-LHH	gldm	DependenceNonUniformity	10.57142857
wavelet-LHH	gldm	GrayLevelNonUniformity	1.142857143
wavelet-LHH	gldm	SmallDependenceEmphasis	0.892857143
wavelet-LHH	gldm	SmallDependenceHighGrayLevelEmphasis	178.5714286
wavelet-LHH	gldm	DependenceNonUniformityNormalized	0.755102041
wavelet-LHH	gldm	LargeDependenceEmphasis	1.428571429
wavelet-LHH	gldm	LargeDependenceLowGrayLevelEmphasis	0.113059758
wavelet-LHH	gldm	DependenceVariance	0.12244898
wavelet-LHH	gldm	LargeDependenceHighGrayLevelEmphasis	212.8571429
wavelet-LHH	gldm	SmallDependenceLowGrayLevelEmphasis	0.104689222
wavelet-LHH	gldm	LowGrayLevelEmphasis	0.106363329
wavelet-LHH	glcm	JointAverage	11.37083333
wavelet-LHH	glcm	SumAverage	22.74166667

wavelet-LHH	glcm	JointEntropy	3.076672354
wavelet-LHH	glcm	ClusterShade	49.75133752
wavelet-LHH	glcm	MaximumProbability	0.135897436
wavelet-LHH	glcm	Idmn	0.885031775
wavelet-LHH	glcm	JointEnergy	0.128685897
wavelet-LHH	glcm	Contrast	136.2224359
wavelet-LHH	glcm	DifferenceEntropy	1.921977923
wavelet-LHH	glcm	InverseVariance	0.090892469
wavelet-LHH	glcm	DifferenceVariance	38.87467415
wavelet-LHH	glcm	Idn	0.780945118
wavelet-LHH	glcm	Idm	0.081787553
wavelet-LHH	glcm	Correlation	-0.317438374
wavelet-LHH	glcm	Autocorrelation	123.1628205
wavelet-LHH	glcm	SumEntropy	1.96256979
wavelet-LHH	glcm	SumSquares	52.43126469
wavelet-LHH	glcm	ClusterProminence	16943.69321
wavelet-LHH	glcm	Imc2	0.997811269
wavelet-LHH	glcm	Imc1	-0.970296312
wavelet-LHH	glcm	DifferenceAverage	9.282692308
wavelet-LHH	glcm	Id	0.171425457
wavelet-LHH	glcm	ClusterTendency	73.50262286
wavelet-LHH	firstorder	InterquartileRange	250.0850004
wavelet-LHH	firstorder	Skewness	0.692440212
wavelet-LHH	firstorder	Uniformity	0.081632653
wavelet-LHH	firstorder	Median	5.427713947
wavelet-LHH	firstorder	Energy	578205.2661
wavelet-LHH	firstorder	RobustMeanAbsoluteDeviation	104.7903966
wavelet-LHH	firstorder	MeanAbsoluteDeviation	164.2150835
wavelet-LHH	firstorder	TotalEnergy	15611542.18
wavelet-LHH	firstorder	Maximum	488.3643101
wavelet-LHH	firstorder	RootMeanSquared	203.2249398
wavelet-LHH	firstorder	90Percentile	294.4083542
wavelet-LHH	firstorder	Minimum	-218.8322862
wavelet-LHH	firstorder	Entropy	3.664497779
wavelet-LHH	firstorder	Range	707.1965963
wavelet-LHH	firstorder	Variance	39805.1812
wavelet-LHH	firstorder	10Percentile	-192.0463784
wavelet-LHH	firstorder	Kurtosis	2.739656119
wavelet-LHH	firstorder	Mean	38.66775073
wavelet-LHH	glrlm	ShortRunLowGrayLevelEmphasis	0.1068309
wavelet-LHH	glrlm	GrayLevelVariance	61.56487975
wavelet-LHH	glrlm	LowGrayLevelRunEmphasis	0.106900242
wavelet-LHH	glrlm	GrayLevelNonUniformityNormalized	0.081270378
wavelet-LHH	glrlm	RunVariance	0.005461994
wavelet-LHH	glrlm	GrayLevelNonUniformity	1.131868132
wavelet-LHH	glrlm	LongRunEmphasis	1.017751479
wavelet-LHH	glrlm	ShortRunHighGrayLevelEmphasis	185.86306
wavelet-LHH	glrlm	RunLengthNonUniformity	13.78106509
wavelet-LHH	glrlm	ShortRunEmphasis	0.99556213
wavelet-LHH	glrlm	LongRunHighGrayLevelEmphasis	187.2831784
wavelet-LHH	glrlm	RunPercentage	0.994505495
wavelet-LHH	glrlm	LongRunLowGrayLevelEmphasis	0.107177609
wavelet-LHH	glrlm	RunEntropy	3.667262544

wavelet-LHH	glrlm	HighGrayLevelRunEmphasis	186.1470837
wavelet-LHH	glrlm	RunLengthNonUniformityNormalized	0.989076013
wavelet-LHH	glszm	GrayLevelVariance	65.15976331
wavelet-LHH	glszm	ZoneVariance	0.071005917
wavelet-LHH	glszm	GrayLevelNonUniformityNormalized	0.076923077
wavelet-LHH	glszm	SizeZoneNonUniformityNormalized	0.857988166
wavelet-LHH	glszm	SizeZoneNonUniformity	11.15384615
wavelet-LHH	glszm	GrayLevelNonUniformity	1
wavelet-LHH	glszm	LargeAreaEmphasis	1.230769231
wavelet-LHH	glszm	SmallAreaHighGrayLevelEmphasis	191.0769231
wavelet-LHH	glszm	ZonePercentage	0.928571429
wavelet-LHH	glszm	LargeAreaLowGrayLevelEmphasis	0.11694897
wavelet-LHH	glszm	LargeAreaHighGrayLevelEmphasis	209.5384615
wavelet-LHH	glszm	HighGrayLevelZoneEmphasis	194.7692308
wavelet-LHH	glszm	SmallAreaEmphasis	0.942307692
wavelet-LHH	glszm	LowGrayLevelZoneEmphasis	0.1133432
wavelet-LHH	glszm	ZoneEntropy	3.700439718
wavelet-LHH	glszm	SmallAreaLowGrayLevelEmphasis	0.112441758
wavelet-LHH	ngtdm	Coarseness	0.125166303
wavelet-LHH	ngtdm	Complexity	1039.13407
wavelet-LHH	ngtdm	Strength	31.46062297
wavelet-LHH	ngtdm	Contrast	5.864512694
wavelet-LHH	ngtdm	Busyness	0.073201041
wavelet-LLH	gldm	GrayLevelVariance	108.7755102
wavelet-LLH	gldm	HighGrayLevelEmphasis	355.7142857
wavelet-LLH	gldm	DependenceEntropy	3.664497779
wavelet-LLH	gldm	DependenceNonUniformity	10.57142857
wavelet-LLH	gldm	GrayLevelNonUniformity	1.142857143
wavelet-LLH	gldm	SmallDependenceEmphasis	0.892857143
wavelet-LLH	gldm	SmallDependenceHighGrayLevelEmphasis	350.4642857
wavelet-LLH	gldm	DependenceNonUniformityNormalized	0.755102041
wavelet-LLH	gldm	LargeDependenceEmphasis	1.428571429
wavelet-LLH	gldm	LargeDependenceLowGrayLevelEmphasis	0.095944074
wavelet-LLH	gldm	DependenceVariance	0.12244898
wavelet-LLH	gldm	LargeDependenceHighGrayLevelEmphasis	376.7142857
wavelet-LLH	gldm	SmallDependenceLowGrayLevelEmphasis	0.08501113
wavelet-LLH	gldm	LowGrayLevelEmphasis	0.087197719
wavelet-LLH	glcm	JointAverage	16.46762821
wavelet-LLH	glcm	SumAverage	32.93525641
wavelet-LLH	glcm	JointEntropy	3.080518508
wavelet-LLH	glcm	ClusterShade	941.3487971
wavelet-LLH	glcm	MaximumProbability	0.133974359
wavelet-LLH	glcm	Idmn	0.82011063
wavelet-LLH	glcm	JointEnergy	0.127820513
wavelet-LLH	glcm	Contrast	322.4019231
wavelet-LLH	glcm	DifferenceEntropy	1.954877482
wavelet-LLH	glcm	InverseVariance	0.035765948
wavelet-LLH	glcm	DifferenceVariance	33.89305021
wavelet-LLH	glcm	Idn	0.716806679
wavelet-LLH	glcm	Idm	0.042893459
wavelet-LLH	glcm	Correlation	-0.341800051
wavelet-LLH	glcm	Autocorrelation	231.0564103
wavelet-LLH	glcm	SumEntropy	2.018980046

wavelet-LLH	glcm	SumSquares	117.4755596
wavelet-LLH	glcm	ClusterProminence	80143.34583
wavelet-LLH	glcm	Imc2	0.997639633
wavelet-LLH	glcm	Imc1	-0.960891446
wavelet-LLH	glcm	DifferenceAverage	15.46730769
wavelet-LLH	glcm	Id	0.110554856
wavelet-LLH	glcm	ClusterTendency	147.5003152
wavelet-LLH	firstorder	InterquartileRange	386.5746601
wavelet-LLH	firstorder	Skewness	0.340612728
wavelet-LLH	firstorder	Uniformity	0.081632653
wavelet-LLH	firstorder	Median	78.7805313
wavelet-LLH	firstorder	Energy	1046827.15
wavelet-LLH	firstorder	RobustMeanAbsoluteDeviation	152.7178351
wavelet-LLH	firstorder	MeanAbsoluteDeviation	222.8484378
wavelet-LLH	firstorder	TotalEnergy	28264333.06
wavelet-LLH	firstorder	Maximum	567.3203821
wavelet-LLH	firstorder	RootMeanSquared	273.447194
wavelet-LLH	firstorder	90Percentile	447.5790465
wavelet-LLH	firstorder	Minimum	-291.6866362
wavelet-LLH	firstorder	Entropy	3.664497779
wavelet-LLH	firstorder	Range	859.0070183
wavelet-LLH	firstorder	Variance	68094.72636
wavelet-LLH	firstorder	10Percentile	-222.6555327
wavelet-LLH	firstorder	Kurtosis	2.016435031
wavelet-LLH	firstorder	Mean	81.7229559
wavelet-LLH	glrlm	ShortRunLowGrayLevelEmphasis	0.087502354
wavelet-LLH	glrlm	GrayLevelVariance	108.9352457
wavelet-LLH	glrlm	LowGrayLevelRunEmphasis	0.087592923
wavelet-LLH	glrlm	GrayLevelNonUniformityNormalized	0.081270378
wavelet-LLH	glrlm	RunVariance	0.005461994
wavelet-LLH	glrlm	GrayLevelNonUniformity	1.131868132
wavelet-LLH	glrlm	LongRunEmphasis	1.017751479
wavelet-LLH	glrlm	ShortRunHighGrayLevelEmphasis	357.3117075
wavelet-LLH	glrlm	RunLengthNonUniformity	13.78106509
wavelet-LLH	glrlm	ShortRunEmphasis	0.99556213
wavelet-LLH	glrlm	LongRunHighGrayLevelEmphasis	358.3989856
wavelet-LLH	glrlm	RunPercentage	0.994505495
wavelet-LLH	glrlm	LongRunLowGrayLevelEmphasis	0.087955198
wavelet-LLH	glrlm	RunEntropy	3.667262544
wavelet-LLH	glrlm	HighGrayLevelRunEmphasis	357.5291631
wavelet-LLH	glrlm	RunLengthNonUniformityNormalized	0.989076013
wavelet-LLH	glszm	GrayLevelVariance	110.852071
wavelet-LLH	glszm	ZoneVariance	0.071005917
wavelet-LLH	glszm	GrayLevelNonUniformityNormalized	0.076923077
wavelet-LLH	glszm	SizeZoneNonUniformityNormalized	0.857988166
wavelet-LLH	glszm	SizeZoneNonUniformity	11.15384615
wavelet-LLH	glszm	GrayLevelNonUniformity	1
wavelet-LLH	glszm	LargeAreaEmphasis	1.230769231
wavelet-LLH	glszm	SmallAreaHighGrayLevelEmphasis	376.4807692
wavelet-LLH	glszm	ZonePercentage	0.928571429
wavelet-LLH	glszm	LargeAreaLowGrayLevelEmphasis	0.097044953
wavelet-LLH	glszm	LargeAreaHighGrayLevelEmphasis	390.6153846
wavelet-LLH	glszm	HighGrayLevelZoneEmphasis	379.3076923

wavelet-LLH	glszm	SmallAreaEmphasis	0.942307692
wavelet-LLH	glszm	LowGrayLevelZoneEmphasis	0.092335377
wavelet-LLH	glszm	ZoneEntropy	3.700439718
wavelet-LLH	glszm	SmallAreaLowGrayLevelEmphasis	0.091157983
wavelet-LLH	ngtdm	Coarseness	0.089121291
wavelet-LLH	ngtdm	Complexity	1774.61593
wavelet-LLH	ngtdm	Strength	41.61274404
wavelet-LLH	ngtdm	Contrast	13.69714297
wavelet-LLH	ngtdm	Busyness	0.079821792
wavelet-LLL	gldm	GrayLevelVariance	177.2040816
wavelet-LLL	gldm	HighGrayLevelEmphasis	890.8571429
wavelet-LLL	gldm	DependenceEntropy	3.324862958
wavelet-LLL	gldm	DependenceNonUniformity	6.714285714
wavelet-LLL	gldm	GrayLevelNonUniformity	1.571428571
wavelet-LLL	gldm	SmallDependenceEmphasis	0.702380952
wavelet-LLL	gldm	SmallDependenceHighGrayLevelEmphasis	662.4285714
wavelet-LLL	gldm	DependenceNonUniformityNormalized	0.479591837
wavelet-LLL	gldm	LargeDependenceEmphasis	3.142857143
wavelet-LLL	gldm	LargeDependenceLowGrayLevelEmphasis	0.077259691
wavelet-LLL	gldm	DependenceVariance	0.673469388
wavelet-LLL	gldm	LargeDependenceHighGrayLevelEmphasis	2841.714286
wavelet-LLL	gldm	SmallDependenceLowGrayLevelEmphasis	0.072777362
wavelet-LLL	gldm	LowGrayLevelEmphasis	0.073498919
wavelet-LLL	glcm	JointAverage	29.44134615
wavelet-LLL	glcm	SumAverage	58.88269231
wavelet-LLL	glcm	JointEntropy	3.035005687
wavelet-LLL	glcm	ClusterShade	-556.399746
wavelet-LLL	glcm	MaximumProbability	0.156730769
wavelet-LLL	glcm	Idmn	0.893961823
wavelet-LLL	glcm	JointEnergy	0.133293269
wavelet-LLL	glcm	Contrast	322.6903846
wavelet-LLL	glcm	DifferenceEntropy	1.936928764
wavelet-LLL	glcm	InverseVariance	0.062392851
wavelet-LLL	glcm	DifferenceVariance	104.8825588
wavelet-LLL	glcm	Idn	0.793766886
wavelet-LLL	glcm	Idm	0.104645476
wavelet-LLL	glcm	Correlation	-0.082956349
wavelet-LLL	glcm	Autocorrelation	857.9442308
wavelet-LLL	glcm	SumEntropy	2.070262097
wavelet-LLL	glcm	SumSquares	147.9971568
wavelet-LLL	glcm	ClusterProminence	136369.8708
wavelet-LLL	glcm	Imc2	0.994187518
wavelet-LLL	glcm	Imc1	-0.87963057
wavelet-LLL	glcm	DifferenceAverage	14.26602564
wavelet-LLL	glcm	Id	0.171974232
wavelet-LLL	glcm	ClusterTendency	269.2982425
wavelet-LLL	firstorder	InterquartileRange	492.8033416
wavelet-LLL	firstorder	Skewness	-0.19540392
wavelet-LLL	firstorder	Uniformity	0.112244898
wavelet-LLL	firstorder	Median	-1165.954412
wavelet-LLL	firstorder	Energy	25125891.4
wavelet-LLL	firstorder	RobustMeanAbsoluteDeviation	222.7981013
wavelet-LLL	firstorder	MeanAbsoluteDeviation	298.6887959

wavelet-LLL	firstorder	TotalEnergy	678399067.8
wavelet-LLL	firstorder	Maximum	-773.5859349
wavelet-LLL	firstorder	RootMeanSquared	1339.666574
wavelet-LLL	firstorder	90Percentile	-886.5622198
wavelet-LLL	firstorder	Minimum	-1933.843033
wavelet-LLL	firstorder	Entropy	3.324862958
wavelet-LLL	firstorder	Range	1160.257099
wavelet-LLL	firstorder	Variance	110959.1299
wavelet-LLL	firstorder	10Percentile	-1638.352163
wavelet-LLL	firstorder	Kurtosis	1.950854794
wavelet-LLL	firstorder	Mean	-1297.592925
wavelet-LLL	glrlm	ShortRunLowGrayLevelEmphasis	0.07515718
wavelet-LLL	glrlm	GrayLevelVariance	179.6128394
wavelet-LLL	glrlm	LowGrayLevelRunEmphasis	0.075192048
wavelet-LLL	glrlm	GrayLevelNonUniformityNormalized	0.109569636
wavelet-LLL	glrlm	RunVariance	0.021847975
wavelet-LLL	glrlm	GrayLevelNonUniformity	1.502113271
wavelet-LLL	glrlm	LongRunEmphasis	1.071005917
wavelet-LLL	glrlm	ShortRunHighGrayLevelEmphasis	876.0830516
wavelet-LLL	glrlm	RunLengthNonUniformity	13.12426036
wavelet-LLL	glrlm	ShortRunEmphasis	0.982248521
wavelet-LLL	glrlm	LongRunHighGrayLevelEmphasis	952.9247675
wavelet-LLL	glrlm	RunPercentage	0.978021978
wavelet-LLL	glrlm	LongRunLowGrayLevelEmphasis	0.075331519
wavelet-LLL	glrlm	RunEntropy	3.37678665
wavelet-LLL	glrlm	HighGrayLevelRunEmphasis	891.4513948
wavelet-LLL	glrlm	RunLengthNonUniformityNormalized	0.956304051
wavelet-LLL	glszm	GrayLevelVariance	203.6528926
wavelet-LLL	glszm	ZoneVariance	0.380165289
wavelet-LLL	glszm	GrayLevelNonUniformityNormalized	0.090909091
wavelet-LLL	glszm	SizeZoneNonUniformityNormalized	0.685950413
wavelet-LLL	glszm	SizeZoneNonUniformity	7.545454545
wavelet-LLL	glszm	GrayLevelNonUniformity	1
wavelet-LLL	glszm	LargeAreaEmphasis	2
wavelet-LLL	glszm	SmallAreaHighGrayLevelEmphasis	816.6363636
wavelet-LLL	glszm	ZonePercentage	0.785714286
wavelet-LLL	glszm	LargeAreaLowGrayLevelEmphasis	0.094972599
wavelet-LLL	glszm	LargeAreaHighGrayLevelEmphasis	1763.454545
wavelet-LLL	glszm	HighGrayLevelZoneEmphasis	918
wavelet-LLL	glszm	SmallAreaEmphasis	0.851010101
wavelet-LLL	glszm	LowGrayLevelZoneEmphasis	0.092913298
wavelet-LLL	glszm	ZoneEntropy	3.459431619
wavelet-LLL	glszm	SmallAreaLowGrayLevelEmphasis	0.092491227
wavelet-LLL	ngtdm	Coarseness	0.069368253
wavelet-LLL	ngtdm	Complexity	2305.673158
wavelet-LLL	ngtdm	Strength	51.26176256
wavelet-LLL	ngtdm	Contrast	37.61614163
wavelet-LLL	ngtdm	Busyness	0.066127598
wavelet-HHL	gldm	GrayLevelVariance	19.22959184
wavelet-HHL	gldm	HighGrayLevelEmphasis	77.64285714
wavelet-HHL	gldm	DependenceEntropy	3.378783493
wavelet-HHL	gldm	DependenceNonUniformity	8.285714286
wavelet-HHL	gldm	GrayLevelNonUniformity	1.428571429

wavelet-HHL	gldm	SmallDependenceEmphasis	0.785714286
wavelet-HHL	gldm	SmallDependenceHighGrayLevelEmphasis	70.35714286
wavelet-HHL	gldm	DependenceNonUniformityNormalized	0.591836735
wavelet-HHL	gldm	LargeDependenceEmphasis	1.857142857
wavelet-HHL	gldm	LargeDependenceLowGrayLevelEmphasis	0.239169988
wavelet-HHL	gldm	DependenceVariance	0.204081633
wavelet-HHL	gldm	LargeDependenceHighGrayLevelEmphasis	106.7857143
wavelet-HHL	gldm	SmallDependenceLowGrayLevelEmphasis	0.096870881
wavelet-HHL	gldm	LowGrayLevelEmphasis	0.125330703
wavelet-HHL	glcm	JointAverage	7.307692308
wavelet-HHL	glcm	SumAverage	14.61538462
wavelet-HHL	glcm	JointEntropy	3.063851841
wavelet-HHL	glcm	ClusterShade	-3.860924145
wavelet-HHL	glcm	MaximumProbability	0.142307692
wavelet-HHL	glcm	Idmn	0.85162489
wavelet-HHL	glcm	JointEnergy	0.129754274
wavelet-HHL	glcm	Contrast	42.63589744
wavelet-HHL	glcm	DifferenceEntropy	1.824686617
wavelet-HHL	glcm	InverseVariance	0.240838855
wavelet-HHL	glcm	DifferenceVariance	9.721826923
wavelet-HHL	glcm	Idn	0.754353808
wavelet-HHL	glcm	Idm	0.170500559
wavelet-HHL	glcm	Correlation	-0.071718612
wavelet-HHL	glcm	Autocorrelation	51.94807692
wavelet-HHL	glcm	SumEntropy	1.981800559
wavelet-HHL	glcm	SumSquares	19.0592281
wavelet-HHL	glcm	ClusterProminence	2580.999898
wavelet-HHL	glcm	Imc2	0.991687418
wavelet-HHL	glcm	Imc1	-0.885640001
wavelet-HHL	glcm	DifferenceAverage	5.276923077
wavelet-HHL	glcm	Id	0.257360517
wavelet-HHL	glcm	ClusterTendency	33.60101496
wavelet-HHL	firstorder	InterquartileRange	203.9338031
wavelet-HHL	firstorder	Skewness	-0.041336484
wavelet-HHL	firstorder	Uniformity	0.102040816
wavelet-HHL	firstorder	Median	30.19755645
wavelet-HHL	firstorder	Energy	177719.83
wavelet-HHL	firstorder	RobustMeanAbsoluteDeviation	71.97380458
wavelet-HHL	firstorder	MeanAbsoluteDeviation	94.63231802
wavelet-HHL	firstorder	TotalEnergy	4798435.409
wavelet-HHL	firstorder	Maximum	192.1366739
wavelet-HHL	firstorder	RootMeanSquared	112.6688669
wavelet-HHL	firstorder	90Percentile	157.8928915
wavelet-HHL	firstorder	Minimum	-133.6551542
wavelet-HHL	firstorder	Entropy	3.378783493
wavelet-HHL	firstorder	Range	325.7918281
wavelet-HHL	firstorder	Variance	12040.2181
wavelet-HHL	firstorder	10Percentile	-119.3477363
wavelet-HHL	firstorder	Kurtosis	1.612954809
wavelet-HHL	firstorder	Mean	25.57450813
wavelet-HHL	glrlm	ShortRunLowGrayLevelEmphasis	0.124063351
wavelet-HHL	glrlm	GrayLevelVariance	19.25344161
wavelet-HHL	glrlm	LowGrayLevelRunEmphasis	0.125242161

wavelet-HHL	glrlm	GrayLevelNonUniformityNormalized	0.101817878
wavelet-HHL	glrlm	RunVariance	0.010923987
wavelet-HHL	glrlm	GrayLevelNonUniformity	1.409974641
wavelet-HHL	glrlm	LongRunEmphasis	1.035502959
wavelet-HHL	glrlm	ShortRunHighGrayLevelEmphasis	77.85756551
wavelet-HHL	glrlm	RunLengthNonUniformity	13.56213018
wavelet-HHL	glrlm	ShortRunEmphasis	0.99112426
wavelet-HHL	glrlm	LongRunHighGrayLevelEmphasis	79.36644125
wavelet-HHL	glrlm	RunPercentage	0.989010989
wavelet-HHL	glrlm	LongRunLowGrayLevelEmphasis	0.129957397
wavelet-HHL	glrlm	RunEntropy	3.380931788
wavelet-HHL	glrlm	HighGrayLevelRunEmphasis	78.15934066
wavelet-HHL	glrlm	RunLengthNonUniformityNormalized	0.978152025
wavelet-HHL	glszm	GrayLevelVariance	19.57638889
wavelet-HHL	glszm	ZoneVariance	0.138888889
wavelet-HHL	glszm	GrayLevelNonUniformityNormalized	0.097222222
wavelet-HHL	glszm	SizeZoneNonUniformityNormalized	0.722222222
wavelet-HHL	glszm	SizeZoneNonUniformity	8.666666667
wavelet-HHL	glszm	GrayLevelNonUniformity	1.166666667
wavelet-HHL	glszm	LargeAreaEmphasis	1.5
wavelet-HHL	glszm	SmallAreaHighGrayLevelEmphasis	80.66666667
wavelet-HHL	glszm	ZonePercentage	0.857142857
wavelet-HHL	glszm	LargeAreaLowGrayLevelEmphasis	0.190489986
wavelet-HHL	glszm	LargeAreaHighGrayLevelEmphasis	101.9166667
wavelet-HHL	glszm	HighGrayLevelZoneEmphasis	84.91666667
wavelet-HHL	glszm	SmallAreaEmphasis	0.875
wavelet-HHL	glszm	LowGrayLevelZoneEmphasis	0.124083736
wavelet-HHL	glszm	ZoneEntropy	3.418295834
wavelet-HHL	glszm	SmallAreaLowGrayLevelEmphasis	0.107482174
wavelet-HHL	ngtdm	Coarseness	0.174106151
wavelet-HHL	ngtdm	Complexity	258.0620748
wavelet-HHL	ngtdm	Strength	15.25881081
wavelet-HHL	ngtdm	Contrast	1.375028792
wavelet-HHL	ngtdm	Busyness	0.088949905
wavelet-HLH	gldm	GrayLevelVariance	25.48979592
wavelet-HLH	gldm	HighGrayLevelEmphasis	85
wavelet-HLH	gldm	DependenceEntropy	3.521640636
wavelet-HLH	gldm	DependenceNonUniformity	10.57142857
wavelet-HLH	gldm	GrayLevelNonUniformity	1.285714286
wavelet-HLH	gldm	SmallDependenceEmphasis	0.892857143
wavelet-HLH	gldm	SmallDependenceHighGrayLevelEmphasis	84.03571429
wavelet-HLH	gldm	DependenceNonUniformityNormalized	0.755102041
wavelet-HLH	gldm	LargeDependenceEmphasis	1.428571429
wavelet-HLH	gldm	LargeDependenceLowGrayLevelEmphasis	0.166038346
wavelet-HLH	gldm	DependenceVariance	0.12244898
wavelet-HLH	gldm	LargeDependenceHighGrayLevelEmphasis	88.85714286
wavelet-HLH	gldm	SmallDependenceLowGrayLevelEmphasis	0.106514537
wavelet-HLH	gldm	LowGrayLevelEmphasis	0.118419298
wavelet-HLH	glcm	JointAverage	7.213141026
wavelet-HLH	glcm	SumAverage	14.42628205
wavelet-HLH	glcm	JointEntropy	3.070262097
wavelet-HLH	glcm	ClusterShade	87.85724938
wavelet-HLH	glcm	MaximumProbability	0.139102564

wavelet-HLH	glcm	Idmn	0.884775381
wavelet-HLH	glcm	JointEnergy	0.130555556
wavelet-HLH	glcm	Contrast	57.64294872
wavelet-HLH	glcm	DifferenceEntropy	1.858723636
wavelet-HLH	glcm	InverseVariance	0.165929987
wavelet-HLH	glcm	DifferenceVariance	14.48033654
wavelet-HLH	glcm	Idn	0.779099591
wavelet-HLH	glcm	Idm	0.129358334
wavelet-HLH	glcm	Correlation	-0.328501277
wavelet-HLH	glcm	Autocorrelation	47.60961538
wavelet-HLH	glcm	SumEntropy	1.910005687
wavelet-HLH	glcm	SumSquares	20.77347089
wavelet-HLH	glcm	ClusterProminence	2765.187341
wavelet-HLH	glcm	Imc2	0.997085323
wavelet-HLH	glcm	Imc1	-0.942068175
wavelet-HLH	glcm	DifferenceAverage	6.109615385
wavelet-HLH	glcm	Id	0.224857621
wavelet-HLH	glcm	ClusterTendency	25.45093483
wavelet-HLH	firstorder	InterquartileRange	172.9857674
wavelet-HLH	firstorder	Skewness	0.698788231
wavelet-HLH	firstorder	Uniformity	0.091836735
wavelet-HLH	firstorder	Median	7.536349735
wavelet-HLH	firstorder	Energy	225008.6487
wavelet-HLH	firstorder	RobustMeanAbsoluteDeviation	67.75933765
wavelet-HLH	firstorder	MeanAbsoluteDeviation	102.7654575
wavelet-HLH	firstorder	TotalEnergy	6075233.515
wavelet-HLH	firstorder	Maximum	295.5367848
wavelet-HLH	firstorder	RootMeanSquared	126.7755747
wavelet-HLH	firstorder	90Percentile	156.2047351
wavelet-HLH	firstorder	Minimum	-155.547354
wavelet-HLH	firstorder	Entropy	3.521640636
wavelet-HLH	firstorder	Range	451.0841388
wavelet-HLH	firstorder	Variance	16059.74546
wavelet-HLH	firstorder	10Percentile	-140.5790801
wavelet-HLH	firstorder	Kurtosis	2.770027135
wavelet-HLH	firstorder	Mean	3.507260843
wavelet-HLH	glrlm	ShortRunLowGrayLevelEmphasis	0.117969446
wavelet-HLH	glrlm	GrayLevelVariance	25.49900142
wavelet-HLH	glrlm	LowGrayLevelRunEmphasis	0.118462542
wavelet-HLH	glrlm	GrayLevelNonUniformityNormalized	0.091599863
wavelet-HLH	glrlm	RunVariance	0.005461994
wavelet-HLH	glrlm	GrayLevelNonUniformity	1.275570583
wavelet-HLH	glrlm	LongRunEmphasis	1.017751479
wavelet-HLH	glrlm	ShortRunHighGrayLevelEmphasis	85.40976331
wavelet-HLH	glrlm	RunLengthNonUniformity	13.78106509
wavelet-HLH	glrlm	ShortRunEmphasis	0.99556213
wavelet-HLH	glrlm	LongRunHighGrayLevelEmphasis	85.60946746
wavelet-HLH	glrlm	RunPercentage	0.994505495
wavelet-HLH	glrlm	LongRunLowGrayLevelEmphasis	0.120434929
wavelet-HLH	glrlm	RunEntropy	3.523560092
wavelet-HLH	glrlm	HighGrayLevelRunEmphasis	85.44970414
wavelet-HLH	glrlm	RunLengthNonUniformityNormalized	0.989076013
wavelet-HLH	glszm	GrayLevelVariance	25.60946746

wavelet-HLH	glszm	ZoneVariance	0.071005917
wavelet-HLH	glszm	GrayLevelNonUniformityNormalized	0.088757396
wavelet-HLH	glszm	SizeZoneNonUniformityNormalized	0.857988166
wavelet-HLH	glszm	SizeZoneNonUniformity	11.15384615
wavelet-HLH	glszm	GrayLevelNonUniformity	1.153846154
wavelet-HLH	glszm	LargeAreaEmphasis	1.230769231
wavelet-HLH	glszm	SmallAreaHighGrayLevelEmphasis	90.32692308
wavelet-HLH	glszm	ZonePercentage	0.928571429
wavelet-HLH	glszm	LargeAreaLowGrayLevelEmphasis	0.144622492
wavelet-HLH	glszm	LargeAreaHighGrayLevelEmphasis	92.92307692
wavelet-HLH	glszm	HighGrayLevelZoneEmphasis	90.84615385
wavelet-HLH	glszm	SmallAreaEmphasis	0.942307692
wavelet-HLH	glszm	LowGrayLevelZoneEmphasis	0.118981467
wavelet-HLH	glszm	ZoneEntropy	3.546593564
wavelet-HLH	glszm	SmallAreaLowGrayLevelEmphasis	0.11257121
wavelet-HLH	ngtdm	Coarseness	0.176074263
wavelet-HLH	ngtdm	Complexity	391.8897392
wavelet-HLH	ngtdm	Strength	19.30706962
wavelet-HLH	ngtdm	Contrast	1.825625844
wavelet-HLH	ngtdm	Busyness	0.084587133

Image type	Feature Class	Feature Name	5: RTSTRUCT: C99-DNU_segment_RLL ITV
general	info	BoundingBox	(0, 0, 1, 6, 6, 5)
general	info	EnabledImageTypes	{u'Original': {}, u'Wavelet': {}}
general	info	GeneralSettings	{'distances': [1], 'additionalInfo': True, 'force2D': False, 'interpolator': 'sitkBSpline', 'resampledPixelSpacing': [3.0], 'label': 1, 'normalizeScale': 1, 'normalize': False, 'force2Ddimension': 0, 'removeOutliers': None, 'minimumROISize': None, u'binWidth': 25.0, 'minimumROIDimensions': 1, u'correctMask': True, 'preCrop': False, u'symmetricalGLCM': True, 'resegmentRange': None, 'padDistance': 5}
general	info	ImageHash	e3bdc7d86304f67a6c6990c4ac4705cf9bef30b3
general	info	ImageSpacing	(0.9765625000000002, 0.9765625000000002, 2.0)
general	info	MaskHash	ac293e89147ec8672d7beb3a90db0b75b9785bf1
general	info	NumpyVersion	1.13.1
general	info	PyWaveletVersion	0.5.2
general	info	SimpleITKVersion	1.1.0
general	info	Version	1.3.0.post102+g1b0eb74
general	info	VolumeNum	1
general	info	VoxelNum	48
original	shape	Maximum3DDiameter	17.49285568
original	shape	Maximum2DDiameterSlice	17.49285568
original	shape	Sphericity	0.805725279
original	shape	MinorAxis	13.21850847
original	shape	Elongation	0.751768025
original	shape	SurfaceVolumeRatio	0.550506098
original	shape	Volume	1296
original	shape	MajorAxis	17.58322784
original	shape	SurfaceArea	713.4559033
original	shape	Flatness	0.55151147
original	shape	LeastAxis	9.697351826
original	shape	Maximum2DDiameterColumn	15
original	shape	Maximum2DDiameterRow	15.29705854
original	gldm	GrayLevelVariance	110.468316
original	gldm	HighGrayLevelEmphasis	833.8541667
original	gldm	DependenceEntropy	4.829448699
original	gldm	DependenceNonUniformity	26.875
original	gldm	GrayLevelNonUniformity	2.208333333
original	gldm	SmallDependenceEmphasis	0.774594907
original	gldm	SmallDependenceHighGrayLevelEmphasis	512.6539352
original	gldm	DependenceNonUniformityNormalized	0.559895833
original	gldm	LargeDependenceEmphasis	2.833333333
original	gldm	LargeDependenceLowGrayLevelEmphasis	0.02564929
original	gldm	DependenceVariance	0.706597222
original	gldm	LargeDependenceHighGrayLevelEmphasis	3485.4375
original	gldm	SmallDependenceLowGrayLevelEmphasis	0.024212397
original	gldm	LowGrayLevelEmphasis	0.024372677
original	glcm	JointAverage	29.25731939
original	glcm	SumAverage	58.51463878

original	glcm	JointEntropy	5.328424272
original	glcm	ClusterShade	-2434.897836
original	glcm	MaximumProbability	0.042034929
original	glcm	Idmn	0.915027384
original	glcm	JointEnergy	0.026179269
original	glcm	Contrast	172.5257406
original	glcm	DifferenceEntropy	3.575673813
original	glcm	InverseVariance	0.134115829
original	glcm	DifferenceVariance	64.27272399
original	glcm	Idn	0.814231771
original	glcm	Idm	0.117393065
original	glcm	Correlation	0.08197597
original	glcm	Autocorrelation	867.8582934
original	glcm	SumEntropy	4.02116507
original	glcm	SumSquares	96.91898424
original	glcm	ClusterProminence	146471.792
original	glcm	Imc2	0.999002228
original	glcm	Imc1	-0.745799059
original	glcm	DifferenceAverage	10.32847849
original	glcm	Id	0.191365034
original	glcm	ClusterTendency	215.1501964
original	firstorder	InterquartileRange	449
original	firstorder	Skewness	-0.589176063
original	firstorder	Uniformity	0.046006944
original	firstorder	Median	-237.5
original	firstorder	Energy	7331516
original	firstorder	RobustMeanAbsoluteDeviation	172.8808864
original	firstorder	MeanAbsoluteDeviation	224.96875
original	firstorder	TotalEnergy	197950932
original	firstorder	Maximum	42
original	firstorder	RootMeanSquared	390.8195449
original	firstorder	90Percentile	7.2
original	firstorder	Minimum	-930
original	firstorder	Entropy	4.621115365
original	firstorder	Range	972
original	firstorder	Variance	69747.90972
original	firstorder	10Percentile	-635.6
original	firstorder	Kurtosis	2.374581729
original	firstorder	Mean	-288.0833333
original	glrlm	ShortRunLowGrayLevelEmphasis	0.024794316
original	glrlm	GrayLevelVariance	110.1483783
original	glrlm	LowGrayLevelRunEmphasis	0.024803895
original	glrlm	GrayLevelNonUniformityNormalized	0.045323009
original	glrlm	RunVariance	0.017606401
original	glrlm	GrayLevelNonUniformity	2.137624825
original	glrlm	LongRunEmphasis	1.054650253
original	glrlm	ShortRunHighGrayLevelEmphasis	803.1556889
original	glrlm	RunLengthNonUniformity	45.51803885
original	glrlm	ShortRunEmphasis	0.986337437
original	glrlm	LongRunHighGrayLevelEmphasis	901.4883847
original	glrlm	RunPercentage	0.982371795
original	glrlm	LongRunLowGrayLevelEmphasis	0.024842213
original	glrlm	RunEntropy	4.671688442
original	glrlm	HighGrayLevelRunEmphasis	822.8222281
original	glrlm	RunLengthNonUniformityNormalized	0.964787198

original	glszm	GrayLevelVariance	103.499375
original	glszm	ZoneVariance	0.36
original	glszm	GrayLevelNonUniformityNormalized	0.0425
original	glszm	SizeZoneNonUniformityNormalized	0.7725
original	glszm	SizeZoneNonUniformity	30.9
original	glszm	GrayLevelNonUniformity	1.7
original	glszm	LargeAreaEmphasis	1.8
original	glszm	SmallAreaHighGrayLevelEmphasis	571.09375
original	glszm	ZonePercentage	0.833333333
original	glszm	LargeAreaLowGrayLevelEmphasis	0.029667275
original	glszm	LargeAreaHighGrayLevelEmphasis	1864.875
original	glszm	HighGrayLevelZoneEmphasis	714.825
original	glszm	SmallAreaEmphasis	0.898090278
original	glszm	LowGrayLevelZoneEmphasis	0.029105557
original	glszm	ZoneEntropy	4.78418372
original	glszm	SmallAreaLowGrayLevelEmphasis	0.029032083
original	ngtdm	Coarseness	0.061181759
original	ngtdm	Complexity	3230.078487
original	ngtdm	Strength	35.99074075
original	ngtdm	Contrast	2.300013529
original	ngtdm	Busyness	0.022978958
wavelet-HHH	gldm	GrayLevelVariance	14.13888889
wavelet-HHH	gldm	HighGrayLevelEmphasis	107.5833333
wavelet-HHH	gldm	DependenceEntropy	4.337581189
wavelet-HHH	gldm	DependenceNonUniformity	17.20833333
wavelet-HHH	gldm	GrayLevelNonUniformity	4.791666667
wavelet-HHH	gldm	SmallDependenceEmphasis	0.579826389
wavelet-HHH	gldm	SmallDependenceHighGrayLevelEmphasis	61.00274306
wavelet-HHH	gldm	DependenceNonUniformityNormalized	0.358506944
wavelet-HHH	gldm	LargeDependenceEmphasis	4.125
wavelet-HHH	gldm	LargeDependenceLowGrayLevelEmphasis	0.067279827
wavelet-HHH	gldm	DependenceVariance	0.914930556
wavelet-HHH	gldm	LargeDependenceHighGrayLevelEmphasis	462.8333333
wavelet-HHH	gldm	SmallDependenceLowGrayLevelEmphasis	0.033115056
wavelet-HHH	gldm	LowGrayLevelEmphasis	0.037464309
wavelet-HHH	glcm	JointAverage	9.511926706
wavelet-HHH	glcm	SumAverage	19.02385341
wavelet-HHH	glcm	JointEntropy	5.048558472
wavelet-HHH	glcm	ClusterShade	16.86978001
wavelet-HHH	glcm	MaximumProbability	0.058691959
wavelet-HHH	glcm	Idmn	0.935487048
wavelet-HHH	glcm	JointEnergy	0.033443441
wavelet-HHH	glcm	Contrast	28.2661769
wavelet-HHH	glcm	DifferenceEntropy	3.004580596
wavelet-HHH	glcm	InverseVariance	0.221640699
wavelet-HHH	glcm	DifferenceVariance	9.90733379
wavelet-HHH	glcm	Idn	0.832716653
wavelet-HHH	glcm	Idm	0.20076313
wavelet-HHH	glcm	Correlation	-0.011814275
wavelet-HHH	glcm	Autocorrelation	90.23731479
wavelet-HHH	glcm	SumEntropy	3.575832272
wavelet-HHH	glcm	SumSquares	13.83504949
wavelet-HHH	glcm	ClusterProminence	2309.034078
wavelet-HHH	glcm	Imc2	0.986033097
wavelet-HHH	glcm	Imc1	-0.533973747

wavelet-HHH	glcm	DifferenceAverage	4.221700547
wavelet-HHH	glcm	Id	0.296338629
wavelet-HHH	glcm	ClusterTendency	27.07402104
wavelet-HHH	firstorder	InterquartileRange	99.2245139
wavelet-HHH	firstorder	Skewness	0.308483749
wavelet-HHH	firstorder	Uniformity	0.099826389
wavelet-HHH	firstorder	Median	7.835259786
wavelet-HHH	firstorder	Energy	414069.9798
wavelet-HHH	firstorder	RobustMeanAbsoluteDeviation	46.02912334
wavelet-HHH	firstorder	MeanAbsoluteDeviation	70.52026133
wavelet-HHH	firstorder	TotalEnergy	11179889.46
wavelet-HHH	firstorder	Maximum	246.3736617
wavelet-HHH	firstorder	RootMeanSquared	92.87872692
wavelet-HHH	firstorder	90Percentile	142.9281309
wavelet-HHH	firstorder	Minimum	-218.8171937
wavelet-HHH	firstorder	Entropy	3.563388283
wavelet-HHH	firstorder	Range	465.1908554
wavelet-HHH	firstorder	Variance	8609.328247
wavelet-HHH	firstorder	10Percentile	-92.68077123
wavelet-HHH	firstorder	Kurtosis	3.168325879
wavelet-HHH	firstorder	Mean	4.138800166
wavelet-HHH	glrlm	ShortRunLowGrayLevelEmphasis	0.038129575
wavelet-HHH	glrlm	GrayLevelVariance	14.48846416
wavelet-HHH	glrlm	LowGrayLevelRunEmphasis	0.03836452
wavelet-HHH	glrlm	GrayLevelNonUniformityNormalized	0.098109546
wavelet-HHH	glrlm	RunVariance	0.033628843
wavelet-HHH	glrlm	GrayLevelNonUniformity	4.568746322
wavelet-HHH	glrlm	LongRunEmphasis	1.101510721
wavelet-HHH	glrlm	ShortRunHighGrayLevelEmphasis	104.8373021
wavelet-HHH	glrlm	RunLengthNonUniformity	43.96458238
wavelet-HHH	glrlm	ShortRunEmphasis	0.976521655
wavelet-HHH	glrlm	LongRunHighGrayLevelEmphasis	118.8176848
wavelet-HHH	glrlm	RunPercentage	0.969551282
wavelet-HHH	glrlm	LongRunLowGrayLevelEmphasis	0.039367088
wavelet-HHH	glrlm	RunEntropy	3.673907888
wavelet-HHH	glrlm	HighGrayLevelRunEmphasis	107.449523
wavelet-HHH	glrlm	RunLengthNonUniformityNormalized	0.942542884
wavelet-HHH	glszm	GrayLevelVariance	19.21484375
wavelet-HHH	glszm	ZoneVariance	1.125
wavelet-HHH	glszm	GrayLevelNonUniformityNormalized	0.087890625
wavelet-HHH	glszm	SizeZoneNonUniformityNormalized	0.5546875
wavelet-HHH	glszm	SizeZoneNonUniformity	17.75
wavelet-HHH	glszm	GrayLevelNonUniformity	2.8125
wavelet-HHH	glszm	LargeAreaEmphasis	3.375
wavelet-HHH	glszm	SmallAreaHighGrayLevelEmphasis	80.87413194
wavelet-HHH	glszm	ZonePercentage	0.666666667
wavelet-HHH	glszm	LargeAreaLowGrayLevelEmphasis	0.07401228
wavelet-HHH	glszm	LargeAreaHighGrayLevelEmphasis	372.875
wavelet-HHH	glszm	HighGrayLevelZoneEmphasis	105.9375
wavelet-HHH	glszm	SmallAreaEmphasis	0.771918403
wavelet-HHH	glszm	LowGrayLevelZoneEmphasis	0.0511212
wavelet-HHH	glszm	ZoneEntropy	4.176108501
wavelet-HHH	glszm	SmallAreaLowGrayLevelEmphasis	0.048633282
wavelet-HHH	ngtdm	Coarseness	0.097028383
wavelet-HHH	ngtdm	Complexity	295.5132997

wavelet-HHH	ngtdm	Strength	8.496372396
wavelet-HHH	ngtdm	Contrast	0.417182855
wavelet-HHH	ngtdm	Busyness	0.081904074
wavelet-HLL	gldm	GrayLevelVariance	172.280816
wavelet-HLL	gldm	HighGrayLevelEmphasis	880.0625
wavelet-HLL	gldm	DependenceEntropy	4.902569011
wavelet-HLL	gldm	DependenceNonUniformity	28.16666667
wavelet-HLL	gldm	GrayLevelNonUniformity	2.125
wavelet-HLL	gldm	SmallDependenceEmphasis	0.78125
wavelet-HLL	gldm	SmallDependenceHighGrayLevelEmphasis	615.90625
wavelet-HLL	gldm	DependenceNonUniformityNormalized	0.586805556
wavelet-HLL	gldm	LargeDependenceEmphasis	1.875
wavelet-HLL	gldm	LargeDependenceLowGrayLevelEmphasis	0.03078856
wavelet-HLL	gldm	DependenceVariance	0.206597222
wavelet-HLL	gldm	LargeDependenceHighGrayLevelEmphasis	1936.6875
wavelet-HLL	gldm	SmallDependenceLowGrayLevelEmphasis	0.02573652
wavelet-HLL	gldm	LowGrayLevelEmphasis	0.026746928
wavelet-HLL	glcm	JointAverage	25.56032472
wavelet-HLL	glcm	SumAverage	51.12064944
wavelet-HLL	glcm	JointEntropy	5.389626966
wavelet-HLL	glcm	ClusterShade	-1611.375184
wavelet-HLL	glcm	MaximumProbability	0.033540855
wavelet-HLL	glcm	Idmn	0.894590305
wavelet-HLL	glcm	JointEnergy	0.024798135
wavelet-HLL	glcm	Contrast	306.0915426
wavelet-HLL	glcm	DifferenceEntropy	3.581433285
wavelet-HLL	glcm	InverseVariance	0.087954243
wavelet-HLL	glcm	DifferenceVariance	72.18706026
wavelet-HLL	glcm	Idn	0.793802626
wavelet-HLL	glcm	Idm	0.076023233
wavelet-HLL	glcm	Correlation	0.212407313
wavelet-HLL	glcm	Autocorrelation	686.6146363
wavelet-HLL	glcm	SumEntropy	4.178379642
wavelet-HLL	glcm	SumSquares	185.5911701
wavelet-HLL	glcm	ClusterProminence	407416.7032
wavelet-HLL	glcm	Imc2	0.999567818
wavelet-HLL	glcm	Imc1	-0.798757849
wavelet-HLL	glcm	DifferenceAverage	13.92953868
wavelet-HLL	glcm	Id	0.14756541
wavelet-HLL	glcm	ClusterTendency	436.2731379
wavelet-HLL	firstorder	InterquartileRange	592.7828171
wavelet-HLL	firstorder	Skewness	-0.285798163
wavelet-HLL	firstorder	Uniformity	0.044270833
wavelet-HLL	firstorder	Median	32.45267764
wavelet-HLL	firstorder	Energy	5425175.688
wavelet-HLL	firstorder	RobustMeanAbsoluteDeviation	241.6856937
wavelet-HLL	firstorder	MeanAbsoluteDeviation	294.551457
wavelet-HLL	firstorder	TotalEnergy	146479743.6
wavelet-HLL	firstorder	Maximum	428.288359
wavelet-HLL	firstorder	RootMeanSquared	336.1911562
wavelet-HLL	firstorder	90Percentile	294.7685453
wavelet-HLL	firstorder	Minimum	-706.3488917
wavelet-HLL	firstorder	Entropy	4.712581189
wavelet-HLL	firstorder	Range	1134.637251
wavelet-HLL	firstorder	Variance	107598.4911

wavelet-HLL	firstorder	10Percentile	-524.8655403
wavelet-HLL	firstorder	Kurtosis	1.70446527
wavelet-HLL	firstorder	Mean	-73.66140407
wavelet-HLL	glrlm	ShortRunLowGrayLevelEmphasis	0.026966232
wavelet-HLL	glrlm	GrayLevelVariance	172.22917
wavelet-HLL	glrlm	LowGrayLevelRunEmphasis	0.027006073
wavelet-HLL	glrlm	GrayLevelNonUniformityNormalized	0.043996277
wavelet-HLL	glrlm	RunVariance	0.011193679
wavelet-HLL	glrlm	GrayLevelNonUniformity	2.088306965
wavelet-HLL	glrlm	LongRunEmphasis	1.03502455
wavelet-HLL	glrlm	ShortRunHighGrayLevelEmphasis	865.7087198
wavelet-HLL	glrlm	RunLengthNonUniformity	46.42847791
wavelet-HLL	glrlm	ShortRunEmphasis	0.991243863
wavelet-HLL	glrlm	LongRunHighGrayLevelEmphasis	918.4838152
wavelet-HLL	glrlm	RunPercentage	0.988782051
wavelet-HLL	glrlm	LongRunLowGrayLevelEmphasis	0.027165436
wavelet-HLL	glrlm	RunEntropy	4.734547558
wavelet-HLL	glrlm	HighGrayLevelRunEmphasis	876.2637389
wavelet-HLL	glrlm	RunLengthNonUniformityNormalized	0.977612641
wavelet-HLL	glszm	GrayLevelVariance	170.7828673
wavelet-HLL	glszm	ZoneVariance	0.141582391
wavelet-HLL	glszm	GrayLevelNonUniformityNormalized	0.039857228
wavelet-HLL	glszm	SizeZoneNonUniformityNormalized	0.716835217
wavelet-HLL	glszm	SizeZoneNonUniformity	29.3902439
wavelet-HLL	glszm	GrayLevelNonUniformity	1.634146341
wavelet-HLL	glszm	LargeAreaEmphasis	1.512195122
wavelet-HLL	glszm	SmallAreaHighGrayLevelEmphasis	669.5182927
wavelet-HLL	glszm	ZonePercentage	0.854166667
wavelet-HLL	glszm	LargeAreaLowGrayLevelEmphasis	0.032890699
wavelet-HLL	glszm	LargeAreaHighGrayLevelEmphasis	1442.658537
wavelet-HLL	glszm	HighGrayLevelZoneEmphasis	824.1463415
wavelet-HLL	glszm	SmallAreaEmphasis	0.87195122
wavelet-HLL	glszm	LowGrayLevelZoneEmphasis	0.030524865
wavelet-HLL	glszm	ZoneEntropy	4.948896212
wavelet-HLL	glszm	SmallAreaLowGrayLevelEmphasis	0.029933407
wavelet-HLL	ngtdm	Coarseness	0.049169952
wavelet-HLL	ngtdm	Complexity	5290.443115
wavelet-HLL	ngtdm	Strength	47.09046734
wavelet-HLL	ngtdm	Contrast	3.803439968
wavelet-HLL	ngtdm	Busyness	0.027190852
wavelet-LHL	gldm	GrayLevelVariance	195.3398438
wavelet-LHL	gldm	HighGrayLevelEmphasis	836.0625
wavelet-LHL	gldm	DependenceEntropy	5.110902344
wavelet-LHL	gldm	DependenceNonUniformity	32.625
wavelet-LHL	gldm	GrayLevelNonUniformity	1.666666667
wavelet-LHL	gldm	SmallDependenceEmphasis	0.850694444
wavelet-LHL	gldm	SmallDependenceHighGrayLevelEmphasis	705.625
wavelet-LHL	gldm	DependenceNonUniformityNormalized	0.6796875
wavelet-LHL	gldm	LargeDependenceEmphasis	1.875
wavelet-LHL	gldm	LargeDependenceLowGrayLevelEmphasis	0.044301537
wavelet-LHL	gldm	DependenceVariance	0.3125
wavelet-LHL	gldm	LargeDependenceHighGrayLevelEmphasis	1367.8125
wavelet-LHL	gldm	SmallDependenceLowGrayLevelEmphasis	0.028431462
wavelet-LHL	gldm	LowGrayLevelEmphasis	0.030062267
wavelet-LHL	glcm	JointAverage	24.28320034

wavelet-LHL	glcm	SumAverage	48.56640068
wavelet-LHL	glcm	JointEntropy	5.412816206
wavelet-LHL	glcm	ClusterShade	2626.370744
wavelet-LHL	glcm	MaximumProbability	0.031997302
wavelet-LHL	glcm	Idmn	0.898541583
wavelet-LHL	glcm	JointEnergy	0.024410702
wavelet-LHL	glcm	Contrast	375.6285112
wavelet-LHL	glcm	DifferenceEntropy	3.833416518
wavelet-LHL	glcm	InverseVariance	0.081627035
wavelet-LHL	glcm	DifferenceVariance	101.9085283
wavelet-LHL	glcm	Idn	0.795838343
wavelet-LHL	glcm	Idm	0.069579644
wavelet-LHL	glcm	Correlation	0.133198191
wavelet-LHL	glcm	Autocorrelation	617.0262775
wavelet-LHL	glcm	SumEntropy	4.273582191
wavelet-LHL	glcm	SumSquares	213.8885378
wavelet-LHL	glcm	ClusterProminence	547639.6188
wavelet-LHL	glcm	Imc2	0.999741709
wavelet-LHL	glcm	Imc1	-0.829624037
wavelet-LHL	glcm	DifferenceAverage	15.43769242
wavelet-LHL	glcm	Id	0.135836501
wavelet-LHL	glcm	ClusterTendency	479.9256399
wavelet-LHL	firstorder	InterquartileRange	539.3265339
wavelet-LHL	firstorder	Skewness	-0.035785255
wavelet-LHL	firstorder	Uniformity	0.034722222
wavelet-LHL	firstorder	Median	0.008843474
wavelet-LHL	firstorder	Energy	5825114.526
wavelet-LHL	firstorder	RobustMeanAbsoluteDeviation	230.0143887
wavelet-LHL	firstorder	MeanAbsoluteDeviation	295.3968102
wavelet-LHL	firstorder	TotalEnergy	157278092.2
wavelet-LHL	firstorder	Maximum	681.3657444
wavelet-LHL	firstorder	RootMeanSquared	348.362674
wavelet-LHL	firstorder	90Percentile	460.6317321
wavelet-LHL	firstorder	Minimum	-606.8942751
wavelet-LHL	firstorder	Entropy	4.996115365
wavelet-LHL	firstorder	Range	1288.26002
wavelet-LHL	firstorder	Variance	121333.2471
wavelet-LHL	firstorder	10Percentile	-499.6760044
wavelet-LHL	firstorder	Kurtosis	1.915682874
wavelet-LHL	firstorder	Mean	-4.827577868
wavelet-LHL	glrlm	ShortRunLowGrayLevelEmphasis	0.03011353
wavelet-LHL	glrlm	GrayLevelVariance	194.4561986
wavelet-LHL	glrlm	LowGrayLevelRunEmphasis	0.030220891
wavelet-LHL	glrlm	GrayLevelNonUniformityNormalized	0.034412323
wavelet-LHL	glrlm	RunVariance	0.009591841
wavelet-LHL	glrlm	GrayLevelNonUniformity	1.63647936
wavelet-LHL	glrlm	LongRunEmphasis	1.030114566
wavelet-LHL	glrlm	ShortRunHighGrayLevelEmphasis	832.0205378
wavelet-LHL	glrlm	RunLengthNonUniformity	46.65597381
wavelet-LHL	glrlm	ShortRunEmphasis	0.992471358
wavelet-LHL	glrlm	LongRunHighGrayLevelEmphasis	858.6659279
wavelet-LHL	glrlm	RunPercentage	0.990384615
wavelet-LHL	glrlm	LongRunLowGrayLevelEmphasis	0.030650333
wavelet-LHL	glrlm	RunEntropy	5.019254482
wavelet-LHL	glrlm	HighGrayLevelRunEmphasis	837.3496158

wavelet-LHL	glrlm	RunLengthNonUniformityNormalized	0.980816319
wavelet-LHL	glszm	GrayLevelVariance	187.5511087
wavelet-LHL	glszm	ZoneVariance	0.149269876
wavelet-LHL	glszm	GrayLevelNonUniformityNormalized	0.03190914
wavelet-LHL	glszm	SizeZoneNonUniformityNormalized	0.828015143
wavelet-LHL	glszm	SizeZoneNonUniformity	35.60465116
wavelet-LHL	glszm	GrayLevelNonUniformity	1.372093023
wavelet-LHL	glszm	LargeAreaEmphasis	1.395348837
wavelet-LHL	glszm	SmallAreaHighGrayLevelEmphasis	763.5930233
wavelet-LHL	glszm	ZonePercentage	0.895833333
wavelet-LHL	glszm	LargeAreaLowGrayLevelEmphasis	0.037564223
wavelet-LHL	glszm	LargeAreaHighGrayLevelEmphasis	1129.465116
wavelet-LHL	glszm	HighGrayLevelZoneEmphasis	836.0232558
wavelet-LHL	glszm	SmallAreaEmphasis	0.927002584
wavelet-LHL	glszm	LowGrayLevelZoneEmphasis	0.032200703
wavelet-LHL	glszm	ZoneEntropy	5.147194987
wavelet-LHL	glszm	SmallAreaLowGrayLevelEmphasis	0.031577595
wavelet-LHL	ngtdm	Coarseness	0.053666584
wavelet-LHL	ngtdm	Complexity	7459.939381
wavelet-LHL	ngtdm	Strength	52.70597463
wavelet-LHL	ngtdm	Contrast	3.518360587
wavelet-LHL	ngtdm	Busyness	0.027192366
wavelet-LHH	gldm	GrayLevelVariance	36.41493056
wavelet-LHH	gldm	HighGrayLevelEmphasis	424.8333333
wavelet-LHH	gldm	DependenceEntropy	4.662782032
wavelet-LHH	gldm	DependenceNonUniformity	22.625
wavelet-LHH	gldm	GrayLevelNonUniformity	3.125
wavelet-LHH	gldm	SmallDependenceEmphasis	0.703269676
wavelet-LHH	gldm	SmallDependenceHighGrayLevelEmphasis	306.7877604
wavelet-LHH	gldm	DependenceNonUniformityNormalized	0.471354167
wavelet-LHH	gldm	LargeDependenceEmphasis	2.791666667
wavelet-LHH	gldm	LargeDependenceLowGrayLevelEmphasis	0.029057208
wavelet-LHH	gldm	DependenceVariance	0.541666667
wavelet-LHH	gldm	LargeDependenceHighGrayLevelEmphasis	1163.708333
wavelet-LHH	gldm	SmallDependenceLowGrayLevelEmphasis	0.023616621
wavelet-LHH	gldm	LowGrayLevelEmphasis	0.024430477
wavelet-LHH	glcm	JointAverage	19.40746652
wavelet-LHH	glcm	SumAverage	38.81493304
wavelet-LHH	glcm	JointEntropy	5.254504792
wavelet-LHH	glcm	ClusterShade	-177.015426
wavelet-LHH	glcm	MaximumProbability	0.051558738
wavelet-LHH	glcm	Idmn	0.944952432
wavelet-LHH	glcm	JointEnergy	0.028301264
wavelet-LHH	glcm	Contrast	68.98018119
wavelet-LHH	glcm	DifferenceEntropy	3.344895133
wavelet-LHH	glcm	InverseVariance	0.171477506
wavelet-LHH	glcm	DifferenceVariance	27.26806036
wavelet-LHH	glcm	Idn	0.848839502
wavelet-LHH	glcm	Idm	0.145109086
wavelet-LHH	glcm	Correlation	-0.004589844
wavelet-LHH	glcm	Autocorrelation	377.0737718
wavelet-LHH	glcm	SumEntropy	3.895373489
wavelet-LHH	glcm	SumSquares	34.50169366
wavelet-LHH	glcm	ClusterProminence	17407.72749
wavelet-LHH	glcm	Imc2	0.997372778

wavelet-LHH	glcm	Imc1	-0.674890096
wavelet-LHH	glcm	DifferenceAverage	6.344328028
wavelet-LHH	glcm	Id	0.237065998
wavelet-LHH	glcm	ClusterTendency	69.02659344
wavelet-LHH	firstorder	InterquartileRange	138.3588168
wavelet-LHH	firstorder	Skewness	-0.656878632
wavelet-LHH	firstorder	Uniformity	0.065104167
wavelet-LHH	firstorder	Median	20.70008959
wavelet-LHH	firstorder	Energy	1110978.15
wavelet-LHH	firstorder	RobustMeanAbsoluteDeviation	66.81193629
wavelet-LHH	firstorder	MeanAbsoluteDeviation	111.3070756
wavelet-LHH	firstorder	TotalEnergy	29996410.04
wavelet-LHH	firstorder	Maximum	321.9912054
wavelet-LHH	firstorder	RootMeanSquared	152.1360513
wavelet-LHH	firstorder	90Percentile	168.4485497
wavelet-LHH	firstorder	Minimum	-472.6782132
wavelet-LHH	firstorder	Entropy	4.215199877
wavelet-LHH	firstorder	Range	794.6694185
wavelet-LHH	firstorder	Variance	23118.98104
wavelet-LHH	firstorder	10Percentile	-161.2298297
wavelet-LHH	firstorder	Kurtosis	4.205452935
wavelet-LHH	firstorder	Mean	5.137808738
wavelet-LHH	glrlm	ShortRunLowGrayLevelEmphasis	0.02482542
wavelet-LHH	glrlm	GrayLevelVariance	36.99906076
wavelet-LHH	glrlm	LowGrayLevelRunEmphasis	0.024864823
wavelet-LHH	glrlm	GrayLevelNonUniformityNormalized	0.064008928
wavelet-LHH	glrlm	RunVariance	0.019198265
wavelet-LHH	glrlm	GrayLevelNonUniformity	3.014387596
wavelet-LHH	glrlm	LongRunEmphasis	1.059787946
wavelet-LHH	glrlm	ShortRunHighGrayLevelEmphasis	419.0768958
wavelet-LHH	glrlm	RunLengthNonUniformity	45.29782964
wavelet-LHH	glrlm	ShortRunEmphasis	0.985053014
wavelet-LHH	glrlm	LongRunHighGrayLevelEmphasis	449.5549135
wavelet-LHH	glrlm	RunPercentage	0.980769231
wavelet-LHH	glrlm	LongRunLowGrayLevelEmphasis	0.025022435
wavelet-LHH	glrlm	RunEntropy	4.281928999
wavelet-LHH	glrlm	HighGrayLevelRunEmphasis	425.1724993
wavelet-LHH	glrlm	RunLengthNonUniformityNormalized	0.961603471
wavelet-LHH	glszm	GrayLevelVariance	45.03871439
wavelet-LHH	glszm	ZoneVariance	0.749452155
wavelet-LHH	glszm	GrayLevelNonUniformityNormalized	0.056245435
wavelet-LHH	glszm	SizeZoneNonUniformityNormalized	0.684441198
wavelet-LHH	glszm	SizeZoneNonUniformity	25.32432432
wavelet-LHH	glszm	GrayLevelNonUniformity	2.081081081
wavelet-LHH	glszm	LargeAreaEmphasis	2.432432432
wavelet-LHH	glszm	SmallAreaHighGrayLevelEmphasis	374.1959459
wavelet-LHH	glszm	ZonePercentage	0.770833333
wavelet-LHH	glszm	LargeAreaLowGrayLevelEmphasis	0.034511484
wavelet-LHH	glszm	LargeAreaHighGrayLevelEmphasis	1029.567568
wavelet-LHH	glszm	HighGrayLevelZoneEmphasis	431.1081081
wavelet-LHH	glszm	SmallAreaEmphasis	0.852102102
wavelet-LHH	glszm	LowGrayLevelZoneEmphasis	0.030897504
wavelet-LHH	glszm	ZoneEntropy	4.60770573
wavelet-LHH	glszm	SmallAreaLowGrayLevelEmphasis	0.030470676
wavelet-LHH	ngtdm	Coarseness	0.094863789

wavelet-LHH	ngtdm	Complexity	1087.238492
wavelet-LHH	ngtdm	Strength	19.94632562
wavelet-LHH	ngtdm	Contrast	0.702957915
wavelet-LHH	ngtdm	Busyness	0.026920017
wavelet-LLH	gldm	GrayLevelVariance	140.2252604
wavelet-LLH	gldm	HighGrayLevelEmphasis	498.8541667
wavelet-LLH	gldm	DependenceEntropy	4.803508855
wavelet-LLH	gldm	DependenceNonUniformity	25.125
wavelet-LLH	gldm	GrayLevelNonUniformity	2.375
wavelet-LLH	gldm	SmallDependenceEmphasis	0.739583333
wavelet-LLH	gldm	SmallDependenceHighGrayLevelEmphasis	455.1990741
wavelet-LLH	gldm	DependenceNonUniformityNormalized	0.5234375
wavelet-LLH	gldm	LargeDependenceEmphasis	2.875
wavelet-LLH	gldm	LargeDependenceLowGrayLevelEmphasis	0.060812723
wavelet-LLH	gldm	DependenceVariance	0.625
wavelet-LLH	gldm	LargeDependenceHighGrayLevelEmphasis	821.4375
wavelet-LLH	gldm	SmallDependenceLowGrayLevelEmphasis	0.03135429
wavelet-LLH	gldm	LowGrayLevelEmphasis	0.035078624
wavelet-LLH	glcm	JointAverage	17.35344537
wavelet-LLH	glcm	SumAverage	34.70689074
wavelet-LLH	glcm	JointEntropy	5.315201637
wavelet-LLH	glcm	ClusterShade	5913.823702
wavelet-LLH	glcm	MaximumProbability	0.048064814
wavelet-LLH	glcm	Idmn	0.935181328
wavelet-LLH	glcm	JointEnergy	0.026962142
wavelet-LLH	glcm	Contrast	209.2276767
wavelet-LLH	glcm	DifferenceEntropy	3.686793662
wavelet-LLH	glcm	InverseVariance	0.103081342
wavelet-LLH	glcm	DifferenceVariance	88.50413872
wavelet-LLH	glcm	Idn	0.841635363
wavelet-LLH	glcm	Idm	0.105892365
wavelet-LLH	glcm	Correlation	0.204244507
wavelet-LLH	glcm	Autocorrelation	329.261294
wavelet-LLH	glcm	SumEntropy	4.121926871
wavelet-LLH	glcm	SumSquares	132.3439686
wavelet-LLH	glcm	ClusterProminence	379700.3778
wavelet-LLH	glcm	Imc2	0.999195551
wavelet-LLH	glcm	Imc1	-0.765383825
wavelet-LLH	glcm	DifferenceAverage	10.71588355
wavelet-LLH	glcm	Id	0.185982317
wavelet-LLH	glcm	ClusterTendency	320.1481977
wavelet-LLH	firstorder	InterquartileRange	302.2251395
wavelet-LLH	firstorder	Skewness	0.859535235
wavelet-LLH	firstorder	Uniformity	0.049479167
wavelet-LLH	firstorder	Median	-64.08211405
wavelet-LLH	firstorder	Energy	4248633.215
wavelet-LLH	firstorder	RobustMeanAbsoluteDeviation	149.0100925
wavelet-LLH	firstorder	MeanAbsoluteDeviation	230.4115341
wavelet-LLH	firstorder	TotalEnergy	114713096.8
wavelet-LLH	firstorder	Maximum	785.4636787
wavelet-LLH	firstorder	RootMeanSquared	297.511667
wavelet-LLH	firstorder	90Percentile	528.2608978
wavelet-LLH	firstorder	Minimum	-448.8575542
wavelet-LLH	firstorder	Entropy	4.595175521
wavelet-LLH	firstorder	Range	1234.321233

wavelet-LLH	firstorder	Variance	88390.97504
wavelet-LLH	firstorder	10Percentile	-337.846511
wavelet-LLH	firstorder	Kurtosis	3.096191391
wavelet-LLH	firstorder	Mean	11.05517687
wavelet-LLH	glrlm	ShortRunLowGrayLevelEmphasis	0.035302046
wavelet-LLH	glrlm	GrayLevelVariance	141.645142
wavelet-LLH	glrlm	LowGrayLevelRunEmphasis	0.035511475
wavelet-LLH	glrlm	GrayLevelNonUniformityNormalized	0.048801426
wavelet-LLH	glrlm	RunVariance	0.019158164
wavelet-LLH	glrlm	GrayLevelNonUniformity	2.298292018
wavelet-LLH	glrlm	LongRunEmphasis	1.060899323
wavelet-LLH	glrlm	ShortRunHighGrayLevelEmphasis	502.9053249
wavelet-LLH	glrlm	RunLengthNonUniformity	45.33339371
wavelet-LLH	glrlm	ShortRunEmphasis	0.984775169
wavelet-LLH	glrlm	LongRunHighGrayLevelEmphasis	515.9241865
wavelet-LLH	glrlm	RunPercentage	0.980769231
wavelet-LLH	glrlm	LongRunLowGrayLevelEmphasis	0.036349188
wavelet-LLH	glrlm	RunEntropy	4.638785177
wavelet-LLH	glrlm	HighGrayLevelRunEmphasis	505.5090972
wavelet-LLH	glrlm	RunLengthNonUniformityNormalized	0.961683671
wavelet-LLH	glszm	GrayLevelVariance	153.0616343
wavelet-LLH	glszm	ZoneVariance	0.56232687
wavelet-LLH	glszm	GrayLevelNonUniformityNormalized	0.04432133
wavelet-LLH	glszm	SizeZoneNonUniformityNormalized	0.760387812
wavelet-LLH	glszm	SizeZoneNonUniformity	28.89473684
wavelet-LLH	glszm	GrayLevelNonUniformity	1.684210526
wavelet-LLH	glszm	LargeAreaEmphasis	2.157894737
wavelet-LLH	glszm	SmallAreaHighGrayLevelEmphasis	568.4161184
wavelet-LLH	glszm	ZonePercentage	0.791666667
wavelet-LLH	glszm	LargeAreaLowGrayLevelEmphasis	0.060138754
wavelet-LLH	glszm	LargeAreaHighGrayLevelEmphasis	757.0263158
wavelet-LLH	glszm	HighGrayLevelZoneEmphasis	588.5526316
wavelet-LLH	glszm	SmallAreaEmphasis	0.887792398
wavelet-LLH	glszm	LowGrayLevelZoneEmphasis	0.040182747
wavelet-LLH	glszm	ZoneEntropy	4.807009421
wavelet-LLH	glszm	SmallAreaLowGrayLevelEmphasis	0.038758498
wavelet-LLH	ngtdm	Coarseness	0.080313262
wavelet-LLH	ngtdm	Complexity	3665.049175
wavelet-LLH	ngtdm	Strength	57.4522212
wavelet-LLH	ngtdm	Contrast	2.4689757
wavelet-LLH	ngtdm	Busyness	0.035206156
wavelet-LLL	gldm	GrayLevelVariance	479.6349826
wavelet-LLL	gldm	HighGrayLevelEmphasis	3370.9375
wavelet-LLL	gldm	DependenceEntropy	5.293295834
wavelet-LLL	gldm	DependenceNonUniformity	48
wavelet-LLL	gldm	GrayLevelNonUniformity	1.291666667
wavelet-LLL	gldm	SmallDependenceEmphasis	1
wavelet-LLL	gldm	SmallDependenceHighGrayLevelEmphasis	3370.9375
wavelet-LLL	gldm	DependenceNonUniformityNormalized	1
wavelet-LLL	gldm	LargeDependenceEmphasis	1
wavelet-LLL	gldm	LargeDependenceLowGrayLevelEmphasis	0.021551644
wavelet-LLL	gldm	DependenceVariance	0
wavelet-LLL	gldm	LargeDependenceHighGrayLevelEmphasis	3370.9375
wavelet-LLL	gldm	SmallDependenceLowGrayLevelEmphasis	0.021551644
wavelet-LLL	gldm	LowGrayLevelEmphasis	0.021551644

wavelet-LLL	glcm	JointAverage	60.62060879
wavelet-LLL	glcm	SumAverage	121.2412176
wavelet-LLL	glcm	JointEntropy	5.432790957
wavelet-LLL	glcm	ClusterShade	-7280.520067
wavelet-LLL	glcm	MaximumProbability	0.025128206
wavelet-LLL	glcm	Idmn	0.933755985
wavelet-LLL	glcm	JointEnergy	0.023967556
wavelet-LLL	glcm	Contrast	777.304666
wavelet-LLL	glcm	DifferenceEntropy	4.002723352
wavelet-LLL	glcm	InverseVariance	0.038023186
wavelet-LLL	glcm	DifferenceVariance	227.0591548
wavelet-LLL	glcm	Idn	0.824570441
wavelet-LLL	glcm	Idm	0.025859368
wavelet-LLL	glcm	Correlation	0.162568817
wavelet-LLL	glcm	Autocorrelation	3761.557559
wavelet-LLL	glcm	SumEntropy	4.285212222
wavelet-LLL	glcm	SumSquares	471.6301982
wavelet-LLL	glcm	ClusterProminence	3259790.993
wavelet-LLL	glcm	Imc2	0.999927406
wavelet-LLL	glcm	Imc1	-0.902089237
wavelet-LLL	glcm	DifferenceAverage	23.13430475
wavelet-LLL	glcm	Id	0.081792228
wavelet-LLL	glcm	ClusterTendency	1109.216127
wavelet-LLL	firstorder	InterquartileRange	794.3621131
wavelet-LLL	firstorder	Skewness	0.047494837
wavelet-LLL	firstorder	Uniformity	0.026909722
wavelet-LLL	firstorder	Median	-1143.337376
wavelet-LLL	firstorder	Energy	66759956.02
wavelet-LLL	firstorder	RobustMeanAbsoluteDeviation	328.9906491
wavelet-LLL	firstorder	MeanAbsoluteDeviation	453.6305366
wavelet-LLL	firstorder	TotalEnergy	1802518813
wavelet-LLL	firstorder	Maximum	123.7826367
wavelet-LLL	firstorder	RootMeanSquared	1179.335583
wavelet-LLL	firstorder	90Percentile	-300.0637549
wavelet-LLL	firstorder	Minimum	-2352.126062
wavelet-LLL	firstorder	Entropy	5.293295834
wavelet-LLL	firstorder	Range	2475.908699
wavelet-LLL	firstorder	Variance	299316.8602
wavelet-LLL	firstorder	10Percentile	-1599.707036
wavelet-LLL	firstorder	Kurtosis	2.59248085
wavelet-LLL	firstorder	Mean	-1044.756219
wavelet-LLL	glrlm	ShortRunLowGrayLevelEmphasis	0.021551644
wavelet-LLL	glrlm	GrayLevelVariance	479.6349826
wavelet-LLL	glrlm	LowGrayLevelRunEmphasis	0.021551644
wavelet-LLL	glrlm	GrayLevelNonUniformityNormalized	0.026909722
wavelet-LLL	glrlm	RunVariance	0
wavelet-LLL	glrlm	GrayLevelNonUniformity	1.291666667
wavelet-LLL	glrlm	LongRunEmphasis	1
wavelet-LLL	glrlm	ShortRunHighGrayLevelEmphasis	3370.9375
wavelet-LLL	glrlm	RunLengthNonUniformity	48
wavelet-LLL	glrlm	ShortRunEmphasis	1
wavelet-LLL	glrlm	LongRunHighGrayLevelEmphasis	3370.9375
wavelet-LLL	glrlm	RunPercentage	1
wavelet-LLL	glrlm	LongRunLowGrayLevelEmphasis	0.021551644
wavelet-LLL	glrlm	RunEntropy	5.293295834

wavelet-LLL	glrlm	HighGrayLevelRunEmphasis	3370.9375
wavelet-LLL	glrlm	RunLengthNonUniformityNormalized	1
wavelet-LLL	glszm	GrayLevelVariance	479.6349826
wavelet-LLL	glszm	ZoneVariance	0
wavelet-LLL	glszm	GrayLevelNonUniformityNormalized	0.026909722
wavelet-LLL	glszm	SizeZoneNonUniformityNormalized	1
wavelet-LLL	glszm	SizeZoneNonUniformity	48
wavelet-LLL	glszm	GrayLevelNonUniformity	1.291666667
wavelet-LLL	glszm	LargeAreaEmphasis	1
wavelet-LLL	glszm	SmallAreaHighGrayLevelEmphasis	3370.9375
wavelet-LLL	glszm	ZonePercentage	1
wavelet-LLL	glszm	LargeAreaLowGrayLevelEmphasis	0.021551644
wavelet-LLL	glszm	LargeAreaHighGrayLevelEmphasis	3370.9375
wavelet-LLL	glszm	HighGrayLevelZoneEmphasis	3370.9375
wavelet-LLL	glszm	SmallAreaEmphasis	1
wavelet-LLL	glszm	LowGrayLevelZoneEmphasis	0.021551644
wavelet-LLL	glszm	ZoneEntropy	5.293295834
wavelet-LLL	glszm	SmallAreaLowGrayLevelEmphasis	0.021551644
wavelet-LLL	ngtdm	Coarseness	0.051243548
wavelet-LLL	ngtdm	Complexity	18263.30948
wavelet-LLL	ngtdm	Strength	110.1072962
wavelet-LLL	ngtdm	Contrast	9.133528156
wavelet-LLL	ngtdm	Busyness	0.016456488
wavelet-HHL	gldm	GrayLevelVariance	20.59331597
wavelet-HHL	gldm	HighGrayLevelEmphasis	105.7708333
wavelet-HHL	gldm	DependenceEntropy	4.735902344
wavelet-HHL	gldm	DependenceNonUniformity	18.91666667
wavelet-HHL	gldm	GrayLevelNonUniformity	3.458333333
wavelet-HHL	gldm	SmallDependenceEmphasis	0.559751157
wavelet-HHL	gldm	SmallDependenceHighGrayLevelEmphasis	61.72395833
wavelet-HHL	gldm	DependenceNonUniformityNormalized	0.394097222
wavelet-HHL	gldm	LargeDependenceEmphasis	3.458333333
wavelet-HHL	gldm	LargeDependenceLowGrayLevelEmphasis	0.11643115
wavelet-HHL	gldm	DependenceVariance	0.539930556
wavelet-HHL	gldm	LargeDependenceHighGrayLevelEmphasis	360.3958333
wavelet-HHL	gldm	SmallDependenceLowGrayLevelEmphasis	0.040113786
wavelet-HHL	gldm	LowGrayLevelEmphasis	0.054071337
wavelet-HHL	glcm	JointAverage	9.037791475
wavelet-HHL	glcm	SumAverage	18.07558295
wavelet-HHL	glcm	JointEntropy	5.177084492
wavelet-HHL	glcm	ClusterShade	42.01845077
wavelet-HHL	glcm	MaximumProbability	0.052519298
wavelet-HHL	glcm	Idmn	0.930518428
wavelet-HHL	glcm	JointEnergy	0.02998224
wavelet-HHL	glcm	Contrast	41.64642884
wavelet-HHL	glcm	DifferenceEntropy	3.196220151
wavelet-HHL	glcm	InverseVariance	0.191527174
wavelet-HHL	glcm	DifferenceVariance	14.57135318
wavelet-HHL	glcm	Idn	0.827806073
wavelet-HHL	glcm	Idm	0.175726982
wavelet-HHL	glcm	Correlation	-0.073680137
wavelet-HHL	glcm	Autocorrelation	80.7104739
wavelet-HHL	glcm	SumEntropy	3.67380891
wavelet-HHL	glcm	SumSquares	19.52585014
wavelet-HHL	glcm	ClusterProminence	4063.054135

wavelet-HHL	glcm	Imc2	0.995851824
wavelet-HHL	glcm	Imc1	-0.644817846
wavelet-HHL	glcm	DifferenceAverage	5.093497935
wavelet-HHL	glcm	Id	0.269411794
wavelet-HHL	glcm	ClusterTendency	36.45697171
wavelet-HHL	firstorder	InterquartileRange	161.9025054
wavelet-HHL	firstorder	Skewness	0.405461894
wavelet-HHL	firstorder	Uniformity	0.072048611
wavelet-HHL	firstorder	Median	19.87160248
wavelet-HHL	firstorder	Energy	624086.8981
wavelet-HHL	firstorder	RobustMeanAbsoluteDeviation	60.41483478
wavelet-HHL	firstorder	MeanAbsoluteDeviation	87.95891766
wavelet-HHL	firstorder	TotalEnergy	16850346.25
wavelet-HHL	firstorder	Maximum	343.618223
wavelet-HHL	firstorder	RootMeanSquared	114.0254813
wavelet-HHL	firstorder	90Percentile	153.9240321
wavelet-HHL	firstorder	Minimum	-178.1764724
wavelet-HHL	firstorder	Entropy	3.957067386
wavelet-HHL	firstorder	Range	521.7946954
wavelet-HHL	firstorder	Variance	12695.85578
wavelet-HHL	firstorder	10Percentile	-128.0525294
wavelet-HHL	firstorder	Kurtosis	3.18939886
wavelet-HHL	firstorder	Mean	17.49155795
wavelet-HHL	glrlm	ShortRunLowGrayLevelEmphasis	0.054239928
wavelet-HHL	glrlm	GrayLevelVariance	20.87524345
wavelet-HHL	glrlm	LowGrayLevelRunEmphasis	0.054831133
wavelet-HHL	glrlm	GrayLevelNonUniformityNormalized	0.071684024
wavelet-HHL	glrlm	RunVariance	0.027152774
wavelet-HHL	glrlm	GrayLevelNonUniformity	3.347186104
wavelet-HHL	glrlm	LongRunEmphasis	1.085890428
wavelet-HHL	glrlm	ShortRunHighGrayLevelEmphasis	103.6522449
wavelet-HHL	glrlm	RunLengthNonUniformity	44.21003215
wavelet-HHL	glrlm	ShortRunEmphasis	0.978527393
wavelet-HHL	glrlm	LongRunHighGrayLevelEmphasis	114.6811502
wavelet-HHL	glrlm	RunPercentage	0.97275641
wavelet-HHL	glrlm	LongRunLowGrayLevelEmphasis	0.057195954
wavelet-HHL	glrlm	RunEntropy	4.033405
wavelet-HHL	glrlm	HighGrayLevelRunEmphasis	105.858026
wavelet-HHL	glrlm	RunLengthNonUniformityNormalized	0.945694451
wavelet-HHL	glszm	GrayLevelVariance	25.30859375
wavelet-HHL	glszm	ZoneVariance	0.625
wavelet-HHL	glszm	GrayLevelNonUniformityNormalized	0.068359375
wavelet-HHL	glszm	SizeZoneNonUniformityNormalized	0.48828125
wavelet-HHL	glszm	SizeZoneNonUniformity	15.625
wavelet-HHL	glszm	GrayLevelNonUniformity	2.1875
wavelet-HHL	glszm	LargeAreaEmphasis	2.875
wavelet-HHL	glszm	SmallAreaHighGrayLevelEmphasis	80.64930556
wavelet-HHL	glszm	ZonePercentage	0.666666667
wavelet-HHL	glszm	LargeAreaLowGrayLevelEmphasis	0.113545548
wavelet-HHL	glszm	LargeAreaHighGrayLevelEmphasis	304.46875
wavelet-HHL	glszm	HighGrayLevelZoneEmphasis	107.4375
wavelet-HHL	glszm	SmallAreaEmphasis	0.723307292
wavelet-HHL	glszm	LowGrayLevelZoneEmphasis	0.066612961
wavelet-HHL	glszm	ZoneEntropy	4.5
wavelet-HHL	glszm	SmallAreaLowGrayLevelEmphasis	0.056633973

wavelet-HHL	ngtdm	Coarseness	0.096396289
wavelet-HHL	ngtdm	Complexity	483.4487963
wavelet-HHL	ngtdm	Strength	10.68848339
wavelet-HHL	ngtdm	Contrast	0.513021243
wavelet-HHL	ngtdm	Busyness	0.084483285
wavelet-HLH	gldm	GrayLevelVariance	52.86414931
wavelet-HLH	gldm	HighGrayLevelEmphasis	351.1458333
wavelet-HLH	gldm	DependenceEntropy	4.777569011
wavelet-HLH	gldm	DependenceNonUniformity	20.29166667
wavelet-HLH	gldm	GrayLevelNonUniformity	2.791666667
wavelet-HLH	gldm	SmallDependenceEmphasis	0.626157407
wavelet-HLH	gldm	SmallDependenceHighGrayLevelEmphasis	213.744213
wavelet-HLH	gldm	DependenceNonUniformityNormalized	0.422743056
wavelet-HLH	gldm	LargeDependenceEmphasis	2.958333333
wavelet-HLH	gldm	LargeDependenceLowGrayLevelEmphasis	0.034971787
wavelet-HLH	gldm	DependenceVariance	0.451388889
wavelet-HLH	gldm	LargeDependenceHighGrayLevelEmphasis	1087.604167
wavelet-HLH	gldm	SmallDependenceLowGrayLevelEmphasis	0.026623978
wavelet-HLH	gldm	LowGrayLevelEmphasis	0.028055567
wavelet-HLH	glcm	JointAverage	17.60236957
wavelet-HLH	glcm	SumAverage	35.20473913
wavelet-HLH	glcm	JointEntropy	5.292567051
wavelet-HLH	glcm	ClusterShade	111.1560583
wavelet-HLH	glcm	MaximumProbability	0.040565147
wavelet-HLH	glcm	Idmn	0.927157189
wavelet-HLH	glcm	JointEnergy	0.026989347
wavelet-HLH	glcm	Contrast	111.3237332
wavelet-HLH	glcm	DifferenceEntropy	3.573219208
wavelet-HLH	glcm	InverseVariance	0.112938158
wavelet-HLH	glcm	DifferenceVariance	34.60257846
wavelet-HLH	glcm	Idn	0.824738966
wavelet-HLH	glcm	Idm	0.114995996
wavelet-HLH	glcm	Correlation	-0.022376922
wavelet-HLH	glcm	Autocorrelation	308.9661208
wavelet-HLH	glcm	SumEntropy	3.917400962
wavelet-HLH	glcm	SumSquares	54.64163853
wavelet-HLH	glcm	ClusterProminence	33409.46636
wavelet-HLH	glcm	Imc2	0.998580048
wavelet-HLH	glcm	Imc1	-0.723663831
wavelet-HLH	glcm	DifferenceAverage	8.309262424
wavelet-HLH	glcm	Id	0.201734417
wavelet-HLH	glcm	ClusterTendency	107.2428209
wavelet-HLH	firstorder	InterquartileRange	209.7829601
wavelet-HLH	firstorder	Skewness	0.200108883
wavelet-HLH	firstorder	Uniformity	0.058159722
wavelet-HLH	firstorder	Median	23.55187836
wavelet-HLH	firstorder	Energy	1623962.84
wavelet-HLH	firstorder	RobustMeanAbsoluteDeviation	91.59520905
wavelet-HLH	firstorder	MeanAbsoluteDeviation	141.0439847
wavelet-HLH	firstorder	TotalEnergy	43846996.67
wavelet-HLH	firstorder	Maximum	463.2229923
wavelet-HLH	firstorder	RootMeanSquared	183.936291
wavelet-HLH	firstorder	90Percentile	269.422826
wavelet-HLH	firstorder	Minimum	-379.6981828
wavelet-HLH	firstorder	Entropy	4.304047012

wavelet-HLH	firstorder	Range	842.9211751
wavelet-HLH	firstorder	Variance	33463.34228
wavelet-HLH	firstorder	10Percentile	-202.6308603
wavelet-HLH	firstorder	Kurtosis	2.94564709
wavelet-HLH	firstorder	Mean	19.21501698
wavelet-HLH	glrlm	ShortRunLowGrayLevelEmphasis	0.028569907
wavelet-HLH	glrlm	GrayLevelVariance	53.45343002
wavelet-HLH	glrlm	LowGrayLevelRunEmphasis	0.028634625
wavelet-HLH	glrlm	GrayLevelNonUniformityNormalized	0.057730878
wavelet-HLH	glrlm	RunVariance	0.022352626
wavelet-HLH	glrlm	GrayLevelNonUniformity	2.709194791
wavelet-HLH	glrlm	LongRunEmphasis	1.071146245
wavelet-HLH	glrlm	ShortRunHighGrayLevelEmphasis	343.94887
wavelet-HLH	glrlm	RunLengthNonUniformity	44.89206446
wavelet-HLH	glrlm	ShortRunEmphasis	0.982213439
wavelet-HLH	glrlm	LongRunHighGrayLevelEmphasis	377.2157254
wavelet-HLH	glrlm	RunPercentage	0.977564103
wavelet-HLH	glrlm	LongRunLowGrayLevelEmphasis	0.028893498
wavelet-HLH	glrlm	RunEntropy	4.360803847
wavelet-HLH	glrlm	HighGrayLevelRunEmphasis	350.6022411
wavelet-HLH	glrlm	RunLengthNonUniformityNormalized	0.955294748
wavelet-HLH	glszm	GrayLevelVariance	61.64571429
wavelet-HLH	glszm	ZoneVariance	0.404897959
wavelet-HLH	glszm	GrayLevelNonUniformityNormalized	0.051428571
wavelet-HLH	glszm	SizeZoneNonUniformityNormalized	0.55755102
wavelet-HLH	glszm	SizeZoneNonUniformity	19.51428571
wavelet-HLH	glszm	GrayLevelNonUniformity	1.8
wavelet-HLH	glszm	LargeAreaEmphasis	2.285714286
wavelet-HLH	glszm	SmallAreaHighGrayLevelEmphasis	261.8880952
wavelet-HLH	glszm	ZonePercentage	0.729166667
wavelet-HLH	glszm	LargeAreaLowGrayLevelEmphasis	0.04169888
wavelet-HLH	glszm	LargeAreaHighGrayLevelEmphasis	826.5428571
wavelet-HLH	glszm	HighGrayLevelZoneEmphasis	343.8857143
wavelet-HLH	glszm	SmallAreaEmphasis	0.773809524
wavelet-HLH	glszm	LowGrayLevelZoneEmphasis	0.03708917
wavelet-HLH	glszm	ZoneEntropy	4.729283017
wavelet-HLH	glszm	SmallAreaLowGrayLevelEmphasis	0.036185967
wavelet-HLH	ngtdm	Coarseness	0.08176811
wavelet-HLH	ngtdm	Complexity	1571.779827
wavelet-HLH	ngtdm	Strength	21.46330704
wavelet-HLH	ngtdm	Contrast	1.279880698
wavelet-HLH	ngtdm	Busyness	0.038247715

Image type	Feature Class	Feature Name	502: RTSTRUCT: C99-DNU_segment_GTV
general	info	BoundingBox	(0, 0, 1, 8, 9, 8)
general	info	EnabledImageTypes	{u'Original': {}, u'Wavelet': {}}
general	info	GeneralSettings	{'distances': [1], 'additionalInfo': True, 'force2D': False, 'interpolator': 'sitkBSpline', 'resampledPixelSpacing': [3.0], 'label': 1, 'normalizeScale': 1, 'normalize': False, 'force2Ddimension': 0, 'removeOutliers': None, 'minimumROISize': None, u'binWidth': 25.0, 'minimumROIDimensions': 1, u'correctMask': True, 'preCrop': False, u'symmetricalGLCM': True, 'resegmentRange': None, 'padDistance': 5}
general	info	ImageHash	2613ada59c4ec173b0181541f10145f9c39ed3b6
general	info	ImageSpacing	(0.9765625000000002, 0.9765625000000002, 2.0)
general	info	MaskHash	6b62bcb21eccdfed8c631916314275b29fe18170
general	info	NumpyVersion	1.13.1
general	info	PyWaveletVersion	0.5.2
general	info	SimpleITKVersion	1.1.0
general	info	Version	1.3.0.post102+g1b0eb74
general	info	VolumeNum	1
general	info	VoxelNum	233
original	shape	Maximum3DDiameter	26.4952826
original	shape	Maximum2DDiameterSlice	24.18677324
original	shape	Sphericity	0.836787372
original	shape	MinorAxis	21.09864038
original	shape	Elongation	0.89313084
original	shape	SurfaceVolumeRatio	0.313061103
original	shape	Volume	6291
original	shape	MajorAxis	23.62323575
original	shape	SurfaceArea	1969.467402
original	shape	Flatness	0.819117552
original	shape	LeastAxis	19.35020704
original	shape	Maximum2DDiameterColumn	25.8069758
original	shape	Maximum2DDiameterRow	25.63201124
original	gldm	GrayLevelVariance	44.62141502
original	gldm	HighGrayLevelEmphasis	486.8841202
original	gldm	DependenceEntropy	6.206215278
original	gldm	DependenceNonUniformity	55.54935622
original	gldm	GrayLevelNonUniformity	11.22317597
original	gldm	SmallDependenceEmphasis	0.370556973
original	gldm	SmallDependenceHighGrayLevelEmphasis	167.1899103
original	gldm	DependenceNonUniformityNormalized	0.238409254
original	gldm	LargeDependenceEmphasis	7.901287554
original	gldm	LargeDependenceLowGrayLevelEmphasis	0.029771437
original	gldm	DependenceVariance	1.726169206
original	gldm	LargeDependenceHighGrayLevelEmphasis	4341.236052
original	gldm	SmallDependenceLowGrayLevelEmphasis	0.00658196

original	gldm	LowGrayLevelEmphasis	0.008705658
original	glcm	JointAverage	21.4543418
original	glcm	SumAverage	42.9086836
original	glcm	JointEntropy	7.428406734
original	glcm	ClusterShade	-879.3975693
original	glcm	MaximumProbability	0.017970421
original	glcm	Idmn	0.974548968
original	glcm	JointEnergy	0.006774966
original	glcm	Contrast	28.39121744
original	glcm	DifferenceEntropy	3.399344399
original	glcm	InverseVariance	0.217712255
original	glcm	DifferenceVariance	10.26929395
original	glcm	Idn	0.891897625
original	glcm	Idm	0.213688204
original	glcm	Correlation	0.635921263
original	glcm	Autocorrelation	485.0668303
original	glcm	SumEntropy	5.140312548
original	glcm	SumSquares	38.93247477
original	glcm	ClusterProminence	44854.52874
original	glcm	Imc2	0.973623958
original	glcm	Imc1	-0.334430958
original	glcm	DifferenceAverage	4.158983924
original	glcm	Id	0.308077352
original	glcm	ClusterTendency	127.3386816
original	firstorder	InterquartileRange	235
original	firstorder	Skewness	-0.675866586
original	firstorder	Uniformity	0.048168137
original	firstorder	Median	-237
original	firstorder	Energy	22528691
original	firstorder	RobustMeanAbsoluteDeviation	98.42974434
original	firstorder	MeanAbsoluteDeviation	136.6271252
original	firstorder	TotalEnergy	608274657
original	firstorder	Maximum	14
original	firstorder	RootMeanSquared	310.9496116
original	firstorder	90Percentile	-64.2
original	firstorder	Minimum	-771
original	firstorder	Entropy	4.544692394
original	firstorder	Range	785
original	firstorder	Variance	28025.41911
original	firstorder	10Percentile	-512.2
original	firstorder	Kurtosis	2.823462193
original	firstorder	Mean	-262.0386266
original	glrlm	ShortRunLowGrayLevelEmphasis	0.0089036
original	glrlm	GrayLevelVariance	44.78613504
original	glrlm	LowGrayLevelRunEmphasis	0.009041351
original	glrlm	GrayLevelNonUniformityNormalized	0.047795599
original	glrlm	RunVariance	0.067493395
original	glrlm	GrayLevelNonUniformity	10.5007065
original	glrlm	LongRunEmphasis	1.195197659
original	glrlm	ShortRunHighGrayLevelEmphasis	461.5445394
original	glrlm	RunLengthNonUniformity	196.796364
original	glrlm	ShortRunEmphasis	0.956990773
original	glrlm	LongRunHighGrayLevelEmphasis	588.4097002

original	glrlm	RunPercentage	0.942885441
original	glrlm	LongRunLowGrayLevelEmphasis	0.009679354
original	glrlm	RunEntropy	4.782810046
original	glrlm	HighGrayLevelRunEmphasis	484.2109525
original	glrlm	RunLengthNonUniformityNormalized	0.894382892
original	glszm	GrayLevelVariance	50.98940904
original	glszm	ZoneVariance	4.121128515
original	glszm	GrayLevelNonUniformityNormalized	0.046635813
original	glszm	SizeZoneNonUniformityNormalized	0.365966536
original	glszm	SizeZoneNonUniformity	38.79245283
original	glszm	GrayLevelNonUniformity	4.943396226
original	glszm	LargeAreaEmphasis	8.952830189
original	glszm	SmallAreaHighGrayLevelEmphasis	272.3884152
original	glszm	ZonePercentage	0.454935622
original	glszm	LargeAreaLowGrayLevelEmphasis	0.038599107
original	glszm	LargeAreaHighGrayLevelEmphasis	4733.490566
original	glszm	HighGrayLevelZoneEmphasis	456.6698113
original	glszm	SmallAreaEmphasis	0.626000247
original	glszm	LowGrayLevelZoneEmphasis	0.015157712
original	glszm	ZoneEntropy	5.72779528
original	glszm	SmallAreaLowGrayLevelEmphasis	0.013827983
original	ngtdm	Coarseness	0.038514986
original	ngtdm	Complexity	1012.354615
original	ngtdm	Strength	14.52877496
original	ngtdm	Contrast	0.24978163
original	ngtdm	Busyness	0.039235175
wavelet-HHH	gldm	GrayLevelVariance	2.580891157
wavelet-HHH	gldm	HighGrayLevelEmphasis	59.76824034
wavelet-HHH	gldm	DependenceEntropy	5.177943716
wavelet-HHH	gldm	DependenceNonUniformity	31.17167382
wavelet-HHH	gldm	GrayLevelNonUniformity	42.31330472
wavelet-HHH	gldm	SmallDependenceEmphasis	0.182033262
wavelet-HHH	gldm	SmallDependenceHighGrayLevelEmphasis	12.40150976
wavelet-HHH	gldm	DependenceNonUniformityNormalized	0.133784008
wavelet-HHH	gldm	LargeDependenceEmphasis	21.9527897
wavelet-HHH	gldm	LargeDependenceLowGrayLevelEmphasis	0.414119644
wavelet-HHH	gldm	DependenceVariance	4.870710457
wavelet-HHH	gldm	LargeDependenceHighGrayLevelEmphasis	1259.914163
wavelet-HHH	gldm	SmallDependenceLowGrayLevelEmphasis	0.007999678
wavelet-HHH	gldm	LowGrayLevelEmphasis	0.024164248
wavelet-HHH	glcm	JointAverage	7.532312389
wavelet-HHH	glcm	SumAverage	15.06462478
wavelet-HHH	glcm	JointEntropy	5.067665377
wavelet-HHH	glcm	ClusterShade	0.45483101
wavelet-HHH	glcm	MaximumProbability	0.077160253
wavelet-HHH	glcm	Idmn	0.968516338
wavelet-HHH	glcm	JointEnergy	0.03843043
wavelet-HHH	glcm	Contrast	5.006421513
wavelet-HHH	glcm	DifferenceEntropy	2.327065494
wavelet-HHH	glcm	InverseVariance	0.416372107
wavelet-HHH	glcm	DifferenceVariance	1.911150183
wavelet-HHH	glcm	Idn	0.88149153
wavelet-HHH	glcm	Idm	0.409558741

wavelet-HHH	glcm	Correlation	-0.051258907
wavelet-HHH	glcm	Autocorrelation	56.61405289
wavelet-HHH	glcm	SumEntropy	3.040767849
wavelet-HHH	glcm	SumSquares	2.380231963
wavelet-HHH	glcm	ClusterProminence	65.24050217
wavelet-HHH	glcm	Imc2	0.581315346
wavelet-HHH	glcm	Imc1	-0.081462782
wavelet-HHH	glcm	DifferenceAverage	1.745046735
wavelet-HHH	glcm	Id	0.477233323
wavelet-HHH	glcm	ClusterTendency	4.514506341
wavelet-HHH	firstorder	InterquartileRange	49.39968858
wavelet-HHH	firstorder	Skewness	0.011234116
wavelet-HHH	firstorder	Uniformity	0.181602166
wavelet-HHH	firstorder	Median	0.045535456
wavelet-HHH	firstorder	Energy	362039.6723
wavelet-HHH	firstorder	RobustMeanAbsoluteDeviation	21.20393994
wavelet-HHH	firstorder	MeanAbsoluteDeviation	30.79777893
wavelet-HHH	firstorder	TotalEnergy	9775071.151
wavelet-HHH	firstorder	Maximum	123.6357343
wavelet-HHH	firstorder	RootMeanSquared	39.41850246
wavelet-HHH	firstorder	90Percentile	53.22993906
wavelet-HHH	firstorder	Minimum	-157.5774285
wavelet-HHH	firstorder	Entropy	2.689494917
wavelet-HHH	firstorder	Range	281.2131628
wavelet-HHH	firstorder	Variance	1552.343188
wavelet-HHH	firstorder	10Percentile	-45.54383765
wavelet-HHH	firstorder	Kurtosis	3.745781169
wavelet-HHH	firstorder	Mean	1.21455679
wavelet-HHH	glrlm	ShortRunLowGrayLevelEmphasis	0.023124696
wavelet-HHH	glrlm	GrayLevelVariance	2.777032194
wavelet-HHH	glrlm	LowGrayLevelRunEmphasis	0.02487644
wavelet-HHH	glrlm	GrayLevelNonUniformityNormalized	0.172954653
wavelet-HHH	glrlm	RunVariance	0.158727532
wavelet-HHH	glrlm	GrayLevelNonUniformity	35.43942923
wavelet-HHH	glrlm	LongRunEmphasis	1.455796264
wavelet-HHH	glrlm	ShortRunHighGrayLevelEmphasis	54.78121126
wavelet-HHH	glrlm	RunLengthNonUniformity	161.7736182
wavelet-HHH	glrlm	ShortRunEmphasis	0.907811741
wavelet-HHH	glrlm	LongRunHighGrayLevelEmphasis	86.16363046
wavelet-HHH	glrlm	RunPercentage	0.879498184
wavelet-HHH	glrlm	LongRunLowGrayLevelEmphasis	0.033552534
wavelet-HHH	glrlm	RunEntropy	3.274164024
wavelet-HHH	glrlm	HighGrayLevelRunEmphasis	60.09460164
wavelet-HHH	glrlm	RunLengthNonUniformityNormalized	0.787813933
wavelet-HHH	glszm	GrayLevelVariance	6.68537415
wavelet-HHH	glszm	ZoneVariance	153.6286848
wavelet-HHH	glszm	GrayLevelNonUniformityNormalized	0.139455782
wavelet-HHH	glszm	SizeZoneNonUniformityNormalized	0.339002268
wavelet-HHH	glszm	SizeZoneNonUniformity	14.23809524
wavelet-HHH	glszm	GrayLevelNonUniformity	5.857142857
wavelet-HHH	glszm	LargeAreaEmphasis	184.4047619
wavelet-HHH	glszm	SmallAreaHighGrayLevelEmphasis	44.34512367
wavelet-HHH	glszm	ZonePercentage	0.180257511

wavelet-HHH	glszm	LargeAreaLowGrayLevelEmphasis	3.515972048
wavelet-HHH	glszm	LargeAreaHighGrayLevelEmphasis	10215.7619
wavelet-HHH	glszm	HighGrayLevelZoneEmphasis	69.54761905
wavelet-HHH	glszm	SmallAreaEmphasis	0.599176953
wavelet-HHH	glszm	LowGrayLevelZoneEmphasis	0.044564998
wavelet-HHH	glszm	ZoneEntropy	4.451608494
wavelet-HHH	glszm	SmallAreaLowGrayLevelEmphasis	0.035876443
wavelet-HHH	ngtdm	Coarseness	0.024848429
wavelet-HHH	ngtdm	Complexity	57.33275983
wavelet-HHH	ngtdm	Strength	0.822615851
wavelet-HHH	ngtdm	Contrast	0.07815665
wavelet-HHH	ngtdm	Busyness	0.525490371
wavelet-HLL	gldm	GrayLevelVariance	20.36729356
wavelet-HLL	gldm	HighGrayLevelEmphasis	109.6008584
wavelet-HLL	gldm	DependenceEntropy	5.736936012
wavelet-HLL	gldm	DependenceNonUniformity	36.44206009
wavelet-HLL	gldm	GrayLevelNonUniformity	26.03004292
wavelet-HLL	gldm	SmallDependenceEmphasis	0.259601311
wavelet-HLL	gldm	SmallDependenceHighGrayLevelEmphasis	42.97905701
wavelet-HLL	gldm	DependenceNonUniformityNormalized	0.156403691
wavelet-HLL	gldm	LargeDependenceEmphasis	16.88841202
wavelet-HLL	gldm	LargeDependenceLowGrayLevelEmphasis	0.282437829
wavelet-HLL	gldm	DependenceVariance	4.533036158
wavelet-HLL	gldm	LargeDependenceHighGrayLevelEmphasis	1312.055794
wavelet-HLL	gldm	SmallDependenceLowGrayLevelEmphasis	0.009585325
wavelet-HLL	gldm	LowGrayLevelEmphasis	0.024308995
wavelet-HLL	glcm	JointAverage	9.117446914
wavelet-HLL	glcm	SumAverage	18.23489383
wavelet-HLL	glcm	JointEntropy	6.246853777
wavelet-HLL	glcm	ClusterShade	535.9834371
wavelet-HLL	glcm	MaximumProbability	0.048790979
wavelet-HLL	glcm	Idmn	0.973806519
wavelet-HLL	glcm	JointEnergy	0.018591862
wavelet-HLL	glcm	Contrast	21.17313599
wavelet-HLL	glcm	DifferenceEntropy	2.971270641
wavelet-HLL	glcm	InverseVariance	0.331669235
wavelet-HLL	glcm	DifferenceVariance	11.24455024
wavelet-HLL	glcm	Idn	0.906166335
wavelet-HLL	glcm	Idm	0.329899765
wavelet-HLL	glcm	Correlation	0.310116774
wavelet-HLL	glcm	Autocorrelation	89.04931544
wavelet-HLL	glcm	SumEntropy	4.131432929
wavelet-HLL	glcm	SumSquares	16.45429754
wavelet-HLL	glcm	ClusterProminence	14530.74012
wavelet-HLL	glcm	Imc2	0.862961215
wavelet-HLL	glcm	Imc1	-0.211494344
wavelet-HLL	glcm	DifferenceAverage	3.025251299
wavelet-HLL	glcm	Id	0.406942185
wavelet-HLL	glcm	ClusterTendency	44.64405419
wavelet-HLL	firstorder	InterquartileRange	76.63003964
wavelet-HLL	firstorder	Skewness	1.799691265
wavelet-HLL	firstorder	Uniformity	0.111716922
wavelet-HLL	firstorder	Median	-0.588105927

wavelet-HLL	firstorder	Energy	3053088.315
wavelet-HLL	firstorder	RobustMeanAbsoluteDeviation	33.93873438
wavelet-HLL	firstorder	MeanAbsoluteDeviation	73.35760674
wavelet-HLL	firstorder	TotalEnergy	82433384.5
wavelet-HLL	firstorder	Maximum	448.7922782
wavelet-HLL	firstorder	RootMeanSquared	114.4700106
wavelet-HLL	firstorder	90Percentile	190.9198335
wavelet-HLL	firstorder	Minimum	-195.2508769
wavelet-HLL	firstorder	Entropy	3.625485313
wavelet-HLL	firstorder	Range	644.0431551
wavelet-HLL	firstorder	Variance	12529.43617
wavelet-HLL	firstorder	10Percentile	-72.43481011
wavelet-HLL	firstorder	Kurtosis	6.60168491
wavelet-HLL	firstorder	Mean	23.95719422
wavelet-HLL	glrlm	ShortRunLowGrayLevelEmphasis	0.023719168
wavelet-HLL	glrlm	GrayLevelVariance	21.78409449
wavelet-HLL	glrlm	LowGrayLevelRunEmphasis	0.025030818
wavelet-HLL	glrlm	GrayLevelNonUniformityNormalized	0.105309974
wavelet-HLL	glrlm	RunVariance	0.12482579
wavelet-HLL	glrlm	GrayLevelNonUniformity	22.16355432
wavelet-HLL	glrlm	LongRunEmphasis	1.355216704
wavelet-HLL	glrlm	ShortRunHighGrayLevelEmphasis	107.2060342
wavelet-HLL	glrlm	RunLengthNonUniformity	174.90801
wavelet-HLL	glrlm	ShortRunEmphasis	0.927312706
wavelet-HLL	glrlm	LongRunHighGrayLevelEmphasis	140.9272953
wavelet-HLL	glrlm	RunPercentage	0.903268405
wavelet-HLL	glrlm	LongRunLowGrayLevelEmphasis	0.031205408
wavelet-HLL	glrlm	RunEntropy	4.10548362
wavelet-HLL	glrlm	HighGrayLevelRunEmphasis	112.9386737
wavelet-HLL	glrlm	RunLengthNonUniformityNormalized	0.829154605
wavelet-HLL	glszm	GrayLevelVariance	40.26381012
wavelet-HLL	glszm	ZoneVariance	45.16236085
wavelet-HLL	glszm	GrayLevelNonUniformityNormalized	0.064902331
wavelet-HLL	glszm	SizeZoneNonUniformityNormalized	0.377021634
wavelet-HLL	glszm	SizeZoneNonUniformity	26.01449275
wavelet-HLL	glszm	GrayLevelNonUniformity	4.47826087
wavelet-HLL	glszm	LargeAreaEmphasis	56.56521739
wavelet-HLL	glszm	SmallAreaHighGrayLevelEmphasis	118.4570423
wavelet-HLL	glszm	ZonePercentage	0.296137339
wavelet-HLL	glszm	LargeAreaLowGrayLevelEmphasis	0.905455233
wavelet-HLL	glszm	LargeAreaHighGrayLevelEmphasis	4185.202899
wavelet-HLL	glszm	HighGrayLevelZoneEmphasis	169.6956522
wavelet-HLL	glszm	SmallAreaEmphasis	0.624880401
wavelet-HLL	glszm	LowGrayLevelZoneEmphasis	0.037279353
wavelet-HLL	glszm	ZoneEntropy	5.124232472
wavelet-HLL	glszm	SmallAreaLowGrayLevelEmphasis	0.027034835
wavelet-HLL	ngtdm	Coarseness	0.025045618
wavelet-HLL	ngtdm	Complexity	591.3025846
wavelet-HLL	ngtdm	Strength	8.218994074
wavelet-HLL	ngtdm	Contrast	0.16956864
wavelet-HLL	ngtdm	Busyness	0.160291958
wavelet-LHL	gldm	GrayLevelVariance	67.33964523
wavelet-LHL	gldm	HighGrayLevelEmphasis	2969.450644

wavelet-LHL	gldm	DependenceEntropy	5.870624234
wavelet-LHL	gldm	DependenceNonUniformity	50.87124464
wavelet-LHL	gldm	GrayLevelNonUniformity	17.25751073
wavelet-LHL	gldm	SmallDependenceEmphasis	0.352426644
wavelet-LHL	gldm	SmallDependenceHighGrayLevelEmphasis	962.5475203
wavelet-LHL	gldm	DependenceNonUniformityNormalized	0.218331522
wavelet-LHL	gldm	LargeDependenceEmphasis	8.545064378
wavelet-LHL	gldm	LargeDependenceLowGrayLevelEmphasis	0.007196088
wavelet-LHL	gldm	DependenceVariance	1.758256737
wavelet-LHL	gldm	LargeDependenceHighGrayLevelEmphasis	26337.38197
wavelet-LHL	gldm	SmallDependenceLowGrayLevelEmphasis	0.004516495
wavelet-LHL	gldm	LowGrayLevelEmphasis	0.004728395
wavelet-LHL	glcm	JointAverage	54.61203906
wavelet-LHL	glcm	SumAverage	109.2240781
wavelet-LHL	glcm	JointEntropy	7.012348584
wavelet-LHL	glcm	ClusterShade	-4812.437166
wavelet-LHL	glcm	MaximumProbability	0.024057572
wavelet-LHL	glcm	Idmn	0.986260007
wavelet-LHL	glcm	JointEnergy	0.009398756
wavelet-LHL	glcm	Contrast	72.53589033
wavelet-LHL	glcm	DifferenceEntropy	3.407963779
wavelet-LHL	glcm	InverseVariance	0.253343261
wavelet-LHL	glcm	DifferenceVariance	47.78509719
wavelet-LHL	glcm	Idn	0.937683019
wavelet-LHL	glcm	Idm	0.238798135
wavelet-LHL	glcm	Correlation	0.266497494
wavelet-LHL	glcm	Autocorrelation	2994.558288
wavelet-LHL	glcm	SumEntropy	4.53878847
wavelet-LHL	glcm	SumSquares	48.32846
wavelet-LHL	glcm	ClusterProminence	333103.5183
wavelet-LHL	glcm	Imc2	0.910115345
wavelet-LHL	glcm	Imc1	-0.228509752
wavelet-LHL	glcm	DifferenceAverage	4.768022967
wavelet-LHL	glcm	Id	0.326752763
wavelet-LHL	glcm	ClusterTendency	120.7779497
wavelet-LHL	firstorder	InterquartileRange	134.580258
wavelet-LHL	firstorder	Skewness	-3.700685784
wavelet-LHL	firstorder	Uniformity	0.07406657
wavelet-LHL	firstorder	Median	-12.6818723
wavelet-LHL	firstorder	Energy	10221739.73
wavelet-LHL	firstorder	RobustMeanAbsoluteDeviation	54.75252468
wavelet-LHL	firstorder	MeanAbsoluteDeviation	109.8157299
wavelet-LHL	firstorder	TotalEnergy	275986972.6
wavelet-LHL	firstorder	Maximum	217.0080702
wavelet-LHL	firstorder	RootMeanSquared	209.4519696
wavelet-LHL	firstorder	90Percentile	115.0579308
wavelet-LHL	firstorder	Minimum	-1367.316887
wavelet-LHL	firstorder	Entropy	4.023912624
wavelet-LHL	firstorder	Range	1584.324957
wavelet-LHL	firstorder	Variance	42215.78931
wavelet-LHL	firstorder	10Percentile	-139.128132
wavelet-LHL	firstorder	Kurtosis	19.54658583
wavelet-LHL	firstorder	Mean	-40.67355738

wavelet-LHL	glrlm	ShortRunLowGrayLevelEmphasis	0.005008352
wavelet-LHL	glrlm	GrayLevelVariance	70.93617262
wavelet-LHL	glrlm	LowGrayLevelRunEmphasis	0.005023185
wavelet-LHL	glrlm	GrayLevelNonUniformityNormalized	0.073395347
wavelet-LHL	glrlm	RunVariance	0.078816269
wavelet-LHL	glrlm	GrayLevelNonUniformity	16.04250524
wavelet-LHL	glrlm	LongRunEmphasis	1.21864792
wavelet-LHL	glrlm	ShortRunHighGrayLevelEmphasis	2818.572041
wavelet-LHL	glrlm	RunLengthNonUniformity	194.5687432
wavelet-LHL	glrlm	ShortRunEmphasis	0.954348218
wavelet-LHL	glrlm	LongRunHighGrayLevelEmphasis	3632.833327
wavelet-LHL	glrlm	RunPercentage	0.938263453
wavelet-LHL	glrlm	LongRunLowGrayLevelEmphasis	0.005095325
wavelet-LHL	glrlm	RunEntropy	4.318086655
wavelet-LHL	glrlm	HighGrayLevelRunEmphasis	2960.996549
wavelet-LHL	glrlm	RunLengthNonUniformityNormalized	0.888495337
wavelet-LHL	glszm	GrayLevelVariance	140.0724826
wavelet-LHL	glszm	ZoneVariance	8.390516493
wavelet-LHL	glszm	GrayLevelNonUniformityNormalized	0.059678819
wavelet-LHL	glszm	SizeZoneNonUniformityNormalized	0.392144097
wavelet-LHL	glszm	SizeZoneNonUniformity	37.64583333
wavelet-LHL	glszm	GrayLevelNonUniformity	5.729166667
wavelet-LHL	glszm	LargeAreaEmphasis	14.28125
wavelet-LHL	glszm	SmallAreaHighGrayLevelEmphasis	1699.098163
wavelet-LHL	glszm	ZonePercentage	0.412017167
wavelet-LHL	glszm	LargeAreaLowGrayLevelEmphasis	0.015406839
wavelet-LHL	glszm	LargeAreaHighGrayLevelEmphasis	43262.75
wavelet-LHL	glszm	HighGrayLevelZoneEmphasis	2803.0625
wavelet-LHL	glszm	SmallAreaEmphasis	0.649802502
wavelet-LHL	glszm	LowGrayLevelZoneEmphasis	0.011007655
wavelet-LHL	glszm	ZoneEntropy	5.561372266
wavelet-LHL	glszm	SmallAreaLowGrayLevelEmphasis	0.010894662
wavelet-LHL	ngtdm	Coarseness	0.024459554
wavelet-LHL	ngtdm	Complexity	2184.603642
wavelet-LHL	ngtdm	Strength	32.62526627
wavelet-LHL	ngtdm	Contrast	0.700997246
wavelet-LHL	ngtdm	Busyness	0.024732653
wavelet-LHH	gldm	GrayLevelVariance	8.85811122
wavelet-LHH	gldm	HighGrayLevelEmphasis	80.48927039
wavelet-LHH	gldm	DependenceEntropy	5.578571863
wavelet-LHH	gldm	DependenceNonUniformity	43.88412017
wavelet-LHH	gldm	GrayLevelNonUniformity	23.31759657
wavelet-LHH	gldm	SmallDependenceEmphasis	0.321788318
wavelet-LHH	gldm	SmallDependenceHighGrayLevelEmphasis	25.04106507
wavelet-LHH	gldm	DependenceNonUniformityNormalized	0.188343863
wavelet-LHH	gldm	LargeDependenceEmphasis	11.59227468
wavelet-LHH	gldm	LargeDependenceLowGrayLevelEmphasis	0.199484557
wavelet-LHH	gldm	DependenceVariance	2.847943414
wavelet-LHH	gldm	LargeDependenceHighGrayLevelEmphasis	954.6351931
wavelet-LHH	gldm	SmallDependenceLowGrayLevelEmphasis	0.018758851
wavelet-LHH	gldm	LowGrayLevelEmphasis	0.032652823
wavelet-LHH	glcm	JointAverage	8.524674947
wavelet-LHH	glcm	SumAverage	17.04934989

wavelet-LHH	glcm	JointEntropy	6.429029144
wavelet-LHH	glcm	ClusterShade	-4.840238794
wavelet-LHH	glcm	MaximumProbability	0.037626115
wavelet-LHH	glcm	Idmn	0.95620666
wavelet-LHH	glcm	JointEnergy	0.014736021
wavelet-LHH	glcm	Contrast	16.41579074
wavelet-LHH	glcm	DifferenceEntropy	3.063852448
wavelet-LHH	glcm	InverseVariance	0.272568501
wavelet-LHH	glcm	DifferenceVariance	6.236617643
wavelet-LHH	glcm	Idn	0.862282577
wavelet-LHH	glcm	Idm	0.269990892
wavelet-LHH	glcm	Correlation	-0.001157765
wavelet-LHH	glcm	Autocorrelation	72.68903254
wavelet-LHH	glcm	SumEntropy	3.917448695
wavelet-LHH	glcm	SumSquares	8.221791683
wavelet-LHH	glcm	ClusterProminence	913.5417815
wavelet-LHH	glcm	Imc2	0.801023869
wavelet-LHH	glcm	Imc1	-0.150106896
wavelet-LHH	glcm	DifferenceAverage	3.150213584
wavelet-LHH	glcm	Id	0.360524291
wavelet-LHH	glcm	ClusterTendency	16.47137599
wavelet-LHH	firstorder	InterquartileRange	101.1451338
wavelet-LHH	firstorder	Skewness	0.051180468
wavelet-LHH	firstorder	Uniformity	0.100075522
wavelet-LHH	firstorder	Median	-4.972836387
wavelet-LHH	firstorder	Energy	1264271.523
wavelet-LHH	firstorder	RobustMeanAbsoluteDeviation	40.10129236
wavelet-LHH	firstorder	MeanAbsoluteDeviation	58.53707806
wavelet-LHH	firstorder	TotalEnergy	34135331.12
wavelet-LHH	firstorder	Maximum	232.8241207
wavelet-LHH	firstorder	RootMeanSquared	73.6617814
wavelet-LHH	firstorder	90Percentile	90.37906201
wavelet-LHH	firstorder	Minimum	-199.3494455
wavelet-LHH	firstorder	Entropy	3.551718891
wavelet-LHH	firstorder	Range	432.1735662
wavelet-LHH	firstorder	Variance	5424.216945
wavelet-LHH	firstorder	10Percentile	-99.19629291
wavelet-LHH	firstorder	Kurtosis	3.096394759
wavelet-LHH	firstorder	Mean	-1.35686918
wavelet-LHH	glrlm	ShortRunLowGrayLevelEmphasis	0.032944568
wavelet-LHH	glrlm	GrayLevelVariance	9.191467391
wavelet-LHH	glrlm	LowGrayLevelRunEmphasis	0.033935189
wavelet-LHH	glrlm	GrayLevelNonUniformityNormalized	0.097294049
wavelet-LHH	glrlm	RunVariance	0.093904404
wavelet-LHH	glrlm	GrayLevelNonUniformity	20.97063588
wavelet-LHH	glrlm	LongRunEmphasis	1.267965518
wavelet-LHH	glrlm	ShortRunHighGrayLevelEmphasis	75.5972459
wavelet-LHH	glrlm	RunLengthNonUniformity	186.2437367
wavelet-LHH	glrlm	ShortRunEmphasis	0.943026307
wavelet-LHH	glrlm	LongRunHighGrayLevelEmphasis	102.3212121
wavelet-LHH	glrlm	RunPercentage	0.924727633
wavelet-LHH	glrlm	LongRunLowGrayLevelEmphasis	0.03849765
wavelet-LHH	glrlm	RunEntropy	3.921249438

wavelet-LHH	glrlm	HighGrayLevelRunEmphasis	80.31709838
wavelet-LHH	glrlm	RunLengthNonUniformityNormalized	0.862595734
wavelet-LHH	glszm	GrayLevelVariance	14.14612234
wavelet-LHH	glszm	ZoneVariance	18.10331616
wavelet-LHH	glszm	GrayLevelNonUniformityNormalized	0.085480248
wavelet-LHH	glszm	SizeZoneNonUniformityNormalized	0.401770379
wavelet-LHH	glszm	SizeZoneNonUniformity	34.95402299
wavelet-LHH	glszm	GrayLevelNonUniformity	7.436781609
wavelet-LHH	glszm	LargeAreaEmphasis	25.27586207
wavelet-LHH	glszm	SmallAreaHighGrayLevelEmphasis	50.6171659
wavelet-LHH	glszm	ZonePercentage	0.373390558
wavelet-LHH	glszm	LargeAreaLowGrayLevelEmphasis	0.403931675
wavelet-LHH	glszm	LargeAreaHighGrayLevelEmphasis	2166.505747
wavelet-LHH	glszm	HighGrayLevelZoneEmphasis	77.22988506
wavelet-LHH	glszm	SmallAreaEmphasis	0.656285081
wavelet-LHH	glszm	LowGrayLevelZoneEmphasis	0.055912426
wavelet-LHH	glszm	ZoneEntropy	5.036005243
wavelet-LHH	glszm	SmallAreaLowGrayLevelEmphasis	0.045350215
wavelet-LHH	ngtdm	Coarseness	0.024560992
wavelet-LHH	ngtdm	Complexity	265.6376437
wavelet-LHH	ngtdm	Strength	2.074446105
wavelet-LHH	ngtdm	Contrast	0.156734255
wavelet-LHH	ngtdm	Busyness	0.285396741
wavelet-LLH	gldm	GrayLevelVariance	20.21956566
wavelet-LLH	gldm	HighGrayLevelEmphasis	247.0257511
wavelet-LLH	gldm	DependenceEntropy	5.87748764
wavelet-LLH	gldm	DependenceNonUniformity	56.95708155
wavelet-LLH	gldm	GrayLevelNonUniformity	15.21459227
wavelet-LLH	gldm	SmallDependenceEmphasis	0.409293038
wavelet-LLH	gldm	SmallDependenceHighGrayLevelEmphasis	103.2561826
wavelet-LLH	gldm	DependenceNonUniformityNormalized	0.244450994
wavelet-LLH	gldm	LargeDependenceEmphasis	7.060085837
wavelet-LLH	gldm	LargeDependenceLowGrayLevelEmphasis	0.043400328
wavelet-LLH	gldm	DependenceVariance	1.508298182
wavelet-LLH	gldm	LargeDependenceHighGrayLevelEmphasis	1767.72103
wavelet-LLH	gldm	SmallDependenceLowGrayLevelEmphasis	0.006876061
wavelet-LLH	gldm	LowGrayLevelEmphasis	0.010312267
wavelet-LLH	glcm	JointAverage	15.05257525
wavelet-LLH	glcm	SumAverage	30.10515051
wavelet-LLH	glcm	JointEntropy	7.233265661
wavelet-LLH	glcm	ClusterShade	17.77334161
wavelet-LLH	glcm	MaximumProbability	0.020036061
wavelet-LLH	glcm	Idmn	0.954689965
wavelet-LLH	glcm	JointEnergy	0.007741768
wavelet-LLH	glcm	Contrast	35.3282803
wavelet-LLH	glcm	DifferenceEntropy	3.462642039
wavelet-LLH	glcm	InverseVariance	0.204752716
wavelet-LLH	glcm	DifferenceVariance	11.90931118
wavelet-LLH	glcm	Idn	0.859247455
wavelet-LLH	glcm	Idm	0.198117406
wavelet-LLH	glcm	Correlation	0.118209802
wavelet-LLH	glcm	Autocorrelation	229.0426738
wavelet-LLH	glcm	SumEntropy	4.548039423

wavelet-LLH	glcm	SumSquares	20.11156092
wavelet-LLH	glcm	ClusterProminence	5515.02019
wavelet-LLH	glcm	Imc2	0.922150981
wavelet-LLH	glcm	Imc1	-0.235500544
wavelet-LLH	glcm	DifferenceAverage	4.66096159
wavelet-LLH	glcm	Id	0.290646187
wavelet-LLH	glcm	ClusterTendency	45.11796339
wavelet-LLH	firstorder	InterquartileRange	165.6934133
wavelet-LLH	firstorder	Skewness	-0.047575109
wavelet-LLH	firstorder	Uniformity	0.065298679
wavelet-LLH	firstorder	Median	-9.237148753
wavelet-LLH	firstorder	Energy	2965349.164
wavelet-LLH	firstorder	RobustMeanAbsoluteDeviation	65.32074725
wavelet-LLH	firstorder	MeanAbsoluteDeviation	90.89498728
wavelet-LLH	firstorder	TotalEnergy	80064427.44
wavelet-LLH	firstorder	Maximum	259.066441
wavelet-LLH	firstorder	RootMeanSquared	112.8132104
wavelet-LLH	firstorder	90Percentile	141.1336876
wavelet-LLH	firstorder	Minimum	-372.7427932
wavelet-LLH	firstorder	Entropy	4.115797982
wavelet-LLH	firstorder	Range	631.8092342
wavelet-LLH	firstorder	Variance	12608.62293
wavelet-LLH	firstorder	10Percentile	-160.3988832
wavelet-LLH	firstorder	Kurtosis	2.671194916
wavelet-LLH	firstorder	Mean	-10.87186809
wavelet-LLH	glrlm	ShortRunLowGrayLevelEmphasis	0.010349693
wavelet-LLH	glrlm	GrayLevelVariance	20.4435117
wavelet-LLH	glrlm	LowGrayLevelRunEmphasis	0.010574566
wavelet-LLH	glrlm	GrayLevelNonUniformityNormalized	0.064807325
wavelet-LLH	glrlm	RunVariance	0.059773416
wavelet-LLH	glrlm	GrayLevelNonUniformity	14.31139635
wavelet-LLH	glrlm	LongRunEmphasis	1.17713381
wavelet-LLH	glrlm	ShortRunHighGrayLevelEmphasis	237.0619185
wavelet-LLH	glrlm	RunLengthNonUniformity	199.7103821
wavelet-LLH	glrlm	ShortRunEmphasis	0.96018954
wavelet-LLH	glrlm	LongRunHighGrayLevelEmphasis	290.6455222
wavelet-LLH	glrlm	RunPercentage	0.94783757
wavelet-LLH	glrlm	LongRunLowGrayLevelEmphasis	0.011578118
wavelet-LLH	glrlm	RunEntropy	4.364860675
wavelet-LLH	glrlm	HighGrayLevelRunEmphasis	246.9885605
wavelet-LLH	glrlm	RunLengthNonUniformityNormalized	0.902302565
wavelet-LLH	glszm	GrayLevelVariance	24.91764987
wavelet-LLH	glszm	ZoneVariance	4.93104273
wavelet-LLH	glszm	GrayLevelNonUniformityNormalized	0.058832908
wavelet-LLH	glszm	SizeZoneNonUniformityNormalized	0.432397959
wavelet-LLH	glszm	SizeZoneNonUniformity	48.42857143
wavelet-LLH	glszm	GrayLevelNonUniformity	6.589285714
wavelet-LLH	glszm	LargeAreaEmphasis	9.258928571
wavelet-LLH	glszm	SmallAreaHighGrayLevelEmphasis	174.4857097
wavelet-LLH	glszm	ZonePercentage	0.480686695
wavelet-LLH	glszm	LargeAreaLowGrayLevelEmphasis	0.059317803
wavelet-LLH	glszm	LargeAreaHighGrayLevelEmphasis	2227.919643
wavelet-LLH	glszm	HighGrayLevelZoneEmphasis	251.2589286

wavelet-LLH	glszm	SmallAreaEmphasis	0.682242665
wavelet-LLH	glszm	LowGrayLevelZoneEmphasis	0.015273597
wavelet-LLH	glszm	ZoneEntropy	5.357179561
wavelet-LLH	glszm	SmallAreaLowGrayLevelEmphasis	0.013311151
wavelet-LLH	ngtdm	Coarseness	0.024050963
wavelet-LLH	ngtdm	Complexity	689.7527841
wavelet-LLH	ngtdm	Strength	3.919178152
wavelet-LLH	ngtdm	Contrast	0.271744134
wavelet-LLH	ngtdm	Busyness	0.141999321
wavelet-LLL	gldm	GrayLevelVariance	353.0049918
wavelet-LLL	gldm	HighGrayLevelEmphasis	3431.111588
wavelet-LLL	gldm	DependenceEntropy	6.610742503
wavelet-LLL	gldm	DependenceNonUniformity	96.70815451
wavelet-LLL	gldm	GrayLevelNonUniformity	4.407725322
wavelet-LLL	gldm	SmallDependenceEmphasis	0.649484979
wavelet-LLL	gldm	SmallDependenceHighGrayLevelEmphasis	1974.432433
wavelet-LLL	gldm	DependenceNonUniformityNormalized	0.415056457
wavelet-LLL	gldm	LargeDependenceEmphasis	3.497854077
wavelet-LLL	gldm	LargeDependenceLowGrayLevelEmphasis	0.005671607
wavelet-LLL	gldm	DependenceVariance	0.79585183
wavelet-LLL	gldm	LargeDependenceHighGrayLevelEmphasis	14144.18455
wavelet-LLL	gldm	SmallDependenceLowGrayLevelEmphasis	0.004846153
wavelet-LLL	gldm	LowGrayLevelEmphasis	0.004954554
wavelet-LLL	glcm	JointAverage	56.5071532
wavelet-LLL	glcm	SumAverage	113.0143064
wavelet-LLL	glcm	JointEntropy	8.148113551
wavelet-LLL	glcm	ClusterShade	-17394.06872
wavelet-LLL	glcm	MaximumProbability	0.010359935
wavelet-LLL	glcm	Idmn	0.982225742
wavelet-LLL	glcm	JointEnergy	0.003739531
wavelet-LLL	glcm	Contrast	174.1654128
wavelet-LLL	glcm	DifferenceEntropy	4.482779295
wavelet-LLL	glcm	InverseVariance	0.108264519
wavelet-LLL	glcm	DifferenceVariance	61.74791241
wavelet-LLL	glcm	Idn	0.909248797
wavelet-LLL	glcm	Idm	0.103631713
wavelet-LLL	glcm	Correlation	0.710321237
wavelet-LLL	glcm	Autocorrelation	3407.427441
wavelet-LLL	glcm	SumEntropy	6.253271004
wavelet-LLL	glcm	SumSquares	301.1201263
wavelet-LLL	glcm	ClusterProminence	2688870.157
wavelet-LLL	glcm	Imc2	0.999521171
wavelet-LLL	glcm	Imc1	-0.599777478
wavelet-LLL	glcm	DifferenceAverage	10.19195229
wavelet-LLL	glcm	Id	0.18326081
wavelet-LLL	glcm	ClusterTendency	1030.315092
wavelet-LLL	firstorder	InterquartileRange	692.2295003
wavelet-LLL	firstorder	Skewness	-0.485296895
wavelet-LLL	firstorder	Uniformity	0.018917276
wavelet-LLL	firstorder	Median	-676.074207
wavelet-LLL	firstorder	Energy	182603118.9
wavelet-LLL	firstorder	RobustMeanAbsoluteDeviation	290.1442178
wavelet-LLL	firstorder	MeanAbsoluteDeviation	390.418424

wavelet-LLL	firstorder	TotalEnergy	4930284209
wavelet-LLL	firstorder	Maximum	268.6031789
wavelet-LLL	firstorder	RootMeanSquared	885.2707907
wavelet-LLL	firstorder	90Percentile	-209.8738253
wavelet-LLL	firstorder	Minimum	-2123.193741
wavelet-LLL	firstorder	Entropy	5.919146854
wavelet-LLL	firstorder	Range	2391.79692
wavelet-LLL	firstorder	Variance	219975.0381
wavelet-LLL	firstorder	10Percentile	-1450.394187
wavelet-LLL	firstorder	Kurtosis	2.563241265
wavelet-LLL	firstorder	Mean	-750.8191092
wavelet-LLL	glrlm	ShortRunLowGrayLevelEmphasis	0.005068083
wavelet-LLL	glrlm	GrayLevelVariance	355.6419321
wavelet-LLL	glrlm	LowGrayLevelRunEmphasis	0.005073563
wavelet-LLL	glrlm	GrayLevelNonUniformityNormalized	0.01871053
wavelet-LLL	glrlm	RunVariance	0.027455737
wavelet-LLL	glrlm	GrayLevelNonUniformity	4.251748496
wavelet-LLL	glrlm	LongRunEmphasis	1.079359652
wavelet-LLL	glrlm	ShortRunHighGrayLevelEmphasis	3332.967721
wavelet-LLL	glrlm	RunLengthNonUniformity	216.6285154
wavelet-LLL	glrlm	ShortRunEmphasis	0.981672847
wavelet-LLL	glrlm	LongRunHighGrayLevelEmphasis	3747.368187
wavelet-LLL	glrlm	RunPercentage	0.975239353
wavelet-LLL	glrlm	LongRunLowGrayLevelEmphasis	0.005096855
wavelet-LLL	glrlm	RunEntropy	6.001585328
wavelet-LLL	glrlm	HighGrayLevelRunEmphasis	3410.487806
wavelet-LLL	glrlm	RunLengthNonUniformityNormalized	0.953058463
wavelet-LLL	glszm	GrayLevelVariance	383.7432526
wavelet-LLL	glszm	ZoneVariance	0.70384083
wavelet-LLL	glszm	GrayLevelNonUniformityNormalized	0.01716263
wavelet-LLL	glszm	SizeZoneNonUniformityNormalized	0.621107266
wavelet-LLL	glszm	SizeZoneNonUniformity	105.5882353
wavelet-LLL	glszm	GrayLevelNonUniformity	2.917647059
wavelet-LLL	glszm	LargeAreaEmphasis	2.582352941
wavelet-LLL	glszm	SmallAreaHighGrayLevelEmphasis	2394.403595
wavelet-LLL	glszm	ZonePercentage	0.729613734
wavelet-LLL	glszm	LargeAreaLowGrayLevelEmphasis	0.007129705
wavelet-LLL	glszm	LargeAreaHighGrayLevelEmphasis	9936.029412
wavelet-LLL	glszm	HighGrayLevelZoneEmphasis	3143.082353
wavelet-LLL	glszm	SmallAreaEmphasis	0.815227124
wavelet-LLL	glszm	LowGrayLevelZoneEmphasis	0.006679326
wavelet-LLL	glszm	ZoneEntropy	6.423961269
wavelet-LLL	glszm	SmallAreaLowGrayLevelEmphasis	0.006618993
wavelet-LLL	ngtdm	Coarseness	0.044284016
wavelet-LLL	ngtdm	Complexity	12158.42876
wavelet-LLL	ngtdm	Strength	95.60913887
wavelet-LLL	ngtdm	Contrast	0.810650387
wavelet-LLL	ngtdm	Busyness	0.006396033
wavelet-HHL	gldm	GrayLevelVariance	6.083552838
wavelet-HHL	gldm	HighGrayLevelEmphasis	63.20600858
wavelet-HHL	gldm	DependenceEntropy	5.361414973
wavelet-HHL	gldm	DependenceNonUniformity	27.93562232
wavelet-HHL	gldm	GrayLevelNonUniformity	40.20171674

wavelet-HHL	gldm	SmallDependenceEmphasis	0.218509423
wavelet-HHL	gldm	SmallDependenceHighGrayLevelEmphasis	17.60156262
wavelet-HHL	gldm	DependenceNonUniformityNormalized	0.119895375
wavelet-HHL	gldm	LargeDependenceEmphasis	26.49356223
wavelet-HHL	gldm	LargeDependenceLowGrayLevelEmphasis	0.567650785
wavelet-HHL	gldm	DependenceVariance	7.291863913
wavelet-HHL	gldm	LargeDependenceHighGrayLevelEmphasis	1525.369099
wavelet-HHL	gldm	SmallDependenceLowGrayLevelEmphasis	0.01377297
wavelet-HHL	gldm	LowGrayLevelEmphasis	0.039131624
wavelet-HHL	glcm	JointAverage	7.549418622
wavelet-HHL	glcm	SumAverage	15.09883724
wavelet-HHL	glcm	JointEntropy	5.254610319
wavelet-HHL	glcm	ClusterShade	45.79768818
wavelet-HHL	glcm	MaximumProbability	0.089550508
wavelet-HHL	glcm	Idmn	0.986844901
wavelet-HHL	glcm	JointEnergy	0.039529575
wavelet-HHL	glcm	Contrast	9.756713808
wavelet-HHL	glcm	DifferenceEntropy	2.5391233
wavelet-HHL	glcm	InverseVariance	0.403207475
wavelet-HHL	glcm	DifferenceVariance	5.571589569
wavelet-HHL	glcm	Idn	0.930677676
wavelet-HHL	glcm	Idm	0.413456077
wavelet-HHL	glcm	Correlation	-0.057878086
wavelet-HHL	glcm	Autocorrelation	56.76416073
wavelet-HHL	glcm	SumEntropy	3.223757344
wavelet-HHL	glcm	SumSquares	4.646496664
wavelet-HHL	glcm	ClusterProminence	1521.272456
wavelet-HHL	glcm	Imc2	0.698821729
wavelet-HHL	glcm	Imc1	-0.123849917
wavelet-HHL	glcm	DifferenceAverage	2.025040958
wavelet-HHL	glcm	Id	0.477832437
wavelet-HHL	glcm	ClusterTendency	8.829272847
wavelet-HHL	firstorder	InterquartileRange	48.83578737
wavelet-HHL	firstorder	Skewness	2.464402161
wavelet-HHL	firstorder	Uniformity	0.172539557
wavelet-HHL	firstorder	Median	0.405341738
wavelet-HHL	firstorder	Energy	887398.9836
wavelet-HHL	firstorder	RobustMeanAbsoluteDeviation	22.10309252
wavelet-HHL	firstorder	MeanAbsoluteDeviation	38.35688447
wavelet-HHL	firstorder	TotalEnergy	23959772.56
wavelet-HHL	firstorder	Maximum	443.7514641
wavelet-HHL	firstorder	RootMeanSquared	61.71368834
wavelet-HHL	firstorder	90Percentile	55.10029813
wavelet-HHL	firstorder	Minimum	-169.9581326
wavelet-HHL	firstorder	Entropy	2.911556863
wavelet-HHL	firstorder	Range	613.7095967
wavelet-HHL	firstorder	Variance	3806.247524
wavelet-HHL	firstorder	10Percentile	-56.96230224
wavelet-HHL	firstorder	Kurtosis	21.74710542
wavelet-HHL	firstorder	Mean	1.527024872
wavelet-HHL	glrlm	ShortRunLowGrayLevelEmphasis	0.039131931
wavelet-HHL	glrlm	GrayLevelVariance	6.81793863
wavelet-HHL	glrlm	LowGrayLevelRunEmphasis	0.041376991

wavelet-HHL	glrlm	GrayLevelNonUniformityNormalized	0.158126017
wavelet-HHL	glrlm	RunVariance	0.175981526
wavelet-HHL	glrlm	GrayLevelNonUniformity	32.06211894
wavelet-HHL	glrlm	LongRunEmphasis	1.504317506
wavelet-HHL	glrlm	ShortRunHighGrayLevelEmphasis	58.24410023
wavelet-HHL	glrlm	RunLengthNonUniformity	157.0479026
wavelet-HHL	glrlm	ShortRunEmphasis	0.900150207
wavelet-HHL	glrlm	LongRunHighGrayLevelEmphasis	93.00994358
wavelet-HHL	glrlm	RunPercentage	0.869924067
wavelet-HHL	glrlm	LongRunLowGrayLevelEmphasis	0.054210322
wavelet-HHL	glrlm	RunEntropy	3.54781909
wavelet-HHL	glrlm	HighGrayLevelRunEmphasis	64.03789109
wavelet-HHL	glrlm	RunLengthNonUniformityNormalized	0.772674984
wavelet-HHL	glszm	GrayLevelVariance	19.30049587
wavelet-HHL	glszm	ZoneVariance	120.1441322
wavelet-HHL	glszm	GrayLevelNonUniformityNormalized	0.110743802
wavelet-HHL	glszm	SizeZoneNonUniformityNormalized	0.393057851
wavelet-HHL	glszm	SizeZoneNonUniformity	21.61818182
wavelet-HHL	glszm	GrayLevelNonUniformity	6.090909091
wavelet-HHL	glszm	LargeAreaEmphasis	138.0909091
wavelet-HHL	glszm	SmallAreaHighGrayLevelEmphasis	58.46784274
wavelet-HHL	glszm	ZonePercentage	0.236051502
wavelet-HHL	glszm	LargeAreaLowGrayLevelEmphasis	2.699404734
wavelet-HHL	glszm	LargeAreaHighGrayLevelEmphasis	7895.509091
wavelet-HHL	glszm	HighGrayLevelZoneEmphasis	80.70909091
wavelet-HHL	glszm	SmallAreaEmphasis	0.645823578
wavelet-HHL	glszm	LowGrayLevelZoneEmphasis	0.067188777
wavelet-HHL	glszm	ZoneEntropy	4.53883796
wavelet-HHL	glszm	SmallAreaLowGrayLevelEmphasis	0.043520668
wavelet-HHL	ngtdm	Coarseness	0.027623058
wavelet-HHL	ngtdm	Complexity	188.5252579
wavelet-HHL	ngtdm	Strength	4.230140857
wavelet-HHL	ngtdm	Contrast	0.110717016
wavelet-HHL	ngtdm	Busyness	0.280586194
wavelet-HLH	gldm	GrayLevelVariance	2.450662197
wavelet-HLH	gldm	HighGrayLevelEmphasis	56.94420601
wavelet-HLH	gldm	DependenceEntropy	5.225870316
wavelet-HLH	gldm	DependenceNonUniformity	28.08154506
wavelet-HLH	gldm	GrayLevelNonUniformity	45.61802575
wavelet-HLH	gldm	SmallDependenceEmphasis	0.205189695
wavelet-HLH	gldm	SmallDependenceHighGrayLevelEmphasis	12.43952178
wavelet-HLH	gldm	DependenceNonUniformityNormalized	0.120521653
wavelet-HLH	gldm	LargeDependenceEmphasis	27.32618026
wavelet-HLH	gldm	LargeDependenceLowGrayLevelEmphasis	0.525643535
wavelet-HLH	gldm	DependenceVariance	6.824329054
wavelet-HLH	gldm	LargeDependenceHighGrayLevelEmphasis	1522.248927
wavelet-HLH	gldm	SmallDependenceLowGrayLevelEmphasis	0.010261398
wavelet-HLH	gldm	LowGrayLevelEmphasis	0.025918356
wavelet-HLH	glcm	JointAverage	7.386624899
wavelet-HLH	glcm	SumAverage	14.7732498
wavelet-HLH	glcm	JointEntropy	5.03573777
wavelet-HLH	glcm	ClusterShade	-1.634797198
wavelet-HLH	glcm	MaximumProbability	0.080009276

wavelet-HLH	glcm	Idmn	0.969348255
wavelet-HLH	glcm	JointEnergy	0.041051746
wavelet-HLH	glcm	Contrast	4.932640985
wavelet-HLH	glcm	DifferenceEntropy	2.333068987
wavelet-HLH	glcm	InverseVariance	0.411505108
wavelet-HLH	glcm	DifferenceVariance	2.017471348
wavelet-HLH	glcm	Idn	0.884972123
wavelet-HLH	glcm	Idm	0.426185436
wavelet-HLH	glcm	Correlation	-0.029774594
wavelet-HLH	glcm	Autocorrelation	54.48279611
wavelet-HLH	glcm	SumEntropy	3.064117824
wavelet-HLH	glcm	SumSquares	2.386499996
wavelet-HLH	glcm	ClusterProminence	82.64507791
wavelet-HLH	glcm	Imc2	0.56890528
wavelet-HLH	glcm	Imc1	-0.076602923
wavelet-HLH	glcm	DifferenceAverage	1.695725283
wavelet-HLH	glcm	Id	0.491364662
wavelet-HLH	glcm	ClusterTendency	4.613358998
wavelet-HLH	firstorder	InterquartileRange	46.29584214
wavelet-HLH	firstorder	Skewness	-0.324923876
wavelet-HLH	firstorder	Uniformity	0.195785518
wavelet-HLH	firstorder	Median	-0.795801572
wavelet-HLH	firstorder	Energy	341261.6378
wavelet-HLH	firstorder	RobustMeanAbsoluteDeviation	18.79412971
wavelet-HLH	firstorder	MeanAbsoluteDeviation	28.83481509
wavelet-HLH	firstorder	TotalEnergy	9214064.221
wavelet-HLH	firstorder	Maximum	103.6618906
wavelet-HLH	firstorder	RootMeanSquared	38.27064439
wavelet-HLH	firstorder	90Percentile	39.96745888
wavelet-HLH	firstorder	Minimum	-160.6221348
wavelet-HLH	firstorder	Entropy	2.631964643
wavelet-HLH	firstorder	Range	264.2840254
wavelet-HLH	firstorder	Variance	1460.711318
wavelet-HLH	firstorder	10Percentile	-45.97684742
wavelet-HLH	firstorder	Kurtosis	4.656296414
wavelet-HLH	firstorder	Mean	-1.982650838
wavelet-HLH	glrlm	ShortRunLowGrayLevelEmphasis	0.024990932
wavelet-HLH	glrlm	GrayLevelVariance	2.697476043
wavelet-HLH	glrlm	LowGrayLevelRunEmphasis	0.026992261
wavelet-HLH	glrlm	GrayLevelNonUniformityNormalized	0.184741979
wavelet-HLH	glrlm	RunVariance	0.185702606
wavelet-HLH	glrlm	GrayLevelNonUniformity	37.2059951
wavelet-HLH	glrlm	LongRunEmphasis	1.530167939
wavelet-HLH	glrlm	ShortRunHighGrayLevelEmphasis	51.22525329
wavelet-HLH	glrlm	RunLengthNonUniformity	153.9116993
wavelet-HLH	glrlm	ShortRunEmphasis	0.895481274
wavelet-HLH	glrlm	LongRunHighGrayLevelEmphasis	86.65131114
wavelet-HLH	glrlm	RunPercentage	0.864311654
wavelet-HLH	glrlm	LongRunLowGrayLevelEmphasis	0.037151785
wavelet-HLH	glrlm	RunEntropy	3.294727796
wavelet-HLH	glrlm	HighGrayLevelRunEmphasis	57.09952167
wavelet-HLH	glrlm	RunLengthNonUniformityNormalized	0.762625948
wavelet-HLH	glszm	GrayLevelVariance	7.982222222

wavelet-HLH	glszm	ZoneVariance	166.0128395
wavelet-HLH	glszm	GrayLevelNonUniformityNormalized	0.130864198
wavelet-HLH	glszm	SizeZoneNonUniformityNormalized	0.53382716
wavelet-HLH	glszm	SizeZoneNonUniformity	24.02222222
wavelet-HLH	glszm	GrayLevelNonUniformity	5.888888889
wavelet-HLH	glszm	LargeAreaEmphasis	192.8222222
wavelet-HLH	glszm	SmallAreaHighGrayLevelEmphasis	46.99523633
wavelet-HLH	glszm	ZonePercentage	0.193133047
wavelet-HLH	glszm	LargeAreaLowGrayLevelEmphasis	3.56017897
wavelet-HLH	glszm	LargeAreaHighGrayLevelEmphasis	11094.13333
wavelet-HLH	glszm	HighGrayLevelZoneEmphasis	59.82222222
wavelet-HLH	glszm	SmallAreaEmphasis	0.75129968
wavelet-HLH	glszm	LowGrayLevelZoneEmphasis	0.053088844
wavelet-HLH	glszm	ZoneEntropy	3.899658863
wavelet-HLH	glszm	SmallAreaLowGrayLevelEmphasis	0.04673681
wavelet-HLH	ngtdm	Coarseness	0.024734464
wavelet-HLH	ngtdm	Complexity	66.32940948
wavelet-HLH	ngtdm	Strength	1.069817615
wavelet-HLH	ngtdm	Contrast	0.057233413
wavelet-HLH	ngtdm	Busyness	0.426246806

Image type	Feature Class	Feature Name	3: RTSTRUCT: C99-DNU_segment_GTVp
general	info	BoundingBox	(0, 1, 1, 6, 6, 8)
general	info	EnabledImageTypes	{u'Original': {}, u'Wavelet': {}}
general	info	GeneralSettings	{'distances': [1], 'additionalInfo': True, 'force2D': False, 'interpolator': 'sitkBSpline', 'resampledPixelSpacing': [3.0], 'label': 1, 'normalizeScale': 1, 'normalize': False, 'force2Ddimension': 0, 'removeOutliers': None, 'minimumROISize': None, u'binWidth': 25.0, 'minimumROIDimensions': 1, u'correctMask': True, 'preCrop': False, u'symmetricalGLCM': True, 'resegmentRange': None, 'padDistance': 5}
general	info	ImageHash	0b6693ea7244013f6d10c816f1260cc7f9a71009
general	info	ImageSpacing	(0.9765625000000002, 0.9765625000000002, 2.0)
general	info	MaskHash	8d143361cfde157532bbb89b9f2636ced632c83f
general	info	NumpyVersion	1.13.1
general	info	PyWaveletVersion	0.5.2
general	info	SimpleITKVersion	1.1.0
general	info	Version	1.3.0.post102+g1b0eb74
general	info	VolumeNum	1
general	info	VoxelNum	122
original	shape	Maximum3DDiameter	25.98076211
original	shape	Maximum2DDiameterSlice	15
original	shape	Sphericity	0.816037497
original	shape	MinorAxis	15.25586653
original	shape	Elongation	0.576940621
original	shape	SurfaceVolumeRatio	0.398290069
original	shape	Volume	3294
original	shape	MajorAxis	26.44269788
original	shape	SurfaceArea	1311.967488
original	shape	Flatness	0.473869799
original	shape	LeastAxis	12.53039592
original	shape	Maximum2DDiameterColumn	25.8069758
original	shape	Maximum2DDiameterRow	22.84731932
original	gldm	GrayLevelVariance	131.6205321
original	gldm	HighGrayLevelEmphasis	1034.57377
original	gldm	DependenceEntropy	5.770369408
original	gldm	DependenceNonUniformity	32.39344262
original	gldm	GrayLevelNonUniformity	7.163934426
original	gldm	SmallDependenceEmphasis	0.508254172
original	gldm	SmallDependenceHighGrayLevelEmphasis	362.2087877
original	gldm	DependenceNonUniformityNormalized	0.265520021
original	gldm	LargeDependenceEmphasis	11.73770492
original	gldm	LargeDependenceLowGrayLevelEmphasis	0.022213398
original	gldm	DependenceVariance	4.943563558
original	gldm	LargeDependenceHighGrayLevelEmphasis	17694.29508
original	gldm	SmallDependenceLowGrayLevelEmphasis	0.014217629

original	gldm	LowGrayLevelEmphasis	0.014711659
original	glcm	JointAverage	32.02772767
original	glcm	SumAverage	64.05545534
original	glcm	JointEntropy	6.550440201
original	glcm	ClusterShade	-3421.567126
original	glcm	MaximumProbability	0.057366541
original	glcm	Idmn	0.911623965
original	glcm	JointEnergy	0.014094141
original	glcm	Contrast	215.1083244
original	glcm	DifferenceEntropy	4.417170919
original	glcm	InverseVariance	0.133917948
original	glcm	DifferenceVariance	102.4925639
original	glcm	Idn	0.826795607
original	glcm	Idm	0.179767185
original	glcm	Correlation	0.030523928
original	glcm	Autocorrelation	1029.636804
original	glcm	SumEntropy	4.924946213
original	glcm	SumSquares	111.1989118
original	glcm	ClusterProminence	181573.1328
original	glcm	Imc2	0.991166394
original	glcm	Imc1	-0.474928984
original	glcm	DifferenceAverage	10.50835503
original	glcm	Id	0.248582826
original	glcm	ClusterTendency	229.6873228
original	firstorder	InterquartileRange	373.25
original	firstorder	Skewness	-1.007583874
original	firstorder	Uniformity	0.058720774
original	firstorder	Median	-111.5
original	firstorder	Energy	15555011
original	firstorder	RobustMeanAbsoluteDeviation	169.59375
original	firstorder	MeanAbsoluteDeviation	236.2639076
original	firstorder	TotalEnergy	419985297
original	firstorder	Maximum	97
original	firstorder	RootMeanSquared	357.0715477
original	firstorder	90Percentile	49.9
original	firstorder	Minimum	-937
original	firstorder	Entropy	4.584700872
original	firstorder	Range	1034
original	firstorder	Variance	82864.86946
original	firstorder	10Percentile	-715.4
original	firstorder	Kurtosis	2.77989594
original	firstorder	Mean	-211.2704918
original	glrlm	ShortRunLowGrayLevelEmphasis	0.015600355
original	glrlm	GrayLevelVariance	134.0399161
original	glrlm	LowGrayLevelRunEmphasis	0.015636766
original	glrlm	GrayLevelNonUniformityNormalized	0.052121093
original	glrlm	RunVariance	0.078164754
original	glrlm	GrayLevelNonUniformity	5.97131766
original	glrlm	LongRunEmphasis	1.216055774
original	glrlm	ShortRunHighGrayLevelEmphasis	936.3586994
original	glrlm	RunLengthNonUniformity	101.711684
original	glrlm	ShortRunEmphasis	0.954559321
original	glrlm	LongRunHighGrayLevelEmphasis	1329.085165

original	glrlm	RunPercentage	0.938209332
original	glrlm	LongRunLowGrayLevelEmphasis	0.015803745
original	glrlm	RunEntropy	4.855701907
original	glrlm	HighGrayLevelRunEmphasis	1003.878932
original	glrlm	RunLengthNonUniformityNormalized	0.888008411
original	glszm	GrayLevelVariance	141.5420408
original	glszm	ZoneVariance	5.733877551
original	glszm	GrayLevelNonUniformityNormalized	0.035918367
original	glszm	SizeZoneNonUniformityNormalized	0.573877551
original	glszm	SizeZoneNonUniformity	40.17142857
original	glszm	GrayLevelNonUniformity	2.514285714
original	glszm	LargeAreaEmphasis	8.771428571
original	glszm	SmallAreaHighGrayLevelEmphasis	504.2821103
original	glszm	ZonePercentage	0.573770492
original	glszm	LargeAreaLowGrayLevelEmphasis	0.030501861
original	glszm	LargeAreaHighGrayLevelEmphasis	12741.91429
original	glszm	HighGrayLevelZoneEmphasis	758
original	glszm	SmallAreaEmphasis	0.784243717
original	glszm	LowGrayLevelZoneEmphasis	0.024952612
original	glszm	ZoneEntropy	5.339508552
original	glszm	SmallAreaLowGrayLevelEmphasis	0.024660241
original	ngtdm	Coarseness	0.019867356
original	ngtdm	Complexity	5065.315141
original	ngtdm	Strength	21.25275265
original	ngtdm	Contrast	2.191213235
original	ngtdm	Busyness	0.042665266
wavelet-HHH	gldm	GrayLevelVariance	21.00080623
wavelet-HHH	gldm	HighGrayLevelEmphasis	178.6885246
wavelet-HHH	gldm	DependenceEntropy	5.322566666
wavelet-HHH	gldm	DependenceNonUniformity	40.62295082
wavelet-HHH	gldm	GrayLevelNonUniformity	8.459016393
wavelet-HHH	gldm	SmallDependenceEmphasis	0.572318919
wavelet-HHH	gldm	SmallDependenceHighGrayLevelEmphasis	102.1882073
wavelet-HHH	gldm	DependenceNonUniformityNormalized	0.332975007
wavelet-HHH	gldm	LargeDependenceEmphasis	5.180327869
wavelet-HHH	gldm	LargeDependenceLowGrayLevelEmphasis	0.049655413
wavelet-HHH	gldm	DependenceVariance	1.438323031
wavelet-HHH	gldm	LargeDependenceHighGrayLevelEmphasis	963.6065574
wavelet-HHH	gldm	SmallDependenceLowGrayLevelEmphasis	0.018352685
wavelet-HHH	gldm	LowGrayLevelEmphasis	0.021535074
wavelet-HHH	glcm	JointAverage	12.49654737
wavelet-HHH	glcm	SumAverage	24.99309474
wavelet-HHH	glcm	JointEntropy	6.592575455
wavelet-HHH	glcm	ClusterShade	-3.349021764
wavelet-HHH	glcm	MaximumProbability	0.027278571
wavelet-HHH	glcm	Idmn	0.940659955
wavelet-HHH	glcm	JointEnergy	0.011727194
wavelet-HHH	glcm	Contrast	40.50781807
wavelet-HHH	glcm	DifferenceEntropy	3.592952241
wavelet-HHH	glcm	InverseVariance	0.166559033
wavelet-HHH	glcm	DifferenceVariance	13.83060063
wavelet-HHH	glcm	Idn	0.836663612
wavelet-HHH	glcm	Idm	0.162858363

wavelet-HHH	glcm	Correlation	-0.037047923
wavelet-HHH	glcm	Autocorrelation	155.3974818
wavelet-HHH	glcm	SumEntropy	4.304982298
wavelet-HHH	glcm	SumSquares	19.47750787
wavelet-HHH	glcm	ClusterProminence	4089.2029
wavelet-HHH	glcm	Imc2	0.967913459
wavelet-HHH	glcm	Imc1	-0.347992034
wavelet-HHH	glcm	DifferenceAverage	5.129320896
wavelet-HHH	glcm	Id	0.260511509
wavelet-HHH	glcm	ClusterTendency	37.40221341
wavelet-HHH	firstorder	InterquartileRange	166.9563597
wavelet-HHH	firstorder	Skewness	-0.005293269
wavelet-HHH	firstorder	Uniformity	0.0693362
wavelet-HHH	firstorder	Median	-3.870859641
wavelet-HHH	firstorder	Energy	1562554.321
wavelet-HHH	firstorder	RobustMeanAbsoluteDeviation	62.51298402
wavelet-HHH	firstorder	MeanAbsoluteDeviation	90.38906366
wavelet-HHH	firstorder	TotalEnergy	42188966.66
wavelet-HHH	firstorder	Maximum	283.8686598
wavelet-HHH	firstorder	RootMeanSquared	113.1716497
wavelet-HHH	firstorder	90Percentile	152.0743549
wavelet-HHH	firstorder	Minimum	-283.045641
wavelet-HHH	firstorder	Entropy	4.068025378
wavelet-HHH	firstorder	Range	566.9143009
wavelet-HHH	firstorder	Variance	12806.41029
wavelet-HHH	firstorder	10Percentile	-140.8298243
wavelet-HHH	firstorder	Kurtosis	2.799595177
wavelet-HHH	firstorder	Mean	1.188279291
wavelet-HHH	glrlm	ShortRunLowGrayLevelEmphasis	0.021894597
wavelet-HHH	glrlm	GrayLevelVariance	21.38391606
wavelet-HHH	glrlm	LowGrayLevelRunEmphasis	0.022081454
wavelet-HHH	glrlm	GrayLevelNonUniformityNormalized	0.068415479
wavelet-HHH	glrlm	RunVariance	0.041211306
wavelet-HHH	glrlm	GrayLevelNonUniformity	8.048880443
wavelet-HHH	glrlm	LongRunEmphasis	1.118492326
wavelet-HHH	glrlm	ShortRunHighGrayLevelEmphasis	173.6627513
wavelet-HHH	glrlm	RunLengthNonUniformity	109.7875232
wavelet-HHH	glrlm	ShortRunEmphasis	0.973363058
wavelet-HHH	glrlm	LongRunHighGrayLevelEmphasis	200.0630969
wavelet-HHH	glrlm	RunPercentage	0.96406053
wavelet-HHH	glrlm	LongRunLowGrayLevelEmphasis	0.022918742
wavelet-HHH	glrlm	RunEntropy	4.220386102
wavelet-HHH	glrlm	HighGrayLevelRunEmphasis	178.549784
wavelet-HHH	glrlm	RunLengthNonUniformityNormalized	0.932783889
wavelet-HHH	glszm	GrayLevelVariance	26.28617209
wavelet-HHH	glszm	ZoneVariance	1.513859958
wavelet-HHH	glszm	GrayLevelNonUniformityNormalized	0.061688832
wavelet-HHH	glszm	SizeZoneNonUniformityNormalized	0.579234097
wavelet-HHH	glszm	SizeZoneNonUniformity	45.75949367
wavelet-HHH	glszm	GrayLevelNonUniformity	4.873417722
wavelet-HHH	glszm	LargeAreaEmphasis	3.898734177
wavelet-HHH	glszm	SmallAreaHighGrayLevelEmphasis	141.0986197
wavelet-HHH	glszm	ZonePercentage	0.647540984

wavelet-HHH	glszm	LargeAreaLowGrayLevelEmphasis	0.050110347
wavelet-HHH	glszm	LargeAreaHighGrayLevelEmphasis	694.278481
wavelet-HHH	glszm	HighGrayLevelZoneEmphasis	179.5443038
wavelet-HHH	glszm	SmallAreaEmphasis	0.787833821
wavelet-HHH	glszm	LowGrayLevelZoneEmphasis	0.029214771
wavelet-HHH	glszm	ZoneEntropy	4.934933084
wavelet-HHH	glszm	SmallAreaLowGrayLevelEmphasis	0.027588798
wavelet-HHH	ngtdm	Coarseness	0.041086988
wavelet-HHH	ngtdm	Complexity	717.7074604
wavelet-HHH	ngtdm	Strength	6.140175739
wavelet-HHH	ngtdm	Contrast	0.351878688
wavelet-HHH	ngtdm	Busyness	0.10095573
wavelet-HLL	gldm	GrayLevelVariance	53.19356356
wavelet-HLL	gldm	HighGrayLevelEmphasis	345.8278689
wavelet-HLL	gldm	DependenceEntropy	5.384487674
wavelet-HLL	gldm	DependenceNonUniformity	51.18032787
wavelet-HLL	gldm	GrayLevelNonUniformity	5.721311475
wavelet-HLL	gldm	SmallDependenceEmphasis	0.65253643
wavelet-HLL	gldm	SmallDependenceHighGrayLevelEmphasis	243.2159927
wavelet-HLL	gldm	DependenceNonUniformityNormalized	0.419510884
wavelet-HLL	gldm	LargeDependenceEmphasis	3.868852459
wavelet-HLL	gldm	LargeDependenceLowGrayLevelEmphasis	0.040152372
wavelet-HLL	gldm	DependenceVariance	1.072829884
wavelet-HLL	gldm	LargeDependenceHighGrayLevelEmphasis	1096.97541
wavelet-HLL	gldm	SmallDependenceLowGrayLevelEmphasis	0.012214712
wavelet-HLL	gldm	LowGrayLevelEmphasis	0.01519949
wavelet-HLL	glcm	JointAverage	16.48098034
wavelet-HLL	glcm	SumAverage	32.96196068
wavelet-HLL	glcm	JointEntropy	6.847634956
wavelet-HLL	glcm	ClusterShade	396.4426849
wavelet-HLL	glcm	MaximumProbability	0.024607248
wavelet-HLL	glcm	Idmn	0.958164961
wavelet-HLL	glcm	JointEnergy	0.009543697
wavelet-HLL	glcm	Contrast	73.97662978
wavelet-HLL	glcm	DifferenceEntropy	3.874931185
wavelet-HLL	glcm	InverseVariance	0.154003687
wavelet-HLL	glcm	DifferenceVariance	28.77572474
wavelet-HLL	glcm	Idn	0.865471738
wavelet-HLL	glcm	Idm	0.137070232
wavelet-HLL	glcm	Correlation	0.114385799
wavelet-HLL	glcm	Autocorrelation	277.588316
wavelet-HLL	glcm	SumEntropy	4.7283888
wavelet-HLL	glcm	SumSquares	42.85610547
wavelet-HLL	glcm	ClusterProminence	37903.65168
wavelet-HLL	glcm	Imc2	0.992965618
wavelet-HLL	glcm	Imc1	-0.477724222
wavelet-HLL	glcm	DifferenceAverage	6.638389138
wavelet-HLL	glcm	Id	0.229005191
wavelet-HLL	glcm	ClusterTendency	97.44779209
wavelet-HLL	firstorder	InterquartileRange	208.6940393
wavelet-HLL	firstorder	Skewness	0.542629201
wavelet-HLL	firstorder	Uniformity	0.046895996
wavelet-HLL	firstorder	Median	-24.02036667

wavelet-HLL	firstorder	Energy	4093737.837
wavelet-HLL	firstorder	RobustMeanAbsoluteDeviation	89.78788296
wavelet-HLL	firstorder	MeanAbsoluteDeviation	140.2604994
wavelet-HLL	firstorder	TotalEnergy	110530921.6
wavelet-HLL	firstorder	Maximum	548.2743151
wavelet-HLL	firstorder	RootMeanSquared	183.1808619
wavelet-HLL	firstorder	90Percentile	198.3987175
wavelet-HLL	firstorder	Minimum	-405.1456597
wavelet-HLL	firstorder	Entropy	4.617465266
wavelet-HLL	firstorder	Range	953.4199748
wavelet-HLL	firstorder	Variance	33441.8695
wavelet-HLL	firstorder	10Percentile	-229.4688611
wavelet-HLL	firstorder	Kurtosis	3.563916725
wavelet-HLL	firstorder	Mean	-10.64700297
wavelet-HLL	glrlm	ShortRunLowGrayLevelEmphasis	0.015179902
wavelet-HLL	glrlm	GrayLevelVariance	53.60636927
wavelet-HLL	glrlm	LowGrayLevelRunEmphasis	0.015361943
wavelet-HLL	glrlm	GrayLevelNonUniformityNormalized	0.046325114
wavelet-HLL	glrlm	RunVariance	0.027135227
wavelet-HLL	glrlm	GrayLevelNonUniformity	5.505813816
wavelet-HLL	glrlm	LongRunEmphasis	1.081713848
wavelet-HLL	glrlm	ShortRunHighGrayLevelEmphasis	342.3793139
wavelet-HLL	glrlm	RunLengthNonUniformity	112.902912
wavelet-HLL	glrlm	ShortRunEmphasis	0.980308349
wavelet-HLL	glrlm	LongRunHighGrayLevelEmphasis	370.084495
wavelet-HLL	glrlm	RunPercentage	0.974148802
wavelet-HLL	glrlm	LongRunLowGrayLevelEmphasis	0.016101617
wavelet-HLL	glrlm	RunEntropy	4.71464113
wavelet-HLL	glrlm	HighGrayLevelRunEmphasis	347.7694512
wavelet-HLL	glrlm	RunLengthNonUniformityNormalized	0.949536827
wavelet-HLL	glszm	GrayLevelVariance	57.08271948
wavelet-HLL	glszm	ZoneVariance	0.752083082
wavelet-HLL	glszm	GrayLevelNonUniformityNormalized	0.043835286
wavelet-HLL	glszm	SizeZoneNonUniformityNormalized	0.615263857
wavelet-HLL	glszm	SizeZoneNonUniformity	55.98901099
wavelet-HLL	glszm	GrayLevelNonUniformity	3.989010989
wavelet-HLL	glszm	LargeAreaEmphasis	2.549450549
wavelet-HLL	glszm	SmallAreaHighGrayLevelEmphasis	305.964591
wavelet-HLL	glszm	ZonePercentage	0.745901639
wavelet-HLL	glszm	LargeAreaLowGrayLevelEmphasis	0.029787033
wavelet-HLL	glszm	LargeAreaHighGrayLevelEmphasis	788.1868132
wavelet-HLL	glszm	HighGrayLevelZoneEmphasis	365.0659341
wavelet-HLL	glszm	SmallAreaEmphasis	0.811527015
wavelet-HLL	glszm	LowGrayLevelZoneEmphasis	0.017430331
wavelet-HLL	glszm	ZoneEntropy	5.209848113
wavelet-HLL	glszm	SmallAreaLowGrayLevelEmphasis	0.015926534
wavelet-HLL	ngtdm	Coarseness	0.047494443
wavelet-HLL	ngtdm	Complexity	1774.619654
wavelet-HLL	ngtdm	Strength	15.28887755
wavelet-HLL	ngtdm	Contrast	0.600220232
wavelet-HLL	ngtdm	Busyness	0.052978624
wavelet-LHL	gldm	GrayLevelVariance	251.2790245
wavelet-LHL	gldm	HighGrayLevelEmphasis	1330.02459

wavelet-LHL	gldm	DependenceEntropy	5.903817563
wavelet-LHL	gldm	DependenceNonUniformity	62.70491803
wavelet-LHL	gldm	GrayLevelNonUniformity	3.278688525
wavelet-LHL	gldm	SmallDependenceEmphasis	0.73712204
wavelet-LHL	gldm	SmallDependenceHighGrayLevelEmphasis	1066.488379
wavelet-LHL	gldm	DependenceNonUniformityNormalized	0.513974738
wavelet-LHL	gldm	LargeDependenceEmphasis	2.508196721
wavelet-LHL	gldm	LargeDependenceLowGrayLevelEmphasis	0.014953349
wavelet-LHL	gldm	DependenceVariance	0.474066111
wavelet-LHL	gldm	LargeDependenceHighGrayLevelEmphasis	2805.270492
wavelet-LHL	gldm	SmallDependenceLowGrayLevelEmphasis	0.010476609
wavelet-LHL	gldm	LowGrayLevelEmphasis	0.011241461
wavelet-LHL	glcm	JointAverage	32.31906077
wavelet-LHL	glcm	SumAverage	64.63812153
wavelet-LHL	glcm	JointEntropy	7.076562646
wavelet-LHL	glcm	ClusterShade	3341.006832
wavelet-LHL	glcm	MaximumProbability	0.014379361
wavelet-LHL	glcm	Idmn	0.918954916
wavelet-LHL	glcm	JointEnergy	0.007698335
wavelet-LHL	glcm	Contrast	497.1136726
wavelet-LHL	glcm	DifferenceEntropy	4.794780438
wavelet-LHL	glcm	InverseVariance	0.082593389
wavelet-LHL	glcm	DifferenceVariance	189.9233675
wavelet-LHL	glcm	Idn	0.825099686
wavelet-LHL	glcm	Idm	0.078647195
wavelet-LHL	glcm	Correlation	0.020206857
wavelet-LHL	glcm	Autocorrelation	1048.963922
wavelet-LHL	glcm	SumEntropy	5.402495865
wavelet-LHL	glcm	SumSquares	252.9716422
wavelet-LHL	glcm	ClusterProminence	726720.3846
wavelet-LHL	glcm	Imc2	0.999673718
wavelet-LHL	glcm	Imc1	-0.685144824
wavelet-LHL	glcm	DifferenceAverage	16.65662848
wavelet-LHL	glcm	Id	0.146434741
wavelet-LHL	glcm	ClusterTendency	514.7728962
wavelet-LHL	firstorder	InterquartileRange	629.1368381
wavelet-LHL	firstorder	Skewness	0.164679388
wavelet-LHL	firstorder	Uniformity	0.026874496
wavelet-LHL	firstorder	Median	-124.9533639
wavelet-LHL	firstorder	Energy	19724455.54
wavelet-LHL	firstorder	RobustMeanAbsoluteDeviation	252.5858775
wavelet-LHL	firstorder	MeanAbsoluteDeviation	339.2993309
wavelet-LHL	firstorder	TotalEnergy	532560299.7
wavelet-LHL	firstorder	Maximum	802.6842682
wavelet-LHL	firstorder	RootMeanSquared	402.0893745
wavelet-LHL	firstorder	90Percentile	511.4188854
wavelet-LHL	firstorder	Minimum	-852.8906077
wavelet-LHL	firstorder	Entropy	5.480180887
wavelet-LHL	firstorder	Range	1655.574876
wavelet-LHL	firstorder	Variance	157182.4944
wavelet-LHL	firstorder	10Percentile	-563.9586072
wavelet-LHL	firstorder	Kurtosis	2.167937284
wavelet-LHL	firstorder	Mean	-67.03260958

wavelet-LHL	glrlm	ShortRunLowGrayLevelEmphasis	0.011352607
wavelet-LHL	glrlm	GrayLevelVariance	252.8383314
wavelet-LHL	glrlm	LowGrayLevelRunEmphasis	0.011385851
wavelet-LHL	glrlm	GrayLevelNonUniformityNormalized	0.026607124
wavelet-LHL	glrlm	RunVariance	0.016381381
wavelet-LHL	glrlm	GrayLevelNonUniformity	3.193037147
wavelet-LHL	glrlm	LongRunEmphasis	1.050362636
wavelet-LHL	glrlm	ShortRunHighGrayLevelEmphasis	1323.197129
wavelet-LHL	glrlm	RunLengthNonUniformity	116.0961611
wavelet-LHL	glrlm	ShortRunEmphasis	0.987409341
wavelet-LHL	glrlm	LongRunHighGrayLevelEmphasis	1385.672791
wavelet-LHL	glrlm	RunPercentage	0.983606557
wavelet-LHL	glrlm	LongRunLowGrayLevelEmphasis	0.011518829
wavelet-LHL	glrlm	RunEntropy	5.531849163
wavelet-LHL	glrlm	HighGrayLevelRunEmphasis	1335.692262
wavelet-LHL	glrlm	RunLengthNonUniformityNormalized	0.967237239
wavelet-LHL	glszm	GrayLevelVariance	271.4777176
wavelet-LHL	glszm	ZoneVariance	0.409412745
wavelet-LHL	glszm	GrayLevelNonUniformityNormalized	0.024156601
wavelet-LHL	glszm	SizeZoneNonUniformityNormalized	0.701374427
wavelet-LHL	glszm	SizeZoneNonUniformity	68.73469388
wavelet-LHL	glszm	GrayLevelNonUniformity	2.367346939
wavelet-LHL	glszm	LargeAreaEmphasis	1.959183673
wavelet-LHL	glszm	SmallAreaHighGrayLevelEmphasis	1271.311567
wavelet-LHL	glszm	ZonePercentage	0.803278689
wavelet-LHL	glszm	LargeAreaLowGrayLevelEmphasis	0.015628721
wavelet-LHL	glszm	LargeAreaHighGrayLevelEmphasis	2402.622449
wavelet-LHL	glszm	HighGrayLevelZoneEmphasis	1406.755102
wavelet-LHL	glszm	SmallAreaEmphasis	0.86300737
wavelet-LHL	glszm	LowGrayLevelZoneEmphasis	0.013322321
wavelet-LHL	glszm	ZoneEntropy	5.813390201
wavelet-LHL	glszm	SmallAreaLowGrayLevelEmphasis	0.012883225
wavelet-LHL	ngtdm	Coarseness	0.030499493
wavelet-LHL	ngtdm	Complexity	16091.91557
wavelet-LHL	ngtdm	Strength	42.90571164
wavelet-LHL	ngtdm	Contrast	2.196547242
wavelet-LHL	ngtdm	Busyness	0.021926583
wavelet-LHH	gldm	GrayLevelVariance	50.69020425
wavelet-LHH	gldm	HighGrayLevelEmphasis	292.4672131
wavelet-LHH	gldm	DependenceEntropy	5.387132625
wavelet-LHH	gldm	DependenceNonUniformity	49.70491803
wavelet-LHH	gldm	GrayLevelNonUniformity	5.754098361
wavelet-LHH	gldm	SmallDependenceEmphasis	0.639615209
wavelet-LHH	gldm	SmallDependenceHighGrayLevelEmphasis	209.8609973
wavelet-LHH	gldm	DependenceNonUniformityNormalized	0.407417361
wavelet-LHH	gldm	LargeDependenceEmphasis	3.393442623
wavelet-LHH	gldm	LargeDependenceLowGrayLevelEmphasis	0.043571021
wavelet-LHH	gldm	DependenceVariance	0.705993013
wavelet-LHH	gldm	LargeDependenceHighGrayLevelEmphasis	856.8114754
wavelet-LHH	gldm	SmallDependenceLowGrayLevelEmphasis	0.017539149
wavelet-LHH	gldm	LowGrayLevelEmphasis	0.021651919
wavelet-LHH	glcm	JointAverage	15.65779215
wavelet-LHH	glcm	SumAverage	31.31558429

wavelet-LHH	glcm	JointEntropy	6.894161509
wavelet-LHH	glcm	ClusterShade	151.3350829
wavelet-LHH	glcm	MaximumProbability	0.023518632
wavelet-LHH	glcm	Idmn	0.919112847
wavelet-LHH	glcm	JointEnergy	0.009177147
wavelet-LHH	glcm	Contrast	101.6804254
wavelet-LHH	glcm	DifferenceEntropy	4.144350408
wavelet-LHH	glcm	InverseVariance	0.111027934
wavelet-LHH	glcm	DifferenceVariance	31.80471498
wavelet-LHH	glcm	Idn	0.809901775
wavelet-LHH	glcm	Idm	0.109659028
wavelet-LHH	glcm	Correlation	-0.007873974
wavelet-LHH	glcm	Autocorrelation	244.7106532
wavelet-LHH	glcm	SumEntropy	4.824776904
wavelet-LHH	glcm	SumSquares	50.3351393
wavelet-LHH	glcm	ClusterProminence	26607.66814
wavelet-LHH	glcm	Imc2	0.993007041
wavelet-LHH	glcm	Imc1	-0.476579414
wavelet-LHH	glcm	DifferenceAverage	8.279417383
wavelet-LHH	glcm	Id	0.194259146
wavelet-LHH	glcm	ClusterTendency	99.66013185
wavelet-LHH	firstorder	InterquartileRange	263.3119538
wavelet-LHH	firstorder	Skewness	0.1689544
wavelet-LHH	firstorder	Uniformity	0.047164741
wavelet-LHH	firstorder	Median	16.18530974
wavelet-LHH	firstorder	Energy	3968633.299
wavelet-LHH	firstorder	RobustMeanAbsoluteDeviation	105.7782779
wavelet-LHH	firstorder	MeanAbsoluteDeviation	147.6737005
wavelet-LHH	firstorder	TotalEnergy	107153099.1
wavelet-LHH	firstorder	Maximum	443.0582815
wavelet-LHH	firstorder	RootMeanSquared	180.3601429
wavelet-LHH	firstorder	90Percentile	288.3167674
wavelet-LHH	firstorder	Minimum	-337.5133952
wavelet-LHH	firstorder	Entropy	4.631988226
wavelet-LHH	firstorder	Range	780.5716767
wavelet-LHH	firstorder	Variance	31836.55997
wavelet-LHH	firstorder	10Percentile	-188.7147389
wavelet-LHH	firstorder	Kurtosis	2.390318165
wavelet-LHH	firstorder	Mean	26.32909362
wavelet-LHH	glrlm	ShortRunLowGrayLevelEmphasis	0.021757173
wavelet-LHH	glrlm	GrayLevelVariance	51.28613059
wavelet-LHH	glrlm	LowGrayLevelRunEmphasis	0.021947803
wavelet-LHH	glrlm	GrayLevelNonUniformityNormalized	0.046542921
wavelet-LHH	glrlm	RunVariance	0.025880542
wavelet-LHH	glrlm	GrayLevelNonUniformity	5.539458136
wavelet-LHH	glrlm	LongRunEmphasis	1.077727084
wavelet-LHH	glrlm	ShortRunHighGrayLevelEmphasis	289.7167817
wavelet-LHH	glrlm	RunLengthNonUniformity	113.3478859
wavelet-LHH	glrlm	ShortRunEmphasis	0.98130504
wavelet-LHH	glrlm	LongRunHighGrayLevelEmphasis	312.2868483
wavelet-LHH	glrlm	RunPercentage	0.975409836
wavelet-LHH	glrlm	LongRunLowGrayLevelEmphasis	0.02271769
wavelet-LHH	glrlm	RunEntropy	4.730227581

wavelet-LHH	glrlm	HighGrayLevelRunEmphasis	293.9950155
wavelet-LHH	glrlm	RunLengthNonUniformityNormalized	0.952046197
wavelet-LHH	glszm	GrayLevelVariance	59.40018496
wavelet-LHH	glszm	ZoneVariance	1.091029198
wavelet-LHH	glszm	GrayLevelNonUniformityNormalized	0.042145594
wavelet-LHH	glszm	SizeZoneNonUniformityNormalized	0.623728366
wavelet-LHH	glszm	SizeZoneNonUniformity	54.26436782
wavelet-LHH	glszm	GrayLevelNonUniformity	3.666666667
wavelet-LHH	glszm	LargeAreaEmphasis	3.057471264
wavelet-LHH	glszm	SmallAreaHighGrayLevelEmphasis	276.1055587
wavelet-LHH	glszm	ZonePercentage	0.713114754
wavelet-LHH	glszm	LargeAreaLowGrayLevelEmphasis	0.043181937
wavelet-LHH	glszm	LargeAreaHighGrayLevelEmphasis	839.0689655
wavelet-LHH	glszm	HighGrayLevelZoneEmphasis	316.8735632
wavelet-LHH	glszm	SmallAreaEmphasis	0.816313059
wavelet-LHH	glszm	LowGrayLevelZoneEmphasis	0.026119392
wavelet-LHH	glszm	ZoneEntropy	5.228109192
wavelet-LHH	glszm	SmallAreaLowGrayLevelEmphasis	0.02364345
wavelet-LHH	ngtdm	Coarseness	0.033065271
wavelet-LHH	ngtdm	Complexity	2268.686702
wavelet-LHH	ngtdm	Strength	10.9296356
wavelet-LHH	ngtdm	Contrast	0.682363074
wavelet-LHH	ngtdm	Busyness	0.072125888
wavelet-LLH	gldm	GrayLevelVariance	169.2610185
wavelet-LLH	gldm	HighGrayLevelEmphasis	933.647541
wavelet-LLH	gldm	DependenceEntropy	5.812674813
wavelet-LLH	gldm	DependenceNonUniformity	59.04918033
wavelet-LLH	gldm	GrayLevelNonUniformity	3.704918033
wavelet-LLH	gldm	SmallDependenceEmphasis	0.709984062
wavelet-LLH	gldm	SmallDependenceHighGrayLevelEmphasis	693.6442395
wavelet-LLH	gldm	DependenceNonUniformityNormalized	0.484009675
wavelet-LLH	gldm	LargeDependenceEmphasis	2.803278689
wavelet-LLH	gldm	LargeDependenceLowGrayLevelEmphasis	0.017952538
wavelet-LLH	gldm	DependenceVariance	0.577801666
wavelet-LLH	gldm	LargeDependenceHighGrayLevelEmphasis	2167.319672
wavelet-LLH	gldm	SmallDependenceLowGrayLevelEmphasis	0.011308366
wavelet-LLH	gldm	LowGrayLevelEmphasis	0.012288026
wavelet-LLH	glcm	JointAverage	26.37964688
wavelet-LLH	glcm	SumAverage	52.75929376
wavelet-LLH	glcm	JointEntropy	7.037467695
wavelet-LLH	glcm	ClusterShade	2886.166358
wavelet-LLH	glcm	MaximumProbability	0.01740936
wavelet-LLH	glcm	Idmn	0.933374627
wavelet-LLH	glcm	JointEnergy	0.00799728
wavelet-LLH	glcm	Contrast	258.187796
wavelet-LLH	glcm	DifferenceEntropy	4.541596102
wavelet-LLH	glcm	InverseVariance	0.097512762
wavelet-LLH	glcm	DifferenceVariance	94.1735534
wavelet-LLH	glcm	Idn	0.835700421
wavelet-LLH	glcm	Idm	0.092053535
wavelet-LLH	glcm	Correlation	0.154516885
wavelet-LLH	glcm	Autocorrelation	720.3866568
wavelet-LLH	glcm	SumEntropy	5.273720587

wavelet-LLH	glcm	SumSquares	153.3587117
wavelet-LLH	glcm	ClusterProminence	373097.0112
wavelet-LLH	glcm	Imc2	0.999366217
wavelet-LLH	glcm	Imc1	-0.645617952
wavelet-LLH	glcm	DifferenceAverage	12.37753709
wavelet-LLH	glcm	Id	0.166394979
wavelet-LLH	glcm	ClusterTendency	355.247051
wavelet-LLH	firstorder	InterquartileRange	501.274393
wavelet-LLH	firstorder	Skewness	0.197169152
wavelet-LLH	firstorder	Uniformity	0.030368181
wavelet-LLH	firstorder	Median	-110.5457948
wavelet-LLH	firstorder	Energy	13604160.92
wavelet-LLH	firstorder	RobustMeanAbsoluteDeviation	207.7551793
wavelet-LLH	firstorder	MeanAbsoluteDeviation	278.8835145
wavelet-LLH	firstorder	TotalEnergy	367312344.8
wavelet-LLH	firstorder	Maximum	632.2598979
wavelet-LLH	firstorder	RootMeanSquared	333.9304055
wavelet-LLH	firstorder	90Percentile	411.6754444
wavelet-LLH	firstorder	Minimum	-747.6573876
wavelet-LLH	firstorder	Entropy	5.317220125
wavelet-LLH	firstorder	Range	1379.917285
wavelet-LLH	firstorder	Variance	106433.907
wavelet-LLH	firstorder	10Percentile	-478.1098607
wavelet-LLH	firstorder	Kurtosis	2.16726832
wavelet-LLH	firstorder	Mean	-71.24330651
wavelet-LLH	glrlm	ShortRunLowGrayLevelEmphasis	0.012418199
wavelet-LLH	glrlm	GrayLevelVariance	170.1689688
wavelet-LLH	glrlm	LowGrayLevelRunEmphasis	0.012465245
wavelet-LLH	glrlm	GrayLevelNonUniformityNormalized	0.029964958
wavelet-LLH	glrlm	RunVariance	0.018892341
wavelet-LLH	glrlm	GrayLevelNonUniformity	3.58720189
wavelet-LLH	glrlm	LongRunEmphasis	1.058580768
wavelet-LLH	glrlm	ShortRunHighGrayLevelEmphasis	926.3820181
wavelet-LLH	glrlm	RunLengthNonUniformity	115.2261076
wavelet-LLH	glrlm	ShortRunEmphasis	0.985354808
wavelet-LLH	glrlm	LongRunHighGrayLevelEmphasis	981.0179889
wavelet-LLH	glrlm	RunPercentage	0.981084489
wavelet-LLH	glrlm	LongRunLowGrayLevelEmphasis	0.01265343
wavelet-LLH	glrlm	RunEntropy	5.379620021
wavelet-LLH	glrlm	HighGrayLevelRunEmphasis	937.3092122
wavelet-LLH	glrlm	RunLengthNonUniformityNormalized	0.962215319
wavelet-LLH	glszm	GrayLevelVariance	182.2803305
wavelet-LLH	glszm	ZoneVariance	0.847442282
wavelet-LLH	glszm	GrayLevelNonUniformityNormalized	0.027387958
wavelet-LLH	glszm	SizeZoneNonUniformityNormalized	0.693526483
wavelet-LLH	glszm	SizeZoneNonUniformity	65.19148936
wavelet-LLH	glszm	GrayLevelNonUniformity	2.574468085
wavelet-LLH	glszm	LargeAreaEmphasis	2.531914894
wavelet-LLH	glszm	SmallAreaHighGrayLevelEmphasis	846.9804588
wavelet-LLH	glszm	ZonePercentage	0.770491803
wavelet-LLH	glszm	LargeAreaLowGrayLevelEmphasis	0.01956879
wavelet-LLH	glszm	LargeAreaHighGrayLevelEmphasis	1944.946809
wavelet-LLH	glszm	HighGrayLevelZoneEmphasis	981.2446809

wavelet-LLH	glszm	SmallAreaEmphasis	0.857639628
wavelet-LLH	glszm	LowGrayLevelZoneEmphasis	0.0149832
wavelet-LLH	glszm	ZoneEntropy	5.664708634
wavelet-LLH	glszm	SmallAreaLowGrayLevelEmphasis	0.01446489
wavelet-LLH	ngtdm	Coarseness	0.034274539
wavelet-LLH	ngtdm	Complexity	8623.081849
wavelet-LLH	ngtdm	Strength	34.29333976
wavelet-LLH	ngtdm	Contrast	1.384946465
wavelet-LLH	ngtdm	Busyness	0.029047131
wavelet-LLL	gldm	GrayLevelVariance	514.6764983
wavelet-LLL	gldm	HighGrayLevelEmphasis	3148.385246
wavelet-LLL	gldm	DependenceEntropy	6.207256497
wavelet-LLL	gldm	DependenceNonUniformity	74.37704918
wavelet-LLL	gldm	GrayLevelNonUniformity	2.37704918
wavelet-LLL	gldm	SmallDependenceEmphasis	0.806010929
wavelet-LLL	gldm	SmallDependenceHighGrayLevelEmphasis	2441.348361
wavelet-LLL	gldm	DependenceNonUniformityNormalized	0.609647944
wavelet-LLL	gldm	LargeDependenceEmphasis	1.885245902
wavelet-LLL	gldm	LargeDependenceLowGrayLevelEmphasis	0.012250181
wavelet-LLL	gldm	DependenceVariance	0.250201559
wavelet-LLL	gldm	LargeDependenceHighGrayLevelEmphasis	6575.221311
wavelet-LLL	gldm	SmallDependenceLowGrayLevelEmphasis	0.011753985
wavelet-LLL	gldm	LowGrayLevelEmphasis	0.011849228
wavelet-LLL	glcm	JointAverage	56.66865172
wavelet-LLL	glcm	SumAverage	113.3373034
wavelet-LLL	glcm	JointEntropy	7.124123789
wavelet-LLL	glcm	ClusterShade	-17572.74598
wavelet-LLL	glcm	MaximumProbability	0.013733639
wavelet-LLL	glcm	Idmn	0.931361933
wavelet-LLL	glcm	JointEnergy	0.007380037
wavelet-LLL	glcm	Contrast	703.8496969
wavelet-LLL	glcm	DifferenceEntropy	5.016911479
wavelet-LLL	glcm	InverseVariance	0.050861432
wavelet-LLL	glcm	DifferenceVariance	232.7607723
wavelet-LLL	glcm	Idn	0.828619912
wavelet-LLL	glcm	Idm	0.050929598
wavelet-LLL	glcm	Correlation	0.249420823
wavelet-LLL	glcm	Autocorrelation	3322.675117
wavelet-LLL	glcm	SumEntropy	5.59912256
wavelet-LLL	glcm	SumSquares	462.3628348
wavelet-LLL	glcm	ClusterProminence	3557671.33
wavelet-LLL	glcm	Imc2	0.999900425
wavelet-LLL	glcm	Imc1	-0.750327496
wavelet-LLL	glcm	DifferenceAverage	21.11740843
wavelet-LLL	glcm	Id	0.108970567
wavelet-LLL	glcm	ClusterTendency	1145.601642
wavelet-LLL	firstorder	InterquartileRange	889.1756494
wavelet-LLL	firstorder	Skewness	-0.329004013
wavelet-LLL	firstorder	Uniformity	0.01948401
wavelet-LLL	firstorder	Median	-600.7122584
wavelet-LLL	firstorder	Energy	95375307.01
wavelet-LLL	firstorder	RobustMeanAbsoluteDeviation	352.821653
wavelet-LLL	firstorder	MeanAbsoluteDeviation	475.4274759

wavelet-LLL	firstorder	TotalEnergy	2575133289
wavelet-LLL	firstorder	Maximum	348.0749654
wavelet-LLL	firstorder	RootMeanSquared	884.1746499
wavelet-LLL	firstorder	90Percentile	-19.54505979
wavelet-LLL	firstorder	Minimum	-1935.384884
wavelet-LLL	firstorder	Entropy	5.881448252
wavelet-LLL	firstorder	Range	2283.459849
wavelet-LLL	firstorder	Variance	321361.8999
wavelet-LLL	firstorder	10Percentile	-1483.645473
wavelet-LLL	firstorder	Kurtosis	2.224982358
wavelet-LLL	firstorder	Mean	-678.5299637
wavelet-LLL	glrlm	ShortRunLowGrayLevelEmphasis	0.011969954
wavelet-LLL	glrlm	GrayLevelVariance	516.2406492
wavelet-LLL	glrlm	LowGrayLevelRunEmphasis	0.011973803
wavelet-LLL	glrlm	GrayLevelNonUniformityNormalized	0.019380873
wavelet-LLL	glrlm	RunVariance	0.010713202
wavelet-LLL	glrlm	GrayLevelNonUniformity	2.33928701
wavelet-LLL	glrlm	LongRunEmphasis	1.03283938
wavelet-LLL	glrlm	ShortRunHighGrayLevelEmphasis	3110.507003
wavelet-LLL	glrlm	RunLengthNonUniformity	118.1324747
wavelet-LLL	glrlm	ShortRunEmphasis	0.991790155
wavelet-LLL	glrlm	LongRunHighGrayLevelEmphasis	3265.525396
wavelet-LLL	glrlm	RunPercentage	0.989281211
wavelet-LLL	glrlm	LongRunLowGrayLevelEmphasis	0.011989199
wavelet-LLL	glrlm	RunEntropy	5.908118234
wavelet-LLL	glrlm	HighGrayLevelRunEmphasis	3141.510681
wavelet-LLL	glrlm	RunLengthNonUniformityNormalized	0.978573596
wavelet-LLL	glszm	GrayLevelVariance	537.1528345
wavelet-LLL	glszm	ZoneVariance	0.211882086
wavelet-LLL	glszm	GrayLevelNonUniformityNormalized	0.018049887
wavelet-LLL	glszm	SizeZoneNonUniformityNormalized	0.764353741
wavelet-LLL	glszm	SizeZoneNonUniformity	80.25714286
wavelet-LLL	glszm	GrayLevelNonUniformity	1.895238095
wavelet-LLL	glszm	LargeAreaEmphasis	1.561904762
wavelet-LLL	glszm	SmallAreaHighGrayLevelEmphasis	2688.591071
wavelet-LLL	glszm	ZonePercentage	0.860655738
wavelet-LLL	glszm	LargeAreaLowGrayLevelEmphasis	0.013933315
wavelet-LLL	glszm	LargeAreaHighGrayLevelEmphasis	5301.171429
wavelet-LLL	glszm	HighGrayLevelZoneEmphasis	3046.685714
wavelet-LLL	glszm	SmallAreaEmphasis	0.896891534
wavelet-LLL	glszm	LowGrayLevelZoneEmphasis	0.013691771
wavelet-LLL	glszm	ZoneEntropy	6.15934397
wavelet-LLL	glszm	SmallAreaLowGrayLevelEmphasis	0.013638188
wavelet-LLL	ngtdm	Coarseness	0.031115353
wavelet-LLL	ngtdm	Complexity	32710.63596
wavelet-LLL	ngtdm	Strength	85.2358517
wavelet-LLL	ngtdm	Contrast	3.343564568
wavelet-LLL	ngtdm	Busyness	0.011042719
wavelet-HHL	gldm	GrayLevelVariance	39.11851653
wavelet-HHL	gldm	HighGrayLevelEmphasis	320.9180328
wavelet-HHL	gldm	DependenceEntropy	5.344827219
wavelet-HHL	gldm	DependenceNonUniformity	51.03278689
wavelet-HHL	gldm	GrayLevelNonUniformity	6.868852459

wavelet-HHL	gldm	SmallDependenceEmphasis	0.643214936
wavelet-HHL	gldm	SmallDependenceHighGrayLevelEmphasis	207.4173497
wavelet-HHL	gldm	DependenceNonUniformityNormalized	0.418301532
wavelet-HHL	gldm	LargeDependenceEmphasis	3.196721311
wavelet-HHL	gldm	LargeDependenceLowGrayLevelEmphasis	0.026322665
wavelet-HHL	gldm	DependenceVariance	0.615694706
wavelet-HHL	gldm	LargeDependenceHighGrayLevelEmphasis	992.2295082
wavelet-HHL	gldm	SmallDependenceLowGrayLevelEmphasis	0.015406782
wavelet-HHL	gldm	LowGrayLevelEmphasis	0.016972974
wavelet-HHL	glcm	JointAverage	16.64446602
wavelet-HHL	glcm	SumAverage	33.28893204
wavelet-HHL	glcm	JointEntropy	6.850969707
wavelet-HHL	glcm	ClusterShade	-16.6182441
wavelet-HHL	glcm	MaximumProbability	0.02394719
wavelet-HHL	glcm	Idmn	0.936366871
wavelet-HHL	glcm	JointEnergy	0.009495648
wavelet-HHL	glcm	Contrast	88.55639055
wavelet-HHL	glcm	DifferenceEntropy	3.995478338
wavelet-HHL	glcm	InverseVariance	0.151599487
wavelet-HHL	glcm	DifferenceVariance	31.10360628
wavelet-HHL	glcm	Idn	0.835418013
wavelet-HHL	glcm	Idm	0.13221535
wavelet-HHL	glcm	Correlation	-0.005798652
wavelet-HHL	glcm	Autocorrelation	276.8715012
wavelet-HHL	glcm	SumEntropy	4.713650816
wavelet-HHL	glcm	SumSquares	44.08990484
wavelet-HHL	glcm	ClusterProminence	21899.43887
wavelet-HHL	glcm	Imc2	0.991792755
wavelet-HHL	glcm	Imc1	-0.46783506
wavelet-HHL	glcm	DifferenceAverage	7.431202068
wavelet-HHL	glcm	Id	0.218213894
wavelet-HHL	glcm	ClusterTendency	87.80322881
wavelet-HHL	firstorder	InterquartileRange	168.6880422
wavelet-HHL	firstorder	Skewness	0.026860859
wavelet-HHL	firstorder	Uniformity	0.056302069
wavelet-HHL	firstorder	Median	-1.158625066
wavelet-HHL	firstorder	Energy	2970059.936
wavelet-HHL	firstorder	RobustMeanAbsoluteDeviation	75.35198508
wavelet-HHL	firstorder	MeanAbsoluteDeviation	118.4801223
wavelet-HHL	firstorder	TotalEnergy	80191618.27
wavelet-HHL	firstorder	Maximum	448.4454213
wavelet-HHL	firstorder	RootMeanSquared	156.0280538
wavelet-HHL	firstorder	90Percentile	235.4143957
wavelet-HHL	firstorder	Minimum	-394.0061341
wavelet-HHL	firstorder	Entropy	4.437295298
wavelet-HHL	firstorder	Range	842.4515554
wavelet-HHL	firstorder	Variance	24278.03473
wavelet-HHL	firstorder	10Percentile	-186.1859283
wavelet-HHL	firstorder	Kurtosis	3.121300714
wavelet-HHL	firstorder	Mean	8.168160144
wavelet-HHL	glrlm	ShortRunLowGrayLevelEmphasis	0.017201556
wavelet-HHL	glrlm	GrayLevelVariance	39.52361472
wavelet-HHL	glrlm	LowGrayLevelRunEmphasis	0.017277447

wavelet-HHL	glrlm	GrayLevelNonUniformityNormalized	0.05553255
wavelet-HHL	glrlm	RunVariance	0.024609152
wavelet-HHL	glrlm	GrayLevelNonUniformity	6.617515373
wavelet-HHL	glrlm	LongRunEmphasis	1.073422098
wavelet-HHL	glrlm	ShortRunHighGrayLevelEmphasis	315.64579
wavelet-HHL	glrlm	RunLengthNonUniformity	113.776966
wavelet-HHL	glrlm	ShortRunEmphasis	0.982368798
wavelet-HHL	glrlm	LongRunHighGrayLevelEmphasis	343.8707212
wavelet-HHL	glrlm	RunPercentage	0.97667087
wavelet-HHL	glrlm	LongRunLowGrayLevelEmphasis	0.017593884
wavelet-HHL	glrlm	RunEntropy	4.543012764
wavelet-HHL	glrlm	HighGrayLevelRunEmphasis	321.1603982
wavelet-HHL	glrlm	RunLengthNonUniformityNormalized	0.954593597
wavelet-HHL	glszm	GrayLevelVariance	45.46360153
wavelet-HHL	glszm	ZoneVariance	0.884132646
wavelet-HHL	glszm	GrayLevelNonUniformityNormalized	0.047958779
wavelet-HHL	glszm	SizeZoneNonUniformityNormalized	0.615801295
wavelet-HHL	glszm	SizeZoneNonUniformity	53.57471264
wavelet-HHL	glszm	GrayLevelNonUniformity	4.172413793
wavelet-HHL	glszm	LargeAreaEmphasis	2.850574713
wavelet-HHL	glszm	SmallAreaHighGrayLevelEmphasis	261.5898679
wavelet-HHL	glszm	ZonePercentage	0.713114754
wavelet-HHL	glszm	LargeAreaLowGrayLevelEmphasis	0.029888352
wavelet-HHL	glszm	LargeAreaHighGrayLevelEmphasis	878.4827586
wavelet-HHL	glszm	HighGrayLevelZoneEmphasis	323.2413793
wavelet-HHL	glszm	SmallAreaEmphasis	0.811358459
wavelet-HHL	glszm	LowGrayLevelZoneEmphasis	0.022066599
wavelet-HHL	glszm	ZoneEntropy	5.211236421
wavelet-HHL	glszm	SmallAreaLowGrayLevelEmphasis	0.02121247
wavelet-HHL	ngtdm	Coarseness	0.043271585
wavelet-HHL	ngtdm	Complexity	1623.0842
wavelet-HHL	ngtdm	Strength	11.35063418
wavelet-HHL	ngtdm	Contrast	0.481142497
wavelet-HHL	ngtdm	Busyness	0.049612905
wavelet-HLH	gldm	GrayLevelVariance	20.73078474
wavelet-HLH	gldm	HighGrayLevelEmphasis	161.795082
wavelet-HLH	gldm	DependenceEntropy	5.428298969
wavelet-HLH	gldm	DependenceNonUniformity	32.42622951
wavelet-HLH	gldm	GrayLevelNonUniformity	8.557377049
wavelet-HLH	gldm	SmallDependenceEmphasis	0.482008057
wavelet-HLH	gldm	SmallDependenceHighGrayLevelEmphasis	85.93831098
wavelet-HLH	gldm	DependenceNonUniformityNormalized	0.265788766
wavelet-HLH	gldm	LargeDependenceEmphasis	7
wavelet-HLH	gldm	LargeDependenceLowGrayLevelEmphasis	0.081793469
wavelet-HLH	gldm	DependenceVariance	1.955925826
wavelet-HLH	gldm	LargeDependenceHighGrayLevelEmphasis	1046.92623
wavelet-HLH	gldm	SmallDependenceLowGrayLevelEmphasis	0.02383334
wavelet-HLH	gldm	LowGrayLevelEmphasis	0.029196326
wavelet-HLH	glcm	JointAverage	11.93774038
wavelet-HLH	glcm	SumAverage	23.87548076
wavelet-HLH	glcm	JointEntropy	6.527564949
wavelet-HLH	glcm	ClusterShade	-9.336841052
wavelet-HLH	glcm	MaximumProbability	0.033899352

wavelet-HLH	glcm	Idmn	0.928988548
wavelet-HLH	glcm	JointEnergy	0.012527467
wavelet-HLH	glcm	Contrast	42.6337061
wavelet-HLH	glcm	DifferenceEntropy	3.625549502
wavelet-HLH	glcm	InverseVariance	0.166786993
wavelet-HLH	glcm	DifferenceVariance	15.4857696
wavelet-HLH	glcm	Idn	0.826500032
wavelet-HLH	glcm	Idm	0.183209976
wavelet-HLH	glcm	Correlation	-0.075869334
wavelet-HLH	glcm	Autocorrelation	141.100644
wavelet-HLH	glcm	SumEntropy	4.284280689
wavelet-HLH	glcm	SumSquares	19.87279954
wavelet-HLH	glcm	ClusterProminence	4187.746532
wavelet-HLH	glcm	Imc2	0.971826045
wavelet-HLH	glcm	Imc1	-0.367791485
wavelet-HLH	glcm	DifferenceAverage	5.150601688
wavelet-HLH	glcm	Id	0.276892661
wavelet-HLH	glcm	ClusterTendency	36.85749206
wavelet-HLH	firstorder	InterquartileRange	160.0769468
wavelet-HLH	firstorder	Skewness	-0.089956044
wavelet-HLH	firstorder	Uniformity	0.070142435
wavelet-HLH	firstorder	Median	7.765154102
wavelet-HLH	firstorder	Energy	1613501.25
wavelet-HLH	firstorder	RobustMeanAbsoluteDeviation	63.11643335
wavelet-HLH	firstorder	MeanAbsoluteDeviation	91.42966237
wavelet-HLH	firstorder	TotalEnergy	43564533.75
wavelet-HLH	firstorder	Maximum	260.9864532
wavelet-HLH	firstorder	RootMeanSquared	115.0018264
wavelet-HLH	firstorder	90Percentile	167.9715397
wavelet-HLH	firstorder	Minimum	-272.0094992
wavelet-HLH	firstorder	Entropy	4.059629118
wavelet-HLH	firstorder	Range	532.9959524
wavelet-HLH	firstorder	Variance	13146.21684
wavelet-HLH	firstorder	10Percentile	-150.0290571
wavelet-HLH	firstorder	Kurtosis	2.609908993
wavelet-HLH	firstorder	Mean	8.899620107
wavelet-HLH	glrlm	ShortRunLowGrayLevelEmphasis	0.029863045
wavelet-HLH	glrlm	GrayLevelVariance	21.34090426
wavelet-HLH	glrlm	LowGrayLevelRunEmphasis	0.030203622
wavelet-HLH	glrlm	GrayLevelNonUniformityNormalized	0.068281127
wavelet-HLH	glrlm	RunVariance	0.054587448
wavelet-HLH	glrlm	GrayLevelNonUniformity	7.932014464
wavelet-HLH	glrlm	LongRunEmphasis	1.159287949
wavelet-HLH	glrlm	ShortRunHighGrayLevelEmphasis	157.1137513
wavelet-HLH	glrlm	RunLengthNonUniformity	105.7734712
wavelet-HLH	glrlm	ShortRunEmphasis	0.963995564
wavelet-HLH	glrlm	LongRunHighGrayLevelEmphasis	185.9682379
wavelet-HLH	glrlm	RunPercentage	0.952080706
wavelet-HLH	glrlm	LongRunLowGrayLevelEmphasis	0.031685571
wavelet-HLH	glrlm	RunEntropy	4.27248488
wavelet-HLH	glrlm	HighGrayLevelRunEmphasis	162.4865139
wavelet-HLH	glrlm	RunLengthNonUniformityNormalized	0.909989903
wavelet-HLH	glszm	GrayLevelVariance	29.54453627

wavelet-HLH	glszm	ZoneVariance	3.886134068
wavelet-HLH	glszm	GrayLevelNonUniformityNormalized	0.058769513
wavelet-HLH	glszm	SizeZoneNonUniformityNormalized	0.529384757
wavelet-HLH	glszm	SizeZoneNonUniformity	34.93939394
wavelet-HLH	glszm	GrayLevelNonUniformity	3.878787879
wavelet-HLH	glszm	LargeAreaEmphasis	7.303030303
wavelet-HLH	glszm	SmallAreaHighGrayLevelEmphasis	139.3471149
wavelet-HLH	glszm	ZonePercentage	0.540983607
wavelet-HLH	glszm	LargeAreaLowGrayLevelEmphasis	0.099076548
wavelet-HLH	glszm	LargeAreaHighGrayLevelEmphasis	1140.893939
wavelet-HLH	glszm	HighGrayLevelZoneEmphasis	172.8181818
wavelet-HLH	glszm	SmallAreaEmphasis	0.753169986
wavelet-HLH	glszm	LowGrayLevelZoneEmphasis	0.045472039
wavelet-HLH	glszm	ZoneEntropy	4.903724233
wavelet-HLH	glszm	SmallAreaLowGrayLevelEmphasis	0.042456277
wavelet-HLH	ngtdm	Coarseness	0.040911566
wavelet-HLH	ngtdm	Complexity	632.3525254
wavelet-HLH	ngtdm	Strength	5.125923276
wavelet-HLH	ngtdm	Contrast	0.38719794
wavelet-HLH	ngtdm	Busyness	0.112343348

Image type	Feature Class	Feature Name	36: RTSTRUCT: C99-DNU_segment_Left_Lung_GTV
general	info	BoundingBox	(0, 1, 1, 31, 33, 25)
general	info	EnabledImageTypes	{u'Original': {}, u'Wavelet': {}}
general	info	GeneralSettings	{'distances': [1], 'additionalInfo': True, 'force2D': False, 'interpolator': 'sitkBSpline', 'resampledPixelSpacing': [3.0], 'label': 1, 'normalizeScale': 1, 'normalize': False, 'force2Ddimension': 0, 'removeOutliers': None, 'minimumROISize': None, u'binWidth': 25.0, 'minimumROIDimensions': 1, u'correctMask': True, 'preCrop': False, u'symmetricalGLCM': True, 'resegmentRange': None, 'padDistance': 5}
general	info	ImageHash	4d527653aaa4d1426738551132625c03e96c48e9
general	info	ImageSpacing	(0.9765624999999998, 0.9765624999999998, 1.999999999999999)
general	info	MaskHash	7f98fb588ee7d20734c970ab4042e348d5c12f4b
general	info	NumpyVersion	1.13.1
general	info	PyWaveletVersion	0.5.2
general	info	SimpleITKVersion	1.1.0
general	info	Version	1.3.0.post102+g1b0eb74
general	info	VolumeNum	1
general	info	VoxelNum	7698
original	shape	Maximum3DDiameter	116.1507641
original	shape	Maximum2DDiameterSlice	89.49860334
original	shape	Sphericity	0.670519105
original	shape	MinorAxis	74.40511291
original	shape	Elongation	0.817139631
original	shape	SurfaceVolumeRatio	0.121756572
original	shape	Volume	207846
original	shape	MajorAxis	91.05556756
original	shape	SurfaceArea	25306.61637
original	shape	Flatness	0.534589989
original	shape	LeastAxis	48.67739482
original	shape	Maximum2DDiameterColumn	90.04998612
original	shape	Maximum2DDiameterRow	115.8015544
original	gldm	GrayLevelVariance	132.1839884
original	gldm	HighGrayLevelEmphasis	1460.520395
original	gldm	DependenceEntropy	7.179779892
original	gldm	DependenceNonUniformity	757.1228891
original	gldm	GrayLevelNonUniformity	846.4250455
original	gldm	SmallDependenceEmphasis	0.278086883
original	gldm	SmallDependenceHighGrayLevelEmphasis	250.8933204
original	gldm	DependenceNonUniformityNormalized	0.098353194
original	gldm	LargeDependenceEmphasis	62.29020525
original	gldm	LargeDependenceLowGrayLevelEmphasis	0.038731668
original	gldm	DependenceVariance	24.85132227
original	gldm	LargeDependenceHighGrayLevelEmphasis	115950.8124
original	gldm	SmallDependenceLowGrayLevelEmphasis	0.001297991
original	gldm	LowGrayLevelEmphasis	0.002206709
original	glcm	JointAverage	37.81862967
original	glcm	SumAverage	75.63725934
original	glcm	JointEntropy	7.371523323
original	glcm	ClusterShade	-10365.899
original	glcm	MaximumProbability	0.089509566
original	glcm	Idmn	0.976094209
original	glcm	JointEnergy	0.031073481
original	glcm	Contrast	86.36953628

original	glcm	DifferenceEntropy	3.561535821
original	glcm	InverseVariance	0.343052132
original	glcm	DifferenceVariance	60.28328217
original	glcm	Idn	0.928479956
original	glcm	Idm	0.407674649
original	glcm	Correlation	0.603712224
original	glcm	Autocorrelation	1497.147702
original	glcm	SumEntropy	4.944892121
original	glcm	SumSquares	110.0038158
original	glcm	ClusterProminence	531279.1308
original	glcm	Imc2	0.799047191
original	glcm	Imc1	-0.134751288
original	glcm	DifferenceAverage	5.048430058
original	glcm	Id	0.454244001
original	glcm	ClusterTendency	353.6457271
original	firstorder	InterquartileRange	276.75
original	firstorder	Skewness	-1.354804255
original	firstorder	Uniformity	0.10995389
original	firstorder	Median	28
original	firstorder	Energy	758987654
original	firstorder	RobustMeanAbsoluteDeviation	157.6002427
original	firstorder	MeanAbsoluteDeviation	234.1415368
original	firstorder	TotalEnergy	20492666658
original	firstorder	Maximum	342
original	firstorder	RootMeanSquared	313.9990994
original	firstorder	90Percentile	73
original	firstorder	Minimum	-1017
original	firstorder	Entropy	4.185412839
original	firstorder	Range	1359
original	firstorder	Variance	82546.25071
original	firstorder	10Percentile	-671
original	firstorder	Kurtosis	3.321852251
original	firstorder	Mean	-126.6853728
original	glrlm	ShortRunLowGrayLevelEmphasis	0.002466657
original	glrlm	GrayLevelVariance	147.146464
original	glrlm	LowGrayLevelRunEmphasis	0.00257048
original	glrlm	GrayLevelNonUniformityNormalized	0.079638881
original	glrlm	RunVariance	0.449873403
original	glrlm	GrayLevelNonUniformity	492.66983
original	glrlm	LongRunEmphasis	2.003135803
original	glrlm	ShortRunHighGrayLevelEmphasis	1133.460889
original	glrlm	RunLengthNonUniformity	4425.980781
original	glrlm	ShortRunEmphasis	0.869757743
original	glrlm	LongRunHighGrayLevelEmphasis	3218.585251
original	glrlm	RunPercentage	0.803125687
original	glrlm	LongRunLowGrayLevelEmphasis	0.00325451
original	glrlm	RunEntropy	5.251257827
original	glrlm	HighGrayLevelRunEmphasis	1369.450794
original	glrlm	RunLengthNonUniformityNormalized	0.715283703
original	glszm	GrayLevelVariance	141.522278
original	glszm	ZoneVariance	2414.986887
original	glszm	GrayLevelNonUniformityNormalized	0.025779836
original	glszm	SizeZoneNonUniformityNormalized	0.592707773
original	glszm	SizeZoneNonUniformity	1356.115385
original	glszm	GrayLevelNonUniformity	58.98426573
original	glszm	LargeAreaEmphasis	2426.306818
original	glszm	SmallAreaHighGrayLevelEmphasis	664.3189413
original	glszm	ZonePercentage	0.297220057

original	glszm	LargeAreaLowGrayLevelEmphasis	1.29561653
original	glszm	LargeAreaHighGrayLevelEmphasis	4582810.228
original	glszm	HighGrayLevelZoneEmphasis	856.8977273
original	glszm	SmallAreaEmphasis	0.797465257
original	glszm	LowGrayLevelZoneEmphasis	0.004775011
original	glszm	ZoneEntropy	6.521097015
original	glszm	SmallAreaLowGrayLevelEmphasis	0.004021076
original	ngtdm	Coarseness	0.000625048
original	ngtdm	Complexity	4384.645107
original	ngtdm	Strength	1.516168279
original	ngtdm	Contrast	0.410462481
original	ngtdm	Busyness	0.574035129
wavelet-HHH	gldm	GrayLevelVariance	5.651918257
wavelet-HHH	gldm	HighGrayLevelEmphasis	311.8291764
wavelet-HHH	gldm	DependenceEntropy	6.1380304
wavelet-HHH	gldm	DependenceNonUniformity	616.0958691
wavelet-HHH	gldm	GrayLevelNonUniformity	1423.858145
wavelet-HHH	gldm	SmallDependenceEmphasis	0.186836077
wavelet-HHH	gldm	SmallDependenceHighGrayLevelEmphasis	60.88344517
wavelet-HHH	gldm	DependenceNonUniformityNormalized	0.080033238
wavelet-HHH	gldm	LargeDependenceEmphasis	65.81995323
wavelet-HHH	gldm	LargeDependenceLowGrayLevelEmphasis	0.21576271
wavelet-HHH	gldm	DependenceVariance	21.74539503
wavelet-HHH	gldm	LargeDependenceHighGrayLevelEmphasis	20258.00637
wavelet-HHH	gldm	SmallDependenceLowGrayLevelEmphasis	0.000894421
wavelet-HHH	gldm	LowGrayLevelEmphasis	0.003635362
wavelet-HHH	glcm	JointAverage	17.49632985
wavelet-HHH	glcm	SumAverage	34.99265971
wavelet-HHH	glcm	JointEntropy	5.709517123
wavelet-HHH	glcm	ClusterShade	0.116298183
wavelet-HHH	glcm	MaximumProbability	0.123921856
wavelet-HHH	glcm	Idmn	0.991614022
wavelet-HHH	glcm	JointEnergy	0.054450713
wavelet-HHH	glcm	Contrast	10.98983748
wavelet-HHH	glcm	DifferenceEntropy	2.737326094
wavelet-HHH	glcm	InverseVariance	0.39025839
wavelet-HHH	glcm	DifferenceVariance	6.55522409
wavelet-HHH	glcm	Idn	0.947517368
wavelet-HHH	glcm	Idm	0.458771012
wavelet-HHH	glcm	Correlation	-0.034957282
wavelet-HHH	glcm	Autocorrelation	305.9268706
wavelet-HHH	glcm	SumEntropy	3.438233929
wavelet-HHH	glcm	SumSquares	5.300213563
wavelet-HHH	glcm	ClusterProminence	883.7486515
wavelet-HHH	glcm	Imc2	0.547944853
wavelet-HHH	glcm	Imc1	-0.062331276
wavelet-HHH	glcm	DifferenceAverage	2.093669147
wavelet-HHH	glcm	Id	0.512046132
wavelet-HHH	glcm	ClusterTendency	10.21101677
wavelet-HHH	firstorder	InterquartileRange	39.20401598
wavelet-HHH	firstorder	Skewness	0.048349561
wavelet-HHH	firstorder	Uniformity	0.184964685
wavelet-HHH	firstorder	Median	0.440137207
wavelet-HHH	firstorder	Energy	26737432.1
wavelet-HHH	firstorder	RobustMeanAbsoluteDeviation	18.8955223
wavelet-HHH	firstorder	MeanAbsoluteDeviation	37.54411111
wavelet-HHH	firstorder	TotalEnergy	721910666.8
wavelet-HHH	firstorder	Maximum	428.8538433

wavelet-HHH	firstorder	RootMeanSquared	58.93467515
wavelet-HHH	firstorder	90Percentile	61.68248425
wavelet-HHH	firstorder	Minimum	-419.5746315
wavelet-HHH	firstorder	Entropy	3.029985068
wavelet-HHH	firstorder	Range	848.4284749
wavelet-HHH	firstorder	Variance	3473.295609
wavelet-HHH	firstorder	10Percentile	-63.50050575
wavelet-HHH	firstorder	Kurtosis	8.003237282
wavelet-HHH	firstorder	Mean	-0.018041958
wavelet-HHH	glrlm	ShortRunLowGrayLevelEmphasis	0.003236187
wavelet-HHH	glrlm	GrayLevelVariance	6.959537009
wavelet-HHH	glrlm	LowGrayLevelRunEmphasis	0.003730803
wavelet-HHH	glrlm	GrayLevelNonUniformityNormalized	0.148965311
wavelet-HHH	glrlm	RunVariance	0.494616897
wavelet-HHH	glrlm	GrayLevelNonUniformity	898.8969936
wavelet-HHH	glrlm	LongRunEmphasis	2.131683553
wavelet-HHH	glrlm	ShortRunHighGrayLevelEmphasis	266.7755955
wavelet-HHH	glrlm	RunLengthNonUniformity	4098.046387
wavelet-HHH	glrlm	ShortRunEmphasis	0.850188492
wavelet-HHH	glrlm	LongRunHighGrayLevelEmphasis	661.1173193
wavelet-HHH	glrlm	RunPercentage	0.783120491
wavelet-HHH	glrlm	LongRunLowGrayLevelEmphasis	0.007450889
wavelet-HHH	glrlm	RunEntropy	4.103958562
wavelet-HHH	glrlm	HighGrayLevelRunEmphasis	312.9301397
wavelet-HHH	glrlm	RunLengthNonUniformityNormalized	0.678998617
wavelet-HHH	glszm	GrayLevelVariance	19.88106854
wavelet-HHH	glszm	ZoneVariance	6106.116746
wavelet-HHH	glszm	GrayLevelNonUniformityNormalized	0.071047249
wavelet-HHH	glszm	SizeZoneNonUniformityNormalized	0.485837383
wavelet-HHH	glszm	SizeZoneNonUniformity	714.1809524
wavelet-HHH	glszm	GrayLevelNonUniformity	104.4394558
wavelet-HHH	glszm	LargeAreaEmphasis	6133.540136
wavelet-HHH	glszm	SmallAreaHighGrayLevelEmphasis	240.1251079
wavelet-HHH	glszm	ZonePercentage	0.190958691
wavelet-HHH	glszm	LargeAreaLowGrayLevelEmphasis	20.01327772
wavelet-HHH	glszm	LargeAreaHighGrayLevelEmphasis	1886508.267
wavelet-HHH	glszm	HighGrayLevelZoneEmphasis	326.655102
wavelet-HHH	glszm	SmallAreaEmphasis	0.724327131
wavelet-HHH	glszm	LowGrayLevelZoneEmphasis	0.004827476
wavelet-HHH	glszm	ZoneEntropy	5.696542262
wavelet-HHH	glszm	SmallAreaLowGrayLevelEmphasis	0.003780316
wavelet-HHH	ngtdm	Coarseness	9.65E-04
wavelet-HHH	ngtdm	Complexity	772.3623778
wavelet-HHH	ngtdm	Strength	0.489300929
wavelet-HHH	ngtdm	Contrast	0.018750448
wavelet-HHH	ngtdm	Busyness	1.147021489
wavelet-HLL	gldm	GrayLevelVariance	26.67140566
wavelet-HLL	gldm	HighGrayLevelEmphasis	761.6673162
wavelet-HLL	gldm	DependenceEntropy	7.026661951
wavelet-HLL	gldm	DependenceNonUniformity	825.0626137
wavelet-HLL	gldm	GrayLevelNonUniformity	799.4572616
wavelet-HLL	gldm	SmallDependenceEmphasis	0.256379658
wavelet-HLL	gldm	SmallDependenceHighGrayLevelEmphasis	210.2974211
wavelet-HLL	gldm	DependenceNonUniformityNormalized	0.107178827
wavelet-HLL	gldm	LargeDependenceEmphasis	50.75136399
wavelet-HLL	gldm	LargeDependenceLowGrayLevelEmphasis	0.068587358
wavelet-HLL	gldm	DependenceVariance	21.28640871
wavelet-HLL	gldm	LargeDependenceHighGrayLevelEmphasis	38269.48389

wavelet-HLL	gldm	SmallDependenceLowGrayLevelEmphasis	0.000589409
wavelet-HLL	gldm	LowGrayLevelEmphasis	0.001693098
wavelet-HLL	glcm	JointAverage	26.97813623
wavelet-HLL	glcm	SumAverage	53.95627246
wavelet-HLL	glcm	JointEntropy	7.449071149
wavelet-HLL	glcm	ClusterShade	223.8275401
wavelet-HLL	glcm	MaximumProbability	0.082668438
wavelet-HLL	glcm	Idmn	0.989995837
wavelet-HLL	glcm	JointEnergy	0.025660392
wavelet-HLL	glcm	Contrast	35.36066385
wavelet-HLL	glcm	DifferenceEntropy	3.401328624
wavelet-HLL	glcm	InverseVariance	0.317980855
wavelet-HLL	glcm	DifferenceVariance	21.7276569
wavelet-HLL	glcm	Idn	0.945017602
wavelet-HLL	glcm	Idm	0.368522057
wavelet-HLL	glcm	Correlation	0.28717474
wavelet-HLL	glcm	Autocorrelation	735.2247872
wavelet-HLL	glcm	SumEntropy	4.638705244
wavelet-HLL	glcm	SumSquares	25.08323066
wavelet-HLL	glcm	ClusterProminence	27433.58039
wavelet-HLL	glcm	Imc2	0.753536394
wavelet-HLL	glcm	Imc1	-0.112946165
wavelet-HLL	glcm	DifferenceAverage	3.628840868
wavelet-HLL	glcm	Id	0.431273947
wavelet-HLL	glcm	ClusterTendency	64.97225878
wavelet-HLL	firstorder	InterquartileRange	81.84523508
wavelet-HLL	firstorder	Skewness	0.376890456
wavelet-HLL	firstorder	Uniformity	0.103852593
wavelet-HLL	firstorder	Median	-4.782951004
wavelet-HLL	firstorder	Energy	128740672
wavelet-HLL	firstorder	RobustMeanAbsoluteDeviation	41.68075728
wavelet-HLL	firstorder	MeanAbsoluteDeviation	83.04694182
wavelet-HLL	firstorder	TotalEnergy	3475998143
wavelet-HLL	firstorder	Maximum	742.083972
wavelet-HLL	firstorder	RootMeanSquared	129.3209637
wavelet-HLL	firstorder	90Percentile	138.8038758
wavelet-HLL	firstorder	Minimum	-665.7291316
wavelet-HLL	firstorder	Entropy	4.049597627
wavelet-HLL	firstorder	Range	1407.813104
wavelet-HLL	firstorder	Variance	16627.21138
wavelet-HLL	firstorder	10Percentile	-162.4680788
wavelet-HLL	firstorder	Kurtosis	6.108410402
wavelet-HLL	firstorder	Mean	-9.833630031
wavelet-HLL	glrlm	ShortRunLowGrayLevelEmphasis	0.001599095
wavelet-HLL	glrlm	GrayLevelVariance	31.10150047
wavelet-HLL	glrlm	LowGrayLevelRunEmphasis	0.001757759
wavelet-HLL	glrlm	GrayLevelNonUniformityNormalized	0.080360152
wavelet-HLL	glrlm	RunVariance	0.377656925
wavelet-HLL	glrlm	GrayLevelNonUniformity	514.4193041
wavelet-HLL	glrlm	LongRunEmphasis	1.840021068
wavelet-HLL	glrlm	ShortRunHighGrayLevelEmphasis	679.8131394
wavelet-HLL	glrlm	RunLengthNonUniformity	4786.415067
wavelet-HLL	glrlm	ShortRunEmphasis	0.886770165
wavelet-HLL	glrlm	LongRunHighGrayLevelEmphasis	1395.018714
wavelet-HLL	glrlm	RunPercentage	0.829686032
wavelet-HLL	glrlm	LongRunLowGrayLevelEmphasis	0.002907137
wavelet-HLL	glrlm	RunEntropy	4.948809415
wavelet-HLL	glrlm	HighGrayLevelRunEmphasis	764.3165969

wavelet-HLL	glrlm	RunLengthNonUniformityNormalized	0.747623654
wavelet-HLL	glszm	GrayLevelVariance	61.15487858
wavelet-HLL	glszm	ZoneVariance	1890.699295
wavelet-HLL	glszm	GrayLevelNonUniformityNormalized	0.03723777
wavelet-HLL	glszm	SizeZoneNonUniformityNormalized	0.450338024
wavelet-HLL	glszm	SizeZoneNonUniformity	990.2933151
wavelet-HLL	glszm	GrayLevelNonUniformity	81.88585721
wavelet-HLL	glszm	LargeAreaEmphasis	1902.95407
wavelet-HLL	glszm	SmallAreaHighGrayLevelEmphasis	588.7854814
wavelet-HLL	glszm	ZonePercentage	0.285658613
wavelet-HLL	glszm	LargeAreaLowGrayLevelEmphasis	2.520951454
wavelet-HLL	glszm	LargeAreaHighGrayLevelEmphasis	1440004.846
wavelet-HLL	glszm	HighGrayLevelZoneEmphasis	814.4938608
wavelet-HLL	glszm	SmallAreaEmphasis	0.697871427
wavelet-HLL	glszm	LowGrayLevelZoneEmphasis	0.002262828
wavelet-HLL	glszm	ZoneEntropy	6.643662979
wavelet-HLL	glszm	SmallAreaLowGrayLevelEmphasis	0.001738427
wavelet-HLL	ngtdm	Coarseness	0.001083096
wavelet-HLL	ngtdm	Complexity	2937.388804
wavelet-HLL	ngtdm	Strength	1.335563419
wavelet-HLL	ngtdm	Contrast	0.05438061
wavelet-HLL	ngtdm	Busyness	0.450415078
wavelet-LHL	gldm	GrayLevelVariance	58.69040759
wavelet-LHL	gldm	HighGrayLevelEmphasis	1754.358275
wavelet-LHL	gldm	DependenceEntropy	7.21290666
wavelet-LHL	gldm	DependenceNonUniformity	1045.291244
wavelet-LHL	gldm	GrayLevelNonUniformity	642.2307093
wavelet-LHL	gldm	SmallDependenceEmphasis	0.336886494
wavelet-LHL	gldm	SmallDependenceHighGrayLevelEmphasis	614.327846
wavelet-LHL	gldm	DependenceNonUniformityNormalized	0.135787379
wavelet-LHL	gldm	LargeDependenceEmphasis	39.76877111
wavelet-LHL	gldm	LargeDependenceLowGrayLevelEmphasis	0.023574962
wavelet-LHL	gldm	DependenceVariance	17.81848713
wavelet-LHL	gldm	LargeDependenceHighGrayLevelEmphasis	68439.14315
wavelet-LHL	gldm	SmallDependenceLowGrayLevelEmphasis	0.000403613
wavelet-LHL	gldm	LowGrayLevelEmphasis	0.000819983
wavelet-LHL	glcm	JointAverage	41.08298829
wavelet-LHL	glcm	SumAverage	82.16597658
wavelet-LHL	glcm	JointEntropy	8.216342663
wavelet-LHL	glcm	ClusterShade	230.096906
wavelet-LHL	glcm	MaximumProbability	0.066982282
wavelet-LHL	glcm	Idmn	0.988741891
wavelet-LHL	glcm	JointEnergy	0.018297539
wavelet-LHL	glcm	Contrast	87.89874737
wavelet-LHL	glcm	DifferenceEntropy	3.875514937
wavelet-LHL	glcm	InverseVariance	0.283742122
wavelet-LHL	glcm	DifferenceVariance	56.33025325
wavelet-LHL	glcm	Idn	0.94444361
wavelet-LHL	glcm	Idm	0.318926426
wavelet-LHL	glcm	Correlation	0.188438563
wavelet-LHL	glcm	Autocorrelation	1698.292801
wavelet-LHL	glcm	SumEntropy	5.038463264
wavelet-LHL	glcm	SumSquares	54.42864203
wavelet-LHL	glcm	ClusterProminence	116736.5437
wavelet-LHL	glcm	Imc2	0.809639433
wavelet-LHL	glcm	Imc1	-0.128050762
wavelet-LHL	glcm	DifferenceAverage	5.482907267
wavelet-LHL	glcm	Id	0.382414888

wavelet-LHL	glcm	ClusterTendency	129.8158208
wavelet-LHL	firstorder	InterquartileRange	97.01236895
wavelet-LHL	firstorder	Skewness	0.100772011
wavelet-LHL	firstorder	Uniformity	0.083428255
wavelet-LHL	firstorder	Median	-3.366613546
wavelet-LHL	firstorder	Energy	282550007.3
wavelet-LHL	firstorder	RobustMeanAbsoluteDeviation	54.06205581
wavelet-LHL	firstorder	MeanAbsoluteDeviation	118.1593262
wavelet-LHL	firstorder	TotalEnergy	7628850198
wavelet-LHL	firstorder	Maximum	1057.697763
wavelet-LHL	firstorder	RootMeanSquared	191.5837669
wavelet-LHL	firstorder	90Percentile	200.454096
wavelet-LHL	firstorder	Minimum	-1013.123305
wavelet-LHL	firstorder	Entropy	4.502924712
wavelet-LHL	firstorder	Range	2070.821068
wavelet-LHL	firstorder	Variance	36637.72376
wavelet-LHL	firstorder	10Percentile	-224.6951795
wavelet-LHL	firstorder	Kurtosis	6.507136692
wavelet-LHL	firstorder	Mean	-8.161861529
wavelet-LHL	glrlm	ShortRunLowGrayLevelEmphasis	0.000799983
wavelet-LHL	glrlm	GrayLevelVariance	67.10347185
wavelet-LHL	glrlm	LowGrayLevelRunEmphasis	0.000857069
wavelet-LHL	glrlm	GrayLevelNonUniformityNormalized	0.065097357
wavelet-LHL	glrlm	RunVariance	0.280993336
wavelet-LHL	glrlm	GrayLevelNonUniformity	430.8580936
wavelet-LHL	glrlm	LongRunEmphasis	1.64541967
wavelet-LHL	glrlm	ShortRunHighGrayLevelEmphasis	1598.244685
wavelet-LHL	glrlm	RunLengthNonUniformity	5189.73811
wavelet-LHL	glrlm	ShortRunEmphasis	0.905212587
wavelet-LHL	glrlm	LongRunHighGrayLevelEmphasis	2869.070847
wavelet-LHL	glrlm	RunPercentage	0.858264884
wavelet-LHL	glrlm	LongRunLowGrayLevelEmphasis	0.001240928
wavelet-LHL	glrlm	RunEntropy	5.291808068
wavelet-LHL	glrlm	HighGrayLevelRunEmphasis	1760.779996
wavelet-LHL	glrlm	RunLengthNonUniformityNormalized	0.783933225
wavelet-LHL	glszm	GrayLevelVariance	124.4891822
wavelet-LHL	glszm	ZoneVariance	1103.574682
wavelet-LHL	glszm	GrayLevelNonUniformityNormalized	0.026895476
wavelet-LHL	glszm	SizeZoneNonUniformityNormalized	0.55166621
wavelet-LHL	glszm	SizeZoneNonUniformity	1571.145365
wavelet-LHL	glszm	GrayLevelNonUniformity	76.59831461
wavelet-LHL	glszm	LargeAreaEmphasis	1110.880618
wavelet-LHL	glszm	SmallAreaHighGrayLevelEmphasis	1419.328196
wavelet-LHL	glszm	ZonePercentage	0.369966225
wavelet-LHL	glszm	LargeAreaLowGrayLevelEmphasis	0.646733788
wavelet-LHL	glszm	LargeAreaHighGrayLevelEmphasis	1912045.102
wavelet-LHL	glszm	HighGrayLevelZoneEmphasis	1824.661517
wavelet-LHL	glszm	SmallAreaEmphasis	0.771111296
wavelet-LHL	glszm	LowGrayLevelZoneEmphasis	0.001159609
wavelet-LHL	glszm	ZoneEntropy	6.787366935
wavelet-LHL	glszm	SmallAreaLowGrayLevelEmphasis	0.000997319
wavelet-LHL	ngtdm	Coarseness	0.00102895
wavelet-LHL	ngtdm	Complexity	9864.092121
wavelet-LHL	ngtdm	Strength	2.599748404
wavelet-LHL	ngtdm	Contrast	0.088236421
wavelet-LHL	ngtdm	Busyness	0.208786846
wavelet-LHH	gldm	GrayLevelVariance	24.85048561
wavelet-LHH	gldm	HighGrayLevelEmphasis	1510.55339

wavelet-LHH	gldm	DependenceEntropy	6.705665752
wavelet-LHH	gldm	DependenceNonUniformity	856.0984671
wavelet-LHH	gldm	GrayLevelNonUniformity	896.5331255
wavelet-LHH	gldm	SmallDependenceEmphasis	0.277365507
wavelet-LHH	gldm	SmallDependenceHighGrayLevelEmphasis	435.7224434
wavelet-LHH	gldm	DependenceNonUniformityNormalized	0.111210505
wavelet-LHH	gldm	LargeDependenceEmphasis	45.56923876
wavelet-LHH	gldm	LargeDependenceLowGrayLevelEmphasis	0.031102316
wavelet-LHH	gldm	DependenceVariance	18.28225489
wavelet-LHH	gldm	LargeDependenceHighGrayLevelEmphasis	67467.04131
wavelet-LHH	gldm	SmallDependenceLowGrayLevelEmphasis	0.000385257
wavelet-LHH	gldm	LowGrayLevelEmphasis	0.000883742
wavelet-LHH	glcm	JointAverage	38.53832886
wavelet-LHH	glcm	SumAverage	77.07665772
wavelet-LHH	glcm	JointEntropy	7.204569319
wavelet-LHH	glcm	ClusterShade	7.799229533
wavelet-LHH	glcm	MaximumProbability	0.08647349
wavelet-LHH	glcm	Idmn	0.992491331
wavelet-LHH	glcm	JointEnergy	0.027824517
wavelet-LHH	glcm	Contrast	45.82084401
wavelet-LHH	glcm	DifferenceEntropy	3.477122367
wavelet-LHH	glcm	InverseVariance	0.323501607
wavelet-LHH	glcm	DifferenceVariance	29.74955368
wavelet-LHH	glcm	Idn	0.95388121
wavelet-LHH	glcm	Idm	0.36373068
wavelet-LHH	glcm	Correlation	-0.013036591
wavelet-LHH	glcm	Autocorrelation	1484.900417
wavelet-LHH	glcm	SumEntropy	4.294033822
wavelet-LHH	glcm	SumSquares	22.60770482
wavelet-LHH	glcm	ClusterProminence	24464.8246
wavelet-LHH	glcm	Imc2	0.722211795
wavelet-LHH	glcm	Imc1	-0.100608084
wavelet-LHH	glcm	DifferenceAverage	3.950877128
wavelet-LHH	glcm	Id	0.425321232
wavelet-LHH	glcm	ClusterTendency	44.60997527
wavelet-LHH	firstorder	InterquartileRange	64.73613838
wavelet-LHH	firstorder	Skewness	-0.019339719
wavelet-LHH	firstorder	Uniformity	0.116463124
wavelet-LHH	firstorder	Median	-0.837194322
wavelet-LHH	firstorder	Energy	119019658.4
wavelet-LHH	firstorder	RobustMeanAbsoluteDeviation	33.49243056
wavelet-LHH	firstorder	MeanAbsoluteDeviation	74.10853371
wavelet-LHH	firstorder	TotalEnergy	3213530777
wavelet-LHH	firstorder	Maximum	913.3792769
wavelet-LHH	firstorder	RootMeanSquared	124.3427295
wavelet-LHH	firstorder	90Percentile	125.8148457
wavelet-LHH	firstorder	Minimum	-933.8643123
wavelet-LHH	firstorder	Entropy	3.901571656
wavelet-LHH	firstorder	Range	1847.243589
wavelet-LHH	firstorder	Variance	15459.83815
wavelet-LHH	firstorder	10Percentile	-117.2612251
wavelet-LHH	firstorder	Kurtosis	10.06871724
wavelet-LHH	firstorder	Mean	1.129697046
wavelet-LHH	glrlm	ShortRunLowGrayLevelEmphasis	0.000848513
wavelet-LHH	glrlm	GrayLevelVariance	29.14555122
wavelet-LHH	glrlm	LowGrayLevelRunEmphasis	0.000923592
wavelet-LHH	glrlm	GrayLevelNonUniformityNormalized	0.092694094
wavelet-LHH	glrlm	RunVariance	0.330466509

wavelet-LHH	glrlm	GrayLevelNonUniformity	598.390547
wavelet-LHH	glrlm	LongRunEmphasis	1.763014622
wavelet-LHH	glrlm	ShortRunHighGrayLevelEmphasis	1352.595355
wavelet-LHH	glrlm	RunLengthNonUniformity	4864.466407
wavelet-LHH	glrlm	ShortRunEmphasis	0.88978399
wavelet-LHH	glrlm	LongRunHighGrayLevelEmphasis	2646.304108
wavelet-LHH	glrlm	RunPercentage	0.837550213
wavelet-LHH	glrlm	LongRunLowGrayLevelEmphasis	0.001442187
wavelet-LHH	glrlm	RunEntropy	4.774039013
wavelet-LHH	glrlm	HighGrayLevelRunEmphasis	1516.107138
wavelet-LHH	glrlm	RunLengthNonUniformityNormalized	0.753033986
wavelet-LHH	glszm	GrayLevelVariance	66.51211147
wavelet-LHH	glszm	ZoneVariance	2066.074147
wavelet-LHH	glszm	GrayLevelNonUniformityNormalized	0.038457657
wavelet-LHH	glszm	SizeZoneNonUniformityNormalized	0.520196084
wavelet-LHH	glszm	SizeZoneNonUniformity	1194.370209
wavelet-LHH	glszm	GrayLevelNonUniformity	88.29878049
wavelet-LHH	glszm	LargeAreaEmphasis	2077.315331
wavelet-LHH	glszm	SmallAreaHighGrayLevelEmphasis	1190.728432
wavelet-LHH	glszm	ZonePercentage	0.298259288
wavelet-LHH	glszm	LargeAreaLowGrayLevelEmphasis	1.406943349
wavelet-LHH	glszm	LargeAreaHighGrayLevelEmphasis	3071299.307
wavelet-LHH	glszm	HighGrayLevelZoneEmphasis	1568.783537
wavelet-LHH	glszm	SmallAreaEmphasis	0.749210908
wavelet-LHH	glszm	LowGrayLevelZoneEmphasis	0.001346855
wavelet-LHH	glszm	ZoneEntropy	6.421407207
wavelet-LHH	glszm	SmallAreaLowGrayLevelEmphasis	0.001164117
wavelet-LHH	ngtdm	Coarseness	1.11E-03
wavelet-LHH	ngtdm	Complexity	5995.083482
wavelet-LHH	ngtdm	Strength	2.263333426
wavelet-LHH	ngtdm	Contrast	0.037232495
wavelet-LHH	ngtdm	Busyness	0.219026043
wavelet-LLH	gldm	GrayLevelVariance	60.55354346
wavelet-LLH	gldm	HighGrayLevelEmphasis	2211.534554
wavelet-LLH	gldm	DependenceEntropy	7.11710481
wavelet-LLH	gldm	DependenceNonUniformity	963.0259808
wavelet-LLH	gldm	GrayLevelNonUniformity	738.0426085
wavelet-LLH	gldm	SmallDependenceEmphasis	0.310283365
wavelet-LLH	gldm	SmallDependenceHighGrayLevelEmphasis	716.5220236
wavelet-LLH	gldm	DependenceNonUniformityNormalized	0.125100803
wavelet-LLH	gldm	LargeDependenceEmphasis	42.87113536
wavelet-LLH	gldm	LargeDependenceLowGrayLevelEmphasis	0.020121078
wavelet-LLH	gldm	DependenceVariance	18.50104757
wavelet-LLH	gldm	LargeDependenceHighGrayLevelEmphasis	92849.53949
wavelet-LLH	gldm	SmallDependenceLowGrayLevelEmphasis	0.000344627
wavelet-LLH	gldm	LowGrayLevelEmphasis	0.00067798
wavelet-LLH	glcm	JointAverage	46.3700077
wavelet-LLH	glcm	SumAverage	92.7400154
wavelet-LLH	glcm	JointEntropy	7.936622409
wavelet-LLH	glcm	ClusterShade	747.0844838
wavelet-LLH	glcm	MaximumProbability	0.075788369
wavelet-LLH	glcm	Idmn	0.992551621
wavelet-LLH	glcm	JointEnergy	0.02246613
wavelet-LLH	glcm	Contrast	88.38882292
wavelet-LLH	glcm	DifferenceEntropy	3.784135848
wavelet-LLH	glcm	InverseVariance	0.299255248
wavelet-LLH	glcm	DifferenceVariance	60.29854866
wavelet-LLH	glcm	Idn	0.956543756

wavelet-LLH	glcm	Idm	0.337654247
wavelet-LLH	glcm	Correlation	0.169671856
wavelet-LLH	glcm	Autocorrelation	2159.84709
wavelet-LLH	glcm	SumEntropy	4.895745915
wavelet-LLH	glcm	SumSquares	53.86165174
wavelet-LLH	glcm	ClusterProminence	162762.0608
wavelet-LLH	glcm	Imc2	0.816401737
wavelet-LLH	glcm	Imc1	-0.134781355
wavelet-LLH	glcm	DifferenceAverage	5.193022045
wavelet-LLH	glcm	Id	0.399242304
wavelet-LLH	glcm	ClusterTendency	127.057784
wavelet-LLH	firstorder	InterquartileRange	79.80413744
wavelet-LLH	firstorder	Skewness	0.292854303
wavelet-LLH	firstorder	Uniformity	0.095874592
wavelet-LLH	firstorder	Median	-2.149769001
wavelet-LLH	firstorder	Energy	290661553.4
wavelet-LLH	firstorder	RobustMeanAbsoluteDeviation	47.26598023
wavelet-LLH	firstorder	MeanAbsoluteDeviation	112.7199116
wavelet-LLH	firstorder	TotalEnergy	7847861942
wavelet-LLH	firstorder	Maximum	1432.25413
wavelet-LLH	firstorder	RootMeanSquared	194.3143355
wavelet-LLH	firstorder	90Percentile	192.0665481
wavelet-LLH	firstorder	Minimum	-1140.618256
wavelet-LLH	firstorder	Entropy	4.389784336
wavelet-LLH	firstorder	Range	2572.872386
wavelet-LLH	firstorder	Variance	37748.98164
wavelet-LLH	firstorder	10Percentile	-195.6219157
wavelet-LLH	firstorder	Kurtosis	8.750814197
wavelet-LLH	firstorder	Mean	-3.013193874
wavelet-LLH	glrlm	ShortRunLowGrayLevelEmphasis	0.000668018
wavelet-LLH	glrlm	GrayLevelVariance	70.27041301
wavelet-LLH	glrlm	LowGrayLevelRunEmphasis	0.000715678
wavelet-LLH	glrlm	GrayLevelNonUniformityNormalized	0.073550674
wavelet-LLH	glrlm	RunVariance	0.310640548
wavelet-LLH	glrlm	GrayLevelNonUniformity	481.4364581
wavelet-LLH	glrlm	LongRunEmphasis	1.7065877
wavelet-LLH	glrlm	ShortRunHighGrayLevelEmphasis	2001.45863
wavelet-LLH	glrlm	RunLengthNonUniformity	5046.563025
wavelet-LLH	glrlm	ShortRunEmphasis	0.89880636
wavelet-LLH	glrlm	LongRunHighGrayLevelEmphasis	3748.725086
wavelet-LLH	glrlm	RunPercentage	0.848592042
wavelet-LLH	glrlm	LongRunLowGrayLevelEmphasis	0.001046085
wavelet-LLH	glrlm	RunEntropy	5.24134701
wavelet-LLH	glrlm	HighGrayLevelRunEmphasis	2220.335161
wavelet-LLH	glrlm	RunLengthNonUniformityNormalized	0.771022858
wavelet-LLH	glszm	GrayLevelVariance	143.1217106
wavelet-LLH	glszm	ZoneVariance	1499.963984
wavelet-LLH	glszm	GrayLevelNonUniformityNormalized	0.026966484
wavelet-LLH	glszm	SizeZoneNonUniformityNormalized	0.516979653
wavelet-LLH	glszm	SizeZoneNonUniformity	1366.377223
wavelet-LLH	glszm	GrayLevelNonUniformity	71.27241771
wavelet-LLH	glszm	LargeAreaEmphasis	1508.447219
wavelet-LLH	glszm	SmallAreaHighGrayLevelEmphasis	1745.896317
wavelet-LLH	glszm	ZonePercentage	0.343335931
wavelet-LLH	glszm	LargeAreaLowGrayLevelEmphasis	0.698318524
wavelet-LLH	glszm	LargeAreaHighGrayLevelEmphasis	3263590.118
wavelet-LLH	glszm	HighGrayLevelZoneEmphasis	2304.896708
wavelet-LLH	glszm	SmallAreaEmphasis	0.747147271

wavelet-LLH	glszm	LowGrayLevelZoneEmphasis	0.001056722
wavelet-LLH	glszm	ZoneEntropy	6.952220033
wavelet-LLH	glszm	SmallAreaLowGrayLevelEmphasis	0.000924538
wavelet-LLH	ngtdm	Coarseness	0.001024978
wavelet-LLH	ngtdm	Complexity	13293.55174
wavelet-LLH	ngtdm	Strength	4.258988607
wavelet-LLH	ngtdm	Contrast	0.063399314
wavelet-LLH	ngtdm	Busyness	0.149988964
wavelet-LLL	gldm	GrayLevelVariance	856.8802827
wavelet-LLL	gldm	HighGrayLevelEmphasis	8478.047805
wavelet-LLL	gldm	DependenceEntropy	8.158674796
wavelet-LLL	gldm	DependenceNonUniformity	1501.157963
wavelet-LLL	gldm	GrayLevelNonUniformity	278.0698883
wavelet-LLL	gldm	SmallDependenceEmphasis	0.444225671
wavelet-LLL	gldm	SmallDependenceHighGrayLevelEmphasis	2605.229066
wavelet-LLL	gldm	DependenceNonUniformityNormalized	0.195006231
wavelet-LLL	gldm	LargeDependenceEmphasis	29.08469732
wavelet-LLL	gldm	LargeDependenceLowGrayLevelEmphasis	0.003504683
wavelet-LLL	gldm	DependenceVariance	13.89313289
wavelet-LLL	gldm	LargeDependenceHighGrayLevelEmphasis	329967.6293
wavelet-LLL	gldm	SmallDependenceLowGrayLevelEmphasis	0.0006909
wavelet-LLL	gldm	LowGrayLevelEmphasis	0.000796864
wavelet-LLL	glcm	JointAverage	90.93086676
wavelet-LLL	glcm	SumAverage	181.8617335
wavelet-LLL	glcm	JointEntropy	9.908905794
wavelet-LLL	glcm	ClusterShade	-169776.9842
wavelet-LLL	glcm	MaximumProbability	0.03098996
wavelet-LLL	glcm	Idmn	0.982314683
wavelet-LLL	glcm	JointEnergy	0.00600679
wavelet-LLL	glcm	Contrast	379.8501902
wavelet-LLL	glcm	DifferenceEntropy	4.717299103
wavelet-LLL	glcm	InverseVariance	0.238169918
wavelet-LLL	glcm	DifferenceVariance	240.1748401
wavelet-LLL	glcm	Idn	0.931644324
wavelet-LLL	glcm	Idm	0.259058454
wavelet-LLL	glcm	Correlation	0.728465131
wavelet-LLL	glcm	Autocorrelation	8787.645042
wavelet-LLL	glcm	SumEntropy	6.548537394
wavelet-LLL	glcm	SumSquares	708.5255622
wavelet-LLL	glcm	ClusterProminence	23089602.64
wavelet-LLL	glcm	Imc2	0.958593807
wavelet-LLL	glcm	Imc1	-0.229835138
wavelet-LLL	glcm	DifferenceAverage	11.59461568
wavelet-LLL	glcm	Id	0.315418282
wavelet-LLL	glcm	ClusterTendency	2454.252059
wavelet-LLL	firstorder	InterquartileRange	926.6227731
wavelet-LLL	firstorder	Skewness	-1.198671782
wavelet-LLL	firstorder	Uniformity	0.036122355
wavelet-LLL	firstorder	Median	55.19957022
wavelet-LLL	firstorder	Energy	5092796489
wavelet-LLL	firstorder	RobustMeanAbsoluteDeviation	433.2165631
wavelet-LLL	firstorder	MeanAbsoluteDeviation	607.9799138
wavelet-LLL	firstorder	TotalEnergy	1.37506E+11
wavelet-LLL	firstorder	Maximum	932.2823549
wavelet-LLL	firstorder	RootMeanSquared	813.3719806
wavelet-LLL	firstorder	90Percentile	202.4727266
wavelet-LLL	firstorder	Minimum	-2502.326196
wavelet-LLL	firstorder	Entropy	5.825538983

wavelet-LLL	firstorder	Range	3434.608551
wavelet-LLL	firstorder	Variance	535521.9412
wavelet-LLL	firstorder	10Percentile	-1649.322756
wavelet-LLL	firstorder	Kurtosis	3.096177528
wavelet-LLL	firstorder	Mean	-355.0380791
wavelet-LLL	glrlm	ShortRunLowGrayLevelEmphasis	0.000873499
wavelet-LLL	glrlm	GrayLevelVariance	903.894497
wavelet-LLL	glrlm	LowGrayLevelRunEmphasis	0.000882642
wavelet-LLL	glrlm	GrayLevelNonUniformityNormalized	0.028640873
wavelet-LLL	glrlm	RunVariance	0.198474889
wavelet-LLL	glrlm	GrayLevelNonUniformity	196.0586716
wavelet-LLL	glrlm	LongRunEmphasis	1.466986864
wavelet-LLL	glrlm	ShortRunHighGrayLevelEmphasis	7300.671465
wavelet-LLL	glrlm	RunLengthNonUniformity	5658.721656
wavelet-LLL	glrlm	ShortRunEmphasis	0.926069331
wavelet-LLL	glrlm	LongRunHighGrayLevelEmphasis	13408.25886
wavelet-LLL	glrlm	RunPercentage	0.888552471
wavelet-LLL	glrlm	LongRunLowGrayLevelEmphasis	0.000934359
wavelet-LLL	glrlm	RunEntropy	6.479122234
wavelet-LLL	glrlm	HighGrayLevelRunEmphasis	8127.813966
wavelet-LLL	glrlm	RunLengthNonUniformityNormalized	0.826767757
wavelet-LLL	glszm	GrayLevelVariance	959.8638898
wavelet-LLL	glszm	ZoneVariance	306.8301014
wavelet-LLL	glszm	GrayLevelNonUniformityNormalized	0.010219994
wavelet-LLL	glszm	SizeZoneNonUniformityNormalized	0.688717358
wavelet-LLL	glszm	SizeZoneNonUniformity	2491.090683
wavelet-LLL	glszm	GrayLevelNonUniformity	36.96571745
wavelet-LLL	glszm	LargeAreaEmphasis	311.3596904
wavelet-LLL	glszm	SmallAreaHighGrayLevelEmphasis	4687.322115
wavelet-LLL	glszm	ZonePercentage	0.469862302
wavelet-LLL	glszm	LargeAreaLowGrayLevelEmphasis	0.028512291
wavelet-LLL	glszm	LargeAreaHighGrayLevelEmphasis	3612624.744
wavelet-LLL	glszm	HighGrayLevelZoneEmphasis	5847.485762
wavelet-LLL	glszm	SmallAreaEmphasis	0.854454286
wavelet-LLL	glszm	LowGrayLevelZoneEmphasis	0.001510106
wavelet-LLL	glszm	ZoneEntropy	7.576194044
wavelet-LLL	glszm	SmallAreaLowGrayLevelEmphasis	0.001441528
wavelet-LLL	ngtdm	Coarseness	0.001143795
wavelet-LLL	ngtdm	Complexity	53471.03235
wavelet-LLL	ngtdm	Strength	13.52323087
wavelet-LLL	ngtdm	Contrast	0.673993961
wavelet-LLL	ngtdm	Busyness	0.050016156
wavelet-HHL	gldm	GrayLevelVariance	9.237051919
wavelet-HHL	gldm	HighGrayLevelEmphasis	314.5375422
wavelet-HHL	gldm	DependenceEntropy	6.42597965
wavelet-HHL	gldm	DependenceNonUniformity	678.4466095
wavelet-HHL	gldm	GrayLevelNonUniformity	1187.524032
wavelet-HHL	gldm	SmallDependenceEmphasis	0.206367339
wavelet-HHL	gldm	SmallDependenceHighGrayLevelEmphasis	69.71132167
wavelet-HHL	gldm	DependenceNonUniformityNormalized	0.088132841
wavelet-HHL	gldm	LargeDependenceEmphasis	60.47778644
wavelet-HHL	gldm	LargeDependenceLowGrayLevelEmphasis	0.201664377
wavelet-HHL	gldm	DependenceVariance	21.9662333
wavelet-HHL	gldm	LargeDependenceHighGrayLevelEmphasis	18391.21044
wavelet-HHL	gldm	SmallDependenceLowGrayLevelEmphasis	0.001180959
wavelet-HHL	gldm	LowGrayLevelEmphasis	0.003942635
wavelet-HHL	glcm	JointAverage	17.50930741
wavelet-HHL	glcm	SumAverage	35.01861482

wavelet-HHL	glcm	JointEntropy	6.288660596
wavelet-HHL	glcm	ClusterShade	14.14496389
wavelet-HHL	glcm	MaximumProbability	0.110168397
wavelet-HHL	glcm	Idmn	0.989107757
wavelet-HHL	glcm	JointEnergy	0.04191324
wavelet-HHL	glcm	Contrast	17.12233671
wavelet-HHL	glcm	DifferenceEntropy	2.988811741
wavelet-HHL	glcm	InverseVariance	0.361857165
wavelet-HHL	glcm	DifferenceVariance	10.36558798
wavelet-HHL	glcm	Idn	0.941575336
wavelet-HHL	glcm	Idm	0.425315859
wavelet-HHL	glcm	Correlation	-0.00720017
wavelet-HHL	glcm	Autocorrelation	306.5424158
wavelet-HHL	glcm	SumEntropy	3.773536717
wavelet-HHL	glcm	SumSquares	8.527551467
wavelet-HHL	glcm	ClusterProminence	2329.537105
wavelet-HHL	glcm	Imc2	0.602808725
wavelet-HHL	glcm	Imc1	-0.071946328
wavelet-HHL	glcm	DifferenceAverage	2.579131605
wavelet-HHL	glcm	Id	0.482496007
wavelet-HHL	glcm	ClusterTendency	16.98786916
wavelet-HHL	firstorder	InterquartileRange	48.18324309
wavelet-HHL	firstorder	Skewness	0.188081499
wavelet-HHL	firstorder	Uniformity	0.154263969
wavelet-HHL	firstorder	Median	-2.022260204
wavelet-HHL	firstorder	Energy	44170424.12
wavelet-HHL	firstorder	RobustMeanAbsoluteDeviation	23.88684979
wavelet-HHL	firstorder	MeanAbsoluteDeviation	48.13631839
wavelet-HHL	firstorder	TotalEnergy	1192601451
wavelet-HHL	firstorder	Maximum	504.240628
wavelet-HHL	firstorder	RootMeanSquared	75.74898734
wavelet-HHL	firstorder	90Percentile	82.64113885
wavelet-HHL	firstorder	Minimum	-420.8987613
wavelet-HHL	firstorder	Entropy	3.350837817
wavelet-HHL	firstorder	Range	925.1393894
wavelet-HHL	firstorder	Variance	5737.459036
wavelet-HHL	firstorder	10Percentile	-77.03300132
wavelet-HHL	firstorder	Kurtosis	7.298715255
wavelet-HHL	firstorder	Mean	-0.670855608
wavelet-HHL	glrlm	ShortRunLowGrayLevelEmphasis	0.003626798
wavelet-HHL	glrlm	GrayLevelVariance	11.17410242
wavelet-HHL	glrlm	LowGrayLevelRunEmphasis	0.004090437
wavelet-HHL	glrlm	GrayLevelNonUniformityNormalized	0.122841207
wavelet-HHL	glrlm	RunVariance	0.448000781
wavelet-HHL	glrlm	GrayLevelNonUniformity	757.1164709
wavelet-HHL	glrlm	LongRunEmphasis	2.019301671
wavelet-HHL	glrlm	ShortRunHighGrayLevelEmphasis	274.9325098
wavelet-HHL	glrlm	RunLengthNonUniformity	4324.382653
wavelet-HHL	glrlm	ShortRunEmphasis	0.862491904
wavelet-HHL	glrlm	LongRunHighGrayLevelEmphasis	627.3083681
wavelet-HHL	glrlm	RunPercentage	0.799778164
wavelet-HHL	glrlm	LongRunLowGrayLevelEmphasis	0.007501997
wavelet-HHL	glrlm	RunEntropy	4.364405853
wavelet-HHL	glrlm	HighGrayLevelRunEmphasis	316.9531237
wavelet-HHL	glrlm	RunLengthNonUniformityNormalized	0.701297918
wavelet-HHL	glszm	GrayLevelVariance	28.28993078
wavelet-HHL	glszm	ZoneVariance	3965.463926
wavelet-HHL	glszm	GrayLevelNonUniformityNormalized	0.056340963

wavelet-HHL	glszm	SizeZoneNonUniformityNormalized	0.446599348
wavelet-HHL	glszm	SizeZoneNonUniformity	759.6654909
wavelet-HHL	glszm	GrayLevelNonUniformity	95.83597884
wavelet-HHL	glszm	LargeAreaEmphasis	3985.944738
wavelet-HHL	glszm	SmallAreaHighGrayLevelEmphasis	240.0145117
wavelet-HHL	glszm	ZonePercentage	0.220966485
wavelet-HHL	glszm	LargeAreaLowGrayLevelEmphasis	13.18547759
wavelet-HHL	glszm	LargeAreaHighGrayLevelEmphasis	1209673.412
wavelet-HHL	glszm	HighGrayLevelZoneEmphasis	339.7989418
wavelet-HHL	glszm	SmallAreaEmphasis	0.694995664
wavelet-HHL	glszm	LowGrayLevelZoneEmphasis	0.005678029
wavelet-HHL	glszm	ZoneEntropy	6.112004795
wavelet-HHL	glszm	SmallAreaLowGrayLevelEmphasis	0.004457541
wavelet-HHL	ngtdm	Coarseness	1.01E-03
wavelet-HHL	ngtdm	Complexity	1072.507267
wavelet-HHL	ngtdm	Strength	0.517478955
wavelet-HHL	ngtdm	Contrast	0.031311057
wavelet-HHL	ngtdm	Busyness	1.038805684
wavelet-HLH	gldm	GrayLevelVariance	9.111229996
wavelet-HLH	gldm	HighGrayLevelEmphasis	821.0354638
wavelet-HLH	gldm	DependenceEntropy	6.425225075
wavelet-HLH	gldm	DependenceNonUniformity	644.0771629
wavelet-HLH	gldm	GrayLevelNonUniformity	1252.191478
wavelet-HLH	gldm	SmallDependenceEmphasis	0.194160827
wavelet-HLH	gldm	SmallDependenceHighGrayLevelEmphasis	164.8910034
wavelet-HLH	gldm	DependenceNonUniformityNormalized	0.083668117
wavelet-HLH	gldm	LargeDependenceEmphasis	63.87529228
wavelet-HLH	gldm	LargeDependenceLowGrayLevelEmphasis	0.079446268
wavelet-HLH	gldm	DependenceVariance	22.57404929
wavelet-HLH	gldm	LargeDependenceHighGrayLevelEmphasis	51676.13445
wavelet-HLH	gldm	SmallDependenceLowGrayLevelEmphasis	0.000421535
wavelet-HLH	gldm	LowGrayLevelEmphasis	0.001434233
wavelet-HLH	glcm	JointAverage	28.50004271
wavelet-HLH	glcm	SumAverage	57.00008543
wavelet-HLH	glcm	JointEntropy	6.139089189
wavelet-HLH	glcm	ClusterShade	-16.34805084
wavelet-HLH	glcm	MaximumProbability	0.115042708
wavelet-HLH	glcm	Idmn	0.993502468
wavelet-HLH	glcm	JointEnergy	0.045822393
wavelet-HLH	glcm	Contrast	16.72564527
wavelet-HLH	glcm	DifferenceEntropy	2.92675635
wavelet-HLH	glcm	InverseVariance	0.370799361
wavelet-HLH	glcm	DifferenceVariance	10.55241915
wavelet-HLH	glcm	Idn	0.955416554
wavelet-HLH	glcm	Idm	0.439188009
wavelet-HLH	glcm	Correlation	-0.005577866
wavelet-HLH	glcm	Autocorrelation	812.2120896
wavelet-HLH	glcm	SumEntropy	3.706522863
wavelet-HLH	glcm	SumSquares	8.322387853
wavelet-HLH	glcm	ClusterProminence	3297.348831
wavelet-HLH	glcm	Imc2	0.608249674
wavelet-HLH	glcm	Imc1	-0.074914214
wavelet-HLH	glcm	DifferenceAverage	2.464499575
wavelet-HLH	glcm	Id	0.494298601
wavelet-HLH	glcm	ClusterTendency	16.56390615
wavelet-HLH	firstorder	InterquartileRange	45.0155171
wavelet-HLH	firstorder	Skewness	-0.175493747
wavelet-HLH	firstorder	Uniformity	0.16266452

wavelet-HLH	firstorder	Median	-1.106514976
wavelet-HLH	firstorder	Energy	43383150.4
wavelet-HLH	firstorder	RobustMeanAbsoluteDeviation	22.34278694
wavelet-HLH	firstorder	MeanAbsoluteDeviation	46.04841644
wavelet-HLH	firstorder	TotalEnergy	1171345061
wavelet-HLH	firstorder	Maximum	505.9433053
wavelet-HLH	firstorder	RootMeanSquared	75.07089435
wavelet-HLH	firstorder	90Percentile	77.55300376
wavelet-HLH	firstorder	Minimum	-676.9170504
wavelet-HLH	firstorder	Entropy	3.288085905
wavelet-HLH	firstorder	Range	1182.860356
wavelet-HLH	firstorder	Variance	5635.61734
wavelet-HLH	firstorder	10Percentile	-72.5980977
wavelet-HLH	firstorder	Kurtosis	10.65224677
wavelet-HLH	firstorder	Mean	-0.147781896
wavelet-HLH	glrlm	ShortRunLowGrayLevelEmphasis	0.001308168
wavelet-HLH	glrlm	GrayLevelVariance	11.136938
wavelet-HLH	glrlm	LowGrayLevelRunEmphasis	0.001484816
wavelet-HLH	glrlm	GrayLevelNonUniformityNormalized	0.129038859
wavelet-HLH	glrlm	RunVariance	0.48138494
wavelet-HLH	glrlm	GrayLevelNonUniformity	786.7248177
wavelet-HLH	glrlm	LongRunEmphasis	2.085856794
wavelet-HLH	glrlm	ShortRunHighGrayLevelEmphasis	708.8213729
wavelet-HLH	glrlm	RunLengthNonUniformity	4231.038871
wavelet-HLH	glrlm	ShortRunEmphasis	0.858130723
wavelet-HLH	glrlm	LongRunHighGrayLevelEmphasis	1702.879035
wavelet-HLH	glrlm	RunPercentage	0.79128445
wavelet-HLH	glrlm	LongRunLowGrayLevelEmphasis	0.002834657
wavelet-HLH	glrlm	RunEntropy	4.342351448
wavelet-HLH	glrlm	HighGrayLevelRunEmphasis	823.9011223
wavelet-HLH	glrlm	RunLengthNonUniformityNormalized	0.693607203
wavelet-HLH	glszm	GrayLevelVariance	30.25983603
wavelet-HLH	glszm	ZoneVariance	4818.299745
wavelet-HLH	glszm	GrayLevelNonUniformityNormalized	0.058117805
wavelet-HLH	glszm	SizeZoneNonUniformityNormalized	0.442877369
wavelet-HLH	glszm	SizeZoneNonUniformity	700.1891208
wavelet-HLH	glszm	GrayLevelNonUniformity	91.88425047
wavelet-HLH	glszm	LargeAreaEmphasis	4842.00759
wavelet-HLH	glszm	SmallAreaHighGrayLevelEmphasis	595.0297248
wavelet-HLH	glszm	ZonePercentage	0.20537802
wavelet-HLH	glszm	LargeAreaLowGrayLevelEmphasis	5.987720018
wavelet-HLH	glszm	LargeAreaHighGrayLevelEmphasis	3921503.258
wavelet-HLH	glszm	HighGrayLevelZoneEmphasis	850.6963947
wavelet-HLH	glszm	SmallAreaEmphasis	0.692150103
wavelet-HLH	glszm	LowGrayLevelZoneEmphasis	0.002133731
wavelet-HLH	glszm	ZoneEntropy	6.142867706
wavelet-HLH	glszm	SmallAreaLowGrayLevelEmphasis	0.001727297
wavelet-HLH	ngtdm	Coarseness	1.01E-03
wavelet-HLH	ngtdm	Complexity	1630.963115
wavelet-HLH	ngtdm	Strength	1.031134647
wavelet-HLH	ngtdm	Contrast	0.020610683
wavelet-HLH	ngtdm	Busyness	0.50180798

Image type	Feature Class	Feature Name	4: RTSTRUCT: C99-DNU_segment_LLL GTV
general	info	BoundingBox	(0, 1, 1, 12, 8, 12)
general	info	EnabledImageTypes	{u'Original': {}, u'Wavelet': {}}
general	info	GeneralSettings	{'distances': [1], 'additionalInfo': True, 'force2D': False, 'interpolator': 'sitkBSpline', 'resampledPixelSpacing': [3.0], 'label': 1, 'normalizeScale': 1, 'normalize': False, 'force2Ddimension': 0, 'removeOutliers': None, 'minimumROISize': None, u'binWidth': 25.0, 'minimumROIDimensions': 1, u'correctMask': True, 'preCrop': False, u'symmetricalGLCM': True, 'resegmentRange': None, 'padDistance': 5}
general	info	ImageHash	81bcdd5954e665c14134ddfe97a0ae7d55f40743
general	info	ImageSpacing	(0.9765625000000002, 0.9765625000000002, 2.0)
general	info	MaskHash	e1fc0199032d4204995cab73655c8bff6f1ef1ff
general	info	NumpyVersion	1.13.1
general	info	PyWaveletVersion	0.5.2
general	info	SimpleITKVersion	1.1.0
general	info	Version	1.3.0.post102+g1b0eb74
general	info	VolumeNum	1
general	info	VoxelNum	445
original	shape	Maximum3DDiameter	39.57271787
original	shape	Maximum2DDiameterSlice	34.98571137
original	shape	Sphericity	0.740832595
original	shape	MinorAxis	30.04289044
original	shape	Elongation	0.834303214
original	shape	SurfaceVolumeRatio	0.285007388
original	shape	Volume	12015
original	shape	MajorAxis	36.00955856
original	shape	SurfaceArea	3424.363764
original	shape	Flatness	0.501833748
original	shape	LeastAxis	18.07081175
original	shape	Maximum2DDiameterColumn	34.20526275
original	shape	Maximum2DDiameterRow	37.58989226
original	gldm	GrayLevelVariance	179.4656432
original	gldm	HighGrayLevelEmphasis	498.1483146
original	gldm	DependenceEntropy	6.575614146
original	gldm	DependenceNonUniformity	130.105618
original	gldm	GrayLevelNonUniformity	16.86966292
original	gldm	SmallDependenceEmphasis	0.506474995
original	gldm	SmallDependenceHighGrayLevelEmphasis	265.829494
original	gldm	DependenceNonUniformityNormalized	0.292372175
original	gldm	LargeDependenceEmphasis	7.269662921
original	gldm	LargeDependenceLowGrayLevelEmphasis	0.250116752
original	gldm	DependenceVariance	2.469132685
original	gldm	LargeDependenceHighGrayLevelEmphasis	2769.680899
original	gldm	SmallDependenceLowGrayLevelEmphasis	0.012745564
original	gldm	LowGrayLevelEmphasis	0.028733258
original	glcm	JointAverage	19.28086632
original	glcm	SumAverage	38.56173263

original	glcm	JointEntropy	8.519128367
original	glcm	ClusterShade	5032.305256
original	glcm	MaximumProbability	0.014193365
original	glcm	Idmn	0.916850734
original	glcm	JointEnergy	0.003506903
original	glcm	Contrast	216.152062
original	glcm	DifferenceEntropy	4.726138627
original	glcm	InverseVariance	0.152813635
original	glcm	DifferenceVariance	100.6975824
original	glcm	Idn	0.830031887
original	glcm	Idm	0.153107947
original	glcm	Correlation	0.431115198
original	glcm	Autocorrelation	453.3934117
original	glcm	SumEntropy	6.025104785
original	glcm	SumSquares	189.6458674
original	glcm	ClusterProminence	558003.9655
original	glcm	Imc2	0.980277535
original	glcm	Imc1	-0.320095778
original	glcm	DifferenceAverage	10.64050471
original	glcm	Id	0.225075632
original	glcm	ClusterTendency	542.4314075
original	firstorder	InterquartileRange	561
original	firstorder	Skewness	0.637468678
original	firstorder	Uniformity	0.037909355
original	firstorder	Median	-716
original	firstorder	Energy	193079199
original	firstorder	RobustMeanAbsoluteDeviation	240.3412656
original	firstorder	MeanAbsoluteDeviation	295.6017978
original	firstorder	TotalEnergy	5213138373
original	firstorder	Maximum	87
original	firstorder	RootMeanSquared	658.7001142
original	firstorder	90Percentile	-4.4
original	firstorder	Minimum	-987
original	firstorder	Entropy	5.04680003
original	firstorder	Range	1074
original	firstorder	Variance	111973.6811
original	firstorder	10Percentile	-899.6
original	firstorder	Kurtosis	1.920649991
original	firstorder	Mean	-567.3730337
original	glrlm	ShortRunLowGrayLevelEmphasis	0.027358637
original	glrlm	GrayLevelVariance	177.8544386
original	glrlm	LowGrayLevelRunEmphasis	0.028506659
original	glrlm	GrayLevelNonUniformityNormalized	0.036773559
original	glrlm	RunVariance	0.051870064
original	glrlm	GrayLevelNonUniformity	15.61609201
original	glrlm	LongRunEmphasis	1.151088132
original	glrlm	ShortRunHighGrayLevelEmphasis	487.0431997
original	glrlm	RunLengthNonUniformity	388.2654641
original	glrlm	ShortRunEmphasis	0.965679274
original	glrlm	LongRunHighGrayLevelEmphasis	564.6098787
original	glrlm	RunPercentage	0.954191876
original	glrlm	LongRunLowGrayLevelEmphasis	0.033554135
original	glrlm	RunEntropy	5.280426595
original	glrlm	HighGrayLevelRunEmphasis	501.8251518
original	glrlm	RunLengthNonUniformityNormalized	0.913925612

original	glszm	GrayLevelVariance	160.0313082
original	glszm	ZoneVariance	3.214966048
original	glszm	GrayLevelNonUniformityNormalized	0.028453819
original	glszm	SizeZoneNonUniformityNormalized	0.521453038
original	glszm	SizeZoneNonUniformity	134.5348837
original	glszm	GrayLevelNonUniformity	7.341085271
original	glszm	LargeAreaEmphasis	6.189922481
original	glszm	SmallAreaHighGrayLevelEmphasis	399.9298661
original	glszm	ZonePercentage	0.579775281
original	glszm	LargeAreaLowGrayLevelEmphasis	0.188492676
original	glszm	LargeAreaHighGrayLevelEmphasis	2422.023256
original	glszm	HighGrayLevelZoneEmphasis	531.1162791
original	glszm	SmallAreaEmphasis	0.74989895
original	glszm	LowGrayLevelZoneEmphasis	0.026075602
original	glszm	ZoneEntropy	6.227957831
original	glszm	SmallAreaLowGrayLevelEmphasis	0.017748835
original	ngtdm	Coarseness	0.008780493
original	ngtdm	Complexity	6370.407487
original	ngtdm	Strength	9.841278052
original	ngtdm	Contrast	1.381200137
original	ngtdm	Busyness	0.178666461
wavelet-HHH	gldm	GrayLevelVariance	16.77538695
wavelet-HHH	gldm	HighGrayLevelEmphasis	289.7303371
wavelet-HHH	gldm	DependenceEntropy	5.85617478
wavelet-HHH	gldm	DependenceNonUniformity	103.8719101
wavelet-HHH	gldm	GrayLevelNonUniformity	35.3505618
wavelet-HHH	gldm	SmallDependenceEmphasis	0.419297871
wavelet-HHH	gldm	SmallDependenceHighGrayLevelEmphasis	123.1720134
wavelet-HHH	gldm	DependenceNonUniformityNormalized	0.233420023
wavelet-HHH	gldm	LargeDependenceEmphasis	7.907865169
wavelet-HHH	gldm	LargeDependenceLowGrayLevelEmphasis	0.034440631
wavelet-HHH	gldm	DependenceVariance	1.96303497
wavelet-HHH	gldm	LargeDependenceHighGrayLevelEmphasis	2174.579775
wavelet-HHH	gldm	SmallDependenceLowGrayLevelEmphasis	0.004736808
wavelet-HHH	gldm	LowGrayLevelEmphasis	0.007001386
wavelet-HHH	glcm	JointAverage	16.5081808
wavelet-HHH	glcm	SumAverage	33.0163616
wavelet-HHH	glcm	JointEntropy	7.457804467
wavelet-HHH	glcm	ClusterShade	7.184006502
wavelet-HHH	glcm	MaximumProbability	0.023160528
wavelet-HHH	glcm	Idmn	0.964672946
wavelet-HHH	glcm	JointEnergy	0.007938502
wavelet-HHH	glcm	Contrast	36.37029236
wavelet-HHH	glcm	DifferenceEntropy	3.619470709
wavelet-HHH	glcm	InverseVariance	0.227362304
wavelet-HHH	glcm	DifferenceVariance	15.19620148
wavelet-HHH	glcm	Idn	0.878208629
wavelet-HHH	glcm	Idm	0.212190442
wavelet-HHH	glcm	Correlation	-0.039411484
wavelet-HHH	glcm	Autocorrelation	271.8250601
wavelet-HHH	glcm	SumEntropy	4.442944162
wavelet-HHH	glcm	SumSquares	17.48804903
wavelet-HHH	glcm	ClusterProminence	4490.653928
wavelet-HHH	glcm	Imc2	0.839229836
wavelet-HHH	glcm	Imc1	-0.151503436

wavelet-HHH	glcm	DifferenceAverage	4.558419335
wavelet-HHH	glcm	Id	0.302274753
wavelet-HHH	glcm	ClusterTendency	33.58190375
wavelet-HHH	firstorder	InterquartileRange	109.1906251
wavelet-HHH	firstorder	Skewness	0.037360773
wavelet-HHH	firstorder	Uniformity	0.079439465
wavelet-HHH	firstorder	Median	-2.62725605
wavelet-HHH	firstorder	Energy	4597948.802
wavelet-HHH	firstorder	RobustMeanAbsoluteDeviation	48.38712642
wavelet-HHH	firstorder	MeanAbsoluteDeviation	76.2352387
wavelet-HHH	firstorder	TotalEnergy	124144617.7
wavelet-HHH	firstorder	Maximum	341.8628868
wavelet-HHH	firstorder	RootMeanSquared	101.6487541
wavelet-HHH	firstorder	90Percentile	136.3812461
wavelet-HHH	firstorder	Minimum	-398.3740039
wavelet-HHH	firstorder	Entropy	3.993611385
wavelet-HHH	firstorder	Range	740.2368907
wavelet-HHH	firstorder	Variance	10331.56023
wavelet-HHH	firstorder	10Percentile	-122.0733506
wavelet-HHH	firstorder	Kurtosis	3.928967658
wavelet-HHH	firstorder	Mean	0.953408744
wavelet-HHH	glrlm	ShortRunLowGrayLevelEmphasis	0.007019594
wavelet-HHH	glrlm	GrayLevelVariance	17.40256795
wavelet-HHH	glrlm	LowGrayLevelRunEmphasis	0.00718136
wavelet-HHH	glrlm	GrayLevelNonUniformityNormalized	0.077162376
wavelet-HHH	glrlm	RunVariance	0.066139839
wavelet-HHH	glrlm	GrayLevelNonUniformity	32.44233288
wavelet-HHH	glrlm	LongRunEmphasis	1.187785171
wavelet-HHH	glrlm	ShortRunHighGrayLevelEmphasis	279.0099367
wavelet-HHH	glrlm	RunLengthNonUniformity	377.9305539
wavelet-HHH	glrlm	ShortRunEmphasis	0.959012195
wavelet-HHH	glrlm	LongRunHighGrayLevelEmphasis	342.5485588
wavelet-HHH	glrlm	RunPercentage	0.944684529
wavelet-HHH	glrlm	LongRunLowGrayLevelEmphasis	0.007922768
wavelet-HHH	glrlm	RunEntropy	4.292594312
wavelet-HHH	glrlm	HighGrayLevelRunEmphasis	290.4185289
wavelet-HHH	glrlm	RunLengthNonUniformityNormalized	0.898460907
wavelet-HHH	glszm	GrayLevelVariance	27.89849133
wavelet-HHH	glszm	ZoneVariance	9.643597903
wavelet-HHH	glszm	GrayLevelNonUniformityNormalized	0.053913601
wavelet-HHH	glszm	SizeZoneNonUniformityNormalized	0.486550216
wavelet-HHH	glszm	SizeZoneNonUniformity	101.6889952
wavelet-HHH	glszm	GrayLevelNonUniformity	11.26794258
wavelet-HHH	glszm	LargeAreaEmphasis	14.17703349
wavelet-HHH	glszm	SmallAreaHighGrayLevelEmphasis	212.705163
wavelet-HHH	glszm	ZonePercentage	0.469662921
wavelet-HHH	glszm	LargeAreaLowGrayLevelEmphasis	0.061608038
wavelet-HHH	glszm	LargeAreaHighGrayLevelEmphasis	3913.023923
wavelet-HHH	glszm	HighGrayLevelZoneEmphasis	297.3923445
wavelet-HHH	glszm	SmallAreaEmphasis	0.723870503
wavelet-HHH	glszm	LowGrayLevelZoneEmphasis	0.010489738
wavelet-HHH	glszm	ZoneEntropy	5.593451667
wavelet-HHH	glszm	SmallAreaLowGrayLevelEmphasis	0.009436062
wavelet-HHH	ngtdm	Coarseness	0.014847354
wavelet-HHH	ngtdm	Complexity	849.5562278

wavelet-HHH	ngtdm	Strength	2.851985599
wavelet-HHH	ngtdm	Contrast	0.167469224
wavelet-HHH	ngtdm	Busyness	0.142692346
wavelet-HLL	gldm	GrayLevelVariance	103.1334629
wavelet-HLL	gldm	HighGrayLevelEmphasis	1342.489888
wavelet-HLL	gldm	DependenceEntropy	6.480030168
wavelet-HLL	gldm	DependenceNonUniformity	176.1325843
wavelet-HLL	gldm	GrayLevelNonUniformity	14.28089888
wavelet-HLL	gldm	SmallDependenceEmphasis	0.633674157
wavelet-HLL	gldm	SmallDependenceHighGrayLevelEmphasis	848.2240462
wavelet-HLL	gldm	DependenceNonUniformityNormalized	0.39580356
wavelet-HLL	gldm	LargeDependenceEmphasis	3.77752809
wavelet-HLL	gldm	LargeDependenceLowGrayLevelEmphasis	0.006223982
wavelet-HLL	gldm	DependenceVariance	0.898977402
wavelet-HLL	gldm	LargeDependenceHighGrayLevelEmphasis	5089.438202
wavelet-HLL	gldm	SmallDependenceLowGrayLevelEmphasis	0.00322849
wavelet-HLL	gldm	LowGrayLevelEmphasis	0.003604343
wavelet-HLL	glcm	JointAverage	34.62037852
wavelet-HLL	glcm	SumAverage	69.24075705
wavelet-HLL	glcm	JointEntropy	8.731412941
wavelet-HLL	glcm	ClusterShade	-2241.367804
wavelet-HLL	glcm	MaximumProbability	0.009799693
wavelet-HLL	glcm	Idmn	0.956998728
wavelet-HLL	glcm	JointEnergy	0.002727529
wavelet-HLL	glcm	Contrast	164.8054463
wavelet-HLL	glcm	DifferenceEntropy	4.580393757
wavelet-HLL	glcm	InverseVariance	0.115714754
wavelet-HLL	glcm	DifferenceVariance	66.8412208
wavelet-HLL	glcm	Idn	0.866794535
wavelet-HLL	glcm	Idm	0.109372223
wavelet-HLL	glcm	Correlation	0.245170186
wavelet-HLL	glcm	Autocorrelation	1225.206742
wavelet-HLL	glcm	SumEntropy	5.811968548
wavelet-HLL	glcm	SumSquares	108.9863226
wavelet-HLL	glcm	ClusterProminence	220494.9899
wavelet-HLL	glcm	Imc2	0.98511789
wavelet-HLL	glcm	Imc1	-0.336318671
wavelet-HLL	glcm	DifferenceAverage	9.68533314
wavelet-HLL	glcm	Id	0.19168239
wavelet-HLL	glcm	ClusterTendency	271.1398443
wavelet-HLL	firstorder	InterquartileRange	340.8335165
wavelet-HLL	firstorder	Skewness	-0.57854514
wavelet-HLL	firstorder	Uniformity	0.032091908
wavelet-HLL	firstorder	Median	15.55491316
wavelet-HLL	firstorder	Energy	29018305.09
wavelet-HLL	firstorder	RobustMeanAbsoluteDeviation	141.2095162
wavelet-HLL	firstorder	MeanAbsoluteDeviation	203.3518564
wavelet-HLL	firstorder	TotalEnergy	783494237.5
wavelet-HLL	firstorder	Maximum	516.204376
wavelet-HLL	firstorder	RootMeanSquared	255.3618499
wavelet-HLL	firstorder	90Percentile	249.0811438
wavelet-HLL	firstorder	Minimum	-898.6488146
wavelet-HLL	firstorder	Entropy	5.231746305
wavelet-HLL	firstorder	Range	1414.853191
wavelet-HLL	firstorder	Variance	64197.42984

wavelet-HLL	firstorder	10Percentile	-401.2369377
wavelet-HLL	firstorder	Kurtosis	3.056377134
wavelet-HLL	firstorder	Mean	-31.8157906
wavelet-HLL	glrlm	ShortRunLowGrayLevelEmphasis	0.003657434
wavelet-HLL	glrlm	GrayLevelVariance	104.2110945
wavelet-HLL	glrlm	LowGrayLevelRunEmphasis	0.003677064
wavelet-HLL	glrlm	GrayLevelNonUniformityNormalized	0.031848222
wavelet-HLL	glrlm	RunVariance	0.028575152
wavelet-HLL	glrlm	GrayLevelNonUniformity	13.79261358
wavelet-HLL	glrlm	LongRunEmphasis	1.084735246
wavelet-HLL	glrlm	ShortRunHighGrayLevelEmphasis	1314.82586
wavelet-HLL	glrlm	RunLengthNonUniformity	410.6539739
wavelet-HLL	glrlm	ShortRunEmphasis	0.979823238
wavelet-HLL	glrlm	LongRunHighGrayLevelEmphasis	1456.598633
wavelet-HLL	glrlm	RunPercentage	0.973206569
wavelet-HLL	glrlm	LongRunLowGrayLevelEmphasis	0.003759582
wavelet-HLL	glrlm	RunEntropy	5.363921542
wavelet-HLL	glrlm	HighGrayLevelRunEmphasis	1342.199919
wavelet-HLL	glrlm	RunLengthNonUniformityNormalized	0.948051145
wavelet-HLL	glszm	GrayLevelVariance	117.3260195
wavelet-HLL	glszm	ZoneVariance	0.937780404
wavelet-HLL	glszm	GrayLevelNonUniformityNormalized	0.028881589
wavelet-HLL	glszm	SizeZoneNonUniformityNormalized	0.616227367
wavelet-HLL	glszm	SizeZoneNonUniformity	194.7278481
wavelet-HLL	glszm	GrayLevelNonUniformity	9.126582278
wavelet-HLL	glszm	LargeAreaEmphasis	2.920886076
wavelet-HLL	glszm	SmallAreaHighGrayLevelEmphasis	1086.587569
wavelet-HLL	glszm	ZonePercentage	0.71011236
wavelet-HLL	glszm	LargeAreaLowGrayLevelEmphasis	0.006465167
wavelet-HLL	glszm	LargeAreaHighGrayLevelEmphasis	3949.949367
wavelet-HLL	glszm	HighGrayLevelZoneEmphasis	1336.794304
wavelet-HLL	glszm	SmallAreaEmphasis	0.812398856
wavelet-HLL	glszm	LowGrayLevelZoneEmphasis	0.004669007
wavelet-HLL	glszm	ZoneEntropy	6.161564271
wavelet-HLL	glszm	SmallAreaLowGrayLevelEmphasis	0.004462968
wavelet-HLL	ngtdm	Coarseness	0.014857714
wavelet-HLL	ngtdm	Complexity	6840.006059
wavelet-HLL	ngtdm	Strength	13.77056479
wavelet-HLL	ngtdm	Contrast	0.469848245
wavelet-HLL	ngtdm	Busyness	0.034134579
wavelet-LHL	gldm	GrayLevelVariance	137.1975559
wavelet-LHL	gldm	HighGrayLevelEmphasis	2332.067416
wavelet-LHL	gldm	DependenceEntropy	6.588277018
wavelet-LHL	gldm	DependenceNonUniformity	172.6179775
wavelet-LHL	gldm	GrayLevelNonUniformity	14.6988764
wavelet-LHL	gldm	SmallDependenceEmphasis	0.628966254
wavelet-LHL	gldm	SmallDependenceHighGrayLevelEmphasis	1451.144833
wavelet-LHL	gldm	DependenceNonUniformityNormalized	0.387905567
wavelet-LHL	gldm	LargeDependenceEmphasis	4.195505618
wavelet-LHL	gldm	LargeDependenceLowGrayLevelEmphasis	0.004322467
wavelet-LHL	gldm	DependenceVariance	1.146754198
wavelet-LHL	gldm	LargeDependenceHighGrayLevelEmphasis	10099.70337
wavelet-LHL	gldm	SmallDependenceLowGrayLevelEmphasis	0.002720638
wavelet-LHL	gldm	LowGrayLevelEmphasis	0.002899041
wavelet-LHL	glcm	JointAverage	46.65792807

wavelet-LHL	glcm	SumAverage	93.31585614
wavelet-LHL	glcm	JointEntropy	8.773653235
wavelet-LHL	glcm	ClusterShade	-4290.802302
wavelet-LHL	glcm	MaximumProbability	0.010119346
wavelet-LHL	glcm	Idmn	0.963819095
wavelet-LHL	glcm	JointEnergy	0.002691244
wavelet-LHL	glcm	Contrast	245.4068371
wavelet-LHL	glcm	DifferenceEntropy	4.800301529
wavelet-LHL	glcm	InverseVariance	0.113284107
wavelet-LHL	glcm	DifferenceVariance	111.5484261
wavelet-LHL	glcm	Idn	0.882359129
wavelet-LHL	glcm	Idm	0.110346584
wavelet-LHL	glcm	Correlation	0.183706157
wavelet-LHL	glcm	Autocorrelation	2205.115816
wavelet-LHL	glcm	SumEntropy	5.931672847
wavelet-LHL	glcm	SumSquares	150.8135401
wavelet-LHL	glcm	ClusterProminence	507485.557
wavelet-LHL	glcm	Imc2	0.991815419
wavelet-LHL	glcm	Imc1	-0.381252653
wavelet-LHL	glcm	DifferenceAverage	11.28872716
wavelet-LHL	glcm	Id	0.18884282
wavelet-LHL	glcm	ClusterTendency	357.8473233
wavelet-LHL	firstorder	InterquartileRange	294.1621331
wavelet-LHL	firstorder	Skewness	-0.580415765
wavelet-LHL	firstorder	Uniformity	0.033031183
wavelet-LHL	firstorder	Median	-4.606720433
wavelet-LHL	firstorder	Energy	38821394.64
wavelet-LHL	firstorder	RobustMeanAbsoluteDeviation	128.4395777
wavelet-LHL	firstorder	MeanAbsoluteDeviation	214.6142021
wavelet-LHL	firstorder	TotalEnergy	1048177655
wavelet-LHL	firstorder	Maximum	683.8044161
wavelet-LHL	firstorder	RootMeanSquared	295.3626399
wavelet-LHL	firstorder	90Percentile	299.0458942
wavelet-LHL	firstorder	Minimum	-1181.288791
wavelet-LHL	firstorder	Entropy	5.356970511
wavelet-LHL	firstorder	Range	1865.093207
wavelet-LHL	firstorder	Variance	85570.75752
wavelet-LHL	firstorder	10Percentile	-427.0390779
wavelet-LHL	firstorder	Kurtosis	4.017226492
wavelet-LHL	firstorder	Mean	-40.84521457
wavelet-LHL	glrlm	ShortRunLowGrayLevelEmphasis	0.002961583
wavelet-LHL	glrlm	GrayLevelVariance	139.8585586
wavelet-LHL	glrlm	LowGrayLevelRunEmphasis	0.002971583
wavelet-LHL	glrlm	GrayLevelNonUniformityNormalized	0.032276755
wavelet-LHL	glrlm	RunVariance	0.030459364
wavelet-LHL	glrlm	GrayLevelNonUniformity	13.9516246
wavelet-LHL	glrlm	LongRunEmphasis	1.090977532
wavelet-LHL	glrlm	ShortRunHighGrayLevelEmphasis	2277.152844
wavelet-LHL	glrlm	RunLengthNonUniformity	408.2739958
wavelet-LHL	glrlm	ShortRunEmphasis	0.978261552
wavelet-LHL	glrlm	LongRunHighGrayLevelEmphasis	2549.634454
wavelet-LHL	glrlm	RunPercentage	0.971305099
wavelet-LHL	glrlm	LongRunLowGrayLevelEmphasis	0.003013165
wavelet-LHL	glrlm	RunEntropy	5.514950699
wavelet-LHL	glrlm	HighGrayLevelRunEmphasis	2329.581534

wavelet-LHL	glrlm	RunLengthNonUniformityNormalized	0.944271262
wavelet-LHL	glszm	GrayLevelVariance	172.2171912
wavelet-LHL	glszm	ZoneVariance	1.349304372
wavelet-LHL	glszm	GrayLevelNonUniformityNormalized	0.025405996
wavelet-LHL	glszm	SizeZoneNonUniformityNormalized	0.619981831
wavelet-LHL	glszm	SizeZoneNonUniformity	194.0543131
wavelet-LHL	glszm	GrayLevelNonUniformity	7.952076677
wavelet-LHL	glszm	LargeAreaEmphasis	3.370607029
wavelet-LHL	glszm	SmallAreaHighGrayLevelEmphasis	1876.379335
wavelet-LHL	glszm	ZonePercentage	0.703370787
wavelet-LHL	glszm	LargeAreaLowGrayLevelEmphasis	0.004971277
wavelet-LHL	glszm	LargeAreaHighGrayLevelEmphasis	8093.214058
wavelet-LHL	glszm	HighGrayLevelZoneEmphasis	2304.709265
wavelet-LHL	glszm	SmallAreaEmphasis	0.815025339
wavelet-LHL	glszm	LowGrayLevelZoneEmphasis	0.003923193
wavelet-LHL	glszm	ZoneEntropy	6.376114853
wavelet-LHL	glszm	SmallAreaLowGrayLevelEmphasis	0.003829103
wavelet-LHL	ngtdm	Coarseness	0.014137543
wavelet-LHL	ngtdm	Complexity	12093.83842
wavelet-LHL	ngtdm	Strength	20.15833999
wavelet-LHL	ngtdm	Contrast	0.51684565
wavelet-LHL	ngtdm	Busyness	0.021213821
wavelet-LHH	gldm	GrayLevelVariance	34.56631233
wavelet-LHH	gldm	HighGrayLevelEmphasis	494.5483146
wavelet-LHH	gldm	DependenceEntropy	6.063954523
wavelet-LHH	gldm	DependenceNonUniformity	127.7370787
wavelet-LHH	gldm	GrayLevelNonUniformity	25.59775281
wavelet-LHH	gldm	SmallDependenceEmphasis	0.503803957
wavelet-LHH	gldm	SmallDependenceHighGrayLevelEmphasis	261.2907147
wavelet-LHH	gldm	DependenceNonUniformityNormalized	0.287049615
wavelet-LHH	gldm	LargeDependenceEmphasis	5.898876404
wavelet-LHH	gldm	LargeDependenceLowGrayLevelEmphasis	0.017355769
wavelet-LHH	gldm	DependenceVariance	1.484156041
wavelet-LHH	gldm	LargeDependenceHighGrayLevelEmphasis	2739.878652
wavelet-LHH	gldm	SmallDependenceLowGrayLevelEmphasis	0.004374152
wavelet-LHH	gldm	LowGrayLevelEmphasis	0.005610097
wavelet-LHH	glcm	JointAverage	21.47621428
wavelet-LHH	glcm	SumAverage	42.95242856
wavelet-LHH	glcm	JointEntropy	7.98278869
wavelet-LHH	glcm	ClusterShade	73.26170214
wavelet-LHH	glcm	MaximumProbability	0.017275035
wavelet-LHH	glcm	Idmn	0.96989212
wavelet-LHH	glcm	JointEnergy	0.005136904
wavelet-LHH	glcm	Contrast	68.99278837
wavelet-LHH	glcm	DifferenceEntropy	4.02225299
wavelet-LHH	glcm	InverseVariance	0.172791492
wavelet-LHH	glcm	DifferenceVariance	29.59179591
wavelet-LHH	glcm	Idn	0.88792826
wavelet-LHH	glcm	Idm	0.165487446
wavelet-LHH	glcm	Correlation	-0.034563086
wavelet-LHH	glcm	Autocorrelation	460.0617731
wavelet-LHH	glcm	SumEntropy	4.874679562
wavelet-LHH	glcm	SumSquares	33.32620405
wavelet-LHH	glcm	ClusterProminence	17647.42119
wavelet-LHH	glcm	Imc2	0.914415121

wavelet-LHH	glcm	Imc1	-0.204268405
wavelet-LHH	glcm	DifferenceAverage	6.202710119
wavelet-LHH	glcm	Id	0.255399065
wavelet-LHH	glcm	ClusterTendency	64.31202783
wavelet-LHH	firstorder	InterquartileRange	159.9308654
wavelet-LHH	firstorder	Skewness	0.226171186
wavelet-LHH	firstorder	Uniformity	0.05752304
wavelet-LHH	firstorder	Median	-8.790470334
wavelet-LHH	firstorder	Energy	9585139.435
wavelet-LHH	firstorder	RobustMeanAbsoluteDeviation	67.76576773
wavelet-LHH	firstorder	MeanAbsoluteDeviation	108.4854119
wavelet-LHH	firstorder	TotalEnergy	258798764.7
wavelet-LHH	firstorder	Maximum	594.5229214
wavelet-LHH	firstorder	RootMeanSquared	146.7638892
wavelet-LHH	firstorder	90Percentile	187.8952285
wavelet-LHH	firstorder	Minimum	-502.0820134
wavelet-LHH	firstorder	Entropy	4.463513729
wavelet-LHH	firstorder	Range	1096.604935
wavelet-LHH	firstorder	Variance	21538.7605
wavelet-LHH	firstorder	10Percentile	-176.8414271
wavelet-LHH	firstorder	Kurtosis	4.29183987
wavelet-LHH	firstorder	Mean	-0.937380474
wavelet-LHH	glrlm	ShortRunLowGrayLevelEmphasis	0.005673445
wavelet-LHH	glrlm	GrayLevelVariance	35.6139315
wavelet-LHH	glrlm	LowGrayLevelRunEmphasis	0.005751708
wavelet-LHH	glrlm	GrayLevelNonUniformityNormalized	0.056044659
wavelet-LHH	glrlm	RunVariance	0.046234044
wavelet-LHH	glrlm	GrayLevelNonUniformity	23.8873039
wavelet-LHH	glrlm	LongRunEmphasis	1.137356529
wavelet-LHH	glrlm	ShortRunHighGrayLevelEmphasis	481.0200897
wavelet-LHH	glrlm	RunLengthNonUniformity	391.6419323
wavelet-LHH	glrlm	ShortRunEmphasis	0.967790248
wavelet-LHH	glrlm	LongRunHighGrayLevelEmphasis	559.6045143
wavelet-LHH	glrlm	RunPercentage	0.957649092
wavelet-LHH	glrlm	LongRunLowGrayLevelEmphasis	0.006084034
wavelet-LHH	glrlm	RunEntropy	4.699835908
wavelet-LHH	glrlm	HighGrayLevelRunEmphasis	495.9366453
wavelet-LHH	glrlm	RunLengthNonUniformityNormalized	0.918633286
wavelet-LHH	glszm	GrayLevelVariance	51.13970178
wavelet-LHH	glszm	ZoneVariance	3.147141794
wavelet-LHH	glszm	GrayLevelNonUniformityNormalized	0.042842086
wavelet-LHH	glszm	SizeZoneNonUniformityNormalized	0.529326059
wavelet-LHH	glszm	SizeZoneNonUniformity	134.4488189
wavelet-LHH	glszm	GrayLevelNonUniformity	10.88188976
wavelet-LHH	glszm	LargeAreaEmphasis	6.216535433
wavelet-LHH	glszm	SmallAreaHighGrayLevelEmphasis	396.9299039
wavelet-LHH	glszm	ZonePercentage	0.570786517
wavelet-LHH	glszm	LargeAreaLowGrayLevelEmphasis	0.020219944
wavelet-LHH	glszm	LargeAreaHighGrayLevelEmphasis	2939.724409
wavelet-LHH	glszm	HighGrayLevelZoneEmphasis	513.7283465
wavelet-LHH	glszm	SmallAreaEmphasis	0.754905549
wavelet-LHH	glszm	LowGrayLevelZoneEmphasis	0.007991644
wavelet-LHH	glszm	ZoneEntropy	5.845507569
wavelet-LHH	glszm	SmallAreaLowGrayLevelEmphasis	0.007343752
wavelet-LHH	ngtdm	Coarseness	0.014061709

wavelet-LHH	ngtdm	Complexity	2500.661685
wavelet-LHH	ngtdm	Strength	5.798858562
wavelet-LHH	ngtdm	Contrast	0.240070447
wavelet-LHH	ngtdm	Busyness	0.080730986
wavelet-LLH	gldm	GrayLevelVariance	85.61307916
wavelet-LLH	gldm	HighGrayLevelEmphasis	1069.739326
wavelet-LLH	gldm	DependenceEntropy	6.449946311
wavelet-LLH	gldm	DependenceNonUniformity	164.6134831
wavelet-LLH	gldm	GrayLevelNonUniformity	15.65168539
wavelet-LLH	gldm	SmallDependenceEmphasis	0.613479363
wavelet-LLH	gldm	SmallDependenceHighGrayLevelEmphasis	674.10168
wavelet-LLH	gldm	DependenceNonUniformityNormalized	0.36991794
wavelet-LLH	gldm	LargeDependenceEmphasis	4.53258427
wavelet-LLH	gldm	LargeDependenceLowGrayLevelEmphasis	0.010145417
wavelet-LLH	gldm	DependenceVariance	1.260143921
wavelet-LLH	gldm	LargeDependenceHighGrayLevelEmphasis	4761.788764
wavelet-LLH	gldm	SmallDependenceLowGrayLevelEmphasis	0.005655044
wavelet-LLH	gldm	LowGrayLevelEmphasis	0.006159712
wavelet-LLH	glcm	JointAverage	30.86348163
wavelet-LLH	glcm	SumAverage	61.72696326
wavelet-LLH	glcm	JointEntropy	8.642022552
wavelet-LLH	glcm	ClusterShade	-619.8186339
wavelet-LLH	glcm	MaximumProbability	0.011194389
wavelet-LLH	glcm	Idmn	0.962811943
wavelet-LLH	glcm	JointEnergy	0.003009795
wavelet-LLH	glcm	Contrast	158.4694824
wavelet-LLH	glcm	DifferenceEntropy	4.554132753
wavelet-LLH	glcm	InverseVariance	0.124586438
wavelet-LLH	glcm	DifferenceVariance	63.95181323
wavelet-LLH	glcm	Idn	0.875907007
wavelet-LLH	glcm	Idm	0.119731956
wavelet-LLH	glcm	Correlation	0.095163857
wavelet-LLH	glcm	Autocorrelation	960.6694381
wavelet-LLH	glcm	SumEntropy	5.571131696
wavelet-LLH	glcm	SumSquares	87.32773955
wavelet-LLH	glcm	ClusterProminence	126253.433
wavelet-LLH	glcm	Imc2	0.978619883
wavelet-LLH	glcm	Imc1	-0.310736212
wavelet-LLH	glcm	DifferenceAverage	9.500397821
wavelet-LLH	glcm	Id	0.200424849
wavelet-LLH	glcm	ClusterTendency	190.8414759
wavelet-LLH	firstorder	InterquartileRange	280.191342
wavelet-LLH	firstorder	Skewness	-0.36051136
wavelet-LLH	firstorder	Uniformity	0.035172327
wavelet-LLH	firstorder	Median	-1.402350075
wavelet-LLH	firstorder	Energy	24111720.28
wavelet-LLH	firstorder	RobustMeanAbsoluteDeviation	118.7304502
wavelet-LLH	firstorder	MeanAbsoluteDeviation	178.7219684
wavelet-LLH	firstorder	TotalEnergy	651016447.6
wavelet-LLH	firstorder	Maximum	704.3705005
wavelet-LLH	firstorder	RootMeanSquared	232.7737981
wavelet-LLH	firstorder	90Percentile	237.3721798
wavelet-LLH	firstorder	Minimum	-799.2603775
wavelet-LLH	firstorder	Entropy	5.107689789
wavelet-LLH	firstorder	Range	1503.630878

wavelet-LLH	firstorder	Variance	53357.27554
wavelet-LLH	firstorder	10Percentile	-339.2949022
wavelet-LLH	firstorder	Kurtosis	3.476623446
wavelet-LLH	firstorder	Mean	-28.74657445
wavelet-LLH	glrlm	ShortRunLowGrayLevelEmphasis	0.006291644
wavelet-LLH	glrlm	GrayLevelVariance	87.27105682
wavelet-LLH	glrlm	LowGrayLevelRunEmphasis	0.00632022
wavelet-LLH	glrlm	GrayLevelNonUniformityNormalized	0.034500345
wavelet-LLH	glrlm	RunVariance	0.032517061
wavelet-LLH	glrlm	GrayLevelNonUniformity	14.87657156
wavelet-LLH	glrlm	LongRunEmphasis	1.098439614
wavelet-LLH	glrlm	ShortRunHighGrayLevelEmphasis	1046.117487
wavelet-LLH	glrlm	RunLengthNonUniformity	405.0610514
wavelet-LLH	glrlm	ShortRunEmphasis	0.976200471
wavelet-LLH	glrlm	LongRunHighGrayLevelEmphasis	1173.166839
wavelet-LLH	glrlm	RunPercentage	0.968885048
wavelet-LLH	glrlm	LongRunLowGrayLevelEmphasis	0.006437336
wavelet-LLH	glrlm	RunEntropy	5.272494191
wavelet-LLH	glrlm	HighGrayLevelRunEmphasis	1070.760475
wavelet-LLH	glrlm	RunLengthNonUniformityNormalized	0.939116496
wavelet-LLH	glszm	GrayLevelVariance	104.79534
wavelet-LLH	glszm	ZoneVariance	1.309340152
wavelet-LLH	glszm	GrayLevelNonUniformityNormalized	0.030334539
wavelet-LLH	glszm	SizeZoneNonUniformityNormalized	0.608462689
wavelet-LLH	glszm	SizeZoneNonUniformity	186.7980456
wavelet-LLH	glszm	GrayLevelNonUniformity	9.312703583
wavelet-LLH	glszm	LargeAreaEmphasis	3.410423453
wavelet-LLH	glszm	SmallAreaHighGrayLevelEmphasis	895.2908317
wavelet-LLH	glszm	ZonePercentage	0.68988764
wavelet-LLH	glszm	LargeAreaLowGrayLevelEmphasis	0.011132634
wavelet-LLH	glszm	LargeAreaHighGrayLevelEmphasis	3599.237785
wavelet-LLH	glszm	HighGrayLevelZoneEmphasis	1089.364821
wavelet-LLH	glszm	SmallAreaEmphasis	0.807614811
wavelet-LLH	glszm	LowGrayLevelZoneEmphasis	0.008356941
wavelet-LLH	glszm	ZoneEntropy	6.126775969
wavelet-LLH	glszm	SmallAreaLowGrayLevelEmphasis	0.008082896
wavelet-LLH	ngtdm	Coarseness	0.013667536
wavelet-LLH	ngtdm	Complexity	6309.603443
wavelet-LLH	ngtdm	Strength	10.4623425
wavelet-LLH	ngtdm	Contrast	0.488523956
wavelet-LLH	ngtdm	Busyness	0.046342973
wavelet-LLL	gldm	GrayLevelVariance	901.018023
wavelet-LLL	gldm	HighGrayLevelEmphasis	3260.139326
wavelet-LLL	gldm	DependenceEntropy	7.14566863
wavelet-LLL	gldm	DependenceNonUniformity	236.5685393
wavelet-LLL	gldm	GrayLevelNonUniformity	5.808988764
wavelet-LLL	gldm	SmallDependenceEmphasis	0.755489388
wavelet-LLL	gldm	SmallDependenceHighGrayLevelEmphasis	2623.5478
wavelet-LLL	gldm	DependenceNonUniformityNormalized	0.531614695
wavelet-LLL	gldm	LargeDependenceEmphasis	2.514606742
wavelet-LLL	gldm	LargeDependenceLowGrayLevelEmphasis	0.008617791
wavelet-LLL	gldm	DependenceVariance	0.503946471
wavelet-LLL	gldm	LargeDependenceHighGrayLevelEmphasis	7013.658427
wavelet-LLL	gldm	SmallDependenceLowGrayLevelEmphasis	0.003519814
wavelet-LLL	gldm	LowGrayLevelEmphasis	0.004133572

wavelet-LLL	glcm	JointAverage	52.63694696
wavelet-LLL	glcm	SumAverage	105.2738939
wavelet-LLL	glcm	JointEntropy	9.17461596
wavelet-LLL	glcm	ClusterShade	42165.20313
wavelet-LLL	glcm	MaximumProbability	0.0057584
wavelet-LLL	glcm	Idmn	0.955733556
wavelet-LLL	glcm	JointEnergy	0.001813913
wavelet-LLL	glcm	Contrast	756.916648
wavelet-LLL	glcm	DifferenceEntropy	5.589879829
wavelet-LLL	glcm	InverseVariance	0.060358531
wavelet-LLL	glcm	DifferenceVariance	295.5283657
wavelet-LLL	glcm	Idn	0.864098382
wavelet-LLL	glcm	Idm	0.060370289
wavelet-LLL	glcm	Correlation	0.603027242
wavelet-LLL	glcm	Autocorrelation	3343.42185
wavelet-LLL	glcm	SumEntropy	7.092112852
wavelet-LLL	glcm	SumSquares	950.7229246
wavelet-LLL	glcm	ClusterProminence	17905099.77
wavelet-LLL	glcm	Imc2	0.999748881
wavelet-LLL	glcm	Imc1	-0.586438819
wavelet-LLL	glcm	DifferenceAverage	21.15396556
wavelet-LLL	glcm	Id	0.120472537
wavelet-LLL	glcm	ClusterTendency	3045.97505
wavelet-LLL	firstorder	InterquartileRange	1276.628228
wavelet-LLL	firstorder	Skewness	0.504871004
wavelet-LLL	firstorder	Uniformity	0.013053907
wavelet-LLL	firstorder	Median	-1765.063067
wavelet-LLL	firstorder	Energy	1422269609
wavelet-LLL	firstorder	RobustMeanAbsoluteDeviation	504.8203993
wavelet-LLL	firstorder	MeanAbsoluteDeviation	642.4923946
wavelet-LLL	firstorder	TotalEnergy	38401279443
wavelet-LLL	firstorder	Maximum	223.1794571
wavelet-LLL	firstorder	RootMeanSquared	1787.767177
wavelet-LLL	firstorder	90Percentile	-498.9959735
wavelet-LLL	firstorder	Minimum	-2819.03277
wavelet-LLL	firstorder	Entropy	6.473104206
wavelet-LLL	firstorder	Range	3042.212227
wavelet-LLL	firstorder	Variance	563218.2164
wavelet-LLL	firstorder	10Percentile	-2483.940641
wavelet-LLL	firstorder	Kurtosis	2.104308572
wavelet-LLL	firstorder	Mean	-1622.61926
wavelet-LLL	glrlm	ShortRunLowGrayLevelEmphasis	0.004123052
wavelet-LLL	glrlm	GrayLevelVariance	896.8393851
wavelet-LLL	glrlm	LowGrayLevelRunEmphasis	0.004156057
wavelet-LLL	glrlm	GrayLevelNonUniformityNormalized	0.012952482
wavelet-LLL	glrlm	RunVariance	0.017492401
wavelet-LLL	glrlm	GrayLevelNonUniformity	5.671300387
wavelet-LLL	glrlm	LongRunEmphasis	1.050663703
wavelet-LLL	glrlm	ShortRunHighGrayLevelEmphasis	3241.778283
wavelet-LLL	glrlm	RunLengthNonUniformity	424.4045924
wavelet-LLL	glrlm	ShortRunEmphasis	0.988125483
wavelet-LLL	glrlm	LongRunHighGrayLevelEmphasis	3402.741718
wavelet-LLL	glrlm	RunPercentage	0.983923941
wavelet-LLL	glrlm	LongRunLowGrayLevelEmphasis	0.004295454
wavelet-LLL	glrlm	RunEntropy	6.537032477

wavelet-LLL	glrlm	HighGrayLevelRunEmphasis	3271.679409
wavelet-LLL	glrlm	RunLengthNonUniformityNormalized	0.969174875
wavelet-LLL	glszm	GrayLevelVariance	848.3604861
wavelet-LLL	glszm	ZoneVariance	0.469251543
wavelet-LLL	glszm	GrayLevelNonUniformityNormalized	0.011990741
wavelet-LLL	glszm	SizeZoneNonUniformityNormalized	0.741188272
wavelet-LLL	glszm	SizeZoneNonUniformity	266.8277778
wavelet-LLL	glszm	GrayLevelNonUniformity	4.316666667
wavelet-LLL	glszm	LargeAreaEmphasis	1.997222222
wavelet-LLL	glszm	SmallAreaHighGrayLevelEmphasis	3117.403085
wavelet-LLL	glszm	ZonePercentage	0.808988764
wavelet-LLL	glszm	LargeAreaLowGrayLevelEmphasis	0.007560346
wavelet-LLL	glszm	LargeAreaHighGrayLevelEmphasis	5596.933333
wavelet-LLL	glszm	HighGrayLevelZoneEmphasis	3443.413889
wavelet-LLL	glszm	SmallAreaEmphasis	0.88258642
wavelet-LLL	glszm	LowGrayLevelZoneEmphasis	0.004474617
wavelet-LLL	glszm	ZoneEntropy	6.945371956
wavelet-LLL	glszm	SmallAreaLowGrayLevelEmphasis	0.004220342
wavelet-LLL	ngtdm	Coarseness	0.015405958
wavelet-LLL	ngtdm	Complexity	56996.25418
wavelet-LLL	ngtdm	Strength	83.36597895
wavelet-LLL	ngtdm	Contrast	1.859204157
wavelet-LLL	ngtdm	Busyness	0.018490047
wavelet-HHL	gldm	GrayLevelVariance	37.68990784
wavelet-HHL	gldm	HighGrayLevelEmphasis	460.1078652
wavelet-HHL	gldm	DependenceEntropy	6.156346603
wavelet-HHL	gldm	DependenceNonUniformity	129.6831461
wavelet-HHL	gldm	GrayLevelNonUniformity	24.91011236
wavelet-HHL	gldm	SmallDependenceEmphasis	0.502431906
wavelet-HHL	gldm	SmallDependenceHighGrayLevelEmphasis	238.7048458
wavelet-HHL	gldm	DependenceNonUniformityNormalized	0.2914228
wavelet-HHL	gldm	LargeDependenceEmphasis	5.782022472
wavelet-HHL	gldm	LargeDependenceLowGrayLevelEmphasis	0.018374409
wavelet-HHL	gldm	DependenceVariance	1.461229643
wavelet-HHL	gldm	LargeDependenceHighGrayLevelEmphasis	2497.977528
wavelet-HHL	gldm	SmallDependenceLowGrayLevelEmphasis	0.00460936
wavelet-HHL	gldm	LowGrayLevelEmphasis	0.005932303
wavelet-HHL	glcm	JointAverage	20.54312602
wavelet-HHL	glcm	SumAverage	41.08625204
wavelet-HHL	glcm	JointEntropy	8.119041981
wavelet-HHL	glcm	ClusterShade	277.6225299
wavelet-HHL	glcm	MaximumProbability	0.015528586
wavelet-HHL	glcm	Idmn	0.96472048
wavelet-HHL	glcm	JointEnergy	0.004602553
wavelet-HHL	glcm	Contrast	82.5164271
wavelet-HHL	glcm	DifferenceEntropy	4.136164487
wavelet-HHL	glcm	InverseVariance	0.155214057
wavelet-HHL	glcm	DifferenceVariance	35.33157374
wavelet-HHL	glcm	Idn	0.879332573
wavelet-HHL	glcm	Idm	0.15405885
wavelet-HHL	glcm	Correlation	-0.017753966
wavelet-HHL	glcm	Autocorrelation	421.3542426
wavelet-HHL	glcm	SumEntropy	4.980673258
wavelet-HHL	glcm	SumSquares	40.58837089
wavelet-HHL	glcm	ClusterProminence	28284.57279

wavelet-HHL	glcm	Imc2	0.937840073
wavelet-HHL	glcm	Imc1	-0.231713328
wavelet-HHL	glcm	DifferenceAverage	6.787461365
wavelet-HHL	glcm	Id	0.243146192
wavelet-HHL	glcm	ClusterTendency	79.83705647
wavelet-HHL	firstorder	InterquartileRange	153.1900785
wavelet-HHL	firstorder	Skewness	0.354705069
wavelet-HHL	firstorder	Uniformity	0.055977781
wavelet-HHL	firstorder	Median	-5.613338562
wavelet-HHL	firstorder	Energy	10488153.56
wavelet-HHL	firstorder	RobustMeanAbsoluteDeviation	68.2417969
wavelet-HHL	firstorder	MeanAbsoluteDeviation	111.993116
wavelet-HHL	firstorder	TotalEnergy	283180146.1
wavelet-HHL	firstorder	Maximum	613.5034722
wavelet-HHL	firstorder	RootMeanSquared	153.5216089
wavelet-HHL	firstorder	90Percentile	184.3437046
wavelet-HHL	firstorder	Minimum	-490.9071754
wavelet-HHL	firstorder	Entropy	4.532572528
wavelet-HHL	firstorder	Range	1104.410648
wavelet-HHL	firstorder	Variance	23567.39319
wavelet-HHL	firstorder	10Percentile	-178.888405
wavelet-HHL	firstorder	Kurtosis	4.534690083
wavelet-HHL	firstorder	Mean	1.221152994
wavelet-HHL	glrlm	ShortRunLowGrayLevelEmphasis	0.005994859
wavelet-HHL	glrlm	GrayLevelVariance	38.76086033
wavelet-HHL	glrlm	LowGrayLevelRunEmphasis	0.006076993
wavelet-HHL	glrlm	GrayLevelNonUniformityNormalized	0.05463215
wavelet-HHL	glrlm	RunVariance	0.045430262
wavelet-HHL	glrlm	GrayLevelNonUniformity	23.30478892
wavelet-HHL	glrlm	LongRunEmphasis	1.135043434
wavelet-HHL	glrlm	ShortRunHighGrayLevelEmphasis	447.5036809
wavelet-HHL	glrlm	RunLengthNonUniformity	392.9115657
wavelet-HHL	glrlm	ShortRunEmphasis	0.968506549
wavelet-HHL	glrlm	LongRunHighGrayLevelEmphasis	519.7827667
wavelet-HHL	glrlm	RunPercentage	0.958513397
wavelet-HHL	glrlm	LongRunLowGrayLevelEmphasis	0.006431529
wavelet-HHL	glrlm	RunEntropy	4.764322557
wavelet-HHL	glrlm	HighGrayLevelRunEmphasis	461.2250334
wavelet-HHL	glrlm	RunLengthNonUniformityNormalized	0.920590401
wavelet-HHL	glszm	GrayLevelVariance	53.67066681
wavelet-HHL	glszm	ZoneVariance	2.457238264
wavelet-HHL	glszm	GrayLevelNonUniformityNormalized	0.042083451
wavelet-HHL	glszm	SizeZoneNonUniformityNormalized	0.502750406
wavelet-HHL	glszm	SizeZoneNonUniformity	130.2123552
wavelet-HHL	glszm	GrayLevelNonUniformity	10.8996139
wavelet-HHL	glszm	LargeAreaEmphasis	5.409266409
wavelet-HHL	glszm	SmallAreaHighGrayLevelEmphasis	353.1121255
wavelet-HHL	glszm	ZonePercentage	0.582022472
wavelet-HHL	glszm	LargeAreaLowGrayLevelEmphasis	0.019942051
wavelet-HHL	glszm	LargeAreaHighGrayLevelEmphasis	2321.088803
wavelet-HHL	glszm	HighGrayLevelZoneEmphasis	474.4749035
wavelet-HHL	glszm	SmallAreaEmphasis	0.736947252
wavelet-HHL	glszm	LowGrayLevelZoneEmphasis	0.008294091
wavelet-HHL	glszm	ZoneEntropy	5.974603386
wavelet-HHL	glszm	SmallAreaLowGrayLevelEmphasis	0.007581076

wavelet-HHL	ngtdm	Coarseness	0.014776387
wavelet-HHL	ngtdm	Complexity	2944.32154
wavelet-HHL	ngtdm	Strength	7.155898051
wavelet-HHL	ngtdm	Contrast	0.226645461
wavelet-HHL	ngtdm	Busyness	0.071725022
wavelet-HLH	gldm	GrayLevelVariance	27.83660649
wavelet-HLH	gldm	HighGrayLevelEmphasis	303.9191011
wavelet-HLH	gldm	DependenceEntropy	6.129841848
wavelet-HLH	gldm	DependenceNonUniformity	124.9011236
wavelet-HLH	gldm	GrayLevelNonUniformity	25.80449438
wavelet-HLH	gldm	SmallDependenceEmphasis	0.476304444
wavelet-HLH	gldm	SmallDependenceHighGrayLevelEmphasis	155.9067941
wavelet-HLH	gldm	DependenceNonUniformityNormalized	0.280676682
wavelet-HLH	gldm	LargeDependenceEmphasis	6.074157303
wavelet-HLH	gldm	LargeDependenceLowGrayLevelEmphasis	0.031881492
wavelet-HLH	gldm	DependenceVariance	1.487822245
wavelet-HLH	gldm	LargeDependenceHighGrayLevelEmphasis	1693.460674
wavelet-HLH	gldm	SmallDependenceLowGrayLevelEmphasis	0.005477309
wavelet-HLH	gldm	LowGrayLevelEmphasis	0.008095004
wavelet-HLH	glcm	JointAverage	16.64659748
wavelet-HLH	glcm	SumAverage	33.29319497
wavelet-HLH	glcm	JointEntropy	8.035869392
wavelet-HLH	glcm	ClusterShade	65.78367154
wavelet-HLH	glcm	MaximumProbability	0.014963269
wavelet-HLH	glcm	Idmn	0.961781713
wavelet-HLH	glcm	JointEnergy	0.004786693
wavelet-HLH	glcm	Contrast	59.54768874
wavelet-HLH	glcm	DifferenceEntropy	3.953236959
wavelet-HLH	glcm	InverseVariance	0.167367901
wavelet-HLH	glcm	DifferenceVariance	23.38635511
wavelet-HLH	glcm	Idn	0.87163217
wavelet-HLH	glcm	Idm	0.164320947
wavelet-HLH	glcm	Correlation	-0.013566604
wavelet-HLH	glcm	Autocorrelation	276.7479537
wavelet-HLH	glcm	SumEntropy	4.828877604
wavelet-HLH	glcm	SumSquares	29.40971582
wavelet-HLH	glcm	ClusterProminence	11870.39803
wavelet-HLH	glcm	Imc2	0.891015235
wavelet-HLH	glcm	Imc1	-0.179826753
wavelet-HLH	glcm	DifferenceAverage	5.953101114
wavelet-HLH	glcm	Id	0.255479297
wavelet-HLH	glcm	ClusterTendency	58.09117454
wavelet-HLH	firstorder	InterquartileRange	155.3972897
wavelet-HLH	firstorder	Skewness	0.16961292
wavelet-HLH	firstorder	Uniformity	0.057987628
wavelet-HLH	firstorder	Median	3.4705715
wavelet-HLH	firstorder	Energy	7685157.316
wavelet-HLH	firstorder	RobustMeanAbsoluteDeviation	65.63697189
wavelet-HLH	firstorder	MeanAbsoluteDeviation	100.7405535
wavelet-HLH	firstorder	TotalEnergy	207499247.5
wavelet-HLH	firstorder	Maximum	509.0736546
wavelet-HLH	firstorder	RootMeanSquared	131.4154346
wavelet-HLH	firstorder	90Percentile	167.5099914
wavelet-HLH	firstorder	Minimum	-377.8708366
wavelet-HLH	firstorder	Entropy	4.384980502

wavelet-HLH	firstorder	Range	886.9444912
wavelet-HLH	firstorder	Variance	17258.19072
wavelet-HLH	firstorder	10Percentile	-169.5750764
wavelet-HLH	firstorder	Kurtosis	3.564564635
wavelet-HLH	firstorder	Mean	3.438855073
wavelet-HLH	glrlm	ShortRunLowGrayLevelEmphasis	0.008086375
wavelet-HLH	glrlm	GrayLevelVariance	28.45537932
wavelet-HLH	glrlm	LowGrayLevelRunEmphasis	0.00824529
wavelet-HLH	glrlm	GrayLevelNonUniformityNormalized	0.057083803
wavelet-HLH	glrlm	RunVariance	0.051097737
wavelet-HLH	glrlm	GrayLevelNonUniformity	24.28721554
wavelet-HLH	glrlm	LongRunEmphasis	1.145757049
wavelet-HLH	glrlm	ShortRunHighGrayLevelEmphasis	296.2016067
wavelet-HLH	glrlm	RunLengthNonUniformity	390.8720158
wavelet-HLH	glrlm	ShortRunEmphasis	0.96752751
wavelet-HLH	glrlm	LongRunHighGrayLevelEmphasis	345.4628229
wavelet-HLH	glrlm	RunPercentage	0.956093345
wavelet-HLH	glrlm	LongRunLowGrayLevelEmphasis	0.008944333
wavelet-HLH	glrlm	RunEntropy	4.617772036
wavelet-HLH	glrlm	HighGrayLevelRunEmphasis	305.1848535
wavelet-HLH	glrlm	RunLengthNonUniformityNormalized	0.918351775
wavelet-HLH	glszm	GrayLevelVariance	38.3713779
wavelet-HLH	glszm	ZoneVariance	6.063566399
wavelet-HLH	glszm	GrayLevelNonUniformityNormalized	0.047123844
wavelet-HLH	glszm	SizeZoneNonUniformityNormalized	0.499629827
wavelet-HLH	glszm	SizeZoneNonUniformity	120.4107884
wavelet-HLH	glszm	GrayLevelNonUniformity	11.35684647
wavelet-HLH	glszm	LargeAreaEmphasis	9.473029046
wavelet-HLH	glszm	SmallAreaHighGrayLevelEmphasis	246.1139054
wavelet-HLH	glszm	ZonePercentage	0.541573034
wavelet-HLH	glszm	LargeAreaLowGrayLevelEmphasis	0.047296714
wavelet-HLH	glszm	LargeAreaHighGrayLevelEmphasis	2601.369295
wavelet-HLH	glszm	HighGrayLevelZoneEmphasis	324.6970954
wavelet-HLH	glszm	SmallAreaEmphasis	0.734268148
wavelet-HLH	glszm	LowGrayLevelZoneEmphasis	0.010829917
wavelet-HLH	glszm	ZoneEntropy	5.772767657
wavelet-HLH	glszm	SmallAreaLowGrayLevelEmphasis	0.009396331
wavelet-HLH	ngtdm	Coarseness	0.01481453
wavelet-HLH	ngtdm	Complexity	1510.95531
wavelet-HLH	ngtdm	Strength	3.928398002
wavelet-HLH	ngtdm	Contrast	0.24752601
wavelet-HLH	ngtdm	Busyness	0.129884279

Image type	Feature Class	Feature Name	5: RTSTRUCT: C99-DNU_segment_LLL GTV
general	info	BoundingBox	(1, 1, 1, 9, 8, 6)
general	info	EnabledImageTypes	{u'Original': {}, u'Wavelet': {}}
general	info	GeneralSettings	{'distances': [1], 'additionalInfo': True, 'force2D': False, 'interpolator': 'sitkBSpline', 'resampledPixelSpacing': [3.0], 'label': 1, 'normalizeScale': 1, 'normalize': False, 'force2Ddimension': 0, 'removeOutliers': None, 'minimumROISize': None, u'binWidth': 25.0, 'minimumROIDimensions': 1, u'correctMask': True, 'preCrop': False, u'symmetricalGLCM': True, 'resegmentRange': None, 'padDistance': 5}
general	info	ImageHash	4075865a58efec90b69044c0e5454e4b5f26a091
general	info	ImageSpacing	(1.6015625, 1.6015625, 2.0)
general	info	MaskHash	d9f2bf06d98723392469aea92f05345f390cd2c9
general	info	NumpyVersion	1.13.1
general	info	PyWaveletVersion	0.5.2
general	info	SimpleITKVersion	1.1.0
general	info	Version	1.3.0.post102+g1b0eb74
general	info	VolumeNum	1
general	info	VoxelNum	197
original	shape	Maximum3DDiameter	25.45584412
original	shape	Maximum2DDiameterSlice	22.84731932
original	shape	Sphericity	0.869271948
original	shape	MinorAxis	21.32120371
original	shape	Elongation	0.940875629
original	shape	SurfaceVolumeRatio	0.31870225
original	shape	Volume	5319
original	shape	MajorAxis	22.66102241
original	shape	SurfaceArea	1695.177267
original	shape	Flatness	0.721036538
original	shape	LeastAxis	16.33942515
original	shape	Maximum2DDiameterColumn	24.73863375
original	shape	Maximum2DDiameterRow	21.84032967
original	gldm	GrayLevelVariance	61.59287794
original	gldm	HighGrayLevelEmphasis	578.751269
original	gldm	DependenceEntropy	6.048019366
original	gldm	DependenceNonUniformity	38.89847716
original	gldm	GrayLevelNonUniformity	15.28426396
original	gldm	SmallDependenceEmphasis	0.436177092
original	gldm	SmallDependenceHighGrayLevelEmphasis	171.2622509
original	gldm	DependenceNonUniformityNormalized	0.197454199
original	gldm	LargeDependenceEmphasis	19.69035533
original	gldm	LargeDependenceLowGrayLevelEmphasis	0.038015059
original	gldm	DependenceVariance	8.157953052
original	gldm	LargeDependenceHighGrayLevelEmphasis	15892.75127
original	gldm	SmallDependenceLowGrayLevelEmphasis	0.010347547
original	gldm	LowGrayLevelEmphasis	0.011751437
original	glcm	JointAverage	24.32666427
original	glcm	SumAverage	48.65332853
original	glcm	JointEntropy	6.53288696

original	glcm	ClusterShade	-1652.538563
original	glcm	MaximumProbability	0.070064053
original	glcm	Idmn	0.969803249
original	glcm	JointEnergy	0.018794343
original	glcm	Contrast	66.61442018
original	glcm	DifferenceEntropy	3.795631561
original	glcm	InverseVariance	0.259807574
original	glcm	DifferenceVariance	35.17894326
original	glcm	Idn	0.899949596
original	glcm	Idm	0.279502319
original	glcm	Correlation	0.298652733
original	glcm	Autocorrelation	606.3858092
original	glcm	SumEntropy	4.669579478
original	glcm	SumSquares	47.78163533
original	glcm	ClusterProminence	60286.37357
original	glcm	Imc2	0.954926509
original	glcm	Imc1	-0.317204655
original	glcm	DifferenceAverage	5.528143881
original	glcm	Id	0.347598456
original	glcm	ClusterTendency	124.5121211
original	firstorder	InterquartileRange	275
original	firstorder	Skewness	-0.919417492
original	firstorder	Uniformity	0.077585096
original	firstorder	Median	-63
original	firstorder	Energy	11551210
original	firstorder	RobustMeanAbsoluteDeviation	119.1574506
original	firstorder	MeanAbsoluteDeviation	162.1375454
original	firstorder	TotalEnergy	311882670
original	firstorder	Maximum	384
original	firstorder	RootMeanSquared	242.1478552
original	firstorder	90Percentile	24.8
original	firstorder	Minimum	-690
original	firstorder	Entropy	4.247973494
original	firstorder	Range	1074
original	firstorder	Variance	38010.54152
original	firstorder	10Percentile	-445.4
original	firstorder	Kurtosis	3.019055797
original	firstorder	Mean	-143.6142132
original	glrlm	ShortRunLowGrayLevelEmphasis	0.012666597
original	glrlm	GrayLevelVariance	63.58676774
original	glrlm	LowGrayLevelRunEmphasis	0.012786927
original	glrlm	GrayLevelNonUniformityNormalized	0.066839947
original	glrlm	RunVariance	0.117095199
original	glrlm	GrayLevelNonUniformity	11.968273
original	glrlm	LongRunEmphasis	1.334621502
original	glrlm	ShortRunHighGrayLevelEmphasis	501.5185109
original	glrlm	RunLengthNonUniformity	149.6391635
original	glrlm	ShortRunEmphasis	0.930266265
original	glrlm	LongRunHighGrayLevelEmphasis	823.5336561
original	glrlm	RunPercentage	0.907848497
original	glrlm	LongRunLowGrayLevelEmphasis	0.013334819
original	glrlm	RunEntropy	4.698708586
original	glrlm	HighGrayLevelRunEmphasis	556.5967312
original	glrlm	RunLengthNonUniformityNormalized	0.834965272
original	glszm	GrayLevelVariance	64.42881394

original	glszm	ZoneVariance	20.40572657
original	glszm	GrayLevelNonUniformityNormalized	0.043005885
original	glszm	SizeZoneNonUniformityNormalized	0.619284744
original	glszm	SizeZoneNonUniformity	58.21276596
original	glszm	GrayLevelNonUniformity	4.042553191
original	glszm	LargeAreaEmphasis	24.79787234
original	glszm	SmallAreaHighGrayLevelEmphasis	296.1383054
original	glszm	ZonePercentage	0.47715736
original	glszm	LargeAreaLowGrayLevelEmphasis	0.055194383
original	glszm	LargeAreaHighGrayLevelEmphasis	19891.19149
original	glszm	HighGrayLevelZoneEmphasis	396.5212766
original	glszm	SmallAreaEmphasis	0.811199881
original	glszm	LowGrayLevelZoneEmphasis	0.022116262
original	glszm	ZoneEntropy	5.366391145
original	glszm	SmallAreaLowGrayLevelEmphasis	0.021265823
original	ngtdm	Coarseness	0.019578331
original	ngtdm	Complexity	1958.665546
original	ngtdm	Strength	12.82397857
original	ngtdm	Contrast	0.614526476
original	ngtdm	Busyness	0.055187052
wavelet-HHH	gldm	GrayLevelVariance	8.879280579
wavelet-HHH	gldm	HighGrayLevelEmphasis	140.8375635
wavelet-HHH	gldm	DependenceEntropy	5.605282737
wavelet-HHH	gldm	DependenceNonUniformity	33.07106599
wavelet-HHH	gldm	GrayLevelNonUniformity	22.36040609
wavelet-HHH	gldm	SmallDependenceEmphasis	0.308676841
wavelet-HHH	gldm	SmallDependenceHighGrayLevelEmphasis	48.50674917
wavelet-HHH	gldm	DependenceNonUniformityNormalized	0.167873431
wavelet-HHH	gldm	LargeDependenceEmphasis	14.87817259
wavelet-HHH	gldm	LargeDependenceLowGrayLevelEmphasis	0.125789395
wavelet-HHH	gldm	DependenceVariance	4.224741684
wavelet-HHH	gldm	LargeDependenceHighGrayLevelEmphasis	2013.22335
wavelet-HHH	gldm	SmallDependenceLowGrayLevelEmphasis	0.008408426
wavelet-HHH	gldm	LowGrayLevelEmphasis	0.014728923
wavelet-HHH	glcm	JointAverage	11.50602701
wavelet-HHH	glcm	SumAverage	23.01205402
wavelet-HHH	glcm	JointEntropy	6.23344852
wavelet-HHH	glcm	ClusterShade	6.580341878
wavelet-HHH	glcm	MaximumProbability	0.044163033
wavelet-HHH	glcm	Idmn	0.96560904
wavelet-HHH	glcm	JointEnergy	0.018245833
wavelet-HHH	glcm	Contrast	17.4985919
wavelet-HHH	glcm	DifferenceEntropy	3.107380477
wavelet-HHH	glcm	InverseVariance	0.298493009
wavelet-HHH	glcm	DifferenceVariance	7.8756505
wavelet-HHH	glcm	Idn	0.882282906
wavelet-HHH	glcm	Idm	0.300851627
wavelet-HHH	glcm	Correlation	-0.034156314
wavelet-HHH	glcm	Autocorrelation	132.0867985
wavelet-HHH	glcm	SumEntropy	3.835792398
wavelet-HHH	glcm	SumSquares	8.443385085
wavelet-HHH	glcm	ClusterProminence	1164.519631
wavelet-HHH	glcm	Imc2	0.838477893
wavelet-HHH	glcm	Imc1	-0.17900954
wavelet-HHH	glcm	DifferenceAverage	3.089615406

wavelet-HHH	glcm	Id	0.383445352
wavelet-HHH	glcm	ClusterTendency	16.27494844
wavelet-HHH	firstorder	InterquartileRange	70.83078711
wavelet-HHH	firstorder	Skewness	0.146278438
wavelet-HHH	firstorder	Uniformity	0.113504599
wavelet-HHH	firstorder	Median	-5.669275023
wavelet-HHH	firstorder	Energy	1060745.169
wavelet-HHH	firstorder	RobustMeanAbsoluteDeviation	31.59345479
wavelet-HHH	firstorder	MeanAbsoluteDeviation	53.13526216
wavelet-HHH	firstorder	TotalEnergy	28640119.56
wavelet-HHH	firstorder	Maximum	248.9024478
wavelet-HHH	firstorder	RootMeanSquared	73.37910631
wavelet-HHH	firstorder	90Percentile	85.73222595
wavelet-HHH	firstorder	Minimum	-251.7538667
wavelet-HHH	firstorder	Entropy	3.475033148
wavelet-HHH	firstorder	Range	500.6563145
wavelet-HHH	firstorder	Variance	5383.605642
wavelet-HHH	firstorder	10Percentile	-79.38683927
wavelet-HHH	firstorder	Kurtosis	4.668692466
wavelet-HHH	firstorder	Mean	-0.942125675
wavelet-HHH	glrlm	ShortRunLowGrayLevelEmphasis	0.014773466
wavelet-HHH	glrlm	GrayLevelVariance	9.418784341
wavelet-HHH	glrlm	LowGrayLevelRunEmphasis	0.015339607
wavelet-HHH	glrlm	GrayLevelNonUniformityNormalized	0.108437177
wavelet-HHH	glrlm	RunVariance	0.100807289
wavelet-HHH	glrlm	GrayLevelNonUniformity	19.506438
wavelet-HHH	glrlm	LongRunEmphasis	1.301890183
wavelet-HHH	glrlm	ShortRunHighGrayLevelEmphasis	132.4308533
wavelet-HHH	glrlm	RunLengthNonUniformity	150.8533709
wavelet-HHH	glrlm	ShortRunEmphasis	0.932654934
wavelet-HHH	glrlm	LongRunHighGrayLevelEmphasis	182.0344989
wavelet-HHH	glrlm	RunPercentage	0.912924639
wavelet-HHH	glrlm	LongRunLowGrayLevelEmphasis	0.017848585
wavelet-HHH	glrlm	RunEntropy	3.913630844
wavelet-HHH	glrlm	HighGrayLevelRunEmphasis	141.465024
wavelet-HHH	glrlm	RunLengthNonUniformityNormalized	0.838236051
wavelet-HHH	glszm	GrayLevelVariance	17.23875433
wavelet-HHH	glszm	ZoneVariance	24.94528547
wavelet-HHH	glszm	GrayLevelNonUniformityNormalized	0.076124567
wavelet-HHH	glszm	SizeZoneNonUniformityNormalized	0.428200692
wavelet-HHH	glszm	SizeZoneNonUniformity	29.11764706
wavelet-HHH	glszm	GrayLevelNonUniformity	5.176470588
wavelet-HHH	glszm	LargeAreaEmphasis	33.33823529
wavelet-HHH	glszm	SmallAreaHighGrayLevelEmphasis	112.1960429
wavelet-HHH	glszm	ZonePercentage	0.345177665
wavelet-HHH	glszm	LargeAreaLowGrayLevelEmphasis	0.278147996
wavelet-HHH	glszm	LargeAreaHighGrayLevelEmphasis	4557.455882
wavelet-HHH	glszm	HighGrayLevelZoneEmphasis	155.6470588
wavelet-HHH	glszm	SmallAreaEmphasis	0.677348357
wavelet-HHH	glszm	LowGrayLevelZoneEmphasis	0.025900764
wavelet-HHH	glszm	ZoneEntropy	4.947377732
wavelet-HHH	glszm	SmallAreaLowGrayLevelEmphasis	0.022189133
wavelet-HHH	ngtdm	Coarseness	0.033415945
wavelet-HHH	ngtdm	Complexity	287.8000731
wavelet-HHH	ngtdm	Strength	3.168995682

wavelet-HHH	ngtdm	Contrast	0.133180788
wavelet-HHH	ngtdm	Busyness	0.13341003
wavelet-HLL	gldm	GrayLevelVariance	78.73668479
wavelet-HLL	gldm	HighGrayLevelEmphasis	464.6497462
wavelet-HLL	gldm	DependenceEntropy	6.04531767
wavelet-HLL	gldm	DependenceNonUniformity	62.59390863
wavelet-HLL	gldm	GrayLevelNonUniformity	7.791878173
wavelet-HLL	gldm	SmallDependenceEmphasis	0.523389735
wavelet-HLL	gldm	SmallDependenceHighGrayLevelEmphasis	275.2635716
wavelet-HLL	gldm	DependenceNonUniformityNormalized	0.317735577
wavelet-HLL	gldm	LargeDependenceEmphasis	4.969543147
wavelet-HLL	gldm	LargeDependenceLowGrayLevelEmphasis	0.063466951
wavelet-HLL	gldm	DependenceVariance	1.150197119
wavelet-HLL	gldm	LargeDependenceHighGrayLevelEmphasis	1883.583756
wavelet-HLL	gldm	SmallDependenceLowGrayLevelEmphasis	0.005044873
wavelet-HLL	gldm	LowGrayLevelEmphasis	0.014651915
wavelet-HLL	glcm	JointAverage	18.77438783
wavelet-HLL	glcm	SumAverage	37.54877567
wavelet-HLL	glcm	JointEntropy	7.548434181
wavelet-HLL	glcm	ClusterShade	2614.143776
wavelet-HLL	glcm	MaximumProbability	0.016398375
wavelet-HLL	glcm	Idmn	0.97008422
wavelet-HLL	glcm	JointEnergy	0.006013038
wavelet-HLL	glcm	Contrast	87.36815313
wavelet-HLL	glcm	DifferenceEntropy	4.099958126
wavelet-HLL	glcm	InverseVariance	0.154559915
wavelet-HLL	glcm	DifferenceVariance	35.80368245
wavelet-HLL	glcm	Idn	0.887412941
wavelet-HLL	glcm	Idm	0.147195962
wavelet-HLL	glcm	Correlation	0.313696079
wavelet-HLL	glcm	Autocorrelation	372.7251971
wavelet-HLL	glcm	SumEntropy	5.13332646
wavelet-HLL	glcm	SumSquares	63.88894332
wavelet-HLL	glcm	ClusterProminence	151556.6468
wavelet-HLL	glcm	Imc2	0.988033508
wavelet-HLL	glcm	Imc1	-0.399452105
wavelet-HLL	glcm	DifferenceAverage	7.046115882
wavelet-HLL	glcm	Id	0.235128618
wavelet-HLL	glcm	ClusterTendency	168.1876201
wavelet-HLL	firstorder	InterquartileRange	253.5633028
wavelet-HLL	firstorder	Skewness	0.915435707
wavelet-HLL	firstorder	Uniformity	0.039552681
wavelet-HLL	firstorder	Median	-101.5654979
wavelet-HLL	firstorder	Energy	10720201.15
wavelet-HLL	firstorder	RobustMeanAbsoluteDeviation	112.9549694
wavelet-HLL	firstorder	MeanAbsoluteDeviation	170.9067067
wavelet-HLL	firstorder	TotalEnergy	289445430.9
wavelet-HLL	firstorder	Maximum	722.0825205
wavelet-HLL	firstorder	RootMeanSquared	233.2750838
wavelet-HLL	firstorder	90Percentile	250.5097186
wavelet-HLL	firstorder	Minimum	-535.8485652
wavelet-HLL	firstorder	Entropy	4.881537864
wavelet-HLL	firstorder	Range	1257.931086
wavelet-HLL	firstorder	Variance	49260.97057
wavelet-HLL	firstorder	10Percentile	-314.9271291

wavelet-HLL	firstorder	Kurtosis	3.990633983
wavelet-HLL	firstorder	Mean	-71.80734035
wavelet-HLL	glrlm	ShortRunLowGrayLevelEmphasis	0.01416709
wavelet-HLL	glrlm	GrayLevelVariance	79.86817633
wavelet-HLL	glrlm	LowGrayLevelRunEmphasis	0.014612825
wavelet-HLL	glrlm	GrayLevelNonUniformityNormalized	0.039299505
wavelet-HLL	glrlm	RunVariance	0.040804032
wavelet-HLL	glrlm	GrayLevelNonUniformity	7.457752378
wavelet-HLL	glrlm	LongRunEmphasis	1.119740373
wavelet-HLL	glrlm	ShortRunHighGrayLevelEmphasis	457.8901871
wavelet-HLL	glrlm	RunLengthNonUniformity	176.632263
wavelet-HLL	glrlm	ShortRunEmphasis	0.972406725
wavelet-HLL	glrlm	LongRunHighGrayLevelEmphasis	512.148639
wavelet-HLL	glrlm	RunPercentage	0.963295588
wavelet-HLL	glrlm	LongRunLowGrayLevelEmphasis	0.016444846
wavelet-HLL	glrlm	RunEntropy	5.025915762
wavelet-HLL	glrlm	HighGrayLevelRunEmphasis	468.2702428
wavelet-HLL	glrlm	RunLengthNonUniformityNormalized	0.93017943
wavelet-HLL	glszm	GrayLevelVariance	95.91926781
wavelet-HLL	glszm	ZoneVariance	1.440202172
wavelet-HLL	glszm	GrayLevelNonUniformityNormalized	0.035994809
wavelet-HLL	glszm	SizeZoneNonUniformityNormalized	0.500717164
wavelet-HLL	glszm	SizeZoneNonUniformity	60.58677686
wavelet-HLL	glszm	GrayLevelNonUniformity	4.355371901
wavelet-HLL	glszm	LargeAreaEmphasis	4.090909091
wavelet-HLL	glszm	SmallAreaHighGrayLevelEmphasis	400.7650666
wavelet-HLL	glszm	ZonePercentage	0.614213198
wavelet-HLL	glszm	LargeAreaLowGrayLevelEmphasis	0.050809759
wavelet-HLL	glszm	LargeAreaHighGrayLevelEmphasis	1624.479339
wavelet-HLL	glszm	HighGrayLevelZoneEmphasis	516.677686
wavelet-HLL	glszm	SmallAreaEmphasis	0.733760904
wavelet-HLL	glszm	LowGrayLevelZoneEmphasis	0.012933727
wavelet-HLL	glszm	ZoneEntropy	5.770368304
wavelet-HLL	glszm	SmallAreaLowGrayLevelEmphasis	0.005688077
wavelet-HLL	ngtdm	Coarseness	0.033177868
wavelet-HLL	ngtdm	Complexity	2927.557381
wavelet-HLL	ngtdm	Strength	19.19720934
wavelet-HLL	ngtdm	Contrast	0.553405226
wavelet-HLL	ngtdm	Busyness	0.056924619
wavelet-LHL	gldm	GrayLevelVariance	53.01893891
wavelet-LHL	gldm	HighGrayLevelEmphasis	500.2233503
wavelet-LHL	gldm	DependenceEntropy	6.043775728
wavelet-LHL	gldm	DependenceNonUniformity	58.88832487
wavelet-LHL	gldm	GrayLevelNonUniformity	9.588832487
wavelet-LHL	gldm	SmallDependenceEmphasis	0.515679639
wavelet-LHL	gldm	SmallDependenceHighGrayLevelEmphasis	280.5566004
wavelet-LHL	gldm	DependenceNonUniformityNormalized	0.298925507
wavelet-LHL	gldm	LargeDependenceEmphasis	5.406091371
wavelet-LHL	gldm	LargeDependenceLowGrayLevelEmphasis	0.055711002
wavelet-LHL	gldm	DependenceVariance	1.262696797
wavelet-LHL	gldm	LargeDependenceHighGrayLevelEmphasis	2534.781726
wavelet-LHL	gldm	SmallDependenceLowGrayLevelEmphasis	0.004563712
wavelet-LHL	gldm	LowGrayLevelEmphasis	0.013592358
wavelet-LHL	glcm	JointAverage	21.0608298
wavelet-LHL	glcm	SumAverage	42.12165959

wavelet-LHL	glcm	JointEntropy	7.459546947
wavelet-LHL	glcm	ClusterShade	443.1266976
wavelet-LHL	glcm	MaximumProbability	0.018722737
wavelet-LHL	glcm	Idmn	0.966410612
wavelet-LHL	glcm	JointEnergy	0.006625003
wavelet-LHL	glcm	Contrast	70.3652918
wavelet-LHL	glcm	DifferenceEntropy	3.960236995
wavelet-LHL	glcm	InverseVariance	0.181549499
wavelet-LHL	glcm	DifferenceVariance	29.11266856
wavelet-LHL	glcm	Idn	0.881666682
wavelet-LHL	glcm	Idm	0.167276245
wavelet-LHL	glcm	Correlation	0.236462444
wavelet-LHL	glcm	Autocorrelation	455.132455
wavelet-LHL	glcm	SumEntropy	5.039657273
wavelet-LHL	glcm	SumSquares	46.73512258
wavelet-LHL	glcm	ClusterProminence	54687.55578
wavelet-LHL	glcm	Imc2	0.984127439
wavelet-LHL	glcm	Imc1	-0.378445068
wavelet-LHL	glcm	DifferenceAverage	6.319724912
wavelet-LHL	glcm	Id	0.254516789
wavelet-LHL	glcm	ClusterTendency	116.5751985
wavelet-LHL	firstorder	InterquartileRange	186.0376496
wavelet-LHL	firstorder	Skewness	0.170560277
wavelet-LHL	firstorder	Uniformity	0.048674277
wavelet-LHL	firstorder	Median	-61.82151147
wavelet-LHL	firstorder	Energy	7256822.146
wavelet-LHL	firstorder	RobustMeanAbsoluteDeviation	85.33566802
wavelet-LHL	firstorder	MeanAbsoluteDeviation	136.7069573
wavelet-LHL	firstorder	TotalEnergy	195934197.9
wavelet-LHL	firstorder	Maximum	494.6537253
wavelet-LHL	firstorder	RootMeanSquared	191.9287905
wavelet-LHL	firstorder	90Percentile	171.321446
wavelet-LHL	firstorder	Minimum	-568.325629
wavelet-LHL	firstorder	Entropy	4.705110138
wavelet-LHL	firstorder	Range	1062.979354
wavelet-LHL	firstorder	Variance	33487.21395
wavelet-LHL	firstorder	10Percentile	-299.5452095
wavelet-LHL	firstorder	Kurtosis	3.516729122
wavelet-LHL	firstorder	Mean	-57.87440444
wavelet-LHL	glrlm	ShortRunLowGrayLevelEmphasis	0.01321041
wavelet-LHL	glrlm	GrayLevelVariance	54.15542414
wavelet-LHL	glrlm	LowGrayLevelRunEmphasis	0.013617407
wavelet-LHL	glrlm	GrayLevelNonUniformityNormalized	0.047699184
wavelet-LHL	glrlm	RunVariance	0.043028812
wavelet-LHL	glrlm	GrayLevelNonUniformity	9.022192977
wavelet-LHL	glrlm	LongRunEmphasis	1.129236075
wavelet-LHL	glrlm	ShortRunHighGrayLevelEmphasis	488.1626942
wavelet-LHL	glrlm	RunLengthNonUniformity	174.8128982
wavelet-LHL	glrlm	ShortRunEmphasis	0.969549189
wavelet-LHL	glrlm	LongRunHighGrayLevelEmphasis	561.3389174
wavelet-LHL	glrlm	RunPercentage	0.960171808
wavelet-LHL	glrlm	LongRunLowGrayLevelEmphasis	0.015259984
wavelet-LHL	glrlm	RunEntropy	4.88697726
wavelet-LHL	glrlm	HighGrayLevelRunEmphasis	502.0235915
wavelet-LHL	glrlm	RunLengthNonUniformityNormalized	0.923403115

wavelet-LHL	glszm	GrayLevelVariance	68.26235277
wavelet-LHL	glszm	ZoneVariance	1.695848894
wavelet-LHL	glszm	GrayLevelNonUniformityNormalized	0.038207412
wavelet-LHL	glszm	SizeZoneNonUniformityNormalized	0.514076415
wavelet-LHL	glszm	SizeZoneNonUniformity	60.66101695
wavelet-LHL	glszm	GrayLevelNonUniformity	4.508474576
wavelet-LHL	glszm	LargeAreaEmphasis	4.483050847
wavelet-LHL	glszm	SmallAreaHighGrayLevelEmphasis	416.3641227
wavelet-LHL	glszm	ZonePercentage	0.598984772
wavelet-LHL	glszm	LargeAreaLowGrayLevelEmphasis	0.047231265
wavelet-LHL	glszm	LargeAreaHighGrayLevelEmphasis	2099.288136
wavelet-LHL	glszm	HighGrayLevelZoneEmphasis	533.4322034
wavelet-LHL	glszm	SmallAreaEmphasis	0.743732273
wavelet-LHL	glszm	LowGrayLevelZoneEmphasis	0.012293226
wavelet-LHL	glszm	ZoneEntropy	5.707488045
wavelet-LHL	glszm	SmallAreaLowGrayLevelEmphasis	0.005140089
wavelet-LHL	ngtdm	Coarseness	0.03196996
wavelet-LHL	ngtdm	Complexity	2263.703442
wavelet-LHL	ngtdm	Strength	13.55040063
wavelet-LHL	ngtdm	Contrast	0.391854789
wavelet-LHL	ngtdm	Busyness	0.044821317
wavelet-LHH	gldm	GrayLevelVariance	27.99216677
wavelet-LHH	gldm	HighGrayLevelEmphasis	372.5939086
wavelet-LHH	gldm	DependenceEntropy	5.964149239
wavelet-LHH	gldm	DependenceNonUniformity	42.38071066
wavelet-LHH	gldm	GrayLevelNonUniformity	14.95939086
wavelet-LHH	gldm	SmallDependenceEmphasis	0.442802546
wavelet-LHH	gldm	SmallDependenceHighGrayLevelEmphasis	170.6708275
wavelet-LHH	gldm	DependenceNonUniformityNormalized	0.215130511
wavelet-LHH	gldm	LargeDependenceEmphasis	13.24365482
wavelet-LHH	gldm	LargeDependenceLowGrayLevelEmphasis	0.046397768
wavelet-LHH	gldm	DependenceVariance	5.076245201
wavelet-LHH	gldm	LargeDependenceHighGrayLevelEmphasis	4554.989848
wavelet-LHH	gldm	SmallDependenceLowGrayLevelEmphasis	0.007678456
wavelet-LHH	gldm	LowGrayLevelEmphasis	0.009370183
wavelet-LHH	glcm	JointAverage	18.48950297
wavelet-LHH	glcm	SumAverage	36.97900594
wavelet-LHH	glcm	JointEntropy	6.782908795
wavelet-LHH	glcm	ClusterShade	-8.675860826
wavelet-LHH	glcm	MaximumProbability	0.039850441
wavelet-LHH	glcm	Idmn	0.984209716
wavelet-LHH	glcm	JointEnergy	0.012588239
wavelet-LHH	glcm	Contrast	46.80372775
wavelet-LHH	glcm	DifferenceEntropy	3.542462282
wavelet-LHH	glcm	InverseVariance	0.263071836
wavelet-LHH	glcm	DifferenceVariance	26.07985842
wavelet-LHH	glcm	Idn	0.925258755
wavelet-LHH	glcm	Idm	0.258935312
wavelet-LHH	glcm	Correlation	-0.044033918
wavelet-LHH	glcm	Autocorrelation	341.0781934
wavelet-LHH	glcm	SumEntropy	4.304524439
wavelet-LHH	glcm	SumSquares	22.58966338
wavelet-LHH	glcm	ClusterProminence	14727.77764
wavelet-LHH	glcm	Imc2	0.945942126
wavelet-LHH	glcm	Imc1	-0.286357057

wavelet-LHH	glcm	DifferenceAverage	4.512428704
wavelet-LHH	glcm	Id	0.34039551
wavelet-LHH	glcm	ClusterTendency	43.55492576
wavelet-LHH	firstorder	InterquartileRange	109.9588952
wavelet-LHH	firstorder	Skewness	0.950319645
wavelet-LHH	firstorder	Uniformity	0.075935994
wavelet-LHH	firstorder	Median	-0.130316494
wavelet-LHH	firstorder	Energy	3400135.22
wavelet-LHH	firstorder	RobustMeanAbsoluteDeviation	48.70483066
wavelet-LHH	firstorder	MeanAbsoluteDeviation	86.67759289
wavelet-LHH	firstorder	TotalEnergy	91803650.93
wavelet-LHH	firstorder	Maximum	814.7863951
wavelet-LHH	firstorder	RootMeanSquared	131.3756813
wavelet-LHH	firstorder	90Percentile	134.1296665
wavelet-LHH	firstorder	Minimum	-441.9530127
wavelet-LHH	firstorder	Entropy	4.090008593
wavelet-LHH	firstorder	Range	1256.739408
wavelet-LHH	firstorder	Variance	17254.75112
wavelet-LHH	firstorder	10Percentile	-131.1529973
wavelet-LHH	firstorder	Kurtosis	10.74887411
wavelet-LHH	firstorder	Mean	2.195113957
wavelet-LHH	glrlm	ShortRunLowGrayLevelEmphasis	0.009705144
wavelet-LHH	glrlm	GrayLevelVariance	29.8169387
wavelet-LHH	glrlm	LowGrayLevelRunEmphasis	0.009862431
wavelet-LHH	glrlm	GrayLevelNonUniformityNormalized	0.071450808
wavelet-LHH	glrlm	RunVariance	0.092097222
wavelet-LHH	glrlm	GrayLevelNonUniformity	13.07364417
wavelet-LHH	glrlm	LongRunEmphasis	1.253304244
wavelet-LHH	glrlm	ShortRunHighGrayLevelEmphasis	356.1626353
wavelet-LHH	glrlm	RunLengthNonUniformity	159.9125029
wavelet-LHH	glrlm	ShortRunEmphasis	0.947991207
wavelet-LHH	glrlm	LongRunHighGrayLevelEmphasis	463.1306191
wavelet-LHH	glrlm	RunPercentage	0.928543538
wavelet-LHH	glrlm	LongRunLowGrayLevelEmphasis	0.010622312
wavelet-LHH	glrlm	RunEntropy	4.428453568
wavelet-LHH	glrlm	HighGrayLevelRunEmphasis	374.3269172
wavelet-LHH	glrlm	RunLengthNonUniformityNormalized	0.873572677
wavelet-LHH	glszm	GrayLevelVariance	51.71978271
wavelet-LHH	glszm	ZoneVariance	12.7674287
wavelet-LHH	glszm	GrayLevelNonUniformityNormalized	0.050475328
wavelet-LHH	glszm	SizeZoneNonUniformityNormalized	0.588727931
wavelet-LHH	glszm	SizeZoneNonUniformity	55.34042553
wavelet-LHH	glszm	GrayLevelNonUniformity	4.744680851
wavelet-LHH	glszm	LargeAreaEmphasis	17.15957447
wavelet-LHH	glszm	SmallAreaHighGrayLevelEmphasis	307.3286026
wavelet-LHH	glszm	ZonePercentage	0.47715736
wavelet-LHH	glszm	LargeAreaLowGrayLevelEmphasis	0.065332837
wavelet-LHH	glszm	LargeAreaHighGrayLevelEmphasis	5872.287234
wavelet-LHH	glszm	HighGrayLevelZoneEmphasis	386.5319149
wavelet-LHH	glszm	SmallAreaEmphasis	0.792523431
wavelet-LHH	glszm	LowGrayLevelZoneEmphasis	0.016348891
wavelet-LHH	glszm	ZoneEntropy	5.280667512
wavelet-LHH	glszm	SmallAreaLowGrayLevelEmphasis	0.015679077
wavelet-LHH	ngtdm	Coarseness	0.033938838
wavelet-LHH	ngtdm	Complexity	1236.789644

wavelet-LHH	ngtdm	Strength	10.60673973
wavelet-LHH	ngtdm	Contrast	0.29316488
wavelet-LHH	ngtdm	Busyness	0.053291952
wavelet-LLH	gldm	GrayLevelVariance	138.5651266
wavelet-LLH	gldm	HighGrayLevelEmphasis	2608.705584
wavelet-LLH	gldm	DependenceEntropy	6.393866525
wavelet-LLH	gldm	DependenceNonUniformity	57.81218274
wavelet-LLH	gldm	GrayLevelNonUniformity	8.076142132
wavelet-LLH	gldm	SmallDependenceEmphasis	0.536691342
wavelet-LLH	gldm	SmallDependenceHighGrayLevelEmphasis	1448.392523
wavelet-LLH	gldm	DependenceNonUniformityNormalized	0.293462857
wavelet-LLH	gldm	LargeDependenceEmphasis	6.85786802
wavelet-LLH	gldm	LargeDependenceLowGrayLevelEmphasis	0.008088113
wavelet-LLH	gldm	DependenceVariance	2.159756757
wavelet-LLH	gldm	LargeDependenceHighGrayLevelEmphasis	17243.80203
wavelet-LLH	gldm	SmallDependenceLowGrayLevelEmphasis	0.005492819
wavelet-LLH	gldm	LowGrayLevelEmphasis	0.005688774
wavelet-LLH	glcm	JointAverage	50.10363873
wavelet-LLH	glcm	SumAverage	100.2072775
wavelet-LLH	glcm	JointEntropy	7.46337174
wavelet-LLH	glcm	ClusterShade	-1216.370915
wavelet-LLH	glcm	MaximumProbability	0.019684314
wavelet-LLH	glcm	Idmn	0.97516286
wavelet-LLH	glcm	JointEnergy	0.006781972
wavelet-LLH	glcm	Contrast	206.566985
wavelet-LLH	glcm	DifferenceEntropy	4.275801963
wavelet-LLH	glcm	InverseVariance	0.184267241
wavelet-LLH	glcm	DifferenceVariance	119.9786202
wavelet-LLH	glcm	Idn	0.912565685
wavelet-LLH	glcm	Idm	0.175218788
wavelet-LLH	glcm	Correlation	0.078734106
wavelet-LLH	glcm	Autocorrelation	2522.913821
wavelet-LLH	glcm	SumEntropy	5.192543166
wavelet-LLH	glcm	SumSquares	115.7082183
wavelet-LLH	glcm	ClusterProminence	489734.2798
wavelet-LLH	glcm	Imc2	0.993829999
wavelet-LLH	glcm	Imc1	-0.45761171
wavelet-LLH	glcm	DifferenceAverage	8.941604881
wavelet-LLH	glcm	Id	0.254088825
wavelet-LLH	glcm	ClusterTendency	256.2658882
wavelet-LLH	firstorder	InterquartileRange	211.2293242
wavelet-LLH	firstorder	Skewness	-0.456216589
wavelet-LLH	firstorder	Uniformity	0.040995645
wavelet-LLH	firstorder	Median	-25.87987158
wavelet-LLH	firstorder	Energy	17149865.25
wavelet-LLH	firstorder	RobustMeanAbsoluteDeviation	102.0890997
wavelet-LLH	firstorder	MeanAbsoluteDeviation	194.2413616
wavelet-LLH	firstorder	TotalEnergy	463046361.8
wavelet-LLH	firstorder	Maximum	799.0397481
wavelet-LLH	firstorder	RootMeanSquared	295.0511033
wavelet-LLH	firstorder	90Percentile	376.5801691
wavelet-LLH	firstorder	Minimum	-1238.859506
wavelet-LLH	firstorder	Entropy	5.080808451
wavelet-LLH	firstorder	Range	2037.899254
wavelet-LLH	firstorder	Variance	86682.26667

wavelet-LLH	firstorder	10Percentile	-307.8457805
wavelet-LLH	firstorder	Kurtosis	5.993708862
wavelet-LLH	firstorder	Mean	-19.31027943
wavelet-LLH	glrlm	ShortRunLowGrayLevelEmphasis	0.005923667
wavelet-LLH	glrlm	GrayLevelVariance	144.1344174
wavelet-LLH	glrlm	LowGrayLevelRunEmphasis	0.005937942
wavelet-LLH	glrlm	GrayLevelNonUniformityNormalized	0.039289176
wavelet-LLH	glrlm	RunVariance	0.048066625
wavelet-LLH	glrlm	GrayLevelNonUniformity	7.393687848
wavelet-LLH	glrlm	LongRunEmphasis	1.145026647
wavelet-LLH	glrlm	ShortRunHighGrayLevelEmphasis	2528.068126
wavelet-LLH	glrlm	RunLengthNonUniformity	171.912087
wavelet-LLH	glrlm	ShortRunEmphasis	0.965574085
wavelet-LLH	glrlm	LongRunHighGrayLevelEmphasis	2975.775514
wavelet-LLH	glrlm	RunPercentage	0.955095666
wavelet-LLH	glrlm	LongRunLowGrayLevelEmphasis	0.005998084
wavelet-LLH	glrlm	RunEntropy	5.302029917
wavelet-LLH	glrlm	HighGrayLevelRunEmphasis	2614.046608
wavelet-LLH	glrlm	RunLengthNonUniformityNormalized	0.913288802
wavelet-LLH	glszm	GrayLevelVariance	210.478356
wavelet-LLH	glszm	ZoneVariance	3.805663442
wavelet-LLH	glszm	GrayLevelNonUniformityNormalized	0.027611044
wavelet-LLH	glszm	SizeZoneNonUniformityNormalized	0.584351388
wavelet-LLH	glszm	SizeZoneNonUniformity	69.53781513
wavelet-LLH	glszm	GrayLevelNonUniformity	3.285714286
wavelet-LLH	glszm	LargeAreaEmphasis	6.546218487
wavelet-LLH	glszm	SmallAreaHighGrayLevelEmphasis	2154.861159
wavelet-LLH	glszm	ZonePercentage	0.604060914
wavelet-LLH	glszm	LargeAreaLowGrayLevelEmphasis	0.011411626
wavelet-LLH	glszm	LargeAreaHighGrayLevelEmphasis	16510.69748
wavelet-LLH	glszm	HighGrayLevelZoneEmphasis	2684.495798
wavelet-LLH	glszm	SmallAreaEmphasis	0.791368292
wavelet-LLH	glszm	LowGrayLevelZoneEmphasis	0.009141797
wavelet-LLH	glszm	ZoneEntropy	6.097390427
wavelet-LLH	glszm	SmallAreaLowGrayLevelEmphasis	0.009051269
wavelet-LLH	ngtdm	Coarseness	0.033707954
wavelet-LLH	ngtdm	Complexity	8987.352237
wavelet-LLH	ngtdm	Strength	40.60959668
wavelet-LLH	ngtdm	Contrast	0.674753121
wavelet-LLH	ngtdm	Busyness	0.010369878
wavelet-LLL	gldm	GrayLevelVariance	430.0213868
wavelet-LLL	gldm	HighGrayLevelEmphasis	3528.045685
wavelet-LLL	gldm	DependenceEntropy	6.405869205
wavelet-LLL	gldm	DependenceNonUniformity	91.64974619
wavelet-LLL	gldm	GrayLevelNonUniformity	3.944162437
wavelet-LLL	gldm	SmallDependenceEmphasis	0.702711506
wavelet-LLL	gldm	SmallDependenceHighGrayLevelEmphasis	2115.364796
wavelet-LLL	gldm	DependenceNonUniformityNormalized	0.465227138
wavelet-LLL	gldm	LargeDependenceEmphasis	3.131979695
wavelet-LLL	gldm	LargeDependenceLowGrayLevelEmphasis	0.006410156
wavelet-LLL	gldm	DependenceVariance	0.734984153
wavelet-LLL	gldm	LargeDependenceHighGrayLevelEmphasis	14093.91371
wavelet-LLL	gldm	SmallDependenceLowGrayLevelEmphasis	0.00568084
wavelet-LLL	gldm	LowGrayLevelEmphasis	0.005780089
wavelet-LLL	glcm	JointAverage	60.33985226

wavelet-LLL	glcm	SumAverage	120.6797045
wavelet-LLL	glcm	JointEntropy	7.925628791
wavelet-LLL	glcm	ClusterShade	-20079.82589
wavelet-LLL	glcm	MaximumProbability	0.010373271
wavelet-LLL	glcm	Idmn	0.96198981
wavelet-LLL	glcm	JointEnergy	0.004307781
wavelet-LLL	glcm	Contrast	437.2061458
wavelet-LLL	glcm	DifferenceEntropy	5.040496515
wavelet-LLL	glcm	InverseVariance	0.087399874
wavelet-LLL	glcm	DifferenceVariance	171.487599
wavelet-LLL	glcm	Idn	0.874034558
wavelet-LLL	glcm	Idm	0.083145656
wavelet-LLL	glcm	Correlation	0.400679155
wavelet-LLL	glcm	Autocorrelation	3787.961829
wavelet-LLL	glcm	SumEntropy	6.081322932
wavelet-LLL	glcm	SumSquares	364.7208964
wavelet-LLL	glcm	ClusterProminence	2703677.614
wavelet-LLL	glcm	Imc2	0.999404876
wavelet-LLL	glcm	Imc1	-0.597169357
wavelet-LLL	glcm	DifferenceAverage	15.97781763
wavelet-LLL	glcm	Id	0.149402561
wavelet-LLL	glcm	ClusterTendency	1021.67744
wavelet-LLL	firstorder	InterquartileRange	809.1114347
wavelet-LLL	firstorder	Skewness	-0.357112931
wavelet-LLL	firstorder	Uniformity	0.020021129
wavelet-LLL	firstorder	Median	-421.2510613
wavelet-LLL	firstorder	Energy	96617423.67
wavelet-LLL	firstorder	RobustMeanAbsoluteDeviation	356.2907405
wavelet-LLL	firstorder	MeanAbsoluteDeviation	450.0437911
wavelet-LLL	firstorder	TotalEnergy	2608670439
wavelet-LLL	firstorder	Maximum	664.7857729
wavelet-LLL	firstorder	RootMeanSquared	700.3169104
wavelet-LLL	firstorder	90Percentile	119.3635116
wavelet-LLL	firstorder	Minimum	-1845.726933
wavelet-LLL	firstorder	Entropy	5.847639462
wavelet-LLL	firstorder	Range	2510.512706
wavelet-LLL	firstorder	Variance	268614.5188
wavelet-LLL	firstorder	10Percentile	-1186.500806
wavelet-LLL	firstorder	Kurtosis	2.154267435
wavelet-LLL	firstorder	Mean	-470.9875329
wavelet-LLL	glrlm	ShortRunLowGrayLevelEmphasis	0.005894069
wavelet-LLL	glrlm	GrayLevelVariance	430.5552632
wavelet-LLL	glrlm	LowGrayLevelRunEmphasis	0.005899035
wavelet-LLL	glrlm	GrayLevelNonUniformityNormalized	0.01980905
wavelet-LLL	glrlm	RunVariance	0.024349055
wavelet-LLL	glrlm	GrayLevelNonUniformity	3.820509362
wavelet-LLL	glrlm	LongRunEmphasis	1.068553166
wavelet-LLL	glrlm	ShortRunHighGrayLevelEmphasis	3424.458029
wavelet-LLL	glrlm	RunLengthNonUniformity	185.2091145
wavelet-LLL	glrlm	ShortRunEmphasis	0.984474702
wavelet-LLL	glrlm	LongRunHighGrayLevelEmphasis	3836.562174
wavelet-LLL	glrlm	RunPercentage	0.978914487
wavelet-LLL	glrlm	LongRunLowGrayLevelEmphasis	0.005919954
wavelet-LLL	glrlm	RunEntropy	5.918397146
wavelet-LLL	glrlm	HighGrayLevelRunEmphasis	3498.992844

wavelet-LLL	glrlm	RunLengthNonUniformityNormalized	0.960057916
wavelet-LLL	glszm	GrayLevelVariance	425.6789614
wavelet-LLL	glszm	ZoneVariance	0.635673874
wavelet-LLL	glszm	GrayLevelNonUniformityNormalized	0.018288672
wavelet-LLL	glszm	SizeZoneNonUniformityNormalized	0.687996141
wavelet-LLL	glszm	SizeZoneNonUniformity	103.8874172
wavelet-LLL	glszm	GrayLevelNonUniformity	2.761589404
wavelet-LLL	glszm	LargeAreaEmphasis	2.337748344
wavelet-LLL	glszm	SmallAreaHighGrayLevelEmphasis	2464.497265
wavelet-LLL	glszm	ZonePercentage	0.766497462
wavelet-LLL	glszm	LargeAreaLowGrayLevelEmphasis	0.007857482
wavelet-LLL	glszm	LargeAreaHighGrayLevelEmphasis	9655.503311
wavelet-LLL	glszm	HighGrayLevelZoneEmphasis	3147.615894
wavelet-LLL	glszm	SmallAreaEmphasis	0.853960633
wavelet-LLL	glszm	LowGrayLevelZoneEmphasis	0.007440113
wavelet-LLL	glszm	ZoneEntropy	6.211526262
wavelet-LLL	glszm	SmallAreaLowGrayLevelEmphasis	0.007389177
wavelet-LLL	ngtdm	Coarseness	0.024760097
wavelet-LLL	ngtdm	Complexity	23669.57008
wavelet-LLL	ngtdm	Strength	60.08799712
wavelet-LLL	ngtdm	Contrast	2.136365228
wavelet-LLL	ngtdm	Busyness	0.011418085
wavelet-HHL	gldm	GrayLevelVariance	18.46324306
wavelet-HHL	gldm	HighGrayLevelEmphasis	156.9137056
wavelet-HHL	gldm	DependenceEntropy	5.719232234
wavelet-HHL	gldm	DependenceNonUniformity	46.16751269
wavelet-HHL	gldm	GrayLevelNonUniformity	15.55837563
wavelet-HHL	gldm	SmallDependenceEmphasis	0.369621275
wavelet-HHL	gldm	SmallDependenceHighGrayLevelEmphasis	64.0352143
wavelet-HHL	gldm	DependenceNonUniformityNormalized	0.234352856
wavelet-HHL	gldm	LargeDependenceEmphasis	8.644670051
wavelet-HHL	gldm	LargeDependenceLowGrayLevelEmphasis	0.133103805
wavelet-HHL	gldm	DependenceVariance	2.125331753
wavelet-HHL	gldm	LargeDependenceHighGrayLevelEmphasis	1151.187817
wavelet-HHL	gldm	SmallDependenceLowGrayLevelEmphasis	0.009086935
wavelet-HHL	gldm	LowGrayLevelEmphasis	0.024099305
wavelet-HHL	glcm	JointAverage	11.77855459
wavelet-HHL	glcm	SumAverage	23.55710918
wavelet-HHL	glcm	JointEntropy	6.925244524
wavelet-HHL	glcm	ClusterShade	82.42460941
wavelet-HHL	glcm	MaximumProbability	0.030114263
wavelet-HHL	glcm	Idmn	0.950103453
wavelet-HHL	glcm	JointEnergy	0.010357792
wavelet-HHL	glcm	Contrast	34.09865124
wavelet-HHL	glcm	DifferenceEntropy	3.525419638
wavelet-HHL	glcm	InverseVariance	0.213944277
wavelet-HHL	glcm	DifferenceVariance	13.74285575
wavelet-HHL	glcm	Idn	0.856543602
wavelet-HHL	glcm	Idm	0.216985231
wavelet-HHL	glcm	Correlation	0.085160409
wavelet-HHL	glcm	Autocorrelation	140.4764956
wavelet-HHL	glcm	SumEntropy	4.477065914
wavelet-HHL	glcm	SumSquares	18.75490636
wavelet-HHL	glcm	ClusterProminence	5711.770594
wavelet-HHL	glcm	Imc2	0.930768627

wavelet-HHL	glcm	Imc1	-0.255670906
wavelet-HHL	glcm	DifferenceAverage	4.461665842
wavelet-HHL	glcm	Id	0.30815037
wavelet-HHL	glcm	ClusterTendency	40.92097419
wavelet-HHL	firstorder	InterquartileRange	133.971468
wavelet-HHL	firstorder	Skewness	0.325195074
wavelet-HHL	firstorder	Uniformity	0.078976526
wavelet-HHL	firstorder	Median	-4.451016223
wavelet-HHL	firstorder	Energy	2261781.053
wavelet-HHL	firstorder	RobustMeanAbsoluteDeviation	53.04223401
wavelet-HHL	firstorder	MeanAbsoluteDeviation	81.64969717
wavelet-HHL	firstorder	TotalEnergy	61068088.44
wavelet-HHL	firstorder	Maximum	307.8501821
wavelet-HHL	firstorder	RootMeanSquared	107.1499981
wavelet-HHL	firstorder	90Percentile	144.7826457
wavelet-HHL	firstorder	Minimum	-264.0223822
wavelet-HHL	firstorder	Entropy	3.981918407
wavelet-HHL	firstorder	Range	571.8725644
wavelet-HHL	firstorder	Variance	11449.32844
wavelet-HHL	firstorder	10Percentile	-106.5432351
wavelet-HHL	firstorder	Kurtosis	3.520212896
wavelet-HHL	firstorder	Mean	5.638586296
wavelet-HHL	glrlm	ShortRunLowGrayLevelEmphasis	0.023686053
wavelet-HHL	glrlm	GrayLevelVariance	18.94795735
wavelet-HHL	glrlm	LowGrayLevelRunEmphasis	0.024509512
wavelet-HHL	glrlm	GrayLevelNonUniformityNormalized	0.076925899
wavelet-HHL	glrlm	RunVariance	0.074612628
wavelet-HHL	glrlm	GrayLevelNonUniformity	14.25079586
wavelet-HHL	glrlm	LongRunEmphasis	1.209500616
wavelet-HHL	glrlm	ShortRunHighGrayLevelEmphasis	151.7767161
wavelet-HHL	glrlm	RunLengthNonUniformity	165.3168871
wavelet-HHL	glrlm	ShortRunEmphasis	0.955294504
wavelet-HHL	glrlm	LongRunHighGrayLevelEmphasis	186.562618
wavelet-HHL	glrlm	RunPercentage	0.940257712
wavelet-HHL	glrlm	LongRunLowGrayLevelEmphasis	0.028113173
wavelet-HHL	glrlm	RunEntropy	4.267734189
wavelet-HHL	glrlm	HighGrayLevelRunEmphasis	158.1035275
wavelet-HHL	glrlm	RunLengthNonUniformityNormalized	0.890828357
wavelet-HHL	glszm	GrayLevelVariance	26.44074514
wavelet-HHL	glszm	ZoneVariance	7.366891267
wavelet-HHL	glszm	GrayLevelNonUniformityNormalized	0.058263971
wavelet-HHL	glszm	SizeZoneNonUniformityNormalized	0.382216938
wavelet-HHL	glszm	SizeZoneNonUniformity	33.25287356
wavelet-HHL	glszm	GrayLevelNonUniformity	5.068965517
wavelet-HHL	glszm	LargeAreaEmphasis	12.49425287
wavelet-HHL	glszm	SmallAreaHighGrayLevelEmphasis	115.2012869
wavelet-HHL	glszm	ZonePercentage	0.441624365
wavelet-HHL	glszm	LargeAreaLowGrayLevelEmphasis	0.182431966
wavelet-HHL	glszm	LargeAreaHighGrayLevelEmphasis	1570.413793
wavelet-HHL	glszm	HighGrayLevelZoneEmphasis	173.7701149
wavelet-HHL	glszm	SmallAreaEmphasis	0.636893192
wavelet-HHL	glszm	LowGrayLevelZoneEmphasis	0.029079777
wavelet-HHL	glszm	ZoneEntropy	5.417434519
wavelet-HHL	glszm	SmallAreaLowGrayLevelEmphasis	0.015240617
wavelet-HHL	ngtdm	Coarseness	0.030592674

wavelet-HHL	ngtdm	Complexity	654.2606951
wavelet-HHL	ngtdm	Strength	5.28546076
wavelet-HHL	ngtdm	Contrast	0.206363839
wavelet-HHL	ngtdm	Busyness	0.115892488
wavelet-HLH	gldm	GrayLevelVariance	18.27163802
wavelet-HLH	gldm	HighGrayLevelEmphasis	176.8781726
wavelet-HLH	gldm	DependenceEntropy	5.830056899
wavelet-HLH	gldm	DependenceNonUniformity	42.82741117
wavelet-HLH	gldm	GrayLevelNonUniformity	15.06091371
wavelet-HLH	gldm	SmallDependenceEmphasis	0.393542818
wavelet-HLH	gldm	SmallDependenceHighGrayLevelEmphasis	76.83664883
wavelet-HLH	gldm	DependenceNonUniformityNormalized	0.217398026
wavelet-HLH	gldm	LargeDependenceEmphasis	10.23857868
wavelet-HLH	gldm	LargeDependenceLowGrayLevelEmphasis	0.119053282
wavelet-HLH	gldm	DependenceVariance	3.027854364
wavelet-HLH	gldm	LargeDependenceHighGrayLevelEmphasis	1632.035533
wavelet-HLH	gldm	SmallDependenceLowGrayLevelEmphasis	0.007808082
wavelet-HLH	gldm	LowGrayLevelEmphasis	0.020594446
wavelet-HLH	glcm	JointAverage	12.55580872
wavelet-HLH	glcm	SumAverage	25.11161744
wavelet-HLH	glcm	JointEntropy	6.881322497
wavelet-HLH	glcm	ClusterShade	12.51119193
wavelet-HLH	glcm	MaximumProbability	0.028262283
wavelet-HLH	glcm	Idmn	0.95723117
wavelet-HLH	glcm	JointEnergy	0.010756925
wavelet-HLH	glcm	Contrast	34.60427646
wavelet-HLH	glcm	DifferenceEntropy	3.493771445
wavelet-HLH	glcm	InverseVariance	0.244532558
wavelet-HLH	glcm	DifferenceVariance	15.60613918
wavelet-HLH	glcm	Idn	0.8709422
wavelet-HLH	glcm	Idm	0.238899539
wavelet-HLH	glcm	Correlation	0.020524317
wavelet-HLH	glcm	Autocorrelation	158.0622337
wavelet-HLH	glcm	SumEntropy	4.370068577
wavelet-HLH	glcm	SumSquares	17.71185329
wavelet-HLH	glcm	ClusterProminence	5533.987619
wavelet-HLH	glcm	Imc2	0.926221786
wavelet-HLH	glcm	Imc1	-0.250630544
wavelet-HLH	glcm	DifferenceAverage	4.306422908
wavelet-HLH	glcm	Id	0.325549496
wavelet-HLH	glcm	ClusterTendency	36.24313672
wavelet-HLH	firstorder	InterquartileRange	118.2118692
wavelet-HLH	firstorder	Skewness	0.244359893
wavelet-HLH	firstorder	Uniformity	0.076451339
wavelet-HLH	firstorder	Median	-3.317269378
wavelet-HLH	firstorder	Energy	2255375.925
wavelet-HLH	firstorder	RobustMeanAbsoluteDeviation	50.66524942
wavelet-HLH	firstorder	MeanAbsoluteDeviation	80.38952216
wavelet-HLH	firstorder	TotalEnergy	60895149.99
wavelet-HLH	firstorder	Maximum	333.9197657
wavelet-HLH	firstorder	RootMeanSquared	106.9981718
wavelet-HLH	firstorder	90Percentile	130.4971307
wavelet-HLH	firstorder	Minimum	-299.0084681
wavelet-HLH	firstorder	Entropy	3.990757248
wavelet-HLH	firstorder	Range	632.9282337

wavelet-HLH	firstorder	Variance	11443.5959
wavelet-HLH	firstorder	10Percentile	-117.8769787
wavelet-HLH	firstorder	Kurtosis	3.906765754
wavelet-HLH	firstorder	Mean	2.238940437
wavelet-HLH	glrlm	ShortRunLowGrayLevelEmphasis	0.020345191
wavelet-HLH	glrlm	GrayLevelVariance	18.99961097
wavelet-HLH	glrlm	LowGrayLevelRunEmphasis	0.021059597
wavelet-HLH	glrlm	GrayLevelNonUniformityNormalized	0.074266463
wavelet-HLH	glrlm	RunVariance	0.074529983
wavelet-HLH	glrlm	GrayLevelNonUniformity	13.68358171
wavelet-HLH	glrlm	LongRunEmphasis	1.221035442
wavelet-HLH	glrlm	ShortRunHighGrayLevelEmphasis	169.9297497
wavelet-HLH	glrlm	RunLengthNonUniformity	162.2372061
wavelet-HLH	glrlm	ShortRunEmphasis	0.950422817
wavelet-HLH	glrlm	LongRunHighGrayLevelEmphasis	213.4578153
wavelet-HLH	glrlm	RunPercentage	0.93518157
wavelet-HLH	glrlm	LongRunLowGrayLevelEmphasis	0.024077953
wavelet-HLH	glrlm	RunEntropy	4.30591975
wavelet-HLH	glrlm	HighGrayLevelRunEmphasis	177.9749829
wavelet-HLH	glrlm	RunLengthNonUniformityNormalized	0.879109653
wavelet-HLH	glszm	GrayLevelVariance	27.63654321
wavelet-HLH	glszm	ZoneVariance	8.597654321
wavelet-HLH	glszm	GrayLevelNonUniformityNormalized	0.058024691
wavelet-HLH	glszm	SizeZoneNonUniformityNormalized	0.444691358
wavelet-HLH	glszm	SizeZoneNonUniformity	40.02222222
wavelet-HLH	glszm	GrayLevelNonUniformity	5.222222222
wavelet-HLH	glszm	LargeAreaEmphasis	13.38888889
wavelet-HLH	glszm	SmallAreaHighGrayLevelEmphasis	140.4058349
wavelet-HLH	glszm	ZonePercentage	0.456852792
wavelet-HLH	glszm	LargeAreaLowGrayLevelEmphasis	0.154819347
wavelet-HLH	glszm	LargeAreaHighGrayLevelEmphasis	1989.455556
wavelet-HLH	glszm	HighGrayLevelZoneEmphasis	194.3333333
wavelet-HLH	glszm	SmallAreaEmphasis	0.691508012
wavelet-HLH	glszm	LowGrayLevelZoneEmphasis	0.024224079
wavelet-HLH	glszm	ZoneEntropy	5.258124625
wavelet-HLH	glszm	SmallAreaLowGrayLevelEmphasis	0.012750138
wavelet-HLH	ngtdm	Coarseness	0.03170504
wavelet-HLH	ngtdm	Complexity	646.0490212
wavelet-HLH	ngtdm	Strength	5.058705563
wavelet-HLH	ngtdm	Contrast	0.229614405
wavelet-HLH	ngtdm	Busyness	0.113775783

Image type	Feature Class	Feature Name	516: RTSTRUCT: C99-DNU-A_segment_ITV 2 VV
general	info	BoundingBox	(0, 1, 1, 4, 4, 3)
general	info	EnabledImageTypes	{u'Original': {}, u'Wavelet': {}}
general	info	GeneralSettings	{'distances': [1], 'additionalInfo': True, 'force2D': False, 'interpolator': 'sitkBSpline', 'resampledPixelSpacing': [3.0], 'label': 1, 'normalizeScale': 1, 'normalize': False, 'force2Ddimension': 0, 'removeOutliers': None, 'minimumROISize': None, u'binWidth': 25.0, 'minimumROIDimensions': 1, u'correctMask': True, 'preCrop': False, u'symmetricalGLCM': True, 'resegmentRange': None, 'padDistance': 5}
general	info	ImageHash	4c6240de0f6117db1228d81e5dcef82752837487
general	info	ImageSpacing	(0.9765625000000002, 0.9765625000000002, 2.0)
general	info	MaskHash	ca8b648c7c82d383da4fbde908ce67d5797735eb
general	info	NumpyVersion	1.13.1
general	info	PyWaveletVersion	0.5.2
general	info	SimpleITKVersion	1.1.0
general	info	Version	1.3.0.post102+g1b0eb74
general	info	VolumeNum	1
general	info	VoxelNum	19
original	shape	Maximum3DDiameter	10.39230485
original	shape	Maximum2DDiameterSlice	9.486832981
original	shape	Sphericity	0.981107832
original	shape	MinorAxis	8.48860303
original	shape	Elongation	0.768400872
original	shape	SurfaceVolumeRatio	0.61573654
original	shape	Volume	513
original	shape	MajorAxis	11.04710229
original	shape	SurfaceArea	315.872845
original	shape	Flatness	0.694144837
original	shape	LeastAxis	7.668289019
original	shape	Maximum2DDiameterColumn	8.485281374
original	shape	Maximum2DDiameterRow	9.486832981
original	gldm	GrayLevelVariance	10.7700831
original	gldm	HighGrayLevelEmphasis	65.84210526
original	gldm	DependenceEntropy	3.471354487
original	gldm	DependenceNonUniformity	8.684210526
original	gldm	GrayLevelNonUniformity	2.052631579
original	gldm	SmallDependenceEmphasis	0.558479532
original	gldm	SmallDependenceHighGrayLevelEmphasis	30.78947368
original	gldm	DependenceNonUniformityNormalized	0.457063712
original	gldm	LargeDependenceEmphasis	3
original	gldm	LargeDependenceLowGrayLevelEmphasis	0.462793526
original	gldm	DependenceVariance	0.337950139
original	gldm	LargeDependenceHighGrayLevelEmphasis	225
original	gldm	SmallDependenceLowGrayLevelEmphasis	0.043348263
original	gldm	LowGrayLevelEmphasis	0.12665974
original	glcm	JointAverage	7.76023421
original	glcm	SumAverage	15.52046842
original	glcm	JointEntropy	3.676768938
original	glcm	ClusterShade	-45.05131998
original	glcm	MaximumProbability	0.107678433
original	glcm	Idmn	0.893673304
original	glcm	JointEnergy	0.084902617

original	glcm	Contrast	20.74061494
original	glcm	DifferenceEntropy	2.160416721
original	glcm	InverseVariance	0.261730353
original	glcm	DifferenceVariance	6.557449591
original	glcm	Idn	0.791346842
original	glcm	Idm	0.237617908
original	glcm	Correlation	-0.000858293
original	glcm	Autocorrelation	60.62113442
original	glcm	SumEntropy	2.543241354
original	glcm	SumSquares	10.57135603
original	glcm	ClusterProminence	1449.507352
original	glcm	Imc2	0.99577651
original	glcm	Imc1	-0.806678521
original	glcm	DifferenceAverage	3.60997891
original	glcm	Id	0.329227063
original	glcm	ClusterTendency	21.54480919
original	firstorder	InterquartileRange	107
original	firstorder	Skewness	-0.488481503
original	firstorder	Uniformity	0.108033241
original	firstorder	Median	-64
original	firstorder	Energy	246277
original	firstorder	RobustMeanAbsoluteDeviation	46.45333333
original	firstorder	MeanAbsoluteDeviation	66.52077562
original	firstorder	TotalEnergy	6649479
original	firstorder	Maximum	43
original	firstorder	RootMeanSquared	113.8505484
original	firstorder	90Percentile	19
original	firstorder	Minimum	-241
original	firstorder	Entropy	3.326360408
original	firstorder	Range	284
original	firstorder	Variance	6620.759003
original	firstorder	10Percentile	-190
original	firstorder	Kurtosis	2.383111789
original	firstorder	Mean	-79.63157895
original	glrlm	ShortRunLowGrayLevelEmphasis	0.121226653
original	glrlm	GrayLevelVariance	10.68460644
original	glrlm	LowGrayLevelRunEmphasis	0.125033976
original	glrlm	GrayLevelNonUniformityNormalized	0.108371897
original	glrlm	RunVariance	0.023827012
original	glrlm	GrayLevelNonUniformity	2.008659469
original	glrlm	LongRunEmphasis	1.081730769
original	glrlm	ShortRunHighGrayLevelEmphasis	63.87880258
original	glrlm	RunLengthNonUniformity	17.7275641
original	glrlm	ShortRunEmphasis	0.979567308
original	glrlm	LongRunHighGrayLevelEmphasis	71.92968399
original	glrlm	RunPercentage	0.975708502
original	glrlm	LongRunLowGrayLevelEmphasis	0.140263267
original	glrlm	RunEntropy	3.343686539
original	glrlm	HighGrayLevelRunEmphasis	65.48897886
original	glrlm	RunLengthNonUniformityNormalized	0.952345976
original	glszm	GrayLevelVariance	9.455621302
original	glszm	ZoneVariance	0.402366864
original	glszm	GrayLevelNonUniformityNormalized	0.112426036
original	glszm	SizeZoneNonUniformityNormalized	0.479289941
original	glszm	SizeZoneNonUniformity	6.230769231
original	glszm	GrayLevelNonUniformity	1.461538462

original	glszm	LargeAreaEmphasis	2.538461538
original	glszm	SmallAreaHighGrayLevelEmphasis	35.82692308
original	glszm	ZonePercentage	0.684210526
original	glszm	LargeAreaLowGrayLevelEmphasis	0.350141791
original	glszm	LargeAreaHighGrayLevelEmphasis	182.0769231
original	glszm	HighGrayLevelZoneEmphasis	59.53846154
original	glszm	SmallAreaEmphasis	0.700854701
original	glszm	LowGrayLevelZoneEmphasis	0.103555895
original	glszm	ZoneEntropy	3.334679141
original	glszm	SmallAreaLowGrayLevelEmphasis	0.042964608
original	ngtdm	Coarseness	0.234095746
original	ngtdm	Complexity	127.1087024
original	ngtdm	Strength	10.33935376
original	ngtdm	Contrast	0.482977321
original	ngtdm	Busyness	0.065243868
wavelet-HHH	gldm	GrayLevelVariance	10.24930748
wavelet-HHH	gldm	HighGrayLevelEmphasis	121.0526316
wavelet-HHH	gldm	DependenceEntropy	3.300559092
wavelet-HHH	gldm	DependenceNonUniformity	6.789473684
wavelet-HHH	gldm	GrayLevelNonUniformity	2.578947368
wavelet-HHH	gldm	SmallDependenceEmphasis	0.486842105
wavelet-HHH	gldm	SmallDependenceHighGrayLevelEmphasis	62.90789474
wavelet-HHH	gldm	DependenceNonUniformityNormalized	0.35734072
wavelet-HHH	gldm	LargeDependenceEmphasis	5.421052632
wavelet-HHH	gldm	LargeDependenceLowGrayLevelEmphasis	0.110999443
wavelet-HHH	gldm	DependenceVariance	1.207756233
wavelet-HHH	gldm	LargeDependenceHighGrayLevelEmphasis	545.4736842
wavelet-HHH	gldm	SmallDependenceLowGrayLevelEmphasis	0.057289836
wavelet-HHH	gldm	LowGrayLevelEmphasis	0.062183804
wavelet-HHH	glcm	JointAverage	10.59424187
wavelet-HHH	glcm	SumAverage	21.18848374
wavelet-HHH	glcm	JointEntropy	3.570070081
wavelet-HHH	glcm	ClusterShade	-53.74613218
wavelet-HHH	glcm	MaximumProbability	0.120668221
wavelet-HHH	glcm	Idmn	0.935539374
wavelet-HHH	glcm	JointEnergy	0.089625719
wavelet-HHH	glcm	Contrast	19.38575314
wavelet-HHH	glcm	DifferenceEntropy	2.18872707
wavelet-HHH	glcm	InverseVariance	0.305454131
wavelet-HHH	glcm	DifferenceVariance	8.382440671
wavelet-HHH	glcm	Idn	0.842773643
wavelet-HHH	glcm	Idm	0.277913724
wavelet-HHH	glcm	Correlation	-0.060559446
wavelet-HHH	glcm	Autocorrelation	111.7433178
wavelet-HHH	glcm	SumEntropy	2.430740864
wavelet-HHH	glcm	SumSquares	8.868567433
wavelet-HHH	glcm	ClusterProminence	1045.064551
wavelet-HHH	glcm	Imc2	0.986497407
wavelet-HHH	glcm	Imc1	-0.688975119
wavelet-HHH	glcm	DifferenceAverage	3.204123654
wavelet-HHH	glcm	Id	0.364878039
wavelet-HHH	glcm	ClusterTendency	16.0885166
wavelet-HHH	firstorder	InterquartileRange	81.04711654
wavelet-HHH	firstorder	Skewness	-1.422219659
wavelet-HHH	firstorder	Uniformity	0.135734072
wavelet-HHH	firstorder	Median	18.69617023

wavelet-HHH	firstorder	Energy	120267.41
wavelet-HHH	firstorder	RobustMeanAbsoluteDeviation	33.05318594
wavelet-HHH	firstorder	MeanAbsoluteDeviation	57.68402379
wavelet-HHH	firstorder	TotalEnergy	3247220.071
wavelet-HHH	firstorder	Maximum	119.4819423
wavelet-HHH	firstorder	RootMeanSquared	79.56044046
wavelet-HHH	firstorder	90Percentile	79.82026383
wavelet-HHH	firstorder	Minimum	-238.614198
wavelet-HHH	firstorder	Entropy	3.010570934
wavelet-HHH	firstorder	Range	358.0961403
wavelet-HHH	firstorder	Variance	6321.618804
wavelet-HHH	firstorder	10Percentile	-57.08803761
wavelet-HHH	firstorder	Kurtosis	5.305063178
wavelet-HHH	firstorder	Mean	2.871390253
wavelet-HHH	glrlm	ShortRunLowGrayLevelEmphasis	0.064099142
wavelet-HHH	glrlm	GrayLevelVariance	10.59338927
wavelet-HHH	glrlm	LowGrayLevelRunEmphasis	0.064443978
wavelet-HHH	glrlm	GrayLevelNonUniformityNormalized	0.133745385
wavelet-HHH	glrlm	RunVariance	0.040186713
wavelet-HHH	glrlm	GrayLevelNonUniformity	2.438861104
wavelet-HHH	glrlm	LongRunEmphasis	1.131221719
wavelet-HHH	glrlm	ShortRunHighGrayLevelEmphasis	118.5297326
wavelet-HHH	glrlm	RunLengthNonUniformity	16.81598793
wavelet-HHH	glrlm	ShortRunEmphasis	0.96719457
wavelet-HHH	glrlm	LongRunHighGrayLevelEmphasis	135.2821836
wavelet-HHH	glrlm	RunPercentage	0.95951417
wavelet-HHH	glrlm	LongRunLowGrayLevelEmphasis	0.065823323
wavelet-HHH	glrlm	RunEntropy	3.111362573
wavelet-HHH	glrlm	HighGrayLevelRunEmphasis	121.8802228
wavelet-HHH	glrlm	RunLengthNonUniformityNormalized	0.919626574
wavelet-HHH	glszm	GrayLevelVariance	14.90972222
wavelet-HHH	glszm	ZoneVariance	0.743055556
wavelet-HHH	glszm	GrayLevelNonUniformityNormalized	0.125
wavelet-HHH	glszm	SizeZoneNonUniformityNormalized	0.458333333
wavelet-HHH	glszm	SizeZoneNonUniformity	5.5
wavelet-HHH	glszm	GrayLevelNonUniformity	1.5
wavelet-HHH	glszm	LargeAreaEmphasis	3.25
wavelet-HHH	glszm	SmallAreaHighGrayLevelEmphasis	87.21354167
wavelet-HHH	glszm	ZonePercentage	0.631578947
wavelet-HHH	glszm	LargeAreaLowGrayLevelEmphasis	0.115991047
wavelet-HHH	glszm	LargeAreaHighGrayLevelEmphasis	361.6666667
wavelet-HHH	glszm	HighGrayLevelZoneEmphasis	126.9166667
wavelet-HHH	glszm	SmallAreaEmphasis	0.671875
wavelet-HHH	glszm	LowGrayLevelZoneEmphasis	0.092777432
wavelet-HHH	glszm	ZoneEntropy	3.418295834
wavelet-HHH	glszm	SmallAreaLowGrayLevelEmphasis	0.089867546
wavelet-HHH	ngtdm	Coarseness	0.199016712
wavelet-HHH	ngtdm	Complexity	110.1217572
wavelet-HHH	ngtdm	Strength	10.74263058
wavelet-HHH	ngtdm	Contrast	0.716219218
wavelet-HHH	ngtdm	Busyness	0.086163691
wavelet-HLL	gldm	GrayLevelVariance	35.36842105
wavelet-HLL	gldm	HighGrayLevelEmphasis	260.3684211
wavelet-HLL	gldm	DependenceEntropy	3.576617645
wavelet-HLL	gldm	DependenceNonUniformity	12.68421053
wavelet-HLL	gldm	GrayLevelNonUniformity	1.736842105

wavelet-HLL	gldm	SmallDependenceEmphasis	0.842105263
wavelet-HLL	gldm	SmallDependenceHighGrayLevelEmphasis	231.5526316
wavelet-HLL	gldm	DependenceNonUniformityNormalized	0.667590028
wavelet-HLL	gldm	LargeDependenceEmphasis	1.631578947
wavelet-HLL	gldm	LargeDependenceLowGrayLevelEmphasis	0.061638336
wavelet-HLL	gldm	DependenceVariance	0.166204986
wavelet-HLL	gldm	LargeDependenceHighGrayLevelEmphasis	375.6315789
wavelet-HLL	gldm	SmallDependenceLowGrayLevelEmphasis	0.057288651
wavelet-HLL	gldm	LowGrayLevelEmphasis	0.058158588
wavelet-HLL	glcm	JointAverage	14.5454157
wavelet-HLL	glcm	SumAverage	29.09083139
wavelet-HLL	glcm	JointEntropy	3.76922648
wavelet-HLL	glcm	ClusterShade	-25.87790621
wavelet-HLL	glcm	MaximumProbability	0.085808636
wavelet-HLL	glcm	Idmn	0.93056419
wavelet-HLL	glcm	JointEnergy	0.077288116
wavelet-HLL	glcm	Contrast	60.703108
wavelet-HLL	glcm	DifferenceEntropy	2.298446684
wavelet-HLL	glcm	InverseVariance	0.195941988
wavelet-HLL	glcm	DifferenceVariance	18.9647581
wavelet-HLL	glcm	Idn	0.825525013
wavelet-HLL	glcm	Idm	0.13573049
wavelet-HLL	glcm	Correlation	0.048593728
wavelet-HLL	glcm	Autocorrelation	215.0132923
wavelet-HLL	glcm	SumEntropy	2.641436724
wavelet-HLL	glcm	SumSquares	32.94400562
wavelet-HLL	glcm	ClusterProminence	14854.64122
wavelet-HLL	glcm	Imc2	0.997644602
wavelet-HLL	glcm	Imc1	-0.843027788
wavelet-HLL	glcm	DifferenceAverage	6.295238095
wavelet-HLL	glcm	Id	0.22456797
wavelet-HLL	glcm	ClusterTendency	71.07291449
wavelet-HLL	firstorder	InterquartileRange	111.9209674
wavelet-HLL	firstorder	Skewness	-0.262793847
wavelet-HLL	firstorder	Uniformity	0.091412742
wavelet-HLL	firstorder	Median	-42.26422493
wavelet-HLL	firstorder	Energy	444779.3348
wavelet-HLL	firstorder	RobustMeanAbsoluteDeviation	59.62184989
wavelet-HLL	firstorder	MeanAbsoluteDeviation	106.3680513
wavelet-HLL	firstorder	TotalEnergy	12009042.04
wavelet-HLL	firstorder	Maximum	258.7379929
wavelet-HLL	firstorder	RootMeanSquared	153.0014336
wavelet-HLL	firstorder	90Percentile	96.35295181
wavelet-HLL	firstorder	Minimum	-396.8664995
wavelet-HLL	firstorder	Entropy	3.576617645
wavelet-HLL	firstorder	Range	655.6044924
wavelet-HLL	firstorder	Variance	21947.97478
wavelet-HLL	firstorder	10Percentile	-219.7591252
wavelet-HLL	firstorder	Kurtosis	3.563701983
wavelet-HLL	firstorder	Mean	-38.2290975
wavelet-HLL	glrlm	ShortRunLowGrayLevelEmphasis	0.058573261
wavelet-HLL	glrlm	GrayLevelVariance	35.64816064
wavelet-HLL	glrlm	LowGrayLevelRunEmphasis	0.058608579
wavelet-HLL	glrlm	GrayLevelNonUniformityNormalized	0.091594258
wavelet-HLL	glrlm	RunVariance	0.008072175
wavelet-HLL	glrlm	GrayLevelNonUniformity	1.726045884

wavelet-HLL	glrlm	LongRunEmphasis	1.025641026
wavelet-HLL	glrlm	ShortRunHighGrayLevelEmphasis	259.8640913
wavelet-HLL	glrlm	RunLengthNonUniformity	18.55555556
wavelet-HLL	glrlm	ShortRunEmphasis	0.993589744
wavelet-HLL	glrlm	LongRunHighGrayLevelEmphasis	265.7134503
wavelet-HLL	glrlm	RunPercentage	0.991902834
wavelet-HLL	glrlm	LongRunLowGrayLevelEmphasis	0.058749851
wavelet-HLL	glrlm	RunEntropy	3.575973584
wavelet-HLL	glrlm	HighGrayLevelRunEmphasis	261.0339631
wavelet-HLL	glrlm	RunLengthNonUniformityNormalized	0.983855651
wavelet-HLL	glszm	GrayLevelVariance	39.20415225
wavelet-HLL	glszm	ZoneVariance	0.103806228
wavelet-HLL	glszm	GrayLevelNonUniformityNormalized	0.093425606
wavelet-HLL	glszm	SizeZoneNonUniformityNormalized	0.792387543
wavelet-HLL	glszm	SizeZoneNonUniformity	13.47058824
wavelet-HLL	glszm	GrayLevelNonUniformity	1.588235294
wavelet-HLL	glszm	LargeAreaEmphasis	1.352941176
wavelet-HLL	glszm	SmallAreaHighGrayLevelEmphasis	253.4264706
wavelet-HLL	glszm	ZonePercentage	0.894736842
wavelet-HLL	glszm	LargeAreaLowGrayLevelEmphasis	0.066297151
wavelet-HLL	glszm	LargeAreaHighGrayLevelEmphasis	333.9411765
wavelet-HLL	glszm	HighGrayLevelZoneEmphasis	269.5294118
wavelet-HLL	glszm	SmallAreaEmphasis	0.911764706
wavelet-HLL	glszm	LowGrayLevelZoneEmphasis	0.064352587
wavelet-HLL	glszm	ZoneEntropy	3.572469459
wavelet-HLL	glszm	SmallAreaLowGrayLevelEmphasis	0.063866445
wavelet-HLL	ngtdm	Coarseness	0.176774386
wavelet-HLL	ngtdm	Complexity	457.1285819
wavelet-HLL	ngtdm	Strength	29.17363613
wavelet-HLL	ngtdm	Contrast	1.807533811
wavelet-HLL	ngtdm	Busyness	0.042855516
wavelet-LHL	gldm	GrayLevelVariance	46.99722992
wavelet-LHL	gldm	HighGrayLevelEmphasis	273.5789474
wavelet-LHL	gldm	DependenceEntropy	3.721611724
wavelet-LHL	gldm	DependenceNonUniformity	9.526315789
wavelet-LHL	gldm	GrayLevelNonUniformity	1.736842105
wavelet-LHL	gldm	SmallDependenceEmphasis	0.605263158
wavelet-LHL	gldm	SmallDependenceHighGrayLevelEmphasis	123.3421053
wavelet-LHL	gldm	DependenceNonUniformityNormalized	0.501385042
wavelet-LHL	gldm	LargeDependenceEmphasis	2.578947368
wavelet-LHL	gldm	LargeDependenceLowGrayLevelEmphasis	0.077692475
wavelet-LHL	gldm	DependenceVariance	0.249307479
wavelet-LHL	gldm	LargeDependenceHighGrayLevelEmphasis	874.5263158
wavelet-LHL	gldm	SmallDependenceLowGrayLevelEmphasis	0.07228179
wavelet-LHL	gldm	LowGrayLevelEmphasis	0.073363927
wavelet-LHL	glcm	JointAverage	15.87951493
wavelet-LHL	glcm	SumAverage	31.75902986
wavelet-LHL	glcm	JointEntropy	3.724870836
wavelet-LHL	glcm	ClusterShade	-967.9691576
wavelet-LHL	glcm	MaximumProbability	0.100643801
wavelet-LHL	glcm	Idmn	0.894110111
wavelet-LHL	glcm	JointEnergy	0.079945069
wavelet-LHL	glcm	Contrast	82.43696304
wavelet-LHL	glcm	DifferenceEntropy	2.324255055
wavelet-LHL	glcm	InverseVariance	0.185785771
wavelet-LHL	glcm	DifferenceVariance	29.63714526

wavelet-LHL	glcm	Idn	0.804985557
wavelet-LHL	glcm	Idm	0.164019949
wavelet-LHL	glcm	Correlation	-0.021277028
wavelet-LHL	glcm	Autocorrelation	252.4049728
wavelet-LHL	glcm	SumEntropy	2.43622973
wavelet-LHL	glcm	SumSquares	40.53478317
wavelet-LHL	glcm	ClusterProminence	29736.05158
wavelet-LHL	glcm	Imc2	0.997083421
wavelet-LHL	glcm	Imc1	-0.831277773
wavelet-LHL	glcm	DifferenceAverage	6.653579754
wavelet-LHL	glcm	Id	0.253149004
wavelet-LHL	glcm	ClusterTendency	79.70216964
wavelet-LHL	firstorder	InterquartileRange	190.000185
wavelet-LHL	firstorder	Skewness	-0.900540313
wavelet-LHL	firstorder	Uniformity	0.091412742
wavelet-LHL	firstorder	Median	-75.89897282
wavelet-LHL	firstorder	Energy	899424.1779
wavelet-LHL	firstorder	RobustMeanAbsoluteDeviation	96.58331384
wavelet-LHL	firstorder	MeanAbsoluteDeviation	139.7150417
wavelet-LHL	firstorder	TotalEnergy	24284452.8
wavelet-LHL	firstorder	Maximum	60.11513303
wavelet-LHL	firstorder	RootMeanSquared	217.5732397
wavelet-LHL	firstorder	90Percentile	49.7529522
wavelet-LHL	firstorder	Minimum	-481.0249289
wavelet-LHL	firstorder	Entropy	3.576617645
wavelet-LHL	firstorder	Range	541.140062
wavelet-LHL	firstorder	Variance	28945.29544
wavelet-LHL	firstorder	10Percentile	-417.7515126
wavelet-LHL	firstorder	Kurtosis	2.481757361
wavelet-LHL	firstorder	Mean	-135.6201283
wavelet-LHL	glrlm	ShortRunLowGrayLevelEmphasis	0.074863027
wavelet-LHL	glrlm	GrayLevelVariance	47.49661667
wavelet-LHL	glrlm	LowGrayLevelRunEmphasis	0.074908194
wavelet-LHL	glrlm	GrayLevelNonUniformityNormalized	0.091307512
wavelet-LHL	glrlm	RunVariance	0.020093357
wavelet-LHL	glrlm	GrayLevelNonUniformity	1.700166706
wavelet-LHL	glrlm	LongRunEmphasis	1.06561086
wavelet-LHL	glrlm	ShortRunHighGrayLevelEmphasis	265.0606891
wavelet-LHL	glrlm	RunLengthNonUniformity	17.90799397
wavelet-LHL	glrlm	ShortRunEmphasis	0.983597285
wavelet-LHL	glrlm	LongRunHighGrayLevelEmphasis	296.1353497
wavelet-LHL	glrlm	RunPercentage	0.979757085
wavelet-LHL	glrlm	LongRunLowGrayLevelEmphasis	0.075088863
wavelet-LHL	glrlm	RunEntropy	3.587798017
wavelet-LHL	glrlm	HighGrayLevelRunEmphasis	271.2756212
wavelet-LHL	glrlm	RunLengthNonUniformityNormalized	0.959813287
wavelet-LHL	glszm	GrayLevelVariance	53.10714286
wavelet-LHL	glszm	ZoneVariance	0.229591837
wavelet-LHL	glszm	GrayLevelNonUniformityNormalized	0.081632653
wavelet-LHL	glszm	SizeZoneNonUniformityNormalized	0.540816327
wavelet-LHL	glszm	SizeZoneNonUniformity	7.571428571
wavelet-LHL	glszm	GrayLevelNonUniformity	1.142857143
wavelet-LHL	glszm	LargeAreaEmphasis	2.071428571
wavelet-LHL	glszm	SmallAreaHighGrayLevelEmphasis	133.4107143
wavelet-LHL	glszm	ZonePercentage	0.736842105
wavelet-LHL	glszm	LargeAreaLowGrayLevelEmphasis	0.101523482

wavelet-LHL	glszm	LargeAreaHighGrayLevelEmphasis	643.1428571
wavelet-LHL	glszm	HighGrayLevelZoneEmphasis	235.3571429
wavelet-LHL	glszm	SmallAreaEmphasis	0.732142857
wavelet-LHL	glszm	LowGrayLevelZoneEmphasis	0.098586253
wavelet-LHL	glszm	ZoneEntropy	3.807354922
wavelet-LHL	glszm	SmallAreaLowGrayLevelEmphasis	0.097851946
wavelet-LHL	ngtdm	Coarseness	0.135059064
wavelet-LHL	ngtdm	Complexity	617.8252088
wavelet-LHL	ngtdm	Strength	27.66916182
wavelet-LHL	ngtdm	Contrast	3.173410427
wavelet-LHL	ngtdm	Busyness	0.042837756
wavelet-LHH	gldm	GrayLevelVariance	7.163434903
wavelet-LHH	gldm	HighGrayLevelEmphasis	51.84210526
wavelet-LHH	gldm	DependenceEntropy	3.366091329
wavelet-LHH	gldm	DependenceNonUniformity	6.789473684
wavelet-LHH	gldm	GrayLevelNonUniformity	2.157894737
wavelet-LHH	gldm	SmallDependenceEmphasis	0.497076023
wavelet-LHH	gldm	SmallDependenceHighGrayLevelEmphasis	25.05263158
wavelet-LHH	gldm	DependenceNonUniformityNormalized	0.35734072
wavelet-LHH	gldm	LargeDependenceEmphasis	3.947368421
wavelet-LHH	gldm	LargeDependenceLowGrayLevelEmphasis	0.148267649
wavelet-LHH	gldm	DependenceVariance	0.55401662
wavelet-LHH	gldm	LargeDependenceHighGrayLevelEmphasis	224.2631579
wavelet-LHH	gldm	SmallDependenceLowGrayLevelEmphasis	0.076966925
wavelet-LHH	gldm	LowGrayLevelEmphasis	0.088194798
wavelet-LHH	glcm	JointAverage	6.662409812
wavelet-LHH	glcm	SumAverage	13.32481962
wavelet-LHH	glcm	JointEntropy	3.650051211
wavelet-LHH	glcm	ClusterShade	-4.875812694
wavelet-LHH	glcm	MaximumProbability	0.120942946
wavelet-LHH	glcm	Idmn	0.892834356
wavelet-LHH	glcm	JointEnergy	0.085854368
wavelet-LHH	glcm	Contrast	17.32617383
wavelet-LHH	glcm	DifferenceEntropy	2.108640001
wavelet-LHH	glcm	InverseVariance	0.255227382
wavelet-LHH	glcm	DifferenceVariance	5.544174559
wavelet-LHH	glcm	Idn	0.787491656
wavelet-LHH	glcm	Idm	0.245015435
wavelet-LHH	glcm	Correlation	-0.251473333
wavelet-LHH	glcm	Autocorrelation	42.86881452
wavelet-LHH	glcm	SumEntropy	2.299881405
wavelet-LHH	glcm	SumSquares	6.880473535
wavelet-LHH	glcm	ClusterProminence	290.9931961
wavelet-LHH	glcm	Imc2	0.992769383
wavelet-LHH	glcm	Imc1	-0.752493363
wavelet-LHH	glcm	DifferenceAverage	3.356132756
wavelet-LHH	glcm	Id	0.338893602
wavelet-LHH	glcm	ClusterTendency	10.19572031
wavelet-LHH	firstorder	InterquartileRange	91.44841137
wavelet-LHH	firstorder	Skewness	-0.340960016
wavelet-LHH	firstorder	Uniformity	0.113573407
wavelet-LHH	firstorder	Median	16.7768505
wavelet-LHH	firstorder	Energy	87923.67276
wavelet-LHH	firstorder	RobustMeanAbsoluteDeviation	39.29640134
wavelet-LHH	firstorder	MeanAbsoluteDeviation	56.06040286
wavelet-LHH	firstorder	TotalEnergy	2373939.164

wavelet-LHH	firstorder	Maximum	123.3974348
wavelet-LHH	firstorder	RootMeanSquared	68.02618411
wavelet-LHH	firstorder	90Percentile	87.02825919
wavelet-LHH	firstorder	Minimum	-147.5416359
wavelet-LHH	firstorder	Entropy	3.22109725
wavelet-LHH	firstorder	Range	270.9390707
wavelet-LHH	firstorder	Variance	4606.259204
wavelet-LHH	firstorder	10Percentile	-70.7134568
wavelet-LHH	firstorder	Kurtosis	2.614311973
wavelet-LHH	firstorder	Mean	4.615465306
wavelet-LHH	glrlm	ShortRunLowGrayLevelEmphasis	0.089980951
wavelet-LHH	glrlm	GrayLevelVariance	7.310477515
wavelet-LHH	glrlm	LowGrayLevelRunEmphasis	0.090526692
wavelet-LHH	glrlm	GrayLevelNonUniformityNormalized	0.113433818
wavelet-LHH	glrlm	RunVariance	0.032201619
wavelet-LHH	glrlm	GrayLevelNonUniformity	2.085443624
wavelet-LHH	glrlm	LongRunEmphasis	1.104072398
wavelet-LHH	glrlm	ShortRunHighGrayLevelEmphasis	50.18894049
wavelet-LHH	glrlm	RunLengthNonUniformity	17.2413273
wavelet-LHH	glrlm	ShortRunEmphasis	0.9739819
wavelet-LHH	glrlm	LongRunHighGrayLevelEmphasis	57.58524516
wavelet-LHH	glrlm	RunPercentage	0.967611336
wavelet-LHH	glrlm	LongRunLowGrayLevelEmphasis	0.092709654
wavelet-LHH	glrlm	RunEntropy	3.266070339
wavelet-LHH	glrlm	HighGrayLevelRunEmphasis	51.66820142
wavelet-LHH	glrlm	RunLengthNonUniformityNormalized	0.935596763
wavelet-LHH	glszm	GrayLevelVariance	9.909722222
wavelet-LHH	glszm	ZoneVariance	0.576388889
wavelet-LHH	glszm	GrayLevelNonUniformityNormalized	0.111111111
wavelet-LHH	glszm	SizeZoneNonUniformityNormalized	0.430555556
wavelet-LHH	glszm	SizeZoneNonUniformity	5.166666667
wavelet-LHH	glszm	GrayLevelNonUniformity	1.333333333
wavelet-LHH	glszm	LargeAreaEmphasis	3.083333333
wavelet-LHH	glszm	SmallAreaHighGrayLevelEmphasis	33.79166667
wavelet-LHH	glszm	ZonePercentage	0.631578947
wavelet-LHH	glszm	LargeAreaLowGrayLevelEmphasis	0.172375686
wavelet-LHH	glszm	LargeAreaHighGrayLevelEmphasis	163.5833333
wavelet-LHH	glszm	HighGrayLevelZoneEmphasis	51.08333333
wavelet-LHH	glszm	SmallAreaEmphasis	0.664351852
wavelet-LHH	glszm	LowGrayLevelZoneEmphasis	0.126618424
wavelet-LHH	glszm	ZoneEntropy	3.251629167
wavelet-LHH	glszm	SmallAreaLowGrayLevelEmphasis	0.118894243
wavelet-LHH	ngtdm	Coarseness	0.188684999
wavelet-LHH	ngtdm	Complexity	105.4179526
wavelet-LHH	ngtdm	Strength	6.469229619
wavelet-LHH	ngtdm	Contrast	0.448649217
wavelet-LHH	ngtdm	Busyness	0.116817784
wavelet-LLH	gldm	GrayLevelVariance	72.84764543
wavelet-LLH	gldm	HighGrayLevelEmphasis	179.2631579
wavelet-LLH	gldm	DependenceEntropy	3.616348566
wavelet-LLH	gldm	DependenceNonUniformity	8.684210526
wavelet-LLH	gldm	GrayLevelNonUniformity	1.842105263
wavelet-LLH	gldm	SmallDependenceEmphasis	0.558479532
wavelet-LLH	gldm	SmallDependenceHighGrayLevelEmphasis	146.6637427
wavelet-LLH	gldm	DependenceNonUniformityNormalized	0.457063712
wavelet-LLH	gldm	LargeDependenceEmphasis	3

wavelet-LLH	gldm	LargeDependenceLowGrayLevelEmphasis	0.108670949
wavelet-LLH	gldm	DependenceVariance	0.337950139
wavelet-LLH	gldm	LargeDependenceHighGrayLevelEmphasis	324.6315789
wavelet-LLH	gldm	SmallDependenceLowGrayLevelEmphasis	0.061229843
wavelet-LLH	gldm	LowGrayLevelEmphasis	0.06998707
wavelet-LLH	glcm	JointAverage	9.37004662
wavelet-LLH	glcm	SumAverage	18.74009324
wavelet-LLH	glcm	JointEntropy	3.693824105
wavelet-LLH	glcm	ClusterShade	1142.213886
wavelet-LLH	glcm	MaximumProbability	0.104551005
wavelet-LLH	glcm	Idmn	0.968521739
wavelet-LLH	glcm	JointEnergy	0.081666988
wavelet-LLH	glcm	Contrast	80.22455322
wavelet-LLH	glcm	DifferenceEntropy	2.294832328
wavelet-LLH	glcm	InverseVariance	0.194646498
wavelet-LLH	glcm	DifferenceVariance	43.82895721
wavelet-LLH	glcm	Idn	0.894628867
wavelet-LLH	glcm	Idm	0.168343231
wavelet-LLH	glcm	Correlation	-0.273128701
wavelet-LLH	glcm	Autocorrelation	84.41990787
wavelet-LLH	glcm	SumEntropy	2.444834476
wavelet-LLH	glcm	SumSquares	35.87831978
wavelet-LLH	glcm	ClusterProminence	40573.29311
wavelet-LLH	glcm	Imc2	0.994147533
wavelet-LLH	glcm	Imc1	-0.768023395
wavelet-LLH	glcm	DifferenceAverage	5.749173049
wavelet-LLH	glcm	Id	0.263667621
wavelet-LLH	glcm	ClusterTendency	63.28872591
wavelet-LLH	firstorder	InterquartileRange	160.2698691
wavelet-LLH	firstorder	Skewness	2.813452063
wavelet-LLH	firstorder	Uniformity	0.096952909
wavelet-LLH	firstorder	Median	-200.4811943
wavelet-LLH	firstorder	Energy	1307523.233
wavelet-LLH	firstorder	RobustMeanAbsoluteDeviation	55.93788585
wavelet-LLH	firstorder	MeanAbsoluteDeviation	127.4130432
wavelet-LLH	firstorder	TotalEnergy	35303127.28
wavelet-LLH	firstorder	Maximum	654.9032972
wavelet-LLH	firstorder	RootMeanSquared	262.3299682
wavelet-LLH	firstorder	90Percentile	-59.30166146
wavelet-LLH	firstorder	Minimum	-395.1708138
wavelet-LLH	firstorder	Entropy	3.471354487
wavelet-LLH	firstorder	Range	1050.074111
wavelet-LLH	firstorder	Variance	44957.89013
wavelet-LLH	firstorder	10Percentile	-296.7056859
wavelet-LLH	firstorder	Kurtosis	11.31990209
wavelet-LLH	firstorder	Mean	-154.4639832
wavelet-LLH	glrlm	ShortRunLowGrayLevelEmphasis	0.071021748
wavelet-LLH	glrlm	GrayLevelVariance	74.5177691
wavelet-LLH	glrlm	LowGrayLevelRunEmphasis	0.071369239
wavelet-LLH	glrlm	GrayLevelNonUniformityNormalized	0.097124806
wavelet-LLH	glrlm	RunVariance	0.033442397
wavelet-LLH	glrlm	GrayLevelNonUniformity	1.800944669
wavelet-LLH	glrlm	LongRunEmphasis	1.091346154
wavelet-LLH	glrlm	ShortRunHighGrayLevelEmphasis	180.8564651
wavelet-LLH	glrlm	RunLengthNonUniformity	17.82371795
wavelet-LLH	glrlm	ShortRunEmphasis	0.982505342

wavelet-LLH	glrlm	LongRunHighGrayLevelEmphasis	188.7377418
wavelet-LLH	glrlm	RunPercentage	0.975708502
wavelet-LLH	glrlm	LongRunLowGrayLevelEmphasis	0.07309307
wavelet-LLH	glrlm	RunEntropy	3.47124237
wavelet-LLH	glrlm	HighGrayLevelRunEmphasis	182.1592161
wavelet-LLH	glrlm	RunLengthNonUniformityNormalized	0.958355591
wavelet-LLH	glszm	GrayLevelVariance	100.3668639
wavelet-LLH	glszm	ZoneVariance	0.402366864
wavelet-LLH	glszm	GrayLevelNonUniformityNormalized	0.088757396
wavelet-LLH	glszm	SizeZoneNonUniformityNormalized	0.479289941
wavelet-LLH	glszm	SizeZoneNonUniformity	6.230769231
wavelet-LLH	glszm	GrayLevelNonUniformity	1.153846154
wavelet-LLH	glszm	LargeAreaEmphasis	2.538461538
wavelet-LLH	glszm	SmallAreaHighGrayLevelEmphasis	205.9123932
wavelet-LLH	glszm	ZonePercentage	0.684210526
wavelet-LLH	glszm	LargeAreaLowGrayLevelEmphasis	0.122737351
wavelet-LLH	glszm	LargeAreaHighGrayLevelEmphasis	339.3846154
wavelet-LLH	glszm	HighGrayLevelZoneEmphasis	228.2307692
wavelet-LLH	glszm	SmallAreaEmphasis	0.700854701
wavelet-LLH	glszm	LowGrayLevelZoneEmphasis	0.093266439
wavelet-LLH	glszm	ZoneEntropy	3.546593564
wavelet-LLH	glszm	SmallAreaLowGrayLevelEmphasis	0.087234181
wavelet-LLH	ngtdm	Coarseness	0.13926128
wavelet-LLH	ngtdm	Complexity	918.9056631
wavelet-LLH	ngtdm	Strength	42.81649011
wavelet-LLH	ngtdm	Contrast	5.939662825
wavelet-LLH	ngtdm	Busyness	0.077696008
wavelet-LLL	gldm	GrayLevelVariance	172.1108033
wavelet-LLL	gldm	HighGrayLevelEmphasis	1152.789474
wavelet-LLL	gldm	DependenceEntropy	3.93213804
wavelet-LLL	gldm	DependenceNonUniformity	15.42105263
wavelet-LLL	gldm	GrayLevelNonUniformity	1.315789474
wavelet-LLL	gldm	SmallDependenceEmphasis	0.921052632
wavelet-LLL	gldm	SmallDependenceHighGrayLevelEmphasis	1066.815789
wavelet-LLL	gldm	DependenceNonUniformityNormalized	0.811634349
wavelet-LLL	gldm	LargeDependenceEmphasis	1.315789474
wavelet-LLL	gldm	LargeDependenceLowGrayLevelEmphasis	0.05447622
wavelet-LLL	gldm	DependenceVariance	0.094182825
wavelet-LLL	gldm	LargeDependenceHighGrayLevelEmphasis	1496.684211
wavelet-LLL	gldm	SmallDependenceLowGrayLevelEmphasis	0.054113744
wavelet-LLL	gldm	LowGrayLevelEmphasis	0.054186239
wavelet-LLL	glcm	JointAverage	33.14558497
wavelet-LLL	glcm	SumAverage	66.29116994
wavelet-LLL	glcm	JointEntropy	3.762666374
wavelet-LLL	glcm	ClusterShade	-3686.603186
wavelet-LLL	glcm	MaximumProbability	0.085242535
wavelet-LLL	glcm	Idmn	0.899152984
wavelet-LLL	glcm	JointEnergy	0.077456036
wavelet-LLL	glcm	Contrast	327.5200633
wavelet-LLL	glcm	DifferenceEntropy	2.502458392
wavelet-LLL	glcm	InverseVariance	0.064394793
wavelet-LLL	glcm	DifferenceVariance	84.38312852
wavelet-LLL	glcm	Idn	0.788055183
wavelet-LLL	glcm	Idm	0.050241288
wavelet-LLL	glcm	Correlation	-0.030775507
wavelet-LLL	glcm	Autocorrelation	1094.541536

wavelet-LLL	glcm	SumEntropy	2.651610763
wavelet-LLL	glcm	SumSquares	157.7305303
wavelet-LLL	glcm	ClusterProminence	284349.3951
wavelet-LLL	glcm	Imc2	0.998694293
wavelet-LLL	glcm	Imc1	-0.903514949
wavelet-LLL	glcm	DifferenceAverage	14.92411477
wavelet-LLL	glcm	Id	0.117591749
wavelet-LLL	glcm	ClusterTendency	303.4020578
wavelet-LLL	firstorder	InterquartileRange	461.9295031
wavelet-LLL	firstorder	Skewness	-0.514814709
wavelet-LLL	firstorder	Uniformity	0.069252078
wavelet-LLL	firstorder	Median	-353.1322204
wavelet-LLL	firstorder	Energy	5196742.973
wavelet-LLL	firstorder	RobustMeanAbsoluteDeviation	196.2427568
wavelet-LLL	firstorder	MeanAbsoluteDeviation	273.7263504
wavelet-LLL	firstorder	TotalEnergy	140312060.3
wavelet-LLL	firstorder	Maximum	60.47465718
wavelet-LLL	firstorder	RootMeanSquared	522.9845008
wavelet-LLL	firstorder	90Percentile	-0.764642069
wavelet-LLL	firstorder	Minimum	-1162.216667
wavelet-LLL	firstorder	Entropy	3.93213804
wavelet-LLL	firstorder	Range	1222.691324
wavelet-LLL	firstorder	Variance	107903.2342
wavelet-LLL	firstorder	10Percentile	-784.3729707
wavelet-LLL	firstorder	Kurtosis	2.54860754
wavelet-LLL	firstorder	Mean	-406.9515375
wavelet-LLL	glrlm	ShortRunLowGrayLevelEmphasis	0.054410937
wavelet-LLL	glrlm	GrayLevelVariance	172.8335241
wavelet-LLL	glrlm	LowGrayLevelRunEmphasis	0.05441388
wavelet-LLL	glrlm	GrayLevelNonUniformityNormalized	0.069148167
wavelet-LLL	glrlm	RunVariance	0.004036087
wavelet-LLL	glrlm	GrayLevelNonUniformity	1.308591993
wavelet-LLL	glrlm	LongRunEmphasis	1.012820513
wavelet-LLL	glrlm	ShortRunHighGrayLevelEmphasis	1149.571694
wavelet-LLL	glrlm	RunLengthNonUniformity	18.77777778
wavelet-LLL	glrlm	ShortRunEmphasis	0.996794872
wavelet-LLL	glrlm	LongRunHighGrayLevelEmphasis	1167.023617
wavelet-LLL	glrlm	RunPercentage	0.995951417
wavelet-LLL	glrlm	LongRunLowGrayLevelEmphasis	0.054425653
wavelet-LLL	glrlm	RunEntropy	3.933335327
wavelet-LLL	glrlm	HighGrayLevelRunEmphasis	1153.062078
wavelet-LLL	glrlm	RunLengthNonUniformityNormalized	0.991927825
wavelet-LLL	glszm	GrayLevelVariance	181.5061728
wavelet-LLL	glszm	ZoneVariance	0.052469136
wavelet-LLL	glszm	GrayLevelNonUniformityNormalized	0.067901235
wavelet-LLL	glszm	SizeZoneNonUniformityNormalized	0.895061728
wavelet-LLL	glszm	SizeZoneNonUniformity	16.11111111
wavelet-LLL	glszm	GrayLevelNonUniformity	1.222222222
wavelet-LLL	glszm	LargeAreaEmphasis	1.166666667
wavelet-LLL	glszm	SmallAreaHighGrayLevelEmphasis	1110.958333
wavelet-LLL	glszm	ZonePercentage	0.947368421
wavelet-LLL	glszm	LargeAreaLowGrayLevelEmphasis	0.057298616
wavelet-LLL	glszm	LargeAreaHighGrayLevelEmphasis	1337.833333
wavelet-LLL	glszm	HighGrayLevelZoneEmphasis	1156.333333
wavelet-LLL	glszm	SmallAreaEmphasis	0.958333333
wavelet-LLL	glszm	LowGrayLevelZoneEmphasis	0.05714557

wavelet-LLL	glszm	ZoneEntropy	3.947702779
wavelet-LLL	glszm	SmallAreaLowGrayLevelEmphasis	0.057107309
wavelet-LLL	ngtdm	Coarseness	0.07880524
wavelet-LLL	ngtdm	Complexity	3105.069506
wavelet-LLL	ngtdm	Strength	56.82764246
wavelet-LLL	ngtdm	Contrast	15.87102709
wavelet-LLL	ngtdm	Busyness	0.045268629
wavelet-HHL	gldm	GrayLevelVariance	8.847645429
wavelet-HHL	gldm	HighGrayLevelEmphasis	27.47368421
wavelet-HHL	gldm	DependenceEntropy	3.576617645
wavelet-HHL	gldm	DependenceNonUniformity	6.789473684
wavelet-HHL	gldm	GrayLevelNonUniformity	2.578947368
wavelet-HHL	gldm	SmallDependenceEmphasis	0.497076023
wavelet-HHL	gldm	SmallDependenceHighGrayLevelEmphasis	22.64912281
wavelet-HHL	gldm	DependenceNonUniformityNormalized	0.35734072
wavelet-HHL	gldm	LargeDependenceEmphasis	3.947368421
wavelet-HHL	gldm	LargeDependenceLowGrayLevelEmphasis	1.690229816
wavelet-HHL	gldm	DependenceVariance	0.55401662
wavelet-HHL	gldm	LargeDependenceHighGrayLevelEmphasis	49.57894737
wavelet-HHL	gldm	SmallDependenceLowGrayLevelEmphasis	0.051734851
wavelet-HHL	gldm	LowGrayLevelEmphasis	0.233884786
wavelet-HHL	glcm	JointAverage	4.067177267
wavelet-HHL	glcm	SumAverage	8.134354534
wavelet-HHL	glcm	JointEntropy	3.590954752
wavelet-HHL	glcm	ClusterShade	65.15070619
wavelet-HHL	glcm	MaximumProbability	0.127658453
wavelet-HHL	glcm	Idmn	0.916202855
wavelet-HHL	glcm	JointEnergy	0.090981798
wavelet-HHL	glcm	Contrast	16.36409146
wavelet-HHL	glcm	DifferenceEntropy	2.124989036
wavelet-HHL	glcm	InverseVariance	0.316549988
wavelet-HHL	glcm	DifferenceVariance	6.537367768
wavelet-HHL	glcm	Idn	0.818266626
wavelet-HHL	glcm	Idm	0.276647522
wavelet-HHL	glcm	Correlation	0.045960595
wavelet-HHL	glcm	Autocorrelation	17.12515818
wavelet-HHL	glcm	SumEntropy	2.46781035
wavelet-HHL	glcm	SumSquares	8.638464862
wavelet-HHL	glcm	ClusterProminence	1038.607114
wavelet-HHL	glcm	Imc2	0.977668862
wavelet-HHL	glcm	Imc1	-0.656307177
wavelet-HHL	glcm	DifferenceAverage	3.058086358
wavelet-HHL	glcm	Id	0.36395226
wavelet-HHL	glcm	ClusterTendency	18.18976798
wavelet-HHL	firstorder	InterquartileRange	78.14842265
wavelet-HHL	firstorder	Skewness	1.126175454
wavelet-HHL	firstorder	Uniformity	0.135734072
wavelet-HHL	firstorder	Median	3.561133683
wavelet-HHL	firstorder	Energy	116197.6693
wavelet-HHL	firstorder	RobustMeanAbsoluteDeviation	38.11952145
wavelet-HHL	firstorder	MeanAbsoluteDeviation	59.32763408
wavelet-HHL	firstorder	TotalEnergy	3137337.072
wavelet-HHL	firstorder	Maximum	211.566385
wavelet-HHL	firstorder	RootMeanSquared	78.20272889
wavelet-HHL	firstorder	90Percentile	128.8423871
wavelet-HHL	firstorder	Minimum	-68.50943329

wavelet-HHL	firstorder	Entropy	2.996641329
wavelet-HHL	firstorder	Range	280.0758183
wavelet-HHL	firstorder	Variance	5673.555963
wavelet-HHL	firstorder	10Percentile	-55.31146655
wavelet-HHL	firstorder	Kurtosis	3.441563497
wavelet-HHL	firstorder	Mean	21.02643202
wavelet-HHL	glrlm	ShortRunLowGrayLevelEmphasis	0.214806887
wavelet-HHL	glrlm	GrayLevelVariance	8.961415863
wavelet-HHL	glrlm	LowGrayLevelRunEmphasis	0.226460408
wavelet-HHL	glrlm	GrayLevelNonUniformityNormalized	0.13570518
wavelet-HHL	glrlm	RunVariance	0.032201619
wavelet-HHL	glrlm	GrayLevelNonUniformity	2.494959117
wavelet-HHL	glrlm	LongRunEmphasis	1.104072398
wavelet-HHL	glrlm	ShortRunHighGrayLevelEmphasis	27.92174063
wavelet-HHL	glrlm	RunLengthNonUniformity	17.2413273
wavelet-HHL	glrlm	ShortRunEmphasis	0.9739819
wavelet-HHL	glrlm	LongRunHighGrayLevelEmphasis	29.00394274
wavelet-HHL	glrlm	RunPercentage	0.967611336
wavelet-HHL	glrlm	LongRunLowGrayLevelEmphasis	0.273074494
wavelet-HHL	glrlm	RunEntropy	3.069839463
wavelet-HHL	glrlm	HighGrayLevelRunEmphasis	28.13818105
wavelet-HHL	glrlm	RunLengthNonUniformityNormalized	0.935596763
wavelet-HHL	glszm	GrayLevelVariance	10.52083333
wavelet-HHL	glszm	ZoneVariance	0.576388889
wavelet-HHL	glszm	GrayLevelNonUniformityNormalized	0.125
wavelet-HHL	glszm	SizeZoneNonUniformityNormalized	0.430555556
wavelet-HHL	glszm	SizeZoneNonUniformity	5.166666667
wavelet-HHL	glszm	GrayLevelNonUniformity	1.5
wavelet-HHL	glszm	LargeAreaEmphasis	3.083333333
wavelet-HHL	glszm	SmallAreaHighGrayLevelEmphasis	34.53009259
wavelet-HHL	glszm	ZonePercentage	0.631578947
wavelet-HHL	glszm	LargeAreaLowGrayLevelEmphasis	0.984623134
wavelet-HHL	glszm	LargeAreaHighGrayLevelEmphasis	56
wavelet-HHL	glszm	HighGrayLevelZoneEmphasis	38.08333333
wavelet-HHL	glszm	SmallAreaEmphasis	0.664351852
wavelet-HHL	glszm	LowGrayLevelZoneEmphasis	0.155757393
wavelet-HHL	glszm	ZoneEntropy	3.584962501
wavelet-HHL	glszm	SmallAreaLowGrayLevelEmphasis	0.051421616
wavelet-HHL	ngtdm	Coarseness	0.226094407
wavelet-HHL	ngtdm	Complexity	82.48844387
wavelet-HHL	ngtdm	Strength	10.24388692
wavelet-HHL	ngtdm	Contrast	0.531405465
wavelet-HHL	ngtdm	Busyness	0.269345167
wavelet-HLH	gldm	GrayLevelVariance	14.45429363
wavelet-HLH	gldm	HighGrayLevelEmphasis	43.84210526
wavelet-HLH	gldm	DependenceEntropy	3.471354487
wavelet-HLH	gldm	DependenceNonUniformity	7.210526316
wavelet-HLH	gldm	GrayLevelNonUniformity	2.684210526
wavelet-HLH	gldm	SmallDependenceEmphasis	0.543859649
wavelet-HLH	gldm	SmallDependenceHighGrayLevelEmphasis	36.4122807
wavelet-HLH	gldm	DependenceNonUniformityNormalized	0.379501385
wavelet-HLH	gldm	LargeDependenceEmphasis	3.526315789
wavelet-HLH	gldm	LargeDependenceLowGrayLevelEmphasis	0.596905071
wavelet-HLH	gldm	DependenceVariance	0.509695291
wavelet-HLH	gldm	LargeDependenceHighGrayLevelEmphasis	76.36842105
wavelet-HLH	gldm	SmallDependenceLowGrayLevelEmphasis	0.079638691

wavelet-HLH	gldm	LowGrayLevelEmphasis	0.148004248
wavelet-HLH	glcm	JointAverage	5.226101676
wavelet-HLH	glcm	SumAverage	10.45220335
wavelet-HLH	glcm	JointEntropy	3.584944096
wavelet-HLH	glcm	ClusterShade	71.16226383
wavelet-HLH	glcm	MaximumProbability	0.135625486
wavelet-HLH	glcm	Idmn	0.887518077
wavelet-HLH	glcm	JointEnergy	0.094036296
wavelet-HLH	glcm	Contrast	26.78184038
wavelet-HLH	glcm	DifferenceEntropy	2.105733441
wavelet-HLH	glcm	InverseVariance	0.247936099
wavelet-HLH	glcm	DifferenceVariance	9.550405074
wavelet-HLH	glcm	Idn	0.786160848
wavelet-HLH	glcm	Idm	0.21234798
wavelet-HLH	glcm	Correlation	-0.074994291
wavelet-HLH	glcm	Autocorrelation	27.00841936
wavelet-HLH	glcm	SumEntropy	2.394848456
wavelet-HLH	glcm	SumSquares	12.75812025
wavelet-HLH	glcm	ClusterProminence	1600.515193
wavelet-HLH	glcm	Imc2	0.989508664
wavelet-HLH	glcm	Imc1	-0.724758316
wavelet-HLH	glcm	DifferenceAverage	4.064923965
wavelet-HLH	glcm	Id	0.305516702
wavelet-HLH	glcm	ClusterTendency	24.25064062
wavelet-HLH	firstorder	InterquartileRange	138.831382
wavelet-HLH	firstorder	Skewness	0.727481688
wavelet-HLH	firstorder	Uniformity	0.141274238
wavelet-HLH	firstorder	Median	-34.55693841
wavelet-HLH	firstorder	Energy	171939.9837
wavelet-HLH	firstorder	RobustMeanAbsoluteDeviation	65.08095228
wavelet-HLH	firstorder	MeanAbsoluteDeviation	82.40368112
wavelet-HLH	firstorder	TotalEnergy	4642379.56
wavelet-HLH	firstorder	Maximum	190.2238424
wavelet-HLH	firstorder	RootMeanSquared	95.12871715
wavelet-HLH	firstorder	90Percentile	154.1217476
wavelet-HLH	firstorder	Minimum	-110.8412944
wavelet-HLH	firstorder	Entropy	3.070847094
wavelet-HLH	firstorder	Range	301.0651368
wavelet-HLH	firstorder	Variance	9044.961901
wavelet-HLH	firstorder	10Percentile	-90.22029918
wavelet-HLH	firstorder	Kurtosis	2.135776343
wavelet-HLH	firstorder	Mean	-2.123893895
wavelet-HLH	glrlm	ShortRunLowGrayLevelEmphasis	0.143572651
wavelet-HLH	glrlm	GrayLevelVariance	14.6463621
wavelet-HLH	glrlm	LowGrayLevelRunEmphasis	0.147433179
wavelet-HLH	glrlm	GrayLevelNonUniformityNormalized	0.138563436
wavelet-HLH	glrlm	RunVariance	0.028078451
wavelet-HLH	glrlm	GrayLevelNonUniformity	2.560318594
wavelet-HLH	glrlm	LongRunEmphasis	1.092760181
wavelet-HLH	glrlm	ShortRunHighGrayLevelEmphasis	44.42124778
wavelet-HLH	glrlm	RunLengthNonUniformity	17.4826546
wavelet-HLH	glrlm	ShortRunEmphasis	0.976809955
wavelet-HLH	glrlm	LongRunHighGrayLevelEmphasis	46.0850864
wavelet-HLH	glrlm	RunPercentage	0.971659919
wavelet-HLH	glrlm	LongRunLowGrayLevelEmphasis	0.162875287
wavelet-HLH	glrlm	RunEntropy	3.150805419

wavelet-HLH	glrlm	HighGrayLevelRunEmphasis	44.75401551
wavelet-HLH	glrlm	RunLengthNonUniformityNormalized	0.943843098
wavelet-HLH	glszm	GrayLevelVariance	16.98224852
wavelet-HLH	glszm	ZoneVariance	0.402366864
wavelet-HLH	glszm	GrayLevelNonUniformityNormalized	0.112426036
wavelet-HLH	glszm	SizeZoneNonUniformityNormalized	0.479289941
wavelet-HLH	glszm	SizeZoneNonUniformity	6.230769231
wavelet-HLH	glszm	GrayLevelNonUniformity	1.461538462
wavelet-HLH	glszm	LargeAreaEmphasis	2.538461538
wavelet-HLH	glszm	SmallAreaHighGrayLevelEmphasis	51.47649573
wavelet-HLH	glszm	ZonePercentage	0.684210526
wavelet-HLH	glszm	LargeAreaLowGrayLevelEmphasis	0.396547635
wavelet-HLH	glszm	LargeAreaHighGrayLevelEmphasis	79.30769231
wavelet-HLH	glszm	HighGrayLevelZoneEmphasis	56.76923077
wavelet-HLH	glszm	SmallAreaEmphasis	0.700854701
wavelet-HLH	glszm	LowGrayLevelZoneEmphasis	0.145427803
wavelet-HLH	glszm	ZoneEntropy	3.546593564
wavelet-HLH	glszm	SmallAreaLowGrayLevelEmphasis	0.104015366
wavelet-HLH	ngtdm	Coarseness	0.110287475
wavelet-HLH	ngtdm	Complexity	185.5971382
wavelet-HLH	ngtdm	Strength	10.21283245
wavelet-HLH	ngtdm	Contrast	1.041812345
wavelet-HLH	ngtdm	Busyness	0.282421381

Image type	Feature Class	Feature Name	4: RTSTRUCT: C99-DNU_segment_ITV
general	info	BoundingBox	(0, 1, 1, 7, 6, 15)
general	info	EnabledImageTypes	{u'Original': {}, u'Wavelet': {}}
general	info	GeneralSettings	{'distances': [1], 'additionalInfo': True, 'force2D': False, 'interpolator': 'sitkBSpline', 'resampledPixelSpacing': [3.0], 'label': 1, 'normalizeScale': 1, 'normalize': False, 'force2Ddimension': 0, 'removeOutliers': None, 'minimumROISize': None, u'binWidth': 25.0, 'minimumROIDimensions': 1, u'correctMask': True, 'preCrop': False, u'symmetricalGLCM': True, 'resegmentRange': None, 'padDistance': 5}
general	info	ImageHash	a3dd8b75e538c3806eb22051287f3de22e6e57f7
general	info	ImageSpacing	(0.9765625000000002, 0.9765625000000002, 2.0)
general	info	MaskHash	137a80e6dcc20888afb4cf6c4703a7c8b99ea9aa
general	info	NumpyVersion	1.13.1
general	info	PyWaveletVersion	0.5.2
general	info	SimpleITKVersion	1.1.0
general	info	Version	1.3.0.post102+g1b0eb74
general	info	VolumeNum	1
general	info	VoxelNum	80
original	shape	Maximum3DDiameter	44.69899328
original	shape	Maximum2DDiameterSlice	13.41640786
original	shape	Sphericity	0.60890928
original	shape	MinorAxis	13.11607348
original	shape	Elongation	0.283684812
original	shape	SurfaceVolumeRatio	0.614393975
original	shape	Volume	2160
original	shape	MajorAxis	46.23466931
original	shape	SurfaceArea	1327.090985
original	shape	Flatness	0.228136557
original	shape	LeastAxis	10.54781829
original	shape	Maximum2DDiameterColumn	28.46049894
original	shape	Maximum2DDiameterRow	44.59820624
original	gldm	GrayLevelVariance	153.1560938
original	gldm	HighGrayLevelEmphasis	790.0875
original	gldm	DependenceEntropy	5.245191026
original	gldm	DependenceNonUniformity	43.975
original	gldm	GrayLevelNonUniformity	2.775
original	gldm	SmallDependenceEmphasis	0.760416667
original	gldm	SmallDependenceHighGrayLevelEmphasis	565.6979167
original	gldm	DependenceNonUniformityNormalized	0.5496875
original	gldm	LargeDependenceEmphasis	2.125
original	gldm	LargeDependenceLowGrayLevelEmphasis	0.031999392
original	gldm	DependenceVariance	0.3025

original	gldm	LargeDependenceHighGrayLevelEmphasis	1967.8125
original	gldm	SmallDependenceLowGrayLevelEmphasis	0.015899541
original	gldm	LowGrayLevelEmphasis	0.019099682
original	glcm	JointAverage	25.81411597
original	glcm	SumAverage	51.62823193
original	glcm	JointEntropy	5.858171973
original	glcm	ClusterShade	-398.384314
original	glcm	MaximumProbability	0.030727493
original	glcm	Idmn	0.952177642
original	glcm	JointEnergy	0.018273415
original	glcm	Contrast	222.9597218
original	glcm	DifferenceEntropy	4.048414288
original	glcm	InverseVariance	0.066074976
original	glcm	DifferenceVariance	74.71578919
original	glcm	Idn	0.851537987
original	glcm	Idm	0.075174118
original	glcm	Correlation	0.197578451
original	glcm	Autocorrelation	694.8298678
original	glcm	SumEntropy	4.50630159
original	glcm	SumSquares	138.8716556
original	glcm	ClusterProminence	280107.1181
original	glcm	Imc2	0.999424462
original	glcm	Imc1	-0.739491428
original	glcm	DifferenceAverage	12.09344292
original	glcm	Id	0.151153001
original	glcm	ClusterTendency	332.5269005
original	firstorder	InterquartileRange	493.25
original	firstorder	Skewness	0.178684315
original	firstorder	Uniformity	0.0346875
original	firstorder	Median	-351
original	firstorder	Energy	17885547
original	firstorder	RobustMeanAbsoluteDeviation	197.6699219
original	firstorder	MeanAbsoluteDeviation	258.0221875
original	firstorder	TotalEnergy	482909769
original	firstorder	Maximum	615
original	firstorder	RootMeanSquared	472.8311935
original	firstorder	90Percentile	29.1
original	firstorder	Minimum	-953
original	firstorder	Entropy	5.001641733
original	firstorder	Range	1568
original	firstorder	Variance	95843.51234
original	firstorder	10Percentile	-749.7
original	firstorder	Kurtosis	2.740521214
original	firstorder	Mean	-357.3875
original	glrlm	ShortRunLowGrayLevelEmphasis	0.019068503
original	glrlm	GrayLevelVariance	152.4351949
original	glrlm	LowGrayLevelRunEmphasis	0.019195118
original	glrlm	GrayLevelNonUniformityNormalized	0.034646661
original	glrlm	RunVariance	0.013447431
original	glrlm	GrayLevelNonUniformity	2.734403757
original	glrlm	LongRunEmphasis	1.041506846
original	glrlm	ShortRunHighGrayLevelEmphasis	776.8107783

original	glrlm	RunLengthNonUniformity	76.82908304
original	glrlm	ShortRunEmphasis	0.989623289
original	glrlm	LongRunHighGrayLevelEmphasis	828.6852104
original	glrlm	RunPercentage	0.986538462
original	glrlm	LongRunLowGrayLevelEmphasis	0.019701578
original	glrlm	RunEntropy	5.026172103
original	glrlm	HighGrayLevelRunEmphasis	787.1856647
original	glrlm	RunLengthNonUniformityNormalized	0.973105138
original	glszm	GrayLevelVariance	143.8578748
original	glszm	ZoneVariance	0.186233014
original	glszm	GrayLevelNonUniformityNormalized	0.034528848
original	glszm	SizeZoneNonUniformityNormalized	0.701047004
original	glszm	SizeZoneNonUniformity	46.97014925
original	glszm	GrayLevelNonUniformity	2.313432836
original	glszm	LargeAreaEmphasis	1.611940299
original	glszm	SmallAreaHighGrayLevelEmphasis	636.3810116
original	glszm	ZonePercentage	0.8375
original	glszm	LargeAreaLowGrayLevelEmphasis	0.027922046
original	glszm	LargeAreaHighGrayLevelEmphasis	1361.955224
original	glszm	HighGrayLevelZoneEmphasis	759.1940299
original	glszm	SmallAreaEmphasis	0.863598673
original	glszm	LowGrayLevelZoneEmphasis	0.020256242
original	glszm	ZoneEntropy	5.209610415
original	glszm	SmallAreaLowGrayLevelEmphasis	0.018349656
original	ngtdm	Coarseness	0.041000662
original	ngtdm	Complexity	5869.81821
original	ngtdm	Strength	33.68424807
original	ngtdm	Contrast	2.227971643
original	ngtdm	Busyness	0.033471507
wavelet-HHH	gldm	GrayLevelVariance	40.724375
wavelet-HHH	gldm	HighGrayLevelEmphasis	897.75
wavelet-HHH	gldm	DependenceEntropy	4.841882745
wavelet-HHH	gldm	DependenceNonUniformity	33.9
wavelet-HHH	gldm	GrayLevelNonUniformity	6.075
wavelet-HHH	gldm	SmallDependenceEmphasis	0.656944444
wavelet-HHH	gldm	SmallDependenceHighGrayLevelEmphasis	591.6381944
wavelet-HHH	gldm	DependenceNonUniformityNormalized	0.42375
wavelet-HHH	gldm	LargeDependenceEmphasis	3.15
wavelet-HHH	gldm	LargeDependenceLowGrayLevelEmphasis	0.016443694
wavelet-HHH	gldm	DependenceVariance	0.59
wavelet-HHH	gldm	LargeDependenceHighGrayLevelEmphasis	2807.975
wavelet-HHH	gldm	SmallDependenceLowGrayLevelEmphasis	0.013548651
wavelet-HHH	gldm	LowGrayLevelEmphasis	0.013945653
wavelet-HHH	glcm	JointAverage	29.47224697
wavelet-HHH	glcm	SumAverage	58.94449395
wavelet-HHH	glcm	JointEntropy	5.62575843
wavelet-HHH	glcm	ClusterShade	-419.5528854
wavelet-HHH	glcm	MaximumProbability	0.041218008
wavelet-HHH	glcm	Idmn	0.980175012
wavelet-HHH	glcm	JointEnergy	0.022258742
wavelet-HHH	glcm	Contrast	63.25672953
wavelet-HHH	glcm	DifferenceEntropy	3.391310432

wavelet-HHH	glcm	InverseVariance	0.200284782
wavelet-HHH	glcm	DifferenceVariance	31.28101598
wavelet-HHH	glcm	Idn	0.910773164
wavelet-HHH	glcm	Idm	0.177567255
wavelet-HHH	glcm	Correlation	-0.08058241
wavelet-HHH	glcm	Autocorrelation	866.842281
wavelet-HHH	glcm	SumEntropy	3.845982385
wavelet-HHH	glcm	SumSquares	29.55617292
wavelet-HHH	glcm	ClusterProminence	28531.27483
wavelet-HHH	glcm	Imc2	0.986432944
wavelet-HHH	glcm	Imc1	-0.49439665
wavelet-HHH	glcm	DifferenceAverage	5.54423314
wavelet-HHH	glcm	Id	0.269408426
wavelet-HHH	glcm	ClusterTendency	54.96796214
wavelet-HHH	firstorder	InterquartileRange	142.6107848
wavelet-HHH	firstorder	Skewness	-1.048720209
wavelet-HHH	firstorder	Uniformity	0.0759375
wavelet-HHH	firstorder	Median	-15.96943222
wavelet-HHH	firstorder	Energy	2067719.54
wavelet-HHH	firstorder	RobustMeanAbsoluteDeviation	61.2090332
wavelet-HHH	firstorder	MeanAbsoluteDeviation	104.0495826
wavelet-HHH	firstorder	TotalEnergy	55828427.58
wavelet-HHH	firstorder	Maximum	554.3859859
wavelet-HHH	firstorder	RootMeanSquared	160.7684492
wavelet-HHH	firstorder	90Percentile	123.6131687
wavelet-HHH	firstorder	Minimum	-722.8156954
wavelet-HHH	firstorder	Entropy	3.958206219
wavelet-HHH	firstorder	Range	1277.201681
wavelet-HHH	firstorder	Variance	25809.16586
wavelet-HHH	firstorder	10Percentile	-117.3215676
wavelet-HHH	firstorder	Kurtosis	9.600523214
wavelet-HHH	firstorder	Mean	-6.10969628
wavelet-HHH	glrlm	ShortRunLowGrayLevelEmphasis	0.014229831
wavelet-HHH	glrlm	GrayLevelVariance	41.51554753
wavelet-HHH	glrlm	LowGrayLevelRunEmphasis	0.014250575
wavelet-HHH	glrlm	GrayLevelNonUniformityNormalized	0.075235641
wavelet-HHH	glrlm	RunVariance	0.023049818
wavelet-HHH	glrlm	GrayLevelNonUniformity	5.880263626
wavelet-HHH	glrlm	LongRunEmphasis	1.071544814
wavelet-HHH	glrlm	ShortRunHighGrayLevelEmphasis	882.0039255
wavelet-HHH	glrlm	RunLengthNonUniformity	74.58495416
wavelet-HHH	glrlm	ShortRunEmphasis	0.982113797
wavelet-HHH	glrlm	LongRunHighGrayLevelEmphasis	961.6236254
wavelet-HHH	glrlm	RunPercentage	0.976923077
wavelet-HHH	glrlm	LongRunLowGrayLevelEmphasis	0.014333549
wavelet-HHH	glrlm	RunEntropy	4.059757547
wavelet-HHH	glrlm	HighGrayLevelRunEmphasis	897.9278655
wavelet-HHH	glrlm	RunLengthNonUniformityNormalized	0.953900364
wavelet-HHH	glszm	GrayLevelVariance	52.28784832
wavelet-HHH	glszm	ZoneVariance	0.602125826
wavelet-HHH	glszm	GrayLevelNonUniformityNormalized	0.068083884
wavelet-HHH	glszm	SizeZoneNonUniformityNormalized	0.629416834

wavelet-HHH	glszm	SizeZoneNonUniformity	37.13559322
wavelet-HHH	glszm	GrayLevelNonUniformity	4.016949153
wavelet-HHH	glszm	LargeAreaEmphasis	2.440677966
wavelet-HHH	glszm	SmallAreaHighGrayLevelEmphasis	736.3308098
wavelet-HHH	glszm	ZonePercentage	0.7375
wavelet-HHH	glszm	LargeAreaLowGrayLevelEmphasis	0.020097647
wavelet-HHH	glszm	LargeAreaHighGrayLevelEmphasis	2243.59322
wavelet-HHH	glszm	HighGrayLevelZoneEmphasis	894.2711864
wavelet-HHH	glszm	SmallAreaEmphasis	0.819416196
wavelet-HHH	glszm	LowGrayLevelZoneEmphasis	0.018503864
wavelet-HHH	glszm	ZoneEntropy	4.706641728
wavelet-HHH	glszm	SmallAreaLowGrayLevelEmphasis	0.018291177
wavelet-HHH	ngtdm	Coarseness	0.049779342
wavelet-HHH	ngtdm	Complexity	1340.316873
wavelet-HHH	ngtdm	Strength	18.02197015
wavelet-HHH	ngtdm	Contrast	0.947314974
wavelet-HHH	ngtdm	Busyness	0.036897152
wavelet-HLL	gldm	GrayLevelVariance	73.71234375
wavelet-HLL	gldm	HighGrayLevelEmphasis	858.4125
wavelet-HLL	gldm	DependenceEntropy	5.178378802
wavelet-HLL	gldm	DependenceNonUniformity	37.7
wavelet-HLL	gldm	GrayLevelNonUniformity	3.875
wavelet-HLL	gldm	SmallDependenceEmphasis	0.689583333
wavelet-HLL	gldm	SmallDependenceHighGrayLevelEmphasis	580.2520833
wavelet-HLL	gldm	DependenceNonUniformityNormalized	0.47125
wavelet-HLL	gldm	LargeDependenceEmphasis	2.575
wavelet-HLL	gldm	LargeDependenceLowGrayLevelEmphasis	0.016943259
wavelet-HLL	gldm	DependenceVariance	0.399375
wavelet-HLL	gldm	LargeDependenceHighGrayLevelEmphasis	2254.3875
wavelet-HLL	gldm	SmallDependenceLowGrayLevelEmphasis	0.014583667
wavelet-HLL	gldm	LowGrayLevelEmphasis	0.014973723
wavelet-HLL	glcm	JointAverage	28.18024193
wavelet-HLL	glcm	SumAverage	56.36048386
wavelet-HLL	glcm	JointEntropy	5.775632037
wavelet-HLL	glcm	ClusterShade	-1556.899995
wavelet-HLL	glcm	MaximumProbability	0.03490667
wavelet-HLL	glcm	Idmn	0.945508516
wavelet-HLL	glcm	JointEnergy	0.019765639
wavelet-HLL	glcm	Contrast	137.4464926
wavelet-HLL	glcm	DifferenceEntropy	3.670256238
wavelet-HLL	glcm	InverseVariance	0.148804543
wavelet-HLL	glcm	DifferenceVariance	59.21585879
wavelet-HLL	glcm	Idn	0.856046696
wavelet-HLL	glcm	Idm	0.135094323
wavelet-HLL	glcm	Correlation	0.108313511
wavelet-HLL	glcm	Autocorrelation	802.67637
wavelet-HLL	glcm	SumEntropy	4.261735515
wavelet-HLL	glcm	SumSquares	76.81202177
wavelet-HLL	glcm	ClusterProminence	114267.1592
wavelet-HLL	glcm	Imc2	0.998556552
wavelet-HLL	glcm	Imc1	-0.690166703
wavelet-HLL	glcm	DifferenceAverage	8.578493696

wavelet-HLL	glcm	Id	0.21806719
wavelet-HLL	glcm	ClusterTendency	169.8015945
wavelet-HLL	firstorder	InterquartileRange	213.2481605
wavelet-HLL	firstorder	Skewness	-0.686340923
wavelet-HLL	firstorder	Uniformity	0.0484375
wavelet-HLL	firstorder	Median	11.39430057
wavelet-HLL	firstorder	Energy	3705868.038
wavelet-HLL	firstorder	RobustMeanAbsoluteDeviation	107.2485599
wavelet-HLL	firstorder	MeanAbsoluteDeviation	162.0370395
wavelet-HLL	firstorder	TotalEnergy	100058437
wavelet-HLL	firstorder	Maximum	439.2871968
wavelet-HLL	firstorder	RootMeanSquared	215.2286005
wavelet-HLL	firstorder	90Percentile	260.9638783
wavelet-HLL	firstorder	Minimum	-658.9825792
wavelet-HLL	firstorder	Entropy	4.613662903
wavelet-HLL	firstorder	Range	1098.269776
wavelet-HLL	firstorder	Variance	46166.73567
wavelet-HLL	firstorder	10Percentile	-273.5448547
wavelet-HLL	firstorder	Kurtosis	3.7908054
wavelet-HLL	firstorder	Mean	12.51458349
wavelet-HLL	glrlm	ShortRunLowGrayLevelEmphasis	0.015215396
wavelet-HLL	glrlm	GrayLevelVariance	74.59785447
wavelet-HLL	glrlm	LowGrayLevelRunEmphasis	0.015233179
wavelet-HLL	glrlm	GrayLevelNonUniformityNormalized	0.04818478
wavelet-HLL	glrlm	RunVariance	0.018238921
wavelet-HLL	glrlm	GrayLevelNonUniformity	3.784383797
wavelet-HLL	glrlm	LongRunEmphasis	1.056732751
wavelet-HLL	glrlm	ShortRunHighGrayLevelEmphasis	845.1935486
wavelet-HLL	glrlm	RunLengthNonUniformity	75.71805442
wavelet-HLL	glrlm	ShortRunEmphasis	0.985816812
wavelet-HLL	glrlm	LongRunHighGrayLevelEmphasis	908.3056607
wavelet-HLL	glrlm	RunPercentage	0.981730769
wavelet-HLL	glrlm	LongRunLowGrayLevelEmphasis	0.015304311
wavelet-HLL	glrlm	RunEntropy	4.669670926
wavelet-HLL	glrlm	HighGrayLevelRunEmphasis	857.815971
wavelet-HLL	glrlm	RunLengthNonUniformityNormalized	0.963522158
wavelet-HLL	glszm	GrayLevelVariance	85.75963719
wavelet-HLL	glszm	ZoneVariance	0.260519022
wavelet-HLL	glszm	GrayLevelNonUniformityNormalized	0.046107332
wavelet-HLL	glszm	SizeZoneNonUniformityNormalized	0.624086672
wavelet-HLL	glszm	SizeZoneNonUniformity	39.31746032
wavelet-HLL	glszm	GrayLevelNonUniformity	2.904761905
wavelet-HLL	glszm	LargeAreaEmphasis	1.873015873
wavelet-HLL	glszm	SmallAreaHighGrayLevelEmphasis	683.9545855
wavelet-HLL	glszm	ZonePercentage	0.7875
wavelet-HLL	glszm	LargeAreaLowGrayLevelEmphasis	0.019769954
wavelet-HLL	glszm	LargeAreaHighGrayLevelEmphasis	1626.968254
wavelet-HLL	glszm	HighGrayLevelZoneEmphasis	848.5714286
wavelet-HLL	glszm	SmallAreaEmphasis	0.8170194
wavelet-HLL	glszm	LowGrayLevelZoneEmphasis	0.018675382
wavelet-HLL	glszm	ZoneEntropy	5.120547091
wavelet-HLL	glszm	SmallAreaLowGrayLevelEmphasis	0.018445053

wavelet-HLL	ngtdm	Coarseness	0.055980623
wavelet-HLL	ngtdm	Complexity	2378.595796
wavelet-HLL	ngtdm	Strength	26.81395533
wavelet-HLL	ngtdm	Contrast	0.946021492
wavelet-HLL	ngtdm	Busyness	0.024164935
wavelet-LHL	gldm	GrayLevelVariance	448.374375
wavelet-LHL	gldm	HighGrayLevelEmphasis	3476.125
wavelet-LHL	gldm	DependenceEntropy	5.543619814
wavelet-LHL	gldm	DependenceNonUniformity	51.3
wavelet-LHL	gldm	GrayLevelNonUniformity	2.375
wavelet-LHL	gldm	SmallDependenceEmphasis	0.827777778
wavelet-LHL	gldm	SmallDependenceHighGrayLevelEmphasis	2847.234028
wavelet-LHL	gldm	DependenceNonUniformityNormalized	0.64125
wavelet-LHL	gldm	LargeDependenceEmphasis	1.8
wavelet-LHL	gldm	LargeDependenceLowGrayLevelEmphasis	0.013381057
wavelet-LHL	gldm	DependenceVariance	0.2375
wavelet-LHL	gldm	LargeDependenceHighGrayLevelEmphasis	6226.8
wavelet-LHL	gldm	SmallDependenceLowGrayLevelEmphasis	0.01298789
wavelet-LHL	gldm	LowGrayLevelEmphasis	0.013056021
wavelet-LHL	glcm	JointAverage	57.87966393
wavelet-LHL	glcm	SumAverage	115.7593279
wavelet-LHL	glcm	JointEntropy	5.863370583
wavelet-LHL	glcm	ClusterShade	4441.600725
wavelet-LHL	glcm	MaximumProbability	0.032169801
wavelet-LHL	glcm	Idmn	0.924712198
wavelet-LHL	glcm	JointEnergy	0.018504851
wavelet-LHL	glcm	Contrast	925.2883913
wavelet-LHL	glcm	DifferenceEntropy	4.251947519
wavelet-LHL	glcm	InverseVariance	0.045152742
wavelet-LHL	glcm	DifferenceVariance	253.4400593
wavelet-LHL	glcm	Idn	0.821849508
wavelet-LHL	glcm	Idm	0.053410597
wavelet-LHL	glcm	Correlation	-0.04948864
wavelet-LHL	glcm	Autocorrelation	3331.042626
wavelet-LHL	glcm	SumEntropy	4.641090319
wavelet-LHL	glcm	SumSquares	439.127337
wavelet-LHL	glcm	ClusterProminence	2364089.769
wavelet-LHL	glcm	Imc2	0.999858585
wavelet-LHL	glcm	Imc1	-0.827136529
wavelet-LHL	glcm	DifferenceAverage	24.19850329
wavelet-LHL	glcm	Id	0.106514386
wavelet-LHL	glcm	ClusterTendency	831.2209567
wavelet-LHL	firstorder	InterquartileRange	649.9451346
wavelet-LHL	firstorder	Skewness	0.101659687
wavelet-LHL	firstorder	Uniformity	0.0296875
wavelet-LHL	firstorder	Median	-164.0450758
wavelet-LHL	firstorder	Energy	23046628.64
wavelet-LHL	firstorder	RobustMeanAbsoluteDeviation	298.6748928
wavelet-LHL	firstorder	MeanAbsoluteDeviation	426.9094932
wavelet-LHL	firstorder	TotalEnergy	622258973.3
wavelet-LHL	firstorder	Maximum	1042.948057
wavelet-LHL	firstorder	RootMeanSquared	536.7335074

wavelet-LHL	firstorder	90Percentile	619.4240523
wavelet-LHL	firstorder	Minimum	-1438.95261
wavelet-LHL	firstorder	Entropy	5.305875438
wavelet-LHL	firstorder	Range	2481.900667
wavelet-LHL	firstorder	Variance	280566.0454
wavelet-LHL	firstorder	10Percentile	-768.3907006
wavelet-LHL	firstorder	Kurtosis	2.69820603
wavelet-LHL	firstorder	Mean	-86.69955366
wavelet-LHL	glrlm	ShortRunLowGrayLevelEmphasis	0.013177814
wavelet-LHL	glrlm	GrayLevelVariance	449.6205284
wavelet-LHL	glrlm	LowGrayLevelRunEmphasis	0.013180497
wavelet-LHL	glrlm	GrayLevelNonUniformityNormalized	0.029522242
wavelet-LHL	glrlm	RunVariance	0.011582406
wavelet-LHL	glrlm	GrayLevelNonUniformity	2.339291064
wavelet-LHL	glrlm	LongRunEmphasis	1.031483285
wavelet-LHL	glrlm	ShortRunHighGrayLevelEmphasis	3450.917235
wavelet-LHL	glrlm	RunLengthNonUniformity	77.87568971
wavelet-LHL	glrlm	ShortRunEmphasis	0.993224949
wavelet-LHL	glrlm	LongRunHighGrayLevelEmphasis	3583.83886
wavelet-LHL	glrlm	RunPercentage	0.990384615
wavelet-LHL	glrlm	LongRunLowGrayLevelEmphasis	0.013193297
wavelet-LHL	glrlm	RunEntropy	5.322963278
wavelet-LHL	glrlm	HighGrayLevelRunEmphasis	3475.64664
wavelet-LHL	glrlm	RunLengthNonUniformityNormalized	0.982676487
wavelet-LHL	glszm	GrayLevelVariance	467.4540816
wavelet-LHL	glszm	ZoneVariance	0.208163265
wavelet-LHL	glszm	GrayLevelNonUniformityNormalized	0.027346939
wavelet-LHL	glszm	SizeZoneNonUniformityNormalized	0.794693878
wavelet-LHL	glszm	SizeZoneNonUniformity	55.62857143
wavelet-LHL	glszm	GrayLevelNonUniformity	1.914285714
wavelet-LHL	glszm	LargeAreaEmphasis	1.514285714
wavelet-LHL	glszm	SmallAreaHighGrayLevelEmphasis	3130.764286
wavelet-LHL	glszm	ZonePercentage	0.875
wavelet-LHL	glszm	LargeAreaLowGrayLevelEmphasis	0.015076496
wavelet-LHL	glszm	LargeAreaHighGrayLevelEmphasis	5161.657143
wavelet-LHL	glszm	HighGrayLevelZoneEmphasis	3468.928571
wavelet-LHL	glszm	SmallAreaEmphasis	0.911607143
wavelet-LHL	glszm	LowGrayLevelZoneEmphasis	0.014863756
wavelet-LHL	glszm	ZoneEntropy	5.496930695
wavelet-LHL	glszm	SmallAreaLowGrayLevelEmphasis	0.014829559
wavelet-LHL	ngtdm	Coarseness	0.024860472
wavelet-LHL	ngtdm	Complexity	26834.51523
wavelet-LHL	ngtdm	Strength	61.85418032
wavelet-LHL	ngtdm	Contrast	7.971305435
wavelet-LHL	ngtdm	Busyness	0.024126254
wavelet-LHH	gldm	GrayLevelVariance	44.71484375
wavelet-LHH	gldm	HighGrayLevelEmphasis	310.8125
wavelet-LHH	gldm	DependenceEntropy	4.986077827
wavelet-LHH	gldm	DependenceNonUniformity	41.3
wavelet-LHH	gldm	GrayLevelNonUniformity	4.525
wavelet-LHH	gldm	SmallDependenceEmphasis	0.742361111
wavelet-LHH	gldm	SmallDependenceHighGrayLevelEmphasis	247.2777778

wavelet-LHH	gldm	DependenceNonUniformityNormalized	0.51625
wavelet-LHH	gldm	LargeDependenceEmphasis	2.475
wavelet-LHH	gldm	LargeDependenceLowGrayLevelEmphasis	0.030422161
wavelet-LHH	gldm	DependenceVariance	0.444375
wavelet-LHH	gldm	LargeDependenceHighGrayLevelEmphasis	684.0625
wavelet-LHH	gldm	SmallDependenceLowGrayLevelEmphasis	0.020007196
wavelet-LHH	gldm	LowGrayLevelEmphasis	0.021526187
wavelet-LHH	glcm	JointAverage	16.29540878
wavelet-LHH	glcm	SumAverage	32.59081756
wavelet-LHH	glcm	JointEntropy	5.769816832
wavelet-LHH	glcm	ClusterShade	330.6575945
wavelet-LHH	glcm	MaximumProbability	0.035481767
wavelet-LHH	glcm	Idmn	0.955550894
wavelet-LHH	glcm	JointEnergy	0.019839127
wavelet-LHH	glcm	Contrast	72.65752995
wavelet-LHH	glcm	DifferenceEntropy	3.595080619
wavelet-LHH	glcm	InverseVariance	0.13636758
wavelet-LHH	glcm	DifferenceVariance	23.64000461
wavelet-LHH	glcm	Idn	0.855839055
wavelet-LHH	glcm	Idm	0.12533154
wavelet-LHH	glcm	Correlation	0.056256593
wavelet-LHH	glcm	Autocorrelation	268.3906708
wavelet-LHH	glcm	SumEntropy	4.14053321
wavelet-LHH	glcm	SumSquares	38.53441745
wavelet-LHH	glcm	ClusterProminence	22192.60069
wavelet-LHH	glcm	Imc2	0.996674596
wavelet-LHH	glcm	Imc1	-0.620054225
wavelet-LHH	glcm	DifferenceAverage	6.91275102
wavelet-LHH	glcm	Id	0.215567194
wavelet-LHH	glcm	ClusterTendency	81.48013986
wavelet-LHH	firstorder	InterquartileRange	222.266468
wavelet-LHH	firstorder	Skewness	0.329980595
wavelet-LHH	firstorder	Uniformity	0.0565625
wavelet-LHH	firstorder	Median	1.408779381
wavelet-LHH	firstorder	Energy	2236550.035
wavelet-LHH	firstorder	RobustMeanAbsoluteDeviation	94.3034337
wavelet-LHH	firstorder	MeanAbsoluteDeviation	132.6876733
wavelet-LHH	firstorder	TotalEnergy	60386850.95
wavelet-LHH	firstorder	Maximum	545.2927523
wavelet-LHH	firstorder	RootMeanSquared	167.2030964
wavelet-LHH	firstorder	90Percentile	187.5524353
wavelet-LHH	firstorder	Minimum	-398.1917508
wavelet-LHH	firstorder	Entropy	4.432769546
wavelet-LHH	firstorder	Range	943.4845031
wavelet-LHH	firstorder	Variance	27933.52951
wavelet-LHH	firstorder	10Percentile	-210.4046123
wavelet-LHH	firstorder	Kurtosis	3.535457376
wavelet-LHH	firstorder	Mean	-4.831762032
wavelet-LHH	glrlm	ShortRunLowGrayLevelEmphasis	0.021710996
wavelet-LHH	glrlm	GrayLevelVariance	45.0939511
wavelet-LHH	glrlm	LowGrayLevelRunEmphasis	0.021786593
wavelet-LHH	glrlm	GrayLevelNonUniformityNormalized	0.056197202

wavelet-LHH	glrlm	RunVariance	0.016332833
wavelet-LHH	glrlm	GrayLevelNonUniformity	4.422423495
wavelet-LHH	glrlm	LongRunEmphasis	1.050339145
wavelet-LHH	glrlm	ShortRunHighGrayLevelEmphasis	308.6913606
wavelet-LHH	glrlm	RunLengthNonUniformity	76.14629285
wavelet-LHH	glrlm	ShortRunEmphasis	0.987415214
wavelet-LHH	glrlm	LongRunHighGrayLevelEmphasis	324.4123989
wavelet-LHH	glrlm	RunPercentage	0.983653846
wavelet-LHH	glrlm	LongRunLowGrayLevelEmphasis	0.022088984
wavelet-LHH	glrlm	RunEntropy	4.493813576
wavelet-LHH	glrlm	HighGrayLevelRunEmphasis	311.8355683
wavelet-LHH	glrlm	RunLengthNonUniformityNormalized	0.967334334
wavelet-LHH	glszm	GrayLevelVariance	50.46059172
wavelet-LHH	glszm	ZoneVariance	0.331360947
wavelet-LHH	glszm	GrayLevelNonUniformityNormalized	0.050887574
wavelet-LHH	glszm	SizeZoneNonUniformityNormalized	0.706508876
wavelet-LHH	glszm	SizeZoneNonUniformity	45.92307692
wavelet-LHH	glszm	GrayLevelNonUniformity	3.307692308
wavelet-LHH	glszm	LargeAreaEmphasis	1.846153846
wavelet-LHH	glszm	SmallAreaHighGrayLevelEmphasis	292.5894231
wavelet-LHH	glszm	ZonePercentage	0.8125
wavelet-LHH	glszm	LargeAreaLowGrayLevelEmphasis	0.029942186
wavelet-LHH	glszm	LargeAreaHighGrayLevelEmphasis	540.3538462
wavelet-LHH	glszm	HighGrayLevelZoneEmphasis	325
wavelet-LHH	glszm	SmallAreaEmphasis	0.865918803
wavelet-LHH	glszm	LowGrayLevelZoneEmphasis	0.025154081
wavelet-LHH	glszm	ZoneEntropy	4.90539306
wavelet-LHH	glszm	SmallAreaLowGrayLevelEmphasis	0.024356575
wavelet-LHH	ngtdm	Coarseness	0.050702179
wavelet-LHH	ngtdm	Complexity	1732.908299
wavelet-LHH	ngtdm	Strength	16.58710894
wavelet-LHH	ngtdm	Contrast	0.642805631
wavelet-LHH	ngtdm	Busyness	0.049602058
wavelet-LLH	gldm	GrayLevelVariance	231.774375
wavelet-LLH	gldm	HighGrayLevelEmphasis	2014.725
wavelet-LLH	gldm	DependenceEntropy	5.370191026
wavelet-LLH	gldm	DependenceNonUniformity	63.825
wavelet-LLH	gldm	GrayLevelNonUniformity	2.625
wavelet-LLH	gldm	SmallDependenceEmphasis	0.913888889
wavelet-LLH	gldm	SmallDependenceHighGrayLevelEmphasis	1879.797222
wavelet-LLH	gldm	DependenceNonUniformityNormalized	0.7978125
wavelet-LLH	gldm	LargeDependenceEmphasis	1.4
wavelet-LLH	gldm	LargeDependenceLowGrayLevelEmphasis	0.016776939
wavelet-LLH	gldm	DependenceVariance	0.134375
wavelet-LLH	gldm	LargeDependenceHighGrayLevelEmphasis	2643.325
wavelet-LLH	gldm	SmallDependenceLowGrayLevelEmphasis	0.01643912
wavelet-LLH	gldm	LowGrayLevelEmphasis	0.016499739
wavelet-LLH	glcm	JointAverage	41.0514451
wavelet-LLH	glcm	SumAverage	82.1028902
wavelet-LLH	glcm	JointEntropy	5.864858923
wavelet-LLH	glcm	ClusterShade	-2021.381207
wavelet-LLH	glcm	MaximumProbability	0.028242456

wavelet-LLH	glcm	Idmn	0.952263715
wavelet-LLH	glcm	JointEnergy	0.018235597
wavelet-LLH	glcm	Contrast	273.8172612
wavelet-LLH	glcm	DifferenceEntropy	4.08877416
wavelet-LLH	glcm	InverseVariance	0.070852621
wavelet-LLH	glcm	DifferenceVariance	98.82971025
wavelet-LLH	glcm	Idn	0.854121062
wavelet-LLH	glcm	Idm	0.05911644
wavelet-LLH	glcm	Correlation	0.255880078
wavelet-LLH	glcm	Autocorrelation	1736.404213
wavelet-LLH	glcm	SumEntropy	4.600109121
wavelet-LLH	glcm	SumSquares	185.9722361
wavelet-LLH	glcm	ClusterProminence	645441.8605
wavelet-LLH	glcm	Imc2	0.999601862
wavelet-LLH	glcm	Imc1	-0.763188993
wavelet-LLH	glcm	DifferenceAverage	12.90203737
wavelet-LLH	glcm	Id	0.136268085
wavelet-LLH	glcm	ClusterTendency	470.0716834
wavelet-LLH	firstorder	InterquartileRange	470.1126643
wavelet-LLH	firstorder	Skewness	-0.488548121
wavelet-LLH	firstorder	Uniformity	0.0328125
wavelet-LLH	firstorder	Median	121.1600309
wavelet-LLH	firstorder	Energy	12170548.83
wavelet-LLH	firstorder	RobustMeanAbsoluteDeviation	210.0757298
wavelet-LLH	firstorder	MeanAbsoluteDeviation	301.9222179
wavelet-LLH	firstorder	TotalEnergy	328604818.4
wavelet-LLH	firstorder	Maximum	765.4454572
wavelet-LLH	firstorder	RootMeanSquared	390.0408445
wavelet-LLH	firstorder	90Percentile	560.6275102
wavelet-LLH	firstorder	Minimum	-929.1799223
wavelet-LLH	firstorder	Entropy	5.173822202
wavelet-LLH	firstorder	Range	1694.62538
wavelet-LLH	firstorder	Variance	143617.4452
wavelet-LLH	firstorder	10Percentile	-432.7216736
wavelet-LLH	firstorder	Kurtosis	2.946604417
wavelet-LLH	firstorder	Mean	92.27358862
wavelet-LLH	glrlm	ShortRunLowGrayLevelEmphasis	0.016574515
wavelet-LLH	glrlm	GrayLevelVariance	232.7007884
wavelet-LLH	glrlm	LowGrayLevelRunEmphasis	0.016577072
wavelet-LLH	glrlm	GrayLevelNonUniformityNormalized	0.03270003
wavelet-LLH	glrlm	RunVariance	0.004805966
wavelet-LLH	glrlm	GrayLevelNonUniformity	2.6035659
wavelet-LLH	glrlm	LongRunEmphasis	1.014680548
wavelet-LLH	glrlm	ShortRunHighGrayLevelEmphasis	2011.125871
wavelet-LLH	glrlm	RunLengthNonUniformity	78.85988565
wavelet-LLH	glrlm	ShortRunEmphasis	0.996329863
wavelet-LLH	glrlm	LongRunHighGrayLevelEmphasis	2039.96491
wavelet-LLH	glrlm	RunPercentage	0.995192308
wavelet-LLH	glrlm	LongRunLowGrayLevelEmphasis	0.016587299
wavelet-LLH	glrlm	RunEntropy	5.188347886
wavelet-LLH	glrlm	HighGrayLevelRunEmphasis	2016.893679
wavelet-LLH	glrlm	RunLengthNonUniformityNormalized	0.990388068

wavelet-LLH	glszm	GrayLevelVariance	244.3779556
wavelet-LLH	glszm	ZoneVariance	0.088888889
wavelet-LLH	glszm	GrayLevelNonUniformityNormalized	0.031466667
wavelet-LLH	glszm	SizeZoneNonUniformityNormalized	0.897955556
wavelet-LLH	glszm	SizeZoneNonUniformity	67.34666667
wavelet-LLH	glszm	GrayLevelNonUniformity	2.36
wavelet-LLH	glszm	LargeAreaEmphasis	1.226666667
wavelet-LLH	glszm	SmallAreaHighGrayLevelEmphasis	1978.957037
wavelet-LLH	glszm	ZonePercentage	0.9375
wavelet-LLH	glszm	LargeAreaLowGrayLevelEmphasis	0.017709393
wavelet-LLH	glszm	LargeAreaHighGrayLevelEmphasis	2400.986667
wavelet-LLH	glszm	HighGrayLevelZoneEmphasis	2044.4
wavelet-LLH	glszm	SmallAreaEmphasis	0.958148148
wavelet-LLH	glszm	LowGrayLevelZoneEmphasis	0.01755322
wavelet-LLH	glszm	ZoneEntropy	5.320299151
wavelet-LLH	glszm	SmallAreaLowGrayLevelEmphasis	0.017523436
wavelet-LLH	ngtdm	Coarseness	0.049516106
wavelet-LLH	ngtdm	Complexity	9145.79827
wavelet-LLH	ngtdm	Strength	64.50675657
wavelet-LLH	ngtdm	Contrast	2.361555826
wavelet-LLH	ngtdm	Busyness	0.012991604
wavelet-LLL	gldm	GrayLevelVariance	562.25
wavelet-LLL	gldm	HighGrayLevelEmphasis	2866.25
wavelet-LLL	gldm	DependenceEntropy	5.718619814
wavelet-LLL	gldm	DependenceNonUniformity	47.675
wavelet-LLL	gldm	GrayLevelNonUniformity	1.975
wavelet-LLL	gldm	SmallDependenceEmphasis	0.797916667
wavelet-LLL	gldm	SmallDependenceHighGrayLevelEmphasis	2380.104167
wavelet-LLL	gldm	DependenceNonUniformityNormalized	0.5959375
wavelet-LLL	gldm	LargeDependenceEmphasis	1.975
wavelet-LLL	gldm	LargeDependenceLowGrayLevelEmphasis	0.014494529
wavelet-LLL	gldm	DependenceVariance	0.285
wavelet-LLL	gldm	LargeDependenceHighGrayLevelEmphasis	5133.5
wavelet-LLL	gldm	SmallDependenceLowGrayLevelEmphasis	0.013409425
wavelet-LLL	gldm	LowGrayLevelEmphasis	0.013609228
wavelet-LLL	glcm	JointAverage	49.72880221
wavelet-LLL	glcm	SumAverage	99.45760441
wavelet-LLL	glcm	JointEntropy	5.888292773
wavelet-LLL	glcm	ClusterShade	9079.201769
wavelet-LLL	glcm	MaximumProbability	0.029765955
wavelet-LLL	glcm	Idmn	0.959015082
wavelet-LLL	glcm	JointEnergy	0.017886047
wavelet-LLL	glcm	Contrast	607.3352572
wavelet-LLL	glcm	DifferenceEntropy	4.298182612
wavelet-LLL	glcm	InverseVariance	0.06024031
wavelet-LLL	glcm	DifferenceVariance	214.168196
wavelet-LLL	glcm	Idn	0.864301061
wavelet-LLL	glcm	Idm	0.06598073
wavelet-LLL	glcm	Correlation	0.471514761
wavelet-LLL	glcm	Autocorrelation	2738.201365
wavelet-LLL	glcm	SumEntropy	4.766383295
wavelet-LLL	glcm	SumSquares	563.9479391

wavelet-LLL	glcm	ClusterProminence	5245340.197
wavelet-LLL	glcm	Imc2	0.999934555
wavelet-LLL	glcm	Imc1	-0.866855244
wavelet-LLL	glcm	DifferenceAverage	19.45147421
wavelet-LLL	glcm	Id	0.123402176
wavelet-LLL	glcm	ClusterTendency	1648.456499
wavelet-LLL	firstorder	InterquartileRange	928.1684194
wavelet-LLL	firstorder	Skewness	0.431009447
wavelet-LLL	firstorder	Uniformity	0.0246875
wavelet-LLL	firstorder	Median	-1333.033882
wavelet-LLL	firstorder	Energy	150448549.6
wavelet-LLL	firstorder	RobustMeanAbsoluteDeviation	343.2282859
wavelet-LLL	firstorder	MeanAbsoluteDeviation	481.5954158
wavelet-LLL	firstorder	TotalEnergy	4062110840
wavelet-LLL	firstorder	Maximum	408.8449784
wavelet-LLL	firstorder	RootMeanSquared	1371.352205
wavelet-LLL	firstorder	90Percentile	-448.2417077
wavelet-LLL	firstorder	Minimum	-2420.617554
wavelet-LLL	firstorder	Entropy	5.567371495
wavelet-LLL	firstorder	Range	2829.462533
wavelet-LLL	firstorder	Variance	351608.019
wavelet-LLL	firstorder	10Percentile	-1926.287344
wavelet-LLL	firstorder	Kurtosis	2.627705686
wavelet-LLL	firstorder	Mean	-1236.526931
wavelet-LLL	glrlm	ShortRunLowGrayLevelEmphasis	0.013750241
wavelet-LLL	glrlm	GrayLevelVariance	564.8158422
wavelet-LLL	glrlm	LowGrayLevelRunEmphasis	0.01375854
wavelet-LLL	glrlm	GrayLevelNonUniformityNormalized	0.024547331
wavelet-LLL	glrlm	RunVariance	0.011532788
wavelet-LLL	glrlm	GrayLevelNonUniformity	1.941274686
wavelet-LLL	glrlm	LongRunEmphasis	1.035353157
wavelet-LLL	glrlm	ShortRunHighGrayLevelEmphasis	2851.467587
wavelet-LLL	glrlm	RunLengthNonUniformity	77.27011709
wavelet-LLL	glrlm	ShortRunEmphasis	0.991161711
wavelet-LLL	glrlm	LongRunHighGrayLevelEmphasis	2955.523463
wavelet-LLL	glrlm	RunPercentage	0.988461538
wavelet-LLL	glrlm	LongRunLowGrayLevelEmphasis	0.013791734
wavelet-LLL	glrlm	RunEntropy	5.585742634
wavelet-LLL	glrlm	HighGrayLevelRunEmphasis	2872.278762
wavelet-LLL	glrlm	RunLengthNonUniformityNormalized	0.976934424
wavelet-LLL	glszm	GrayLevelVariance	592.4515858
wavelet-LLL	glszm	ZoneVariance	0.162990968
wavelet-LLL	glszm	GrayLevelNonUniformityNormalized	0.02289435
wavelet-LLL	glszm	SizeZoneNonUniformityNormalized	0.748372191
wavelet-LLL	glszm	SizeZoneNonUniformity	51.63768116
wavelet-LLL	glszm	GrayLevelNonUniformity	1.579710145
wavelet-LLL	glszm	LargeAreaEmphasis	1.507246377
wavelet-LLL	glszm	SmallAreaHighGrayLevelEmphasis	2671.834944
wavelet-LLL	glszm	ZonePercentage	0.8625
wavelet-LLL	glszm	LargeAreaLowGrayLevelEmphasis	0.016105988
wavelet-LLL	glszm	LargeAreaHighGrayLevelEmphasis	4143.304348
wavelet-LLL	glszm	HighGrayLevelZoneEmphasis	2941.188406

wavelet-LLL	glszm	SmallAreaEmphasis	0.889291465
wavelet-LLL	glszm	LowGrayLevelZoneEmphasis	0.015622714
wavelet-LLL	glszm	ZoneEntropy	5.738817573
wavelet-LLL	glszm	SmallAreaLowGrayLevelEmphasis	0.015510213
wavelet-LLL	ngtdm	Coarseness	0.037319558
wavelet-LLL	ngtdm	Complexity	24913.47998
wavelet-LLL	ngtdm	Strength	127.3508513
wavelet-LLL	ngtdm	Contrast	5.289053619
wavelet-LLL	ngtdm	Busyness	0.014890582
wavelet-HHL	gldm	GrayLevelVariance	35.69359375
wavelet-HHL	gldm	HighGrayLevelEmphasis	208.2875
wavelet-HHL	gldm	DependenceEntropy	4.866359695
wavelet-HHL	gldm	DependenceNonUniformity	40.25
wavelet-HHL	gldm	GrayLevelNonUniformity	5.125
wavelet-HHL	gldm	SmallDependenceEmphasis	0.731944444
wavelet-HHL	gldm	SmallDependenceHighGrayLevelEmphasis	167.2486111
wavelet-HHL	gldm	DependenceNonUniformityNormalized	0.503125
wavelet-HHL	gldm	LargeDependenceEmphasis	2.85
wavelet-HHL	gldm	LargeDependenceLowGrayLevelEmphasis	0.074990444
wavelet-HHL	gldm	DependenceVariance	0.674375
wavelet-HHL	gldm	LargeDependenceHighGrayLevelEmphasis	512.3875
wavelet-HHL	gldm	SmallDependenceLowGrayLevelEmphasis	0.035275464
wavelet-HHL	gldm	LowGrayLevelEmphasis	0.042319395
wavelet-HHL	glcm	JointAverage	12.65105825
wavelet-HHL	glcm	SumAverage	25.30211651
wavelet-HHL	glcm	JointEntropy	5.692340368
wavelet-HHL	glcm	ClusterShade	135.4621611
wavelet-HHL	glcm	MaximumProbability	0.038837282
wavelet-HHL	glcm	Idmn	0.947886777
wavelet-HHL	glcm	JointEnergy	0.021276555
wavelet-HHL	glcm	Contrast	72.31721541
wavelet-HHL	glcm	DifferenceEntropy	3.535481482
wavelet-HHL	glcm	InverseVariance	0.158158065
wavelet-HHL	glcm	DifferenceVariance	26.29927307
wavelet-HHL	glcm	Idn	0.849787384
wavelet-HHL	glcm	Idm	0.139562054
wavelet-HHL	glcm	Correlation	-0.087186512
wavelet-HHL	glcm	Autocorrelation	157.4418185
wavelet-HHL	glcm	SumEntropy	4.069120674
wavelet-HHL	glcm	SumSquares	33.44315175
wavelet-HHL	glcm	ClusterProminence	14689.77696
wavelet-HHL	glcm	Imc2	0.995243306
wavelet-HHL	glcm	Imc1	-0.590474168
wavelet-HHL	glcm	DifferenceAverage	6.623193825
wavelet-HHL	glcm	Id	0.229970128
wavelet-HHL	glcm	ClusterTendency	61.45539158
wavelet-HHL	firstorder	InterquartileRange	181.5981632
wavelet-HHL	firstorder	Skewness	0.410159212
wavelet-HHL	firstorder	Uniformity	0.0640625
wavelet-HHL	firstorder	Median	5.853889355
wavelet-HHL	firstorder	Energy	1775186.254
wavelet-HHL	firstorder	RobustMeanAbsoluteDeviation	74.96479727

wavelet-HHL	firstorder	MeanAbsoluteDeviation	113.3640093
wavelet-HHL	firstorder	TotalEnergy	47930028.86
wavelet-HHL	firstorder	Maximum	519.8967379
wavelet-HHL	firstorder	RootMeanSquared	148.9625059
wavelet-HHL	firstorder	90Percentile	173.4245082
wavelet-HHL	firstorder	Minimum	-316.238285
wavelet-HHL	firstorder	Entropy	4.244179226
wavelet-HHL	firstorder	Range	836.1350229
wavelet-HHL	firstorder	Variance	22098.20215
wavelet-HHL	firstorder	10Percentile	-177.8423948
wavelet-HHL	firstorder	Kurtosis	4.023787698
wavelet-HHL	firstorder	Mean	-9.572148656
wavelet-HHL	glrlm	ShortRunLowGrayLevelEmphasis	0.042404819
wavelet-HHL	glrlm	GrayLevelVariance	36.02605313
wavelet-HHL	glrlm	LowGrayLevelRunEmphasis	0.042713059
wavelet-HHL	glrlm	GrayLevelNonUniformityNormalized	0.063211967
wavelet-HHL	glrlm	RunVariance	0.018231844
wavelet-HHL	glrlm	GrayLevelNonUniformity	4.964653696
wavelet-HHL	glrlm	LongRunEmphasis	1.057041262
wavelet-HHL	glrlm	ShortRunHighGrayLevelEmphasis	206.980507
wavelet-HHL	glrlm	RunLengthNonUniformity	75.73450836
wavelet-HHL	glrlm	ShortRunEmphasis	0.985739684
wavelet-HHL	glrlm	LongRunHighGrayLevelEmphasis	218.2676219
wavelet-HHL	glrlm	RunPercentage	0.981730769
wavelet-HHL	glrlm	LongRunLowGrayLevelEmphasis	0.04394602
wavelet-HHL	glrlm	RunEntropy	4.313365961
wavelet-HHL	glrlm	HighGrayLevelRunEmphasis	209.23793
wavelet-HHL	glrlm	RunLengthNonUniformityNormalized	0.963536313
wavelet-HHL	glszm	GrayLevelVariance	39.99546485
wavelet-HHL	glszm	ZoneVariance	0.895439657
wavelet-HHL	glszm	GrayLevelNonUniformityNormalized	0.057193248
wavelet-HHL	glszm	SizeZoneNonUniformityNormalized	0.72436382
wavelet-HHL	glszm	SizeZoneNonUniformity	45.63492063
wavelet-HHL	glszm	GrayLevelNonUniformity	3.603174603
wavelet-HHL	glszm	LargeAreaEmphasis	2.507936508
wavelet-HHL	glszm	SmallAreaHighGrayLevelEmphasis	203.8810626
wavelet-HHL	glszm	ZonePercentage	0.7875
wavelet-HHL	glszm	LargeAreaLowGrayLevelEmphasis	0.069789099
wavelet-HHL	glszm	LargeAreaHighGrayLevelEmphasis	484.7777778
wavelet-HHL	glszm	HighGrayLevelZoneEmphasis	221.6031746
wavelet-HHL	glszm	SmallAreaEmphasis	0.875027557
wavelet-HHL	glszm	LowGrayLevelZoneEmphasis	0.047545685
wavelet-HHL	glszm	ZoneEntropy	4.660840289
wavelet-HHL	glszm	SmallAreaLowGrayLevelEmphasis	0.04332638
wavelet-HHL	ngtdm	Coarseness	0.055543777
wavelet-HHL	ngtdm	Complexity	1096.115888
wavelet-HHL	ngtdm	Strength	11.28472009
wavelet-HHL	ngtdm	Contrast	0.703990667
wavelet-HHL	ngtdm	Busyness	0.075125463
wavelet-HLH	gldm	GrayLevelVariance	41.8775
wavelet-HLH	gldm	HighGrayLevelEmphasis	360.5
wavelet-HLH	gldm	DependenceEntropy	5.115311532

wavelet-HLH	gldm	DependenceNonUniformity	33.375
wavelet-HLH	gldm	GrayLevelNonUniformity	4.325
wavelet-HLH	gldm	SmallDependenceEmphasis	0.627517361
wavelet-HLH	gldm	SmallDependenceHighGrayLevelEmphasis	227.8858507
wavelet-HLH	gldm	DependenceNonUniformityNormalized	0.4171875
wavelet-HLH	gldm	LargeDependenceEmphasis	3.075
wavelet-HLH	gldm	LargeDependenceLowGrayLevelEmphasis	0.028670344
wavelet-HLH	gldm	DependenceVariance	0.515
wavelet-HLH	gldm	LargeDependenceHighGrayLevelEmphasis	1064.875
wavelet-HLH	gldm	SmallDependenceLowGrayLevelEmphasis	0.018849654
wavelet-HLH	gldm	LowGrayLevelEmphasis	0.020373912
wavelet-HLH	glcm	JointAverage	17.86747509
wavelet-HLH	glcm	SumAverage	35.73495018
wavelet-HLH	glcm	JointEntropy	5.772443844
wavelet-HLH	glcm	ClusterShade	11.92819704
wavelet-HLH	glcm	MaximumProbability	0.035214673
wavelet-HLH	glcm	Idmn	0.95153579
wavelet-HLH	glcm	JointEnergy	0.019813336
wavelet-HLH	glcm	Contrast	73.42156286
wavelet-HLH	glcm	DifferenceEntropy	3.526361257
wavelet-HLH	glcm	InverseVariance	0.127152939
wavelet-HLH	glcm	DifferenceVariance	26.3685038
wavelet-HLH	glcm	Idn	0.854187527
wavelet-HLH	glcm	Idm	0.138921593
wavelet-HLH	glcm	Correlation	-0.051352486
wavelet-HLH	glcm	Autocorrelation	317.9234952
wavelet-HLH	glcm	SumEntropy	4.112714956
wavelet-HLH	glcm	SumSquares	34.71693416
wavelet-HLH	glcm	ClusterProminence	15325.87521
wavelet-HLH	glcm	Imc2	0.997093287
wavelet-HLH	glcm	Imc1	-0.624570305
wavelet-HLH	glcm	DifferenceAverage	6.733615343
wavelet-HLH	glcm	Id	0.231050806
wavelet-HLH	glcm	ClusterTendency	65.44617377
wavelet-HLH	firstorder	InterquartileRange	181.3594037
wavelet-HLH	firstorder	Skewness	-0.164945258
wavelet-HLH	firstorder	Uniformity	0.0540625
wavelet-HLH	firstorder	Median	-6.187844462
wavelet-HLH	firstorder	Energy	2103123.801
wavelet-HLH	firstorder	RobustMeanAbsoluteDeviation	85.42299761
wavelet-HLH	firstorder	MeanAbsoluteDeviation	125.4574094
wavelet-HLH	firstorder	TotalEnergy	56784342.64
wavelet-HLH	firstorder	Maximum	441.8283419
wavelet-HLH	firstorder	RootMeanSquared	162.1389759
wavelet-HLH	firstorder	90Percentile	168.7366909
wavelet-HLH	firstorder	Minimum	-432.05882
wavelet-HLH	firstorder	Entropy	4.433460515
wavelet-HLH	firstorder	Range	873.8871619
wavelet-HLH	firstorder	Variance	26029.16086
wavelet-HLH	firstorder	10Percentile	-239.1288259
wavelet-HLH	firstorder	Kurtosis	3.230313745
wavelet-HLH	firstorder	Mean	-16.12100045

wavelet-HLH	glrlm	ShortRunLowGrayLevelEmphasis	0.020693707
wavelet-HLH	glrlm	GrayLevelVariance	42.37430356
wavelet-HLH	glrlm	LowGrayLevelRunEmphasis	0.020765838
wavelet-HLH	glrlm	GrayLevelNonUniformityNormalized	0.05369434
wavelet-HLH	glrlm	RunVariance	0.023036006
wavelet-HLH	glrlm	GrayLevelNonUniformity	4.196940745
wavelet-HLH	glrlm	LongRunEmphasis	1.071871459
wavelet-HLH	glrlm	ShortRunHighGrayLevelEmphasis	354.5790974
wavelet-HLH	glrlm	RunLengthNonUniformity	74.60237523
wavelet-HLH	glrlm	ShortRunEmphasis	0.982032135
wavelet-HLH	glrlm	LongRunHighGrayLevelEmphasis	385.7736465
wavelet-HLH	glrlm	RunPercentage	0.976923077
wavelet-HLH	glrlm	LongRunLowGrayLevelEmphasis	0.021054363
wavelet-HLH	glrlm	RunEntropy	4.510301138
wavelet-HLH	glrlm	HighGrayLevelRunEmphasis	360.8180072
wavelet-HLH	glrlm	RunLengthNonUniformityNormalized	0.953927988
wavelet-HLH	glszm	GrayLevelVariance	49.52794293
wavelet-HLH	glszm	ZoneVariance	0.476813317
wavelet-HLH	glszm	GrayLevelNonUniformityNormalized	0.048156956
wavelet-HLH	glszm	SizeZoneNonUniformityNormalized	0.565398335
wavelet-HLH	glszm	SizeZoneNonUniformity	32.79310345
wavelet-HLH	glszm	GrayLevelNonUniformity	2.793103448
wavelet-HLH	glszm	LargeAreaEmphasis	2.379310345
wavelet-HLH	glszm	SmallAreaHighGrayLevelEmphasis	284.2898707
wavelet-HLH	glszm	ZonePercentage	0.725
wavelet-HLH	glszm	LargeAreaLowGrayLevelEmphasis	0.032112013
wavelet-HLH	glszm	LargeAreaHighGrayLevelEmphasis	829.5862069
wavelet-HLH	glszm	HighGrayLevelZoneEmphasis	364.8965517
wavelet-HLH	glszm	SmallAreaEmphasis	0.780292146
wavelet-HLH	glszm	LowGrayLevelZoneEmphasis	0.026561282
wavelet-HLH	glszm	ZoneEntropy	5.086345004
wavelet-HLH	glszm	SmallAreaLowGrayLevelEmphasis	0.025649651
wavelet-HLH	ngtdm	Coarseness	0.054071094
wavelet-HLH	ngtdm	Complexity	1565.828522
wavelet-HLH	ngtdm	Strength	13.092956
wavelet-HLH	ngtdm	Contrast	0.744998773
wavelet-HLH	ngtdm	Busyness	0.046339689

Image type	Feature Class	Feature Name	4: RTSTRUCT: C99-DNU_segment_GTV
general	info	BoundingBox	(1, 1, 1, 9, 8, 6)
general	info	EnabledImageTypes	{u'Original': {}, u'Wavelet': {}}
general	info	GeneralSettings	{'distances': [1], 'additionalInfo': True, 'force2D': False, 'interpolator': 'sitkBSpline', 'resampledPixelSpacing': [3.0], 'label': 1, 'normalizeScale': 1, 'normalize': False, 'force2Ddimension': 0, 'removeOutliers': None, 'minimumROISize': None, u'binWidth': 25.0, 'minimumROIDimensions': 1, u'correctMask': True, 'preCrop': False, u'symmetricalGLCM': True, 'resegmentRange': None, 'padDistance': 5}
general	info	ImageHash	fb903779eda4d6a3b7f16a49487ba60614877219
general	info	ImageSpacing	(0.9765625000000002, 0.9765625000000002, 2.0)
general	info	MaskHash	6a4dd7c53befe42661a583ae26ecc80d128598 1b
general	info	NumpyVersion	1.13.1
general	info	PyWaveletVersion	0.5.2
general	info	SimpleITKVersion	1.1.0
general	info	Version	1.3.0.post102+g1b0eb74
general	info	VolumeNum	1
general	info	VoxelNum	210
original	shape	Maximum3DDiameter	29.69848481
original	shape	Maximum2DDiameterSlice	28.3019434
original	shape	Sphericity	0.830953464
original	shape	MinorAxis	19.43341552
original	shape	Elongation	0.722978895
original	shape	SurfaceVolumeRatio	0.326372133
original	shape	Volume	5670
original	shape	MajorAxis	26.87964429
original	shape	SurfaceArea	1850.529991
original	shape	Flatness	0.590661645
original	shape	LeastAxis	15.87677492
original	shape	Maximum2DDiameterColumn	25.63201124
original	shape	Maximum2DDiameterRow	21.63330765
original	gldm	GrayLevelVariance	75.53868481
original	gldm	HighGrayLevelEmphasis	567.5333333
original	gldm	DependenceEntropy	5.902540643
original	gldm	DependenceNonUniformity	52.36190476
original	gldm	GrayLevelNonUniformity	13.28571429
original	gldm	SmallDependenceEmphasis	0.504589006
original	gldm	SmallDependenceHighGrayLevelEmphasis	183.2284815
original	gldm	DependenceNonUniformityNormalized	0.249342404
original	gldm	LargeDependenceEmphasis	15.62857143
original	gldm	LargeDependenceLowGrayLevelEmphasis	0.032395015
original	gldm	DependenceVariance	7.079909297
original	gldm	LargeDependenceHighGrayLevelEmphasis	13515.1619
original	gldm	SmallDependenceLowGrayLevelEmphasis	0.00922689

original	gldm	LowGrayLevelEmphasis	0.010972732
original	glcm	JointAverage	24.21689344
original	glcm	SumAverage	48.43378687
original	glcm	JointEntropy	6.902932769
original	glcm	ClusterShade	-1888.063444
original	glcm	MaximumProbability	0.055566513
original	glcm	Idmn	0.928698439
original	glcm	JointEnergy	0.014008235
original	glcm	Contrast	99.52699988
original	glcm	DifferenceEntropy	4.05244682
original	glcm	InverseVariance	0.231437564
original	glcm	DifferenceVariance	47.23036405
original	glcm	Idn	0.846148057
original	glcm	Idm	0.237877878
original	glcm	Correlation	0.229286644
original	glcm	Autocorrelation	601.9067016
original	glcm	SumEntropy	4.923828017
original	glcm	SumSquares	65.06161498
original	glcm	ClusterProminence	79161.47308
original	glcm	Imc2	0.964928512
original	glcm	Imc1	-0.328949172
original	glcm	DifferenceAverage	7.082793872
original	glcm	Id	0.304545644
original	glcm	ClusterTendency	160.71946
original	firstorder	InterquartileRange	373
original	firstorder	Skewness	-0.649206355
original	firstorder	Uniformity	0.063265306
original	firstorder	Median	-95.5
original	firstorder	Energy	16915690
original	firstorder	RobustMeanAbsoluteDeviation	156.1955782
original	firstorder	MeanAbsoluteDeviation	191.4767347
original	firstorder	TotalEnergy	456723630
original	firstorder	Maximum	83
original	firstorder	RootMeanSquared	283.8149129
original	firstorder	90Percentile	32.2
original	firstorder	Minimum	-718
original	firstorder	Entropy	4.469079255
original	firstorder	Range	801
original	firstorder	Variance	47142.02821
original	firstorder	10Percentile	-534.2
original	firstorder	Kurtosis	2.033779665
original	firstorder	Mean	-182.7809524
original	glrlm	ShortRunLowGrayLevelEmphasis	0.011580892
original	glrlm	GrayLevelVariance	76.00819797
original	glrlm	LowGrayLevelRunEmphasis	0.011694864
original	glrlm	GrayLevelNonUniformityNormalized	0.055378826
original	glrlm	RunVariance	0.093416299
original	glrlm	GrayLevelNonUniformity	10.77549168
original	glrlm	LongRunEmphasis	1.261299175
original	glrlm	ShortRunHighGrayLevelEmphasis	497.7757005
original	glrlm	RunLengthNonUniformity	168.8084243
original	glrlm	ShortRunEmphasis	0.945378538
original	glrlm	LongRunHighGrayLevelEmphasis	769.0788251

original	glrlm	RunPercentage	0.926007326
original	glrlm	LongRunLowGrayLevelEmphasis	0.012198085
original	glrlm	RunEntropy	4.832397006
original	glrlm	HighGrayLevelRunEmphasis	544.2860912
original	glrlm	RunLengthNonUniformityNormalized	0.86735631
original	glszm	GrayLevelVariance	60.17194707
original	glszm	ZoneVariance	14.19584121
original	glszm	GrayLevelNonUniformityNormalized	0.042722117
original	glszm	SizeZoneNonUniformityNormalized	0.657013233
original	glszm	SizeZoneNonUniformity	75.55652174
original	glszm	GrayLevelNonUniformity	4.913043478
original	glszm	LargeAreaEmphasis	17.53043478
original	glszm	SmallAreaHighGrayLevelEmphasis	277.5755438
original	glszm	ZonePercentage	0.547619048
original	glszm	LargeAreaLowGrayLevelEmphasis	0.040780464
original	glszm	LargeAreaHighGrayLevelEmphasis	15106.62609
original	glszm	HighGrayLevelZoneEmphasis	372.0782609
original	glszm	SmallAreaEmphasis	0.835678125
original	glszm	LowGrayLevelZoneEmphasis	0.017591414
original	glszm	ZoneEntropy	5.182155305
original	glszm	SmallAreaLowGrayLevelEmphasis	0.016371817
original	ngtdm	Coarseness	0.015854388
original	ngtdm	Complexity	2519.81904
original	ngtdm	Strength	9.588214394
original	ngtdm	Contrast	0.896612329
original	ngtdm	Busyness	0.07340041
wavelet-HHH	gldm	GrayLevelVariance	8.791746032
wavelet-HHH	gldm	HighGrayLevelEmphasis	98.40952381
wavelet-HHH	gldm	DependenceEntropy	5.600279495
wavelet-HHH	gldm	DependenceNonUniformity	38.2
wavelet-HHH	gldm	GrayLevelNonUniformity	22.66666667
wavelet-HHH	gldm	SmallDependenceEmphasis	0.3381721
wavelet-HHH	gldm	SmallDependenceHighGrayLevelEmphasis	31.60821233
wavelet-HHH	gldm	DependenceNonUniformityNormalized	0.181904762
wavelet-HHH	gldm	LargeDependenceEmphasis	12.34285714
wavelet-HHH	gldm	LargeDependenceLowGrayLevelEmphasis	0.15764324
wavelet-HHH	gldm	DependenceVariance	3.342857143
wavelet-HHH	gldm	LargeDependenceHighGrayLevelEmphasis	1220.904762
wavelet-HHH	gldm	SmallDependenceLowGrayLevelEmphasis	0.019243292
wavelet-HHH	gldm	LowGrayLevelEmphasis	0.028067596
wavelet-HHH	glcm	JointAverage	9.464795729
wavelet-HHH	glcm	SumAverage	18.92959146
wavelet-HHH	glcm	JointEntropy	6.355092073
wavelet-HHH	glcm	ClusterShade	-15.0429497
wavelet-HHH	glcm	MaximumProbability	0.043208701
wavelet-HHH	glcm	Idmn	0.95107267
wavelet-HHH	glcm	JointEnergy	0.016403952
wavelet-HHH	glcm	Contrast	16.69195452
wavelet-HHH	glcm	DifferenceEntropy	3.095279874
wavelet-HHH	glcm	InverseVariance	0.277569309
wavelet-HHH	glcm	DifferenceVariance	6.606516909
wavelet-HHH	glcm	Idn	0.856371838
wavelet-HHH	glcm	Idm	0.276427922

wavelet-HHH	glcm	Correlation	-0.028593553
wavelet-HHH	glcm	Autocorrelation	89.330958
wavelet-HHH	glcm	SumEntropy	3.879813335
wavelet-HHH	glcm	SumSquares	8.089935396
wavelet-HHH	glcm	ClusterProminence	840.6767448
wavelet-HHH	glcm	Imc2	0.819724535
wavelet-HHH	glcm	Imc1	-0.162895372
wavelet-HHH	glcm	DifferenceAverage	3.150171901
wavelet-HHH	glcm	Id	0.365031764
wavelet-HHH	glcm	ClusterTendency	15.66778707
wavelet-HHH	firstorder	InterquartileRange	84.95398056
wavelet-HHH	firstorder	Skewness	-0.319557242
wavelet-HHH	firstorder	Uniformity	0.107936508
wavelet-HHH	firstorder	Median	3.811263588
wavelet-HHH	firstorder	Energy	1159017.267
wavelet-HHH	firstorder	RobustMeanAbsoluteDeviation	37.51105762
wavelet-HHH	firstorder	MeanAbsoluteDeviation	57.06841513
wavelet-HHH	firstorder	TotalEnergy	31293466.22
wavelet-HHH	firstorder	Maximum	187.4856989
wavelet-HHH	firstorder	RootMeanSquared	74.29084631
wavelet-HHH	firstorder	90Percentile	96.55990029
wavelet-HHH	firstorder	Minimum	-203.9240533
wavelet-HHH	firstorder	Entropy	3.514888855
wavelet-HHH	firstorder	Range	391.4097521
wavelet-HHH	firstorder	Variance	5518.738713
wavelet-HHH	firstorder	10Percentile	-97.57614013
wavelet-HHH	firstorder	Kurtosis	3.358742101
wavelet-HHH	firstorder	Mean	-0.625405518
wavelet-HHH	glrlm	ShortRunLowGrayLevelEmphasis	0.028694442
wavelet-HHH	glrlm	GrayLevelVariance	9.249280073
wavelet-HHH	glrlm	LowGrayLevelRunEmphasis	0.029423226
wavelet-HHH	glrlm	GrayLevelNonUniformityNormalized	0.103049694
wavelet-HHH	glrlm	RunVariance	0.085802322
wavelet-HHH	glrlm	GrayLevelNonUniformity	19.98246951
wavelet-HHH	glrlm	LongRunEmphasis	1.262330128
wavelet-HHH	glrlm	ShortRunHighGrayLevelEmphasis	92.30163047
wavelet-HHH	glrlm	RunLengthNonUniformity	165.8744864
wavelet-HHH	glrlm	ShortRunEmphasis	0.939565099
wavelet-HHH	glrlm	LongRunHighGrayLevelEmphasis	124.4838167
wavelet-HHH	glrlm	RunPercentage	0.923076923
wavelet-HHH	glrlm	LongRunLowGrayLevelEmphasis	0.032544412
wavelet-HHH	glrlm	RunEntropy	3.901941636
wavelet-HHH	glrlm	HighGrayLevelRunEmphasis	98.31011132
wavelet-HHH	glrlm	RunLengthNonUniformityNormalized	0.854289975
wavelet-HHH	glszm	GrayLevelVariance	15.13146936
wavelet-HHH	glszm	ZoneVariance	23.41701368
wavelet-HHH	glszm	GrayLevelNonUniformityNormalized	0.076145152
wavelet-HHH	glszm	SizeZoneNonUniformityNormalized	0.436644854
wavelet-HHH	glszm	SizeZoneNonUniformity	35.80487805
wavelet-HHH	glszm	GrayLevelNonUniformity	6.243902439
wavelet-HHH	glszm	LargeAreaEmphasis	29.97560976
wavelet-HHH	glszm	SmallAreaHighGrayLevelEmphasis	61.88904261
wavelet-HHH	glszm	ZonePercentage	0.39047619

wavelet-HHH	glszm	LargeAreaLowGrayLevelEmphasis	0.371743309
wavelet-HHH	glszm	LargeAreaHighGrayLevelEmphasis	2935.341463
wavelet-HHH	glszm	HighGrayLevelZoneEmphasis	93.95121951
wavelet-HHH	glszm	SmallAreaEmphasis	0.682803535
wavelet-HHH	glszm	LowGrayLevelZoneEmphasis	0.052119418
wavelet-HHH	glszm	ZoneEntropy	5.043034322
wavelet-HHH	glszm	SmallAreaLowGrayLevelEmphasis	0.046579688
wavelet-HHH	ngtdm	Coarseness	0.029941522
wavelet-HHH	ngtdm	Complexity	216.3631055
wavelet-HHH	ngtdm	Strength	2.051811884
wavelet-HHH	ngtdm	Contrast	0.173328313
wavelet-HHH	ngtdm	Busyness	0.216204422
wavelet-HLL	gldm	GrayLevelVariance	101.1509297
wavelet-HLL	gldm	HighGrayLevelEmphasis	529.8380952
wavelet-HLL	gldm	DependenceEntropy	6.232598125
wavelet-HLL	gldm	DependenceNonUniformity	72.41904762
wavelet-HLL	gldm	GrayLevelNonUniformity	7.114285714
wavelet-HLL	gldm	SmallDependenceEmphasis	0.581797619
wavelet-HLL	gldm	SmallDependenceHighGrayLevelEmphasis	321.7134775
wavelet-HLL	gldm	DependenceNonUniformityNormalized	0.344852608
wavelet-HLL	gldm	LargeDependenceEmphasis	4.6
wavelet-HLL	gldm	LargeDependenceLowGrayLevelEmphasis	0.027126021
wavelet-HLL	gldm	DependenceVariance	1.151020408
wavelet-HLL	gldm	LargeDependenceHighGrayLevelEmphasis	2065.590476
wavelet-HLL	gldm	SmallDependenceLowGrayLevelEmphasis	0.009159129
wavelet-HLL	gldm	LowGrayLevelEmphasis	0.011051417
wavelet-HLL	glcm	JointAverage	20.02457273
wavelet-HLL	glcm	SumAverage	40.04914546
wavelet-HLL	glcm	JointEntropy	7.752147002
wavelet-HLL	glcm	ClusterShade	2730.253183
wavelet-HLL	glcm	MaximumProbability	0.013533228
wavelet-HLL	glcm	Idmn	0.943253915
wavelet-HLL	glcm	JointEnergy	0.005107239
wavelet-HLL	glcm	Contrast	130.4994852
wavelet-HLL	glcm	DifferenceEntropy	4.260793023
wavelet-HLL	glcm	InverseVariance	0.137398078
wavelet-HLL	glcm	DifferenceVariance	52.09388997
wavelet-HLL	glcm	Idn	0.85159763
wavelet-HLL	glcm	Idm	0.132734595
wavelet-HLL	glcm	Correlation	0.31884249
wavelet-HLL	glcm	Autocorrelation	433.250502
wavelet-HLL	glcm	SumEntropy	5.420184322
wavelet-HLL	glcm	SumSquares	97.4018759
wavelet-HLL	glcm	ClusterProminence	175296.7805
wavelet-HLL	glcm	Imc2	0.994722151
wavelet-HLL	glcm	Imc1	-0.45599131
wavelet-HLL	glcm	DifferenceAverage	8.470249981
wavelet-HLL	glcm	Id	0.217117232
wavelet-HLL	glcm	ClusterTendency	259.1080184
wavelet-HLL	firstorder	InterquartileRange	407.9334018
wavelet-HLL	firstorder	Skewness	0.383218444
wavelet-HLL	firstorder	Uniformity	0.033877551
wavelet-HLL	firstorder	Median	-117.2942973

wavelet-HLL	firstorder	Energy	14400079.24
wavelet-HLL	firstorder	RobustMeanAbsoluteDeviation	163.8501122
wavelet-HLL	firstorder	MeanAbsoluteDeviation	213.1401341
wavelet-HLL	firstorder	TotalEnergy	388802139.5
wavelet-HLL	firstorder	Maximum	496.9633659
wavelet-HLL	firstorder	RootMeanSquared	261.8621888
wavelet-HLL	firstorder	90Percentile	300.589066
wavelet-HLL	firstorder	Minimum	-571.8838035
wavelet-HLL	firstorder	Entropy	5.096459549
wavelet-HLL	firstorder	Range	1068.847169
wavelet-HLL	firstorder	Variance	63653.49075
wavelet-HLL	firstorder	10Percentile	-355.1593875
wavelet-HLL	firstorder	Kurtosis	2.081220501
wavelet-HLL	firstorder	Mean	-70.13070055
wavelet-HLL	glrlm	ShortRunLowGrayLevelEmphasis	0.011167442
wavelet-HLL	glrlm	GrayLevelVariance	101.907067
wavelet-HLL	glrlm	LowGrayLevelRunEmphasis	0.01127665
wavelet-HLL	glrlm	GrayLevelNonUniformityNormalized	0.033291426
wavelet-HLL	glrlm	RunVariance	0.036773672
wavelet-HLL	glrlm	GrayLevelNonUniformity	6.761711635
wavelet-HLL	glrlm	LongRunEmphasis	1.106931095
wavelet-HLL	glrlm	ShortRunHighGrayLevelEmphasis	520.9680217
wavelet-HLL	glrlm	RunLengthNonUniformity	190.342944
wavelet-HLL	glrlm	ShortRunEmphasis	0.975216732
wavelet-HLL	glrlm	LongRunHighGrayLevelEmphasis	579.7463353
wavelet-HLL	glrlm	RunPercentage	0.967032967
wavelet-HLL	glrlm	LongRunLowGrayLevelEmphasis	0.011765404
wavelet-HLL	glrlm	RunEntropy	5.240033068
wavelet-HLL	glrlm	HighGrayLevelRunEmphasis	532.4851115
wavelet-HLL	glrlm	RunLengthNonUniformityNormalized	0.936867264
wavelet-HLL	glszm	GrayLevelVariance	109.2482158
wavelet-HLL	glszm	ZoneVariance	1.733347751
wavelet-HLL	glszm	GrayLevelNonUniformityNormalized	0.029736159
wavelet-HLL	glszm	SizeZoneNonUniformityNormalized	0.594290657
wavelet-HLL	glszm	SizeZoneNonUniformity	80.82352941
wavelet-HLL	glszm	GrayLevelNonUniformity	4.044117647
wavelet-HLL	glszm	LargeAreaEmphasis	4.117647059
wavelet-HLL	glszm	SmallAreaHighGrayLevelEmphasis	442.8921474
wavelet-HLL	glszm	ZonePercentage	0.647619048
wavelet-HLL	glszm	LargeAreaLowGrayLevelEmphasis	0.029000675
wavelet-HLL	glszm	LargeAreaHighGrayLevelEmphasis	1899.044118
wavelet-HLL	glszm	HighGrayLevelZoneEmphasis	554.2720588
wavelet-HLL	glszm	SmallAreaEmphasis	0.797574312
wavelet-HLL	glszm	LowGrayLevelZoneEmphasis	0.014599775
wavelet-HLL	glszm	ZoneEntropy	5.848656504
wavelet-HLL	glszm	SmallAreaLowGrayLevelEmphasis	0.01368346
wavelet-HLL	ngtdm	Coarseness	0.027215117
wavelet-HLL	ngtdm	Complexity	3987.568804
wavelet-HLL	ngtdm	Strength	16.52016105
wavelet-HLL	ngtdm	Contrast	0.717407834
wavelet-HLL	ngtdm	Busyness	0.055029952
wavelet-LHL	gldm	GrayLevelVariance	55.21045351
wavelet-LHL	gldm	HighGrayLevelEmphasis	386.6238095

wavelet-LHL	gldm	DependenceEntropy	6.048051042
wavelet-LHL	gldm	DependenceNonUniformity	71.43809524
wavelet-LHL	gldm	GrayLevelNonUniformity	9.133333333
wavelet-LHL	gldm	SmallDependenceEmphasis	0.561888889
wavelet-LHL	gldm	SmallDependenceHighGrayLevelEmphasis	235.8572526
wavelet-LHL	gldm	DependenceNonUniformityNormalized	0.340181406
wavelet-LHL	gldm	LargeDependenceEmphasis	4.285714286
wavelet-LHL	gldm	LargeDependenceLowGrayLevelEmphasis	0.024892588
wavelet-LHL	gldm	DependenceVariance	0.907120181
wavelet-LHL	gldm	LargeDependenceHighGrayLevelEmphasis	1528.7
wavelet-LHL	gldm	SmallDependenceLowGrayLevelEmphasis	0.009387984
wavelet-LHL	gldm	LowGrayLevelEmphasis	0.011411906
wavelet-LHL	glcm	JointAverage	17.68770267
wavelet-LHL	glcm	SumAverage	35.37540534
wavelet-LHL	glcm	JointEntropy	7.603638718
wavelet-LHL	glcm	ClusterShade	627.0253853
wavelet-LHL	glcm	MaximumProbability	0.013541238
wavelet-LHL	glcm	Idmn	0.960278492
wavelet-LHL	glcm	JointEnergy	0.005744421
wavelet-LHL	glcm	Contrast	77.37291641
wavelet-LHL	glcm	DifferenceEntropy	4.007296899
wavelet-LHL	glcm	InverseVariance	0.153155158
wavelet-LHL	glcm	DifferenceVariance	30.53422879
wavelet-LHL	glcm	Idn	0.870214275
wavelet-LHL	glcm	Idm	0.142077211
wavelet-LHL	glcm	Correlation	0.159615898
wavelet-LHL	glcm	Autocorrelation	321.3211539
wavelet-LHL	glcm	SumEntropy	5.060228629
wavelet-LHL	glcm	SumSquares	47.12994726
wavelet-LHL	glcm	ClusterProminence	46902.53753
wavelet-LHL	glcm	Imc2	0.983095453
wavelet-LHL	glcm	Imc1	-0.366888457
wavelet-LHL	glcm	DifferenceAverage	6.710012168
wavelet-LHL	glcm	Id	0.233365686
wavelet-LHL	glcm	ClusterTendency	111.1468726
wavelet-LHL	firstorder	InterquartileRange	223.728592
wavelet-LHL	firstorder	Skewness	0.518884932
wavelet-LHL	firstorder	Uniformity	0.043492063
wavelet-LHL	firstorder	Median	-75.85099663
wavelet-LHL	firstorder	Energy	7927199.42
wavelet-LHL	firstorder	RobustMeanAbsoluteDeviation	97.99524903
wavelet-LHL	firstorder	MeanAbsoluteDeviation	145.0405234
wavelet-LHL	firstorder	TotalEnergy	214034384.3
wavelet-LHL	firstorder	Maximum	509.2613036
wavelet-LHL	firstorder	RootMeanSquared	194.2899088
wavelet-LHL	firstorder	90Percentile	210.1425056
wavelet-LHL	firstorder	Minimum	-479.7042011
wavelet-LHL	firstorder	Entropy	4.78432658
wavelet-LHL	firstorder	Range	988.9655047
wavelet-LHL	firstorder	Variance	34474.82132
wavelet-LHL	firstorder	10Percentile	-266.1637197
wavelet-LHL	firstorder	Kurtosis	3.21481848
wavelet-LHL	firstorder	Mean	-57.21667015

wavelet-LHL	glrlm	ShortRunLowGrayLevelEmphasis	0.011546211
wavelet-LHL	glrlm	GrayLevelVariance	56.09907423
wavelet-LHL	glrlm	LowGrayLevelRunEmphasis	0.011651463
wavelet-LHL	glrlm	GrayLevelNonUniformityNormalized	0.042964976
wavelet-LHL	glrlm	RunVariance	0.034420513
wavelet-LHL	glrlm	GrayLevelNonUniformity	8.731628233
wavelet-LHL	glrlm	LongRunEmphasis	1.103224393
wavelet-LHL	glrlm	ShortRunHighGrayLevelEmphasis	379.4100582
wavelet-LHL	glrlm	RunLengthNonUniformity	190.7176336
wavelet-LHL	glrlm	ShortRunEmphasis	0.975452981
wavelet-LHL	glrlm	LongRunHighGrayLevelEmphasis	423.5313097
wavelet-LHL	glrlm	RunPercentage	0.967765568
wavelet-LHL	glrlm	LongRunLowGrayLevelEmphasis	0.0120951
wavelet-LHL	glrlm	RunEntropy	4.934144974
wavelet-LHL	glrlm	HighGrayLevelRunEmphasis	387.9764939
wavelet-LHL	glrlm	RunLengthNonUniformityNormalized	0.937800713
wavelet-LHL	glszm	GrayLevelVariance	68.49689986
wavelet-LHL	glszm	ZoneVariance	1.491358025
wavelet-LHL	glszm	GrayLevelNonUniformityNormalized	0.037037037
wavelet-LHL	glszm	SizeZoneNonUniformityNormalized	0.546776406
wavelet-LHL	glszm	SizeZoneNonUniformity	73.81481481
wavelet-LHL	glszm	GrayLevelNonUniformity	5
wavelet-LHL	glszm	LargeAreaEmphasis	3.911111111
wavelet-LHL	glszm	SmallAreaHighGrayLevelEmphasis	331.6148583
wavelet-LHL	glszm	ZonePercentage	0.642857143
wavelet-LHL	glszm	LargeAreaLowGrayLevelEmphasis	0.027789438
wavelet-LHL	glszm	LargeAreaHighGrayLevelEmphasis	1363.962963
wavelet-LHL	glszm	HighGrayLevelZoneEmphasis	413.3555556
wavelet-LHL	glszm	SmallAreaEmphasis	0.767913403
wavelet-LHL	glszm	LowGrayLevelZoneEmphasis	0.015238309
wavelet-LHL	glszm	ZoneEntropy	5.793420182
wavelet-LHL	glszm	SmallAreaLowGrayLevelEmphasis	0.014084907
wavelet-LHL	ngtdm	Coarseness	0.029248906
wavelet-LHL	ngtdm	Complexity	2669.621513
wavelet-LHL	ngtdm	Strength	13.78814952
wavelet-LHL	ngtdm	Contrast	0.385304755
wavelet-LHL	ngtdm	Busyness	0.057963895
wavelet-LHH	gldm	GrayLevelVariance	21.01789116
wavelet-LHH	gldm	HighGrayLevelEmphasis	374.9952381
wavelet-LHH	gldm	DependenceEntropy	5.749804521
wavelet-LHH	gldm	DependenceNonUniformity	44.56190476
wavelet-LHH	gldm	GrayLevelNonUniformity	16.01904762
wavelet-LHH	gldm	SmallDependenceEmphasis	0.407089785
wavelet-LHH	gldm	SmallDependenceHighGrayLevelEmphasis	167.6598646
wavelet-LHH	gldm	DependenceNonUniformityNormalized	0.212199546
wavelet-LHH	gldm	LargeDependenceEmphasis	9.733333333
wavelet-LHH	gldm	LargeDependenceLowGrayLevelEmphasis	0.037590286
wavelet-LHH	gldm	DependenceVariance	2.723446712
wavelet-LHH	gldm	LargeDependenceHighGrayLevelEmphasis	3242.080952
wavelet-LHH	gldm	SmallDependenceLowGrayLevelEmphasis	0.007248079
wavelet-LHH	gldm	LowGrayLevelEmphasis	0.009149675
wavelet-LHH	glcm	JointAverage	18.77490796
wavelet-LHH	glcm	SumAverage	37.54981591

wavelet-LHH	glcm	JointEntropy	6.902689289
wavelet-LHH	glcm	ClusterShade	88.87984182
wavelet-LHH	glcm	MaximumProbability	0.03118518
wavelet-LHH	glcm	Idmn	0.969121405
wavelet-LHH	glcm	JointEnergy	0.010744009
wavelet-LHH	glcm	Contrast	38.62644193
wavelet-LHH	glcm	DifferenceEntropy	3.568471298
wavelet-LHH	glcm	InverseVariance	0.224190014
wavelet-LHH	glcm	DifferenceVariance	17.35701336
wavelet-LHH	glcm	Idn	0.888890757
wavelet-LHH	glcm	Idm	0.227051761
wavelet-LHH	glcm	Correlation	0.005134007
wavelet-LHH	glcm	Autocorrelation	352.7800507
wavelet-LHH	glcm	SumEntropy	4.383114189
wavelet-LHH	glcm	SumSquares	19.54396817
wavelet-LHH	glcm	ClusterProminence	7600.199171
wavelet-LHH	glcm	Imc2	0.92605727
wavelet-LHH	glcm	Imc1	-0.250089112
wavelet-LHH	glcm	DifferenceAverage	4.539192131
wavelet-LHH	glcm	Id	0.315735781
wavelet-LHH	glcm	ClusterTendency	39.54943076
wavelet-LHH	firstorder	InterquartileRange	130.2827432
wavelet-LHH	firstorder	Skewness	0.0779981
wavelet-LHH	firstorder	Uniformity	0.076281179
wavelet-LHH	firstorder	Median	-2.94823443
wavelet-LHH	firstorder	Energy	2756846.363
wavelet-LHH	firstorder	RobustMeanAbsoluteDeviation	55.8106921
wavelet-LHH	firstorder	MeanAbsoluteDeviation	85.7787895
wavelet-LHH	firstorder	TotalEnergy	74434851.81
wavelet-LHH	firstorder	Maximum	353.0052541
wavelet-LHH	firstorder	RootMeanSquared	114.5767857
wavelet-LHH	firstorder	90Percentile	140.1267214
wavelet-LHH	firstorder	Minimum	-444.8218981
wavelet-LHH	firstorder	Entropy	4.042669481
wavelet-LHH	firstorder	Range	797.8271522
wavelet-LHH	firstorder	Variance	13068.89174
wavelet-LHH	firstorder	10Percentile	-125.8626234
wavelet-LHH	firstorder	Kurtosis	4.798048468
wavelet-LHH	firstorder	Mean	7.677765808
wavelet-LHH	glrlm	ShortRunLowGrayLevelEmphasis	0.009393956
wavelet-LHH	glrlm	GrayLevelVariance	21.93486825
wavelet-LHH	glrlm	LowGrayLevelRunEmphasis	0.009552694
wavelet-LHH	glrlm	GrayLevelNonUniformityNormalized	0.073592438
wavelet-LHH	glrlm	RunVariance	0.069282867
wavelet-LHH	glrlm	GrayLevelNonUniformity	14.47639615
wavelet-LHH	glrlm	LongRunEmphasis	1.210772778
wavelet-LHH	glrlm	ShortRunHighGrayLevelEmphasis	361.0193954
wavelet-LHH	glrlm	RunLengthNonUniformity	173.0617664
wavelet-LHH	glrlm	ShortRunEmphasis	0.950809537
wavelet-LHH	glrlm	LongRunHighGrayLevelEmphasis	448.1975697
wavelet-LHH	glrlm	RunPercentage	0.936630037
wavelet-LHH	glrlm	LongRunLowGrayLevelEmphasis	0.010236381
wavelet-LHH	glrlm	RunEntropy	4.36631235

wavelet-LHH	glrlm	HighGrayLevelRunEmphasis	377.6438676
wavelet-LHH	glrlm	RunLengthNonUniformityNormalized	0.879033531
wavelet-LHH	glszm	GrayLevelVariance	34.62817576
wavelet-LHH	glszm	ZoneVariance	9.285714286
wavelet-LHH	glszm	GrayLevelNonUniformityNormalized	0.056226572
wavelet-LHH	glszm	SizeZoneNonUniformityNormalized	0.485422741
wavelet-LHH	glszm	SizeZoneNonUniformity	47.57142857
wavelet-LHH	glszm	GrayLevelNonUniformity	5.510204082
wavelet-LHH	glszm	LargeAreaEmphasis	13.87755102
wavelet-LHH	glszm	SmallAreaHighGrayLevelEmphasis	303.7198267
wavelet-LHH	glszm	ZonePercentage	0.466666667
wavelet-LHH	glszm	LargeAreaLowGrayLevelEmphasis	0.057731172
wavelet-LHH	glszm	LargeAreaHighGrayLevelEmphasis	4650.132653
wavelet-LHH	glszm	HighGrayLevelZoneEmphasis	406.1734694
wavelet-LHH	glszm	SmallAreaEmphasis	0.722258231
wavelet-LHH	glszm	LowGrayLevelZoneEmphasis	0.015959999
wavelet-LHH	glszm	ZoneEntropy	5.298447411
wavelet-LHH	glszm	SmallAreaLowGrayLevelEmphasis	0.01506427
wavelet-LHH	ngtdm	Coarseness	0.025591266
wavelet-LHH	ngtdm	Complexity	1012.951911
wavelet-LHH	ngtdm	Strength	6.550453936
wavelet-LHH	ngtdm	Contrast	0.232209547
wavelet-LHH	ngtdm	Busyness	0.077971956
wavelet-LLH	gldm	GrayLevelVariance	41.76419501
wavelet-LLH	gldm	HighGrayLevelEmphasis	361.8333333
wavelet-LLH	gldm	DependenceEntropy	6.014857562
wavelet-LLH	gldm	DependenceNonUniformity	56.02857143
wavelet-LLH	gldm	GrayLevelNonUniformity	11.35238095
wavelet-LLH	gldm	SmallDependenceEmphasis	0.493291545
wavelet-LLH	gldm	SmallDependenceHighGrayLevelEmphasis	208.3206359
wavelet-LLH	gldm	DependenceNonUniformityNormalized	0.266802721
wavelet-LLH	gldm	LargeDependenceEmphasis	6.971428571
wavelet-LLH	gldm	LargeDependenceLowGrayLevelEmphasis	0.032798892
wavelet-LLH	gldm	DependenceVariance	1.919637188
wavelet-LLH	gldm	LargeDependenceHighGrayLevelEmphasis	2337.957143
wavelet-LLH	gldm	SmallDependenceLowGrayLevelEmphasis	0.008812968
wavelet-LLH	gldm	LowGrayLevelEmphasis	0.011004147
wavelet-LLH	glcm	JointAverage	17.43995235
wavelet-LLH	glcm	SumAverage	34.8799047
wavelet-LLH	glcm	JointEntropy	7.325478253
wavelet-LLH	glcm	ClusterShade	392.7656109
wavelet-LLH	glcm	MaximumProbability	0.02547928
wavelet-LLH	glcm	Idmn	0.973617136
wavelet-LLH	glcm	JointEnergy	0.007590436
wavelet-LLH	glcm	Contrast	55.9967005
wavelet-LLH	glcm	DifferenceEntropy	3.756799721
wavelet-LLH	glcm	InverseVariance	0.19850255
wavelet-LLH	glcm	DifferenceVariance	25.64504045
wavelet-LLH	glcm	Idn	0.896905598
wavelet-LLH	glcm	Idm	0.190913331
wavelet-LLH	glcm	Correlation	0.195098123
wavelet-LLH	glcm	Autocorrelation	311.6692747
wavelet-LLH	glcm	SumEntropy	4.859641254

wavelet-LLH	glcm	SumSquares	35.48482603
wavelet-LLH	glcm	ClusterProminence	36817.00884
wavelet-LLH	glcm	Imc2	0.970767815
wavelet-LLH	glcm	Imc1	-0.329744873
wavelet-LLH	glcm	DifferenceAverage	5.410443365
wavelet-LLH	glcm	Id	0.28123382
wavelet-LLH	glcm	ClusterTendency	85.94260362
wavelet-LLH	firstorder	InterquartileRange	177.7772846
wavelet-LLH	firstorder	Skewness	0.535949971
wavelet-LLH	firstorder	Uniformity	0.054058957
wavelet-LLH	firstorder	Median	-43.45421306
wavelet-LLH	firstorder	Energy	5866003.753
wavelet-LLH	firstorder	RobustMeanAbsoluteDeviation	78.47164545
wavelet-LLH	firstorder	MeanAbsoluteDeviation	121.8657361
wavelet-LLH	firstorder	TotalEnergy	158382101.3
wavelet-LLH	firstorder	Maximum	592.4221682
wavelet-LLH	firstorder	RootMeanSquared	167.1327353
wavelet-LLH	firstorder	90Percentile	158.4690064
wavelet-LLH	firstorder	Minimum	-456.8788246
wavelet-LLH	firstorder	Entropy	4.528496158
wavelet-LLH	firstorder	Range	1049.300993
wavelet-LLH	firstorder	Variance	26328.47253
wavelet-LLH	firstorder	10Percentile	-230.2496445
wavelet-LLH	firstorder	Kurtosis	4.176507077
wavelet-LLH	firstorder	Mean	-40.06093703
wavelet-LLH	glrlm	ShortRunLowGrayLevelEmphasis	0.011225022
wavelet-LLH	glrlm	GrayLevelVariance	43.25161345
wavelet-LLH	glrlm	LowGrayLevelRunEmphasis	0.011364051
wavelet-LLH	glrlm	GrayLevelNonUniformityNormalized	0.052443036
wavelet-LLH	glrlm	RunVariance	0.061110278
wavelet-LLH	glrlm	GrayLevelNonUniformity	10.48716676
wavelet-LLH	glrlm	LongRunEmphasis	1.165658655
wavelet-LLH	glrlm	ShortRunHighGrayLevelEmphasis	353.1580056
wavelet-LLH	glrlm	RunLengthNonUniformity	182.7609467
wavelet-LLH	glrlm	ShortRunEmphasis	0.965364491
wavelet-LLH	glrlm	LongRunHighGrayLevelEmphasis	417.2723728
wavelet-LLH	glrlm	RunPercentage	0.952014652
wavelet-LLH	glrlm	LongRunLowGrayLevelEmphasis	0.012000102
wavelet-LLH	glrlm	RunEntropy	4.754643708
wavelet-LLH	glrlm	HighGrayLevelRunEmphasis	364.0468554
wavelet-LLH	glrlm	RunLengthNonUniformityNormalized	0.913615654
wavelet-LLH	glszm	GrayLevelVariance	64.00635161
wavelet-LLH	glszm	ZoneVariance	4.821928166
wavelet-LLH	glszm	GrayLevelNonUniformityNormalized	0.039848771
wavelet-LLH	glszm	SizeZoneNonUniformityNormalized	0.562192817
wavelet-LLH	glszm	SizeZoneNonUniformity	64.65217391
wavelet-LLH	glszm	GrayLevelNonUniformity	4.582608696
wavelet-LLH	glszm	LargeAreaEmphasis	8.156521739
wavelet-LLH	glszm	SmallAreaHighGrayLevelEmphasis	343.4932537
wavelet-LLH	glszm	ZonePercentage	0.547619048
wavelet-LLH	glszm	LargeAreaLowGrayLevelEmphasis	0.04125327
wavelet-LLH	glszm	LargeAreaHighGrayLevelEmphasis	2854.773913
wavelet-LLH	glszm	HighGrayLevelZoneEmphasis	406.7391304

wavelet-LLH	glszm	SmallAreaEmphasis	0.774015987
wavelet-LLH	glszm	LowGrayLevelZoneEmphasis	0.016633715
wavelet-LLH	glszm	ZoneEntropy	5.561673343
wavelet-LLH	glszm	SmallAreaLowGrayLevelEmphasis	0.015497166
wavelet-LLH	ngtdm	Coarseness	0.035340976
wavelet-LLH	ngtdm	Complexity	1731.914543
wavelet-LLH	ngtdm	Strength	13.74156756
wavelet-LLH	ngtdm	Contrast	0.283502097
wavelet-LLH	ngtdm	Busyness	0.048041904
wavelet-LLL	gldm	GrayLevelVariance	488.9808617
wavelet-LLL	gldm	HighGrayLevelEmphasis	2793.895238
wavelet-LLL	gldm	DependenceEntropy	6.45504119
wavelet-LLL	gldm	DependenceNonUniformity	124.3238095
wavelet-LLL	gldm	GrayLevelNonUniformity	3.695238095
wavelet-LLL	gldm	SmallDependenceEmphasis	0.797669312
wavelet-LLL	gldm	SmallDependenceHighGrayLevelEmphasis	1892.004614
wavelet-LLL	gldm	DependenceNonUniformityNormalized	0.592018141
wavelet-LLL	gldm	LargeDependenceEmphasis	2.619047619
wavelet-LLL	gldm	LargeDependenceLowGrayLevelEmphasis	0.007306611
wavelet-LLL	gldm	DependenceVariance	0.685623583
wavelet-LLL	gldm	LargeDependenceHighGrayLevelEmphasis	11213.57143
wavelet-LLL	gldm	SmallDependenceLowGrayLevelEmphasis	0.006664833
wavelet-LLL	gldm	LowGrayLevelEmphasis	0.006765905
wavelet-LLL	glcm	JointAverage	54.31830851
wavelet-LLL	glcm	SumAverage	108.636617
wavelet-LLL	glcm	JointEntropy	8.028899915
wavelet-LLL	glcm	ClusterShade	-16326.34315
wavelet-LLL	glcm	MaximumProbability	0.010080484
wavelet-LLL	glcm	Idmn	0.931011792
wavelet-LLL	glcm	JointEnergy	0.003996823
wavelet-LLL	glcm	Contrast	602.2980365
wavelet-LLL	glcm	DifferenceEntropy	5.223584344
wavelet-LLL	glcm	InverseVariance	0.069006272
wavelet-LLL	glcm	DifferenceVariance	204.5434621
wavelet-LLL	glcm	Idn	0.830395087
wavelet-LLL	glcm	Idm	0.063928995
wavelet-LLL	glcm	Correlation	0.327815601
wavelet-LLL	glcm	Autocorrelation	3100.556404
wavelet-LLL	glcm	SumEntropy	6.206737574
wavelet-LLL	glcm	SumSquares	449.7663662
wavelet-LLL	glcm	ClusterProminence	3302350.67
wavelet-LLL	glcm	Imc2	0.999718609
wavelet-LLL	glcm	Imc1	-0.637290561
wavelet-LLL	glcm	DifferenceAverage	19.37418193
wavelet-LLL	glcm	Id	0.124380414
wavelet-LLL	glcm	ClusterTendency	1196.767428
wavelet-LLL	firstorder	InterquartileRange	950.6804834
wavelet-LLL	firstorder	Skewness	-0.143802534
wavelet-LLL	firstorder	Uniformity	0.017596372
wavelet-LLL	firstorder	Median	-574.2372694
wavelet-LLL	firstorder	Energy	142682974.9
wavelet-LLL	firstorder	RobustMeanAbsoluteDeviation	383.9538246
wavelet-LLL	firstorder	MeanAbsoluteDeviation	480.5271885

wavelet-LLL	firstorder	TotalEnergy	3852440322
wavelet-LLL	firstorder	Maximum	322.7714677
wavelet-LLL	firstorder	RootMeanSquared	824.2831659
wavelet-LLL	firstorder	90Percentile	113.6960563
wavelet-LLL	firstorder	Minimum	-1784.691668
wavelet-LLL	firstorder	Entropy	6.03324344
wavelet-LLL	firstorder	Range	2107.463136
wavelet-LLL	firstorder	Variance	305609.175
wavelet-LLL	firstorder	10Percentile	-1363.04134
wavelet-LLL	firstorder	Kurtosis	1.886347267
wavelet-LLL	firstorder	Mean	-611.4193018
wavelet-LLL	glrlm	ShortRunLowGrayLevelEmphasis	0.006859206
wavelet-LLL	glrlm	GrayLevelVariance	486.0731769
wavelet-LLL	glrlm	LowGrayLevelRunEmphasis	0.006863704
wavelet-LLL	glrlm	GrayLevelNonUniformityNormalized	0.017443992
wavelet-LLL	glrlm	RunVariance	0.016514104
wavelet-LLL	glrlm	GrayLevelNonUniformity	3.608492337
wavelet-LLL	glrlm	LongRunEmphasis	1.047574605
wavelet-LLL	glrlm	ShortRunHighGrayLevelEmphasis	2707.161965
wavelet-LLL	glrlm	RunLengthNonUniformity	200.9678869
wavelet-LLL	glrlm	ShortRunEmphasis	0.988942287
wavelet-LLL	glrlm	LongRunHighGrayLevelEmphasis	2996.683985
wavelet-LLL	glrlm	RunPercentage	0.984981685
wavelet-LLL	glrlm	LongRunLowGrayLevelEmphasis	0.006882273
wavelet-LLL	glrlm	RunEntropy	6.079778039
wavelet-LLL	glrlm	HighGrayLevelRunEmphasis	2761.203666
wavelet-LLL	glrlm	RunLengthNonUniformityNormalized	0.971386031
wavelet-LLL	glszm	GrayLevelVariance	459.3798571
wavelet-LLL	glszm	ZoneVariance	0.366655223
wavelet-LLL	glszm	GrayLevelNonUniformityNormalized	0.017009457
wavelet-LLL	glszm	SizeZoneNonUniformityNormalized	0.779594894
wavelet-LLL	glszm	SizeZoneNonUniformity	139.547486
wavelet-LLL	glszm	GrayLevelNonUniformity	3.044692737
wavelet-LLL	glszm	LargeAreaEmphasis	1.74301676
wavelet-LLL	glszm	SmallAreaHighGrayLevelEmphasis	2089.833425
wavelet-LLL	glszm	ZonePercentage	0.852380952
wavelet-LLL	glszm	LargeAreaLowGrayLevelEmphasis	0.008138866
wavelet-LLL	glszm	LargeAreaHighGrayLevelEmphasis	6178.726257
wavelet-LLL	glszm	HighGrayLevelZoneEmphasis	2483.877095
wavelet-LLL	glszm	SmallAreaEmphasis	0.903939276
wavelet-LLL	glszm	LowGrayLevelZoneEmphasis	0.007856079
wavelet-LLL	glszm	ZoneEntropy	6.327820616
wavelet-LLL	glszm	SmallAreaLowGrayLevelEmphasis	0.007800588
wavelet-LLL	ngtdm	Coarseness	0.020587013
wavelet-LLL	ngtdm	Complexity	33932.04501
wavelet-LLL	ngtdm	Strength	58.55958541
wavelet-LLL	ngtdm	Contrast	2.219440205
wavelet-LLL	ngtdm	Busyness	0.014364137
wavelet-HHL	gldm	GrayLevelVariance	20.41188209
wavelet-HHL	gldm	HighGrayLevelEmphasis	230.8
wavelet-HHL	gldm	DependenceEntropy	5.773002055
wavelet-HHL	gldm	DependenceNonUniformity	54.62857143
wavelet-HHL	gldm	GrayLevelNonUniformity	14.18095238

wavelet-HHL	gldm	SmallDependenceEmphasis	0.426213584
wavelet-HHL	gldm	SmallDependenceHighGrayLevelEmphasis	110.5665547
wavelet-HHL	gldm	DependenceNonUniformityNormalized	0.260136054
wavelet-HHL	gldm	LargeDependenceEmphasis	7.095238095
wavelet-HHL	gldm	LargeDependenceLowGrayLevelEmphasis	0.049164484
wavelet-HHL	gldm	DependenceVariance	1.783310658
wavelet-HHL	gldm	LargeDependenceHighGrayLevelEmphasis	1430.2
wavelet-HHL	gldm	SmallDependenceLowGrayLevelEmphasis	0.008541659
wavelet-HHL	gldm	LowGrayLevelEmphasis	0.012173509
wavelet-HHL	glcm	JointAverage	14.63688261
wavelet-HHL	glcm	SumAverage	29.27376523
wavelet-HHL	glcm	JointEntropy	7.12682541
wavelet-HHL	glcm	ClusterShade	48.25657842
wavelet-HHL	glcm	MaximumProbability	0.023818998
wavelet-HHL	glcm	Idmn	0.956989212
wavelet-HHL	glcm	JointEnergy	0.008647817
wavelet-HHL	glcm	Contrast	39.28997058
wavelet-HHL	glcm	DifferenceEntropy	3.620251922
wavelet-HHL	glcm	InverseVariance	0.194110064
wavelet-HHL	glcm	DifferenceVariance	15.2649305
wavelet-HHL	glcm	Idn	0.864066132
wavelet-HHL	glcm	Idm	0.192866817
wavelet-HHL	glcm	Correlation	-0.005465002
wavelet-HHL	glcm	Autocorrelation	214.1510007
wavelet-HHL	glcm	SumEntropy	4.443206648
wavelet-HHL	glcm	SumSquares	19.45988582
wavelet-HHL	glcm	ClusterProminence	5395.268187
wavelet-HHL	glcm	Imc2	0.930666133
wavelet-HHL	glcm	Imc1	-0.249230262
wavelet-HHL	glcm	DifferenceAverage	4.833646613
wavelet-HHL	glcm	Id	0.286472363
wavelet-HHL	glcm	ClusterTendency	38.5495727
wavelet-HHL	firstorder	InterquartileRange	144.1884671
wavelet-HHL	firstorder	Skewness	0.274454621
wavelet-HHL	firstorder	Uniformity	0.067528345
wavelet-HHL	firstorder	Median	-8.693651397
wavelet-HHL	firstorder	Energy	2643807.73
wavelet-HHL	firstorder	RobustMeanAbsoluteDeviation	62.74315796
wavelet-HHL	firstorder	MeanAbsoluteDeviation	89.65184987
wavelet-HHL	firstorder	TotalEnergy	71382808.7
wavelet-HHL	firstorder	Maximum	345.5427407
wavelet-HHL	firstorder	RootMeanSquared	112.2032113
wavelet-HHL	firstorder	90Percentile	154.3463953
wavelet-HHL	firstorder	Minimum	-327.1175584
wavelet-HHL	firstorder	Entropy	4.127539612
wavelet-HHL	firstorder	Range	672.6602991
wavelet-HHL	firstorder	Variance	12589.33252
wavelet-HHL	firstorder	10Percentile	-130.3580811
wavelet-HHL	firstorder	Kurtosis	3.253362758
wavelet-HHL	firstorder	Mean	0.47759828
wavelet-HHL	glrlm	ShortRunLowGrayLevelEmphasis	0.012261329
wavelet-HHL	glrlm	GrayLevelVariance	20.88501569
wavelet-HHL	glrlm	LowGrayLevelRunEmphasis	0.012492525

wavelet-HHL	glrlm	GrayLevelNonUniformityNormalized	0.066412858
wavelet-HHL	glrlm	RunVariance	0.058541836
wavelet-HHL	glrlm	GrayLevelNonUniformity	13.24770805
wavelet-HHL	glrlm	LongRunEmphasis	1.167931492
wavelet-HHL	glrlm	ShortRunHighGrayLevelEmphasis	224.6504711
wavelet-HHL	glrlm	RunLengthNonUniformity	180.9349368
wavelet-HHL	glrlm	ShortRunEmphasis	0.96256198
wavelet-HHL	glrlm	LongRunHighGrayLevelEmphasis	266.0682648
wavelet-HHL	glrlm	RunPercentage	0.94981685
wavelet-HHL	glrlm	LongRunLowGrayLevelEmphasis	0.013527453
wavelet-HHL	glrlm	RunEntropy	4.364407349
wavelet-HHL	glrlm	HighGrayLevelRunEmphasis	232.3013934
wavelet-HHL	glrlm	RunLengthNonUniformityNormalized	0.906636571
wavelet-HHL	glszm	GrayLevelVariance	27.63691206
wavelet-HHL	glszm	ZoneVariance	5.357715148
wavelet-HHL	glszm	GrayLevelNonUniformityNormalized	0.057027053
wavelet-HHL	glszm	SizeZoneNonUniformityNormalized	0.441794703
wavelet-HHL	glszm	SizeZoneNonUniformity	45.50485437
wavelet-HHL	glszm	GrayLevelNonUniformity	5.873786408
wavelet-HHL	glszm	LargeAreaEmphasis	9.514563107
wavelet-HHL	glszm	SmallAreaHighGrayLevelEmphasis	187.1896001
wavelet-HHL	glszm	ZonePercentage	0.49047619
wavelet-HHL	glszm	LargeAreaLowGrayLevelEmphasis	0.070845515
wavelet-HHL	glszm	LargeAreaHighGrayLevelEmphasis	1854.524272
wavelet-HHL	glszm	HighGrayLevelZoneEmphasis	256.1456311
wavelet-HHL	glszm	SmallAreaEmphasis	0.69104945
wavelet-HHL	glszm	LowGrayLevelZoneEmphasis	0.018318992
wavelet-HHL	glszm	ZoneEntropy	5.403848741
wavelet-HHL	glszm	SmallAreaLowGrayLevelEmphasis	0.016282967
wavelet-HHL	ngtdm	Coarseness	0.025864202
wavelet-HHL	ngtdm	Complexity	889.4237706
wavelet-HHL	ngtdm	Strength	5.029964996
wavelet-HHL	ngtdm	Contrast	0.249725987
wavelet-HHL	ngtdm	Busyness	0.115858025
wavelet-HLH	gldm	GrayLevelVariance	12.06249433
wavelet-HLH	gldm	HighGrayLevelEmphasis	146.1809524
wavelet-HLH	gldm	DependenceEntropy	5.74052594
wavelet-HLH	gldm	DependenceNonUniformity	45.59047619
wavelet-HLH	gldm	GrayLevelNonUniformity	18.54285714
wavelet-HLH	gldm	SmallDependenceEmphasis	0.40413208
wavelet-HLH	gldm	SmallDependenceHighGrayLevelEmphasis	62.09230956
wavelet-HLH	gldm	DependenceNonUniformityNormalized	0.217097506
wavelet-HLH	gldm	LargeDependenceEmphasis	10.43809524
wavelet-HLH	gldm	LargeDependenceLowGrayLevelEmphasis	0.097111617
wavelet-HLH	gldm	DependenceVariance	3.225034014
wavelet-HLH	gldm	LargeDependenceHighGrayLevelEmphasis	1392.104762
wavelet-HLH	gldm	SmallDependenceLowGrayLevelEmphasis	0.010919062
wavelet-HLH	gldm	LowGrayLevelEmphasis	0.016236103
wavelet-HLH	glcm	JointAverage	11.63227131
wavelet-HLH	glcm	SumAverage	23.26454262
wavelet-HLH	glcm	JointEntropy	6.707513729
wavelet-HLH	glcm	ClusterShade	-9.832132464
wavelet-HLH	glcm	MaximumProbability	0.032621522

wavelet-HLH	glcm	Idmn	0.960616225
wavelet-HLH	glcm	JointEnergy	0.012125836
wavelet-HLH	glcm	Contrast	24.01896071
wavelet-HLH	glcm	DifferenceEntropy	3.310193492
wavelet-HLH	glcm	InverseVariance	0.257095443
wavelet-HLH	glcm	DifferenceVariance	9.675623103
wavelet-HLH	glcm	Idn	0.870837058
wavelet-HLH	glcm	Idm	0.246050332
wavelet-HLH	glcm	Correlation	-0.029214195
wavelet-HLH	glcm	Autocorrelation	134.9749845
wavelet-HLH	glcm	SumEntropy	4.091966911
wavelet-HLH	glcm	SumSquares	11.66581462
wavelet-HLH	glcm	ClusterProminence	1903.876901
wavelet-HLH	glcm	Imc2	0.872332844
wavelet-HLH	glcm	Imc1	-0.194207235
wavelet-HLH	glcm	DifferenceAverage	3.743336189
wavelet-HLH	glcm	Id	0.335237296
wavelet-HLH	glcm	ClusterTendency	22.64429777
wavelet-HLH	firstorder	InterquartileRange	107.1236522
wavelet-HLH	firstorder	Skewness	-0.035350773
wavelet-HLH	firstorder	Uniformity	0.08829932
wavelet-HLH	firstorder	Median	4.282480806
wavelet-HLH	firstorder	Energy	1602844.483
wavelet-HLH	firstorder	RobustMeanAbsoluteDeviation	45.08077923
wavelet-HLH	firstorder	MeanAbsoluteDeviation	67.49279193
wavelet-HLH	firstorder	TotalEnergy	43276801.04
wavelet-HLH	firstorder	Maximum	297.634035
wavelet-HLH	firstorder	RootMeanSquared	87.36471127
wavelet-HLH	firstorder	90Percentile	113.0608064
wavelet-HLH	firstorder	Minimum	-263.7094208
wavelet-HLH	firstorder	Entropy	3.761997558
wavelet-HLH	firstorder	Range	561.3434558
wavelet-HLH	firstorder	Variance	7628.555592
wavelet-HLH	firstorder	10Percentile	-105.7235689
wavelet-HLH	firstorder	Kurtosis	3.568419539
wavelet-HLH	firstorder	Mean	2.009274256
wavelet-HLH	glrlm	ShortRunLowGrayLevelEmphasis	0.016340647
wavelet-HLH	glrlm	GrayLevelVariance	12.58292152
wavelet-HLH	glrlm	LowGrayLevelRunEmphasis	0.016764853
wavelet-HLH	glrlm	GrayLevelNonUniformityNormalized	0.085678856
wavelet-HLH	glrlm	RunVariance	0.077236409
wavelet-HLH	glrlm	GrayLevelNonUniformity	16.82911461
wavelet-HLH	glrlm	LongRunEmphasis	1.222694147
wavelet-HLH	glrlm	ShortRunHighGrayLevelEmphasis	140.2711908
wavelet-HLH	glrlm	RunLengthNonUniformity	172.9415949
wavelet-HLH	glrlm	ShortRunEmphasis	0.951048831
wavelet-HLH	glrlm	LongRunHighGrayLevelEmphasis	176.9631576
wavelet-HLH	glrlm	RunPercentage	0.935164835
wavelet-HLH	glrlm	LongRunLowGrayLevelEmphasis	0.018696525
wavelet-HLH	glrlm	RunEntropy	4.084735606
wavelet-HLH	glrlm	HighGrayLevelRunEmphasis	146.937515
wavelet-HLH	glrlm	RunLengthNonUniformityNormalized	0.879786336
wavelet-HLH	glszm	GrayLevelVariance	18.8264

wavelet-HLH	glszm	ZoneVariance	7.69
wavelet-HLH	glszm	GrayLevelNonUniformityNormalized	0.0672
wavelet-HLH	glszm	SizeZoneNonUniformityNormalized	0.4468
wavelet-HLH	glszm	SizeZoneNonUniformity	44.68
wavelet-HLH	glszm	GrayLevelNonUniformity	6.72
wavelet-HLH	glszm	LargeAreaEmphasis	12.1
wavelet-HLH	glszm	SmallAreaHighGrayLevelEmphasis	107.6833024
wavelet-HLH	glszm	ZonePercentage	0.476190476
wavelet-HLH	glszm	LargeAreaLowGrayLevelEmphasis	0.114057209
wavelet-HLH	glszm	LargeAreaHighGrayLevelEmphasis	1697.72
wavelet-HLH	glszm	HighGrayLevelZoneEmphasis	152.46
wavelet-HLH	glszm	SmallAreaEmphasis	0.694520213
wavelet-HLH	glszm	LowGrayLevelZoneEmphasis	0.024397395
wavelet-HLH	glszm	ZoneEntropy	5.254177321
wavelet-HLH	glszm	SmallAreaLowGrayLevelEmphasis	0.021516693
wavelet-HLH	ngtdm	Coarseness	0.028705213
wavelet-HLH	ngtdm	Complexity	487.7063974
wavelet-HLH	ngtdm	Strength	3.689371639
wavelet-HLH	ngtdm	Contrast	0.161610089
wavelet-HLH	ngtdm	Busyness	0.136223454

Image type	Feature Class	Feature Name	5: RTSTRUCT: C99-DNU_segment_IGTV LEFT LOWER
general	info	BoundingBox	(0, 0, 1, 12, 13, 12)
general	info	EnabledImageTypes	{u'Original': {}, u'Wavelet': {}}
general	info	GeneralSettings	{'distances': [1], 'additionalInfo': True, 'force2D': False, 'interpolator': 'sitkBSpline', 'resampledPixelSpacing': [3.0], 'label': 1, 'normalizeScale': 1, 'normalize': False, 'force2Ddimension': 0, 'removeOutliers': None, 'minimumROISize': None, u'binWidth': 25.0, 'minimumROIDimensions': 1, u'correctMask': True, 'preCrop': False, u'symmetricalGLCM': True, 'resegmentRange': None, 'padDistance': 5}
general	info	ImageHash	e262a1c16809a94e3f8653bb6abeb9a454c0328c
general	info	ImageSpacing	(0.9765625000000002, 0.9765625000000002, 2.0)
general	info	MaskHash	d1fac83645e9cd07a48486b9c19078da4fdb891d
general	info	NumpyVersion	1.13.1
general	info	PyWaveletVersion	0.5.2
general	info	SimpleITKVersion	1.1.0
general	info	Version	1.3.0.post102+g1b0eb74
general	info	VolumeNum	1
general	info	VoxelNum	745
original	shape	Maximum3DDiameter	42.10700654
original	shape	Maximum2DDiameterSlice	40.24922359
original	shape	Sphericity	0.765569656
original	shape	MinorAxis	31.27071515
original	shape	Elongation	0.875084812
original	shape	SurfaceVolumeRatio	0.232269794
original	shape	Volume	20115
original	shape	MajorAxis	35.73449649
original	shape	SurfaceArea	4672.106901
original	shape	Flatness	0.752748719
original	shape	LeastAxis	26.89909646
original	shape	Maximum2DDiameterColumn	40.80441153
original	shape	Maximum2DDiameterRow	36.61966685
original	gldm	GrayLevelVariance	120.305997
original	gldm	HighGrayLevelEmphasis	771.5489933
original	gldm	DependenceEntropy	6.62428044
original	gldm	DependenceNonUniformity	286.3557047
original	gldm	GrayLevelNonUniformity	19.92080537
original	gldm	SmallDependenceEmphasis	0.618056301
original	gldm	SmallDependenceHighGrayLevelEmphasis	404.0507878
original	gldm	DependenceNonUniformityNormalized	0.384370073
original	gldm	LargeDependenceEmphasis	3.773154362
original	gldm	LargeDependenceLowGrayLevelEmphasis	0.026261042
original	gldm	DependenceVariance	0.844232242
original	gldm	LargeDependenceHighGrayLevelEmphasis	3816.491275
original	gldm	SmallDependenceLowGrayLevelEmphasis	0.004529207
original	gldm	LowGrayLevelEmphasis	0.008138322
original	glcm	JointAverage	27.18301999
original	glcm	SumAverage	54.36603999
original	glcm	JointEntropy	9.382295584
original	glcm	ClusterShade	-906.268337
original	glcm	MaximumProbability	0.005338594
original	glcm	Idmn	0.936230491
original	glcm	JointEnergy	0.001729776
original	glcm	Contrast	157.8932734
original	glcm	DifferenceEntropy	4.660728349

original	glcm	InverseVariance	0.109057118
original	glcm	DifferenceVariance	60.84866903
original	glcm	Idn	0.836514802
original	glcm	Idm	0.103720465
original	glcm	Correlation	0.330550795
original	glcm	Autocorrelation	777.7819808
original	glcm	SumEntropy	6.01007142
original	glcm	SumSquares	117.7551456
original	glcm	ClusterProminence	222533.9164
original	glcm	Imc2	0.952399773
original	glcm	Imc1	-0.22503475
original	glcm	DifferenceAverage	9.800649564
original	glcm	Id	0.185091122
original	glcm	ClusterTendency	313.1273092
original	firstorder	InterquartileRange	464
original	firstorder	Skewness	-0.049681532
original	firstorder	Uniformity	0.026739336
original	firstorder	Median	-432
original	firstorder	Energy	190906958
original	firstorder	RobustMeanAbsoluteDeviation	181.4338987
original	firstorder	MeanAbsoluteDeviation	233.8633575
original	firstorder	TotalEnergy	5154487866
original	firstorder	Maximum	70
original	firstorder	RootMeanSquared	506.212357
original	firstorder	90Percentile	-50.4
original	firstorder	Minimum	-1043
original	firstorder	Entropy	5.32141217
original	firstorder	Range	1113
original	firstorder	Variance	75127.01566
original	firstorder	10Percentile	-784.6
original	firstorder	Kurtosis	1.999167248
original	firstorder	Mean	-425.5865772
original	glrlm	ShortRunLowGrayLevelEmphasis	0.00798757
original	glrlm	GrayLevelVariance	119.6852796
original	glrlm	LowGrayLevelRunEmphasis	0.00815002
original	glrlm	GrayLevelNonUniformityNormalized	0.02673168
original	glrlm	RunVariance	0.029254053
original	glrlm	GrayLevelNonUniformity	19.3703148
original	glrlm	LongRunEmphasis	1.086455026
original	glrlm	ShortRunHighGrayLevelEmphasis	742.7241736
original	glrlm	RunLengthNonUniformity	686.3242374
original	glrlm	ShortRunEmphasis	0.979451687
original	glrlm	LongRunHighGrayLevelEmphasis	854.201649
original	glrlm	RunPercentage	0.9726381
original	glrlm	LongRunLowGrayLevelEmphasis	0.008805449
original	glrlm	RunEntropy	5.457638476
original	glrlm	HighGrayLevelRunEmphasis	763.9915759
original	glrlm	RunLengthNonUniformityNormalized	0.947073219
original	glszm	GrayLevelVariance	111.4519919
original	glszm	ZoneVariance	1.058935778
original	glszm	GrayLevelNonUniformityNormalized	0.027023214
original	glszm	SizeZoneNonUniformityNormalized	0.596541444
original	glszm	SizeZoneNonUniformity	309.6050096
original	glszm	GrayLevelNonUniformity	14.02504817
original	glszm	LargeAreaEmphasis	3.119460501
original	glszm	SmallAreaHighGrayLevelEmphasis	497.5537075
original	glszm	ZonePercentage	0.696644295
original	glszm	LargeAreaLowGrayLevelEmphasis	0.020122026

original	glszm	LargeAreaHighGrayLevelEmphasis	3139.456647
original	glszm	HighGrayLevelZoneEmphasis	667.9210019
original	glszm	SmallAreaEmphasis	0.800635389
original	glszm	LowGrayLevelZoneEmphasis	0.008095397
original	glszm	ZoneEntropy	6.225768635
original	glszm	SmallAreaLowGrayLevelEmphasis	0.005698034
original	ngtdm	Coarseness	0.00791479
original	ngtdm	Complexity	5086.222526
original	ngtdm	Strength	5.134527626
original	ngtdm	Contrast	0.844366919
original	ngtdm	Busyness	0.144891019
wavelet-HHH	gldm	GrayLevelVariance	21.46005315
wavelet-HHH	gldm	HighGrayLevelEmphasis	328.6268456
wavelet-HHH	gldm	DependenceEntropy	6.176262853
wavelet-HHH	gldm	DependenceNonUniformity	185.6201342
wavelet-HHH	gldm	GrayLevelNonUniformity	48.23221477
wavelet-HHH	gldm	SmallDependenceEmphasis	0.430983549
wavelet-HHH	gldm	SmallDependenceHighGrayLevelEmphasis	147.1140417
wavelet-HHH	gldm	DependenceNonUniformityNormalized	0.249154543
wavelet-HHH	gldm	LargeDependenceEmphasis	7.252348993
wavelet-HHH	gldm	LargeDependenceLowGrayLevelEmphasis	0.03162078
wavelet-HHH	gldm	DependenceVariance	1.778633395
wavelet-HHH	gldm	LargeDependenceHighGrayLevelEmphasis	2251.120805
wavelet-HHH	gldm	SmallDependenceLowGrayLevelEmphasis	0.006063817
wavelet-HHH	gldm	LowGrayLevelEmphasis	0.008278681
wavelet-HHH	glcm	JointAverage	17.53903098
wavelet-HHH	glcm	SumAverage	35.07806197
wavelet-HHH	glcm	JointEntropy	8.005322174
wavelet-HHH	glcm	ClusterShade	-6.776899346
wavelet-HHH	glcm	MaximumProbability	0.014586534
wavelet-HHH	glcm	Idmn	0.960606599
wavelet-HHH	glcm	JointEnergy	0.005109108
wavelet-HHH	glcm	Contrast	46.39818081
wavelet-HHH	glcm	DifferenceEntropy	3.813186836
wavelet-HHH	glcm	InverseVariance	0.188737491
wavelet-HHH	glcm	DifferenceVariance	18.18548852
wavelet-HHH	glcm	Idn	0.869447583
wavelet-HHH	glcm	Idm	0.181730494
wavelet-HHH	glcm	Correlation	-0.039238163
wavelet-HHH	glcm	Autocorrelation	306.7764567
wavelet-HHH	glcm	SumEntropy	4.646239598
wavelet-HHH	glcm	SumSquares	22.35722963
wavelet-HHH	glcm	ClusterProminence	6967.859251
wavelet-HHH	glcm	Imc2	0.782780887
wavelet-HHH	glcm	Imc1	-0.112813263
wavelet-HHH	glcm	DifferenceAverage	5.25331262
wavelet-HHH	glcm	Id	0.273579268
wavelet-HHH	glcm	ClusterTendency	43.0307377
wavelet-HHH	firstorder	InterquartileRange	142.7021341
wavelet-HHH	firstorder	Skewness	-0.058032497
wavelet-HHH	firstorder	Uniformity	0.064741228
wavelet-HHH	firstorder	Median	2.546534637
wavelet-HHH	firstorder	Energy	9976823.411
wavelet-HHH	firstorder	RobustMeanAbsoluteDeviation	59.6781191
wavelet-HHH	firstorder	MeanAbsoluteDeviation	89.20642211
wavelet-HHH	firstorder	TotalEnergy	269374232.1
wavelet-HHH	firstorder	Maximum	367.7859074
wavelet-HHH	firstorder	RootMeanSquared	115.722553

wavelet-HHH	firstorder	90Percentile	150.6711451
wavelet-HHH	firstorder	Minimum	-424.3727511
wavelet-HHH	firstorder	Entropy	4.210585555
wavelet-HHH	firstorder	Range	792.1586584
wavelet-HHH	firstorder	Variance	13391.44655
wavelet-HHH	firstorder	10Percentile	-139.9950438
wavelet-HHH	firstorder	Kurtosis	3.614682051
wavelet-HHH	firstorder	Mean	0.512566773
wavelet-HHH	glrlm	ShortRunLowGrayLevelEmphasis	0.008372595
wavelet-HHH	glrlm	GrayLevelVariance	22.04936441
wavelet-HHH	glrlm	LowGrayLevelRunEmphasis	0.008522899
wavelet-HHH	glrlm	GrayLevelNonUniformityNormalized	0.063554775
wavelet-HHH	glrlm	RunVariance	0.056158405
wavelet-HHH	glrlm	GrayLevelNonUniformity	44.90911808
wavelet-HHH	glrlm	LongRunEmphasis	1.168088251
wavelet-HHH	glrlm	ShortRunHighGrayLevelEmphasis	317.1907358
wavelet-HHH	glrlm	RunLengthNonUniformity	636.5143171
wavelet-HHH	glrlm	ShortRunEmphasis	0.96041272
wavelet-HHH	glrlm	LongRunHighGrayLevelEmphasis	381.8139534
wavelet-HHH	glrlm	RunPercentage	0.948477026
wavelet-HHH	glrlm	LongRunLowGrayLevelEmphasis	0.009159626
wavelet-HHH	glrlm	RunEntropy	4.499694207
wavelet-HHH	glrlm	HighGrayLevelRunEmphasis	329.5524913
wavelet-HHH	glrlm	RunLengthNonUniformityNormalized	0.900612645
wavelet-HHH	glszm	GrayLevelVariance	30.78167757
wavelet-HHH	glszm	ZoneVariance	6.538935525
wavelet-HHH	glszm	GrayLevelNonUniformityNormalized	0.051696154
wavelet-HHH	glszm	SizeZoneNonUniformityNormalized	0.462738963
wavelet-HHH	glszm	SizeZoneNonUniformity	170.7506775
wavelet-HHH	glszm	GrayLevelNonUniformity	19.07588076
wavelet-HHH	glszm	LargeAreaEmphasis	10.61517615
wavelet-HHH	glszm	SmallAreaHighGrayLevelEmphasis	243.772913
wavelet-HHH	glszm	ZonePercentage	0.495302013
wavelet-HHH	glszm	LargeAreaLowGrayLevelEmphasis	0.047060365
wavelet-HHH	glszm	LargeAreaHighGrayLevelEmphasis	3353.840108
wavelet-HHH	glszm	HighGrayLevelZoneEmphasis	340.598916
wavelet-HHH	glszm	SmallAreaEmphasis	0.706987027
wavelet-HHH	glszm	LowGrayLevelZoneEmphasis	0.012812732
wavelet-HHH	glszm	ZoneEntropy	5.858257705
wavelet-HHH	glszm	SmallAreaLowGrayLevelEmphasis	0.011602655
wavelet-HHH	ngtdm	Coarseness	0.008297858
wavelet-HHH	ngtdm	Complexity	1225.548941
wavelet-HHH	ngtdm	Strength	1.956564478
wavelet-HHH	ngtdm	Contrast	0.199370337
wavelet-HHH	ngtdm	Busyness	0.225345635
wavelet-HLL	gldm	GrayLevelVariance	72.48339444
wavelet-HLL	gldm	HighGrayLevelEmphasis	1697.601342
wavelet-HLL	gldm	DependenceEntropy	6.534201556
wavelet-HLL	gldm	DependenceNonUniformity	259.1798658
wavelet-HLL	gldm	GrayLevelNonUniformity	26.87651007
wavelet-HLL	gldm	SmallDependenceEmphasis	0.557216804
wavelet-HLL	gldm	SmallDependenceHighGrayLevelEmphasis	962.7883001
wavelet-HLL	gldm	DependenceNonUniformityNormalized	0.347892437
wavelet-HLL	gldm	LargeDependenceEmphasis	4.175838926
wavelet-HLL	gldm	LargeDependenceLowGrayLevelEmphasis	0.004301696
wavelet-HLL	gldm	DependenceVariance	0.867834782
wavelet-HLL	gldm	LargeDependenceHighGrayLevelEmphasis	6896.855034
wavelet-HLL	gldm	SmallDependenceLowGrayLevelEmphasis	0.00174944

wavelet-HLL	gldm	LowGrayLevelEmphasis	0.002063295
wavelet-HLL	glcm	JointAverage	40.13034395
wavelet-HLL	glcm	SumAverage	80.2606879
wavelet-HLL	glcm	JointEntropy	9.047325957
wavelet-HLL	glcm	ClusterShade	371.156729
wavelet-HLL	glcm	MaximumProbability	0.007064277
wavelet-HLL	glcm	Idmn	0.976292482
wavelet-HLL	glcm	JointEnergy	0.002272763
wavelet-HLL	glcm	Contrast	135.2342717
wavelet-HLL	glcm	DifferenceEntropy	4.478871136
wavelet-HLL	glcm	InverseVariance	0.121763525
wavelet-HLL	glcm	DifferenceVariance	53.23226252
wavelet-HLL	glcm	Idn	0.897732596
wavelet-HLL	glcm	Idm	0.116084604
wavelet-HLL	glcm	Correlation	0.113920805
wavelet-HLL	glcm	Autocorrelation	1619.066415
wavelet-HLL	glcm	SumEntropy	5.583281415
wavelet-HLL	glcm	SumSquares	76.23103519
wavelet-HLL	glcm	ClusterProminence	117657.0677
wavelet-HLL	glcm	Imc2	0.938163972
wavelet-HLL	glcm	Imc1	-0.210629567
wavelet-HLL	glcm	DifferenceAverage	8.832180273
wavelet-HLL	glcm	Id	0.200223333
wavelet-HLL	glcm	ClusterTendency	169.6898691
wavelet-HLL	firstorder	InterquartileRange	288.3268519
wavelet-HLL	firstorder	Skewness	0.002652261
wavelet-HLL	firstorder	Uniformity	0.036075852
wavelet-HLL	firstorder	Median	-31.06271404
wavelet-HLL	firstorder	Energy	34254515.28
wavelet-HLL	firstorder	RobustMeanAbsoluteDeviation	116.3900694
wavelet-HLL	firstorder	MeanAbsoluteDeviation	166.4539238
wavelet-HLL	firstorder	TotalEnergy	924871912.6
wavelet-HLL	firstorder	Maximum	752.2210277
wavelet-HLL	firstorder	RootMeanSquared	214.4276455
wavelet-HLL	firstorder	90Percentile	209.7020122
wavelet-HLL	firstorder	Minimum	-1019.189535
wavelet-HLL	firstorder	Entropy	5.026101422
wavelet-HLL	firstorder	Range	1771.410563
wavelet-HLL	firstorder	Variance	45101.68684
wavelet-HLL	firstorder	10Percentile	-277.3604278
wavelet-HLL	firstorder	Kurtosis	4.118113274
wavelet-HLL	firstorder	Mean	-29.62310419
wavelet-HLL	glrlm	ShortRunLowGrayLevelEmphasis	0.002090674
wavelet-HLL	glrlm	GrayLevelVariance	73.2917411
wavelet-HLL	glrlm	LowGrayLevelRunEmphasis	0.002107664
wavelet-HLL	glrlm	GrayLevelNonUniformityNormalized	0.03586604
wavelet-HLL	glrlm	RunVariance	0.033577899
wavelet-HLL	glrlm	GrayLevelNonUniformity	25.87895381
wavelet-HLL	glrlm	LongRunEmphasis	1.100384414
wavelet-HLL	glrlm	ShortRunHighGrayLevelEmphasis	1659.860388
wavelet-HLL	glrlm	RunLengthNonUniformity	677.8522716
wavelet-HLL	glrlm	ShortRunEmphasis	0.976095792
wavelet-HLL	glrlm	LongRunHighGrayLevelEmphasis	1864.566449
wavelet-HLL	glrlm	RunPercentage	0.968508002
wavelet-HLL	glrlm	LongRunLowGrayLevelEmphasis	0.00217859
wavelet-HLL	glrlm	RunEntropy	5.199779488
wavelet-HLL	glrlm	HighGrayLevelRunEmphasis	1699.249845
wavelet-HLL	glrlm	RunLengthNonUniformityNormalized	0.939039845

wavelet-HLL	glszm	GrayLevelVariance	84.52221074
wavelet-HLL	glszm	ZoneVariance	1.145153166
wavelet-HLL	glszm	GrayLevelNonUniformityNormalized	0.033459122
wavelet-HLL	glszm	SizeZoneNonUniformityNormalized	0.519909842
wavelet-HLL	glszm	SizeZoneNonUniformity	251.6363636
wavelet-HLL	glszm	GrayLevelNonUniformity	16.19421488
wavelet-HLL	glszm	LargeAreaEmphasis	3.51446281
wavelet-HLL	glszm	SmallAreaHighGrayLevelEmphasis	1300.539441
wavelet-HLL	glszm	ZonePercentage	0.64966443
wavelet-HLL	glszm	LargeAreaLowGrayLevelEmphasis	0.004535753
wavelet-HLL	glszm	LargeAreaHighGrayLevelEmphasis	5859.927686
wavelet-HLL	glszm	HighGrayLevelZoneEmphasis	1719.254132
wavelet-HLL	glszm	SmallAreaEmphasis	0.749254478
wavelet-HLL	glszm	LowGrayLevelZoneEmphasis	0.002796514
wavelet-HLL	glszm	ZoneEntropy	6.322038116
wavelet-HLL	glszm	SmallAreaLowGrayLevelEmphasis	0.002617333
wavelet-HLL	ngtdm	Coarseness	0.008000476
wavelet-HLL	ngtdm	Complexity	7146.057585
wavelet-HLL	ngtdm	Strength	8.306243699
wavelet-HLL	ngtdm	Contrast	0.317320476
wavelet-HLL	ngtdm	Busyness	0.054764814
wavelet-LHL	gldm	GrayLevelVariance	100.7884257
wavelet-LHL	gldm	HighGrayLevelEmphasis	1207.4
wavelet-LHL	gldm	DependenceEntropy	6.605612021
wavelet-LHL	gldm	DependenceNonUniformity	297.5637584
wavelet-LHL	gldm	GrayLevelNonUniformity	22.27516779
wavelet-LHL	gldm	SmallDependenceEmphasis	0.617944072
wavelet-LHL	gldm	SmallDependenceHighGrayLevelEmphasis	739.2250455
wavelet-LHL	gldm	DependenceNonUniformityNormalized	0.399414441
wavelet-LHL	gldm	LargeDependenceEmphasis	3.416107383
wavelet-LHL	gldm	LargeDependenceLowGrayLevelEmphasis	0.005571815
wavelet-LHL	gldm	DependenceVariance	0.668078015
wavelet-LHL	gldm	LargeDependenceHighGrayLevelEmphasis	4112.524832
wavelet-LHL	gldm	SmallDependenceLowGrayLevelEmphasis	0.002518679
wavelet-LHL	gldm	LowGrayLevelEmphasis	0.002941867
wavelet-LHL	glcm	JointAverage	33.1198587
wavelet-LHL	glcm	SumAverage	66.23971741
wavelet-LHL	glcm	JointEntropy	9.349376518
wavelet-LHL	glcm	ClusterShade	37.72557853
wavelet-LHL	glcm	MaximumProbability	0.006123646
wavelet-LHL	glcm	Idmn	0.964046897
wavelet-LHL	glcm	JointEnergy	0.001810591
wavelet-LHL	glcm	Contrast	196.7434303
wavelet-LHL	glcm	DifferenceEntropy	4.726693321
wavelet-LHL	glcm	InverseVariance	0.099945429
wavelet-LHL	glcm	DifferenceVariance	76.04678977
wavelet-LHL	glcm	Idn	0.875819294
wavelet-LHL	glcm	Idm	0.095789682
wavelet-LHL	glcm	Correlation	0.073100242
wavelet-LHL	glcm	Autocorrelation	1104.884773
wavelet-LHL	glcm	SumEntropy	5.786835442
wavelet-LHL	glcm	SumSquares	106.3270466
wavelet-LHL	glcm	ClusterProminence	195260.6605
wavelet-LHL	glcm	Imc2	0.963716618
wavelet-LHL	glcm	Imc1	-0.248781845
wavelet-LHL	glcm	DifferenceAverage	10.69000697
wavelet-LHL	glcm	Id	0.175689908
wavelet-LHL	glcm	ClusterTendency	228.5647559

wavelet-LHL	firstorder	InterquartileRange	325.8462207
wavelet-LHL	firstorder	Skewness	-0.147711465
wavelet-LHL	firstorder	Uniformity	0.029899554
wavelet-LHL	firstorder	Median	-29.11142277
wavelet-LHL	firstorder	Energy	47623506.21
wavelet-LHL	firstorder	RobustMeanAbsoluteDeviation	138.1882874
wavelet-LHL	firstorder	MeanAbsoluteDeviation	199.1048684
wavelet-LHL	firstorder	TotalEnergy	1285834668
wavelet-LHL	firstorder	Maximum	873.7564843
wavelet-LHL	firstorder	RootMeanSquared	252.832295
wavelet-LHL	firstorder	90Percentile	274.232678
wavelet-LHL	firstorder	Minimum	-837.5207163
wavelet-LHL	firstorder	Entropy	5.304394582
wavelet-LHL	firstorder	Range	1711.277201
wavelet-LHL	firstorder	Variance	62983.3634
wavelet-LHL	firstorder	10Percentile	-342.5260755
wavelet-LHL	firstorder	Kurtosis	3.178136377
wavelet-LHL	firstorder	Mean	-30.67256112
wavelet-LHL	glrlm	ShortRunLowGrayLevelEmphasis	0.002968848
wavelet-LHL	glrlm	GrayLevelVariance	101.7672747
wavelet-LHL	glrlm	LowGrayLevelRunEmphasis	0.002989914
wavelet-LHL	glrlm	GrayLevelNonUniformityNormalized	0.029696752
wavelet-LHL	glrlm	RunVariance	0.026764506
wavelet-LHL	glrlm	GrayLevelNonUniformity	21.56404752
wavelet-LHL	glrlm	LongRunEmphasis	1.079763679
wavelet-LHL	glrlm	ShortRunHighGrayLevelEmphasis	1184.117042
wavelet-LHL	glrlm	RunLengthNonUniformity	690.618112
wavelet-LHL	glrlm	ShortRunEmphasis	0.980890277
wavelet-LHL	glrlm	LongRunHighGrayLevelEmphasis	1303.837722
wavelet-LHL	glrlm	RunPercentage	0.974703149
wavelet-LHL	glrlm	LongRunLowGrayLevelEmphasis	0.003077539
wavelet-LHL	glrlm	RunEntropy	5.44502853
wavelet-LHL	glrlm	HighGrayLevelRunEmphasis	1207.29415
wavelet-LHL	glrlm	RunLengthNonUniformityNormalized	0.950818908
wavelet-LHL	glszm	GrayLevelVariance	115.6498323
wavelet-LHL	glszm	ZoneVariance	0.779124319
wavelet-LHL	glszm	GrayLevelNonUniformityNormalized	0.027237635
wavelet-LHL	glszm	SizeZoneNonUniformityNormalized	0.573804739
wavelet-LHL	glszm	SizeZoneNonUniformity	301.8212928
wavelet-LHL	glszm	GrayLevelNonUniformity	14.3269962
wavelet-LHL	glszm	LargeAreaEmphasis	2.785171103
wavelet-LHL	glszm	SmallAreaHighGrayLevelEmphasis	936.5555646
wavelet-LHL	glszm	ZonePercentage	0.706040268
wavelet-LHL	glszm	LargeAreaLowGrayLevelEmphasis	0.005598562
wavelet-LHL	glszm	LargeAreaHighGrayLevelEmphasis	3350.511407
wavelet-LHL	glszm	HighGrayLevelZoneEmphasis	1201.515209
wavelet-LHL	glszm	SmallAreaEmphasis	0.786125604
wavelet-LHL	glszm	LowGrayLevelZoneEmphasis	0.003712518
wavelet-LHL	glszm	ZoneEntropy	6.38430863
wavelet-LHL	glszm	SmallAreaLowGrayLevelEmphasis	0.003471013
wavelet-LHL	ngtdm	Coarseness	0.007961013
wavelet-LHL	ngtdm	Complexity	9981.632664
wavelet-LHL	ngtdm	Strength	8.376949526
wavelet-LHL	ngtdm	Contrast	0.462503212
wavelet-LHL	ngtdm	Busyness	0.063422679
wavelet-LHH	gldm	GrayLevelVariance	44.72572587
wavelet-LHH	gldm	HighGrayLevelEmphasis	601.052349
wavelet-LHH	gldm	DependenceEntropy	6.291040022

wavelet-LHH	gldm	DependenceNonUniformity	244.2697987
wavelet-LHH	gldm	GrayLevelNonUniformity	33.41610738
wavelet-LHH	gldm	SmallDependenceEmphasis	0.523488441
wavelet-LHH	gldm	SmallDependenceHighGrayLevelEmphasis	317.8847983
wavelet-LHH	gldm	DependenceNonUniformityNormalized	0.327878924
wavelet-LHH	gldm	LargeDependenceEmphasis	4.49261745
wavelet-LHH	gldm	LargeDependenceLowGrayLevelEmphasis	0.01134281
wavelet-LHH	gldm	DependenceVariance	0.895357867
wavelet-LHH	gldm	LargeDependenceHighGrayLevelEmphasis	2680.512752
wavelet-LHH	gldm	SmallDependenceLowGrayLevelEmphasis	0.003026452
wavelet-LHH	gldm	LowGrayLevelEmphasis	0.00409595
wavelet-LHH	glcm	JointAverage	23.58953777
wavelet-LHH	glcm	SumAverage	47.17907555
wavelet-LHH	glcm	JointEntropy	8.805723933
wavelet-LHH	glcm	ClusterShade	-92.21116376
wavelet-LHH	glcm	MaximumProbability	0.009064502
wavelet-LHH	glcm	Idmn	0.955434826
wavelet-LHH	glcm	JointEnergy	0.00279364
wavelet-LHH	glcm	Contrast	100.2256539
wavelet-LHH	glcm	DifferenceEntropy	4.317766978
wavelet-LHH	glcm	InverseVariance	0.124876775
wavelet-LHH	glcm	DifferenceVariance	37.35267879
wavelet-LHH	glcm	Idn	0.860697226
wavelet-LHH	glcm	Idm	0.123948475
wavelet-LHH	glcm	Correlation	-0.012371562
wavelet-LHH	glcm	Autocorrelation	555.95175
wavelet-LHH	glcm	SumEntropy	5.216304645
wavelet-LHH	glcm	SumSquares	49.5946004
wavelet-LHH	glcm	ClusterProminence	34426.816
wavelet-LHH	glcm	Imc2	0.894744409
wavelet-LHH	glcm	Imc1	-0.168820777
wavelet-LHH	glcm	DifferenceAverage	7.799377147
wavelet-LHH	glcm	Id	0.21180116
wavelet-LHH	glcm	ClusterTendency	98.15274767
wavelet-LHH	firstorder	InterquartileRange	205.3568206
wavelet-LHH	firstorder	Skewness	-0.163220109
wavelet-LHH	firstorder	Uniformity	0.044853835
wavelet-LHH	firstorder	Median	10.13107109
wavelet-LHH	firstorder	Energy	20817398.87
wavelet-LHH	firstorder	RobustMeanAbsoluteDeviation	87.97498274
wavelet-LHH	firstorder	MeanAbsoluteDeviation	130.4160472
wavelet-LHH	firstorder	TotalEnergy	562069769.5
wavelet-LHH	firstorder	Maximum	521.53899
wavelet-LHH	firstorder	RootMeanSquared	167.1610519
wavelet-LHH	firstorder	90Percentile	209.1391678
wavelet-LHH	firstorder	Minimum	-565.913002
wavelet-LHH	firstorder	Entropy	4.734933152
wavelet-LHH	firstorder	Range	1087.451992
wavelet-LHH	firstorder	Variance	27936.16167
wavelet-LHH	firstorder	10Percentile	-222.4180675
wavelet-LHH	firstorder	Kurtosis	3.295627272
wavelet-LHH	firstorder	Mean	2.57984601
wavelet-LHH	glrlm	ShortRunLowGrayLevelEmphasis	0.004109732
wavelet-LHH	glrlm	GrayLevelVariance	45.4015178
wavelet-LHH	glrlm	LowGrayLevelRunEmphasis	0.004165943
wavelet-LHH	glrlm	GrayLevelNonUniformityNormalized	0.044334868
wavelet-LHH	glrlm	RunVariance	0.036362854
wavelet-LHH	glrlm	GrayLevelNonUniformity	31.88982161

wavelet-LHH	glrlm	LongRunEmphasis	1.109390053
wavelet-LHH	glrlm	ShortRunHighGrayLevelEmphasis	585.6261154
wavelet-LHH	glrlm	RunLengthNonUniformity	671.1378156
wavelet-LHH	glrlm	ShortRunEmphasis	0.973726146
wavelet-LHH	glrlm	LongRunHighGrayLevelEmphasis	666.3182443
wavelet-LHH	glrlm	RunPercentage	0.965513681
wavelet-LHH	glrlm	LongRunLowGrayLevelEmphasis	0.004400457
wavelet-LHH	glrlm	RunEntropy	4.933669157
wavelet-LHH	glrlm	HighGrayLevelRunEmphasis	601.2697841
wavelet-LHH	glrlm	RunLengthNonUniformityNormalized	0.932853783
wavelet-LHH	glszm	GrayLevelVariance	55.20924526
wavelet-LHH	glszm	ZoneVariance	1.633329308
wavelet-LHH	glszm	GrayLevelNonUniformityNormalized	0.038782756
wavelet-LHH	glszm	SizeZoneNonUniformityNormalized	0.494090086
wavelet-LHH	glszm	SizeZoneNonUniformity	224.810989
wavelet-LHH	glszm	GrayLevelNonUniformity	17.64615385
wavelet-LHH	glszm	LargeAreaEmphasis	4.314285714
wavelet-LHH	glszm	SmallAreaHighGrayLevelEmphasis	445.3649483
wavelet-LHH	glszm	ZonePercentage	0.610738255
wavelet-LHH	glszm	LargeAreaLowGrayLevelEmphasis	0.012061587
wavelet-LHH	glszm	LargeAreaHighGrayLevelEmphasis	2593.393407
wavelet-LHH	glszm	HighGrayLevelZoneEmphasis	604.7252747
wavelet-LHH	glszm	SmallAreaEmphasis	0.730738805
wavelet-LHH	glszm	LowGrayLevelZoneEmphasis	0.00530878
wavelet-LHH	glszm	ZoneEntropy	6.150305379
wavelet-LHH	glszm	SmallAreaLowGrayLevelEmphasis	0.004665306
wavelet-LHH	ngtdm	Coarseness	0.008226013
wavelet-LHH	ngtdm	Complexity	3315.276482
wavelet-LHH	ngtdm	Strength	3.691024996
wavelet-LHH	ngtdm	Contrast	0.308695084
wavelet-LHH	ngtdm	Busyness	0.123722502
wavelet-LLH	gldm	GrayLevelVariance	98.26176118
wavelet-LLH	gldm	HighGrayLevelEmphasis	1108.051007
wavelet-LLH	gldm	DependenceEntropy	6.662454977
wavelet-LLH	gldm	DependenceNonUniformity	278.9409396
wavelet-LLH	gldm	GrayLevelNonUniformity	22.38255034
wavelet-LLH	gldm	SmallDependenceEmphasis	0.596372483
wavelet-LLH	gldm	SmallDependenceHighGrayLevelEmphasis	670.7019165
wavelet-LLH	gldm	DependenceNonUniformityNormalized	0.374417369
wavelet-LLH	gldm	LargeDependenceEmphasis	3.802684564
wavelet-LLH	gldm	LargeDependenceLowGrayLevelEmphasis	0.006662299
wavelet-LLH	gldm	DependenceVariance	0.799791
wavelet-LLH	gldm	LargeDependenceHighGrayLevelEmphasis	4040.757047
wavelet-LLH	gldm	SmallDependenceLowGrayLevelEmphasis	0.00255808
wavelet-LLH	gldm	LowGrayLevelEmphasis	0.003097885
wavelet-LLH	glcm	JointAverage	31.2573221
wavelet-LLH	glcm	SumAverage	62.5146442
wavelet-LLH	glcm	JointEntropy	9.281157006
wavelet-LLH	glcm	ClusterShade	574.2131701
wavelet-LLH	glcm	MaximumProbability	0.006461875
wavelet-LLH	glcm	Idmn	0.961582747
wavelet-LLH	glcm	JointEnergy	0.001905611
wavelet-LLH	glcm	Contrast	165.2592701
wavelet-LLH	glcm	DifferenceEntropy	4.626006716
wavelet-LLH	glcm	InverseVariance	0.114140256
wavelet-LLH	glcm	DifferenceVariance	64.35038216
wavelet-LLH	glcm	Idn	0.871984597
wavelet-LLH	glcm	Idm	0.10706443

wavelet-LLH	glcm	Correlation	0.157594287
wavelet-LLH	glcm	Autocorrelation	992.9990711
wavelet-LLH	glcm	SumEntropy	5.795809829
wavelet-LLH	glcm	SumSquares	98.57674425
wavelet-LLH	glcm	ClusterProminence	171158.1881
wavelet-LLH	glcm	Imc2	0.960857826
wavelet-LLH	glcm	Imc1	-0.243582665
wavelet-LLH	glcm	DifferenceAverage	9.810971739
wavelet-LLH	glcm	Id	0.187947029
wavelet-LLH	glcm	ClusterTendency	229.0477069
wavelet-LLH	firstorder	InterquartileRange	333.5986201
wavelet-LLH	firstorder	Skewness	0.037847151
wavelet-LLH	firstorder	Uniformity	0.030043692
wavelet-LLH	firstorder	Median	-28.9722785
wavelet-LLH	firstorder	Energy	46025218.41
wavelet-LLH	firstorder	RobustMeanAbsoluteDeviation	136.7447323
wavelet-LLH	firstorder	MeanAbsoluteDeviation	197.4885529
wavelet-LLH	firstorder	TotalEnergy	1242680897
wavelet-LLH	firstorder	Maximum	709.1926995
wavelet-LLH	firstorder	RootMeanSquared	248.5534483
wavelet-LLH	firstorder	90Percentile	292.5785192
wavelet-LLH	firstorder	Minimum	-783.8310482
wavelet-LLH	firstorder	Entropy	5.2936449
wavelet-LLH	firstorder	Range	1493.023748
wavelet-LLH	firstorder	Variance	61445.04814
wavelet-LLH	firstorder	10Percentile	-321.0545905
wavelet-LLH	firstorder	Kurtosis	2.996684294
wavelet-LLH	firstorder	Mean	-18.26933296
wavelet-LLH	glrlm	ShortRunLowGrayLevelEmphasis	0.003122212
wavelet-LLH	glrlm	GrayLevelVariance	99.30512623
wavelet-LLH	glrlm	LowGrayLevelRunEmphasis	0.003150315
wavelet-LLH	glrlm	GrayLevelNonUniformityNormalized	0.029839599
wavelet-LLH	glrlm	RunVariance	0.029012552
wavelet-LLH	glrlm	GrayLevelNonUniformity	21.60345871
wavelet-LLH	glrlm	LongRunEmphasis	1.088245011
wavelet-LLH	glrlm	ShortRunHighGrayLevelEmphasis	1086.512876
wavelet-LLH	glrlm	RunLengthNonUniformity	683.942799
wavelet-LLH	glrlm	ShortRunEmphasis	0.978416615
wavelet-LLH	glrlm	LongRunHighGrayLevelEmphasis	1203.08451
wavelet-LLH	glrlm	RunPercentage	0.971812081
wavelet-LLH	glrlm	LongRunLowGrayLevelEmphasis	0.003264554
wavelet-LLH	glrlm	RunEntropy	5.449702734
wavelet-LLH	glrlm	HighGrayLevelRunEmphasis	1109.413314
wavelet-LLH	glrlm	RunLengthNonUniformityNormalized	0.944452061
wavelet-LLH	glszm	GrayLevelVariance	113.5786851
wavelet-LLH	glszm	ZoneVariance	1.115128797
wavelet-LLH	glszm	GrayLevelNonUniformityNormalized	0.027474048
wavelet-LLH	glszm	SizeZoneNonUniformityNormalized	0.561245675
wavelet-LLH	glszm	SizeZoneNonUniformity	286.2352941
wavelet-LLH	glszm	GrayLevelNonUniformity	14.01176471
wavelet-LLH	glszm	LargeAreaEmphasis	3.249019608
wavelet-LLH	glszm	SmallAreaHighGrayLevelEmphasis	878.1813371
wavelet-LLH	glszm	ZonePercentage	0.684563758
wavelet-LLH	glszm	LargeAreaLowGrayLevelEmphasis	0.006711001
wavelet-LLH	glszm	LargeAreaHighGrayLevelEmphasis	3464.062745
wavelet-LLH	glszm	HighGrayLevelZoneEmphasis	1124.070588
wavelet-LLH	glszm	SmallAreaEmphasis	0.777790305
wavelet-LLH	glszm	LowGrayLevelZoneEmphasis	0.003921569

wavelet-LLH	glszm	ZoneEntropy	6.420834556
wavelet-LLH	glszm	SmallAreaLowGrayLevelEmphasis	0.003611601
wavelet-LLH	ngtdm	Coarseness	0.008742465
wavelet-LLH	ngtdm	Complexity	8810.811316
wavelet-LLH	ngtdm	Strength	8.657740958
wavelet-LLH	ngtdm	Contrast	0.410058936
wavelet-LLH	ngtdm	Busyness	0.062064821
wavelet-LLL	gldm	GrayLevelVariance	597.8870429
wavelet-LLL	gldm	HighGrayLevelEmphasis	4186.138255
wavelet-LLL	gldm	DependenceEntropy	7.228893058
wavelet-LLL	gldm	DependenceNonUniformity	402.6456376
wavelet-LLL	gldm	GrayLevelNonUniformity	9.053691275
wavelet-LLL	gldm	SmallDependenceEmphasis	0.758356823
wavelet-LLL	gldm	SmallDependenceHighGrayLevelEmphasis	2911.371018
wavelet-LLL	gldm	DependenceNonUniformityNormalized	0.540463943
wavelet-LLL	gldm	LargeDependenceEmphasis	2.32885906
wavelet-LLL	gldm	LargeDependenceLowGrayLevelEmphasis	0.002469612
wavelet-LLL	gldm	DependenceVariance	0.413700284
wavelet-LLL	gldm	LargeDependenceHighGrayLevelEmphasis	11930.08188
wavelet-LLL	gldm	SmallDependenceLowGrayLevelEmphasis	0.001923098
wavelet-LLL	gldm	LowGrayLevelEmphasis	0.002014954
wavelet-LLL	glcm	JointAverage	64.76127446
wavelet-LLL	glcm	SumAverage	129.5225489
wavelet-LLL	glcm	JointEntropy	9.954030991
wavelet-LLL	glcm	ClusterShade	-1875.406949
wavelet-LLL	glcm	MaximumProbability	0.003008567
wavelet-LLL	glcm	Idmn	0.963254726
wavelet-LLL	glcm	JointEnergy	0.001067909
wavelet-LLL	glcm	Contrast	497.143868
wavelet-LLL	glcm	DifferenceEntropy	5.410558159
wavelet-LLL	glcm	InverseVariance	0.060551968
wavelet-LLL	glcm	DifferenceVariance	177.3123152
wavelet-LLL	glcm	Idn	0.871335888
wavelet-LLL	glcm	Idm	0.05779308
wavelet-LLL	glcm	Correlation	0.565065881
wavelet-LLL	glcm	Autocorrelation	4516.246135
wavelet-LLL	glcm	SumEntropy	7.121001936
wavelet-LLL	glcm	SumSquares	570.1962109
wavelet-LLL	glcm	ClusterProminence	6698135.901
wavelet-LLL	glcm	Imc2	0.998509658
wavelet-LLL	glcm	Imc1	-0.452538342
wavelet-LLL	glcm	DifferenceAverage	17.59194153
wavelet-LLL	glcm	Id	0.122099763
wavelet-LLL	glcm	ClusterTendency	1783.640976
wavelet-LLL	firstorder	InterquartileRange	999.9296159
wavelet-LLL	firstorder	Skewness	0.100710351
wavelet-LLL	firstorder	Uniformity	0.012152606
wavelet-LLL	firstorder	Median	-1287.721694
wavelet-LLL	firstorder	Energy	1423550657
wavelet-LLL	firstorder	RobustMeanAbsoluteDeviation	406.7090693
wavelet-LLL	firstorder	MeanAbsoluteDeviation	522.8711173
wavelet-LLL	firstorder	TotalEnergy	38435867748
wavelet-LLL	firstorder	Maximum	14.59499595
wavelet-LLL	firstorder	RootMeanSquared	1382.319157
wavelet-LLL	firstorder	90Percentile	-364.4000029
wavelet-LLL	firstorder	Minimum	-2723.821531
wavelet-LLL	firstorder	Entropy	6.479233968
wavelet-LLL	firstorder	Range	2738.416527

wavelet-LLL	firstorder	Variance	373596.5936
wavelet-LLL	firstorder	10Percentile	-2001.268739
wavelet-LLL	firstorder	Kurtosis	2.006915773
wavelet-LLL	firstorder	Mean	-1239.842594
wavelet-LLL	glrlm	ShortRunLowGrayLevelEmphasis	0.002035774
wavelet-LLL	glrlm	GrayLevelVariance	595.6787759
wavelet-LLL	glrlm	LowGrayLevelRunEmphasis	0.0020398
wavelet-LLL	glrlm	GrayLevelNonUniformityNormalized	0.012155081
wavelet-LLL	glrlm	RunVariance	0.015180943
wavelet-LLL	glrlm	GrayLevelNonUniformity	8.921842953
wavelet-LLL	glrlm	LongRunEmphasis	1.045438364
wavelet-LLL	glrlm	ShortRunHighGrayLevelEmphasis	4103.473897
wavelet-LLL	glrlm	RunLengthNonUniformity	712.652279
wavelet-LLL	glrlm	ShortRunEmphasis	0.988873777
wavelet-LLL	glrlm	LongRunHighGrayLevelEmphasis	4420.053696
wavelet-LLL	glrlm	RunPercentage	0.985234899
wavelet-LLL	glrlm	LongRunLowGrayLevelEmphasis	0.002056027
wavelet-LLL	glrlm	RunEntropy	6.540353095
wavelet-LLL	glrlm	HighGrayLevelRunEmphasis	4165.197901
wavelet-LLL	glrlm	RunLengthNonUniformityNormalized	0.970878487
wavelet-LLL	glszm	GrayLevelVariance	569.4383223
wavelet-LLL	glszm	ZoneVariance	0.366170597
wavelet-LLL	glszm	GrayLevelNonUniformityNormalized	0.012377718
wavelet-LLL	glszm	SizeZoneNonUniformityNormalized	0.722051348
wavelet-LLL	glszm	SizeZoneNonUniformity	441.8954248
wavelet-LLL	glszm	GrayLevelNonUniformity	7.575163399
wavelet-LLL	glszm	LargeAreaEmphasis	1.848039216
wavelet-LLL	glszm	SmallAreaHighGrayLevelEmphasis	3290.518824
wavelet-LLL	glszm	ZonePercentage	0.82147651
wavelet-LLL	glszm	LargeAreaLowGrayLevelEmphasis	0.002659482
wavelet-LLL	glszm	LargeAreaHighGrayLevelEmphasis	8900.75
wavelet-LLL	glszm	HighGrayLevelZoneEmphasis	3915.455882
wavelet-LLL	glszm	SmallAreaEmphasis	0.874485961
wavelet-LLL	glszm	LowGrayLevelZoneEmphasis	0.002372561
wavelet-LLL	glszm	ZoneEntropy	6.988738195
wavelet-LLL	glszm	SmallAreaLowGrayLevelEmphasis	0.002322343
wavelet-LLL	ngtdm	Coarseness	0.010575572
wavelet-LLL	ngtdm	Complexity	43354.07422
wavelet-LLL	ngtdm	Strength	38.97578296
wavelet-LLL	ngtdm	Contrast	1.211935801
wavelet-LLL	ngtdm	Busyness	0.021361282
wavelet-HHL	gldm	GrayLevelVariance	34.03056439
wavelet-HHL	gldm	HighGrayLevelEmphasis	489.0080537
wavelet-HHL	gldm	DependenceEntropy	6.229359184
wavelet-HHL	gldm	DependenceNonUniformity	212.642953
wavelet-HHL	gldm	GrayLevelNonUniformity	39.33825503
wavelet-HHL	gldm	SmallDependenceEmphasis	0.491754463
wavelet-HHL	gldm	SmallDependenceHighGrayLevelEmphasis	255.9826949
wavelet-HHL	gldm	DependenceNonUniformityNormalized	0.285426783
wavelet-HHL	gldm	LargeDependenceEmphasis	5.582550336
wavelet-HHL	gldm	LargeDependenceLowGrayLevelEmphasis	0.016021711
wavelet-HHL	gldm	DependenceVariance	1.248329355
wavelet-HHL	gldm	LargeDependenceHighGrayLevelEmphasis	2612.406711
wavelet-HHL	gldm	SmallDependenceLowGrayLevelEmphasis	0.00316561
wavelet-HHL	gldm	LowGrayLevelEmphasis	0.004538851
wavelet-HHL	glcm	JointAverage	21.38880739
wavelet-HHL	glcm	SumAverage	42.77761478
wavelet-HHL	glcm	JointEntropy	8.458305906

wavelet-HHL	glcm	ClusterShade	85.40737215
wavelet-HHL	glcm	MaximumProbability	0.010302046
wavelet-HHL	glcm	Idmn	0.967353598
wavelet-HHL	glcm	JointEnergy	0.003667222
wavelet-HHL	glcm	Contrast	74.85308001
wavelet-HHL	glcm	DifferenceEntropy	4.109531119
wavelet-HHL	glcm	InverseVariance	0.153398286
wavelet-HHL	glcm	DifferenceVariance	30.09987442
wavelet-HHL	glcm	Idn	0.881576692
wavelet-HHL	glcm	Idm	0.149475839
wavelet-HHL	glcm	Correlation	-0.0327055
wavelet-HHL	glcm	Autocorrelation	456.2977763
wavelet-HHL	glcm	SumEntropy	4.980379326
wavelet-HHL	glcm	SumSquares	36.23075462
wavelet-HHL	glcm	ClusterProminence	19681.50351
wavelet-HHL	glcm	Imc2	0.858731518
wavelet-HHL	glcm	Imc1	-0.147594965
wavelet-HHL	glcm	DifferenceAverage	6.584813247
wavelet-HHL	glcm	Id	0.240200729
wavelet-HHL	glcm	ClusterTendency	70.06993846
wavelet-HHL	firstorder	InterquartileRange	174.486683
wavelet-HHL	firstorder	Skewness	0.26647365
wavelet-HHL	firstorder	Uniformity	0.052803027
wavelet-HHL	firstorder	Median	-7.036698103
wavelet-HHL	firstorder	Energy	15815281.26
wavelet-HHL	firstorder	RobustMeanAbsoluteDeviation	73.42350091
wavelet-HHL	firstorder	MeanAbsoluteDeviation	111.6610238
wavelet-HHL	firstorder	TotalEnergy	427012594.1
wavelet-HHL	firstorder	Maximum	597.4892334
wavelet-HHL	firstorder	RootMeanSquared	145.7002589
wavelet-HHL	firstorder	90Percentile	180.3254577
wavelet-HHL	firstorder	Minimum	-507.3279116
wavelet-HHL	firstorder	Entropy	4.520579274
wavelet-HHL	firstorder	Range	1104.817145
wavelet-HHL	firstorder	Variance	21212.70699
wavelet-HHL	firstorder	10Percentile	-189.8270844
wavelet-HHL	firstorder	Kurtosis	3.945055613
wavelet-HHL	firstorder	Mean	-3.982268975
wavelet-HHL	glrlm	ShortRunLowGrayLevelEmphasis	0.004543157
wavelet-HHL	glrlm	GrayLevelVariance	34.85687578
wavelet-HHL	glrlm	LowGrayLevelRunEmphasis	0.004624306
wavelet-HHL	glrlm	GrayLevelNonUniformityNormalized	0.052005667
wavelet-HHL	glrlm	RunVariance	0.046527719
wavelet-HHL	glrlm	GrayLevelNonUniformity	37.13187087
wavelet-HHL	glrlm	LongRunEmphasis	1.135712735
wavelet-HHL	glrlm	ShortRunHighGrayLevelEmphasis	475.9174474
wavelet-HHL	glrlm	RunLengthNonUniformity	657.7975618
wavelet-HHL	glrlm	ShortRunEmphasis	0.968801733
wavelet-HHL	glrlm	LongRunHighGrayLevelEmphasis	553.1780673
wavelet-HHL	glrlm	RunPercentage	0.958389262
wavelet-HHL	glrlm	LongRunLowGrayLevelEmphasis	0.004971437
wavelet-HHL	glrlm	RunEntropy	4.759732446
wavelet-HHL	glrlm	HighGrayLevelRunEmphasis	490.2138677
wavelet-HHL	glrlm	RunLengthNonUniformityNormalized	0.921066203
wavelet-HHL	glszm	GrayLevelVariance	47.39081066
wavelet-HHL	glszm	ZoneVariance	2.808361678
wavelet-HHL	glszm	GrayLevelNonUniformityNormalized	0.042834467
wavelet-HHL	glszm	SizeZoneNonUniformityNormalized	0.505170068

wavelet-HHL	glszm	SizeZoneNonUniformity	212.1714286
wavelet-HHL	glszm	GrayLevelNonUniformity	17.99047619
wavelet-HHL	glszm	LargeAreaEmphasis	5.954761905
wavelet-HHL	glszm	SmallAreaHighGrayLevelEmphasis	392.861501
wavelet-HHL	glszm	ZonePercentage	0.563758389
wavelet-HHL	glszm	LargeAreaLowGrayLevelEmphasis	0.018177897
wavelet-HHL	glszm	LargeAreaHighGrayLevelEmphasis	2820.411905
wavelet-HHL	glszm	HighGrayLevelZoneEmphasis	511.5880952
wavelet-HHL	glszm	SmallAreaEmphasis	0.738378339
wavelet-HHL	glszm	LowGrayLevelZoneEmphasis	0.006010473
wavelet-HHL	glszm	ZoneEntropy	6.041474407
wavelet-HHL	glszm	SmallAreaLowGrayLevelEmphasis	0.005243672
wavelet-HHL	ngtdm	Coarseness	0.008055962
wavelet-HHL	ngtdm	Complexity	2807.280215
wavelet-HHL	ngtdm	Strength	3.762681633
wavelet-HHL	ngtdm	Contrast	0.228178446
wavelet-HHL	ngtdm	Busyness	0.139505764
wavelet-HLH	gldm	GrayLevelVariance	31.7514094
wavelet-HLH	gldm	HighGrayLevelEmphasis	377.7114094
wavelet-HLH	gldm	DependenceEntropy	6.188980521
wavelet-HLH	gldm	DependenceNonUniformity	216.1865772
wavelet-HLH	gldm	GrayLevelNonUniformity	40.72885906
wavelet-HLH	gldm	SmallDependenceEmphasis	0.495799008
wavelet-HLH	gldm	SmallDependenceHighGrayLevelEmphasis	188.3367552
wavelet-HLH	gldm	DependenceNonUniformityNormalized	0.290183325
wavelet-HLH	gldm	LargeDependenceEmphasis	5.569127517
wavelet-HLH	gldm	LargeDependenceLowGrayLevelEmphasis	0.021943371
wavelet-HLH	gldm	DependenceVariance	1.290615738
wavelet-HLH	gldm	LargeDependenceHighGrayLevelEmphasis	2063
wavelet-HLH	gldm	SmallDependenceLowGrayLevelEmphasis	0.004480527
wavelet-HLH	gldm	LowGrayLevelEmphasis	0.006421686
wavelet-HLH	glcm	JointAverage	18.55773496
wavelet-HLH	glcm	SumAverage	37.11546993
wavelet-HLH	glcm	JointEntropy	8.40428387
wavelet-HLH	glcm	ClusterShade	-12.40069154
wavelet-HLH	glcm	MaximumProbability	0.011652841
wavelet-HLH	glcm	Idmn	0.961038525
wavelet-HLH	glcm	JointEnergy	0.003835322
wavelet-HLH	glcm	Contrast	68.27239429
wavelet-HLH	glcm	DifferenceEntropy	4.059880049
wavelet-HLH	glcm	InverseVariance	0.159224727
wavelet-HLH	glcm	DifferenceVariance	26.7475353
wavelet-HLH	glcm	Idn	0.870010559
wavelet-HLH	glcm	Idm	0.151313819
wavelet-HLH	glcm	Correlation	-0.020976018
wavelet-HLH	glcm	Autocorrelation	343.6837179
wavelet-HLH	glcm	SumEntropy	4.952612239
wavelet-HLH	glcm	SumSquares	33.42607024
wavelet-HLH	glcm	ClusterProminence	16019.23872
wavelet-HLH	glcm	Imc2	0.842864323
wavelet-HLH	glcm	Imc1	-0.138308875
wavelet-HLH	glcm	DifferenceAverage	6.367360301
wavelet-HLH	glcm	Id	0.241995686
wavelet-HLH	glcm	ClusterTendency	65.43188668
wavelet-HLH	firstorder	InterquartileRange	169.7015255
wavelet-HLH	firstorder	Skewness	0.006071139
wavelet-HLH	firstorder	Uniformity	0.054669609
wavelet-HLH	firstorder	Median	6.837569449

wavelet-HLH	firstorder	Energy	14760356.12
wavelet-HLH	firstorder	RobustMeanAbsoluteDeviation	71.22478984
wavelet-HLH	firstorder	MeanAbsoluteDeviation	108.4108029
wavelet-HLH	firstorder	TotalEnergy	398529615.4
wavelet-HLH	firstorder	Maximum	500.176589
wavelet-HLH	firstorder	RootMeanSquared	140.7570906
wavelet-HLH	firstorder	90Percentile	178.2408368
wavelet-HLH	firstorder	Minimum	-432.1579467
wavelet-HLH	firstorder	Entropy	4.478585929
wavelet-HLH	firstorder	Range	932.3345357
wavelet-HLH	firstorder	Variance	19806.2031
wavelet-HLH	firstorder	10Percentile	-174.9215735
wavelet-HLH	firstorder	Kurtosis	3.61963724
wavelet-HLH	firstorder	Mean	2.521002541
wavelet-HLH	glrlm	ShortRunLowGrayLevelEmphasis	0.00643318
wavelet-HLH	glrlm	GrayLevelVariance	32.47297746
wavelet-HLH	glrlm	LowGrayLevelRunEmphasis	0.006544543
wavelet-HLH	glrlm	GrayLevelNonUniformityNormalized	0.053838446
wavelet-HLH	glrlm	RunVariance	0.045339003
wavelet-HLH	glrlm	GrayLevelNonUniformity	38.46162463
wavelet-HLH	glrlm	LongRunEmphasis	1.133477967
wavelet-HLH	glrlm	ShortRunHighGrayLevelEmphasis	366.4469211
wavelet-HLH	glrlm	RunLengthNonUniformity	658.5597975
wavelet-HLH	glrlm	ShortRunEmphasis	0.968989629
wavelet-HLH	glrlm	LongRunHighGrayLevelEmphasis	427.4320604
wavelet-HLH	glrlm	RunPercentage	0.958905524
wavelet-HLH	glrlm	LongRunLowGrayLevelEmphasis	0.007018284
wavelet-HLH	glrlm	RunEntropy	4.71595178
wavelet-HLH	glrlm	HighGrayLevelRunEmphasis	377.9892436
wavelet-HLH	glrlm	RunLengthNonUniformityNormalized	0.921551171
wavelet-HLH	glszm	GrayLevelVariance	43.35573598
wavelet-HLH	glszm	ZoneVariance	3.170191998
wavelet-HLH	glszm	GrayLevelNonUniformityNormalized	0.044058655
wavelet-HLH	glszm	SizeZoneNonUniformityNormalized	0.508330465
wavelet-HLH	glszm	SizeZoneNonUniformity	214.0071259
wavelet-HLH	glszm	GrayLevelNonUniformity	18.54869359
wavelet-HLH	glszm	LargeAreaEmphasis	6.301662708
wavelet-HLH	glszm	SmallAreaHighGrayLevelEmphasis	280.9845122
wavelet-HLH	glszm	ZonePercentage	0.565100671
wavelet-HLH	glszm	LargeAreaLowGrayLevelEmphasis	0.025665949
wavelet-HLH	glszm	LargeAreaHighGrayLevelEmphasis	2360.235154
wavelet-HLH	glszm	HighGrayLevelZoneEmphasis	379.263658
wavelet-HLH	glszm	SmallAreaEmphasis	0.740551595
wavelet-HLH	glszm	LowGrayLevelZoneEmphasis	0.008554677
wavelet-HLH	glszm	ZoneEntropy	5.990910846
wavelet-HLH	glszm	SmallAreaLowGrayLevelEmphasis	0.007398044
wavelet-HLH	ngtdm	Coarseness	0.008238153
wavelet-HLH	ngtdm	Complexity	2266.290822
wavelet-HLH	ngtdm	Strength	3.127972924
wavelet-HLH	ngtdm	Contrast	0.226533923
wavelet-HLH	ngtdm	Busyness	0.162997946

Image type	Feature Class	Feature Name	4: RTSTRUCT: C99-DNu_segment_GTV
general	info	BoundingBox	(0, 0, 1, 10, 10, 10)
general	info	EnabledImageTypes	{u'Original': {}, u'Wavelet': {}}
general	info	GeneralSettings	{'distances': [1], 'additionalInfo': True, 'force2D': False, 'interpolator': 'sitkBSpline', 'resampledPixelSpacing': [3.0], 'label': 1, 'normalizeScale': 1, 'normalize': False, 'force2Ddimension': 0, 'removeOutliers': None, 'minimumROISize': None, u'binWidth': 25.0, 'minimumROIDimensions': 1, u'correctMask': True, 'preCrop': False, u'symmetricalGLCM': True, 'resegmentRange': None, 'padDistance': 5}
general	info	ImageHash	92feba319139b5a5d9736c33c57ad7efd275d5cc
general	info	ImageSpacing	(0.9765625000000002, 0.9765625000000002, 2.0)
general	info	MaskHash	5cfc521b1794931be3281555344829d6f904cff7
general	info	NumpyVersion	1.13.1
general	info	PyWaveletVersion	0.5.2
general	info	SimpleITKVersion	1.1.0
general	info	Version	1.3.0.post102+g1b0eb74
general	info	VolumeNum	1
general	info	VoxelNum	383
original	shape	Maximum3DDiameter	33.27160952
original	shape	Maximum2DDiameterSlice	27
original	shape	Sphericity	0.839350093
original	shape	MinorAxis	23.34676671
original	shape	Elongation	0.778284464
original	shape	SurfaceVolumeRatio	0.264456027
original	shape	Volume	10341
original	shape	MajorAxis	29.99772937
original	shape	SurfaceArea	2734.739773
original	shape	Flatness	0.728591265
original	shape	LeastAxis	21.85608359
original	shape	Maximum2DDiameterColumn	30
original	shape	Maximum2DDiameterRow	30.88689042
original	gldm	GrayLevelVariance	96.35067387
original	gldm	HighGrayLevelEmphasis	962.9843342
original	gldm	DependenceEntropy	6.479280795
original	gldm	DependenceNonUniformity	98.92167102
original	gldm	GrayLevelNonUniformity	17.37597911
original	gldm	SmallDependenceEmphasis	0.516220852
original	gldm	SmallDependenceHighGrayLevelEmphasis	356.9896067
original	gldm	DependenceNonUniformityNormalized	0.258281125
original	gldm	LargeDependenceEmphasis	15.19321149
original	gldm	LargeDependenceLowGrayLevelEmphasis	0.015195106
original	gldm	DependenceVariance	6.989399341
original	gldm	LargeDependenceHighGrayLevelEmphasis	21953.02089

original	gldm	SmallDependenceLowGrayLevelEmphasis	0.004263368
original	gldm	LowGrayLevelEmphasis	0.00477943
original	glcm	JointAverage	31.51763249
original	glcm	SumAverage	63.03526498
original	glcm	JointEntropy	7.835241082
original	glcm	ClusterShade	-2816.957045
original	glcm	MaximumProbability	0.042530836
original	glcm	Idmn	0.959859779
original	glcm	JointEnergy	0.008241152
original	glcm	Contrast	86.78854776
original	glcm	DifferenceEntropy	4.137435585
original	glcm	InverseVariance	0.204317832
original	glcm	DifferenceVariance	41.52791046
original	glcm	Idn	0.879144406
original	glcm	Idm	0.215415041
original	glcm	Correlation	0.473890753
original	glcm	Autocorrelation	1033.030091
original	glcm	SumEntropy	5.408064635
original	glcm	SumSquares	82.93830618
original	glcm	ClusterProminence	156148.1646
original	glcm	Imc2	0.968335027
original	glcm	Imc1	-0.303233482
original	glcm	DifferenceAverage	6.6613039
original	glcm	Id	0.290891584
original	glcm	ClusterTendency	244.964677
original	firstorder	InterquartileRange	432.5
original	firstorder	Skewness	-0.598191176
original	firstorder	Uniformity	0.045368092
original	firstorder	Median	-175
original	firstorder	Energy	42726667
original	firstorder	RobustMeanAbsoluteDeviation	167.3160323
original	firstorder	MeanAbsoluteDeviation	211.2375979
original	firstorder	TotalEnergy	1153620009
original	firstorder	Maximum	103
original	firstorder	RootMeanSquared	334.0028103
original	firstorder	90Percentile	34
original	firstorder	Minimum	-936
original	firstorder	Entropy	4.853226677
original	firstorder	Range	1039
original	firstorder	Variance	60028.87728
original	firstorder	10Percentile	-603.6
original	firstorder	Kurtosis	2.242123677
original	firstorder	Mean	-227
original	glrlm	ShortRunLowGrayLevelEmphasis	0.005041844
original	glrlm	GrayLevelVariance	96.64550679
original	glrlm	LowGrayLevelRunEmphasis	0.005086116
original	glrlm	GrayLevelNonUniformityNormalized	0.03990722
original	glrlm	RunVariance	0.09445664
original	glrlm	GrayLevelNonUniformity	14.19694292
original	glrlm	LongRunEmphasis	1.256242388
original	glrlm	ShortRunHighGrayLevelEmphasis	855.5339351
original	glrlm	RunLengthNonUniformity	310.5539222

original	glrlm	ShortRunEmphasis	0.947937583
original	glrlm	LongRunHighGrayLevelEmphasis	1292.763415
original	glrlm	RunPercentage	0.928298855
original	glrlm	LongRunLowGrayLevelEmphasis	0.005294761
original	glrlm	RunEntropy	5.202745988
original	glrlm	HighGrayLevelRunEmphasis	928.1992393
original	glrlm	RunLengthNonUniformityNormalized	0.872977856
original	glszm	GrayLevelVariance	87.77850453
original	glszm	ZoneVariance	15.79269044
original	glszm	GrayLevelNonUniformityNormalized	0.032173119
original	glszm	SizeZoneNonUniformityNormalized	0.644630381
original	glszm	SizeZoneNonUniformity	139.8847926
original	glszm	GrayLevelNonUniformity	6.98156682
original	glszm	LargeAreaEmphasis	18.9078341
original	glszm	SmallAreaHighGrayLevelEmphasis	539.7492534
original	glszm	ZonePercentage	0.566579634
original	glszm	LargeAreaLowGrayLevelEmphasis	0.020176809
original	glszm	LargeAreaHighGrayLevelEmphasis	27742.14286
original	glszm	HighGrayLevelZoneEmphasis	701.5391705
original	glszm	SmallAreaEmphasis	0.828471427
original	glszm	LowGrayLevelZoneEmphasis	0.007684289
original	glszm	ZoneEntropy	5.798614981
original	glszm	SmallAreaLowGrayLevelEmphasis	0.007417118
original	ngtdm	Coarseness	0.015192695
original	ngtdm	Complexity	2876.318192
original	ngtdm	Strength	11.08278377
original	ngtdm	Contrast	0.622248126
original	ngtdm	Busyness	0.052532014
wavelet-HHH	gldm	GrayLevelVariance	5.766431021
wavelet-HHH	gldm	HighGrayLevelEmphasis	114.8407311
wavelet-HHH	gldm	DependenceEntropy	5.660984456
wavelet-HHH	gldm	DependenceNonUniformity	59.54308094
wavelet-HHH	gldm	GrayLevelNonUniformity	49.44908616
wavelet-HHH	gldm	SmallDependenceEmphasis	0.283750579
wavelet-HHH	gldm	SmallDependenceHighGrayLevelEmphasis	33.55135914
wavelet-HHH	gldm	DependenceNonUniformityNormalized	0.155464963
wavelet-HHH	gldm	LargeDependenceEmphasis	17.09921671
wavelet-HHH	gldm	LargeDependenceLowGrayLevelEmphasis	0.16393624
wavelet-HHH	gldm	DependenceVariance	4.949532685
wavelet-HHH	gldm	LargeDependenceHighGrayLevelEmphasis	1953.10705
wavelet-HHH	gldm	SmallDependenceLowGrayLevelEmphasis	0.006790401
wavelet-HHH	gldm	LowGrayLevelEmphasis	0.013971329
wavelet-HHH	glcm	JointAverage	10.46295952
wavelet-HHH	glcm	SumAverage	20.92591903
wavelet-HHH	glcm	JointEntropy	6.192632503
wavelet-HHH	glcm	ClusterShade	-5.298069955
wavelet-HHH	glcm	MaximumProbability	0.053753255
wavelet-HHH	glcm	Idmn	0.967739444
wavelet-HHH	glcm	JointEnergy	0.019967772
wavelet-HHH	glcm	Contrast	11.76664201
wavelet-HHH	glcm	DifferenceEntropy	2.88380452
wavelet-HHH	glcm	InverseVariance	0.336568854

wavelet-HHH	glcm	DifferenceVariance	4.822189149
wavelet-HHH	glcm	Idn	0.882805313
wavelet-HHH	glcm	Idm	0.323386854
wavelet-HHH	glcm	Correlation	-0.028390147
wavelet-HHH	glcm	Autocorrelation	109.3117471
wavelet-HHH	glcm	SumEntropy	3.669867342
wavelet-HHH	glcm	SumSquares	5.721041562
wavelet-HHH	glcm	ClusterProminence	486.8447125
wavelet-HHH	glcm	Imc2	0.687081744
wavelet-HHH	glcm	Imc1	-0.099894547
wavelet-HHH	glcm	DifferenceAverage	2.605625481
wavelet-HHH	glcm	Id	0.404078443
wavelet-HHH	glcm	ClusterTendency	11.11752424
wavelet-HHH	firstorder	InterquartileRange	67.85407595
wavelet-HHH	firstorder	Skewness	-0.217921684
wavelet-HHH	firstorder	Uniformity	0.129109886
wavelet-HHH	firstorder	Median	3.595869644
wavelet-HHH	firstorder	Energy	1347115.061
wavelet-HHH	firstorder	RobustMeanAbsoluteDeviation	29.7207068
wavelet-HHH	firstorder	MeanAbsoluteDeviation	45.32147551
wavelet-HHH	firstorder	TotalEnergy	36372106.64
wavelet-HHH	firstorder	Maximum	188.7905106
wavelet-HHH	firstorder	RootMeanSquared	59.30659069
wavelet-HHH	firstorder	90Percentile	71.70943954
wavelet-HHH	firstorder	Minimum	-225.6332496
wavelet-HHH	firstorder	Entropy	3.264586548
wavelet-HHH	firstorder	Range	414.4237602
wavelet-HHH	firstorder	Variance	3515.220436
wavelet-HHH	firstorder	10Percentile	-77.26651944
wavelet-HHH	firstorder	Kurtosis	3.835006043
wavelet-HHH	firstorder	Mean	-1.432222878
wavelet-HHH	glrlm	ShortRunLowGrayLevelEmphasis	0.013749603
wavelet-HHH	glrlm	GrayLevelVariance	6.146580231
wavelet-HHH	glrlm	LowGrayLevelRunEmphasis	0.014440133
wavelet-HHH	glrlm	GrayLevelNonUniformityNormalized	0.122439209
wavelet-HHH	glrlm	RunVariance	0.121135783
wavelet-HHH	glrlm	GrayLevelNonUniformity	42.41105618
wavelet-HHH	glrlm	LongRunEmphasis	1.345297938
wavelet-HHH	glrlm	ShortRunHighGrayLevelEmphasis	106.7015715
wavelet-HHH	glrlm	RunLengthNonUniformity	287.3105333
wavelet-HHH	glrlm	ShortRunEmphasis	0.927964938
wavelet-HHH	glrlm	LongRunHighGrayLevelEmphasis	154.2666768
wavelet-HHH	glrlm	RunPercentage	0.904398474
wavelet-HHH	glrlm	LongRunLowGrayLevelEmphasis	0.017732093
wavelet-HHH	glrlm	RunEntropy	3.757072423
wavelet-HHH	glrlm	HighGrayLevelRunEmphasis	114.9264613
wavelet-HHH	glrlm	RunLengthNonUniformityNormalized	0.828765965
wavelet-HHH	glszm	GrayLevelVariance	12.80009739
wavelet-HHH	glszm	ZoneVariance	95.58087818
wavelet-HHH	glszm	GrayLevelNonUniformityNormalized	0.085463842
wavelet-HHH	glszm	SizeZoneNonUniformityNormalized	0.487054622
wavelet-HHH	glszm	SizeZoneNonUniformity	54.06306306

wavelet-HHH	glszm	GrayLevelNonUniformity	9.486486486
wavelet-HHH	glszm	LargeAreaEmphasis	107.4864865
wavelet-HHH	glszm	SmallAreaHighGrayLevelEmphasis	86.95302033
wavelet-HHH	glszm	ZonePercentage	0.289817232
wavelet-HHH	glszm	LargeAreaLowGrayLevelEmphasis	0.971427309
wavelet-HHH	glszm	LargeAreaHighGrayLevelEmphasis	12537.24324
wavelet-HHH	glszm	HighGrayLevelZoneEmphasis	117.1711712
wavelet-HHH	glszm	SmallAreaEmphasis	0.724419572
wavelet-HHH	glszm	LowGrayLevelZoneEmphasis	0.024068499
wavelet-HHH	glszm	ZoneEntropy	4.888048917
wavelet-HHH	glszm	SmallAreaLowGrayLevelEmphasis	0.020742152
wavelet-HHH	ngtdm	Coarseness	0.016681785
wavelet-HHH	ngtdm	Complexity	223.7427044
wavelet-HHH	ngtdm	Strength	1.424330984
wavelet-HHH	ngtdm	Contrast	0.081963128
wavelet-HHH	ngtdm	Busyness	0.28506548
wavelet-HLL	gldm	GrayLevelVariance	47.83055307
wavelet-HLL	gldm	HighGrayLevelEmphasis	494.8825065
wavelet-HLL	gldm	DependenceEntropy	6.416201809
wavelet-HLL	gldm	DependenceNonUniformity	102.2375979
wavelet-HLL	gldm	GrayLevelNonUniformity	16.35770235
wavelet-HLL	gldm	SmallDependenceEmphasis	0.470978962
wavelet-HLL	gldm	SmallDependenceHighGrayLevelEmphasis	252.201166
wavelet-HLL	gldm	DependenceNonUniformityNormalized	0.266938898
wavelet-HLL	gldm	LargeDependenceEmphasis	7.866840731
wavelet-HLL	gldm	LargeDependenceLowGrayLevelEmphasis	0.024062612
wavelet-HLL	gldm	DependenceVariance	2.551588735
wavelet-HLL	gldm	LargeDependenceHighGrayLevelEmphasis	3867.373368
wavelet-HLL	gldm	SmallDependenceLowGrayLevelEmphasis	0.004970149
wavelet-HLL	gldm	LowGrayLevelEmphasis	0.006686106
wavelet-HLL	glcm	JointAverage	20.96263026
wavelet-HLL	glcm	SumAverage	41.92526052
wavelet-HLL	glcm	JointEntropy	8.151721725
wavelet-HLL	glcm	ClusterShade	-46.00690252
wavelet-HLL	glcm	MaximumProbability	0.014632914
wavelet-HLL	glcm	Idmn	0.961369326
wavelet-HLL	glcm	JointEnergy	0.004286534
wavelet-HLL	glcm	Contrast	63.19014893
wavelet-HLL	glcm	DifferenceEntropy	3.945924564
wavelet-HLL	glcm	InverseVariance	0.167756354
wavelet-HLL	glcm	DifferenceVariance	23.23117027
wavelet-HLL	glcm	Idn	0.871324485
wavelet-HLL	glcm	Idm	0.169318025
wavelet-HLL	glcm	Correlation	0.234763887
wavelet-HLL	glcm	Autocorrelation	449.7987134
wavelet-HLL	glcm	SumEntropy	5.219370431
wavelet-HLL	glcm	SumSquares	41.94536773
wavelet-HLL	glcm	ClusterProminence	33131.03593
wavelet-HLL	glcm	Imc2	0.945891213
wavelet-HLL	glcm	Imc1	-0.246924831
wavelet-HLL	glcm	DifferenceAverage	6.136882733
wavelet-HLL	glcm	Id	0.25758053

wavelet-HLL	glcm	ClusterTendency	104.591322
wavelet-HLL	firstorder	InterquartileRange	232.3443078
wavelet-HLL	firstorder	Skewness	-0.084169091
wavelet-HLL	firstorder	Uniformity	0.042709406
wavelet-HLL	firstorder	Median	-54.13327058
wavelet-HLL	firstorder	Energy	12755560.67
wavelet-HLL	firstorder	RobustMeanAbsoluteDeviation	96.23543335
wavelet-HLL	firstorder	MeanAbsoluteDeviation	137.9873484
wavelet-HLL	firstorder	TotalEnergy	344400138
wavelet-HLL	firstorder	Maximum	361.7266677
wavelet-HLL	firstorder	RootMeanSquared	182.4947559
wavelet-HLL	firstorder	90Percentile	171.782493
wavelet-HLL	firstorder	Minimum	-568.6950422
wavelet-HLL	firstorder	Entropy	4.752089858
wavelet-HLL	firstorder	Range	930.4217099
wavelet-HLL	firstorder	Variance	29847.35402
wavelet-HLL	firstorder	10Percentile	-279.5096328
wavelet-HLL	firstorder	Kurtosis	2.785089546
wavelet-HLL	firstorder	Mean	-58.79610467
wavelet-HLL	glrlm	ShortRunLowGrayLevelEmphasis	0.006783904
wavelet-HLL	glrlm	GrayLevelVariance	49.12377839
wavelet-HLL	glrlm	LowGrayLevelRunEmphasis	0.006892163
wavelet-HLL	glrlm	GrayLevelNonUniformityNormalized	0.041756155
wavelet-HLL	glrlm	RunVariance	0.058155463
wavelet-HLL	glrlm	GrayLevelNonUniformity	15.18916684
wavelet-HLL	glrlm	LongRunEmphasis	1.168891044
wavelet-HLL	glrlm	ShortRunHighGrayLevelEmphasis	478.1129249
wavelet-HLL	glrlm	RunLengthNonUniformity	330.1940374
wavelet-HLL	glrlm	ShortRunEmphasis	0.962345937
wavelet-HLL	glrlm	LongRunHighGrayLevelEmphasis	576.0621414
wavelet-HLL	glrlm	RunPercentage	0.949789114
wavelet-HLL	glrlm	LongRunLowGrayLevelEmphasis	0.007367079
wavelet-HLL	glrlm	RunEntropy	4.996167014
wavelet-HLL	glrlm	HighGrayLevelRunEmphasis	495.983079
wavelet-HLL	glrlm	RunLengthNonUniformityNormalized	0.906646122
wavelet-HLL	glszm	GrayLevelVariance	64.23836451
wavelet-HLL	glszm	ZoneVariance	4.895400746
wavelet-HLL	glszm	GrayLevelNonUniformityNormalized	0.037293102
wavelet-HLL	glszm	SizeZoneNonUniformityNormalized	0.491128866
wavelet-HLL	glszm	SizeZoneNonUniformity	102.645933
wavelet-HLL	glszm	GrayLevelNonUniformity	7.794258373
wavelet-HLL	glszm	LargeAreaEmphasis	8.253588517
wavelet-HLL	glszm	SmallAreaHighGrayLevelEmphasis	402.4788504
wavelet-HLL	glszm	ZonePercentage	0.545691906
wavelet-HLL	glszm	LargeAreaLowGrayLevelEmphasis	0.027816212
wavelet-HLL	glszm	LargeAreaHighGrayLevelEmphasis	4048.478469
wavelet-HLL	glszm	HighGrayLevelZoneEmphasis	524.5358852
wavelet-HLL	glszm	SmallAreaEmphasis	0.72811925
wavelet-HLL	glszm	LowGrayLevelZoneEmphasis	0.009673957
wavelet-HLL	glszm	ZoneEntropy	5.966164026
wavelet-HLL	glszm	SmallAreaLowGrayLevelEmphasis	0.008639706
wavelet-HLL	ngtdm	Coarseness	0.016665385

wavelet-HLL	ngtdm	Complexity	2284.011546
wavelet-HLL	ngtdm	Strength	6.622759477
wavelet-HLL	ngtdm	Contrast	0.350486119
wavelet-HLL	ngtdm	Busyness	0.087872288
wavelet-LHL	gldm	GrayLevelVariance	36.39701682
wavelet-LHL	gldm	HighGrayLevelEmphasis	293.9869452
wavelet-LHL	gldm	DependenceEntropy	6.291058896
wavelet-LHL	gldm	DependenceNonUniformity	94.16449086
wavelet-LHL	gldm	GrayLevelNonUniformity	19.29765013
wavelet-LHL	gldm	SmallDependenceEmphasis	0.436406228
wavelet-LHL	gldm	SmallDependenceHighGrayLevelEmphasis	134.7159401
wavelet-LHL	gldm	DependenceNonUniformityNormalized	0.245860289
wavelet-LHL	gldm	LargeDependenceEmphasis	9.412532637
wavelet-LHL	gldm	LargeDependenceLowGrayLevelEmphasis	0.049596164
wavelet-LHL	gldm	DependenceVariance	3.169058348
wavelet-LHL	gldm	LargeDependenceHighGrayLevelEmphasis	2648.624021
wavelet-LHL	gldm	SmallDependenceLowGrayLevelEmphasis	0.007518078
wavelet-LHL	gldm	LowGrayLevelEmphasis	0.011022866
wavelet-LHL	glcm	JointAverage	15.81851851
wavelet-LHL	glcm	SumAverage	31.63703701
wavelet-LHL	glcm	JointEntropy	7.939514884
wavelet-LHL	glcm	ClusterShade	134.0780447
wavelet-LHL	glcm	MaximumProbability	0.01871355
wavelet-LHL	glcm	Idmn	0.972537539
wavelet-LHL	glcm	JointEnergy	0.005160577
wavelet-LHL	glcm	Contrast	53.56163009
wavelet-LHL	glcm	DifferenceEntropy	3.858428813
wavelet-LHL	glcm	InverseVariance	0.177677357
wavelet-LHL	glcm	DifferenceVariance	21.68191103
wavelet-LHL	glcm	Idn	0.891486369
wavelet-LHL	glcm	Idm	0.186539268
wavelet-LHL	glcm	Correlation	0.167070664
wavelet-LHL	glcm	Autocorrelation	255.5507389
wavelet-LHL	glcm	SumEntropy	4.97771902
wavelet-LHL	glcm	SumSquares	32.09416207
wavelet-LHL	glcm	ClusterProminence	19363.84835
wavelet-LHL	glcm	Imc2	0.922062936
wavelet-LHL	glcm	Imc1	-0.215778739
wavelet-LHL	glcm	DifferenceAverage	5.544140919
wavelet-LHL	glcm	Id	0.276517202
wavelet-LHL	glcm	ClusterTendency	74.81501821
wavelet-LHL	firstorder	InterquartileRange	192.7653021
wavelet-LHL	firstorder	Skewness	0.166906442
wavelet-LHL	firstorder	Uniformity	0.050385509
wavelet-LHL	firstorder	Median	-31.95474877
wavelet-LHL	firstorder	Energy	9306240.597
wavelet-LHL	firstorder	RobustMeanAbsoluteDeviation	82.6923674
wavelet-LHL	firstorder	MeanAbsoluteDeviation	119.9498935
wavelet-LHL	firstorder	TotalEnergy	251268496.1
wavelet-LHL	firstorder	Maximum	617.4777078
wavelet-LHL	firstorder	RootMeanSquared	155.8790503
wavelet-LHL	firstorder	90Percentile	152.9276997

wavelet-LHL	firstorder	Minimum	-421.6775041
wavelet-LHL	firstorder	Entropy	4.537202504
wavelet-LHL	firstorder	Range	1039.155212
wavelet-LHL	firstorder	Variance	22953.91851
wavelet-LHL	firstorder	10Percentile	-242.5417453
wavelet-LHL	firstorder	Kurtosis	3.345598954
wavelet-LHL	firstorder	Mean	-36.66551261
wavelet-LHL	glrlm	ShortRunLowGrayLevelEmphasis	0.011152023
wavelet-LHL	glrlm	GrayLevelVariance	37.69586076
wavelet-LHL	glrlm	LowGrayLevelRunEmphasis	0.011381938
wavelet-LHL	glrlm	GrayLevelNonUniformityNormalized	0.048878033
wavelet-LHL	glrlm	RunVariance	0.069022258
wavelet-LHL	glrlm	GrayLevelNonUniformity	17.64298756
wavelet-LHL	glrlm	LongRunEmphasis	1.197049562
wavelet-LHL	glrlm	ShortRunHighGrayLevelEmphasis	282.9090879
wavelet-LHL	glrlm	RunLengthNonUniformity	322.979876
wavelet-LHL	glrlm	ShortRunEmphasis	0.95702069
wavelet-LHL	glrlm	LongRunHighGrayLevelEmphasis	350.1809588
wavelet-LHL	glrlm	RunPercentage	0.942357903
wavelet-LHL	glrlm	LongRunLowGrayLevelEmphasis	0.012387801
wavelet-LHL	glrlm	RunEntropy	4.822720333
wavelet-LHL	glrlm	HighGrayLevelRunEmphasis	294.8707676
wavelet-LHL	glrlm	RunLengthNonUniformityNormalized	0.893957484
wavelet-LHL	glszm	GrayLevelVariance	51.68126233
wavelet-LHL	glszm	ZoneVariance	6.885890861
wavelet-LHL	glszm	GrayLevelNonUniformityNormalized	0.040631164
wavelet-LHL	glszm	SizeZoneNonUniformityNormalized	0.464930966
wavelet-LHL	glszm	SizeZoneNonUniformity	90.66153846
wavelet-LHL	glszm	GrayLevelNonUniformity	7.923076923
wavelet-LHL	glszm	LargeAreaEmphasis	10.74358974
wavelet-LHL	glszm	SmallAreaHighGrayLevelEmphasis	221.5536023
wavelet-LHL	glszm	ZonePercentage	0.509138381
wavelet-LHL	glszm	LargeAreaLowGrayLevelEmphasis	0.059997338
wavelet-LHL	glszm	LargeAreaHighGrayLevelEmphasis	3024.415385
wavelet-LHL	glszm	HighGrayLevelZoneEmphasis	305.225641
wavelet-LHL	glszm	SmallAreaEmphasis	0.708394282
wavelet-LHL	glszm	LowGrayLevelZoneEmphasis	0.016027379
wavelet-LHL	glszm	ZoneEntropy	5.890589133
wavelet-LHL	glszm	SmallAreaLowGrayLevelEmphasis	0.013759542
wavelet-LHL	ngtdm	Coarseness	0.017052832
wavelet-LHL	ngtdm	Complexity	1658.787547
wavelet-LHL	ngtdm	Strength	5.501357155
wavelet-LHL	ngtdm	Contrast	0.302932124
wavelet-LHL	ngtdm	Busyness	0.118389189
wavelet-LHH	gldm	GrayLevelVariance	13.73920335
wavelet-LHH	gldm	HighGrayLevelEmphasis	177.4856397
wavelet-LHH	gldm	DependenceEntropy	5.939698396
wavelet-LHH	gldm	DependenceNonUniformity	75.6997389
wavelet-LHH	gldm	GrayLevelNonUniformity	34.59791123
wavelet-LHH	gldm	SmallDependenceEmphasis	0.384255026
wavelet-LHH	gldm	SmallDependenceHighGrayLevelEmphasis	70.89805632
wavelet-LHH	gldm	DependenceNonUniformityNormalized	0.197649449

wavelet-LHH	gldm	LargeDependenceEmphasis	12.83289817
wavelet-LHH	gldm	LargeDependenceLowGrayLevelEmphasis	0.093276403
wavelet-LHH	gldm	DependenceVariance	4.35765463
wavelet-LHH	gldm	LargeDependenceHighGrayLevelEmphasis	2014.462141
wavelet-LHH	gldm	SmallDependenceLowGrayLevelEmphasis	0.006676768
wavelet-LHH	gldm	LowGrayLevelEmphasis	0.011137599
wavelet-LHH	glcm	JointAverage	12.74006127
wavelet-LHH	glcm	SumAverage	25.48012255
wavelet-LHH	glcm	JointEntropy	7.0057945
wavelet-LHH	glcm	ClusterShade	37.29225804
wavelet-LHH	glcm	MaximumProbability	0.039066173
wavelet-LHH	glcm	Idmn	0.957855148
wavelet-LHH	glcm	JointEnergy	0.011935392
wavelet-LHH	glcm	Contrast	25.99302643
wavelet-LHH	glcm	DifferenceEntropy	3.380447937
wavelet-LHH	glcm	InverseVariance	0.267860395
wavelet-LHH	glcm	DifferenceVariance	10.98960975
wavelet-LHH	glcm	Idn	0.869732591
wavelet-LHH	glcm	Idm	0.256533412
wavelet-LHH	glcm	Correlation	0.002280376
wavelet-LHH	glcm	Autocorrelation	162.3523087
wavelet-LHH	glcm	SumEntropy	4.238824552
wavelet-LHH	glcm	SumSquares	13.03290091
wavelet-LHH	glcm	ClusterProminence	2571.597746
wavelet-LHH	glcm	Imc2	0.814978382
wavelet-LHH	glcm	Imc1	-0.146263001
wavelet-LHH	glcm	DifferenceAverage	3.820530986
wavelet-LHH	glcm	Id	0.341848838
wavelet-LHH	glcm	ClusterTendency	26.1385772
wavelet-LHH	firstorder	InterquartileRange	106.6127336
wavelet-LHH	firstorder	Skewness	0.252659326
wavelet-LHH	firstorder	Uniformity	0.090333972
wavelet-LHH	firstorder	Median	-7.126531482
wavelet-LHH	firstorder	Energy	3303898.315
wavelet-LHH	firstorder	RobustMeanAbsoluteDeviation	44.32119923
wavelet-LHH	firstorder	MeanAbsoluteDeviation	70.15658801
wavelet-LHH	firstorder	TotalEnergy	89205254.51
wavelet-LHH	firstorder	Maximum	269.3045495
wavelet-LHH	firstorder	RootMeanSquared	92.87823404
wavelet-LHH	firstorder	90Percentile	133.6166895
wavelet-LHH	firstorder	Minimum	-281.4819955
wavelet-LHH	firstorder	Entropy	3.830376559
wavelet-LHH	firstorder	Range	550.7865451
wavelet-LHH	firstorder	Variance	8577.079645
wavelet-LHH	firstorder	10Percentile	-88.83854476
wavelet-LHH	firstorder	Kurtosis	3.470839487
wavelet-LHH	firstorder	Mean	7.020449678
wavelet-LHH	glrlm	ShortRunLowGrayLevelEmphasis	0.011077089
wavelet-LHH	glrlm	GrayLevelVariance	14.46040578
wavelet-LHH	glrlm	LowGrayLevelRunEmphasis	0.011464544
wavelet-LHH	glrlm	GrayLevelNonUniformityNormalized	0.085363023
wavelet-LHH	glrlm	RunVariance	0.09146406

wavelet-LHH	glrlm	GrayLevelNonUniformity	30.29759878
wavelet-LHH	glrlm	LongRunEmphasis	1.257844063
wavelet-LHH	glrlm	ShortRunHighGrayLevelEmphasis	169.8807928
wavelet-LHH	glrlm	RunLengthNonUniformity	307.4659543
wavelet-LHH	glrlm	ShortRunEmphasis	0.945004138
wavelet-LHH	glrlm	LongRunHighGrayLevelEmphasis	220.0563845
wavelet-LHH	glrlm	RunPercentage	0.926491263
wavelet-LHH	glrlm	LongRunLowGrayLevelEmphasis	0.013277798
wavelet-LHH	glrlm	RunEntropy	4.216201693
wavelet-LHH	glrlm	HighGrayLevelRunEmphasis	178.8014876
wavelet-LHH	glrlm	RunLengthNonUniformityNormalized	0.865928274
wavelet-LHH	glszm	GrayLevelVariance	22.80756657
wavelet-LHH	glszm	ZoneVariance	18.59379247
wavelet-LHH	glszm	GrayLevelNonUniformityNormalized	0.058879706
wavelet-LHH	glszm	SizeZoneNonUniformityNormalized	0.484591368
wavelet-LHH	glszm	SizeZoneNonUniformity	79.95757576
wavelet-LHH	glszm	GrayLevelNonUniformity	9.715151515
wavelet-LHH	glszm	LargeAreaEmphasis	23.98181818
wavelet-LHH	glszm	SmallAreaHighGrayLevelEmphasis	133.4552287
wavelet-LHH	glszm	ZonePercentage	0.430809399
wavelet-LHH	glszm	LargeAreaLowGrayLevelEmphasis	0.175736578
wavelet-LHH	glszm	LargeAreaHighGrayLevelEmphasis	3710.357576
wavelet-LHH	glszm	HighGrayLevelZoneEmphasis	189.1393939
wavelet-LHH	glszm	SmallAreaEmphasis	0.723033951
wavelet-LHH	glszm	LowGrayLevelZoneEmphasis	0.016412895
wavelet-LHH	glszm	ZoneEntropy	5.39464487
wavelet-LHH	glszm	SmallAreaLowGrayLevelEmphasis	0.014255346
wavelet-LHH	ngtdm	Coarseness	0.016881586
wavelet-LHH	ngtdm	Complexity	511.4553229
wavelet-LHH	ngtdm	Strength	2.209185302
wavelet-LHH	ngtdm	Contrast	0.170433189
wavelet-LHH	ngtdm	Busyness	0.206110811
wavelet-LLH	gldm	GrayLevelVariance	33.63837779
wavelet-LLH	gldm	HighGrayLevelEmphasis	382.537859
wavelet-LLH	gldm	DependenceEntropy	6.226406683
wavelet-LLH	gldm	DependenceNonUniformity	94.45169713
wavelet-LLH	gldm	GrayLevelNonUniformity	20.16971279
wavelet-LLH	gldm	SmallDependenceEmphasis	0.43218503
wavelet-LLH	gldm	SmallDependenceHighGrayLevelEmphasis	183.0652009
wavelet-LLH	gldm	DependenceNonUniformityNormalized	0.246610175
wavelet-LLH	gldm	LargeDependenceEmphasis	7.234986945
wavelet-LLH	gldm	LargeDependenceLowGrayLevelEmphasis	0.045304044
wavelet-LLH	gldm	DependenceVariance	1.749851727
wavelet-LLH	gldm	LargeDependenceHighGrayLevelEmphasis	2587.56658
wavelet-LLH	gldm	SmallDependenceLowGrayLevelEmphasis	0.003343652
wavelet-LLH	gldm	LowGrayLevelEmphasis	0.009267868
wavelet-LLH	glcm	JointAverage	18.47111323
wavelet-LLH	glcm	SumAverage	36.94222647
wavelet-LLH	glcm	JointEntropy	7.827623145
wavelet-LLH	glcm	ClusterShade	168.5781053
wavelet-LLH	glcm	MaximumProbability	0.016232621
wavelet-LLH	glcm	Idmn	0.973141744

wavelet-LLH	glcm	JointEnergy	0.005678118
wavelet-LLH	glcm	Contrast	49.36584718
wavelet-LLH	glcm	DifferenceEntropy	3.743601849
wavelet-LLH	glcm	InverseVariance	0.210306735
wavelet-LLH	glcm	DifferenceVariance	21.95390314
wavelet-LLH	glcm	Idn	0.894874512
wavelet-LLH	glcm	Idm	0.195420276
wavelet-LLH	glcm	Correlation	0.157941806
wavelet-LLH	glcm	Autocorrelation	345.8477983
wavelet-LLH	glcm	SumEntropy	4.908386301
wavelet-LLH	glcm	SumSquares	29.34182536
wavelet-LLH	glcm	ClusterProminence	17766.32304
wavelet-LLH	glcm	Imc2	0.922633995
wavelet-LLH	glcm	Imc1	-0.219344642
wavelet-LLH	glcm	DifferenceAverage	5.131858997
wavelet-LLH	glcm	Id	0.285659324
wavelet-LLH	glcm	ClusterTendency	68.00145426
wavelet-LLH	firstorder	InterquartileRange	171.9375926
wavelet-LLH	firstorder	Skewness	0.233604498
wavelet-LLH	firstorder	Uniformity	0.052662435
wavelet-LLH	firstorder	Median	-42.83457871
wavelet-LLH	firstorder	Energy	8739182.149
wavelet-LLH	firstorder	RobustMeanAbsoluteDeviation	75.5563364
wavelet-LLH	firstorder	MeanAbsoluteDeviation	111.9387859
wavelet-LLH	firstorder	TotalEnergy	235957918
wavelet-LLH	firstorder	Maximum	476.436598
wavelet-LLH	firstorder	RootMeanSquared	151.0553142
wavelet-LLH	firstorder	90Percentile	136.7164811
wavelet-LLH	firstorder	Minimum	-479.0376452
wavelet-LLH	firstorder	Entropy	4.513105723
wavelet-LLH	firstorder	Range	955.4742432
wavelet-LLH	firstorder	Variance	20766.05485
wavelet-LLH	firstorder	10Percentile	-218.0729222
wavelet-LLH	firstorder	Kurtosis	3.498927231
wavelet-LLH	firstorder	Mean	-45.29517762
wavelet-LLH	glrlm	ShortRunLowGrayLevelEmphasis	0.009076619
wavelet-LLH	glrlm	GrayLevelVariance	34.6795248
wavelet-LLH	glrlm	LowGrayLevelRunEmphasis	0.009371741
wavelet-LLH	glrlm	GrayLevelNonUniformityNormalized	0.051414791
wavelet-LLH	glrlm	RunVariance	0.05998246
wavelet-LLH	glrlm	GrayLevelNonUniformity	18.67759146
wavelet-LLH	glrlm	LongRunEmphasis	1.172963457
wavelet-LLH	glrlm	ShortRunHighGrayLevelEmphasis	370.6724906
wavelet-LLH	glrlm	RunLengthNonUniformity	328.5392611
wavelet-LLH	glrlm	ShortRunEmphasis	0.961413465
wavelet-LLH	glrlm	LongRunHighGrayLevelEmphasis	444.9952262
wavelet-LLH	glrlm	RunPercentage	0.948383209
wavelet-LLH	glrlm	LongRunLowGrayLevelEmphasis	0.010610144
wavelet-LLH	glrlm	RunEntropy	4.779165593
wavelet-LLH	glrlm	HighGrayLevelRunEmphasis	384.2448333
wavelet-LLH	glrlm	RunLengthNonUniformityNormalized	0.903919169
wavelet-LLH	glszm	GrayLevelVariance	48.86005823

wavelet-LLH	glszm	ZoneVariance	4.448658816
wavelet-LLH	glszm	GrayLevelNonUniformityNormalized	0.040944111
wavelet-LLH	glszm	SizeZoneNonUniformityNormalized	0.444974104
wavelet-LLH	glszm	SizeZoneNonUniformity	87.65989848
wavelet-LLH	glszm	GrayLevelNonUniformity	8.065989848
wavelet-LLH	glszm	LargeAreaEmphasis	8.228426396
wavelet-LLH	glszm	SmallAreaHighGrayLevelEmphasis	304.8792435
wavelet-LLH	glszm	ZonePercentage	0.514360313
wavelet-LLH	glszm	LargeAreaLowGrayLevelEmphasis	0.04798401
wavelet-LLH	glszm	LargeAreaHighGrayLevelEmphasis	2985.025381
wavelet-LLH	glszm	HighGrayLevelZoneEmphasis	415.6700508
wavelet-LLH	glszm	SmallAreaEmphasis	0.693530126
wavelet-LLH	glszm	LowGrayLevelZoneEmphasis	0.009638066
wavelet-LLH	glszm	ZoneEntropy	6.008636497
wavelet-LLH	glszm	SmallAreaLowGrayLevelEmphasis	0.00469312
wavelet-LLH	ngtdm	Coarseness	0.01833718
wavelet-LLH	ngtdm	Complexity	1697.139078
wavelet-LLH	ngtdm	Strength	6.453902332
wavelet-LLH	ngtdm	Contrast	0.230204794
wavelet-LLH	ngtdm	Busyness	0.086850598
wavelet-LLL	gldm	GrayLevelVariance	629.1656089
wavelet-LLL	gldm	HighGrayLevelEmphasis	5329.467363
wavelet-LLL	gldm	DependenceEntropy	6.988372713
wavelet-LLL	gldm	DependenceNonUniformity	185.1462141
wavelet-LLL	gldm	GrayLevelNonUniformity	5.851174935
wavelet-LLL	gldm	SmallDependenceEmphasis	0.718185319
wavelet-LLL	gldm	SmallDependenceHighGrayLevelEmphasis	3243.657427
wavelet-LLL	gldm	DependenceNonUniformityNormalized	0.483410481
wavelet-LLL	gldm	LargeDependenceEmphasis	4.315926893
wavelet-LLL	gldm	LargeDependenceLowGrayLevelEmphasis	0.003580674
wavelet-LLL	gldm	DependenceVariance	1.686956759
wavelet-LLL	gldm	LargeDependenceHighGrayLevelEmphasis	34759.08616
wavelet-LLL	gldm	SmallDependenceLowGrayLevelEmphasis	0.00304751
wavelet-LLL	gldm	LowGrayLevelEmphasis	0.003106154
wavelet-LLL	glcm	JointAverage	74.40740235
wavelet-LLL	glcm	SumAverage	148.8148047
wavelet-LLL	glcm	JointEntropy	8.856732466
wavelet-LLL	glcm	ClusterShade	-39065.27488
wavelet-LLL	glcm	MaximumProbability	0.011384428
wavelet-LLL	glcm	Idmn	0.962835537
wavelet-LLL	glcm	JointEnergy	0.002416703
wavelet-LLL	glcm	Contrast	463.6183607
wavelet-LLL	glcm	DifferenceEntropy	5.25288102
wavelet-LLL	glcm	InverseVariance	0.08504197
wavelet-LLL	glcm	DifferenceVariance	187.0822279
wavelet-LLL	glcm	Idn	0.87584541
wavelet-LLL	glcm	Idm	0.08473227
wavelet-LLL	glcm	Correlation	0.579819055
wavelet-LLL	glcm	Autocorrelation	5862.232657
wavelet-LLL	glcm	SumEntropy	6.678123018
wavelet-LLL	glcm	SumSquares	556.3927072
wavelet-LLL	glcm	ClusterProminence	7319626.447

wavelet-LLL	glcm	Imc2	0.999300566
wavelet-LLL	glcm	Imc1	-0.541767466
wavelet-LLL	glcm	DifferenceAverage	16.3504778
wavelet-LLL	glcm	Id	0.150692623
wavelet-LLL	glcm	ClusterTendency	1761.952468
wavelet-LLL	firstorder	InterquartileRange	1112.655838
wavelet-LLL	firstorder	Skewness	-0.351002854
wavelet-LLL	firstorder	Uniformity	0.015277219
wavelet-LLL	firstorder	Median	-637.9026481
wavelet-LLL	firstorder	Energy	324236308.2
wavelet-LLL	firstorder	RobustMeanAbsoluteDeviation	424.5964339
wavelet-LLL	firstorder	MeanAbsoluteDeviation	538.1143526
wavelet-LLL	firstorder	TotalEnergy	8754380322
wavelet-LLL	firstorder	Maximum	238.6988349
wavelet-LLL	firstorder	RootMeanSquared	920.0923841
wavelet-LLL	firstorder	90Percentile	99.2661003
wavelet-LLL	firstorder	Minimum	-2350.774437
wavelet-LLL	firstorder	Entropy	6.259512248
wavelet-LLL	firstorder	Range	2589.473271
wavelet-LLL	firstorder	Variance	392772.1962
wavelet-LLL	firstorder	10Percentile	-1539.210951
wavelet-LLL	firstorder	Kurtosis	2.085252785
wavelet-LLL	firstorder	Mean	-673.6451582
wavelet-LLL	glrlm	ShortRunLowGrayLevelEmphasis	0.003175682
wavelet-LLL	glrlm	GrayLevelVariance	625.5786959
wavelet-LLL	glrlm	LowGrayLevelRunEmphasis	0.003178612
wavelet-LLL	glrlm	GrayLevelNonUniformityNormalized	0.014860584
wavelet-LLL	glrlm	RunVariance	0.028901317
wavelet-LLL	glrlm	GrayLevelNonUniformity	5.556454143
wavelet-LLL	glrlm	LongRunEmphasis	1.079105584
wavelet-LLL	glrlm	ShortRunHighGrayLevelEmphasis	5118.758589
wavelet-LLL	glrlm	RunLengthNonUniformity	357.7948947
wavelet-LLL	glrlm	ShortRunEmphasis	0.983019225
wavelet-LLL	glrlm	LongRunHighGrayLevelEmphasis	5912.716552
wavelet-LLL	glrlm	RunPercentage	0.976099618
wavelet-LLL	glrlm	LongRunLowGrayLevelEmphasis	0.003192132
wavelet-LLL	glrlm	RunEntropy	6.356105777
wavelet-LLL	glrlm	HighGrayLevelRunEmphasis	5257.634252
wavelet-LLL	glrlm	RunLengthNonUniformityNormalized	0.956759663
wavelet-LLL	glszm	GrayLevelVariance	593.1431192
wavelet-LLL	glszm	ZoneVariance	0.951175043
wavelet-LLL	glszm	GrayLevelNonUniformityNormalized	0.013389112
wavelet-LLL	glszm	SizeZoneNonUniformityNormalized	0.709488708
wavelet-LLL	glszm	SizeZoneNonUniformity	212.1371237
wavelet-LLL	glszm	GrayLevelNonUniformity	4.003344482
wavelet-LLL	glszm	LargeAreaEmphasis	2.591973244
wavelet-LLL	glszm	SmallAreaHighGrayLevelEmphasis	3791.153501
wavelet-LLL	glszm	ZonePercentage	0.780678851
wavelet-LLL	glszm	LargeAreaLowGrayLevelEmphasis	0.004178574
wavelet-LLL	glszm	LargeAreaHighGrayLevelEmphasis	18168.04013
wavelet-LLL	glszm	HighGrayLevelZoneEmphasis	4697.280936
wavelet-LLL	glszm	SmallAreaEmphasis	0.867071304

wavelet-LLL	glszm	LowGrayLevelZoneEmphasis	0.003922614
wavelet-LLL	glszm	ZoneEntropy	6.80075893
wavelet-LLL	glszm	SmallAreaLowGrayLevelEmphasis	0.003891454
wavelet-LLL	ngtdm	Coarseness	0.018582396
wavelet-LLL	ngtdm	Complexity	33532.47174
wavelet-LLL	ngtdm	Strength	68.00575951
wavelet-LLL	ngtdm	Contrast	1.556801145
wavelet-LLL	ngtdm	Busyness	0.008999923
wavelet-HHL	gldm	GrayLevelVariance	17.30365603
wavelet-HHL	gldm	HighGrayLevelEmphasis	368.156658
wavelet-HHL	gldm	DependenceEntropy	6.040541326
wavelet-HHL	gldm	DependenceNonUniformity	76.94778068
wavelet-HHL	gldm	GrayLevelNonUniformity	31.60574413
wavelet-HHL	gldm	SmallDependenceEmphasis	0.416762102
wavelet-HHL	gldm	SmallDependenceHighGrayLevelEmphasis	157.710626
wavelet-HHL	gldm	DependenceNonUniformityNormalized	0.200908044
wavelet-HHL	gldm	LargeDependenceEmphasis	13.66318538
wavelet-HHL	gldm	LargeDependenceLowGrayLevelEmphasis	0.0444387
wavelet-HHL	gldm	DependenceVariance	5.004533401
wavelet-HHL	gldm	LargeDependenceHighGrayLevelEmphasis	4726.955614
wavelet-HHL	gldm	SmallDependenceLowGrayLevelEmphasis	0.004470835
wavelet-HHL	gldm	LowGrayLevelEmphasis	0.006212057
wavelet-HHL	glcm	JointAverage	18.66837259
wavelet-HHL	glcm	SumAverage	37.33674519
wavelet-HHL	glcm	JointEntropy	7.096709348
wavelet-HHL	glcm	ClusterShade	-0.329141866
wavelet-HHL	glcm	MaximumProbability	0.031444402
wavelet-HHL	glcm	Idmn	0.972059809
wavelet-HHL	glcm	JointEnergy	0.01056269
wavelet-HHL	glcm	Contrast	33.26684017
wavelet-HHL	glcm	DifferenceEntropy	3.483232769
wavelet-HHL	glcm	InverseVariance	0.249128001
wavelet-HHL	glcm	DifferenceVariance	15.97612547
wavelet-HHL	glcm	Idn	0.896030189
wavelet-HHL	glcm	Idm	0.249217595
wavelet-HHL	glcm	Correlation	-0.016000155
wavelet-HHL	glcm	Autocorrelation	348.1618949
wavelet-HHL	glcm	SumEntropy	4.329587092
wavelet-HHL	glcm	SumSquares	16.28008147
wavelet-HHL	glcm	ClusterProminence	5464.12202
wavelet-HHL	glcm	Imc2	0.852710988
wavelet-HHL	glcm	Imc1	-0.170281804
wavelet-HHL	glcm	DifferenceAverage	4.096548576
wavelet-HHL	glcm	Id	0.336561934
wavelet-HHL	glcm	ClusterTendency	31.8534857
wavelet-HHL	firstorder	InterquartileRange	107.4916215
wavelet-HHL	firstorder	Skewness	-0.027505102
wavelet-HHL	firstorder	Uniformity	0.082521525
wavelet-HHL	firstorder	Median	1.081527419
wavelet-HHL	firstorder	Energy	4148808.665
wavelet-HHL	firstorder	RobustMeanAbsoluteDeviation	46.92202199
wavelet-HHL	firstorder	MeanAbsoluteDeviation	76.19027294

wavelet-HHL	firstorder	TotalEnergy	112017834
wavelet-HHL	firstorder	Maximum	327.0871994
wavelet-HHL	firstorder	RootMeanSquared	104.0788096
wavelet-HHL	firstorder	90Percentile	129.6539125
wavelet-HHL	firstorder	Minimum	-433.5068442
wavelet-HHL	firstorder	Entropy	3.960090691
wavelet-HHL	firstorder	Range	760.5940436
wavelet-HHL	firstorder	Variance	10797.07224
wavelet-HHL	firstorder	10Percentile	-104.3859171
wavelet-HHL	firstorder	Kurtosis	4.673149247
wavelet-HHL	firstorder	Mean	5.9435986
wavelet-HHL	glrlm	ShortRunLowGrayLevelEmphasis	0.006307327
wavelet-HHL	glrlm	GrayLevelVariance	18.30096863
wavelet-HHL	glrlm	LowGrayLevelRunEmphasis	0.006473907
wavelet-HHL	glrlm	GrayLevelNonUniformityNormalized	0.078579923
wavelet-HHL	glrlm	RunVariance	0.092732753
wavelet-HHL	glrlm	GrayLevelNonUniformity	27.85248339
wavelet-HHL	glrlm	LongRunEmphasis	1.262505917
wavelet-HHL	glrlm	ShortRunHighGrayLevelEmphasis	349.6405734
wavelet-HHL	glrlm	RunLengthNonUniformity	306.4440365
wavelet-HHL	glrlm	ShortRunEmphasis	0.944129683
wavelet-HHL	glrlm	LongRunHighGrayLevelEmphasis	461.4255917
wavelet-HHL	glrlm	RunPercentage	0.925286202
wavelet-HHL	glrlm	LongRunLowGrayLevelEmphasis	0.007261413
wavelet-HHL	glrlm	RunEntropy	4.351165914
wavelet-HHL	glrlm	HighGrayLevelRunEmphasis	369.4442343
wavelet-HHL	glrlm	RunLengthNonUniformityNormalized	0.86406241
wavelet-HHL	glszm	GrayLevelVariance	31.10495537
wavelet-HHL	glszm	ZoneVariance	23.83140111
wavelet-HHL	glszm	GrayLevelNonUniformityNormalized	0.058240142
wavelet-HHL	glszm	SizeZoneNonUniformityNormalized	0.577511029
wavelet-HHL	glszm	SizeZoneNonUniformity	98.75438596
wavelet-HHL	glszm	GrayLevelNonUniformity	9.959064327
wavelet-HHL	glszm	LargeAreaEmphasis	28.84795322
wavelet-HHL	glszm	SmallAreaHighGrayLevelEmphasis	298.868343
wavelet-HHL	glszm	ZonePercentage	0.446475196
wavelet-HHL	glszm	LargeAreaLowGrayLevelEmphasis	0.0948144
wavelet-HHL	glszm	LargeAreaHighGrayLevelEmphasis	9750.643275
wavelet-HHL	glszm	HighGrayLevelZoneEmphasis	378.8947368
wavelet-HHL	glszm	SmallAreaEmphasis	0.785182894
wavelet-HHL	glszm	LowGrayLevelZoneEmphasis	0.010233387
wavelet-HHL	glszm	ZoneEntropy	5.327434993
wavelet-HHL	glszm	SmallAreaLowGrayLevelEmphasis	0.009571389
wavelet-HHL	ngtdm	Coarseness	0.018492476
wavelet-HHL	ngtdm	Complexity	956.2485781
wavelet-HHL	ngtdm	Strength	4.304376573
wavelet-HHL	ngtdm	Contrast	0.140183329
wavelet-HHL	ngtdm	Busyness	0.087381342
wavelet-HLH	gldm	GrayLevelVariance	5.884094922
wavelet-HLH	gldm	HighGrayLevelEmphasis	64.04960836
wavelet-HLH	gldm	DependenceEntropy	5.704973539
wavelet-HLH	gldm	DependenceNonUniformity	59.52741514

wavelet-HLH	gldm	GrayLevelNonUniformity	44.84856397
wavelet-HLH	gldm	SmallDependenceEmphasis	0.264833267
wavelet-HLH	gldm	SmallDependenceHighGrayLevelEmphasis	20.01741772
wavelet-HLH	gldm	DependenceNonUniformityNormalized	0.15542406
wavelet-HLH	gldm	LargeDependenceEmphasis	16.27415144
wavelet-HLH	gldm	LargeDependenceLowGrayLevelEmphasis	0.354333591
wavelet-HLH	gldm	DependenceVariance	4.124467411
wavelet-HLH	gldm	LargeDependenceHighGrayLevelEmphasis	913.2663185
wavelet-HLH	gldm	SmallDependenceLowGrayLevelEmphasis	0.015577164
wavelet-HLH	gldm	LowGrayLevelEmphasis	0.031744184
wavelet-HLH	glcm	JointAverage	7.600946658
wavelet-HLH	glcm	SumAverage	15.20189332
wavelet-HLH	glcm	JointEntropy	6.24416465
wavelet-HLH	glcm	ClusterShade	5.150941648
wavelet-HLH	glcm	MaximumProbability	0.040797949
wavelet-HLH	glcm	Idmn	0.957795833
wavelet-HLH	glcm	JointEnergy	0.017112001
wavelet-HLH	glcm	Contrast	10.85848213
wavelet-HLH	glcm	DifferenceEntropy	2.842011708
wavelet-HLH	glcm	InverseVariance	0.317493246
wavelet-HLH	glcm	DifferenceVariance	4.144824899
wavelet-HLH	glcm	Idn	0.864534603
wavelet-HLH	glcm	Idm	0.315760646
wavelet-HLH	glcm	Correlation	-0.007176979
wavelet-HLH	glcm	Autocorrelation	57.72063479
wavelet-HLH	glcm	SumEntropy	3.681186705
wavelet-HLH	glcm	SumSquares	5.373842196
wavelet-HLH	glcm	ClusterProminence	354.987328
wavelet-HLH	glcm	Imc2	0.610741868
wavelet-HLH	glcm	Imc1	-0.073154676
wavelet-HLH	glcm	DifferenceAverage	2.57044182
wavelet-HLH	glcm	Id	0.400462114
wavelet-HLH	glcm	ClusterTendency	10.63688666
wavelet-HLH	firstorder	InterquartileRange	80.674366
wavelet-HLH	firstorder	Skewness	0.146107428
wavelet-HLH	firstorder	Uniformity	0.117098078
wavelet-HLH	firstorder	Median	0.531692878
wavelet-HLH	firstorder	Energy	1410262.546
wavelet-HLH	firstorder	RobustMeanAbsoluteDeviation	33.6301653
wavelet-HLH	firstorder	MeanAbsoluteDeviation	48.3220186
wavelet-HLH	firstorder	TotalEnergy	38077088.75
wavelet-HLH	firstorder	Maximum	190.5743336
wavelet-HLH	firstorder	RootMeanSquared	60.68070238
wavelet-HLH	firstorder	90Percentile	79.42822378
wavelet-HLH	firstorder	Minimum	-164.4439856
wavelet-HLH	firstorder	Entropy	3.302919134
wavelet-HLH	firstorder	Range	355.0183191
wavelet-HLH	firstorder	Variance	3675.052658
wavelet-HLH	firstorder	10Percentile	-74.2845524
wavelet-HLH	firstorder	Kurtosis	3.00507471
wavelet-HLH	firstorder	Mean	2.663640892
wavelet-HLH	glrlm	ShortRunLowGrayLevelEmphasis	0.031311987

wavelet-HLH	glrlm	GrayLevelVariance	6.223108004
wavelet-HLH	glrlm	LowGrayLevelRunEmphasis	0.032847593
wavelet-HLH	glrlm	GrayLevelNonUniformityNormalized	0.113208095
wavelet-HLH	glrlm	RunVariance	0.120984224
wavelet-HLH	glrlm	GrayLevelNonUniformity	39.21620687
wavelet-HLH	glrlm	LongRunEmphasis	1.345233836
wavelet-HLH	glrlm	ShortRunHighGrayLevelEmphasis	60.60290448
wavelet-HLH	glrlm	RunLengthNonUniformity	287.1794446
wavelet-HLH	glrlm	ShortRunEmphasis	0.928003129
wavelet-HLH	glrlm	LongRunHighGrayLevelEmphasis	84.48932964
wavelet-HLH	glrlm	RunPercentage	0.904398474
wavelet-HLH	glrlm	LongRunLowGrayLevelEmphasis	0.040235316
wavelet-HLH	glrlm	RunEntropy	3.783767958
wavelet-HLH	glrlm	HighGrayLevelRunEmphasis	64.75437061
wavelet-HLH	glrlm	RunLengthNonUniformityNormalized	0.828534237
wavelet-HLH	glszm	GrayLevelVariance	12.30889656
wavelet-HLH	glszm	ZoneVariance	65.80944365
wavelet-HLH	glszm	GrayLevelNonUniformityNormalized	0.089638919
wavelet-HLH	glszm	SizeZoneNonUniformityNormalized	0.418230789
wavelet-HLH	glszm	SizeZoneNonUniformity	45.58715596
wavelet-HLH	glszm	GrayLevelNonUniformity	9.770642202
wavelet-HLH	glszm	LargeAreaEmphasis	78.1559633
wavelet-HLH	glszm	SmallAreaHighGrayLevelEmphasis	54.08580686
wavelet-HLH	glszm	ZonePercentage	0.2845953
wavelet-HLH	glszm	LargeAreaLowGrayLevelEmphasis	1.634354904
wavelet-HLH	glszm	LargeAreaHighGrayLevelEmphasis	4272.541284
wavelet-HLH	glszm	HighGrayLevelZoneEmphasis	75.43119266
wavelet-HLH	glszm	SmallAreaEmphasis	0.671597992
wavelet-HLH	glszm	LowGrayLevelZoneEmphasis	0.057150599
wavelet-HLH	glszm	ZoneEntropy	5.018082138
wavelet-HLH	glszm	SmallAreaLowGrayLevelEmphasis	0.048838327
wavelet-HLH	ngtdm	Coarseness	0.015659374
wavelet-HLH	ngtdm	Complexity	170.1744054
wavelet-HLH	ngtdm	Strength	0.983421424
wavelet-HLH	ngtdm	Contrast	0.110203956
wavelet-HLH	ngtdm	Busyness	0.563604794

Image type	Feature Class	Feature Name	4: RTSTRUCT: C99-DNU_segment_RUL GTV
general	info	BoundingBox	(0, 0, 1, 8, 8, 4)
general	info	EnabledImageTypes	{u'Original': {}, u'Wavelet': {}}
general	info	GeneralSettings	{'distances': [1], 'additionalInfo': True, 'force2D': False, 'interpolator': 'sitkBSpline', 'resampledPixelSpacing': [3.0], 'label': 1, 'normalizeScale': 1, 'normalize': False, 'force2Ddimension': 0, 'removeOutliers': None, 'minimumROISize': None, u'binWidth': 25.0, 'minimumROIDimensions': 1, u'correctMask': True, 'preCrop': False, u'symmetricalGLCM': True, 'resegmentRange': None, 'padDistance': 5}
general	info	ImageHash	11d74446fca7dd494ac9097143277cd3bef2b0f2
general	info	ImageSpacing	(0.9765625000000002, 0.9765625000000002, 2.0)
general	info	MaskHash	8add2e3aec4d7fb2d851ea015eb4644316fd5885
general	info	NumpyVersion	1.13.1
general	info	PyWaveletVersion	0.5.2
general	info	SimpleITKVersion	1.1.0
general	info	Version	1.3.0.post102+g1b0eb74
general	info	VolumeNum	1
general	info	VoxelNum	108
original	shape	Maximum3DDiameter	24.18677324
original	shape	Maximum2DDiameterSlice	24.18677324
original	shape	Sphericity	0.805724557
original	shape	MinorAxis	18.85452012
original	shape	Elongation	0.879326704
original	shape	SurfaceVolumeRatio	0.420115158
original	shape	Volume	2916
original	shape	MajorAxis	21.4419965
original	shape	SurfaceArea	1225.0558
original	shape	Flatness	0.506309523
original	shape	LeastAxis	10.85628703
original	shape	Maximum2DDiameterColumn	21.21320344
original	shape	Maximum2DDiameterRow	21.84032967
original	gldm	GrayLevelVariance	47.15046296
original	gldm	HighGrayLevelEmphasis	227.1574074
original	gldm	DependenceEntropy	5.403837474
original	gldm	DependenceNonUniformity	45.44444444
original	gldm	GrayLevelNonUniformity	5.314814815
original	gldm	SmallDependenceEmphasis	0.652584877
original	gldm	SmallDependenceHighGrayLevelEmphasis	158.9236754
original	gldm	DependenceNonUniformityNormalized	0.420781893
original	gldm	LargeDependenceEmphasis	3.111111111
original	gldm	LargeDependenceLowGrayLevelEmphasis	0.042071975
original	gldm	DependenceVariance	0.574759945
original	gldm	LargeDependenceHighGrayLevelEmphasis	631.8425926

original	gldm	SmallDependenceLowGrayLevelEmphasis	0.02359765
original	gldm	LowGrayLevelEmphasis	0.026113219
original	glcm	JointAverage	14.70621157
original	glcm	SumAverage	29.41242314
original	glcm	JointEntropy	6.65747825
original	glcm	ClusterShade	355.6410821
original	glcm	MaximumProbability	0.026176385
original	glcm	Idmn	0.940569046
original	glcm	JointEnergy	0.010871506
original	glcm	Contrast	78.14909683
original	glcm	DifferenceEntropy	3.888728682
original	glcm	InverseVariance	0.153150446
original	glcm	DifferenceVariance	29.54562628
original	glcm	Idn	0.841058997
original	glcm	Idm	0.133816074
original	glcm	Correlation	0.098740729
original	glcm	Autocorrelation	220.79793
original	glcm	SumEntropy	4.688476401
original	glcm	SumSquares	43.32961664
original	glcm	ClusterProminence	26090.04132
original	glcm	Imc2	0.993371551
original	glcm	Imc1	-0.493809344
original	glcm	DifferenceAverage	6.914112356
original	glcm	Id	0.223934011
original	glcm	ClusterTendency	95.16936972
original	firstorder	InterquartileRange	199.5
original	firstorder	Skewness	0.649277997
original	firstorder	Uniformity	0.049211248
original	firstorder	Median	-528
original	firstorder	Energy	30441681
original	firstorder	RobustMeanAbsoluteDeviation	92.66428855
original	firstorder	MeanAbsoluteDeviation	134.1815844
original	firstorder	TotalEnergy	821925387
original	firstorder	Maximum	-6
original	firstorder	RootMeanSquared	530.9118728
original	firstorder	90Percentile	-264.8
original	firstorder	Minimum	-816
original	firstorder	Entropy	4.571197806
original	firstorder	Range	810
original	firstorder	Variance	29333.24923
original	firstorder	10Percentile	-706
original	firstorder	Kurtosis	3.049486223
original	firstorder	Mean	-502.5277778
original	glrlm	ShortRunLowGrayLevelEmphasis	0.026419853
original	glrlm	GrayLevelVariance	47.84040685
original	glrlm	LowGrayLevelRunEmphasis	0.026551628
original	glrlm	GrayLevelNonUniformityNormalized	0.048693937
original	glrlm	RunVariance	0.022764876
original	glrlm	GrayLevelNonUniformity	5.139760908
original	glrlm	LongRunEmphasis	1.070551458
original	glrlm	ShortRunHighGrayLevelEmphasis	224.5638726
original	glrlm	RunLengthNonUniformity	100.7720127
original	glrlm	ShortRunEmphasis	0.982362135

original	glrlm	LongRunHighGrayLevelEmphasis	241.5687946
original	glrlm	RunPercentage	0.977207977
original	glrlm	LongRunLowGrayLevelEmphasis	0.027078726
original	glrlm	RunEntropy	4.664539512
original	glrlm	HighGrayLevelRunEmphasis	227.964857
original	glrlm	RunLengthNonUniformityNormalized	0.954470249
original	glszm	GrayLevelVariance	57.88655664
original	glszm	ZoneVariance	0.612081397
original	glszm	GrayLevelNonUniformityNormalized	0.042781606
original	glszm	SizeZoneNonUniformityNormalized	0.618330396
original	glszm	SizeZoneNonUniformity	48.84810127
original	glszm	GrayLevelNonUniformity	3.379746835
original	glszm	LargeAreaEmphasis	2.481012658
original	glszm	SmallAreaHighGrayLevelEmphasis	201.0047433
original	glszm	ZonePercentage	0.731481481
original	glszm	LargeAreaLowGrayLevelEmphasis	0.043397424
original	glszm	LargeAreaHighGrayLevelEmphasis	533.9113924
original	glszm	HighGrayLevelZoneEmphasis	237.5822785
original	glszm	SmallAreaEmphasis	0.813533755
original	glszm	LowGrayLevelZoneEmphasis	0.033078772
original	glszm	ZoneEntropy	5.25449147
original	glszm	SmallAreaLowGrayLevelEmphasis	0.031730151
original	ngtdm	Coarseness	0.047075928
original	ngtdm	Complexity	1787.913795
original	ngtdm	Strength	15.86148198
original	ngtdm	Contrast	0.537988497
original	ngtdm	Busyness	0.069667967
wavelet-HHH	gldm	GrayLevelVariance	30.88571674
wavelet-HHH	gldm	HighGrayLevelEmphasis	331.0092593
wavelet-HHH	gldm	DependenceEntropy	5.197683192
wavelet-HHH	gldm	DependenceNonUniformity	38.59259259
wavelet-HHH	gldm	GrayLevelNonUniformity	7.092592593
wavelet-HHH	gldm	SmallDependenceEmphasis	0.568207305
wavelet-HHH	gldm	SmallDependenceHighGrayLevelEmphasis	183.0807382
wavelet-HHH	gldm	DependenceNonUniformityNormalized	0.35733882
wavelet-HHH	gldm	LargeDependenceEmphasis	3.925925926
wavelet-HHH	gldm	LargeDependenceLowGrayLevelEmphasis	0.02929863
wavelet-HHH	gldm	DependenceVariance	0.765432099
wavelet-HHH	gldm	LargeDependenceHighGrayLevelEmphasis	1318.546296
wavelet-HHH	gldm	SmallDependenceLowGrayLevelEmphasis	0.01798821
wavelet-HHH	gldm	LowGrayLevelEmphasis	0.019480921
wavelet-HHH	glcm	JointAverage	17.47769937
wavelet-HHH	glcm	SumAverage	34.95539873
wavelet-HHH	glcm	JointEntropy	6.550390625
wavelet-HHH	glcm	ClusterShade	-289.6964348
wavelet-HHH	glcm	MaximumProbability	0.02668882
wavelet-HHH	glcm	Idmn	0.934935808
wavelet-HHH	glcm	JointEnergy	0.011908158
wavelet-HHH	glcm	Contrast	64.35598553
wavelet-HHH	glcm	DifferenceEntropy	3.716438975
wavelet-HHH	glcm	InverseVariance	0.161553622
wavelet-HHH	glcm	DifferenceVariance	25.5480148
wavelet-HHH	glcm	Idn	0.836031259

wavelet-HHH	glcm	Idm	0.147695935
wavelet-HHH	glcm	Correlation	-0.037688107
wavelet-HHH	glcm	Autocorrelation	304.3489141
wavelet-HHH	glcm	SumEntropy	4.426274678
wavelet-HHH	glcm	SumSquares	31.02574113
wavelet-HHH	glcm	ClusterProminence	13893.64854
wavelet-HHH	glcm	Imc2	0.987486994
wavelet-HHH	glcm	Imc1	-0.445336695
wavelet-HHH	glcm	DifferenceAverage	6.156812454
wavelet-HHH	glcm	Id	0.241665679
wavelet-HHH	glcm	ClusterTendency	59.74697901
wavelet-HHH	firstorder	InterquartileRange	135.3754609
wavelet-HHH	firstorder	Skewness	-0.839531348
wavelet-HHH	firstorder	Uniformity	0.065672154
wavelet-HHH	firstorder	Median	5.445777029
wavelet-HHH	firstorder	Energy	2075450.972
wavelet-HHH	firstorder	RobustMeanAbsoluteDeviation	64.19258121
wavelet-HHH	firstorder	MeanAbsoluteDeviation	102.9773188
wavelet-HHH	firstorder	TotalEnergy	56037176.24
wavelet-HHH	firstorder	Maximum	259.3367368
wavelet-HHH	firstorder	RootMeanSquared	138.6258945
wavelet-HHH	firstorder	90Percentile	151.4555611
wavelet-HHH	firstorder	Minimum	-401.9561848
wavelet-HHH	firstorder	Entropy	4.236815407
wavelet-HHH	firstorder	Range	661.2929215
wavelet-HHH	firstorder	Variance	19191.42175
wavelet-HHH	firstorder	10Percentile	-193.2543972
wavelet-HHH	firstorder	Kurtosis	3.750270495
wavelet-HHH	firstorder	Mean	-5.07118138
wavelet-HHH	glrlm	ShortRunLowGrayLevelEmphasis	0.019916518
wavelet-HHH	glrlm	GrayLevelVariance	31.56538303
wavelet-HHH	glrlm	LowGrayLevelRunEmphasis	0.019989023
wavelet-HHH	glrlm	GrayLevelNonUniformityNormalized	0.064784595
wavelet-HHH	glrlm	RunVariance	0.037438422
wavelet-HHH	glrlm	GrayLevelNonUniformity	6.789981164
wavelet-HHH	glrlm	LongRunEmphasis	1.102385525
wavelet-HHH	glrlm	ShortRunHighGrayLevelEmphasis	323.4432109
wavelet-HHH	glrlm	RunLengthNonUniformity	99.31398169
wavelet-HHH	glrlm	ShortRunEmphasis	0.978643274
wavelet-HHH	glrlm	LongRunHighGrayLevelEmphasis	365.4083769
wavelet-HHH	glrlm	RunPercentage	0.97008547
wavelet-HHH	glrlm	LongRunLowGrayLevelEmphasis	0.020336442
wavelet-HHH	glrlm	RunEntropy	4.361978396
wavelet-HHH	glrlm	HighGrayLevelRunEmphasis	330.733047
wavelet-HHH	glrlm	RunLengthNonUniformityNormalized	0.946789327
wavelet-HHH	glszm	GrayLevelVariance	41.4449514
wavelet-HHH	glszm	ZoneVariance	1.094624083
wavelet-HHH	glszm	GrayLevelNonUniformityNormalized	0.054949415
wavelet-HHH	glszm	SizeZoneNonUniformityNormalized	0.532235668
wavelet-HHH	glszm	SizeZoneNonUniformity	37.78873239
wavelet-HHH	glszm	GrayLevelNonUniformity	3.901408451
wavelet-HHH	glszm	LargeAreaEmphasis	3.408450704
wavelet-HHH	glszm	SmallAreaHighGrayLevelEmphasis	241.8292553

wavelet-HHH	glszm	ZonePercentage	0.657407407
wavelet-HHH	glszm	LargeAreaLowGrayLevelEmphasis	0.036071873
wavelet-HHH	glszm	LargeAreaHighGrayLevelEmphasis	1126.042254
wavelet-HHH	glszm	HighGrayLevelZoneEmphasis	325.6760563
wavelet-HHH	glszm	SmallAreaEmphasis	0.757599645
wavelet-HHH	glszm	LowGrayLevelZoneEmphasis	0.027846077
wavelet-HHH	glszm	ZoneEntropy	5.161874366
wavelet-HHH	glszm	SmallAreaLowGrayLevelEmphasis	0.026990528
wavelet-HHH	ngtdm	Coarseness	0.050713792
wavelet-HHH	ngtdm	Complexity	1226.042596
wavelet-HHH	ngtdm	Strength	11.48983744
wavelet-HHH	ngtdm	Contrast	0.387505683
wavelet-HHH	ngtdm	Busyness	0.039046538
wavelet-HLL	gldm	GrayLevelVariance	86.78626543
wavelet-HLL	gldm	HighGrayLevelEmphasis	832.3796296
wavelet-HLL	gldm	DependenceEntropy	5.756976044
wavelet-HLL	gldm	DependenceNonUniformity	39.07407407
wavelet-HLL	gldm	GrayLevelNonUniformity	4.111111111
wavelet-HLL	gldm	SmallDependenceEmphasis	0.576195988
wavelet-HLL	gldm	SmallDependenceHighGrayLevelEmphasis	414.5467464
wavelet-HLL	gldm	DependenceNonUniformityNormalized	0.361796982
wavelet-HLL	gldm	LargeDependenceEmphasis	3.833333333
wavelet-HLL	gldm	LargeDependenceLowGrayLevelEmphasis	0.079750108
wavelet-HLL	gldm	DependenceVariance	0.738340192
wavelet-HLL	gldm	LargeDependenceHighGrayLevelEmphasis	3793.787037
wavelet-HLL	gldm	SmallDependenceLowGrayLevelEmphasis	0.006779501
wavelet-HLL	gldm	LowGrayLevelEmphasis	0.021130897
wavelet-HLL	glcm	JointAverage	27.2626055
wavelet-HLL	glcm	SumAverage	54.525211
wavelet-HLL	glcm	JointEntropy	6.756345836
wavelet-HLL	glcm	ClusterShade	-1270.356173
wavelet-HLL	glcm	MaximumProbability	0.021148397
wavelet-HLL	glcm	Idmn	0.944052262
wavelet-HLL	glcm	JointEnergy	0.009832025
wavelet-HLL	glcm	Contrast	125.482999
wavelet-HLL	glcm	DifferenceEntropy	4.044854068
wavelet-HLL	glcm	InverseVariance	0.153579157
wavelet-HLL	glcm	DifferenceVariance	54.20567272
wavelet-HLL	glcm	Idn	0.853323187
wavelet-HLL	glcm	Idm	0.140653536
wavelet-HLL	glcm	Correlation	0.200902598
wavelet-HLL	glcm	Autocorrelation	759.5393956
wavelet-HLL	glcm	SumEntropy	4.971298142
wavelet-HLL	glcm	SumSquares	78.93230278
wavelet-HLL	glcm	ClusterProminence	112565.4933
wavelet-HLL	glcm	Imc2	0.997762987
wavelet-HLL	glcm	Imc1	-0.575877807
wavelet-HLL	glcm	DifferenceAverage	8.189317574
wavelet-HLL	glcm	Id	0.223663247
wavelet-HLL	glcm	ClusterTendency	190.2462121
wavelet-HLL	firstorder	InterquartileRange	300.1924268
wavelet-HLL	firstorder	Skewness	-0.830739324
wavelet-HLL	firstorder	Uniformity	0.038065844

wavelet-HLL	firstorder	Median	19.81347538
wavelet-HLL	firstorder	Energy	5983893.314
wavelet-HLL	firstorder	RobustMeanAbsoluteDeviation	126.8977312
wavelet-HLL	firstorder	MeanAbsoluteDeviation	186.1879831
wavelet-HLL	firstorder	TotalEnergy	161565119.5
wavelet-HLL	firstorder	Maximum	328.3418901
wavelet-HLL	firstorder	RootMeanSquared	235.3856826
wavelet-HLL	firstorder	90Percentile	247.1766429
wavelet-HLL	firstorder	Minimum	-692.2047207
wavelet-HLL	firstorder	Entropy	4.902079032
wavelet-HLL	firstorder	Range	1020.546611
wavelet-HLL	firstorder	Variance	54507.31487
wavelet-HLL	firstorder	10Percentile	-376.5254512
wavelet-HLL	firstorder	Kurtosis	3.182376125
wavelet-HLL	firstorder	Mean	-29.98507462
wavelet-HLL	glrlm	ShortRunLowGrayLevelEmphasis	0.02037534
wavelet-HLL	glrlm	GrayLevelVariance	87.23390053
wavelet-HLL	glrlm	LowGrayLevelRunEmphasis	0.020974853
wavelet-HLL	glrlm	GrayLevelNonUniformityNormalized	0.037883275
wavelet-HLL	glrlm	RunVariance	0.035174027
wavelet-HLL	glrlm	GrayLevelNonUniformity	3.972143408
wavelet-HLL	glrlm	LongRunEmphasis	1.097814383
wavelet-HLL	glrlm	ShortRunHighGrayLevelEmphasis	805.0018881
wavelet-HLL	glrlm	RunLengthNonUniformity	99.36657711
wavelet-HLL	glrlm	ShortRunEmphasis	0.97890744
wavelet-HLL	glrlm	LongRunHighGrayLevelEmphasis	926.4399884
wavelet-HLL	glrlm	RunPercentage	0.970797721
wavelet-HLL	glrlm	LongRunLowGrayLevelEmphasis	0.023389534
wavelet-HLL	glrlm	RunEntropy	4.990222858
wavelet-HLL	glrlm	HighGrayLevelRunEmphasis	826.5842846
wavelet-HLL	glrlm	RunLengthNonUniformityNormalized	0.946945662
wavelet-HLL	glszm	GrayLevelVariance	92.99305556
wavelet-HLL	glszm	ZoneVariance	0.833333333
wavelet-HLL	glszm	GrayLevelNonUniformityNormalized	0.035108025
wavelet-HLL	glszm	SizeZoneNonUniformityNormalized	0.533950617
wavelet-HLL	glszm	SizeZoneNonUniformity	38.44444444
wavelet-HLL	glszm	GrayLevelNonUniformity	2.527777778
wavelet-HLL	glszm	LargeAreaEmphasis	3.083333333
wavelet-HLL	glszm	SmallAreaHighGrayLevelEmphasis	518.5260417
wavelet-HLL	glszm	ZonePercentage	0.666666667
wavelet-HLL	glszm	LargeAreaLowGrayLevelEmphasis	0.061187261
wavelet-HLL	glszm	LargeAreaHighGrayLevelEmphasis	2890.041667
wavelet-HLL	glszm	HighGrayLevelZoneEmphasis	747.5
wavelet-HLL	glszm	SmallAreaEmphasis	0.758946759
wavelet-HLL	glszm	LowGrayLevelZoneEmphasis	0.017263897
wavelet-HLL	glszm	ZoneEntropy	5.489097918
wavelet-HLL	glszm	SmallAreaLowGrayLevelEmphasis	0.006582877
wavelet-HLL	ngtdm	Coarseness	0.053721435
wavelet-HLL	ngtdm	Complexity	2937.740358
wavelet-HLL	ngtdm	Strength	28.19577266
wavelet-HLL	ngtdm	Contrast	0.755915707
wavelet-HLL	ngtdm	Busyness	0.019984996
wavelet-LHL	gldm	GrayLevelVariance	29.60459534

wavelet-LHL	gldm	HighGrayLevelEmphasis	245.2592593
wavelet-LHL	gldm	DependenceEntropy	5.303867738
wavelet-LHL	gldm	DependenceNonUniformity	32.18518519
wavelet-LHL	gldm	GrayLevelNonUniformity	6.833333333
wavelet-LHL	gldm	SmallDependenceEmphasis	0.527651749
wavelet-LHL	gldm	SmallDependenceHighGrayLevelEmphasis	136.2878704
wavelet-LHL	gldm	DependenceNonUniformityNormalized	0.298010974
wavelet-LHL	gldm	LargeDependenceEmphasis	5.481481481
wavelet-LHL	gldm	LargeDependenceLowGrayLevelEmphasis	0.052039356
wavelet-LHL	gldm	DependenceVariance	1.331961591
wavelet-LHL	gldm	LargeDependenceHighGrayLevelEmphasis	1353.759259
wavelet-LHL	gldm	SmallDependenceLowGrayLevelEmphasis	0.024810801
wavelet-LHL	gldm	LowGrayLevelEmphasis	0.02804593
wavelet-LHL	glcm	JointAverage	14.48135102
wavelet-LHL	glcm	SumAverage	28.96270204
wavelet-LHL	glcm	JointEntropy	6.516253317
wavelet-LHL	glcm	ClusterShade	-135.9886187
wavelet-LHL	glcm	MaximumProbability	0.033878918
wavelet-LHL	glcm	Idmn	0.941093371
wavelet-LHL	glcm	JointEnergy	0.012341498
wavelet-LHL	glcm	Contrast	56.422569
wavelet-LHL	glcm	DifferenceEntropy	3.722722409
wavelet-LHL	glcm	InverseVariance	0.174863879
wavelet-LHL	glcm	DifferenceVariance	22.41683032
wavelet-LHL	glcm	Idn	0.844231506
wavelet-LHL	glcm	Idm	0.175940233
wavelet-LHL	glcm	Correlation	-0.001828624
wavelet-LHL	glcm	Autocorrelation	209.7394102
wavelet-LHL	glcm	SumEntropy	4.458465845
wavelet-LHL	glcm	SumSquares	28.23251115
wavelet-LHL	glcm	ClusterProminence	10957.08325
wavelet-LHL	glcm	Imc2	0.985436781
wavelet-LHL	glcm	Imc1	-0.433189015
wavelet-LHL	glcm	DifferenceAverage	5.769776669
wavelet-LHL	glcm	Id	0.265681146
wavelet-LHL	glcm	ClusterTendency	56.50747561
wavelet-LHL	firstorder	InterquartileRange	166.541338
wavelet-LHL	firstorder	Skewness	-0.316078308
wavelet-LHL	firstorder	Uniformity	0.063271605
wavelet-LHL	firstorder	Median	-33.75123629
wavelet-LHL	firstorder	Energy	2249095.837
wavelet-LHL	firstorder	RobustMeanAbsoluteDeviation	67.45728658
wavelet-LHL	firstorder	MeanAbsoluteDeviation	105.1172057
wavelet-LHL	firstorder	TotalEnergy	60725587.6
wavelet-LHL	firstorder	Maximum	295.9062397
wavelet-LHL	firstorder	RootMeanSquared	144.3085633
wavelet-LHL	firstorder	90Percentile	89.2353182
wavelet-LHL	firstorder	Minimum	-393.4976701
wavelet-LHL	firstorder	Entropy	4.24375376
wavelet-LHL	firstorder	Range	689.4039098
wavelet-LHL	firstorder	Variance	18737.17069
wavelet-LHL	firstorder	10Percentile	-234.4430595
wavelet-LHL	firstorder	Kurtosis	3.279029358

wavelet-LHL	firstorder	Mean	-45.6923491
wavelet-LHL	glrlm	ShortRunLowGrayLevelEmphasis	0.028807352
wavelet-LHL	glrlm	GrayLevelVariance	30.37799495
wavelet-LHL	glrlm	LowGrayLevelRunEmphasis	0.02898022
wavelet-LHL	glrlm	GrayLevelNonUniformityNormalized	0.062484048
wavelet-LHL	glrlm	RunVariance	0.04741959
wavelet-LHL	glrlm	GrayLevelNonUniformity	6.479608867
wavelet-LHL	glrlm	LongRunEmphasis	1.134031261
wavelet-LHL	glrlm	ShortRunHighGrayLevelEmphasis	238.3749182
wavelet-LHL	glrlm	RunLengthNonUniformity	96.18984325
wavelet-LHL	glrlm	ShortRunEmphasis	0.970757568
wavelet-LHL	glrlm	LongRunHighGrayLevelEmphasis	277.5576638
wavelet-LHL	glrlm	RunPercentage	0.96011396
wavelet-LHL	glrlm	LongRunLowGrayLevelEmphasis	0.02976144
wavelet-LHL	glrlm	RunEntropy	4.398117024
wavelet-LHL	glrlm	HighGrayLevelRunEmphasis	245.4553644
wavelet-LHL	glrlm	RunLengthNonUniformityNormalized	0.926810709
wavelet-LHL	glszm	GrayLevelVariance	41.58764648
wavelet-LHL	glszm	ZoneVariance	2.46484375
wavelet-LHL	glszm	GrayLevelNonUniformityNormalized	0.052734375
wavelet-LHL	glszm	SizeZoneNonUniformityNormalized	0.559570313
wavelet-LHL	glszm	SizeZoneNonUniformity	35.8125
wavelet-LHL	glszm	GrayLevelNonUniformity	3.375
wavelet-LHL	glszm	LargeAreaEmphasis	5.3125
wavelet-LHL	glszm	SmallAreaHighGrayLevelEmphasis	203.638303
wavelet-LHL	glszm	ZonePercentage	0.592592593
wavelet-LHL	glszm	LargeAreaLowGrayLevelEmphasis	0.066792637
wavelet-LHL	glszm	LargeAreaHighGrayLevelEmphasis	1257.90625
wavelet-LHL	glszm	HighGrayLevelZoneEmphasis	254.109375
wavelet-LHL	glszm	SmallAreaEmphasis	0.773515625
wavelet-LHL	glszm	LowGrayLevelZoneEmphasis	0.042937265
wavelet-LHL	glszm	ZoneEntropy	5.042434179
wavelet-LHL	glszm	SmallAreaLowGrayLevelEmphasis	0.041015621
wavelet-LHL	ngtdm	Coarseness	0.044950483
wavelet-LHL	ngtdm	Complexity	1064.951528
wavelet-LHL	ngtdm	Strength	9.286308094
wavelet-LHL	ngtdm	Contrast	0.434903898
wavelet-LHL	ngtdm	Busyness	0.060820267
wavelet-LHH	gldm	GrayLevelVariance	28.41246571
wavelet-LHH	gldm	HighGrayLevelEmphasis	236.787037
wavelet-LHH	gldm	DependenceEntropy	5.151567914
wavelet-LHH	gldm	DependenceNonUniformity	39.05555556
wavelet-LHH	gldm	GrayLevelNonUniformity	6.722222222
wavelet-LHH	gldm	SmallDependenceEmphasis	0.603171296
wavelet-LHH	gldm	SmallDependenceHighGrayLevelEmphasis	163.3668673
wavelet-LHH	gldm	DependenceNonUniformityNormalized	0.361625514
wavelet-LHH	gldm	LargeDependenceEmphasis	4.222222222
wavelet-LHH	gldm	LargeDependenceLowGrayLevelEmphasis	0.03816091
wavelet-LHH	gldm	DependenceVariance	0.995541838
wavelet-LHH	gldm	LargeDependenceHighGrayLevelEmphasis	830.787037
wavelet-LHH	gldm	SmallDependenceLowGrayLevelEmphasis	0.013807864
wavelet-LHH	gldm	LowGrayLevelEmphasis	0.016521486
wavelet-LHH	glcm	JointAverage	14.55335681

wavelet-LHH	glcm	SumAverage	29.10671361
wavelet-LHH	glcm	JointEntropy	6.536671466
wavelet-LHH	glcm	ClusterShade	152.8596989
wavelet-LHH	glcm	MaximumProbability	0.024468411
wavelet-LHH	glcm	Idmn	0.951395101
wavelet-LHH	glcm	JointEnergy	0.011774602
wavelet-LHH	glcm	Contrast	61.91516995
wavelet-LHH	glcm	DifferenceEntropy	3.778994439
wavelet-LHH	glcm	InverseVariance	0.155634216
wavelet-LHH	glcm	DifferenceVariance	22.91679982
wavelet-LHH	glcm	Idn	0.853952712
wavelet-LHH	glcm	Idm	0.149218578
wavelet-LHH	glcm	Correlation	-0.049183083
wavelet-LHH	glcm	Autocorrelation	210.5078739
wavelet-LHH	glcm	SumEntropy	4.445999416
wavelet-LHH	glcm	SumSquares	29.64934417
wavelet-LHH	glcm	ClusterProminence	10423.88951
wavelet-LHH	glcm	Imc2	0.985651648
wavelet-LHH	glcm	Imc1	-0.430986748
wavelet-LHH	glcm	DifferenceAverage	6.184862167
wavelet-LHH	glcm	Id	0.241336511
wavelet-LHH	glcm	ClusterTendency	56.68220672
wavelet-LHH	firstorder	InterquartileRange	184.2536154
wavelet-LHH	firstorder	Skewness	0.44202462
wavelet-LHH	firstorder	Uniformity	0.062242798
wavelet-LHH	firstorder	Median	-17.67923385
wavelet-LHH	firstorder	Energy	1933172.585
wavelet-LHH	firstorder	RobustMeanAbsoluteDeviation	76.14499278
wavelet-LHH	firstorder	MeanAbsoluteDeviation	107.0172276
wavelet-LHH	firstorder	TotalEnergy	52195659.79
wavelet-LHH	firstorder	Maximum	468.2353464
wavelet-LHH	firstorder	RootMeanSquared	133.7899329
wavelet-LHH	firstorder	90Percentile	192.6846704
wavelet-LHH	firstorder	Minimum	-347.8061576
wavelet-LHH	firstorder	Entropy	4.205779407
wavelet-LHH	firstorder	Range	816.041504
wavelet-LHH	firstorder	Variance	17896.5454
wavelet-LHH	firstorder	10Percentile	-151.1434939
wavelet-LHH	firstorder	Kurtosis	3.458156741
wavelet-LHH	firstorder	Mean	-1.789066468
wavelet-LHH	glrlm	ShortRunLowGrayLevelEmphasis	0.016668796
wavelet-LHH	glrlm	GrayLevelVariance	28.92742106
wavelet-LHH	glrlm	LowGrayLevelRunEmphasis	0.016830761
wavelet-LHH	glrlm	GrayLevelNonUniformityNormalized	0.061353739
wavelet-LHH	glrlm	RunVariance	0.030574943
wavelet-LHH	glrlm	GrayLevelNonUniformity	6.423742308
wavelet-LHH	glrlm	LongRunEmphasis	1.095371956
wavelet-LHH	glrlm	ShortRunHighGrayLevelEmphasis	234.0678339
wavelet-LHH	glrlm	RunLengthNonUniformity	98.32831931
wavelet-LHH	glrlm	ShortRunEmphasis	0.976157011
wavelet-LHH	glrlm	LongRunHighGrayLevelEmphasis	256.0252998
wavelet-LHH	glrlm	RunPercentage	0.969373219
wavelet-LHH	glrlm	LongRunLowGrayLevelEmphasis	0.017478621

wavelet-LHH	glrlm	RunEntropy	4.343319938
wavelet-LHH	glrlm	HighGrayLevelRunEmphasis	238.4593271
wavelet-LHH	glrlm	RunLengthNonUniformityNormalized	0.938850114
wavelet-LHH	glszm	GrayLevelVariance	35.78626543
wavelet-LHH	glszm	ZoneVariance	1.388888889
wavelet-LHH	glszm	GrayLevelNonUniformityNormalized	0.057484568
wavelet-LHH	glszm	SizeZoneNonUniformityNormalized	0.61882716
wavelet-LHH	glszm	SizeZoneNonUniformity	44.55555556
wavelet-LHH	glszm	GrayLevelNonUniformity	4.138888889
wavelet-LHH	glszm	LargeAreaEmphasis	3.638888889
wavelet-LHH	glszm	SmallAreaHighGrayLevelEmphasis	228.5662506
wavelet-LHH	glszm	ZonePercentage	0.666666667
wavelet-LHH	glszm	LargeAreaLowGrayLevelEmphasis	0.03974623
wavelet-LHH	glszm	LargeAreaHighGrayLevelEmphasis	734.9027778
wavelet-LHH	glszm	HighGrayLevelZoneEmphasis	264.9722222
wavelet-LHH	glszm	SmallAreaEmphasis	0.811217089
wavelet-LHH	glszm	LowGrayLevelZoneEmphasis	0.021294259
wavelet-LHH	glszm	ZoneEntropy	4.800075134
wavelet-LHH	glszm	SmallAreaLowGrayLevelEmphasis	0.02004197
wavelet-LHH	ngtdm	Coarseness	0.040630983
wavelet-LHH	ngtdm	Complexity	985.6320576
wavelet-LHH	ngtdm	Strength	8.23836778
wavelet-LHH	ngtdm	Contrast	0.510294921
wavelet-LHH	ngtdm	Busyness	0.099463779
wavelet-LLH	gldm	GrayLevelVariance	42.07098765
wavelet-LLH	gldm	HighGrayLevelEmphasis	388.4444444
wavelet-LLH	gldm	DependenceEntropy	5.402912794
wavelet-LLH	gldm	DependenceNonUniformity	41.42592593
wavelet-LLH	gldm	GrayLevelNonUniformity	5.796296296
wavelet-LLH	gldm	SmallDependenceEmphasis	0.606610082
wavelet-LLH	gldm	SmallDependenceHighGrayLevelEmphasis	234.1395319
wavelet-LLH	gldm	DependenceNonUniformityNormalized	0.383573388
wavelet-LLH	gldm	LargeDependenceEmphasis	3.481481481
wavelet-LLH	gldm	LargeDependenceLowGrayLevelEmphasis	0.026995855
wavelet-LLH	gldm	DependenceVariance	0.641632373
wavelet-LLH	gldm	LargeDependenceHighGrayLevelEmphasis	1399.185185
wavelet-LLH	gldm	SmallDependenceLowGrayLevelEmphasis	0.012296103
wavelet-LLH	gldm	LowGrayLevelEmphasis	0.014440652
wavelet-LLH	glcm	JointAverage	17.94128741
wavelet-LLH	glcm	SumAverage	35.88257481
wavelet-LLH	glcm	JointEntropy	6.668618272
wavelet-LLH	glcm	ClusterShade	101.1578406
wavelet-LLH	glcm	MaximumProbability	0.023202544
wavelet-LLH	glcm	Idmn	0.942126569
wavelet-LLH	glcm	JointEnergy	0.010608246
wavelet-LLH	glcm	Contrast	78.56236556
wavelet-LLH	glcm	DifferenceEntropy	3.860803453
wavelet-LLH	glcm	InverseVariance	0.130185363
wavelet-LLH	glcm	DifferenceVariance	24.81965946
wavelet-LLH	glcm	Idn	0.838258534
wavelet-LLH	glcm	Idm	0.126153866
wavelet-LLH	glcm	Correlation	0.138643661
wavelet-LLH	glcm	Autocorrelation	328.4459916

wavelet-LLH	glcm	SumEntropy	4.731519184
wavelet-LLH	glcm	SumSquares	45.77692727
wavelet-LLH	glcm	ClusterProminence	34233.96009
wavelet-LLH	glcm	Imc2	0.994118589
wavelet-LLH	glcm	Imc1	-0.50508095
wavelet-LLH	glcm	DifferenceAverage	7.167921049
wavelet-LLH	glcm	Id	0.215022933
wavelet-LLH	glcm	ClusterTendency	104.5453435
wavelet-LLH	firstorder	InterquartileRange	204.3290552
wavelet-LLH	firstorder	Skewness	-0.126156915
wavelet-LLH	firstorder	Uniformity	0.05366941
wavelet-LLH	firstorder	Median	-13.13080217
wavelet-LLH	firstorder	Energy	2891439.205
wavelet-LLH	firstorder	RobustMeanAbsoluteDeviation	85.1678498
wavelet-LLH	firstorder	MeanAbsoluteDeviation	126.0925326
wavelet-LLH	firstorder	TotalEnergy	78068858.52
wavelet-LLH	firstorder	Maximum	371.2662019
wavelet-LLH	firstorder	RootMeanSquared	163.6233028
wavelet-LLH	firstorder	90Percentile	189.6068086
wavelet-LLH	firstorder	Minimum	-472.4845521
wavelet-LLH	firstorder	Entropy	4.47585975
wavelet-LLH	firstorder	Range	843.750754
wavelet-LLH	firstorder	Variance	26282.21188
wavelet-LLH	firstorder	10Percentile	-230.9346357
wavelet-LLH	firstorder	Kurtosis	2.977123507
wavelet-LLH	firstorder	Mean	-22.14437503
wavelet-LLH	glrlm	ShortRunLowGrayLevelEmphasis	0.014588803
wavelet-LLH	glrlm	GrayLevelVariance	42.12300249
wavelet-LLH	glrlm	LowGrayLevelRunEmphasis	0.014693909
wavelet-LLH	glrlm	GrayLevelNonUniformityNormalized	0.05328338
wavelet-LLH	glrlm	RunVariance	0.029302389
wavelet-LLH	glrlm	GrayLevelNonUniformity	5.604058741
wavelet-LLH	glrlm	LongRunEmphasis	1.085320522
wavelet-LLH	glrlm	ShortRunHighGrayLevelEmphasis	380.1669974
wavelet-LLH	glrlm	RunLengthNonUniformity	99.96308754
wavelet-LLH	glrlm	ShortRunEmphasis	0.980337936
wavelet-LLH	glrlm	LongRunHighGrayLevelEmphasis	422.5520459
wavelet-LLH	glrlm	RunPercentage	0.973646724
wavelet-LLH	glrlm	LongRunLowGrayLevelEmphasis	0.015129839
wavelet-LLH	glrlm	RunEntropy	4.56609105
wavelet-LLH	glrlm	HighGrayLevelRunEmphasis	388.0485351
wavelet-LLH	glrlm	RunLengthNonUniformityNormalized	0.950003297
wavelet-LLH	glszm	GrayLevelVariance	43.07306667
wavelet-LLH	glszm	ZoneVariance	0.619733333
wavelet-LLH	glszm	GrayLevelNonUniformityNormalized	0.048888889
wavelet-LLH	glszm	SizeZoneNonUniformityNormalized	0.570844444
wavelet-LLH	glszm	SizeZoneNonUniformity	42.81333333
wavelet-LLH	glszm	GrayLevelNonUniformity	3.666666667
wavelet-LLH	glszm	LargeAreaEmphasis	2.693333333
wavelet-LLH	glszm	SmallAreaHighGrayLevelEmphasis	301.2063889
wavelet-LLH	glszm	ZonePercentage	0.694444444
wavelet-LLH	glszm	LargeAreaLowGrayLevelEmphasis	0.02773803
wavelet-LLH	glszm	LargeAreaHighGrayLevelEmphasis	1050.346667

wavelet-LLH	glszm	HighGrayLevelZoneEmphasis	387.5466667
wavelet-LLH	glszm	SmallAreaEmphasis	0.77712963
wavelet-LLH	glszm	LowGrayLevelZoneEmphasis	0.018351244
wavelet-LLH	glszm	ZoneEntropy	5.112170611
wavelet-LLH	glszm	SmallAreaLowGrayLevelEmphasis	0.017128035
wavelet-LLH	ngtdm	Coarseness	0.046072034
wavelet-LLH	ngtdm	Complexity	1469.325987
wavelet-LLH	ngtdm	Strength	12.90915593
wavelet-LLH	ngtdm	Contrast	0.533623103
wavelet-LLH	ngtdm	Busyness	0.049350637
wavelet-LLL	gldm	GrayLevelVariance	169.9811385
wavelet-LLL	gldm	HighGrayLevelEmphasis	1327.240741
wavelet-LLL	gldm	DependenceEntropy	5.807992479
wavelet-LLL	gldm	DependenceNonUniformity	46.11111111
wavelet-LLL	gldm	GrayLevelNonUniformity	3.148148148
wavelet-LLL	gldm	SmallDependenceEmphasis	0.655156893
wavelet-LLL	gldm	SmallDependenceHighGrayLevelEmphasis	801.1242284
wavelet-LLL	gldm	DependenceNonUniformityNormalized	0.426954733
wavelet-LLL	gldm	LargeDependenceEmphasis	3.018518519
wavelet-LLL	gldm	LargeDependenceLowGrayLevelEmphasis	0.012730396
wavelet-LLL	gldm	DependenceVariance	0.540809328
wavelet-LLL	gldm	LargeDependenceHighGrayLevelEmphasis	4234.962963
wavelet-LLL	gldm	SmallDependenceLowGrayLevelEmphasis	0.01037053
wavelet-LLL	gldm	LowGrayLevelEmphasis	0.010717569
wavelet-LLL	glcm	JointAverage	37.49006598
wavelet-LLL	glcm	SumAverage	74.98013197
wavelet-LLL	glcm	JointEntropy	6.828881544
wavelet-LLL	glcm	ClusterShade	-160.668952
wavelet-LLL	glcm	MaximumProbability	0.019081543
wavelet-LLL	glcm	Idmn	0.956909522
wavelet-LLL	glcm	JointEnergy	0.009267811
wavelet-LLL	glcm	Contrast	205.6322793
wavelet-LLL	glcm	DifferenceEntropy	4.390472871
wavelet-LLL	glcm	InverseVariance	0.081311766
wavelet-LLL	glcm	DifferenceVariance	64.484652
wavelet-LLL	glcm	Idn	0.858410545
wavelet-LLL	glcm	Idm	0.088062687
wavelet-LLL	glcm	Correlation	0.30456815
wavelet-LLL	glcm	Autocorrelation	1454.288076
wavelet-LLL	glcm	SumEntropy	5.235827574
wavelet-LLL	glcm	SumSquares	149.8626648
wavelet-LLL	glcm	ClusterProminence	405822.6466
wavelet-LLL	glcm	Imc2	0.99948648
wavelet-LLL	glcm	Imc1	-0.6737606
wavelet-LLL	glcm	DifferenceAverage	11.58372415
wavelet-LLL	glcm	Id	0.16211002
wavelet-LLL	glcm	ClusterTendency	393.8183799
wavelet-LLL	firstorder	InterquartileRange	488.068099
wavelet-LLL	firstorder	Skewness	0.059614167
wavelet-LLL	firstorder	Uniformity	0.02914952
wavelet-LLL	firstorder	Median	-1456.071233
wavelet-LLL	firstorder	Energy	242365769.3
wavelet-LLL	firstorder	RobustMeanAbsoluteDeviation	194.0672947

wavelet-LLL	firstorder	MeanAbsoluteDeviation	265.595534
wavelet-LLL	firstorder	TotalEnergy	6543875771
wavelet-LLL	firstorder	Maximum	-675.0156335
wavelet-LLL	firstorder	RootMeanSquared	1498.041219
wavelet-LLL	firstorder	90Percentile	-1040.046905
wavelet-LLL	firstorder	Minimum	-2289.098079
wavelet-LLL	firstorder	Entropy	5.324836836
wavelet-LLL	firstorder	Range	1614.082445
wavelet-LLL	firstorder	Variance	106697.6046
wavelet-LLL	firstorder	10Percentile	-1901.219594
wavelet-LLL	firstorder	Kurtosis	2.501954256
wavelet-LLL	firstorder	Mean	-1461.995174
wavelet-LLL	glrlm	ShortRunLowGrayLevelEmphasis	0.010923534
wavelet-LLL	glrlm	GrayLevelVariance	171.201227
wavelet-LLL	glrlm	LowGrayLevelRunEmphasis	0.010940867
wavelet-LLL	glrlm	GrayLevelNonUniformityNormalized	0.028813872
wavelet-LLL	glrlm	RunVariance	0.022029297
wavelet-LLL	glrlm	GrayLevelNonUniformity	3.043578539
wavelet-LLL	glrlm	LongRunEmphasis	1.068973098
wavelet-LLL	glrlm	ShortRunHighGrayLevelEmphasis	1298.510539
wavelet-LLL	glrlm	RunLengthNonUniformity	101.0429862
wavelet-LLL	glrlm	ShortRunEmphasis	0.982756725
wavelet-LLL	glrlm	LongRunHighGrayLevelEmphasis	1425.463761
wavelet-LLL	glrlm	RunPercentage	0.977920228
wavelet-LLL	glrlm	LongRunLowGrayLevelEmphasis	0.011010197
wavelet-LLL	glrlm	RunEntropy	5.388833283
wavelet-LLL	glrlm	HighGrayLevelRunEmphasis	1323.901184
wavelet-LLL	glrlm	RunLengthNonUniformityNormalized	0.955941407
wavelet-LLL	glszm	GrayLevelVariance	184.7623438
wavelet-LLL	glszm	ZoneVariance	0.6025
wavelet-LLL	glszm	GrayLevelNonUniformityNormalized	0.0259375
wavelet-LLL	glszm	SizeZoneNonUniformityNormalized	0.611875
wavelet-LLL	glszm	SizeZoneNonUniformity	48.95
wavelet-LLL	glszm	GrayLevelNonUniformity	2.075
wavelet-LLL	glszm	LargeAreaEmphasis	2.425
wavelet-LLL	glszm	SmallAreaHighGrayLevelEmphasis	969.7128472
wavelet-LLL	glszm	ZonePercentage	0.740740741
wavelet-LLL	glszm	LargeAreaLowGrayLevelEmphasis	0.015573752
wavelet-LLL	glszm	LargeAreaHighGrayLevelEmphasis	3268.225
wavelet-LLL	glszm	HighGrayLevelZoneEmphasis	1272.9375
wavelet-LLL	glszm	SmallAreaEmphasis	0.810416667
wavelet-LLL	glszm	LowGrayLevelZoneEmphasis	0.014108873
wavelet-LLL	glszm	ZoneEntropy	5.703055907
wavelet-LLL	glszm	SmallAreaLowGrayLevelEmphasis	0.013920689
wavelet-LLL	ngtdm	Coarseness	0.051226312
wavelet-LLL	ngtdm	Complexity	6774.394676
wavelet-LLL	ngtdm	Strength	50.33696091
wavelet-LLL	ngtdm	Contrast	1.078655167
wavelet-LLL	ngtdm	Busyness	0.014994961
wavelet-HHL	gldm	GrayLevelVariance	33.47839506
wavelet-HHL	gldm	HighGrayLevelEmphasis	309.4074074
wavelet-HHL	gldm	DependenceEntropy	5.517399164
wavelet-HHL	gldm	DependenceNonUniformity	39.16666667

wavelet-HHL	gldm	GrayLevelNonUniformity	6.444444444
wavelet-HHL	gldm	SmallDependenceEmphasis	0.588121063
wavelet-HHL	gldm	SmallDependenceHighGrayLevelEmphasis	189.4477066
wavelet-HHL	gldm	DependenceNonUniformityNormalized	0.362654321
wavelet-HHL	gldm	LargeDependenceEmphasis	4.833333333
wavelet-HHL	gldm	LargeDependenceLowGrayLevelEmphasis	0.033584995
wavelet-HHL	gldm	DependenceVariance	1.403978052
wavelet-HHL	gldm	LargeDependenceHighGrayLevelEmphasis	1223.240741
wavelet-HHL	gldm	SmallDependenceLowGrayLevelEmphasis	0.012798616
wavelet-HHL	gldm	LowGrayLevelEmphasis	0.014655606
wavelet-HHL	glcm	JointAverage	16.66330404
wavelet-HHL	glcm	SumAverage	33.32660807
wavelet-HHL	glcm	JointEntropy	6.563307272
wavelet-HHL	glcm	ClusterShade	155.7084813
wavelet-HHL	glcm	MaximumProbability	0.031462059
wavelet-HHL	glcm	Idmn	0.951071176
wavelet-HHL	glcm	JointEnergy	0.011880861
wavelet-HHL	glcm	Contrast	71.3208551
wavelet-HHL	glcm	DifferenceEntropy	3.81053218
wavelet-HHL	glcm	InverseVariance	0.152828364
wavelet-HHL	glcm	DifferenceVariance	27.87735414
wavelet-HHL	glcm	Idn	0.856097374
wavelet-HHL	glcm	Idm	0.148056104
wavelet-HHL	glcm	Correlation	-0.029506672
wavelet-HHL	glcm	Autocorrelation	276.7106884
wavelet-HHL	glcm	SumEntropy	4.455783262
wavelet-HHL	glcm	SumSquares	34.67219061
wavelet-HHL	glcm	ClusterProminence	16981.46111
wavelet-HHL	glcm	Imc2	0.990980492
wavelet-HHL	glcm	Imc1	-0.47553996
wavelet-HHL	glcm	DifferenceAverage	6.497656199
wavelet-HHL	glcm	Id	0.239120837
wavelet-HHL	glcm	ClusterTendency	67.36790735
wavelet-HHL	firstorder	InterquartileRange	155.455983
wavelet-HHL	firstorder	Skewness	0.426768872
wavelet-HHL	firstorder	Uniformity	0.059670782
wavelet-HHL	firstorder	Median	-11.69049201
wavelet-HHL	firstorder	Energy	2249923.252
wavelet-HHL	firstorder	RobustMeanAbsoluteDeviation	71.96989374
wavelet-HHL	firstorder	MeanAbsoluteDeviation	110.3764512
wavelet-HHL	firstorder	TotalEnergy	60747927.81
wavelet-HHL	firstorder	Maximum	459.1958461
wavelet-HHL	firstorder	RootMeanSquared	144.3351056
wavelet-HHL	firstorder	90Percentile	201.0308059
wavelet-HHL	firstorder	Minimum	-378.4188353
wavelet-HHL	firstorder	Entropy	4.369139496
wavelet-HHL	firstorder	Range	837.6146814
wavelet-HHL	firstorder	Variance	20824.04402
wavelet-HHL	firstorder	10Percentile	-168.0643731
wavelet-HHL	firstorder	Kurtosis	3.540177496
wavelet-HHL	firstorder	Mean	2.928939285
wavelet-HHL	glrlm	ShortRunLowGrayLevelEmphasis	0.014878737
wavelet-HHL	glrlm	GrayLevelVariance	34.08105491

wavelet-HHL	glrlm	LowGrayLevelRunEmphasis	0.014997252
wavelet-HHL	glrlm	GrayLevelNonUniformityNormalized	0.058218823
wavelet-HHL	glrlm	RunVariance	0.035601602
wavelet-HHL	glrlm	GrayLevelNonUniformity	6.0839398
wavelet-HHL	glrlm	LongRunEmphasis	1.106539899
wavelet-HHL	glrlm	ShortRunHighGrayLevelEmphasis	304.6161349
wavelet-HHL	glrlm	RunLengthNonUniformity	98.04096806
wavelet-HHL	glrlm	ShortRunEmphasis	0.97501666
wavelet-HHL	glrlm	LongRunHighGrayLevelEmphasis	338.5616394
wavelet-HHL	glrlm	RunPercentage	0.967236467
wavelet-HHL	glrlm	LongRunLowGrayLevelEmphasis	0.015505562
wavelet-HHL	glrlm	RunEntropy	4.50199379
wavelet-HHL	glrlm	HighGrayLevelRunEmphasis	311.1451169
wavelet-HHL	glrlm	RunLengthNonUniformityNormalized	0.937413588
wavelet-HHL	glszm	GrayLevelVariance	41.41666667
wavelet-HHL	glszm	ZoneVariance	1.861111111
wavelet-HHL	glszm	GrayLevelNonUniformityNormalized	0.049768519
wavelet-HHL	glszm	SizeZoneNonUniformityNormalized	0.586033951
wavelet-HHL	glszm	SizeZoneNonUniformity	42.19444444
wavelet-HHL	glszm	GrayLevelNonUniformity	3.583333333
wavelet-HHL	glszm	LargeAreaEmphasis	4.111111111
wavelet-HHL	glszm	SmallAreaHighGrayLevelEmphasis	256.0514298
wavelet-HHL	glszm	ZonePercentage	0.666666667
wavelet-HHL	glszm	LargeAreaLowGrayLevelEmphasis	0.034971537
wavelet-HHL	glszm	LargeAreaHighGrayLevelEmphasis	1063.722222
wavelet-HHL	glszm	HighGrayLevelZoneEmphasis	324.7777778
wavelet-HHL	glszm	SmallAreaEmphasis	0.790715545
wavelet-HHL	glszm	LowGrayLevelZoneEmphasis	0.019700674
wavelet-HHL	glszm	ZoneEntropy	5.206257252
wavelet-HHL	glszm	SmallAreaLowGrayLevelEmphasis	0.018800856
wavelet-HHL	ngtdm	Coarseness	0.047546553
wavelet-HHL	ngtdm	Complexity	1376.286357
wavelet-HHL	ngtdm	Strength	12.01990866
wavelet-HHL	ngtdm	Contrast	0.412972601
wavelet-HHL	ngtdm	Busyness	0.053350668
wavelet-HLH	gldm	GrayLevelVariance	42.69718793
wavelet-HLH	gldm	HighGrayLevelEmphasis	258.3518519
wavelet-HLH	gldm	DependenceEntropy	5.465458049
wavelet-HLH	gldm	DependenceNonUniformity	37.77777778
wavelet-HLH	gldm	GrayLevelNonUniformity	5.888888889
wavelet-HLH	gldm	SmallDependenceEmphasis	0.573302469
wavelet-HLH	gldm	SmallDependenceHighGrayLevelEmphasis	160.2632459
wavelet-HLH	gldm	DependenceNonUniformityNormalized	0.349794239
wavelet-HLH	gldm	LargeDependenceEmphasis	4.111111111
wavelet-HLH	gldm	LargeDependenceLowGrayLevelEmphasis	0.054231739
wavelet-HLH	gldm	DependenceVariance	0.817558299
wavelet-HLH	gldm	LargeDependenceHighGrayLevelEmphasis	940.0555556
wavelet-HLH	gldm	SmallDependenceLowGrayLevelEmphasis	0.015588881
wavelet-HLH	gldm	LowGrayLevelEmphasis	0.020492068
wavelet-HLH	glcm	JointAverage	14.62060845
wavelet-HLH	glcm	SumAverage	29.2412169
wavelet-HLH	glcm	JointEntropy	6.633031113
wavelet-HLH	glcm	ClusterShade	233.7000129

wavelet-HLH	glcm	MaximumProbability	0.025488595
wavelet-HLH	glcm	Idmn	0.949272983
wavelet-HLH	glcm	JointEnergy	0.011013977
wavelet-HLH	glcm	Contrast	86.34089909
wavelet-HLH	glcm	DifferenceEntropy	3.975954229
wavelet-HLH	glcm	InverseVariance	0.126054901
wavelet-HLH	glcm	DifferenceVariance	31.15414303
wavelet-HLH	glcm	Idn	0.850877968
wavelet-HLH	glcm	Idm	0.13111574
wavelet-HLH	glcm	Correlation	-0.033301362
wavelet-HLH	glcm	Autocorrelation	212.4076908
wavelet-HLH	glcm	SumEntropy	4.624508802
wavelet-HLH	glcm	SumSquares	41.7662124
wavelet-HLH	glcm	ClusterProminence	23533.43856
wavelet-HLH	glcm	Imc2	0.992569373
wavelet-HLH	glcm	Imc1	-0.490806836
wavelet-HLH	glcm	DifferenceAverage	7.308635975
wavelet-HLH	glcm	Id	0.220107452
wavelet-HLH	glcm	ClusterTendency	80.72395051
wavelet-HLH	firstorder	InterquartileRange	218.2607168
wavelet-HLH	firstorder	Skewness	0.517525833
wavelet-HLH	firstorder	Uniformity	0.054526749
wavelet-HLH	firstorder	Median	11.11416607
wavelet-HLH	firstorder	Energy	2850671.672
wavelet-HLH	firstorder	RobustMeanAbsoluteDeviation	83.72733491
wavelet-HLH	firstorder	MeanAbsoluteDeviation	125.2139391
wavelet-HLH	firstorder	TotalEnergy	76968135.14
wavelet-HLH	firstorder	Maximum	589.8484952
wavelet-HLH	firstorder	RootMeanSquared	162.4657135
wavelet-HLH	firstorder	90Percentile	176.4377069
wavelet-HLH	firstorder	Minimum	-332.7244381
wavelet-HLH	firstorder	Entropy	4.42227324
wavelet-HLH	firstorder	Range	922.5729334
wavelet-HLH	firstorder	Variance	26381.32818
wavelet-HLH	firstorder	10Percentile	-202.2965333
wavelet-HLH	firstorder	Kurtosis	3.882104792
wavelet-HLH	firstorder	Mean	3.71212824
wavelet-HLH	glrlm	ShortRunLowGrayLevelEmphasis	0.020525897
wavelet-HLH	glrlm	GrayLevelVariance	43.10714145
wavelet-HLH	glrlm	LowGrayLevelRunEmphasis	0.020800085
wavelet-HLH	glrlm	GrayLevelNonUniformityNormalized	0.054368639
wavelet-HLH	glrlm	RunVariance	0.031252812
wavelet-HLH	glrlm	GrayLevelNonUniformity	5.688066385
wavelet-HLH	glrlm	LongRunEmphasis	1.098613676
wavelet-HLH	glrlm	ShortRunHighGrayLevelEmphasis	254.034104
wavelet-HLH	glrlm	RunLengthNonUniformity	98.17710777
wavelet-HLH	glrlm	ShortRunEmphasis	0.975346581
wavelet-HLH	glrlm	LongRunHighGrayLevelEmphasis	281.4842964
wavelet-HLH	glrlm	RunPercentage	0.968660969
wavelet-HLH	glrlm	LongRunLowGrayLevelEmphasis	0.021896834
wavelet-HLH	glrlm	RunEntropy	4.53366278
wavelet-HLH	glrlm	HighGrayLevelRunEmphasis	259.5241425
wavelet-HLH	glrlm	RunLengthNonUniformityNormalized	0.937494375

wavelet-HLH	glszm	GrayLevelVariance	48.9581535
wavelet-HLH	glszm	ZoneVariance	0.742728467
wavelet-HLH	glszm	GrayLevelNonUniformityNormalized	0.050478514
wavelet-HLH	glszm	SizeZoneNonUniformityNormalized	0.539876149
wavelet-HLH	glszm	SizeZoneNonUniformity	39.4109589
wavelet-HLH	glszm	GrayLevelNonUniformity	3.684931507
wavelet-HLH	glszm	LargeAreaEmphasis	2.931506849
wavelet-HLH	glszm	SmallAreaHighGrayLevelEmphasis	217.3845129
wavelet-HLH	glszm	ZonePercentage	0.675925926
wavelet-HLH	glszm	LargeAreaLowGrayLevelEmphasis	0.045250931
wavelet-HLH	glszm	LargeAreaHighGrayLevelEmphasis	708.4109589
wavelet-HLH	glszm	HighGrayLevelZoneEmphasis	273.1369863
wavelet-HLH	glszm	SmallAreaEmphasis	0.762557078
wavelet-HLH	glszm	LowGrayLevelZoneEmphasis	0.024968515
wavelet-HLH	glszm	ZoneEntropy	5.282443313
wavelet-HLH	glszm	SmallAreaLowGrayLevelEmphasis	0.022083191
wavelet-HLH	ngtdm	Coarseness	0.039875911
wavelet-HLH	ngtdm	Complexity	1773.517043
wavelet-HLH	ngtdm	Strength	12.13134553
wavelet-HLH	ngtdm	Contrast	0.614580166
wavelet-HLH	ngtdm	Busyness	0.068783068

Image type	Feature Class	Feature Name	11: RTSTRUCT: C99-DNU_segment_ITVp RLL
general	info	BoundingBox	(0, 0, 1, 6, 6, 14)
general	info	EnabledImageTypes	{u'Original': {}, u'Wavelet': {}}
general	info	GeneralSettings	{'distances': [1], 'additionalInfo': True, 'force2D': False, 'interpolator': 'sitkBSpline', 'resampledPixelSpacing': [3.0], 'label': 1, 'normalizeScale': 1, 'normalize': False, 'force2Ddimension': 0, 'removeOutliers': None, 'minimumROISize': None, u'binWidth': 25.0, 'minimumROIDimensions': 1, u'correctMask': True, 'preCrop': False, u'symmetricalGLCM': True, 'resegmentRange': None, 'padDistance': 5}
general	info	ImageHash	df67948ff51d1c9dfdf52362da6b02592924fb0d
general	info	ImageSpacing	(0.9765625000000002, 0.9765625000000002, 2.0)
general	info	MaskHash	cf35b0cd870305e050716ba8f6768dc01624dff4
general	info	NumpyVersion	1.13.1
general	info	PyWaveletVersion	0.5.2
general	info	SimpleITKVersion	1.1.0
general	info	Version	1.3.0.post102+g1b0eb74
general	info	VolumeNum	1
general	info	VoxelNum	219
original	shape	Maximum3DDiameter	40.13726448
original	shape	Maximum2DDiameterSlice	16.15549442
original	shape	Sphericity	0.756013893
original	shape	MinorAxis	15.31584871
original	shape	Elongation	0.352393167
original	shape	SurfaceVolumeRatio	0.35374072
original	shape	Volume	5913
original	shape	MajorAxis	43.46238839
original	shape	SurfaceArea	2091.668876
original	shape	Flatness	0.300921319
original	shape	LeastAxis	13.07875925
original	shape	Maximum2DDiameterColumn	40.02499219
original	shape	Maximum2DDiameterRow	40.02499219
original	gldm	GrayLevelVariance	11.69541919
original	gldm	HighGrayLevelEmphasis	197.3515982
original	gldm	DependenceEntropy	5.672337935
original	gldm	DependenceNonUniformity	33.32876712
original	gldm	GrayLevelNonUniformity	21.87671233
original	gldm	SmallDependenceEmphasis	0.278709802
original	gldm	SmallDependenceHighGrayLevelEmphasis	40.3399949
original	gldm	DependenceNonUniformityNormalized	0.152186151
original	gldm	LargeDependenceEmphasis	16.8173516
original	gldm	LargeDependenceLowGrayLevelEmphasis	0.082358849
original	gldm	DependenceVariance	4.615249891
original	gldm	LargeDependenceHighGrayLevelEmphasis	4033.205479
original	gldm	SmallDependenceLowGrayLevelEmphasis	0.007782615
original	gldm	LowGrayLevelEmphasis	0.011796912
original	glcm	JointAverage	14.3234171
original	glcm	SumAverage	28.64683419

original	glcm	JointEntropy	6.285063171
original	glcm	ClusterShade	-99.45041692
original	glcm	MaximumProbability	0.049963376
original	glcm	Idmn	0.963824455
original	glcm	JointEnergy	0.017700775
original	glcm	Contrast	15.26715841
original	glcm	DifferenceEntropy	3.008104917
original	glcm	InverseVariance	0.319113156
original	glcm	DifferenceVariance	7.069978605
original	glcm	Idn	0.881726417
original	glcm	Idm	0.3297096
original	glcm	Correlation	0.234460061
original	glcm	Autocorrelation	207.5558271
original	glcm	SumEntropy	4.087487592
original	glcm	SumSquares	10.01452469
original	glcm	ClusterProminence	1977.582853
original	glcm	Imc2	0.829677395
original	glcm	Imc1	-0.171698269
original	glcm	DifferenceAverage	2.834598218
original	glcm	Id	0.407673447
original	glcm	ClusterTendency	24.79094036
original	firstorder	InterquartileRange	102
original	firstorder	Skewness	-0.897335235
original	firstorder	Uniformity	0.099893664
original	firstorder	Median	-47
original	firstorder	Energy	2754914
original	firstorder	RobustMeanAbsoluteDeviation	48.13068182
original	firstorder	MeanAbsoluteDeviation	68.31654886
original	firstorder	TotalEnergy	74382678
original	firstorder	Maximum	72
original	firstorder	RootMeanSquared	112.1584414
original	firstorder	90Percentile	17
original	firstorder	Minimum	-376
original	firstorder	Entropy	3.581499903
original	firstorder	Range	448
original	firstorder	Variance	7277.814266
original	firstorder	10Percentile	-202.4
original	firstorder	Kurtosis	3.294602862
original	firstorder	Mean	-72.81278539
original	glrlm	ShortRunLowGrayLevelEmphasis	0.012190909
original	glrlm	GrayLevelVariance	12.15116807
original	glrlm	LowGrayLevelRunEmphasis	0.012544897
original	glrlm	GrayLevelNonUniformityNormalized	0.095073685
original	glrlm	RunVariance	0.118191451
original	glrlm	GrayLevelNonUniformity	18.82545072
original	glrlm	LongRunEmphasis	1.344227576
original	glrlm	ShortRunHighGrayLevelEmphasis	176.2304443
original	glrlm	RunLengthNonUniformity	163.9073314
original	glrlm	ShortRunEmphasis	0.926868437
original	glrlm	LongRunHighGrayLevelEmphasis	274.5339048
original	glrlm	RunPercentage	0.904109589
original	glrlm	LongRunLowGrayLevelEmphasis	0.01418504
original	glrlm	RunEntropy	4.025701949
original	glrlm	HighGrayLevelRunEmphasis	193.3300964
original	glrlm	RunLengthNonUniformityNormalized	0.826738666

original	glszm	GrayLevelVariance	14.57684369
original	glszm	ZoneVariance	26.08219178
original	glszm	GrayLevelNonUniformityNormalized	0.085006568
original	glszm	SizeZoneNonUniformityNormalized	0.376618503
original	glszm	SizeZoneNonUniformity	27.49315068
original	glszm	GrayLevelNonUniformity	6.205479452
original	glszm	LargeAreaEmphasis	35.08219178
original	glszm	SmallAreaHighGrayLevelEmphasis	80.4980896
original	glszm	ZonePercentage	0.333333333
original	glszm	LargeAreaLowGrayLevelEmphasis	0.170431204
original	glszm	LargeAreaHighGrayLevelEmphasis	8571.09589
original	glszm	HighGrayLevelZoneEmphasis	142.9178082
original	glszm	SmallAreaEmphasis	0.629752882
original	glszm	LowGrayLevelZoneEmphasis	0.024905457
original	glszm	ZoneEntropy	4.876258594
original	glszm	SmallAreaLowGrayLevelEmphasis	0.022012207
original	ngtdm	Coarseness	0.031996862
original	ngtdm	Complexity	203.6877739
original	ngtdm	Strength	3.00349847
original	ngtdm	Contrast	0.202822328
original	ngtdm	Busyness	0.141565754
wavelet-HHH	gldm	GrayLevelVariance	2.770417631
wavelet-HHH	gldm	HighGrayLevelEmphasis	58.84931507
wavelet-HHH	gldm	DependenceEntropy	5.301928675
wavelet-HHH	gldm	DependenceNonUniformity	27.72146119
wavelet-HHH	gldm	GrayLevelNonUniformity	41.34703196
wavelet-HHH	gldm	SmallDependenceEmphasis	0.190273529
wavelet-HHH	gldm	SmallDependenceHighGrayLevelEmphasis	12.97750832
wavelet-HHH	gldm	DependenceNonUniformityNormalized	0.126582015
wavelet-HHH	gldm	LargeDependenceEmphasis	25.03652968
wavelet-HHH	gldm	LargeDependenceLowGrayLevelEmphasis	0.463741534
wavelet-HHH	gldm	DependenceVariance	6.337816142
wavelet-HHH	gldm	LargeDependenceHighGrayLevelEmphasis	1463.525114
wavelet-HHH	gldm	SmallDependenceLowGrayLevelEmphasis	0.009462271
wavelet-HHH	gldm	LowGrayLevelEmphasis	0.025602349
wavelet-HHH	glcm	JointAverage	7.494033243
wavelet-HHH	glcm	SumAverage	14.98806649
wavelet-HHH	glcm	JointEntropy	5.069286648
wavelet-HHH	glcm	ClusterShade	-0.518666175
wavelet-HHH	glcm	MaximumProbability	0.088573471
wavelet-HHH	glcm	Idmn	0.975885167
wavelet-HHH	glcm	JointEnergy	0.041183926
wavelet-HHH	glcm	Contrast	5.189995203
wavelet-HHH	glcm	DifferenceEntropy	2.351331836
wavelet-HHH	glcm	InverseVariance	0.411832525
wavelet-HHH	glcm	DifferenceVariance	2.180265324
wavelet-HHH	glcm	Idn	0.898031691
wavelet-HHH	glcm	Idm	0.427397501
wavelet-HHH	glcm	Correlation	-0.022370706
wavelet-HHH	glcm	Autocorrelation	56.10673325
wavelet-HHH	glcm	SumEntropy	3.078367748
wavelet-HHH	glcm	SumSquares	2.540298884
wavelet-HHH	glcm	ClusterProminence	106.7023153
wavelet-HHH	glcm	Imc2	0.64196815
wavelet-HHH	glcm	Imc1	-0.102147263

wavelet-HHH	glcm	DifferenceAverage	1.718304835
wavelet-HHH	glcm	Id	0.491779319
wavelet-HHH	glcm	ClusterTendency	4.971200333
wavelet-HHH	firstorder	InterquartileRange	47.80228909
wavelet-HHH	firstorder	Skewness	-0.061891999
wavelet-HHH	firstorder	Uniformity	0.188799233
wavelet-HHH	firstorder	Median	1.497335065
wavelet-HHH	firstorder	Energy	360806.8663
wavelet-HHH	firstorder	RobustMeanAbsoluteDeviation	19.7705291
wavelet-HHH	firstorder	MeanAbsoluteDeviation	30.47912561
wavelet-HHH	firstorder	TotalEnergy	9741785.389
wavelet-HHH	firstorder	Maximum	155.5669052
wavelet-HHH	firstorder	RootMeanSquared	40.58965308
wavelet-HHH	firstorder	90Percentile	43.9379285
wavelet-HHH	firstorder	Minimum	-154.4465919
wavelet-HHH	firstorder	Entropy	2.708728163
wavelet-HHH	firstorder	Range	310.0134971
wavelet-HHH	firstorder	Variance	1647.506138
wavelet-HHH	firstorder	10Percentile	-47.91396316
wavelet-HHH	firstorder	Kurtosis	4.723284612
wavelet-HHH	firstorder	Mean	0.117468683
wavelet-HHH	glrlm	ShortRunLowGrayLevelEmphasis	0.024755688
wavelet-HHH	glrlm	GrayLevelVariance	3.024238514
wavelet-HHH	glrlm	LowGrayLevelRunEmphasis	0.026593471
wavelet-HHH	glrlm	GrayLevelNonUniformityNormalized	0.179472366
wavelet-HHH	glrlm	RunVariance	0.173747578
wavelet-HHH	glrlm	GrayLevelNonUniformity	34.28692187
wavelet-HHH	glrlm	LongRunEmphasis	1.492975981
wavelet-HHH	glrlm	ShortRunHighGrayLevelEmphasis	53.45362165
wavelet-HHH	glrlm	RunLengthNonUniformity	148.9369756
wavelet-HHH	glrlm	ShortRunEmphasis	0.903007716
wavelet-HHH	glrlm	LongRunHighGrayLevelEmphasis	87.39310021
wavelet-HHH	glrlm	RunPercentage	0.872146119
wavelet-HHH	glrlm	LongRunLowGrayLevelEmphasis	0.03589333
wavelet-HHH	glrlm	RunEntropy	3.317362784
wavelet-HHH	glrlm	HighGrayLevelRunEmphasis	59.04165681
wavelet-HHH	glrlm	RunLengthNonUniformityNormalized	0.778361159
wavelet-HHH	glszm	GrayLevelVariance	9.304601899
wavelet-HHH	glszm	ZoneVariance	168.128561
wavelet-HHH	glszm	GrayLevelNonUniformityNormalized	0.11468225
wavelet-HHH	glszm	SizeZoneNonUniformityNormalized	0.474068663
wavelet-HHH	glszm	SizeZoneNonUniformity	17.54054054
wavelet-HHH	glszm	GrayLevelNonUniformity	4.243243243
wavelet-HHH	glszm	LargeAreaEmphasis	203.1621622
wavelet-HHH	glszm	SmallAreaHighGrayLevelEmphasis	53.57260245
wavelet-HHH	glszm	ZonePercentage	0.168949772
wavelet-HHH	glszm	LargeAreaLowGrayLevelEmphasis	3.742569217
wavelet-HHH	glszm	LargeAreaHighGrayLevelEmphasis	11774.10811
wavelet-HHH	glszm	HighGrayLevelZoneEmphasis	69.89189189
wavelet-HHH	glszm	SmallAreaEmphasis	0.706397386
wavelet-HHH	glszm	LowGrayLevelZoneEmphasis	0.054103524
wavelet-HHH	glszm	ZoneEntropy	4.208924987
wavelet-HHH	glszm	SmallAreaLowGrayLevelEmphasis	0.046973857
wavelet-HHH	ngtdm	Coarseness	0.028107315
wavelet-HHH	ngtdm	Complexity	78.45489985

wavelet-HHH	ngtdm	Strength	1.392439184
wavelet-HHH	ngtdm	Contrast	0.055128484
wavelet-HHH	ngtdm	Busyness	0.329202546
wavelet-HLL	gldm	GrayLevelVariance	3.865682534
wavelet-HLL	gldm	HighGrayLevelEmphasis	86.76712329
wavelet-HLL	gldm	DependenceEntropy	5.288087653
wavelet-HLL	gldm	DependenceNonUniformity	33.0456621
wavelet-HLL	gldm	GrayLevelNonUniformity	34.17808219
wavelet-HLL	gldm	SmallDependenceEmphasis	0.236189701
wavelet-HLL	gldm	SmallDependenceHighGrayLevelEmphasis	19.53804255
wavelet-HLL	gldm	DependenceNonUniformityNormalized	0.150893434
wavelet-HLL	gldm	LargeDependenceEmphasis	18.43378995
wavelet-HLL	gldm	LargeDependenceLowGrayLevelEmphasis	0.228180704
wavelet-HLL	gldm	DependenceVariance	4.922291028
wavelet-HLL	gldm	LargeDependenceHighGrayLevelEmphasis	1615.260274
wavelet-HLL	gldm	SmallDependenceLowGrayLevelEmphasis	0.009253344
wavelet-HLL	gldm	LowGrayLevelEmphasis	0.018849467
wavelet-HLL	glcm	JointAverage	9.175594613
wavelet-HLL	glcm	SumAverage	18.35118923
wavelet-HLL	glcm	JointEntropy	5.43404815
wavelet-HLL	glcm	ClusterShade	-5.833013264
wavelet-HLL	glcm	MaximumProbability	0.067496561
wavelet-HLL	glcm	Idmn	0.967561146
wavelet-HLL	glcm	JointEnergy	0.031997983
wavelet-HLL	glcm	Contrast	7.215461352
wavelet-HLL	glcm	DifferenceEntropy	2.51756971
wavelet-HLL	glcm	InverseVariance	0.388169192
wavelet-HLL	glcm	DifferenceVariance	2.924733991
wavelet-HLL	glcm	Idn	0.882488986
wavelet-HLL	glcm	Idm	0.378763843
wavelet-HLL	glcm	Correlation	-0.027576174
wavelet-HLL	glcm	Autocorrelation	84.12116576
wavelet-HLL	glcm	SumEntropy	3.300457079
wavelet-HLL	glcm	SumSquares	3.533602399
wavelet-HLL	glcm	ClusterProminence	207.7109168
wavelet-HLL	glcm	Imc2	0.715033829
wavelet-HLL	glcm	Imc1	-0.127013961
wavelet-HLL	glcm	DifferenceAverage	2.034149464
wavelet-HLL	glcm	Id	0.451404492
wavelet-HLL	glcm	ClusterTendency	6.918948244
wavelet-HLL	firstorder	InterquartileRange	56.83353007
wavelet-HLL	firstorder	Skewness	-0.516952987
wavelet-HLL	firstorder	Uniformity	0.156064302
wavelet-HLL	firstorder	Median	-6.810096345
wavelet-HLL	firstorder	Energy	551447.086
wavelet-HLL	firstorder	RobustMeanAbsoluteDeviation	24.54402844
wavelet-HLL	firstorder	MeanAbsoluteDeviation	37.45009528
wavelet-HLL	firstorder	TotalEnergy	14889071.32
wavelet-HLL	firstorder	Maximum	119.7802768
wavelet-HLL	firstorder	RootMeanSquared	50.17990857
wavelet-HLL	firstorder	90Percentile	47.02154807
wavelet-HLL	firstorder	Minimum	-216.0199944
wavelet-HLL	firstorder	Entropy	2.962746721
wavelet-HLL	firstorder	Range	335.8002712
wavelet-HLL	firstorder	Variance	2413.323989

wavelet-HLL	firstorder	10Percentile	-69.10273775
wavelet-HLL	firstorder	Kurtosis	4.357227866
wavelet-HLL	firstorder	Mean	-10.23226437
wavelet-HLL	glrlm	ShortRunLowGrayLevelEmphasis	0.01864494
wavelet-HLL	glrlm	GrayLevelVariance	4.152124214
wavelet-HLL	glrlm	LowGrayLevelRunEmphasis	0.019611328
wavelet-HLL	glrlm	GrayLevelNonUniformityNormalized	0.149173454
wavelet-HLL	glrlm	RunVariance	0.130688337
wavelet-HLL	glrlm	GrayLevelNonUniformity	29.30706879
wavelet-HLL	glrlm	LongRunEmphasis	1.377081901
wavelet-HLL	glrlm	ShortRunHighGrayLevelEmphasis	79.86683497
wavelet-HLL	glrlm	RunLengthNonUniformity	160.3913577
wavelet-HLL	glrlm	ShortRunEmphasis	0.921308018
wavelet-HLL	glrlm	LongRunHighGrayLevelEmphasis	119.5859209
wavelet-HLL	glrlm	RunPercentage	0.897084651
wavelet-HLL	glrlm	LongRunLowGrayLevelEmphasis	0.024236524
wavelet-HLL	glrlm	RunEntropy	3.467459066
wavelet-HLL	glrlm	HighGrayLevelRunEmphasis	86.72330068
wavelet-HLL	glrlm	RunLengthNonUniformityNormalized	0.814973369
wavelet-HLL	glszm	GrayLevelVariance	9.647891659
wavelet-HLL	glszm	ZoneVariance	60.27331487
wavelet-HLL	glszm	GrayLevelNonUniformityNormalized	0.106802093
wavelet-HLL	glszm	SizeZoneNonUniformityNormalized	0.35426285
wavelet-HLL	glszm	SizeZoneNonUniformity	20.19298246
wavelet-HLL	glszm	GrayLevelNonUniformity	6.087719298
wavelet-HLL	glszm	LargeAreaEmphasis	75.03508772
wavelet-HLL	glszm	SmallAreaHighGrayLevelEmphasis	48.33750142
wavelet-HLL	glszm	ZonePercentage	0.260273973
wavelet-HLL	glszm	LargeAreaLowGrayLevelEmphasis	0.920066398
wavelet-HLL	glszm	LargeAreaHighGrayLevelEmphasis	6547.719298
wavelet-HLL	glszm	HighGrayLevelZoneEmphasis	85.36842105
wavelet-HLL	glszm	SmallAreaEmphasis	0.610211752
wavelet-HLL	glszm	LowGrayLevelZoneEmphasis	0.036895689
wavelet-HLL	glszm	ZoneEntropy	4.684048901
wavelet-HLL	glszm	SmallAreaLowGrayLevelEmphasis	0.031747333
wavelet-HLL	ngtdm	Coarseness	0.026766138
wavelet-HLL	ngtdm	Complexity	109.1814133
wavelet-HLL	ngtdm	Strength	1.514001602
wavelet-HLL	ngtdm	Contrast	0.078723518
wavelet-HLL	ngtdm	Busyness	0.279401031
wavelet-LHL	gldm	GrayLevelVariance	90.1897792
wavelet-LHL	gldm	HighGrayLevelEmphasis	4221.333333
wavelet-LHL	gldm	DependenceEntropy	5.859825946
wavelet-LHL	gldm	DependenceNonUniformity	45.16438356
wavelet-LHL	gldm	GrayLevelNonUniformity	16.10502283
wavelet-LHL	gldm	SmallDependenceEmphasis	0.399435981
wavelet-LHL	gldm	SmallDependenceHighGrayLevelEmphasis	1578.437204
wavelet-LHL	gldm	DependenceNonUniformityNormalized	0.206230062
wavelet-LHL	gldm	LargeDependenceEmphasis	10.9543379
wavelet-LHL	gldm	LargeDependenceLowGrayLevelEmphasis	0.007126652
wavelet-LHL	gldm	DependenceVariance	3.372990555
wavelet-LHL	gldm	LargeDependenceHighGrayLevelEmphasis	48537.23288
wavelet-LHL	gldm	SmallDependenceLowGrayLevelEmphasis	0.00472269
wavelet-LHL	gldm	LowGrayLevelEmphasis	0.004864962
wavelet-LHL	glcm	JointAverage	65.13918101

wavelet-LHL	glcm	SumAverage	130.278362
wavelet-LHL	glcm	JointEntropy	6.779592623
wavelet-LHL	glcm	ClusterShade	-5272.783488
wavelet-LHL	glcm	MaximumProbability	0.043882885
wavelet-LHL	glcm	Idmn	0.986205884
wavelet-LHL	glcm	JointEnergy	0.012704501
wavelet-LHL	glcm	Contrast	140.4595666
wavelet-LHL	glcm	DifferenceEntropy	3.752355388
wavelet-LHL	glcm	InverseVariance	0.237330238
wavelet-LHL	glcm	DifferenceVariance	94.18997864
wavelet-LHL	glcm	Idn	0.940372311
wavelet-LHL	glcm	Idm	0.237077078
wavelet-LHL	glcm	Correlation	-0.064094258
wavelet-LHL	glcm	Autocorrelation	4243.919719
wavelet-LHL	glcm	SumEntropy	4.616347361
wavelet-LHL	glcm	SumSquares	70.76321421
wavelet-LHL	glcm	ClusterProminence	466795.3081
wavelet-LHL	glcm	Imc2	0.970913047
wavelet-LHL	glcm	Imc1	-0.356496798
wavelet-LHL	glcm	DifferenceAverage	6.459329292
wavelet-LHL	glcm	Id	0.316048285
wavelet-LHL	glcm	ClusterTendency	142.5932902
wavelet-LHL	firstorder	InterquartileRange	144.5570649
wavelet-LHL	firstorder	Skewness	-3.063684756
wavelet-LHL	firstorder	Uniformity	0.073538917
wavelet-LHL	firstorder	Median	-56.19118477
wavelet-LHL	firstorder	Energy	14752048.16
wavelet-LHL	firstorder	RobustMeanAbsoluteDeviation	64.19578394
wavelet-LHL	firstorder	MeanAbsoluteDeviation	136.5228182
wavelet-LHL	firstorder	TotalEnergy	398305300.3
wavelet-LHL	firstorder	Maximum	530.288741
wavelet-LHL	firstorder	RootMeanSquared	259.5398823
wavelet-LHL	firstorder	90Percentile	50.8496649
wavelet-LHL	firstorder	Minimum	-1685.686782
wavelet-LHL	firstorder	Entropy	4.315206669
wavelet-LHL	firstorder	Range	2215.975523
wavelet-LHL	firstorder	Variance	56077.32423
wavelet-LHL	firstorder	10Percentile	-314.1078604
wavelet-LHL	firstorder	Kurtosis	18.92535174
wavelet-LHL	firstorder	Mean	-106.2244146
wavelet-LHL	glrlm	ShortRunLowGrayLevelEmphasis	0.005203546
wavelet-LHL	glrlm	GrayLevelVariance	95.45690031
wavelet-LHL	glrlm	LowGrayLevelRunEmphasis	0.005215054
wavelet-LHL	glrlm	GrayLevelNonUniformityNormalized	0.06811151
wavelet-LHL	glrlm	RunVariance	0.093354003
wavelet-LHL	glrlm	GrayLevelNonUniformity	13.92703337
wavelet-LHL	glrlm	LongRunEmphasis	1.254025845
wavelet-LHL	glrlm	ShortRunHighGrayLevelEmphasis	3984.479088
wavelet-LHL	glrlm	RunLengthNonUniformity	180.6250783
wavelet-LHL	glrlm	ShortRunEmphasis	0.950201761
wavelet-LHL	glrlm	LongRunHighGrayLevelEmphasis	5331.055312
wavelet-LHL	glrlm	RunPercentage	0.93256059
wavelet-LHL	glrlm	LongRunLowGrayLevelEmphasis	0.005273584
wavelet-LHL	glrlm	RunEntropy	4.648342783
wavelet-LHL	glrlm	HighGrayLevelRunEmphasis	4205.368349

wavelet-LHL	glrlm	RunLengthNonUniformityNormalized	0.880753132
wavelet-LHL	glszm	GrayLevelVariance	160.0954808
wavelet-LHL	glszm	ZoneVariance	10.53602588
wavelet-LHL	glszm	GrayLevelNonUniformityNormalized	0.042446819
wavelet-LHL	glszm	SizeZoneNonUniformityNormalized	0.471620429
wavelet-LHL	glszm	SizeZoneNonUniformity	47.63366337
wavelet-LHL	glszm	GrayLevelNonUniformity	4.287128713
wavelet-LHL	glszm	LargeAreaEmphasis	15.23762376
wavelet-LHL	glszm	SmallAreaHighGrayLevelEmphasis	2758.206081
wavelet-LHL	glszm	ZonePercentage	0.461187215
wavelet-LHL	glszm	LargeAreaLowGrayLevelEmphasis	0.013489253
wavelet-LHL	glszm	LargeAreaHighGrayLevelEmphasis	67645.68317
wavelet-LHL	glszm	HighGrayLevelZoneEmphasis	3996.732673
wavelet-LHL	glszm	SmallAreaEmphasis	0.71349478
wavelet-LHL	glszm	LowGrayLevelZoneEmphasis	0.010275395
wavelet-LHL	glszm	ZoneEntropy	5.554251523
wavelet-LHL	glszm	SmallAreaLowGrayLevelEmphasis	0.010203122
wavelet-LHL	ngtdm	Coarseness	0.026656544
wavelet-LHL	ngtdm	Complexity	4251.16143
wavelet-LHL	ngtdm	Strength	32.98392927
wavelet-LHL	ngtdm	Contrast	0.691703307
wavelet-LHL	ngtdm	Busyness	0.012728316
wavelet-LHH	gldm	GrayLevelVariance	6.802902358
wavelet-LHH	gldm	HighGrayLevelEmphasis	150.1461187
wavelet-LHH	gldm	DependenceEntropy	5.473141026
wavelet-LHH	gldm	DependenceNonUniformity	35.75799087
wavelet-LHH	gldm	GrayLevelNonUniformity	28.76255708
wavelet-LHH	gldm	SmallDependenceEmphasis	0.271747289
wavelet-LHH	gldm	SmallDependenceHighGrayLevelEmphasis	42.03604159
wavelet-LHH	gldm	DependenceNonUniformityNormalized	0.163278497
wavelet-LHH	gldm	LargeDependenceEmphasis	15.15525114
wavelet-LHH	gldm	LargeDependenceLowGrayLevelEmphasis	0.116825662
wavelet-LHH	gldm	DependenceVariance	3.706761744
wavelet-LHH	gldm	LargeDependenceHighGrayLevelEmphasis	2216.684932
wavelet-LHH	gldm	SmallDependenceLowGrayLevelEmphasis	0.008046913
wavelet-LHH	gldm	LowGrayLevelEmphasis	0.013656109
wavelet-LHH	glcm	JointAverage	12.15119259
wavelet-LHH	glcm	SumAverage	24.30238518
wavelet-LHH	glcm	JointEntropy	5.877492332
wavelet-LHH	glcm	ClusterShade	-28.01433226
wavelet-LHH	glcm	MaximumProbability	0.05451921
wavelet-LHH	glcm	Idmn	0.971268496
wavelet-LHH	glcm	JointEnergy	0.023705262
wavelet-LHH	glcm	Contrast	13.59058509
wavelet-LHH	glcm	DifferenceEntropy	2.827383034
wavelet-LHH	glcm	InverseVariance	0.342046241
wavelet-LHH	glcm	DifferenceVariance	6.586878744
wavelet-LHH	glcm	Idn	0.89408261
wavelet-LHH	glcm	Idm	0.336416869
wavelet-LHH	glcm	Correlation	-0.041343402
wavelet-LHH	glcm	Autocorrelation	147.4358815
wavelet-LHH	glcm	SumEntropy	3.64313701
wavelet-LHH	glcm	SumSquares	6.569706221
wavelet-LHH	glcm	ClusterProminence	993.6458969
wavelet-LHH	glcm	Imc2	0.819451007

wavelet-LHH	glcm	Imc1	-0.17714107
wavelet-LHH	glcm	DifferenceAverage	2.615526383
wavelet-LHH	glcm	Id	0.414775861
wavelet-LHH	glcm	ClusterTendency	12.6882398
wavelet-LHH	firstorder	InterquartileRange	69.20655074
wavelet-LHH	firstorder	Skewness	-0.431024056
wavelet-LHH	firstorder	Uniformity	0.131335877
wavelet-LHH	firstorder	Median	-12.90763215
wavelet-LHH	firstorder	Energy	958001.1338
wavelet-LHH	firstorder	RobustMeanAbsoluteDeviation	29.24918441
wavelet-LHH	firstorder	MeanAbsoluteDeviation	47.04306698
wavelet-LHH	firstorder	TotalEnergy	25866030.61
wavelet-LHH	firstorder	Maximum	188.601719
wavelet-LHH	firstorder	RootMeanSquared	66.13950711
wavelet-LHH	firstorder	90Percentile	61.91446273
wavelet-LHH	firstorder	Minimum	-279.9944139
wavelet-LHH	firstorder	Entropy	3.261933548
wavelet-LHH	firstorder	Range	468.5961329
wavelet-LHH	firstorder	Variance	4226.083775
wavelet-LHH	firstorder	10Percentile	-86.05764138
wavelet-LHH	firstorder	Kurtosis	5.48374803
wavelet-LHH	firstorder	Mean	-12.17992718
wavelet-LHH	glrlm	ShortRunLowGrayLevelEmphasis	0.013790148
wavelet-LHH	glrlm	GrayLevelVariance	7.256465416
wavelet-LHH	glrlm	LowGrayLevelRunEmphasis	0.014303565
wavelet-LHH	glrlm	GrayLevelNonUniformityNormalized	0.12570881
wavelet-LHH	glrlm	RunVariance	0.11946105
wavelet-LHH	glrlm	GrayLevelNonUniformity	25.01911038
wavelet-LHH	glrlm	LongRunEmphasis	1.339448066
wavelet-LHH	glrlm	ShortRunHighGrayLevelEmphasis	140.2414088
wavelet-LHH	glrlm	RunLengthNonUniformity	166.8312228
wavelet-LHH	glrlm	ShortRunEmphasis	0.930766784
wavelet-LHH	glrlm	LongRunHighGrayLevelEmphasis	200.3859365
wavelet-LHH	glrlm	RunPercentage	0.908324552
wavelet-LHH	glrlm	LongRunLowGrayLevelEmphasis	0.016805872
wavelet-LHH	glrlm	RunEntropy	3.724056186
wavelet-LHH	glrlm	HighGrayLevelRunEmphasis	150.4369555
wavelet-LHH	glrlm	RunLengthNonUniformityNormalized	0.836234637
wavelet-LHH	glszm	GrayLevelVariance	15.41804408
wavelet-LHH	glszm	ZoneVariance	45.33815427
wavelet-LHH	glszm	GrayLevelNonUniformityNormalized	0.090449954
wavelet-LHH	glszm	SizeZoneNonUniformityNormalized	0.396235078
wavelet-LHH	glszm	SizeZoneNonUniformity	26.15151515
wavelet-LHH	glszm	GrayLevelNonUniformity	5.96969697
wavelet-LHH	glszm	LargeAreaEmphasis	56.34848485
wavelet-LHH	glszm	SmallAreaHighGrayLevelEmphasis	101.8204102
wavelet-LHH	glszm	ZonePercentage	0.301369863
wavelet-LHH	glszm	LargeAreaLowGrayLevelEmphasis	0.44113009
wavelet-LHH	glszm	LargeAreaHighGrayLevelEmphasis	7837.363636
wavelet-LHH	glszm	HighGrayLevelZoneEmphasis	154.0151515
wavelet-LHH	glszm	SmallAreaEmphasis	0.650886545
wavelet-LHH	glszm	LowGrayLevelZoneEmphasis	0.027768549
wavelet-LHH	glszm	ZoneEntropy	4.8604704
wavelet-LHH	glszm	SmallAreaLowGrayLevelEmphasis	0.024639241
wavelet-LHH	ngtdm	Coarseness	0.027637176

wavelet-LHH	ngtdm	Complexity	239.5754783
wavelet-LHH	ngtdm	Strength	3.08961357
wavelet-LHH	ngtdm	Contrast	0.09679732
wavelet-LHH	ngtdm	Busyness	0.150180223
wavelet-LLH	gldm	GrayLevelVariance	43.77510894
wavelet-LLH	gldm	HighGrayLevelEmphasis	183.7488584
wavelet-LLH	gldm	DependenceEntropy	5.985522166
wavelet-LLH	gldm	DependenceNonUniformity	44.8630137
wavelet-LLH	gldm	GrayLevelNonUniformity	16.44292237
wavelet-LLH	gldm	SmallDependenceEmphasis	0.357490864
wavelet-LLH	gldm	SmallDependenceHighGrayLevelEmphasis	97.22011124
wavelet-LLH	gldm	DependenceNonUniformityNormalized	0.204853944
wavelet-LLH	gldm	LargeDependenceEmphasis	10.82648402
wavelet-LLH	gldm	LargeDependenceLowGrayLevelEmphasis	0.137851208
wavelet-LLH	gldm	DependenceVariance	2.99159734
wavelet-LLH	gldm	LargeDependenceHighGrayLevelEmphasis	1255.420091
wavelet-LLH	gldm	SmallDependenceLowGrayLevelEmphasis	0.009476202
wavelet-LLH	gldm	LowGrayLevelEmphasis	0.017815661
wavelet-LLH	glcm	JointAverage	11.18626336
wavelet-LLH	glcm	SumAverage	22.37252671
wavelet-LLH	glcm	JointEntropy	6.719774797
wavelet-LLH	glcm	ClusterShade	1707.209565
wavelet-LLH	glcm	MaximumProbability	0.032353434
wavelet-LLH	glcm	Idmn	0.969321552
wavelet-LLH	glcm	JointEnergy	0.012443457
wavelet-LLH	glcm	Contrast	59.70910409
wavelet-LLH	glcm	DifferenceEntropy	3.455910442
wavelet-LLH	glcm	InverseVariance	0.258727788
wavelet-LLH	glcm	DifferenceVariance	34.11461921
wavelet-LLH	glcm	Idn	0.903891254
wavelet-LLH	glcm	Idm	0.253588094
wavelet-LLH	glcm	Correlation	0.16074211
wavelet-LLH	glcm	Autocorrelation	133.144635
wavelet-LLH	glcm	SumEntropy	4.488115803
wavelet-LLH	glcm	SumSquares	37.80714358
wavelet-LLH	glcm	ClusterProminence	64994.85801
wavelet-LLH	glcm	Imc2	0.952517082
wavelet-LLH	glcm	Imc1	-0.309949551
wavelet-LLH	glcm	DifferenceAverage	4.787695487
wavelet-LLH	glcm	Id	0.33775886
wavelet-LLH	glcm	ClusterTendency	91.51947024
wavelet-LLH	firstorder	InterquartileRange	130.1261544
wavelet-LLH	firstorder	Skewness	1.839180483
wavelet-LLH	firstorder	Uniformity	0.075081837
wavelet-LLH	firstorder	Median	-31.46888023
wavelet-LLH	firstorder	Energy	5988383.872
wavelet-LLH	firstorder	RobustMeanAbsoluteDeviation	57.93448207
wavelet-LLH	firstorder	MeanAbsoluteDeviation	113.2643773
wavelet-LLH	firstorder	TotalEnergy	161686364.5
wavelet-LLH	firstorder	Maximum	677.6236698
wavelet-LLH	firstorder	RootMeanSquared	165.3608738
wavelet-LLH	firstorder	90Percentile	241.7847248
wavelet-LLH	firstorder	Minimum	-250.5477395
wavelet-LLH	firstorder	Entropy	4.152419114
wavelet-LLH	firstorder	Range	928.1714093

wavelet-LLH	firstorder	Variance	27279.02666
wavelet-LLH	firstorder	10Percentile	-135.4044205
wavelet-LLH	firstorder	Kurtosis	6.357560259
wavelet-LLH	firstorder	Mean	8.074151946
wavelet-LLH	glrlm	ShortRunLowGrayLevelEmphasis	0.017576539
wavelet-LLH	glrlm	GrayLevelVariance	45.95246204
wavelet-LLH	glrlm	LowGrayLevelRunEmphasis	0.018222239
wavelet-LLH	glrlm	GrayLevelNonUniformityNormalized	0.072502055
wavelet-LLH	glrlm	RunVariance	0.094901338
wavelet-LLH	glrlm	GrayLevelNonUniformity	14.79974178
wavelet-LLH	glrlm	LongRunEmphasis	1.265923981
wavelet-LLH	glrlm	ShortRunHighGrayLevelEmphasis	182.8338047
wavelet-LLH	glrlm	RunLengthNonUniformity	180.1059381
wavelet-LLH	glrlm	ShortRunEmphasis	0.948487598
wavelet-LLH	glrlm	LongRunHighGrayLevelEmphasis	219.4139864
wavelet-LLH	glrlm	RunPercentage	0.930804355
wavelet-LLH	glrlm	LongRunLowGrayLevelEmphasis	0.021626458
wavelet-LLH	glrlm	RunEntropy	4.474637774
wavelet-LLH	glrlm	HighGrayLevelRunEmphasis	189.0429362
wavelet-LLH	glrlm	RunLengthNonUniformityNormalized	0.87849217
wavelet-LLH	glszm	GrayLevelVariance	73.23630602
wavelet-LLH	glszm	ZoneVariance	7.774219104
wavelet-LLH	glszm	GrayLevelNonUniformityNormalized	0.051833409
wavelet-LLH	glszm	SizeZoneNonUniformityNormalized	0.390448167
wavelet-LLH	glszm	SizeZoneNonUniformity	36.70212766
wavelet-LLH	glszm	GrayLevelNonUniformity	4.872340426
wavelet-LLH	glszm	LargeAreaEmphasis	13.20212766
wavelet-LLH	glszm	SmallAreaHighGrayLevelEmphasis	198.9766289
wavelet-LLH	glszm	ZonePercentage	0.429223744
wavelet-LLH	glszm	LargeAreaLowGrayLevelEmphasis	0.16597458
wavelet-LLH	glszm	LargeAreaHighGrayLevelEmphasis	1623.117021
wavelet-LLH	glszm	HighGrayLevelZoneEmphasis	258.6595745
wavelet-LLH	glszm	SmallAreaEmphasis	0.644757064
wavelet-LLH	glszm	LowGrayLevelZoneEmphasis	0.024418592
wavelet-LLH	glszm	ZoneEntropy	5.739384698
wavelet-LLH	glszm	SmallAreaLowGrayLevelEmphasis	0.019573381
wavelet-LLH	ngtdm	Coarseness	0.026989055
wavelet-LLH	ngtdm	Complexity	1855.564986
wavelet-LLH	ngtdm	Strength	17.69063965
wavelet-LLH	ngtdm	Contrast	0.29904935
wavelet-LLH	ngtdm	Busyness	0.101232601
wavelet-LLL	gldm	GrayLevelVariance	121.7679781
wavelet-LLL	gldm	HighGrayLevelEmphasis	1108.703196
wavelet-LLL	gldm	DependenceEntropy	6.177905227
wavelet-LLL	gldm	DependenceNonUniformity	75.79452055
wavelet-LLL	gldm	GrayLevelNonUniformity	7.484018265
wavelet-LLL	gldm	SmallDependenceEmphasis	0.571982496
wavelet-LLL	gldm	SmallDependenceHighGrayLevelEmphasis	538.6668176
wavelet-LLL	gldm	DependenceNonUniformityNormalized	0.346093701
wavelet-LLL	gldm	LargeDependenceEmphasis	4.726027397
wavelet-LLL	gldm	LargeDependenceLowGrayLevelEmphasis	0.018560243
wavelet-LLL	gldm	DependenceVariance	1.203978232
wavelet-LLL	gldm	LargeDependenceHighGrayLevelEmphasis	6680.502283
wavelet-LLL	gldm	SmallDependenceLowGrayLevelEmphasis	0.006698038
wavelet-LLL	gldm	LowGrayLevelEmphasis	0.008812936

wavelet-LLL	glcm	JointAverage	33.4057249
wavelet-LLL	glcm	SumAverage	66.8114498
wavelet-LLL	glcm	JointEntropy	7.673149544
wavelet-LLL	glcm	ClusterShade	-2692.886395
wavelet-LLL	glcm	MaximumProbability	0.015809133
wavelet-LLL	glcm	Idmn	0.973559912
wavelet-LLL	glcm	JointEnergy	0.005554512
wavelet-LLL	glcm	Contrast	119.2797682
wavelet-LLL	glcm	DifferenceEntropy	4.230072921
wavelet-LLL	glcm	InverseVariance	0.155726272
wavelet-LLL	glcm	DifferenceVariance	49.87527165
wavelet-LLL	glcm	Idn	0.895577436
wavelet-LLL	glcm	Idm	0.145584082
wavelet-LLL	glcm	Correlation	0.393487153
wavelet-LLL	glcm	Autocorrelation	1155.910341
wavelet-LLL	glcm	SumEntropy	5.532845202
wavelet-LLL	glcm	SumSquares	99.44230945
wavelet-LLL	glcm	ClusterProminence	253129.6238
wavelet-LLL	glcm	Imc2	0.993473008
wavelet-LLL	glcm	Imc1	-0.443185195
wavelet-LLL	glcm	DifferenceAverage	8.128449496
wavelet-LLL	glcm	Id	0.22820956
wavelet-LLL	glcm	ClusterTendency	278.4894696
wavelet-LLL	firstorder	InterquartileRange	383.5263615
wavelet-LLL	firstorder	Skewness	-0.480409093
wavelet-LLL	firstorder	Uniformity	0.034173599
wavelet-LLL	firstorder	Median	-176.6804362
wavelet-LLL	firstorder	Energy	27989260.77
wavelet-LLL	firstorder	RobustMeanAbsoluteDeviation	159.6673196
wavelet-LLL	firstorder	MeanAbsoluteDeviation	223.0840845
wavelet-LLL	firstorder	TotalEnergy	755710040.8
wavelet-LLL	firstorder	Maximum	594.8182236
wavelet-LLL	firstorder	RootMeanSquared	357.4980331
wavelet-LLL	firstorder	90Percentile	48.52743279
wavelet-LLL	firstorder	Minimum	-991.9556113
wavelet-LLL	firstorder	Entropy	5.145130974
wavelet-LLL	firstorder	Range	1586.773835
wavelet-LLL	firstorder	Variance	76335.97464
wavelet-LLL	firstorder	10Percentile	-621.3794269
wavelet-LLL	firstorder	Kurtosis	3.070468925
wavelet-LLL	firstorder	Mean	-226.8675143
wavelet-LLL	glrlm	ShortRunLowGrayLevelEmphasis	0.008916679
wavelet-LLL	glrlm	GrayLevelVariance	122.9448896
wavelet-LLL	glrlm	LowGrayLevelRunEmphasis	0.009005393
wavelet-LLL	glrlm	GrayLevelNonUniformityNormalized	0.03344101
wavelet-LLL	glrlm	RunVariance	0.037403052
wavelet-LLL	glrlm	GrayLevelNonUniformity	7.077830364
wavelet-LLL	glrlm	LongRunEmphasis	1.109908766
wavelet-LLL	glrlm	ShortRunHighGrayLevelEmphasis	1061.217138
wavelet-LLL	glrlm	RunLengthNonUniformity	198.1856578
wavelet-LLL	glrlm	ShortRunEmphasis	0.974629198
wavelet-LLL	glrlm	LongRunHighGrayLevelEmphasis	1255.136176
wavelet-LLL	glrlm	RunPercentage	0.966280295
wavelet-LLL	glrlm	LongRunLowGrayLevelEmphasis	0.009365658
wavelet-LLL	glrlm	RunEntropy	5.292669765

wavelet-LLL	glrlm	HighGrayLevelRunEmphasis	1097.324257
wavelet-LLL	glrlm	RunLengthNonUniformityNormalized	0.935804949
wavelet-LLL	glszm	GrayLevelVariance	135.0131422
wavelet-LLL	glszm	ZoneVariance	2.135538584
wavelet-LLL	glszm	GrayLevelNonUniformityNormalized	0.029160881
wavelet-LLL	glszm	SizeZoneNonUniformityNormalized	0.565959135
wavelet-LLL	glszm	SizeZoneNonUniformity	80.36619718
wavelet-LLL	glszm	GrayLevelNonUniformity	4.14084507
wavelet-LLL	glszm	LargeAreaEmphasis	4.514084507
wavelet-LLL	glszm	SmallAreaHighGrayLevelEmphasis	700.010804
wavelet-LLL	glszm	ZonePercentage	0.648401826
wavelet-LLL	glszm	LargeAreaLowGrayLevelEmphasis	0.019244058
wavelet-LLL	glszm	LargeAreaHighGrayLevelEmphasis	6368.802817
wavelet-LLL	glszm	HighGrayLevelZoneEmphasis	965.0211268
wavelet-LLL	glszm	SmallAreaEmphasis	0.780573161
wavelet-LLL	glszm	LowGrayLevelZoneEmphasis	0.011346427
wavelet-LLL	glszm	ZoneEntropy	5.939271264
wavelet-LLL	glszm	SmallAreaLowGrayLevelEmphasis	0.009790273
wavelet-LLL	ngtdm	Coarseness	0.032674755
wavelet-LLL	ngtdm	Complexity	4914.941723
wavelet-LLL	ngtdm	Strength	28.82514747
wavelet-LLL	ngtdm	Contrast	0.607273526
wavelet-LLL	ngtdm	Busyness	0.019871275
wavelet-HHL	gldm	GrayLevelVariance	5.8479181
wavelet-HHL	gldm	HighGrayLevelEmphasis	138.4657534
wavelet-HHL	gldm	DependenceEntropy	5.476482578
wavelet-HHL	gldm	DependenceNonUniformity	29.14611872
wavelet-HHL	gldm	GrayLevelNonUniformity	34.75342466
wavelet-HHL	gldm	SmallDependenceEmphasis	0.249812026
wavelet-HHL	gldm	SmallDependenceHighGrayLevelEmphasis	37.33832809
wavelet-HHL	gldm	DependenceNonUniformityNormalized	0.1330873
wavelet-HHL	gldm	LargeDependenceEmphasis	22.00456621
wavelet-HHL	gldm	LargeDependenceLowGrayLevelEmphasis	0.173741336
wavelet-HHL	gldm	DependenceVariance	6.475928358
wavelet-HHL	gldm	LargeDependenceHighGrayLevelEmphasis	2956.721461
wavelet-HHL	gldm	SmallDependenceLowGrayLevelEmphasis	0.007214661
wavelet-HHL	gldm	LowGrayLevelEmphasis	0.013192109
wavelet-HHL	glcm	JointAverage	11.50498054
wavelet-HHL	glcm	SumAverage	23.00996108
wavelet-HHL	glcm	JointEntropy	5.386719923
wavelet-HHL	glcm	ClusterShade	9.409450668
wavelet-HHL	glcm	MaximumProbability	0.079098278
wavelet-HHL	glcm	Idmn	0.984393387
wavelet-HHL	glcm	JointEnergy	0.035118696
wavelet-HHL	glcm	Contrast	10.19878195
wavelet-HHL	glcm	DifferenceEntropy	2.631625379
wavelet-HHL	glcm	InverseVariance	0.390055524
wavelet-HHL	glcm	DifferenceVariance	5.445730267
wavelet-HHL	glcm	Idn	0.923652877
wavelet-HHL	glcm	Idm	0.395317344
wavelet-HHL	glcm	Correlation	-0.044707702
wavelet-HHL	glcm	Autocorrelation	132.1827039
wavelet-HHL	glcm	SumEntropy	3.341905249
wavelet-HHL	glcm	SumSquares	4.915747879
wavelet-HHL	glcm	ClusterProminence	833.373857

wavelet-HHL	glcm	Imc2	0.796007599
wavelet-HHL	glcm	Imc1	-0.172860697
wavelet-HHL	glcm	DifferenceAverage	2.158557942
wavelet-HHL	glcm	Id	0.46285918
wavelet-HHL	glcm	ClusterTendency	9.464209567
wavelet-HHL	firstorder	InterquartileRange	57.50025319
wavelet-HHL	firstorder	Skewness	0.347427784
wavelet-HHL	firstorder	Uniformity	0.158691437
wavelet-HHL	firstorder	Median	0.909364549
wavelet-HHL	firstorder	Energy	793013.0248
wavelet-HHL	firstorder	RobustMeanAbsoluteDeviation	23.55128627
wavelet-HHL	firstorder	MeanAbsoluteDeviation	40.65555665
wavelet-HHL	firstorder	TotalEnergy	21411351.67
wavelet-HHL	firstorder	Maximum	310.5092795
wavelet-HHL	firstorder	RootMeanSquared	60.17527765
wavelet-HHL	firstorder	90Percentile	60.32327203
wavelet-HHL	firstorder	Minimum	-251.7009232
wavelet-HHL	firstorder	Entropy	3.075178551
wavelet-HHL	firstorder	Range	562.2102027
wavelet-HHL	firstorder	Variance	3620.873235
wavelet-HHL	firstorder	10Percentile	-64.095261
wavelet-HHL	firstorder	Kurtosis	8.371050424
wavelet-HHL	firstorder	Mean	0.436812176
wavelet-HHL	glrlm	ShortRunLowGrayLevelEmphasis	0.013220564
wavelet-HHL	glrlm	GrayLevelVariance	6.451202115
wavelet-HHL	glrlm	LowGrayLevelRunEmphasis	0.013891603
wavelet-HHL	glrlm	GrayLevelNonUniformityNormalized	0.14760798
wavelet-HHL	glrlm	RunVariance	0.146625222
wavelet-HHL	glrlm	GrayLevelNonUniformity	28.67508181
wavelet-HHL	glrlm	LongRunEmphasis	1.420838451
wavelet-HHL	glrlm	ShortRunHighGrayLevelEmphasis	127.4118243
wavelet-HHL	glrlm	RunLengthNonUniformity	155.4877917
wavelet-HHL	glrlm	ShortRunEmphasis	0.913619939
wavelet-HHL	glrlm	LongRunHighGrayLevelEmphasis	195.4912225
wavelet-HHL	glrlm	RunPercentage	0.88689849
wavelet-HHL	glrlm	LongRunLowGrayLevelEmphasis	0.017143034
wavelet-HHL	glrlm	RunEntropy	3.642007169
wavelet-HHL	glrlm	HighGrayLevelRunEmphasis	139.0074588
wavelet-HHL	glrlm	RunLengthNonUniformityNormalized	0.799344465
wavelet-HHL	glszm	GrayLevelVariance	16.68305556
wavelet-HHL	glszm	ZoneVariance	55.09416667
wavelet-HHL	glszm	GrayLevelNonUniformityNormalized	0.089444444
wavelet-HHL	glszm	SizeZoneNonUniformityNormalized	0.406666667
wavelet-HHL	glszm	SizeZoneNonUniformity	24.4
wavelet-HHL	glszm	GrayLevelNonUniformity	5.366666667
wavelet-HHL	glszm	LargeAreaEmphasis	68.41666667
wavelet-HHL	glszm	SmallAreaHighGrayLevelEmphasis	102.0967497
wavelet-HHL	glszm	ZonePercentage	0.273972603
wavelet-HHL	glszm	LargeAreaLowGrayLevelEmphasis	0.540412072
wavelet-HHL	glszm	LargeAreaHighGrayLevelEmphasis	9241.433333
wavelet-HHL	glszm	HighGrayLevelZoneEmphasis	149.3166667
wavelet-HHL	glszm	SmallAreaEmphasis	0.657164168
wavelet-HHL	glszm	LowGrayLevelZoneEmphasis	0.027185362
wavelet-HHL	glszm	ZoneEntropy	4.837907837
wavelet-HHL	glszm	SmallAreaLowGrayLevelEmphasis	0.024206292

wavelet-HHL	ngtdm	Coarseness	0.030126106
wavelet-HHL	ngtdm	Complexity	267.499143
wavelet-HHL	ngtdm	Strength	4.260020997
wavelet-HHL	ngtdm	Contrast	0.059199851
wavelet-HHL	ngtdm	Busyness	0.112641666
wavelet-HLH	gldm	GrayLevelVariance	2.942557495
wavelet-HLH	gldm	HighGrayLevelEmphasis	33.62100457
wavelet-HLH	gldm	DependenceEntropy	5.240920557
wavelet-HLH	gldm	DependenceNonUniformity	27.38356164
wavelet-HLH	gldm	GrayLevelNonUniformity	39.75799087
wavelet-HLH	gldm	SmallDependenceEmphasis	0.182375743
wavelet-HLH	gldm	SmallDependenceHighGrayLevelEmphasis	6.738218784
wavelet-HLH	gldm	DependenceNonUniformityNormalized	0.125039094
wavelet-HLH	gldm	LargeDependenceEmphasis	24.96347032
wavelet-HLH	gldm	LargeDependenceLowGrayLevelEmphasis	0.965111033
wavelet-HLH	gldm	DependenceVariance	6.185692542
wavelet-HLH	gldm	LargeDependenceHighGrayLevelEmphasis	790.5159817
wavelet-HLH	gldm	SmallDependenceLowGrayLevelEmphasis	0.014731071
wavelet-HLH	gldm	LowGrayLevelEmphasis	0.060579869
wavelet-HLH	glcm	JointAverage	5.504508887
wavelet-HLH	glcm	SumAverage	11.00901777
wavelet-HLH	glcm	JointEntropy	5.155990108
wavelet-HLH	glcm	ClusterShade	-0.26960186
wavelet-HLH	glcm	MaximumProbability	0.094798262
wavelet-HLH	glcm	Idmn	0.958590156
wavelet-HLH	glcm	JointEnergy	0.03991349
wavelet-HLH	glcm	Contrast	5.818496308
wavelet-HLH	glcm	DifferenceEntropy	2.428597256
wavelet-HLH	glcm	InverseVariance	0.391963628
wavelet-HLH	glcm	DifferenceVariance	2.442496742
wavelet-HLH	glcm	Idn	0.870533003
wavelet-HLH	glcm	Idm	0.419653798
wavelet-HLH	glcm	Correlation	-0.034218555
wavelet-HLH	glcm	Autocorrelation	30.19910447
wavelet-HLH	glcm	SumEntropy	3.150859757
wavelet-HLH	glcm	SumSquares	2.806949135
wavelet-HLH	glcm	ClusterProminence	107.6309682
wavelet-HLH	glcm	Imc2	0.654203399
wavelet-HLH	glcm	Imc1	-0.106006744
wavelet-HLH	glcm	DifferenceAverage	1.812433657
wavelet-HLH	glcm	Id	0.486017055
wavelet-HLH	glcm	ClusterTendency	5.409300233
wavelet-HLH	firstorder	InterquartileRange	48.81430619
wavelet-HLH	firstorder	Skewness	-0.073441523
wavelet-HLH	firstorder	Uniformity	0.181543337
wavelet-HLH	firstorder	Median	5.974657662
wavelet-HLH	firstorder	Energy	396770.7223
wavelet-HLH	firstorder	RobustMeanAbsoluteDeviation	21.73205309
wavelet-HLH	firstorder	MeanAbsoluteDeviation	32.86407132
wavelet-HLH	firstorder	TotalEnergy	10712809.5
wavelet-HLH	firstorder	Maximum	127.9070053
wavelet-HLH	firstorder	RootMeanSquared	42.56452112
wavelet-HLH	firstorder	90Percentile	52.16743241
wavelet-HLH	firstorder	Minimum	-124.645338
wavelet-HLH	firstorder	Entropy	2.75929453

wavelet-HLH	firstorder	Range	252.5523433
wavelet-HLH	firstorder	Variance	1809.132337
wavelet-HLH	firstorder	10Percentile	-53.97029619
wavelet-HLH	firstorder	Kurtosis	3.584530676
wavelet-HLH	firstorder	Mean	1.614348444
wavelet-HLH	glrlm	ShortRunLowGrayLevelEmphasis	0.058919426
wavelet-HLH	glrlm	GrayLevelVariance	3.18590917
wavelet-HLH	glrlm	LowGrayLevelRunEmphasis	0.063264918
wavelet-HLH	glrlm	GrayLevelNonUniformityNormalized	0.170193342
wavelet-HLH	glrlm	RunVariance	0.165894851
wavelet-HLH	glrlm	GrayLevelNonUniformity	32.49887014
wavelet-HLH	glrlm	LongRunEmphasis	1.486868218
wavelet-HLH	glrlm	ShortRunHighGrayLevelEmphasis	30.67585329
wavelet-HLH	glrlm	RunLengthNonUniformity	147.8843936
wavelet-HLH	glrlm	ShortRunEmphasis	0.90049023
wavelet-HLH	glrlm	LongRunHighGrayLevelEmphasis	49.46039158
wavelet-HLH	glrlm	RunPercentage	0.871794872
wavelet-HLH	glrlm	LongRunLowGrayLevelEmphasis	0.083567569
wavelet-HLH	glrlm	RunEntropy	3.375423866
wavelet-HLH	glrlm	HighGrayLevelRunEmphasis	33.85779523
wavelet-HLH	glrlm	RunLengthNonUniformityNormalized	0.772816617
wavelet-HLH	glszm	GrayLevelVariance	6.578231293
wavelet-HLH	glszm	ZoneVariance	132.0731293
wavelet-HLH	glszm	GrayLevelNonUniformityNormalized	0.124716553
wavelet-HLH	glszm	SizeZoneNonUniformityNormalized	0.323129252
wavelet-HLH	glszm	SizeZoneNonUniformity	13.57142857
wavelet-HLH	glszm	GrayLevelNonUniformity	5.238095238
wavelet-HLH	glszm	LargeAreaEmphasis	159.2619048
wavelet-HLH	glszm	SmallAreaHighGrayLevelEmphasis	22.58116768
wavelet-HLH	glszm	ZonePercentage	0.191780822
wavelet-HLH	glszm	LargeAreaLowGrayLevelEmphasis	5.42975259
wavelet-HLH	glszm	LargeAreaHighGrayLevelEmphasis	5280.214286
wavelet-HLH	glszm	HighGrayLevelZoneEmphasis	37.61904762
wavelet-HLH	glszm	SmallAreaEmphasis	0.578352705
wavelet-HLH	glszm	LowGrayLevelZoneEmphasis	0.103023288
wavelet-HLH	glszm	ZoneEntropy	4.403989446
wavelet-HLH	glszm	SmallAreaLowGrayLevelEmphasis	0.048361431
wavelet-HLH	ngtdm	Coarseness	0.027391489
wavelet-HLH	ngtdm	Complexity	65.40084856
wavelet-HLH	ngtdm	Strength	0.937299825
wavelet-HLH	ngtdm	Contrast	0.075433011
wavelet-HLH	ngtdm	Busyness	0.540361195

Image type	Feature Class	Feature Name	11: RTSTRUCT: C99-DNU_segment_ITV LLL
general	info	BoundingBox	(0, 1, 1, 7, 7, 5)
general	info	EnabledImageTypes	{u'Original': {}, u'Wavelet': {}}
general	info	GeneralSettings	{'distances': [1], 'additionalInfo': True, 'force2D': False, 'interpolator': 'sitkBSpline', 'resampledPixelSpacing': [3.0], 'label': 1, 'normalizeScale': 1, 'normalize': False, 'force2Ddimension': 0, 'removeOutliers': None, 'minimumROISize': None, u'binWidth': 25.0, 'minimumROIDimensions': 1, u'correctMask': True, 'preCrop': False, u'symmetricalGLCM': True, 'resegmentRange': None, 'padDistance': 5}
general	info	ImageHash	820cf1b0ebcde051b80ab7bd37f54451116a3cfa
general	info	ImageSpacing	(0.9765625000000002, 0.9765625000000002, 2.0)
general	info	MaskHash	6688eac8574a2e734d3d16a17182aea1faad7cf8
general	info	NumpyVersion	1.13.1
general	info	PyWaveletVersion	0.5.2
general	info	SimpleITKVersion	1.1.0
general	info	Version	1.3.0.post102+g1b0eb74
general	info	VolumeNum	1
general	info	VoxelNum	108
original	shape	Maximum3DDiameter	22.44994432
original	shape	Maximum2DDiameterSlice	20.1246118
original	shape	Sphericity	0.881798369
original	shape	MinorAxis	16.15068387
original	shape	Elongation	0.820449784
original	shape	SurfaceVolumeRatio	0.383871315
original	shape	Volume	2916
original	shape	MajorAxis	19.68515831
original	shape	SurfaceArea	1119.368753
original	shape	Flatness	0.670513334
original	shape	LeastAxis	13.19916112
original	shape	Maximum2DDiameterColumn	18
original	shape	Maximum2DDiameterRow	21.63330765
original	gldm	GrayLevelVariance	81.71879287
original	gldm	HighGrayLevelEmphasis	750.037037
original	gldm	DependenceEntropy	5.629434956
original	gldm	DependenceNonUniformity	30.44444444
original	gldm	GrayLevelNonUniformity	6.611111111
original	gldm	SmallDependenceEmphasis	0.537815614
original	gldm	SmallDependenceHighGrayLevelEmphasis	294.7568754
original	gldm	DependenceNonUniformityNormalized	0.281893004
original	gldm	LargeDependenceEmphasis	12
original	gldm	LargeDependenceLowGrayLevelEmphasis	0.022887647
original	gldm	DependenceVariance	4.888888889
original	gldm	LargeDependenceHighGrayLevelEmphasis	13238.57407

original	gldm	SmallDependenceLowGrayLevelEmphasis	0.011975768
original	gldm	LowGrayLevelEmphasis	0.012571114
original	glcm	JointAverage	27.96679941
original	glcm	SumAverage	55.93359882
original	glcm	JointEntropy	6.345550738
original	glcm	ClusterShade	-1750.648301
original	glcm	MaximumProbability	0.050464276
original	glcm	Idmn	0.930318639
original	glcm	JointEnergy	0.015769056
original	glcm	Contrast	116.2242794
original	glcm	DifferenceEntropy	4.045646516
original	glcm	InverseVariance	0.198535256
original	glcm	DifferenceVariance	55.79303059
original	glcm	Idn	0.844894381
original	glcm	Idm	0.215463865
original	glcm	Correlation	0.158389493
original	glcm	Autocorrelation	793.038339
original	glcm	SumEntropy	4.684556418
original	glcm	SumSquares	68.96696317
original	glcm	ClusterProminence	76002.91334
original	glcm	Imc2	0.987918495
original	glcm	Imc1	-0.457183857
original	glcm	DifferenceAverage	7.733197164
original	glcm	Id	0.284814056
original	glcm	ClusterTendency	159.6435733
original	firstorder	InterquartileRange	366
original	firstorder	Skewness	-0.772493775
original	firstorder	Uniformity	0.061213992
original	firstorder	Median	-100
original	firstorder	Energy	9465300
original	firstorder	RobustMeanAbsoluteDeviation	153.1179016
original	firstorder	MeanAbsoluteDeviation	195.4128944
original	firstorder	TotalEnergy	255563100
original	firstorder	Maximum	67
original	firstorder	RootMeanSquared	296.0433527
original	firstorder	90Percentile	29.6
original	firstorder	Minimum	-801
original	firstorder	Entropy	4.452841682
original	firstorder	Range	868
original	firstorder	Variance	50763.44307
original	firstorder	10Percentile	-525.6
original	firstorder	Kurtosis	2.496036864
original	firstorder	Mean	-192.037037
original	glrlm	ShortRunLowGrayLevelEmphasis	0.013318423
original	glrlm	GrayLevelVariance	82.57514469
original	glrlm	LowGrayLevelRunEmphasis	0.01336842
original	glrlm	GrayLevelNonUniformityNormalized	0.055689765
original	glrlm	RunVariance	0.075973171
original	glrlm	GrayLevelNonUniformity	5.632571637
original	glrlm	LongRunEmphasis	1.219550187
original	glrlm	ShortRunHighGrayLevelEmphasis	673.9683035
original	glrlm	RunLengthNonUniformity	89.29152927
original	glrlm	ShortRunEmphasis	0.951944656

original	glrlm	LongRunHighGrayLevelEmphasis	966.673071
original	glrlm	RunPercentage	0.935897436
original	glrlm	LongRunLowGrayLevelEmphasis	0.013592927
original	glrlm	RunEntropy	4.747093148
original	glrlm	HighGrayLevelRunEmphasis	726.3601672
original	glrlm	RunLengthNonUniformityNormalized	0.882472321
original	glszm	GrayLevelVariance	81.96484375
original	glszm	ZoneVariance	4.52734375
original	glszm	GrayLevelNonUniformityNormalized	0.038574219
original	glszm	SizeZoneNonUniformityNormalized	0.671386719
original	glszm	SizeZoneNonUniformity	42.96875
original	glszm	GrayLevelNonUniformity	2.46875
original	glszm	LargeAreaEmphasis	7.375
original	glszm	SmallAreaHighGrayLevelEmphasis	442.9841305
original	glszm	ZonePercentage	0.592592593
original	glszm	LargeAreaLowGrayLevelEmphasis	0.026709725
original	glszm	LargeAreaHighGrayLevelEmphasis	7582.90625
original	glszm	HighGrayLevelZoneEmphasis	568.71875
original	glszm	SmallAreaEmphasis	0.840258264
original	glszm	LowGrayLevelZoneEmphasis	0.020383148
original	glszm	ZoneEntropy	5.159774414
original	glszm	SmallAreaLowGrayLevelEmphasis	0.020093228
original	ngtdm	Coarseness	0.027992519
original	ngtdm	Complexity	2667.800315
original	ngtdm	Strength	17.09700721
original	ngtdm	Contrast	1.219507793
original	ngtdm	Busyness	0.040340586
wavelet-HHH	gldm	GrayLevelVariance	22.33024691
wavelet-HHH	gldm	HighGrayLevelEmphasis	298.2592593
wavelet-HHH	gldm	DependenceEntropy	5.405363373
wavelet-HHH	gldm	DependenceNonUniformity	33.01851852
wavelet-HHH	gldm	GrayLevelNonUniformity	7.259259259
wavelet-HHH	gldm	SmallDependenceEmphasis	0.486027442
wavelet-HHH	gldm	SmallDependenceHighGrayLevelEmphasis	144.1959399
wavelet-HHH	gldm	DependenceNonUniformityNormalized	0.305727023
wavelet-HHH	gldm	LargeDependenceEmphasis	5.592592593
wavelet-HHH	gldm	LargeDependenceLowGrayLevelEmphasis	0.031215176
wavelet-HHH	gldm	DependenceVariance	1.367283951
wavelet-HHH	gldm	LargeDependenceHighGrayLevelEmphasis	1740.037037
wavelet-HHH	gldm	SmallDependenceLowGrayLevelEmphasis	0.012636155
wavelet-HHH	gldm	LowGrayLevelEmphasis	0.014694506
wavelet-HHH	glcm	JointAverage	16.4848446
wavelet-HHH	glcm	SumAverage	32.9696892
wavelet-HHH	glcm	JointEntropy	6.503740938
wavelet-HHH	glcm	ClusterShade	-57.5350488
wavelet-HHH	glcm	MaximumProbability	0.031590348
wavelet-HHH	glcm	Idmn	0.955978845
wavelet-HHH	glcm	JointEnergy	0.012475715
wavelet-HHH	glcm	Contrast	43.64331905
wavelet-HHH	glcm	DifferenceEntropy	3.571737112
wavelet-HHH	glcm	InverseVariance	0.189939032
wavelet-HHH	glcm	DifferenceVariance	17.12334938
wavelet-HHH	glcm	Idn	0.862238028

wavelet-HHH	glcm	Idm	0.181279008
wavelet-HHH	glcm	Correlation	-0.027715223
wavelet-HHH	glcm	Autocorrelation	271.2137406
wavelet-HHH	glcm	SumEntropy	4.260461331
wavelet-HHH	glcm	SumSquares	21.26579039
wavelet-HHH	glcm	ClusterProminence	6845.734509
wavelet-HHH	glcm	Imc2	0.976170902
wavelet-HHH	glcm	Imc1	-0.383297952
wavelet-HHH	glcm	DifferenceAverage	5.087689332
wavelet-HHH	glcm	Id	0.274900236
wavelet-HHH	glcm	ClusterTendency	41.41984252
wavelet-HHH	firstorder	InterquartileRange	153.1862648
wavelet-HHH	firstorder	Skewness	-0.382133102
wavelet-HHH	firstorder	Uniformity	0.067215364
wavelet-HHH	firstorder	Median	8.195818109
wavelet-HHH	firstorder	Energy	1500026.466
wavelet-HHH	firstorder	RobustMeanAbsoluteDeviation	63.49450897
wavelet-HHH	firstorder	MeanAbsoluteDeviation	92.08444283
wavelet-HHH	firstorder	TotalEnergy	40500714.58
wavelet-HHH	firstorder	Maximum	303.2806768
wavelet-HHH	firstorder	RootMeanSquared	117.8521699
wavelet-HHH	firstorder	90Percentile	128.3701114
wavelet-HHH	firstorder	Minimum	-397.6128636
wavelet-HHH	firstorder	Entropy	4.11458322
wavelet-HHH	firstorder	Range	700.8935404
wavelet-HHH	firstorder	Variance	13884.62211
wavelet-HHH	firstorder	10Percentile	-138.8643557
wavelet-HHH	firstorder	Kurtosis	3.882909882
wavelet-HHH	firstorder	Mean	2.124107954
wavelet-HHH	glrlm	ShortRunLowGrayLevelEmphasis	0.015045516
wavelet-HHH	glrlm	GrayLevelVariance	22.8793637
wavelet-HHH	glrlm	LowGrayLevelRunEmphasis	0.015164766
wavelet-HHH	glrlm	GrayLevelNonUniformityNormalized	0.066352172
wavelet-HHH	glrlm	RunVariance	0.043492998
wavelet-HHH	glrlm	GrayLevelNonUniformity	6.876378634
wavelet-HHH	glrlm	LongRunEmphasis	1.132418309
wavelet-HHH	glrlm	ShortRunHighGrayLevelEmphasis	288.2836347
wavelet-HHH	glrlm	RunLengthNonUniformity	95.62622702
wavelet-HHH	glrlm	ShortRunEmphasis	0.968613805
wavelet-HHH	glrlm	LongRunHighGrayLevelEmphasis	338.5024587
wavelet-HHH	glrlm	RunPercentage	0.959401709
wavelet-HHH	glrlm	LongRunLowGrayLevelEmphasis	0.015664654
wavelet-HHH	glrlm	RunEntropy	4.28167792
wavelet-HHH	glrlm	HighGrayLevelRunEmphasis	297.9022147
wavelet-HHH	glrlm	RunLengthNonUniformityNormalized	0.921589289
wavelet-HHH	glszm	GrayLevelVariance	30.93860562
wavelet-HHH	glszm	ZoneVariance	2.094693028
wavelet-HHH	glszm	GrayLevelNonUniformityNormalized	0.059833507
wavelet-HHH	glszm	SizeZoneNonUniformityNormalized	0.46566077
wavelet-HHH	glszm	SizeZoneNonUniformity	28.87096774
wavelet-HHH	glszm	GrayLevelNonUniformity	3.709677419
wavelet-HHH	glszm	LargeAreaEmphasis	5.129032258
wavelet-HHH	glszm	SmallAreaHighGrayLevelEmphasis	210.1371098

wavelet-HHH	glszm	ZonePercentage	0.574074074
wavelet-HHH	glszm	LargeAreaLowGrayLevelEmphasis	0.038515658
wavelet-HHH	glszm	LargeAreaHighGrayLevelEmphasis	1525.677419
wavelet-HHH	glszm	HighGrayLevelZoneEmphasis	298.4193548
wavelet-HHH	glszm	SmallAreaEmphasis	0.707431538
wavelet-HHH	glszm	LowGrayLevelZoneEmphasis	0.022646264
wavelet-HHH	glszm	ZoneEntropy	5.073080335
wavelet-HHH	glszm	SmallAreaLowGrayLevelEmphasis	0.021433099
wavelet-HHH	ngtdm	Coarseness	0.045263671
wavelet-HHH	ngtdm	Complexity	894.7789269
wavelet-HHH	ngtdm	Strength	9.20867061
wavelet-HHH	ngtdm	Contrast	0.314593974
wavelet-HHH	ngtdm	Busyness	0.057756087
wavelet-HLL	gldm	GrayLevelVariance	104.2663752
wavelet-HLL	gldm	HighGrayLevelEmphasis	547.212963
wavelet-HLL	gldm	DependenceEntropy	5.760590484
wavelet-HLL	gldm	DependenceNonUniformity	36.01851852
wavelet-HLL	gldm	GrayLevelNonUniformity	4.592592593
wavelet-HLL	gldm	SmallDependenceEmphasis	0.546489198
wavelet-HLL	gldm	SmallDependenceHighGrayLevelEmphasis	380.820409
wavelet-HLL	gldm	DependenceNonUniformityNormalized	0.333504801
wavelet-HLL	gldm	LargeDependenceEmphasis	4.777777778
wavelet-HLL	gldm	LargeDependenceLowGrayLevelEmphasis	0.031670512
wavelet-HLL	gldm	DependenceVariance	0.99691358
wavelet-HLL	gldm	LargeDependenceHighGrayLevelEmphasis	1798.990741
wavelet-HLL	gldm	SmallDependenceLowGrayLevelEmphasis	0.013419937
wavelet-HLL	gldm	LowGrayLevelEmphasis	0.015315843
wavelet-HLL	glcm	JointAverage	20.07764754
wavelet-HLL	glcm	SumAverage	40.15529507
wavelet-HLL	glcm	JointEntropy	6.819221654
wavelet-HLL	glcm	ClusterShade	2964.889545
wavelet-HLL	glcm	MaximumProbability	0.02300524
wavelet-HLL	glcm	Idmn	0.950947316
wavelet-HLL	glcm	JointEnergy	0.009519708
wavelet-HLL	glcm	Contrast	155.2023476
wavelet-HLL	glcm	DifferenceEntropy	4.106096091
wavelet-HLL	glcm	InverseVariance	0.12845326
wavelet-HLL	glcm	DifferenceVariance	72.07143305
wavelet-HLL	glcm	Idn	0.867332104
wavelet-HLL	glcm	Idm	0.139341637
wavelet-HLL	glcm	Correlation	0.116644513
wavelet-HLL	glcm	Autocorrelation	416.7878969
wavelet-HLL	glcm	SumEntropy	5.002821277
wavelet-HLL	glcm	SumSquares	91.02896343
wavelet-HLL	glcm	ClusterProminence	186032.1242
wavelet-HLL	glcm	Imc2	0.997799456
wavelet-HLL	glcm	Imc1	-0.571363221
wavelet-HLL	glcm	DifferenceAverage	8.745867505
wavelet-HLL	glcm	Id	0.22503072
wavelet-HLL	glcm	ClusterTendency	208.9135061
wavelet-HLL	firstorder	InterquartileRange	291.056665
wavelet-HLL	firstorder	Skewness	0.902275338
wavelet-HLL	firstorder	Uniformity	0.042524005

wavelet-HLL	firstorder	Median	-178.1653489
wavelet-HLL	firstorder	Energy	8403556.435
wavelet-HLL	firstorder	RobustMeanAbsoluteDeviation	135.4231268
wavelet-HLL	firstorder	MeanAbsoluteDeviation	200.5198089
wavelet-HLL	firstorder	TotalEnergy	226896023.7
wavelet-HLL	firstorder	Maximum	623.6447993
wavelet-HLL	firstorder	RootMeanSquared	278.9457075
wavelet-HLL	firstorder	90Percentile	267.827858
wavelet-HLL	firstorder	Minimum	-601.028402
wavelet-HLL	firstorder	Entropy	4.891624684
wavelet-HLL	firstorder	Range	1224.673201
wavelet-HLL	firstorder	Variance	65348.23346
wavelet-HLL	firstorder	10Percentile	-351.9853041
wavelet-HLL	firstorder	Kurtosis	3.269401672
wavelet-HLL	firstorder	Mean	-111.6354526
wavelet-HLL	glrlm	ShortRunLowGrayLevelEmphasis	0.015614783
wavelet-HLL	glrlm	GrayLevelVariance	106.3521388
wavelet-HLL	glrlm	LowGrayLevelRunEmphasis	0.015737963
wavelet-HLL	glrlm	GrayLevelNonUniformityNormalized	0.041683396
wavelet-HLL	glrlm	RunVariance	0.036196321
wavelet-HLL	glrlm	GrayLevelNonUniformity	4.339779918
wavelet-HLL	glrlm	LongRunEmphasis	1.114853386
wavelet-HLL	glrlm	ShortRunHighGrayLevelEmphasis	545.2058645
wavelet-HLL	glrlm	RunLengthNonUniformity	96.65405918
wavelet-HLL	glrlm	ShortRunEmphasis	0.971286653
wavelet-HLL	glrlm	LongRunHighGrayLevelEmphasis	594.3991196
wavelet-HLL	glrlm	RunPercentage	0.963675214
wavelet-HLL	glrlm	LongRunLowGrayLevelEmphasis	0.01623068
wavelet-HLL	glrlm	RunEntropy	5.032932834
wavelet-HLL	glrlm	HighGrayLevelRunEmphasis	555.0445155
wavelet-HLL	glrlm	RunLengthNonUniformityNormalized	0.927607358
wavelet-HLL	glszm	GrayLevelVariance	133.475607
wavelet-HLL	glszm	ZoneVariance	1.640454444
wavelet-HLL	glszm	GrayLevelNonUniformityNormalized	0.032746714
wavelet-HLL	glszm	SizeZoneNonUniformityNormalized	0.577634217
wavelet-HLL	glszm	SizeZoneNonUniformity	38.70149254
wavelet-HLL	glszm	GrayLevelNonUniformity	2.194029851
wavelet-HLL	glszm	LargeAreaEmphasis	4.23880597
wavelet-HLL	glszm	SmallAreaHighGrayLevelEmphasis	572.6841177
wavelet-HLL	glszm	ZonePercentage	0.62037037
wavelet-HLL	glszm	LargeAreaLowGrayLevelEmphasis	0.035316317
wavelet-HLL	glszm	LargeAreaHighGrayLevelEmphasis	1791.597015
wavelet-HLL	glszm	HighGrayLevelZoneEmphasis	660.4179104
wavelet-HLL	glszm	SmallAreaEmphasis	0.777868735
wavelet-HLL	glszm	LowGrayLevelZoneEmphasis	0.022137575
wavelet-HLL	glszm	ZoneEntropy	5.487658034
wavelet-HLL	glszm	SmallAreaLowGrayLevelEmphasis	0.021217727
wavelet-HLL	ngtdm	Coarseness	0.045075149
wavelet-HLL	ngtdm	Complexity	4476.587934
wavelet-HLL	ngtdm	Strength	33.14602109
wavelet-HLL	ngtdm	Contrast	0.884761285
wavelet-HLL	ngtdm	Busyness	0.035946811
wavelet-LHL	gldm	GrayLevelVariance	51.08256173

wavelet-LHL	gldm	HighGrayLevelEmphasis	333.5092593
wavelet-LHL	gldm	DependenceEntropy	5.513784724
wavelet-LHL	gldm	DependenceNonUniformity	46.66666667
wavelet-LHL	gldm	GrayLevelNonUniformity	4.537037037
wavelet-LHL	gldm	SmallDependenceEmphasis	0.651234568
wavelet-LHL	gldm	SmallDependenceHighGrayLevelEmphasis	221.2345679
wavelet-LHL	gldm	DependenceNonUniformityNormalized	0.432098765
wavelet-LHL	gldm	LargeDependenceEmphasis	2.888888889
wavelet-LHL	gldm	LargeDependenceLowGrayLevelEmphasis	0.029007948
wavelet-LHL	gldm	DependenceVariance	0.469135802
wavelet-LHL	gldm	LargeDependenceHighGrayLevelEmphasis	943.8425926
wavelet-LHL	gldm	SmallDependenceLowGrayLevelEmphasis	0.014853942
wavelet-LHL	gldm	LowGrayLevelEmphasis	0.017054393
wavelet-LHL	glcm	JointAverage	16.21086004
wavelet-LHL	glcm	SumAverage	32.42172007
wavelet-LHL	glcm	JointEntropy	6.870290769
wavelet-LHL	glcm	ClusterShade	137.273631
wavelet-LHL	glcm	MaximumProbability	0.018263625
wavelet-LHL	glcm	Idmn	0.932656477
wavelet-LHL	glcm	JointEnergy	0.008998355
wavelet-LHL	glcm	Contrast	72.78066121
wavelet-LHL	glcm	DifferenceEntropy	3.863853561
wavelet-LHL	glcm	InverseVariance	0.142428869
wavelet-LHL	glcm	DifferenceVariance	23.17145919
wavelet-LHL	glcm	Idn	0.82663227
wavelet-LHL	glcm	Idm	0.122154727
wavelet-LHL	glcm	Correlation	0.206820514
wavelet-LHL	glcm	Autocorrelation	272.4798331
wavelet-LHL	glcm	SumEntropy	4.827897298
wavelet-LHL	glcm	SumSquares	45.9790172
wavelet-LHL	glcm	ClusterProminence	28829.28609
wavelet-LHL	glcm	Imc2	0.995727553
wavelet-LHL	glcm	Imc1	-0.517782447
wavelet-LHL	glcm	DifferenceAverage	6.913934363
wavelet-LHL	glcm	Id	0.213095251
wavelet-LHL	glcm	ClusterTendency	111.1354076
wavelet-LHL	firstorder	InterquartileRange	285.1760299
wavelet-LHL	firstorder	Skewness	-0.033538258
wavelet-LHL	firstorder	Uniformity	0.042009602
wavelet-LHL	firstorder	Median	-39.80885794
wavelet-LHL	firstorder	Energy	3625890.354
wavelet-LHL	firstorder	RobustMeanAbsoluteDeviation	122.1269065
wavelet-LHL	firstorder	MeanAbsoluteDeviation	154.7474562
wavelet-LHL	firstorder	TotalEnergy	97899039.57
wavelet-LHL	firstorder	Maximum	294.5734247
wavelet-LHL	firstorder	RootMeanSquared	183.229525
wavelet-LHL	firstorder	90Percentile	194.1463769
wavelet-LHL	firstorder	Minimum	-440.1334847
wavelet-LHL	firstorder	Entropy	4.683083805
wavelet-LHL	firstorder	Range	734.7069094
wavelet-LHL	firstorder	Variance	31771.54174
wavelet-LHL	firstorder	10Percentile	-271.0873082
wavelet-LHL	firstorder	Kurtosis	1.965632303

wavelet-LHL	firstorder	Mean	-42.44428227
wavelet-LHL	glrlm	ShortRunLowGrayLevelEmphasis	0.017186634
wavelet-LHL	glrlm	GrayLevelVariance	51.25907605
wavelet-LHL	glrlm	LowGrayLevelRunEmphasis	0.017292424
wavelet-LHL	glrlm	GrayLevelNonUniformityNormalized	0.041885035
wavelet-LHL	glrlm	RunVariance	0.021322289
wavelet-LHL	glrlm	GrayLevelNonUniformity	4.427334252
wavelet-LHL	glrlm	LongRunEmphasis	1.066683947
wavelet-LHL	glrlm	ShortRunHighGrayLevelEmphasis	328.3823776
wavelet-LHL	glrlm	RunLengthNonUniformity	101.2627827
wavelet-LHL	glrlm	ShortRunEmphasis	0.983329013
wavelet-LHL	glrlm	LongRunHighGrayLevelEmphasis	355.2540626
wavelet-LHL	glrlm	RunPercentage	0.978632479
wavelet-LHL	glrlm	LongRunLowGrayLevelEmphasis	0.017715585
wavelet-LHL	glrlm	RunEntropy	4.754443017
wavelet-LHL	glrlm	HighGrayLevelRunEmphasis	333.7567146
wavelet-LHL	glrlm	RunLengthNonUniformityNormalized	0.957355421
wavelet-LHL	glszm	GrayLevelVariance	53.63114885
wavelet-LHL	glszm	ZoneVariance	0.561448486
wavelet-LHL	glszm	GrayLevelNonUniformityNormalized	0.040217914
wavelet-LHL	glszm	SizeZoneNonUniformityNormalized	0.604230091
wavelet-LHL	glszm	SizeZoneNonUniformity	47.73417722
wavelet-LHL	glszm	GrayLevelNonUniformity	3.17721519
wavelet-LHL	glszm	LargeAreaEmphasis	2.430379747
wavelet-LHL	glszm	SmallAreaHighGrayLevelEmphasis	273.9125035
wavelet-LHL	glszm	ZonePercentage	0.731481481
wavelet-LHL	glszm	LargeAreaLowGrayLevelEmphasis	0.030978581
wavelet-LHL	glszm	LargeAreaHighGrayLevelEmphasis	783.1012658
wavelet-LHL	glszm	HighGrayLevelZoneEmphasis	337.0632911
wavelet-LHL	glszm	SmallAreaEmphasis	0.804655415
wavelet-LHL	glszm	LowGrayLevelZoneEmphasis	0.020958226
wavelet-LHL	glszm	ZoneEntropy	5.309738659
wavelet-LHL	glszm	SmallAreaLowGrayLevelEmphasis	0.019770537
wavelet-LHL	ngtdm	Coarseness	0.048113889
wavelet-LHL	ngtdm	Complexity	1528.685448
wavelet-LHL	ngtdm	Strength	13.33479867
wavelet-LHL	ngtdm	Contrast	0.622181265
wavelet-LHL	ngtdm	Busyness	0.059051722
wavelet-LHH	gldm	GrayLevelVariance	38.06678669
wavelet-LHH	gldm	HighGrayLevelEmphasis	226.6203704
wavelet-LHH	gldm	DependenceEntropy	5.34490666
wavelet-LHH	gldm	DependenceNonUniformity	40.22222222
wavelet-LHH	gldm	GrayLevelNonUniformity	6.314814815
wavelet-LHH	gldm	SmallDependenceEmphasis	0.609151235
wavelet-LHH	gldm	SmallDependenceHighGrayLevelEmphasis	150.6512963
wavelet-LHH	gldm	DependenceNonUniformityNormalized	0.372427984
wavelet-LHH	gldm	LargeDependenceEmphasis	4.055555556
wavelet-LHH	gldm	LargeDependenceLowGrayLevelEmphasis	0.048261613
wavelet-LHH	gldm	DependenceVariance	0.960562414
wavelet-LHH	gldm	LargeDependenceHighGrayLevelEmphasis	766.3981481
wavelet-LHH	gldm	SmallDependenceLowGrayLevelEmphasis	0.020531201
wavelet-LHH	gldm	LowGrayLevelEmphasis	0.023843624
wavelet-LHH	glcm	JointAverage	13.76493005

wavelet-LHH	glcm	SumAverage	27.5298601
wavelet-LHH	glcm	JointEntropy	6.664401421
wavelet-LHH	glcm	ClusterShade	271.7202784
wavelet-LHH	glcm	MaximumProbability	0.027088858
wavelet-LHH	glcm	Idmn	0.949949585
wavelet-LHH	glcm	JointEnergy	0.011025447
wavelet-LHH	glcm	Contrast	64.44564825
wavelet-LHH	glcm	DifferenceEntropy	3.77092619
wavelet-LHH	glcm	InverseVariance	0.153001006
wavelet-LHH	glcm	DifferenceVariance	22.44733886
wavelet-LHH	glcm	Idn	0.851679047
wavelet-LHH	glcm	Idm	0.141102124
wavelet-LHH	glcm	Correlation	0.028473274
wavelet-LHH	glcm	Autocorrelation	190.8367833
wavelet-LHH	glcm	SumEntropy	4.553025503
wavelet-LHH	glcm	SumSquares	33.55096651
wavelet-LHH	glcm	ClusterProminence	17715.35315
wavelet-LHH	glcm	Imc2	0.989612481
wavelet-LHH	glcm	Imc1	-0.454270753
wavelet-LHH	glcm	DifferenceAverage	6.303299599
wavelet-LHH	glcm	Id	0.234006184
wavelet-LHH	glcm	ClusterTendency	69.7582178
wavelet-LHH	firstorder	InterquartileRange	195.7605149
wavelet-LHH	firstorder	Skewness	0.531781068
wavelet-LHH	firstorder	Uniformity	0.058470508
wavelet-LHH	firstorder	Median	-0.469691246
wavelet-LHH	firstorder	Energy	2579277.738
wavelet-LHH	firstorder	RobustMeanAbsoluteDeviation	82.21876454
wavelet-LHH	firstorder	MeanAbsoluteDeviation	121.7908676
wavelet-LHH	firstorder	TotalEnergy	69640498.92
wavelet-LHH	firstorder	Maximum	492.1086184
wavelet-LHH	firstorder	RootMeanSquared	154.5386724
wavelet-LHH	firstorder	90Percentile	191.3391107
wavelet-LHH	firstorder	Minimum	-308.1565776
wavelet-LHH	firstorder	Entropy	4.42353119
wavelet-LHH	firstorder	Range	800.265196
wavelet-LHH	firstorder	Variance	23847.40358
wavelet-LHH	firstorder	10Percentile	-160.4676118
wavelet-LHH	firstorder	Kurtosis	3.300241525
wavelet-LHH	firstorder	Mean	5.898957219
wavelet-LHH	glrlm	ShortRunLowGrayLevelEmphasis	0.024141074
wavelet-LHH	glrlm	GrayLevelVariance	38.66230999
wavelet-LHH	glrlm	LowGrayLevelRunEmphasis	0.024323885
wavelet-LHH	glrlm	GrayLevelNonUniformityNormalized	0.057199233
wavelet-LHH	glrlm	RunVariance	0.030672189
wavelet-LHH	glrlm	GrayLevelNonUniformity	5.998881076
wavelet-LHH	glrlm	LongRunEmphasis	1.093469321
wavelet-LHH	glrlm	ShortRunHighGrayLevelEmphasis	223.7581508
wavelet-LHH	glrlm	RunLengthNonUniformity	98.95899051
wavelet-LHH	glrlm	ShortRunEmphasis	0.977504813
wavelet-LHH	glrlm	LongRunHighGrayLevelEmphasis	244.9792064
wavelet-LHH	glrlm	RunPercentage	0.970797721
wavelet-LHH	glrlm	LongRunLowGrayLevelEmphasis	0.025090016

wavelet-LHH	glrlm	RunEntropy	4.546640546
wavelet-LHH	glrlm	HighGrayLevelRunEmphasis	227.9325904
wavelet-LHH	glrlm	RunLengthNonUniformityNormalized	0.942932686
wavelet-LHH	glszm	GrayLevelVariance	46.17092768
wavelet-LHH	glszm	ZoneVariance	1.437545654
wavelet-LHH	glszm	GrayLevelNonUniformityNormalized	0.048210373
wavelet-LHH	glszm	SizeZoneNonUniformityNormalized	0.601168736
wavelet-LHH	glszm	SizeZoneNonUniformity	44.48648649
wavelet-LHH	glszm	GrayLevelNonUniformity	3.567567568
wavelet-LHH	glszm	LargeAreaEmphasis	3.567567568
wavelet-LHH	glszm	SmallAreaHighGrayLevelEmphasis	203.4784889
wavelet-LHH	glszm	ZonePercentage	0.685185185
wavelet-LHH	glszm	LargeAreaLowGrayLevelEmphasis	0.052728096
wavelet-LHH	glszm	LargeAreaHighGrayLevelEmphasis	654.472973
wavelet-LHH	glszm	HighGrayLevelZoneEmphasis	245.972973
wavelet-LHH	glszm	SmallAreaEmphasis	0.803188605
wavelet-LHH	glszm	LowGrayLevelZoneEmphasis	0.030926701
wavelet-LHH	glszm	ZoneEntropy	5.1535214
wavelet-LHH	glszm	SmallAreaLowGrayLevelEmphasis	0.029211779
wavelet-LHH	ngtdm	Coarseness	0.044503102
wavelet-LHH	ngtdm	Complexity	1561.321051
wavelet-LHH	ngtdm	Strength	12.63329782
wavelet-LHH	ngtdm	Contrast	0.463969569
wavelet-LHH	ngtdm	Busyness	0.06405863
wavelet-LLH	gldm	GrayLevelVariance	109.0154321
wavelet-LLH	gldm	HighGrayLevelEmphasis	753.6111111
wavelet-LLH	gldm	DependenceEntropy	5.684065831
wavelet-LLH	gldm	DependenceNonUniformity	44.40740741
wavelet-LLH	gldm	GrayLevelNonUniformity	4.240740741
wavelet-LLH	gldm	SmallDependenceEmphasis	0.649112654
wavelet-LLH	gldm	SmallDependenceHighGrayLevelEmphasis	478.2022248
wavelet-LLH	gldm	DependenceNonUniformityNormalized	0.411179698
wavelet-LLH	gldm	LargeDependenceEmphasis	3.333333333
wavelet-LLH	gldm	LargeDependenceLowGrayLevelEmphasis	0.018038073
wavelet-LLH	gldm	DependenceVariance	0.677640604
wavelet-LLH	gldm	LargeDependenceHighGrayLevelEmphasis	2419.574074
wavelet-LLH	gldm	SmallDependenceLowGrayLevelEmphasis	0.011136713
wavelet-LLH	gldm	LowGrayLevelEmphasis	0.012128719
wavelet-LLH	glcm	JointAverage	24.80891306
wavelet-LLH	glcm	SumAverage	49.61782611
wavelet-LLH	glcm	JointEntropy	6.848264597
wavelet-LLH	glcm	ClusterShade	2321.648345
wavelet-LLH	glcm	MaximumProbability	0.022987731
wavelet-LLH	glcm	Idmn	0.954238089
wavelet-LLH	glcm	JointEnergy	0.009305652
wavelet-LLH	glcm	Contrast	118.2308133
wavelet-LLH	glcm	DifferenceEntropy	4.092360915
wavelet-LLH	glcm	InverseVariance	0.114948155
wavelet-LLH	glcm	DifferenceVariance	43.66286371
wavelet-LLH	glcm	Idn	0.860133049
wavelet-LLH	glcm	Idm	0.112077962
wavelet-LLH	glcm	Correlation	0.372116127
wavelet-LLH	glcm	Autocorrelation	651.2503346

wavelet-LLH	glcm	SumEntropy	5.056834908
wavelet-LLH	glcm	SumSquares	94.77108072
wavelet-LLH	glcm	ClusterProminence	177408.6605
wavelet-LLH	glcm	Imc2	0.998095592
wavelet-LLH	glcm	Imc1	-0.579860796
wavelet-LLH	glcm	DifferenceAverage	8.40396355
wavelet-LLH	glcm	Id	0.200157077
wavelet-LLH	glcm	ClusterTendency	260.8535095
wavelet-LLH	firstorder	InterquartileRange	389.177991
wavelet-LLH	firstorder	Skewness	0.28857691
wavelet-LLH	firstorder	Uniformity	0.039266118
wavelet-LLH	firstorder	Median	-115.6995127
wavelet-LLH	firstorder	Energy	7701862.5
wavelet-LLH	firstorder	RobustMeanAbsoluteDeviation	161.5336242
wavelet-LLH	firstorder	MeanAbsoluteDeviation	219.0248711
wavelet-LLH	firstorder	TotalEnergy	207950287.5
wavelet-LLH	firstorder	Maximum	477.0718024
wavelet-LLH	firstorder	RootMeanSquared	267.0459542
wavelet-LLH	firstorder	90Percentile	338.0870213
wavelet-LLH	firstorder	Minimum	-652.9188297
wavelet-LLH	firstorder	Entropy	4.899241009
wavelet-LLH	firstorder	Range	1129.990632
wavelet-LLH	firstorder	Variance	68571.59919
wavelet-LLH	firstorder	10Percentile	-350.3750027
wavelet-LLH	firstorder	Kurtosis	2.300262982
wavelet-LLH	firstorder	Mean	-52.36356058
wavelet-LLH	glrlm	ShortRunLowGrayLevelEmphasis	0.012321746
wavelet-LLH	glrlm	GrayLevelVariance	109.7109334
wavelet-LLH	glrlm	LowGrayLevelRunEmphasis	0.01236817
wavelet-LLH	glrlm	GrayLevelNonUniformityNormalized	0.038796048
wavelet-LLH	glrlm	RunVariance	0.031735602
wavelet-LLH	glrlm	GrayLevelNonUniformity	4.088730853
wavelet-LLH	glrlm	LongRunEmphasis	1.083228123
wavelet-LLH	glrlm	ShortRunHighGrayLevelEmphasis	741.1878954
wavelet-LLH	glrlm	RunLengthNonUniformity	100.908057
wavelet-LLH	glrlm	ShortRunEmphasis	0.983000238
wavelet-LLH	glrlm	LongRunHighGrayLevelEmphasis	815.6870478
wavelet-LLH	glrlm	RunPercentage	0.975783476
wavelet-LLH	glrlm	LongRunLowGrayLevelEmphasis	0.012579841
wavelet-LLH	glrlm	RunEntropy	4.975553454
wavelet-LLH	glrlm	HighGrayLevelRunEmphasis	753.8941896
wavelet-LLH	glrlm	RunLengthNonUniformityNormalized	0.956947613
wavelet-LLH	glszm	GrayLevelVariance	117.5792242
wavelet-LLH	glszm	ZoneVariance	0.775147929
wavelet-LLH	glszm	GrayLevelNonUniformityNormalized	0.033859303
wavelet-LLH	glszm	SizeZoneNonUniformityNormalized	0.630834977
wavelet-LLH	glszm	SizeZoneNonUniformity	49.20512821
wavelet-LLH	glszm	GrayLevelNonUniformity	2.641025641
wavelet-LLH	glszm	LargeAreaEmphasis	2.692307692
wavelet-LLH	glszm	SmallAreaHighGrayLevelEmphasis	598.3318376
wavelet-LLH	glszm	ZonePercentage	0.722222222
wavelet-LLH	glszm	LargeAreaLowGrayLevelEmphasis	0.019890724
wavelet-LLH	glszm	LargeAreaHighGrayLevelEmphasis	1986.410256

wavelet-LLH	glszm	HighGrayLevelZoneEmphasis	747.7179487
wavelet-LLH	glszm	SmallAreaEmphasis	0.821004274
wavelet-LLH	glszm	LowGrayLevelZoneEmphasis	0.015761801
wavelet-LLH	glszm	ZoneEntropy	5.45810318
wavelet-LLH	glszm	SmallAreaLowGrayLevelEmphasis	0.015201643
wavelet-LLH	ngtdm	Coarseness	0.049829885
wavelet-LLH	ngtdm	Complexity	3340.227221
wavelet-LLH	ngtdm	Strength	29.3441976
wavelet-LLH	ngtdm	Contrast	1.023346056
wavelet-LLH	ngtdm	Busyness	0.028125799
wavelet-LLL	gldm	GrayLevelVariance	489.7252229
wavelet-LLL	gldm	HighGrayLevelEmphasis	2545.675926
wavelet-LLL	gldm	DependenceEntropy	6.016235303
wavelet-LLL	gldm	DependenceNonUniformity	66.16666667
wavelet-LLL	gldm	GrayLevelNonUniformity	2.166666667
wavelet-LLL	gldm	SmallDependenceEmphasis	0.808641975
wavelet-LLL	gldm	SmallDependenceHighGrayLevelEmphasis	1884.049383
wavelet-LLL	gldm	DependenceNonUniformityNormalized	0.612654321
wavelet-LLL	gldm	LargeDependenceEmphasis	1.888888889
wavelet-LLL	gldm	LargeDependenceLowGrayLevelEmphasis	0.02108391
wavelet-LLL	gldm	DependenceVariance	0.25617284
wavelet-LLL	gldm	LargeDependenceHighGrayLevelEmphasis	5962.675926
wavelet-LLL	gldm	SmallDependenceLowGrayLevelEmphasis	0.020094189
wavelet-LLL	gldm	LowGrayLevelEmphasis	0.020288177
wavelet-LLL	glcm	JointAverage	52.08848599
wavelet-LLL	glcm	SumAverage	104.176972
wavelet-LLL	glcm	JointEntropy	7.029243723
wavelet-LLL	glcm	ClusterShade	-9572.577422
wavelet-LLL	glcm	MaximumProbability	0.013879555
wavelet-LLL	glcm	Idmn	0.920833965
wavelet-LLL	glcm	JointEnergy	0.007799299
wavelet-LLL	glcm	Contrast	690.9835268
wavelet-LLL	glcm	DifferenceEntropy	4.986192165
wavelet-LLL	glcm	InverseVariance	0.048249875
wavelet-LLL	glcm	DifferenceVariance	234.8749423
wavelet-LLL	glcm	Idn	0.815413844
wavelet-LLL	glcm	Idm	0.048182295
wavelet-LLL	glcm	Correlation	0.273775871
wavelet-LLL	glcm	Autocorrelation	2842.26378
wavelet-LLL	glcm	SumEntropy	5.581624489
wavelet-LLL	glcm	SumSquares	473.8571355
wavelet-LLL	glcm	ClusterProminence	3300354.533
wavelet-LLL	glcm	Imc2	0.999916934
wavelet-LLL	glcm	Imc1	-0.766751811
wavelet-LLL	glcm	DifferenceAverage	21.15277462
wavelet-LLL	glcm	Id	0.106382524
wavelet-LLL	glcm	ClusterTendency	1204.445015
wavelet-LLL	firstorder	InterquartileRange	885.4584205
wavelet-LLL	firstorder	Skewness	0.030518355
wavelet-LLL	firstorder	Uniformity	0.020061728
wavelet-LLL	firstorder	Median	-721.1454345
wavelet-LLL	firstorder	Energy	82662238.28
wavelet-LLL	firstorder	RobustMeanAbsoluteDeviation	373.8271466

wavelet-LLL	firstorder	MeanAbsoluteDeviation	477.1030321
wavelet-LLL	firstorder	TotalEnergy	2231880433
wavelet-LLL	firstorder	Maximum	294.0856171
wavelet-LLL	firstorder	RootMeanSquared	874.8663299
wavelet-LLL	firstorder	90Percentile	132.9159222
wavelet-LLL	firstorder	Minimum	-1781.152152
wavelet-LLL	firstorder	Entropy	5.82068516
wavelet-LLL	firstorder	Range	2075.237769
wavelet-LLL	firstorder	Variance	305196.3138
wavelet-LLL	firstorder	10Percentile	-1404.758476
wavelet-LLL	firstorder	Kurtosis	1.956548118
wavelet-LLL	firstorder	Mean	-678.3765778
wavelet-LLL	glrlm	ShortRunLowGrayLevelEmphasis	0.020491545
wavelet-LLL	glrlm	GrayLevelVariance	487.9744016
wavelet-LLL	glrlm	LowGrayLevelRunEmphasis	0.020499218
wavelet-LLL	glrlm	GrayLevelNonUniformityNormalized	0.020015825
wavelet-LLL	glrlm	RunVariance	0.010678564
wavelet-LLL	glrlm	GrayLevelNonUniformity	2.138668297
wavelet-LLL	glrlm	LongRunEmphasis	1.032719388
wavelet-LLL	glrlm	ShortRunHighGrayLevelEmphasis	2503.045279
wavelet-LLL	glrlm	RunLengthNonUniformity	104.5865652
wavelet-LLL	glrlm	ShortRunEmphasis	0.991820153
wavelet-LLL	glrlm	LongRunHighGrayLevelEmphasis	2653.891635
wavelet-LLL	glrlm	RunPercentage	0.989316239
wavelet-LLL	glrlm	LongRunLowGrayLevelEmphasis	0.020529911
wavelet-LLL	glrlm	RunEntropy	5.838094045
wavelet-LLL	glrlm	HighGrayLevelRunEmphasis	2533.21455
wavelet-LLL	glrlm	RunLengthNonUniformityNormalized	0.978642872
wavelet-LLL	glszm	GrayLevelVariance	470.0947261
wavelet-LLL	glszm	ZoneVariance	0.148030783
wavelet-LLL	glszm	GrayLevelNonUniformityNormalized	0.019239475
wavelet-LLL	glszm	SizeZoneNonUniformityNormalized	0.758940697
wavelet-LLL	glszm	SizeZoneNonUniformity	71.34042553
wavelet-LLL	glszm	GrayLevelNonUniformity	1.808510638
wavelet-LLL	glszm	LargeAreaEmphasis	1.468085106
wavelet-LLL	glszm	SmallAreaHighGrayLevelEmphasis	2052.712175
wavelet-LLL	glszm	ZonePercentage	0.87037037
wavelet-LLL	glszm	LargeAreaLowGrayLevelEmphasis	0.02361116
wavelet-LLL	glszm	LargeAreaHighGrayLevelEmphasis	4100.670213
wavelet-LLL	glszm	HighGrayLevelZoneEmphasis	2403.287234
wavelet-LLL	glszm	SmallAreaEmphasis	0.894799054
wavelet-LLL	glszm	LowGrayLevelZoneEmphasis	0.023160855
wavelet-LLL	glszm	ZoneEntropy	6.011827681
wavelet-LLL	glszm	SmallAreaLowGrayLevelEmphasis	0.023050173
wavelet-LLL	ngtdm	Coarseness	0.029994138
wavelet-LLL	ngtdm	Complexity	30298.25977
wavelet-LLL	ngtdm	Strength	79.76586594
wavelet-LLL	ngtdm	Contrast	3.603856083
wavelet-LLL	ngtdm	Busyness	0.015229212
wavelet-HHL	gldm	GrayLevelVariance	22.46262003
wavelet-HHL	gldm	HighGrayLevelEmphasis	217.9444444
wavelet-HHL	gldm	DependenceEntropy	5.255689326
wavelet-HHL	gldm	DependenceNonUniformity	34.75925926

wavelet-HHL	gldm	GrayLevelNonUniformity	7.12962963
wavelet-HHL	gldm	SmallDependenceEmphasis	0.572755837
wavelet-HHL	gldm	SmallDependenceHighGrayLevelEmphasis	130.7547996
wavelet-HHL	gldm	DependenceNonUniformityNormalized	0.321844993
wavelet-HHL	gldm	LargeDependenceEmphasis	6.148148148
wavelet-HHL	gldm	LargeDependenceLowGrayLevelEmphasis	0.046437909
wavelet-HHL	gldm	DependenceVariance	1.846364883
wavelet-HHL	gldm	LargeDependenceHighGrayLevelEmphasis	1392.740741
wavelet-HHL	gldm	SmallDependenceLowGrayLevelEmphasis	0.01435663
wavelet-HHL	gldm	LowGrayLevelEmphasis	0.017112479
wavelet-HHL	glcm	JointAverage	13.93341558
wavelet-HHL	glcm	SumAverage	27.86683117
wavelet-HHL	glcm	JointEntropy	6.537726464
wavelet-HHL	glcm	ClusterShade	-23.08526723
wavelet-HHL	glcm	MaximumProbability	0.0303986
wavelet-HHL	glcm	Idmn	0.941403916
wavelet-HHL	glcm	JointEnergy	0.012163751
wavelet-HHL	glcm	Contrast	43.85521277
wavelet-HHL	glcm	DifferenceEntropy	3.584143478
wavelet-HHL	glcm	InverseVariance	0.181264912
wavelet-HHL	glcm	DifferenceVariance	15.66746018
wavelet-HHL	glcm	Idn	0.84108496
wavelet-HHL	glcm	Idm	0.176729477
wavelet-HHL	glcm	Correlation	-0.041174262
wavelet-HHL	glcm	Autocorrelation	193.2034369
wavelet-HHL	glcm	SumEntropy	4.32302579
wavelet-HHL	glcm	SumSquares	20.9225015
wavelet-HHL	glcm	ClusterProminence	4732.096806
wavelet-HHL	glcm	Imc2	0.975150854
wavelet-HHL	glcm	Imc1	-0.378526554
wavelet-HHL	glcm	DifferenceAverage	5.218573478
wavelet-HHL	glcm	Id	0.269857959
wavelet-HHL	glcm	ClusterTendency	39.83479323
wavelet-HHL	firstorder	InterquartileRange	162.6824149
wavelet-HHL	firstorder	Skewness	-0.117947612
wavelet-HHL	firstorder	Uniformity	0.066015089
wavelet-HHL	firstorder	Median	13.52887559
wavelet-HHL	firstorder	Energy	1525774.184
wavelet-HHL	firstorder	RobustMeanAbsoluteDeviation	70.85847054
wavelet-HHL	firstorder	MeanAbsoluteDeviation	97.18259684
wavelet-HHL	firstorder	TotalEnergy	41195902.98
wavelet-HHL	firstorder	Maximum	282.7848763
wavelet-HHL	firstorder	RootMeanSquared	118.8593233
wavelet-HHL	firstorder	90Percentile	153.0290958
wavelet-HHL	firstorder	Minimum	-319.2236449
wavelet-HHL	firstorder	Entropy	4.117113473
wavelet-HHL	firstorder	Range	602.0085212
wavelet-HHL	firstorder	Variance	14004.05338
wavelet-HHL	firstorder	10Percentile	-150.3032768
wavelet-HHL	firstorder	Kurtosis	2.726517662
wavelet-HHL	firstorder	Mean	11.11239689
wavelet-HHL	glrlm	ShortRunLowGrayLevelEmphasis	0.017395475
wavelet-HHL	glrlm	GrayLevelVariance	22.95181587

wavelet-HHL	glrlm	LowGrayLevelRunEmphasis	0.01759293
wavelet-HHL	glrlm	GrayLevelNonUniformityNormalized	0.06439922
wavelet-HHL	glrlm	RunVariance	0.041183789
wavelet-HHL	glrlm	GrayLevelNonUniformity	6.669272291
wavelet-HHL	glrlm	LongRunEmphasis	1.130382716
wavelet-HHL	glrlm	ShortRunHighGrayLevelEmphasis	210.8918456
wavelet-HHL	glrlm	RunLengthNonUniformity	95.07986324
wavelet-HHL	glrlm	ShortRunEmphasis	0.967404321
wavelet-HHL	glrlm	LongRunHighGrayLevelEmphasis	246.301835
wavelet-HHL	glrlm	RunPercentage	0.958689459
wavelet-HHL	glrlm	LongRunLowGrayLevelEmphasis	0.018382749
wavelet-HHL	glrlm	RunEntropy	4.309231588
wavelet-HHL	glrlm	HighGrayLevelRunEmphasis	217.9738435
wavelet-HHL	glrlm	RunLengthNonUniformityNormalized	0.917632423
wavelet-HHL	glszm	GrayLevelVariance	29.30968858
wavelet-HHL	glszm	ZoneVariance	2.565743945
wavelet-HHL	glszm	GrayLevelNonUniformityNormalized	0.0566609
wavelet-HHL	glszm	SizeZoneNonUniformityNormalized	0.641435986
wavelet-HHL	glszm	SizeZoneNonUniformity	43.61764706
wavelet-HHL	glszm	GrayLevelNonUniformity	3.852941176
wavelet-HHL	glszm	LargeAreaEmphasis	5.088235294
wavelet-HHL	glszm	SmallAreaHighGrayLevelEmphasis	190.1041204
wavelet-HHL	glszm	ZonePercentage	0.62962963
wavelet-HHL	glszm	LargeAreaLowGrayLevelEmphasis	0.044884237
wavelet-HHL	glszm	LargeAreaHighGrayLevelEmphasis	1215.470588
wavelet-HHL	glszm	HighGrayLevelZoneEmphasis	222.0294118
wavelet-HHL	glszm	SmallAreaEmphasis	0.822809445
wavelet-HHL	glszm	LowGrayLevelZoneEmphasis	0.023516999
wavelet-HHL	glszm	ZoneEntropy	4.850125113
wavelet-HHL	glszm	SmallAreaLowGrayLevelEmphasis	0.022249669
wavelet-HHL	ngtdm	Coarseness	0.041589529
wavelet-HHL	ngtdm	Complexity	755.1901129
wavelet-HHL	ngtdm	Strength	6.653484529
wavelet-HHL	ngtdm	Contrast	0.398785432
wavelet-HHL	ngtdm	Busyness	0.094718686
wavelet-HLH	gldm	GrayLevelVariance	29.32407407
wavelet-HLH	gldm	HighGrayLevelEmphasis	211.5740741
wavelet-HLH	gldm	DependenceEntropy	5.408376595
wavelet-HLH	gldm	DependenceNonUniformity	34.92592593
wavelet-HLH	gldm	GrayLevelNonUniformity	6.62962963
wavelet-HLH	gldm	SmallDependenceEmphasis	0.561715535
wavelet-HLH	gldm	SmallDependenceHighGrayLevelEmphasis	118.019856
wavelet-HLH	gldm	DependenceNonUniformityNormalized	0.323388203
wavelet-HLH	gldm	LargeDependenceEmphasis	5.074074074
wavelet-HLH	gldm	LargeDependenceLowGrayLevelEmphasis	0.047912821
wavelet-HLH	gldm	DependenceVariance	1.293209877
wavelet-HLH	gldm	LargeDependenceHighGrayLevelEmphasis	1014.314815
wavelet-HLH	gldm	SmallDependenceLowGrayLevelEmphasis	0.022192845
wavelet-HLH	gldm	LowGrayLevelEmphasis	0.024660833
wavelet-HLH	glcm	JointAverage	13.62501401
wavelet-HLH	glcm	SumAverage	27.25002802
wavelet-HLH	glcm	JointEntropy	6.622088705
wavelet-HLH	glcm	ClusterShade	-86.36407979

wavelet-HLH	glcm	MaximumProbability	0.025671812
wavelet-HLH	glcm	Idmn	0.936272863
wavelet-HLH	glcm	JointEnergy	0.011181905
wavelet-HLH	glcm	Contrast	58.69230549
wavelet-HLH	glcm	DifferenceEntropy	3.717812382
wavelet-HLH	glcm	InverseVariance	0.182818171
wavelet-HLH	glcm	DifferenceVariance	24.42041846
wavelet-HLH	glcm	Idn	0.840852855
wavelet-HLH	glcm	Idm	0.171057282
wavelet-HLH	glcm	Correlation	-0.040500438
wavelet-HLH	glcm	Autocorrelation	184.6729175
wavelet-HLH	glcm	SumEntropy	4.426804921
wavelet-HLH	glcm	SumSquares	28.28447226
wavelet-HLH	glcm	ClusterProminence	10201.34073
wavelet-HLH	glcm	Imc2	0.988173597
wavelet-HLH	glcm	Imc1	-0.443215132
wavelet-HLH	glcm	DifferenceAverage	5.773996006
wavelet-HLH	glcm	Id	0.262638824
wavelet-HLH	glcm	ClusterTendency	54.44558354
wavelet-HLH	firstorder	InterquartileRange	167.8033465
wavelet-HLH	firstorder	Skewness	-0.143463378
wavelet-HLH	firstorder	Uniformity	0.06138546
wavelet-HLH	firstorder	Median	7.905251013
wavelet-HLH	firstorder	Energy	1970843.837
wavelet-HLH	firstorder	RobustMeanAbsoluteDeviation	71.04483163
wavelet-HLH	firstorder	MeanAbsoluteDeviation	105.4691019
wavelet-HLH	firstorder	TotalEnergy	53212783.59
wavelet-HLH	firstorder	Maximum	342.5037039
wavelet-HLH	firstorder	RootMeanSquared	135.087209
wavelet-HLH	firstorder	90Percentile	174.8134435
wavelet-HLH	firstorder	Minimum	-302.4061039
wavelet-HLH	firstorder	Entropy	4.307131477
wavelet-HLH	firstorder	Range	644.9098078
wavelet-HLH	firstorder	Variance	18248.17382
wavelet-HLH	firstorder	10Percentile	-209.2636311
wavelet-HLH	firstorder	Kurtosis	2.782231649
wavelet-HLH	firstorder	Mean	-0.616625362
wavelet-HLH	glrlm	ShortRunLowGrayLevelEmphasis	0.025225718
wavelet-HLH	glrlm	GrayLevelVariance	30.16676234
wavelet-HLH	glrlm	LowGrayLevelRunEmphasis	0.025383518
wavelet-HLH	glrlm	GrayLevelNonUniformityNormalized	0.060002762
wavelet-HLH	glrlm	RunVariance	0.039236624
wavelet-HLH	glrlm	GrayLevelNonUniformity	6.245679791
wavelet-HLH	glrlm	LongRunEmphasis	1.117411776
wavelet-HLH	glrlm	ShortRunHighGrayLevelEmphasis	206.3016752
wavelet-HLH	glrlm	RunLengthNonUniformity	96.88339817
wavelet-HLH	glrlm	ShortRunEmphasis	0.972323101
wavelet-HLH	glrlm	LongRunHighGrayLevelEmphasis	235.5754224
wavelet-HLH	glrlm	RunPercentage	0.963675214
wavelet-HLH	glrlm	LongRunLowGrayLevelEmphasis	0.026054388
wavelet-HLH	glrlm	RunEntropy	4.473217404
wavelet-HLH	glrlm	HighGrayLevelRunEmphasis	211.9298234
wavelet-HLH	glrlm	RunLengthNonUniformityNormalized	0.930131912

wavelet-HLH	glszm	GrayLevelVariance	41.47988754
wavelet-HLH	glszm	ZoneVariance	2.212802768
wavelet-HLH	glszm	GrayLevelNonUniformityNormalized	0.047145329
wavelet-HLH	glszm	SizeZoneNonUniformityNormalized	0.58650519
wavelet-HLH	glszm	SizeZoneNonUniformity	39.88235294
wavelet-HLH	glszm	GrayLevelNonUniformity	3.205882353
wavelet-HLH	glszm	LargeAreaEmphasis	4.735294118
wavelet-HLH	glszm	SmallAreaHighGrayLevelEmphasis	165.0256786
wavelet-HLH	glszm	ZonePercentage	0.62962963
wavelet-HLH	glszm	LargeAreaLowGrayLevelEmphasis	0.056487274
wavelet-HLH	glszm	LargeAreaHighGrayLevelEmphasis	957.3235294
wavelet-HLH	glszm	HighGrayLevelZoneEmphasis	212.3970588
wavelet-HLH	glszm	SmallAreaEmphasis	0.792690745
wavelet-HLH	glszm	LowGrayLevelZoneEmphasis	0.035888083
wavelet-HLH	glszm	ZoneEntropy	5.138502179
wavelet-HLH	glszm	SmallAreaLowGrayLevelEmphasis	0.034713774
wavelet-HLH	ngtdm	Coarseness	0.049901485
wavelet-HLH	ngtdm	Complexity	1088.294126
wavelet-HLH	ngtdm	Strength	9.305354131
wavelet-HLH	ngtdm	Contrast	0.404180619
wavelet-HLH	ngtdm	Busyness	0.059152297

Image type	Feature Class	Feature Name	502: RTSTRUCT: C99-DNU_segment_ITVp
general	info	BoundingBox	(0, 0, 1, 5, 5, 4)
general	info	EnabledImageTypes	{u'Original': {}, u'Wavelet': {}}
general	info	GeneralSettings	{'distances': [1], 'additionalInfo': True, 'force2D': False, 'interpolator': 'sitkBSpline', 'resampledPixelSpacing': [3.0], 'label': 1, 'normalizeScale': 1, 'normalize': False, 'force2Ddimension': 0, 'removeOutliers': None, 'minimumROISize': None, u'binWidth': 25.0, 'minimumROIDimensions': 1, u'correctMask': True, 'preCrop': False, u'symmetricalGLCM': True, 'resegmentRange': None, 'padDistance': 5}
general	info	ImageHash	642adbafa5d7080cbe199e9b94065faf49b83188
general	info	ImageSpacing	(0.9765625000000002, 0.9765625000000002, 2.0)
general	info	MaskHash	b0ac6997fd95c65a05c261b1da5b0086d2209f4b
general	info	NumpyVersion	1.13.1
general	info	PyWaveletVersion	0.5.2
general	info	SimpleITKVersion	1.1.0
general	info	Version	1.3.0.post102+g1b0eb74
general	info	VolumeNum	1
general	info	VoxelNum	45
original	shape	Maximum3DDiameter	14.69693846
original	shape	Maximum2DDiameterSlice	13.41640786
original	shape	Sphericity	0.917871287
original	shape	MinorAxis	12.21353278
original	shape	Elongation	0.862970258
original	shape	SurfaceVolumeRatio	0.493753569
original	shape	Volume	1215
original	shape	MajorAxis	14.15290117
original	shape	SurfaceArea	599.9105857
original	shape	Flatness	0.717848766
original	shape	LeastAxis	10.15964265
original	shape	Maximum2DDiameterColumn	13.41640786
original	shape	Maximum2DDiameterRow	13.41640786
original	gldm	GrayLevelVariance	83.10617284
original	gldm	HighGrayLevelEmphasis	346.2666667
original	gldm	DependenceEntropy	4.691853096
original	gldm	DependenceNonUniformity	20.73333333
original	gldm	GrayLevelNonUniformity	2.511111111
original	gldm	SmallDependenceEmphasis	0.627160494
original	gldm	SmallDependenceHighGrayLevelEmphasis	185.9506173
original	gldm	DependenceNonUniformityNormalized	0.460740741
original	gldm	LargeDependenceEmphasis	2.688888889
original	gldm	LargeDependenceLowGrayLevelEmphasis	0.04911702
original	gldm	DependenceVariance	0.337777778
original	gldm	LargeDependenceHighGrayLevelEmphasis	1090.444444
original	gldm	SmallDependenceLowGrayLevelEmphasis	0.032077263
original	gldm	LowGrayLevelEmphasis	0.035220794
original	glcm	JointAverage	18.22490759
original	glcm	SumAverage	36.44981518

original	glcm	JointEntropy	5.434539186
original	glcm	ClusterShade	20.16485518
original	glcm	MaximumProbability	0.036309585
original	glcm	Idmn	0.883873979
original	glcm	JointEnergy	0.023880008
original	glcm	Contrast	161.5622026
original	glcm	DifferenceEntropy	3.761629745
original	glcm	InverseVariance	0.087842704
original	glcm	DifferenceVariance	52.56489626
original	glcm	Idn	0.778435994
original	glcm	Idm	0.094263469
original	glcm	Correlation	0.06726969
original	glcm	Autocorrelation	337.8114928
original	glcm	SumEntropy	4.140964115
original	glcm	SumSquares	86.18594788
original	glcm	ClusterProminence	73346.15672
original	glcm	Imc2	0.998705723
original	glcm	Imc1	-0.717784456
original	glcm	DifferenceAverage	10.31041339
original	glcm	Id	0.173409334
original	glcm	ClusterTendency	183.181589
original	firstorder	InterquartileRange	379
original	firstorder	Skewness	0.214635759
original	firstorder	Uniformity	0.055802469
original	firstorder	Median	-426
original	firstorder	Energy	8908500
original	firstorder	RobustMeanAbsoluteDeviation	155.2489796
original	firstorder	MeanAbsoluteDeviation	198.8197531
original	firstorder	TotalEnergy	240529500
original	firstorder	Maximum	16
original	firstorder	RootMeanSquared	444.9344521
original	firstorder	90Percentile	-51
original	firstorder	Minimum	-752
original	firstorder	Entropy	4.385754485
original	firstorder	Range	768
original	firstorder	Variance	51872.83951
original	firstorder	10Percentile	-648
original	firstorder	Kurtosis	1.789727895
original	firstorder	Mean	-382.2222222
original	glrlm	ShortRunLowGrayLevelEmphasis	0.035661591
original	glrlm	GrayLevelVariance	82.71106578
original	glrlm	LowGrayLevelRunEmphasis	0.035795573
original	glrlm	GrayLevelNonUniformityNormalized	0.055659489
original	glrlm	RunVariance	0.020467449
original	glrlm	GrayLevelNonUniformity	2.453878163
original	glrlm	LongRunEmphasis	1.064174198
original	glrlm	ShortRunHighGrayLevelEmphasis	337.2551126
original	glrlm	RunLengthNonUniformity	42.30984132
original	glrlm	ShortRunEmphasis	0.983956451
original	glrlm	LongRunHighGrayLevelEmphasis	372.4380039
original	glrlm	RunPercentage	0.979487179
original	glrlm	LongRunLowGrayLevelEmphasis	0.036331502
original	glrlm	RunEntropy	4.419141571
original	glrlm	HighGrayLevelRunEmphasis	344.2916909
original	glrlm	RunLengthNonUniformityNormalized	0.959065101

original	glszm	GrayLevelVariance	76.60238751
original	glszm	ZoneVariance	0.35261708
original	glszm	GrayLevelNonUniformityNormalized	0.052341598
original	glszm	SizeZoneNonUniformityNormalized	0.548209366
original	glszm	SizeZoneNonUniformity	18.09090909
original	glszm	GrayLevelNonUniformity	1.727272727
original	glszm	LargeAreaEmphasis	2.212121212
original	glszm	SmallAreaHighGrayLevelEmphasis	213.9175084
original	glszm	ZonePercentage	0.733333333
original	glszm	LargeAreaLowGrayLevelEmphasis	0.054885681
original	glszm	LargeAreaHighGrayLevelEmphasis	852.5454545
original	glszm	HighGrayLevelZoneEmphasis	313.5757576
original	glszm	SmallAreaEmphasis	0.764309764
original	glszm	LowGrayLevelZoneEmphasis	0.045005337
original	glszm	ZoneEntropy	4.620151695
original	glszm	SmallAreaLowGrayLevelEmphasis	0.042985967
original	ngtdm	Coarseness	0.046998219
original	ngtdm	Complexity	2403.830544
original	ngtdm	Strength	23.0477821
original	ngtdm	Contrast	2.272176804
original	ngtdm	Busyness	0.073449147
wavelet-HHH	gldm	GrayLevelVariance	8.158024691
wavelet-HHH	gldm	HighGrayLevelEmphasis	63.57777778
wavelet-HHH	gldm	DependenceEntropy	4.119087818
wavelet-HHH	gldm	DependenceNonUniformity	15
wavelet-HHH	gldm	GrayLevelNonUniformity	5.222222222
wavelet-HHH	gldm	SmallDependenceEmphasis	0.482561728
wavelet-HHH	gldm	SmallDependenceHighGrayLevelEmphasis	31.78117284
wavelet-HHH	gldm	DependenceNonUniformityNormalized	0.333333333
wavelet-HHH	gldm	LargeDependenceEmphasis	4.511111111
wavelet-HHH	gldm	LargeDependenceLowGrayLevelEmphasis	0.144375273
wavelet-HHH	gldm	DependenceVariance	0.773333333
wavelet-HHH	gldm	LargeDependenceHighGrayLevelEmphasis	254.2888889
wavelet-HHH	gldm	SmallDependenceLowGrayLevelEmphasis	0.057734756
wavelet-HHH	gldm	LowGrayLevelEmphasis	0.068374567
wavelet-HHH	glcm	JointAverage	7.517344992
wavelet-HHH	glcm	SumAverage	15.03468998
wavelet-HHH	glcm	JointEntropy	4.985865198
wavelet-HHH	glcm	ClusterShade	1.903978492
wavelet-HHH	glcm	MaximumProbability	0.063419068
wavelet-HHH	glcm	Idmn	0.945808181
wavelet-HHH	glcm	JointEnergy	0.035313074
wavelet-HHH	glcm	Contrast	14.41756483
wavelet-HHH	glcm	DifferenceEntropy	2.626098385
wavelet-HHH	glcm	InverseVariance	0.324227057
wavelet-HHH	glcm	DifferenceVariance	4.781837577
wavelet-HHH	glcm	Idn	0.845023584
wavelet-HHH	glcm	Idm	0.258350676
wavelet-HHH	glcm	Correlation	-0.005570471
wavelet-HHH	glcm	Autocorrelation	56.34578353
wavelet-HHH	glcm	SumEntropy	3.226097203
wavelet-HHH	glcm	SumSquares	7.015858781
wavelet-HHH	glcm	ClusterProminence	614.7205582
wavelet-HHH	glcm	Imc2	0.975433944
wavelet-HHH	glcm	Imc1	-0.469718025

wavelet-HHH	glcm	DifferenceAverage	3.015888772
wavelet-HHH	glcm	Id	0.34832803
wavelet-HHH	glcm	ClusterTendency	13.64587029
wavelet-HHH	firstorder	InterquartileRange	80.11389782
wavelet-HHH	firstorder	Skewness	-0.002822205
wavelet-HHH	firstorder	Uniformity	0.116049383
wavelet-HHH	firstorder	Median	3.01298646
wavelet-HHH	firstorder	Energy	225184.225
wavelet-HHH	firstorder	RobustMeanAbsoluteDeviation	35.6863122
wavelet-HHH	firstorder	MeanAbsoluteDeviation	55.28562515
wavelet-HHH	firstorder	TotalEnergy	6079974.075
wavelet-HHH	firstorder	Maximum	176.0404188
wavelet-HHH	firstorder	RootMeanSquared	70.73962036
wavelet-HHH	firstorder	90Percentile	76.01611698
wavelet-HHH	firstorder	Minimum	-170.4602046
wavelet-HHH	firstorder	Entropy	3.351352676
wavelet-HHH	firstorder	Range	346.5006234
wavelet-HHH	firstorder	Variance	4993.265243
wavelet-HHH	firstorder	10Percentile	-94.69255171
wavelet-HHH	firstorder	Kurtosis	3.226442913
wavelet-HHH	firstorder	Mean	-3.290690751
wavelet-HHH	glrlm	ShortRunLowGrayLevelEmphasis	0.06963225
wavelet-HHH	glrlm	GrayLevelVariance	8.379065887
wavelet-HHH	glrlm	LowGrayLevelRunEmphasis	0.070219736
wavelet-HHH	glrlm	GrayLevelNonUniformityNormalized	0.11504078
wavelet-HHH	glrlm	RunVariance	0.039291826
wavelet-HHH	glrlm	GrayLevelNonUniformity	4.993132619
wavelet-HHH	glrlm	LongRunEmphasis	1.12094276
wavelet-HHH	glrlm	ShortRunHighGrayLevelEmphasis	62.19569585
wavelet-HHH	glrlm	RunLengthNonUniformity	40.55886441
wavelet-HHH	glrlm	ShortRunEmphasis	0.971848946
wavelet-HHH	glrlm	LongRunHighGrayLevelEmphasis	70.6614897
wavelet-HHH	glrlm	RunPercentage	0.964102564
wavelet-HHH	glrlm	LongRunLowGrayLevelEmphasis	0.072801306
wavelet-HHH	glrlm	RunEntropy	3.478640916
wavelet-HHH	glrlm	HighGrayLevelRunEmphasis	63.8288171
wavelet-HHH	glrlm	RunLengthNonUniformityNormalized	0.931849698
wavelet-HHH	glszm	GrayLevelVariance	11.79698217
wavelet-HHH	glszm	ZoneVariance	1.407407407
wavelet-HHH	glszm	GrayLevelNonUniformityNormalized	0.102880658
wavelet-HHH	glszm	SizeZoneNonUniformityNormalized	0.465020576
wavelet-HHH	glszm	SizeZoneNonUniformity	12.55555556
wavelet-HHH	glszm	GrayLevelNonUniformity	2.777777778
wavelet-HHH	glszm	LargeAreaEmphasis	4.185185185
wavelet-HHH	glszm	SmallAreaHighGrayLevelEmphasis	44.95559671
wavelet-HHH	glszm	ZonePercentage	0.6
wavelet-HHH	glszm	LargeAreaLowGrayLevelEmphasis	0.16940774
wavelet-HHH	glszm	LargeAreaHighGrayLevelEmphasis	236.8518519
wavelet-HHH	glszm	HighGrayLevelZoneEmphasis	66.66666667
wavelet-HHH	glszm	SmallAreaEmphasis	0.678436214
wavelet-HHH	glszm	LowGrayLevelZoneEmphasis	0.099963966
wavelet-HHH	glszm	ZoneEntropy	4.032303243
wavelet-HHH	glszm	SmallAreaLowGrayLevelEmphasis	0.093667103
wavelet-HHH	ngtdm	Coarseness	0.116035098
wavelet-HHH	ngtdm	Complexity	151.3778477

wavelet-HHH	ngtdm	Strength	5.962200691
wavelet-HHH	ngtdm	Contrast	0.247981602
wavelet-HHH	ngtdm	Busyness	0.10347217
wavelet-HLL	gldm	GrayLevelVariance	79.81432099
wavelet-HLL	gldm	HighGrayLevelEmphasis	392.7111111
wavelet-HLL	gldm	DependenceEntropy	4.763966707
wavelet-HLL	gldm	DependenceNonUniformity	20.42222222
wavelet-HLL	gldm	GrayLevelNonUniformity	2.333333333
wavelet-HLL	gldm	SmallDependenceEmphasis	0.65632716
wavelet-HLL	gldm	SmallDependenceHighGrayLevelEmphasis	230.7222222
wavelet-HLL	gldm	DependenceNonUniformityNormalized	0.45382716
wavelet-HLL	gldm	LargeDependenceEmphasis	2.822222222
wavelet-HLL	gldm	LargeDependenceLowGrayLevelEmphasis	0.043962099
wavelet-HLL	gldm	DependenceVariance	0.471111111
wavelet-HLL	gldm	LargeDependenceHighGrayLevelEmphasis	1298.444444
wavelet-HLL	gldm	SmallDependenceLowGrayLevelEmphasis	0.034967225
wavelet-HLL	gldm	LowGrayLevelEmphasis	0.036610807
wavelet-HLL	glcm	JointAverage	17.9948908
wavelet-HLL	glcm	SumAverage	35.98978161
wavelet-HLL	glcm	JointEntropy	5.386718162
wavelet-HLL	glcm	ClusterShade	447.3275094
wavelet-HLL	glcm	MaximumProbability	0.03807984
wavelet-HLL	glcm	Idmn	0.942179916
wavelet-HLL	glcm	JointEnergy	0.024698479
wavelet-HLL	glcm	Contrast	91.92714836
wavelet-HLL	glcm	DifferenceEntropy	3.317788653
wavelet-HLL	glcm	InverseVariance	0.171715378
wavelet-HLL	glcm	DifferenceVariance	25.16583714
wavelet-HLL	glcm	Idn	0.843841161
wavelet-HLL	glcm	Idm	0.137913433
wavelet-HLL	glcm	Correlation	0.313659712
wavelet-HLL	glcm	Autocorrelation	348.1352549
wavelet-HLL	glcm	SumEntropy	4.033376366
wavelet-HLL	glcm	SumSquares	69.17513294
wavelet-HLL	glcm	ClusterProminence	94370.56771
wavelet-HLL	glcm	Imc2	0.998500979
wavelet-HLL	glcm	Imc1	-0.707011135
wavelet-HLL	glcm	DifferenceAverage	7.576874921
wavelet-HLL	glcm	Id	0.220782978
wavelet-HLL	glcm	ClusterTendency	184.7733834
wavelet-HLL	firstorder	InterquartileRange	325.7820695
wavelet-HLL	firstorder	Skewness	0.153542452
wavelet-HLL	firstorder	Uniformity	0.051851852
wavelet-HLL	firstorder	Median	22.53977182
wavelet-HLL	firstorder	Energy	2257379.047
wavelet-HLL	firstorder	RobustMeanAbsoluteDeviation	115.0132027
wavelet-HLL	firstorder	MeanAbsoluteDeviation	175.9119773
wavelet-HLL	firstorder	TotalEnergy	60949234.27
wavelet-HLL	firstorder	Maximum	465.4523869
wavelet-HLL	firstorder	RootMeanSquared	223.9731654
wavelet-HLL	firstorder	90Percentile	265.3172587
wavelet-HLL	firstorder	Minimum	-446.5018306
wavelet-HLL	firstorder	Entropy	4.446974207
wavelet-HLL	firstorder	Range	911.9542175
wavelet-HLL	firstorder	Variance	49729.94992

wavelet-HLL	firstorder	10Percentile	-308.0722534
wavelet-HLL	firstorder	Kurtosis	2.676298045
wavelet-HLL	firstorder	Mean	-20.83336041
wavelet-HLL	glrlm	ShortRunLowGrayLevelEmphasis	0.037231492
wavelet-HLL	glrlm	GrayLevelVariance	80.207752
wavelet-HLL	glrlm	LowGrayLevelRunEmphasis	0.037301123
wavelet-HLL	glrlm	GrayLevelNonUniformityNormalized	0.051388759
wavelet-HLL	glrlm	RunVariance	0.020467449
wavelet-HLL	glrlm	GrayLevelNonUniformity	2.265552277
wavelet-HLL	glrlm	LongRunEmphasis	1.064174198
wavelet-HLL	glrlm	ShortRunHighGrayLevelEmphasis	382.8375841
wavelet-HLL	glrlm	RunLengthNonUniformity	42.30984132
wavelet-HLL	glrlm	ShortRunEmphasis	0.983956451
wavelet-HLL	glrlm	LongRunHighGrayLevelEmphasis	421.9965138
wavelet-HLL	glrlm	RunPercentage	0.979487179
wavelet-HLL	glrlm	LongRunLowGrayLevelEmphasis	0.037579646
wavelet-HLL	glrlm	RunEntropy	4.494760079
wavelet-HLL	glrlm	HighGrayLevelRunEmphasis	390.66937
wavelet-HLL	glrlm	RunLengthNonUniformityNormalized	0.959065101
wavelet-HLL	glszm	GrayLevelVariance	85.07698962
wavelet-HLL	glszm	ZoneVariance	0.39532872
wavelet-HLL	glszm	GrayLevelNonUniformityNormalized	0.046712803
wavelet-HLL	glszm	SizeZoneNonUniformityNormalized	0.596885813
wavelet-HLL	glszm	SizeZoneNonUniformity	20.29411765
wavelet-HLL	glszm	GrayLevelNonUniformity	1.588235294
wavelet-HLL	glszm	LargeAreaEmphasis	2.147058824
wavelet-HLL	glszm	SmallAreaHighGrayLevelEmphasis	271.4191176
wavelet-HLL	glszm	ZonePercentage	0.755555556
wavelet-HLL	glszm	LargeAreaLowGrayLevelEmphasis	0.051732739
wavelet-HLL	glszm	LargeAreaHighGrayLevelEmphasis	930.6470588
wavelet-HLL	glszm	HighGrayLevelZoneEmphasis	365.1470588
wavelet-HLL	glszm	SmallAreaEmphasis	0.795955882
wavelet-HLL	glszm	LowGrayLevelZoneEmphasis	0.046970037
wavelet-HLL	glszm	ZoneEntropy	4.712319091
wavelet-HLL	glszm	SmallAreaLowGrayLevelEmphasis	0.045922974
wavelet-HLL	ngtdm	Coarseness	0.091186654
wavelet-HLL	ngtdm	Complexity	1719.114163
wavelet-HLL	ngtdm	Strength	39.95442121
wavelet-HLL	ngtdm	Contrast	1.398791195
wavelet-HLL	ngtdm	Busyness	0.027138873
wavelet-LHL	gldm	GrayLevelVariance	179.2069136
wavelet-LHL	gldm	HighGrayLevelEmphasis	927.5333333
wavelet-LHL	gldm	DependenceEntropy	4.802529763
wavelet-LHL	gldm	DependenceNonUniformity	16.91111111
wavelet-LHL	gldm	GrayLevelNonUniformity	2.288888889
wavelet-LHL	gldm	SmallDependenceEmphasis	0.613888889
wavelet-LHL	gldm	SmallDependenceHighGrayLevelEmphasis	567.0296296
wavelet-LHL	gldm	DependenceNonUniformityNormalized	0.375802469
wavelet-LHL	gldm	LargeDependenceEmphasis	3.933333333
wavelet-LHL	gldm	LargeDependenceLowGrayLevelEmphasis	0.036232932
wavelet-LHL	gldm	DependenceVariance	0.851358025
wavelet-LHL	gldm	LargeDependenceHighGrayLevelEmphasis	3655.4
wavelet-LHL	gldm	SmallDependenceLowGrayLevelEmphasis	0.027281624
wavelet-LHL	gldm	LowGrayLevelEmphasis	0.028468179
wavelet-LHL	glcm	JointAverage	27.75315304

wavelet-LHL	glcm	SumAverage	55.50630608
wavelet-LHL	glcm	JointEntropy	5.395913499
wavelet-LHL	glcm	ClusterShade	-3210.369461
wavelet-LHL	glcm	MaximumProbability	0.039355452
wavelet-LHL	glcm	Idmn	0.92328694
wavelet-LHL	glcm	JointEnergy	0.024656817
wavelet-LHL	glcm	Contrast	226.3910868
wavelet-LHL	glcm	DifferenceEntropy	3.709527624
wavelet-LHL	glcm	InverseVariance	0.13520854
wavelet-LHL	glcm	DifferenceVariance	80.63202526
wavelet-LHL	glcm	Idn	0.831240684
wavelet-LHL	glcm	Idm	0.134519407
wavelet-LHL	glcm	Correlation	0.337233139
wavelet-LHL	glcm	Autocorrelation	832.0823143
wavelet-LHL	glcm	SumEntropy	4.173605394
wavelet-LHL	glcm	SumSquares	173.0343647
wavelet-LHL	glcm	ClusterProminence	489400.9803
wavelet-LHL	glcm	Imc2	0.999319746
wavelet-LHL	glcm	Imc1	-0.770292118
wavelet-LHL	glcm	DifferenceAverage	11.24499442
wavelet-LHL	glcm	Id	0.205585524
wavelet-LHL	glcm	ClusterTendency	465.7463718
wavelet-LHL	firstorder	InterquartileRange	543.9933359
wavelet-LHL	firstorder	Skewness	-0.386535011
wavelet-LHL	firstorder	Uniformity	0.050864198
wavelet-LHL	firstorder	Median	89.75112292
wavelet-LHL	firstorder	Energy	5067402.359
wavelet-LHL	firstorder	RobustMeanAbsoluteDeviation	223.08081
wavelet-LHL	firstorder	MeanAbsoluteDeviation	291.3763096
wavelet-LHL	firstorder	TotalEnergy	136819863.7
wavelet-LHL	firstorder	Maximum	491.5211792
wavelet-LHL	firstorder	RootMeanSquared	335.5725575
wavelet-LHL	firstorder	90Percentile	385.9066872
wavelet-LHL	firstorder	Minimum	-682.6771497
wavelet-LHL	firstorder	Entropy	4.56131303
wavelet-LHL	firstorder	Range	1174.198329
wavelet-LHL	firstorder	Variance	111677.5667
wavelet-LHL	firstorder	10Percentile	-517.6632996
wavelet-LHL	firstorder	Kurtosis	1.905389555
wavelet-LHL	firstorder	Mean	-30.51843129
wavelet-LHL	glrlm	ShortRunLowGrayLevelEmphasis	0.029188761
wavelet-LHL	glrlm	GrayLevelVariance	181.3998464
wavelet-LHL	glrlm	LowGrayLevelRunEmphasis	0.029252758
wavelet-LHL	glrlm	GrayLevelNonUniformityNormalized	0.049601376
wavelet-LHL	glrlm	RunVariance	0.028960749
wavelet-LHL	glrlm	GrayLevelNonUniformity	2.167444505
wavelet-LHL	glrlm	LongRunEmphasis	1.091671658
wavelet-LHL	glrlm	ShortRunHighGrayLevelEmphasis	906.0869187
wavelet-LHL	glrlm	RunLengthNonUniformity	41.2116882
wavelet-LHL	glrlm	ShortRunEmphasis	0.977082086
wavelet-LHL	glrlm	LongRunHighGrayLevelEmphasis	1012.778177
wavelet-LHL	glrlm	RunPercentage	0.970940171
wavelet-LHL	glrlm	LongRunLowGrayLevelEmphasis	0.029508747
wavelet-LHL	glrlm	RunEntropy	4.643866864
wavelet-LHL	glrlm	HighGrayLevelRunEmphasis	927.4251705

wavelet-LHL	glrlm	RunLengthNonUniformityNormalized	0.942078502
wavelet-LHL	glszm	GrayLevelVariance	207.125
wavelet-LHL	glszm	ZoneVariance	0.741210938
wavelet-LHL	glszm	GrayLevelNonUniformityNormalized	0.041015625
wavelet-LHL	glszm	SizeZoneNonUniformityNormalized	0.591796875
wavelet-LHL	glszm	SizeZoneNonUniformity	18.9375
wavelet-LHL	glszm	GrayLevelNonUniformity	1.3125
wavelet-LHL	glszm	LargeAreaEmphasis	2.71875
wavelet-LHL	glszm	SmallAreaHighGrayLevelEmphasis	733.6871875
wavelet-LHL	glszm	ZonePercentage	0.711111111
wavelet-LHL	glszm	LargeAreaLowGrayLevelEmphasis	0.043235751
wavelet-LHL	glszm	LargeAreaHighGrayLevelEmphasis	2578.21875
wavelet-LHL	glszm	HighGrayLevelZoneEmphasis	922.6875
wavelet-LHL	glszm	SmallAreaEmphasis	0.797256944
wavelet-LHL	glszm	LowGrayLevelZoneEmphasis	0.038840098
wavelet-LHL	glszm	ZoneEntropy	4.851409766
wavelet-LHL	glszm	SmallAreaLowGrayLevelEmphasis	0.038150441
wavelet-LHL	ngtdm	Coarseness	0.069819008
wavelet-LHL	ngtdm	Complexity	3902.96708
wavelet-LHL	ngtdm	Strength	68.20332125
wavelet-LHL	ngtdm	Contrast	3.474764237
wavelet-LHL	ngtdm	Busyness	0.021612354
wavelet-LHH	gldm	GrayLevelVariance	14.40987654
wavelet-LHH	gldm	HighGrayLevelEmphasis	80.2
wavelet-LHH	gldm	DependenceEntropy	4.463749485
wavelet-LHH	gldm	DependenceNonUniformity	14.15555556
wavelet-LHH	gldm	GrayLevelNonUniformity	4.022222222
wavelet-LHH	gldm	SmallDependenceEmphasis	0.477395062
wavelet-LHH	gldm	SmallDependenceHighGrayLevelEmphasis	37.15161728
wavelet-LHH	gldm	DependenceNonUniformityNormalized	0.314567901
wavelet-LHH	gldm	LargeDependenceEmphasis	5.177777778
wavelet-LHH	gldm	LargeDependenceLowGrayLevelEmphasis	0.136149194
wavelet-LHH	gldm	DependenceVariance	1.088395062
wavelet-LHH	gldm	LargeDependenceHighGrayLevelEmphasis	413.0444444
wavelet-LHH	gldm	SmallDependenceLowGrayLevelEmphasis	0.040611582
wavelet-LHH	gldm	LowGrayLevelEmphasis	0.052872111
wavelet-LHH	glcm	JointAverage	7.909034983
wavelet-LHH	glcm	SumAverage	15.81806997
wavelet-LHH	glcm	JointEntropy	5.156071404
wavelet-LHH	glcm	ClusterShade	33.37620278
wavelet-LHH	glcm	MaximumProbability	0.054025934
wavelet-LHH	glcm	Idmn	0.939773695
wavelet-LHH	glcm	JointEnergy	0.030686181
wavelet-LHH	glcm	Contrast	26.2513886
wavelet-LHH	glcm	DifferenceEntropy	2.921317592
wavelet-LHH	glcm	InverseVariance	0.245844386
wavelet-LHH	glcm	DifferenceVariance	9.344941975
wavelet-LHH	glcm	Idn	0.840550818
wavelet-LHH	glcm	Idm	0.222860049
wavelet-LHH	glcm	Correlation	-0.073946149
wavelet-LHH	glcm	Autocorrelation	61.80876926
wavelet-LHH	glcm	SumEntropy	3.47716489
wavelet-LHH	glcm	SumSquares	12.32902216
wavelet-LHH	glcm	ClusterProminence	1830.407281
wavelet-LHH	glcm	Imc2	0.988570303

wavelet-LHH	glcm	Imc1	-0.554093105
wavelet-LHH	glcm	DifferenceAverage	4.002733207
wavelet-LHH	glcm	Id	0.314396485
wavelet-LHH	glcm	ClusterTendency	23.06470006
wavelet-LHH	firstorder	InterquartileRange	136.0983992
wavelet-LHH	firstorder	Skewness	0.444081639
wavelet-LHH	firstorder	Uniformity	0.089382716
wavelet-LHH	firstorder	Median	-27.13207427
wavelet-LHH	firstorder	Energy	394492.2215
wavelet-LHH	firstorder	RobustMeanAbsoluteDeviation	53.5486816
wavelet-LHH	firstorder	MeanAbsoluteDeviation	76.18482888
wavelet-LHH	firstorder	TotalEnergy	10651289.98
wavelet-LHH	firstorder	Maximum	251.1586874
wavelet-LHH	firstorder	RootMeanSquared	93.62955628
wavelet-LHH	firstorder	90Percentile	109.2799862
wavelet-LHH	firstorder	Minimum	-186.774129
wavelet-LHH	firstorder	Entropy	3.68331803
wavelet-LHH	firstorder	Range	437.9328164
wavelet-LHH	firstorder	Variance	8658.114452
wavelet-LHH	firstorder	10Percentile	-125.4573268
wavelet-LHH	firstorder	Kurtosis	2.90395307
wavelet-LHH	firstorder	Mean	-10.41054071
wavelet-LHH	glrlm	ShortRunLowGrayLevelEmphasis	0.053536605
wavelet-LHH	glrlm	GrayLevelVariance	14.62214563
wavelet-LHH	glrlm	LowGrayLevelRunEmphasis	0.054195488
wavelet-LHH	glrlm	GrayLevelNonUniformityNormalized	0.088201828
wavelet-LHH	glrlm	RunVariance	0.039138057
wavelet-LHH	glrlm	GrayLevelNonUniformity	3.812702707
wavelet-LHH	glrlm	LongRunEmphasis	1.124936606
wavelet-LHH	glrlm	ShortRunHighGrayLevelEmphasis	77.52814595
wavelet-LHH	glrlm	RunLengthNonUniformity	39.90194434
wavelet-LHH	glrlm	ShortRunEmphasis	0.968765848
wavelet-LHH	glrlm	LongRunHighGrayLevelEmphasis	90.41063021
wavelet-LHH	glrlm	RunPercentage	0.960683761
wavelet-LHH	glrlm	LongRunLowGrayLevelEmphasis	0.056831019
wavelet-LHH	glrlm	RunEntropy	3.800881856
wavelet-LHH	glrlm	HighGrayLevelRunEmphasis	80.1046428
wavelet-LHH	glrlm	RunLengthNonUniformityNormalized	0.921723887
wavelet-LHH	glszm	GrayLevelVariance	17.58024691
wavelet-LHH	glszm	ZoneVariance	1.185185185
wavelet-LHH	glszm	GrayLevelNonUniformityNormalized	0.0781893
wavelet-LHH	glszm	SizeZoneNonUniformityNormalized	0.432098765
wavelet-LHH	glszm	SizeZoneNonUniformity	11.66666667
wavelet-LHH	glszm	GrayLevelNonUniformity	2.111111111
wavelet-LHH	glszm	LargeAreaEmphasis	3.962962963
wavelet-LHH	glszm	SmallAreaHighGrayLevelEmphasis	52.47942387
wavelet-LHH	glszm	ZonePercentage	0.6
wavelet-LHH	glszm	LargeAreaLowGrayLevelEmphasis	0.135618061
wavelet-LHH	glszm	LargeAreaHighGrayLevelEmphasis	303.8888889
wavelet-LHH	glszm	HighGrayLevelZoneEmphasis	79.81481481
wavelet-LHH	glszm	SmallAreaEmphasis	0.670781893
wavelet-LHH	glszm	LowGrayLevelZoneEmphasis	0.073033774
wavelet-LHH	glszm	ZoneEntropy	4.282484261
wavelet-LHH	glszm	SmallAreaLowGrayLevelEmphasis	0.064493208
wavelet-LHH	ngtdm	Coarseness	0.095808375

wavelet-LHH	ngtdm	Complexity	297.4969328
wavelet-LHH	ngtdm	Strength	7.667871473
wavelet-LHH	ngtdm	Contrast	0.461538545
wavelet-LHH	ngtdm	Busyness	0.117657199
wavelet-LLH	gldm	GrayLevelVariance	61.89333333
wavelet-LLH	gldm	HighGrayLevelEmphasis	273.1111111
wavelet-LLH	gldm	DependenceEntropy	4.791635874
wavelet-LLH	gldm	DependenceNonUniformity	14.77777778
wavelet-LLH	gldm	GrayLevelNonUniformity	2.644444444
wavelet-LLH	gldm	SmallDependenceEmphasis	0.513888889
wavelet-LLH	gldm	SmallDependenceHighGrayLevelEmphasis	179.3447531
wavelet-LLH	gldm	DependenceNonUniformityNormalized	0.328395062
wavelet-LLH	gldm	LargeDependenceEmphasis	4.333333333
wavelet-LLH	gldm	LargeDependenceLowGrayLevelEmphasis	0.071871031
wavelet-LLH	gldm	DependenceVariance	0.765432099
wavelet-LLH	gldm	LargeDependenceHighGrayLevelEmphasis	961.4666667
wavelet-LLH	gldm	SmallDependenceLowGrayLevelEmphasis	0.03172859
wavelet-LLH	gldm	LowGrayLevelEmphasis	0.037659044
wavelet-LLH	glcm	JointAverage	13.84583868
wavelet-LLH	glcm	SumAverage	27.69167735
wavelet-LLH	glcm	JointEntropy	5.326845993
wavelet-LLH	glcm	ClusterShade	1028.823828
wavelet-LLH	glcm	MaximumProbability	0.048906088
wavelet-LLH	glcm	Idmn	0.941209462
wavelet-LLH	glcm	JointEnergy	0.026278939
wavelet-LLH	glcm	Contrast	82.6178923
wavelet-LLH	glcm	DifferenceEntropy	3.489884142
wavelet-LLH	glcm	InverseVariance	0.131247592
wavelet-LLH	glcm	DifferenceVariance	29.75760271
wavelet-LLH	glcm	Idn	0.843336262
wavelet-LLH	glcm	Idm	0.147035607
wavelet-LLH	glcm	Correlation	0.256599564
wavelet-LLH	glcm	Autocorrelation	207.4389541
wavelet-LLH	glcm	SumEntropy	4.046973055
wavelet-LLH	glcm	SumSquares	56.66512442
wavelet-LLH	glcm	ClusterProminence	60218.06717
wavelet-LLH	glcm	Imc2	0.997922797
wavelet-LLH	glcm	Imc1	-0.683523432
wavelet-LLH	glcm	DifferenceAverage	7.030934464
wavelet-LLH	glcm	Id	0.235606485
wavelet-LLH	glcm	ClusterTendency	144.0426054
wavelet-LLH	firstorder	InterquartileRange	267.7835107
wavelet-LLH	firstorder	Skewness	0.549171226
wavelet-LLH	firstorder	Uniformity	0.058765432
wavelet-LLH	firstorder	Median	-17.35750501
wavelet-LLH	firstorder	Energy	1734357.187
wavelet-LLH	firstorder	RobustMeanAbsoluteDeviation	107.9778643
wavelet-LLH	firstorder	MeanAbsoluteDeviation	156.6482169
wavelet-LLH	firstorder	TotalEnergy	46827644.04
wavelet-LLH	firstorder	Maximum	452.3343716
wavelet-LLH	firstorder	RootMeanSquared	196.3193083
wavelet-LLH	firstorder	90Percentile	232.2318757
wavelet-LLH	firstorder	Minimum	-352.5377665
wavelet-LLH	firstorder	Entropy	4.294646363
wavelet-LLH	firstorder	Range	804.8721381

wavelet-LLH	firstorder	Variance	37966.92288
wavelet-LLH	firstorder	10Percentile	-249.6564601
wavelet-LLH	firstorder	Kurtosis	2.748354271
wavelet-LLH	firstorder	Mean	-23.96555719
wavelet-LLH	glrlm	ShortRunLowGrayLevelEmphasis	0.038356156
wavelet-LLH	glrlm	GrayLevelVariance	63.00350232
wavelet-LLH	glrlm	LowGrayLevelRunEmphasis	0.038632805
wavelet-LLH	glrlm	GrayLevelNonUniformityNormalized	0.0580017
wavelet-LLH	glrlm	RunVariance	0.037829106
wavelet-LLH	glrlm	GrayLevelNonUniformity	2.521974537
wavelet-LLH	glrlm	LongRunEmphasis	1.113350022
wavelet-LLH	glrlm	ShortRunHighGrayLevelEmphasis	270.241197
wavelet-LLH	glrlm	RunLengthNonUniformity	40.70050066
wavelet-LLH	glrlm	ShortRunEmphasis	0.973799247
wavelet-LLH	glrlm	LongRunHighGrayLevelEmphasis	298.0072315
wavelet-LLH	glrlm	RunPercentage	0.965811966
wavelet-LLH	glrlm	LongRunLowGrayLevelEmphasis	0.039913826
wavelet-LLH	glrlm	RunEntropy	4.375080753
wavelet-LLH	glrlm	HighGrayLevelRunEmphasis	275.7106432
wavelet-LLH	glrlm	RunLengthNonUniformityNormalized	0.934726404
wavelet-LLH	glszm	GrayLevelVariance	79.45280612
wavelet-LLH	glszm	ZoneVariance	0.952806122
wavelet-LLH	glszm	GrayLevelNonUniformityNormalized	0.048469388
wavelet-LLH	glszm	SizeZoneNonUniformityNormalized	0.466836735
wavelet-LLH	glszm	SizeZoneNonUniformity	13.07142857
wavelet-LLH	glszm	GrayLevelNonUniformity	1.357142857
wavelet-LLH	glszm	LargeAreaEmphasis	3.535714286
wavelet-LLH	glszm	SmallAreaHighGrayLevelEmphasis	267.3065476
wavelet-LLH	glszm	ZonePercentage	0.622222222
wavelet-LLH	glszm	LargeAreaLowGrayLevelEmphasis	0.080384325
wavelet-LLH	glszm	LargeAreaHighGrayLevelEmphasis	815.5714286
wavelet-LLH	glszm	HighGrayLevelZoneEmphasis	323.0357143
wavelet-LLH	glszm	SmallAreaEmphasis	0.704801587
wavelet-LLH	glszm	LowGrayLevelZoneEmphasis	0.053347018
wavelet-LLH	glszm	ZoneEntropy	4.664497779
wavelet-LLH	glszm	SmallAreaLowGrayLevelEmphasis	0.049389476
wavelet-LLH	ngtdm	Coarseness	0.087818387
wavelet-LLH	ngtdm	Complexity	1383.205809
wavelet-LLH	ngtdm	Strength	31.07169493
wavelet-LLH	ngtdm	Contrast	1.251552176
wavelet-LLH	ngtdm	Busyness	0.05371291
wavelet-LLL	gldm	GrayLevelVariance	333.5377778
wavelet-LLL	gldm	HighGrayLevelEmphasis	1069.755556
wavelet-LLL	gldm	DependenceEntropy	5.002964207
wavelet-LLL	gldm	DependenceNonUniformity	23.4
wavelet-LLL	gldm	GrayLevelNonUniformity	1.977777778
wavelet-LLL	gldm	SmallDependenceEmphasis	0.740740741
wavelet-LLL	gldm	SmallDependenceHighGrayLevelEmphasis	737.7234568
wavelet-LLL	gldm	DependenceNonUniformityNormalized	0.52
wavelet-LLL	gldm	LargeDependenceEmphasis	2.333333333
wavelet-LLL	gldm	LargeDependenceLowGrayLevelEmphasis	0.076850872
wavelet-LLL	gldm	DependenceVariance	0.373333333
wavelet-LLL	gldm	LargeDependenceHighGrayLevelEmphasis	2707.711111
wavelet-LLL	gldm	SmallDependenceLowGrayLevelEmphasis	0.032807589
wavelet-LLL	gldm	LowGrayLevelEmphasis	0.041559526

wavelet-LLL	glcm	JointAverage	32.07494257
wavelet-LLL	glcm	SumAverage	64.14988514
wavelet-LLL	glcm	JointEntropy	5.437646168
wavelet-LLL	glcm	ClusterShade	420.1552953
wavelet-LLL	glcm	MaximumProbability	0.039319002
wavelet-LLL	glcm	Idmn	0.894989873
wavelet-LLL	glcm	JointEnergy	0.023817085
wavelet-LLL	glcm	Contrast	613.2562196
wavelet-LLL	glcm	DifferenceEntropy	4.042694702
wavelet-LLL	glcm	InverseVariance	0.069862056
wavelet-LLL	glcm	DifferenceVariance	194.5614517
wavelet-LLL	glcm	Idn	0.788375679
wavelet-LLL	glcm	Idm	0.070071446
wavelet-LLL	glcm	Correlation	0.155082694
wavelet-LLL	glcm	Autocorrelation	1084.464939
wavelet-LLL	glcm	SumEntropy	4.289363638
wavelet-LLL	glcm	SumSquares	361.1384947
wavelet-LLL	glcm	ClusterProminence	1513796.43
wavelet-LLL	glcm	Imc2	0.999522137
wavelet-LLL	glcm	Imc1	-0.786438074
wavelet-LLL	glcm	DifferenceAverage	20.20146309
wavelet-LLL	glcm	Id	0.122834292
wavelet-LLL	glcm	ClusterTendency	831.2977592
wavelet-LLL	firstorder	InterquartileRange	635.7983043
wavelet-LLL	firstorder	Skewness	0.424428501
wavelet-LLL	firstorder	Uniformity	0.043950617
wavelet-LLL	firstorder	Median	-1220.315058
wavelet-LLL	firstorder	Energy	75076740.91
wavelet-LLL	firstorder	RobustMeanAbsoluteDeviation	282.441122
wavelet-LLL	firstorder	MeanAbsoluteDeviation	380.6671534
wavelet-LLL	firstorder	TotalEnergy	2027072005
wavelet-LLL	firstorder	Maximum	-209.7458023
wavelet-LLL	firstorder	RootMeanSquared	1291.654761
wavelet-LLL	firstorder	90Percentile	-490.4110667
wavelet-LLL	firstorder	Minimum	-1859.303001
wavelet-LLL	firstorder	Entropy	4.719522263
wavelet-LLL	firstorder	Range	1649.557199
wavelet-LLL	firstorder	Variance	209460.883
wavelet-LLL	firstorder	10Percentile	-1790.869536
wavelet-LLL	firstorder	Kurtosis	2.163650394
wavelet-LLL	firstorder	Mean	-1207.853939
wavelet-LLL	glrlm	ShortRunLowGrayLevelEmphasis	0.041392097
wavelet-LLL	glrlm	GrayLevelVariance	334.311818
wavelet-LLL	glrlm	LowGrayLevelRunEmphasis	0.041745547
wavelet-LLL	glrlm	GrayLevelNonUniformityNormalized	0.043394752
wavelet-LLL	glrlm	RunVariance	0.015371039
wavelet-LLL	glrlm	GrayLevelNonUniformity	1.922986574
wavelet-LLL	glrlm	LongRunEmphasis	1.047446739
wavelet-LLL	glrlm	ShortRunHighGrayLevelEmphasis	1052.216165
wavelet-LLL	glrlm	RunLengthNonUniformity	42.96186372
wavelet-LLL	glrlm	ShortRunEmphasis	0.988138315
wavelet-LLL	glrlm	LongRunHighGrayLevelEmphasis	1126.055154
wavelet-LLL	glrlm	RunPercentage	0.984615385
wavelet-LLL	glrlm	LongRunLowGrayLevelEmphasis	0.043159348
wavelet-LLL	glrlm	RunEntropy	4.756379487

wavelet-LLL	glrlm	HighGrayLevelRunEmphasis	1066.983963
wavelet-LLL	glrlm	RunLengthNonUniformityNormalized	0.969257923
wavelet-LLL	glszm	GrayLevelVariance	346.1080247
wavelet-LLL	glszm	ZoneVariance	0.409722222
wavelet-LLL	glszm	GrayLevelNonUniformityNormalized	0.037037037
wavelet-LLL	glszm	SizeZoneNonUniformityNormalized	0.708333333
wavelet-LLL	glszm	SizeZoneNonUniformity	25.5
wavelet-LLL	glszm	GrayLevelNonUniformity	1.333333333
wavelet-LLL	glszm	LargeAreaEmphasis	1.972222222
wavelet-LLL	glszm	SmallAreaHighGrayLevelEmphasis	846.6736111
wavelet-LLL	glszm	ZonePercentage	0.8
wavelet-LLL	glszm	LargeAreaLowGrayLevelEmphasis	0.066814737
wavelet-LLL	glszm	LargeAreaHighGrayLevelEmphasis	2192.75
wavelet-LLL	glszm	HighGrayLevelZoneEmphasis	1025
wavelet-LLL	glszm	SmallAreaEmphasis	0.865933642
wavelet-LLL	glszm	LowGrayLevelZoneEmphasis	0.044623631
wavelet-LLL	glszm	ZoneEntropy	4.947702779
wavelet-LLL	glszm	SmallAreaLowGrayLevelEmphasis	0.03918049
wavelet-LLL	ngtdm	Coarseness	0.044377632
wavelet-LLL	ngtdm	Complexity	10039.35714
wavelet-LLL	ngtdm	Strength	70.58219689
wavelet-LLL	ngtdm	Contrast	10.39381648
wavelet-LLL	ngtdm	Busyness	0.029475738
wavelet-HHL	gldm	GrayLevelVariance	12.23506173
wavelet-HHL	gldm	HighGrayLevelEmphasis	108.7111111
wavelet-HHL	gldm	DependenceEntropy	4.341310041
wavelet-HHL	gldm	DependenceNonUniformity	13.08888889
wavelet-HHL	gldm	GrayLevelNonUniformity	4.244444444
wavelet-HHL	gldm	SmallDependenceEmphasis	0.482216049
wavelet-HHL	gldm	SmallDependenceHighGrayLevelEmphasis	53.93101235
wavelet-HHL	gldm	DependenceNonUniformityNormalized	0.290864198
wavelet-HHL	gldm	LargeDependenceEmphasis	5.355555556
wavelet-HHL	gldm	LargeDependenceLowGrayLevelEmphasis	0.235699771
wavelet-HHL	gldm	DependenceVariance	1.084444444
wavelet-HHL	gldm	LargeDependenceHighGrayLevelEmphasis	563.8666667
wavelet-HHL	gldm	SmallDependenceLowGrayLevelEmphasis	0.0193317
wavelet-HHL	gldm	LowGrayLevelEmphasis	0.057616193
wavelet-HHL	glcm	JointAverage	9.991043285
wavelet-HHL	glcm	SumAverage	19.98208657
wavelet-HHL	glcm	JointEntropy	5.094904316
wavelet-HHL	glcm	ClusterShade	-23.32119487
wavelet-HHL	glcm	MaximumProbability	0.058139985
wavelet-HHL	glcm	Idmn	0.931371366
wavelet-HHL	glcm	JointEnergy	0.032438755
wavelet-HHL	glcm	Contrast	21.93187563
wavelet-HHL	glcm	DifferenceEntropy	2.836891495
wavelet-HHL	glcm	InverseVariance	0.213700217
wavelet-HHL	glcm	DifferenceVariance	7.311588779
wavelet-HHL	glcm	Idn	0.826305295
wavelet-HHL	glcm	Idm	0.214513633
wavelet-HHL	glcm	Correlation	0.001200315
wavelet-HHL	glcm	Autocorrelation	100.2772448
wavelet-HHL	glcm	SumEntropy	3.553829712
wavelet-HHL	glcm	SumSquares	11.28324732
wavelet-HHL	glcm	ClusterProminence	1559.321261

wavelet-HHL	glcm	Imc2	0.986599132
wavelet-HHL	glcm	Imc1	-0.535009706
wavelet-HHL	glcm	DifferenceAverage	3.722219374
wavelet-HHL	glcm	Id	0.314939348
wavelet-HHL	glcm	ClusterTendency	23.20111365
wavelet-HHL	firstorder	InterquartileRange	132.8731622
wavelet-HHL	firstorder	Skewness	-0.556134472
wavelet-HHL	firstorder	Uniformity	0.094320988
wavelet-HHL	firstorder	Median	12.07839806
wavelet-HHL	firstorder	Energy	349343.1303
wavelet-HHL	firstorder	RobustMeanAbsoluteDeviation	47.09744845
wavelet-HHL	firstorder	MeanAbsoluteDeviation	69.53619871
wavelet-HHL	firstorder	TotalEnergy	9432264.518
wavelet-HHL	firstorder	Maximum	166.7158201
wavelet-HHL	firstorder	RootMeanSquared	88.1089137
wavelet-HHL	firstorder	90Percentile	110.9630722
wavelet-HHL	firstorder	Minimum	-212.2875159
wavelet-HHL	firstorder	Entropy	3.583179421
wavelet-HHL	firstorder	Range	379.003336
wavelet-HHL	firstorder	Variance	7696.793443
wavelet-HHL	firstorder	10Percentile	-103.5114093
wavelet-HHL	firstorder	Kurtosis	2.978096254
wavelet-HHL	firstorder	Mean	8.147835958
wavelet-HHL	glrlm	ShortRunLowGrayLevelEmphasis	0.055986653
wavelet-HHL	glrlm	GrayLevelVariance	12.44596923
wavelet-HHL	glrlm	LowGrayLevelRunEmphasis	0.057748406
wavelet-HHL	glrlm	GrayLevelNonUniformityNormalized	0.092764872
wavelet-HHL	glrlm	RunVariance	0.056182509
wavelet-HHL	glrlm	GrayLevelNonUniformity	4.00525517
wavelet-HHL	glrlm	LongRunEmphasis	1.148141513
wavelet-HHL	glrlm	ShortRunHighGrayLevelEmphasis	105.7299349
wavelet-HHL	glrlm	RunLengthNonUniformity	40.09738711
wavelet-HHL	glrlm	ShortRunEmphasis	0.970546961
wavelet-HHL	glrlm	LongRunHighGrayLevelEmphasis	123.9628417
wavelet-HHL	glrlm	RunPercentage	0.958974359
wavelet-HHL	glrlm	LongRunLowGrayLevelEmphasis	0.065209402
wavelet-HHL	glrlm	RunEntropy	3.720208364
wavelet-HHL	glrlm	HighGrayLevelRunEmphasis	108.8955044
wavelet-HHL	glrlm	RunLengthNonUniformityNormalized	0.927332487
wavelet-HHL	glszm	GrayLevelVariance	15.0591716
wavelet-HHL	glszm	ZoneVariance	1.658284024
wavelet-HHL	glszm	GrayLevelNonUniformityNormalized	0.082840237
wavelet-HHL	glszm	SizeZoneNonUniformityNormalized	0.479289941
wavelet-HHL	glszm	SizeZoneNonUniformity	12.46153846
wavelet-HHL	glszm	GrayLevelNonUniformity	2.153846154
wavelet-HHL	glszm	LargeAreaEmphasis	4.653846154
wavelet-HHL	glszm	SmallAreaHighGrayLevelEmphasis	79.76042735
wavelet-HHL	glszm	ZonePercentage	0.577777778
wavelet-HHL	glszm	LargeAreaLowGrayLevelEmphasis	0.20519256
wavelet-HHL	glszm	LargeAreaHighGrayLevelEmphasis	517.5384615
wavelet-HHL	glszm	HighGrayLevelZoneEmphasis	109
wavelet-HHL	glszm	SmallAreaEmphasis	0.710811966
wavelet-HHL	glszm	LowGrayLevelZoneEmphasis	0.054247287
wavelet-HHL	glszm	ZoneEntropy	4.132944045
wavelet-HHL	glszm	SmallAreaLowGrayLevelEmphasis	0.022716218

wavelet-HHL	ngtdm	Coarseness	0.108224325
wavelet-HHL	ngtdm	Complexity	208.1461888
wavelet-HHL	ngtdm	Strength	6.932579604
wavelet-HHL	ngtdm	Contrast	0.371964709
wavelet-HHL	ngtdm	Busyness	0.085485816
wavelet-HLH	gldm	GrayLevelVariance	8.017777778
wavelet-HLH	gldm	HighGrayLevelEmphasis	57.95555556
wavelet-HLH	gldm	DependenceEntropy	4.58618893
wavelet-HLH	gldm	DependenceNonUniformity	11.13333333
wavelet-HLH	gldm	GrayLevelNonUniformity	5.4
wavelet-HLH	gldm	SmallDependenceEmphasis	0.401895062
wavelet-HLH	gldm	SmallDependenceHighGrayLevelEmphasis	30.01967901
wavelet-HLH	gldm	DependenceNonUniformityNormalized	0.247407407
wavelet-HLH	gldm	LargeDependenceEmphasis	7.177777778
wavelet-HLH	gldm	LargeDependenceLowGrayLevelEmphasis	0.231197816
wavelet-HLH	gldm	DependenceVariance	1.523950617
wavelet-HLH	gldm	LargeDependenceHighGrayLevelEmphasis	359.3777778
wavelet-HLH	gldm	SmallDependenceLowGrayLevelEmphasis	0.032397751
wavelet-HLH	gldm	LowGrayLevelEmphasis	0.053706005
wavelet-HLH	glcm	JointAverage	7.098516926
wavelet-HLH	glcm	SumAverage	14.19703385
wavelet-HLH	glcm	JointEntropy	5.023501218
wavelet-HLH	glcm	ClusterShade	17.39371678
wavelet-HLH	glcm	MaximumProbability	0.068284232
wavelet-HLH	glcm	Idmn	0.930491048
wavelet-HLH	glcm	JointEnergy	0.034456797
wavelet-HLH	glcm	Contrast	16.37780619
wavelet-HLH	glcm	DifferenceEntropy	2.850585683
wavelet-HLH	glcm	InverseVariance	0.216584747
wavelet-HLH	glcm	DifferenceVariance	5.405355274
wavelet-HLH	glcm	Idn	0.825164324
wavelet-HLH	glcm	Idm	0.247973211
wavelet-HLH	glcm	Correlation	0.000516732
wavelet-HLH	glcm	Autocorrelation	50.48216428
wavelet-HLH	glcm	SumEntropy	3.444552677
wavelet-HLH	glcm	SumSquares	8.249854971
wavelet-HLH	glcm	ClusterProminence	796.0516601
wavelet-HLH	glcm	Imc2	0.966099562
wavelet-HLH	glcm	Imc1	-0.434144497
wavelet-HLH	glcm	DifferenceAverage	3.272260189
wavelet-HLH	glcm	Id	0.346494466
wavelet-HLH	glcm	ClusterTendency	16.62161369
wavelet-HLH	firstorder	InterquartileRange	83.7698416
wavelet-HLH	firstorder	Skewness	0.24034703
wavelet-HLH	firstorder	Uniformity	0.12
wavelet-HLH	firstorder	Median	-10.29730626
wavelet-HLH	firstorder	Energy	232983.6405
wavelet-HLH	firstorder	RobustMeanAbsoluteDeviation	35.73972157
wavelet-HLH	firstorder	MeanAbsoluteDeviation	54.93968171
wavelet-HLH	firstorder	TotalEnergy	6290558.294
wavelet-HLH	firstorder	Maximum	165.8041768
wavelet-HLH	firstorder	RootMeanSquared	71.95425098
wavelet-HLH	firstorder	90Percentile	78.03451854
wavelet-HLH	firstorder	Minimum	-172.4798069
wavelet-HLH	firstorder	Entropy	3.297874075

wavelet-HLH	firstorder	Range	338.2839838
wavelet-HLH	firstorder	Variance	5068.331764
wavelet-HLH	firstorder	10Percentile	-104.2915496
wavelet-HLH	firstorder	Kurtosis	2.996057895
wavelet-HLH	firstorder	Mean	-10.44425533
wavelet-HLH	glrlm	ShortRunLowGrayLevelEmphasis	0.053676456
wavelet-HLH	glrlm	GrayLevelVariance	8.283294237
wavelet-HLH	glrlm	LowGrayLevelRunEmphasis	0.054993032
wavelet-HLH	glrlm	GrayLevelNonUniformityNormalized	0.117161944
wavelet-HLH	glrlm	RunVariance	0.052686958
wavelet-HLH	glrlm	GrayLevelNonUniformity	4.994876677
wavelet-HLH	glrlm	LongRunEmphasis	1.169950937
wavelet-HLH	glrlm	ShortRunHighGrayLevelEmphasis	56.49459021
wavelet-HLH	glrlm	RunLengthNonUniformity	38.17545118
wavelet-HLH	glrlm	ShortRunEmphasis	0.957512266
wavelet-HLH	glrlm	LongRunHighGrayLevelEmphasis	66.6606595
wavelet-HLH	glrlm	RunPercentage	0.947008547
wavelet-HLH	glrlm	LongRunLowGrayLevelEmphasis	0.060259336
wavelet-HLH	glrlm	RunEntropy	3.524036333
wavelet-HLH	glrlm	HighGrayLevelRunEmphasis	58.52780406
wavelet-HLH	glrlm	RunLengthNonUniformityNormalized	0.894626084
wavelet-HLH	glszm	GrayLevelVariance	12.20408163
wavelet-HLH	glszm	ZoneVariance	3.836734694
wavelet-HLH	glszm	GrayLevelNonUniformityNormalized	0.088435374
wavelet-HLH	glszm	SizeZoneNonUniformityNormalized	0.424036281
wavelet-HLH	glszm	SizeZoneNonUniformity	8.904761905
wavelet-HLH	glszm	GrayLevelNonUniformity	1.857142857
wavelet-HLH	glszm	LargeAreaEmphasis	8.428571429
wavelet-HLH	glszm	SmallAreaHighGrayLevelEmphasis	55.91669366
wavelet-HLH	glszm	ZonePercentage	0.466666667
wavelet-HLH	glszm	LargeAreaLowGrayLevelEmphasis	0.296462792
wavelet-HLH	glszm	LargeAreaHighGrayLevelEmphasis	418.6666667
wavelet-HLH	glszm	HighGrayLevelZoneEmphasis	71.71428571
wavelet-HLH	glszm	SmallAreaEmphasis	0.668610301
wavelet-HLH	glszm	LowGrayLevelZoneEmphasis	0.075794398
wavelet-HLH	glszm	ZoneEntropy	4.106603137
wavelet-HLH	glszm	SmallAreaLowGrayLevelEmphasis	0.061700264
wavelet-HLH	ngtdm	Coarseness	0.100603097
wavelet-HLH	ngtdm	Complexity	158.7619489
wavelet-HLH	ngtdm	Strength	5.692229588
wavelet-HLH	ngtdm	Contrast	0.247751038
wavelet-HLH	ngtdm	Busyness	0.129128848

Image type	Feature Class	Feature Name	11: RTSTRUCT: C99-DNU_segment_GTV RLL
general	info	BoundingBox	(1, 0, 1, 5, 7, 5)
general	info	EnabledImageTypes	{u'Original': {}, u'Wavelet': {}}
general	info	GeneralSettings	{'distances': [1], 'additionalInfo': True, 'force2D': False, 'interpolator': 'sitkBSpline', 'resampledPixelSpacing': [3.0], 'label': 1, 'normalizeScale': 1, 'normalize': False, 'force2Ddimension': 0, 'removeOutliers': None, 'minimumROISize': None, u'binWidth': 25.0, 'minimumROIDimensions': 1, u'correctMask': True, 'preCrop': False, u'symmetricalGLCM': True, 'resegmentRange': None, 'padDistance': 5}
general	info	ImageHash	3a081fbf94e08190510786c9b43559c48a9791fb
general	info	ImageSpacing	(1.26953125, 1.26953125, 2.0)
general	info	MaskHash	f677c81c0826f6b15ba759d23aba66b368686682
general	info	NumpyVersion	1.13.1
general	info	PyWaveletVersion	0.5.2
general	info	SimpleITKVersion	1.1.0
general	info	Version	1.3.0.post102+g1b0eb74
general	info	VolumeNum	1
general	info	VoxelNum	110
original	shape	Maximum3DDiameter	20.34698995
original	shape	Maximum2DDiameterSlice	17.49285568
original	shape	Sphericity	0.916448828
original	shape	MinorAxis	15.65513004
original	shape	Elongation	0.815104627
original	shape	SurfaceVolumeRatio	0.367105103
original	shape	Volume	2970
original	shape	MajorAxis	19.20628287
original	shape	SurfaceArea	1090.302156
original	shape	Flatness	0.719001573
original	shape	LeastAxis	13.8093476
original	shape	Maximum2DDiameterColumn	16.97056275
original	shape	Maximum2DDiameterRow	20.1246118
original	gldm	GrayLevelVariance	59.9292562
original	gldm	HighGrayLevelEmphasis	782.0727273
original	gldm	DependenceEntropy	5.361241537
original	gldm	DependenceNonUniformity	25.85454545
original	gldm	GrayLevelNonUniformity	8.963636364
original	gldm	SmallDependenceEmphasis	0.474290908
original	gldm	SmallDependenceHighGrayLevelEmphasis	268.2191194
original	gldm	DependenceNonUniformityNormalized	0.235041322
original	gldm	LargeDependenceEmphasis	12.78181818
original	gldm	LargeDependenceLowGrayLevelEmphasis	0.023270861
original	gldm	DependenceVariance	4.736859504
original	gldm	LargeDependenceHighGrayLevelEmphasis	13065.2
original	gldm	SmallDependenceLowGrayLevelEmphasis	0.011148598
original	gldm	LowGrayLevelEmphasis	0.011731831
original	glcm	JointAverage	28.50064765
original	glcm	SumAverage	57.0012953
original	glcm	JointEntropy	6.062832543
original	glcm	ClusterShade	-1723.770545
original	glcm	MaximumProbability	0.059639838
original	glcm	Idmn	0.953387623
original	glcm	JointEnergy	0.020588645

original	glcm	Contrast	72.41923794
original	glcm	DifferenceEntropy	3.653319024
original	glcm	InverseVariance	0.273041486
original	glcm	DifferenceVariance	40.4989274
original	glcm	Idn	0.879100821
original	glcm	Idm	0.264901232
original	glcm	Correlation	0.200756714
original	glcm	Autocorrelation	821.6542029
original	glcm	SumEntropy	4.318739521
original	glcm	SumSquares	45.52948578
original	glcm	ClusterProminence	61309.63283
original	glcm	Imc2	0.970847411
original	glcm	Imc1	-0.383068838
original	glcm	DifferenceAverage	5.633352438
original	glcm	Id	0.335159222
original	glcm	ClusterTendency	109.6987052
original	firstorder	InterquartileRange	217.25
original	firstorder	Skewness	-1.386387329
original	firstorder	Uniformity	0.081487603
original	firstorder	Median	-53.5
original	firstorder	Energy	6360220
original	firstorder	RobustMeanAbsoluteDeviation	103.3306401
original	firstorder	MeanAbsoluteDeviation	154.6981818
original	firstorder	TotalEnergy	171725940
original	firstorder	Maximum	72
original	firstorder	RootMeanSquared	240.4582746
original	firstorder	90Percentile	23
original	firstorder	Minimum	-781
original	firstorder	Entropy	4.136812194
original	firstorder	Range	853
original	firstorder	Variance	37826.22182
original	firstorder	10Percentile	-426.2
original	firstorder	Kurtosis	4.111826286
original	firstorder	Mean	-141.4
original	glrlm	ShortRunLowGrayLevelEmphasis	0.012500793
original	glrlm	GrayLevelVariance	61.93737655
original	glrlm	LowGrayLevelRunEmphasis	0.012555209
original	glrlm	GrayLevelNonUniformityNormalized	0.073983114
original	glrlm	RunVariance	0.083856581
original	glrlm	GrayLevelNonUniformity	7.570768607
original	glrlm	LongRunEmphasis	1.244343396
original	glrlm	ShortRunHighGrayLevelEmphasis	708.9192913
original	glrlm	RunLengthNonUniformity	89.1074329
original	glrlm	ShortRunEmphasis	0.946603158
original	glrlm	LongRunHighGrayLevelEmphasis	1013.713811
original	glrlm	RunPercentage	0.929370629
original	glrlm	LongRunLowGrayLevelEmphasis	0.012802737
original	glrlm	RunEntropy	4.471504661
original	glrlm	HighGrayLevelRunEmphasis	763.5295444
original	glrlm	RunLengthNonUniformityNormalized	0.870436018
original	glszm	GrayLevelVariance	68.89158163
original	glszm	ZoneVariance	10.49872449
original	glszm	GrayLevelNonUniformityNormalized	0.051658163
original	glszm	SizeZoneNonUniformityNormalized	0.65880102
original	glszm	SizeZoneNonUniformity	36.89285714
original	glszm	GrayLevelNonUniformity	2.892857143

original	glszm	LargeAreaEmphasis	14.35714286
original	glszm	SmallAreaHighGrayLevelEmphasis	436.7283236
original	glszm	ZonePercentage	0.509090909
original	glszm	LargeAreaLowGrayLevelEmphasis	0.035119499
original	glszm	LargeAreaHighGrayLevelEmphasis	14451.78571
original	glszm	HighGrayLevelZoneEmphasis	576.75
original	glszm	SmallAreaEmphasis	0.833101623
original	glszm	LowGrayLevelZoneEmphasis	0.022009056
original	glszm	ZoneEntropy	4.857010619
original	glszm	SmallAreaLowGrayLevelEmphasis	0.021776125
original	ngtdm	Coarseness	0.034304122
original	ngtdm	Complexity	1865.399869
original	ngtdm	Strength	21.54000184
original	ngtdm	Contrast	0.705465351
original	ngtdm	Busyness	0.028647855
wavelet-HHH	gldm	GrayLevelVariance	14.58545455
wavelet-HHH	gldm	HighGrayLevelEmphasis	149.1454545
wavelet-HHH	gldm	DependenceEntropy	5.332292928
wavelet-HHH	gldm	DependenceNonUniformity	26.65454545
wavelet-HHH	gldm	GrayLevelNonUniformity	9.672727273
wavelet-HHH	gldm	SmallDependenceEmphasis	0.450566842
wavelet-HHH	gldm	SmallDependenceHighGrayLevelEmphasis	77.35631463
wavelet-HHH	gldm	DependenceNonUniformityNormalized	0.24231405
wavelet-HHH	gldm	LargeDependenceEmphasis	8.163636364
wavelet-HHH	gldm	LargeDependenceLowGrayLevelEmphasis	0.079443313
wavelet-HHH	gldm	DependenceVariance	2.316033058
wavelet-HHH	gldm	LargeDependenceHighGrayLevelEmphasis	1119.018182
wavelet-HHH	gldm	SmallDependenceLowGrayLevelEmphasis	0.015835417
wavelet-HHH	gldm	LowGrayLevelEmphasis	0.021009
wavelet-HHH	glcm	JointAverage	11.54390132
wavelet-HHH	glcm	SumAverage	23.08780264
wavelet-HHH	glcm	JointEntropy	6.255708139
wavelet-HHH	glcm	ClusterShade	-1.080958065
wavelet-HHH	glcm	MaximumProbability	0.03894739
wavelet-HHH	glcm	Idmn	0.956163544
wavelet-HHH	glcm	JointEnergy	0.015661065
wavelet-HHH	glcm	Contrast	27.33314932
wavelet-HHH	glcm	DifferenceEntropy	3.295092338
wavelet-HHH	glcm	InverseVariance	0.252356527
wavelet-HHH	glcm	DifferenceVariance	11.38590047
wavelet-HHH	glcm	Idn	0.865772199
wavelet-HHH	glcm	Idm	0.236628799
wavelet-HHH	glcm	Correlation	-0.036323595
wavelet-HHH	glcm	Autocorrelation	132.6776952
wavelet-HHH	glcm	SumEntropy	3.998237732
wavelet-HHH	glcm	SumSquares	13.06985862
wavelet-HHH	glcm	ClusterProminence	2291.778979
wavelet-HHH	glcm	Imc2	0.958072185
wavelet-HHH	glcm	Imc1	-0.335676302
wavelet-HHH	glcm	DifferenceAverage	3.947021677
wavelet-HHH	glcm	Id	0.326418045
wavelet-HHH	glcm	ClusterTendency	24.94628517
wavelet-HHH	firstorder	InterquartileRange	103.5797128
wavelet-HHH	firstorder	Skewness	0.077791002
wavelet-HHH	firstorder	Uniformity	0.087933884
wavelet-HHH	firstorder	Median	0.023507155

wavelet-HHH	firstorder	Energy	1019416.594
wavelet-HHH	firstorder	RobustMeanAbsoluteDeviation	45.26666926
wavelet-HHH	firstorder	MeanAbsoluteDeviation	72.08507233
wavelet-HHH	firstorder	TotalEnergy	27524248.04
wavelet-HHH	firstorder	Maximum	297.1305783
wavelet-HHH	firstorder	RootMeanSquared	96.26745859
wavelet-HHH	firstorder	90Percentile	111.2527884
wavelet-HHH	firstorder	Minimum	-259.2300021
wavelet-HHH	firstorder	Entropy	3.839552118
wavelet-HHH	firstorder	Range	556.3605805
wavelet-HHH	firstorder	Variance	9258.85464
wavelet-HHH	firstorder	10Percentile	-115.374511
wavelet-HHH	firstorder	Kurtosis	3.809082077
wavelet-HHH	firstorder	Mean	2.927275655
wavelet-HHH	glrlm	ShortRunLowGrayLevelEmphasis	0.021366044
wavelet-HHH	glrlm	GrayLevelVariance	15.24524376
wavelet-HHH	glrlm	LowGrayLevelRunEmphasis	0.021729862
wavelet-HHH	glrlm	GrayLevelNonUniformityNormalized	0.085276212
wavelet-HHH	glrlm	RunVariance	0.058761084
wavelet-HHH	glrlm	GrayLevelNonUniformity	8.870916525
wavelet-HHH	glrlm	LongRunEmphasis	1.178479333
wavelet-HHH	glrlm	ShortRunHighGrayLevelEmphasis	144.46085
wavelet-HHH	glrlm	RunLengthNonUniformity	93.1520392
wavelet-HHH	glrlm	ShortRunEmphasis	0.957853937
wavelet-HHH	glrlm	LongRunHighGrayLevelEmphasis	173.9092859
wavelet-HHH	glrlm	RunPercentage	0.945454545
wavelet-HHH	glrlm	LongRunLowGrayLevelEmphasis	0.023254705
wavelet-HHH	glrlm	RunEntropy	4.101579903
wavelet-HHH	glrlm	HighGrayLevelRunEmphasis	150.0637776
wavelet-HHH	glrlm	RunLengthNonUniformityNormalized	0.89514485
wavelet-HHH	glszm	GrayLevelVariance	24.66709184
wavelet-HHH	glszm	ZoneVariance	4.891581633
wavelet-HHH	glszm	GrayLevelNonUniformityNormalized	0.066326531
wavelet-HHH	glszm	SizeZoneNonUniformityNormalized	0.510204082
wavelet-HHH	glszm	SizeZoneNonUniformity	28.57142857
wavelet-HHH	glszm	GrayLevelNonUniformity	3.714285714
wavelet-HHH	glszm	LargeAreaEmphasis	8.75
wavelet-HHH	glszm	SmallAreaHighGrayLevelEmphasis	133.1297199
wavelet-HHH	glszm	ZonePercentage	0.509090909
wavelet-HHH	glszm	LargeAreaLowGrayLevelEmphasis	0.095790245
wavelet-HHH	glszm	LargeAreaHighGrayLevelEmphasis	1211.785714
wavelet-HHH	glszm	HighGrayLevelZoneEmphasis	166.1071429
wavelet-HHH	glszm	SmallAreaEmphasis	0.740836636
wavelet-HHH	glszm	LowGrayLevelZoneEmphasis	0.032556676
wavelet-HHH	glszm	ZoneEntropy	4.840587681
wavelet-HHH	glszm	SmallAreaLowGrayLevelEmphasis	0.029691938
wavelet-HHH	ngtdm	Coarseness	0.058938716
wavelet-HHH	ngtdm	Complexity	479.82203
wavelet-HHH	ngtdm	Strength	6.852646364
wavelet-HHH	ngtdm	Contrast	0.210347951
wavelet-HHH	ngtdm	Busyness	0.06858538
wavelet-HLL	gldm	GrayLevelVariance	60.55247934
wavelet-HLL	gldm	HighGrayLevelEmphasis	257.8272727
wavelet-HLL	gldm	DependenceEntropy	5.660594145
wavelet-HLL	gldm	DependenceNonUniformity	28.29090909
wavelet-HLL	gldm	GrayLevelNonUniformity	6.018181818

wavelet-HLL	gldm	SmallDependenceEmphasis	0.46099732
wavelet-HLL	gldm	SmallDependenceHighGrayLevelEmphasis	155.5793962
wavelet-HLL	gldm	DependenceNonUniformityNormalized	0.257190083
wavelet-HLL	gldm	LargeDependenceEmphasis	7.472727273
wavelet-HLL	gldm	LargeDependenceLowGrayLevelEmphasis	0.154853185
wavelet-HLL	gldm	DependenceVariance	1.971570248
wavelet-HLL	gldm	LargeDependenceHighGrayLevelEmphasis	1250.409091
wavelet-HLL	gldm	SmallDependenceLowGrayLevelEmphasis	0.010497068
wavelet-HLL	gldm	LowGrayLevelEmphasis	0.030072692
wavelet-HLL	glcm	JointAverage	13.61456008
wavelet-HLL	glcm	SumAverage	27.22912016
wavelet-HLL	glcm	JointEntropy	6.686958822
wavelet-HLL	glcm	ClusterShade	1817.340078
wavelet-HLL	glcm	MaximumProbability	0.02721413
wavelet-HLL	glcm	Idmn	0.953106127
wavelet-HLL	glcm	JointEnergy	0.010828807
wavelet-HLL	glcm	Contrast	80.4589059
wavelet-HLL	glcm	DifferenceEntropy	3.792459761
wavelet-HLL	glcm	InverseVariance	0.175260521
wavelet-HLL	glcm	DifferenceVariance	38.17385858
wavelet-HLL	glcm	Idn	0.869150017
wavelet-HLL	glcm	Idm	0.187510012
wavelet-HLL	glcm	Correlation	0.209559764
wavelet-HLL	glcm	Autocorrelation	196.6226246
wavelet-HLL	glcm	SumEntropy	4.730775138
wavelet-HLL	glcm	SumSquares	51.48340032
wavelet-HLL	glcm	ClusterProminence	80184.9359
wavelet-HLL	glcm	Imc2	0.989882265
wavelet-HLL	glcm	Imc1	-0.453539842
wavelet-HLL	glcm	DifferenceAverage	6.336618493
wavelet-HLL	glcm	Id	0.274537597
wavelet-HLL	glcm	ClusterTendency	125.4746954
wavelet-HLL	firstorder	InterquartileRange	244.5204602
wavelet-HLL	firstorder	Skewness	1.155191866
wavelet-HLL	firstorder	Uniformity	0.054710744
wavelet-HLL	firstorder	Median	-77.29850124
wavelet-HLL	firstorder	Energy	4254072.377
wavelet-HLL	firstorder	RobustMeanAbsoluteDeviation	92.57931314
wavelet-HLL	firstorder	MeanAbsoluteDeviation	146.862023
wavelet-HLL	firstorder	TotalEnergy	114859954.2
wavelet-HLL	firstorder	Maximum	536.7129949
wavelet-HLL	firstorder	RootMeanSquared	196.6554989
wavelet-HLL	firstorder	90Percentile	204.5055218
wavelet-HLL	firstorder	Minimum	-361.4296498
wavelet-HLL	firstorder	Entropy	4.441263214
wavelet-HLL	firstorder	Range	898.1426448
wavelet-HLL	firstorder	Variance	37362.83226
wavelet-HLL	firstorder	10Percentile	-222.4682333
wavelet-HLL	firstorder	Kurtosis	4.193381229
wavelet-HLL	firstorder	Mean	-36.20156054
wavelet-HLL	glrlm	ShortRunLowGrayLevelEmphasis	0.029357105
wavelet-HLL	glrlm	GrayLevelVariance	61.88511583
wavelet-HLL	glrlm	LowGrayLevelRunEmphasis	0.0303711
wavelet-HLL	glrlm	GrayLevelNonUniformityNormalized	0.053719169
wavelet-HLL	glrlm	RunVariance	0.054572932
wavelet-HLL	glrlm	GrayLevelNonUniformity	5.603016026

wavelet-HLL	glrlm	LongRunEmphasis	1.169773331
wavelet-HLL	glrlm	ShortRunHighGrayLevelEmphasis	255.8491847
wavelet-HLL	glrlm	RunLengthNonUniformity	93.9588997
wavelet-HLL	glrlm	ShortRunEmphasis	0.959318937
wavelet-HLL	glrlm	LongRunHighGrayLevelEmphasis	291.0884646
wavelet-HLL	glrlm	RunPercentage	0.948251748
wavelet-HLL	glrlm	LongRunLowGrayLevelEmphasis	0.034534253
wavelet-HLL	glrlm	RunEntropy	4.644982388
wavelet-HLL	glrlm	HighGrayLevelRunEmphasis	262.7920094
wavelet-HLL	glrlm	RunLengthNonUniformityNormalized	0.899291229
wavelet-HLL	glszm	GrayLevelVariance	74.34333333
wavelet-HLL	glszm	ZoneVariance	2.505555556
wavelet-HLL	glszm	GrayLevelNonUniformityNormalized	0.047222222
wavelet-HLL	glszm	SizeZoneNonUniformityNormalized	0.49
wavelet-HLL	glszm	SizeZoneNonUniformity	29.4
wavelet-HLL	glszm	GrayLevelNonUniformity	2.833333333
wavelet-HLL	glszm	LargeAreaEmphasis	5.866666667
wavelet-HLL	glszm	SmallAreaHighGrayLevelEmphasis	259.3620854
wavelet-HLL	glszm	ZonePercentage	0.545454545
wavelet-HLL	glszm	LargeAreaLowGrayLevelEmphasis	0.130934054
wavelet-HLL	glszm	LargeAreaHighGrayLevelEmphasis	1094.883333
wavelet-HLL	glszm	HighGrayLevelZoneEmphasis	320.8333333
wavelet-HLL	glszm	SmallAreaEmphasis	0.723557613
wavelet-HLL	glszm	LowGrayLevelZoneEmphasis	0.029319204
wavelet-HLL	glszm	ZoneEntropy	5.202479554
wavelet-HLL	glszm	SmallAreaLowGrayLevelEmphasis	0.013781774
wavelet-HLL	ngtdm	Coarseness	0.045334553
wavelet-HLL	ngtdm	Complexity	1836.773752
wavelet-HLL	ngtdm	Strength	18.58327621
wavelet-HLL	ngtdm	Contrast	0.705478727
wavelet-HLL	ngtdm	Busyness	0.071255882
wavelet-LHL	gldm	GrayLevelVariance	116.271405
wavelet-LHL	gldm	HighGrayLevelEmphasis	656.2
wavelet-LHL	gldm	DependenceEntropy	5.587866872
wavelet-LHL	gldm	DependenceNonUniformity	51.65454545
wavelet-LHL	gldm	GrayLevelNonUniformity	4.363636364
wavelet-LHL	gldm	SmallDependenceEmphasis	0.69730303
wavelet-LHL	gldm	SmallDependenceHighGrayLevelEmphasis	487.6495051
wavelet-LHL	gldm	DependenceNonUniformityNormalized	0.469586777
wavelet-LHL	gldm	LargeDependenceEmphasis	3.290909091
wavelet-LHL	gldm	LargeDependenceLowGrayLevelEmphasis	0.021691129
wavelet-LHL	gldm	DependenceVariance	0.845950413
wavelet-LHL	gldm	LargeDependenceHighGrayLevelEmphasis	1916.018182
wavelet-LHL	gldm	SmallDependenceLowGrayLevelEmphasis	0.015788215
wavelet-LHL	gldm	LowGrayLevelEmphasis	0.01655653
wavelet-LHL	glcm	JointAverage	22.49659911
wavelet-LHL	glcm	SumAverage	44.99319821
wavelet-LHL	glcm	JointEntropy	6.854449591
wavelet-LHL	glcm	ClusterShade	5270.453309
wavelet-LHL	glcm	MaximumProbability	0.022121187
wavelet-LHL	glcm	Idmn	0.966558064
wavelet-LHL	glcm	JointEnergy	0.009350957
wavelet-LHL	glcm	Contrast	143.6824555
wavelet-LHL	glcm	DifferenceEntropy	4.14653526
wavelet-LHL	glcm	InverseVariance	0.132180369
wavelet-LHL	glcm	DifferenceVariance	60.61614017

wavelet-LHL	glcm	Idn	0.884008071
wavelet-LHL	glcm	Idm	0.115704611
wavelet-LHL	glcm	Correlation	0.292031969
wavelet-LHL	glcm	Autocorrelation	535.2962705
wavelet-LHL	glcm	SumEntropy	4.993945904
wavelet-LHL	glcm	SumSquares	100.9688349
wavelet-LHL	glcm	ClusterProminence	371190.1825
wavelet-LHL	glcm	Imc2	0.997850719
wavelet-LHL	glcm	Imc1	-0.571462012
wavelet-LHL	glcm	DifferenceAverage	8.817577105
wavelet-LHL	glcm	Id	0.202084036
wavelet-LHL	glcm	ClusterTendency	260.1928839
wavelet-LHL	firstorder	InterquartileRange	272.5663317
wavelet-LHL	firstorder	Skewness	0.94874831
wavelet-LHL	firstorder	Uniformity	0.039669421
wavelet-LHL	firstorder	Median	-79.31286197
wavelet-LHL	firstorder	Energy	8065697.426
wavelet-LHL	firstorder	RobustMeanAbsoluteDeviation	123.8001572
wavelet-LHL	firstorder	MeanAbsoluteDeviation	199.0023033
wavelet-LHL	firstorder	TotalEnergy	217773830.5
wavelet-LHL	firstorder	Maximum	905.9182736
wavelet-LHL	firstorder	RootMeanSquared	270.7850108
wavelet-LHL	firstorder	90Percentile	352.1680592
wavelet-LHL	firstorder	Minimum	-597.9567209
wavelet-LHL	firstorder	Entropy	4.932674828
wavelet-LHL	firstorder	Range	1503.874995
wavelet-LHL	firstorder	Variance	72341.65723
wavelet-LHL	firstorder	10Percentile	-302.1897972
wavelet-LHL	firstorder	Kurtosis	4.506583826
wavelet-LHL	firstorder	Mean	-31.35067511
wavelet-LHL	glrlm	ShortRunLowGrayLevelEmphasis	0.016835583
wavelet-LHL	glrlm	GrayLevelVariance	118.0107671
wavelet-LHL	glrlm	LowGrayLevelRunEmphasis	0.016873087
wavelet-LHL	glrlm	GrayLevelNonUniformityNormalized	0.038978628
wavelet-LHL	glrlm	RunVariance	0.024573555
wavelet-LHL	glrlm	GrayLevelNonUniformity	4.195344516
wavelet-LHL	glrlm	LongRunEmphasis	1.06982508
wavelet-LHL	glrlm	ShortRunHighGrayLevelEmphasis	649.6918451
wavelet-LHL	glrlm	RunLengthNonUniformity	103.2605939
wavelet-LHL	glrlm	ShortRunEmphasis	0.984164052
wavelet-LHL	glrlm	LongRunHighGrayLevelEmphasis	696.7342264
wavelet-LHL	glrlm	RunPercentage	0.978321678
wavelet-LHL	glrlm	LongRunLowGrayLevelEmphasis	0.017037801
wavelet-LHL	glrlm	RunEntropy	5.0168815
wavelet-LHL	glrlm	HighGrayLevelRunEmphasis	658.5286717
wavelet-LHL	glrlm	RunLengthNonUniformityNormalized	0.95931195
wavelet-LHL	glszm	GrayLevelVariance	136.8608997
wavelet-LHL	glszm	ZoneVariance	0.607612457
wavelet-LHL	glszm	GrayLevelNonUniformityNormalized	0.034740484
wavelet-LHL	glszm	SizeZoneNonUniformityNormalized	0.667543253
wavelet-LHL	glszm	SizeZoneNonUniformity	56.74117647
wavelet-LHL	glszm	GrayLevelNonUniformity	2.952941176
wavelet-LHL	glszm	LargeAreaEmphasis	2.282352941
wavelet-LHL	glszm	SmallAreaHighGrayLevelEmphasis	598.6336601
wavelet-LHL	glszm	ZonePercentage	0.772727273
wavelet-LHL	glszm	LargeAreaLowGrayLevelEmphasis	0.023716972

wavelet-LHL	glszm	LargeAreaHighGrayLevelEmphasis	1372.635294
wavelet-LHL	glszm	HighGrayLevelZoneEmphasis	687.7294118
wavelet-LHL	glszm	SmallAreaEmphasis	0.843281046
wavelet-LHL	glszm	LowGrayLevelZoneEmphasis	0.020692964
wavelet-LHL	glszm	ZoneEntropy	5.429576671
wavelet-LHL	glszm	SmallAreaLowGrayLevelEmphasis	0.020275534
wavelet-LHL	ngtdm	Coarseness	0.050353845
wavelet-LHL	ngtdm	Complexity	4356.09057
wavelet-LHL	ngtdm	Strength	39.51487587
wavelet-LHL	ngtdm	Contrast	0.965305054
wavelet-LHL	ngtdm	Busyness	0.028636938
wavelet-LHH	gldm	GrayLevelVariance	32.76561983
wavelet-LHH	gldm	HighGrayLevelEmphasis	419.7818182
wavelet-LHH	gldm	DependenceEntropy	5.534464118
wavelet-LHH	gldm	DependenceNonUniformity	31.03636364
wavelet-LHH	gldm	GrayLevelNonUniformity	7.145454545
wavelet-LHH	gldm	SmallDependenceEmphasis	0.510834364
wavelet-LHH	gldm	SmallDependenceHighGrayLevelEmphasis	231.1868701
wavelet-LHH	gldm	DependenceNonUniformityNormalized	0.28214876
wavelet-LHH	gldm	LargeDependenceEmphasis	6.981818182
wavelet-LHH	gldm	LargeDependenceLowGrayLevelEmphasis	0.032449999
wavelet-LHH	gldm	DependenceVariance	2.141818182
wavelet-LHH	gldm	LargeDependenceHighGrayLevelEmphasis	2566.890909
wavelet-LHH	gldm	SmallDependenceLowGrayLevelEmphasis	0.013067519
wavelet-LHH	gldm	LowGrayLevelEmphasis	0.014567316
wavelet-LHH	glcm	JointAverage	19.68993802
wavelet-LHH	glcm	SumAverage	39.37987603
wavelet-LHH	glcm	JointEntropy	6.564892261
wavelet-LHH	glcm	ClusterShade	-51.57098346
wavelet-LHH	glcm	MaximumProbability	0.036612727
wavelet-LHH	glcm	Idmn	0.952841964
wavelet-LHH	glcm	JointEnergy	0.012358015
wavelet-LHH	glcm	Contrast	57.90709858
wavelet-LHH	glcm	DifferenceEntropy	3.728783858
wavelet-LHH	glcm	InverseVariance	0.192728457
wavelet-LHH	glcm	DifferenceVariance	24.45861183
wavelet-LHH	glcm	Idn	0.861812875
wavelet-LHH	glcm	Idm	0.188078723
wavelet-LHH	glcm	Correlation	-0.028698671
wavelet-LHH	glcm	Autocorrelation	386.8260329
wavelet-LHH	glcm	SumEntropy	4.420717488
wavelet-LHH	glcm	SumSquares	28.07436077
wavelet-LHH	glcm	ClusterProminence	12425.59349
wavelet-LHH	glcm	Imc2	0.981501777
wavelet-LHH	glcm	Imc1	-0.404330412
wavelet-LHH	glcm	DifferenceAverage	5.712502894
wavelet-LHH	glcm	Id	0.274784747
wavelet-LHH	glcm	ClusterTendency	54.39034449
wavelet-LHH	firstorder	InterquartileRange	152.4870077
wavelet-LHH	firstorder	Skewness	-0.300615281
wavelet-LHH	firstorder	Uniformity	0.064958678
wavelet-LHH	firstorder	Median	-4.418919448
wavelet-LHH	firstorder	Energy	2238356.326
wavelet-LHH	firstorder	RobustMeanAbsoluteDeviation	63.74632433
wavelet-LHH	firstorder	MeanAbsoluteDeviation	103.9374529
wavelet-LHH	firstorder	TotalEnergy	60435620.81

wavelet-LHH	firstorder	Maximum	311.5921287
wavelet-LHH	firstorder	RootMeanSquared	142.6488481
wavelet-LHH	firstorder	90Percentile	190.0157717
wavelet-LHH	firstorder	Minimum	-466.9636306
wavelet-LHH	firstorder	Entropy	4.257682055
wavelet-LHH	firstorder	Range	778.5557593
wavelet-LHH	firstorder	Variance	20331.7572
wavelet-LHH	firstorder	10Percentile	-143.789586
wavelet-LHH	firstorder	Kurtosis	4.060100206
wavelet-LHH	firstorder	Mean	4.115419259
wavelet-LHH	glrlm	ShortRunLowGrayLevelEmphasis	0.015029448
wavelet-LHH	glrlm	GrayLevelVariance	33.88754497
wavelet-LHH	glrlm	LowGrayLevelRunEmphasis	0.015131795
wavelet-LHH	glrlm	GrayLevelNonUniformityNormalized	0.062537932
wavelet-LHH	glrlm	RunVariance	0.053459379
wavelet-LHH	glrlm	GrayLevelNonUniformity	6.563170466
wavelet-LHH	glrlm	LongRunEmphasis	1.154005098
wavelet-LHH	glrlm	ShortRunHighGrayLevelEmphasis	409.450192
wavelet-LHH	glrlm	RunLengthNonUniformity	95.9981516
wavelet-LHH	glrlm	ShortRunEmphasis	0.965657763
wavelet-LHH	glrlm	LongRunHighGrayLevelEmphasis	478.6053823
wavelet-LHH	glrlm	RunPercentage	0.953846154
wavelet-LHH	glrlm	LongRunLowGrayLevelEmphasis	0.015598567
wavelet-LHH	glrlm	RunEntropy	4.464235191
wavelet-LHH	glrlm	HighGrayLevelRunEmphasis	422.2655954
wavelet-LHH	glrlm	RunLengthNonUniformityNormalized	0.914253987
wavelet-LHH	glszm	GrayLevelVariance	47.77676997
wavelet-LHH	glszm	ZoneVariance	3.586293777
wavelet-LHH	glszm	GrayLevelNonUniformityNormalized	0.049634669
wavelet-LHH	glszm	SizeZoneNonUniformityNormalized	0.560594608
wavelet-LHH	glszm	SizeZoneNonUniformity	35.31746032
wavelet-LHH	glszm	GrayLevelNonUniformity	3.126984127
wavelet-LHH	glszm	LargeAreaEmphasis	6.634920635
wavelet-LHH	glszm	SmallAreaHighGrayLevelEmphasis	357.3154938
wavelet-LHH	glszm	ZonePercentage	0.572727273
wavelet-LHH	glszm	LargeAreaLowGrayLevelEmphasis	0.039632585
wavelet-LHH	glszm	LargeAreaHighGrayLevelEmphasis	2516.539683
wavelet-LHH	glszm	HighGrayLevelZoneEmphasis	449.047619
wavelet-LHH	glszm	SmallAreaEmphasis	0.774549027
wavelet-LHH	glszm	LowGrayLevelZoneEmphasis	0.023184075
wavelet-LHH	glszm	ZoneEntropy	5.135699765
wavelet-LHH	glszm	SmallAreaLowGrayLevelEmphasis	0.022451193
wavelet-LHH	ngtdm	Coarseness	0.050514626
wavelet-LHH	ngtdm	Complexity	1230.39406
wavelet-LHH	ngtdm	Strength	11.50721556
wavelet-LHH	ngtdm	Contrast	0.452730814
wavelet-LHH	ngtdm	Busyness	0.04195413
wavelet-LLH	gldm	GrayLevelVariance	60.56198347
wavelet-LLH	gldm	HighGrayLevelEmphasis	249
wavelet-LLH	gldm	DependenceEntropy	5.494551759
wavelet-LLH	gldm	DependenceNonUniformity	39.4
wavelet-LLH	gldm	GrayLevelNonUniformity	5.418181818
wavelet-LLH	gldm	SmallDependenceEmphasis	0.53655303
wavelet-LLH	gldm	SmallDependenceHighGrayLevelEmphasis	160.5825758
wavelet-LLH	gldm	DependenceNonUniformityNormalized	0.358181818
wavelet-LLH	gldm	LargeDependenceEmphasis	4.054545455

wavelet-LLH	gldm	LargeDependenceLowGrayLevelEmphasis	0.090384709
wavelet-LLH	gldm	DependenceVariance	0.748760331
wavelet-LLH	gldm	LargeDependenceHighGrayLevelEmphasis	772.7454545
wavelet-LLH	gldm	SmallDependenceLowGrayLevelEmphasis	0.037534027
wavelet-LLH	gldm	LowGrayLevelEmphasis	0.045868464
wavelet-LLH	glcm	JointAverage	13.47949087
wavelet-LLH	glcm	SumAverage	26.95898173
wavelet-LLH	glcm	JointEntropy	6.809877151
wavelet-LLH	glcm	ClusterShade	1209.2845
wavelet-LLH	glcm	MaximumProbability	0.023566804
wavelet-LLH	glcm	Idmn	0.955551927
wavelet-LLH	glcm	JointEnergy	0.009654293
wavelet-LLH	glcm	Contrast	85.13715822
wavelet-LLH	glcm	DifferenceEntropy	3.932138542
wavelet-LLH	glcm	InverseVariance	0.156625851
wavelet-LLH	glcm	DifferenceVariance	35.75342627
wavelet-LLH	glcm	Idn	0.864573598
wavelet-LLH	glcm	Idm	0.145282181
wavelet-LLH	glcm	Correlation	0.152082367
wavelet-LLH	glcm	Autocorrelation	189.7240324
wavelet-LLH	glcm	SumEntropy	4.792318498
wavelet-LLH	glcm	SumSquares	50.52169609
wavelet-LLH	glcm	ClusterProminence	64565.4398
wavelet-LLH	glcm	Imc2	0.992774025
wavelet-LLH	glcm	Imc1	-0.476288787
wavelet-LLH	glcm	DifferenceAverage	6.940370384
wavelet-LLH	glcm	Id	0.234017396
wavelet-LLH	glcm	ClusterTendency	116.9496261
wavelet-LLH	firstorder	InterquartileRange	218.1778009
wavelet-LLH	firstorder	Skewness	0.928901975
wavelet-LLH	firstorder	Uniformity	0.049256198
wavelet-LLH	firstorder	Median	-112.110237
wavelet-LLH	firstorder	Energy	4690710.721
wavelet-LLH	firstorder	RobustMeanAbsoluteDeviation	95.60447501
wavelet-LLH	firstorder	MeanAbsoluteDeviation	148.3220158
wavelet-LLH	firstorder	TotalEnergy	126649189.5
wavelet-LLH	firstorder	Maximum	595.221916
wavelet-LLH	firstorder	RootMeanSquared	206.5013916
wavelet-LLH	firstorder	90Percentile	155.3113638
wavelet-LLH	firstorder	Minimum	-386.2400596
wavelet-LLH	firstorder	Entropy	4.574955336
wavelet-LLH	firstorder	Range	981.4619756
wavelet-LLH	firstorder	Variance	37676.1217
wavelet-LLH	firstorder	10Percentile	-298.5852312
wavelet-LLH	firstorder	Kurtosis	4.104505521
wavelet-LLH	firstorder	Mean	-70.47483977
wavelet-LLH	glrlm	ShortRunLowGrayLevelEmphasis	0.046457264
wavelet-LLH	glrlm	GrayLevelVariance	61.60678923
wavelet-LLH	glrlm	LowGrayLevelRunEmphasis	0.046849814
wavelet-LLH	glrlm	GrayLevelNonUniformityNormalized	0.048556069
wavelet-LLH	glrlm	RunVariance	0.032895794
wavelet-LLH	glrlm	GrayLevelNonUniformity	5.173632037
wavelet-LLH	glrlm	LongRunEmphasis	1.100204683
wavelet-LLH	glrlm	ShortRunHighGrayLevelEmphasis	246.9099051
wavelet-LLH	glrlm	RunLengthNonUniformity	100.0522147
wavelet-LLH	glrlm	ShortRunEmphasis	0.975786771

wavelet-LLH	glrlm	LongRunHighGrayLevelEmphasis	269.2355776
wavelet-LLH	glrlm	RunPercentage	0.968531469
wavelet-LLH	glrlm	LongRunLowGrayLevelEmphasis	0.048443289
wavelet-LLH	glrlm	RunEntropy	4.697864146
wavelet-LLH	glrlm	HighGrayLevelRunEmphasis	251.2785087
wavelet-LLH	glrlm	RunLengthNonUniformityNormalized	0.938422768
wavelet-LLH	glszm	GrayLevelVariance	78.36588952
wavelet-LLH	glszm	ZoneVariance	1.400546104
wavelet-LLH	glszm	GrayLevelNonUniformityNormalized	0.041797942
wavelet-LLH	glszm	SizeZoneNonUniformityNormalized	0.496324302
wavelet-LLH	glszm	SizeZoneNonUniformity	34.24637681
wavelet-LLH	glszm	GrayLevelNonUniformity	2.884057971
wavelet-LLH	glszm	LargeAreaEmphasis	3.942028986
wavelet-LLH	glszm	SmallAreaHighGrayLevelEmphasis	232.2450765
wavelet-LLH	glszm	ZonePercentage	0.627272727
wavelet-LLH	glszm	LargeAreaLowGrayLevelEmphasis	0.103895355
wavelet-LLH	glszm	LargeAreaHighGrayLevelEmphasis	761.3913043
wavelet-LLH	glszm	HighGrayLevelZoneEmphasis	288.4057971
wavelet-LLH	glszm	SmallAreaEmphasis	0.729747383
wavelet-LLH	glszm	LowGrayLevelZoneEmphasis	0.06332453
wavelet-LLH	glszm	ZoneEntropy	5.329184783
wavelet-LLH	glszm	SmallAreaLowGrayLevelEmphasis	0.057592258
wavelet-LLH	ngtdm	Coarseness	0.046716709
wavelet-LLH	ngtdm	Complexity	2085.798828
wavelet-LLH	ngtdm	Strength	19.01857476
wavelet-LLH	ngtdm	Contrast	0.727487201
wavelet-LLH	ngtdm	Busyness	0.071136493
wavelet-LLL	gldm	GrayLevelVariance	374.6595868
wavelet-LLL	gldm	HighGrayLevelEmphasis	3895.463636
wavelet-LLL	gldm	DependenceEntropy	5.883232554
wavelet-LLL	gldm	DependenceNonUniformity	52.83636364
wavelet-LLL	gldm	GrayLevelNonUniformity	2.781818182
wavelet-LLL	gldm	SmallDependenceEmphasis	0.718704545
wavelet-LLL	gldm	SmallDependenceHighGrayLevelEmphasis	2544.463932
wavelet-LLL	gldm	DependenceNonUniformityNormalized	0.480330579
wavelet-LLL	gldm	LargeDependenceEmphasis	3.345454545
wavelet-LLL	gldm	LargeDependenceLowGrayLevelEmphasis	0.01044493
wavelet-LLL	gldm	DependenceVariance	0.900495868
wavelet-LLL	gldm	LargeDependenceHighGrayLevelEmphasis	17341.35455
wavelet-LLL	gldm	SmallDependenceLowGrayLevelEmphasis	0.009852751
wavelet-LLL	gldm	LowGrayLevelEmphasis	0.009932723
wavelet-LLL	glcm	JointAverage	64.1923217
wavelet-LLL	glcm	SumAverage	128.3846434
wavelet-LLL	glcm	JointEntropy	6.980552225
wavelet-LLL	glcm	ClusterShade	-20107.0889
wavelet-LLL	glcm	MaximumProbability	0.016062779
wavelet-LLL	glcm	Idmn	0.94805303
wavelet-LLL	glcm	JointEnergy	0.008265977
wavelet-LLL	glcm	Contrast	444.917756
wavelet-LLL	glcm	DifferenceEntropy	4.810766186
wavelet-LLL	glcm	InverseVariance	0.089760756
wavelet-LLL	glcm	DifferenceVariance	177.3661114
wavelet-LLL	glcm	Idn	0.853435416
wavelet-LLL	glcm	Idm	0.088111068
wavelet-LLL	glcm	Correlation	0.320813379
wavelet-LLL	glcm	Autocorrelation	4225.471364

wavelet-LLL	glcm	SumEntropy	5.499003821
wavelet-LLL	glcm	SumSquares	326.8521403
wavelet-LLL	glcm	ClusterProminence	2442246.446
wavelet-LLL	glcm	Imc2	0.999620406
wavelet-LLL	glcm	Imc1	-0.680898128
wavelet-LLL	glcm	DifferenceAverage	16.21104137
wavelet-LLL	glcm	Id	0.151396715
wavelet-LLL	glcm	ClusterTendency	862.4908053
wavelet-LLL	firstorder	InterquartileRange	756.8999137
wavelet-LLL	firstorder	Skewness	-0.698986892
wavelet-LLL	firstorder	Uniformity	0.025289256
wavelet-LLL	firstorder	Median	-416.5325411
wavelet-LLL	firstorder	Energy	51102202.2
wavelet-LLL	firstorder	RobustMeanAbsoluteDeviation	287.3746433
wavelet-LLL	firstorder	MeanAbsoluteDeviation	391.5737549
wavelet-LLL	firstorder	TotalEnergy	1379759459
wavelet-LLL	firstorder	Maximum	170.4098527
wavelet-LLL	firstorder	RootMeanSquared	681.5904009
wavelet-LLL	firstorder	90Percentile	111.2191727
wavelet-LLL	firstorder	Minimum	-1935.407199
wavelet-LLL	firstorder	Entropy	5.472148182
wavelet-LLL	firstorder	Range	2105.817052
wavelet-LLL	firstorder	Variance	234801.6674
wavelet-LLL	firstorder	10Percentile	-1176.774897
wavelet-LLL	firstorder	Kurtosis	3.039670673
wavelet-LLL	firstorder	Mean	-479.336841
wavelet-LLL	glrlm	ShortRunLowGrayLevelEmphasis	0.010146095
wavelet-LLL	glrlm	GrayLevelVariance	373.9825271
wavelet-LLL	glrlm	LowGrayLevelRunEmphasis	0.010150186
wavelet-LLL	glrlm	GrayLevelNonUniformityNormalized	0.024941518
wavelet-LLL	glrlm	RunVariance	0.021643189
wavelet-LLL	glrlm	GrayLevelNonUniformity	2.684425912
wavelet-LLL	glrlm	LongRunEmphasis	1.06732819
wavelet-LLL	glrlm	ShortRunHighGrayLevelEmphasis	3772.0291
wavelet-LLL	glrlm	RunLengthNonUniformity	103.0143237
wavelet-LLL	glrlm	ShortRunEmphasis	0.983167953
wavelet-LLL	glrlm	LongRunHighGrayLevelEmphasis	4223.870618
wavelet-LLL	glrlm	RunPercentage	0.978321678
wavelet-LLL	glrlm	LongRunLowGrayLevelEmphasis	0.010166549
wavelet-LLL	glrlm	RunEntropy	5.539575882
wavelet-LLL	glrlm	HighGrayLevelRunEmphasis	3862.397403
wavelet-LLL	glrlm	RunLengthNonUniformityNormalized	0.956713621
wavelet-LLL	glszm	GrayLevelVariance	370.2932666
wavelet-LLL	glszm	ZoneVariance	0.550027042
wavelet-LLL	glszm	GrayLevelNonUniformityNormalized	0.023796647
wavelet-LLL	glszm	SizeZoneNonUniformityNormalized	0.711465657
wavelet-LLL	glszm	SizeZoneNonUniformity	61.18604651
wavelet-LLL	glszm	GrayLevelNonUniformity	2.046511628
wavelet-LLL	glszm	LargeAreaEmphasis	2.186046512
wavelet-LLL	glszm	SmallAreaHighGrayLevelEmphasis	3011.624399
wavelet-LLL	glszm	ZonePercentage	0.781818182
wavelet-LLL	glszm	LargeAreaLowGrayLevelEmphasis	0.012908727
wavelet-LLL	glszm	LargeAreaHighGrayLevelEmphasis	10079.31395
wavelet-LLL	glszm	HighGrayLevelZoneEmphasis	3580.965116
wavelet-LLL	glszm	SmallAreaEmphasis	0.86625969
wavelet-LLL	glszm	LowGrayLevelZoneEmphasis	0.012630047

wavelet-LLL	glszm	ZoneEntropy	5.713657632
wavelet-LLL	glszm	SmallAreaLowGrayLevelEmphasis	0.012586471
wavelet-LLL	ngtdm	Coarseness	0.036807343
wavelet-LLL	ngtdm	Complexity	15921.42252
wavelet-LLL	ngtdm	Strength	69.2519305
wavelet-LLL	ngtdm	Contrast	3.301016623
wavelet-LLL	ngtdm	Busyness	0.01096404
wavelet-HHL	gldm	GrayLevelVariance	14.07966942
wavelet-HHL	gldm	HighGrayLevelEmphasis	102.7818182
wavelet-HHL	gldm	DependenceEntropy	5.326313237
wavelet-HHL	gldm	DependenceNonUniformity	23.09090909
wavelet-HHL	gldm	GrayLevelNonUniformity	10.16363636
wavelet-HHL	gldm	SmallDependenceEmphasis	0.35142843
wavelet-HHL	gldm	SmallDependenceHighGrayLevelEmphasis	44.71367013
wavelet-HHL	gldm	DependenceNonUniformityNormalized	0.209917355
wavelet-HHL	gldm	LargeDependenceEmphasis	9.909090909
wavelet-HHL	gldm	LargeDependenceLowGrayLevelEmphasis	0.155508986
wavelet-HHL	gldm	DependenceVariance	2.371570248
wavelet-HHL	gldm	LargeDependenceHighGrayLevelEmphasis	838.5818182
wavelet-HHL	gldm	SmallDependenceLowGrayLevelEmphasis	0.02017564
wavelet-HHL	gldm	LowGrayLevelEmphasis	0.029955886
wavelet-HHL	glcm	JointAverage	9.423213761
wavelet-HHL	glcm	SumAverage	18.84642752
wavelet-HHL	glcm	JointEntropy	6.200760381
wavelet-HHL	glcm	ClusterShade	79.13514691
wavelet-HHL	glcm	MaximumProbability	0.044968965
wavelet-HHL	glcm	Idmn	0.965628406
wavelet-HHL	glcm	JointEnergy	0.016675158
wavelet-HHL	glcm	Contrast	24.83406814
wavelet-HHL	glcm	DifferenceEntropy	3.250891043
wavelet-HHL	glcm	InverseVariance	0.263995468
wavelet-HHL	glcm	DifferenceVariance	10.9180784
wavelet-HHL	glcm	Idn	0.881558461
wavelet-HHL	glcm	Idm	0.261208156
wavelet-HHL	glcm	Correlation	0.003717178
wavelet-HHL	glcm	Autocorrelation	88.87363657
wavelet-HHL	glcm	SumEntropy	4.007868773
wavelet-HHL	glcm	SumSquares	12.47798534
wavelet-HHL	glcm	ClusterProminence	2723.414798
wavelet-HHL	glcm	Imc2	0.950387422
wavelet-HHL	glcm	Imc1	-0.320088843
wavelet-HHL	glcm	DifferenceAverage	3.696912252
wavelet-HHL	glcm	Id	0.347664774
wavelet-HHL	glcm	ClusterTendency	25.07787323
wavelet-HHL	firstorder	InterquartileRange	91.0329612
wavelet-HHL	firstorder	Skewness	0.891036832
wavelet-HHL	firstorder	Uniformity	0.092396694
wavelet-HHL	firstorder	Median	-9.05181226
wavelet-HHL	firstorder	Energy	931133.6915
wavelet-HHL	firstorder	RobustMeanAbsoluteDeviation	40.72746213
wavelet-HHL	firstorder	MeanAbsoluteDeviation	66.58572628
wavelet-HHL	firstorder	TotalEnergy	25140609.67
wavelet-HHL	firstorder	Maximum	378.143802
wavelet-HHL	firstorder	RootMeanSquared	92.00462891
wavelet-HHL	firstorder	90Percentile	113.1699822
wavelet-HHL	firstorder	Minimum	-200.5537255

wavelet-HHL	firstorder	Entropy	3.771912507
wavelet-HHL	firstorder	Range	578.6975275
wavelet-HHL	firstorder	Variance	8460.033616
wavelet-HHL	firstorder	10Percentile	-105.1494842
wavelet-HHL	firstorder	Kurtosis	5.294152003
wavelet-HHL	firstorder	Mean	-2.195022728
wavelet-HHL	glrlm	ShortRunLowGrayLevelEmphasis	0.030371369
wavelet-HHL	glrlm	GrayLevelVariance	14.81713709
wavelet-HHL	glrlm	LowGrayLevelRunEmphasis	0.031087597
wavelet-HHL	glrlm	GrayLevelNonUniformityNormalized	0.088888513
wavelet-HHL	glrlm	RunVariance	0.07872842
wavelet-HHL	glrlm	GrayLevelNonUniformity	9.120280864
wavelet-HHL	glrlm	LongRunEmphasis	1.229771959
wavelet-HHL	glrlm	ShortRunHighGrayLevelEmphasis	99.65455693
wavelet-HHL	glrlm	RunLengthNonUniformity	90.03511212
wavelet-HHL	glrlm	ShortRunEmphasis	0.949264084
wavelet-HHL	glrlm	LongRunHighGrayLevelEmphasis	123.5478462
wavelet-HHL	glrlm	RunPercentage	0.932867133
wavelet-HHL	glrlm	LongRunLowGrayLevelEmphasis	0.03442765
wavelet-HHL	glrlm	RunEntropy	4.086029101
wavelet-HHL	glrlm	HighGrayLevelRunEmphasis	104.0347915
wavelet-HHL	glrlm	RunLengthNonUniformityNormalized	0.876416173
wavelet-HHL	glszm	GrayLevelVariance	28.3634397
wavelet-HHL	glszm	ZoneVariance	11.73499189
wavelet-HHL	glszm	GrayLevelNonUniformityNormalized	0.061114116
wavelet-HHL	glszm	SizeZoneNonUniformityNormalized	0.454840454
wavelet-HHL	glszm	SizeZoneNonUniformity	19.55813953
wavelet-HHL	glszm	GrayLevelNonUniformity	2.627906977
wavelet-HHL	glszm	LargeAreaEmphasis	18.27906977
wavelet-HHL	glszm	SmallAreaHighGrayLevelEmphasis	96.09792313
wavelet-HHL	glszm	ZonePercentage	0.390909091
wavelet-HHL	glszm	LargeAreaLowGrayLevelEmphasis	0.28430927
wavelet-HHL	glszm	LargeAreaHighGrayLevelEmphasis	1602.418605
wavelet-HHL	glszm	HighGrayLevelZoneEmphasis	126.5116279
wavelet-HHL	glszm	SmallAreaEmphasis	0.69358083
wavelet-HHL	glszm	LowGrayLevelZoneEmphasis	0.053572042
wavelet-HHL	glszm	ZoneEntropy	4.804058069
wavelet-HHL	glszm	SmallAreaLowGrayLevelEmphasis	0.048215233
wavelet-HHL	ngtdm	Coarseness	0.058760382
wavelet-HHL	ngtdm	Complexity	434.7650979
wavelet-HHL	ngtdm	Strength	7.377067832
wavelet-HHL	ngtdm	Contrast	0.2087824
wavelet-HHL	ngtdm	Busyness	0.094565045
wavelet-HLH	gldm	GrayLevelVariance	31.32595041
wavelet-HLH	gldm	HighGrayLevelEmphasis	421.9272727
wavelet-HLH	gldm	DependenceEntropy	5.485283122
wavelet-HLH	gldm	DependenceNonUniformity	36.87272727
wavelet-HLH	gldm	GrayLevelNonUniformity	6.8
wavelet-HLH	gldm	SmallDependenceEmphasis	0.571386364
wavelet-HLH	gldm	SmallDependenceHighGrayLevelEmphasis	240.7054066
wavelet-HLH	gldm	DependenceNonUniformityNormalized	0.335206612
wavelet-HLH	gldm	LargeDependenceEmphasis	4.545454545
wavelet-HLH	gldm	LargeDependenceLowGrayLevelEmphasis	0.023496049
wavelet-HLH	gldm	DependenceVariance	1.038347107
wavelet-HLH	gldm	LargeDependenceHighGrayLevelEmphasis	1770.054545
wavelet-HLH	gldm	SmallDependenceLowGrayLevelEmphasis	0.012132441

wavelet-HLH	gldm	LowGrayLevelEmphasis	0.013274647
wavelet-HLH	glcm	JointAverage	19.69465383
wavelet-HLH	glcm	SumAverage	39.38930766
wavelet-HLH	glcm	JointEntropy	6.595345915
wavelet-HLH	glcm	ClusterShade	-135.3300267
wavelet-HLH	glcm	MaximumProbability	0.026214296
wavelet-HLH	glcm	Idmn	0.955553344
wavelet-HLH	glcm	JointEnergy	0.01153308
wavelet-HLH	glcm	Contrast	67.80836499
wavelet-HLH	glcm	DifferenceEntropy	3.70343143
wavelet-HLH	glcm	InverseVariance	0.199710945
wavelet-HLH	glcm	DifferenceVariance	30.76730083
wavelet-HLH	glcm	Idn	0.869170954
wavelet-HLH	glcm	Idm	0.173018639
wavelet-HLH	glcm	Correlation	-0.012611572
wavelet-HLH	glcm	Autocorrelation	387.4793945
wavelet-HLH	glcm	SumEntropy	4.403164177
wavelet-HLH	glcm	SumSquares	33.41841719
wavelet-HLH	glcm	ClusterProminence	22463.46896
wavelet-HLH	glcm	Imc2	0.988274926
wavelet-HLH	glcm	Imc1	-0.446716279
wavelet-HLH	glcm	DifferenceAverage	5.923095126
wavelet-HLH	glcm	Id	0.262922673
wavelet-HLH	glcm	ClusterTendency	65.86530378
wavelet-HLH	firstorder	InterquartileRange	160.9341865
wavelet-HLH	firstorder	Skewness	-0.292581163
wavelet-HLH	firstorder	Uniformity	0.061818182
wavelet-HLH	firstorder	Median	0.675727493
wavelet-HLH	firstorder	Energy	2151836.371
wavelet-HLH	firstorder	RobustMeanAbsoluteDeviation	66.74099756
wavelet-HLH	firstorder	MeanAbsoluteDeviation	104.3116951
wavelet-HLH	firstorder	TotalEnergy	58099582.02
wavelet-HLH	firstorder	Maximum	385.6975544
wavelet-HLH	firstorder	RootMeanSquared	139.8647519
wavelet-HLH	firstorder	90Percentile	155.2837132
wavelet-HLH	firstorder	Minimum	-469.2969141
wavelet-HLH	firstorder	Entropy	4.259249052
wavelet-HLH	firstorder	Range	854.9944685
wavelet-HLH	firstorder	Variance	19522.8546
wavelet-HLH	firstorder	10Percentile	-127.84268
wavelet-HLH	firstorder	Kurtosis	4.469626538
wavelet-HLH	firstorder	Mean	6.268510666
wavelet-HLH	glrlm	ShortRunLowGrayLevelEmphasis	0.013575863
wavelet-HLH	glrlm	GrayLevelVariance	32.03001274
wavelet-HLH	glrlm	LowGrayLevelRunEmphasis	0.013645149
wavelet-HLH	glrlm	GrayLevelNonUniformityNormalized	0.061277408
wavelet-HLH	glrlm	RunVariance	0.039451453
wavelet-HLH	glrlm	GrayLevelNonUniformity	6.514887833
wavelet-HLH	glrlm	LongRunEmphasis	1.112148156
wavelet-HLH	glrlm	ShortRunHighGrayLevelEmphasis	413.060785
wavelet-HLH	glrlm	RunLengthNonUniformity	99.82948457
wavelet-HLH	glrlm	ShortRunEmphasis	0.975298619
wavelet-HLH	glrlm	LongRunHighGrayLevelEmphasis	467.0824968
wavelet-HLH	glrlm	RunPercentage	0.966433566
wavelet-HLH	glrlm	LongRunLowGrayLevelEmphasis	0.013959341
wavelet-HLH	glrlm	RunEntropy	4.386111082

wavelet-HLH	glrlm	HighGrayLevelRunEmphasis	422.8660928
wavelet-HLH	glrlm	RunLengthNonUniformityNormalized	0.938051623
wavelet-HLH	glszm	GrayLevelVariance	41.71196191
wavelet-HLH	glszm	ZoneVariance	1.402499504
wavelet-HLH	glszm	GrayLevelNonUniformityNormalized	0.052965681
wavelet-HLH	glszm	SizeZoneNonUniformityNormalized	0.577464789
wavelet-HLH	glszm	SizeZoneNonUniformity	41
wavelet-HLH	glszm	GrayLevelNonUniformity	3.76056338
wavelet-HLH	glszm	LargeAreaEmphasis	3.802816901
wavelet-HLH	glszm	SmallAreaHighGrayLevelEmphasis	329.8418662
wavelet-HLH	glszm	ZonePercentage	0.645454545
wavelet-HLH	glszm	LargeAreaLowGrayLevelEmphasis	0.027181201
wavelet-HLH	glszm	LargeAreaHighGrayLevelEmphasis	1489.464789
wavelet-HLH	glszm	HighGrayLevelZoneEmphasis	428.8591549
wavelet-HLH	glszm	SmallAreaEmphasis	0.786533646
wavelet-HLH	glszm	LowGrayLevelZoneEmphasis	0.019068305
wavelet-HLH	glszm	ZoneEntropy	5.106942894
wavelet-HLH	glszm	SmallAreaLowGrayLevelEmphasis	0.018541186
wavelet-HLH	ngtdm	Coarseness	0.046703869
wavelet-HLH	ngtdm	Complexity	1260.352561
wavelet-HLH	ngtdm	Strength	12.02039362
wavelet-HLH	ngtdm	Contrast	0.429577911
wavelet-HLH	ngtdm	Busyness	0.04607687

Image type	Feature Class	Feature Name	3: RTSTRUCT: C99-DNU_segment_GTV_T9
general	info	BoundingBox	(0, 1, 1, 11, 7, 11)
general	info	EnabledImageTypes	{u'Original': {}, u'Wavelet': {}}
general	info	GeneralSettings	{'distances': [1], 'additionalInfo': True, 'force2D': False, 'interpolator': 'sitkBSpline', 'resampledPixelSpacing': [3.0], 'label': 1, 'normalizeScale': 1, 'normalize': False, 'force2Ddimension': 0, 'removeOutliers': None, 'minimumROISize': None, u'binWidth': 25.0, 'minimumROIDimensions': 1, u'correctMask': True, 'preCrop': False, u'symmetricalGLCM': True, 'resegmentRange': None, 'padDistance': 5}
general	info	ImageHash	f704d6044e19d5f7eda935496a80564c2d20cf2b
general	info	ImageSpacing	(0.9765624999999998, 0.9765624999999998, 1.999999999999999)
general	info	MaskHash	5d6ceebf00269009033aaa5a787ff207d67f1e69
general	info	NumpyVersion	1.13.1
general	info	PyWaveletVersion	0.5.2
general	info	SimpleITKVersion	1.1.0
general	info	Version	1.3.0.post102+g1b0eb74
general	info	VolumeNum	1
general	info	VoxelNum	242
original	shape	Maximum3DDiameter	36.12478374
original	shape	Maximum2DDiameterSlice	30.59411708
original	shape	Sphericity	0.650484259
original	shape	MinorAxis	23.98294987
original	shape	Elongation	0.657773085
original	shape	SurfaceVolumeRatio	0.397668285
original	shape	Volume	6534
original	shape	MajorAxis	36.46082581
original	shape	SurfaceArea	2598.364575
original	shape	Flatness	0.467987664
original	shape	LeastAxis	17.06321671
original	shape	Maximum2DDiameterColumn	34.98571137
original	shape	Maximum2DDiameterRow	32.31098884
original	gldm	GrayLevelVariance	87.66942149
original	gldm	HighGrayLevelEmphasis	487.6694215
original	gldm	DependenceEntropy	6.121594038
original	gldm	DependenceNonUniformity	89.36363636
original	gldm	GrayLevelNonUniformity	8.280991736
original	gldm	SmallDependenceEmphasis	0.57443067
original	gldm	SmallDependenceHighGrayLevelEmphasis	273.1549885
original	gldm	DependenceNonUniformityNormalized	0.369271225
original	gldm	LargeDependenceEmphasis	3.669421488
original	gldm	LargeDependenceLowGrayLevelEmphasis	0.053732814
original	gldm	DependenceVariance	0.657332149
original	gldm	LargeDependenceHighGrayLevelEmphasis	1945.958678
original	gldm	SmallDependenceLowGrayLevelEmphasis	0.011407452

original	gldm	LowGrayLevelEmphasis	0.018796996
original	glcm	JointAverage	20.98956277
original	glcm	SumAverage	41.97912555
original	glcm	JointEntropy	7.83063399
original	glcm	ClusterShade	-1362.677498
original	glcm	MaximumProbability	0.013186912
original	glcm	Idmn	0.932007282
original	glcm	JointEnergy	0.004803818
original	glcm	Contrast	128.456335
original	glcm	DifferenceEntropy	4.38696607
original	glcm	InverseVariance	0.133536824
original	glcm	DifferenceVariance	50.16653063
original	glcm	Idn	0.834352423
original	glcm	Idm	0.127451233
original	glcm	Correlation	0.198138715
original	glcm	Autocorrelation	456.5389697
original	glcm	SumEntropy	5.387701976
original	glcm	SumSquares	80.12970498
original	glcm	ClusterProminence	108190.9788
original	glcm	Imc2	0.990940192
original	glcm	Imc1	-0.410627121
original	glcm	DifferenceAverage	8.720412437
original	glcm	Id	0.209478019
original	glcm	ClusterTendency	192.0624849
original	firstorder	InterquartileRange	381.5
original	firstorder	Skewness	-0.361733567
original	firstorder	Uniformity	0.034218974
original	firstorder	Median	436.5
original	firstorder	Energy	49528583
original	firstorder	RobustMeanAbsoluteDeviation	152.2858615
original	firstorder	MeanAbsoluteDeviation	199.3631583
original	firstorder	TotalEnergy	1337271741
original	firstorder	Maximum	852
original	firstorder	RootMeanSquared	452.3975753
original	firstorder	90Percentile	662.6
original	firstorder	Minimum	-76
original	firstorder	Entropy	5.013166884
original	firstorder	Range	928
original	firstorder	Variance	54801.79969
original	firstorder	10Percentile	11
original	firstorder	Kurtosis	2.06825824
original	firstorder	Mean	387.1198347
original	glrlm	ShortRunLowGrayLevelEmphasis	0.018599568
original	glrlm	GrayLevelVariance	87.83626036
original	glrlm	LowGrayLevelRunEmphasis	0.018909739
original	glrlm	GrayLevelNonUniformityNormalized	0.034092732
original	glrlm	RunVariance	0.030941021
original	glrlm	GrayLevelNonUniformity	8.017013636
original	glrlm	LongRunEmphasis	1.090690262
original	glrlm	ShortRunHighGrayLevelEmphasis	474.8936909
original	glrlm	RunLengthNonUniformity	222.3630848
original	glrlm	ShortRunEmphasis	0.978649975
original	glrlm	LongRunHighGrayLevelEmphasis	533.1838924

original	glrlm	RunPercentage	0.971710108
original	glrlm	LongRunLowGrayLevelEmphasis	0.02031008
original	glrlm	RunEntropy	5.129966565
original	glrlm	HighGrayLevelRunEmphasis	486.4668393
original	glrlm	RunLengthNonUniformityNormalized	0.94525734
original	glszm	GrayLevelVariance	88.86464646
original	glszm	ZoneVariance	0.733737374
original	glszm	GrayLevelNonUniformityNormalized	0.032800735
original	glszm	SizeZoneNonUniformityNormalized	0.520440771
original	glszm	SizeZoneNonUniformity	85.87272727
original	glszm	GrayLevelNonUniformity	5.412121212
original	glszm	LargeAreaEmphasis	2.884848485
original	glszm	SmallAreaHighGrayLevelEmphasis	354.7356988
original	glszm	ZonePercentage	0.681818182
original	glszm	LargeAreaLowGrayLevelEmphasis	0.046659572
original	glszm	LargeAreaHighGrayLevelEmphasis	1519.939394
original	glszm	HighGrayLevelZoneEmphasis	475.6424242
original	glszm	SmallAreaEmphasis	0.747756682
original	glszm	LowGrayLevelZoneEmphasis	0.019791488
original	glszm	ZoneEntropy	5.937348694
original	glszm	SmallAreaLowGrayLevelEmphasis	0.014854248
original	ngtdm	Coarseness	0.02063193
original	ngtdm	Complexity	3614.468362
original	ngtdm	Strength	9.924696307
original	ngtdm	Contrast	0.825310496
original	ngtdm	Busyness	0.069447424
wavelet-HHH	gldm	GrayLevelVariance	10.10197391
wavelet-HHH	gldm	HighGrayLevelEmphasis	222.8760331
wavelet-HHH	gldm	DependenceEntropy	5.701012918
wavelet-HHH	gldm	DependenceNonUniformity	45.91735537
wavelet-HHH	gldm	GrayLevelNonUniformity	24.54545455
wavelet-HHH	gldm	SmallDependenceEmphasis	0.343847464
wavelet-HHH	gldm	SmallDependenceHighGrayLevelEmphasis	81.26958289
wavelet-HHH	gldm	DependenceNonUniformityNormalized	0.189741138
wavelet-HHH	gldm	LargeDependenceEmphasis	12.27272727
wavelet-HHH	gldm	LargeDependenceLowGrayLevelEmphasis	0.064545896
wavelet-HHH	gldm	DependenceVariance	3.567789085
wavelet-HHH	gldm	LargeDependenceHighGrayLevelEmphasis	2642.438017
wavelet-HHH	gldm	SmallDependenceLowGrayLevelEmphasis	0.006088236
wavelet-HHH	gldm	LowGrayLevelEmphasis	0.009484429
wavelet-HHH	glcm	JointAverage	14.52737728
wavelet-HHH	glcm	SumAverage	29.05475455
wavelet-HHH	glcm	JointEntropy	6.462061352
wavelet-HHH	glcm	ClusterShade	3.290422374
wavelet-HHH	glcm	MaximumProbability	0.045682502
wavelet-HHH	glcm	Idmn	0.969245173
wavelet-HHH	glcm	JointEnergy	0.015711438
wavelet-HHH	glcm	Contrast	20.24280684
wavelet-HHH	glcm	DifferenceEntropy	3.170445272
wavelet-HHH	glcm	InverseVariance	0.285453527
wavelet-HHH	glcm	DifferenceVariance	9.061539274
wavelet-HHH	glcm	Idn	0.888578866
wavelet-HHH	glcm	Idm	0.289695806

wavelet-HHH	glcm	Correlation	-0.035122708
wavelet-HHH	glcm	Autocorrelation	210.6554264
wavelet-HHH	glcm	SumEntropy	3.940997106
wavelet-HHH	glcm	SumSquares	9.727284423
wavelet-HHH	glcm	ClusterProminence	1598.556995
wavelet-HHH	glcm	Imc2	0.860776916
wavelet-HHH	glcm	Imc1	-0.19150889
wavelet-HHH	glcm	DifferenceAverage	3.306532582
wavelet-HHH	glcm	Id	0.373074625
wavelet-HHH	glcm	ClusterTendency	18.66633085
wavelet-HHH	firstorder	InterquartileRange	84.28697621
wavelet-HHH	firstorder	Skewness	0.038496219
wavelet-HHH	firstorder	Uniformity	0.101427498
wavelet-HHH	firstorder	Median	-0.576210215
wavelet-HHH	firstorder	Energy	1528415.918
wavelet-HHH	firstorder	RobustMeanAbsoluteDeviation	37.09376961
wavelet-HHH	firstorder	MeanAbsoluteDeviation	59.01329775
wavelet-HHH	firstorder	TotalEnergy	41267229.77
wavelet-HHH	firstorder	Maximum	244.3471176
wavelet-HHH	firstorder	RootMeanSquared	79.47180792
wavelet-HHH	firstorder	90Percentile	104.6312772
wavelet-HHH	firstorder	Minimum	-348.4063433
wavelet-HHH	firstorder	Entropy	3.602906714
wavelet-HHH	firstorder	Range	592.7534609
wavelet-HHH	firstorder	Variance	6311.81164
wavelet-HHH	firstorder	10Percentile	-91.75744936
wavelet-HHH	firstorder	Kurtosis	4.583129237
wavelet-HHH	firstorder	Mean	1.989123872
wavelet-HHH	glrlm	ShortRunLowGrayLevelEmphasis	0.009570806
wavelet-HHH	glrlm	GrayLevelVariance	10.65548613
wavelet-HHH	glrlm	LowGrayLevelRunEmphasis	0.009851364
wavelet-HHH	glrlm	GrayLevelNonUniformityNormalized	0.097107572
wavelet-HHH	glrlm	RunVariance	0.092617143
wavelet-HHH	glrlm	GrayLevelNonUniformity	21.74173164
wavelet-HHH	glrlm	LongRunEmphasis	1.263604906
wavelet-HHH	glrlm	ShortRunHighGrayLevelEmphasis	211.5160124
wavelet-HHH	glrlm	RunLengthNonUniformity	193.5325881
wavelet-HHH	glrlm	ShortRunEmphasis	0.943891889
wavelet-HHH	glrlm	LongRunHighGrayLevelEmphasis	279.997296
wavelet-HHH	glrlm	RunPercentage	0.924984107
wavelet-HHH	glrlm	LongRunLowGrayLevelEmphasis	0.011167865
wavelet-HHH	glrlm	RunEntropy	3.982936115
wavelet-HHH	glrlm	HighGrayLevelRunEmphasis	223.5701225
wavelet-HHH	glrlm	RunLengthNonUniformityNormalized	0.8637456
wavelet-HHH	glszm	GrayLevelVariance	18.32541233
wavelet-HHH	glszm	ZoneVariance	22.27039931
wavelet-HHH	glszm	GrayLevelNonUniformityNormalized	0.072048611
wavelet-HHH	glszm	SizeZoneNonUniformityNormalized	0.427083333
wavelet-HHH	glszm	SizeZoneNonUniformity	41
wavelet-HHH	glszm	GrayLevelNonUniformity	6.916666667
wavelet-HHH	glszm	LargeAreaEmphasis	28.625
wavelet-HHH	glszm	SmallAreaHighGrayLevelEmphasis	164.0560879
wavelet-HHH	glszm	ZonePercentage	0.396694215

wavelet-HHH	glszm	LargeAreaLowGrayLevelEmphasis	0.151945287
wavelet-HHH	glszm	LargeAreaHighGrayLevelEmphasis	6026.979167
wavelet-HHH	glszm	HighGrayLevelZoneEmphasis	237.4270833
wavelet-HHH	glszm	SmallAreaEmphasis	0.676819511
wavelet-HHH	glszm	LowGrayLevelZoneEmphasis	0.016057478
wavelet-HHH	glszm	ZoneEntropy	5.173671423
wavelet-HHH	glszm	SmallAreaLowGrayLevelEmphasis	0.014313897
wavelet-HHH	ngtdm	Coarseness	0.027488211
wavelet-HHH	ngtdm	Complexity	323.5636768
wavelet-HHH	ngtdm	Strength	2.661099716
wavelet-HHH	ngtdm	Contrast	0.166297117
wavelet-HHH	ngtdm	Busyness	0.141996356
wavelet-HLL	gldm	GrayLevelVariance	34.82222867
wavelet-HLL	gldm	HighGrayLevelEmphasis	268.9628099
wavelet-HLL	gldm	DependenceEntropy	5.922941687
wavelet-HLL	gldm	DependenceNonUniformity	66.37190083
wavelet-HLL	gldm	GrayLevelNonUniformity	13.75206612
wavelet-HLL	gldm	SmallDependenceEmphasis	0.46769682
wavelet-HLL	gldm	SmallDependenceHighGrayLevelEmphasis	144.5136026
wavelet-HLL	gldm	DependenceNonUniformityNormalized	0.274264053
wavelet-HLL	gldm	LargeDependenceEmphasis	6.371900826
wavelet-HLL	gldm	LargeDependenceLowGrayLevelEmphasis	0.069434844
wavelet-HLL	gldm	DependenceVariance	1.575438836
wavelet-HLL	gldm	LargeDependenceHighGrayLevelEmphasis	1479.028926
wavelet-HLL	gldm	SmallDependenceLowGrayLevelEmphasis	0.007644594
wavelet-HLL	gldm	LowGrayLevelEmphasis	0.016746394
wavelet-HLL	glcm	JointAverage	15.18502947
wavelet-HLL	glcm	SumAverage	30.37005893
wavelet-HLL	glcm	JointEntropy	7.340872833
wavelet-HLL	glcm	ClusterShade	477.6342937
wavelet-HLL	glcm	MaximumProbability	0.021854607
wavelet-HLL	glcm	Idmn	0.978858403
wavelet-HLL	glcm	JointEnergy	0.00741383
wavelet-HLL	glcm	Contrast	43.42685866
wavelet-HLL	glcm	DifferenceEntropy	3.609012085
wavelet-HLL	glcm	InverseVariance	0.222814925
wavelet-HLL	glcm	DifferenceVariance	19.43547077
wavelet-HLL	glcm	Idn	0.906677795
wavelet-HLL	glcm	Idm	0.208168436
wavelet-HLL	glcm	Correlation	0.271892314
wavelet-HLL	glcm	Autocorrelation	238.90796
wavelet-HLL	glcm	SumEntropy	4.823030788
wavelet-HLL	glcm	SumSquares	29.95784633
wavelet-HLL	glcm	ClusterProminence	30453.06161
wavelet-HLL	glcm	Imc2	0.959619782
wavelet-HLL	glcm	Imc1	-0.298247844
wavelet-HLL	glcm	DifferenceAverage	4.796938502
wavelet-HLL	glcm	Id	0.298684643
wavelet-HLL	glcm	ClusterTendency	76.40452666
wavelet-HLL	firstorder	InterquartileRange	169.0446595
wavelet-HLL	firstorder	Skewness	0.88051861
wavelet-HLL	firstorder	Uniformity	0.056826719
wavelet-HLL	firstorder	Median	-44.86344239

wavelet-HLL	firstorder	Energy	5462991.523
wavelet-HLL	firstorder	RobustMeanAbsoluteDeviation	72.41087562
wavelet-HLL	firstorder	MeanAbsoluteDeviation	110.4072247
wavelet-HLL	firstorder	TotalEnergy	147500771.1
wavelet-HLL	firstorder	Maximum	655.4647488
wavelet-HLL	firstorder	RootMeanSquared	150.2476127
wavelet-HLL	firstorder	90Percentile	145.2572604
wavelet-HLL	firstorder	Minimum	-394.65713
wavelet-HLL	firstorder	Entropy	4.39613828
wavelet-HLL	firstorder	Range	1050.121879
wavelet-HLL	firstorder	Variance	21671.00747
wavelet-HLL	firstorder	10Percentile	-195.573439
wavelet-HLL	firstorder	Kurtosis	5.71688784
wavelet-HLL	firstorder	Mean	-30.0555763
wavelet-HLL	glrlm	ShortRunLowGrayLevelEmphasis	0.016490454
wavelet-HLL	glrlm	GrayLevelVariance	35.87498802
wavelet-HLL	glrlm	LowGrayLevelRunEmphasis	0.01694346
wavelet-HLL	glrlm	GrayLevelNonUniformityNormalized	0.055584243
wavelet-HLL	glrlm	RunVariance	0.052526482
wavelet-HLL	glrlm	GrayLevelNonUniformity	12.83807218
wavelet-HLL	glrlm	LongRunEmphasis	1.152563443
wavelet-HLL	glrlm	ShortRunHighGrayLevelEmphasis	263.0460666
wavelet-HLL	glrlm	RunLengthNonUniformity	211.1676854
wavelet-HLL	glrlm	ShortRunEmphasis	0.965548379
wavelet-HLL	glrlm	LongRunHighGrayLevelEmphasis	306.0273215
wavelet-HLL	glrlm	RunPercentage	0.954227591
wavelet-HLL	glrlm	LongRunLowGrayLevelEmphasis	0.018821507
wavelet-HLL	glrlm	RunEntropy	4.62468639
wavelet-HLL	glrlm	HighGrayLevelRunEmphasis	270.8916041
wavelet-HLL	glrlm	RunLengthNonUniformityNormalized	0.913691874
wavelet-HLL	glszm	GrayLevelVariance	50.95362718
wavelet-HLL	glszm	ZoneVariance	3.76010101
wavelet-HLL	glszm	GrayLevelNonUniformityNormalized	0.045454545
wavelet-HLL	glszm	SizeZoneNonUniformityNormalized	0.47761708
wavelet-HLL	glszm	SizeZoneNonUniformity	63.04545455
wavelet-HLL	glszm	GrayLevelNonUniformity	6
wavelet-HLL	glszm	LargeAreaEmphasis	7.121212121
wavelet-HLL	glszm	SmallAreaHighGrayLevelEmphasis	230.6521698
wavelet-HLL	glszm	ZonePercentage	0.545454545
wavelet-HLL	glszm	LargeAreaLowGrayLevelEmphasis	0.073184536
wavelet-HLL	glszm	LargeAreaHighGrayLevelEmphasis	1667.204545
wavelet-HLL	glszm	HighGrayLevelZoneEmphasis	297.3484848
wavelet-HLL	glszm	SmallAreaEmphasis	0.716800178
wavelet-HLL	glszm	LowGrayLevelZoneEmphasis	0.018689218
wavelet-HLL	glszm	ZoneEntropy	5.561207522
wavelet-HLL	glszm	SmallAreaLowGrayLevelEmphasis	0.011360789
wavelet-HLL	ngtdm	Coarseness	0.032071514
wavelet-HLL	ngtdm	Complexity	1270.416047
wavelet-HLL	ngtdm	Strength	12.16865752
wavelet-HLL	ngtdm	Contrast	0.257263808
wavelet-HLL	ngtdm	Busyness	0.074252001
wavelet-LHL	gldm	GrayLevelVariance	138.7094632
wavelet-LHL	gldm	HighGrayLevelEmphasis	1127.318182

wavelet-LHL	gldm	DependenceEntropy	6.314940407
wavelet-LHL	gldm	DependenceNonUniformity	104.9421488
wavelet-LHL	gldm	GrayLevelNonUniformity	7.115702479
wavelet-LHL	gldm	SmallDependenceEmphasis	0.669515611
wavelet-LHL	gldm	SmallDependenceHighGrayLevelEmphasis	780.7391563
wavelet-LHL	gldm	DependenceNonUniformityNormalized	0.433645243
wavelet-LHL	gldm	LargeDependenceEmphasis	3.289256198
wavelet-LHL	gldm	LargeDependenceLowGrayLevelEmphasis	0.010916782
wavelet-LHL	gldm	DependenceVariance	0.745099378
wavelet-LHL	gldm	LargeDependenceHighGrayLevelEmphasis	3343.896694
wavelet-LHL	gldm	SmallDependenceLowGrayLevelEmphasis	0.006647467
wavelet-LHL	gldm	LowGrayLevelEmphasis	0.007185633
wavelet-LHL	glcm	JointAverage	31.5141467
wavelet-LHL	glcm	SumAverage	63.0282934
wavelet-LHL	glcm	JointEntropy	7.954344909
wavelet-LHL	glcm	ClusterShade	-103.7731558
wavelet-LHL	glcm	MaximumProbability	0.012004625
wavelet-LHL	glcm	Idmn	0.944879694
wavelet-LHL	glcm	JointEnergy	0.004348809
wavelet-LHL	glcm	Contrast	253.2992985
wavelet-LHL	glcm	DifferenceEntropy	4.723967027
wavelet-LHL	glcm	InverseVariance	0.102339021
wavelet-LHL	glcm	DifferenceVariance	100.8967079
wavelet-LHL	glcm	Idn	0.851320065
wavelet-LHL	glcm	Idm	0.099752431
wavelet-LHL	glcm	Correlation	0.160856934
wavelet-LHL	glcm	Autocorrelation	1017.365252
wavelet-LHL	glcm	SumEntropy	5.750608954
wavelet-LHL	glcm	SumSquares	150.8021512
wavelet-LHL	glcm	ClusterProminence	318739.2867
wavelet-LHL	glcm	Imc2	0.997815567
wavelet-LHL	glcm	Imc1	-0.511920281
wavelet-LHL	glcm	DifferenceAverage	11.95268388
wavelet-LHL	glcm	Id	0.174602185
wavelet-LHL	glcm	ClusterTendency	349.9093062
wavelet-LHL	firstorder	InterquartileRange	428.912118
wavelet-LHL	firstorder	Skewness	-0.040471239
wavelet-LHL	firstorder	Uniformity	0.029403729
wavelet-LHL	firstorder	Median	-3.503292958
wavelet-LHL	firstorder	Energy	21070429.89
wavelet-LHL	firstorder	RobustMeanAbsoluteDeviation	179.8487075
wavelet-LHL	firstorder	MeanAbsoluteDeviation	245.3949626
wavelet-LHL	firstorder	TotalEnergy	568901607
wavelet-LHL	firstorder	Maximum	716.734068
wavelet-LHL	firstorder	RootMeanSquared	295.0726895
wavelet-LHL	firstorder	90Percentile	347.8194638
wavelet-LHL	firstorder	Minimum	-783.1332724
wavelet-LHL	firstorder	Entropy	5.344516079
wavelet-LHL	firstorder	Range	1499.86734
wavelet-LHL	firstorder	Variance	86377.064
wavelet-LHL	firstorder	10Percentile	-422.1743357
wavelet-LHL	firstorder	Kurtosis	2.478951577
wavelet-LHL	firstorder	Mean	-26.28360892

wavelet-LHL	glrlm	ShortRunLowGrayLevelEmphasis	0.007289085
wavelet-LHL	glrlm	GrayLevelVariance	139.3995125
wavelet-LHL	glrlm	LowGrayLevelRunEmphasis	0.007317452
wavelet-LHL	glrlm	GrayLevelNonUniformityNormalized	0.029070826
wavelet-LHL	glrlm	RunVariance	0.023502969
wavelet-LHL	glrlm	GrayLevelNonUniformity	6.874752764
wavelet-LHL	glrlm	LongRunEmphasis	1.07169045
wavelet-LHL	glrlm	ShortRunHighGrayLevelEmphasis	1112.185708
wavelet-LHL	glrlm	RunLengthNonUniformity	225.9101946
wavelet-LHL	glrlm	ShortRunEmphasis	0.98244109
wavelet-LHL	glrlm	LongRunHighGrayLevelEmphasis	1202.279644
wavelet-LHL	glrlm	RunPercentage	0.977113795
wavelet-LHL	glrlm	LongRunLowGrayLevelEmphasis	0.007433669
wavelet-LHL	glrlm	RunEntropy	5.445510484
wavelet-LHL	glrlm	HighGrayLevelRunEmphasis	1130.050576
wavelet-LHL	glrlm	RunLengthNonUniformityNormalized	0.954907911
wavelet-LHL	glszm	GrayLevelVariance	149.3030556
wavelet-LHL	glszm	ZoneVariance	0.692469136
wavelet-LHL	glszm	GrayLevelNonUniformityNormalized	0.026049383
wavelet-LHL	glszm	SizeZoneNonUniformityNormalized	0.642037037
wavelet-LHL	glszm	SizeZoneNonUniformity	115.5666667
wavelet-LHL	glszm	GrayLevelNonUniformity	4.688888889
wavelet-LHL	glszm	LargeAreaEmphasis	2.5
wavelet-LHL	glszm	SmallAreaHighGrayLevelEmphasis	973.6172936
wavelet-LHL	glszm	ZonePercentage	0.743801653
wavelet-LHL	glszm	LargeAreaLowGrayLevelEmphasis	0.011531814
wavelet-LHL	glszm	LargeAreaHighGrayLevelEmphasis	2613.172222
wavelet-LHL	glszm	HighGrayLevelZoneEmphasis	1159.483333
wavelet-LHL	glszm	SmallAreaEmphasis	0.827980324
wavelet-LHL	glszm	LowGrayLevelZoneEmphasis	0.009104096
wavelet-LHL	glszm	ZoneEntropy	6.110739486
wavelet-LHL	glszm	SmallAreaLowGrayLevelEmphasis	0.008820102
wavelet-LHL	ngtdm	Coarseness	0.020209025
wavelet-LHL	ngtdm	Complexity	9180.140021
wavelet-LHL	ngtdm	Strength	20.61378895
wavelet-LHL	ngtdm	Contrast	0.847291246
wavelet-LHL	ngtdm	Busyness	0.032528326
wavelet-LHH	gldm	GrayLevelVariance	51.22081825
wavelet-LHH	gldm	HighGrayLevelEmphasis	708.8677686
wavelet-LHH	gldm	DependenceEntropy	5.87686905
wavelet-LHH	gldm	DependenceNonUniformity	93.73553719
wavelet-LHH	gldm	GrayLevelNonUniformity	10.96694215
wavelet-LHH	gldm	SmallDependenceEmphasis	0.631967401
wavelet-LHH	gldm	SmallDependenceHighGrayLevelEmphasis	455.4301297
wavelet-LHH	gldm	DependenceNonUniformityNormalized	0.387336931
wavelet-LHH	gldm	LargeDependenceEmphasis	4.033057851
wavelet-LHH	gldm	LargeDependenceLowGrayLevelEmphasis	0.011990317
wavelet-LHH	gldm	DependenceVariance	1.020968513
wavelet-LHH	gldm	LargeDependenceHighGrayLevelEmphasis	2695.85124
wavelet-LHH	gldm	SmallDependenceLowGrayLevelEmphasis	0.005547485
wavelet-LHH	gldm	LowGrayLevelEmphasis	0.006301124
wavelet-LHH	glcm	JointAverage	25.60366658
wavelet-LHH	glcm	SumAverage	51.20733316

wavelet-LHH	glcm	JointEntropy	7.62839358
wavelet-LHH	glcm	ClusterShade	18.71410845
wavelet-LHH	glcm	MaximumProbability	0.016457749
wavelet-LHH	glcm	Idmn	0.966346174
wavelet-LHH	glcm	JointEnergy	0.005761317
wavelet-LHH	glcm	Contrast	102.3004818
wavelet-LHH	glcm	DifferenceEntropy	4.128616472
wavelet-LHH	glcm	InverseVariance	0.148410017
wavelet-LHH	glcm	DifferenceVariance	42.55088758
wavelet-LHH	glcm	Idn	0.881516258
wavelet-LHH	glcm	Idm	0.138402139
wavelet-LHH	glcm	Correlation	-0.028847071
wavelet-LHH	glcm	Autocorrelation	654.0878086
wavelet-LHH	glcm	SumEntropy	4.912555861
wavelet-LHH	glcm	SumSquares	49.68080845
wavelet-LHH	glcm	ClusterProminence	48457.39033
wavelet-LHH	glcm	Imc2	0.983425012
wavelet-LHH	glcm	Imc1	-0.368994525
wavelet-LHH	glcm	DifferenceAverage	7.520303247
wavelet-LHH	glcm	Id	0.225345297
wavelet-LHH	glcm	ClusterTendency	96.42275199
wavelet-LHH	firstorder	InterquartileRange	202.7791744
wavelet-LHH	firstorder	Skewness	0.007410039
wavelet-LHH	firstorder	Uniformity	0.045317943
wavelet-LHH	firstorder	Median	8.653072689
wavelet-LHH	firstorder	Energy	7680304.632
wavelet-LHH	firstorder	RobustMeanAbsoluteDeviation	89.75899766
wavelet-LHH	firstorder	MeanAbsoluteDeviation	135.9782967
wavelet-LHH	firstorder	TotalEnergy	207368225.1
wavelet-LHH	firstorder	Maximum	631.0895985
wavelet-LHH	firstorder	RootMeanSquared	178.1482416
wavelet-LHH	firstorder	90Percentile	205.4111466
wavelet-LHH	firstorder	Minimum	-608.4332665
wavelet-LHH	firstorder	Entropy	4.704009667
wavelet-LHH	firstorder	Range	1239.522865
wavelet-LHH	firstorder	Variance	31724.76988
wavelet-LHH	firstorder	10Percentile	-222.0659823
wavelet-LHH	firstorder	Kurtosis	4.343602045
wavelet-LHH	firstorder	Mean	3.467869171
wavelet-LHH	glrlm	ShortRunLowGrayLevelEmphasis	0.006387757
wavelet-LHH	glrlm	GrayLevelVariance	52.0647885
wavelet-LHH	glrlm	LowGrayLevelRunEmphasis	0.006429212
wavelet-LHH	glrlm	GrayLevelNonUniformityNormalized	0.044825696
wavelet-LHH	glrlm	RunVariance	0.030224616
wavelet-LHH	glrlm	GrayLevelNonUniformity	10.54117844
wavelet-LHH	glrlm	LongRunEmphasis	1.090088071
wavelet-LHH	glrlm	ShortRunHighGrayLevelEmphasis	695.623385
wavelet-LHH	glrlm	RunLengthNonUniformity	222.3949731
wavelet-LHH	glrlm	ShortRunEmphasis	0.978580269
wavelet-LHH	glrlm	LongRunHighGrayLevelEmphasis	769.9169093
wavelet-LHH	glrlm	RunPercentage	0.971710108
wavelet-LHH	glrlm	LongRunLowGrayLevelEmphasis	0.006604251
wavelet-LHH	glrlm	RunEntropy	4.840144765

wavelet-LHH	glrlm	HighGrayLevelRunEmphasis	710.0529771
wavelet-LHH	glrlm	RunLengthNonUniformityNormalized	0.945287261
wavelet-LHH	glszm	GrayLevelVariance	62.83319161
wavelet-LHH	glszm	ZoneVariance	1.627409297
wavelet-LHH	glszm	GrayLevelNonUniformityNormalized	0.04180839
wavelet-LHH	glszm	SizeZoneNonUniformityNormalized	0.642502834
wavelet-LHH	glszm	SizeZoneNonUniformity	107.9404762
wavelet-LHH	glszm	GrayLevelNonUniformity	7.023809524
wavelet-LHH	glszm	LargeAreaEmphasis	3.702380952
wavelet-LHH	glszm	SmallAreaHighGrayLevelEmphasis	597.3452735
wavelet-LHH	glszm	ZonePercentage	0.694214876
wavelet-LHH	glszm	LargeAreaLowGrayLevelEmphasis	0.013234488
wavelet-LHH	glszm	LargeAreaHighGrayLevelEmphasis	2451.892857
wavelet-LHH	glszm	HighGrayLevelZoneEmphasis	721
wavelet-LHH	glszm	SmallAreaEmphasis	0.827218537
wavelet-LHH	glszm	LowGrayLevelZoneEmphasis	0.008207505
wavelet-LHH	glszm	ZoneEntropy	5.476337289
wavelet-LHH	glszm	SmallAreaLowGrayLevelEmphasis	0.007820857
wavelet-LHH	ngtdm	Coarseness	0.022303119
wavelet-LHH	ngtdm	Complexity	3290.290835
wavelet-LHH	ngtdm	Strength	10.92739136
wavelet-LHH	ngtdm	Contrast	0.443770808
wavelet-LHH	ngtdm	Busyness	0.048715497
wavelet-LLH	gldm	GrayLevelVariance	76.92008743
wavelet-LLH	gldm	HighGrayLevelEmphasis	564.1983471
wavelet-LLH	gldm	DependenceEntropy	6.267831913
wavelet-LLH	gldm	DependenceNonUniformity	77.3553719
wavelet-LLH	gldm	GrayLevelNonUniformity	9.79338843
wavelet-LLH	gldm	SmallDependenceEmphasis	0.557649149
wavelet-LLH	gldm	SmallDependenceHighGrayLevelEmphasis	338.8593553
wavelet-LLH	gldm	DependenceNonUniformityNormalized	0.319650297
wavelet-LLH	gldm	LargeDependenceEmphasis	5.545454545
wavelet-LLH	gldm	LargeDependenceLowGrayLevelEmphasis	0.022348705
wavelet-LLH	gldm	DependenceVariance	1.578444095
wavelet-LLH	gldm	LargeDependenceHighGrayLevelEmphasis	2911.92562
wavelet-LLH	gldm	SmallDependenceLowGrayLevelEmphasis	0.008721749
wavelet-LLH	gldm	LowGrayLevelEmphasis	0.010087989
wavelet-LLH	glcm	JointAverage	21.71881796
wavelet-LLH	glcm	SumAverage	43.43763591
wavelet-LLH	glcm	JointEntropy	7.636952192
wavelet-LLH	glcm	ClusterShade	-45.04676395
wavelet-LLH	glcm	MaximumProbability	0.015861429
wavelet-LLH	glcm	Idmn	0.96378652
wavelet-LLH	glcm	JointEnergy	0.00572279
wavelet-LLH	glcm	Contrast	118.2833995
wavelet-LLH	glcm	DifferenceEntropy	4.258147727
wavelet-LLH	glcm	InverseVariance	0.149262586
wavelet-LLH	glcm	DifferenceVariance	49.7964731
wavelet-LLH	glcm	Idn	0.879161302
wavelet-LLH	glcm	Idm	0.152128378
wavelet-LLH	glcm	Correlation	0.163130755
wavelet-LLH	glcm	Autocorrelation	483.6370982
wavelet-LLH	glcm	SumEntropy	5.272615468

wavelet-LLH	glcm	SumSquares	71.04853185
wavelet-LLH	glcm	ClusterProminence	100157.201
wavelet-LLH	glcm	Imc2	0.990233444
wavelet-LLH	glcm	Imc1	-0.41186947
wavelet-LLH	glcm	DifferenceAverage	8.046486661
wavelet-LLH	glcm	Id	0.233368516
wavelet-LLH	glcm	ClusterTendency	165.9107279
wavelet-LLH	firstorder	InterquartileRange	322.7507555
wavelet-LLH	firstorder	Skewness	0.247413381
wavelet-LLH	firstorder	Uniformity	0.040468547
wavelet-LLH	firstorder	Median	-18.61286189
wavelet-LLH	firstorder	Energy	11908123.72
wavelet-LLH	firstorder	RobustMeanAbsoluteDeviation	117.202428
wavelet-LLH	firstorder	MeanAbsoluteDeviation	170.3523385
wavelet-LLH	firstorder	TotalEnergy	321519340.4
wavelet-LLH	firstorder	Maximum	731.8179541
wavelet-LLH	firstorder	RootMeanSquared	221.8267856
wavelet-LLH	firstorder	90Percentile	188.8471062
wavelet-LLH	firstorder	Minimum	-556.3371849
wavelet-LLH	firstorder	Entropy	4.910121968
wavelet-LLH	firstorder	Range	1288.155139
wavelet-LLH	firstorder	Variance	47907.73398
wavelet-LLH	firstorder	10Percentile	-302.1413996
wavelet-LLH	firstorder	Kurtosis	3.512512792
wavelet-LLH	firstorder	Mean	-36.04703632
wavelet-LLH	glrlm	ShortRunLowGrayLevelEmphasis	0.010295225
wavelet-LLH	glrlm	GrayLevelVariance	78.64735526
wavelet-LLH	glrlm	LowGrayLevelRunEmphasis	0.010376857
wavelet-LLH	glrlm	GrayLevelNonUniformityNormalized	0.039757995
wavelet-LLH	glrlm	RunVariance	0.041391489
wavelet-LLH	glrlm	GrayLevelNonUniformity	9.25529011
wavelet-LLH	glrlm	LongRunEmphasis	1.123096197
wavelet-LLH	glrlm	ShortRunHighGrayLevelEmphasis	551.0736218
wavelet-LLH	glrlm	RunLengthNonUniformity	215.8301159
wavelet-LLH	glrlm	ShortRunEmphasis	0.971083346
wavelet-LLH	glrlm	LongRunHighGrayLevelEmphasis	629.3883238
wavelet-LLH	glrlm	RunPercentage	0.961856325
wavelet-LLH	glrlm	LongRunLowGrayLevelEmphasis	0.010730533
wavelet-LLH	glrlm	RunEntropy	5.092984145
wavelet-LLH	glrlm	HighGrayLevelRunEmphasis	566.1007911
wavelet-LLH	glrlm	RunLengthNonUniformityNormalized	0.926783855
wavelet-LLH	glszm	GrayLevelVariance	99.64406852
wavelet-LLH	glszm	ZoneVariance	1.942671622
wavelet-LLH	glszm	GrayLevelNonUniformityNormalized	0.033961297
wavelet-LLH	glszm	SizeZoneNonUniformityNormalized	0.568371139
wavelet-LLH	glszm	SizeZoneNonUniformity	86.96078431
wavelet-LLH	glszm	GrayLevelNonUniformity	5.196078431
wavelet-LLH	glszm	LargeAreaEmphasis	4.444444444
wavelet-LLH	glszm	SmallAreaHighGrayLevelEmphasis	486.5614953
wavelet-LLH	glszm	ZonePercentage	0.632231405
wavelet-LLH	glszm	LargeAreaLowGrayLevelEmphasis	0.023500538
wavelet-LLH	glszm	LargeAreaHighGrayLevelEmphasis	2381.751634
wavelet-LLH	glszm	HighGrayLevelZoneEmphasis	595.2156863

wavelet-LLH	glszm	SmallAreaEmphasis	0.781222715
wavelet-LLH	glszm	LowGrayLevelZoneEmphasis	0.014201429
wavelet-LLH	glszm	ZoneEntropy	5.9082578
wavelet-LLH	glszm	SmallAreaLowGrayLevelEmphasis	0.013461849
wavelet-LLH	ngtdm	Coarseness	0.021899803
wavelet-LLH	ngtdm	Complexity	4706.787707
wavelet-LLH	ngtdm	Strength	15.60523955
wavelet-LLH	ngtdm	Contrast	0.493180052
wavelet-LLH	ngtdm	Busyness	0.045814723
wavelet-LLL	gldm	GrayLevelVariance	354.2899563
wavelet-LLL	gldm	HighGrayLevelEmphasis	2443.384298
wavelet-LLL	gldm	DependenceEntropy	6.472311018
wavelet-LLL	gldm	DependenceNonUniformity	116.5702479
wavelet-LLL	gldm	GrayLevelNonUniformity	5.008264463
wavelet-LLL	gldm	SmallDependenceEmphasis	0.710571625
wavelet-LLL	gldm	SmallDependenceHighGrayLevelEmphasis	1659.219697
wavelet-LLL	gldm	DependenceNonUniformityNormalized	0.481695239
wavelet-LLL	gldm	LargeDependenceEmphasis	2.636363636
wavelet-LLL	gldm	LargeDependenceLowGrayLevelEmphasis	0.007
wavelet-LLL	gldm	DependenceVariance	0.472303804
wavelet-LLL	gldm	LargeDependenceHighGrayLevelEmphasis	7446.764463
wavelet-LLL	gldm	SmallDependenceLowGrayLevelEmphasis	0.005619133
wavelet-LLL	gldm	LowGrayLevelEmphasis	0.005862324
wavelet-LLL	glcm	JointAverage	48.29544572
wavelet-LLL	glcm	SumAverage	96.59089144
wavelet-LLL	glcm	JointEntropy	8.055026231
wavelet-LLL	glcm	ClusterShade	-8987.887505
wavelet-LLL	glcm	MaximumProbability	0.009615911
wavelet-LLL	glcm	Idmn	0.95227406
wavelet-LLL	glcm	JointEnergy	0.003938831
wavelet-LLL	glcm	Contrast	425.6990736
wavelet-LLL	glcm	DifferenceEntropy	5.10010795
wavelet-LLL	glcm	InverseVariance	0.055931592
wavelet-LLL	glcm	DifferenceVariance	146.2216385
wavelet-LLL	glcm	Idn	0.85476814
wavelet-LLL	glcm	Idm	0.065674402
wavelet-LLL	glcm	Correlation	0.363345094
wavelet-LLL	glcm	Autocorrelation	2452.578892
wavelet-LLL	glcm	SumEntropy	6.148797109
wavelet-LLL	glcm	SumSquares	332.4836431
wavelet-LLL	glcm	ClusterProminence	2365763.675
wavelet-LLL	glcm	Imc2	0.999329456
wavelet-LLL	glcm	Imc1	-0.584617718
wavelet-LLL	glcm	DifferenceAverage	16.34149874
wavelet-LLL	glcm	Id	0.132054016
wavelet-LLL	glcm	ClusterTendency	904.2354986
wavelet-LLL	firstorder	InterquartileRange	642.289664
wavelet-LLL	firstorder	Skewness	-0.26643609
wavelet-LLL	firstorder	Uniformity	0.020695308
wavelet-LLL	firstorder	Median	1076.661142
wavelet-LLL	firstorder	Energy	322964584.7
wavelet-LLL	firstorder	RobustMeanAbsoluteDeviation	273.3107679
wavelet-LLL	firstorder	MeanAbsoluteDeviation	382.0769687

wavelet-LLL	firstorder	TotalEnergy	8720043788
wavelet-LLL	firstorder	Maximum	2123.166482
wavelet-LLL	firstorder	RootMeanSquared	1155.233483
wavelet-LLL	firstorder	90Percentile	1610.438185
wavelet-LLL	firstorder	Minimum	-67.08234373
wavelet-LLL	firstorder	Entropy	5.804284132
wavelet-LLL	firstorder	Range	2190.248826
wavelet-LLL	firstorder	Variance	221394.2841
wavelet-LLL	firstorder	10Percentile	338.7232753
wavelet-LLL	firstorder	Kurtosis	2.416931387
wavelet-LLL	firstorder	Mean	1055.068773
wavelet-LLL	glrlm	ShortRunLowGrayLevelEmphasis	0.005946615
wavelet-LLL	glrlm	GrayLevelVariance	354.894987
wavelet-LLL	glrlm	LowGrayLevelRunEmphasis	0.005957106
wavelet-LLL	glrlm	GrayLevelNonUniformityNormalized	0.020576184
wavelet-LLL	glrlm	RunVariance	0.018106579
wavelet-LLL	glrlm	GrayLevelNonUniformity	4.889331518
wavelet-LLL	glrlm	LongRunEmphasis	1.055661526
wavelet-LLL	glrlm	ShortRunHighGrayLevelEmphasis	2394.277715
wavelet-LLL	glrlm	RunLengthNonUniformity	229.0569826
wavelet-LLL	glrlm	ShortRunEmphasis	0.986084619
wavelet-LLL	glrlm	LongRunHighGrayLevelEmphasis	2596.650748
wavelet-LLL	glrlm	RunPercentage	0.981881755
wavelet-LLL	glrlm	LongRunLowGrayLevelEmphasis	0.005999067
wavelet-LLL	glrlm	RunEntropy	5.868124794
wavelet-LLL	glrlm	HighGrayLevelRunEmphasis	2434.752322
wavelet-LLL	glrlm	RunLengthNonUniformityNormalized	0.963786842
wavelet-LLL	glszm	GrayLevelVariance	367.1505167
wavelet-LLL	glszm	ZoneVariance	0.384199995
wavelet-LLL	glszm	GrayLevelNonUniformityNormalized	0.019051013
wavelet-LLL	glszm	SizeZoneNonUniformityNormalized	0.665853458
wavelet-LLL	glszm	SizeZoneNonUniformity	127.1780105
wavelet-LLL	glszm	GrayLevelNonUniformity	3.638743455
wavelet-LLL	glszm	LargeAreaEmphasis	1.989528796
wavelet-LLL	glszm	SmallAreaHighGrayLevelEmphasis	1951.754543
wavelet-LLL	glszm	ZonePercentage	0.789256198
wavelet-LLL	glszm	LargeAreaLowGrayLevelEmphasis	0.007910068
wavelet-LLL	glszm	LargeAreaHighGrayLevelEmphasis	5332.675393
wavelet-LLL	glszm	HighGrayLevelZoneEmphasis	2347.633508
wavelet-LLL	glszm	SmallAreaEmphasis	0.843359511
wavelet-LLL	glszm	LowGrayLevelZoneEmphasis	0.007215173
wavelet-LLL	glszm	ZoneEntropy	6.359200771
wavelet-LLL	glszm	SmallAreaLowGrayLevelEmphasis	0.007070021
wavelet-LLL	ngtdm	Coarseness	0.021238385
wavelet-LLL	ngtdm	Complexity	20915.81375
wavelet-LLL	ngtdm	Strength	44.20985234
wavelet-LLL	ngtdm	Contrast	1.74239451
wavelet-LLL	ngtdm	Busyness	0.01747049
wavelet-HHL	gldm	GrayLevelVariance	18.89495253
wavelet-HHL	gldm	HighGrayLevelEmphasis	297.8677686
wavelet-HHL	gldm	DependenceEntropy	5.843473299
wavelet-HHL	gldm	DependenceNonUniformity	49.40495868
wavelet-HHL	gldm	GrayLevelNonUniformity	21.4214876

wavelet-HHL	gldm	SmallDependenceEmphasis	0.39898115
wavelet-HHL	gldm	SmallDependenceHighGrayLevelEmphasis	122.9071353
wavelet-HHL	gldm	DependenceNonUniformityNormalized	0.204152722
wavelet-HHL	gldm	LargeDependenceEmphasis	10.19008264
wavelet-HHL	gldm	LargeDependenceLowGrayLevelEmphasis	0.04412097
wavelet-HHL	gldm	DependenceVariance	2.797076702
wavelet-HHL	gldm	LargeDependenceHighGrayLevelEmphasis	2810.595041
wavelet-HHL	gldm	SmallDependenceLowGrayLevelEmphasis	0.006323314
wavelet-HHL	gldm	LowGrayLevelEmphasis	0.00862598
wavelet-HHL	glcm	JointAverage	16.82114966
wavelet-HHL	glcm	SumAverage	33.64229933
wavelet-HHL	glcm	JointEntropy	6.866237958
wavelet-HHL	glcm	ClusterShade	76.71679631
wavelet-HHL	glcm	MaximumProbability	0.039004423
wavelet-HHL	glcm	Idmn	0.972291275
wavelet-HHL	glcm	JointEnergy	0.011690195
wavelet-HHL	glcm	Contrast	35.84103535
wavelet-HHL	glcm	DifferenceEntropy	3.579447914
wavelet-HHL	glcm	InverseVariance	0.221039648
wavelet-HHL	glcm	DifferenceVariance	15.76002732
wavelet-HHL	glcm	Idn	0.893226334
wavelet-HHL	glcm	Idm	0.238827772
wavelet-HHL	glcm	Correlation	-0.008892444
wavelet-HHL	glcm	Autocorrelation	282.746004
wavelet-HHL	glcm	SumEntropy	4.357962121
wavelet-HHL	glcm	SumSquares	17.70414246
wavelet-HHL	glcm	ClusterProminence	5152.154629
wavelet-HHL	glcm	Imc2	0.917841233
wavelet-HHL	glcm	Imc1	-0.23874069
wavelet-HHL	glcm	DifferenceAverage	4.437249367
wavelet-HHL	glcm	Id	0.325431017
wavelet-HHL	glcm	ClusterTendency	34.97553448
wavelet-HHL	firstorder	InterquartileRange	109.474137
wavelet-HHL	firstorder	Skewness	0.40730617
wavelet-HHL	firstorder	Uniformity	0.088518544
wavelet-HHL	firstorder	Median	-8.122860226
wavelet-HHL	firstorder	Energy	2843651.96
wavelet-HHL	firstorder	RobustMeanAbsoluteDeviation	47.13261816
wavelet-HHL	firstorder	MeanAbsoluteDeviation	78.78673151
wavelet-HHL	firstorder	TotalEnergy	76778602.91
wavelet-HHL	firstorder	Maximum	447.0712696
wavelet-HHL	firstorder	RootMeanSquared	108.4003133
wavelet-HHL	firstorder	90Percentile	142.7077614
wavelet-HHL	firstorder	Minimum	-384.618205
wavelet-HHL	firstorder	Entropy	3.945506307
wavelet-HHL	firstorder	Range	831.6894746
wavelet-HHL	firstorder	Variance	11726.86425
wavelet-HHL	firstorder	10Percentile	-111.3352048
wavelet-HHL	firstorder	Kurtosis	4.884104125
wavelet-HHL	firstorder	Mean	4.874800904
wavelet-HHL	glrlm	ShortRunLowGrayLevelEmphasis	0.008774742
wavelet-HHL	glrlm	GrayLevelVariance	19.84165213
wavelet-HHL	glrlm	LowGrayLevelRunEmphasis	0.008967024

wavelet-HHL	glrlm	GrayLevelNonUniformityNormalized	0.084533883
wavelet-HHL	glrlm	RunVariance	0.079038201
wavelet-HHL	glrlm	GrayLevelNonUniformity	19.11503664
wavelet-HHL	glrlm	LongRunEmphasis	1.228205238
wavelet-HHL	glrlm	ShortRunHighGrayLevelEmphasis	285.3901008
wavelet-HHL	glrlm	RunLengthNonUniformity	198.6669693
wavelet-HHL	glrlm	ShortRunEmphasis	0.950238336
wavelet-HHL	glrlm	LongRunHighGrayLevelEmphasis	362.5864564
wavelet-HHL	glrlm	RunPercentage	0.933884298
wavelet-HHL	glrlm	LongRunLowGrayLevelEmphasis	0.009847816
wavelet-HHL	glrlm	RunEntropy	4.285840085
wavelet-HHL	glrlm	HighGrayLevelRunEmphasis	299.2788175
wavelet-HHL	glrlm	RunLengthNonUniformityNormalized	0.878106279
wavelet-HHL	glszm	GrayLevelVariance	32.6892562
wavelet-HHL	glszm	ZoneVariance	10.46909091
wavelet-HHL	glszm	GrayLevelNonUniformityNormalized	0.053553719
wavelet-HHL	glszm	SizeZoneNonUniformityNormalized	0.49322314
wavelet-HHL	glszm	SizeZoneNonUniformity	54.25454545
wavelet-HHL	glszm	GrayLevelNonUniformity	5.890909091
wavelet-HHL	glszm	LargeAreaEmphasis	15.30909091
wavelet-HHL	glszm	SmallAreaHighGrayLevelEmphasis	224.034386
wavelet-HHL	glszm	ZonePercentage	0.454545455
wavelet-HHL	glszm	LargeAreaLowGrayLevelEmphasis	0.070689124
wavelet-HHL	glszm	LargeAreaHighGrayLevelEmphasis	4097.236364
wavelet-HHL	glszm	HighGrayLevelZoneEmphasis	312.4909091
wavelet-HHL	glszm	SmallAreaEmphasis	0.727721651
wavelet-HHL	glszm	LowGrayLevelZoneEmphasis	0.014371337
wavelet-HHL	glszm	ZoneEntropy	5.416460027
wavelet-HHL	glszm	SmallAreaLowGrayLevelEmphasis	0.013351432
wavelet-HHL	ngtdm	Coarseness	0.024794244
wavelet-HHL	ngtdm	Complexity	943.1745206
wavelet-HHL	ngtdm	Strength	5.964789207
wavelet-HHL	ngtdm	Contrast	0.177794284
wavelet-HHL	ngtdm	Busyness	0.078529944
wavelet-HLH	gldm	GrayLevelVariance	8.162232771
wavelet-HLH	gldm	HighGrayLevelEmphasis	120.8553719
wavelet-HLH	gldm	DependenceEntropy	5.414097414
wavelet-HLH	gldm	DependenceNonUniformity	54.85950413
wavelet-HLH	gldm	GrayLevelNonUniformity	26.04132231
wavelet-HLH	gldm	SmallDependenceEmphasis	0.346444565
wavelet-HLH	gldm	SmallDependenceHighGrayLevelEmphasis	43.6515749
wavelet-HLH	gldm	DependenceNonUniformityNormalized	0.226692166
wavelet-HLH	gldm	LargeDependenceEmphasis	9.884297521
wavelet-HLH	gldm	LargeDependenceLowGrayLevelEmphasis	0.097397253
wavelet-HLH	gldm	DependenceVariance	2.625503722
wavelet-HLH	gldm	LargeDependenceHighGrayLevelEmphasis	1176.954545
wavelet-HLH	gldm	SmallDependenceLowGrayLevelEmphasis	0.009518171
wavelet-HLH	gldm	LowGrayLevelEmphasis	0.016208618
wavelet-HLH	glcm	JointAverage	10.60942777
wavelet-HLH	glcm	SumAverage	21.21885555
wavelet-HLH	glcm	JointEntropy	6.367095735
wavelet-HLH	glcm	ClusterShade	-5.178722579
wavelet-HLH	glcm	MaximumProbability	0.044758449

wavelet-HLH	glcm	Idmn	0.967931864
wavelet-HLH	glcm	JointEnergy	0.016151933
wavelet-HLH	glcm	Contrast	15.8874139
wavelet-HLH	glcm	DifferenceEntropy	3.053837541
wavelet-HLH	glcm	InverseVariance	0.298590697
wavelet-HLH	glcm	DifferenceVariance	6.418131903
wavelet-HLH	glcm	Idn	0.881943049
wavelet-HLH	glcm	Idm	0.281400094
wavelet-HLH	glcm	Correlation	-0.020367395
wavelet-HLH	glcm	Autocorrelation	112.4100538
wavelet-HLH	glcm	SumEntropy	3.869559975
wavelet-HLH	glcm	SumSquares	7.788144194
wavelet-HLH	glcm	ClusterProminence	900.5792903
wavelet-HLH	glcm	Imc2	0.805832659
wavelet-HLH	glcm	Imc1	-0.15374494
wavelet-HLH	glcm	DifferenceAverage	3.054953121
wavelet-HLH	glcm	Id	0.36842369
wavelet-HLH	glcm	ClusterTendency	15.26516287
wavelet-HLH	firstorder	InterquartileRange	81.91817019
wavelet-HLH	firstorder	Skewness	-0.121482135
wavelet-HLH	firstorder	Uniformity	0.10760877
wavelet-HLH	firstorder	Median	7.995711151
wavelet-HLH	firstorder	Energy	1209147.739
wavelet-HLH	firstorder	RobustMeanAbsoluteDeviation	35.54544069
wavelet-HLH	firstorder	MeanAbsoluteDeviation	54.2338237
wavelet-HLH	firstorder	TotalEnergy	32646988.96
wavelet-HLH	firstorder	Maximum	257.9546445
wavelet-HLH	firstorder	RootMeanSquared	70.68577128
wavelet-HLH	firstorder	90Percentile	90.52880915
wavelet-HLH	firstorder	Minimum	-225.3613377
wavelet-HLH	firstorder	Entropy	3.483722536
wavelet-HLH	firstorder	Range	483.3159822
wavelet-HLH	firstorder	Variance	4985.004453
wavelet-HLH	firstorder	10Percentile	-88.55649906
wavelet-HLH	firstorder	Kurtosis	3.623491138
wavelet-HLH	firstorder	Mean	3.387301117
wavelet-HLH	glrlm	ShortRunLowGrayLevelEmphasis	0.016200731
wavelet-HLH	glrlm	GrayLevelVariance	8.530657831
wavelet-HLH	glrlm	LowGrayLevelRunEmphasis	0.016677361
wavelet-HLH	glrlm	GrayLevelNonUniformityNormalized	0.103754765
wavelet-HLH	glrlm	RunVariance	0.075021383
wavelet-HLH	glrlm	GrayLevelNonUniformity	23.47615035
wavelet-HLH	glrlm	LongRunEmphasis	1.221360903
wavelet-HLH	glrlm	ShortRunHighGrayLevelEmphasis	115.1651133
wavelet-HLH	glrlm	RunLengthNonUniformity	198.9987678
wavelet-HLH	glrlm	ShortRunEmphasis	0.950396469
wavelet-HLH	glrlm	LongRunHighGrayLevelEmphasis	147.249156
wavelet-HLH	glrlm	RunPercentage	0.934837889
wavelet-HLH	glrlm	LongRunLowGrayLevelEmphasis	0.018779941
wavelet-HLH	glrlm	RunEntropy	3.817347419
wavelet-HLH	glrlm	HighGrayLevelRunEmphasis	121.0308187
wavelet-HLH	glrlm	RunLengthNonUniformityNormalized	0.878636017
wavelet-HLH	glszm	GrayLevelVariance	14.57774471

wavelet-HLH	glszm	ZoneVariance	18.97162291
wavelet-HLH	glszm	GrayLevelNonUniformityNormalized	0.080880009
wavelet-HLH	glszm	SizeZoneNonUniformityNormalized	0.380805612
wavelet-HLH	glszm	SizeZoneNonUniformity	36.93814433
wavelet-HLH	glszm	GrayLevelNonUniformity	7.845360825
wavelet-HLH	glszm	LargeAreaEmphasis	25.19587629
wavelet-HLH	glszm	SmallAreaHighGrayLevelEmphasis	81.44710872
wavelet-HLH	glszm	ZonePercentage	0.400826446
wavelet-HLH	glszm	LargeAreaLowGrayLevelEmphasis	0.234373509
wavelet-HLH	glszm	LargeAreaHighGrayLevelEmphasis	3068.298969
wavelet-HLH	glszm	HighGrayLevelZoneEmphasis	123.8556701
wavelet-HLH	glszm	SmallAreaEmphasis	0.635084839
wavelet-HLH	glszm	LowGrayLevelZoneEmphasis	0.025671815
wavelet-HLH	glszm	ZoneEntropy	5.180688585
wavelet-HLH	glszm	SmallAreaLowGrayLevelEmphasis	0.021322191
wavelet-HLH	ngtdm	Coarseness	0.025310236
wavelet-HLH	ngtdm	Complexity	269.6247004
wavelet-HLH	ngtdm	Strength	2.224359043
wavelet-HLH	ngtdm	Contrast	0.139308909
wavelet-HLH	ngtdm	Busyness	0.208817787

Image type	Feature Class	Feature Name	11: RTSTRUCT: C99-DNU-B_1_segment_ITV-RUL
general	info	BoundingBox	(1, 1, 1, 14, 12, 9)
general	info	EnabledImageTypes	{u'Original': {}, u'Wavelet': {}}
general	info	GeneralSettings	{'distances': [1], 'additionalInfo': True, 'force2D': False, 'interpolator': 'sitkBSpline', 'resampledPixelSpacing': [3.0], 'label': 1, 'normalizeScale': 1, 'normalize': False, 'force2Ddimension': 0, 'removeOutliers': None, 'minimumROISize': None, u'binWidth': 25.0, 'minimumROIDimensions': 1, u'correctMask': True, 'preCrop': False, u'symmetricalGLCM': True, 'resegmentRange': None, 'padDistance': 5}
general	info	ImageHash	e65113bd6793fcf11f5b95888366fdf387e0c3a3
general	info	ImageSpacing	(0.9765625000000002, 0.9765625000000002, 2.0)
general	info	MaskHash	399efe7a869f8195ecbc3bc4331bd5c42068416d
general	info	NumpyVersion	1.13.1
general	info	PyWaveletVersion	0.5.2
general	info	SimpleITKVersion	1.1.0
general	info	Version	1.3.0.post102+g1b0eb74
general	info	VolumeNum	1
general	info	VoxelNum	285
original	shape	Maximum3DDiameter	48.18713521
original	shape	Maximum2DDiameterSlice	34.20526275
original	shape	Sphericity	0.670166998
original	shape	MinorAxis	21.12011631
original	shape	Elongation	0.477725185
original	shape	SurfaceVolumeRatio	0.365509115
original	shape	Volume	7695
original	shape	MajorAxis	44.20976114
original	shape	SurfaceArea	2812.592643
original	shape	Flatness	0.361618712
original	shape	LeastAxis	15.98707687
original	shape	Maximum2DDiameterColumn	37.94733192
original	shape	Maximum2DDiameterRow	27.16615541
original	gldm	GrayLevelVariance	66.92360726
original	gldm	HighGrayLevelEmphasis	384.6385965
original	gldm	DependenceEntropy	6.160858561
original	gldm	DependenceNonUniformity	95.20350877
original	gldm	GrayLevelNonUniformity	10.25614035
original	gldm	SmallDependenceEmphasis	0.573828127
original	gldm	SmallDependenceHighGrayLevelEmphasis	192.5659566
original	gldm	DependenceNonUniformityNormalized	0.334047399
original	gldm	LargeDependenceEmphasis	5.4
original	gldm	LargeDependenceLowGrayLevelEmphasis	0.052213157
original	gldm	DependenceVariance	1.607756233
original	gldm	LargeDependenceHighGrayLevelEmphasis	3118.45614
original	gldm	SmallDependenceLowGrayLevelEmphasis	0.024079845
original	gldm	LowGrayLevelEmphasis	0.028597083
original	glcm	JointAverage	19.37451922

original	glcm	SumAverage	38.74903844
original	glcm	JointEntropy	8.010715874
original	glcm	ClusterShade	-427.5825814
original	glcm	MaximumProbability	0.013253403
original	glcm	Idmn	0.931418848
original	glcm	JointEnergy	0.004352648
original	glcm	Contrast	84.99715634
original	glcm	DifferenceEntropy	4.161007392
original	glcm	InverseVariance	0.139720413
original	glcm	DifferenceVariance	29.48408129
original	glcm	Idn	0.829184448
original	glcm	Idm	0.140597978
original	glcm	Correlation	0.322185046
original	glcm	Autocorrelation	395.2069702
original	glcm	SumEntropy	5.413877863
original	glcm	SumSquares	62.24742063
original	glcm	ClusterProminence	64455.39293
original	glcm	Imc2	0.979736661
original	glcm	Imc1	-0.33563435
original	glcm	DifferenceAverage	7.325794673
original	glcm	Id	0.226053546
original	glcm	ClusterTendency	163.9925262
original	firstorder	InterquartileRange	350
original	firstorder	Skewness	-0.11114576
original	firstorder	Uniformity	0.035986457
original	firstorder	Median	-306
original	firstorder	Energy	40584082
original	firstorder	RobustMeanAbsoluteDeviation	136.1508926
original	firstorder	MeanAbsoluteDeviation	174.9500769
original	firstorder	TotalEnergy	1095770214
original	firstorder	Maximum	39
original	firstorder	RootMeanSquared	377.3596265
original	firstorder	90Percentile	-33.6
original	firstorder	Minimum	-743
original	firstorder	Entropy	4.875271191
original	firstorder	Range	782
original	firstorder	Variance	41922.41022
original	firstorder	10Percentile	-582
original	firstorder	Kurtosis	1.989002593
original	firstorder	Mean	-316.9824561
original	glrlm	ShortRunLowGrayLevelEmphasis	0.029202431
original	glrlm	GrayLevelVariance	66.40938505
original	glrlm	LowGrayLevelRunEmphasis	0.029408091
original	glrlm	GrayLevelNonUniformityNormalized	0.035975924
original	glrlm	RunVariance	0.041488708
original	glrlm	GrayLevelNonUniformity	9.879572216
original	glrlm	LongRunEmphasis	1.119451236
original	glrlm	ShortRunHighGrayLevelEmphasis	363.8957599
original	glrlm	RunLengthNonUniformity	255.989205
original	glrlm	ShortRunEmphasis	0.972991124
original	glrlm	LongRunHighGrayLevelEmphasis	444.5199521
original	glrlm	RunPercentage	0.963562753
original	glrlm	LongRunLowGrayLevelEmphasis	0.030255071
original	glrlm	RunEntropy	5.026023168
original	glrlm	HighGrayLevelRunEmphasis	378.4498401

original	glrlm	RunLengthNonUniformityNormalized	0.931720465
original	glszm	GrayLevelVariance	64.73418892
original	glszm	ZoneVariance	1.711325009
original	glszm	GrayLevelNonUniformityNormalized	0.036709446
original	glszm	SizeZoneNonUniformityNormalized	0.577465603
original	glszm	SizeZoneNonUniformity	107.4086022
original	glszm	GrayLevelNonUniformity	6.827956989
original	glszm	LargeAreaEmphasis	4.059139785
original	glszm	SmallAreaHighGrayLevelEmphasis	258.719697
original	glszm	ZonePercentage	0.652631579
original	glszm	LargeAreaLowGrayLevelEmphasis	0.056222888
original	glszm	LargeAreaHighGrayLevelEmphasis	2130.225806
original	glszm	HighGrayLevelZoneEmphasis	333.6236559
original	glszm	SmallAreaEmphasis	0.787914717
original	glszm	LowGrayLevelZoneEmphasis	0.038972691
original	glszm	ZoneEntropy	5.681925565
original	glszm	SmallAreaLowGrayLevelEmphasis	0.035790611
original	ngtdm	Coarseness	0.019819568
original	ngtdm	Complexity	1893.992043
original	ngtdm	Strength	6.581884746
original	ngtdm	Contrast	0.708598667
original	ngtdm	Busyness	0.118715147
wavelet-HHH	gldm	GrayLevelVariance	3.751726685
wavelet-HHH	gldm	HighGrayLevelEmphasis	59.92280702
wavelet-HHH	gldm	DependenceEntropy	5.339686826
wavelet-HHH	gldm	DependenceNonUniformity	48.12982456
wavelet-HHH	gldm	GrayLevelNonUniformity	42.73333333
wavelet-HHH	gldm	SmallDependenceEmphasis	0.230785382
wavelet-HHH	gldm	SmallDependenceHighGrayLevelEmphasis	15.51706349
wavelet-HHH	gldm	DependenceNonUniformityNormalized	0.168876577
wavelet-HHH	gldm	LargeDependenceEmphasis	14.74736842
wavelet-HHH	gldm	LargeDependenceLowGrayLevelEmphasis	0.305423622
wavelet-HHH	gldm	DependenceVariance	3.187368421
wavelet-HHH	gldm	LargeDependenceHighGrayLevelEmphasis	846.4421053
wavelet-HHH	gldm	SmallDependenceLowGrayLevelEmphasis	0.008959699
wavelet-HHH	gldm	LowGrayLevelEmphasis	0.025542398
wavelet-HHH	glcm	JointAverage	7.513996365
wavelet-HHH	glcm	SumAverage	15.02799273
wavelet-HHH	glcm	JointEntropy	5.698127116
wavelet-HHH	glcm	ClusterShade	1.441205345
wavelet-HHH	glcm	MaximumProbability	0.055189059
wavelet-HHH	glcm	Idmn	0.95813446
wavelet-HHH	glcm	JointEnergy	0.024130182
wavelet-HHH	glcm	Contrast	7.982929259
wavelet-HHH	glcm	DifferenceEntropy	2.642130832
wavelet-HHH	glcm	InverseVariance	0.345570768
wavelet-HHH	glcm	DifferenceVariance	2.927851129
wavelet-HHH	glcm	Idn	0.86385332
wavelet-HHH	glcm	Idm	0.344532595
wavelet-HHH	glcm	Correlation	-0.046927356
wavelet-HHH	glcm	Autocorrelation	56.28830546
wavelet-HHH	glcm	SumEntropy	3.391202292
wavelet-HHH	glcm	SumSquares	3.819187638
wavelet-HHH	glcm	ClusterProminence	156.6436855
wavelet-HHH	glcm	Imc2	0.610333509

wavelet-HHH	glcm	Imc1	-0.079876785
wavelet-HHH	glcm	DifferenceAverage	2.231871331
wavelet-HHH	glcm	Id	0.425580407
wavelet-HHH	glcm	ClusterTendency	7.293821293
wavelet-HHH	firstorder	InterquartileRange	59.5657757
wavelet-HHH	firstorder	Skewness	0.043504184
wavelet-HHH	firstorder	Uniformity	0.14994152
wavelet-HHH	firstorder	Median	0.783785521
wavelet-HHH	firstorder	Energy	642447.0457
wavelet-HHH	firstorder	RobustMeanAbsoluteDeviation	25.05931977
wavelet-HHH	firstorder	MeanAbsoluteDeviation	37.1229389
wavelet-HHH	firstorder	TotalEnergy	17346070.23
wavelet-HHH	firstorder	Maximum	129.4494087
wavelet-HHH	firstorder	RootMeanSquared	47.47841784
wavelet-HHH	firstorder	90Percentile	60.37577838
wavelet-HHH	firstorder	Minimum	-153.9748087
wavelet-HHH	firstorder	Entropy	2.957467035
wavelet-HHH	firstorder	Range	283.4242174
wavelet-HHH	firstorder	Variance	2254.162913
wavelet-HHH	firstorder	10Percentile	-62.48988125
wavelet-HHH	firstorder	Kurtosis	3.027822763
wavelet-HHH	firstorder	Mean	-0.192994304
wavelet-HHH	glrlm	ShortRunLowGrayLevelEmphasis	0.024527353
wavelet-HHH	glrlm	GrayLevelVariance	3.913989969
wavelet-HHH	glrlm	LowGrayLevelRunEmphasis	0.02603721
wavelet-HHH	glrlm	GrayLevelNonUniformityNormalized	0.145571914
wavelet-HHH	glrlm	RunVariance	0.105103915
wavelet-HHH	glrlm	GrayLevelNonUniformity	37.65727617
wavelet-HHH	glrlm	LongRunEmphasis	1.321140168
wavelet-HHH	glrlm	ShortRunHighGrayLevelEmphasis	55.9816016
wavelet-HHH	glrlm	RunLengthNonUniformity	214.0147713
wavelet-HHH	glrlm	ShortRunEmphasis	0.9271559
wavelet-HHH	glrlm	LongRunHighGrayLevelEmphasis	78.61114183
wavelet-HHH	glrlm	RunPercentage	0.907692308
wavelet-HHH	glrlm	LongRunLowGrayLevelEmphasis	0.03268095
wavelet-HHH	glrlm	RunEntropy	3.423770046
wavelet-HHH	glrlm	HighGrayLevelRunEmphasis	60.17369907
wavelet-HHH	glrlm	RunLengthNonUniformityNormalized	0.826352085
wavelet-HHH	glszm	GrayLevelVariance	7.05315097
wavelet-HHH	glszm	ZoneVariance	40.42434211
wavelet-HHH	glszm	GrayLevelNonUniformityNormalized	0.108033241
wavelet-HHH	glszm	SizeZoneNonUniformityNormalized	0.278739612
wavelet-HHH	glszm	SizeZoneNonUniformity	21.18421053
wavelet-HHH	glszm	GrayLevelNonUniformity	8.210526316
wavelet-HHH	glszm	LargeAreaEmphasis	54.48684211
wavelet-HHH	glszm	SmallAreaHighGrayLevelEmphasis	39.61455398
wavelet-HHH	glszm	ZonePercentage	0.266666667
wavelet-HHH	glszm	LargeAreaLowGrayLevelEmphasis	1.041879365
wavelet-HHH	glszm	LargeAreaHighGrayLevelEmphasis	3182.921053
wavelet-HHH	glszm	HighGrayLevelZoneEmphasis	66.30263158
wavelet-HHH	glszm	SmallAreaEmphasis	0.542042958
wavelet-HHH	glszm	LowGrayLevelZoneEmphasis	0.037226856
wavelet-HHH	glszm	ZoneEntropy	5.159387304
wavelet-HHH	glszm	SmallAreaLowGrayLevelEmphasis	0.026135132
wavelet-HHH	ngtdm	Coarseness	0.020619757

wavelet-HHH	ngtdm	Complexity	96.27116132
wavelet-HHH	ngtdm	Strength	0.850652715
wavelet-HHH	ngtdm	Contrast	0.093793545
wavelet-HHH	ngtdm	Busyness	0.535475563
wavelet-HLL	gldm	GrayLevelVariance	66.58676516
wavelet-HLL	gldm	HighGrayLevelEmphasis	396.9333333
wavelet-HLL	gldm	DependenceEntropy	6.202334193
wavelet-HLL	gldm	DependenceNonUniformity	102.8596491
wavelet-HLL	gldm	GrayLevelNonUniformity	10.4877193
wavelet-HLL	gldm	SmallDependenceEmphasis	0.583876218
wavelet-HLL	gldm	SmallDependenceHighGrayLevelEmphasis	244.899501
wavelet-HLL	gldm	DependenceNonUniformityNormalized	0.36091105
wavelet-HLL	gldm	LargeDependenceEmphasis	3.98245614
wavelet-HLL	gldm	LargeDependenceLowGrayLevelEmphasis	0.056838422
wavelet-HLL	gldm	DependenceVariance	0.842720837
wavelet-HLL	gldm	LargeDependenceHighGrayLevelEmphasis	1653.445614
wavelet-HLL	gldm	SmallDependenceLowGrayLevelEmphasis	0.009907378
wavelet-HLL	gldm	LowGrayLevelEmphasis	0.016981814
wavelet-HLL	glcm	JointAverage	18.34166083
wavelet-HLL	glcm	SumAverage	36.68332166
wavelet-HLL	glcm	JointEntropy	8.027407585
wavelet-HLL	glcm	ClusterShade	26.62837367
wavelet-HLL	glcm	MaximumProbability	0.011928198
wavelet-HLL	glcm	Idmn	0.953466006
wavelet-HLL	glcm	JointEnergy	0.004270643
wavelet-HLL	glcm	Contrast	103.6140842
wavelet-HLL	glcm	DifferenceEntropy	4.133533677
wavelet-HLL	glcm	InverseVariance	0.138075268
wavelet-HLL	glcm	DifferenceVariance	39.41292016
wavelet-HLL	glcm	Idn	0.861490758
wavelet-HLL	glcm	Idm	0.13099661
wavelet-HLL	glcm	Correlation	0.266308805
wavelet-HLL	glcm	Autocorrelation	355.6955933
wavelet-HLL	glcm	SumEntropy	5.432448012
wavelet-HLL	glcm	SumSquares	71.03654969
wavelet-HLL	glcm	ClusterProminence	97081.80282
wavelet-HLL	glcm	Imc2	0.988710008
wavelet-HLL	glcm	Imc1	-0.384762362
wavelet-HLL	glcm	DifferenceAverage	7.670164454
wavelet-HLL	glcm	Id	0.218998536
wavelet-HLL	glcm	ClusterTendency	180.5321146
wavelet-HLL	firstorder	InterquartileRange	273.1591492
wavelet-HLL	firstorder	Skewness	0.143592617
wavelet-HLL	firstorder	Uniformity	0.036799015
wavelet-HLL	firstorder	Median	-28.50764189
wavelet-HLL	firstorder	Energy	12191003.12
wavelet-HLL	firstorder	RobustMeanAbsoluteDeviation	114.5801189
wavelet-HLL	firstorder	MeanAbsoluteDeviation	163.3260591
wavelet-HLL	firstorder	TotalEnergy	329157084.2
wavelet-HLL	firstorder	Maximum	587.1795205
wavelet-HLL	firstorder	RootMeanSquared	206.8222656
wavelet-HLL	firstorder	90Percentile	208.0332893
wavelet-HLL	firstorder	Minimum	-464.3567435
wavelet-HLL	firstorder	Entropy	4.945208891
wavelet-HLL	firstorder	Range	1051.536264

wavelet-HLL	firstorder	Variance	41671.78734
wavelet-HLL	firstorder	10Percentile	-302.595624
wavelet-HLL	firstorder	Kurtosis	2.972550189
wavelet-HLL	firstorder	Mean	-33.22141166
wavelet-HLL	glrlm	ShortRunLowGrayLevelEmphasis	0.016689933
wavelet-HLL	glrlm	GrayLevelVariance	67.07784929
wavelet-HLL	glrlm	LowGrayLevelRunEmphasis	0.017039752
wavelet-HLL	glrlm	GrayLevelNonUniformityNormalized	0.036654567
wavelet-HLL	glrlm	RunVariance	0.031375684
wavelet-HLL	glrlm	GrayLevelNonUniformity	10.13705227
wavelet-HLL	glrlm	LongRunEmphasis	1.095217025
wavelet-HLL	glrlm	ShortRunHighGrayLevelEmphasis	387.8649709
wavelet-HLL	glrlm	RunLengthNonUniformity	260.7691755
wavelet-HLL	glrlm	ShortRunEmphasis	0.977193449
wavelet-HLL	glrlm	LongRunHighGrayLevelEmphasis	434.5774263
wavelet-HLL	glrlm	RunPercentage	0.970310391
wavelet-HLL	glrlm	LongRunLowGrayLevelEmphasis	0.018463334
wavelet-HLL	glrlm	RunEntropy	5.077215447
wavelet-HLL	glrlm	HighGrayLevelRunEmphasis	396.9461962
wavelet-HLL	glrlm	RunLengthNonUniformityNormalized	0.942112154
wavelet-HLL	glszm	GrayLevelVariance	74.50442696
wavelet-HLL	glszm	ZoneVariance	0.982922617
wavelet-HLL	glszm	GrayLevelNonUniformityNormalized	0.035223815
wavelet-HLL	glszm	SizeZoneNonUniformityNormalized	0.552040788
wavelet-HLL	glszm	SizeZoneNonUniformity	105.4397906
wavelet-HLL	glszm	GrayLevelNonUniformity	6.727748691
wavelet-HLL	glszm	LargeAreaEmphasis	3.209424084
wavelet-HLL	glszm	SmallAreaHighGrayLevelEmphasis	332.2326571
wavelet-HLL	glszm	ZonePercentage	0.670175439
wavelet-HLL	glszm	LargeAreaLowGrayLevelEmphasis	0.052600587
wavelet-HLL	glszm	LargeAreaHighGrayLevelEmphasis	1258.209424
wavelet-HLL	glszm	HighGrayLevelZoneEmphasis	411.8324607
wavelet-HLL	glszm	SmallAreaEmphasis	0.771192465
wavelet-HLL	glszm	LowGrayLevelZoneEmphasis	0.016935041
wavelet-HLL	glszm	ZoneEntropy	5.939110188
wavelet-HLL	glszm	SmallAreaLowGrayLevelEmphasis	0.012859255
wavelet-HLL	ngtdm	Coarseness	0.024253518
wavelet-HLL	ngtdm	Complexity	3088.742912
wavelet-HLL	ngtdm	Strength	12.77266745
wavelet-HLL	ngtdm	Contrast	0.420214311
wavelet-HLL	ngtdm	Busyness	0.063117011
wavelet-LHL	gldm	GrayLevelVariance	28.37998153
wavelet-LHL	gldm	HighGrayLevelEmphasis	266.8385965
wavelet-LHL	gldm	DependenceEntropy	5.910777758
wavelet-LHL	gldm	DependenceNonUniformity	85.66666667
wavelet-LHL	gldm	GrayLevelNonUniformity	15.40701754
wavelet-LHL	gldm	SmallDependenceEmphasis	0.506006823
wavelet-LHL	gldm	SmallDependenceHighGrayLevelEmphasis	141.7112817
wavelet-LHL	gldm	DependenceNonUniformityNormalized	0.300584795
wavelet-LHL	gldm	LargeDependenceEmphasis	5.070175439
wavelet-LHL	gldm	LargeDependenceLowGrayLevelEmphasis	0.035161177
wavelet-LHL	gldm	DependenceVariance	1.056128039
wavelet-LHL	gldm	LargeDependenceHighGrayLevelEmphasis	1286.214035
wavelet-LHL	gldm	SmallDependenceLowGrayLevelEmphasis	0.008609521
wavelet-LHL	gldm	LowGrayLevelEmphasis	0.011846821

wavelet-LHL	glcm	JointAverage	15.13647702
wavelet-LHL	glcm	SumAverage	30.27295405
wavelet-LHL	glcm	JointEntropy	7.586929534
wavelet-LHL	glcm	ClusterShade	128.9509535
wavelet-LHL	glcm	MaximumProbability	0.015596387
wavelet-LHL	glcm	Idmn	0.956510166
wavelet-LHL	glcm	JointEnergy	0.006083709
wavelet-LHL	glcm	Contrast	43.54036373
wavelet-LHL	glcm	DifferenceEntropy	3.623983755
wavelet-LHL	glcm	InverseVariance	0.214273217
wavelet-LHL	glcm	DifferenceVariance	17.33191227
wavelet-LHL	glcm	Idn	0.865655314
wavelet-LHL	glcm	Idm	0.188549779
wavelet-LHL	glcm	Correlation	0.245976323
wavelet-LHL	glcm	Autocorrelation	236.4772581
wavelet-LHL	glcm	SumEntropy	4.887622097
wavelet-LHL	glcm	SumSquares	29.06886365
wavelet-LHL	glcm	ClusterProminence	15837.52189
wavelet-LHL	glcm	Imc2	0.955074342
wavelet-LHL	glcm	Imc1	-0.279780387
wavelet-LHL	glcm	DifferenceAverage	4.97579272
wavelet-LHL	glcm	Id	0.28068255
wavelet-LHL	glcm	ClusterTendency	72.73509089
wavelet-LHL	firstorder	InterquartileRange	182.6294714
wavelet-LHL	firstorder	Skewness	-0.066583428
wavelet-LHL	firstorder	Uniformity	0.054059711
wavelet-LHL	firstorder	Median	-25.12281992
wavelet-LHL	firstorder	Energy	5253737.478
wavelet-LHL	firstorder	RobustMeanAbsoluteDeviation	75.67247825
wavelet-LHL	firstorder	MeanAbsoluteDeviation	107.5778347
wavelet-LHL	firstorder	TotalEnergy	141850911.9
wavelet-LHL	firstorder	Maximum	320.758313
wavelet-LHL	firstorder	RootMeanSquared	135.772481
wavelet-LHL	firstorder	90Percentile	152.5243307
wavelet-LHL	firstorder	Minimum	-383.7523664
wavelet-LHL	firstorder	Entropy	4.407865603
wavelet-LHL	firstorder	Range	704.5106794
wavelet-LHL	firstorder	Variance	17765.47032
wavelet-LHL	firstorder	10Percentile	-195.7163477
wavelet-LHL	firstorder	Kurtosis	2.695787738
wavelet-LHL	firstorder	Mean	-25.85916212
wavelet-LHL	glrlm	ShortRunLowGrayLevelEmphasis	0.011904091
wavelet-LHL	glrlm	GrayLevelVariance	28.81550924
wavelet-LHL	glrlm	LowGrayLevelRunEmphasis	0.012081658
wavelet-LHL	glrlm	GrayLevelNonUniformityNormalized	0.053654716
wavelet-LHL	glrlm	RunVariance	0.041901897
wavelet-LHL	glrlm	GrayLevelNonUniformity	14.70165683
wavelet-LHL	glrlm	LongRunEmphasis	1.124283012
wavelet-LHL	glrlm	ShortRunHighGrayLevelEmphasis	260.139367
wavelet-LHL	glrlm	RunLengthNonUniformity	253.7781553
wavelet-LHL	glrlm	ShortRunEmphasis	0.970810205
wavelet-LHL	glrlm	LongRunHighGrayLevelEmphasis	298.8694415
wavelet-LHL	glrlm	RunPercentage	0.961403509
wavelet-LHL	glrlm	LongRunLowGrayLevelEmphasis	0.012823689
wavelet-LHL	glrlm	RunEntropy	4.601863962

wavelet-LHL	glrlm	HighGrayLevelRunEmphasis	267.4467105
wavelet-LHL	glrlm	RunLengthNonUniformityNormalized	0.925928979
wavelet-LHL	glszm	GrayLevelVariance	35.338162
wavelet-LHL	glszm	ZoneVariance	1.752232063
wavelet-LHL	glszm	GrayLevelNonUniformityNormalized	0.04872889
wavelet-LHL	glszm	SizeZoneNonUniformityNormalized	0.500089641
wavelet-LHL	glszm	SizeZoneNonUniformity	83.51497006
wavelet-LHL	glszm	GrayLevelNonUniformity	8.137724551
wavelet-LHL	glszm	LargeAreaEmphasis	4.664670659
wavelet-LHL	glszm	SmallAreaHighGrayLevelEmphasis	209.6778654
wavelet-LHL	glszm	ZonePercentage	0.585964912
wavelet-LHL	glszm	LargeAreaLowGrayLevelEmphasis	0.037297008
wavelet-LHL	glszm	LargeAreaHighGrayLevelEmphasis	1163.383234
wavelet-LHL	glszm	HighGrayLevelZoneEmphasis	279.2215569
wavelet-LHL	glszm	SmallAreaEmphasis	0.733862174
wavelet-LHL	glszm	LowGrayLevelZoneEmphasis	0.015751653
wavelet-LHL	glszm	ZoneEntropy	5.603045699
wavelet-LHL	glszm	SmallAreaLowGrayLevelEmphasis	0.013804181
wavelet-LHL	ngtdm	Coarseness	0.022928954
wavelet-LHL	ngtdm	Complexity	1121.416969
wavelet-LHL	ngtdm	Strength	5.576477541
wavelet-LHL	ngtdm	Contrast	0.251465245
wavelet-LHL	ngtdm	Busyness	0.103257287
wavelet-LHH	gldm	GrayLevelVariance	9.243139428
wavelet-LHH	gldm	HighGrayLevelEmphasis	98.39649123
wavelet-LHH	gldm	DependenceEntropy	5.549070508
wavelet-LHH	gldm	DependenceNonUniformity	58.13684211
wavelet-LHH	gldm	GrayLevelNonUniformity	29.42105263
wavelet-LHH	gldm	SmallDependenceEmphasis	0.371072976
wavelet-LHH	gldm	SmallDependenceHighGrayLevelEmphasis	40.15067575
wavelet-LHH	gldm	DependenceNonUniformityNormalized	0.20398892
wavelet-LHH	gldm	LargeDependenceEmphasis	12.2
wavelet-LHH	gldm	LargeDependenceLowGrayLevelEmphasis	0.177956309
wavelet-LHH	gldm	DependenceVariance	4.062493075
wavelet-LHH	gldm	LargeDependenceHighGrayLevelEmphasis	1104.207018
wavelet-LHH	gldm	SmallDependenceLowGrayLevelEmphasis	0.010516571
wavelet-LHH	gldm	LowGrayLevelEmphasis	0.022575183
wavelet-LHH	glcm	JointAverage	9.470332357
wavelet-LHH	glcm	SumAverage	18.94066471
wavelet-LHH	glcm	JointEntropy	6.656303625
wavelet-LHH	glcm	ClusterShade	-0.38811868
wavelet-LHH	glcm	MaximumProbability	0.04712928
wavelet-LHH	glcm	Idmn	0.952244018
wavelet-LHH	glcm	JointEnergy	0.014162305
wavelet-LHH	glcm	Contrast	20.439459
wavelet-LHH	glcm	DifferenceEntropy	3.209052635
wavelet-LHH	glcm	InverseVariance	0.274191097
wavelet-LHH	glcm	DifferenceVariance	8.246491137
wavelet-LHH	glcm	Idn	0.860294053
wavelet-LHH	glcm	Idm	0.270566125
wavelet-LHH	glcm	Correlation	-0.016562806
wavelet-LHH	glcm	Autocorrelation	89.49452551
wavelet-LHH	glcm	SumEntropy	4.044805788
wavelet-LHH	glcm	SumSquares	10.02299412
wavelet-LHH	glcm	ClusterProminence	1414.241401

wavelet-LHH	glcm	Imc2	0.832043573
wavelet-LHH	glcm	Imc1	-0.165086354
wavelet-LHH	glcm	DifferenceAverage	3.427448394
wavelet-LHH	glcm	Id	0.357014913
wavelet-LHH	glcm	ClusterTendency	19.65251749
wavelet-LHH	firstorder	InterquartileRange	80.81561198
wavelet-LHH	firstorder	Skewness	0.078645914
wavelet-LHH	firstorder	Uniformity	0.103231764
wavelet-LHH	firstorder	Median	-2.9316811
wavelet-LHH	firstorder	Energy	1631463.62
wavelet-LHH	firstorder	RobustMeanAbsoluteDeviation	36.93678973
wavelet-LHH	firstorder	MeanAbsoluteDeviation	57.32254665
wavelet-LHH	firstorder	TotalEnergy	44049517.74
wavelet-LHH	firstorder	Maximum	236.2282851
wavelet-LHH	firstorder	RootMeanSquared	75.6599878
wavelet-LHH	firstorder	90Percentile	98.98303918
wavelet-LHH	firstorder	Minimum	-208.8349773
wavelet-LHH	firstorder	Entropy	3.592067965
wavelet-LHH	firstorder	Range	445.0632624
wavelet-LHH	firstorder	Variance	5720.696456
wavelet-LHH	firstorder	10Percentile	-96.30569335
wavelet-LHH	firstorder	Kurtosis	3.323714213
wavelet-LHH	firstorder	Mean	-1.933209393
wavelet-LHH	glrlm	ShortRunLowGrayLevelEmphasis	0.022242073
wavelet-LHH	glrlm	GrayLevelVariance	9.67600934
wavelet-LHH	glrlm	LowGrayLevelRunEmphasis	0.023107946
wavelet-LHH	glrlm	GrayLevelNonUniformityNormalized	0.098100685
wavelet-LHH	glrlm	RunVariance	0.086075831
wavelet-LHH	glrlm	GrayLevelNonUniformity	25.97280725
wavelet-LHH	glrlm	LongRunEmphasis	1.248439802
wavelet-LHH	glrlm	ShortRunHighGrayLevelEmphasis	93.99005943
wavelet-LHH	glrlm	RunLengthNonUniformity	230.3646874
wavelet-LHH	glrlm	ShortRunEmphasis	0.946013606
wavelet-LHH	glrlm	LongRunHighGrayLevelEmphasis	121.6037408
wavelet-LHH	glrlm	RunPercentage	0.928744939
wavelet-LHH	glrlm	LongRunLowGrayLevelEmphasis	0.026949653
wavelet-LHH	glrlm	RunEntropy	3.952840986
wavelet-LHH	glrlm	HighGrayLevelRunEmphasis	98.93684565
wavelet-LHH	glrlm	RunLengthNonUniformityNormalized	0.868952603
wavelet-LHH	glszm	GrayLevelVariance	14.697856
wavelet-LHH	glszm	ZoneVariance	12.3456
wavelet-LHH	glszm	GrayLevelNonUniformityNormalized	0.079424
wavelet-LHH	glszm	SizeZoneNonUniformityNormalized	0.4272
wavelet-LHH	glszm	SizeZoneNonUniformity	53.4
wavelet-LHH	glszm	GrayLevelNonUniformity	9.928
wavelet-LHH	glszm	LargeAreaEmphasis	17.544
wavelet-LHH	glszm	SmallAreaHighGrayLevelEmphasis	75.57814339
wavelet-LHH	glszm	ZonePercentage	0.438596491
wavelet-LHH	glszm	LargeAreaLowGrayLevelEmphasis	0.240028519
wavelet-LHH	glszm	LargeAreaHighGrayLevelEmphasis	1633.56
wavelet-LHH	glszm	HighGrayLevelZoneEmphasis	105.176
wavelet-LHH	glszm	SmallAreaEmphasis	0.677760611
wavelet-LHH	glszm	LowGrayLevelZoneEmphasis	0.029220015
wavelet-LHH	glszm	ZoneEntropy	5.102282031
wavelet-LHH	glszm	SmallAreaLowGrayLevelEmphasis	0.020467912

wavelet-LHH	ngtdm	Coarseness	0.023928624
wavelet-LHH	ngtdm	Complexity	283.451124
wavelet-LHH	ngtdm	Strength	1.962644721
wavelet-LHH	ngtdm	Contrast	0.144521281
wavelet-LHH	ngtdm	Busyness	0.241052852
wavelet-LLH	gldm	GrayLevelVariance	45.09675592
wavelet-LLH	gldm	HighGrayLevelEmphasis	772.0140351
wavelet-LLH	gldm	DependenceEntropy	5.953313856
wavelet-LLH	gldm	DependenceNonUniformity	96.34035088
wavelet-LLH	gldm	GrayLevelNonUniformity	13.49824561
wavelet-LLH	gldm	SmallDependenceEmphasis	0.533109162
wavelet-LLH	gldm	SmallDependenceHighGrayLevelEmphasis	427.5588567
wavelet-LLH	gldm	DependenceNonUniformityNormalized	0.338036319
wavelet-LLH	gldm	LargeDependenceEmphasis	4.333333333
wavelet-LLH	gldm	LargeDependenceLowGrayLevelEmphasis	0.012340904
wavelet-LLH	gldm	DependenceVariance	0.861975993
wavelet-LLH	gldm	LargeDependenceHighGrayLevelEmphasis	3127.003509
wavelet-LLH	gldm	SmallDependenceLowGrayLevelEmphasis	0.004541735
wavelet-LLH	gldm	LowGrayLevelEmphasis	0.005610045
wavelet-LLH	glcm	JointAverage	26.67191576
wavelet-LLH	glcm	SumAverage	53.34383151
wavelet-LLH	glcm	JointEntropy	7.770199942
wavelet-LLH	glcm	ClusterShade	-233.4436327
wavelet-LLH	glcm	MaximumProbability	0.014042865
wavelet-LLH	glcm	Idmn	0.965558988
wavelet-LLH	glcm	JointEnergy	0.005330908
wavelet-LLH	glcm	Contrast	74.0860365
wavelet-LLH	glcm	DifferenceEntropy	3.966985322
wavelet-LLH	glcm	InverseVariance	0.156436582
wavelet-LLH	glcm	DifferenceVariance	29.36249582
wavelet-LLH	glcm	Idn	0.878230743
wavelet-LLH	glcm	Idm	0.147276592
wavelet-LLH	glcm	Correlation	0.114890649
wavelet-LLH	glcm	Autocorrelation	716.7516241
wavelet-LLH	glcm	SumEntropy	5.040760552
wavelet-LLH	glcm	SumSquares	42.38414185
wavelet-LLH	glcm	ClusterProminence	35196.87115
wavelet-LLH	glcm	Imc2	0.97016921
wavelet-LLH	glcm	Imc1	-0.312701507
wavelet-LLH	glcm	DifferenceAverage	6.510311134
wavelet-LLH	glcm	Id	0.238281888
wavelet-LLH	glcm	ClusterTendency	95.45053088
wavelet-LLH	firstorder	InterquartileRange	232.0559317
wavelet-LLH	firstorder	Skewness	-0.559773212
wavelet-LLH	firstorder	Uniformity	0.047362265
wavelet-LLH	firstorder	Median	-14.97380405
wavelet-LLH	firstorder	Energy	8115403.694
wavelet-LLH	firstorder	RobustMeanAbsoluteDeviation	88.22683209
wavelet-LLH	firstorder	MeanAbsoluteDeviation	129.8230202
wavelet-LLH	firstorder	TotalEnergy	219115899.7
wavelet-LLH	firstorder	Maximum	381.3607272
wavelet-LLH	firstorder	RootMeanSquared	168.7456686
wavelet-LLH	firstorder	90Percentile	175.0807467
wavelet-LLH	firstorder	Minimum	-669.2314008
wavelet-LLH	firstorder	Entropy	4.646273683

wavelet-LLH	firstorder	Range	1050.592128
wavelet-LLH	firstorder	Variance	28279.66221
wavelet-LLH	firstorder	10Percentile	-211.4003213
wavelet-LLH	firstorder	Kurtosis	3.967095986
wavelet-LLH	firstorder	Mean	-13.97993101
wavelet-LLH	glrlm	ShortRunLowGrayLevelEmphasis	0.00567633
wavelet-LLH	glrlm	GrayLevelVariance	45.59336099
wavelet-LLH	glrlm	LowGrayLevelRunEmphasis	0.005730848
wavelet-LLH	glrlm	GrayLevelNonUniformityNormalized	0.04705889
wavelet-LLH	glrlm	RunVariance	0.035988841
wavelet-LLH	glrlm	GrayLevelNonUniformity	12.96648255
wavelet-LLH	glrlm	LongRunEmphasis	1.107306089
wavelet-LLH	glrlm	ShortRunHighGrayLevelEmphasis	755.8010163
wavelet-LLH	glrlm	RunLengthNonUniformity	258.1516201
wavelet-LLH	glrlm	ShortRunEmphasis	0.974781613
wavelet-LLH	glrlm	LongRunHighGrayLevelEmphasis	851.3426416
wavelet-LLH	glrlm	RunPercentage	0.966801619
wavelet-LLH	glrlm	LongRunLowGrayLevelEmphasis	0.005958502
wavelet-LLH	glrlm	RunEntropy	4.801782669
wavelet-LLH	glrlm	HighGrayLevelRunEmphasis	773.8476667
wavelet-LLH	glrlm	RunLengthNonUniformityNormalized	0.936147108
wavelet-LLH	glszm	GrayLevelVariance	52.69042789
wavelet-LLH	glszm	ZoneVariance	1.381167879
wavelet-LLH	glszm	GrayLevelNonUniformityNormalized	0.044287007
wavelet-LLH	glszm	SizeZoneNonUniformityNormalized	0.495646203
wavelet-LLH	glszm	SizeZoneNonUniformity	88.72067039
wavelet-LLH	glszm	GrayLevelNonUniformity	7.927374302
wavelet-LLH	glszm	LargeAreaEmphasis	3.916201117
wavelet-LLH	glszm	SmallAreaHighGrayLevelEmphasis	591.8115864
wavelet-LLH	glszm	ZonePercentage	0.628070175
wavelet-LLH	glszm	LargeAreaLowGrayLevelEmphasis	0.013361297
wavelet-LLH	glszm	LargeAreaHighGrayLevelEmphasis	2860.335196
wavelet-LLH	glszm	HighGrayLevelZoneEmphasis	796.547486
wavelet-LLH	glszm	SmallAreaEmphasis	0.73057025
wavelet-LLH	glszm	LowGrayLevelZoneEmphasis	0.007640958
wavelet-LLH	glszm	ZoneEntropy	5.749003432
wavelet-LLH	glszm	SmallAreaLowGrayLevelEmphasis	0.006959894
wavelet-LLH	ngtdm	Coarseness	0.021355888
wavelet-LLH	ngtdm	Complexity	2237.846707
wavelet-LLH	ngtdm	Strength	8.724039771
wavelet-LLH	ngtdm	Contrast	0.353888587
wavelet-LLH	ngtdm	Busyness	0.050738598
wavelet-LLL	gldm	GrayLevelVariance	369.7403016
wavelet-LLL	gldm	HighGrayLevelEmphasis	2248.126316
wavelet-LLL	gldm	DependenceEntropy	6.493174822
wavelet-LLL	gldm	DependenceNonUniformity	173.7859649
wavelet-LLL	gldm	GrayLevelNonUniformity	4.915789474
wavelet-LLL	gldm	SmallDependenceEmphasis	0.80662768
wavelet-LLL	gldm	SmallDependenceHighGrayLevelEmphasis	1618.887524
wavelet-LLL	gldm	DependenceNonUniformityNormalized	0.609775315
wavelet-LLL	gldm	LargeDependenceEmphasis	1.898245614
wavelet-LLL	gldm	LargeDependenceLowGrayLevelEmphasis	0.006823953
wavelet-LLL	gldm	DependenceVariance	0.25804863
wavelet-LLL	gldm	LargeDependenceHighGrayLevelEmphasis	5112.512281
wavelet-LLL	gldm	SmallDependenceLowGrayLevelEmphasis	0.006093561

wavelet-LLL	gldm	LowGrayLevelEmphasis	0.006228722
wavelet-LLL	glcm	JointAverage	47.31158591
wavelet-LLL	glcm	SumAverage	94.62317182
wavelet-LLL	glcm	JointEntropy	8.38493142
wavelet-LLL	glcm	ClusterShade	1637.986156
wavelet-LLL	glcm	MaximumProbability	0.006722081
wavelet-LLL	glcm	Idmn	0.953887346
wavelet-LLL	glcm	JointEnergy	0.003113054
wavelet-LLL	glcm	Contrast	361.4249863
wavelet-LLL	glcm	DifferenceEntropy	5.015720547
wavelet-LLL	glcm	InverseVariance	0.064199432
wavelet-LLL	glcm	DifferenceVariance	114.0669963
wavelet-LLL	glcm	Idn	0.85487105
wavelet-LLL	glcm	Idm	0.058390315
wavelet-LLL	glcm	Correlation	0.487989676
wavelet-LLL	glcm	Autocorrelation	2406.291563
wavelet-LLL	glcm	SumEntropy	6.393090926
wavelet-LLL	glcm	SumSquares	347.9420573
wavelet-LLL	glcm	ClusterProminence	2501994.759
wavelet-LLL	glcm	Imc2	0.999548302
wavelet-LLL	glcm	Imc1	-0.593345973
wavelet-LLL	glcm	DifferenceAverage	15.29047522
wavelet-LLL	glcm	Id	0.127204072
wavelet-LLL	glcm	ClusterTendency	1030.343243
wavelet-LLL	firstorder	InterquartileRange	684.4624862
wavelet-LLL	firstorder	Skewness	0.12713916
wavelet-LLL	firstorder	Uniformity	0.017248384
wavelet-LLL	firstorder	Median	-952.8497841
wavelet-LLL	firstorder	Energy	311499333.4
wavelet-LLL	firstorder	RobustMeanAbsoluteDeviation	292.2934177
wavelet-LLL	firstorder	MeanAbsoluteDeviation	395.6140697
wavelet-LLL	firstorder	TotalEnergy	8410482002
wavelet-LLL	firstorder	Maximum	56.21531538
wavelet-LLL	firstorder	RootMeanSquared	1045.456894
wavelet-LLL	firstorder	90Percentile	-223.3856374
wavelet-LLL	firstorder	Minimum	-1997.106101
wavelet-LLL	firstorder	Entropy	6.040680746
wavelet-LLL	firstorder	Range	2053.321416
wavelet-LLL	firstorder	Variance	231016.8375
wavelet-LLL	firstorder	10Percentile	-1523.014538
wavelet-LLL	firstorder	Kurtosis	2.235902001
wavelet-LLL	firstorder	Mean	-928.4197756
wavelet-LLL	glrlm	ShortRunLowGrayLevelEmphasis	0.00628396
wavelet-LLL	glrlm	GrayLevelVariance	369.425701
wavelet-LLL	glrlm	LowGrayLevelRunEmphasis	0.006289543
wavelet-LLL	glrlm	GrayLevelNonUniformityNormalized	0.017237264
wavelet-LLL	glrlm	RunVariance	0.010793439
wavelet-LLL	glrlm	GrayLevelNonUniformity	4.859558122
wavelet-LLL	glrlm	LongRunEmphasis	1.032879852
wavelet-LLL	glrlm	ShortRunHighGrayLevelEmphasis	2211.12221
wavelet-LLL	glrlm	RunLengthNonUniformity	275.8625565
wavelet-LLL	glrlm	ShortRunEmphasis	0.991780037
wavelet-LLL	glrlm	LongRunHighGrayLevelEmphasis	2343.215338
wavelet-LLL	glrlm	RunPercentage	0.989203779
wavelet-LLL	glrlm	LongRunLowGrayLevelEmphasis	0.006311878

wavelet-LLL	glrlm	RunEntropy	6.075279725
wavelet-LLL	glrlm	HighGrayLevelRunEmphasis	2237.540835
wavelet-LLL	glrlm	RunLengthNonUniformityNormalized	0.978413122
wavelet-LLL	glszm	GrayLevelVariance	359.4165123
wavelet-LLL	glszm	ZoneVariance	0.162566179
wavelet-LLL	glszm	GrayLevelNonUniformityNormalized	0.017259749
wavelet-LLL	glszm	SizeZoneNonUniformityNormalized	0.758658559
wavelet-LLL	glszm	SizeZoneNonUniformity	187.388664
wavelet-LLL	glszm	GrayLevelNonUniformity	4.263157895
wavelet-LLL	glszm	LargeAreaEmphasis	1.493927126
wavelet-LLL	glszm	SmallAreaHighGrayLevelEmphasis	1746.030589
wavelet-LLL	glszm	ZonePercentage	0.866666667
wavelet-LLL	glszm	LargeAreaLowGrayLevelEmphasis	0.007409685
wavelet-LLL	glszm	LargeAreaHighGrayLevelEmphasis	3721.838057
wavelet-LLL	glszm	HighGrayLevelZoneEmphasis	2088.898785
wavelet-LLL	glszm	SmallAreaEmphasis	0.894511921
wavelet-LLL	glszm	LowGrayLevelZoneEmphasis	0.007081432
wavelet-LLL	glszm	ZoneEntropy	6.369182771
wavelet-LLL	glszm	SmallAreaLowGrayLevelEmphasis	0.007005807
wavelet-LLL	ngtdm	Coarseness	0.023307596
wavelet-LLL	ngtdm	Complexity	21864.09242
wavelet-LLL	ngtdm	Strength	51.51057859
wavelet-LLL	ngtdm	Contrast	1.189356282
wavelet-LLL	ngtdm	Busyness	0.015718872
wavelet-HHL	gldm	GrayLevelVariance	5.414022776
wavelet-HHL	gldm	HighGrayLevelEmphasis	62.1122807
wavelet-HHL	gldm	DependenceEntropy	5.374824616
wavelet-HHL	gldm	DependenceNonUniformity	46.65614035
wavelet-HHL	gldm	GrayLevelNonUniformity	36.97192982
wavelet-HHL	gldm	SmallDependenceEmphasis	0.265345478
wavelet-HHL	gldm	SmallDependenceHighGrayLevelEmphasis	17.62440774
wavelet-HHL	gldm	DependenceNonUniformityNormalized	0.163705756
wavelet-HHL	gldm	LargeDependenceEmphasis	14.60701754
wavelet-HHL	gldm	LargeDependenceLowGrayLevelEmphasis	0.295830097
wavelet-HHL	gldm	DependenceVariance	3.425620191
wavelet-HHL	gldm	LargeDependenceHighGrayLevelEmphasis	860.9192982
wavelet-HHL	gldm	SmallDependenceLowGrayLevelEmphasis	0.013222087
wavelet-HHL	gldm	LowGrayLevelEmphasis	0.029540894
wavelet-HHL	glcm	JointAverage	7.552644758
wavelet-HHL	glcm	SumAverage	15.10528952
wavelet-HHL	glcm	JointEntropy	5.989764239
wavelet-HHL	glcm	ClusterShade	-0.318262284
wavelet-HHL	glcm	MaximumProbability	0.055139337
wavelet-HHL	glcm	Idmn	0.95675209
wavelet-HHL	glcm	JointEnergy	0.021387061
wavelet-HHL	glcm	Contrast	9.76616641
wavelet-HHL	glcm	DifferenceEntropy	2.774115609
wavelet-HHL	glcm	InverseVariance	0.334756252
wavelet-HHL	glcm	DifferenceVariance	3.828035478
wavelet-HHL	glcm	Idn	0.864081959
wavelet-HHL	glcm	Idm	0.334422723
wavelet-HHL	glcm	Correlation	0.010668547
wavelet-HHL	glcm	Autocorrelation	57.10605263
wavelet-HHL	glcm	SumEntropy	3.613466505
wavelet-HHL	glcm	SumSquares	4.943181996

wavelet-HHL	glcm	ClusterProminence	316.1936066
wavelet-HHL	glcm	Imc2	0.68973068
wavelet-HHL	glcm	Imc1	-0.103367701
wavelet-HHL	glcm	DifferenceAverage	2.41796581
wavelet-HHL	glcm	Id	0.415726795
wavelet-HHL	glcm	ClusterTendency	10.00656158
wavelet-HHL	firstorder	InterquartileRange	71.90877427
wavelet-HHL	firstorder	Skewness	0.084535505
wavelet-HHL	firstorder	Uniformity	0.12972607
wavelet-HHL	firstorder	Median	-1.724580135
wavelet-HHL	firstorder	Energy	954285.1377
wavelet-HHL	firstorder	RobustMeanAbsoluteDeviation	30.44362679
wavelet-HHL	firstorder	MeanAbsoluteDeviation	45.20712242
wavelet-HHL	firstorder	TotalEnergy	25765698.72
wavelet-HHL	firstorder	Maximum	160.8234464
wavelet-HHL	firstorder	RootMeanSquared	57.86509228
wavelet-HHL	firstorder	90Percentile	74.80403514
wavelet-HHL	firstorder	Minimum	-153.1352332
wavelet-HHL	firstorder	Entropy	3.226286451
wavelet-HHL	firstorder	Range	313.9586796
wavelet-HHL	firstorder	Variance	3347.960248
wavelet-HHL	firstorder	10Percentile	-75.89017513
wavelet-HHL	firstorder	Kurtosis	3.00192207
wavelet-HHL	firstorder	Mean	0.639262181
wavelet-HHL	glrlm	ShortRunLowGrayLevelEmphasis	0.029031469
wavelet-HHL	glrlm	GrayLevelVariance	5.71925764
wavelet-HHL	glrlm	LowGrayLevelRunEmphasis	0.030457433
wavelet-HHL	glrlm	GrayLevelNonUniformityNormalized	0.123686651
wavelet-HHL	glrlm	RunVariance	0.103511293
wavelet-HHL	glrlm	GrayLevelNonUniformity	32.0722713
wavelet-HHL	glrlm	LongRunEmphasis	1.312754421
wavelet-HHL	glrlm	ShortRunHighGrayLevelEmphasis	58.15723134
wavelet-HHL	glrlm	RunLengthNonUniformity	215.8353309
wavelet-HHL	glrlm	ShortRunEmphasis	0.929791635
wavelet-HHL	glrlm	LongRunHighGrayLevelEmphasis	80.92726084
wavelet-HHL	glrlm	RunPercentage	0.909851552
wavelet-HHL	glrlm	LongRunLowGrayLevelEmphasis	0.036818079
wavelet-HHL	glrlm	RunEntropy	3.682517208
wavelet-HHL	glrlm	HighGrayLevelRunEmphasis	62.36318772
wavelet-HHL	glrlm	RunLengthNonUniformityNormalized	0.831818195
wavelet-HHL	glszm	GrayLevelVariance	11.15121882
wavelet-HHL	glszm	ZoneVariance	69.28613946
wavelet-HHL	glszm	GrayLevelNonUniformityNormalized	0.108843537
wavelet-HHL	glszm	SizeZoneNonUniformityNormalized	0.387188209
wavelet-HHL	glszm	SizeZoneNonUniformity	32.52380952
wavelet-HHL	glszm	GrayLevelNonUniformity	9.142857143
wavelet-HHL	glszm	LargeAreaEmphasis	80.79761905
wavelet-HHL	glszm	SmallAreaHighGrayLevelEmphasis	44.42300888
wavelet-HHL	glszm	ZonePercentage	0.294736842
wavelet-HHL	glszm	LargeAreaLowGrayLevelEmphasis	1.636365398
wavelet-HHL	glszm	LargeAreaHighGrayLevelEmphasis	4368.97619
wavelet-HHL	glszm	HighGrayLevelZoneEmphasis	68.29761905
wavelet-HHL	glszm	SmallAreaEmphasis	0.64401499
wavelet-HHL	glszm	LowGrayLevelZoneEmphasis	0.048493496
wavelet-HHL	glszm	ZoneEntropy	4.738661215

wavelet-HHL	glszm	SmallAreaLowGrayLevelEmphasis	0.038463656
wavelet-HHL	ngtdm	Coarseness	0.023308332
wavelet-HHL	ngtdm	Complexity	132.9611734
wavelet-HHL	ngtdm	Strength	1.164343836
wavelet-HHL	ngtdm	Contrast	0.108759586
wavelet-HHL	ngtdm	Busyness	0.401055726
wavelet-HLH	gldm	GrayLevelVariance	20.82248076
wavelet-HLH	gldm	HighGrayLevelEmphasis	254.5368421
wavelet-HLH	gldm	DependenceEntropy	5.95307161
wavelet-HLH	gldm	DependenceNonUniformity	70.54385965
wavelet-HLH	gldm	GrayLevelNonUniformity	20.29824561
wavelet-HLH	gldm	SmallDependenceEmphasis	0.455417438
wavelet-HLH	gldm	SmallDependenceHighGrayLevelEmphasis	122.5308705
wavelet-HLH	gldm	DependenceNonUniformityNormalized	0.247522315
wavelet-HLH	gldm	LargeDependenceEmphasis	8.29122807
wavelet-HLH	gldm	LargeDependenceLowGrayLevelEmphasis	0.04222517
wavelet-HLH	gldm	DependenceVariance	2.514373653
wavelet-HLH	gldm	LargeDependenceHighGrayLevelEmphasis	2142.207018
wavelet-HLH	gldm	SmallDependenceLowGrayLevelEmphasis	0.008069648
wavelet-HLH	gldm	LowGrayLevelEmphasis	0.010842398
wavelet-HLH	glcm	JointAverage	15.21608868
wavelet-HLH	glcm	SumAverage	30.43217737
wavelet-HLH	glcm	JointEntropy	7.25295522
wavelet-HLH	glcm	ClusterShade	-33.96039735
wavelet-HLH	glcm	MaximumProbability	0.029222834
wavelet-HLH	glcm	Idmn	0.965202237
wavelet-HLH	glcm	JointEnergy	0.00841722
wavelet-HLH	glcm	Contrast	41.14914448
wavelet-HLH	glcm	DifferenceEntropy	3.64940944
wavelet-HLH	glcm	InverseVariance	0.215830917
wavelet-HLH	glcm	DifferenceVariance	17.14789167
wavelet-HLH	glcm	Idn	0.879943777
wavelet-HLH	glcm	Idm	0.210778762
wavelet-HLH	glcm	Correlation	-0.009449932
wavelet-HLH	glcm	Autocorrelation	231.2699017
wavelet-HLH	glcm	SumEntropy	4.460414821
wavelet-HLH	glcm	SumSquares	20.31188758
wavelet-HLH	glcm	ClusterProminence	6905.901839
wavelet-HLH	glcm	Imc2	0.922763493
wavelet-HLH	glcm	Imc1	-0.23710943
wavelet-HLH	glcm	DifferenceAverage	4.795313816
wavelet-HLH	glcm	Id	0.299738144
wavelet-HLH	glcm	ClusterTendency	40.09840584
wavelet-HLH	firstorder	InterquartileRange	137.3748175
wavelet-HLH	firstorder	Skewness	-0.012016879
wavelet-HLH	firstorder	Uniformity	0.071221914
wavelet-HLH	firstorder	Median	0.296960006
wavelet-HLH	firstorder	Energy	3701226.675
wavelet-HLH	firstorder	RobustMeanAbsoluteDeviation	55.95900397
wavelet-HLH	firstorder	MeanAbsoluteDeviation	86.23354259
wavelet-HLH	firstorder	TotalEnergy	99933120.24
wavelet-HLH	firstorder	Maximum	403.2767336
wavelet-HLH	firstorder	RootMeanSquared	113.9594676
wavelet-HLH	firstorder	90Percentile	134.2884436
wavelet-HLH	firstorder	Minimum	-365.1235121

wavelet-HLH	firstorder	Entropy	4.135552628
wavelet-HLH	firstorder	Range	768.4002457
wavelet-HLH	firstorder	Variance	12957.40203
wavelet-HLH	firstorder	10Percentile	-146.4146171
wavelet-HLH	firstorder	Kurtosis	3.816593781
wavelet-HLH	firstorder	Mean	-5.41832383
wavelet-HLH	glrlm	ShortRunLowGrayLevelEmphasis	0.011017334
wavelet-HLH	glrlm	GrayLevelVariance	21.62315583
wavelet-HLH	glrlm	LowGrayLevelRunEmphasis	0.011206632
wavelet-HLH	glrlm	GrayLevelNonUniformityNormalized	0.069139925
wavelet-HLH	glrlm	RunVariance	0.064252968
wavelet-HLH	glrlm	GrayLevelNonUniformity	18.65080071
wavelet-HLH	glrlm	LongRunEmphasis	1.185797549
wavelet-HLH	glrlm	ShortRunHighGrayLevelEmphasis	244.3866397
wavelet-HLH	glrlm	RunLengthNonUniformity	243.2305175
wavelet-HLH	glrlm	ShortRunEmphasis	0.959386834
wavelet-HLH	glrlm	LongRunHighGrayLevelEmphasis	301.8080599
wavelet-HLH	glrlm	RunPercentage	0.946018893
wavelet-HLH	glrlm	LongRunLowGrayLevelEmphasis	0.012052207
wavelet-HLH	glrlm	RunEntropy	4.411501867
wavelet-HLH	glrlm	HighGrayLevelRunEmphasis	254.6190226
wavelet-HLH	glrlm	RunLengthNonUniformityNormalized	0.900410202
wavelet-HLH	glszm	GrayLevelVariance	31.894239
wavelet-HLH	glszm	ZoneVariance	4.965542093
wavelet-HLH	glszm	GrayLevelNonUniformityNormalized	0.052475114
wavelet-HLH	glszm	SizeZoneNonUniformityNormalized	0.495428134
wavelet-HLH	glszm	SizeZoneNonUniformity	73.81879195
wavelet-HLH	glszm	GrayLevelNonUniformity	7.818791946
wavelet-HLH	glszm	LargeAreaEmphasis	8.624161074
wavelet-HLH	glszm	SmallAreaHighGrayLevelEmphasis	202.0415007
wavelet-HLH	glszm	ZonePercentage	0.522807018
wavelet-HLH	glszm	LargeAreaLowGrayLevelEmphasis	0.049263078
wavelet-HLH	glszm	LargeAreaHighGrayLevelEmphasis	2232.114094
wavelet-HLH	glszm	HighGrayLevelZoneEmphasis	263.7919463
wavelet-HLH	glszm	SmallAreaEmphasis	0.72982688
wavelet-HLH	glszm	LowGrayLevelZoneEmphasis	0.016221063
wavelet-HLH	glszm	ZoneEntropy	5.565518796
wavelet-HLH	glszm	SmallAreaLowGrayLevelEmphasis	0.014646756
wavelet-HLH	ngtdm	Coarseness	0.022346515
wavelet-HLH	ngtdm	Complexity	1080.234428
wavelet-HLH	ngtdm	Strength	5.182415542
wavelet-HLH	ngtdm	Contrast	0.194862901
wavelet-HLH	ngtdm	Busyness	0.094999376

Image type	Feature Class	Feature Name	502: RTSTRUCT: C99-DNU_segment_GTV_LUL
general	info	BoundingBox	(0, 0, 1, 8, 7, 6)
general	info	EnabledImageTypes	{u'Original': {}, u'Wavelet': {}}
general	info	GeneralSettings	{'distances': [1], 'additionalInfo': True, 'force2D': False, 'interpolator': 'sitkBSpline', 'resampledPixelSpacing': [3.0], 'label': 1, 'normalizeScale': 1, 'normalize': False, 'force2Ddimension': 0, 'removeOutliers': None, 'minimumROISize': None, u'binWidth': 25.0, 'minimumROIDimensions': 1, u'correctMask': True, 'preCrop': False, u'symmetricalGLCM': True, 'resegmentRange': None, 'padDistance': 5}
general	info	ImageHash	56506f19380279cec30721b0943a23e0ddfc8dfa
general	info	ImageSpacing	(0.9765625000000002, 0.9765625000000002, 2.0)
general	info	MaskHash	d25f3cd83a126bf6f0628f61cda64ef3f9ef1c4c
general	info	NumpyVersion	1.13.1
general	info	PyWaveletVersion	0.5.2
general	info	SimpleITKVersion	1.1.0
general	info	Version	1.3.0.post102+g1b0eb74
general	info	VolumeNum	1
general	info	VoxelNum	139
original	shape	Maximum3DDiameter	22.04540769
original	shape	Maximum2DDiameterSlice	20.1246118
original	shape	Sphericity	0.870004181
original	shape	MinorAxis	17.94730634
original	shape	Elongation	0.896398011
original	shape	SurfaceVolumeRatio	0.357687088
original	shape	Volume	3753
original	shape	MajorAxis	20.02158208
original	shape	SurfaceArea	1342.399642
original	shape	Flatness	0.754797767
original	shape	LeastAxis	15.11224544
original	shape	Maximum2DDiameterColumn	19.20937271
original	shape	Maximum2DDiameterRow	18.97366596
original	gldm	GrayLevelVariance	68.6307127
original	gldm	HighGrayLevelEmphasis	591.6906475
original	gldm	DependenceEntropy	5.622161443
original	gldm	DependenceNonUniformity	46.20863309
original	gldm	GrayLevelNonUniformity	7.273381295
original	gldm	SmallDependenceEmphasis	0.59388715
original	gldm	SmallDependenceHighGrayLevelEmphasis	261.9389633
original	gldm	DependenceNonUniformityNormalized	0.332436209
original	gldm	LargeDependenceEmphasis	11.79136691
original	gldm	LargeDependenceLowGrayLevelEmphasis	0.024194533
original	gldm	DependenceVariance	5.702189328
original	gldm	LargeDependenceHighGrayLevelEmphasis	11206.97122
original	gldm	SmallDependenceLowGrayLevelEmphasis	0.011507858
original	gldm	LowGrayLevelEmphasis	0.012200866
original	glcm	JointAverage	24.81735347
original	glcm	SumAverage	49.63470695
original	glcm	JointEntropy	6.742173297

original	glcm	ClusterShade	-978.5442388
original	glcm	MaximumProbability	0.05066662
original	glcm	Idmn	0.934705247
original	glcm	JointEnergy	0.012752145
original	glcm	Contrast	93.50943944
original	glcm	DifferenceEntropy	4.106394905
original	glcm	InverseVariance	0.167928538
original	glcm	DifferenceVariance	40.44155382
original	glcm	Idn	0.842588786
original	glcm	Idm	0.185825259
original	glcm	Correlation	0.23889078
original	glcm	Autocorrelation	631.1821992
original	glcm	SumEntropy	4.883458019
original	glcm	SumSquares	61.85345152
original	glcm	ClusterProminence	54341.68924
original	glcm	Imc2	0.988372483
original	glcm	Imc1	-0.43835587
original	glcm	DifferenceAverage	7.240499558
original	glcm	Id	0.264149516
original	glcm	ClusterTendency	153.9043667
original	firstorder	InterquartileRange	353
original	firstorder	Skewness	-0.460913951
original	firstorder	Uniformity	0.053568656
original	firstorder	Median	-173
original	firstorder	Energy	12436572
original	firstorder	RobustMeanAbsoluteDeviation	149.3520408
original	firstorder	MeanAbsoluteDeviation	182.5525594
original	firstorder	TotalEnergy	335787444
original	firstorder	Maximum	50
original	firstorder	RootMeanSquared	299.1182726
original	firstorder	90Percentile	24
original	firstorder	Minimum	-766
original	firstorder	Entropy	4.528111152
original	firstorder	Range	816
original	firstorder	Variance	42766.00114
original	firstorder	10Percentile	-484.4
original	firstorder	Kurtosis	2.139035965
original	firstorder	Mean	-216.1151079
original	glrlm	ShortRunLowGrayLevelEmphasis	0.01280578
original	glrlm	GrayLevelVariance	67.98024375
original	glrlm	LowGrayLevelRunEmphasis	0.012859617
original	glrlm	GrayLevelNonUniformityNormalized	0.047994821
original	glrlm	RunVariance	0.071853572
original	glrlm	GrayLevelNonUniformity	6.298149574
original	glrlm	LongRunEmphasis	1.19665256
original	glrlm	ShortRunHighGrayLevelEmphasis	533.1829762
original	glrlm	RunLengthNonUniformity	117.8854216
original	glrlm	ShortRunEmphasis	0.958926986
original	glrlm	LongRunHighGrayLevelEmphasis	756.1961937
original	glrlm	RunPercentage	0.94355285
original	glrlm	LongRunLowGrayLevelEmphasis	0.013106257
original	glrlm	RunEntropy	4.801854395
original	glrlm	HighGrayLevelRunEmphasis	571.0892778
original	glrlm	RunLengthNonUniformityNormalized	0.8982664
original	glszm	GrayLevelVariance	59.55102041

original	glszm	ZoneVariance	4.271223282
original	glszm	GrayLevelNonUniformityNormalized	0.041420118
original	glszm	SizeZoneNonUniformityNormalized	0.677092139
original	glszm	SizeZoneNonUniformity	61.61538462
original	glszm	GrayLevelNonUniformity	3.769230769
original	glszm	LargeAreaEmphasis	6.604395604
original	glszm	SmallAreaHighGrayLevelEmphasis	358.3435003
original	glszm	ZonePercentage	0.654676259
original	glszm	LargeAreaLowGrayLevelEmphasis	0.024229575
original	glszm	LargeAreaHighGrayLevelEmphasis	5952.450549
original	glszm	HighGrayLevelZoneEmphasis	453.8571429
original	glszm	SmallAreaEmphasis	0.847336559
original	glszm	LowGrayLevelZoneEmphasis	0.017812493
original	glszm	ZoneEntropy	5.225725514
original	glszm	SmallAreaLowGrayLevelEmphasis	0.017450459
original	ngtdm	Coarseness	0.030042399
original	ngtdm	Complexity	2149.631067
original	ngtdm	Strength	13.81179231
original	ngtdm	Contrast	0.816503748
original	ngtdm	Busyness	0.043378908
wavelet-HHH	gldm	GrayLevelVariance	13.57724755
wavelet-HHH	gldm	HighGrayLevelEmphasis	198.6546763
wavelet-HHH	gldm	DependenceEntropy	5.57319861
wavelet-HHH	gldm	DependenceNonUniformity	28.55395683
wavelet-HHH	gldm	GrayLevelNonUniformity	12.49640288
wavelet-HHH	gldm	SmallDependenceEmphasis	0.384703054
wavelet-HHH	gldm	SmallDependenceHighGrayLevelEmphasis	81.23109431
wavelet-HHH	gldm	DependenceNonUniformityNormalized	0.20542415
wavelet-HHH	gldm	LargeDependenceEmphasis	9.978417266
wavelet-HHH	gldm	LargeDependenceLowGrayLevelEmphasis	0.070364061
wavelet-HHH	gldm	DependenceVariance	2.622224523
wavelet-HHH	gldm	LargeDependenceHighGrayLevelEmphasis	1768.582734
wavelet-HHH	gldm	SmallDependenceLowGrayLevelEmphasis	0.010839343
wavelet-HHH	gldm	LowGrayLevelEmphasis	0.014493721
wavelet-HHH	glcm	JointAverage	13.55340034
wavelet-HHH	glcm	SumAverage	27.10680067
wavelet-HHH	glcm	JointEntropy	6.440952788
wavelet-HHH	glcm	ClusterShade	-22.38090679
wavelet-HHH	glcm	MaximumProbability	0.04046747
wavelet-HHH	glcm	Idmn	0.956769173
wavelet-HHH	glcm	JointEnergy	0.014177883
wavelet-HHH	glcm	Contrast	26.67498106
wavelet-HHH	glcm	DifferenceEntropy	3.353471491
wavelet-HHH	glcm	InverseVariance	0.235046989
wavelet-HHH	glcm	DifferenceVariance	11.09132895
wavelet-HHH	glcm	Idn	0.866164214
wavelet-HHH	glcm	Idm	0.242530365
wavelet-HHH	glcm	Correlation	-0.042181815
wavelet-HHH	glcm	Autocorrelation	183.1800423
wavelet-HHH	glcm	SumEntropy	4.079983543
wavelet-HHH	glcm	SumSquares	12.81106056
wavelet-HHH	glcm	ClusterProminence	2262.478727
wavelet-HHH	glcm	Imc2	0.928077585
wavelet-HHH	glcm	Imc1	-0.268025092
wavelet-HHH	glcm	DifferenceAverage	3.924737204

wavelet-HHH	glcm	Id	0.332620016
wavelet-HHH	glcm	ClusterTendency	24.56926119
wavelet-HHH	firstorder	InterquartileRange	118.8039488
wavelet-HHH	firstorder	Skewness	-0.308456916
wavelet-HHH	firstorder	Uniformity	0.089902179
wavelet-HHH	firstorder	Median	7.995267163
wavelet-HHH	firstorder	Energy	1198217.506
wavelet-HHH	firstorder	RobustMeanAbsoluteDeviation	46.90875385
wavelet-HHH	firstorder	MeanAbsoluteDeviation	71.59932453
wavelet-HHH	firstorder	TotalEnergy	32351872.65
wavelet-HHH	firstorder	Maximum	240.765582
wavelet-HHH	firstorder	RootMeanSquared	92.8454082
wavelet-HHH	firstorder	90Percentile	121.7597063
wavelet-HHH	firstorder	Minimum	-319.0299667
wavelet-HHH	firstorder	Entropy	3.772231241
wavelet-HHH	firstorder	Range	559.7955487
wavelet-HHH	firstorder	Variance	8617.407666
wavelet-HHH	firstorder	10Percentile	-106.9747467
wavelet-HHH	firstorder	Kurtosis	3.700094243
wavelet-HHH	firstorder	Mean	1.691791264
wavelet-HHH	glrlm	ShortRunLowGrayLevelEmphasis	0.014787697
wavelet-HHH	glrlm	GrayLevelVariance	14.21817609
wavelet-HHH	glrlm	LowGrayLevelRunEmphasis	0.015092102
wavelet-HHH	glrlm	GrayLevelNonUniformityNormalized	0.086992715
wavelet-HHH	glrlm	RunVariance	0.078780959
wavelet-HHH	glrlm	GrayLevelNonUniformity	11.29955573
wavelet-HHH	glrlm	LongRunEmphasis	1.227207999
wavelet-HHH	glrlm	ShortRunHighGrayLevelEmphasis	190.8325424
wavelet-HHH	glrlm	RunLengthNonUniformity	114.0928202
wavelet-HHH	glrlm	ShortRunEmphasis	0.950011434
wavelet-HHH	glrlm	LongRunHighGrayLevelEmphasis	240.8176537
wavelet-HHH	glrlm	RunPercentage	0.934144992
wavelet-HHH	glrlm	LongRunLowGrayLevelEmphasis	0.016479885
wavelet-HHH	glrlm	RunEntropy	4.081772411
wavelet-HHH	glrlm	HighGrayLevelRunEmphasis	199.9215325
wavelet-HHH	glrlm	RunLengthNonUniformityNormalized	0.877576849
wavelet-HHH	glszm	GrayLevelVariance	24.12201021
wavelet-HHH	glszm	ZoneVariance	7.479709755
wavelet-HHH	glszm	GrayLevelNonUniformityNormalized	0.064230046
wavelet-HHH	glszm	SizeZoneNonUniformityNormalized	0.457135179
wavelet-HHH	glszm	SizeZoneNonUniformity	27.8852459
wavelet-HHH	glszm	GrayLevelNonUniformity	3.918032787
wavelet-HHH	glszm	LargeAreaEmphasis	12.67213115
wavelet-HHH	glszm	SmallAreaHighGrayLevelEmphasis	150.0104307
wavelet-HHH	glszm	ZonePercentage	0.438848921
wavelet-HHH	glszm	LargeAreaLowGrayLevelEmphasis	0.093748016
wavelet-HHH	glszm	LargeAreaHighGrayLevelEmphasis	2368.04918
wavelet-HHH	glszm	HighGrayLevelZoneEmphasis	211.0491803
wavelet-HHH	glszm	SmallAreaEmphasis	0.701349186
wavelet-HHH	glszm	LowGrayLevelZoneEmphasis	0.025435491
wavelet-HHH	glszm	ZoneEntropy	5.080338928
wavelet-HHH	glszm	SmallAreaLowGrayLevelEmphasis	0.023677964
wavelet-HHH	ngtdm	Coarseness	0.039153364
wavelet-HHH	ngtdm	Complexity	473.9074441
wavelet-HHH	ngtdm	Strength	4.98351925

wavelet-HHH	ngtdm	Contrast	0.21704954
wavelet-HHH	ngtdm	Busyness	0.093439542
wavelet-HLL	gldm	GrayLevelVariance	61.36245536
wavelet-HLL	gldm	HighGrayLevelEmphasis	320.8273381
wavelet-HLL	gldm	DependenceEntropy	5.851472976
wavelet-HLL	gldm	DependenceNonUniformity	45.30215827
wavelet-HLL	gldm	GrayLevelNonUniformity	6.611510791
wavelet-HLL	gldm	SmallDependenceEmphasis	0.556078576
wavelet-HLL	gldm	SmallDependenceHighGrayLevelEmphasis	195.3329868
wavelet-HLL	gldm	DependenceNonUniformityNormalized	0.325914808
wavelet-HLL	gldm	LargeDependenceEmphasis	5.431654676
wavelet-HLL	gldm	LargeDependenceLowGrayLevelEmphasis	0.046157062
wavelet-HLL	gldm	DependenceVariance	1.574245639
wavelet-HLL	gldm	LargeDependenceHighGrayLevelEmphasis	1360.453237
wavelet-HLL	gldm	SmallDependenceLowGrayLevelEmphasis	0.013248403
wavelet-HLL	gldm	LowGrayLevelEmphasis	0.016287765
wavelet-HLL	glcm	JointAverage	15.5334962
wavelet-HLL	glcm	SumAverage	31.0669924
wavelet-HLL	glcm	JointEntropy	7.050682937
wavelet-HLL	glcm	ClusterShade	1442.808569
wavelet-HLL	glcm	MaximumProbability	0.024295099
wavelet-HLL	glcm	Idmn	0.958716973
wavelet-HLL	glcm	JointEnergy	0.008531976
wavelet-HLL	glcm	Contrast	61.38641656
wavelet-HLL	glcm	DifferenceEntropy	3.8127049
wavelet-HLL	glcm	InverseVariance	0.179246177
wavelet-HLL	glcm	DifferenceVariance	23.65595757
wavelet-HLL	glcm	Idn	0.867398003
wavelet-HLL	glcm	Idm	0.164699812
wavelet-HLL	glcm	Correlation	0.431851672
wavelet-HLL	glcm	Autocorrelation	266.4252939
wavelet-HLL	glcm	SumEntropy	5.060013679
wavelet-HLL	glcm	SumSquares	55.627812
wavelet-HLL	glcm	ClusterProminence	73096.58896
wavelet-HLL	glcm	Imc2	0.991927639
wavelet-HLL	glcm	Imc1	-0.45487092
wavelet-HLL	glcm	DifferenceAverage	6.03638716
wavelet-HLL	glcm	Id	0.253853982
wavelet-HLL	glcm	ClusterTendency	161.1248314
wavelet-HLL	firstorder	InterquartileRange	316.8487539
wavelet-HLL	firstorder	Skewness	0.510296566
wavelet-HLL	firstorder	Uniformity	0.047564826
wavelet-HLL	firstorder	Median	-71.49733479
wavelet-HLL	firstorder	Energy	5550852.756
wavelet-HLL	firstorder	RobustMeanAbsoluteDeviation	118.2675375
wavelet-HLL	firstorder	MeanAbsoluteDeviation	162.1392625
wavelet-HLL	firstorder	TotalEnergy	149873024.4
wavelet-HLL	firstorder	Maximum	457.6282812
wavelet-HLL	firstorder	RootMeanSquared	199.8354135
wavelet-HLL	firstorder	90Percentile	249.302902
wavelet-HLL	firstorder	Minimum	-411.0795453
wavelet-HLL	firstorder	Entropy	4.668668506
wavelet-HLL	firstorder	Range	868.7078265
wavelet-HLL	firstorder	Variance	38761.62087
wavelet-HLL	firstorder	10Percentile	-259.4207336

wavelet-HLL	firstorder	Kurtosis	2.393037255
wavelet-HLL	firstorder	Mean	-34.24283308
wavelet-HLL	glrlm	ShortRunLowGrayLevelEmphasis	0.016473502
wavelet-HLL	glrlm	GrayLevelVariance	62.33967586
wavelet-HLL	glrlm	LowGrayLevelRunEmphasis	0.016658526
wavelet-HLL	glrlm	GrayLevelNonUniformityNormalized	0.046186446
wavelet-HLL	glrlm	RunVariance	0.041653369
wavelet-HLL	glrlm	GrayLevelNonUniformity	6.182079018
wavelet-HLL	glrlm	LongRunEmphasis	1.121610949
wavelet-HLL	glrlm	ShortRunHighGrayLevelEmphasis	316.1351725
wavelet-HLL	glrlm	RunLengthNonUniformity	124.5850906
wavelet-HLL	glrlm	ShortRunEmphasis	0.972203072
wavelet-HLL	glrlm	LongRunHighGrayLevelEmphasis	353.7244609
wavelet-HLL	glrlm	RunPercentage	0.96292197
wavelet-HLL	glrlm	LongRunLowGrayLevelEmphasis	0.017493156
wavelet-HLL	glrlm	RunEntropy	4.823864904
wavelet-HLL	glrlm	HighGrayLevelRunEmphasis	323.4089792
wavelet-HLL	glrlm	RunLengthNonUniformityNormalized	0.930029548
wavelet-HLL	glszm	GrayLevelVariance	73.80358988
wavelet-HLL	glszm	ZoneVariance	2.107308884
wavelet-HLL	glszm	GrayLevelNonUniformityNormalized	0.039256198
wavelet-HLL	glszm	SizeZoneNonUniformityNormalized	0.556818182
wavelet-HLL	glszm	SizeZoneNonUniformity	49
wavelet-HLL	glszm	GrayLevelNonUniformity	3.454545455
wavelet-HLL	glszm	LargeAreaEmphasis	4.602272727
wavelet-HLL	glszm	SmallAreaHighGrayLevelEmphasis	278.9238889
wavelet-HLL	glszm	ZonePercentage	0.633093525
wavelet-HLL	glszm	LargeAreaLowGrayLevelEmphasis	0.046655572
wavelet-HLL	glszm	LargeAreaHighGrayLevelEmphasis	1154.056818
wavelet-HLL	glszm	HighGrayLevelZoneEmphasis	352.4659091
wavelet-HLL	glszm	SmallAreaEmphasis	0.773933081
wavelet-HLL	glszm	LowGrayLevelZoneEmphasis	0.021706597
wavelet-HLL	glszm	ZoneEntropy	5.440695931
wavelet-HLL	glszm	SmallAreaLowGrayLevelEmphasis	0.020173853
wavelet-HLL	ngtdm	Coarseness	0.046019621
wavelet-HLL	ngtdm	Complexity	1762.254856
wavelet-HLL	ngtdm	Strength	19.33878788
wavelet-HLL	ngtdm	Contrast	0.478692962
wavelet-HLL	ngtdm	Busyness	0.051908483
wavelet-LHL	gldm	GrayLevelVariance	84.62170695
wavelet-LHL	gldm	HighGrayLevelEmphasis	810.1294964
wavelet-LHL	gldm	DependenceEntropy	5.737736491
wavelet-LHL	gldm	DependenceNonUniformity	60.68345324
wavelet-LHL	gldm	GrayLevelNonUniformity	5.489208633
wavelet-LHL	gldm	SmallDependenceEmphasis	0.684466427
wavelet-LHL	gldm	SmallDependenceHighGrayLevelEmphasis	603.5635931
wavelet-LHL	gldm	DependenceNonUniformityNormalized	0.436571606
wavelet-LHL	gldm	LargeDependenceEmphasis	3.935251799
wavelet-LHL	gldm	LargeDependenceLowGrayLevelEmphasis	0.015638919
wavelet-LHL	gldm	DependenceVariance	1.125407588
wavelet-LHL	gldm	LargeDependenceHighGrayLevelEmphasis	2485.827338
wavelet-LHL	gldm	SmallDependenceLowGrayLevelEmphasis	0.008537589
wavelet-LHL	gldm	LowGrayLevelEmphasis	0.009202339
wavelet-LHL	glcm	JointAverage	26.31484114
wavelet-LHL	glcm	SumAverage	52.62968227

wavelet-LHL	glcm	JointEntropy	7.192084013
wavelet-LHL	glcm	ClusterShade	1828.648644
wavelet-LHL	glcm	MaximumProbability	0.018574555
wavelet-LHL	glcm	Idmn	0.964620938
wavelet-LHL	glcm	JointEnergy	0.007400427
wavelet-LHL	glcm	Contrast	111.7318927
wavelet-LHL	glcm	DifferenceEntropy	4.167441458
wavelet-LHL	glcm	InverseVariance	0.134896338
wavelet-LHL	glcm	DifferenceVariance	41.24690632
wavelet-LHL	glcm	Idn	0.876343947
wavelet-LHL	glcm	Idm	0.122958347
wavelet-LHL	glcm	Correlation	0.266233534
wavelet-LHL	glcm	Autocorrelation	714.3764562
wavelet-LHL	glcm	SumEntropy	5.171419673
wavelet-LHL	glcm	SumSquares	77.66107851
wavelet-LHL	glcm	ClusterProminence	140209.594
wavelet-LHL	glcm	Imc2	0.995692345
wavelet-LHL	glcm	Imc1	-0.499171958
wavelet-LHL	glcm	DifferenceAverage	8.148939347
wavelet-LHL	glcm	Id	0.209136811
wavelet-LHL	glcm	ClusterTendency	198.9124214
wavelet-LHL	firstorder	InterquartileRange	293.1778961
wavelet-LHL	firstorder	Skewness	0.407179435
wavelet-LHL	firstorder	Uniformity	0.03949071
wavelet-LHL	firstorder	Median	-43.4805139
wavelet-LHL	firstorder	Energy	7335793.802
wavelet-LHL	firstorder	RobustMeanAbsoluteDeviation	120.3796016
wavelet-LHL	firstorder	MeanAbsoluteDeviation	179.5351859
wavelet-LHL	firstorder	TotalEnergy	198066432.6
wavelet-LHL	firstorder	Maximum	627.184419
wavelet-LHL	firstorder	RootMeanSquared	229.7291775
wavelet-LHL	firstorder	90Percentile	291.3486844
wavelet-LHL	firstorder	Minimum	-654.4678538
wavelet-LHL	firstorder	Entropy	4.922637964
wavelet-LHL	firstorder	Range	1281.652273
wavelet-LHL	firstorder	Variance	52572.78529
wavelet-LHL	firstorder	10Percentile	-237.4358067
wavelet-LHL	firstorder	Kurtosis	3.338994981
wavelet-LHL	firstorder	Mean	-14.2376151
wavelet-LHL	glrlm	ShortRunLowGrayLevelEmphasis	0.00935138
wavelet-LHL	glrlm	GrayLevelVariance	85.73920245
wavelet-LHL	glrlm	LowGrayLevelRunEmphasis	0.009392879
wavelet-LHL	glrlm	GrayLevelNonUniformityNormalized	0.038854226
wavelet-LHL	glrlm	RunVariance	0.028282657
wavelet-LHL	glrlm	GrayLevelNonUniformity	5.261254545
wavelet-LHL	glrlm	LongRunEmphasis	1.083243833
wavelet-LHL	glrlm	ShortRunHighGrayLevelEmphasis	803.6727937
wavelet-LHL	glrlm	RunLengthNonUniformity	128.6955473
wavelet-LHL	glrlm	ShortRunEmphasis	0.980474379
wavelet-LHL	glrlm	LongRunHighGrayLevelEmphasis	865.5907837
wavelet-LHL	glrlm	RunPercentage	0.973990039
wavelet-LHL	glrlm	LongRunLowGrayLevelEmphasis	0.009571836
wavelet-LHL	glrlm	RunEntropy	5.032178412
wavelet-LHL	glrlm	HighGrayLevelRunEmphasis	815.6443648
wavelet-LHL	glrlm	RunLengthNonUniformityNormalized	0.95011376

wavelet-LHL	glszm	GrayLevelVariance	98.03174603
wavelet-LHL	glszm	ZoneVariance	0.828480726
wavelet-LHL	glszm	GrayLevelNonUniformityNormalized	0.034195011
wavelet-LHL	glszm	SizeZoneNonUniformityNormalized	0.685623583
wavelet-LHL	glszm	SizeZoneNonUniformity	71.99047619
wavelet-LHL	glszm	GrayLevelNonUniformity	3.59047619
wavelet-LHL	glszm	LargeAreaEmphasis	2.580952381
wavelet-LHL	glszm	SmallAreaHighGrayLevelEmphasis	761.5880952
wavelet-LHL	glszm	ZonePercentage	0.755395683
wavelet-LHL	glszm	LargeAreaLowGrayLevelEmphasis	0.014884558
wavelet-LHL	glszm	LargeAreaHighGrayLevelEmphasis	1806.07619
wavelet-LHL	glszm	HighGrayLevelZoneEmphasis	863.4761905
wavelet-LHL	glszm	SmallAreaEmphasis	0.852906004
wavelet-LHL	glszm	LowGrayLevelZoneEmphasis	0.011503579
wavelet-LHL	glszm	ZoneEntropy	5.493169333
wavelet-LHL	glszm	SmallAreaLowGrayLevelEmphasis	0.011192012
wavelet-LHL	ngtdm	Coarseness	0.038001018
wavelet-LHL	ngtdm	Complexity	3679.603268
wavelet-LHL	ngtdm	Strength	24.48731826
wavelet-LHL	ngtdm	Contrast	0.66892904
wavelet-LHL	ngtdm	Busyness	0.032458353
wavelet-LHH	gldm	GrayLevelVariance	27.55923606
wavelet-LHH	gldm	HighGrayLevelEmphasis	209.5179856
wavelet-LHH	gldm	DependenceEntropy	5.60513071
wavelet-LHH	gldm	DependenceNonUniformity	39.58992806
wavelet-LHH	gldm	GrayLevelNonUniformity	8.841726619
wavelet-LHH	gldm	SmallDependenceEmphasis	0.50198479
wavelet-LHH	gldm	SmallDependenceHighGrayLevelEmphasis	115.461031
wavelet-LHH	gldm	DependenceNonUniformityNormalized	0.284819626
wavelet-LHH	gldm	LargeDependenceEmphasis	7.014388489
wavelet-LHH	gldm	LargeDependenceLowGrayLevelEmphasis	0.060021046
wavelet-LHH	gldm	DependenceVariance	2.136328347
wavelet-LHH	gldm	LargeDependenceHighGrayLevelEmphasis	1270.035971
wavelet-LHH	gldm	SmallDependenceLowGrayLevelEmphasis	0.016202337
wavelet-LHH	gldm	LowGrayLevelEmphasis	0.019634576
wavelet-LHH	glcm	JointAverage	13.5719321
wavelet-LHH	glcm	SumAverage	27.1438642
wavelet-LHH	glcm	JointEntropy	6.858969846
wavelet-LHH	glcm	ClusterShade	114.125692
wavelet-LHH	glcm	MaximumProbability	0.024598342
wavelet-LHH	glcm	Idmn	0.955495696
wavelet-LHH	glcm	JointEnergy	0.009864422
wavelet-LHH	glcm	Contrast	54.21807151
wavelet-LHH	glcm	DifferenceEntropy	3.733309925
wavelet-LHH	glcm	InverseVariance	0.184744419
wavelet-LHH	glcm	DifferenceVariance	22.10062803
wavelet-LHH	glcm	Idn	0.863897078
wavelet-LHH	glcm	Idm	0.183454239
wavelet-LHH	glcm	Correlation	-0.032475534
wavelet-LHH	glcm	Autocorrelation	183.3455529
wavelet-LHH	glcm	SumEntropy	4.511915553
wavelet-LHH	glcm	SumSquares	26.23932895
wavelet-LHH	glcm	ClusterProminence	9879.755907
wavelet-LHH	glcm	Imc2	0.974046774
wavelet-LHH	glcm	Imc1	-0.358479759

wavelet-LHH	glcm	DifferenceAverage	5.575019506
wavelet-LHH	glcm	Id	0.272632339
wavelet-LHH	glcm	ClusterTendency	50.73924431
wavelet-LHH	firstorder	InterquartileRange	140.758373
wavelet-LHH	firstorder	Skewness	0.494208548
wavelet-LHH	firstorder	Uniformity	0.063609544
wavelet-LHH	firstorder	Median	-5.499026234
wavelet-LHH	firstorder	Energy	2414069.625
wavelet-LHH	firstorder	RobustMeanAbsoluteDeviation	63.18112304
wavelet-LHH	firstorder	MeanAbsoluteDeviation	98.50380901
wavelet-LHH	firstorder	TotalEnergy	65179879.86
wavelet-LHH	firstorder	Maximum	474.5818351
wavelet-LHH	firstorder	RootMeanSquared	131.7854596
wavelet-LHH	firstorder	90Percentile	149.2065766
wavelet-LHH	firstorder	Minimum	-318.2064006
wavelet-LHH	firstorder	Entropy	4.232444071
wavelet-LHH	firstorder	Range	792.7882358
wavelet-LHH	firstorder	Variance	17367.40722
wavelet-LHH	firstorder	10Percentile	-156.7813233
wavelet-LHH	firstorder	Kurtosis	4.297188576
wavelet-LHH	firstorder	Mean	0.012344199
wavelet-LHH	glrlm	ShortRunLowGrayLevelEmphasis	0.020038322
wavelet-LHH	glrlm	GrayLevelVariance	28.5256882
wavelet-LHH	glrlm	LowGrayLevelRunEmphasis	0.02026675
wavelet-LHH	glrlm	GrayLevelNonUniformityNormalized	0.062290966
wavelet-LHH	glrlm	RunVariance	0.059325474
wavelet-LHH	glrlm	GrayLevelNonUniformity	8.257634861
wavelet-LHH	glrlm	LongRunEmphasis	1.161255374
wavelet-LHH	glrlm	ShortRunHighGrayLevelEmphasis	204.7975514
wavelet-LHH	glrlm	RunLengthNonUniformity	121.5544821
wavelet-LHH	glrlm	ShortRunEmphasis	0.966343954
wavelet-LHH	glrlm	LongRunHighGrayLevelEmphasis	240.0521386
wavelet-LHH	glrlm	RunPercentage	0.953514112
wavelet-LHH	glrlm	LongRunLowGrayLevelEmphasis	0.021370553
wavelet-LHH	glrlm	RunEntropy	4.43963535
wavelet-LHH	glrlm	HighGrayLevelRunEmphasis	210.9269463
wavelet-LHH	glrlm	RunLengthNonUniformityNormalized	0.916244673
wavelet-LHH	glszm	GrayLevelVariance	41.24146771
wavelet-LHH	glszm	ZoneVariance	3.245954174
wavelet-LHH	glszm	GrayLevelNonUniformityNormalized	0.052074988
wavelet-LHH	glszm	SizeZoneNonUniformityNormalized	0.530523954
wavelet-LHH	glszm	SizeZoneNonUniformity	41.91139241
wavelet-LHH	glszm	GrayLevelNonUniformity	4.113924051
wavelet-LHH	glszm	LargeAreaEmphasis	6.341772152
wavelet-LHH	glszm	SmallAreaHighGrayLevelEmphasis	178.486931
wavelet-LHH	glszm	ZonePercentage	0.568345324
wavelet-LHH	glszm	LargeAreaLowGrayLevelEmphasis	0.064515481
wavelet-LHH	glszm	LargeAreaHighGrayLevelEmphasis	1188.21519
wavelet-LHH	glszm	HighGrayLevelZoneEmphasis	227.443038
wavelet-LHH	glszm	SmallAreaEmphasis	0.755021761
wavelet-LHH	glszm	LowGrayLevelZoneEmphasis	0.029300242
wavelet-LHH	glszm	ZoneEntropy	5.13125935
wavelet-LHH	glszm	SmallAreaLowGrayLevelEmphasis	0.027612288
wavelet-LHH	ngtdm	Coarseness	0.042037664
wavelet-LHH	ngtdm	Complexity	1118.427546

wavelet-LHH	ngtdm	Strength	9.300162281
wavelet-LHH	ngtdm	Contrast	0.349743681
wavelet-LHH	ngtdm	Busyness	0.072249238
wavelet-LLH	gldm	GrayLevelVariance	40.53537602
wavelet-LLH	gldm	HighGrayLevelEmphasis	294.4676259
wavelet-LLH	gldm	DependenceEntropy	5.583894203
wavelet-LLH	gldm	DependenceNonUniformity	55.44604317
wavelet-LLH	gldm	GrayLevelNonUniformity	6.81294964
wavelet-LLH	gldm	SmallDependenceEmphasis	0.645809353
wavelet-LLH	gldm	SmallDependenceHighGrayLevelEmphasis	206.9063689
wavelet-LLH	gldm	DependenceNonUniformityNormalized	0.398892397
wavelet-LLH	gldm	LargeDependenceEmphasis	3.848920863
wavelet-LLH	gldm	LargeDependenceLowGrayLevelEmphasis	0.032522815
wavelet-LLH	gldm	DependenceVariance	0.9417732
wavelet-LLH	gldm	LargeDependenceHighGrayLevelEmphasis	926.0359712
wavelet-LLH	gldm	SmallDependenceLowGrayLevelEmphasis	0.012758561
wavelet-LLH	gldm	LowGrayLevelEmphasis	0.015176334
wavelet-LLH	glcm	JointAverage	15.52990868
wavelet-LLH	glcm	SumAverage	31.05981735
wavelet-LLH	glcm	JointEntropy	7.077165654
wavelet-LLH	glcm	ClusterShade	271.4938214
wavelet-LLH	glcm	MaximumProbability	0.021045336
wavelet-LLH	glcm	Idmn	0.948220448
wavelet-LLH	glcm	JointEnergy	0.008254592
wavelet-LLH	glcm	Contrast	62.7991904
wavelet-LLH	glcm	DifferenceEntropy	3.833828529
wavelet-LLH	glcm	InverseVariance	0.163287647
wavelet-LLH	glcm	DifferenceVariance	22.7553858
wavelet-LLH	glcm	Idn	0.849296351
wavelet-LLH	glcm	Idm	0.142224054
wavelet-LLH	glcm	Correlation	0.147721386
wavelet-LLH	glcm	Autocorrelation	246.9851191
wavelet-LLH	glcm	SumEntropy	4.803309492
wavelet-LLH	glcm	SumSquares	37.15040549
wavelet-LLH	glcm	ClusterProminence	24018.62756
wavelet-LLH	glcm	Imc2	0.988648579
wavelet-LLH	glcm	Imc1	-0.425583965
wavelet-LLH	glcm	DifferenceAverage	6.234541527
wavelet-LLH	glcm	Id	0.234528596
wavelet-LLH	glcm	ClusterTendency	85.80243158
wavelet-LLH	firstorder	InterquartileRange	204.5731135
wavelet-LLH	firstorder	Skewness	0.226409706
wavelet-LLH	firstorder	Uniformity	0.049014026
wavelet-LLH	firstorder	Median	-62.69186869
wavelet-LLH	firstorder	Energy	3724061.226
wavelet-LLH	firstorder	RobustMeanAbsoluteDeviation	90.94953446
wavelet-LLH	firstorder	MeanAbsoluteDeviation	129.2077661
wavelet-LLH	firstorder	TotalEnergy	100549653.1
wavelet-LLH	firstorder	Maximum	359.1560923
wavelet-LLH	firstorder	RootMeanSquared	163.6820313
wavelet-LLH	firstorder	90Percentile	172.7537353
wavelet-LLH	firstorder	Minimum	-406.38519
wavelet-LLH	firstorder	Entropy	4.592167234
wavelet-LLH	firstorder	Range	765.5412823
wavelet-LLH	firstorder	Variance	25204.59825

wavelet-LLH	firstorder	10Percentile	-231.7908983
wavelet-LLH	firstorder	Kurtosis	2.70071891
wavelet-LLH	firstorder	Mean	-39.83979331
wavelet-LLH	glrlm	ShortRunLowGrayLevelEmphasis	0.015293666
wavelet-LLH	glrlm	GrayLevelVariance	41.22902085
wavelet-LLH	glrlm	LowGrayLevelRunEmphasis	0.015424173
wavelet-LLH	glrlm	GrayLevelNonUniformityNormalized	0.048265837
wavelet-LLH	glrlm	RunVariance	0.028226596
wavelet-LLH	glrlm	GrayLevelNonUniformity	6.527682904
wavelet-LLH	glrlm	LongRunEmphasis	1.085072339
wavelet-LLH	glrlm	ShortRunHighGrayLevelEmphasis	291.4599856
wavelet-LLH	glrlm	RunLengthNonUniformity	128.075203
wavelet-LLH	glrlm	ShortRunEmphasis	0.979365027
wavelet-LLH	glrlm	LongRunHighGrayLevelEmphasis	315.8186864
wavelet-LLH	glrlm	RunPercentage	0.972883232
wavelet-LLH	glrlm	LongRunLowGrayLevelEmphasis	0.015961189
wavelet-LLH	glrlm	RunEntropy	4.712950981
wavelet-LLH	glrlm	HighGrayLevelRunEmphasis	296.246129
wavelet-LLH	glrlm	RunLengthNonUniformityNormalized	0.946889637
wavelet-LLH	glszm	GrayLevelVariance	49.44352617
wavelet-LLH	glszm	ZoneVariance	1.230690746
wavelet-LLH	glszm	GrayLevelNonUniformityNormalized	0.042342618
wavelet-LLH	glszm	SizeZoneNonUniformityNormalized	0.653504744
wavelet-LLH	glszm	SizeZoneNonUniformity	64.6969697
wavelet-LLH	glszm	GrayLevelNonUniformity	4.191919192
wavelet-LLH	glszm	LargeAreaEmphasis	3.202020202
wavelet-LLH	glszm	SmallAreaHighGrayLevelEmphasis	271.982033
wavelet-LLH	glszm	ZonePercentage	0.712230216
wavelet-LLH	glszm	LargeAreaLowGrayLevelEmphasis	0.031998015
wavelet-LLH	glszm	LargeAreaHighGrayLevelEmphasis	799.2424242
wavelet-LLH	glszm	HighGrayLevelZoneEmphasis	317.2121212
wavelet-LLH	glszm	SmallAreaEmphasis	0.834171491
wavelet-LLH	glszm	LowGrayLevelZoneEmphasis	0.018644447
wavelet-LLH	glszm	ZoneEntropy	5.357764989
wavelet-LLH	glszm	SmallAreaLowGrayLevelEmphasis	0.017400397
wavelet-LLH	ngtdm	Coarseness	0.0447769
wavelet-LLH	ngtdm	Complexity	1511.508838
wavelet-LLH	ngtdm	Strength	12.42429285
wavelet-LLH	ngtdm	Contrast	0.383688028
wavelet-LLH	ngtdm	Busyness	0.055724119
wavelet-LLL	gldm	GrayLevelVariance	393.3218777
wavelet-LLL	gldm	HighGrayLevelEmphasis	2238.611511
wavelet-LLL	gldm	DependenceEntropy	6.033810947
wavelet-LLL	gldm	DependenceNonUniformity	75.77697842
wavelet-LLL	gldm	GrayLevelNonUniformity	2.726618705
wavelet-LLL	gldm	SmallDependenceEmphasis	0.763389289
wavelet-LLL	gldm	SmallDependenceHighGrayLevelEmphasis	1544.923261
wavelet-LLL	gldm	DependenceNonUniformityNormalized	0.545158118
wavelet-LLL	gldm	LargeDependenceEmphasis	2.26618705
wavelet-LLL	gldm	LargeDependenceLowGrayLevelEmphasis	0.009927323
wavelet-LLL	gldm	DependenceVariance	0.378034263
wavelet-LLL	gldm	LargeDependenceHighGrayLevelEmphasis	6491.446043
wavelet-LLL	gldm	SmallDependenceLowGrayLevelEmphasis	0.008130277
wavelet-LLL	gldm	LowGrayLevelEmphasis	0.008470201
wavelet-LLL	glcm	JointAverage	48.67487258

wavelet-LLL	glcm	SumAverage	97.34974516
wavelet-LLL	glcm	JointEntropy	7.425164939
wavelet-LLL	glcm	ClusterShade	-1322.658753
wavelet-LLL	glcm	MaximumProbability	0.011209192
wavelet-LLL	glcm	Idmn	0.936463995
wavelet-LLL	glcm	JointEnergy	0.005948415
wavelet-LLL	glcm	Contrast	466.8719554
wavelet-LLL	glcm	DifferenceEntropy	4.969865737
wavelet-LLL	glcm	InverseVariance	0.056392178
wavelet-LLL	glcm	DifferenceVariance	146.940305
wavelet-LLL	glcm	Idn	0.831369775
wavelet-LLL	glcm	Idm	0.058056569
wavelet-LLL	glcm	Correlation	0.380183118
wavelet-LLL	glcm	Autocorrelation	2517.681615
wavelet-LLL	glcm	SumEntropy	5.896989154
wavelet-LLL	glcm	SumSquares	380.3456533
wavelet-LLL	glcm	ClusterProminence	2312811.052
wavelet-LLL	glcm	Imc2	0.999819547
wavelet-LLL	glcm	Imc1	-0.697657453
wavelet-LLL	glcm	DifferenceAverage	17.56092167
wavelet-LLL	glcm	Id	0.120530054
wavelet-LLL	glcm	ClusterTendency	1054.510658
wavelet-LLL	firstorder	InterquartileRange	778.0407519
wavelet-LLL	firstorder	Skewness	0.132670183
wavelet-LLL	firstorder	Uniformity	0.019615962
wavelet-LLL	firstorder	Median	-692.8060958
wavelet-LLL	firstorder	Energy	99987221.59
wavelet-LLL	firstorder	RobustMeanAbsoluteDeviation	330.0690545
wavelet-LLL	firstorder	MeanAbsoluteDeviation	423.8517822
wavelet-LLL	firstorder	TotalEnergy	2699654983
wavelet-LLL	firstorder	Maximum	206.3070261
wavelet-LLL	firstorder	RootMeanSquared	848.1347354
wavelet-LLL	firstorder	90Percentile	57.60829707
wavelet-LLL	firstorder	Minimum	-1740.123131
wavelet-LLL	firstorder	Entropy	5.813457058
wavelet-LLL	firstorder	Range	1946.430158
wavelet-LLL	firstorder	Variance	244915.1051
wavelet-LLL	firstorder	10Percentile	-1297.995982
wavelet-LLL	firstorder	Kurtosis	1.971022195
wavelet-LLL	firstorder	Mean	-688.7796631
wavelet-LLL	glrlm	ShortRunLowGrayLevelEmphasis	0.008562465
wavelet-LLL	glrlm	GrayLevelVariance	389.4655641
wavelet-LLL	glrlm	LowGrayLevelRunEmphasis	0.008576435
wavelet-LLL	glrlm	GrayLevelNonUniformityNormalized	0.019632515
wavelet-LLL	glrlm	RunVariance	0.01437798
wavelet-LLL	glrlm	GrayLevelNonUniformity	2.689670968
wavelet-LLL	glrlm	LongRunEmphasis	1.044223175
wavelet-LLL	glrlm	ShortRunHighGrayLevelEmphasis	2189.16085
wavelet-LLL	glrlm	RunLengthNonUniformity	133.0980143
wavelet-LLL	glrlm	ShortRunEmphasis	0.988944206
wavelet-LLL	glrlm	LongRunHighGrayLevelEmphasis	2365.839773
wavelet-LLL	glrlm	RunPercentage	0.985611511
wavelet-LLL	glrlm	LongRunLowGrayLevelEmphasis	0.008632313
wavelet-LLL	glrlm	RunEntropy	5.838991665
wavelet-LLL	glrlm	HighGrayLevelRunEmphasis	2224.496635

wavelet-LLL	glrlm	RunLengthNonUniformityNormalized	0.971244041
wavelet-LLL	glszm	GrayLevelVariance	345.9473098
wavelet-LLL	glszm	ZoneVariance	0.227928062
wavelet-LLL	glszm	GrayLevelNonUniformityNormalized	0.020065398
wavelet-LLL	glszm	SizeZoneNonUniformityNormalized	0.7171522
wavelet-LLL	glszm	SizeZoneNonUniformity	83.18965517
wavelet-LLL	glszm	GrayLevelNonUniformity	2.327586207
wavelet-LLL	glszm	LargeAreaEmphasis	1.663793103
wavelet-LLL	glszm	SmallAreaHighGrayLevelEmphasis	1740.860153
wavelet-LLL	glszm	ZonePercentage	0.834532374
wavelet-LLL	glszm	LargeAreaLowGrayLevelEmphasis	0.010733294
wavelet-LLL	glszm	LargeAreaHighGrayLevelEmphasis	4131.008621
wavelet-LLL	glszm	HighGrayLevelZoneEmphasis	2096.215517
wavelet-LLL	glszm	SmallAreaEmphasis	0.8723659
wavelet-LLL	glszm	LowGrayLevelZoneEmphasis	0.00987295
wavelet-LLL	glszm	ZoneEntropy	5.973770435
wavelet-LLL	glszm	SmallAreaLowGrayLevelEmphasis	0.009674688
wavelet-LLL	ngtdm	Coarseness	0.033170648
wavelet-LLL	ngtdm	Complexity	20082.14556
wavelet-LLL	ngtdm	Strength	63.95470051
wavelet-LLL	ngtdm	Contrast	2.340363358
wavelet-LLL	ngtdm	Busyness	0.015316203
wavelet-HHL	gldm	GrayLevelVariance	20.56084054
wavelet-HHL	gldm	HighGrayLevelEmphasis	204.8561151
wavelet-HHL	gldm	DependenceEntropy	5.579995727
wavelet-HHL	gldm	DependenceNonUniformity	38.00719424
wavelet-HHL	gldm	GrayLevelNonUniformity	9.949640288
wavelet-HHL	gldm	SmallDependenceEmphasis	0.45675786
wavelet-HHL	gldm	SmallDependenceHighGrayLevelEmphasis	94.17415505
wavelet-HHL	gldm	DependenceNonUniformityNormalized	0.273433052
wavelet-HHL	gldm	LargeDependenceEmphasis	7.73381295
wavelet-HHL	gldm	LargeDependenceLowGrayLevelEmphasis	0.109270948
wavelet-HHL	gldm	DependenceVariance	2.400703897
wavelet-HHL	gldm	LargeDependenceHighGrayLevelEmphasis	1518.640288
wavelet-HHL	gldm	SmallDependenceLowGrayLevelEmphasis	0.009790809
wavelet-HHL	gldm	LowGrayLevelEmphasis	0.024718479
wavelet-HHL	glcm	JointAverage	13.54841391
wavelet-HHL	glcm	SumAverage	27.09682782
wavelet-HHL	glcm	JointEntropy	6.698718853
wavelet-HHL	glcm	ClusterShade	-54.58393918
wavelet-HHL	glcm	MaximumProbability	0.032148718
wavelet-HHL	glcm	Idmn	0.944580556
wavelet-HHL	glcm	JointEnergy	0.011464655
wavelet-HHL	glcm	Contrast	38.98639258
wavelet-HHL	glcm	DifferenceEntropy	3.552655879
wavelet-HHL	glcm	InverseVariance	0.217212569
wavelet-HHL	glcm	DifferenceVariance	15.84347492
wavelet-HHL	glcm	Idn	0.850019434
wavelet-HHL	glcm	Idm	0.207397246
wavelet-HHL	glcm	Correlation	-0.031325889
wavelet-HHL	glcm	Autocorrelation	182.9105007
wavelet-HHL	glcm	SumEntropy	4.308383073
wavelet-HHL	glcm	SumSquares	18.81220592
wavelet-HHL	glcm	ClusterProminence	4721.329793
wavelet-HHL	glcm	Imc2	0.961521266

wavelet-HHL	glcm	Imc1	-0.324829549
wavelet-HHL	glcm	DifferenceAverage	4.73627589
wavelet-HHL	glcm	Id	0.297382431
wavelet-HHL	glcm	ClusterTendency	36.26243109
wavelet-HHL	firstorder	InterquartileRange	140.3900628
wavelet-HHL	firstorder	Skewness	-0.295718294
wavelet-HHL	firstorder	Uniformity	0.071580146
wavelet-HHL	firstorder	Median	2.127199373
wavelet-HHL	firstorder	Energy	1783835.55
wavelet-HHL	firstorder	RobustMeanAbsoluteDeviation	56.3972431
wavelet-HHL	firstorder	MeanAbsoluteDeviation	86.44505292
wavelet-HHL	firstorder	TotalEnergy	48163559.84
wavelet-HHL	firstorder	Maximum	256.3135943
wavelet-HHL	firstorder	RootMeanSquared	113.2843735
wavelet-HHL	firstorder	90Percentile	147.3534176
wavelet-HHL	firstorder	Minimum	-317.3435704
wavelet-HHL	firstorder	Entropy	4.099367214
wavelet-HHL	firstorder	Range	573.6571647
wavelet-HHL	firstorder	Variance	12831.42966
wavelet-HHL	firstorder	10Percentile	-130.2872541
wavelet-HHL	firstorder	Kurtosis	3.264561327
wavelet-HHL	firstorder	Mean	1.385503146
wavelet-HHL	glrlm	ShortRunLowGrayLevelEmphasis	0.024341032
wavelet-HHL	glrlm	GrayLevelVariance	21.18208476
wavelet-HHL	glrlm	LowGrayLevelRunEmphasis	0.025065966
wavelet-HHL	glrlm	GrayLevelNonUniformityNormalized	0.069404534
wavelet-HHL	glrlm	RunVariance	0.053708402
wavelet-HHL	glrlm	GrayLevelNonUniformity	9.161669333
wavelet-HHL	glrlm	LongRunEmphasis	1.163925603
wavelet-HHL	glrlm	ShortRunHighGrayLevelEmphasis	197.5963164
wavelet-HHL	glrlm	RunLengthNonUniformity	119.2273172
wavelet-HHL	glrlm	ShortRunEmphasis	0.961002454
wavelet-HHL	glrlm	LongRunHighGrayLevelEmphasis	237.6116721
wavelet-HHL	glrlm	RunPercentage	0.949640288
wavelet-HHL	glrlm	LongRunLowGrayLevelEmphasis	0.028004843
wavelet-HHL	glrlm	RunEntropy	4.336114854
wavelet-HHL	glrlm	HighGrayLevelRunEmphasis	205.2717479
wavelet-HHL	glrlm	RunLengthNonUniformityNormalized	0.902563075
wavelet-HHL	glszm	GrayLevelVariance	27.23359757
wavelet-HHL	glszm	ZoneVariance	2.910102884
wavelet-HHL	glszm	GrayLevelNonUniformityNormalized	0.056839265
wavelet-HHL	glszm	SizeZoneNonUniformityNormalized	0.447124304
wavelet-HHL	glszm	SizeZoneNonUniformity	34.42857143
wavelet-HHL	glszm	GrayLevelNonUniformity	4.376623377
wavelet-HHL	glszm	LargeAreaEmphasis	6.168831169
wavelet-HHL	glszm	SmallAreaHighGrayLevelEmphasis	142.089228
wavelet-HHL	glszm	ZonePercentage	0.553956835
wavelet-HHL	glszm	LargeAreaLowGrayLevelEmphasis	0.097143563
wavelet-HHL	glszm	LargeAreaHighGrayLevelEmphasis	1224.766234
wavelet-HHL	glszm	HighGrayLevelZoneEmphasis	207.9090909
wavelet-HHL	glszm	SmallAreaEmphasis	0.690758478
wavelet-HHL	glszm	LowGrayLevelZoneEmphasis	0.025785302
wavelet-HHL	glszm	ZoneEntropy	5.237121443
wavelet-HHL	glszm	SmallAreaLowGrayLevelEmphasis	0.013339339
wavelet-HHL	ngtdm	Coarseness	0.042780471

wavelet-HHL	ngtdm	Complexity	766.2850782
wavelet-HHL	ngtdm	Strength	6.515640628
wavelet-HHL	ngtdm	Contrast	0.27493135
wavelet-HHL	ngtdm	Busyness	0.070434556
wavelet-HLH	gldm	GrayLevelVariance	21.44920035
wavelet-HLH	gldm	HighGrayLevelEmphasis	234.5251799
wavelet-HLH	gldm	DependenceEntropy	5.496357937
wavelet-HLH	gldm	DependenceNonUniformity	42.42446043
wavelet-HLH	gldm	GrayLevelNonUniformity	9.690647482
wavelet-HLH	gldm	SmallDependenceEmphasis	0.47251199
wavelet-HLH	gldm	SmallDependenceHighGrayLevelEmphasis	100.0940927
wavelet-HLH	gldm	DependenceNonUniformityNormalized	0.305211946
wavelet-HLH	gldm	LargeDependenceEmphasis	5.618705036
wavelet-HLH	gldm	LargeDependenceLowGrayLevelEmphasis	0.038066455
wavelet-HLH	gldm	DependenceVariance	1.295895658
wavelet-HLH	gldm	LargeDependenceHighGrayLevelEmphasis	1413.359712
wavelet-HLH	gldm	SmallDependenceLowGrayLevelEmphasis	0.01413622
wavelet-HLH	gldm	LowGrayLevelEmphasis	0.01673819
wavelet-HLH	glcm	JointAverage	14.65971838
wavelet-HLH	glcm	SumAverage	29.31943676
wavelet-HLH	glcm	JointEntropy	6.79126655
wavelet-HLH	glcm	ClusterShade	-59.23664723
wavelet-HLH	glcm	MaximumProbability	0.025499024
wavelet-HLH	glcm	Idmn	0.953742046
wavelet-HLH	glcm	JointEnergy	0.010331373
wavelet-HLH	glcm	Contrast	45.54622258
wavelet-HLH	glcm	DifferenceEntropy	3.659023156
wavelet-HLH	glcm	InverseVariance	0.18240524
wavelet-HLH	glcm	DifferenceVariance	17.03740419
wavelet-HLH	glcm	Idn	0.858438978
wavelet-HLH	glcm	Idm	0.177123167
wavelet-HLH	glcm	Correlation	-0.038514568
wavelet-HLH	glcm	Autocorrelation	214.0396022
wavelet-HLH	glcm	SumEntropy	4.403856721
wavelet-HLH	glcm	SumSquares	21.89097274
wavelet-HLH	glcm	ClusterProminence	6146.031907
wavelet-HLH	glcm	Imc2	0.96131554
wavelet-HLH	glcm	Imc1	-0.320623047
wavelet-HLH	glcm	DifferenceAverage	5.248861987
wavelet-HLH	glcm	Id	0.270284994
wavelet-HLH	glcm	ClusterTendency	42.01766839
wavelet-HLH	firstorder	InterquartileRange	158.0928037
wavelet-HLH	firstorder	Skewness	-0.17175611
wavelet-HLH	firstorder	Uniformity	0.069716888
wavelet-HLH	firstorder	Median	3.509490703
wavelet-HLH	firstorder	Energy	1864447.206
wavelet-HLH	firstorder	RobustMeanAbsoluteDeviation	66.64011876
wavelet-HLH	firstorder	MeanAbsoluteDeviation	92.99046751
wavelet-HLH	firstorder	TotalEnergy	50340074.57
wavelet-HLH	firstorder	Maximum	351.6022318
wavelet-HLH	firstorder	RootMeanSquared	115.8157556
wavelet-HLH	firstorder	90Percentile	147.3233538
wavelet-HLH	firstorder	Minimum	-340.5875701
wavelet-HLH	firstorder	Entropy	4.051964161
wavelet-HLH	firstorder	Range	692.1898019

wavelet-HLH	firstorder	Variance	13410.59451
wavelet-HLH	firstorder	10Percentile	-134.7318019
wavelet-HLH	firstorder	Kurtosis	3.269164651
wavelet-HLH	firstorder	Mean	1.641566283
wavelet-HLH	glrlm	ShortRunLowGrayLevelEmphasis	0.017112574
wavelet-HLH	glrlm	GrayLevelVariance	21.86482426
wavelet-HLH	glrlm	LowGrayLevelRunEmphasis	0.017259821
wavelet-HLH	glrlm	GrayLevelNonUniformityNormalized	0.069196257
wavelet-HLH	glrlm	RunVariance	0.047203411
wavelet-HLH	glrlm	GrayLevelNonUniformity	9.219207129
wavelet-HLH	glrlm	LongRunEmphasis	1.136111838
wavelet-HLH	glrlm	ShortRunHighGrayLevelEmphasis	225.7801919
wavelet-HLH	glrlm	RunLengthNonUniformity	122.9154094
wavelet-HLH	glrlm	ShortRunEmphasis	0.969190721
wavelet-HLH	glrlm	LongRunHighGrayLevelEmphasis	268.5387763
wavelet-HLH	glrlm	RunPercentage	0.958494743
wavelet-HLH	glrlm	LongRunLowGrayLevelEmphasis	0.017898645
wavelet-HLH	glrlm	RunEntropy	4.229200436
wavelet-HLH	glrlm	HighGrayLevelRunEmphasis	233.6109319
wavelet-HLH	glrlm	RunLengthNonUniformityNormalized	0.922339662
wavelet-HLH	glszm	GrayLevelVariance	27.26758711
wavelet-HLH	glszm	ZoneVariance	1.862754767
wavelet-HLH	glszm	GrayLevelNonUniformityNormalized	0.063445102
wavelet-HLH	glszm	SizeZoneNonUniformityNormalized	0.440828402
wavelet-HLH	glszm	SizeZoneNonUniformity	34.38461538
wavelet-HLH	glszm	GrayLevelNonUniformity	4.948717949
wavelet-HLH	glszm	LargeAreaEmphasis	5.038461538
wavelet-HLH	glszm	SmallAreaHighGrayLevelEmphasis	138.5956088
wavelet-HLH	glszm	ZonePercentage	0.561151079
wavelet-HLH	glszm	LargeAreaLowGrayLevelEmphasis	0.044632543
wavelet-HLH	glszm	LargeAreaHighGrayLevelEmphasis	1260.730769
wavelet-HLH	glszm	HighGrayLevelZoneEmphasis	216.1538462
wavelet-HLH	glszm	SmallAreaEmphasis	0.688803201
wavelet-HLH	glszm	LowGrayLevelZoneEmphasis	0.026080467
wavelet-HLH	glszm	ZoneEntropy	5.256162797
wavelet-HLH	glszm	SmallAreaLowGrayLevelEmphasis	0.024438334
wavelet-HLH	ngtdm	Coarseness	0.034422025
wavelet-HLH	ngtdm	Complexity	772.3612481
wavelet-HLH	ngtdm	Strength	5.85213285
wavelet-HLH	ngtdm	Contrast	0.364357035
wavelet-HLH	ngtdm	Busyness	0.099652344

Image type	Feature Class	Feature Name	502: RTSTRUCT: C99-DNU_segment_GTVp_RUL
general	info	BoundingBox	(1, 0, 1, 5, 6, 4)
general	info	EnabledImageTypes	{u'Original': {}, u'Wavelet': {}}
general	info	GeneralSettings	{'distances': [1], 'additionalInfo': True, 'force2D': False, 'interpolator': 'sitkBSpline', 'resampledPixelSpacing': [3.0], 'label': 1, 'normalizeScale': 1, 'normalize': False, 'force2Ddimension': 0, 'removeOutliers': None, 'minimumROISize': None, u'binWidth': 25.0, 'minimumROIDimensions': 1, u'correctMask': True, 'preCrop': False, u'symmetricalGLCM': True, 'resegmentRange': None, 'padDistance': 5}
general	info	ImageHash	865f4fff9208af09a0a146636c02c83a725d85a8
general	info	ImageSpacing	(0.9765625000000002, 0.9765625000000002, 2.0)
general	info	MaskHash	8b6f86499f2b37e5c2cda4ea1ed85c80f9a28d3a
general	info	NumpyVersion	1.13.1
general	info	PyWaveletVersion	0.5.2
general	info	SimpleITKVersion	1.1.0
general	info	Version	1.3.0.post102+g1b0eb74
general	info	VolumeNum	1
general	info	VoxelNum	63
original	shape	Maximum3DDiameter	18
original	shape	Maximum2DDiameterSlice	15
original	shape	Sphericity	0.899432551
original	shape	MinorAxis	12.84316708
original	shape	Elongation	0.814706374
original	shape	SurfaceVolumeRatio	0.450416286
original	shape	Volume	1701
original	shape	MajorAxis	15.76416669
original	shape	SurfaceArea	766.1581033
original	shape	Flatness	0.772747855
original	shape	LeastAxis	12.18172599
original	shape	Maximum2DDiameterColumn	15
original	shape	Maximum2DDiameterRow	13.41640786
original	gldm	GrayLevelVariance	76.33308138
original	gldm	HighGrayLevelEmphasis	476.968254
original	gldm	DependenceEntropy	4.875945352
original	gldm	DependenceNonUniformity	26.74603175
original	gldm	GrayLevelNonUniformity	2.936507937
original	gldm	SmallDependenceEmphasis	0.670943563
original	gldm	SmallDependenceHighGrayLevelEmphasis	259.7465608
original	gldm	DependenceNonUniformityNormalized	0.424540186
original	gldm	LargeDependenceEmphasis	4.555555556
original	gldm	LargeDependenceLowGrayLevelEmphasis	0.031541208
original	gldm	DependenceVariance	1.451247166
original	gldm	LargeDependenceHighGrayLevelEmphasis	3281.920635
original	gldm	SmallDependenceLowGrayLevelEmphasis	0.020268254
original	gldm	LowGrayLevelEmphasis	0.021433441
original	glcm	JointAverage	22.15959586
original	glcm	SumAverage	44.31919171
original	glcm	JointEntropy	5.911916588
original	glcm	ClusterShade	-554.1411611

original	glcm	MaximumProbability	0.031163934
original	glcm	Idmn	0.903768177
original	glcm	JointEnergy	0.017487563
original	glcm	Contrast	142.1366756
original	glcm	DifferenceEntropy	3.926792204
original	glcm	InverseVariance	0.122944174
original	glcm	DifferenceVariance	46.23137556
original	glcm	Idn	0.800111881
original	glcm	Idm	0.122626781
original	glcm	Correlation	0.055853908
original	glcm	Autocorrelation	495.3521278
original	glcm	SumEntropy	4.484437008
original	glcm	SumSquares	74.84154815
original	glcm	ClusterProminence	61219.60992
original	glcm	Imc2	0.998089345
original	glcm	Imc1	-0.64372656
original	glcm	DifferenceAverage	9.58310381
original	glcm	Id	0.19838695
original	glcm	ClusterTendency	157.229517
original	firstorder	InterquartileRange	381
original	firstorder	Skewness	-0.126828055
original	firstorder	Uniformity	0.046611237
original	firstorder	Median	-263
original	firstorder	Energy	6535661
original	firstorder	RobustMeanAbsoluteDeviation	149.6101624
original	firstorder	MeanAbsoluteDeviation	188.6606198
original	firstorder	TotalEnergy	176462847
original	firstorder	Maximum	103
original	firstorder	RootMeanSquared	322.0879551
original	firstorder	90Percentile	36.8
original	firstorder	Minimum	-711
original	firstorder	Entropy	4.59642725
original	firstorder	Range	814
original	firstorder	Variance	47511.44419
original	firstorder	10Percentile	-523
original	firstorder	Kurtosis	1.88939294
original	firstorder	Mean	-237.1269841
original	glrlm	ShortRunLowGrayLevelEmphasis	0.021926661
original	glrlm	GrayLevelVariance	75.43408179
original	glrlm	LowGrayLevelRunEmphasis	0.022003167
original	glrlm	GrayLevelNonUniformityNormalized	0.045580145
original	glrlm	RunVariance	0.029109116
original	glrlm	GrayLevelNonUniformity	2.789106724
original	glrlm	LongRunEmphasis	1.094021345
original	glrlm	ShortRunHighGrayLevelEmphasis	451.6762159
original	glrlm	RunLengthNonUniformity	57.71812726
original	glrlm	ShortRunEmphasis	0.976494664
original	glrlm	LongRunHighGrayLevelEmphasis	537.9057237
original	glrlm	RunPercentage	0.970695971
original	glrlm	LongRunLowGrayLevelEmphasis	0.022309189
original	glrlm	RunEntropy	4.690680378
original	glrlm	HighGrayLevelRunEmphasis	468.9221175
original	glrlm	RunLengthNonUniformityNormalized	0.941781768
original	glszm	GrayLevelVariance	67.68764147
original	glszm	ZoneVariance	0.777727478

original	glszm	GrayLevelNonUniformityNormalized	0.043005885
original	glszm	SizeZoneNonUniformityNormalized	0.672249887
original	glszm	SizeZoneNonUniformity	31.59574468
original	glszm	GrayLevelNonUniformity	2.021276596
original	glszm	LargeAreaEmphasis	2.574468085
original	glszm	SmallAreaHighGrayLevelEmphasis	313.3095981
original	glszm	ZonePercentage	0.746031746
original	glszm	LargeAreaLowGrayLevelEmphasis	0.032264556
original	glszm	LargeAreaHighGrayLevelEmphasis	1583.085106
original	glszm	HighGrayLevelZoneEmphasis	406.4042553
original	glszm	SmallAreaEmphasis	0.844491726
original	glszm	LowGrayLevelZoneEmphasis	0.027569749
original	glszm	ZoneEntropy	4.809492043
original	glszm	SmallAreaLowGrayLevelEmphasis	0.026996168
original	ngtdm	Coarseness	0.047790728
original	ngtdm	Complexity	2550.905395
original	ngtdm	Strength	19.83043694
original	ngtdm	Contrast	1.467427198
original	ngtdm	Busyness	0.045154735
wavelet-HHH	gldm	GrayLevelVariance	10.53061224
wavelet-HHH	gldm	HighGrayLevelEmphasis	99.42857143
wavelet-HHH	gldm	DependenceEntropy	4.677023623
wavelet-HHH	gldm	DependenceNonUniformity	17.57142857
wavelet-HHH	gldm	GrayLevelNonUniformity	6.492063492
wavelet-HHH	gldm	SmallDependenceEmphasis	0.447588183
wavelet-HHH	gldm	SmallDependenceHighGrayLevelEmphasis	42.17615961
wavelet-HHH	gldm	DependenceNonUniformityNormalized	0.278911565
wavelet-HHH	gldm	LargeDependenceEmphasis	5.73015873
wavelet-HHH	gldm	LargeDependenceLowGrayLevelEmphasis	0.095680729
wavelet-HHH	gldm	DependenceVariance	1.138321995
wavelet-HHH	gldm	LargeDependenceHighGrayLevelEmphasis	565.968254
wavelet-HHH	gldm	SmallDependenceLowGrayLevelEmphasis	0.024952558
wavelet-HHH	gldm	LowGrayLevelEmphasis	0.033386331
wavelet-HHH	glcm	JointAverage	9.439107811
wavelet-HHH	glcm	SumAverage	18.87821562
wavelet-HHH	glcm	JointEntropy	5.596149832
wavelet-HHH	glcm	ClusterShade	-17.81023586
wavelet-HHH	glcm	MaximumProbability	0.046201062
wavelet-HHH	glcm	Idmn	0.927717888
wavelet-HHH	glcm	JointEnergy	0.022961591
wavelet-HHH	glcm	Contrast	22.77769117
wavelet-HHH	glcm	DifferenceEntropy	3.086057691
wavelet-HHH	glcm	InverseVariance	0.231197397
wavelet-HHH	glcm	DifferenceVariance	7.966752798
wavelet-HHH	glcm	Idn	0.823203735
wavelet-HHH	glcm	Idm	0.220359005
wavelet-HHH	glcm	Correlation	-0.044322884
wavelet-HHH	glcm	Autocorrelation	88.66259764
wavelet-HHH	glcm	SumEntropy	3.711445899
wavelet-HHH	glcm	SumSquares	10.92654727
wavelet-HHH	glcm	ClusterProminence	1279.481801
wavelet-HHH	glcm	Imc2	0.966202679
wavelet-HHH	glcm	Imc1	-0.39688083
wavelet-HHH	glcm	DifferenceAverage	3.81364353
wavelet-HHH	glcm	Id	0.315784973

wavelet-HHH	glcm	ClusterTendency	20.92849793
wavelet-HHH	firstorder	InterquartileRange	111.2367759
wavelet-HHH	firstorder	Skewness	-0.210767316
wavelet-HHH	firstorder	Uniformity	0.103048627
wavelet-HHH	firstorder	Median	-5.835692177
wavelet-HHH	firstorder	Energy	426675.5983
wavelet-HHH	firstorder	RobustMeanAbsoluteDeviation	42.40243483
wavelet-HHH	firstorder	MeanAbsoluteDeviation	64.77471002
wavelet-HHH	firstorder	TotalEnergy	11520241.15
wavelet-HHH	firstorder	Maximum	174.9208109
wavelet-HHH	firstorder	RootMeanSquared	82.29598134
wavelet-HHH	firstorder	90Percentile	104.5190666
wavelet-HHH	firstorder	Minimum	-214.5076378
wavelet-HHH	firstorder	Entropy	3.532692705
wavelet-HHH	firstorder	Range	389.4284488
wavelet-HHH	firstorder	Variance	6767.579326
wavelet-HHH	firstorder	10Percentile	-116.7552392
wavelet-HHH	firstorder	Kurtosis	2.840237554
wavelet-HHH	firstorder	Mean	-2.247046588
wavelet-HHH	glrlm	ShortRunLowGrayLevelEmphasis	0.033808077
wavelet-HHH	glrlm	GrayLevelVariance	10.78810182
wavelet-HHH	glrlm	LowGrayLevelRunEmphasis	0.034294847
wavelet-HHH	glrlm	GrayLevelNonUniformityNormalized	0.100954558
wavelet-HHH	glrlm	RunVariance	0.043766363
wavelet-HHH	glrlm	GrayLevelNonUniformity	6.081103312
wavelet-HHH	glrlm	LongRunEmphasis	1.139956235
wavelet-HHH	glrlm	ShortRunHighGrayLevelEmphasis	95.86646718
wavelet-HHH	glrlm	RunLengthNonUniformity	55.03200803
wavelet-HHH	glrlm	ShortRunEmphasis	0.965010941
wavelet-HHH	glrlm	LongRunHighGrayLevelEmphasis	113.4392967
wavelet-HHH	glrlm	RunPercentage	0.956043956
wavelet-HHH	glrlm	LongRunLowGrayLevelEmphasis	0.036241924
wavelet-HHH	glrlm	RunEntropy	3.71815838
wavelet-HHH	glrlm	HighGrayLevelRunEmphasis	99.38103307
wavelet-HHH	glrlm	RunLengthNonUniformityNormalized	0.912467274
wavelet-HHH	glszm	GrayLevelVariance	14.71366782
wavelet-HHH	glszm	ZoneVariance	3.360726644
wavelet-HHH	glszm	GrayLevelNonUniformityNormalized	0.089965398
wavelet-HHH	glszm	SizeZoneNonUniformityNormalized	0.446366782
wavelet-HHH	glszm	SizeZoneNonUniformity	15.17647059
wavelet-HHH	glszm	GrayLevelNonUniformity	3.058823529
wavelet-HHH	glszm	LargeAreaEmphasis	6.794117647
wavelet-HHH	glszm	SmallAreaHighGrayLevelEmphasis	63.18472155
wavelet-HHH	glszm	ZonePercentage	0.53968254
wavelet-HHH	glszm	LargeAreaLowGrayLevelEmphasis	0.125728141
wavelet-HHH	glszm	LargeAreaHighGrayLevelEmphasis	630.5882353
wavelet-HHH	glszm	HighGrayLevelZoneEmphasis	98.38235294
wavelet-HHH	glszm	SmallAreaEmphasis	0.68549634
wavelet-HHH	glszm	LowGrayLevelZoneEmphasis	0.049438051
wavelet-HHH	glszm	ZoneEntropy	4.359377915
wavelet-HHH	glszm	SmallAreaLowGrayLevelEmphasis	0.043919628
wavelet-HHH	ngtdm	Coarseness	0.077418174
wavelet-HHH	ngtdm	Complexity	229.6572407
wavelet-HHH	ngtdm	Strength	5.225343329
wavelet-HHH	ngtdm	Contrast	0.278193942

wavelet-HHH	ngtdm	Busyness	0.100912997
wavelet-HLL	gldm	GrayLevelVariance	135.3716301
wavelet-HLL	gldm	HighGrayLevelEmphasis	453.6825397
wavelet-HLL	gldm	DependenceEntropy	4.988951947
wavelet-HLL	gldm	DependenceNonUniformity	30.3968254
wavelet-HLL	gldm	GrayLevelNonUniformity	2.777777778
wavelet-HLL	gldm	SmallDependenceEmphasis	0.712191358
wavelet-HLL	gldm	SmallDependenceHighGrayLevelEmphasis	314.707231
wavelet-HLL	gldm	DependenceNonUniformityNormalized	0.482489292
wavelet-HLL	gldm	LargeDependenceEmphasis	2.682539683
wavelet-HLL	gldm	LargeDependenceLowGrayLevelEmphasis	0.09147105
wavelet-HLL	gldm	DependenceVariance	0.503401361
wavelet-HLL	gldm	LargeDependenceHighGrayLevelEmphasis	1108.031746
wavelet-HLL	gldm	SmallDependenceLowGrayLevelEmphasis	0.043436616
wavelet-HLL	gldm	LowGrayLevelEmphasis	0.048951709
wavelet-HLL	glcm	JointAverage	16.47516204
wavelet-HLL	glcm	SumAverage	32.95032408
wavelet-HLL	glcm	JointEntropy	5.937992106
wavelet-HLL	glcm	ClusterShade	4143.419692
wavelet-HLL	glcm	MaximumProbability	0.030991906
wavelet-HLL	glcm	Idmn	0.936049867
wavelet-HLL	glcm	JointEnergy	0.017064451
wavelet-HLL	glcm	Contrast	141.913005
wavelet-HLL	glcm	DifferenceEntropy	3.869348247
wavelet-HLL	glcm	InverseVariance	0.130149763
wavelet-HLL	glcm	DifferenceVariance	44.68006528
wavelet-HLL	glcm	Idn	0.834805827
wavelet-HLL	glcm	Idm	0.109974442
wavelet-HLL	glcm	Correlation	0.427757441
wavelet-HLL	glcm	Autocorrelation	329.7464307
wavelet-HLL	glcm	SumEntropy	4.513152025
wavelet-HLL	glcm	SumSquares	128.9704107
wavelet-HLL	glcm	ClusterProminence	336315.7923
wavelet-HLL	glcm	Imc2	0.998619872
wavelet-HLL	glcm	Imc1	-0.669640653
wavelet-HLL	glcm	DifferenceAverage	9.433041461
wavelet-HLL	glcm	Id	0.189035242
wavelet-HLL	glcm	ClusterTendency	373.9686376
wavelet-HLL	firstorder	InterquartileRange	450.50684
wavelet-HLL	firstorder	Skewness	0.520039455
wavelet-HLL	firstorder	Uniformity	0.044091711
wavelet-HLL	firstorder	Median	-143.1591556
wavelet-HLL	firstorder	Energy	5618779.371
wavelet-HLL	firstorder	RobustMeanAbsoluteDeviation	173.891852
wavelet-HLL	firstorder	MeanAbsoluteDeviation	242.2014465
wavelet-HLL	firstorder	TotalEnergy	151707043
wavelet-HLL	firstorder	Maximum	573.5288922
wavelet-HLL	firstorder	RootMeanSquared	298.6418828
wavelet-HLL	firstorder	90Percentile	386.8902691
wavelet-HLL	firstorder	Minimum	-492.5726445
wavelet-HLL	firstorder	Entropy	4.676102646
wavelet-HLL	firstorder	Range	1066.101537
wavelet-HLL	firstorder	Variance	84683.59795
wavelet-HLL	firstorder	10Percentile	-413.5500321
wavelet-HLL	firstorder	Kurtosis	2.331666212

wavelet-HLL	firstorder	Mean	-67.10719923
wavelet-HLL	glrlm	ShortRunLowGrayLevelEmphasis	0.049133885
wavelet-HLL	glrlm	GrayLevelVariance	134.9872059
wavelet-HLL	glrlm	LowGrayLevelRunEmphasis	0.049452303
wavelet-HLL	glrlm	GrayLevelNonUniformityNormalized	0.043962148
wavelet-HLL	glrlm	RunVariance	0.018291855
wavelet-HLL	glrlm	GrayLevelNonUniformity	2.718573811
wavelet-HLL	glrlm	LongRunEmphasis	1.056697718
wavelet-HLL	glrlm	ShortRunHighGrayLevelEmphasis	447.9928661
wavelet-HLL	glrlm	RunLengthNonUniformity	59.61207338
wavelet-HLL	glrlm	ShortRunEmphasis	0.985825571
wavelet-HLL	glrlm	LongRunHighGrayLevelEmphasis	478.5584271
wavelet-HLL	glrlm	RunPercentage	0.981684982
wavelet-HLL	glrlm	LongRunLowGrayLevelEmphasis	0.050725973
wavelet-HLL	glrlm	RunEntropy	4.711744064
wavelet-HLL	glrlm	HighGrayLevelRunEmphasis	454.1059783
wavelet-HLL	glrlm	RunLengthNonUniformityNormalized	0.963416291
wavelet-HLL	glszm	GrayLevelVariance	128.72
wavelet-HLL	glszm	ZoneVariance	0.3524
wavelet-HLL	glszm	GrayLevelNonUniformityNormalized	0.044
wavelet-HLL	glszm	SizeZoneNonUniformityNormalized	0.6664
wavelet-HLL	glszm	SizeZoneNonUniformity	33.32
wavelet-HLL	glszm	GrayLevelNonUniformity	2.2
wavelet-HLL	glszm	LargeAreaEmphasis	1.94
wavelet-HLL	glszm	SmallAreaHighGrayLevelEmphasis	369.34
wavelet-HLL	glszm	ZonePercentage	0.793650794
wavelet-HLL	glszm	LargeAreaLowGrayLevelEmphasis	0.080279907
wavelet-HLL	glszm	LargeAreaHighGrayLevelEmphasis	829.04
wavelet-HLL	glszm	HighGrayLevelZoneEmphasis	452.72
wavelet-HLL	glszm	SmallAreaEmphasis	0.843472222
wavelet-HLL	glszm	LowGrayLevelZoneEmphasis	0.056174129
wavelet-HLL	glszm	ZoneEntropy	4.85366069
wavelet-HLL	glszm	SmallAreaLowGrayLevelEmphasis	0.053713699
wavelet-HLL	ngtdm	Coarseness	0.075674798
wavelet-HLL	ngtdm	Complexity	2580.211956
wavelet-HLL	ngtdm	Strength	46.45857672
wavelet-HLL	ngtdm	Contrast	1.863518922
wavelet-HLL	ngtdm	Busyness	0.032843209
wavelet-LHL	gldm	GrayLevelVariance	127.8342152
wavelet-LHL	gldm	HighGrayLevelEmphasis	943.2539683
wavelet-LHL	gldm	DependenceEntropy	5.223156511
wavelet-LHL	gldm	DependenceNonUniformity	32.07936508
wavelet-LHL	gldm	GrayLevelNonUniformity	2.587301587
wavelet-LHL	gldm	SmallDependenceEmphasis	0.700176367
wavelet-LHL	gldm	SmallDependenceHighGrayLevelEmphasis	694.0555556
wavelet-LHL	gldm	DependenceNonUniformityNormalized	0.509196271
wavelet-LHL	gldm	LargeDependenceEmphasis	2.26984127
wavelet-LHL	gldm	LargeDependenceLowGrayLevelEmphasis	0.020187297
wavelet-LHL	gldm	DependenceVariance	0.274124465
wavelet-LHL	gldm	LargeDependenceHighGrayLevelEmphasis	2003.539683
wavelet-LHL	gldm	SmallDependenceLowGrayLevelEmphasis	0.01708869
wavelet-LHL	gldm	LowGrayLevelEmphasis	0.017692735
wavelet-LHL	glcm	JointAverage	27.50237668
wavelet-LHL	glcm	SumAverage	55.00475336
wavelet-LHL	glcm	JointEntropy	6.034807428

wavelet-LHL	glcm	ClusterShade	3325.308308
wavelet-LHL	glcm	MaximumProbability	0.027386443
wavelet-LHL	glcm	Idmn	0.937313272
wavelet-LHL	glcm	JointEnergy	0.015689082
wavelet-LHL	glcm	Contrast	223.8200174
wavelet-LHL	glcm	DifferenceEntropy	4.11638172
wavelet-LHL	glcm	InverseVariance	0.093084474
wavelet-LHL	glcm	DifferenceVariance	79.47582491
wavelet-LHL	glcm	Idn	0.837785159
wavelet-LHL	glcm	Idm	0.085821765
wavelet-LHL	glcm	Correlation	0.123067267
wavelet-LHL	glcm	Autocorrelation	774.1090155
wavelet-LHL	glcm	SumEntropy	4.60269813
wavelet-LHL	glcm	SumSquares	129.4352256
wavelet-LHL	glcm	ClusterProminence	276581.9814
wavelet-LHL	glcm	Imc2	0.999339139
wavelet-LHL	glcm	Imc1	-0.713847804
wavelet-LHL	glcm	DifferenceAverage	11.64493465
wavelet-LHL	glcm	Id	0.162742093
wavelet-LHL	glcm	ClusterTendency	293.920885
wavelet-LHL	firstorder	InterquartileRange	396.9788529
wavelet-LHL	firstorder	Skewness	0.38880193
wavelet-LHL	firstorder	Uniformity	0.041068279
wavelet-LHL	firstorder	Median	-70.88033767
wavelet-LHL	firstorder	Energy	5195448.229
wavelet-LHL	firstorder	RobustMeanAbsoluteDeviation	151.2982806
wavelet-LHL	firstorder	MeanAbsoluteDeviation	225.2684762
wavelet-LHL	firstorder	TotalEnergy	140277102.2
wavelet-LHL	firstorder	Maximum	593.2526325
wavelet-LHL	firstorder	RootMeanSquared	287.1714335
wavelet-LHL	firstorder	90Percentile	294.1079114
wavelet-LHL	firstorder	Minimum	-746.5495357
wavelet-LHL	firstorder	Entropy	4.845784487
wavelet-LHL	firstorder	Range	1339.802168
wavelet-LHL	firstorder	Variance	79912.87211
wavelet-LHL	firstorder	10Percentile	-369.3468092
wavelet-LHL	firstorder	Kurtosis	2.916123378
wavelet-LHL	firstorder	Mean	-50.54265614
wavelet-LHL	glrlm	ShortRunLowGrayLevelEmphasis	0.01792914
wavelet-LHL	glrlm	GrayLevelVariance	128.1503142
wavelet-LHL	glrlm	LowGrayLevelRunEmphasis	0.017953984
wavelet-LHL	glrlm	GrayLevelNonUniformityNormalized	0.040825833
wavelet-LHL	glrlm	RunVariance	0.015821125
wavelet-LHL	glrlm	GrayLevelNonUniformity	2.531756864
wavelet-LHL	glrlm	LongRunEmphasis	1.049914645
wavelet-LHL	glrlm	ShortRunHighGrayLevelEmphasis	934.757853
wavelet-LHL	glrlm	RunLengthNonUniformity	60.0964151
wavelet-LHL	glrlm	ShortRunEmphasis	0.987521339
wavelet-LHL	glrlm	LongRunHighGrayLevelEmphasis	986.593948
wavelet-LHL	glrlm	RunPercentage	0.984126984
wavelet-LHL	glrlm	LongRunLowGrayLevelEmphasis	0.018053362
wavelet-LHL	glrlm	RunEntropy	4.878879508
wavelet-LHL	glrlm	HighGrayLevelRunEmphasis	945.125072
wavelet-LHL	glrlm	RunLengthNonUniformityNormalized	0.96835775
wavelet-LHL	glszm	GrayLevelVariance	133.1584

wavelet-LHL	glszm	ZoneVariance	0.2324
wavelet-LHL	glszm	GrayLevelNonUniformityNormalized	0.0368
wavelet-LHL	glszm	SizeZoneNonUniformityNormalized	0.6264
wavelet-LHL	glszm	SizeZoneNonUniformity	31.32
wavelet-LHL	glszm	GrayLevelNonUniformity	1.84
wavelet-LHL	glszm	LargeAreaEmphasis	1.82
wavelet-LHL	glszm	SmallAreaHighGrayLevelEmphasis	820.345
wavelet-LHL	glszm	ZonePercentage	0.793650794
wavelet-LHL	glszm	LargeAreaLowGrayLevelEmphasis	0.023370191
wavelet-LHL	glszm	LargeAreaHighGrayLevelEmphasis	1657.82
wavelet-LHL	glszm	HighGrayLevelZoneEmphasis	971.84
wavelet-LHL	glszm	SmallAreaEmphasis	0.817222222
wavelet-LHL	glszm	LowGrayLevelZoneEmphasis	0.021776395
wavelet-LHL	glszm	ZoneEntropy	5.25366069
wavelet-LHL	glszm	SmallAreaLowGrayLevelEmphasis	0.021402637
wavelet-LHL	ngtdm	Coarseness	0.056441217
wavelet-LHL	ngtdm	Complexity	4624.660035
wavelet-LHL	ngtdm	Strength	41.18300254
wavelet-LHL	ngtdm	Contrast	1.805278373
wavelet-LHL	ngtdm	Busyness	0.026592785
wavelet-LHH	gldm	GrayLevelVariance	40.19551524
wavelet-LHH	gldm	HighGrayLevelEmphasis	322.2222222
wavelet-LHH	gldm	DependenceEntropy	4.925459884
wavelet-LHH	gldm	DependenceNonUniformity	29.12698413
wavelet-LHH	gldm	GrayLevelNonUniformity	3.412698413
wavelet-LHH	gldm	SmallDependenceEmphasis	0.668981481
wavelet-LHH	gldm	SmallDependenceHighGrayLevelEmphasis	213.4329806
wavelet-LHH	gldm	DependenceNonUniformityNormalized	0.462333081
wavelet-LHH	gldm	LargeDependenceEmphasis	2.714285714
wavelet-LHH	gldm	LargeDependenceLowGrayLevelEmphasis	0.029958392
wavelet-LHH	gldm	DependenceVariance	0.440413202
wavelet-LHH	gldm	LargeDependenceHighGrayLevelEmphasis	840.7301587
wavelet-LHH	gldm	SmallDependenceLowGrayLevelEmphasis	0.020793577
wavelet-LHH	gldm	LowGrayLevelEmphasis	0.022155889
wavelet-LHH	glcm	JointAverage	17.02444224
wavelet-LHH	glcm	SumAverage	34.04888448
wavelet-LHH	glcm	JointEntropy	5.959107912
wavelet-LHH	glcm	ClusterShade	3.33926906
wavelet-LHH	glcm	MaximumProbability	0.029911009
wavelet-LHH	glcm	Idmn	0.921692787
wavelet-LHH	glcm	JointEnergy	0.016860183
wavelet-LHH	glcm	Contrast	88.71905048
wavelet-LHH	glcm	DifferenceEntropy	3.748816748
wavelet-LHH	glcm	InverseVariance	0.139290099
wavelet-LHH	glcm	DifferenceVariance	30.71228474
wavelet-LHH	glcm	Idn	0.817581858
wavelet-LHH	glcm	Idm	0.11917595
wavelet-LHH	glcm	Correlation	-0.028078144
wavelet-LHH	glcm	Autocorrelation	288.761312
wavelet-LHH	glcm	SumEntropy	4.303938201
wavelet-LHH	glcm	SumSquares	43.08427779
wavelet-LHH	glcm	ClusterProminence	18725.61208
wavelet-LHH	glcm	Imc2	0.99790895
wavelet-LHH	glcm	Imc1	-0.636247411
wavelet-LHH	glcm	DifferenceAverage	7.478959706

wavelet-LHH	glcm	Id	0.208233926
wavelet-LHH	glcm	ClusterTendency	83.61806069
wavelet-LHH	firstorder	InterquartileRange	203.028079
wavelet-LHH	firstorder	Skewness	-0.044071194
wavelet-LHH	firstorder	Uniformity	0.054169816
wavelet-LHH	firstorder	Median	5.212556309
wavelet-LHH	firstorder	Energy	1556441.703
wavelet-LHH	firstorder	RobustMeanAbsoluteDeviation	84.07838618
wavelet-LHH	firstorder	MeanAbsoluteDeviation	125.9998951
wavelet-LHH	firstorder	TotalEnergy	42023925.99
wavelet-LHH	firstorder	Maximum	325.4581488
wavelet-LHH	firstorder	RootMeanSquared	157.1795911
wavelet-LHH	firstorder	90Percentile	211.8321048
wavelet-LHH	firstorder	Minimum	-376.9978574
wavelet-LHH	firstorder	Entropy	4.435447734
wavelet-LHH	firstorder	Range	702.4560061
wavelet-LHH	firstorder	Variance	24666.35432
wavelet-LHH	firstorder	10Percentile	-190.287595
wavelet-LHH	firstorder	Kurtosis	2.666128068
wavelet-LHH	firstorder	Mean	6.250563292
wavelet-LHH	glrlm	ShortRunLowGrayLevelEmphasis	0.022449541
wavelet-LHH	glrlm	GrayLevelVariance	40.4909659
wavelet-LHH	glrlm	LowGrayLevelRunEmphasis	0.022515313
wavelet-LHH	glrlm	GrayLevelNonUniformityNormalized	0.053631383
wavelet-LHH	glrlm	RunVariance	0.019510548
wavelet-LHH	glrlm	GrayLevelNonUniformity	3.312884436
wavelet-LHH	glrlm	LongRunEmphasis	1.060541838
wavelet-LHH	glrlm	ShortRunHighGrayLevelEmphasis	317.6085188
wavelet-LHH	glrlm	RunLengthNonUniformity	59.38891104
wavelet-LHH	glrlm	ShortRunEmphasis	0.984864541
wavelet-LHH	glrlm	LongRunHighGrayLevelEmphasis	341.4292587
wavelet-LHH	glrlm	RunPercentage	0.98046398
wavelet-LHH	glrlm	LongRunLowGrayLevelEmphasis	0.022778401
wavelet-LHH	glrlm	RunEntropy	4.498005703
wavelet-LHH	glrlm	HighGrayLevelRunEmphasis	322.3726667
wavelet-LHH	glrlm	RunLengthNonUniformityNormalized	0.960978903
wavelet-LHH	glszm	GrayLevelVariance	44.77604167
wavelet-LHH	glszm	ZoneVariance	0.381510417
wavelet-LHH	glszm	GrayLevelNonUniformityNormalized	0.048611111
wavelet-LHH	glszm	SizeZoneNonUniformityNormalized	0.606770833
wavelet-LHH	glszm	SizeZoneNonUniformity	29.125
wavelet-LHH	glszm	GrayLevelNonUniformity	2.333333333
wavelet-LHH	glszm	LargeAreaEmphasis	2.104166667
wavelet-LHH	glszm	SmallAreaHighGrayLevelEmphasis	255.5266204
wavelet-LHH	glszm	ZonePercentage	0.761904762
wavelet-LHH	glszm	LargeAreaLowGrayLevelEmphasis	0.03264216
wavelet-LHH	glszm	LargeAreaHighGrayLevelEmphasis	666.4583333
wavelet-LHH	glszm	HighGrayLevelZoneEmphasis	321.1666667
wavelet-LHH	glszm	SmallAreaEmphasis	0.805700231
wavelet-LHH	glszm	LowGrayLevelZoneEmphasis	0.027771737
wavelet-LHH	glszm	ZoneEntropy	4.845175521
wavelet-LHH	glszm	SmallAreaLowGrayLevelEmphasis	0.027004791
wavelet-LHH	ngtdm	Coarseness	0.07355921
wavelet-LHH	ngtdm	Complexity	1354.601902
wavelet-LHH	ngtdm	Strength	16.90302338

wavelet-LHH	ngtdm	Contrast	0.651036742
wavelet-LHH	ngtdm	Busyness	0.039931598
wavelet-LLH	gldm	GrayLevelVariance	88.56739733
wavelet-LLH	gldm	HighGrayLevelEmphasis	532.2380952
wavelet-LLH	gldm	DependenceEntropy	5.20339282
wavelet-LLH	gldm	DependenceNonUniformity	25.98412698
wavelet-LLH	gldm	GrayLevelNonUniformity	2.777777778
wavelet-LLH	gldm	SmallDependenceEmphasis	0.634920635
wavelet-LLH	gldm	SmallDependenceHighGrayLevelEmphasis	351.14903
wavelet-LLH	gldm	DependenceNonUniformityNormalized	0.41244646
wavelet-LLH	gldm	LargeDependenceEmphasis	3.095238095
wavelet-LLH	gldm	LargeDependenceLowGrayLevelEmphasis	0.029394369
wavelet-LLH	gldm	DependenceVariance	0.525069287
wavelet-LLH	gldm	LargeDependenceHighGrayLevelEmphasis	1588.587302
wavelet-LLH	gldm	SmallDependenceLowGrayLevelEmphasis	0.021189108
wavelet-LLH	gldm	LowGrayLevelEmphasis	0.022544279
wavelet-LLH	glcm	JointAverage	20.27520636
wavelet-LLH	glcm	SumAverage	40.55041271
wavelet-LLH	glcm	JointEntropy	5.962802146
wavelet-LLH	glcm	ClusterShade	905.6608851
wavelet-LLH	glcm	MaximumProbability	0.030204289
wavelet-LLH	glcm	Idmn	0.926844162
wavelet-LLH	glcm	JointEnergy	0.016904788
wavelet-LLH	glcm	Contrast	137.0579171
wavelet-LLH	glcm	DifferenceEntropy	3.797102434
wavelet-LLH	glcm	InverseVariance	0.118999698
wavelet-LLH	glcm	DifferenceVariance	52.31883756
wavelet-LLH	glcm	Idn	0.829716028
wavelet-LLH	glcm	Idm	0.119709939
wavelet-LLH	glcm	Correlation	0.263366242
wavelet-LLH	glcm	Autocorrelation	436.5326905
wavelet-LLH	glcm	SumEntropy	4.601476248
wavelet-LLH	glcm	SumSquares	93.77692828
wavelet-LLH	glcm	ClusterProminence	134351.9009
wavelet-LLH	glcm	Imc2	0.99904045
wavelet-LLH	glcm	Imc1	-0.696250315
wavelet-LLH	glcm	DifferenceAverage	8.868355705
wavelet-LLH	glcm	Id	0.204477322
wavelet-LLH	glcm	ClusterTendency	238.049796
wavelet-LLH	firstorder	InterquartileRange	356.1599411
wavelet-LLH	firstorder	Skewness	-0.084277755
wavelet-LLH	firstorder	Uniformity	0.044091711
wavelet-LLH	firstorder	Median	-59.43696288
wavelet-LLH	firstorder	Energy	3736833.696
wavelet-LLH	firstorder	RobustMeanAbsoluteDeviation	140.6478656
wavelet-LLH	firstorder	MeanAbsoluteDeviation	194.0676647
wavelet-LLH	firstorder	TotalEnergy	100894509.8
wavelet-LLH	firstorder	Maximum	369.0670796
wavelet-LLH	firstorder	RootMeanSquared	243.5463417
wavelet-LLH	firstorder	90Percentile	242.9436439
wavelet-LLH	firstorder	Minimum	-570.918381
wavelet-LLH	firstorder	Entropy	4.661504479
wavelet-LLH	firstorder	Range	939.9854606
wavelet-LLH	firstorder	Variance	55687.2661
wavelet-LLH	firstorder	10Percentile	-386.8515

wavelet-LLH	firstorder	Kurtosis	2.177513661
wavelet-LLH	firstorder	Mean	-60.22918294
wavelet-LLH	glrlm	ShortRunLowGrayLevelEmphasis	0.022950608
wavelet-LLH	glrlm	GrayLevelVariance	89.56252552
wavelet-LLH	glrlm	LowGrayLevelRunEmphasis	0.023014086
wavelet-LLH	glrlm	GrayLevelNonUniformityNormalized	0.043774189
wavelet-LLH	glrlm	RunVariance	0.02309523
wavelet-LLH	glrlm	GrayLevelNonUniformity	2.694591456
wavelet-LLH	glrlm	LongRunEmphasis	1.073780802
wavelet-LLH	glrlm	ShortRunHighGrayLevelEmphasis	523.6444554
wavelet-LLH	glrlm	RunLengthNonUniformity	58.79110136
wavelet-LLH	glrlm	ShortRunEmphasis	0.9815548
wavelet-LLH	glrlm	LongRunHighGrayLevelEmphasis	570.1086504
wavelet-LLH	glrlm	RunPercentage	0.976800977
wavelet-LLH	glrlm	LongRunLowGrayLevelEmphasis	0.023268
wavelet-LLH	glrlm	RunEntropy	4.725942114
wavelet-LLH	glrlm	HighGrayLevelRunEmphasis	532.9372944
wavelet-LLH	glrlm	RunLengthNonUniformityNormalized	0.95380954
wavelet-LLH	glszm	GrayLevelVariance	104.1995062
wavelet-LLH	glszm	ZoneVariance	0.64
wavelet-LLH	glszm	GrayLevelNonUniformityNormalized	0.040987654
wavelet-LLH	glszm	SizeZoneNonUniformityNormalized	0.595061728
wavelet-LLH	glszm	SizeZoneNonUniformity	26.77777778
wavelet-LLH	glszm	GrayLevelNonUniformity	1.844444444
wavelet-LLH	glszm	LargeAreaEmphasis	2.6
wavelet-LLH	glszm	SmallAreaHighGrayLevelEmphasis	447.1280864
wavelet-LLH	glszm	ZonePercentage	0.714285714
wavelet-LLH	glszm	LargeAreaLowGrayLevelEmphasis	0.035220431
wavelet-LLH	glszm	LargeAreaHighGrayLevelEmphasis	1373.888889
wavelet-LLH	glszm	HighGrayLevelZoneEmphasis	544.2666667
wavelet-LLH	glszm	SmallAreaEmphasis	0.799074074
wavelet-LLH	glszm	LowGrayLevelZoneEmphasis	0.030159638
wavelet-LLH	glszm	ZoneEntropy	5.047408652
wavelet-LLH	glszm	SmallAreaLowGrayLevelEmphasis	0.029333095
wavelet-LLH	ngtdm	Coarseness	0.073810194
wavelet-LLH	ngtdm	Complexity	2305.552794
wavelet-LLH	ngtdm	Strength	32.14984696
wavelet-LLH	ngtdm	Contrast	1.27504455
wavelet-LLH	ngtdm	Busyness	0.02689164
wavelet-LLL	gldm	GrayLevelVariance	486.6293777
wavelet-LLL	gldm	HighGrayLevelEmphasis	2043.746032
wavelet-LLL	gldm	DependenceEntropy	5.357921987
wavelet-LLL	gldm	DependenceNonUniformity	53.6031746
wavelet-LLL	gldm	GrayLevelNonUniformity	1.698412698
wavelet-LLL	gldm	SmallDependenceEmphasis	0.933862434
wavelet-LLL	gldm	SmallDependenceHighGrayLevelEmphasis	1808.656085
wavelet-LLL	gldm	DependenceNonUniformityNormalized	0.850844041
wavelet-LLL	gldm	LargeDependenceEmphasis	1.476190476
wavelet-LLL	gldm	LargeDependenceLowGrayLevelEmphasis	0.025666385
wavelet-LLL	gldm	DependenceVariance	0.206097254
wavelet-LLL	gldm	LargeDependenceHighGrayLevelEmphasis	4111.936508
wavelet-LLL	gldm	SmallDependenceLowGrayLevelEmphasis	0.025289337
wavelet-LLL	gldm	LowGrayLevelEmphasis	0.025356803
wavelet-LLL	glcm	JointAverage	46.4191287
wavelet-LLL	glcm	SumAverage	92.8382574

wavelet-LLL	glcm	JointEntropy	6.088362039
wavelet-LLL	glcm	ClusterShade	2697.770862
wavelet-LLL	glcm	MaximumProbability	0.023104367
wavelet-LLL	glcm	Idmn	0.902398242
wavelet-LLL	glcm	JointEnergy	0.014989188
wavelet-LLL	glcm	Contrast	908.7973834
wavelet-LLL	glcm	DifferenceEntropy	4.437430409
wavelet-LLL	glcm	InverseVariance	0.037936291
wavelet-LLL	glcm	DifferenceVariance	278.7206023
wavelet-LLL	glcm	Idn	0.795291179
wavelet-LLL	glcm	Idm	0.034037228
wavelet-LLL	glcm	Correlation	0.140086483
wavelet-LLL	glcm	Autocorrelation	2223.593919
wavelet-LLL	glcm	SumEntropy	4.871484443
wavelet-LLL	glcm	SumSquares	520.022252
wavelet-LLL	glcm	ClusterProminence	3243006.604
wavelet-LLL	glcm	Imc2	0.999904423
wavelet-LLL	glcm	Imc1	-0.828864565
wavelet-LLL	glcm	DifferenceAverage	24.49244975
wavelet-LLL	glcm	Id	0.088605446
wavelet-LLL	glcm	ClusterTendency	1171.291624
wavelet-LLL	firstorder	InterquartileRange	783.5796793
wavelet-LLL	firstorder	Skewness	0.362216764
wavelet-LLL	firstorder	Uniformity	0.026958932
wavelet-LLL	firstorder	Median	-935.7855398
wavelet-LLL	firstorder	Energy	64728504.57
wavelet-LLL	firstorder	RobustMeanAbsoluteDeviation	324.0269238
wavelet-LLL	firstorder	MeanAbsoluteDeviation	456.2052349
wavelet-LLL	firstorder	TotalEnergy	1747669623
wavelet-LLL	firstorder	Maximum	295.8623223
wavelet-LLL	firstorder	RootMeanSquared	1013.625464
wavelet-LLL	firstorder	90Percentile	-15.55164379
wavelet-LLL	firstorder	Minimum	-1807.807023
wavelet-LLL	firstorder	Entropy	5.357921987
wavelet-LLL	firstorder	Range	2103.669345
wavelet-LLL	firstorder	Variance	304590.6171
wavelet-LLL	firstorder	10Percentile	-1489.083463
wavelet-LLL	firstorder	Kurtosis	2.262940357
wavelet-LLL	firstorder	Mean	-850.2034835
wavelet-LLL	glrlm	ShortRunLowGrayLevelEmphasis	0.025475993
wavelet-LLL	glrlm	GrayLevelVariance	484.312434
wavelet-LLL	glrlm	LowGrayLevelRunEmphasis	0.025478843
wavelet-LLL	glrlm	GrayLevelNonUniformityNormalized	0.02686847
wavelet-LLL	glrlm	RunVariance	0.004882734
wavelet-LLL	glrlm	GrayLevelNonUniformity	1.684509039
wavelet-LLL	glrlm	LongRunEmphasis	1.014888337
wavelet-LLL	glrlm	ShortRunHighGrayLevelEmphasis	2018.308977
wavelet-LLL	glrlm	RunLengthNonUniformity	62.08684864
wavelet-LLL	glrlm	ShortRunEmphasis	0.996277916
wavelet-LLL	glrlm	LongRunHighGrayLevelEmphasis	2094.551223
wavelet-LLL	glrlm	RunPercentage	0.995115995
wavelet-LLL	glrlm	LongRunLowGrayLevelEmphasis	0.025490244
wavelet-LLL	glrlm	RunEntropy	5.367925082
wavelet-LLL	glrlm	HighGrayLevelRunEmphasis	2033.557426
wavelet-LLL	glrlm	RunLengthNonUniformityNormalized	0.990234531

wavelet-LLL	glszm	GrayLevelVariance	466.5222222
wavelet-LLL	glszm	ZoneVariance	0.080833333
wavelet-LLL	glszm	GrayLevelNonUniformityNormalized	0.026666667
wavelet-LLL	glszm	SizeZoneNonUniformityNormalized	0.935
wavelet-LLL	glszm	SizeZoneNonUniformity	56.1
wavelet-LLL	glszm	GrayLevelNonUniformity	1.6
wavelet-LLL	glszm	LargeAreaEmphasis	1.183333333
wavelet-LLL	glszm	SmallAreaHighGrayLevelEmphasis	1877.685185
wavelet-LLL	glszm	ZonePercentage	0.952380952
wavelet-LLL	glszm	LargeAreaLowGrayLevelEmphasis	0.026726742
wavelet-LLL	glszm	LargeAreaHighGrayLevelEmphasis	2692.166667
wavelet-LLL	glszm	HighGrayLevelZoneEmphasis	1961.633333
wavelet-LLL	glszm	SmallAreaEmphasis	0.972685185
wavelet-LLL	glszm	LowGrayLevelZoneEmphasis	0.026576722
wavelet-LLL	glszm	ZoneEntropy	5.369146221
wavelet-LLL	glszm	SmallAreaLowGrayLevelEmphasis	0.026542692
wavelet-LLL	ngtdm	Coarseness	0.034537611
wavelet-LLL	ngtdm	Complexity	23480.09624
wavelet-LLL	ngtdm	Strength	85.69189586
wavelet-LLL	ngtdm	Contrast	8.101119044
wavelet-LLL	ngtdm	Busyness	0.023783488
wavelet-HHL	gldm	GrayLevelVariance	25.13378685
wavelet-HHL	gldm	HighGrayLevelEmphasis	295.031746
wavelet-HHL	gldm	DependenceEntropy	4.88951286
wavelet-HHL	gldm	DependenceNonUniformity	20.04761905
wavelet-HHL	gldm	GrayLevelNonUniformity	4.650793651
wavelet-HHL	gldm	SmallDependenceEmphasis	0.508708113
wavelet-HHL	gldm	SmallDependenceHighGrayLevelEmphasis	160.9521605
wavelet-HHL	gldm	DependenceNonUniformityNormalized	0.318216175
wavelet-HHL	gldm	LargeDependenceEmphasis	4.841269841
wavelet-HHL	gldm	LargeDependenceLowGrayLevelEmphasis	0.036054034
wavelet-HHL	gldm	DependenceVariance	1.029478458
wavelet-HHL	gldm	LargeDependenceHighGrayLevelEmphasis	1411.063492
wavelet-HHL	gldm	SmallDependenceLowGrayLevelEmphasis	0.018215293
wavelet-HHL	gldm	LowGrayLevelEmphasis	0.020552661
wavelet-HHL	glcm	JointAverage	16.52602019
wavelet-HHL	glcm	SumAverage	33.05204039
wavelet-HHL	glcm	JointEntropy	5.82266732
wavelet-HHL	glcm	ClusterShade	-5.184873948
wavelet-HHL	glcm	MaximumProbability	0.03213056
wavelet-HHL	glcm	Idmn	0.953945748
wavelet-HHL	glcm	JointEnergy	0.018902074
wavelet-HHL	glcm	Contrast	55.75092328
wavelet-HHL	glcm	DifferenceEntropy	3.486361849
wavelet-HHL	glcm	InverseVariance	0.180182997
wavelet-HHL	glcm	DifferenceVariance	22.27245398
wavelet-HHL	glcm	Idn	0.86121527
wavelet-HHL	glcm	Idm	0.175483426
wavelet-HHL	glcm	Correlation	0.048208531
wavelet-HHL	glcm	Autocorrelation	274.8345986
wavelet-HHL	glcm	SumEntropy	4.089715475
wavelet-HHL	glcm	SumSquares	29.47173466
wavelet-HHL	glcm	ClusterProminence	12473.86227
wavelet-HHL	glcm	Imc2	0.992066575
wavelet-HHL	glcm	Imc1	-0.534961488

wavelet-HHL	glcm	DifferenceAverage	5.698963595
wavelet-HHL	glcm	Id	0.26575699
wavelet-HHL	glcm	ClusterTendency	62.13601534
wavelet-HHL	firstorder	InterquartileRange	150.5862237
wavelet-HHL	firstorder	Skewness	-0.157722492
wavelet-HHL	firstorder	Uniformity	0.073822121
wavelet-HHL	firstorder	Median	10.72821103
wavelet-HHL	firstorder	Energy	990023.6209
wavelet-HHL	firstorder	RobustMeanAbsoluteDeviation	62.44740269
wavelet-HHL	firstorder	MeanAbsoluteDeviation	96.50965172
wavelet-HHL	firstorder	TotalEnergy	26730637.76
wavelet-HHL	firstorder	Maximum	377.6460654
wavelet-HHL	firstorder	RootMeanSquared	125.3581296
wavelet-HHL	firstorder	90Percentile	122.8892327
wavelet-HHL	firstorder	Minimum	-390.8759323
wavelet-HHL	firstorder	Entropy	3.959924069
wavelet-HHL	firstorder	Range	768.5219977
wavelet-HHL	firstorder	Variance	15703.52731
wavelet-HHL	firstorder	10Percentile	-181.9240836
wavelet-HHL	firstorder	Kurtosis	4.135016567
wavelet-HHL	firstorder	Mean	-3.336665678
wavelet-HHL	glrlm	ShortRunLowGrayLevelEmphasis	0.021055508
wavelet-HHL	glrlm	GrayLevelVariance	25.74687071
wavelet-HHL	glrlm	LowGrayLevelRunEmphasis	0.021178992
wavelet-HHL	glrlm	GrayLevelNonUniformityNormalized	0.073010462
wavelet-HHL	glrlm	RunVariance	0.03651081
wavelet-HHL	glrlm	GrayLevelNonUniformity	4.43200511
wavelet-HHL	glrlm	LongRunEmphasis	1.115455943
wavelet-HHL	glrlm	ShortRunHighGrayLevelEmphasis	287.1921762
wavelet-HHL	glrlm	RunLengthNonUniformity	56.31068807
wavelet-HHL	glrlm	ShortRunEmphasis	0.971136014
wavelet-HHL	glrlm	LongRunHighGrayLevelEmphasis	328.322089
wavelet-HHL	glrlm	RunPercentage	0.963369963
wavelet-HHL	glrlm	LongRunLowGrayLevelEmphasis	0.021672927
wavelet-HHL	glrlm	RunEntropy	4.089885987
wavelet-HHL	glrlm	HighGrayLevelRunEmphasis	295.4181588
wavelet-HHL	glrlm	RunLengthNonUniformityNormalized	0.92697838
wavelet-HHL	glszm	GrayLevelVariance	34.7700831
wavelet-HHL	glszm	ZoneVariance	1.593490305
wavelet-HHL	glszm	GrayLevelNonUniformityNormalized	0.069252078
wavelet-HHL	glszm	SizeZoneNonUniformityNormalized	0.48199446
wavelet-HHL	glszm	SizeZoneNonUniformity	18.31578947
wavelet-HHL	glszm	GrayLevelNonUniformity	2.631578947
wavelet-HHL	glszm	LargeAreaEmphasis	4.342105263
wavelet-HHL	glszm	SmallAreaHighGrayLevelEmphasis	233.0739302
wavelet-HHL	glszm	ZonePercentage	0.603174603
wavelet-HHL	glszm	LargeAreaLowGrayLevelEmphasis	0.044454976
wavelet-HHL	glszm	LargeAreaHighGrayLevelEmphasis	1280.552632
wavelet-HHL	glszm	HighGrayLevelZoneEmphasis	304.4210526
wavelet-HHL	glszm	SmallAreaEmphasis	0.719608694
wavelet-HHL	glszm	LowGrayLevelZoneEmphasis	0.031124213
wavelet-HHL	glszm	ZoneEntropy	4.523986066
wavelet-HHL	glszm	SmallAreaLowGrayLevelEmphasis	0.029596716
wavelet-HHL	ngtdm	Coarseness	0.074657245
wavelet-HHL	ngtdm	Complexity	615.8002964

wavelet-HHL	ngtdm	Strength	12.34608142
wavelet-HHL	ngtdm	Contrast	0.55228939
wavelet-HHL	ngtdm	Busyness	0.054938573
wavelet-HLH	gldm	GrayLevelVariance	19.73444192
wavelet-HLH	gldm	HighGrayLevelEmphasis	134.1904762
wavelet-HLH	gldm	DependenceEntropy	4.838003138
wavelet-HLH	gldm	DependenceNonUniformity	19.25396825
wavelet-HLH	gldm	GrayLevelNonUniformity	5
wavelet-HLH	gldm	SmallDependenceEmphasis	0.505458554
wavelet-HLH	gldm	SmallDependenceHighGrayLevelEmphasis	62.09291887
wavelet-HLH	gldm	DependenceNonUniformityNormalized	0.305618544
wavelet-HLH	gldm	LargeDependenceEmphasis	5.253968254
wavelet-HLH	gldm	LargeDependenceLowGrayLevelEmphasis	0.090340043
wavelet-HLH	gldm	DependenceVariance	1.190224238
wavelet-HLH	gldm	LargeDependenceHighGrayLevelEmphasis	663.1111111
wavelet-HLH	gldm	SmallDependenceLowGrayLevelEmphasis	0.029527415
wavelet-HLH	gldm	LowGrayLevelEmphasis	0.036799189
wavelet-HLH	glcm	JointAverage	10.52666728
wavelet-HLH	glcm	SumAverage	21.05333457
wavelet-HLH	glcm	JointEntropy	5.746213444
wavelet-HLH	glcm	ClusterShade	-30.42210228
wavelet-HLH	glcm	MaximumProbability	0.039222011
wavelet-HLH	glcm	Idmn	0.910305363
wavelet-HLH	glcm	JointEnergy	0.020388211
wavelet-HLH	glcm	Contrast	42.56143947
wavelet-HLH	glcm	DifferenceEntropy	3.428638833
wavelet-HLH	glcm	InverseVariance	0.198127676
wavelet-HLH	glcm	DifferenceVariance	15.74256066
wavelet-HLH	glcm	Idn	0.809014367
wavelet-HLH	glcm	Idm	0.190437894
wavelet-HLH	glcm	Correlation	-0.00961269
wavelet-HLH	glcm	Autocorrelation	110.7934653
wavelet-HLH	glcm	SumEntropy	4.020095152
wavelet-HLH	glcm	SumSquares	21.24052387
wavelet-HLH	glcm	ClusterProminence	5157.280935
wavelet-HLH	glcm	Imc2	0.987453468
wavelet-HLH	glcm	Imc1	-0.493055337
wavelet-HLH	glcm	DifferenceAverage	5.097989659
wavelet-HLH	glcm	Id	0.280537037
wavelet-HLH	glcm	ClusterTendency	42.40065601
wavelet-HLH	firstorder	InterquartileRange	142.1399314
wavelet-HLH	firstorder	Skewness	-0.136094914
wavelet-HLH	firstorder	Uniformity	0.079365079
wavelet-HLH	firstorder	Median	12.06292467
wavelet-HLH	firstorder	Energy	792339.6987
wavelet-HLH	firstorder	RobustMeanAbsoluteDeviation	59.26383795
wavelet-HLH	firstorder	MeanAbsoluteDeviation	88.50402106
wavelet-HLH	firstorder	TotalEnergy	21393171.86
wavelet-HLH	firstorder	Maximum	219.2380599
wavelet-HLH	firstorder	RootMeanSquared	112.1464249
wavelet-HLH	firstorder	90Percentile	167.729175
wavelet-HLH	firstorder	Minimum	-227.537623
wavelet-HLH	firstorder	Entropy	3.862067527
wavelet-HLH	firstorder	Range	446.7756828
wavelet-HLH	firstorder	Variance	12558.74872

wavelet-HLH	firstorder	10Percentile	-158.2301833
wavelet-HLH	firstorder	Kurtosis	2.402770567
wavelet-HLH	firstorder	Mean	4.251104722
wavelet-HLH	glrlm	ShortRunLowGrayLevelEmphasis	0.037347111
wavelet-HLH	glrlm	GrayLevelVariance	20.06984465
wavelet-HLH	glrlm	LowGrayLevelRunEmphasis	0.037758102
wavelet-HLH	glrlm	GrayLevelNonUniformityNormalized	0.077941042
wavelet-HLH	glrlm	RunVariance	0.038972526
wavelet-HLH	glrlm	GrayLevelNonUniformity	4.718785041
wavelet-HLH	glrlm	LongRunEmphasis	1.122847917
wavelet-HLH	glrlm	ShortRunHighGrayLevelEmphasis	130.2447383
wavelet-HLH	glrlm	RunLengthNonUniformity	55.8519202
wavelet-HLH	glrlm	ShortRunEmphasis	0.969288021
wavelet-HLH	glrlm	LongRunHighGrayLevelEmphasis	150.4749055
wavelet-HLH	glrlm	RunPercentage	0.960927961
wavelet-HLH	glrlm	LongRunLowGrayLevelEmphasis	0.039402067
wavelet-HLH	glrlm	RunEntropy	4.017627797
wavelet-HLH	glrlm	HighGrayLevelRunEmphasis	134.2907718
wavelet-HLH	glrlm	RunLengthNonUniformityNormalized	0.922054948
wavelet-HLH	glszm	GrayLevelVariance	23.86997809
wavelet-HLH	glszm	ZoneVariance	2.208911614
wavelet-HLH	glszm	GrayLevelNonUniformityNormalized	0.069393718
wavelet-HLH	glszm	SizeZoneNonUniformityNormalized	0.507669832
wavelet-HLH	glszm	SizeZoneNonUniformity	18.78378378
wavelet-HLH	glszm	GrayLevelNonUniformity	2.567567568
wavelet-HLH	glszm	LargeAreaEmphasis	5.108108108
wavelet-HLH	glszm	SmallAreaHighGrayLevelEmphasis	87.24865416
wavelet-HLH	glszm	ZonePercentage	0.587301587
wavelet-HLH	glszm	LargeAreaLowGrayLevelEmphasis	0.107144237
wavelet-HLH	glszm	LargeAreaHighGrayLevelEmphasis	617.5675676
wavelet-HLH	glszm	HighGrayLevelZoneEmphasis	133.2702703
wavelet-HLH	glszm	SmallAreaEmphasis	0.734916958
wavelet-HLH	glszm	LowGrayLevelZoneEmphasis	0.052511704
wavelet-HLH	glszm	ZoneEntropy	4.553651676
wavelet-HLH	glszm	SmallAreaLowGrayLevelEmphasis	0.048285945
wavelet-HLH	ngtdm	Coarseness	0.069027812
wavelet-HLH	ngtdm	Complexity	483.9962821
wavelet-HLH	ngtdm	Strength	7.534635996
wavelet-HLH	ngtdm	Contrast	0.478875436
wavelet-HLH	ngtdm	Busyness	0.080454478

Image type	Feature Class	Feature Name	3: RTSTRUCT: C99-DNU_segment_ITV-RUL
general	info	BoundingBox	(0, 0, 1, 4, 5, 4)
general	info	EnabledImageTypes	{u'Original': {}, u'Wavelet': {}}
general	info	GeneralSettings	{'distances': [1], 'additionalInfo': True, 'force2D': False, 'interpolator': 'sitkBSpline', 'resampledPixelSpacing': [3.0], 'label': 1, 'normalizeScale': 1, 'normalize': False, 'force2Ddimension': 0, 'removeOutliers': None, 'minimumROISize': None, u'binWidth': 25.0, 'minimumROIDimensions': 1, u'correctMask': True, 'preCrop': False, u'symmetricalGLCM': True, 'resegmentRange': None, 'padDistance': 5}
general	info	ImageHash	238e8e078a6b269bd13474f72c26df1f9eb8f449
general	info	ImageSpacing	(0.9765625000000002, 0.9765625000000002, 2.0)
general	info	MaskHash	8dc0712b47997a65fb206e06706dd79573dc73c7
general	info	NumpyVersion	1.13.1
general	info	PyWaveletVersion	0.5.2
general	info	SimpleITKVersion	1.1.0
general	info	Version	1.3.0.post102+g1b0eb74
general	info	VolumeNum	1
general	info	VoxelNum	35
original	shape	Maximum3DDiameter	14.07124728
original	shape	Maximum2DDiameterSlice	12.36931688
original	shape	Sphericity	0.873709674
original	shape	MinorAxis	11.275846
original	shape	Elongation	0.82654471
original	shape	SurfaceVolumeRatio	0.564035419
original	shape	Volume	945
original	shape	MajorAxis	13.6421489
original	shape	SurfaceArea	533.0134714
original	shape	Flatness	0.707806996
original	shape	LeastAxis	9.656008437
original	shape	Maximum2DDiameterColumn	10.81665383
original	shape	Maximum2DDiameterRow	13.41640786
original	gldm	GrayLevelVariance	103.5477551
original	gldm	HighGrayLevelEmphasis	400.3714286
original	gldm	DependenceEntropy	4.536286231
original	gldm	DependenceNonUniformity	17.97142857
original	gldm	GrayLevelNonUniformity	1.857142857
original	gldm	SmallDependenceEmphasis	0.717460317
original	gldm	SmallDependenceHighGrayLevelEmphasis	259.531746
original	gldm	DependenceNonUniformityNormalized	0.513469388
original	gldm	LargeDependenceEmphasis	2.257142857
original	gldm	LargeDependenceLowGrayLevelEmphasis	0.05117022
original	gldm	DependenceVariance	0.297142857
original	gldm	LargeDependenceHighGrayLevelEmphasis	976.4285714
original	gldm	SmallDependenceLowGrayLevelEmphasis	0.043997753
original	gldm	LowGrayLevelEmphasis	0.045178278
original	glcm	JointAverage	20.29840556

original	glcm	SumAverage	40.59681111
original	glcm	JointEntropy	4.893555421
original	glcm	ClusterShade	-449.0538434
original	glcm	MaximumProbability	0.050098649
original	glcm	Idmn	0.891693837
original	glcm	JointEnergy	0.034869217
original	glcm	Contrast	173.6295547
original	glcm	DifferenceEntropy	3.482767232
original	glcm	InverseVariance	0.09502363
original	glcm	DifferenceVariance	53.28293022
original	glcm	Idn	0.784484567
original	glcm	Idm	0.092687632
original	glcm	Correlation	0.150578887
original	glcm	Autocorrelation	427.303602
original	glcm	SumEntropy	3.761645139
original	glcm	SumSquares	101.3765226
original	glcm	ClusterProminence	112969.1549
original	glcm	Imc2	0.999098736
original	glcm	Imc1	-0.789115773
original	glcm	DifferenceAverage	10.79129939
original	glcm	Id	0.168399629
original	glcm	ClusterTendency	231.8765355
original	firstorder	InterquartileRange	438
original	firstorder	Skewness	0.270573424
original	firstorder	Uniformity	0.053061224
original	firstorder	Median	-456
original	firstorder	Energy	8817494
original	firstorder	RobustMeanAbsoluteDeviation	169.4019204
original	firstorder	MeanAbsoluteDeviation	218.5485714
original	firstorder	TotalEnergy	238072338
original	firstorder	Maximum	12
original	firstorder	RootMeanSquared	501.9246955
original	firstorder	90Percentile	-76.6
original	firstorder	Minimum	-840
original	firstorder	Entropy	4.378864088
original	firstorder	Range	852
original	firstorder	Variance	64612.56
original	firstorder	10Percentile	-702.8
original	firstorder	Kurtosis	1.824848113
original	firstorder	Mean	-432.8
original	glrlm	ShortRunLowGrayLevelEmphasis	0.045774344
original	glrlm	GrayLevelVariance	103.7413837
original	glrlm	LowGrayLevelRunEmphasis	0.045828182
original	glrlm	GrayLevelNonUniformityNormalized	0.052959696
original	glrlm	RunVariance	0.015358964
original	glrlm	GrayLevelNonUniformity	1.825174825
original	glrlm	LongRunEmphasis	1.047922666
original	glrlm	ShortRunHighGrayLevelEmphasis	393.4042541
original	glrlm	RunLengthNonUniformity	33.42588784
original	glrlm	ShortRunEmphasis	0.988019334
original	glrlm	LongRunHighGrayLevelEmphasis	422.038818
original	glrlm	RunPercentage	0.984615385
original	glrlm	LongRunLowGrayLevelEmphasis	0.046043533
original	glrlm	RunEntropy	4.395097779

original	glrlm	HighGrayLevelRunEmphasis	399.1311669
original	glrlm	RunLengthNonUniformityNormalized	0.969282072
original	glszm	GrayLevelVariance	107.0306122
original	glszm	ZoneVariance	0.258928571
original	glszm	GrayLevelNonUniformityNormalized	0.051020408
original	glszm	SizeZoneNonUniformityNormalized	0.650510204
original	glszm	SizeZoneNonUniformity	18.21428571
original	glszm	GrayLevelNonUniformity	1.428571429
original	glszm	LargeAreaEmphasis	1.821428571
original	glszm	SmallAreaHighGrayLevelEmphasis	294.7093254
original	glszm	ZonePercentage	0.8
original	glszm	LargeAreaLowGrayLevelEmphasis	0.05944568
original	glszm	LargeAreaHighGrayLevelEmphasis	745.25
original	glszm	HighGrayLevelZoneEmphasis	381.6428571
original	glszm	SmallAreaEmphasis	0.834325397
original	glszm	LowGrayLevelZoneEmphasis	0.055343574
original	glszm	ZoneEntropy	4.494680368
original	glszm	SmallAreaLowGrayLevelEmphasis	0.054714872
original	ngtdm	Coarseness	0.073146291
original	ngtdm	Complexity	2432.519362
original	ngtdm	Strength	37.32757588
original	ngtdm	Contrast	3.162262576
original	ngtdm	Busyness	0.043626291
wavelet-HHH	gldm	GrayLevelVariance	2.053877551
wavelet-HHH	gldm	HighGrayLevelEmphasis	20.91428571
wavelet-HHH	gldm	DependenceEntropy	4.204582218
wavelet-HHH	gldm	DependenceNonUniformity	6.942857143
wavelet-HHH	gldm	GrayLevelNonUniformity	6.828571429
wavelet-HHH	gldm	SmallDependenceEmphasis	0.352488095
wavelet-HHH	gldm	SmallDependenceHighGrayLevelEmphasis	7.307670635
wavelet-HHH	gldm	DependenceNonUniformityNormalized	0.198367347
wavelet-HHH	gldm	LargeDependenceEmphasis	13.68571429
wavelet-HHH	gldm	LargeDependenceLowGrayLevelEmphasis	0.790078879
wavelet-HHH	gldm	DependenceVariance	3.627755102
wavelet-HHH	gldm	LargeDependenceHighGrayLevelEmphasis	290.3428571
wavelet-HHH	gldm	SmallDependenceLowGrayLevelEmphasis	0.059168134
wavelet-HHH	gldm	LowGrayLevelEmphasis	0.096967768
wavelet-HHH	glcm	JointAverage	4.486809307
wavelet-HHH	glcm	SumAverage	8.973618614
wavelet-HHH	glcm	JointEntropy	4.00529255
wavelet-HHH	glcm	ClusterShade	-1.275476642
wavelet-HHH	glcm	MaximumProbability	0.135200647
wavelet-HHH	glcm	Idmn	0.937126756
wavelet-HHH	glcm	JointEnergy	0.07305041
wavelet-HHH	glcm	Contrast	3.688641695
wavelet-HHH	glcm	DifferenceEntropy	1.897059119
wavelet-HHH	glcm	InverseVariance	0.466517572
wavelet-HHH	glcm	DifferenceVariance	1.373443035
wavelet-HHH	glcm	Idn	0.837372721
wavelet-HHH	glcm	Idm	0.446009932
wavelet-HHH	glcm	Correlation	-0.059974305
wavelet-HHH	glcm	Autocorrelation	20.05496798
wavelet-HHH	glcm	SumEntropy	2.526278212
wavelet-HHH	glcm	SumSquares	1.737738363

wavelet-HHH	glcm	ClusterProminence	29.51204514
wavelet-HHH	glcm	Imc2	0.867413815
wavelet-HHH	glcm	Imc1	-0.305328475
wavelet-HHH	glcm	DifferenceAverage	1.511890114
wavelet-HHH	glcm	Id	0.504256849
wavelet-HHH	glcm	ClusterTendency	3.262311758
wavelet-HHH	firstorder	InterquartileRange	46.76723386
wavelet-HHH	firstorder	Skewness	-0.072724721
wavelet-HHH	firstorder	Uniformity	0.195102041
wavelet-HHH	firstorder	Median	-2.340322939
wavelet-HHH	firstorder	Energy	44823.41271
wavelet-HHH	firstorder	RobustMeanAbsoluteDeviation	20.36161275
wavelet-HHH	firstorder	MeanAbsoluteDeviation	29.3672158
wavelet-HHH	firstorder	TotalEnergy	1210232.143
wavelet-HHH	firstorder	Maximum	66.37209858
wavelet-HHH	firstorder	RootMeanSquared	35.78643506
wavelet-HHH	firstorder	90Percentile	36.79828697
wavelet-HHH	firstorder	Minimum	-89.84014109
wavelet-HHH	firstorder	Entropy	2.509552032
wavelet-HHH	firstorder	Range	156.2122397
wavelet-HHH	firstorder	Variance	1258.278884
wavelet-HHH	firstorder	10Percentile	-41.27220708
wavelet-HHH	firstorder	Kurtosis	2.800117677
wavelet-HHH	firstorder	Mean	-4.731812645
wavelet-HHH	glrlm	ShortRunLowGrayLevelEmphasis	0.096981274
wavelet-HHH	glrlm	GrayLevelVariance	2.169700494
wavelet-HHH	glrlm	LowGrayLevelRunEmphasis	0.100763489
wavelet-HHH	glrlm	GrayLevelNonUniformityNormalized	0.190901107
wavelet-HHH	glrlm	RunVariance	0.092421928
wavelet-HHH	glrlm	GrayLevelNonUniformity	6.126377204
wavelet-HHH	glrlm	LongRunEmphasis	1.290333721
wavelet-HHH	glrlm	ShortRunHighGrayLevelEmphasis	19.4905824
wavelet-HHH	glrlm	RunLengthNonUniformity	27.10436803
wavelet-HHH	glrlm	ShortRunEmphasis	0.933114575
wavelet-HHH	glrlm	LongRunHighGrayLevelEmphasis	27.00906658
wavelet-HHH	glrlm	RunPercentage	0.916483516
wavelet-HHH	glrlm	LongRunLowGrayLevelEmphasis	0.117060439
wavelet-HHH	glrlm	RunEntropy	2.856207364
wavelet-HHH	glrlm	HighGrayLevelRunEmphasis	20.90083194
wavelet-HHH	glrlm	RunLengthNonUniformityNormalized	0.841822811
wavelet-HHH	glszm	GrayLevelVariance	3.693877551
wavelet-HHH	glszm	ZoneVariance	6.678571429
wavelet-HHH	glszm	GrayLevelNonUniformityNormalized	0.183673469
wavelet-HHH	glszm	SizeZoneNonUniformityNormalized	0.530612245
wavelet-HHH	glszm	SizeZoneNonUniformity	7.428571429
wavelet-HHH	glszm	GrayLevelNonUniformity	2.571428571
wavelet-HHH	glszm	LargeAreaEmphasis	12.92857143
wavelet-HHH	glszm	SmallAreaHighGrayLevelEmphasis	14.94608376
wavelet-HHH	glszm	ZonePercentage	0.4
wavelet-HHH	glszm	LargeAreaLowGrayLevelEmphasis	0.818173388
wavelet-HHH	glszm	LargeAreaHighGrayLevelEmphasis	272.5714286
wavelet-HHH	glszm	HighGrayLevelZoneEmphasis	20.85714286
wavelet-HHH	glszm	SmallAreaEmphasis	0.723946703
wavelet-HHH	glszm	LowGrayLevelZoneEmphasis	0.15507815

wavelet-HHH	glszm	ZoneEntropy	3.039148672
wavelet-HHH	glszm	SmallAreaLowGrayLevelEmphasis	0.138403936
wavelet-HHH	ngtdm	Coarseness	0.137064656
wavelet-HHH	ngtdm	Complexity	22.0902173
wavelet-HHH	ngtdm	Strength	1.916875372
wavelet-HHH	ngtdm	Contrast	0.125952582
wavelet-HHH	ngtdm	Busyness	0.303992787
wavelet-HLL	gldm	GrayLevelVariance	103.8742857
wavelet-HLL	gldm	HighGrayLevelEmphasis	623.7142857
wavelet-HLL	gldm	DependenceEntropy	4.378864088
wavelet-HLL	gldm	DependenceNonUniformity	16.25714286
wavelet-HLL	gldm	GrayLevelNonUniformity	1.971428571
wavelet-HLL	gldm	SmallDependenceEmphasis	0.693650794
wavelet-HLL	gldm	SmallDependenceHighGrayLevelEmphasis	340.2555556
wavelet-HLL	gldm	DependenceNonUniformityNormalized	0.464489796
wavelet-HLL	gldm	LargeDependenceEmphasis	3.114285714
wavelet-HLL	gldm	LargeDependenceLowGrayLevelEmphasis	0.037098412
wavelet-HLL	gldm	DependenceVariance	0.644897959
wavelet-HLL	gldm	LargeDependenceHighGrayLevelEmphasis	2617.485714
wavelet-HLL	gldm	SmallDependenceLowGrayLevelEmphasis	0.034116987
wavelet-HLL	gldm	LowGrayLevelEmphasis	0.034500238
wavelet-HLL	glcm	JointAverage	22.89932355
wavelet-HLL	glcm	SumAverage	45.7986471
wavelet-HLL	glcm	JointEntropy	4.854113038
wavelet-HLL	glcm	ClusterShade	-921.9291433
wavelet-HLL	glcm	MaximumProbability	0.054638943
wavelet-HLL	glcm	Idmn	0.920368938
wavelet-HLL	glcm	JointEnergy	0.036073426
wavelet-HLL	glcm	Contrast	133.3698131
wavelet-HLL	glcm	DifferenceEntropy	3.261680922
wavelet-HLL	glcm	InverseVariance	0.127415141
wavelet-HLL	glcm	DifferenceVariance	32.48562492
wavelet-HLL	glcm	Idn	0.815610674
wavelet-HLL	glcm	Idm	0.117430424
wavelet-HLL	glcm	Correlation	0.341617382
wavelet-HLL	glcm	Autocorrelation	563.7391658
wavelet-HLL	glcm	SumEntropy	3.703364621
wavelet-HLL	glcm	SumSquares	104.0210319
wavelet-HLL	glcm	ClusterProminence	148181.0372
wavelet-HLL	glcm	Imc2	0.99868639
wavelet-HLL	glcm	Imc1	-0.769805986
wavelet-HLL	glcm	DifferenceAverage	9.314808181
wavelet-HLL	glcm	Id	0.1948683
wavelet-HLL	glcm	ClusterTendency	282.7143143
wavelet-HLL	firstorder	InterquartileRange	382.7159913
wavelet-HLL	firstorder	Skewness	-0.529839721
wavelet-HLL	firstorder	Uniformity	0.056326531
wavelet-HLL	firstorder	Median	-0.611200853
wavelet-HLL	firstorder	Energy	2346087.833
wavelet-HLL	firstorder	RobustMeanAbsoluteDeviation	148.3078169
wavelet-HLL	firstorder	MeanAbsoluteDeviation	209.8122529
wavelet-HLL	firstorder	TotalEnergy	63344371.49
wavelet-HLL	firstorder	Maximum	302.9870718
wavelet-HLL	firstorder	RootMeanSquared	258.9036132

wavelet-HLL	firstorder	90Percentile	268.9147609
wavelet-HLL	firstorder	Minimum	-578.9342289
wavelet-HLL	firstorder	Entropy	4.300153017
wavelet-HLL	firstorder	Range	881.9213006
wavelet-HLL	firstorder	Variance	65297.13017
wavelet-HLL	firstorder	10Percentile	-428.0859448
wavelet-HLL	firstorder	Kurtosis	2.191720157
wavelet-HLL	firstorder	Mean	-41.64073448
wavelet-HLL	glrlm	ShortRunLowGrayLevelEmphasis	0.035264648
wavelet-HLL	glrlm	GrayLevelVariance	104.2121396
wavelet-HLL	glrlm	LowGrayLevelRunEmphasis	0.035286541
wavelet-HLL	glrlm	GrayLevelNonUniformityNormalized	0.055755647
wavelet-HLL	glrlm	RunVariance	0.021807904
wavelet-HLL	glrlm	GrayLevelNonUniformity	1.909465993
wavelet-HLL	glrlm	LongRunEmphasis	1.07091201
wavelet-HLL	glrlm	ShortRunHighGrayLevelEmphasis	599.6913512
wavelet-HLL	glrlm	RunLengthNonUniformity	32.80845972
wavelet-HLL	glrlm	ShortRunEmphasis	0.982271997
wavelet-HLL	glrlm	LongRunHighGrayLevelEmphasis	682.7610922
wavelet-HLL	glrlm	RunPercentage	0.978021978
wavelet-HLL	glrlm	LongRunLowGrayLevelEmphasis	0.035374113
wavelet-HLL	glrlm	RunEntropy	4.346824001
wavelet-HLL	glrlm	HighGrayLevelRunEmphasis	616.3052994
wavelet-HLL	glrlm	RunLengthNonUniformityNormalized	0.956384192
wavelet-HLL	glszm	GrayLevelVariance	105.122085
wavelet-HLL	glszm	ZoneVariance	0.430727023
wavelet-HLL	glszm	GrayLevelNonUniformityNormalized	0.053497942
wavelet-HLL	glszm	SizeZoneNonUniformityNormalized	0.68175583
wavelet-HLL	glszm	SizeZoneNonUniformity	18.40740741
wavelet-HLL	glszm	GrayLevelNonUniformity	1.444444444
wavelet-HLL	glszm	LargeAreaEmphasis	2.111111111
wavelet-HLL	glszm	SmallAreaHighGrayLevelEmphasis	390.877572
wavelet-HLL	glszm	ZonePercentage	0.771428571
wavelet-HLL	glszm	LargeAreaLowGrayLevelEmphasis	0.045702161
wavelet-HLL	glszm	LargeAreaHighGrayLevelEmphasis	1588.444444
wavelet-HLL	glszm	HighGrayLevelZoneEmphasis	530.7037037
wavelet-HLL	glszm	SmallAreaEmphasis	0.845679012
wavelet-HLL	glszm	LowGrayLevelZoneEmphasis	0.044357633
wavelet-HLL	glszm	ZoneEntropy	4.356558335
wavelet-HLL	glszm	SmallAreaLowGrayLevelEmphasis	0.044160299
wavelet-HLL	ngtdm	Coarseness	0.115096935
wavelet-HLL	ngtdm	Complexity	1532.015417
wavelet-HLL	ngtdm	Strength	57.97920918
wavelet-HLL	ngtdm	Contrast	2.280418327
wavelet-HLL	ngtdm	Busyness	0.018488053
wavelet-LHL	gldm	GrayLevelVariance	80.57632653
wavelet-LHL	gldm	HighGrayLevelEmphasis	391.3428571
wavelet-LHL	gldm	DependenceEntropy	4.45757516
wavelet-LHL	gldm	DependenceNonUniformity	18.54285714
wavelet-LHL	gldm	GrayLevelNonUniformity	1.742857143
wavelet-LHL	gldm	SmallDependenceEmphasis	0.752380952
wavelet-LHL	gldm	SmallDependenceHighGrayLevelEmphasis	302.2095238
wavelet-LHL	gldm	DependenceNonUniformityNormalized	0.529795918
wavelet-LHL	gldm	LargeDependenceEmphasis	2.371428571

wavelet-LHL	gldm	LargeDependenceLowGrayLevelEmphasis	0.050871676
wavelet-LHL	gldm	DependenceVariance	0.411428571
wavelet-LHL	gldm	LargeDependenceHighGrayLevelEmphasis	772.2571429
wavelet-LHL	gldm	SmallDependenceLowGrayLevelEmphasis	0.033279065
wavelet-LHL	gldm	LowGrayLevelEmphasis	0.035607111
wavelet-LHL	glcm	JointAverage	17.01589894
wavelet-LHL	glcm	SumAverage	34.03179788
wavelet-LHL	glcm	JointEntropy	4.849452156
wavelet-LHL	glcm	ClusterShade	1011.543692
wavelet-LHL	glcm	MaximumProbability	0.052378387
wavelet-LHL	glcm	Idmn	0.936829161
wavelet-LHL	glcm	JointEnergy	0.036161493
wavelet-LHL	glcm	Contrast	89.60838524
wavelet-LHL	glcm	DifferenceEntropy	3.154070515
wavelet-LHL	glcm	InverseVariance	0.144825266
wavelet-LHL	glcm	DifferenceVariance	29.01985391
wavelet-LHL	glcm	Idn	0.836810253
wavelet-LHL	glcm	Idm	0.123024329
wavelet-LHL	glcm	Correlation	0.446731357
wavelet-LHL	glcm	Autocorrelation	328.1631638
wavelet-LHL	glcm	SumEntropy	3.70014814
wavelet-LHL	glcm	SumSquares	82.96228568
wavelet-LHL	glcm	ClusterProminence	123087.0088
wavelet-LHL	glcm	Imc2	0.999188308
wavelet-LHL	glcm	Imc1	-0.803794706
wavelet-LHL	glcm	DifferenceAverage	7.380161059
wavelet-LHL	glcm	Id	0.212642332
wavelet-LHL	glcm	ClusterTendency	242.2407575
wavelet-LHL	firstorder	InterquartileRange	314.8139617
wavelet-LHL	firstorder	Skewness	0.185728774
wavelet-LHL	firstorder	Uniformity	0.049795918
wavelet-LHL	firstorder	Median	-6.512358271
wavelet-LHL	firstorder	Energy	1819566.019
wavelet-LHL	firstorder	RobustMeanAbsoluteDeviation	143.2242317
wavelet-LHL	firstorder	MeanAbsoluteDeviation	190.9818025
wavelet-LHL	firstorder	TotalEnergy	49128282.51
wavelet-LHL	firstorder	Maximum	388.7192676
wavelet-LHL	firstorder	RootMeanSquared	228.0078958
wavelet-LHL	firstorder	90Percentile	308.9315483
wavelet-LHL	firstorder	Minimum	-446.897996
wavelet-LHL	firstorder	Entropy	4.45757516
wavelet-LHL	firstorder	Range	835.6172636
wavelet-LHL	firstorder	Variance	51562.06714
wavelet-LHL	firstorder	10Percentile	-292.1331729
wavelet-LHL	firstorder	Kurtosis	2.016194513
wavelet-LHL	firstorder	Mean	-20.62846076
wavelet-LHL	glrlm	ShortRunLowGrayLevelEmphasis	0.035885346
wavelet-LHL	glrlm	GrayLevelVariance	80.31385925
wavelet-LHL	glrlm	LowGrayLevelRunEmphasis	0.036011965
wavelet-LHL	glrlm	GrayLevelNonUniformityNormalized	0.049584594
wavelet-LHL	glrlm	RunVariance	0.015346621
wavelet-LHL	glrlm	GrayLevelNonUniformity	1.708891109
wavelet-LHL	glrlm	LongRunEmphasis	1.048334019
wavelet-LHL	glrlm	ShortRunHighGrayLevelEmphasis	388.9082329

wavelet-LHL	glrlm	RunLengthNonUniformity	33.43548608
wavelet-LHL	glrlm	ShortRunEmphasis	0.987916495
wavelet-LHL	glrlm	LongRunHighGrayLevelEmphasis	407.6361227
wavelet-LHL	glrlm	RunPercentage	0.984615385
wavelet-LHL	glrlm	LongRunLowGrayLevelEmphasis	0.036518438
wavelet-LHL	glrlm	RunEntropy	4.475152289
wavelet-LHL	glrlm	HighGrayLevelRunEmphasis	392.6538109
wavelet-LHL	glrlm	RunLengthNonUniformityNormalized	0.969306758
wavelet-LHL	glszm	GrayLevelVariance	77.34363853
wavelet-LHL	glszm	ZoneVariance	0.233055886
wavelet-LHL	glszm	GrayLevelNonUniformityNormalized	0.048751486
wavelet-LHL	glszm	SizeZoneNonUniformityNormalized	0.705112961
wavelet-LHL	glszm	SizeZoneNonUniformity	20.44827586
wavelet-LHL	glszm	GrayLevelNonUniformity	1.413793103
wavelet-LHL	glszm	LargeAreaEmphasis	1.689655172
wavelet-LHL	glszm	SmallAreaHighGrayLevelEmphasis	347.2969349
wavelet-LHL	glszm	ZonePercentage	0.828571429
wavelet-LHL	glszm	LargeAreaLowGrayLevelEmphasis	0.04803743
wavelet-LHL	glszm	LargeAreaHighGrayLevelEmphasis	621.137931
wavelet-LHL	glszm	HighGrayLevelZoneEmphasis	400.1034483
wavelet-LHL	glszm	SmallAreaEmphasis	0.865900383
wavelet-LHL	glszm	LowGrayLevelZoneEmphasis	0.040981227
wavelet-LHL	glszm	ZoneEntropy	4.487122805
wavelet-LHL	glszm	SmallAreaLowGrayLevelEmphasis	0.039815835
wavelet-LHL	ngtdm	Coarseness	0.142945402
wavelet-LHL	ngtdm	Complexity	1242.235178
wavelet-LHL	ngtdm	Strength	57.7055248
wavelet-LHL	ngtdm	Contrast	1.180240875
wavelet-LHL	ngtdm	Busyness	0.029450172
wavelet-LHH	gldm	GrayLevelVariance	12.85387755
wavelet-LHH	gldm	HighGrayLevelEmphasis	199.3714286
wavelet-LHH	gldm	DependenceEntropy	4.079143374
wavelet-LHH	gldm	DependenceNonUniformity	12.6
wavelet-LHH	gldm	GrayLevelNonUniformity	4.028571429
wavelet-LHH	gldm	SmallDependenceEmphasis	0.54484127
wavelet-LHH	gldm	SmallDependenceHighGrayLevelEmphasis	112.2031746
wavelet-LHH	gldm	DependenceNonUniformityNormalized	0.36
wavelet-LHH	gldm	LargeDependenceEmphasis	3.971428571
wavelet-LHH	gldm	LargeDependenceLowGrayLevelEmphasis	0.055348198
wavelet-LHH	gldm	DependenceVariance	0.731428571
wavelet-LHH	gldm	LargeDependenceHighGrayLevelEmphasis	702.7428571
wavelet-LHH	gldm	SmallDependenceLowGrayLevelEmphasis	0.031520116
wavelet-LHH	gldm	LowGrayLevelEmphasis	0.034293669
wavelet-LHH	glcm	JointAverage	13.39419831
wavelet-LHH	glcm	SumAverage	26.78839663
wavelet-LHH	glcm	JointEntropy	4.592943823
wavelet-LHH	glcm	ClusterShade	-60.2179925
wavelet-LHH	glcm	MaximumProbability	0.07502308
wavelet-LHH	glcm	Idmn	0.955158577
wavelet-LHH	glcm	JointEnergy	0.045218687
wavelet-LHH	glcm	Contrast	23.97987875
wavelet-LHH	glcm	DifferenceEntropy	2.747133081
wavelet-LHH	glcm	InverseVariance	0.277116544
wavelet-LHH	glcm	DifferenceVariance	9.815536246

wavelet-LHH	glcm	Idn	0.862985731
wavelet-LHH	glcm	Idm	0.233282702
wavelet-LHH	glcm	Correlation	-0.005126687
wavelet-LHH	glcm	Autocorrelation	179.5785634
wavelet-LHH	glcm	SumEntropy	3.235110955
wavelet-LHH	glcm	SumSquares	12.01458574
wavelet-LHH	glcm	ClusterProminence	2191.707943
wavelet-LHH	glcm	Imc2	0.986098887
wavelet-LHH	glcm	Imc1	-0.572396776
wavelet-LHH	glcm	DifferenceAverage	3.688032223
wavelet-LHH	glcm	Id	0.325103441
wavelet-LHH	glcm	ClusterTendency	24.0784642
wavelet-LHH	firstorder	InterquartileRange	102.6584311
wavelet-LHH	firstorder	Skewness	-1.078001823
wavelet-LHH	firstorder	Uniformity	0.115102041
wavelet-LHH	firstorder	Median	3.840195234
wavelet-LHH	firstorder	Energy	273369.7259
wavelet-LHH	firstorder	RobustMeanAbsoluteDeviation	45.821738
wavelet-LHH	firstorder	MeanAbsoluteDeviation	67.07426072
wavelet-LHH	firstorder	TotalEnergy	7380982.598
wavelet-LHH	firstorder	Maximum	188.0453823
wavelet-LHH	firstorder	RootMeanSquared	88.37739301
wavelet-LHH	firstorder	90Percentile	86.47963624
wavelet-LHH	firstorder	Minimum	-306.2083479
wavelet-LHH	firstorder	Entropy	3.293887847
wavelet-LHH	firstorder	Range	494.2537303
wavelet-LHH	firstorder	Variance	7790.968545
wavelet-LHH	firstorder	10Percentile	-92.51732864
wavelet-LHH	firstorder	Kurtosis	5.486405709
wavelet-LHH	firstorder	Mean	4.426629703
wavelet-LHH	glrlm	ShortRunLowGrayLevelEmphasis	0.035018377
wavelet-LHH	glrlm	GrayLevelVariance	13.00266069
wavelet-LHH	glrlm	LowGrayLevelRunEmphasis	0.035179358
wavelet-LHH	glrlm	GrayLevelNonUniformityNormalized	0.11473167
wavelet-LHH	glrlm	RunVariance	0.030705585
wavelet-LHH	glrlm	GrayLevelNonUniformity	3.892096139
wavelet-LHH	glrlm	LongRunEmphasis	1.096256684
wavelet-LHH	glrlm	ShortRunHighGrayLevelEmphasis	195.7646021
wavelet-LHH	glrlm	RunLengthNonUniformity	31.86137392
wavelet-LHH	glrlm	ShortRunEmphasis	0.975935829
wavelet-LHH	glrlm	LongRunHighGrayLevelEmphasis	217.2012536
wavelet-LHH	glrlm	RunPercentage	0.969230769
wavelet-LHH	glrlm	LongRunLowGrayLevelEmphasis	0.035823285
wavelet-LHH	glrlm	RunEntropy	3.397487306
wavelet-LHH	glrlm	HighGrayLevelRunEmphasis	200.0519324
wavelet-LHH	glrlm	RunLengthNonUniformityNormalized	0.938588831
wavelet-LHH	glszm	GrayLevelVariance	15.86389414
wavelet-LHH	glszm	ZoneVariance	0.771266541
wavelet-LHH	glszm	GrayLevelNonUniformityNormalized	0.103969754
wavelet-LHH	glszm	SizeZoneNonUniformityNormalized	0.50094518
wavelet-LHH	glszm	SizeZoneNonUniformity	11.52173913
wavelet-LHH	glszm	GrayLevelNonUniformity	2.391304348
wavelet-LHH	glszm	LargeAreaEmphasis	3.086956522
wavelet-LHH	glszm	SmallAreaHighGrayLevelEmphasis	148.7826087

wavelet-LHH	glszm	ZonePercentage	0.657142857
wavelet-LHH	glszm	LargeAreaLowGrayLevelEmphasis	0.062209149
wavelet-LHH	glszm	LargeAreaHighGrayLevelEmphasis	596.173913
wavelet-LHH	glszm	HighGrayLevelZoneEmphasis	203.4347826
wavelet-LHH	glszm	SmallAreaEmphasis	0.722826087
wavelet-LHH	glszm	LowGrayLevelZoneEmphasis	0.048988311
wavelet-LHH	glszm	ZoneEntropy	3.96900163
wavelet-LHH	glszm	SmallAreaLowGrayLevelEmphasis	0.047383594
wavelet-LHH	ngtdm	Coarseness	0.119754632
wavelet-LHH	ngtdm	Complexity	189.0608881
wavelet-LHH	ngtdm	Strength	10.70557516
wavelet-LHH	ngtdm	Contrast	0.494881541
wavelet-LHH	ngtdm	Busyness	0.061972916
wavelet-LLH	gldm	GrayLevelVariance	132.884898
wavelet-LLH	gldm	HighGrayLevelEmphasis	766.4857143
wavelet-LLH	gldm	DependenceEntropy	4.514718017
wavelet-LLH	gldm	DependenceNonUniformity	27.91428571
wavelet-LLH	gldm	GrayLevelNonUniformity	1.685714286
wavelet-LLH	gldm	SmallDependenceEmphasis	0.914285714
wavelet-LLH	gldm	SmallDependenceHighGrayLevelEmphasis	711.8857143
wavelet-LLH	gldm	DependenceNonUniformityNormalized	0.79755102
wavelet-LLH	gldm	LargeDependenceEmphasis	1.342857143
wavelet-LLH	gldm	LargeDependenceLowGrayLevelEmphasis	0.038814274
wavelet-LLH	gldm	DependenceVariance	0.10122449
wavelet-LLH	gldm	LargeDependenceHighGrayLevelEmphasis	984.8857143
wavelet-LLH	gldm	SmallDependenceLowGrayLevelEmphasis	0.034266169
wavelet-LLH	gldm	LowGrayLevelEmphasis	0.03517579
wavelet-LLH	glcm	JointAverage	26.19899703
wavelet-LLH	glcm	SumAverage	52.39799406
wavelet-LLH	glcm	JointEntropy	4.885331459
wavelet-LLH	glcm	ClusterShade	-3800.13219
wavelet-LLH	glcm	MaximumProbability	0.043568641
wavelet-LLH	glcm	Idmn	0.891603262
wavelet-LLH	glcm	JointEnergy	0.034946946
wavelet-LLH	glcm	Contrast	241.9559831
wavelet-LLH	glcm	DifferenceEntropy	3.294075799
wavelet-LLH	glcm	InverseVariance	0.146563228
wavelet-LLH	glcm	DifferenceVariance	78.06443298
wavelet-LLH	glcm	Idn	0.800945801
wavelet-LLH	glcm	Idm	0.096778762
wavelet-LLH	glcm	Correlation	0.141788623
wavelet-LLH	glcm	Autocorrelation	710.5241729
wavelet-LLH	glcm	SumEntropy	3.631777062
wavelet-LLH	glcm	SumSquares	144.6641184
wavelet-LLH	glcm	ClusterProminence	273671.0199
wavelet-LLH	glcm	Imc2	0.999397822
wavelet-LLH	glcm	Imc1	-0.820378079
wavelet-LLH	glcm	DifferenceAverage	11.73684638
wavelet-LLH	glcm	Id	0.173849302
wavelet-LLH	glcm	ClusterTendency	336.7004907
wavelet-LLH	firstorder	InterquartileRange	473.4577397
wavelet-LLH	firstorder	Skewness	-0.65429091
wavelet-LLH	firstorder	Uniformity	0.048163265
wavelet-LLH	firstorder	Median	119.2560783

wavelet-LLH	firstorder	Energy	2899148.866
wavelet-LLH	firstorder	RobustMeanAbsoluteDeviation	197.9094626
wavelet-LLH	firstorder	MeanAbsoluteDeviation	254.4887094
wavelet-LLH	firstorder	TotalEnergy	78277019.38
wavelet-LLH	firstorder	Maximum	362.5551489
wavelet-LLH	firstorder	RootMeanSquared	287.8069227
wavelet-LLH	firstorder	90Percentile	277.6206171
wavelet-LLH	firstorder	Minimum	-617.2931817
wavelet-LLH	firstorder	Entropy	4.514718017
wavelet-LLH	firstorder	Range	979.8483306
wavelet-LLH	firstorder	Variance	82746.87348
wavelet-LLH	firstorder	10Percentile	-448.9215775
wavelet-LLH	firstorder	Kurtosis	2.026399692
wavelet-LLH	firstorder	Mean	-9.270990281
wavelet-LLH	glrlm	ShortRunLowGrayLevelEmphasis	0.035250923
wavelet-LLH	glrlm	GrayLevelVariance	132.4921721
wavelet-LLH	glrlm	LowGrayLevelRunEmphasis	0.035286938
wavelet-LLH	glrlm	GrayLevelNonUniformityNormalized	0.048206287
wavelet-LLH	glrlm	RunVariance	0.004391802
wavelet-LLH	glrlm	GrayLevelNonUniformity	1.679767292
wavelet-LLH	glrlm	LongRunEmphasis	1.013574661
wavelet-LLH	glrlm	ShortRunHighGrayLevelEmphasis	764.9098578
wavelet-LLH	glrlm	RunLengthNonUniformity	34.54751131
wavelet-LLH	glrlm	ShortRunEmphasis	0.996606335
wavelet-LLH	glrlm	LongRunHighGrayLevelEmphasis	775.7186813
wavelet-LLH	glrlm	RunPercentage	0.995604396
wavelet-LLH	glrlm	LongRunLowGrayLevelEmphasis	0.035430995
wavelet-LLH	glrlm	RunEntropy	4.51455308
wavelet-LLH	glrlm	HighGrayLevelRunEmphasis	767.0716225
wavelet-LLH	glrlm	RunLengthNonUniformityNormalized	0.991216396
wavelet-LLH	glszm	GrayLevelVariance	127.9412305
wavelet-LLH	glszm	ZoneVariance	0.056932966
wavelet-LLH	glszm	GrayLevelNonUniformityNormalized	0.048668503
wavelet-LLH	glszm	SizeZoneNonUniformityNormalized	0.886134068
wavelet-LLH	glszm	SizeZoneNonUniformity	29.24242424
wavelet-LLH	glszm	GrayLevelNonUniformity	1.606060606
wavelet-LLH	glszm	LargeAreaEmphasis	1.181818182
wavelet-LLH	glszm	SmallAreaHighGrayLevelEmphasis	745.3787879
wavelet-LLH	glszm	ZonePercentage	0.942857143
wavelet-LLH	glszm	LargeAreaLowGrayLevelEmphasis	0.038593989
wavelet-LLH	glszm	LargeAreaHighGrayLevelEmphasis	890.1515152
wavelet-LLH	glszm	HighGrayLevelZoneEmphasis	774.3333333
wavelet-LLH	glszm	SmallAreaEmphasis	0.954545455
wavelet-LLH	glszm	LowGrayLevelZoneEmphasis	0.03666449
wavelet-LLH	glszm	ZoneEntropy	4.513794877
wavelet-LLH	glszm	SmallAreaLowGrayLevelEmphasis	0.036182115
wavelet-LLH	ngtdm	Coarseness	0.076686389
wavelet-LLH	ngtdm	Complexity	3090.671583
wavelet-LLH	ngtdm	Strength	50.08408734
wavelet-LLH	ngtdm	Contrast	3.585427756
wavelet-LLH	ngtdm	Busyness	0.024543145
wavelet-LLL	gldm	GrayLevelVariance	468.7053061
wavelet-LLL	gldm	HighGrayLevelEmphasis	1932.314286
wavelet-LLL	gldm	DependenceEntropy	4.786425874

wavelet-LLL	gldm	DependenceNonUniformity	27.91428571
wavelet-LLL	gldm	GrayLevelNonUniformity	1.457142857
wavelet-LLL	gldm	SmallDependenceEmphasis	0.914285714
wavelet-LLL	gldm	SmallDependenceHighGrayLevelEmphasis	1880.457143
wavelet-LLL	gldm	DependenceNonUniformityNormalized	0.79755102
wavelet-LLL	gldm	LargeDependenceEmphasis	1.342857143
wavelet-LLL	gldm	LargeDependenceLowGrayLevelEmphasis	0.032908536
wavelet-LLL	gldm	DependenceVariance	0.10122449
wavelet-LLL	gldm	LargeDependenceHighGrayLevelEmphasis	2139.742857
wavelet-LLL	gldm	SmallDependenceLowGrayLevelEmphasis	0.030940807
wavelet-LLL	gldm	LowGrayLevelEmphasis	0.031334353
wavelet-LLL	glcm	JointAverage	44.98156305
wavelet-LLL	glcm	SumAverage	89.9631261
wavelet-LLL	glcm	JointEntropy	4.930993691
wavelet-LLL	glcm	ClusterShade	-5179.511079
wavelet-LLL	glcm	MaximumProbability	0.038797096
wavelet-LLL	glcm	Idmn	0.913669328
wavelet-LLL	glcm	JointEnergy	0.03355393
wavelet-LLL	glcm	Contrast	789.1583832
wavelet-LLL	glcm	DifferenceEntropy	3.700136968
wavelet-LLL	glcm	InverseVariance	0.03790827
wavelet-LLL	glcm	DifferenceVariance	232.9222895
wavelet-LLL	glcm	Idn	0.803858432
wavelet-LLL	glcm	Idm	0.032667511
wavelet-LLL	glcm	Correlation	0.176529044
wavelet-LLL	glcm	Autocorrelation	2105.997638
wavelet-LLL	glcm	SumEntropy	3.825063605
wavelet-LLL	glcm	SumSquares	472.7731538
wavelet-LLL	glcm	ClusterProminence	2778240.56
wavelet-LLL	glcm	Imc2	0.999826395
wavelet-LLL	glcm	Imc1	-0.896526168
wavelet-LLL	glcm	DifferenceAverage	23.16188136
wavelet-LLL	glcm	Id	0.087820682
wavelet-LLL	glcm	ClusterTendency	1101.934232
wavelet-LLL	firstorder	InterquartileRange	836.3382213
wavelet-LLL	firstorder	Skewness	0.29879863
wavelet-LLL	firstorder	Uniformity	0.041632653
wavelet-LLL	firstorder	Median	-1424.127935
wavelet-LLL	firstorder	Energy	76849086.16
wavelet-LLL	firstorder	RobustMeanAbsoluteDeviation	327.0906557
wavelet-LLL	firstorder	MeanAbsoluteDeviation	450.2071015
wavelet-LLL	firstorder	TotalEnergy	2074925326
wavelet-LLL	firstorder	Maximum	-175.210354
wavelet-LLL	firstorder	RootMeanSquared	1481.785469
wavelet-LLL	firstorder	90Percentile	-670.9241842
wavelet-LLL	firstorder	Minimum	-2305.630822
wavelet-LLL	firstorder	Entropy	4.707714803
wavelet-LLL	firstorder	Range	2130.420468
wavelet-LLL	firstorder	Variance	292061.8235
wavelet-LLL	firstorder	10Percentile	-2056.375262
wavelet-LLL	firstorder	Kurtosis	2.229015642
wavelet-LLL	firstorder	Mean	-1379.719664
wavelet-LLL	glrlm	ShortRunLowGrayLevelEmphasis	0.03143978
wavelet-LLL	glrlm	GrayLevelVariance	469.0314528

wavelet-LLL	glrlm	LowGrayLevelRunEmphasis	0.031455362
wavelet-LLL	glrlm	GrayLevelNonUniformityNormalized	0.04148262
wavelet-LLL	glrlm	RunVariance	0.004391802
wavelet-LLL	glrlm	GrayLevelNonUniformity	1.445636716
wavelet-LLL	glrlm	LongRunEmphasis	1.013574661
wavelet-LLL	glrlm	ShortRunHighGrayLevelEmphasis	1936.267065
wavelet-LLL	glrlm	RunLengthNonUniformity	34.54751131
wavelet-LLL	glrlm	ShortRunEmphasis	0.996606335
wavelet-LLL	glrlm	LongRunHighGrayLevelEmphasis	1946.532902
wavelet-LLL	glrlm	RunPercentage	0.995604396
wavelet-LLL	glrlm	LongRunLowGrayLevelEmphasis	0.031517688
wavelet-LLL	glrlm	RunEntropy	4.714655932
wavelet-LLL	glrlm	HighGrayLevelRunEmphasis	1938.320233
wavelet-LLL	glrlm	RunLengthNonUniformityNormalized	0.991216396
wavelet-LLL	glszm	GrayLevelVariance	472.7897153
wavelet-LLL	glszm	ZoneVariance	0.056932966
wavelet-LLL	glszm	GrayLevelNonUniformityNormalized	0.039485767
wavelet-LLL	glszm	SizeZoneNonUniformityNormalized	0.886134068
wavelet-LLL	glszm	SizeZoneNonUniformity	29.24242424
wavelet-LLL	glszm	GrayLevelNonUniformity	1.303030303
wavelet-LLL	glszm	LargeAreaEmphasis	1.181818182
wavelet-LLL	glszm	SmallAreaHighGrayLevelEmphasis	1985.257576
wavelet-LLL	glszm	ZonePercentage	0.942857143
wavelet-LLL	glszm	LargeAreaLowGrayLevelEmphasis	0.033789934
wavelet-LLL	glszm	LargeAreaHighGrayLevelEmphasis	2122.757576
wavelet-LLL	glszm	HighGrayLevelZoneEmphasis	2012.757576
wavelet-LLL	glszm	SmallAreaEmphasis	0.954545455
wavelet-LLL	glszm	LowGrayLevelZoneEmphasis	0.03295514
wavelet-LLL	glszm	ZoneEntropy	4.801969877
wavelet-LLL	glszm	SmallAreaLowGrayLevelEmphasis	0.032746441
wavelet-LLL	ngtdm	Coarseness	0.043472852
wavelet-LLL	ngtdm	Complexity	13190.82664
wavelet-LLL	ngtdm	Strength	99.33890412
wavelet-LLL	ngtdm	Contrast	19.49839151
wavelet-LLL	ngtdm	Busyness	0.037463944
wavelet-HHL	gldm	GrayLevelVariance	12.07836735
wavelet-HHL	gldm	HighGrayLevelEmphasis	49.11428571
wavelet-HHL	gldm	DependenceEntropy	4.164299088
wavelet-HHL	gldm	DependenceNonUniformity	9.971428571
wavelet-HHL	gldm	GrayLevelNonUniformity	3.571428571
wavelet-HHL	gldm	SmallDependenceEmphasis	0.467460317
wavelet-HHL	gldm	SmallDependenceHighGrayLevelEmphasis	34.29186508
wavelet-HHL	gldm	DependenceNonUniformityNormalized	0.284897959
wavelet-HHL	gldm	LargeDependenceEmphasis	5.685714286
wavelet-HHL	gldm	LargeDependenceLowGrayLevelEmphasis	0.63122039
wavelet-HHL	gldm	DependenceVariance	1.093877551
wavelet-HHL	gldm	LargeDependenceHighGrayLevelEmphasis	167.1142857
wavelet-HHL	gldm	SmallDependenceLowGrayLevelEmphasis	0.033466329
wavelet-HHL	gldm	LowGrayLevelEmphasis	0.114904694
wavelet-HHL	glcm	JointAverage	6.071612435
wavelet-HHL	glcm	SumAverage	12.14322487
wavelet-HHL	glcm	JointEntropy	4.6342216
wavelet-HHL	glcm	ClusterShade	41.13217132
wavelet-HHL	glcm	MaximumProbability	0.069363961

wavelet-HHL	glcm	Idmn	0.900928586
wavelet-HHL	glcm	JointEnergy	0.043655379
wavelet-HHL	glcm	Contrast	26.16771728
wavelet-HHL	glcm	DifferenceEntropy	2.699006712
wavelet-HHL	glcm	InverseVariance	0.218991252
wavelet-HHL	glcm	DifferenceVariance	7.408522988
wavelet-HHL	glcm	Idn	0.801020078
wavelet-HHL	glcm	Idm	0.231622046
wavelet-HHL	glcm	Correlation	-0.024860229
wavelet-HHL	glcm	Autocorrelation	36.40158452
wavelet-HHL	glcm	SumEntropy	3.188967132
wavelet-HHL	glcm	SumSquares	12.49794016
wavelet-HHL	glcm	ClusterProminence	1635.488591
wavelet-HHL	glcm	Imc2	0.992249647
wavelet-HHL	glcm	Imc1	-0.635779358
wavelet-HHL	glcm	DifferenceAverage	3.987501591
wavelet-HHL	glcm	Id	0.324270263
wavelet-HHL	glcm	ClusterTendency	23.82404335
wavelet-HHL	firstorder	InterquartileRange	112.4398842
wavelet-HHL	firstorder	Skewness	0.535537317
wavelet-HHL	firstorder	Uniformity	0.102040816
wavelet-HHL	firstorder	Median	-3.957621768
wavelet-HHL	firstorder	Energy	268014.7496
wavelet-HHL	firstorder	RobustMeanAbsoluteDeviation	50.73380342
wavelet-HHL	firstorder	MeanAbsoluteDeviation	72.11921831
wavelet-HHL	firstorder	TotalEnergy	7236398.238
wavelet-HHL	firstorder	Maximum	203.6614502
wavelet-HHL	firstorder	RootMeanSquared	87.50750981
wavelet-HHL	firstorder	90Percentile	133.2469618
wavelet-HHL	firstorder	Minimum	-119.4187245
wavelet-HHL	firstorder	Entropy	3.475731503
wavelet-HHL	firstorder	Range	323.0801748
wavelet-HHL	firstorder	Variance	7442.07748
wavelet-HHL	firstorder	10Percentile	-84.78762946
wavelet-HHL	firstorder	Kurtosis	2.494136292
wavelet-HHL	firstorder	Mean	14.67946842
wavelet-HHL	glrlm	ShortRunLowGrayLevelEmphasis	0.1099564
wavelet-HHL	glrlm	GrayLevelVariance	12.30269462
wavelet-HHL	glrlm	LowGrayLevelRunEmphasis	0.11446916
wavelet-HHL	glrlm	GrayLevelNonUniformityNormalized	0.100376621
wavelet-HHL	glrlm	RunVariance	0.043248114
wavelet-HHL	glrlm	GrayLevelNonUniformity	3.359431939
wavelet-HHL	glrlm	LongRunEmphasis	1.145724585
wavelet-HHL	glrlm	ShortRunHighGrayLevelEmphasis	49.25192599
wavelet-HHL	glrlm	RunLengthNonUniformity	30.70793262
wavelet-HHL	glrlm	ShortRunEmphasis	0.963568854
wavelet-HHL	glrlm	LongRunHighGrayLevelEmphasis	54.06923889
wavelet-HHL	glrlm	RunPercentage	0.956043956
wavelet-HHL	glrlm	LongRunLowGrayLevelEmphasis	0.132520202
wavelet-HHL	glrlm	RunEntropy	3.619644146
wavelet-HHL	glrlm	HighGrayLevelRunEmphasis	50.21538857
wavelet-HHL	glrlm	RunLengthNonUniformityNormalized	0.913503771
wavelet-HHL	glszm	GrayLevelVariance	14.06
wavelet-HHL	glszm	ZoneVariance	1.5875

wavelet-HHL	glszm	GrayLevelNonUniformityNormalized	0.09
wavelet-HHL	glszm	SizeZoneNonUniformityNormalized	0.465
wavelet-HHL	glszm	SizeZoneNonUniformity	9.3
wavelet-HHL	glszm	GrayLevelNonUniformity	1.8
wavelet-HHL	glszm	LargeAreaEmphasis	4.65
wavelet-HHL	glszm	SmallAreaHighGrayLevelEmphasis	56.66394444
wavelet-HHL	glszm	ZonePercentage	0.571428571
wavelet-HHL	glszm	LargeAreaLowGrayLevelEmphasis	0.496643221
wavelet-HHL	glszm	LargeAreaHighGrayLevelEmphasis	155.95
wavelet-HHL	glszm	HighGrayLevelZoneEmphasis	65.9
wavelet-HHL	glszm	SmallAreaEmphasis	0.702611111
wavelet-HHL	glszm	LowGrayLevelZoneEmphasis	0.09757362
wavelet-HHL	glszm	ZoneEntropy	3.88418372
wavelet-HHL	glszm	SmallAreaLowGrayLevelEmphasis	0.038658895
wavelet-HHL	ngtdm	Coarseness	0.108175727
wavelet-HHL	ngtdm	Complexity	210.9316304
wavelet-HHL	ngtdm	Strength	6.879690563
wavelet-HHL	ngtdm	Contrast	0.485593579
wavelet-HHL	ngtdm	Busyness	0.19443968
wavelet-HLH	gldm	GrayLevelVariance	8.82122449
wavelet-HLH	gldm	HighGrayLevelEmphasis	64.85714286
wavelet-HLH	gldm	DependenceEntropy	4.286146588
wavelet-HLH	gldm	DependenceNonUniformity	10.48571429
wavelet-HLH	gldm	GrayLevelNonUniformity	4.314285714
wavelet-HLH	gldm	SmallDependenceEmphasis	0.503968254
wavelet-HLH	gldm	SmallDependenceHighGrayLevelEmphasis	38.79047619
wavelet-HLH	gldm	DependenceNonUniformityNormalized	0.299591837
wavelet-HLH	gldm	LargeDependenceEmphasis	6.428571429
wavelet-HLH	gldm	LargeDependenceLowGrayLevelEmphasis	0.18957805
wavelet-HLH	gldm	DependenceVariance	1.588571429
wavelet-HLH	gldm	LargeDependenceHighGrayLevelEmphasis	359.8857143
wavelet-HLH	gldm	SmallDependenceLowGrayLevelEmphasis	0.044707876
wavelet-HLH	gldm	LowGrayLevelEmphasis	0.057595381
wavelet-HLH	glcm	JointAverage	7.466076158
wavelet-HLH	glcm	SumAverage	14.93215232
wavelet-HLH	glcm	JointEntropy	4.519784623
wavelet-HLH	glcm	ClusterShade	13.22528463
wavelet-HLH	glcm	MaximumProbability	0.090046484
wavelet-HLH	glcm	Idmn	0.939177532
wavelet-HLH	glcm	JointEnergy	0.049236665
wavelet-HLH	glcm	Contrast	13.90998868
wavelet-HLH	glcm	DifferenceEntropy	2.597294302
wavelet-HLH	glcm	InverseVariance	0.268586909
wavelet-HLH	glcm	DifferenceVariance	4.309648586
wavelet-HLH	glcm	Idn	0.835881769
wavelet-HLH	glcm	Idm	0.266572259
wavelet-HLH	glcm	Correlation	0.032201852
wavelet-HLH	glcm	Autocorrelation	56.61489202
wavelet-HLH	glcm	SumEntropy	3.091645177
wavelet-HLH	glcm	SumSquares	7.375637795
wavelet-HLH	glcm	ClusterProminence	734.2004933
wavelet-HLH	glcm	Imc2	0.977938452
wavelet-HLH	glcm	Imc1	-0.52627319
wavelet-HLH	glcm	DifferenceAverage	3.015920679

wavelet-HLH	glcm	Id	0.359490255
wavelet-HLH	glcm	ClusterTendency	15.5925625
wavelet-HLH	firstorder	InterquartileRange	84.17655585
wavelet-HLH	firstorder	Skewness	0.278531541
wavelet-HLH	firstorder	Uniformity	0.123265306
wavelet-HLH	firstorder	Median	0.749414932
wavelet-HLH	firstorder	Energy	196963.1413
wavelet-HLH	firstorder	RobustMeanAbsoluteDeviation	38.45826227
wavelet-HLH	firstorder	MeanAbsoluteDeviation	59.4767245
wavelet-HLH	firstorder	TotalEnergy	5318004.814
wavelet-HLH	firstorder	Maximum	174.784112
wavelet-HLH	firstorder	RootMeanSquared	75.01678693
wavelet-HLH	firstorder	90Percentile	96.95534013
wavelet-HLH	firstorder	Minimum	-153.3077171
wavelet-HLH	firstorder	Entropy	3.257117662
wavelet-HLH	firstorder	Range	328.0918291
wavelet-HLH	firstorder	Variance	5627.203034
wavelet-HLH	firstorder	10Percentile	-92.87758844
wavelet-HLH	firstorder	Kurtosis	2.838055822
wavelet-HLH	firstorder	Mean	-0.561504865
wavelet-HLH	glrlm	ShortRunLowGrayLevelEmphasis	0.058303106
wavelet-HLH	glrlm	GrayLevelVariance	9.088395761
wavelet-HLH	glrlm	LowGrayLevelRunEmphasis	0.059213222
wavelet-HLH	glrlm	GrayLevelNonUniformityNormalized	0.120161424
wavelet-HLH	glrlm	RunVariance	0.050461371
wavelet-HLH	glrlm	GrayLevelNonUniformity	4.015002202
wavelet-HLH	glrlm	LongRunEmphasis	1.153684709
wavelet-HLH	glrlm	ShortRunHighGrayLevelEmphasis	63.47597952
wavelet-HLH	glrlm	RunLengthNonUniformity	30.54013349
wavelet-HLH	glrlm	ShortRunEmphasis	0.964168825
wavelet-HLH	glrlm	LongRunHighGrayLevelEmphasis	73.74188051
wavelet-HLH	glrlm	RunPercentage	0.953846154
wavelet-HLH	glrlm	LongRunLowGrayLevelEmphasis	0.063015561
wavelet-HLH	glrlm	RunEntropy	3.430397065
wavelet-HLH	glrlm	HighGrayLevelRunEmphasis	65.39655158
wavelet-HLH	glrlm	RunLengthNonUniformityNormalized	0.912639454
wavelet-HLH	glszm	GrayLevelVariance	12.8275
wavelet-HLH	glszm	ZoneVariance	2.3875
wavelet-HLH	glszm	GrayLevelNonUniformityNormalized	0.095
wavelet-HLH	glszm	SizeZoneNonUniformityNormalized	0.585
wavelet-HLH	glszm	SizeZoneNonUniformity	11.7
wavelet-HLH	glszm	GrayLevelNonUniformity	1.9
wavelet-HLH	glszm	LargeAreaEmphasis	5.45
wavelet-HLH	glszm	SmallAreaHighGrayLevelEmphasis	62.59583333
wavelet-HLH	glszm	ZonePercentage	0.571428571
wavelet-HLH	glszm	LargeAreaLowGrayLevelEmphasis	0.189686899
wavelet-HLH	glszm	LargeAreaHighGrayLevelEmphasis	303.2
wavelet-HLH	glszm	HighGrayLevelZoneEmphasis	74.45
wavelet-HLH	glszm	SmallAreaEmphasis	0.776388889
wavelet-HLH	glszm	LowGrayLevelZoneEmphasis	0.081670813
wavelet-HLH	glszm	ZoneEntropy	3.921928095
wavelet-HLH	glszm	SmallAreaLowGrayLevelEmphasis	0.075573428
wavelet-HLH	ngtdm	Coarseness	0.145966051
wavelet-HLH	ngtdm	Complexity	121.6813103

wavelet-HLH	ngtdm	Strength	8.465815954
wavelet-HLH	ngtdm	Contrast	0.271443618
wavelet-HLH	ngtdm	Busyness	0.091380253

Image type	Feature Class	Feature Name	2: RTSTRUCT: C99-Dnu-B_segment_ITV
general	info	BoundingBox	(1, 0, 3, 15, 13, 14)
general	info	EnabledImageTypes	{u'Original': {}, u'Wavelet': {}}
general	info	GeneralSettings	{'distances': [1], 'additionalInfo': True, 'force2D': False, 'interpolator': 'sitkBSpline', 'resampledPixelSpacing': [3.0], 'label': 1, 'normalizeScale': 1, 'normalize': False, 'force2Ddimension': 0, 'removeOutliers': None, 'minimumROISize': None, u'binWidth': 25.0, 'minimumROIDimensions': 1, u'correctMask': True, 'preCrop': False, u'symmetricalGLCM': True, 'resegmentRange': None, 'padDistance': 5}
general	info	ImageHash	1d096944131e4e1a0c9a38bc8ad9d42d5202ee5c
general	info	ImageSpacing	(0.9765625000000002, 0.9765625000000002, 2.0)
general	info	MaskHash	a7426d3f791e249534fae605229875e87312c255
general	info	NumpyVersion	1.13.1
general	info	PyWaveletVersion	0.5.2
general	info	SimpleITKVersion	1.1.0
general	info	Version	1.3.0.post102+g1b0eb74
general	info	VolumeNum	1
general	info	VoxelNum	688
original	shape	Maximum3DDiameter	50.28916384
original	shape	Maximum2DDiameterSlice	44.59820624
original	shape	Sphericity	0.699592721
original	shape	MinorAxis	34.04193959
original	shape	Elongation	0.86312351
original	shape	SurfaceVolumeRatio	0.261008574
original	shape	Volume	18576
original	shape	MajorAxis	39.44040362
original	shape	SurfaceArea	4848.495279
original	shape	Flatness	0.681583351
original	shape	LeastAxis	26.88192246
original	shape	Maximum2DDiameterColumn	40.80441153
original	shape	Maximum2DDiameterRow	45.79301257
original	gldm	GrayLevelVariance	165.1370483
original	gldm	HighGrayLevelEmphasis	1242.178779
original	gldm	DependenceEntropy	6.659780213
original	gldm	DependenceNonUniformity	149.2877907
original	gldm	GrayLevelNonUniformity	56.60755814
original	gldm	SmallDependenceEmphasis	0.474978518
original	gldm	SmallDependenceHighGrayLevelEmphasis	389.7137091
original	gldm	DependenceNonUniformityNormalized	0.216988068
original	gldm	LargeDependenceEmphasis	32.48546512
original	gldm	LargeDependenceLowGrayLevelEmphasis	0.038954679
original	gldm	DependenceVariance	16.57835316
original	gldm	LargeDependenceHighGrayLevelEmphasis	58501.29506

original	gldm	SmallDependenceLowGrayLevelEmphasis	0.007197101
original	gldm	LowGrayLevelEmphasis	0.010194441
original	glcm	JointAverage	34.72490243
original	glcm	SumAverage	69.44980487
original	glcm	JointEntropy	7.504284724
original	glcm	ClusterShade	-7771.291707
original	glcm	MaximumProbability	0.094534925
original	glcm	Idmn	0.929981757
original	glcm	JointEnergy	0.020507693
original	glcm	Contrast	200.5254135
original	glcm	DifferenceEntropy	4.381129951
original	glcm	InverseVariance	0.244485317
original	glcm	DifferenceVariance	115.0786914
original	glcm	Idn	0.861089505
original	glcm	Idm	0.286987469
original	glcm	Correlation	0.307836365
original	glcm	Autocorrelation	1250.609985
original	glcm	SumEntropy	5.303332192
original	glcm	SumSquares	144.8670955
original	glcm	ClusterProminence	450671.8274
original	glcm	Imc2	0.939383121
original	glcm	Imc1	-0.251629067
original	glcm	DifferenceAverage	9.174433722
original	glcm	Id	0.338842814
original	glcm	ClusterTendency	378.9429686
original	firstorder	InterquartileRange	479.75
original	firstorder	Skewness	-0.970513925
original	firstorder	Uniformity	0.082278428
original	firstorder	Median	-31.5
original	firstorder	Energy	103590872
original	firstorder	RobustMeanAbsoluteDeviation	212.6137295
original	firstorder	MeanAbsoluteDeviation	275.2077643
original	firstorder	TotalEnergy	2796953544
original	firstorder	Maximum	104
original	firstorder	RootMeanSquared	388.0310914
original	firstorder	90Percentile	49
original	firstorder	Minimum	-1020
original	firstorder	Entropy	4.586612813
original	firstorder	Range	1124
original	firstorder	Variance	103239.1135
original	firstorder	10Percentile	-762.6
original	firstorder	Kurtosis	2.493420593
original	firstorder	Mean	-217.5523256
original	glrlm	ShortRunLowGrayLevelEmphasis	0.01111478
original	glrlm	GrayLevelVariance	171.349303
original	glrlm	LowGrayLevelRunEmphasis	0.011283356
original	glrlm	GrayLevelNonUniformityNormalized	0.061551526
original	glrlm	RunVariance	0.19766371
original	glrlm	GrayLevelNonUniformity	37.51311884
original	glrlm	LongRunEmphasis	1.475904704
original	glrlm	ShortRunHighGrayLevelEmphasis	1036.756742
original	glrlm	RunLengthNonUniformity	499.8483276

original	glrlm	ShortRunEmphasis	0.923008297
original	glrlm	LongRunHighGrayLevelEmphasis	2022.963071
original	glrlm	RunPercentage	0.885062612
original	glrlm	LongRunLowGrayLevelEmphasis	0.012049574
original	glrlm	RunEntropy	5.225062914
original	glrlm	HighGrayLevelRunEmphasis	1172.440629
original	glrlm	RunLengthNonUniformityNormalized	0.82045303
original	glszm	GrayLevelVariance	159.3437981
original	glszm	ZoneVariance	84.6255942
original	glszm	GrayLevelNonUniformityNormalized	0.025700215
original	glszm	SizeZoneNonUniformityNormalized	0.730275982
original	glszm	SizeZoneNonUniformity	249.754386
original	glszm	GrayLevelNonUniformity	8.789473684
original	glszm	LargeAreaEmphasis	88.67251462
original	glszm	SmallAreaHighGrayLevelEmphasis	675.5192492
original	glszm	ZonePercentage	0.497093023
original	glszm	LargeAreaLowGrayLevelEmphasis	0.075384201
original	glszm	LargeAreaHighGrayLevelEmphasis	161398.6871
original	glszm	HighGrayLevelZoneEmphasis	802.9005848
original	glszm	SmallAreaEmphasis	0.877021337
original	glszm	LowGrayLevelZoneEmphasis	0.01627145
original	glszm	ZoneEntropy	5.975339245
original	glszm	SmallAreaLowGrayLevelEmphasis	0.01354055
original	ngtdm	Coarseness	0.003654249
original	ngtdm	Complexity	7188.973889
original	ngtdm	Strength	7.552206742
original	ngtdm	Contrast	1.20974721
original	ngtdm	Busyness	0.128455928
wavelet-HHH	gldm	GrayLevelVariance	22.95641014
wavelet-HHH	gldm	HighGrayLevelEmphasis	441.0043605
wavelet-HHH	gldm	DependenceEntropy	6.206621174
wavelet-HHH	gldm	DependenceNonUniformity	158.3953488
wavelet-HHH	gldm	GrayLevelNonUniformity	48.86627907
wavelet-HHH	gldm	SmallDependenceEmphasis	0.444377109
wavelet-HHH	gldm	SmallDependenceHighGrayLevelEmphasis	202.8364574
wavelet-HHH	gldm	DependenceNonUniformityNormalized	0.230225798
wavelet-HHH	gldm	LargeDependenceEmphasis	10.76453488
wavelet-HHH	gldm	LargeDependenceLowGrayLevelEmphasis	0.028096705
wavelet-HHH	gldm	DependenceVariance	3.858639805
wavelet-HHH	gldm	LargeDependenceHighGrayLevelEmphasis	4622.443314
wavelet-HHH	gldm	SmallDependenceLowGrayLevelEmphasis	0.003032203
wavelet-HHH	gldm	LowGrayLevelEmphasis	0.004455463
wavelet-HHH	glcm	JointAverage	20.43436251
wavelet-HHH	glcm	SumAverage	40.86872501
wavelet-HHH	glcm	JointEntropy	7.689528828
wavelet-HHH	glcm	ClusterShade	15.61238113
wavelet-HHH	glcm	MaximumProbability	0.029039705
wavelet-HHH	glcm	Idmn	0.979948562
wavelet-HHH	glcm	JointEnergy	0.007582828
wavelet-HHH	glcm	Contrast	42.85325181
wavelet-HHH	glcm	DifferenceEntropy	3.7237518
wavelet-HHH	glcm	InverseVariance	0.228305738

wavelet-HHH	glcm	DifferenceVariance	20.02372575
wavelet-HHH	glcm	Idn	0.909298199
wavelet-HHH	glcm	Idm	0.220129192
wavelet-HHH	glcm	Correlation	-0.03728522
wavelet-HHH	glcm	Autocorrelation	416.7494577
wavelet-HHH	glcm	SumEntropy	4.557036536
wavelet-HHH	glcm	SumSquares	20.61039079
wavelet-HHH	glcm	ClusterProminence	7901.94063
wavelet-HHH	glcm	Imc2	0.80486553
wavelet-HHH	glcm	Imc1	-0.127396849
wavelet-HHH	glcm	DifferenceAverage	4.752324129
wavelet-HHH	glcm	Id	0.307606032
wavelet-HHH	glcm	ClusterTendency	39.58831133
wavelet-HHH	firstorder	InterquartileRange	120.698204
wavelet-HHH	firstorder	Skewness	0.17317451
wavelet-HHH	firstorder	Uniformity	0.071026568
wavelet-HHH	firstorder	Median	0.618667368
wavelet-HHH	firstorder	Energy	9756527.574
wavelet-HHH	firstorder	RobustMeanAbsoluteDeviation	52.87116595
wavelet-HHH	firstorder	MeanAbsoluteDeviation	86.08480275
wavelet-HHH	firstorder	TotalEnergy	263426244.5
wavelet-HHH	firstorder	Maximum	577.1334812
wavelet-HHH	firstorder	RootMeanSquared	119.0840014
wavelet-HHH	firstorder	90Percentile	135.6444107
wavelet-HHH	firstorder	Minimum	-479.9298527
wavelet-HHH	firstorder	Entropy	4.202792289
wavelet-HHH	firstorder	Range	1057.063334
wavelet-HHH	firstorder	Variance	14178.02556
wavelet-HHH	firstorder	10Percentile	-143.2588623
wavelet-HHH	firstorder	Kurtosis	5.13456101
wavelet-HHH	firstorder	Mean	-1.724478444
wavelet-HHH	glrlm	ShortRunLowGrayLevelEmphasis	0.004474134
wavelet-HHH	glrlm	GrayLevelVariance	24.13191255
wavelet-HHH	glrlm	LowGrayLevelRunEmphasis	0.00458818
wavelet-HHH	glrlm	GrayLevelNonUniformityNormalized	0.067485936
wavelet-HHH	glrlm	RunVariance	0.07762119
wavelet-HHH	glrlm	GrayLevelNonUniformity	43.5252346
wavelet-HHH	glrlm	LongRunEmphasis	1.215984117
wavelet-HHH	glrlm	ShortRunHighGrayLevelEmphasis	422.1968698
wavelet-HHH	glrlm	RunLengthNonUniformity	571.8668491
wavelet-HHH	glrlm	ShortRunEmphasis	0.954071954
wavelet-HHH	glrlm	LongRunHighGrayLevelEmphasis	534.3730076
wavelet-HHH	glrlm	RunPercentage	0.937388193
wavelet-HHH	glrlm	LongRunLowGrayLevelEmphasis	0.005120056
wavelet-HHH	glrlm	RunEntropy	4.556268949
wavelet-HHH	glrlm	HighGrayLevelRunEmphasis	441.8574547
wavelet-HHH	glrlm	RunLengthNonUniformityNormalized	0.886576736
wavelet-HHH	glszm	GrayLevelVariance	39.65781619
wavelet-HHH	glszm	ZoneVariance	29.96966074
wavelet-HHH	glszm	GrayLevelNonUniformityNormalized	0.047562545
wavelet-HHH	glszm	SizeZoneNonUniformityNormalized	0.557817106
wavelet-HHH	glszm	SizeZoneNonUniformity	184.6374622

wavelet-HHH	glszm	GrayLevelNonUniformity	15.74320242
wavelet-HHH	glszm	LargeAreaEmphasis	34.29003021
wavelet-HHH	glszm	SmallAreaHighGrayLevelEmphasis	357.4156062
wavelet-HHH	glszm	ZonePercentage	0.481104651
wavelet-HHH	glszm	LargeAreaLowGrayLevelEmphasis	0.085433886
wavelet-HHH	glszm	LargeAreaHighGrayLevelEmphasis	14711.08459
wavelet-HHH	glszm	HighGrayLevelZoneEmphasis	453.5528701
wavelet-HHH	glszm	SmallAreaEmphasis	0.774398695
wavelet-HHH	glszm	LowGrayLevelZoneEmphasis	0.006543449
wavelet-HHH	glszm	ZoneEntropy	5.739886651
wavelet-HHH	glszm	SmallAreaLowGrayLevelEmphasis	0.005911951
wavelet-HHH	ngtdm	Coarseness	0.01019009
wavelet-HHH	ngtdm	Complexity	1935.894621
wavelet-HHH	ngtdm	Strength	4.037421682
wavelet-HHH	ngtdm	Contrast	0.133954926
wavelet-HHH	ngtdm	Busyness	0.106301542
wavelet-HLL	gldm	GrayLevelVariance	89.60829967
wavelet-HLL	gldm	HighGrayLevelEmphasis	1165.696221
wavelet-HLL	gldm	DependenceEntropy	6.732762642
wavelet-HLL	gldm	DependenceNonUniformity	218.7296512
wavelet-HLL	gldm	GrayLevelNonUniformity	23.59593023
wavelet-HLL	gldm	SmallDependenceEmphasis	0.547529657
wavelet-HLL	gldm	SmallDependenceHighGrayLevelEmphasis	636.1253224
wavelet-HLL	gldm	DependenceNonUniformityNormalized	0.317921005
wavelet-HLL	gldm	LargeDependenceEmphasis	7.212209302
wavelet-HLL	gldm	LargeDependenceLowGrayLevelEmphasis	0.008964148
wavelet-HLL	gldm	DependenceVariance	2.770407991
wavelet-HLL	gldm	LargeDependenceHighGrayLevelEmphasis	8553.75436
wavelet-HLL	gldm	SmallDependenceLowGrayLevelEmphasis	0.00238463
wavelet-HLL	gldm	LowGrayLevelEmphasis	0.002931125
wavelet-HLL	glcm	JointAverage	32.40308435
wavelet-HLL	glcm	SumAverage	64.8061687
wavelet-HLL	glcm	JointEntropy	8.881690168
wavelet-HLL	glcm	ClusterShade	-547.2447622
wavelet-HLL	glcm	MaximumProbability	0.018090279
wavelet-HLL	glcm	Idmn	0.966567558
wavelet-HLL	glcm	JointEnergy	0.00301932
wavelet-HLL	glcm	Contrast	127.2423908
wavelet-HLL	glcm	DifferenceEntropy	4.40371927
wavelet-HLL	glcm	InverseVariance	0.158746337
wavelet-HLL	glcm	DifferenceVariance	59.70860485
wavelet-HLL	glcm	Idn	0.887266365
wavelet-HLL	glcm	Idm	0.152924515
wavelet-HLL	glcm	Correlation	0.209246749
wavelet-HLL	glcm	Autocorrelation	1068.102779
wavelet-HLL	glcm	SumEntropy	5.664199266
wavelet-HLL	glcm	SumSquares	81.71884363
wavelet-HLL	glcm	ClusterProminence	144837.5155
wavelet-HLL	glcm	Imc2	0.963056252
wavelet-HLL	glcm	Imc1	-0.258509453
wavelet-HLL	glcm	DifferenceAverage	8.056440105
wavelet-HLL	glcm	Id	0.23564152

wavelet-HLL	glcm	ClusterTendency	199.6329837
wavelet-HLL	firstorder	InterquartileRange	285.6655496
wavelet-HLL	firstorder	Skewness	-0.262819608
wavelet-HLL	firstorder	Uniformity	0.03429641
wavelet-HLL	firstorder	Median	-26.18652819
wavelet-HLL	firstorder	Energy	39766737.69
wavelet-HLL	firstorder	RobustMeanAbsoluteDeviation	121.0781227
wavelet-HLL	firstorder	MeanAbsoluteDeviation	182.5793777
wavelet-HLL	firstorder	TotalEnergy	1073701918
wavelet-HLL	firstorder	Maximum	553.5307785
wavelet-HLL	firstorder	RootMeanSquared	240.4173264
wavelet-HLL	firstorder	90Percentile	256.6845998
wavelet-HLL	firstorder	Minimum	-832.3926514
wavelet-HLL	firstorder	Entropy	5.178235877
wavelet-HLL	firstorder	Range	1385.92343
wavelet-HLL	firstorder	Variance	55972.22999
wavelet-HLL	firstorder	10Percentile	-343.4260794
wavelet-HLL	firstorder	Kurtosis	3.170600452
wavelet-HLL	firstorder	Mean	-42.75816688
wavelet-HLL	glrlm	ShortRunLowGrayLevelEmphasis	0.002980621
wavelet-HLL	glrlm	GrayLevelVariance	91.81876061
wavelet-HLL	glrlm	LowGrayLevelRunEmphasis	0.00301445
wavelet-HLL	glrlm	GrayLevelNonUniformityNormalized	0.033104346
wavelet-HLL	glrlm	RunVariance	0.050261299
wavelet-HLL	glrlm	GrayLevelNonUniformity	21.80779499
wavelet-HLL	glrlm	LongRunEmphasis	1.141828634
wavelet-HLL	glrlm	ShortRunHighGrayLevelEmphasis	1128.330653
wavelet-HLL	glrlm	RunLengthNonUniformity	607.0461423
wavelet-HLL	glrlm	ShortRunEmphasis	0.968739102
wavelet-HLL	glrlm	LongRunHighGrayLevelEmphasis	1332.393648
wavelet-HLL	glrlm	RunPercentage	0.95740161
wavelet-HLL	glrlm	LongRunLowGrayLevelEmphasis	0.003163984
wavelet-HLL	glrlm	RunEntropy	5.402778578
wavelet-HLL	glrlm	HighGrayLevelRunEmphasis	1165.130519
wavelet-HLL	glrlm	RunLengthNonUniformityNormalized	0.921297229
wavelet-HLL	glszm	GrayLevelVariance	113.0811006
wavelet-HLL	glszm	ZoneVariance	6.186103143
wavelet-HLL	glszm	GrayLevelNonUniformityNormalized	0.027538132
wavelet-HLL	glszm	SizeZoneNonUniformityNormalized	0.565861712
wavelet-HLL	glszm	SizeZoneNonUniformity	241.6229508
wavelet-HLL	glszm	GrayLevelNonUniformity	11.7587822
wavelet-HLL	glszm	LargeAreaEmphasis	8.782201405
wavelet-HLL	glszm	SmallAreaHighGrayLevelEmphasis	906.5602853
wavelet-HLL	glszm	ZonePercentage	0.620639535
wavelet-HLL	glszm	LargeAreaLowGrayLevelEmphasis	0.011239526
wavelet-HLL	glszm	LargeAreaHighGrayLevelEmphasis	10355.0281
wavelet-HLL	glszm	HighGrayLevelZoneEmphasis	1163.782201
wavelet-HLL	glszm	SmallAreaEmphasis	0.780544391
wavelet-HLL	glszm	LowGrayLevelZoneEmphasis	0.004018995
wavelet-HLL	glszm	ZoneEntropy	6.338160304
wavelet-HLL	glszm	SmallAreaLowGrayLevelEmphasis	0.003713428
wavelet-HLL	ngtdm	Coarseness	0.010844496

wavelet-HLL	ngtdm	Complexity	5769.136086
wavelet-HLL	ngtdm	Strength	8.609393416
wavelet-HLL	ngtdm	Contrast	0.387442349
wavelet-HLL	ngtdm	Busyness	0.052875562
wavelet-LHL	gldm	GrayLevelVariance	141.9944227
wavelet-LHL	gldm	HighGrayLevelEmphasis	1724.831395
wavelet-LHL	gldm	DependenceEntropy	6.814849561
wavelet-LHL	gldm	DependenceNonUniformity	262.2761628
wavelet-LHL	gldm	GrayLevelNonUniformity	21.05813953
wavelet-LHL	gldm	SmallDependenceEmphasis	0.631869268
wavelet-LHL	gldm	SmallDependenceHighGrayLevelEmphasis	1123.702455
wavelet-LHL	gldm	DependenceNonUniformityNormalized	0.381215353
wavelet-LHL	gldm	LargeDependenceEmphasis	5.75872093
wavelet-LHL	gldm	LargeDependenceLowGrayLevelEmphasis	0.008781003
wavelet-LHL	gldm	DependenceVariance	2.188378853
wavelet-LHL	gldm	LargeDependenceHighGrayLevelEmphasis	9759.607558
wavelet-LHL	gldm	SmallDependenceLowGrayLevelEmphasis	0.005480158
wavelet-LHL	gldm	LowGrayLevelEmphasis	0.005747524
wavelet-LHL	glcm	JointAverage	39.82821663
wavelet-LHL	glcm	SumAverage	79.65643325
wavelet-LHL	glcm	JointEntropy	9.027676434
wavelet-LHL	glcm	ClusterShade	2217.944823
wavelet-LHL	glcm	MaximumProbability	0.013738818
wavelet-LHL	glcm	Idmn	0.975143566
wavelet-LHL	glcm	JointEnergy	0.0026797
wavelet-LHL	glcm	Contrast	230.4413753
wavelet-LHL	glcm	DifferenceEntropy	4.745197759
wavelet-LHL	glcm	InverseVariance	0.140417709
wavelet-LHL	glcm	DifferenceVariance	115.7704303
wavelet-LHL	glcm	Idn	0.904739693
wavelet-LHL	glcm	Idm	0.130955642
wavelet-LHL	glcm	Correlation	0.117466129
wavelet-LHL	glcm	Autocorrelation	1603.365017
wavelet-LHL	glcm	SumEntropy	5.847386423
wavelet-LHL	glcm	SumSquares	132.2593879
wavelet-LHL	glcm	ClusterProminence	476372.7915
wavelet-LHL	glcm	Imc2	0.98111265
wavelet-LHL	glcm	Imc1	-0.30913184
wavelet-LHL	glcm	DifferenceAverage	10.49090099
wavelet-LHL	glcm	Id	0.208967038
wavelet-LHL	glcm	ClusterTendency	298.5961765
wavelet-LHL	firstorder	InterquartileRange	293.0515419
wavelet-LHL	firstorder	Skewness	0.23734304
wavelet-LHL	firstorder	Uniformity	0.030607761
wavelet-LHL	firstorder	Median	-26.98051922
wavelet-LHL	firstorder	Energy	62241606.78
wavelet-LHL	firstorder	RobustMeanAbsoluteDeviation	135.1904459
wavelet-LHL	firstorder	MeanAbsoluteDeviation	218.0864985
wavelet-LHL	firstorder	TotalEnergy	1680523383
wavelet-LHL	firstorder	Maximum	1216.559916
wavelet-LHL	firstorder	RootMeanSquared	300.7780772
wavelet-LHL	firstorder	90Percentile	306.5403253

wavelet-LHL	firstorder	Minimum	-1017.035666
wavelet-LHL	firstorder	Entropy	5.43780966
wavelet-LHL	firstorder	Range	2233.595582
wavelet-LHL	firstorder	Variance	88655.92976
wavelet-LHL	firstorder	10Percentile	-409.5932289
wavelet-LHL	firstorder	Kurtosis	4.489853013
wavelet-LHL	firstorder	Mean	-42.56197777
wavelet-LHL	glrlm	ShortRunLowGrayLevelEmphasis	0.005911226
wavelet-LHL	glrlm	GrayLevelVariance	145.8187803
wavelet-LHL	glrlm	LowGrayLevelRunEmphasis	0.00592787
wavelet-LHL	glrlm	GrayLevelNonUniformityNormalized	0.029410127
wavelet-LHL	glrlm	RunVariance	0.041604278
wavelet-LHL	glrlm	GrayLevelNonUniformity	19.54334401
wavelet-LHL	glrlm	LongRunEmphasis	1.114089582
wavelet-LHL	glrlm	ShortRunHighGrayLevelEmphasis	1686.27587
wavelet-LHL	glrlm	RunLengthNonUniformity	623.207696
wavelet-LHL	glrlm	ShortRunEmphasis	0.975433936
wavelet-LHL	glrlm	LongRunHighGrayLevelEmphasis	1916.401771
wavelet-LHL	glrlm	RunPercentage	0.96578712
wavelet-LHL	glrlm	LongRunLowGrayLevelEmphasis	0.00600447
wavelet-LHL	glrlm	RunEntropy	5.630633342
wavelet-LHL	glrlm	HighGrayLevelRunEmphasis	1727.117458
wavelet-LHL	glrlm	RunLengthNonUniformityNormalized	0.937718179
wavelet-LHL	glszm	GrayLevelVariance	186.8594334
wavelet-LHL	glszm	ZoneVariance	4.015153691
wavelet-LHL	glszm	GrayLevelNonUniformityNormalized	0.023125539
wavelet-LHL	glszm	SizeZoneNonUniformityNormalized	0.672050057
wavelet-LHL	glszm	SizeZoneNonUniformity	317.2076271
wavelet-LHL	glszm	GrayLevelNonUniformity	10.91525424
wavelet-LHL	glszm	LargeAreaEmphasis	6.139830508
wavelet-LHL	glszm	SmallAreaHighGrayLevelEmphasis	1515.689299
wavelet-LHL	glszm	ZonePercentage	0.686046512
wavelet-LHL	glszm	LargeAreaLowGrayLevelEmphasis	0.011210679
wavelet-LHL	glszm	LargeAreaHighGrayLevelEmphasis	10563.41949
wavelet-LHL	glszm	HighGrayLevelZoneEmphasis	1763.555085
wavelet-LHL	glszm	SmallAreaEmphasis	0.845148903
wavelet-LHL	glszm	LowGrayLevelZoneEmphasis	0.008055703
wavelet-LHL	glszm	ZoneEntropy	6.435990297
wavelet-LHL	glszm	SmallAreaLowGrayLevelEmphasis	0.00793092
wavelet-LHL	ngtdm	Coarseness	0.010911921
wavelet-LHL	ngtdm	Complexity	15462.33745
wavelet-LHL	ngtdm	Strength	17.27344513
wavelet-LHL	ngtdm	Contrast	0.440148988
wavelet-LHL	ngtdm	Busyness	0.029332437
wavelet-LHH	gldm	GrayLevelVariance	59.85275825
wavelet-LHH	gldm	HighGrayLevelEmphasis	1059.44186
wavelet-LHH	gldm	DependenceEntropy	6.404701498
wavelet-LHH	gldm	DependenceNonUniformity	198.4331395
wavelet-LHH	gldm	GrayLevelNonUniformity	32.77325581
wavelet-LHH	gldm	SmallDependenceEmphasis	0.525247565
wavelet-LHH	gldm	SmallDependenceHighGrayLevelEmphasis	601.5946386
wavelet-LHH	gldm	DependenceNonUniformityNormalized	0.288420261

wavelet-LHH	gldm	LargeDependenceEmphasis	6.965116279
wavelet-LHH	gldm	LargeDependenceLowGrayLevelEmphasis	0.00931343
wavelet-LHH	gldm	DependenceVariance	2.19901805
wavelet-LHH	gldm	LargeDependenceHighGrayLevelEmphasis	6843.921512
wavelet-LHH	gldm	SmallDependenceLowGrayLevelEmphasis	0.002254471
wavelet-LHH	gldm	LowGrayLevelEmphasis	0.002813671
wavelet-LHH	glcm	JointAverage	31.55303179
wavelet-LHH	glcm	SumAverage	63.10606359
wavelet-LHH	glcm	JointEntropy	8.444834618
wavelet-LHH	glcm	ClusterShade	126.6298118
wavelet-LHH	glcm	MaximumProbability	0.019074644
wavelet-LHH	glcm	Idmn	0.979586717
wavelet-LHH	glcm	JointEnergy	0.004314229
wavelet-LHH	glcm	Contrast	105.5593936
wavelet-LHH	glcm	DifferenceEntropy	4.279128868
wavelet-LHH	glcm	InverseVariance	0.177295508
wavelet-LHH	glcm	DifferenceVariance	51.7131544
wavelet-LHH	glcm	Idn	0.910910135
wavelet-LHH	glcm	Idm	0.167527215
wavelet-LHH	glcm	Correlation	0.001005133
wavelet-LHH	glcm	Autocorrelation	995.7097494
wavelet-LHH	glcm	SumEntropy	5.170260695
wavelet-LHH	glcm	SumSquares	52.88713573
wavelet-LHH	glcm	ClusterProminence	66363.52679
wavelet-LHH	glcm	Imc2	0.925737082
wavelet-LHH	glcm	Imc1	-0.206870151
wavelet-LHH	glcm	DifferenceAverage	7.255056762
wavelet-LHH	glcm	Id	0.251132485
wavelet-LHH	glcm	ClusterTendency	105.9891494
wavelet-LHH	firstorder	InterquartileRange	180.6859106
wavelet-LHH	firstorder	Skewness	0.155448954
wavelet-LHH	firstorder	Uniformity	0.047635546
wavelet-LHH	firstorder	Median	-5.247357554
wavelet-LHH	firstorder	Energy	25770202.53
wavelet-LHH	firstorder	RobustMeanAbsoluteDeviation	80.57998324
wavelet-LHH	firstorder	MeanAbsoluteDeviation	136.8110336
wavelet-LHH	firstorder	TotalEnergy	695795468.2
wavelet-LHH	firstorder	Maximum	907.4676395
wavelet-LHH	firstorder	RootMeanSquared	193.5373083
wavelet-LHH	firstorder	90Percentile	230.723806
wavelet-LHH	firstorder	Minimum	-772.5918958
wavelet-LHH	firstorder	Entropy	4.81332802
wavelet-LHH	firstorder	Range	1680.059535
wavelet-LHH	firstorder	Variance	37446.69402
wavelet-LHH	firstorder	10Percentile	-218.6208859
wavelet-LHH	firstorder	Kurtosis	5.244701343
wavelet-LHH	firstorder	Mean	3.161597153
wavelet-LHH	glrlm	ShortRunLowGrayLevelEmphasis	0.002856931
wavelet-LHH	glrlm	GrayLevelVariance	61.93056063
wavelet-LHH	glrlm	LowGrayLevelRunEmphasis	0.002894768
wavelet-LHH	glrlm	GrayLevelNonUniformityNormalized	0.045748604
wavelet-LHH	glrlm	RunVariance	0.054644811

wavelet-LHH	glrlm	GrayLevelNonUniformity	30.04728528
wavelet-LHH	glrlm	LongRunEmphasis	1.152959327
wavelet-LHH	glrlm	ShortRunHighGrayLevelEmphasis	1031.33231
wavelet-LHH	glrlm	RunLengthNonUniformity	601.4039257
wavelet-LHH	glrlm	ShortRunEmphasis	0.966400162
wavelet-LHH	glrlm	LongRunHighGrayLevelEmphasis	1211.908457
wavelet-LHH	glrlm	RunPercentage	0.954494633
wavelet-LHH	glrlm	LongRunLowGrayLevelEmphasis	0.003065332
wavelet-LHH	glrlm	RunEntropy	5.073721177
wavelet-LHH	glrlm	HighGrayLevelRunEmphasis	1063.837109
wavelet-LHH	glrlm	RunLengthNonUniformityNormalized	0.915502256
wavelet-LHH	glszm	GrayLevelVariance	87.46173076
wavelet-LHH	glszm	ZoneVariance	12.50928007
wavelet-LHH	glszm	GrayLevelNonUniformityNormalized	0.034026919
wavelet-LHH	glszm	SizeZoneNonUniformityNormalized	0.592586046
wavelet-LHH	glszm	SizeZoneNonUniformity	235.8492462
wavelet-LHH	glszm	GrayLevelNonUniformity	13.54271357
wavelet-LHH	glszm	LargeAreaEmphasis	15.49748744
wavelet-LHH	glszm	SmallAreaHighGrayLevelEmphasis	934.1804735
wavelet-LHH	glszm	ZonePercentage	0.578488372
wavelet-LHH	glszm	LargeAreaLowGrayLevelEmphasis	0.019303865
wavelet-LHH	glszm	LargeAreaHighGrayLevelEmphasis	15177.63819
wavelet-LHH	glszm	HighGrayLevelZoneEmphasis	1128.577889
wavelet-LHH	glszm	SmallAreaEmphasis	0.797530072
wavelet-LHH	glszm	LowGrayLevelZoneEmphasis	0.004025166
wavelet-LHH	glszm	ZoneEntropy	6.068238024
wavelet-LHH	glszm	SmallAreaLowGrayLevelEmphasis	0.003762281
wavelet-LHH	ngtdm	Coarseness	0.010438203
wavelet-LHH	ngtdm	Complexity	5878.041734
wavelet-LHH	ngtdm	Strength	8.396151829
wavelet-LHH	ngtdm	Contrast	0.262772743
wavelet-LHH	ngtdm	Busyness	0.049724134
wavelet-LLH	gldm	GrayLevelVariance	139.7643912
wavelet-LLH	gldm	HighGrayLevelEmphasis	2720.936047
wavelet-LLH	gldm	DependenceEntropy	6.844033012
wavelet-LLH	gldm	DependenceNonUniformity	243.5901163
wavelet-LLH	gldm	GrayLevelNonUniformity	21.10755814
wavelet-LLH	gldm	SmallDependenceEmphasis	0.605038747
wavelet-LLH	gldm	SmallDependenceHighGrayLevelEmphasis	1658.645493
wavelet-LLH	gldm	DependenceNonUniformityNormalized	0.354055402
wavelet-LLH	gldm	LargeDependenceEmphasis	5.098837209
wavelet-LLH	gldm	LargeDependenceLowGrayLevelEmphasis	0.003859152
wavelet-LLH	gldm	DependenceVariance	1.495462074
wavelet-LLH	gldm	LargeDependenceHighGrayLevelEmphasis	13936.125
wavelet-LLH	gldm	SmallDependenceLowGrayLevelEmphasis	0.002127905
wavelet-LLH	gldm	LowGrayLevelEmphasis	0.002287633
wavelet-LLH	glcm	JointAverage	50.8294092
wavelet-LLH	glcm	SumAverage	101.6588184
wavelet-LLH	glcm	JointEntropy	9.067416464
wavelet-LLH	glcm	ClusterShade	714.4870104
wavelet-LLH	glcm	MaximumProbability	0.010973052
wavelet-LLH	glcm	Idmn	0.978472555

wavelet-LLH	glcm	JointEnergy	0.00246499
wavelet-LLH	glcm	Contrast	214.8747359
wavelet-LLH	glcm	DifferenceEntropy	4.720546343
wavelet-LLH	glcm	InverseVariance	0.132635701
wavelet-LLH	glcm	DifferenceVariance	105.5797828
wavelet-LLH	glcm	Idn	0.909500698
wavelet-LLH	glcm	Idm	0.127129954
wavelet-LLH	glcm	Correlation	0.133957297
wavelet-LLH	glcm	Autocorrelation	2601.523223
wavelet-LLH	glcm	SumEntropy	5.850752069
wavelet-LLH	glcm	SumSquares	125.3013617
wavelet-LLH	glcm	ClusterProminence	409240.3738
wavelet-LLH	glcm	Imc2	0.977445443
wavelet-LLH	glcm	Imc1	-0.293964722
wavelet-LLH	glcm	DifferenceAverage	10.24993366
wavelet-LLH	glcm	Id	0.206498285
wavelet-LLH	glcm	ClusterTendency	286.3307109
wavelet-LLH	firstorder	InterquartileRange	294.0494826
wavelet-LLH	firstorder	Skewness	0.049198306
wavelet-LLH	firstorder	Uniformity	0.03067959
wavelet-LLH	firstorder	Median	-31.95347876
wavelet-LLH	firstorder	Energy	61270285.44
wavelet-LLH	firstorder	RobustMeanAbsoluteDeviation	128.8357071
wavelet-LLH	firstorder	MeanAbsoluteDeviation	212.4208445
wavelet-LLH	firstorder	TotalEnergy	1654297707
wavelet-LLH	firstorder	Maximum	1028.306903
wavelet-LLH	firstorder	RootMeanSquared	298.4219286
wavelet-LLH	firstorder	90Percentile	283.0095648
wavelet-LLH	firstorder	Minimum	-1293.384378
wavelet-LLH	firstorder	Entropy	5.408447765
wavelet-LLH	firstorder	Range	2321.691281
wavelet-LLH	firstorder	Variance	87277.39196
wavelet-LLH	firstorder	10Percentile	-387.1273936
wavelet-LLH	firstorder	Kurtosis	4.935686822
wavelet-LLH	firstorder	Mean	-42.16936666
wavelet-LLH	glrlm	ShortRunLowGrayLevelEmphasis	0.002345813
wavelet-LLH	glrlm	GrayLevelVariance	143.4194453
wavelet-LLH	glrlm	LowGrayLevelRunEmphasis	0.002355916
wavelet-LLH	glrlm	GrayLevelNonUniformityNormalized	0.029882327
wavelet-LLH	glrlm	RunVariance	0.039422903
wavelet-LLH	glrlm	GrayLevelNonUniformity	19.85234425
wavelet-LLH	glrlm	LongRunEmphasis	1.112984449
wavelet-LLH	glrlm	ShortRunHighGrayLevelEmphasis	2651.785636
wavelet-LLH	glrlm	RunLengthNonUniformity	621.2392761
wavelet-LLH	glrlm	ShortRunEmphasis	0.974378665
wavelet-LLH	glrlm	LongRunHighGrayLevelEmphasis	3029.299607
wavelet-LLH	glrlm	RunPercentage	0.965451699
wavelet-LLH	glrlm	LongRunLowGrayLevelEmphasis	0.002399898
wavelet-LLH	glrlm	RunEntropy	5.600052574
wavelet-LLH	glrlm	HighGrayLevelRunEmphasis	2721.06325
wavelet-LLH	glrlm	RunLengthNonUniformityNormalized	0.934919554
wavelet-LLH	glszm	GrayLevelVariance	187.6343287

wavelet-LLH	glszm	ZoneVariance	3.043027136
wavelet-LLH	glszm	GrayLevelNonUniformityNormalized	0.023870211
wavelet-LLH	glszm	SizeZoneNonUniformityNormalized	0.644087507
wavelet-LLH	glszm	SizeZoneNonUniformity	295.6361656
wavelet-LLH	glszm	GrayLevelNonUniformity	10.95642702
wavelet-LLH	glszm	LargeAreaEmphasis	5.289760349
wavelet-LLH	glszm	SmallAreaHighGrayLevelEmphasis	2274.323653
wavelet-LLH	glszm	ZonePercentage	0.667151163
wavelet-LLH	glszm	LargeAreaLowGrayLevelEmphasis	0.004848316
wavelet-LLH	glszm	LargeAreaHighGrayLevelEmphasis	14475.878
wavelet-LLH	glszm	HighGrayLevelZoneEmphasis	2730.298475
wavelet-LLH	glszm	SmallAreaEmphasis	0.827827535
wavelet-LLH	glszm	LowGrayLevelZoneEmphasis	0.003230499
wavelet-LLH	glszm	ZoneEntropy	6.450267903
wavelet-LLH	glszm	SmallAreaLowGrayLevelEmphasis	0.003157128
wavelet-LLH	ngtdm	Coarseness	0.010125148
wavelet-LLH	ngtdm	Complexity	15861.49462
wavelet-LLH	ngtdm	Strength	17.60789545
wavelet-LLH	ngtdm	Contrast	0.432354785
wavelet-LLH	ngtdm	Busyness	0.024255629
wavelet-LLL	gldm	GrayLevelVariance	813.2438797
wavelet-LLL	gldm	HighGrayLevelEmphasis	5513.263081
wavelet-LLL	gldm	DependenceEntropy	7.320737082
wavelet-LLL	gldm	DependenceNonUniformity	341.6976744
wavelet-LLL	gldm	GrayLevelNonUniformity	9.488372093
wavelet-LLL	gldm	SmallDependenceEmphasis	0.732235068
wavelet-LLL	gldm	SmallDependenceHighGrayLevelEmphasis	3403.824456
wavelet-LLL	gldm	DependenceNonUniformityNormalized	0.496653597
wavelet-LLL	gldm	LargeDependenceEmphasis	3.845930233
wavelet-LLL	gldm	LargeDependenceLowGrayLevelEmphasis	0.003323177
wavelet-LLL	gldm	DependenceVariance	1.280345795
wavelet-LLL	gldm	LargeDependenceHighGrayLevelEmphasis	31091.71948
wavelet-LLL	gldm	SmallDependenceLowGrayLevelEmphasis	0.002784187
wavelet-LLL	gldm	LowGrayLevelEmphasis	0.002854917
wavelet-LLL	glcm	JointAverage	73.59266163
wavelet-LLL	glcm	SumAverage	147.1853233
wavelet-LLL	glcm	JointEntropy	9.592210303
wavelet-LLL	glcm	ClusterShade	-61863.34041
wavelet-LLL	glcm	MaximumProbability	0.007961585
wavelet-LLL	glcm	Idmn	0.963234432
wavelet-LLL	glcm	JointEnergy	0.001541344
wavelet-LLL	glcm	Contrast	694.8736185
wavelet-LLL	glcm	DifferenceEntropy	5.556734363
wavelet-LLL	glcm	InverseVariance	0.090469267
wavelet-LLL	glcm	DifferenceVariance	302.2778322
wavelet-LLL	glcm	Idn	0.88011872
wavelet-LLL	glcm	Idm	0.086526128
wavelet-LLL	glcm	Correlation	0.524938583
wavelet-LLL	glcm	Autocorrelation	5798.867526
wavelet-LLL	glcm	SumEntropy	6.978389247
wavelet-LLL	glcm	SumSquares	729.1128031
wavelet-LLL	glcm	ClusterProminence	11649761.24

wavelet-LLL	glcm	Imc2	0.998715851
wavelet-LLL	glcm	Imc1	-0.474615037
wavelet-LLL	glcm	DifferenceAverage	19.48567837
wavelet-LLL	glcm	Id	0.148218969
wavelet-LLL	glcm	ClusterTendency	2221.577594
wavelet-LLL	firstorder	InterquartileRange	1235.655174
wavelet-LLL	firstorder	Skewness	-0.467658894
wavelet-LLL	firstorder	Uniformity	0.013791239
wavelet-LLL	firstorder	Median	-550.5167871
wavelet-LLL	firstorder	Energy	638591195.4
wavelet-LLL	firstorder	RobustMeanAbsoluteDeviation	491.6736575
wavelet-LLL	firstorder	MeanAbsoluteDeviation	614.9668677
wavelet-LLL	firstorder	TotalEnergy	17241962276
wavelet-LLL	firstorder	Maximum	853.0418305
wavelet-LLL	firstorder	RootMeanSquared	963.423519
wavelet-LLL	firstorder	90Percentile	150.7951918
wavelet-LLL	firstorder	Minimum	-2338.867749
wavelet-LLL	firstorder	Entropy	6.447156024
wavelet-LLL	firstorder	Range	3191.90958
wavelet-LLL	firstorder	Variance	507832.8574
wavelet-LLL	firstorder	10Percentile	-1703.893047
wavelet-LLL	firstorder	Kurtosis	2.053137249
wavelet-LLL	firstorder	Mean	-648.345602
wavelet-LLL	glrlm	ShortRunLowGrayLevelEmphasis	0.002914485
wavelet-LLL	glrlm	GrayLevelVariance	812.2207715
wavelet-LLL	glrlm	LowGrayLevelRunEmphasis	0.002917943
wavelet-LLL	glrlm	GrayLevelNonUniformityNormalized	0.013362887
wavelet-LLL	glrlm	RunVariance	0.026106431
wavelet-LLL	glrlm	GrayLevelNonUniformity	8.981332131
wavelet-LLL	glrlm	LongRunEmphasis	1.074181165
wavelet-LLL	glrlm	ShortRunHighGrayLevelEmphasis	5296.041352
wavelet-LLL	glrlm	RunLengthNonUniformity	642.9073886
wavelet-LLL	glrlm	ShortRunEmphasis	0.983109338
wavelet-LLL	glrlm	LongRunHighGrayLevelEmphasis	6081.804861
wavelet-LLL	glrlm	RunPercentage	0.976855993
wavelet-LLL	glrlm	LongRunLowGrayLevelEmphasis	0.002932444
wavelet-LLL	glrlm	RunEntropy	6.562309569
wavelet-LLL	glrlm	HighGrayLevelRunEmphasis	5440.054342
wavelet-LLL	glrlm	RunLengthNonUniformityNormalized	0.95651992
wavelet-LLL	glszm	GrayLevelVariance	774.5045804
wavelet-LLL	glszm	ZoneVariance	1.654576342
wavelet-LLL	glszm	GrayLevelNonUniformityNormalized	0.011600895
wavelet-LLL	glszm	SizeZoneNonUniformityNormalized	0.77044343
wavelet-LLL	glszm	SizeZoneNonUniformity	409.1054614
wavelet-LLL	glszm	GrayLevelNonUniformity	6.16007533
wavelet-LLL	glszm	LargeAreaEmphasis	3.333333333
wavelet-LLL	glszm	SmallAreaHighGrayLevelEmphasis	4015.131947
wavelet-LLL	glszm	ZonePercentage	0.771802326
wavelet-LLL	glszm	LargeAreaLowGrayLevelEmphasis	0.003984214
wavelet-LLL	glszm	LargeAreaHighGrayLevelEmphasis	26126.25424
wavelet-LLL	glszm	HighGrayLevelZoneEmphasis	4703.032015
wavelet-LLL	glszm	SmallAreaEmphasis	0.896857813

wavelet-LLL	glszm	LowGrayLevelZoneEmphasis	0.00363107
wavelet-LLL	glszm	ZoneEntropy	7.005930477
wavelet-LLL	glszm	SmallAreaLowGrayLevelEmphasis	0.003592662
wavelet-LLL	ngtdm	Coarseness	0.009897309
wavelet-LLL	ngtdm	Complexity	59978.8186
wavelet-LLL	ngtdm	Strength	48.42167828
wavelet-LLL	ngtdm	Contrast	1.765971575
wavelet-LLL	ngtdm	Busyness	0.012488443
wavelet-HHL	gldm	GrayLevelVariance	42.63060269
wavelet-HHL	gldm	HighGrayLevelEmphasis	758.9709302
wavelet-HHL	gldm	DependenceEntropy	6.501432194
wavelet-HHL	gldm	DependenceNonUniformity	169.0261628
wavelet-HHL	gldm	GrayLevelNonUniformity	36.95348837
wavelet-HHL	gldm	SmallDependenceEmphasis	0.455748768
wavelet-HHL	gldm	SmallDependenceHighGrayLevelEmphasis	347.1130399
wavelet-HHL	gldm	DependenceNonUniformityNormalized	0.245677562
wavelet-HHL	gldm	LargeDependenceEmphasis	9.281976744
wavelet-HHL	gldm	LargeDependenceLowGrayLevelEmphasis	0.01560574
wavelet-HHL	gldm	DependenceVariance	3.118881828
wavelet-HHL	gldm	LargeDependenceHighGrayLevelEmphasis	6606.276163
wavelet-HHL	gldm	SmallDependenceLowGrayLevelEmphasis	0.002415306
wavelet-HHL	gldm	LowGrayLevelEmphasis	0.003230089
wavelet-HHL	glcm	JointAverage	26.79812262
wavelet-HHL	glcm	SumAverage	53.59624524
wavelet-HHL	glcm	JointEntropy	8.240095466
wavelet-HHL	glcm	ClusterShade	191.7654564
wavelet-HHL	glcm	MaximumProbability	0.019494959
wavelet-HHL	glcm	Idmn	0.968070464
wavelet-HHL	glcm	JointEnergy	0.005101804
wavelet-HHL	glcm	Contrast	81.79678034
wavelet-HHL	glcm	DifferenceEntropy	4.126389088
wavelet-HHL	glcm	InverseVariance	0.194577813
wavelet-HHL	glcm	DifferenceVariance	39.36762859
wavelet-HHL	glcm	Idn	0.890446689
wavelet-HHL	glcm	Idm	0.192688363
wavelet-HHL	glcm	Correlation	-0.029731644
wavelet-HHL	glcm	Autocorrelation	717.0756104
wavelet-HHL	glcm	SumEntropy	4.970120184
wavelet-HHL	glcm	SumSquares	39.80915183
wavelet-HHL	glcm	ClusterProminence	28843.9975
wavelet-HHL	glcm	Imc2	0.899017728
wavelet-HHL	glcm	Imc1	-0.183092705
wavelet-HHL	glcm	DifferenceAverage	6.431619094
wavelet-HHL	glcm	Id	0.275675423
wavelet-HHL	glcm	ClusterTendency	77.43982697
wavelet-HHL	firstorder	InterquartileRange	162.0632824
wavelet-HHL	firstorder	Skewness	0.101766871
wavelet-HHL	firstorder	Uniformity	0.053711466
wavelet-HHL	firstorder	Median	-2.109107525
wavelet-HHL	firstorder	Energy	18342109.93
wavelet-HHL	firstorder	RobustMeanAbsoluteDeviation	70.75709956
wavelet-HHL	firstorder	MeanAbsoluteDeviation	118.4808324

wavelet-HHL	firstorder	TotalEnergy	495236968.1
wavelet-HHL	firstorder	Maximum	519.1388679
wavelet-HHL	firstorder	RootMeanSquared	163.2790357
wavelet-HHL	firstorder	90Percentile	204.0423845
wavelet-HHL	firstorder	Minimum	-632.0131387
wavelet-HHL	firstorder	Entropy	4.620493688
wavelet-HHL	firstorder	Range	1151.152007
wavelet-HHL	firstorder	Variance	26617.15722
wavelet-HHL	firstorder	10Percentile	-182.0074158
wavelet-HHL	firstorder	Kurtosis	4.091233163
wavelet-HHL	firstorder	Mean	6.548762216
wavelet-HHL	glrlm	ShortRunLowGrayLevelEmphasis	0.003272418
wavelet-HHL	glrlm	GrayLevelVariance	44.36053424
wavelet-HHL	glrlm	LowGrayLevelRunEmphasis	0.003335266
wavelet-HHL	glrlm	GrayLevelNonUniformityNormalized	0.051348696
wavelet-HHL	glrlm	RunVariance	0.070380932
wavelet-HHL	glrlm	GrayLevelNonUniformity	33.31268721
wavelet-HHL	glrlm	LongRunEmphasis	1.19559918
wavelet-HHL	glrlm	ShortRunHighGrayLevelEmphasis	730.7115414
wavelet-HHL	glrlm	RunLengthNonUniformity	581.4428249
wavelet-HHL	glrlm	ShortRunEmphasis	0.958091729
wavelet-HHL	glrlm	LongRunHighGrayLevelEmphasis	902.4075792
wavelet-HHL	glrlm	RunPercentage	0.942978533
wavelet-HHL	glrlm	LongRunLowGrayLevelEmphasis	0.003627623
wavelet-HHL	glrlm	RunEntropy	4.936347405
wavelet-HHL	glrlm	HighGrayLevelRunEmphasis	761.0948472
wavelet-HHL	glrlm	RunLengthNonUniformityNormalized	0.895942767
wavelet-HHL	glszm	GrayLevelVariance	65.94902639
wavelet-HHL	glszm	ZoneVariance	18.64228375
wavelet-HHL	glszm	GrayLevelNonUniformityNormalized	0.037150672
wavelet-HHL	glszm	SizeZoneNonUniformityNormalized	0.531302506
wavelet-HHL	glszm	SizeZoneNonUniformity	186.4871795
wavelet-HHL	glszm	GrayLevelNonUniformity	13.03988604
wavelet-HHL	glszm	LargeAreaEmphasis	22.48433048
wavelet-HHL	glszm	SmallAreaHighGrayLevelEmphasis	573.1968347
wavelet-HHL	glszm	ZonePercentage	0.510174419
wavelet-HHL	glszm	LargeAreaLowGrayLevelEmphasis	0.037243798
wavelet-HHL	glszm	LargeAreaHighGrayLevelEmphasis	15367.7037
wavelet-HHL	glszm	HighGrayLevelZoneEmphasis	774.9344729
wavelet-HHL	glszm	SmallAreaEmphasis	0.756009823
wavelet-HHL	glszm	LowGrayLevelZoneEmphasis	0.004896778
wavelet-HHL	glszm	ZoneEntropy	6.035387442
wavelet-HHL	glszm	SmallAreaLowGrayLevelEmphasis	0.004529853
wavelet-HHL	ngtdm	Coarseness	0.010095415
wavelet-HHL	ngtdm	Complexity	3133.168995
wavelet-HHL	ngtdm	Strength	4.579226835
wavelet-HHL	ngtdm	Contrast	0.259302692
wavelet-HHL	ngtdm	Busyness	0.080589932
wavelet-HLH	gldm	GrayLevelVariance	33.42313413
wavelet-HLH	gldm	HighGrayLevelEmphasis	504.2122093
wavelet-HLH	gldm	DependenceEntropy	6.36513909
wavelet-HLH	gldm	DependenceNonUniformity	167.1133721

wavelet-HLH	gldm	GrayLevelNonUniformity	41.6627907
wavelet-HLH	gldm	SmallDependenceEmphasis	0.466273419
wavelet-HLH	gldm	SmallDependenceHighGrayLevelEmphasis	250.4180394
wavelet-HLH	gldm	DependenceNonUniformityNormalized	0.242897343
wavelet-HLH	gldm	LargeDependenceEmphasis	10.34593023
wavelet-HLH	gldm	LargeDependenceLowGrayLevelEmphasis	0.026303563
wavelet-HLH	gldm	DependenceVariance	3.801852353
wavelet-HLH	gldm	LargeDependenceHighGrayLevelEmphasis	4785.005814
wavelet-HLH	gldm	SmallDependenceLowGrayLevelEmphasis	0.003488374
wavelet-HLH	gldm	LowGrayLevelEmphasis	0.00480066
wavelet-HLH	glcm	JointAverage	21.72844304
wavelet-HLH	glcm	SumAverage	43.45688608
wavelet-HLH	glcm	JointEntropy	7.990613855
wavelet-HLH	glcm	ClusterShade	36.62919175
wavelet-HLH	glcm	MaximumProbability	0.021434003
wavelet-HLH	glcm	Idmn	0.97375523
wavelet-HLH	glcm	JointEnergy	0.006077257
wavelet-HLH	glcm	Contrast	60.87751838
wavelet-HLH	glcm	DifferenceEntropy	3.930821015
wavelet-HLH	glcm	InverseVariance	0.206881222
wavelet-HLH	glcm	DifferenceVariance	29.27101155
wavelet-HLH	glcm	Idn	0.898990855
wavelet-HLH	glcm	Idm	0.203936451
wavelet-HLH	glcm	Correlation	-0.013655844
wavelet-HLH	glcm	Autocorrelation	471.6904925
wavelet-HLH	glcm	SumEntropy	4.811160576
wavelet-HLH	glcm	SumSquares	29.98929046
wavelet-HLH	glcm	ClusterProminence	18088.37043
wavelet-HLH	glcm	Imc2	0.86606405
wavelet-HLH	glcm	Imc1	-0.160737554
wavelet-HLH	glcm	DifferenceAverage	5.560862487
wavelet-HLH	glcm	Id	0.289903376
wavelet-HLH	glcm	ClusterTendency	59.07964347
wavelet-HLH	firstorder	InterquartileRange	145.7991438
wavelet-HLH	firstorder	Skewness	0.073244215
wavelet-HLH	firstorder	Uniformity	0.060556382
wavelet-HLH	firstorder	Median	0.47423188
wavelet-HLH	firstorder	Energy	14520103.33
wavelet-HLH	firstorder	RobustMeanAbsoluteDeviation	62.51266467
wavelet-HLH	firstorder	MeanAbsoluteDeviation	104.2456245
wavelet-HLH	firstorder	TotalEnergy	392042789.9
wavelet-HLH	firstorder	Maximum	577.1614622
wavelet-HLH	firstorder	RootMeanSquared	145.2749164
wavelet-HLH	firstorder	90Percentile	176.6247856
wavelet-HLH	firstorder	Minimum	-506.5502279
wavelet-HLH	firstorder	Entropy	4.444517625
wavelet-HLH	firstorder	Range	1083.71169
wavelet-HLH	firstorder	Variance	21084.88296
wavelet-HLH	firstorder	10Percentile	-164.7128295
wavelet-HLH	firstorder	Kurtosis	4.651506618
wavelet-HLH	firstorder	Mean	4.463001888
wavelet-HLH	glrlm	ShortRunLowGrayLevelEmphasis	0.004854493

wavelet-HLH	glrlm	GrayLevelVariance	35.07923601
wavelet-HLH	glrlm	LowGrayLevelRunEmphasis	0.004958064
wavelet-HLH	glrlm	GrayLevelNonUniformityNormalized	0.05760049
wavelet-HLH	glrlm	RunVariance	0.073497184
wavelet-HLH	glrlm	GrayLevelNonUniformity	37.25624045
wavelet-HLH	glrlm	LongRunEmphasis	1.205955613
wavelet-HLH	glrlm	ShortRunHighGrayLevelEmphasis	486.0178354
wavelet-HLH	glrlm	RunLengthNonUniformity	576.6539372
wavelet-HLH	glrlm	ShortRunEmphasis	0.955904579
wavelet-HLH	glrlm	LongRunHighGrayLevelEmphasis	602.3503876
wavelet-HLH	glrlm	RunPercentage	0.940071556
wavelet-HLH	glrlm	LongRunLowGrayLevelEmphasis	0.005439567
wavelet-HLH	glrlm	RunEntropy	4.78173554
wavelet-HLH	glrlm	HighGrayLevelRunEmphasis	506.6509088
wavelet-HLH	glrlm	RunLengthNonUniformityNormalized	0.891157526
wavelet-HLH	glszm	GrayLevelVariance	54.9252926
wavelet-HLH	glszm	ZoneVariance	16.42432851
wavelet-HLH	glszm	GrayLevelNonUniformityNormalized	0.040761754
wavelet-HLH	glszm	SizeZoneNonUniformityNormalized	0.556437215
wavelet-HLH	glszm	SizeZoneNonUniformity	197.5352113
wavelet-HLH	glszm	GrayLevelNonUniformity	14.47042254
wavelet-HLH	glszm	LargeAreaEmphasis	20.18028169
wavelet-HLH	glszm	SmallAreaHighGrayLevelEmphasis	422.3289841
wavelet-HLH	glszm	ZonePercentage	0.515988372
wavelet-HLH	glszm	LargeAreaLowGrayLevelEmphasis	0.05129116
wavelet-HLH	glszm	LargeAreaHighGrayLevelEmphasis	9098.090141
wavelet-HLH	glszm	HighGrayLevelZoneEmphasis	532.1323944
wavelet-HLH	glszm	SmallAreaEmphasis	0.773295228
wavelet-HLH	glszm	LowGrayLevelZoneEmphasis	0.007059332
wavelet-HLH	glszm	ZoneEntropy	5.89341006
wavelet-HLH	glszm	SmallAreaLowGrayLevelEmphasis	0.006430898
wavelet-HLH	ngtdm	Coarseness	0.010423797
wavelet-HLH	ngtdm	Complexity	2809.607661
wavelet-HLH	ngtdm	Strength	5.00231429
wavelet-HLH	ngtdm	Contrast	0.174393409
wavelet-HLH	ngtdm	Busyness	0.087386695

Image type	Feature Class	Feature Name	3: RTSTRUCT: C99-DNU_segment_RUL GTV
general	info	BoundingBox	(0, 1, 1, 4, 4, 4)
general	info	EnabledImageTypes	{u'Original': {}, u'Wavelet': {}}
general	info	GeneralSettings	{'distances': [1], 'additionalInfo': True, 'force2D': False, 'interpolator': 'sitkBSpline', 'resampledPixelSpacing': [3.0], 'label': 1, 'normalizeScale': 1, 'normalize': False, 'force2Ddimension': 0, 'removeOutliers': None, 'minimumROISize': None, u'binWidth': 25.0, 'minimumROIDimensions': 1, u'correctMask': True, 'preCrop': False, u'symmetricalGLCM': True, 'resegmentRange': None, 'padDistance': 5}
general	info	ImageHash	b95bbe59cf23c56c380ff660fdc7505d715a6071
general	info	ImageSpacing	(0.9765625000000002, 0.9765625000000002, 2.0)
general	info	MaskHash	9fbeeb14750e84851604cfb767679f29630d9eff
general	info	NumpyVersion	1.13.1
general	info	PyWaveletVersion	0.5.2
general	info	SimpleITKVersion	1.1.0
general	info	Version	1.3.0.post102+g1b0eb74
general	info	VolumeNum	1
general	info	VoxelNum	42
original	shape	Maximum3DDiameter	13.07669683
original	shape	Maximum2DDiameterSlice	10.81665383
original	shape	Sphericity	0.944357774
original	shape	MinorAxis	12.15805051
original	shape	Elongation	0.914761319
original	shape	SurfaceVolumeRatio	0.491069785
original	shape	Volume	1134
original	shape	MajorAxis	13.2909539
original	shape	SurfaceArea	556.873136
original	shape	Flatness	0.73821467
original	shape	LeastAxis	9.811577149
original	shape	Maximum2DDiameterColumn	12.72792206
original	shape	Maximum2DDiameterRow	12.72792206
original	gldm	GrayLevelVariance	71.91609977
original	gldm	HighGrayLevelEmphasis	245.9047619
original	gldm	DependenceEntropy	4.820888851
original	gldm	DependenceNonUniformity	26.76190476
original	gldm	GrayLevelNonUniformity	2.142857143
original	gldm	SmallDependenceEmphasis	0.821428571
original	gldm	SmallDependenceHighGrayLevelEmphasis	233.547619
original	gldm	DependenceNonUniformityNormalized	0.637188209
original	gldm	LargeDependenceEmphasis	1.714285714
original	gldm	LargeDependenceLowGrayLevelEmphasis	0.064097231
original	gldm	DependenceVariance	0.181405896
original	gldm	LargeDependenceHighGrayLevelEmphasis	295.3333333
original	gldm	SmallDependenceLowGrayLevelEmphasis	0.037990992
original	gldm	LowGrayLevelEmphasis	0.043212239

original	glcm	JointAverage	15.0472715
original	glcm	SumAverage	30.094543
original	glcm	JointEntropy	5.319491261
original	glcm	ClusterShade	473.3636944
original	glcm	MaximumProbability	0.036649262
original	glcm	Idmn	0.90554542
original	glcm	JointEnergy	0.026129262
original	glcm	Contrast	121.2981257
original	glcm	DifferenceEntropy	3.563574493
original	glcm	InverseVariance	0.116802863
original	glcm	DifferenceVariance	34.9832248
original	glcm	Idn	0.79535656
original	glcm	Idm	0.09439937
original	glcm	Correlation	0.171965992
original	glcm	Autocorrelation	239.6106206
original	glcm	SumEntropy	4.032508437
original	glcm	SumSquares	73.42271533
original	glcm	ClusterProminence	66716.03399
original	glcm	Imc2	0.999564695
original	glcm	Imc1	-0.805080681
original	glcm	DifferenceAverage	9.115300811
original	glcm	Id	0.176135482
original	glcm	ClusterTendency	172.3927356
original	firstorder	InterquartileRange	316.5
original	firstorder	Skewness	0.66382111
original	firstorder	Uniformity	0.051020408
original	firstorder	Median	-612
original	firstorder	Energy	15070081
original	firstorder	RobustMeanAbsoluteDeviation	127.3632813
original	firstorder	MeanAbsoluteDeviation	176.9195011
original	firstorder	TotalEnergy	406892187
original	firstorder	Maximum	-99
original	firstorder	RootMeanSquared	599.0087248
original	firstorder	90Percentile	-240.2
original	firstorder	Minimum	-851
original	firstorder	Entropy	4.499227542
original	firstorder	Range	752
original	firstorder	Variance	44597.81916
original	firstorder	10Percentile	-786.8
original	firstorder	Kurtosis	2.366050028
original	firstorder	Mean	-560.547619
original	glrlm	ShortRunLowGrayLevelEmphasis	0.043135993
original	glrlm	GrayLevelVariance	72.17869953
original	glrlm	LowGrayLevelRunEmphasis	0.043345874
original	glrlm	GrayLevelNonUniformityNormalized	0.050736881
original	glrlm	RunVariance	0.009138105
original	glrlm	GrayLevelNonUniformity	2.111766282
original	glrlm	LongRunEmphasis	1.028705441
original	glrlm	ShortRunHighGrayLevelEmphasis	247.0962287
original	glrlm	RunLengthNonUniformity	40.88067542
original	glrlm	ShortRunEmphasis	0.99282364
original	glrlm	LongRunHighGrayLevelEmphasis	249.5850889
original	glrlm	RunPercentage	0.990842491
original	glrlm	LongRunLowGrayLevelEmphasis	0.044185397

original	glrlm	RunEntropy	4.522108318
original	glrlm	HighGrayLevelRunEmphasis	247.5940007
original	glrlm	RunLengthNonUniformityNormalized	0.981723791
original	glszm	GrayLevelVariance	75.29437546
original	glszm	ZoneVariance	0.11687363
original	glszm	GrayLevelNonUniformityNormalized	0.046018992
original	glszm	SizeZoneNonUniformityNormalized	0.766252739
original	glszm	SizeZoneNonUniformity	28.35135135
original	glszm	GrayLevelNonUniformity	1.702702703
original	glszm	LargeAreaEmphasis	1.405405405
original	glszm	SmallAreaHighGrayLevelEmphasis	262.7702703
original	glszm	ZonePercentage	0.880952381
original	glszm	LargeAreaLowGrayLevelEmphasis	0.05695416
original	glszm	LargeAreaHighGrayLevelEmphasis	297.8378378
original	glszm	HighGrayLevelZoneEmphasis	269.7837838
original	glszm	SmallAreaEmphasis	0.898648649
original	glszm	LowGrayLevelZoneEmphasis	0.045100517
original	glszm	ZoneEntropy	4.831074987
original	glszm	SmallAreaLowGrayLevelEmphasis	0.042137106
original	ngtdm	Coarseness	0.073659008
original	ngtdm	Complexity	2089.501123
original	ngtdm	Strength	31.3693065
original	ngtdm	Contrast	1.41002399
original	ngtdm	Busyness	0.076188539
wavelet-HHH	gldm	GrayLevelVariance	1.725623583
wavelet-HHH	gldm	HighGrayLevelEmphasis	21.76190476
wavelet-HHH	gldm	DependenceEntropy	4.302449863
wavelet-HHH	gldm	DependenceNonUniformity	7.80952381
wavelet-HHH	gldm	GrayLevelNonUniformity	9.904761905
wavelet-HHH	gldm	SmallDependenceEmphasis	0.233949661
wavelet-HHH	gldm	SmallDependenceHighGrayLevelEmphasis	5.165760717
wavelet-HHH	gldm	DependenceNonUniformityNormalized	0.185941043
wavelet-HHH	gldm	LargeDependenceEmphasis	13.9047619
wavelet-HHH	gldm	LargeDependenceLowGrayLevelEmphasis	0.845642884
wavelet-HHH	gldm	DependenceVariance	3.108843537
wavelet-HHH	gldm	LargeDependenceHighGrayLevelEmphasis	267
wavelet-HHH	gldm	SmallDependenceLowGrayLevelEmphasis	0.038614712
wavelet-HHH	gldm	LowGrayLevelEmphasis	0.083021677
wavelet-HHH	glcm	JointAverage	4.469861582
wavelet-HHH	glcm	SumAverage	8.939723164
wavelet-HHH	glcm	JointEntropy	4.211024519
wavelet-HHH	glcm	ClusterShade	-1.192130355
wavelet-HHH	glcm	MaximumProbability	0.127794658
wavelet-HHH	glcm	Idmn	0.923337699
wavelet-HHH	glcm	JointEnergy	0.066428419
wavelet-HHH	glcm	Contrast	4.611044211
wavelet-HHH	glcm	DifferenceEntropy	2.043525536
wavelet-HHH	glcm	InverseVariance	0.414457866
wavelet-HHH	glcm	DifferenceVariance	1.598014039
wavelet-HHH	glcm	Idn	0.819965728
wavelet-HHH	glcm	Idm	0.405817468
wavelet-HHH	glcm	Correlation	-0.049885543
wavelet-HHH	glcm	Autocorrelation	19.88502866
wavelet-HHH	glcm	SumEntropy	2.684962035

wavelet-HHH	glcm	SumSquares	2.202561065
wavelet-HHH	glcm	ClusterProminence	51.87014399
wavelet-HHH	glcm	Imc2	0.864448626
wavelet-HHH	glcm	Imc1	-0.306957885
wavelet-HHH	glcm	DifferenceAverage	1.716022915
wavelet-HHH	glcm	Id	0.47517634
wavelet-HHH	glcm	ClusterTendency	4.199200049
wavelet-HHH	firstorder	InterquartileRange	37.00632936
wavelet-HHH	firstorder	Skewness	0.015796301
wavelet-HHH	firstorder	Uniformity	0.235827664
wavelet-HHH	firstorder	Median	-5.510520666
wavelet-HHH	firstorder	Energy	44278.26972
wavelet-HHH	firstorder	RobustMeanAbsoluteDeviation	15.1642361
wavelet-HHH	firstorder	MeanAbsoluteDeviation	24.99646755
wavelet-HHH	firstorder	TotalEnergy	1195513.283
wavelet-HHH	firstorder	Maximum	70.08417579
wavelet-HHH	firstorder	RootMeanSquared	32.46913176
wavelet-HHH	firstorder	90Percentile	46.58325999
wavelet-HHH	firstorder	Minimum	-88.4433181
wavelet-HHH	firstorder	Entropy	2.340725
wavelet-HHH	firstorder	Range	158.5274939
wavelet-HHH	firstorder	Variance	1054.207068
wavelet-HHH	firstorder	10Percentile	-35.55297546
wavelet-HHH	firstorder	Kurtosis	3.371011664
wavelet-HHH	firstorder	Mean	-0.193517045
wavelet-HHH	glrlm	ShortRunLowGrayLevelEmphasis	0.081286984
wavelet-HHH	glrlm	GrayLevelVariance	1.807556963
wavelet-HHH	glrlm	LowGrayLevelRunEmphasis	0.08547913
wavelet-HHH	glrlm	GrayLevelNonUniformityNormalized	0.227230196
wavelet-HHH	glrlm	RunVariance	0.098241321
wavelet-HHH	glrlm	GrayLevelNonUniformity	8.712494977
wavelet-HHH	glrlm	LongRunEmphasis	1.311868785
wavelet-HHH	glrlm	ShortRunHighGrayLevelEmphasis	20.52874398
wavelet-HHH	glrlm	RunLengthNonUniformity	32.26109983
wavelet-HHH	glrlm	ShortRunEmphasis	0.928860771
wavelet-HHH	glrlm	LongRunHighGrayLevelEmphasis	28.27351755
wavelet-HHH	glrlm	RunPercentage	0.912087912
wavelet-HHH	glrlm	LongRunLowGrayLevelEmphasis	0.10357601
wavelet-HHH	glrlm	RunEntropy	2.707957236
wavelet-HHH	glrlm	HighGrayLevelRunEmphasis	21.9309215
wavelet-HHH	glrlm	RunLengthNonUniformityNormalized	0.836880449
wavelet-HHH	glszm	GrayLevelVariance	2.852071006
wavelet-HHH	glszm	ZoneVariance	6.023668639
wavelet-HHH	glszm	GrayLevelNonUniformityNormalized	0.171597633
wavelet-HHH	glszm	SizeZoneNonUniformityNormalized	0.25443787
wavelet-HHH	glszm	SizeZoneNonUniformity	3.307692308
wavelet-HHH	glszm	GrayLevelNonUniformity	2.230769231
wavelet-HHH	glszm	LargeAreaEmphasis	16.46153846
wavelet-HHH	glszm	SmallAreaHighGrayLevelEmphasis	9.213378443
wavelet-HHH	glszm	ZonePercentage	0.30952381
wavelet-HHH	glszm	LargeAreaLowGrayLevelEmphasis	1.030609192
wavelet-HHH	glszm	LargeAreaHighGrayLevelEmphasis	337.0769231
wavelet-HHH	glszm	HighGrayLevelZoneEmphasis	22.07692308
wavelet-HHH	glszm	SmallAreaEmphasis	0.442723172

wavelet-HHH	glszm	LowGrayLevelZoneEmphasis	0.140074132
wavelet-HHH	glszm	ZoneEntropy	3.546593564
wavelet-HHH	glszm	SmallAreaLowGrayLevelEmphasis	0.107278197
wavelet-HHH	ngtdm	Coarseness	0.132693026
wavelet-HHH	ngtdm	Complexity	20.40199518
wavelet-HHH	ngtdm	Strength	1.769467303
wavelet-HHH	ngtdm	Contrast	0.092141164
wavelet-HHH	ngtdm	Busyness	0.270068281
wavelet-HLL	gldm	GrayLevelVariance	70.89569161
wavelet-HLL	gldm	HighGrayLevelEmphasis	474.7142857
wavelet-HLL	gldm	DependenceEntropy	4.546846589
wavelet-HLL	gldm	DependenceNonUniformity	13.19047619
wavelet-HLL	gldm	GrayLevelNonUniformity	2.80952381
wavelet-HLL	gldm	SmallDependenceEmphasis	0.529265873
wavelet-HLL	gldm	SmallDependenceHighGrayLevelEmphasis	193.5709325
wavelet-HLL	gldm	DependenceNonUniformityNormalized	0.314058957
wavelet-HLL	gldm	LargeDependenceEmphasis	4.857142857
wavelet-HLL	gldm	LargeDependenceLowGrayLevelEmphasis	0.081458245
wavelet-HLL	gldm	DependenceVariance	1.045351474
wavelet-HLL	gldm	LargeDependenceHighGrayLevelEmphasis	3041.857143
wavelet-HLL	gldm	SmallDependenceLowGrayLevelEmphasis	0.029765363
wavelet-HLL	gldm	LowGrayLevelEmphasis	0.039466073
wavelet-HLL	glcm	JointAverage	20.52214638
wavelet-HLL	glcm	SumAverage	41.04429276
wavelet-HLL	glcm	JointEntropy	5.129290381
wavelet-HLL	glcm	ClusterShade	-1142.461733
wavelet-HLL	glcm	MaximumProbability	0.051953095
wavelet-HLL	glcm	Idmn	0.944645665
wavelet-HLL	glcm	JointEnergy	0.030846067
wavelet-HLL	glcm	Contrast	66.42976223
wavelet-HLL	glcm	DifferenceEntropy	3.225897505
wavelet-HLL	glcm	InverseVariance	0.19304285
wavelet-HLL	glcm	DifferenceVariance	20.37392793
wavelet-HLL	glcm	Idn	0.848476092
wavelet-HLL	glcm	Idm	0.180050152
wavelet-HLL	glcm	Correlation	0.473998871
wavelet-HLL	glcm	Autocorrelation	455.8079144
wavelet-HLL	glcm	SumEntropy	3.898632584
wavelet-HLL	glcm	SumSquares	67.14037164
wavelet-HLL	glcm	ClusterProminence	89610.50116
wavelet-HLL	glcm	Imc2	0.997499128
wavelet-HLL	glcm	Imc1	-0.693482141
wavelet-HLL	glcm	DifferenceAverage	6.345175227
wavelet-HLL	glcm	Id	0.260886708
wavelet-HLL	glcm	ClusterTendency	202.1317243
wavelet-HLL	firstorder	InterquartileRange	259.6379082
wavelet-HLL	firstorder	Skewness	-0.727040828
wavelet-HLL	firstorder	Uniformity	0.066893424
wavelet-HLL	firstorder	Median	4.774272904
wavelet-HLL	firstorder	Energy	1864496.785
wavelet-HLL	firstorder	RobustMeanAbsoluteDeviation	113.612342
wavelet-HLL	firstorder	MeanAbsoluteDeviation	166.2080459
wavelet-HLL	firstorder	TotalEnergy	50341413.19
wavelet-HLL	firstorder	Maximum	276.8004479

wavelet-HLL	firstorder	RootMeanSquared	210.6959435
wavelet-HLL	firstorder	90Percentile	227.3116629
wavelet-HLL	firstorder	Minimum	-490.6901872
wavelet-HLL	firstorder	Entropy	4.13686371
wavelet-HLL	firstorder	Range	767.490635
wavelet-HLL	firstorder	Variance	44289.41989
wavelet-HLL	firstorder	10Percentile	-292.4952571
wavelet-HLL	firstorder	Kurtosis	2.660079395
wavelet-HLL	firstorder	Mean	-10.16664638
wavelet-HLL	glrlm	ShortRunLowGrayLevelEmphasis	0.040017748
wavelet-HLL	glrlm	GrayLevelVariance	71.03071398
wavelet-HLL	glrlm	LowGrayLevelRunEmphasis	0.040445673
wavelet-HLL	glrlm	GrayLevelNonUniformityNormalized	0.065345245
wavelet-HLL	glrlm	RunVariance	0.036404434
wavelet-HLL	glrlm	GrayLevelNonUniformity	2.645520787
wavelet-HLL	glrlm	LongRunEmphasis	1.117835413
wavelet-HLL	glrlm	ShortRunHighGrayLevelEmphasis	449.5594557
wavelet-HLL	glrlm	RunLengthNonUniformity	37.60708388
wavelet-HLL	glrlm	ShortRunEmphasis	0.970541147
wavelet-HLL	glrlm	LongRunHighGrayLevelEmphasis	543.4184872
wavelet-HLL	glrlm	RunPercentage	0.963369963
wavelet-HLL	glrlm	LongRunLowGrayLevelEmphasis	0.042157372
wavelet-HLL	glrlm	RunEntropy	4.246625656
wavelet-HLL	glrlm	HighGrayLevelRunEmphasis	468.331262
wavelet-HLL	glrlm	RunLengthNonUniformityNormalized	0.927191131
wavelet-HLL	glszm	GrayLevelVariance	72.51851852
wavelet-HLL	glszm	ZoneVariance	0.987654321
wavelet-HLL	glszm	GrayLevelNonUniformityNormalized	0.053497942
wavelet-HLL	glszm	SizeZoneNonUniformityNormalized	0.497942387
wavelet-HLL	glszm	SizeZoneNonUniformity	13.44444444
wavelet-HLL	glszm	GrayLevelNonUniformity	1.444444444
wavelet-HLL	glszm	LargeAreaEmphasis	3.407407407
wavelet-HLL	glszm	SmallAreaHighGrayLevelEmphasis	251.3290535
wavelet-HLL	glszm	ZonePercentage	0.642857143
wavelet-HLL	glszm	LargeAreaLowGrayLevelEmphasis	0.083363665
wavelet-HLL	glszm	LargeAreaHighGrayLevelEmphasis	1820.814815
wavelet-HLL	glszm	HighGrayLevelZoneEmphasis	408.6296296
wavelet-HLL	glszm	SmallAreaEmphasis	0.730133745
wavelet-HLL	glszm	LowGrayLevelZoneEmphasis	0.05118874
wavelet-HLL	glszm	ZoneEntropy	4.53266528
wavelet-HLL	glszm	SmallAreaLowGrayLevelEmphasis	0.043826765
wavelet-HLL	ngtdm	Coarseness	0.139205192
wavelet-HLL	ngtdm	Complexity	804.9503816
wavelet-HLL	ngtdm	Strength	51.50790262
wavelet-HLL	ngtdm	Contrast	1.031507986
wavelet-HLL	ngtdm	Busyness	0.017134989
wavelet-LHL	gldm	GrayLevelVariance	62.53514739
wavelet-LHL	gldm	HighGrayLevelEmphasis	272.0952381
wavelet-LHL	gldm	DependenceEntropy	4.755296292
wavelet-LHL	gldm	DependenceNonUniformity	16.9047619
wavelet-LHL	gldm	GrayLevelNonUniformity	2.285714286
wavelet-LHL	gldm	SmallDependenceEmphasis	0.572751323
wavelet-LHL	gldm	SmallDependenceHighGrayLevelEmphasis	153.707672
wavelet-LHL	gldm	DependenceNonUniformityNormalized	0.402494331

wavelet-LHL	gldm	LargeDependenceEmphasis	3.238095238
wavelet-LHL	gldm	LargeDependenceLowGrayLevelEmphasis	0.057777519
wavelet-LHL	gldm	DependenceVariance	0.46031746
wavelet-LHL	gldm	LargeDependenceHighGrayLevelEmphasis	836.3333333
wavelet-LHL	gldm	SmallDependenceLowGrayLevelEmphasis	0.037325719
wavelet-LHL	gldm	LowGrayLevelEmphasis	0.040755269
wavelet-LHL	glcm	JointAverage	13.73222146
wavelet-LHL	glcm	SumAverage	27.46444291
wavelet-LHL	glcm	JointEntropy	5.229935265
wavelet-LHL	glcm	ClusterShade	1356.312268
wavelet-LHL	glcm	MaximumProbability	0.042786732
wavelet-LHL	glcm	Idmn	0.909056659
wavelet-LHL	glcm	JointEnergy	0.028115927
wavelet-LHL	glcm	Contrast	105.7551711
wavelet-LHL	glcm	DifferenceEntropy	3.296031883
wavelet-LHL	glcm	InverseVariance	0.118406433
wavelet-LHL	glcm	DifferenceVariance	28.28981704
wavelet-LHL	glcm	Idn	0.805578765
wavelet-LHL	glcm	Idm	0.118713898
wavelet-LHL	glcm	Correlation	0.236770596
wavelet-LHL	glcm	Autocorrelation	204.735303
wavelet-LHL	glcm	SumEntropy	3.833033829
wavelet-LHL	glcm	SumSquares	68.59432534
wavelet-LHL	glcm	ClusterProminence	72844.46255
wavelet-LHL	glcm	Imc2	0.999243978
wavelet-LHL	glcm	Imc1	-0.770935008
wavelet-LHL	glcm	DifferenceAverage	8.169348926
wavelet-LHL	glcm	Id	0.205235824
wavelet-LHL	glcm	ClusterTendency	168.6221303
wavelet-LHL	firstorder	InterquartileRange	246.748834
wavelet-LHL	firstorder	Skewness	0.330215857
wavelet-LHL	firstorder	Uniformity	0.054421769
wavelet-LHL	firstorder	Median	-17.94011049
wavelet-LHL	firstorder	Energy	1658242.374
wavelet-LHL	firstorder	RobustMeanAbsoluteDeviation	115.7449084
wavelet-LHL	firstorder	MeanAbsoluteDeviation	164.1624838
wavelet-LHL	firstorder	TotalEnergy	44772544.1
wavelet-LHL	firstorder	Maximum	375.733863
wavelet-LHL	firstorder	RootMeanSquared	198.7006827
wavelet-LHL	firstorder	90Percentile	294.1513766
wavelet-LHL	firstorder	Minimum	-337.6373352
wavelet-LHL	firstorder	Entropy	4.350068911
wavelet-LHL	firstorder	Range	713.3711982
wavelet-LHL	firstorder	Variance	39481.9588
wavelet-LHL	firstorder	10Percentile	-241.4328707
wavelet-LHL	firstorder	Kurtosis	2.135704768
wavelet-LHL	firstorder	Mean	-0.049908329
wavelet-LHL	glrlm	ShortRunLowGrayLevelEmphasis	0.041537663
wavelet-LHL	glrlm	GrayLevelVariance	63.25185603
wavelet-LHL	glrlm	LowGrayLevelRunEmphasis	0.041697308
wavelet-LHL	glrlm	GrayLevelNonUniformityNormalized	0.054251698
wavelet-LHL	glrlm	RunVariance	0.029430969
wavelet-LHL	glrlm	GrayLevelNonUniformity	2.220584108
wavelet-LHL	glrlm	LongRunEmphasis	1.089908064

wavelet-LHL	glrlm	ShortRunHighGrayLevelEmphasis	266.996956
wavelet-LHL	glrlm	RunLengthNonUniformity	39.12033638
wavelet-LHL	glrlm	ShortRunEmphasis	0.979832986
wavelet-LHL	glrlm	LongRunHighGrayLevelEmphasis	295.535782
wavelet-LHL	glrlm	RunPercentage	0.974358974
wavelet-LHL	glrlm	LongRunLowGrayLevelEmphasis	0.042390562
wavelet-LHL	glrlm	RunEntropy	4.389874117
wavelet-LHL	glrlm	HighGrayLevelRunEmphasis	272.3924089
wavelet-LHL	glrlm	RunLengthNonUniformityNormalized	0.952263533
wavelet-LHL	glszm	GrayLevelVariance	72.64684899
wavelet-LHL	glszm	ZoneVariance	0.454221165
wavelet-LHL	glszm	GrayLevelNonUniformityNormalized	0.048751486
wavelet-LHL	glszm	SizeZoneNonUniformityNormalized	0.498216409
wavelet-LHL	glszm	SizeZoneNonUniformity	14.44827586
wavelet-LHL	glszm	GrayLevelNonUniformity	1.413793103
wavelet-LHL	glszm	LargeAreaEmphasis	2.551724138
wavelet-LHL	glszm	SmallAreaHighGrayLevelEmphasis	194.591954
wavelet-LHL	glszm	ZonePercentage	0.69047619
wavelet-LHL	glszm	LargeAreaLowGrayLevelEmphasis	0.067622469
wavelet-LHL	glszm	LargeAreaHighGrayLevelEmphasis	665.3103448
wavelet-LHL	glszm	HighGrayLevelZoneEmphasis	274.4827586
wavelet-LHL	glszm	SmallAreaEmphasis	0.727011494
wavelet-LHL	glszm	LowGrayLevelZoneEmphasis	0.055450874
wavelet-LHL	glszm	ZoneEntropy	4.720049961
wavelet-LHL	glszm	SmallAreaLowGrayLevelEmphasis	0.053213309
wavelet-LHL	ngtdm	Coarseness	0.091936835
wavelet-LHL	ngtdm	Complexity	1336.940789
wavelet-LHL	ngtdm	Strength	29.14951092
wavelet-LHL	ngtdm	Contrast	1.301157805
wavelet-LHL	ngtdm	Busyness	0.043458466
wavelet-LHH	gldm	GrayLevelVariance	8.868480726
wavelet-LHH	gldm	HighGrayLevelEmphasis	47.19047619
wavelet-LHH	gldm	DependenceEntropy	4.576492125
wavelet-LHH	gldm	DependenceNonUniformity	9.523809524
wavelet-LHH	gldm	GrayLevelNonUniformity	4.619047619
wavelet-LHH	gldm	SmallDependenceEmphasis	0.367109788
wavelet-LHH	gldm	SmallDependenceHighGrayLevelEmphasis	24.89412037
wavelet-LHH	gldm	DependenceNonUniformityNormalized	0.22675737
wavelet-LHH	gldm	LargeDependenceEmphasis	7.904761905
wavelet-LHH	gldm	LargeDependenceLowGrayLevelEmphasis	0.385208854
wavelet-LHH	gldm	DependenceVariance	1.535147392
wavelet-LHH	gldm	LargeDependenceHighGrayLevelEmphasis	281.7142857
wavelet-LHH	gldm	SmallDependenceLowGrayLevelEmphasis	0.044415607
wavelet-LHH	gldm	LowGrayLevelEmphasis	0.072653627
wavelet-LHH	glcm	JointAverage	6.339469914
wavelet-LHH	glcm	SumAverage	12.67893983
wavelet-LHH	glcm	JointEntropy	4.901042185
wavelet-LHH	glcm	ClusterShade	27.75292429
wavelet-LHH	glcm	MaximumProbability	0.076492145
wavelet-LHH	glcm	Idmn	0.915315866
wavelet-LHH	glcm	JointEnergy	0.038293842
wavelet-LHH	glcm	Contrast	18.26569513
wavelet-LHH	glcm	DifferenceEntropy	2.76091094
wavelet-LHH	glcm	InverseVariance	0.245694527

wavelet-LHH	glcm	DifferenceVariance	6.292860281
wavelet-LHH	glcm	Idn	0.813150052
wavelet-LHH	glcm	Idm	0.262230553
wavelet-LHH	glcm	Correlation	-0.007693109
wavelet-LHH	glcm	Autocorrelation	40.06579819
wavelet-LHH	glcm	SumEntropy	3.298061154
wavelet-LHH	glcm	SumSquares	8.942901786
wavelet-LHH	glcm	ClusterProminence	821.1822074
wavelet-LHH	glcm	Imc2	0.983664686
wavelet-LHH	glcm	Imc1	-0.529313721
wavelet-LHH	glcm	DifferenceAverage	3.371090846
wavelet-LHH	glcm	Id	0.353114042
wavelet-LHH	glcm	ClusterTendency	17.50591202
wavelet-LHH	firstorder	InterquartileRange	104.8883889
wavelet-LHH	firstorder	Skewness	0.463908224
wavelet-LHH	firstorder	Uniformity	0.109977324
wavelet-LHH	firstorder	Median	-14.76951845
wavelet-LHH	firstorder	Energy	223982.4089
wavelet-LHH	firstorder	RobustMeanAbsoluteDeviation	41.01843778
wavelet-LHH	firstorder	MeanAbsoluteDeviation	59.59318571
wavelet-LHH	firstorder	TotalEnergy	6047525.041
wavelet-LHH	firstorder	Maximum	151.94909
wavelet-LHH	firstorder	RootMeanSquared	73.02680671
wavelet-LHH	firstorder	90Percentile	105.86323
wavelet-LHH	firstorder	Minimum	-128.6559477
wavelet-LHH	firstorder	Entropy	3.391554926
wavelet-LHH	firstorder	Range	280.6050377
wavelet-LHH	firstorder	Variance	5258.563169
wavelet-LHH	firstorder	10Percentile	-89.89845374
wavelet-LHH	firstorder	Kurtosis	2.446872975
wavelet-LHH	firstorder	Mean	-8.622721721
wavelet-LHH	glrlm	ShortRunLowGrayLevelEmphasis	0.072211833
wavelet-LHH	glrlm	GrayLevelVariance	9.201943398
wavelet-LHH	glrlm	LowGrayLevelRunEmphasis	0.074410905
wavelet-LHH	glrlm	GrayLevelNonUniformityNormalized	0.107310249
wavelet-LHH	glrlm	RunVariance	0.057790574
wavelet-LHH	glrlm	GrayLevelNonUniformity	4.249650203
wavelet-LHH	glrlm	LongRunEmphasis	1.193632715
wavelet-LHH	glrlm	ShortRunHighGrayLevelEmphasis	46.38911334
wavelet-LHH	glrlm	RunLengthNonUniformity	35.11402371
wavelet-LHH	glrlm	ShortRunEmphasis	0.951591821
wavelet-LHH	glrlm	LongRunHighGrayLevelEmphasis	54.63319089
wavelet-LHH	glrlm	RunPercentage	0.941391941
wavelet-LHH	glrlm	LongRunLowGrayLevelEmphasis	0.083207192
wavelet-LHH	glrlm	RunEntropy	3.599364596
wavelet-LHH	glrlm	HighGrayLevelRunEmphasis	48.03792885
wavelet-LHH	glrlm	RunLengthNonUniformityNormalized	0.884418852
wavelet-LHH	glszm	GrayLevelVariance	14.42105263
wavelet-LHH	glszm	ZoneVariance	3.218836565
wavelet-LHH	glszm	GrayLevelNonUniformityNormalized	0.091412742
wavelet-LHH	glszm	SizeZoneNonUniformityNormalized	0.385041551
wavelet-LHH	glszm	SizeZoneNonUniformity	7.315789474
wavelet-LHH	glszm	GrayLevelNonUniformity	1.736842105
wavelet-LHH	glszm	LargeAreaEmphasis	8.105263158

wavelet-LHH	glszm	SmallAreaHighGrayLevelEmphasis	48.70258056
wavelet-LHH	glszm	ZonePercentage	0.452380952
wavelet-LHH	glszm	LargeAreaLowGrayLevelEmphasis	0.448763799
wavelet-LHH	glszm	LargeAreaHighGrayLevelEmphasis	281.1578947
wavelet-LHH	glszm	HighGrayLevelZoneEmphasis	63.42105263
wavelet-LHH	glszm	SmallAreaEmphasis	0.63028464
wavelet-LHH	glszm	LowGrayLevelZoneEmphasis	0.104951565
wavelet-LHH	glszm	ZoneEntropy	3.892407119
wavelet-LHH	glszm	SmallAreaLowGrayLevelEmphasis	0.090034189
wavelet-LHH	ngtdm	Coarseness	0.104492739
wavelet-LHH	ngtdm	Complexity	157.9619647
wavelet-LHH	ngtdm	Strength	5.837226494
wavelet-LHH	ngtdm	Contrast	0.283648042
wavelet-LHH	ngtdm	Busyness	0.163657087
wavelet-LLH	gldm	GrayLevelVariance	102.3718821
wavelet-LLH	gldm	HighGrayLevelEmphasis	732.1428571
wavelet-LLH	gldm	DependenceEntropy	4.784941827
wavelet-LLH	gldm	DependenceNonUniformity	22.14285714
wavelet-LLH	gldm	GrayLevelNonUniformity	2.047619048
wavelet-LLH	gldm	SmallDependenceEmphasis	0.751322751
wavelet-LLH	gldm	SmallDependenceHighGrayLevelEmphasis	526.5674603
wavelet-LLH	gldm	DependenceNonUniformityNormalized	0.527210884
wavelet-LLH	gldm	LargeDependenceEmphasis	2.523809524
wavelet-LLH	gldm	LargeDependenceLowGrayLevelEmphasis	0.029639753
wavelet-LLH	gldm	DependenceVariance	0.482993197
wavelet-LLH	gldm	LargeDependenceHighGrayLevelEmphasis	2050
wavelet-LLH	gldm	SmallDependenceLowGrayLevelEmphasis	0.026999835
wavelet-LLH	gldm	LowGrayLevelEmphasis	0.027414719
wavelet-LLH	glcm	JointAverage	25.02582266
wavelet-LLH	glcm	SumAverage	50.05164533
wavelet-LLH	glcm	JointEntropy	5.272811415
wavelet-LLH	glcm	ClusterShade	-74.08000361
wavelet-LLH	glcm	MaximumProbability	0.042784704
wavelet-LLH	glcm	Idmn	0.951193174
wavelet-LLH	glcm	JointEnergy	0.027046246
wavelet-LLH	glcm	Contrast	114.8797523
wavelet-LLH	glcm	DifferenceEntropy	3.381485907
wavelet-LLH	glcm	InverseVariance	0.113158933
wavelet-LLH	glcm	DifferenceVariance	22.40860027
wavelet-LLH	glcm	Idn	0.846352376
wavelet-LLH	glcm	Idm	0.09660343
wavelet-LLH	glcm	Correlation	0.423266525
wavelet-LLH	glcm	Autocorrelation	669.6267301
wavelet-LLH	glcm	SumEntropy	4.00692649
wavelet-LLH	glcm	SumSquares	100.0940699
wavelet-LLH	glcm	ClusterProminence	188080.9168
wavelet-LLH	glcm	Imc2	0.999353518
wavelet-LLH	glcm	Imc1	-0.777639323
wavelet-LLH	glcm	DifferenceAverage	8.963380649
wavelet-LLH	glcm	Id	0.178491335
wavelet-LLH	glcm	ClusterTendency	285.4965275
wavelet-LLH	firstorder	InterquartileRange	381.2161769
wavelet-LLH	firstorder	Skewness	-0.208168783
wavelet-LLH	firstorder	Uniformity	0.048752834

wavelet-LLH	firstorder	Median	55.12752586
wavelet-LLH	firstorder	Energy	2676664.117
wavelet-LLH	firstorder	RobustMeanAbsoluteDeviation	154.0590715
wavelet-LLH	firstorder	MeanAbsoluteDeviation	213.3809871
wavelet-LLH	firstorder	TotalEnergy	72269931.16
wavelet-LLH	firstorder	Maximum	545.4966459
wavelet-LLH	firstorder	RootMeanSquared	252.4482086
wavelet-LLH	firstorder	90Percentile	330.4485943
wavelet-LLH	firstorder	Minimum	-589.4914569
wavelet-LLH	firstorder	Entropy	4.558518613
wavelet-LLH	firstorder	Range	1134.988103
wavelet-LLH	firstorder	Variance	63526.06132
wavelet-LLH	firstorder	10Percentile	-271.9824783
wavelet-LLH	firstorder	Kurtosis	2.526029773
wavelet-LLH	firstorder	Mean	14.28414141
wavelet-LLH	glrlm	ShortRunLowGrayLevelEmphasis	0.027874216
wavelet-LLH	glrlm	GrayLevelVariance	103.4525414
wavelet-LLH	glrlm	LowGrayLevelRunEmphasis	0.027894322
wavelet-LLH	glrlm	GrayLevelNonUniformityNormalized	0.048090294
wavelet-LLH	glrlm	RunVariance	0.020441523
wavelet-LLH	glrlm	GrayLevelNonUniformity	1.989027368
wavelet-LLH	glrlm	LongRunEmphasis	1.059902022
wavelet-LLH	glrlm	ShortRunHighGrayLevelEmphasis	719.3700749
wavelet-LLH	glrlm	RunLengthNonUniformity	40.18580363
wavelet-LLH	glrlm	ShortRunEmphasis	0.987398664
wavelet-LLH	glrlm	LongRunHighGrayLevelEmphasis	781.0210221
wavelet-LLH	glrlm	RunPercentage	0.983516484
wavelet-LLH	glrlm	LongRunLowGrayLevelEmphasis	0.0279853
wavelet-LLH	glrlm	RunEntropy	4.599312715
wavelet-LLH	glrlm	HighGrayLevelRunEmphasis	729.9908626
wavelet-LLH	glrlm	RunLengthNonUniformityNormalized	0.970156839
wavelet-LLH	glszm	GrayLevelVariance	115.6306228
wavelet-LLH	glszm	ZoneVariance	0.415224913
wavelet-LLH	glszm	GrayLevelNonUniformityNormalized	0.043252595
wavelet-LLH	glszm	SizeZoneNonUniformityNormalized	0.737024221
wavelet-LLH	glszm	SizeZoneNonUniformity	25.05882353
wavelet-LLH	glszm	GrayLevelNonUniformity	1.470588235
wavelet-LLH	glszm	LargeAreaEmphasis	1.941176471
wavelet-LLH	glszm	SmallAreaHighGrayLevelEmphasis	611.4199346
wavelet-LLH	glszm	ZonePercentage	0.80952381
wavelet-LLH	glszm	LargeAreaLowGrayLevelEmphasis	0.034872726
wavelet-LLH	glszm	LargeAreaHighGrayLevelEmphasis	1514.058824
wavelet-LLH	glszm	HighGrayLevelZoneEmphasis	707.2647059
wavelet-LLH	glszm	SmallAreaEmphasis	0.880106209
wavelet-LLH	glszm	LowGrayLevelZoneEmphasis	0.033490143
wavelet-LLH	glszm	ZoneEntropy	4.771142621
wavelet-LLH	glszm	SmallAreaLowGrayLevelEmphasis	0.033270415
wavelet-LLH	ngtdm	Coarseness	0.10836105
wavelet-LLH	ngtdm	Complexity	1960.683629
wavelet-LLH	ngtdm	Strength	61.42929812
wavelet-LLH	ngtdm	Contrast	1.421096237
wavelet-LLH	ngtdm	Busyness	0.019025778
wavelet-LLL	gldm	GrayLevelVariance	323.4875283
wavelet-LLL	gldm	HighGrayLevelEmphasis	958.047619

wavelet-LLL	gldm	DependenceEntropy	4.766968315
wavelet-LLL	gldm	DependenceNonUniformity	28.28571429
wavelet-LLL	gldm	GrayLevelNonUniformity	1.80952381
wavelet-LLL	gldm	SmallDependenceEmphasis	0.843915344
wavelet-LLL	gldm	SmallDependenceHighGrayLevelEmphasis	834.9484127
wavelet-LLL	gldm	DependenceNonUniformityNormalized	0.673469388
wavelet-LLL	gldm	LargeDependenceEmphasis	2.047619048
wavelet-LLL	gldm	LargeDependenceLowGrayLevelEmphasis	0.040811946
wavelet-LLL	gldm	DependenceVariance	0.394557823
wavelet-LLL	gldm	LargeDependenceHighGrayLevelEmphasis	1673.619048
wavelet-LLL	gldm	SmallDependenceLowGrayLevelEmphasis	0.037051847
wavelet-LLL	gldm	LowGrayLevelEmphasis	0.037466013
wavelet-LLL	glcm	JointAverage	29.63836866
wavelet-LLL	glcm	SumAverage	59.27673731
wavelet-LLL	glcm	JointEntropy	5.331001552
wavelet-LLL	glcm	ClusterShade	2701.607508
wavelet-LLL	glcm	MaximumProbability	0.036196385
wavelet-LLL	glcm	Idmn	0.903795298
wavelet-LLL	glcm	JointEnergy	0.025609455
wavelet-LLL	glcm	Contrast	524.3190794
wavelet-LLL	glcm	DifferenceEntropy	3.957779503
wavelet-LLL	glcm	InverseVariance	0.060580939
wavelet-LLL	glcm	DifferenceVariance	150.3313171
wavelet-LLL	glcm	Idn	0.794509466
wavelet-LLL	glcm	Idm	0.056999004
wavelet-LLL	glcm	Correlation	0.232059899
wavelet-LLL	glcm	Autocorrelation	960.2332311
wavelet-LLL	glcm	SumEntropy	4.224246097
wavelet-LLL	glcm	SumSquares	341.7321199
wavelet-LLL	glcm	ClusterProminence	1432711.392
wavelet-LLL	glcm	Imc2	0.999776723
wavelet-LLL	glcm	Imc1	-0.844057861
wavelet-LLL	glcm	DifferenceAverage	18.9433259
wavelet-LLL	glcm	Id	0.115188626
wavelet-LLL	glcm	ClusterTendency	842.6094003
wavelet-LLL	firstorder	InterquartileRange	782.7993437
wavelet-LLL	firstorder	Skewness	0.437208125
wavelet-LLL	firstorder	Uniformity	0.0430839
wavelet-LLL	firstorder	Median	-1784.184237
wavelet-LLL	firstorder	Energy	127533134.7
wavelet-LLL	firstorder	RobustMeanAbsoluteDeviation	309.2336048
wavelet-LLL	firstorder	MeanAbsoluteDeviation	397.4358188
wavelet-LLL	firstorder	TotalEnergy	3443394637
wavelet-LLL	firstorder	Maximum	-669.5559434
wavelet-LLL	firstorder	RootMeanSquared	1742.556515
wavelet-LLL	firstorder	90Percentile	-1086.245463
wavelet-LLL	firstorder	Minimum	-2292.082486
wavelet-LLL	firstorder	Entropy	4.701375756
wavelet-LLL	firstorder	Range	1622.526543
wavelet-LLL	firstorder	Variance	203543.5575
wavelet-LLL	firstorder	10Percentile	-2211.007892
wavelet-LLL	firstorder	Kurtosis	2.021714169
wavelet-LLL	firstorder	Mean	-1683.139819
wavelet-LLL	glrlm	ShortRunLowGrayLevelEmphasis	0.037831315

wavelet-LLL	glrlm	GrayLevelVariance	325.6977316
wavelet-LLL	glrlm	LowGrayLevelRunEmphasis	0.037857016
wavelet-LLL	glrlm	GrayLevelNonUniformityNormalized	0.042778327
wavelet-LLL	glrlm	RunVariance	0.010975495
wavelet-LLL	glrlm	GrayLevelNonUniformity	1.777155365
wavelet-LLL	glrlm	LongRunEmphasis	1.034052533
wavelet-LLL	glrlm	ShortRunHighGrayLevelEmphasis	954.7127937
wavelet-LLL	glrlm	RunLengthNonUniformity	40.64577861
wavelet-LLL	glrlm	ShortRunEmphasis	0.991486867
wavelet-LLL	glrlm	LongRunHighGrayLevelEmphasis	985.161668
wavelet-LLL	glrlm	RunPercentage	0.989010989
wavelet-LLL	glrlm	LongRunLowGrayLevelEmphasis	0.03795982
wavelet-LLL	glrlm	RunEntropy	4.726095496
wavelet-LLL	glrlm	HighGrayLevelRunEmphasis	960.8025686
wavelet-LLL	glrlm	RunLengthNonUniformityNormalized	0.978049009
wavelet-LLL	glszm	GrayLevelVariance	350.0803506
wavelet-LLL	glszm	ZoneVariance	0.224981738
wavelet-LLL	glszm	GrayLevelNonUniformityNormalized	0.041636231
wavelet-LLL	glszm	SizeZoneNonUniformityNormalized	0.84806428
wavelet-LLL	glszm	SizeZoneNonUniformity	31.37837838
wavelet-LLL	glszm	GrayLevelNonUniformity	1.540540541
wavelet-LLL	glszm	LargeAreaEmphasis	1.513513514
wavelet-LLL	glszm	SmallAreaHighGrayLevelEmphasis	921.4804805
wavelet-LLL	glszm	ZonePercentage	0.880952381
wavelet-LLL	glszm	LargeAreaLowGrayLevelEmphasis	0.043542923
wavelet-LLL	glszm	LargeAreaHighGrayLevelEmphasis	1402.486486
wavelet-LLL	glszm	HighGrayLevelZoneEmphasis	976.4324324
wavelet-LLL	glszm	SmallAreaEmphasis	0.931681682
wavelet-LLL	glszm	LowGrayLevelZoneEmphasis	0.042177683
wavelet-LLL	glszm	ZoneEntropy	4.736216203
wavelet-LLL	glszm	SmallAreaLowGrayLevelEmphasis	0.042009331
wavelet-LLL	ngtdm	Coarseness	0.044692289
wavelet-LLL	ngtdm	Complexity	9107.814964
wavelet-LLL	ngtdm	Strength	76.38151207
wavelet-LLL	ngtdm	Contrast	9.969524054
wavelet-LLL	ngtdm	Busyness	0.042833155
wavelet-HHL	gldm	GrayLevelVariance	5.283446712
wavelet-HHL	gldm	HighGrayLevelEmphasis	34.23809524
wavelet-HHL	gldm	DependenceEntropy	4.474952542
wavelet-HHL	gldm	DependenceNonUniformity	8.761904762
wavelet-HHL	gldm	GrayLevelNonUniformity	5.952380952
wavelet-HHL	gldm	SmallDependenceEmphasis	0.280390212
wavelet-HHL	gldm	SmallDependenceHighGrayLevelEmphasis	10.27621032
wavelet-HHL	gldm	DependenceNonUniformityNormalized	0.20861678
wavelet-HHL	gldm	LargeDependenceEmphasis	9.952380952
wavelet-HHL	gldm	LargeDependenceLowGrayLevelEmphasis	0.565605581
wavelet-HHL	gldm	DependenceVariance	1.789115646
wavelet-HHL	gldm	LargeDependenceHighGrayLevelEmphasis	291.0952381
wavelet-HHL	gldm	SmallDependenceLowGrayLevelEmphasis	0.063351477
wavelet-HHL	gldm	LowGrayLevelEmphasis	0.102202668
wavelet-HHL	glcm	JointAverage	5.424981701
wavelet-HHL	glcm	SumAverage	10.8499634
wavelet-HHL	glcm	JointEntropy	4.63501912
wavelet-HHL	glcm	ClusterShade	4.405888069

wavelet-HHL	glcm	MaximumProbability	0.079569853
wavelet-HHL	glcm	Idmn	0.933435297
wavelet-HHL	glcm	JointEnergy	0.045244809
wavelet-HHL	glcm	Contrast	10.18501652
wavelet-HHL	glcm	DifferenceEntropy	2.471386361
wavelet-HHL	glcm	InverseVariance	0.356182532
wavelet-HHL	glcm	DifferenceVariance	3.964792767
wavelet-HHL	glcm	Idn	0.837512142
wavelet-HHL	glcm	Idm	0.34488626
wavelet-HHL	glcm	Correlation	-0.066154885
wavelet-HHL	glcm	Autocorrelation	29.0289215
wavelet-HHL	glcm	SumEntropy	3.015527511
wavelet-HHL	glcm	SumSquares	4.680685657
wavelet-HHL	glcm	ClusterProminence	251.5130734
wavelet-HHL	glcm	Imc2	0.946512888
wavelet-HHL	glcm	Imc1	-0.411681732
wavelet-HHL	glcm	DifferenceAverage	2.416166692
wavelet-HHL	glcm	Id	0.421670882
wavelet-HHL	glcm	ClusterTendency	8.537726108
wavelet-HHL	firstorder	InterquartileRange	71.47477029
wavelet-HHL	firstorder	Skewness	0.270283272
wavelet-HHL	firstorder	Uniformity	0.141723356
wavelet-HHL	firstorder	Median	-6.132305792
wavelet-HHL	firstorder	Energy	141506.4599
wavelet-HHL	firstorder	RobustMeanAbsoluteDeviation	27.01439492
wavelet-HHL	firstorder	MeanAbsoluteDeviation	44.77495541
wavelet-HHL	firstorder	TotalEnergy	3820674.418
wavelet-HHL	firstorder	Maximum	131.288632
wavelet-HHL	firstorder	RootMeanSquared	58.04482257
wavelet-HHL	firstorder	90Percentile	65.1519612
wavelet-HHL	firstorder	Minimum	-123.7233071
wavelet-HHL	firstorder	Entropy	3.063973377
wavelet-HHL	firstorder	Range	255.0119391
wavelet-HHL	firstorder	Variance	3341.440718
wavelet-HHL	firstorder	10Percentile	-86.38198579
wavelet-HHL	firstorder	Kurtosis	3.083201261
wavelet-HHL	firstorder	Mean	-5.268843206
wavelet-HHL	glrlm	ShortRunLowGrayLevelEmphasis	0.102827313
wavelet-HHL	glrlm	GrayLevelVariance	5.507853999
wavelet-HHL	glrlm	LowGrayLevelRunEmphasis	0.105987112
wavelet-HHL	glrlm	GrayLevelNonUniformityNormalized	0.138411291
wavelet-HHL	glrlm	RunVariance	0.070671436
wavelet-HHL	glrlm	GrayLevelNonUniformity	5.398873621
wavelet-HHL	glrlm	LongRunEmphasis	1.234754388
wavelet-HHL	glrlm	ShortRunHighGrayLevelEmphasis	32.80801246
wavelet-HHL	glrlm	RunLengthNonUniformity	33.57312288
wavelet-HHL	glrlm	ShortRunEmphasis	0.941311403
wavelet-HHL	glrlm	LongRunHighGrayLevelEmphasis	41.61357778
wavelet-HHL	glrlm	RunPercentage	0.928571429
wavelet-HHL	glrlm	LongRunLowGrayLevelEmphasis	0.118626307
wavelet-HHL	glrlm	RunEntropy	3.354270883
wavelet-HHL	glrlm	HighGrayLevelRunEmphasis	34.56912552
wavelet-HHL	glrlm	RunLengthNonUniformityNormalized	0.858657129
wavelet-HHL	glszm	GrayLevelVariance	9.795555556

wavelet-HHL	glszm	ZoneVariance	5.626666667
wavelet-HHL	glszm	GrayLevelNonUniformityNormalized	0.102222222
wavelet-HHL	glszm	SizeZoneNonUniformityNormalized	0.28
wavelet-HHL	glszm	SizeZoneNonUniformity	4.2
wavelet-HHL	glszm	GrayLevelNonUniformity	1.533333333
wavelet-HHL	glszm	LargeAreaEmphasis	13.46666667
wavelet-HHL	glszm	SmallAreaHighGrayLevelEmphasis	19.13161385
wavelet-HHL	glszm	ZonePercentage	0.357142857
wavelet-HHL	glszm	LargeAreaLowGrayLevelEmphasis	0.772609837
wavelet-HHL	glszm	LargeAreaHighGrayLevelEmphasis	415.6
wavelet-HHL	glszm	HighGrayLevelZoneEmphasis	37.53333333
wavelet-HHL	glszm	SmallAreaEmphasis	0.530994803
wavelet-HHL	glszm	LowGrayLevelZoneEmphasis	0.193220665
wavelet-HHL	glszm	ZoneEntropy	3.773557262
wavelet-HHL	glszm	SmallAreaLowGrayLevelEmphasis	0.165939604
wavelet-HHL	ngtdm	Coarseness	0.136714423
wavelet-HHL	ngtdm	Complexity	81.45958878
wavelet-HHL	ngtdm	Strength	4.404918529
wavelet-HHL	ngtdm	Contrast	0.178964444
wavelet-HHL	ngtdm	Busyness	0.143288117
wavelet-HLH	gldm	GrayLevelVariance	8.092970522
wavelet-HLH	gldm	HighGrayLevelEmphasis	78.33333333
wavelet-HLH	gldm	DependenceEntropy	4.403989446
wavelet-HLH	gldm	DependenceNonUniformity	10.9047619
wavelet-HLH	gldm	GrayLevelNonUniformity	5.19047619
wavelet-HLH	gldm	SmallDependenceEmphasis	0.38504795
wavelet-HLH	gldm	SmallDependenceHighGrayLevelEmphasis	27.54321594
wavelet-HLH	gldm	DependenceNonUniformityNormalized	0.259637188
wavelet-HLH	gldm	LargeDependenceEmphasis	9.380952381
wavelet-HLH	gldm	LargeDependenceLowGrayLevelEmphasis	0.159391316
wavelet-HLH	gldm	DependenceVariance	2.768707483
wavelet-HLH	gldm	LargeDependenceHighGrayLevelEmphasis	765.6666667
wavelet-HLH	gldm	SmallDependenceLowGrayLevelEmphasis	0.033271183
wavelet-HLH	gldm	LowGrayLevelEmphasis	0.043767419
wavelet-HLH	glcm	JointAverage	8.507588523
wavelet-HLH	glcm	SumAverage	17.01517705
wavelet-HLH	glcm	JointEntropy	4.843446544
wavelet-HLH	glcm	ClusterShade	-14.70934127
wavelet-HLH	glcm	MaximumProbability	0.075150104
wavelet-HLH	glcm	Idmn	0.953117665
wavelet-HLH	glcm	JointEnergy	0.039301435
wavelet-HLH	glcm	Contrast	12.20525102
wavelet-HLH	glcm	DifferenceEntropy	2.553714328
wavelet-HLH	glcm	InverseVariance	0.348674856
wavelet-HLH	glcm	DifferenceVariance	4.752470884
wavelet-HLH	glcm	Idn	0.860317873
wavelet-HLH	glcm	Idm	0.31448791
wavelet-HLH	glcm	Correlation	0.066931457
wavelet-HLH	glcm	Autocorrelation	73.14129952
wavelet-HLH	glcm	SumEntropy	3.373601499
wavelet-HLH	glcm	SumSquares	6.771927183
wavelet-HLH	glcm	ClusterProminence	758.6774827
wavelet-HLH	glcm	Imc2	0.964965535
wavelet-HLH	glcm	Imc1	-0.44469567

wavelet-HLH	glcm	DifferenceAverage	2.690322379
wavelet-HLH	glcm	Id	0.394239256
wavelet-HLH	glcm	ClusterTendency	14.88245771
wavelet-HLH	firstorder	InterquartileRange	71.81783454
wavelet-HLH	firstorder	Skewness	-0.174465399
wavelet-HLH	firstorder	Uniformity	0.123582766
wavelet-HLH	firstorder	Median	3.780994058
wavelet-HLH	firstorder	Energy	204883.6162
wavelet-HLH	firstorder	RobustMeanAbsoluteDeviation	31.52981515
wavelet-HLH	firstorder	MeanAbsoluteDeviation	52.81062976
wavelet-HLH	firstorder	TotalEnergy	5531857.639
wavelet-HLH	firstorder	Maximum	165.2726008
wavelet-HLH	firstorder	RootMeanSquared	69.84397855
wavelet-HLH	firstorder	90Percentile	63.70944944
wavelet-HLH	firstorder	Minimum	-188.2697636
wavelet-HLH	firstorder	Entropy	3.26849934
wavelet-HLH	firstorder	Range	353.5423644
wavelet-HLH	firstorder	Variance	4862.332597
wavelet-HLH	firstorder	10Percentile	-101.7825146
wavelet-HLH	firstorder	Kurtosis	3.607990632
wavelet-HLH	firstorder	Mean	-3.981047872
wavelet-HLH	glrlm	ShortRunLowGrayLevelEmphasis	0.044948043
wavelet-HLH	glrlm	GrayLevelVariance	8.390731198
wavelet-HLH	glrlm	LowGrayLevelRunEmphasis	0.045669378
wavelet-HLH	glrlm	GrayLevelNonUniformityNormalized	0.118468509
wavelet-HLH	glrlm	RunVariance	0.063989863
wavelet-HLH	glrlm	GrayLevelNonUniformity	4.676210248
wavelet-HLH	glrlm	LongRunEmphasis	1.201883974
wavelet-HLH	glrlm	ShortRunHighGrayLevelEmphasis	74.07437907
wavelet-HLH	glrlm	RunLengthNonUniformity	34.94796956
wavelet-HLH	glrlm	ShortRunEmphasis	0.951839009
wavelet-HLH	glrlm	LongRunHighGrayLevelEmphasis	94.71835886
wavelet-HLH	glrlm	RunPercentage	0.93956044
wavelet-HLH	glrlm	LongRunLowGrayLevelEmphasis	0.04866879
wavelet-HLH	glrlm	RunEntropy	3.507627062
wavelet-HLH	glrlm	HighGrayLevelRunEmphasis	78.05348688
wavelet-HLH	glrlm	RunLengthNonUniformityNormalized	0.883145745
wavelet-HLH	glszm	GrayLevelVariance	11.59
wavelet-HLH	glszm	ZoneVariance	3.99
wavelet-HLH	glszm	GrayLevelNonUniformityNormalized	0.105
wavelet-HLH	glszm	SizeZoneNonUniformityNormalized	0.4
wavelet-HLH	glszm	SizeZoneNonUniformity	8
wavelet-HLH	glszm	GrayLevelNonUniformity	2.1
wavelet-HLH	glszm	LargeAreaEmphasis	8.4
wavelet-HLH	glszm	SmallAreaHighGrayLevelEmphasis	43.52951389
wavelet-HLH	glszm	ZonePercentage	0.476190476
wavelet-HLH	glszm	LargeAreaLowGrayLevelEmphasis	0.178246813
wavelet-HLH	glszm	LargeAreaHighGrayLevelEmphasis	687
wavelet-HLH	glszm	HighGrayLevelZoneEmphasis	74
wavelet-HLH	glszm	SmallAreaEmphasis	0.630131173
wavelet-HLH	glszm	LowGrayLevelZoneEmphasis	0.074089138
wavelet-HLH	glszm	ZoneEntropy	3.921928095
wavelet-HLH	glszm	SmallAreaLowGrayLevelEmphasis	0.066544522
wavelet-HLH	ngtdm	Coarseness	0.150105267

wavelet-HLH	ngtdm	Complexity	109.0991824
wavelet-HLH	ngtdm	Strength	6.982649274
wavelet-HLH	ngtdm	Contrast	0.238882352
wavelet-HLH	ngtdm	Busyness	0.073170408

Image type	Feature Class	Feature Name	3: RTSTRUCT: C99-DNU-A_segment_GTV LUL
general	info	BoundingBox	(0, 0, 1, 5, 5, 3)
general	info	EnabledImageTypes	{u'Original': {}, u'Wavelet': {}}
general	info	GeneralSettings	{'distances': [1], 'additionalInfo': True, 'force2D': False, 'interpolator': 'sitkBSpline', 'resampledPixelSpacing': [3.0], 'label': 1, 'normalizeScale': 1, 'normalize': False, 'force2Ddimension': 0, 'removeOutliers': None, 'minimumROISize': None, u'binWidth': 25.0, 'minimumROIDimensions': 1, u'correctMask': True, 'preCrop': False, u'symmetricalGLCM': True, 'resegmentRange': None, 'padDistance': 5}
general	info	ImageHash	525b135c269d171cc15012c9eca7023d55d92887
general	info	ImageSpacing	(0.9375, 0.9375, 2.0)
general	info	MaskHash	743209826dbc4da7c3f50119046ff4983a2f0eac
general	info	NumpyVersion	1.13.1
general	info	PyWaveletVersion	0.5.2
general	info	SimpleITKVersion	1.1.0
general	info	Version	1.3.0.post102+g1b0eb74
general	info	VolumeNum	1
general	info	VoxelNum	37
original	shape	Maximum3DDiameter	15.29705854
original	shape	Maximum2DDiameterSlice	13.41640786
original	shape	Sphericity	0.880314135
original	shape	MinorAxis	13.16705797
original	shape	Elongation	0.86650253
original	shape	SurfaceVolumeRatio	0.549529848
original	shape	Volume	999
original	shape	MajorAxis	15.19563707
original	shape	SurfaceArea	548.9803181
original	shape	Flatness	0.459025789
original	shape	LeastAxis	6.975189297
original	shape	Maximum2DDiameterColumn	13.41640786
original	shape	Maximum2DDiameterRow	13.41640786
original	gldm	GrayLevelVariance	89.10591673
original	gldm	HighGrayLevelEmphasis	392.0540541
original	gldm	DependenceEntropy	4.594456406
original	gldm	DependenceNonUniformity	24.45945946
original	gldm	GrayLevelNonUniformity	1.756756757
original	gldm	SmallDependenceEmphasis	0.837837838
original	gldm	SmallDependenceHighGrayLevelEmphasis	332.2972973
original	gldm	DependenceNonUniformityNormalized	0.661066472
original	gldm	LargeDependenceEmphasis	1.648648649
original	gldm	LargeDependenceLowGrayLevelEmphasis	0.0466436
original	gldm	DependenceVariance	0.169466764
original	gldm	LargeDependenceHighGrayLevelEmphasis	631.0810811
original	gldm	SmallDependenceLowGrayLevelEmphasis	0.034608807
original	gldm	LowGrayLevelEmphasis	0.037015766
original	glcm	JointAverage	19.73113814
original	glcm	SumAverage	39.46227628

original	glcm	JointEntropy	4.924174793
original	glcm	ClusterShade	-440.4319037
original	glcm	MaximumProbability	0.046532801
original	glcm	Idmn	0.880088171
original	glcm	JointEnergy	0.036017823
original	glcm	Contrast	197.151978
original	glcm	DifferenceEntropy	3.479337987
original	glcm	InverseVariance	0.091016954
original	glcm	DifferenceVariance	59.05593789
original	glcm	Idn	0.772483447
original	glcm	Idm	0.071087495
original	glcm	Correlation	-0.045415534
original	glcm	Autocorrelation	386.3203841
original	glcm	SumEntropy	3.679270697
original	glcm	SumSquares	94.0089715
original	glcm	ClusterProminence	87964.80329
original	glcm	Imc2	0.999535688
original	glcm	Imc1	-0.842945504
original	glcm	DifferenceAverage	11.58144954
original	glcm	Id	0.14737739
original	glcm	ClusterTendency	178.8839081
original	firstorder	InterquartileRange	399
original	firstorder	Skewness	0.135084802
original	firstorder	Uniformity	0.047479912
original	firstorder	Median	-410
original	firstorder	Energy	8055267
original	firstorder	RobustMeanAbsoluteDeviation	151.4268728
original	firstorder	MeanAbsoluteDeviation	198.3403944
original	firstorder	TotalEnergy	217492209
original	firstorder	Maximum	26
original	firstorder	RootMeanSquared	466.5939551
original	firstorder	90Percentile	-46.4
original	firstorder	Minimum	-811
original	firstorder	Entropy	4.519999987
original	firstorder	Range	837
original	firstorder	Variance	55779.80862
original	firstorder	10Percentile	-705.8
original	firstorder	Kurtosis	1.906600822
original	firstorder	Mean	-402.4054054
original	glrlm	ShortRunLowGrayLevelEmphasis	0.037112827
original	glrlm	GrayLevelVariance	88.87386046
original	glrlm	LowGrayLevelRunEmphasis	0.037210692
original	glrlm	GrayLevelNonUniformityNormalized	0.047459455
original	glrlm	RunVariance	0.008288854
original	glrlm	GrayLevelNonUniformity	1.741431541
original	glrlm	LongRunEmphasis	1.026373626
original	glrlm	ShortRunHighGrayLevelEmphasis	389.8314523
original	glrlm	RunLengthNonUniformity	36.11208791
original	glrlm	ShortRunEmphasis	0.993406593
original	glrlm	LongRunHighGrayLevelEmphasis	401.979804
original	glrlm	RunPercentage	0.991683992
original	glrlm	LongRunLowGrayLevelEmphasis	0.037602153
original	glrlm	RunEntropy	4.525242074
original	glrlm	HighGrayLevelRunEmphasis	392.2611227
original	glrlm	RunLengthNonUniformityNormalized	0.983422292

original	glszm	GrayLevelVariance	86.00183655
original	glszm	ZoneVariance	0.106519743
original	glszm	GrayLevelNonUniformityNormalized	0.046831956
original	glszm	SizeZoneNonUniformityNormalized	0.786960514
original	glszm	SizeZoneNonUniformity	25.96969697
original	glszm	GrayLevelNonUniformity	1.545454545
original	glszm	LargeAreaEmphasis	1.363636364
original	glszm	SmallAreaHighGrayLevelEmphasis	361.4090909
original	glszm	ZonePercentage	0.891891892
original	glszm	LargeAreaLowGrayLevelEmphasis	0.045100807
original	glszm	LargeAreaHighGrayLevelEmphasis	528.9090909
original	glszm	HighGrayLevelZoneEmphasis	394.9090909
original	glszm	SmallAreaEmphasis	0.909090909
original	glszm	LowGrayLevelZoneEmphasis	0.039703384
original	glszm	ZoneEntropy	4.597276316
original	glszm	SmallAreaLowGrayLevelEmphasis	0.038354029
original	ngtdm	Coarseness	0.06724255
original	ngtdm	Complexity	2842.334894
original	ngtdm	Strength	29.50717956
original	ngtdm	Contrast	2.553370895
original	ngtdm	Busyness	0.052047562
wavelet-HHH	gldm	GrayLevelVariance	14.12710007
wavelet-HHH	gldm	HighGrayLevelEmphasis	193.1081081
wavelet-HHH	gldm	DependenceEntropy	4.270132082
wavelet-HHH	gldm	DependenceNonUniformity	11.86486486
wavelet-HHH	gldm	GrayLevelNonUniformity	4.297297297
wavelet-HHH	gldm	SmallDependenceEmphasis	0.470908408
wavelet-HHH	gldm	SmallDependenceHighGrayLevelEmphasis	100.0760135
wavelet-HHH	gldm	DependenceNonUniformityNormalized	0.320672023
wavelet-HHH	gldm	LargeDependenceEmphasis	4.621621622
wavelet-HHH	gldm	LargeDependenceLowGrayLevelEmphasis	0.057778426
wavelet-HHH	gldm	DependenceVariance	0.72899927
wavelet-HHH	gldm	LargeDependenceHighGrayLevelEmphasis	795.5405405
wavelet-HHH	gldm	SmallDependenceLowGrayLevelEmphasis	0.029746509
wavelet-HHH	gldm	LowGrayLevelEmphasis	0.033163264
wavelet-HHH	glcm	JointAverage	13.55762681
wavelet-HHH	glcm	SumAverage	27.11525363
wavelet-HHH	glcm	JointEntropy	4.554720842
wavelet-HHH	glcm	ClusterShade	-8.007795321
wavelet-HHH	glcm	MaximumProbability	0.089128501
wavelet-HHH	glcm	Idmn	0.962697711
wavelet-HHH	glcm	JointEnergy	0.050062406
wavelet-HHH	glcm	Contrast	20.67708929
wavelet-HHH	glcm	DifferenceEntropy	2.608209827
wavelet-HHH	glcm	InverseVariance	0.239821454
wavelet-HHH	glcm	DifferenceVariance	7.205386074
wavelet-HHH	glcm	Idn	0.871260934
wavelet-HHH	glcm	Idm	0.243848086
wavelet-HHH	glcm	Correlation	-0.106429154
wavelet-HHH	glcm	Autocorrelation	183.1917966
wavelet-HHH	glcm	SumEntropy	3.022137024
wavelet-HHH	glcm	SumSquares	9.569469086
wavelet-HHH	glcm	ClusterProminence	1201.253689
wavelet-HHH	glcm	Imc2	0.981620729
wavelet-HHH	glcm	Imc1	-0.550339467

wavelet-HHH	glcm	DifferenceAverage	3.534947395
wavelet-HHH	glcm	Id	0.337737212
wavelet-HHH	glcm	ClusterTendency	17.60078705
wavelet-HHH	firstorder	InterquartileRange	102.6843246
wavelet-HHH	firstorder	Skewness	-0.68652119
wavelet-HHH	firstorder	Uniformity	0.11614317
wavelet-HHH	firstorder	Median	6.402888221
wavelet-HHH	firstorder	Energy	322758.6561
wavelet-HHH	firstorder	RobustMeanAbsoluteDeviation	43.59596729
wavelet-HHH	firstorder	MeanAbsoluteDeviation	68.99572422
wavelet-HHH	firstorder	TotalEnergy	8714483.715
wavelet-HHH	firstorder	Maximum	204.0486551
wavelet-HHH	firstorder	RootMeanSquared	93.39810984
wavelet-HHH	firstorder	90Percentile	105.014925
wavelet-HHH	firstorder	Minimum	-322.2620546
wavelet-HHH	firstorder	Entropy	3.418516541
wavelet-HHH	firstorder	Range	526.3107097
wavelet-HHH	firstorder	Variance	8714.065456
wavelet-HHH	firstorder	10Percentile	-85.83109045
wavelet-HHH	firstorder	Kurtosis	5.232481489
wavelet-HHH	firstorder	Mean	-3.023485793
wavelet-HHH	glrlm	ShortRunLowGrayLevelEmphasis	0.034034373
wavelet-HHH	glrlm	GrayLevelVariance	14.46898611
wavelet-HHH	glrlm	LowGrayLevelRunEmphasis	0.034229627
wavelet-HHH	glrlm	GrayLevelNonUniformityNormalized	0.114110684
wavelet-HHH	glrlm	RunVariance	0.041802899
wavelet-HHH	glrlm	GrayLevelNonUniformity	4.067319467
wavelet-HHH	glrlm	LongRunEmphasis	1.125441225
wavelet-HHH	glrlm	ShortRunHighGrayLevelEmphasis	189.2487811
wavelet-HHH	glrlm	RunLengthNonUniformity	33.16803197
wavelet-HHH	glrlm	ShortRunEmphasis	0.971229696
wavelet-HHH	glrlm	LongRunHighGrayLevelEmphasis	215.5936238
wavelet-HHH	glrlm	RunPercentage	0.962577963
wavelet-HHH	glrlm	LongRunLowGrayLevelEmphasis	0.03505669
wavelet-HHH	glrlm	RunEntropy	3.548782617
wavelet-HHH	glrlm	HighGrayLevelRunEmphasis	194.0515492
wavelet-HHH	glrlm	RunLengthNonUniformityNormalized	0.92910876
wavelet-HHH	glszm	GrayLevelVariance	21.00680272
wavelet-HHH	glszm	ZoneVariance	1.324263039
wavelet-HHH	glszm	GrayLevelNonUniformityNormalized	0.083900227
wavelet-HHH	glszm	SizeZoneNonUniformityNormalized	0.428571429
wavelet-HHH	glszm	SizeZoneNonUniformity	9
wavelet-HHH	glszm	GrayLevelNonUniformity	1.761904762
wavelet-HHH	glszm	LargeAreaEmphasis	4.428571429
wavelet-HHH	glszm	SmallAreaHighGrayLevelEmphasis	147.7000661
wavelet-HHH	glszm	ZonePercentage	0.567567568
wavelet-HHH	glszm	LargeAreaLowGrayLevelEmphasis	0.074971301
wavelet-HHH	glszm	LargeAreaHighGrayLevelEmphasis	820.5238095
wavelet-HHH	glszm	HighGrayLevelZoneEmphasis	205.1904762
wavelet-HHH	glszm	SmallAreaEmphasis	0.675515873
wavelet-HHH	glszm	LowGrayLevelZoneEmphasis	0.053580197
wavelet-HHH	glszm	ZoneEntropy	4.201841232
wavelet-HHH	glszm	SmallAreaLowGrayLevelEmphasis	0.051483269
wavelet-HHH	ngtdm	Coarseness	0.100110672
wavelet-HHH	ngtdm	Complexity	291.2330809

wavelet-HHH	ngtdm	Strength	10.56022766
wavelet-HHH	ngtdm	Contrast	0.480710037
wavelet-HHH	ngtdm	Busyness	0.066833809
wavelet-HLL	gldm	GrayLevelVariance	124.9306063
wavelet-HLL	gldm	HighGrayLevelEmphasis	873.027027
wavelet-HLL	gldm	DependenceEntropy	4.722966879
wavelet-HLL	gldm	DependenceNonUniformity	29.86486486
wavelet-HLL	gldm	GrayLevelNonUniformity	1.702702703
wavelet-HLL	gldm	SmallDependenceEmphasis	0.918918919
wavelet-HLL	gldm	SmallDependenceHighGrayLevelEmphasis	822.7162162
wavelet-HLL	gldm	DependenceNonUniformityNormalized	0.80715851
wavelet-HLL	gldm	LargeDependenceEmphasis	1.324324324
wavelet-HLL	gldm	LargeDependenceLowGrayLevelEmphasis	0.030592649
wavelet-HLL	gldm	DependenceVariance	0.096420745
wavelet-HLL	gldm	LargeDependenceHighGrayLevelEmphasis	1074.27027
wavelet-HLL	gldm	SmallDependenceLowGrayLevelEmphasis	0.029844867
wavelet-HLL	gldm	LowGrayLevelEmphasis	0.029994423
wavelet-HLL	glcm	JointAverage	27.37480073
wavelet-HLL	glcm	SumAverage	54.74960147
wavelet-HLL	glcm	JointEntropy	4.931912164
wavelet-HLL	glcm	ClusterShade	-995.0772117
wavelet-HLL	glcm	MaximumProbability	0.041543403
wavelet-HLL	glcm	Idmn	0.909874519
wavelet-HLL	glcm	JointEnergy	0.035636872
wavelet-HLL	glcm	Contrast	229.5069536
wavelet-HLL	glcm	DifferenceEntropy	3.343969588
wavelet-HLL	glcm	InverseVariance	0.068055437
wavelet-HLL	glcm	DifferenceVariance	35.27415275
wavelet-HLL	glcm	Idn	0.794418367
wavelet-HLL	glcm	Idm	0.050904981
wavelet-HLL	glcm	Correlation	0.155910634
wavelet-HLL	glcm	Autocorrelation	767.0395145
wavelet-HLL	glcm	SumEntropy	3.71930941
wavelet-HLL	glcm	SumSquares	131.117313
wavelet-HLL	glcm	ClusterProminence	216742.7201
wavelet-HLL	glcm	Imc2	0.999587999
wavelet-HLL	glcm	Imc1	-0.844022477
wavelet-HLL	glcm	DifferenceAverage	12.94322665
wavelet-HLL	glcm	Id	0.124549628
wavelet-HLL	glcm	ClusterTendency	294.9622983
wavelet-HLL	firstorder	InterquartileRange	344.1570597
wavelet-HLL	firstorder	Skewness	-0.270786426
wavelet-HLL	firstorder	Uniformity	0.046018992
wavelet-HLL	firstorder	Median	-35.6110544
wavelet-HLL	firstorder	Energy	2981494.748
wavelet-HLL	firstorder	RobustMeanAbsoluteDeviation	145.9829146
wavelet-HLL	firstorder	MeanAbsoluteDeviation	218.1956508
wavelet-HLL	firstorder	TotalEnergy	80500358.19
wavelet-HLL	firstorder	Maximum	420.5486304
wavelet-HLL	firstorder	RootMeanSquared	283.8678198
wavelet-HLL	firstorder	90Percentile	330.1534579
wavelet-HLL	firstorder	Minimum	-706.157451
wavelet-HLL	firstorder	Entropy	4.614858771
wavelet-HLL	firstorder	Range	1126.706081
wavelet-HLL	firstorder	Variance	77856.53363

wavelet-HLL	firstorder	10Percentile	-434.3378682
wavelet-HLL	firstorder	Kurtosis	2.602140742
wavelet-HLL	firstorder	Mean	-52.19583799
wavelet-HLL	glrlm	ShortRunLowGrayLevelEmphasis	0.03010881
wavelet-HLL	glrlm	GrayLevelVariance	125.3398459
wavelet-HLL	glrlm	LowGrayLevelRunEmphasis	0.030114722
wavelet-HLL	glrlm	GrayLevelNonUniformityNormalized	0.045824237
wavelet-HLL	glrlm	RunVariance	0.004154796
wavelet-HLL	glrlm	GrayLevelNonUniformity	1.688611689
wavelet-HLL	glrlm	LongRunEmphasis	1.012820513
wavelet-HLL	glrlm	ShortRunHighGrayLevelEmphasis	872.1174203
wavelet-HLL	glrlm	RunLengthNonUniformity	36.54700855
wavelet-HLL	glrlm	ShortRunEmphasis	0.996794872
wavelet-HLL	glrlm	LongRunHighGrayLevelEmphasis	882.0613306
wavelet-HLL	glrlm	RunPercentage	0.995841996
wavelet-HLL	glrlm	LongRunLowGrayLevelEmphasis	0.03013837
wavelet-HLL	glrlm	RunEntropy	4.623330499
wavelet-HLL	glrlm	HighGrayLevelRunEmphasis	874.1062024
wavelet-HLL	glrlm	RunLengthNonUniformityNormalized	0.991690408
wavelet-HLL	glszm	GrayLevelVariance	130.4212245
wavelet-HLL	glszm	ZoneVariance	0.053877551
wavelet-HLL	glszm	GrayLevelNonUniformityNormalized	0.043265306
wavelet-HLL	glszm	SizeZoneNonUniformityNormalized	0.892244898
wavelet-HLL	glszm	SizeZoneNonUniformity	31.22857143
wavelet-HLL	glszm	GrayLevelNonUniformity	1.514285714
wavelet-HLL	glszm	LargeAreaEmphasis	1.171428571
wavelet-HLL	glszm	SmallAreaHighGrayLevelEmphasis	860.8642857
wavelet-HLL	glszm	ZonePercentage	0.945945946
wavelet-HLL	glszm	LargeAreaLowGrayLevelEmphasis	0.031919194
wavelet-HLL	glszm	LargeAreaHighGrayLevelEmphasis	993.8285714
wavelet-HLL	glszm	HighGrayLevelZoneEmphasis	887.4571429
wavelet-HLL	glszm	SmallAreaEmphasis	0.957142857
wavelet-HLL	glszm	LowGrayLevelZoneEmphasis	0.031602988
wavelet-HLL	glszm	ZoneEntropy	4.729283017
wavelet-HLL	glszm	SmallAreaLowGrayLevelEmphasis	0.031523937
wavelet-HLL	ngtdm	Coarseness	0.105327005
wavelet-HLL	ngtdm	Complexity	2990.963717
wavelet-HLL	ngtdm	Strength	57.56054837
wavelet-HLL	ngtdm	Contrast	2.507976668
wavelet-HLL	ngtdm	Busyness	0.017277539
wavelet-LHL	gldm	GrayLevelVariance	157.8407597
wavelet-LHL	gldm	HighGrayLevelEmphasis	850.8108108
wavelet-LHL	gldm	DependenceEntropy	4.722966879
wavelet-LHL	gldm	DependenceNonUniformity	24.45945946
wavelet-LHL	gldm	GrayLevelNonUniformity	1.810810811
wavelet-LHL	gldm	SmallDependenceEmphasis	0.837837838
wavelet-LHL	gldm	SmallDependenceHighGrayLevelEmphasis	708.5135135
wavelet-LHL	gldm	DependenceNonUniformityNormalized	0.661066472
wavelet-LHL	gldm	LargeDependenceEmphasis	1.648648649
wavelet-LHL	gldm	LargeDependenceLowGrayLevelEmphasis	0.031926872
wavelet-LHL	gldm	DependenceVariance	0.169466764
wavelet-LHL	gldm	LargeDependenceHighGrayLevelEmphasis	1420
wavelet-LHL	gldm	SmallDependenceLowGrayLevelEmphasis	0.030255525
wavelet-LHL	gldm	LowGrayLevelEmphasis	0.030589794
wavelet-LHL	glcm	JointAverage	27.0058669

wavelet-LHL	glcm	SumAverage	54.01173381
wavelet-LHL	glcm	JointEntropy	4.926183668
wavelet-LHL	glcm	ClusterShade	-266.535354
wavelet-LHL	glcm	MaximumProbability	0.04389383
wavelet-LHL	glcm	Idmn	0.887517802
wavelet-LHL	glcm	JointEnergy	0.035913421
wavelet-LHL	glcm	Contrast	405.8635922
wavelet-LHL	glcm	DifferenceEntropy	3.527477598
wavelet-LHL	glcm	InverseVariance	0.067449216
wavelet-LHL	glcm	DifferenceVariance	111.804176
wavelet-LHL	glcm	Idn	0.783476702
wavelet-LHL	glcm	Idm	0.05572148
wavelet-LHL	glcm	Correlation	-0.002059387
wavelet-LHL	glcm	Autocorrelation	726.311275
wavelet-LHL	glcm	SumEntropy	3.73462449
wavelet-LHL	glcm	SumSquares	199.7382931
wavelet-LHL	glcm	ClusterProminence	368231.3467
wavelet-LHL	glcm	Imc2	0.999657519
wavelet-LHL	glcm	Imc1	-0.859851851
wavelet-LHL	glcm	DifferenceAverage	16.05645112
wavelet-LHL	glcm	Id	0.122176017
wavelet-LHL	glcm	ClusterTendency	393.0895804
wavelet-LHL	firstorder	InterquartileRange	456.6132621
wavelet-LHL	firstorder	Skewness	-0.007663335
wavelet-LHL	firstorder	Uniformity	0.048940833
wavelet-LHL	firstorder	Median	-58.84267853
wavelet-LHL	firstorder	Energy	3686708.575
wavelet-LHL	firstorder	RobustMeanAbsoluteDeviation	188.1455558
wavelet-LHL	firstorder	MeanAbsoluteDeviation	261.2195966
wavelet-LHL	firstorder	TotalEnergy	99541131.52
wavelet-LHL	firstorder	Maximum	582.1536367
wavelet-LHL	firstorder	RootMeanSquared	315.6592661
wavelet-LHL	firstorder	90Percentile	356.0626447
wavelet-LHL	firstorder	Minimum	-672.8750838
wavelet-LHL	firstorder	Entropy	4.540402352
wavelet-LHL	firstorder	Range	1255.028721
wavelet-LHL	firstorder	Variance	98655.68596
wavelet-LHL	firstorder	10Percentile	-476.0900507
wavelet-LHL	firstorder	Kurtosis	2.244817645
wavelet-LHL	firstorder	Mean	-31.38608498
wavelet-LHL	glrlm	ShortRunLowGrayLevelEmphasis	0.030827003
wavelet-LHL	glrlm	GrayLevelVariance	158.3701248
wavelet-LHL	glrlm	LowGrayLevelRunEmphasis	0.030840594
wavelet-LHL	glrlm	GrayLevelNonUniformityNormalized	0.048695618
wavelet-LHL	glrlm	RunVariance	0.008288854
wavelet-LHL	glrlm	GrayLevelNonUniformity	1.787169587
wavelet-LHL	glrlm	LongRunEmphasis	1.026373626
wavelet-LHL	glrlm	ShortRunHighGrayLevelEmphasis	844.7904663
wavelet-LHL	glrlm	RunLengthNonUniformity	36.11208791
wavelet-LHL	glrlm	ShortRunEmphasis	0.993406593
wavelet-LHL	glrlm	LongRunHighGrayLevelEmphasis	873.7190377
wavelet-LHL	glrlm	RunPercentage	0.991683992
wavelet-LHL	glrlm	LongRunLowGrayLevelEmphasis	0.030894959
wavelet-LHL	glrlm	RunEntropy	4.554615009
wavelet-LHL	glrlm	HighGrayLevelRunEmphasis	850.5761806

wavelet-LHL	glrlm	RunLengthNonUniformityNormalized	0.983422292
wavelet-LHL	glszm	GrayLevelVariance	165.2580349
wavelet-LHL	glszm	ZoneVariance	0.106519743
wavelet-LHL	glszm	GrayLevelNonUniformityNormalized	0.044995409
wavelet-LHL	glszm	SizeZoneNonUniformityNormalized	0.786960514
wavelet-LHL	glszm	SizeZoneNonUniformity	25.96969697
wavelet-LHL	glszm	GrayLevelNonUniformity	1.484848485
wavelet-LHL	glszm	LargeAreaEmphasis	1.363636364
wavelet-LHL	glszm	SmallAreaHighGrayLevelEmphasis	767.8030303
wavelet-LHL	glszm	ZonePercentage	0.891891892
wavelet-LHL	glszm	LargeAreaLowGrayLevelEmphasis	0.034797364
wavelet-LHL	glszm	LargeAreaHighGrayLevelEmphasis	1166.666667
wavelet-LHL	glszm	HighGrayLevelZoneEmphasis	847.5757576
wavelet-LHL	glszm	SmallAreaEmphasis	0.909090909
wavelet-LHL	glszm	LowGrayLevelZoneEmphasis	0.03404779
wavelet-LHL	glszm	ZoneEntropy	4.741363816
wavelet-LHL	glszm	SmallAreaLowGrayLevelEmphasis	0.033860396
wavelet-LHL	ngtdm	Coarseness	0.054150525
wavelet-LHL	ngtdm	Complexity	4992.060045
wavelet-LHL	ngtdm	Strength	45.42697947
wavelet-LHL	ngtdm	Contrast	5.185795059
wavelet-LHL	ngtdm	Busyness	0.032780682
wavelet-LHH	gldm	GrayLevelVariance	26.17092768
wavelet-LHH	gldm	HighGrayLevelEmphasis	123.4864865
wavelet-LHH	gldm	DependenceEntropy	4.391489514
wavelet-LHH	gldm	DependenceNonUniformity	20.83783784
wavelet-LHH	gldm	GrayLevelNonUniformity	3.054054054
wavelet-LHH	gldm	SmallDependenceEmphasis	0.777214715
wavelet-LHH	gldm	SmallDependenceHighGrayLevelEmphasis	111.4934309
wavelet-LHH	gldm	DependenceNonUniformityNormalized	0.563184806
wavelet-LHH	gldm	LargeDependenceEmphasis	2.675675676
wavelet-LHH	gldm	LargeDependenceLowGrayLevelEmphasis	0.126962237
wavelet-LHH	gldm	DependenceVariance	0.623813002
wavelet-LHH	gldm	LargeDependenceHighGrayLevelEmphasis	200.0810811
wavelet-LHH	gldm	SmallDependenceLowGrayLevelEmphasis	0.069252965
wavelet-LHH	gldm	LowGrayLevelEmphasis	0.075239264
wavelet-LHH	glcm	JointAverage	9.652285459
wavelet-LHH	glcm	SumAverage	19.30457092
wavelet-LHH	glcm	JointEntropy	4.811487561
wavelet-LHH	glcm	ClusterShade	61.22346381
wavelet-LHH	glcm	MaximumProbability	0.062114477
wavelet-LHH	glcm	Idmn	0.900332702
wavelet-LHH	glcm	JointEnergy	0.039568667
wavelet-LHH	glcm	Contrast	56.16578397
wavelet-LHH	glcm	DifferenceEntropy	2.918343777
wavelet-LHH	glcm	InverseVariance	0.138299983
wavelet-LHH	glcm	DifferenceVariance	15.68142122
wavelet-LHH	glcm	Idn	0.78897649
wavelet-LHH	glcm	Idm	0.127030928
wavelet-LHH	glcm	Correlation	-0.084423944
wavelet-LHH	glcm	Autocorrelation	91.32911107
wavelet-LHH	glcm	SumEntropy	3.45470766
wavelet-LHH	glcm	SumSquares	25.84413337
wavelet-LHH	glcm	ClusterProminence	6051.28676
wavelet-LHH	glcm	Imc2	0.995538402

wavelet-LHH	glcm	Imc1	-0.672618731
wavelet-LHH	glcm	DifferenceAverage	6.2182653
wavelet-LHH	glcm	Id	0.221969568
wavelet-LHH	glcm	ClusterTendency	47.21074952
wavelet-LHH	firstorder	InterquartileRange	191.2852128
wavelet-LHH	firstorder	Skewness	0.343467768
wavelet-LHH	firstorder	Uniformity	0.082542001
wavelet-LHH	firstorder	Median	2.311493306
wavelet-LHH	firstorder	Energy	611642.5632
wavelet-LHH	firstorder	RobustMeanAbsoluteDeviation	74.22886175
wavelet-LHH	firstorder	MeanAbsoluteDeviation	105.0531165
wavelet-LHH	firstorder	TotalEnergy	16514349.21
wavelet-LHH	firstorder	Maximum	278.4845834
wavelet-LHH	firstorder	RootMeanSquared	128.5724702
wavelet-LHH	firstorder	90Percentile	178.7385026
wavelet-LHH	firstorder	Minimum	-219.7788565
wavelet-LHH	firstorder	Entropy	3.830546609
wavelet-LHH	firstorder	Range	498.2634399
wavelet-LHH	firstorder	Variance	16467.78269
wavelet-LHH	firstorder	10Percentile	-122.0917318
wavelet-LHH	firstorder	Kurtosis	2.402605079
wavelet-LHH	firstorder	Mean	7.943386985
wavelet-LHH	glrlm	ShortRunLowGrayLevelEmphasis	0.075648174
wavelet-LHH	glrlm	GrayLevelVariance	26.35245163
wavelet-LHH	glrlm	LowGrayLevelRunEmphasis	0.076028067
wavelet-LHH	glrlm	GrayLevelNonUniformityNormalized	0.080861392
wavelet-LHH	glrlm	RunVariance	0.016608814
wavelet-LHH	glrlm	GrayLevelNonUniformity	2.943140943
wavelet-LHH	glrlm	LongRunEmphasis	1.051648352
wavelet-LHH	glrlm	ShortRunHighGrayLevelEmphasis	124.1444956
wavelet-LHH	glrlm	RunLengthNonUniformity	35.1970696
wavelet-LHH	glrlm	ShortRunEmphasis	0.987087912
wavelet-LHH	glrlm	LongRunHighGrayLevelEmphasis	127.2901
wavelet-LHH	glrlm	RunPercentage	0.983367983
wavelet-LHH	glrlm	LongRunLowGrayLevelEmphasis	0.077547641
wavelet-LHH	glrlm	RunEntropy	3.901915328
wavelet-LHH	glrlm	HighGrayLevelRunEmphasis	124.7736165
wavelet-LHH	glrlm	RunLengthNonUniformityNormalized	0.966782371
wavelet-LHH	glszm	GrayLevelVariance	28.51222222
wavelet-LHH	glszm	ZoneVariance	0.645555556
wavelet-LHH	glszm	GrayLevelNonUniformityNormalized	0.068888889
wavelet-LHH	glszm	SizeZoneNonUniformityNormalized	0.813333333
wavelet-LHH	glszm	SizeZoneNonUniformity	24.4
wavelet-LHH	glszm	GrayLevelNonUniformity	2.066666667
wavelet-LHH	glszm	LargeAreaEmphasis	2.166666667
wavelet-LHH	glszm	SmallAreaHighGrayLevelEmphasis	134.8037037
wavelet-LHH	glszm	ZonePercentage	0.810810811
wavelet-LHH	glszm	LargeAreaLowGrayLevelEmphasis	0.122503426
wavelet-LHH	glszm	LargeAreaHighGrayLevelEmphasis	193.2333333
wavelet-LHH	glszm	HighGrayLevelZoneEmphasis	140.1666667
wavelet-LHH	glszm	SmallAreaEmphasis	0.91337037
wavelet-LHH	glszm	LowGrayLevelZoneEmphasis	0.086274259
wavelet-LHH	glszm	ZoneEntropy	4.189898095
wavelet-LHH	glszm	SmallAreaLowGrayLevelEmphasis	0.084307338
wavelet-LHH	ngtdm	Coarseness	0.076202036

wavelet-LHH	ngtdm	Complexity	628.8977318
wavelet-LHH	ngtdm	Strength	12.69542212
wavelet-LHH	ngtdm	Contrast	0.888513094
wavelet-LHH	ngtdm	Busyness	0.132664298
wavelet-LLH	gldm	GrayLevelVariance	416.8224982
wavelet-LLH	gldm	HighGrayLevelEmphasis	3263.189189
wavelet-LLH	gldm	DependenceEntropy	4.722966879
wavelet-LLH	gldm	DependenceNonUniformity	19.59459459
wavelet-LLH	gldm	GrayLevelNonUniformity	1.594594595
wavelet-LLH	gldm	SmallDependenceEmphasis	0.716216216
wavelet-LLH	gldm	SmallDependenceHighGrayLevelEmphasis	2099.027027
wavelet-LLH	gldm	DependenceNonUniformityNormalized	0.529583638
wavelet-LLH	gldm	LargeDependenceEmphasis	2.135135135
wavelet-LLH	gldm	LargeDependenceLowGrayLevelEmphasis	0.028274106
wavelet-LLH	gldm	DependenceVariance	0.235208181
wavelet-LLH	gldm	LargeDependenceHighGrayLevelEmphasis	7919.837838
wavelet-LLH	gldm	SmallDependenceLowGrayLevelEmphasis	0.02789032
wavelet-LLH	gldm	LowGrayLevelEmphasis	0.027967077
wavelet-LLH	glcm	JointAverage	57.54584173
wavelet-LLH	glcm	SumAverage	115.0916835
wavelet-LLH	glcm	JointEntropy	4.920376729
wavelet-LLH	glcm	ClusterShade	-7425.802903
wavelet-LLH	glcm	MaximumProbability	0.049612238
wavelet-LLH	glcm	Idmn	0.933129607
wavelet-LLH	glcm	JointEnergy	0.036149783
wavelet-LLH	glcm	Contrast	624.5936156
wavelet-LLH	glcm	DifferenceEntropy	3.44452824
wavelet-LLH	glcm	InverseVariance	0.088122045
wavelet-LLH	glcm	DifferenceVariance	267.1722227
wavelet-LLH	glcm	Idn	0.851937396
wavelet-LLH	glcm	Idm	0.087068907
wavelet-LLH	glcm	Correlation	-0.066982178
wavelet-LLH	glcm	Autocorrelation	3288.757162
wavelet-LLH	glcm	SumEntropy	3.692628872
wavelet-LLH	glcm	SumSquares	284.961836
wavelet-LLH	glcm	ClusterProminence	1366121.099
wavelet-LLH	glcm	Imc2	0.999459127
wavelet-LLH	glcm	Imc1	-0.826454265
wavelet-LLH	glcm	DifferenceAverage	16.80259834
wavelet-LLH	glcm	Id	0.15986084
wavelet-LLH	glcm	ClusterTendency	515.2537283
wavelet-LLH	firstorder	InterquartileRange	449.1548787
wavelet-LLH	firstorder	Skewness	-0.831275922
wavelet-LLH	firstorder	Uniformity	0.043097151
wavelet-LLH	firstorder	Median	-33.17241607
wavelet-LLH	firstorder	Energy	10050192.04
wavelet-LLH	firstorder	RobustMeanAbsoluteDeviation	236.9047852
wavelet-LLH	firstorder	MeanAbsoluteDeviation	383.443621
wavelet-LLH	firstorder	TotalEnergy	271355185.1
wavelet-LLH	firstorder	Maximum	583.3740835
wavelet-LLH	firstorder	RootMeanSquared	521.1782918
wavelet-LLH	firstorder	90Percentile	510.3032135
wavelet-LLH	firstorder	Minimum	-1421.092393
wavelet-LLH	firstorder	Entropy	4.64851046
wavelet-LLH	firstorder	Range	2004.466477

wavelet-LLH	firstorder	Variance	261071.1565
wavelet-LLH	firstorder	10Percentile	-877.9817992
wavelet-LLH	firstorder	Kurtosis	3.195636482
wavelet-LLH	firstorder	Mean	-102.7407189
wavelet-LLH	glrlm	ShortRunLowGrayLevelEmphasis	0.028399701
wavelet-LLH	glrlm	GrayLevelVariance	419.7374531
wavelet-LLH	glrlm	LowGrayLevelRunEmphasis	0.02840288
wavelet-LLH	glrlm	GrayLevelNonUniformityNormalized	0.043009203
wavelet-LLH	glrlm	RunVariance	0.01442602
wavelet-LLH	glrlm	GrayLevelNonUniformity	1.568208068
wavelet-LLH	glrlm	LongRunEmphasis	1.047202797
wavelet-LLH	glrlm	ShortRunHighGrayLevelEmphasis	3200.832706
wavelet-LLH	glrlm	RunLengthNonUniformity	35.47202797
wavelet-LLH	glrlm	ShortRunEmphasis	0.988199301
wavelet-LLH	glrlm	LongRunHighGrayLevelEmphasis	3444.476354
wavelet-LLH	glrlm	RunPercentage	0.985446985
wavelet-LLH	glrlm	LongRunLowGrayLevelEmphasis	0.028415596
wavelet-LLH	glrlm	RunEntropy	4.655220683
wavelet-LLH	glrlm	HighGrayLevelRunEmphasis	3249.561436
wavelet-LLH	glrlm	RunLengthNonUniformityNormalized	0.97114796
wavelet-LLH	glszm	GrayLevelVariance	459.5955556
wavelet-LLH	glszm	ZoneVariance	0.178888889
wavelet-LLH	glszm	GrayLevelNonUniformityNormalized	0.04
wavelet-LLH	glszm	SizeZoneNonUniformityNormalized	0.642222222
wavelet-LLH	glszm	SizeZoneNonUniformity	19.26666667
wavelet-LLH	glszm	GrayLevelNonUniformity	1.2
wavelet-LLH	glszm	LargeAreaEmphasis	1.7
wavelet-LLH	glszm	SmallAreaHighGrayLevelEmphasis	2349.5
wavelet-LLH	glszm	ZonePercentage	0.810810811
wavelet-LLH	glszm	LargeAreaLowGrayLevelEmphasis	0.034618952
wavelet-LLH	glszm	LargeAreaHighGrayLevelEmphasis	5939
wavelet-LLH	glszm	HighGrayLevelZoneEmphasis	3067.4
wavelet-LLH	glszm	SmallAreaEmphasis	0.825
wavelet-LLH	glszm	LowGrayLevelZoneEmphasis	0.034429617
wavelet-LLH	glszm	ZoneEntropy	4.773557262
wavelet-LLH	glszm	SmallAreaLowGrayLevelEmphasis	0.034382283
wavelet-LLH	ngtdm	Coarseness	0.045801859
wavelet-LLH	ngtdm	Complexity	13080.5226
wavelet-LLH	ngtdm	Strength	84.24709468
wavelet-LLH	ngtdm	Contrast	18.81624933
wavelet-LLH	ngtdm	Busyness	0.0200553
wavelet-LLL	gldm	GrayLevelVariance	214.8327246
wavelet-LLL	gldm	HighGrayLevelEmphasis	1160.810811
wavelet-LLL	gldm	DependenceEntropy	4.831074987
wavelet-LLL	gldm	DependenceNonUniformity	26.94594595
wavelet-LLL	gldm	GrayLevelNonUniformity	1.378378378
wavelet-LLL	gldm	SmallDependenceEmphasis	0.878378378
wavelet-LLL	gldm	SmallDependenceHighGrayLevelEmphasis	1015.067568
wavelet-LLL	gldm	DependenceNonUniformityNormalized	0.728268809
wavelet-LLL	gldm	LargeDependenceEmphasis	1.486486486
wavelet-LLL	gldm	LargeDependenceLowGrayLevelEmphasis	0.030575592
wavelet-LLL	gldm	DependenceVariance	0.135865595
wavelet-LLL	gldm	LargeDependenceHighGrayLevelEmphasis	1743.783784
wavelet-LLL	gldm	SmallDependenceLowGrayLevelEmphasis	0.029431794
wavelet-LLL	gldm	LowGrayLevelEmphasis	0.029660553

wavelet-LLL	glcm	JointAverage	33.4814355
wavelet-LLL	glcm	SumAverage	66.962871
wavelet-LLL	glcm	JointEntropy	4.958694101
wavelet-LLL	glcm	ClusterShade	-1799.982692
wavelet-LLL	glcm	MaximumProbability	0.041009394
wavelet-LLL	glcm	Idmn	0.919903243
wavelet-LLL	glcm	JointEnergy	0.03513821
wavelet-LLL	glcm	Contrast	433.5432457
wavelet-LLL	glcm	DifferenceEntropy	3.682110635
wavelet-LLL	glcm	InverseVariance	0.050808746
wavelet-LLL	glcm	DifferenceVariance	145.9139159
wavelet-LLL	glcm	Idn	0.813209476
wavelet-LLL	glcm	Idm	0.046685283
wavelet-LLL	glcm	Correlation	0.040157412
wavelet-LLL	glcm	Autocorrelation	1133.715423
wavelet-LLL	glcm	SumEntropy	3.908436056
wavelet-LLL	glcm	SumSquares	226.2313673
wavelet-LLL	glcm	ClusterProminence	590639.0142
wavelet-LLL	glcm	Imc2	0.999799705
wavelet-LLL	glcm	Imc1	-0.890945113
wavelet-LLL	glcm	DifferenceAverage	16.82415257
wavelet-LLL	glcm	Id	0.114005364
wavelet-LLL	glcm	ClusterTendency	471.3822233
wavelet-LLL	firstorder	InterquartileRange	455.1350158
wavelet-LLL	firstorder	Skewness	0.109468148
wavelet-LLL	firstorder	Uniformity	0.03725347
wavelet-LLL	firstorder	Median	-1137.971137
wavelet-LLL	firstorder	Energy	55485268.82
wavelet-LLL	firstorder	RobustMeanAbsoluteDeviation	212.7567733
wavelet-LLL	firstorder	MeanAbsoluteDeviation	299.8523635
wavelet-LLL	firstorder	TotalEnergy	1498102258
wavelet-LLL	firstorder	Maximum	-290.7839299
wavelet-LLL	firstorder	RootMeanSquared	1224.582321
wavelet-LLL	firstorder	90Percentile	-783.9819129
wavelet-LLL	firstorder	Minimum	-1914.887492
wavelet-LLL	firstorder	Entropy	4.831074987
wavelet-LLL	firstorder	Range	1624.103563
wavelet-LLL	firstorder	Variance	132950.2751
wavelet-LLL	firstorder	10Percentile	-1639.829141
wavelet-LLL	firstorder	Kurtosis	2.673648852
wavelet-LLL	firstorder	Mean	-1169.038744
wavelet-LLL	glrlm	ShortRunLowGrayLevelEmphasis	0.029829585
wavelet-LLL	glrlm	GrayLevelVariance	215.1862008
wavelet-LLL	glrlm	LowGrayLevelRunEmphasis	0.029838628
wavelet-LLL	glrlm	GrayLevelNonUniformityNormalized	0.037203524
wavelet-LLL	glrlm	RunVariance	0.006232194
wavelet-LLL	glrlm	GrayLevelNonUniformity	1.367983368
wavelet-LLL	glrlm	LongRunEmphasis	1.019230769
wavelet-LLL	glrlm	ShortRunHighGrayLevelEmphasis	1154.809064
wavelet-LLL	glrlm	RunLengthNonUniformity	36.32051282
wavelet-LLL	glrlm	ShortRunEmphasis	0.995192308
wavelet-LLL	glrlm	LongRunHighGrayLevelEmphasis	1183.615154
wavelet-LLL	glrlm	RunPercentage	0.993762994
wavelet-LLL	glrlm	LongRunLowGrayLevelEmphasis	0.029874799
wavelet-LLL	glrlm	RunEntropy	4.832348067

wavelet-LLL	glrlm	HighGrayLevelRunEmphasis	1160.570282
wavelet-LLL	glrlm	RunLengthNonUniformityNormalized	0.987535613
wavelet-LLL	glszm	GrayLevelVariance	220.0596886
wavelet-LLL	glszm	ZoneVariance	0.080449827
wavelet-LLL	glszm	GrayLevelNonUniformityNormalized	0.03633218
wavelet-LLL	glszm	SizeZoneNonUniformityNormalized	0.839100346
wavelet-LLL	glszm	SizeZoneNonUniformity	28.52941176
wavelet-LLL	glszm	GrayLevelNonUniformity	1.235294118
wavelet-LLL	glszm	LargeAreaEmphasis	1.264705882
wavelet-LLL	glszm	SmallAreaHighGrayLevelEmphasis	1078.198529
wavelet-LLL	glszm	ZonePercentage	0.918918919
wavelet-LLL	glszm	LargeAreaLowGrayLevelEmphasis	0.032609587
wavelet-LLL	glszm	LargeAreaHighGrayLevelEmphasis	1474.705882
wavelet-LLL	glszm	HighGrayLevelZoneEmphasis	1157.5
wavelet-LLL	glszm	SmallAreaEmphasis	0.933823529
wavelet-LLL	glszm	LowGrayLevelZoneEmphasis	0.032111698
wavelet-LLL	glszm	ZoneEntropy	4.852168724
wavelet-LLL	glszm	SmallAreaLowGrayLevelEmphasis	0.031987226
wavelet-LLL	ngtdm	Coarseness	0.067318295
wavelet-LLL	ngtdm	Complexity	7029.703067
wavelet-LLL	ngtdm	Strength	63.00348155
wavelet-LLL	ngtdm	Contrast	5.777574301
wavelet-LLL	ngtdm	Busyness	0.02378517
wavelet-HHL	gldm	GrayLevelVariance	16.35792549
wavelet-HHL	gldm	HighGrayLevelEmphasis	106.3513514
wavelet-HHL	gldm	DependenceEntropy	4.391489514
wavelet-HHL	gldm	DependenceNonUniformity	17.10810811
wavelet-HHL	gldm	GrayLevelNonUniformity	3.378378378
wavelet-HHL	gldm	SmallDependenceEmphasis	0.705660661
wavelet-HHL	gldm	SmallDependenceHighGrayLevelEmphasis	88.53238739
wavelet-HHL	gldm	DependenceNonUniformityNormalized	0.4623813
wavelet-HHL	gldm	LargeDependenceEmphasis	3.810810811
wavelet-HHL	gldm	LargeDependenceLowGrayLevelEmphasis	0.099347643
wavelet-HHL	gldm	DependenceVariance	1.092768444
wavelet-HHL	gldm	LargeDependenceHighGrayLevelEmphasis	289.7567568
wavelet-HHL	gldm	SmallDependenceLowGrayLevelEmphasis	0.038782399
wavelet-HHL	gldm	LowGrayLevelEmphasis	0.044869125
wavelet-HHL	glcm	JointAverage	9.482204865
wavelet-HHL	glcm	SumAverage	18.96440973
wavelet-HHL	glcm	JointEntropy	4.75626229
wavelet-HHL	glcm	ClusterShade	11.8027627
wavelet-HHL	glcm	MaximumProbability	0.063322206
wavelet-HHL	glcm	Idmn	0.912395887
wavelet-HHL	glcm	JointEnergy	0.0412171
wavelet-HHL	glcm	Contrast	40.10345487
wavelet-HHL	glcm	DifferenceEntropy	2.878260221
wavelet-HHL	glcm	InverseVariance	0.209836795
wavelet-HHL	glcm	DifferenceVariance	12.32690589
wavelet-HHL	glcm	Idn	0.806021423
wavelet-HHL	glcm	Idm	0.169700392
wavelet-HHL	glcm	Correlation	-0.043295573
wavelet-HHL	glcm	Autocorrelation	89.33163303
wavelet-HHL	glcm	SumEntropy	3.260513133
wavelet-HHL	glcm	SumSquares	19.01643296
wavelet-HHL	glcm	ClusterProminence	3569.38465

wavelet-HHL	glcm	Imc2	0.996055081
wavelet-HHL	glcm	Imc1	-0.698328293
wavelet-HHL	glcm	DifferenceAverage	5.10629499
wavelet-HHL	glcm	Id	0.261617749
wavelet-HHL	glcm	ClusterTendency	35.96227699
wavelet-HHL	firstorder	InterquartileRange	143.152841
wavelet-HHL	firstorder	Skewness	0.263408276
wavelet-HHL	firstorder	Uniformity	0.091307524
wavelet-HHL	firstorder	Median	-43.09377854
wavelet-HHL	firstorder	Energy	410053.4296
wavelet-HHL	firstorder	RobustMeanAbsoluteDeviation	55.80358776
wavelet-HHL	firstorder	MeanAbsoluteDeviation	81.9404176
wavelet-HHL	firstorder	TotalEnergy	11071442.6
wavelet-HHL	firstorder	Maximum	201.8758687
wavelet-HHL	firstorder	RootMeanSquared	105.2735728
wavelet-HHL	firstorder	90Percentile	119.3111048
wavelet-HHL	firstorder	Minimum	-249.8554256
wavelet-HHL	firstorder	Entropy	3.752260205
wavelet-HHL	firstorder	Range	451.7312943
wavelet-HHL	firstorder	Variance	10322.91492
wavelet-HHL	firstorder	10Percentile	-146.2344519
wavelet-HHL	firstorder	Kurtosis	2.60462211
wavelet-HHL	firstorder	Mean	-27.56102689
wavelet-HHL	glrlm	ShortRunLowGrayLevelEmphasis	0.045119969
wavelet-HHL	glrlm	GrayLevelVariance	16.62757452
wavelet-HHL	glrlm	LowGrayLevelRunEmphasis	0.045517479
wavelet-HHL	glrlm	GrayLevelNonUniformityNormalized	0.088901226
wavelet-HHL	glrlm	RunVariance	0.024864292
wavelet-HHL	glrlm	GrayLevelNonUniformity	3.20923768
wavelet-HHL	glrlm	LongRunEmphasis	1.078786899
wavelet-HHL	glrlm	ShortRunHighGrayLevelEmphasis	106.252257
wavelet-HHL	glrlm	RunLengthNonUniformity	34.32802557
wavelet-HHL	glrlm	ShortRunEmphasis	0.980303275
wavelet-HHL	glrlm	LongRunHighGrayLevelEmphasis	112.4496686
wavelet-HHL	glrlm	RunPercentage	0.975051975
wavelet-HHL	glrlm	LongRunLowGrayLevelEmphasis	0.047107517
wavelet-HHL	glrlm	RunEntropy	3.850477965
wavelet-HHL	glrlm	HighGrayLevelRunEmphasis	107.4917393
wavelet-HHL	glrlm	RunLengthNonUniformityNormalized	0.950271416
wavelet-HHL	glszm	GrayLevelVariance	19.53443878
wavelet-HHL	glszm	ZoneVariance	1.003826531
wavelet-HHL	glszm	GrayLevelNonUniformityNormalized	0.073979592
wavelet-HHL	glszm	SizeZoneNonUniformityNormalized	0.742346939
wavelet-HHL	glszm	SizeZoneNonUniformity	20.78571429
wavelet-HHL	glszm	GrayLevelNonUniformity	2.071428571
wavelet-HHL	glszm	LargeAreaEmphasis	2.75
wavelet-HHL	glszm	SmallAreaHighGrayLevelEmphasis	113.6170635
wavelet-HHL	glszm	ZonePercentage	0.756756757
wavelet-HHL	glszm	LargeAreaLowGrayLevelEmphasis	0.083664083
wavelet-HHL	glszm	LargeAreaHighGrayLevelEmphasis	240.75
wavelet-HHL	glszm	HighGrayLevelZoneEmphasis	120.25
wavelet-HHL	glszm	SmallAreaEmphasis	0.879960317
wavelet-HHL	glszm	LowGrayLevelZoneEmphasis	0.052942717
wavelet-HHL	glszm	ZoneEntropy	4.182005815
wavelet-HHL	glszm	SmallAreaLowGrayLevelEmphasis	0.050279032

wavelet-HHL	ngtdm	Coarseness	0.131474427
wavelet-HHL	ngtdm	Complexity	360.8577631
wavelet-HHL	ngtdm	Strength	11.66317279
wavelet-HHL	ngtdm	Contrast	0.403905503
wavelet-HHL	ngtdm	Busyness	0.058924529
wavelet-HLH	gldm	GrayLevelVariance	143.7048941
wavelet-HLH	gldm	HighGrayLevelEmphasis	521.3243243
wavelet-HLH	gldm	DependenceEntropy	4.411891879
wavelet-HLH	gldm	DependenceNonUniformity	15.91891892
wavelet-HLH	gldm	GrayLevelNonUniformity	2.513513514
wavelet-HLH	gldm	SmallDependenceEmphasis	0.603603604
wavelet-HLH	gldm	SmallDependenceHighGrayLevelEmphasis	411.5900901
wavelet-HLH	gldm	DependenceNonUniformityNormalized	0.430241052
wavelet-HLH	gldm	LargeDependenceEmphasis	2.945945946
wavelet-HLH	gldm	LargeDependenceLowGrayLevelEmphasis	0.226148479
wavelet-HLH	gldm	DependenceVariance	0.403214025
wavelet-HLH	gldm	LargeDependenceHighGrayLevelEmphasis	1090.351351
wavelet-HLH	gldm	SmallDependenceLowGrayLevelEmphasis	0.015787809
wavelet-HLH	gldm	LowGrayLevelEmphasis	0.057660297
wavelet-HLH	glcm	JointAverage	18.09663596
wavelet-HLH	glcm	SumAverage	36.19327192
wavelet-HLH	glcm	JointEntropy	4.844779633
wavelet-HLH	glcm	ClusterShade	4268.834155
wavelet-HLH	glcm	MaximumProbability	0.053781143
wavelet-HLH	glcm	Idmn	0.969730442
wavelet-HLH	glcm	JointEnergy	0.03810165
wavelet-HLH	glcm	Contrast	170.3189069
wavelet-HLH	glcm	DifferenceEntropy	3.124554701
wavelet-HLH	glcm	InverseVariance	0.179070133
wavelet-HLH	glcm	DifferenceVariance	100.5960141
wavelet-HLH	glcm	Idn	0.903274045
wavelet-HLH	glcm	Idm	0.153433717
wavelet-HLH	glcm	Correlation	-0.109759486
wavelet-HLH	glcm	Autocorrelation	326.2052615
wavelet-HLH	glcm	SumEntropy	3.408243367
wavelet-HLH	glcm	SumSquares	83.06817583
wavelet-HLH	glcm	ClusterProminence	326351.4249
wavelet-HLH	glcm	Imc2	0.998009589
wavelet-HLH	glcm	Imc1	-0.739630556
wavelet-HLH	glcm	DifferenceAverage	7.921812969
wavelet-HLH	glcm	Id	0.240926236
wavelet-HLH	glcm	ClusterTendency	161.9537965
wavelet-HLH	firstorder	InterquartileRange	167.6458962
wavelet-HLH	firstorder	Skewness	2.139273875
wavelet-HLH	firstorder	Uniformity	0.067932798
wavelet-HLH	firstorder	Median	17.42683478
wavelet-HLH	firstorder	Energy	3394052.156
wavelet-HLH	firstorder	RobustMeanAbsoluteDeviation	74.70273447
wavelet-HLH	firstorder	MeanAbsoluteDeviation	175.2986804
wavelet-HLH	firstorder	TotalEnergy	91639408.2
wavelet-HLH	firstorder	Maximum	1164.034999
wavelet-HLH	firstorder	RootMeanSquared	302.8714898
wavelet-HLH	firstorder	90Percentile	172.232053
wavelet-HLH	firstorder	Minimum	-416.6172575
wavelet-HLH	firstorder	Entropy	4.098117812

wavelet-HLH	firstorder	Range	1580.652256
wavelet-HLH	firstorder	Variance	89470.44266
wavelet-HLH	firstorder	10Percentile	-150.749319
wavelet-HLH	firstorder	Kurtosis	8.297571308
wavelet-HLH	firstorder	Mean	47.54678415
wavelet-HLH	glrlm	ShortRunLowGrayLevelEmphasis	0.054959657
wavelet-HLH	glrlm	GrayLevelVariance	146.112357
wavelet-HLH	glrlm	LowGrayLevelRunEmphasis	0.056716538
wavelet-HLH	glrlm	GrayLevelNonUniformityNormalized	0.067360871
wavelet-HLH	glrlm	RunVariance	0.022763886
wavelet-HLH	glrlm	GrayLevelNonUniformity	2.435849877
wavelet-HLH	glrlm	LongRunEmphasis	1.072775264
wavelet-HLH	glrlm	ShortRunHighGrayLevelEmphasis	521.8835997
wavelet-HLH	glrlm	RunLengthNonUniformity	34.56435395
wavelet-HLH	glrlm	ShortRunEmphasis	0.981806184
wavelet-HLH	glrlm	LongRunHighGrayLevelEmphasis	547.7848063
wavelet-HLH	glrlm	RunPercentage	0.977130977
wavelet-HLH	glrlm	LongRunLowGrayLevelEmphasis	0.06374406
wavelet-HLH	glrlm	RunEntropy	4.14319822
wavelet-HLH	glrlm	HighGrayLevelRunEmphasis	527.063841
wavelet-HLH	glrlm	RunLengthNonUniformityNormalized	0.954472228
wavelet-HLH	glszm	GrayLevelVariance	177.9286694
wavelet-HLH	glszm	ZoneVariance	0.307270233
wavelet-HLH	glszm	GrayLevelNonUniformityNormalized	0.061728395
wavelet-HLH	glszm	SizeZoneNonUniformityNormalized	0.533607682
wavelet-HLH	glszm	SizeZoneNonUniformity	14.40740741
wavelet-HLH	glszm	GrayLevelNonUniformity	1.666666667
wavelet-HLH	glszm	LargeAreaEmphasis	2.185185185
wavelet-HLH	glszm	SmallAreaHighGrayLevelEmphasis	541.9393004
wavelet-HLH	glszm	ZonePercentage	0.72972973
wavelet-HLH	glszm	LargeAreaLowGrayLevelEmphasis	0.155774509
wavelet-HLH	glszm	LargeAreaHighGrayLevelEmphasis	947.5925926
wavelet-HLH	glszm	HighGrayLevelZoneEmphasis	611.1851852
wavelet-HLH	glszm	SmallAreaEmphasis	0.744855967
wavelet-HLH	glszm	LowGrayLevelZoneEmphasis	0.040739285
wavelet-HLH	glszm	ZoneEntropy	4.430632409
wavelet-HLH	glszm	SmallAreaLowGrayLevelEmphasis	0.012094475
wavelet-HLH	ngtdm	Coarseness	0.081177617
wavelet-HLH	ngtdm	Complexity	2909.9883
wavelet-HLH	ngtdm	Strength	57.58135841
wavelet-HLH	ngtdm	Contrast	5.404939762
wavelet-HLH	ngtdm	Busyness	0.045533533

Image type	Feature Class	Feature Name	7: RTSTRUCT: C99_DNU_segment_Tumor
general	info	BoundingBox	(0, 0, 1, 25, 24, 20)
general	info	EnabledImageTypes	{u'Original': {}, u'Wavelet': {}}
general	info	GeneralSettings	{'distances': [1], 'additionalInfo': True, 'force2D': False, 'interpolator': 'sitkBSpline', 'resampledPixelSpacing': [3.0], 'label': 1, 'normalizeScale': 1, 'normalize': False, 'force2Ddimension': 0, 'removeOutliers': None, 'minimumROISize': None, u'binWidth': 25.0, 'minimumROIDimensions': 1, u'correctMask': True, 'preCrop': False, u'symmetricalGLCM': True, 'resegmentRange': None, 'padDistance': 5}
general	info	ImageHash	4dc4e8a678c240e442e6879347266657b859bbb0
general	info	ImageSpacing	(0.9765624999999998, 0.9765624999999998, 1.999999999999999)
general	info	MaskHash	44577aaf0a2507e783147c287d2522b156056df6
general	info	NumpyVersion	1.13.1
general	info	PyWaveletVersion	0.5.2
general	info	SimpleITKVersion	1.1.0
general	info	Version	1.3.0.post102+g1b0eb74
general	info	VolumeNum	1
general	info	VoxelNum	4698
original	shape	Maximum3DDiameter	86.1684397
original	shape	Maximum2DDiameterSlice	76.4852927
original	shape	Sphericity	0.802412988
original	shape	MinorAxis	52.52326729
original	shape	Elongation	0.727645538
original	shape	SurfaceVolumeRatio	0.11994825
original	shape	Volume	126846
original	shape	MajorAxis	72.18249069
original	shape	SurfaceArea	15214.95572
original	shape	Flatness	0.657468495
original	shape	LeastAxis	47.4577135
original	shape	Maximum2DDiameterColumn	67.08203932
original	shape	Maximum2DDiameterRow	70.29224708
original	gldm	GrayLevelVariance	178.0301662
original	gldm	HighGrayLevelEmphasis	1324.727544
original	gldm	DependenceEntropy	7.036291477
original	gldm	DependenceNonUniformity	419.0791826
original	gldm	GrayLevelNonUniformity	731.2005109
original	gldm	SmallDependenceEmphasis	0.27680686
original	gldm	SmallDependenceHighGrayLevelEmphasis	222.2744472
original	gldm	DependenceNonUniformityNormalized	0.089203743
original	gldm	LargeDependenceEmphasis	113.49255
original	gldm	LargeDependenceLowGrayLevelEmphasis	0.222898849
original	gldm	DependenceVariance	48.45302866

original	gldm	LargeDependenceHighGrayLevelEmphasis	196679.6701
original	gldm	SmallDependenceLowGrayLevelEmphasis	0.004525609
original	gldm	LowGrayLevelEmphasis	0.0120954
original	glcm	JointAverage	35.60937297
original	glcm	SumAverage	71.21874594
original	glcm	JointEntropy	6.688864932
original	glcm	ClusterShade	-15739.61989
original	glcm	MaximumProbability	0.215482855
original	glcm	Idmn	0.98306387
original	glcm	JointEnergy	0.066313033
original	glcm	Contrast	113.2663353
original	glcm	DifferenceEntropy	3.505971116
original	glcm	InverseVariance	0.311062219
original	glcm	DifferenceVariance	81.78916823
original	glcm	Idn	0.942187681
original	glcm	Idm	0.470958637
original	glcm	Correlation	0.599502177
original	glcm	Autocorrelation	1354.333521
original	glcm	SumEntropy	4.707259872
original	glcm	SumSquares	142.831991
original	glcm	ClusterProminence	950198.8142
original	glcm	Imc2	0.83045137
original	glcm	Imc1	-0.166036434
original	glcm	DifferenceAverage	5.554139677
original	glcm	Id	0.506034699
original	glcm	ClusterTendency	458.0616287
original	firstorder	InterquartileRange	341.5
original	firstorder	Skewness	-1.280414712
original	firstorder	Uniformity	0.155640807
original	firstorder	Median	-4
original	firstorder	Energy	694883622
original	firstorder	RobustMeanAbsoluteDeviation	186.9796975
original	firstorder	MeanAbsoluteDeviation	273.5375631
original	firstorder	TotalEnergy	18761857794
original	firstorder	Maximum	878
original	firstorder	RootMeanSquared	384.5913673
original	firstorder	90Percentile	27
original	firstorder	Minimum	-1012
original	firstorder	Entropy	3.950904826
original	firstorder	Range	1890
original	firstorder	Variance	111196.7623
original	firstorder	10Percentile	-832
original	firstorder	Kurtosis	3.240799689
original	firstorder	Mean	-191.608344
original	glrlm	ShortRunLowGrayLevelEmphasis	0.014330086
original	glrlm	GrayLevelVariance	205.4624622
original	glrlm	LowGrayLevelRunEmphasis	0.015218192
original	glrlm	GrayLevelNonUniformityNormalized	0.084854362
original	glrlm	RunVariance	0.993845051
original	glrlm	GrayLevelNonUniformity	290.7521026
original	glrlm	LongRunEmphasis	2.885805093
original	glrlm	ShortRunHighGrayLevelEmphasis	917.5128033
original	glrlm	RunLengthNonUniformity	2271.320459

original	glrlm	ShortRunEmphasis	0.839253346
original	glrlm	LongRunHighGrayLevelEmphasis	4427.996261
original	glrlm	RunPercentage	0.728280447
original	glrlm	LongRunLowGrayLevelEmphasis	0.020330196
original	glrlm	RunEntropy	5.407386765
original	glrlm	HighGrayLevelRunEmphasis	1184.755005
original	glrlm	RunLengthNonUniformityNormalized	0.663140178
original	glszm	GrayLevelVariance	190.7109555
original	glszm	ZoneVariance	2241.503098
original	glszm	GrayLevelNonUniformityNormalized	0.024273455
original	glszm	SizeZoneNonUniformityNormalized	0.626656234
original	glszm	SizeZoneNonUniformity	878.5720399
original	glszm	GrayLevelNonUniformity	34.03138374
original	glszm	LargeAreaEmphasis	2252.731812
original	glszm	SmallAreaHighGrayLevelEmphasis	631.4337558
original	glszm	ZonePercentage	0.298424862
original	glszm	LargeAreaLowGrayLevelEmphasis	1.561491132
original	glszm	LargeAreaHighGrayLevelEmphasis	3948505.153
original	glszm	HighGrayLevelZoneEmphasis	762.2346648
original	glszm	SmallAreaEmphasis	0.81863106
original	glszm	LowGrayLevelZoneEmphasis	0.018786011
original	glszm	ZoneEntropy	6.490865536
original	glszm	SmallAreaLowGrayLevelEmphasis	0.012413931
original	ngtdm	Coarseness	0.000735893
original	ngtdm	Complexity	10595.80385
original	ngtdm	Strength	3.615994507
original	ngtdm	Contrast	0.388751879
original	ngtdm	Busyness	0.365035885
wavelet-HHH	gldm	GrayLevelVariance	7.875090276
wavelet-HHH	gldm	HighGrayLevelEmphasis	279.247765
wavelet-HHH	gldm	DependenceEntropy	6.247285041
wavelet-HHH	gldm	DependenceNonUniformity	394.1281396
wavelet-HHH	gldm	GrayLevelNonUniformity	831.3639847
wavelet-HHH	gldm	SmallDependenceEmphasis	0.196198084
wavelet-HHH	gldm	SmallDependenceHighGrayLevelEmphasis	56.89897855
wavelet-HHH	gldm	DependenceNonUniformityNormalized	0.08389275
wavelet-HHH	gldm	LargeDependenceEmphasis	70.94848872
wavelet-HHH	gldm	LargeDependenceLowGrayLevelEmphasis	0.264175771
wavelet-HHH	gldm	DependenceVariance	24.69806668
wavelet-HHH	gldm	LargeDependenceHighGrayLevelEmphasis	19301.82205
wavelet-HHH	gldm	SmallDependenceLowGrayLevelEmphasis	0.001553661
wavelet-HHH	gldm	LowGrayLevelEmphasis	0.00467067
wavelet-HHH	glcm	JointAverage	16.4816208
wavelet-HHH	glcm	SumAverage	32.9632416
wavelet-HHH	glcm	JointEntropy	5.889860659
wavelet-HHH	glcm	ClusterShade	-3.54529676
wavelet-HHH	glcm	MaximumProbability	0.126932328
wavelet-HHH	glcm	Idmn	0.989070152
wavelet-HHH	glcm	JointEnergy	0.056074048
wavelet-HHH	glcm	Contrast	14.56945377
wavelet-HHH	glcm	DifferenceEntropy	2.867013345
wavelet-HHH	glcm	InverseVariance	0.373197912
wavelet-HHH	glcm	DifferenceVariance	8.98079047

wavelet-HHH	glcm	Idn	0.942400061
wavelet-HHH	glcm	Idm	0.455193289
wavelet-HHH	glcm	Correlation	-0.027518534
wavelet-HHH	glcm	Autocorrelation	271.4457375
wavelet-HHH	glcm	SumEntropy	3.58442157
wavelet-HHH	glcm	SumSquares	7.086494699
wavelet-HHH	glcm	ClusterProminence	1568.242583
wavelet-HHH	glcm	Imc2	0.615453847
wavelet-HHH	glcm	Imc1	-0.079270972
wavelet-HHH	glcm	DifferenceAverage	2.347283196
wavelet-HHH	glcm	Id	0.507504643
wavelet-HHH	glcm	ClusterTendency	13.77652503
wavelet-HHH	firstorder	InterquartileRange	37.4934186
wavelet-HHH	firstorder	Skewness	-0.116252512
wavelet-HHH	firstorder	Uniformity	0.176961257
wavelet-HHH	firstorder	Median	-0.051041827
wavelet-HHH	firstorder	Energy	22900980.45
wavelet-HHH	firstorder	RobustMeanAbsoluteDeviation	19.70339672
wavelet-HHH	firstorder	MeanAbsoluteDeviation	42.70361999
wavelet-HHH	firstorder	TotalEnergy	618326472.2
wavelet-HHH	firstorder	Maximum	468.4457846
wavelet-HHH	firstorder	RootMeanSquared	69.81850285
wavelet-HHH	firstorder	90Percentile	69.64862404
wavelet-HHH	firstorder	Minimum	-397.6850149
wavelet-HHH	firstorder	Entropy	3.185454674
wavelet-HHH	firstorder	Range	866.1307994
wavelet-HHH	firstorder	Variance	4874.297467
wavelet-HHH	firstorder	10Percentile	-74.19422485
wavelet-HHH	firstorder	Kurtosis	7.483152313
wavelet-HHH	firstorder	Mean	-0.570853523
wavelet-HHH	glrlm	ShortRunLowGrayLevelEmphasis	0.004375142
wavelet-HHH	glrlm	GrayLevelVariance	9.814983016
wavelet-HHH	glrlm	LowGrayLevelRunEmphasis	0.004940404
wavelet-HHH	glrlm	GrayLevelNonUniformityNormalized	0.136714661
wavelet-HHH	glrlm	RunVariance	0.544461566
wavelet-HHH	glrlm	GrayLevelNonUniformity	499.4989372
wavelet-HHH	glrlm	LongRunEmphasis	2.206975458
wavelet-HHH	glrlm	ShortRunHighGrayLevelEmphasis	239.6667015
wavelet-HHH	glrlm	RunLengthNonUniformity	2474.626294
wavelet-HHH	glrlm	ShortRunEmphasis	0.849184872
wavelet-HHH	glrlm	LongRunHighGrayLevelEmphasis	609.8205997
wavelet-HHH	glrlm	RunPercentage	0.776893604
wavelet-HHH	glrlm	LongRunLowGrayLevelEmphasis	0.009442159
wavelet-HHH	glrlm	RunEntropy	4.299989651
wavelet-HHH	glrlm	HighGrayLevelRunEmphasis	281.0036302
wavelet-HHH	glrlm	RunLengthNonUniformityNormalized	0.677487124
wavelet-HHH	glszm	GrayLevelVariance	25.56453454
wavelet-HHH	glszm	ZoneVariance	3281.29628
wavelet-HHH	glszm	GrayLevelNonUniformityNormalized	0.060302578
wavelet-HHH	glszm	SizeZoneNonUniformityNormalized	0.44680498
wavelet-HHH	glszm	SizeZoneNonUniformity	441.4433198
wavelet-HHH	glszm	GrayLevelNonUniformity	59.57894737
wavelet-HHH	glszm	LargeAreaEmphasis	3303.906883

wavelet-HHH	glszm	SmallAreaHighGrayLevelEmphasis	203.3282586
wavelet-HHH	glszm	ZonePercentage	0.210302256
wavelet-HHH	glszm	LargeAreaLowGrayLevelEmphasis	12.19884875
wavelet-HHH	glszm	LargeAreaHighGrayLevelEmphasis	898804.5526
wavelet-HHH	glszm	HighGrayLevelZoneEmphasis	290.3512146
wavelet-HHH	glszm	SmallAreaEmphasis	0.695040369
wavelet-HHH	glszm	LowGrayLevelZoneEmphasis	0.00780705
wavelet-HHH	glszm	ZoneEntropy	5.914106832
wavelet-HHH	glszm	SmallAreaLowGrayLevelEmphasis	0.006379916
wavelet-HHH	ngtdm	Coarseness	1.64E-03
wavelet-HHH	ngtdm	Complexity	781.1897933
wavelet-HHH	ngtdm	Strength	0.656354135
wavelet-HHH	ngtdm	Contrast	0.031775795
wavelet-HHH	ngtdm	Busyness	0.768960897
wavelet-HLL	gldm	GrayLevelVariance	46.9360068
wavelet-HLL	gldm	HighGrayLevelEmphasis	1846.497871
wavelet-HLL	gldm	DependenceEntropy	7.178338361
wavelet-HLL	gldm	DependenceNonUniformity	611.9778629
wavelet-HLL	gldm	GrayLevelNonUniformity	423.308642
wavelet-HLL	gldm	SmallDependenceEmphasis	0.305172558
wavelet-HLL	gldm	SmallDependenceHighGrayLevelEmphasis	570.5544156
wavelet-HLL	gldm	DependenceNonUniformityNormalized	0.130263487
wavelet-HLL	gldm	LargeDependenceEmphasis	57.51809281
wavelet-HLL	gldm	LargeDependenceLowGrayLevelEmphasis	0.030989883
wavelet-HLL	gldm	DependenceVariance	27.11570896
wavelet-HLL	gldm	LargeDependenceHighGrayLevelEmphasis	108581.9121
wavelet-HLL	gldm	SmallDependenceLowGrayLevelEmphasis	0.000428595
wavelet-HLL	gldm	LowGrayLevelEmphasis	0.000835246
wavelet-HLL	glcm	JointAverage	42.41529774
wavelet-HLL	glcm	SumAverage	84.83059549
wavelet-HLL	glcm	JointEntropy	7.86244418
wavelet-HLL	glcm	ClusterShade	15.333422
wavelet-HLL	glcm	MaximumProbability	0.092632941
wavelet-HLL	glcm	Idmn	0.989132369
wavelet-HLL	glcm	JointEnergy	0.027566555
wavelet-HLL	glcm	Contrast	56.31464186
wavelet-HLL	glcm	DifferenceEntropy	3.684867379
wavelet-HLL	glcm	InverseVariance	0.280524533
wavelet-HLL	glcm	DifferenceVariance	34.34451255
wavelet-HLL	glcm	Idn	0.942730463
wavelet-HLL	glcm	Idm	0.350097619
wavelet-HLL	glcm	Correlation	0.330656454
wavelet-HLL	glcm	Autocorrelation	1813.560712
wavelet-HLL	glcm	SumEntropy	4.972368194
wavelet-HLL	glcm	SumSquares	42.65809642
wavelet-HLL	glcm	ClusterProminence	70093.63219
wavelet-HLL	glcm	Imc2	0.834682879
wavelet-HLL	glcm	Imc1	-0.147340975
wavelet-HLL	glcm	DifferenceAverage	4.601770052
wavelet-HLL	glcm	Id	0.410857203
wavelet-HLL	glcm	ClusterTendency	114.3177438
wavelet-HLL	firstorder	InterquartileRange	122.9433607
wavelet-HLL	firstorder	Skewness	-0.146924371

wavelet-HLL	firstorder	Uniformity	0.090104011
wavelet-HLL	firstorder	Median	-5.480106918
wavelet-HLL	firstorder	Energy	140992927
wavelet-HLL	firstorder	RobustMeanAbsoluteDeviation	64.09421664
wavelet-HLL	firstorder	MeanAbsoluteDeviation	116.839729
wavelet-HLL	firstorder	TotalEnergy	3806809029
wavelet-HLL	firstorder	Maximum	626.540058
wavelet-HLL	firstorder	RootMeanSquared	173.2375994
wavelet-HLL	firstorder	90Percentile	175.8995233
wavelet-HLL	firstorder	Minimum	-1054.760761
wavelet-HLL	firstorder	Entropy	4.39634981
wavelet-HLL	firstorder	Range	1681.300819
wavelet-HLL	firstorder	Variance	29290.66635
wavelet-HLL	firstorder	10Percentile	-246.2205258
wavelet-HLL	firstorder	Kurtosis	5.096236738
wavelet-HLL	firstorder	Mean	-26.84398443
wavelet-HLL	glrlm	ShortRunLowGrayLevelEmphasis	0.000837273
wavelet-HLL	glrlm	GrayLevelVariance	54.87513555
wavelet-HLL	glrlm	LowGrayLevelRunEmphasis	0.000897031
wavelet-HLL	glrlm	GrayLevelNonUniformityNormalized	0.062485991
wavelet-HLL	glrlm	RunVariance	0.469237243
wavelet-HLL	glrlm	GrayLevelNonUniformity	243.6545236
wavelet-HLL	glrlm	LongRunEmphasis	1.944498595
wavelet-HLL	glrlm	ShortRunHighGrayLevelEmphasis	1637.50088
wavelet-HLL	glrlm	RunLengthNonUniformity	2946.354739
wavelet-HLL	glrlm	ShortRunEmphasis	0.89169626
wavelet-HLL	glrlm	LongRunHighGrayLevelEmphasis	3616.723948
wavelet-HLL	glrlm	RunPercentage	0.826390936
wavelet-HLL	glrlm	LongRunLowGrayLevelEmphasis	0.001407456
wavelet-HLL	glrlm	RunEntropy	5.309799974
wavelet-HLL	glrlm	HighGrayLevelRunEmphasis	1840.108632
wavelet-HLL	glrlm	RunLengthNonUniformityNormalized	0.757786176
wavelet-HLL	glszm	GrayLevelVariance	88.56436605
wavelet-HLL	glszm	ZoneVariance	854.3818441
wavelet-HLL	glszm	GrayLevelNonUniformityNormalized	0.030671924
wavelet-HLL	glszm	SizeZoneNonUniformityNormalized	0.462353603
wavelet-HLL	glszm	SizeZoneNonUniformity	761.9587379
wavelet-HLL	glszm	GrayLevelNonUniformity	50.5473301
wavelet-HLL	glszm	LargeAreaEmphasis	862.5084951
wavelet-HLL	glszm	SmallAreaHighGrayLevelEmphasis	1338.232524
wavelet-HLL	glszm	ZonePercentage	0.350787569
wavelet-HLL	glszm	LargeAreaLowGrayLevelEmphasis	0.456917061
wavelet-HLL	glszm	LargeAreaHighGrayLevelEmphasis	1632882.845
wavelet-HLL	glszm	HighGrayLevelZoneEmphasis	1860.831311
wavelet-HLL	glszm	SmallAreaEmphasis	0.706864296
wavelet-HLL	glszm	LowGrayLevelZoneEmphasis	0.001310356
wavelet-HLL	glszm	ZoneEntropy	6.778303019
wavelet-HLL	glszm	SmallAreaLowGrayLevelEmphasis	0.001117097
wavelet-HLL	ngtdm	Coarseness	0.001558657
wavelet-HLL	ngtdm	Complexity	5329.763973
wavelet-HLL	ngtdm	Strength	2.881530391
wavelet-HLL	ngtdm	Contrast	0.090432146
wavelet-HLL	ngtdm	Busyness	0.177794599

wavelet-LHL	gldm	GrayLevelVariance	62.30351874
wavelet-LHL	gldm	HighGrayLevelEmphasis	1473.011069
wavelet-LHL	gldm	DependenceEntropy	7.136688435
wavelet-LHL	gldm	DependenceNonUniformity	671.9297573
wavelet-LHL	gldm	GrayLevelNonUniformity	430.7220094
wavelet-LHL	gldm	SmallDependenceEmphasis	0.351775078
wavelet-LHL	gldm	SmallDependenceHighGrayLevelEmphasis	522.192861
wavelet-LHL	gldm	DependenceNonUniformityNormalized	0.14302464
wavelet-LHL	gldm	LargeDependenceEmphasis	53.45806726
wavelet-LHL	gldm	LargeDependenceLowGrayLevelEmphasis	0.037017419
wavelet-LHL	gldm	DependenceVariance	25.55142819
wavelet-LHL	gldm	LargeDependenceHighGrayLevelEmphasis	79410.58961
wavelet-LHL	gldm	SmallDependenceLowGrayLevelEmphasis	0.00099077
wavelet-LHL	gldm	LowGrayLevelEmphasis	0.001470237
wavelet-LHL	glcm	JointAverage	37.51597713
wavelet-LHL	glcm	SumAverage	75.03195426
wavelet-LHL	glcm	JointEntropy	7.975671917
wavelet-LHL	glcm	ClusterShade	-316.0025737
wavelet-LHL	glcm	MaximumProbability	0.094120918
wavelet-LHL	glcm	Idmn	0.984382204
wavelet-LHL	glcm	JointEnergy	0.027647461
wavelet-LHL	glcm	Contrast	87.58015757
wavelet-LHL	glcm	DifferenceEntropy	3.877466746
wavelet-LHL	glcm	InverseVariance	0.27869846
wavelet-LHL	glcm	DifferenceVariance	56.21785963
wavelet-LHL	glcm	Idn	0.934952306
wavelet-LHL	glcm	Idm	0.339108408
wavelet-LHL	glcm	Correlation	0.200396467
wavelet-LHL	glcm	Autocorrelation	1419.031214
wavelet-LHL	glcm	SumEntropy	4.988140354
wavelet-LHL	glcm	SumSquares	55.36967218
wavelet-LHL	glcm	ClusterProminence	116687.61
wavelet-LHL	glcm	Imc2	0.852868909
wavelet-LHL	glcm	Imc1	-0.154550081
wavelet-LHL	glcm	DifferenceAverage	5.494285064
wavelet-LHL	glcm	Id	0.397733196
wavelet-LHL	glcm	ClusterTendency	133.8985311
wavelet-LHL	firstorder	InterquartileRange	113.9561248
wavelet-LHL	firstorder	Skewness	-0.262713831
wavelet-LHL	firstorder	Uniformity	0.091681994
wavelet-LHL	firstorder	Median	-3.414887742
wavelet-LHL	firstorder	Energy	185214391.9
wavelet-LHL	firstorder	RobustMeanAbsoluteDeviation	62.14935196
wavelet-LHL	firstorder	MeanAbsoluteDeviation	127.0091958
wavelet-LHL	firstorder	TotalEnergy	5000788582
wavelet-LHL	firstorder	Maximum	799.0676849
wavelet-LHL	firstorder	RootMeanSquared	198.5550141
wavelet-LHL	firstorder	90Percentile	193.5548485
wavelet-LHL	firstorder	Minimum	-945.7575643
wavelet-LHL	firstorder	Entropy	4.49439159
wavelet-LHL	firstorder	Range	1744.825249
wavelet-LHL	firstorder	Variance	38869.73932
wavelet-LHL	firstorder	10Percentile	-259.1776787

wavelet-LHL	firstorder	Kurtosis	5.822197532
wavelet-LHL	firstorder	Mean	-23.54473012
wavelet-LHL	glrlm	ShortRunLowGrayLevelEmphasis	0.001552843
wavelet-LHL	glrlm	GrayLevelVariance	73.27921392
wavelet-LHL	glrlm	LowGrayLevelRunEmphasis	0.001625577
wavelet-LHL	glrlm	GrayLevelNonUniformityNormalized	0.063412558
wavelet-LHL	glrlm	RunVariance	0.388328313
wavelet-LHL	glrlm	GrayLevelNonUniformity	249.6022125
wavelet-LHL	glrlm	LongRunEmphasis	1.828704948
wavelet-LHL	glrlm	ShortRunHighGrayLevelEmphasis	1317.2284
wavelet-LHL	glrlm	RunLengthNonUniformity	3008.092109
wavelet-LHL	glrlm	ShortRunEmphasis	0.895608819
wavelet-LHL	glrlm	LongRunHighGrayLevelEmphasis	2699.909892
wavelet-LHL	glrlm	RunPercentage	0.835281789
wavelet-LHL	glrlm	LongRunLowGrayLevelEmphasis	0.00219375
wavelet-LHL	glrlm	RunEntropy	5.398140943
wavelet-LHL	glrlm	HighGrayLevelRunEmphasis	1471.543063
wavelet-LHL	glrlm	RunLengthNonUniformityNormalized	0.765542145
wavelet-LHL	glszm	GrayLevelVariance	127.6753569
wavelet-LHL	glszm	ZoneVariance	818.2429243
wavelet-LHL	glszm	GrayLevelNonUniformityNormalized	0.026276171
wavelet-LHL	glszm	SizeZoneNonUniformityNormalized	0.549496446
wavelet-LHL	glszm	SizeZoneNonUniformity	1016.018929
wavelet-LHL	glszm	GrayLevelNonUniformity	48.58464035
wavelet-LHL	glszm	LargeAreaEmphasis	824.6987561
wavelet-LHL	glszm	SmallAreaHighGrayLevelEmphasis	1146.799358
wavelet-LHL	glszm	ZonePercentage	0.393571733
wavelet-LHL	glszm	LargeAreaLowGrayLevelEmphasis	0.557357587
wavelet-LHL	glszm	LargeAreaHighGrayLevelEmphasis	1226008.501
wavelet-LHL	glszm	HighGrayLevelZoneEmphasis	1482.186047
wavelet-LHL	glszm	SmallAreaEmphasis	0.76972716
wavelet-LHL	glszm	LowGrayLevelZoneEmphasis	0.00261501
wavelet-LHL	glszm	ZoneEntropy	6.740833786
wavelet-LHL	glszm	SmallAreaLowGrayLevelEmphasis	0.002416425
wavelet-LHL	ngtdm	Coarseness	0.001537991
wavelet-LHL	ngtdm	Complexity	8395.11866
wavelet-LHL	ngtdm	Strength	3.304353061
wavelet-LHL	ngtdm	Contrast	0.110466294
wavelet-LHL	ngtdm	Busyness	0.168455214
wavelet-LHH	gldm	GrayLevelVariance	20.55196105
wavelet-LHH	gldm	HighGrayLevelEmphasis	888.1666667
wavelet-LHH	gldm	DependenceEntropy	6.628244256
wavelet-LHH	gldm	DependenceNonUniformity	468.8761175
wavelet-LHH	gldm	GrayLevelNonUniformity	635.2916135
wavelet-LHH	gldm	SmallDependenceEmphasis	0.253467041
wavelet-LHH	gldm	SmallDependenceHighGrayLevelEmphasis	233.5072009
wavelet-LHH	gldm	DependenceNonUniformityNormalized	0.099803346
wavelet-LHH	gldm	LargeDependenceEmphasis	59.1890166
wavelet-LHH	gldm	LargeDependenceLowGrayLevelEmphasis	0.068994682
wavelet-LHH	gldm	DependenceVariance	23.56097547
wavelet-LHH	gldm	LargeDependenceHighGrayLevelEmphasis	51319.54896
wavelet-LHH	gldm	SmallDependenceLowGrayLevelEmphasis	0.00069348
wavelet-LHH	gldm	LowGrayLevelEmphasis	0.001575505

wavelet-LHH	glcm	JointAverage	29.476927
wavelet-LHH	glcm	SumAverage	58.95385399
wavelet-LHH	glcm	JointEntropy	6.75303416
wavelet-LHH	glcm	ClusterShade	1.546405292
wavelet-LHH	glcm	MaximumProbability	0.109257281
wavelet-LHH	glcm	Idmn	0.991041081
wavelet-LHH	glcm	JointEnergy	0.038895138
wavelet-LHH	glcm	Contrast	36.85753489
wavelet-LHH	glcm	DifferenceEntropy	3.308266807
wavelet-LHH	glcm	InverseVariance	0.336967416
wavelet-LHH	glcm	DifferenceVariance	24.61373252
wavelet-LHH	glcm	Idn	0.951023363
wavelet-LHH	glcm	Idm	0.400910777
wavelet-LHH	glcm	Correlation	-0.008565653
wavelet-LHH	glcm	Autocorrelation	868.7835211
wavelet-LHH	glcm	SumEntropy	4.108981225
wavelet-LHH	glcm	SumSquares	18.3227978
wavelet-LHH	glcm	ClusterProminence	16262.48481
wavelet-LHH	glcm	Imc2	0.730975967
wavelet-LHH	glcm	Imc1	-0.109347717
wavelet-LHH	glcm	DifferenceAverage	3.462111024
wavelet-LHH	glcm	Id	0.457893258
wavelet-LHH	glcm	ClusterTendency	36.43365632
wavelet-LHH	firstorder	InterquartileRange	54.23149705
wavelet-LHH	firstorder	Skewness	0.158143671
wavelet-LHH	firstorder	Uniformity	0.135225971
wavelet-LHH	firstorder	Median	-1.54079164
wavelet-LHH	firstorder	Energy	60289103.04
wavelet-LHH	firstorder	RobustMeanAbsoluteDeviation	28.46704748
wavelet-LHH	firstorder	MeanAbsoluteDeviation	65.37979031
wavelet-LHH	firstorder	TotalEnergy	1627805782
wavelet-LHH	firstorder	Maximum	788.0309055
wavelet-LHH	firstorder	RootMeanSquared	113.282521
wavelet-LHH	firstorder	90Percentile	104.3453089
wavelet-LHH	firstorder	Minimum	-714.6204321
wavelet-LHH	firstorder	Entropy	3.712092944
wavelet-LHH	firstorder	Range	1502.651338
wavelet-LHH	firstorder	Variance	12832.0364
wavelet-LHH	firstorder	10Percentile	-104.0907407
wavelet-LHH	firstorder	Kurtosis	10.62610383
wavelet-LHH	firstorder	Mean	-0.945068257
wavelet-LHH	glrlm	ShortRunLowGrayLevelEmphasis	0.001525258
wavelet-LHH	glrlm	GrayLevelVariance	24.95781539
wavelet-LHH	glrlm	LowGrayLevelRunEmphasis	0.001673688
wavelet-LHH	glrlm	GrayLevelNonUniformityNormalized	0.103029378
wavelet-LHH	glrlm	RunVariance	0.442563803
wavelet-LHH	glrlm	GrayLevelNonUniformity	392.230321
wavelet-LHH	glrlm	LongRunEmphasis	1.97841765
wavelet-LHH	glrlm	ShortRunHighGrayLevelEmphasis	782.0560316
wavelet-LHH	glrlm	RunLengthNonUniformity	2740.677295
wavelet-LHH	glrlm	ShortRunEmphasis	0.872657609
wavelet-LHH	glrlm	LongRunHighGrayLevelEmphasis	1741.805357
wavelet-LHH	glrlm	RunPercentage	0.808887579

wavelet-LHH	glrlm	LongRunLowGrayLevelEmphasis	0.002810501
wavelet-LHH	glrlm	RunEntropy	4.713827735
wavelet-LHH	glrlm	HighGrayLevelRunEmphasis	892.8266031
wavelet-LHH	glrlm	RunLengthNonUniformityNormalized	0.720350766
wavelet-LHH	glszm	GrayLevelVariance	60.28572862
wavelet-LHH	glszm	ZoneVariance	1830.873286
wavelet-LHH	glszm	GrayLevelNonUniformityNormalized	0.04303227
wavelet-LHH	glszm	SizeZoneNonUniformityNormalized	0.499383451
wavelet-LHH	glszm	SizeZoneNonUniformity	644.2046512
wavelet-LHH	glszm	GrayLevelNonUniformity	55.51162791
wavelet-LHH	glszm	LargeAreaEmphasis	1844.136434
wavelet-LHH	glszm	SmallAreaHighGrayLevelEmphasis	681.738387
wavelet-LHH	glszm	ZonePercentage	0.27458493
wavelet-LHH	glszm	LargeAreaLowGrayLevelEmphasis	2.134225563
wavelet-LHH	glszm	LargeAreaHighGrayLevelEmphasis	1596871.593
wavelet-LHH	glszm	HighGrayLevelZoneEmphasis	923.5976744
wavelet-LHH	glszm	SmallAreaEmphasis	0.73459226
wavelet-LHH	glszm	LowGrayLevelZoneEmphasis	0.002630118
wavelet-LHH	glszm	ZoneEntropy	6.323300884
wavelet-LHH	glszm	SmallAreaLowGrayLevelEmphasis	0.002297244
wavelet-LHH	ngtdm	Coarseness	0.001784249
wavelet-LHH	ngtdm	Complexity	3876.996194
wavelet-LHH	ngtdm	Strength	2.668945031
wavelet-LHH	ngtdm	Contrast	0.0355112
wavelet-LHH	ngtdm	Busyness	0.204460633
wavelet-LLH	gldm	GrayLevelVariance	50.78562746
wavelet-LLH	gldm	HighGrayLevelEmphasis	2089.52533
wavelet-LLH	gldm	DependenceEntropy	7.048055888
wavelet-LLH	gldm	DependenceNonUniformity	590.1179225
wavelet-LLH	gldm	GrayLevelNonUniformity	475.4171988
wavelet-LLH	gldm	SmallDependenceEmphasis	0.319084311
wavelet-LLH	gldm	SmallDependenceHighGrayLevelEmphasis	691.6995247
wavelet-LLH	gldm	DependenceNonUniformityNormalized	0.125610456
wavelet-LLH	gldm	LargeDependenceEmphasis	54.2899106
wavelet-LLH	gldm	LargeDependenceLowGrayLevelEmphasis	0.026582926
wavelet-LLH	gldm	DependenceVariance	24.76833688
wavelet-LLH	gldm	LargeDependenceHighGrayLevelEmphasis	112619.3035
wavelet-LLH	gldm	SmallDependenceLowGrayLevelEmphasis	0.000437367
wavelet-LLH	gldm	LowGrayLevelEmphasis	0.000784024
wavelet-LLH	glcm	JointAverage	45.01842841
wavelet-LLH	glcm	SumAverage	90.03685682
wavelet-LLH	glcm	JointEntropy	7.697753885
wavelet-LLH	glcm	ClusterShade	502.3832711
wavelet-LLH	glcm	MaximumProbability	0.097975505
wavelet-LLH	glcm	Idmn	0.99178896
wavelet-LLH	glcm	JointEnergy	0.029568245
wavelet-LLH	glcm	Contrast	74.13227131
wavelet-LLH	glcm	DifferenceEntropy	3.73067968
wavelet-LLH	glcm	InverseVariance	0.293864736
wavelet-LLH	glcm	DifferenceVariance	49.16798531
wavelet-LLH	glcm	Idn	0.953200873
wavelet-LLH	glcm	Idm	0.354538256
wavelet-LLH	glcm	Correlation	0.191727623

wavelet-LLH	glcm	Autocorrelation	2035.648886
wavelet-LLH	glcm	SumEntropy	4.819621153
wavelet-LLH	glcm	SumSquares	46.05263647
wavelet-LLH	glcm	ClusterProminence	114012.7705
wavelet-LLH	glcm	Imc2	0.835288229
wavelet-LLH	glcm	Imc1	-0.147958724
wavelet-LLH	glcm	DifferenceAverage	4.908265382
wavelet-LLH	glcm	Id	0.413694868
wavelet-LLH	glcm	ClusterTendency	110.0782746
wavelet-LLH	firstorder	InterquartileRange	88.4061709
wavelet-LLH	firstorder	Skewness	0.227079637
wavelet-LLH	firstorder	Uniformity	0.101195657
wavelet-LLH	firstorder	Median	-2.659327765
wavelet-LLH	firstorder	Energy	149146107.8
wavelet-LLH	firstorder	RobustMeanAbsoluteDeviation	47.66112768
wavelet-LLH	firstorder	MeanAbsoluteDeviation	106.2305799
wavelet-LLH	firstorder	TotalEnergy	4026944911
wavelet-LLH	firstorder	Maximum	1126.144272
wavelet-LLH	firstorder	RootMeanSquared	178.176103
wavelet-LLH	firstorder	90Percentile	168.2697782
wavelet-LLH	firstorder	Minimum	-1117.470666
wavelet-LLH	firstorder	Entropy	4.306875898
wavelet-LLH	firstorder	Range	2243.614938
wavelet-LLH	firstorder	Variance	31668.49075
wavelet-LLH	firstorder	10Percentile	-193.7026408
wavelet-LLH	firstorder	Kurtosis	8.452075551
wavelet-LLH	firstorder	Mean	-8.844937851
wavelet-LLH	glrlm	ShortRunLowGrayLevelEmphasis	0.000791166
wavelet-LLH	glrlm	GrayLevelVariance	60.13652367
wavelet-LLH	glrlm	LowGrayLevelRunEmphasis	0.00084515
wavelet-LLH	glrlm	GrayLevelNonUniformityNormalized	0.072476567
wavelet-LLH	glrlm	RunVariance	0.408571178
wavelet-LLH	glrlm	GrayLevelNonUniformity	283.307877
wavelet-LLH	glrlm	LongRunEmphasis	1.8707756
wavelet-LLH	glrlm	ShortRunHighGrayLevelEmphasis	1868.888558
wavelet-LLH	glrlm	RunLengthNonUniformity	2949.83144
wavelet-LLH	glrlm	ShortRunEmphasis	0.890601226
wavelet-LLH	glrlm	LongRunHighGrayLevelEmphasis	3896.663472
wavelet-LLH	glrlm	RunPercentage	0.829485542
wavelet-LLH	glrlm	LongRunLowGrayLevelEmphasis	0.001269807
wavelet-LLH	glrlm	RunEntropy	5.234836893
wavelet-LLH	glrlm	HighGrayLevelRunEmphasis	2094.400764
wavelet-LLH	glrlm	RunLengthNonUniformityNormalized	0.755552868
wavelet-LLH	glszm	GrayLevelVariance	113.7336548
wavelet-LLH	glszm	ZoneVariance	979.317546
wavelet-LLH	glszm	GrayLevelNonUniformityNormalized	0.029719856
wavelet-LLH	glszm	SizeZoneNonUniformityNormalized	0.529023462
wavelet-LLH	glszm	SizeZoneNonUniformity	883.9982047
wavelet-LLH	glszm	GrayLevelNonUniformity	49.66187911
wavelet-LLH	glszm	LargeAreaEmphasis	987.2220227
wavelet-LLH	glszm	SmallAreaHighGrayLevelEmphasis	1655.972693
wavelet-LLH	glszm	ZonePercentage	0.355683269
wavelet-LLH	glszm	LargeAreaLowGrayLevelEmphasis	0.477671515

wavelet-LLH	glszm	LargeAreaHighGrayLevelEmphasis	2045460.705
wavelet-LLH	glszm	HighGrayLevelZoneEmphasis	2161.255536
wavelet-LLH	glszm	SmallAreaEmphasis	0.755567651
wavelet-LLH	glszm	LowGrayLevelZoneEmphasis	0.001289145
wavelet-LLH	glszm	ZoneEntropy	6.699733797
wavelet-LLH	glszm	SmallAreaLowGrayLevelEmphasis	0.001153799
wavelet-LLH	ngtdm	Coarseness	0.001594732
wavelet-LLH	ngtdm	Complexity	9388.252178
wavelet-LLH	ngtdm	Strength	5.036290597
wavelet-LLH	ngtdm	Contrast	0.066618124
wavelet-LLH	ngtdm	Busyness	0.117156111
wavelet-LLL	gldm	GrayLevelVariance	1166.800227
wavelet-LLL	gldm	HighGrayLevelEmphasis	10371.07471
wavelet-LLL	gldm	DependenceEntropy	8.141861951
wavelet-LLL	gldm	DependenceNonUniformity	992.2383993
wavelet-LLL	gldm	GrayLevelNonUniformity	201.6879523
wavelet-LLL	gldm	SmallDependenceEmphasis	0.471047501
wavelet-LLL	gldm	SmallDependenceHighGrayLevelEmphasis	3427.195317
wavelet-LLL	gldm	DependenceNonUniformityNormalized	0.211204427
wavelet-LLL	gldm	LargeDependenceEmphasis	46.96636867
wavelet-LLL	gldm	LargeDependenceLowGrayLevelEmphasis	0.010359035
wavelet-LLL	gldm	DependenceVariance	25.49740739
wavelet-LLL	gldm	LargeDependenceHighGrayLevelEmphasis	654409.9053
wavelet-LLL	gldm	SmallDependenceLowGrayLevelEmphasis	0.001056343
wavelet-LLL	gldm	LowGrayLevelEmphasis	0.001870302
wavelet-LLL	glcm	JointAverage	100.5556864
wavelet-LLL	glcm	SumAverage	201.1113727
wavelet-LLL	glcm	JointEntropy	9.49933811
wavelet-LLL	glcm	ClusterShade	-265553.357
wavelet-LLL	glcm	MaximumProbability	0.048118009
wavelet-LLL	glcm	Idmn	0.98271874
wavelet-LLL	glcm	JointEnergy	0.010051473
wavelet-LLL	glcm	Contrast	532.7955257
wavelet-LLL	glcm	DifferenceEntropy	4.85861631
wavelet-LLL	glcm	InverseVariance	0.227531401
wavelet-LLL	glcm	DifferenceVariance	340.2018761
wavelet-LLL	glcm	Idn	0.932897806
wavelet-LLL	glcm	Idm	0.28227949
wavelet-LLL	glcm	Correlation	0.712442936
wavelet-LLL	glcm	Autocorrelation	10784.69268
wavelet-LLL	glcm	SumEntropy	6.522147848
wavelet-LLL	glcm	SumSquares	938.7651778
wavelet-LLL	glcm	ClusterProminence	43143125.69
wavelet-LLL	glcm	Imc2	0.979895306
wavelet-LLL	glcm	Imc1	-0.293802213
wavelet-LLL	glcm	DifferenceAverage	13.66433046
wavelet-LLL	glcm	Id	0.33042532
wavelet-LLL	glcm	ClusterTendency	3222.265186
wavelet-LLL	firstorder	InterquartileRange	1095.93707
wavelet-LLL	firstorder	Skewness	-1.169976785
wavelet-LLL	firstorder	Uniformity	0.042930599
wavelet-LLL	firstorder	Median	-20.76101136
wavelet-LLL	firstorder	Energy	4792332302

wavelet-LLL	firstorder	RobustMeanAbsoluteDeviation	510.8336148
wavelet-LLL	firstorder	MeanAbsoluteDeviation	710.5301929
wavelet-LLL	firstorder	TotalEnergy	1.29393E+11
wavelet-LLL	firstorder	Maximum	1249.575096
wavelet-LLL	firstorder	RootMeanSquared	1009.989726
wavelet-LLL	firstorder	90Percentile	94.63780427
wavelet-LLL	firstorder	Minimum	-2908.899116
wavelet-LLL	firstorder	Entropy	5.864958921
wavelet-LLL	firstorder	Range	4158.474212
wavelet-LLL	firstorder	Variance	729449.5834
wavelet-LLL	firstorder	10Percentile	-2005.660737
wavelet-LLL	firstorder	Kurtosis	3.114330676
wavelet-LLL	firstorder	Mean	-539.1007916
wavelet-LLL	glrlm	ShortRunLowGrayLevelEmphasis	0.002048472
wavelet-LLL	glrlm	GrayLevelVariance	1250.449897
wavelet-LLL	glrlm	LowGrayLevelRunEmphasis	0.002100676
wavelet-LLL	glrlm	GrayLevelNonUniformityNormalized	0.029066996
wavelet-LLL	glrlm	RunVariance	0.321272309
wavelet-LLL	glrlm	GrayLevelNonUniformity	117.6609727
wavelet-LLL	glrlm	LongRunEmphasis	1.675329338
wavelet-LLL	glrlm	ShortRunHighGrayLevelEmphasis	8622.761145
wavelet-LLL	glrlm	RunLengthNonUniformity	3252.77215
wavelet-LLL	glrlm	ShortRunEmphasis	0.914524172
wavelet-LLL	glrlm	LongRunHighGrayLevelEmphasis	19196.70558
wavelet-LLL	glrlm	RunPercentage	0.860251498
wavelet-LLL	glrlm	LongRunLowGrayLevelEmphasis	0.002343848
wavelet-LLL	glrlm	RunEntropy	6.67924528
wavelet-LLL	glrlm	HighGrayLevelRunEmphasis	9801.161457
wavelet-LLL	glrlm	RunLengthNonUniformityNormalized	0.804337579
wavelet-LLL	glszm	GrayLevelVariance	1301.403637
wavelet-LLL	glszm	ZoneVariance	295.6692026
wavelet-LLL	glszm	GrayLevelNonUniformityNormalized	0.008926501
wavelet-LLL	glszm	SizeZoneNonUniformityNormalized	0.737543475
wavelet-LLL	glszm	SizeZoneNonUniformity	1711.100862
wavelet-LLL	glszm	GrayLevelNonUniformity	20.70948276
wavelet-LLL	glszm	LargeAreaEmphasis	299.7698276
wavelet-LLL	glszm	SmallAreaHighGrayLevelEmphasis	6073.601409
wavelet-LLL	glszm	ZonePercentage	0.49382716
wavelet-LLL	glszm	LargeAreaLowGrayLevelEmphasis	0.028538141
wavelet-LLL	glszm	LargeAreaHighGrayLevelEmphasis	4212944.803
wavelet-LLL	glszm	HighGrayLevelZoneEmphasis	7278.626293
wavelet-LLL	glszm	SmallAreaEmphasis	0.880443728
wavelet-LLL	glszm	LowGrayLevelZoneEmphasis	0.002547876
wavelet-LLL	glszm	ZoneEntropy	7.630026027
wavelet-LLL	glszm	SmallAreaLowGrayLevelEmphasis	0.001898047
wavelet-LLL	ngtdm	Coarseness	0.001577966
wavelet-LLL	ngtdm	Complexity	94916.65307
wavelet-LLL	ngtdm	Strength	27.13595842
wavelet-LLL	ngtdm	Contrast	0.834774966
wavelet-LLL	ngtdm	Busyness	0.027435269
wavelet-HHL	gldm	GrayLevelVariance	16.11629307
wavelet-HHL	gldm	HighGrayLevelEmphasis	358.1536824
wavelet-HHL	gldm	DependenceEntropy	6.624954201

wavelet-HHL	gldm	DependenceNonUniformity	463.9463602
wavelet-HHL	gldm	GrayLevelNonUniformity	626.6734781
wavelet-HHL	gldm	SmallDependenceEmphasis	0.242945846
wavelet-HHL	gldm	SmallDependenceHighGrayLevelEmphasis	94.81283593
wavelet-HHL	gldm	DependenceNonUniformityNormalized	0.098754015
wavelet-HHL	gldm	LargeDependenceEmphasis	60.44870158
wavelet-HHL	gldm	LargeDependenceLowGrayLevelEmphasis	0.181722485
wavelet-HHL	gldm	DependenceVariance	23.90001107
wavelet-HHL	gldm	LargeDependenceHighGrayLevelEmphasis	20525.50702
wavelet-HHL	gldm	SmallDependenceLowGrayLevelEmphasis	0.001537611
wavelet-HHL	gldm	LowGrayLevelEmphasis	0.003992755
wavelet-HHL	glcm	JointAverage	18.57069275
wavelet-HHL	glcm	SumAverage	37.1413855
wavelet-HHL	glcm	JointEntropy	6.726680114
wavelet-HHL	glcm	ClusterShade	48.26092175
wavelet-HHL	glcm	MaximumProbability	0.111358669
wavelet-HHL	glcm	Idmn	0.98556241
wavelet-HHL	glcm	JointEnergy	0.039101263
wavelet-HHL	glcm	Contrast	28.38582806
wavelet-HHL	glcm	DifferenceEntropy	3.23895315
wavelet-HHL	glcm	InverseVariance	0.339257461
wavelet-HHL	glcm	DifferenceVariance	17.75333565
wavelet-HHL	glcm	Idn	0.935527859
wavelet-HHL	glcm	Idm	0.404985216
wavelet-HHL	glcm	Correlation	0.011608492
wavelet-HHL	glcm	Autocorrelation	345.1306287
wavelet-HHL	glcm	SumEntropy	4.081533639
wavelet-HHL	glcm	SumSquares	14.45238211
wavelet-HHL	glcm	ClusterProminence	6576.149645
wavelet-HHL	glcm	Imc2	0.702966476
wavelet-HHL	glcm	Imc1	-0.098787543
wavelet-HHL	glcm	DifferenceAverage	3.228887633
wavelet-HHL	glcm	Id	0.461823697
wavelet-HHL	glcm	ClusterTendency	29.42370036
wavelet-HHL	firstorder	InterquartileRange	55.16639203
wavelet-HHL	firstorder	Skewness	0.276990745
wavelet-HHL	firstorder	Uniformity	0.133391545
wavelet-HHL	firstorder	Median	-2.085110239
wavelet-HHL	firstorder	Energy	47117025.58
wavelet-HHL	firstorder	RobustMeanAbsoluteDeviation	29.3317874
wavelet-HHL	firstorder	MeanAbsoluteDeviation	62.54234978
wavelet-HHL	firstorder	TotalEnergy	1272159691
wavelet-HHL	firstorder	Maximum	589.0694817
wavelet-HHL	firstorder	RootMeanSquared	100.1457278
wavelet-HHL	firstorder	90Percentile	115.8058877
wavelet-HHL	firstorder	Minimum	-434.5557982
wavelet-HHL	firstorder	Entropy	3.676047139
wavelet-HHL	firstorder	Range	1023.62528
wavelet-HHL	firstorder	Variance	10029.15656
wavelet-HHL	firstorder	10Percentile	-105.2541726
wavelet-HHL	firstorder	Kurtosis	6.306445213
wavelet-HHL	firstorder	Mean	-0.101132795
wavelet-HHL	glrlm	ShortRunLowGrayLevelEmphasis	0.003832753

wavelet-HHL	glrlm	GrayLevelVariance	19.43026957
wavelet-HHL	glrlm	LowGrayLevelRunEmphasis	0.004222833
wavelet-HHL	glrlm	GrayLevelNonUniformityNormalized	0.101177882
wavelet-HHL	glrlm	RunVariance	0.473754063
wavelet-HHL	glrlm	GrayLevelNonUniformity	383.9248541
wavelet-HHL	glrlm	LongRunEmphasis	2.022700121
wavelet-HHL	glrlm	ShortRunHighGrayLevelEmphasis	319.2337719
wavelet-HHL	glrlm	RunLengthNonUniformity	2739.632817
wavelet-HHL	glrlm	ShortRunEmphasis	0.873371764
wavelet-HHL	glrlm	LongRunHighGrayLevelEmphasis	710.248904
wavelet-HHL	glrlm	RunPercentage	0.805940335
wavelet-HHL	glrlm	LongRunLowGrayLevelEmphasis	0.007316043
wavelet-HHL	glrlm	RunEntropy	4.674851502
wavelet-HHL	glrlm	HighGrayLevelRunEmphasis	362.4372951
wavelet-HHL	glrlm	RunLengthNonUniformityNormalized	0.722336526
wavelet-HHL	glszm	GrayLevelVariance	42.36460935
wavelet-HHL	glszm	ZoneVariance	1837.281496
wavelet-HHL	glszm	GrayLevelNonUniformityNormalized	0.045590386
wavelet-HHL	glszm	SizeZoneNonUniformityNormalized	0.471786919
wavelet-HHL	glszm	SizeZoneNonUniformity	590.2054357
wavelet-HHL	glszm	GrayLevelNonUniformity	57.03357314
wavelet-HHL	glszm	LargeAreaEmphasis	1851.384492
wavelet-HHL	glszm	SmallAreaHighGrayLevelEmphasis	283.9260247
wavelet-HHL	glszm	ZonePercentage	0.266283525
wavelet-HHL	glszm	LargeAreaLowGrayLevelEmphasis	5.51645672
wavelet-HHL	glszm	LargeAreaHighGrayLevelEmphasis	624535.96
wavelet-HHL	glszm	HighGrayLevelZoneEmphasis	394.1894484
wavelet-HHL	glszm	SmallAreaEmphasis	0.714178736
wavelet-HHL	glszm	LowGrayLevelZoneEmphasis	0.006182124
wavelet-HHL	glszm	ZoneEntropy	6.20458113
wavelet-HHL	glszm	SmallAreaLowGrayLevelEmphasis	0.005069516
wavelet-HHL	ngtdm	Coarseness	0.001756496
wavelet-HHL	ngtdm	Complexity	1732.150702
wavelet-HHL	ngtdm	Strength	1.028047015
wavelet-HHL	ngtdm	Contrast	0.05326666
wavelet-HHL	ngtdm	Busyness	0.514485141
wavelet-HLH	gldm	GrayLevelVariance	10.20133618
wavelet-HLH	gldm	HighGrayLevelEmphasis	353.3260962
wavelet-HLH	gldm	DependenceEntropy	6.429576149
wavelet-HLH	gldm	DependenceNonUniformity	410.7650064
wavelet-HLH	gldm	GrayLevelNonUniformity	745.4618987
wavelet-HLH	gldm	SmallDependenceEmphasis	0.206335365
wavelet-HLH	gldm	SmallDependenceHighGrayLevelEmphasis	78.41210939
wavelet-HLH	gldm	DependenceNonUniformityNormalized	0.087434016
wavelet-HLH	gldm	LargeDependenceEmphasis	65.08173691
wavelet-HLH	gldm	LargeDependenceLowGrayLevelEmphasis	0.193610862
wavelet-HLH	gldm	DependenceVariance	23.77553377
wavelet-HLH	gldm	LargeDependenceHighGrayLevelEmphasis	22176.05321
wavelet-HLH	gldm	SmallDependenceLowGrayLevelEmphasis	0.001206729
wavelet-HLH	gldm	LowGrayLevelEmphasis	0.003671583
wavelet-HLH	glcm	JointAverage	18.51885379
wavelet-HLH	glcm	SumAverage	37.03770758
wavelet-HLH	glcm	JointEntropy	6.247773616

wavelet-HLH	glcm	ClusterShade	-3.522462419
wavelet-HLH	glcm	MaximumProbability	0.119041801
wavelet-HLH	glcm	Idmn	0.989220895
wavelet-HLH	glcm	JointEnergy	0.04582696
wavelet-HLH	glcm	Contrast	18.96054348
wavelet-HLH	glcm	DifferenceEntropy	3.000845735
wavelet-HLH	glcm	InverseVariance	0.361100092
wavelet-HLH	glcm	DifferenceVariance	11.96948157
wavelet-HLH	glcm	Idn	0.943694396
wavelet-HLH	glcm	Idm	0.433470523
wavelet-HLH	glcm	Correlation	0.009333968
wavelet-HLH	glcm	Autocorrelation	343.0413852
wavelet-HLH	glcm	SumEntropy	3.807538463
wavelet-HLH	glcm	SumSquares	9.573558085
wavelet-HLH	glcm	ClusterProminence	3386.265233
wavelet-HLH	glcm	Imc2	0.646531968
wavelet-HLH	glcm	Imc1	-0.084622982
wavelet-HLH	glcm	DifferenceAverage	2.625549491
wavelet-HLH	glcm	Id	0.488863656
wavelet-HLH	glcm	ClusterTendency	19.33368886
wavelet-HLH	firstorder	InterquartileRange	45.72101111
wavelet-HLH	firstorder	Skewness	0.016025263
wavelet-HLH	firstorder	Uniformity	0.158676437
wavelet-HLH	firstorder	Median	-0.645670971
wavelet-HLH	firstorder	Energy	29634797.22
wavelet-HLH	firstorder	RobustMeanAbsoluteDeviation	22.77613863
wavelet-HLH	firstorder	MeanAbsoluteDeviation	48.33076993
wavelet-HLH	firstorder	TotalEnergy	800139524.9
wavelet-HLH	firstorder	Maximum	539.7477807
wavelet-HLH	firstorder	RootMeanSquared	79.42266831
wavelet-HLH	firstorder	90Percentile	81.54714719
wavelet-HLH	firstorder	Minimum	-425.5692443
wavelet-HLH	firstorder	Entropy	3.352113047
wavelet-HLH	firstorder	Range	965.317025
wavelet-HLH	firstorder	Variance	6307.450783
wavelet-HLH	firstorder	10Percentile	-75.84332568
wavelet-HLH	firstorder	Kurtosis	8.406499153
wavelet-HLH	firstorder	Mean	0.71376394
wavelet-HLH	glrlm	ShortRunLowGrayLevelEmphasis	0.003431949
wavelet-HLH	glrlm	GrayLevelVariance	12.52067193
wavelet-HLH	glrlm	LowGrayLevelRunEmphasis	0.003853044
wavelet-HLH	glrlm	GrayLevelNonUniformityNormalized	0.124197934
wavelet-HLH	glrlm	RunVariance	0.488315398
wavelet-HLH	glrlm	GrayLevelNonUniformity	462.4216979
wavelet-HLH	glrlm	LongRunEmphasis	2.094102368
wavelet-HLH	glrlm	ShortRunHighGrayLevelEmphasis	308.2297499
wavelet-HLH	glrlm	RunLengthNonUniformity	2594.750231
wavelet-HLH	glrlm	ShortRunEmphasis	0.859714701
wavelet-HLH	glrlm	LongRunHighGrayLevelEmphasis	729.9950492
wavelet-HLH	glrlm	RunPercentage	0.791269607
wavelet-HLH	glrlm	LongRunLowGrayLevelEmphasis	0.007114687
wavelet-HLH	glrlm	RunEntropy	4.4067826
wavelet-HLH	glrlm	HighGrayLevelRunEmphasis	356.3184632

wavelet-HLH	glrlm	RunLengthNonUniformityNormalized	0.696858597
wavelet-HLH	glszm	GrayLevelVariance	33.19795256
wavelet-HLH	glszm	ZoneVariance	2671.858546
wavelet-HLH	glszm	GrayLevelNonUniformityNormalized	0.05419323
wavelet-HLH	glszm	SizeZoneNonUniformityNormalized	0.459128526
wavelet-HLH	glszm	SizeZoneNonUniformity	474.2797677
wavelet-HLH	glszm	GrayLevelNonUniformity	55.98160697
wavelet-HLH	glszm	LargeAreaEmphasis	2692.54211
wavelet-HLH	glszm	SmallAreaHighGrayLevelEmphasis	273.9253406
wavelet-HLH	glszm	ZonePercentage	0.2198808
wavelet-HLH	glszm	LargeAreaLowGrayLevelEmphasis	7.931078385
wavelet-HLH	glszm	LargeAreaHighGrayLevelEmphasis	917650.4511
wavelet-HLH	glszm	HighGrayLevelZoneEmphasis	379.8857696
wavelet-HLH	glszm	SmallAreaEmphasis	0.704653975
wavelet-HLH	glszm	LowGrayLevelZoneEmphasis	0.005811155
wavelet-HLH	glszm	ZoneEntropy	6.091913959
wavelet-HLH	glszm	SmallAreaLowGrayLevelEmphasis	0.004703386
wavelet-HLH	ngtdm	Coarseness	0.001682305
wavelet-HLH	ngtdm	Complexity	1196.763664
wavelet-HLH	ngtdm	Strength	1.006732173
wavelet-HLH	ngtdm	Contrast	0.031049065
wavelet-HLH	ngtdm	Busyness	0.550298428

Image type	Feature Class	Feature Name	9: RTSTRUCT: C99-DNU_segment_GTV/ITV Left Lun
general	info	BoundingBox	(0, 0, 1, 8, 10, 8)
general	info	EnabledImageTypes	{u'Original': {}, u'Wavelet': {}}
general	info	GeneralSettings	{'distances': [1], 'additionalInfo': True, 'force2D': False, 'interpolator': 'sitkBSpline', 'resampledPixelSpacing': [3.0], 'label': 1, 'normalizeScale': 1, 'normalize': False, 'force2Ddimension': 0, 'removeOutliers': None, 'minimumROISize': None, u'binWidth': 25.0, 'minimumROIDimensions': 1, u'correctMask': True, 'preCrop': False, u'symmetricalGLCM': True, 'resegmentRange': None, 'padDistance': 5}
general	info	ImageHash	f1059adcd825097f815b994a024245d90eb323c3
general	info	ImageSpacing	(0.9765624999999998, 0.9765624999999998, 1.999999999999999)
general	info	MaskHash	5ab5a502df9926f285aed950da104f1e523cbb58
general	info	NumpyVersion	1.13.1
general	info	PyWaveletVersion	0.5.2
general	info	SimpleITKVersion	1.1.0
general	info	Version	1.3.0.post102+g1b0eb74
general	info	VolumeNum	1
general	info	VoxelNum	324
original	shape	Maximum3DDiameter	32.31098884
original	shape	Maximum2DDiameterSlice	27.65863337
original	shape	Sphericity	0.828473762
original	shape	MinorAxis	22.94784445
original	shape	Elongation	0.81174244
original	shape	SurfaceVolumeRatio	0.283292955
original	shape	Volume	8748
original	shape	MajorAxis	28.26985916
original	shape	SurfaceArea	2478.246768
original	shape	Flatness	0.692919896
original	shape	LeastAxis	19.58874787
original	shape	Maximum2DDiameterColumn	23.43074903
original	shape	Maximum2DDiameterRow	30
original	gldm	GrayLevelVariance	185.4966754
original	gldm	HighGrayLevelEmphasis	1374.682099
original	gldm	DependenceEntropy	6.571536752
original	gldm	DependenceNonUniformity	78.16049383
original	gldm	GrayLevelNonUniformity	19.5
original	gldm	SmallDependenceEmphasis	0.491118921
original	gldm	SmallDependenceHighGrayLevelEmphasis	614.2032283
original	gldm	DependenceNonUniformityNormalized	0.241236092
original	gldm	LargeDependenceEmphasis	14.17283951
original	gldm	LargeDependenceLowGrayLevelEmphasis	0.12000018
original	gldm	DependenceVariance	6.180003048
original	gldm	LargeDependenceHighGrayLevelEmphasis	22785.04012
original	gldm	SmallDependenceLowGrayLevelEmphasis	0.009822394
original	gldm	LowGrayLevelEmphasis	0.014855004
original	glcm	JointAverage	33.33994621
original	glcm	SumAverage	66.67989242
original	glcm	JointEntropy	7.840748315
original	glcm	ClusterShade	-9985.984881
original	glcm	MaximumProbability	0.038422111
original	glcm	Idmn	0.957004605
original	glcm	JointEnergy	0.007961067
original	glcm	Contrast	216.5173942

original	glcm	DifferenceEntropy	4.623362468
original	glcm	InverseVariance	0.164631354
original	glcm	DifferenceVariance	108.1854492
original	glcm	Idn	0.878891272
original	glcm	Idm	0.192168365
original	glcm	Correlation	0.459742892
original	glcm	Autocorrelation	1202.957306
original	glcm	SumEntropy	5.819254562
original	glcm	SumSquares	199.5129292
original	glcm	ClusterProminence	1002485.827
original	glcm	Imc2	0.992419695
original	glcm	Imc1	-0.422098314
original	glcm	DifferenceAverage	10.24515702
original	glcm	Id	0.259433995
original	glcm	ClusterTendency	581.5343225
original	firstorder	InterquartileRange	291.25
original	firstorder	Skewness	-0.785171749
original	firstorder	Uniformity	0.060185185
original	firstorder	Median	-29.5
original	firstorder	Energy	44820380
original	firstorder	RobustMeanAbsoluteDeviation	160.5565771
original	firstorder	MeanAbsoluteDeviation	260.9720317
original	firstorder	TotalEnergy	1210150260
original	firstorder	Maximum	623
original	firstorder	RootMeanSquared	371.9334701
original	firstorder	90Percentile	76.1
original	firstorder	Minimum	-997
original	firstorder	Entropy	4.905493456
original	firstorder	Range	1620
original	firstorder	Variance	115554.0077
original	firstorder	10Percentile	-729.5
original	firstorder	Kurtosis	3.342626942
original	firstorder	Mean	-150.9320988
original	glrlm	ShortRunLowGrayLevelEmphasis	0.014674587
original	glrlm	GrayLevelVariance	191.4805453
original	glrlm	LowGrayLevelRunEmphasis	0.0152347
original	glrlm	GrayLevelNonUniformityNormalized	0.052861643
original	glrlm	RunVariance	0.094147852
original	glrlm	GrayLevelNonUniformity	15.93753457
original	glrlm	LongRunEmphasis	1.252948416
original	glrlm	ShortRunHighGrayLevelEmphasis	1280.769471
original	glrlm	RunLengthNonUniformity	264.3147826
original	glrlm	ShortRunEmphasis	0.949372916
original	glrlm	LongRunHighGrayLevelEmphasis	1760.595345
original	glrlm	RunPercentage	0.929724596
original	glrlm	LongRunLowGrayLevelEmphasis	0.017504864
original	glrlm	RunEntropy	5.293100007
original	glrlm	HighGrayLevelRunEmphasis	1359.574683
original	glrlm	RunLengthNonUniformityNormalized	0.876688388
original	glszm	GrayLevelVariance	235.606405
original	glszm	ZoneVariance	16.08832645
original	glszm	GrayLevelNonUniformityNormalized	0.026407541
original	glszm	SizeZoneNonUniformityNormalized	0.613636364
original	glszm	SizeZoneNonUniformity	108
original	glszm	GrayLevelNonUniformity	4.647727273
original	glszm	LargeAreaEmphasis	19.47727273
original	glszm	SmallAreaHighGrayLevelEmphasis	994.0160764
original	glszm	ZonePercentage	0.543209877

original	glszm	LargeAreaLowGrayLevelEmphasis	0.069197659
original	glszm	LargeAreaHighGrayLevelEmphasis	32182.46023
original	glszm	HighGrayLevelZoneEmphasis	1250.897727
original	glszm	SmallAreaEmphasis	0.808781692
original	glszm	LowGrayLevelZoneEmphasis	0.019827242
original	glszm	ZoneEntropy	6.202059568
original	glszm	SmallAreaLowGrayLevelEmphasis	0.017480023
original	ngtdm	Coarseness	0.013135283
original	ngtdm	Complexity	9976.523907
original	ngtdm	Strength	31.08516302
original	ngtdm	Contrast	0.688721625
original	ngtdm	Busyness	0.02745322
wavelet-HHH	gldm	GrayLevelVariance	13.73551097
wavelet-HHH	gldm	HighGrayLevelEmphasis	252.5524691
wavelet-HHH	gldm	DependenceEntropy	5.835897879
wavelet-HHH	gldm	DependenceNonUniformity	53.89506173
wavelet-HHH	gldm	GrayLevelNonUniformity	32.51851852
wavelet-HHH	gldm	SmallDependenceEmphasis	0.328504428
wavelet-HHH	gldm	SmallDependenceHighGrayLevelEmphasis	90.18280522
wavelet-HHH	gldm	DependenceNonUniformityNormalized	0.166342783
wavelet-HHH	gldm	LargeDependenceEmphasis	14.54320988
wavelet-HHH	gldm	LargeDependenceLowGrayLevelEmphasis	0.070083148
wavelet-HHH	gldm	DependenceVariance	4.200236244
wavelet-HHH	gldm	LargeDependenceHighGrayLevelEmphasis	3452.570988
wavelet-HHH	gldm	SmallDependenceLowGrayLevelEmphasis	0.006396864
wavelet-HHH	gldm	LowGrayLevelEmphasis	0.009554682
wavelet-HHH	glcm	JointAverage	15.47522988
wavelet-HHH	glcm	SumAverage	30.95045976
wavelet-HHH	glcm	JointEntropy	6.807032842
wavelet-HHH	glcm	ClusterShade	5.30251015
wavelet-HHH	glcm	MaximumProbability	0.042245024
wavelet-HHH	glcm	Idmn	0.973240595
wavelet-HHH	glcm	JointEnergy	0.013459943
wavelet-HHH	glcm	Contrast	30.23678717
wavelet-HHH	glcm	DifferenceEntropy	3.404035901
wavelet-HHH	glcm	InverseVariance	0.268357615
wavelet-HHH	glcm	DifferenceVariance	15.59718957
wavelet-HHH	glcm	Idn	0.900213138
wavelet-HHH	glcm	Idm	0.275209798
wavelet-HHH	glcm	Correlation	-0.039913811
wavelet-HHH	glcm	Autocorrelation	238.9125621
wavelet-HHH	glcm	SumEntropy	4.158128869
wavelet-HHH	glcm	SumSquares	14.54696518
wavelet-HHH	glcm	ClusterProminence	5281.282758
wavelet-HHH	glcm	Imc2	0.857805264
wavelet-HHH	glcm	Imc1	-0.17905042
wavelet-HHH	glcm	DifferenceAverage	3.809433774
wavelet-HHH	glcm	Id	0.358820526
wavelet-HHH	glcm	ClusterTendency	27.95107355
wavelet-HHH	firstorder	InterquartileRange	86.14125064
wavelet-HHH	firstorder	Skewness	-0.00136046
wavelet-HHH	firstorder	Uniformity	0.100365798
wavelet-HHH	firstorder	Median	-0.522185681
wavelet-HHH	firstorder	Energy	2754685.594
wavelet-HHH	firstorder	RobustMeanAbsoluteDeviation	36.73151567
wavelet-HHH	firstorder	MeanAbsoluteDeviation	63.70960409
wavelet-HHH	firstorder	TotalEnergy	74376511.03
wavelet-HHH	firstorder	Maximum	378.6259717

wavelet-HHH	firstorder	RootMeanSquared	92.20691964
wavelet-HHH	firstorder	90Percentile	98.42069556
wavelet-HHH	firstorder	Minimum	-350.1401229
wavelet-HHH	firstorder	Entropy	3.719795512
wavelet-HHH	firstorder	Range	728.7660946
wavelet-HHH	firstorder	Variance	8499.826943
wavelet-HHH	firstorder	10Percentile	-97.80464423
wavelet-HHH	firstorder	Kurtosis	6.175840976
wavelet-HHH	firstorder	Mean	-1.512972717
wavelet-HHH	glrlm	ShortRunLowGrayLevelEmphasis	0.009739487
wavelet-HHH	glrlm	GrayLevelVariance	14.64867737
wavelet-HHH	glrlm	LowGrayLevelRunEmphasis	0.010024951
wavelet-HHH	glrlm	GrayLevelNonUniformityNormalized	0.094805603
wavelet-HHH	glrlm	RunVariance	0.111014461
wavelet-HHH	glrlm	GrayLevelNonUniformity	28.10379877
wavelet-HHH	glrlm	LongRunEmphasis	1.307560426
wavelet-HHH	glrlm	ShortRunHighGrayLevelEmphasis	238.8581407
wavelet-HHH	glrlm	RunLengthNonUniformity	251.8991323
wavelet-HHH	glrlm	ShortRunEmphasis	0.937238049
wavelet-HHH	glrlm	LongRunHighGrayLevelEmphasis	327.1271835
wavelet-HHH	glrlm	RunPercentage	0.914767331
wavelet-HHH	glrlm	LongRunLowGrayLevelEmphasis	0.011416178
wavelet-HHH	glrlm	RunEntropy	4.154954048
wavelet-HHH	glrlm	HighGrayLevelRunEmphasis	253.8560648
wavelet-HHH	glrlm	RunLengthNonUniformityNormalized	0.84925605
wavelet-HHH	glszm	GrayLevelVariance	28.69760702
wavelet-HHH	glszm	ZoneVariance	45.56064209
wavelet-HHH	glszm	GrayLevelNonUniformityNormalized	0.067776457
wavelet-HHH	glszm	SizeZoneNonUniformityNormalized	0.480529132
wavelet-HHH	glszm	SizeZoneNonUniformity	55.74137931
wavelet-HHH	glszm	GrayLevelNonUniformity	7.862068966
wavelet-HHH	glszm	LargeAreaEmphasis	53.36206897
wavelet-HHH	glszm	SmallAreaHighGrayLevelEmphasis	202.3575516
wavelet-HHH	glszm	ZonePercentage	0.358024691
wavelet-HHH	glszm	LargeAreaLowGrayLevelEmphasis	0.240256051
wavelet-HHH	glszm	LargeAreaHighGrayLevelEmphasis	12956.4569
wavelet-HHH	glszm	HighGrayLevelZoneEmphasis	269.75
wavelet-HHH	glszm	SmallAreaEmphasis	0.719414587
wavelet-HHH	glszm	LowGrayLevelZoneEmphasis	0.018478465
wavelet-HHH	glszm	ZoneEntropy	5.231415832
wavelet-HHH	glszm	SmallAreaLowGrayLevelEmphasis	0.016924446
wavelet-HHH	ngtdm	Coarseness	0.021723862
wavelet-HHH	ngtdm	Complexity	741.5150415
wavelet-HHH	ngtdm	Strength	4.759810169
wavelet-HHH	ngtdm	Contrast	0.124902827
wavelet-HHH	ngtdm	Busyness	0.095851384
wavelet-HLL	gldm	GrayLevelVariance	79.01039285
wavelet-HLL	gldm	HighGrayLevelEmphasis	777.5030864
wavelet-HLL	gldm	DependenceEntropy	6.377525056
wavelet-HLL	gldm	DependenceNonUniformity	89.75925926
wavelet-HLL	gldm	GrayLevelNonUniformity	15.58024691
wavelet-HLL	gldm	SmallDependenceEmphasis	0.480875362
wavelet-HLL	gldm	SmallDependenceHighGrayLevelEmphasis	408.2472706
wavelet-HLL	gldm	DependenceNonUniformityNormalized	0.277034751
wavelet-HLL	gldm	LargeDependenceEmphasis	7.382716049
wavelet-HLL	gldm	LargeDependenceLowGrayLevelEmphasis	0.015513515
wavelet-HLL	gldm	DependenceVariance	2.361796982
wavelet-HLL	gldm	LargeDependenceHighGrayLevelEmphasis	5338.645062

wavelet-HLL	gldm	SmallDependenceLowGrayLevelEmphasis	0.004264971
wavelet-HLL	gldm	LowGrayLevelEmphasis	0.005230232
wavelet-HLL	glcm	JointAverage	26.40928501
wavelet-HLL	glcm	SumAverage	52.81857002
wavelet-HLL	glcm	JointEntropy	8.068780113
wavelet-HLL	glcm	ClusterShade	2156.797946
wavelet-HLL	glcm	MaximumProbability	0.018094679
wavelet-HLL	glcm	Idmn	0.968982999
wavelet-HLL	glcm	JointEnergy	0.004766493
wavelet-HLL	glcm	Contrast	130.1044977
wavelet-HLL	glcm	DifferenceEntropy	4.338080688
wavelet-HLL	glcm	InverseVariance	0.156610616
wavelet-HLL	glcm	DifferenceVariance	62.7011503
wavelet-HLL	glcm	Idn	0.892776374
wavelet-HLL	glcm	Idm	0.161316922
wavelet-HLL	glcm	Correlation	0.247328622
wavelet-HLL	glcm	Autocorrelation	719.0239182
wavelet-HLL	glcm	SumEntropy	5.500804487
wavelet-HLL	glcm	SumSquares	86.58695049
wavelet-HLL	glcm	ClusterProminence	190612.2082
wavelet-HLL	glcm	Imc2	0.986068203
wavelet-HLL	glcm	Imc1	-0.364740277
wavelet-HLL	glcm	DifferenceAverage	8.02571597
wavelet-HLL	glcm	Id	0.244066222
wavelet-HLL	glcm	ClusterTendency	216.2433043
wavelet-HLL	firstorder	InterquartileRange	161.1079033
wavelet-HLL	firstorder	Skewness	0.744801744
wavelet-HLL	firstorder	Uniformity	0.048087182
wavelet-HLL	firstorder	Median	-17.19240913
wavelet-HLL	firstorder	Energy	16073262.12
wavelet-HLL	firstorder	RobustMeanAbsoluteDeviation	85.90893009
wavelet-HLL	firstorder	MeanAbsoluteDeviation	154.9334188
wavelet-HLL	firstorder	TotalEnergy	433978077.4
wavelet-HLL	firstorder	Maximum	848.0056457
wavelet-HLL	firstorder	RootMeanSquared	222.7304059
wavelet-HLL	firstorder	90Percentile	305.7362734
wavelet-HLL	firstorder	Minimum	-642.7045959
wavelet-HLL	firstorder	Entropy	4.861144999
wavelet-HLL	firstorder	Range	1490.710242
wavelet-HLL	firstorder	Variance	49607.2008
wavelet-HLL	firstorder	10Percentile	-269.350898
wavelet-HLL	firstorder	Kurtosis	4.559237868
wavelet-HLL	firstorder	Mean	-1.277857135
wavelet-HLL	glrlm	ShortRunLowGrayLevelEmphasis	0.005348825
wavelet-HLL	glrlm	GrayLevelVariance	81.35568481
wavelet-HLL	glrlm	LowGrayLevelRunEmphasis	0.005407818
wavelet-HLL	glrlm	GrayLevelNonUniformityNormalized	0.046389779
wavelet-HLL	glrlm	RunVariance	0.058137701
wavelet-HLL	glrlm	GrayLevelNonUniformity	14.31568037
wavelet-HLL	glrlm	LongRunEmphasis	1.161567058
wavelet-HLL	glrlm	ShortRunHighGrayLevelEmphasis	755.3666616
wavelet-HLL	glrlm	RunLengthNonUniformity	281.8127462
wavelet-HLL	glrlm	ShortRunEmphasis	0.965215358
wavelet-HLL	glrlm	LongRunHighGrayLevelEmphasis	895.7371124
wavelet-HLL	glrlm	RunPercentage	0.952279202
wavelet-HLL	glrlm	LongRunLowGrayLevelEmphasis	0.00568341
wavelet-HLL	glrlm	RunEntropy	5.099540382
wavelet-HLL	glrlm	HighGrayLevelRunEmphasis	780.5208019

wavelet-HLL	glrlm	RunLengthNonUniformityNormalized	0.913047147
wavelet-HLL	glszm	GrayLevelVariance	112.5507196
wavelet-HLL	glszm	ZoneVariance	4.102512309
wavelet-HLL	glszm	GrayLevelNonUniformityNormalized	0.032382275
wavelet-HLL	glszm	SizeZoneNonUniformityNormalized	0.50284055
wavelet-HLL	glszm	SizeZoneNonUniformity	89.50561798
wavelet-HLL	glszm	GrayLevelNonUniformity	5.764044944
wavelet-HLL	glszm	LargeAreaEmphasis	7.415730337
wavelet-HLL	glszm	SmallAreaHighGrayLevelEmphasis	647.8603079
wavelet-HLL	glszm	ZonePercentage	0.549382716
wavelet-HLL	glszm	LargeAreaLowGrayLevelEmphasis	0.018471797
wavelet-HLL	glszm	LargeAreaHighGrayLevelEmphasis	5313.162921
wavelet-HLL	glszm	HighGrayLevelZoneEmphasis	834.5898876
wavelet-HLL	glszm	SmallAreaEmphasis	0.736889111
wavelet-HLL	glszm	LowGrayLevelZoneEmphasis	0.008042207
wavelet-HLL	glszm	ZoneEntropy	6.09407178
wavelet-HLL	glszm	SmallAreaLowGrayLevelEmphasis	0.007490809
wavelet-HLL	ngtdm	Coarseness	0.021308646
wavelet-HLL	ngtdm	Complexity	4838.824843
wavelet-HLL	ngtdm	Strength	19.26990107
wavelet-HLL	ngtdm	Contrast	0.336707329
wavelet-HLL	ngtdm	Busyness	0.033276496
wavelet-LHL	gldm	GrayLevelVariance	147.0895062
wavelet-LHL	gldm	HighGrayLevelEmphasis	1104.648148
wavelet-LHL	gldm	DependenceEntropy	6.486321812
wavelet-LHL	gldm	DependenceNonUniformity	120.9567901
wavelet-LHL	gldm	GrayLevelNonUniformity	10.64814815
wavelet-LHL	gldm	SmallDependenceEmphasis	0.601868544
wavelet-LHL	gldm	SmallDependenceHighGrayLevelEmphasis	663.9647123
wavelet-LHL	gldm	DependenceNonUniformityNormalized	0.373323426
wavelet-LHL	gldm	LargeDependenceEmphasis	4.12962963
wavelet-LHL	gldm	LargeDependenceLowGrayLevelEmphasis	0.010284012
wavelet-LHL	gldm	DependenceVariance	1.012879134
wavelet-LHL	gldm	LargeDependenceHighGrayLevelEmphasis	4367.950617
wavelet-LHL	gldm	SmallDependenceLowGrayLevelEmphasis	0.005697165
wavelet-LHL	gldm	LowGrayLevelEmphasis	0.006265768
wavelet-LHL	glcm	JointAverage	31.58978057
wavelet-LHL	glcm	SumAverage	63.17956115
wavelet-LHL	glcm	JointEntropy	8.391103896
wavelet-LHL	glcm	ClusterShade	4170.379006
wavelet-LHL	glcm	MaximumProbability	0.014462857
wavelet-LHL	glcm	Idmn	0.962479129
wavelet-LHL	glcm	JointEnergy	0.003531167
wavelet-LHL	glcm	Contrast	247.7411574
wavelet-LHL	glcm	DifferenceEntropy	4.701835734
wavelet-LHL	glcm	InverseVariance	0.122668361
wavelet-LHL	glcm	DifferenceVariance	116.2428959
wavelet-LHL	glcm	Idn	0.882668349
wavelet-LHL	glcm	Idm	0.115673706
wavelet-LHL	glcm	Correlation	0.208660228
wavelet-LHL	glcm	Autocorrelation	1030.035021
wavelet-LHL	glcm	SumEntropy	5.840513283
wavelet-LHL	glcm	SumSquares	155.9484372
wavelet-LHL	glcm	ClusterProminence	566913.726
wavelet-LHL	glcm	Imc2	0.995533146
wavelet-LHL	glcm	Imc1	-0.440037866
wavelet-LHL	glcm	DifferenceAverage	11.06307531
wavelet-LHL	glcm	Id	0.19440322

wavelet-LHL	glcm	ClusterTendency	376.0525913
wavelet-LHL	firstorder	InterquartileRange	340.4662443
wavelet-LHL	firstorder	Skewness	0.459552285
wavelet-LHL	firstorder	Uniformity	0.032864655
wavelet-LHL	firstorder	Median	-11.1571158
wavelet-LHL	firstorder	Energy	29706452.47
wavelet-LHL	firstorder	RobustMeanAbsoluteDeviation	135.9114588
wavelet-LHL	firstorder	MeanAbsoluteDeviation	223.5349634
wavelet-LHL	firstorder	TotalEnergy	802074216.8
wavelet-LHL	firstorder	Maximum	1077.838499
wavelet-LHL	firstorder	RootMeanSquared	302.7979222
wavelet-LHL	firstorder	90Percentile	414.0552
wavelet-LHL	firstorder	Minimum	-729.8141771
wavelet-LHL	firstorder	Entropy	5.357578874
wavelet-LHL	firstorder	Range	1807.652676
wavelet-LHL	firstorder	Variance	91557.98441
wavelet-LHL	firstorder	10Percentile	-363.3690972
wavelet-LHL	firstorder	Kurtosis	3.590777011
wavelet-LHL	firstorder	Mean	11.34007505
wavelet-LHL	glrlm	ShortRunLowGrayLevelEmphasis	0.006387004
wavelet-LHL	glrlm	GrayLevelVariance	149.0873299
wavelet-LHL	glrlm	LowGrayLevelRunEmphasis	0.006416269
wavelet-LHL	glrlm	GrayLevelNonUniformityNormalized	0.031863222
wavelet-LHL	glrlm	RunVariance	0.032395496
wavelet-LHL	glrlm	GrayLevelNonUniformity	10.02131783
wavelet-LHL	glrlm	LongRunEmphasis	1.09467719
wavelet-LHL	glrlm	ShortRunHighGrayLevelEmphasis	1081.794249
wavelet-LHL	glrlm	RunLengthNonUniformity	296.8932041
wavelet-LHL	glrlm	ShortRunEmphasis	0.978002652
wavelet-LHL	glrlm	LongRunHighGrayLevelEmphasis	1206.323198
wavelet-LHL	glrlm	RunPercentage	0.970560304
wavelet-LHL	glrlm	LongRunLowGrayLevelEmphasis	0.006543881
wavelet-LHL	glrlm	RunEntropy	5.50043977
wavelet-LHL	glrlm	HighGrayLevelRunEmphasis	1105.63691
wavelet-LHL	glrlm	RunLengthNonUniformityNormalized	0.943785383
wavelet-LHL	glszm	GrayLevelVariance	170.8945763
wavelet-LHL	glszm	ZoneVariance	2.176450114
wavelet-LHL	glszm	GrayLevelNonUniformityNormalized	0.025572777
wavelet-LHL	glszm	SizeZoneNonUniformityNormalized	0.582645728
wavelet-LHL	glszm	SizeZoneNonUniformity	128.7647059
wavelet-LHL	glszm	GrayLevelNonUniformity	5.65158371
wavelet-LHL	glszm	LargeAreaEmphasis	4.325791855
wavelet-LHL	glszm	SmallAreaHighGrayLevelEmphasis	869.858503
wavelet-LHL	glszm	ZonePercentage	0.682098765
wavelet-LHL	glszm	LargeAreaLowGrayLevelEmphasis	0.012644973
wavelet-LHL	glszm	LargeAreaHighGrayLevelEmphasis	4463.99095
wavelet-LHL	glszm	HighGrayLevelZoneEmphasis	1106.257919
wavelet-LHL	glszm	SmallAreaEmphasis	0.791677979
wavelet-LHL	glszm	LowGrayLevelZoneEmphasis	0.008540271
wavelet-LHL	glszm	ZoneEntropy	6.275579669
wavelet-LHL	glszm	SmallAreaLowGrayLevelEmphasis	0.008219602
wavelet-LHL	ngtdm	Coarseness	0.020926513
wavelet-LHL	ngtdm	Complexity	10680.22738
wavelet-LHL	ngtdm	Strength	24.25374759
wavelet-LHL	ngtdm	Contrast	0.628819058
wavelet-LHL	ngtdm	Busyness	0.026658915
wavelet-LHH	gldm	GrayLevelVariance	49.31575789
wavelet-LHH	gldm	HighGrayLevelEmphasis	762.8981481

wavelet-LHH	gldm	DependenceEntropy	6.023253573
wavelet-LHH	gldm	DependenceNonUniformity	89.24074074
wavelet-LHH	gldm	GrayLevelNonUniformity	19.53703704
wavelet-LHH	gldm	SmallDependenceEmphasis	0.505013612
wavelet-LHH	gldm	SmallDependenceHighGrayLevelEmphasis	436.2983427
wavelet-LHH	gldm	DependenceNonUniformityNormalized	0.275434385
wavelet-LHH	gldm	LargeDependenceEmphasis	6.783950617
wavelet-LHH	gldm	LargeDependenceLowGrayLevelEmphasis	0.014201047
wavelet-LHH	gldm	DependenceVariance	1.927640604
wavelet-LHH	gldm	LargeDependenceHighGrayLevelEmphasis	4688.533951
wavelet-LHH	gldm	SmallDependenceLowGrayLevelEmphasis	0.004121975
wavelet-LHH	gldm	LowGrayLevelEmphasis	0.004969357
wavelet-LHH	glcm	JointAverage	26.56983718
wavelet-LHH	glcm	SumAverage	53.13967437
wavelet-LHH	glcm	JointEntropy	7.767275547
wavelet-LHH	glcm	ClusterShade	549.1111856
wavelet-LHH	glcm	MaximumProbability	0.022034008
wavelet-LHH	glcm	Idmn	0.97600002
wavelet-LHH	glcm	JointEnergy	0.006133203
wavelet-LHH	glcm	Contrast	100.3134302
wavelet-LHH	glcm	DifferenceEntropy	4.161306553
wavelet-LHH	glcm	InverseVariance	0.178901723
wavelet-LHH	glcm	DifferenceVariance	51.00071929
wavelet-LHH	glcm	Idn	0.904742261
wavelet-LHH	glcm	Idm	0.172170086
wavelet-LHH	glcm	Correlation	-0.016087865
wavelet-LHH	glcm	Autocorrelation	705.3336575
wavelet-LHH	glcm	SumEntropy	4.975825146
wavelet-LHH	glcm	SumSquares	49.52564368
wavelet-LHH	glcm	ClusterProminence	68646.92958
wavelet-LHH	glcm	Imc2	0.961511625
wavelet-LHH	glcm	Imc1	-0.286675079
wavelet-LHH	glcm	DifferenceAverage	6.960169575
wavelet-LHH	glcm	Id	0.257138451
wavelet-LHH	glcm	ClusterTendency	97.7891445
wavelet-LHH	firstorder	InterquartileRange	145.620803
wavelet-LHH	firstorder	Skewness	0.584272375
wavelet-LHH	firstorder	Uniformity	0.060299497
wavelet-LHH	firstorder	Median	-3.597443214
wavelet-LHH	firstorder	Energy	10034645.62
wavelet-LHH	firstorder	RobustMeanAbsoluteDeviation	65.1871417
wavelet-LHH	firstorder	MeanAbsoluteDeviation	118.3223722
wavelet-LHH	firstorder	TotalEnergy	270935431.8
wavelet-LHH	firstorder	Maximum	846.6142801
wavelet-LHH	firstorder	RootMeanSquared	175.9861599
wavelet-LHH	firstorder	90Percentile	214.9643312
wavelet-LHH	firstorder	Minimum	-649.7104158
wavelet-LHH	firstorder	Entropy	4.515407915
wavelet-LHH	firstorder	Range	1496.324696
wavelet-LHH	firstorder	Variance	30949.39894
wavelet-LHH	firstorder	10Percentile	-179.4827218
wavelet-LHH	firstorder	Kurtosis	6.629526224
wavelet-LHH	firstorder	Mean	4.661493739
wavelet-LHH	glrlm	ShortRunLowGrayLevelEmphasis	0.005073527
wavelet-LHH	glrlm	GrayLevelVariance	51.10489159
wavelet-LHH	glrlm	LowGrayLevelRunEmphasis	0.005131295
wavelet-LHH	glrlm	GrayLevelNonUniformityNormalized	0.058019333
wavelet-LHH	glrlm	RunVariance	0.051190899

wavelet-LHH	glrlm	GrayLevelNonUniformity	17.9287822
wavelet-LHH	glrlm	LongRunEmphasis	1.150988502
wavelet-LHH	glrlm	ShortRunHighGrayLevelEmphasis	743.794567
wavelet-LHH	glrlm	RunLengthNonUniformity	281.7045791
wavelet-LHH	glrlm	ShortRunEmphasis	0.964910579
wavelet-LHH	glrlm	LongRunHighGrayLevelEmphasis	868.6775737
wavelet-LHH	glrlm	RunPercentage	0.953703704
wavelet-LHH	glrlm	LongRunLowGrayLevelEmphasis	0.005378539
wavelet-LHH	glrlm	RunEntropy	4.769389348
wavelet-LHH	glrlm	HighGrayLevelRunEmphasis	767.3522819
wavelet-LHH	glrlm	RunLengthNonUniformityNormalized	0.911490991
wavelet-LHH	glszm	GrayLevelVariance	76.23454717
wavelet-LHH	glszm	ZoneVariance	5.56918287
wavelet-LHH	glszm	GrayLevelNonUniformityNormalized	0.041665395
wavelet-LHH	glszm	SizeZoneNonUniformityNormalized	0.569610207
wavelet-LHH	glszm	SizeZoneNonUniformity	103.0994475
wavelet-LHH	glszm	GrayLevelNonUniformity	7.541436464
wavelet-LHH	glszm	LargeAreaEmphasis	8.773480663
wavelet-LHH	glszm	SmallAreaHighGrayLevelEmphasis	700.2720702
wavelet-LHH	glszm	ZonePercentage	0.558641975
wavelet-LHH	glszm	LargeAreaLowGrayLevelEmphasis	0.019605059
wavelet-LHH	glszm	LargeAreaHighGrayLevelEmphasis	6139.033149
wavelet-LHH	glszm	HighGrayLevelZoneEmphasis	839.3370166
wavelet-LHH	glszm	SmallAreaEmphasis	0.781141254
wavelet-LHH	glszm	LowGrayLevelZoneEmphasis	0.007572301
wavelet-LHH	glszm	ZoneEntropy	5.744812036
wavelet-LHH	glszm	SmallAreaLowGrayLevelEmphasis	0.007164751
wavelet-LHH	ngtdm	Coarseness	0.021492714
wavelet-LHH	ngtdm	Complexity	3530.141805
wavelet-LHH	ngtdm	Strength	12.66272183
wavelet-LHH	ngtdm	Contrast	0.296622563
wavelet-LHH	ngtdm	Busyness	0.036385323
wavelet-LLH	gldm	GrayLevelVariance	333.2895424
wavelet-LLH	gldm	HighGrayLevelEmphasis	5716.953704
wavelet-LLH	gldm	DependenceEntropy	6.623358891
wavelet-LLH	gldm	DependenceNonUniformity	123.5308642
wavelet-LLH	gldm	GrayLevelNonUniformity	9.734567901
wavelet-LLH	gldm	SmallDependenceEmphasis	0.626234568
wavelet-LLH	gldm	SmallDependenceHighGrayLevelEmphasis	3601.177906
wavelet-LLH	gldm	DependenceNonUniformityNormalized	0.381268099
wavelet-LLH	gldm	LargeDependenceEmphasis	4.166666667
wavelet-LLH	gldm	LargeDependenceLowGrayLevelEmphasis	0.003963189
wavelet-LLH	gldm	DependenceVariance	1.071673525
wavelet-LLH	gldm	LargeDependenceHighGrayLevelEmphasis	23162.15741
wavelet-LLH	gldm	SmallDependenceLowGrayLevelEmphasis	0.003271409
wavelet-LLH	gldm	LowGrayLevelEmphasis	0.003345376
wavelet-LLH	glcm	JointAverage	72.38303502
wavelet-LLH	glcm	SumAverage	144.76607
wavelet-LLH	glcm	JointEntropy	8.44420892
wavelet-LLH	glcm	ClusterShade	-10548.3719
wavelet-LLH	glcm	MaximumProbability	0.013762481
wavelet-LLH	glcm	Idmn	0.970435438
wavelet-LLH	glcm	JointEnergy	0.003334911
wavelet-LLH	glcm	Contrast	601.9091097
wavelet-LLH	glcm	DifferenceEntropy	5.077834851
wavelet-LLH	glcm	InverseVariance	0.112144041
wavelet-LLH	glcm	DifferenceVariance	332.8314911
wavelet-LLH	glcm	Idn	0.903094076

wavelet-LLH	glcm	Idm	0.109188712
wavelet-LLH	glcm	Correlation	0.047687801
wavelet-LLH	glcm	Autocorrelation	5258.613155
wavelet-LLH	glcm	SumEntropy	6.009221955
wavelet-LLH	glcm	SumSquares	320.2149047
wavelet-LLH	glcm	ClusterProminence	2636262.745
wavelet-LLH	glcm	Imc2	0.998198287
wavelet-LLH	glcm	Imc1	-0.500550445
wavelet-LLH	glcm	DifferenceAverage	15.89507388
wavelet-LLH	glcm	Id	0.180733021
wavelet-LLH	glcm	ClusterTendency	678.9505091
wavelet-LLH	firstorder	InterquartileRange	315.9310026
wavelet-LLH	firstorder	Skewness	-0.275890425
wavelet-LLH	firstorder	Uniformity	0.030044963
wavelet-LLH	firstorder	Median	-21.49115454
wavelet-LLH	firstorder	Energy	67832322.23
wavelet-LLH	firstorder	RobustMeanAbsoluteDeviation	146.0235665
wavelet-LLH	firstorder	MeanAbsoluteDeviation	298.360666
wavelet-LLH	firstorder	TotalEnergy	1831472700
wavelet-LLH	firstorder	Maximum	1391.542257
wavelet-LLH	firstorder	RootMeanSquared	457.5576676
wavelet-LLH	firstorder	90Percentile	463.3241355
wavelet-LLH	firstorder	Minimum	-1849.901037
wavelet-LLH	firstorder	Entropy	5.627369088
wavelet-LLH	firstorder	Range	3241.443293
wavelet-LLH	firstorder	Variance	208568.3529
wavelet-LLH	firstorder	10Percentile	-488.5356326
wavelet-LLH	firstorder	Kurtosis	5.142331477
wavelet-LLH	firstorder	Mean	-28.11879006
wavelet-LLH	glrlm	ShortRunLowGrayLevelEmphasis	0.003436292
wavelet-LLH	glrlm	GrayLevelVariance	340.6873874
wavelet-LLH	glrlm	LowGrayLevelRunEmphasis	0.003440693
wavelet-LLH	glrlm	GrayLevelNonUniformityNormalized	0.02901003
wavelet-LLH	glrlm	RunVariance	0.03015642
wavelet-LLH	glrlm	GrayLevelNonUniformity	9.126126249
wavelet-LLH	glrlm	LongRunEmphasis	1.091805647
wavelet-LLH	glrlm	ShortRunHighGrayLevelEmphasis	5596.846064
wavelet-LLH	glrlm	RunLengthNonUniformity	296.5692305
wavelet-LLH	glrlm	ShortRunEmphasis	0.977603612
wavelet-LLH	glrlm	LongRunHighGrayLevelEmphasis	6232.214867
wavelet-LLH	glrlm	RunPercentage	0.970797721
wavelet-LLH	glrlm	LongRunLowGrayLevelEmphasis	0.003458712
wavelet-LLH	glrlm	RunEntropy	5.778724853
wavelet-LLH	glrlm	HighGrayLevelRunEmphasis	5721.553646
wavelet-LLH	glrlm	RunLengthNonUniformityNormalized	0.94253806
wavelet-LLH	glszm	GrayLevelVariance	429.1626851
wavelet-LLH	glszm	ZoneVariance	1.0729104
wavelet-LLH	glszm	GrayLevelNonUniformityNormalized	0.021211357
wavelet-LLH	glszm	SizeZoneNonUniformityNormalized	0.620369113
wavelet-LLH	glszm	SizeZoneNonUniformity	140.8237885
wavelet-LLH	glszm	GrayLevelNonUniformity	4.814977974
wavelet-LLH	glszm	LargeAreaEmphasis	3.110132159
wavelet-LLH	glszm	SmallAreaHighGrayLevelEmphasis	4689.826175
wavelet-LLH	glszm	ZonePercentage	0.700617284
wavelet-LLH	glszm	LargeAreaLowGrayLevelEmphasis	0.005103539
wavelet-LLH	glszm	LargeAreaHighGrayLevelEmphasis	17457.28194
wavelet-LLH	glszm	HighGrayLevelZoneEmphasis	5760.735683
wavelet-LLH	glszm	SmallAreaEmphasis	0.814643226

wavelet-LLH	glszm	LowGrayLevelZoneEmphasis	0.00468998
wavelet-LLH	glszm	ZoneEntropy	6.573100142
wavelet-LLH	glszm	SmallAreaLowGrayLevelEmphasis	0.004653258
wavelet-LLH	ngtdm	Coarseness	0.018773243
wavelet-LLH	ngtdm	Complexity	35297.78658
wavelet-LLH	ngtdm	Strength	53.9605448
wavelet-LLH	ngtdm	Contrast	1.162208733
wavelet-LLH	ngtdm	Busyness	0.00794937
wavelet-LLL	gldm	GrayLevelVariance	917.8688462
wavelet-LLL	gldm	HighGrayLevelEmphasis	11410.2037
wavelet-LLL	gldm	DependenceEntropy	6.861076238
wavelet-LLL	gldm	DependenceNonUniformity	172.5987654
wavelet-LLL	gldm	GrayLevelNonUniformity	5.827160494
wavelet-LLL	gldm	SmallDependenceEmphasis	0.75654321
wavelet-LLL	gldm	SmallDependenceHighGrayLevelEmphasis	8202.792332
wavelet-LLL	gldm	DependenceNonUniformityNormalized	0.532712239
wavelet-LLL	gldm	LargeDependenceEmphasis	2.685185185
wavelet-LLL	gldm	LargeDependenceLowGrayLevelEmphasis	0.003541178
wavelet-LLL	gldm	DependenceVariance	0.616559976
wavelet-LLL	gldm	LargeDependenceHighGrayLevelEmphasis	34106.49383
wavelet-LLL	gldm	SmallDependenceLowGrayLevelEmphasis	0.003237644
wavelet-LLL	gldm	LowGrayLevelEmphasis	0.003272061
wavelet-LLL	glcm	JointAverage	99.84610951
wavelet-LLL	glcm	SumAverage	199.692219
wavelet-LLL	glcm	JointEntropy	8.68444809
wavelet-LLL	glcm	ClusterShade	-148337.2172
wavelet-LLL	glcm	MaximumProbability	0.010396981
wavelet-LLL	glcm	Idmn	0.972612991
wavelet-LLL	glcm	JointEnergy	0.002628939
wavelet-LLL	glcm	Contrast	843.8871104
wavelet-LLL	glcm	DifferenceEntropy	5.543176817
wavelet-LLL	glcm	InverseVariance	0.073585703
wavelet-LLL	glcm	DifferenceVariance	360.5591278
wavelet-LLL	glcm	Idn	0.894320657
wavelet-LLL	glcm	Idm	0.068544316
wavelet-LLL	glcm	Correlation	0.579718182
wavelet-LLL	glcm	Autocorrelation	10546.56798
wavelet-LLL	glcm	SumEntropy	6.770749264
wavelet-LLL	glcm	SumSquares	998.2298541
wavelet-LLL	glcm	ClusterProminence	29567117.34
wavelet-LLL	glcm	Imc2	0.999796646
wavelet-LLL	glcm	Imc1	-0.620869914
wavelet-LLL	glcm	DifferenceAverage	21.55138863
wavelet-LLL	glcm	Id	0.128618622
wavelet-LLL	glcm	ClusterTendency	3149.032306
wavelet-LLL	firstorder	InterquartileRange	968.6177679
wavelet-LLL	firstorder	Skewness	-1.040199233
wavelet-LLL	firstorder	Uniformity	0.017985063
wavelet-LLL	firstorder	Median	-173.1626398
wavelet-LLL	firstorder	Energy	251848722.3
wavelet-LLL	firstorder	RobustMeanAbsoluteDeviation	411.3022029
wavelet-LLL	firstorder	MeanAbsoluteDeviation	607.9913338
wavelet-LLL	firstorder	TotalEnergy	6799915502
wavelet-LLL	firstorder	Maximum	1157.795535
wavelet-LLL	firstorder	RootMeanSquared	881.6523529
wavelet-LLL	firstorder	90Percentile	214.982701
wavelet-LLL	firstorder	Minimum	-2984.213336
wavelet-LLL	firstorder	Entropy	6.268350098

wavelet-LLL	firstorder	Range	4142.008871
wavelet-LLL	firstorder	Variance	572806.6537
wavelet-LLL	firstorder	10Percentile	-1664.397079
wavelet-LLL	firstorder	Kurtosis	3.49439817
wavelet-LLL	firstorder	Mean	-452.2214254
wavelet-LLL	glrlm	ShortRunLowGrayLevelEmphasis	0.003323858
wavelet-LLL	glrlm	GrayLevelVariance	919.9607713
wavelet-LLL	glrlm	LowGrayLevelRunEmphasis	0.00332583
wavelet-LLL	glrlm	GrayLevelNonUniformityNormalized	0.017529402
wavelet-LLL	glrlm	RunVariance	0.017823043
wavelet-LLL	glrlm	GrayLevelNonUniformity	5.58503291
wavelet-LLL	glrlm	LongRunEmphasis	1.05270897
wavelet-LLL	glrlm	ShortRunHighGrayLevelEmphasis	11209.05912
wavelet-LLL	glrlm	RunLengthNonUniformity	308.1506937
wavelet-LLL	glrlm	ShortRunEmphasis	0.98736543
wavelet-LLL	glrlm	LongRunHighGrayLevelEmphasis	12081.49056
wavelet-LLL	glrlm	RunPercentage	0.9831434
wavelet-LLL	glrlm	LongRunLowGrayLevelEmphasis	0.00333386
wavelet-LLL	glrlm	RunEntropy	6.343454772
wavelet-LLL	glrlm	HighGrayLevelRunEmphasis	11377.02875
wavelet-LLL	glrlm	RunLengthNonUniformityNormalized	0.967209862
wavelet-LLL	glszm	GrayLevelVariance	948.0642218
wavelet-LLL	glszm	ZoneVariance	0.493801653
wavelet-LLL	glszm	GrayLevelNonUniformityNormalized	0.014520202
wavelet-LLL	glszm	SizeZoneNonUniformityNormalized	0.738894628
wavelet-LLL	glszm	SizeZoneNonUniformity	195.0681818
wavelet-LLL	glszm	GrayLevelNonUniformity	3.833333333
wavelet-LLL	glszm	LargeAreaEmphasis	2
wavelet-LLL	glszm	SmallAreaHighGrayLevelEmphasis	9472.349547
wavelet-LLL	glszm	ZonePercentage	0.814814815
wavelet-LLL	glszm	LargeAreaLowGrayLevelEmphasis	0.004136011
wavelet-LLL	glszm	LargeAreaHighGrayLevelEmphasis	24466.31818
wavelet-LLL	glszm	HighGrayLevelZoneEmphasis	11007.24242
wavelet-LLL	glszm	SmallAreaEmphasis	0.882646748
wavelet-LLL	glszm	LowGrayLevelZoneEmphasis	0.003981851
wavelet-LLL	glszm	ZoneEntropy	6.773133041
wavelet-LLL	glszm	SmallAreaLowGrayLevelEmphasis	0.003967092
wavelet-LLL	ngtdm	Coarseness	0.019683455
wavelet-LLL	ngtdm	Complexity	62810.26598
wavelet-LLL	ngtdm	Strength	148.6895684
wavelet-LLL	ngtdm	Contrast	1.767919061
wavelet-LLL	ngtdm	Busyness	0.004168104
wavelet-HHL	gldm	GrayLevelVariance	15.44623533
wavelet-HHL	gldm	HighGrayLevelEmphasis	223.1975309
wavelet-HHL	gldm	DependenceEntropy	5.964639153
wavelet-HHL	gldm	DependenceNonUniformity	61.32098765
wavelet-HHL	gldm	GrayLevelNonUniformity	29.02469136
wavelet-HHL	gldm	SmallDependenceEmphasis	0.372936198
wavelet-HHL	gldm	SmallDependenceHighGrayLevelEmphasis	87.62855491
wavelet-HHL	gldm	DependenceNonUniformityNormalized	0.189262308
wavelet-HHL	gldm	LargeDependenceEmphasis	14.25308642
wavelet-HHL	gldm	LargeDependenceLowGrayLevelEmphasis	0.100242226
wavelet-HHL	gldm	DependenceVariance	5.029492455
wavelet-HHL	gldm	LargeDependenceHighGrayLevelEmphasis	2906.907407
wavelet-HHL	gldm	SmallDependenceLowGrayLevelEmphasis	0.004716249
wavelet-HHL	gldm	LowGrayLevelEmphasis	0.012738132
wavelet-HHL	glcm	JointAverage	14.41327424
wavelet-HHL	glcm	SumAverage	28.82654848

wavelet-HHL	glcm	JointEntropy	7.110852631
wavelet-HHL	glcm	ClusterShade	-7.950913661
wavelet-HHL	glcm	MaximumProbability	0.047170746
wavelet-HHL	glcm	Idmn	0.969230728
wavelet-HHL	glcm	JointEnergy	0.011281978
wavelet-HHL	glcm	Contrast	31.70777054
wavelet-HHL	glcm	DifferenceEntropy	3.487291427
wavelet-HHL	glcm	InverseVariance	0.251486176
wavelet-HHL	glcm	DifferenceVariance	14.72735224
wavelet-HHL	glcm	Idn	0.890182966
wavelet-HHL	glcm	Idm	0.256282008
wavelet-HHL	glcm	Correlation	0.028397546
wavelet-HHL	glcm	Autocorrelation	208.1524951
wavelet-HHL	glcm	SumEntropy	4.38215541
wavelet-HHL	glcm	SumSquares	16.2607969
wavelet-HHL	glcm	ClusterProminence	4952.937069
wavelet-HHL	glcm	Imc2	0.882354552
wavelet-HHL	glcm	Imc1	-0.192626198
wavelet-HHL	glcm	DifferenceAverage	4.083845602
wavelet-HHL	glcm	Id	0.341178727
wavelet-HHL	glcm	ClusterTendency	33.33541707
wavelet-HHL	firstorder	InterquartileRange	97.27460443
wavelet-HHL	firstorder	Skewness	-0.004187898
wavelet-HHL	firstorder	Uniformity	0.089582381
wavelet-HHL	firstorder	Median	-3.798021501
wavelet-HHL	firstorder	Energy	3059505.973
wavelet-HHL	firstorder	RobustMeanAbsoluteDeviation	41.79782706
wavelet-HHL	firstorder	MeanAbsoluteDeviation	69.81814883
wavelet-HHL	firstorder	TotalEnergy	82606661.26
wavelet-HHL	firstorder	Maximum	385.5146349
wavelet-HHL	firstorder	RootMeanSquared	97.17468636
wavelet-HHL	firstorder	90Percentile	114.8968368
wavelet-HHL	firstorder	Minimum	-336.5069334
wavelet-HHL	firstorder	Entropy	3.891753109
wavelet-HHL	firstorder	Range	722.0215683
wavelet-HHL	firstorder	Variance	9440.739905
wavelet-HHL	firstorder	10Percentile	-110.6814874
wavelet-HHL	firstorder	Kurtosis	4.608756314
wavelet-HHL	firstorder	Mean	-1.476402145
wavelet-HHL	glrlm	ShortRunLowGrayLevelEmphasis	0.012590706
wavelet-HHL	glrlm	GrayLevelVariance	16.3489094
wavelet-HHL	glrlm	LowGrayLevelRunEmphasis	0.013102357
wavelet-HHL	glrlm	GrayLevelNonUniformityNormalized	0.083774817
wavelet-HHL	glrlm	RunVariance	0.102355203
wavelet-HHL	glrlm	GrayLevelNonUniformity	25.02627138
wavelet-HHL	glrlm	LongRunEmphasis	1.28180458
wavelet-HHL	glrlm	ShortRunHighGrayLevelEmphasis	212.4794557
wavelet-HHL	glrlm	RunLengthNonUniformity	257.1648121
wavelet-HHL	glrlm	ShortRunEmphasis	0.942310859
wavelet-HHL	glrlm	LongRunHighGrayLevelEmphasis	282.8091763
wavelet-HHL	glrlm	RunPercentage	0.921652422
wavelet-HHL	glrlm	LongRunLowGrayLevelEmphasis	0.015411569
wavelet-HHL	glrlm	RunEntropy	4.29571909
wavelet-HHL	glrlm	HighGrayLevelRunEmphasis	224.5220929
wavelet-HHL	glrlm	RunLengthNonUniformityNormalized	0.860378143
wavelet-HHL	glszm	GrayLevelVariance	26.88213668
wavelet-HHL	glszm	ZoneVariance	27.50086505
wavelet-HHL	glszm	GrayLevelNonUniformityNormalized	0.058282872

wavelet-HHL	glszm	SizeZoneNonUniformityNormalized	0.478265571
wavelet-HHL	glszm	SizeZoneNonUniformity	65.04411765
wavelet-HHL	glszm	GrayLevelNonUniformity	7.926470588
wavelet-HHL	glszm	LargeAreaEmphasis	33.17647059
wavelet-HHL	glszm	SmallAreaHighGrayLevelEmphasis	171.2796732
wavelet-HHL	glszm	ZonePercentage	0.419753086
wavelet-HHL	glszm	LargeAreaLowGrayLevelEmphasis	0.204209241
wavelet-HHL	glszm	LargeAreaHighGrayLevelEmphasis	6634.735294
wavelet-HHL	glszm	HighGrayLevelZoneEmphasis	236.7058824
wavelet-HHL	glszm	SmallAreaEmphasis	0.718793025
wavelet-HHL	glszm	LowGrayLevelZoneEmphasis	0.015610358
wavelet-HHL	glszm	ZoneEntropy	5.453615927
wavelet-HHL	glszm	SmallAreaLowGrayLevelEmphasis	0.008447192
wavelet-HHL	ngtdm	Coarseness	0.021535133
wavelet-HHL	ngtdm	Complexity	755.14006
wavelet-HHL	ngtdm	Strength	4.207460129
wavelet-HHL	ngtdm	Contrast	0.137686902
wavelet-HHL	ngtdm	Busyness	0.104897108
wavelet-HLH	gldm	GrayLevelVariance	44.612902
wavelet-HLH	gldm	HighGrayLevelEmphasis	510.5864198
wavelet-HLH	gldm	DependenceEntropy	6.110067714
wavelet-HLH	gldm	DependenceNonUniformity	81.85185185
wavelet-HLH	gldm	GrayLevelNonUniformity	21.32098765
wavelet-HLH	gldm	SmallDependenceEmphasis	0.475413408
wavelet-HLH	gldm	SmallDependenceHighGrayLevelEmphasis	267.2604012
wavelet-HLH	gldm	DependenceNonUniformityNormalized	0.252629172
wavelet-HLH	gldm	LargeDependenceEmphasis	8.672839506
wavelet-HLH	gldm	LargeDependenceLowGrayLevelEmphasis	0.024478038
wavelet-HLH	gldm	DependenceVariance	2.847469898
wavelet-HLH	gldm	LargeDependenceHighGrayLevelEmphasis	4026.703704
wavelet-HLH	gldm	SmallDependenceLowGrayLevelEmphasis	0.00538243
wavelet-HLH	gldm	LowGrayLevelEmphasis	0.006695016
wavelet-HLH	glcm	JointAverage	21.6936217
wavelet-HLH	glcm	SumAverage	43.38724341
wavelet-HLH	glcm	JointEntropy	7.678008203
wavelet-HLH	glcm	ClusterShade	623.4882339
wavelet-HLH	glcm	MaximumProbability	0.030393407
wavelet-HLH	glcm	Idmn	0.977856047
wavelet-HLH	glcm	JointEnergy	0.006921643
wavelet-HLH	glcm	Contrast	92.74762614
wavelet-HLH	glcm	DifferenceEntropy	4.078915849
wavelet-HLH	glcm	InverseVariance	0.190221429
wavelet-HLH	glcm	DifferenceVariance	48.68932889
wavelet-HLH	glcm	Idn	0.909636021
wavelet-HLH	glcm	Idm	0.188344537
wavelet-HLH	glcm	Correlation	-0.017978134
wavelet-HLH	glcm	Autocorrelation	470.2262313
wavelet-HLH	glcm	SumEntropy	4.917883707
wavelet-HLH	glcm	SumSquares	45.97516706
wavelet-HLH	glcm	ClusterProminence	63637.5513
wavelet-HLH	glcm	Imc2	0.954629323
wavelet-HLH	glcm	Imc1	-0.273452129
wavelet-HLH	glcm	DifferenceAverage	6.573151062
wavelet-HLH	glcm	Id	0.272583432
wavelet-HLH	glcm	ClusterTendency	91.15304209
wavelet-HLH	firstorder	InterquartileRange	135.8823141
wavelet-HLH	firstorder	Skewness	1.068862674
wavelet-HLH	firstorder	Uniformity	0.065805517

wavelet-HLH	firstorder	Median	-6.511149945
wavelet-HLH	firstorder	Energy	8995787.577
wavelet-HLH	firstorder	RobustMeanAbsoluteDeviation	60.02441621
wavelet-HLH	firstorder	MeanAbsoluteDeviation	110.2016928
wavelet-HLH	firstorder	TotalEnergy	242886264.6
wavelet-HLH	firstorder	Maximum	960.7901698
wavelet-HLH	firstorder	RootMeanSquared	166.6276582
wavelet-HLH	firstorder	90Percentile	178.0860677
wavelet-HLH	firstorder	Minimum	-516.882979
wavelet-HLH	firstorder	Entropy	4.413567045
wavelet-HLH	firstorder	Range	1477.673149
wavelet-HLH	firstorder	Variance	27760.97047
wavelet-HLH	firstorder	10Percentile	-169.2645117
wavelet-HLH	firstorder	Kurtosis	9.306002268
wavelet-HLH	firstorder	Mean	1.950897419
wavelet-HLH	glrlm	ShortRunLowGrayLevelEmphasis	0.006848069
wavelet-HLH	glrlm	GrayLevelVariance	46.76714943
wavelet-HLH	glrlm	LowGrayLevelRunEmphasis	0.006945189
wavelet-HLH	glrlm	GrayLevelNonUniformityNormalized	0.062074747
wavelet-HLH	glrlm	RunVariance	0.06536868
wavelet-HLH	glrlm	GrayLevelNonUniformity	19.02679773
wavelet-HLH	glrlm	LongRunEmphasis	1.185151567
wavelet-HLH	glrlm	ShortRunHighGrayLevelEmphasis	494.7315261
wavelet-HLH	glrlm	RunLengthNonUniformity	275.6230505
wavelet-HLH	glrlm	ShortRunEmphasis	0.959278009
wavelet-HLH	glrlm	LongRunHighGrayLevelEmphasis	598.6237082
wavelet-HLH	glrlm	RunPercentage	0.945631529
wavelet-HLH	glrlm	LongRunLowGrayLevelEmphasis	0.007384505
wavelet-HLH	glrlm	RunEntropy	4.709643493
wavelet-HLH	glrlm	HighGrayLevelRunEmphasis	513.5004706
wavelet-HLH	glrlm	RunLengthNonUniformityNormalized	0.898881082
wavelet-HLH	glszm	GrayLevelVariance	74.33310213
wavelet-HLH	glszm	ZoneVariance	8.854538248
wavelet-HLH	glszm	GrayLevelNonUniformityNormalized	0.041088651
wavelet-HLH	glszm	SizeZoneNonUniformityNormalized	0.533557934
wavelet-HLH	glszm	SizeZoneNonUniformity	92.83908046
wavelet-HLH	glszm	GrayLevelNonUniformity	7.149425287
wavelet-HLH	glszm	LargeAreaEmphasis	12.32183908
wavelet-HLH	glszm	SmallAreaHighGrayLevelEmphasis	438.3388701
wavelet-HLH	glszm	ZonePercentage	0.537037037
wavelet-HLH	glszm	LargeAreaLowGrayLevelEmphasis	0.036538171
wavelet-HLH	glszm	LargeAreaHighGrayLevelEmphasis	5643.931034
wavelet-HLH	glszm	HighGrayLevelZoneEmphasis	552.5344828
wavelet-HLH	glszm	SmallAreaEmphasis	0.757849219
wavelet-HLH	glszm	LowGrayLevelZoneEmphasis	0.010353524
wavelet-HLH	glszm	ZoneEntropy	5.839214583
wavelet-HLH	glszm	SmallAreaLowGrayLevelEmphasis	0.009693685
wavelet-HLH	ngtdm	Coarseness	0.022097347
wavelet-HLH	ngtdm	Complexity	2607.647552
wavelet-HLH	ngtdm	Strength	11.39497455
wavelet-HLH	ngtdm	Contrast	0.32288717
wavelet-HLH	ngtdm	Busyness	0.051622329

Image type	Feature Class	Feature Name	15: RTSTRUCT: C99-DNU_segment_Tumor(4D)
general	info	BoundingBox	(0, 0, 2, 8, 7, 8)
general	info	EnabledImageTypes	{u'Original': {}, u'Wavelet': {}}
general	info	GeneralSettings	{'distances': [1], 'additionalInfo': True, 'force2D': False, 'interpolator': 'sitkBSpline', 'resampledPixelSpacing': [3.0], 'label': 1, 'normalizeScale': 1, 'normalize': False, 'force2Ddimension': 0, 'removeOutliers': None, 'minimumROISize': None, u'binWidth': 25.0, 'minimumROIDimensions': 1, u'correctMask': True, 'preCrop': False, u'symmetricalGLCM': True, 'resegmentRange': None, 'padDistance': 5}
general	info	ImageHash	c3cb87f6b106b5033f8e97f85cc4a4f252836c72
general	info	ImageSpacing	(0.9765625, 0.9765625, 1.9999999999999999)
general	info	MaskHash	fa20b17072dee1e33ce49f6d12dcda21474d0328
general	info	NumpyVersion	1.13.1
general	info	PyWaveletVersion	0.5.2
general	info	SimpleITKVersion	1.1.0
general	info	Version	1.3.0.post102+g1b0eb74
general	info	VolumeNum	1
general	info	VoxelNum	222
original	shape	Maximum3DDiameter	25.98076211
original	shape	Maximum2DDiameterSlice	21.84032967
original	shape	Sphericity	0.874322917
original	shape	MinorAxis	20.92364996
original	shape	Elongation	0.926712664
original	shape	SurfaceVolumeRatio	0.304490221
original	shape	Volume	5994
original	shape	MajorAxis	22.57835764
original	shape	SurfaceArea	1825.114386
original	shape	Flatness	0.833187148
original	shape	LeastAxis	18.8119974
original	shape	Maximum2DDiameterColumn	25.8069758
original	shape	Maximum2DDiameterRow	24.18677324
original	gldm	GrayLevelVariance	98.78242432
original	gldm	HighGrayLevelEmphasis	753.6351351
original	gldm	DependenceEntropy	6.415468108
original	gldm	DependenceNonUniformity	47.78378378
original	gldm	GrayLevelNonUniformity	9.342342342
original	gldm	SmallDependenceEmphasis	0.42435885
original	gldm	SmallDependenceHighGrayLevelEmphasis	230.167591
original	gldm	DependenceNonUniformityNormalized	0.215242269
original	gldm	LargeDependenceEmphasis	12.67567568
original	gldm	LargeDependenceLowGrayLevelEmphasis	0.042044056
original	gldm	DependenceVariance	4.724291859
original	gldm	LargeDependenceHighGrayLevelEmphasis	14207.01351
original	gldm	SmallDependenceLowGrayLevelEmphasis	0.007545765
original	gldm	LowGrayLevelEmphasis	0.011433816
original	glcm	JointAverage	26.99622884
original	glcm	SumAverage	53.99245768
original	glcm	JointEntropy	7.439898857
original	glcm	ClusterShade	-3437.567762
original	glcm	MaximumProbability	0.037782821
original	glcm	Idmn	0.97520037
original	glcm	JointEnergy	0.008020887

original	glcm	Contrast	45.3193906
original	glcm	DifferenceEntropy	3.703007686
original	glcm	InverseVariance	0.207679563
original	glcm	DifferenceVariance	18.43511239
original	glcm	Idn	0.897475945
original	glcm	Idm	0.222242052
original	glcm	Correlation	0.727207965
original	glcm	Autocorrelation	790.3860205
original	glcm	SumEntropy	5.452435374
original	glcm	SumSquares	84.08762508
original	glcm	ClusterProminence	221756.2277
original	glcm	Imc2	0.992878392
original	glcm	Imc1	-0.44695808
original	glcm	DifferenceAverage	5.074250793
original	glcm	Id	0.306233358
original	glcm	ClusterTendency	291.0311097
original	firstorder	InterquartileRange	422
original	firstorder	Skewness	-0.599940858
original	firstorder	Uniformity	0.042123204
original	firstorder	Median	-146.5
original	firstorder	Energy	22396285
original	firstorder	RobustMeanAbsoluteDeviation	167.805656
original	firstorder	MeanAbsoluteDeviation	213.4726889
original	firstorder	TotalEnergy	604699695
original	firstorder	Maximum	175
original	firstorder	RootMeanSquared	317.6226797
original	firstorder	90Percentile	68.9
original	firstorder	Minimum	-804
original	firstorder	Entropy	4.907365383
original	firstorder	Range	979
original	firstorder	Variance	61753.26769
original	firstorder	10Percentile	-548.3
original	firstorder	Kurtosis	2.251906822
original	firstorder	Mean	-197.8153153
original	glrlm	ShortRunLowGrayLevelEmphasis	0.011753957
original	glrlm	GrayLevelVariance	98.90628055
original	glrlm	LowGrayLevelRunEmphasis	0.011989869
original	glrlm	GrayLevelNonUniformityNormalized	0.038754927
original	glrlm	RunVariance	0.099138436
original	glrlm	GrayLevelNonUniformity	8.013949263
original	glrlm	LongRunEmphasis	1.260675359
original	glrlm	ShortRunHighGrayLevelEmphasis	676.6350705
original	glrlm	RunLengthNonUniformity	181.4054412
original	glrlm	ShortRunEmphasis	0.949121224
original	glrlm	LongRunHighGrayLevelEmphasis	1008.958159
original	glrlm	RunPercentage	0.93000693
original	glrlm	LongRunLowGrayLevelEmphasis	0.012989253
original	glrlm	RunEntropy	5.208233813
original	glrlm	HighGrayLevelRunEmphasis	729.9758971
original	glrlm	RunLengthNonUniformityNormalized	0.876708075
original	glszm	GrayLevelVariance	94.53386488
original	glszm	ZoneVariance	9.274691358
original	glszm	GrayLevelNonUniformityNormalized	0.032578875
original	glszm	SizeZoneNonUniformityNormalized	0.50154321
original	glszm	SizeZoneNonUniformity	54.16666667
original	glszm	GrayLevelNonUniformity	3.518518519

original	glszm	LargeAreaEmphasis	13.5
original	glszm	SmallAreaHighGrayLevelEmphasis	365.3972166
original	glszm	ZonePercentage	0.486486486
original	glszm	LargeAreaLowGrayLevelEmphasis	0.046009755
original	glszm	LargeAreaHighGrayLevelEmphasis	14578.89815
original	glszm	HighGrayLevelZoneEmphasis	549.25
original	glszm	SmallAreaEmphasis	0.735262036
original	glszm	LowGrayLevelZoneEmphasis	0.017638771
original	glszm	ZoneEntropy	5.81277078
original	glszm	SmallAreaLowGrayLevelEmphasis	0.014457064
original	ngtdm	Coarseness	0.04793935
original	ngtdm	Complexity	1795.950707
original	ngtdm	Strength	30.79255371
original	ngtdm	Contrast	0.382087061
original	ngtdm	Busyness	0.018290602
wavelet-HHH	gldm	GrayLevelVariance	1.177420664
wavelet-HHH	gldm	HighGrayLevelEmphasis	31.18018018
wavelet-HHH	gldm	DependenceEntropy	5.061630627
wavelet-HHH	gldm	DependenceNonUniformity	18.54054054
wavelet-HHH	gldm	GrayLevelNonUniformity	63.8018018
wavelet-HHH	gldm	SmallDependenceEmphasis	0.137887194
wavelet-HHH	gldm	SmallDependenceHighGrayLevelEmphasis	4.68941791
wavelet-HHH	gldm	DependenceNonUniformityNormalized	0.083515948
wavelet-HHH	gldm	LargeDependenceEmphasis	52.42342342
wavelet-HHH	gldm	LargeDependenceLowGrayLevelEmphasis	1.791562546
wavelet-HHH	gldm	DependenceVariance	11.96923951
wavelet-HHH	gldm	LargeDependenceHighGrayLevelEmphasis	1609.711712
wavelet-HHH	gldm	SmallDependenceLowGrayLevelEmphasis	0.011613937
wavelet-HHH	gldm	LowGrayLevelEmphasis	0.042148591
wavelet-HHH	glcm	JointAverage	5.486782318
wavelet-HHH	glcm	SumAverage	10.97356464
wavelet-HHH	glcm	JointEntropy	4.00570409
wavelet-HHH	glcm	ClusterShade	-0.245903856
wavelet-HHH	glcm	MaximumProbability	0.160713594
wavelet-HHH	glcm	Idmn	0.97450311
wavelet-HHH	glcm	JointEnergy	0.092125189
wavelet-HHH	glcm	Contrast	2.284160235
wavelet-HHH	glcm	DifferenceEntropy	1.885033555
wavelet-HHH	glcm	InverseVariance	0.486209168
wavelet-HHH	glcm	DifferenceVariance	1.048249994
wavelet-HHH	glcm	Idn	0.899004674
wavelet-HHH	glcm	Idm	0.555773966
wavelet-HHH	glcm	Correlation	-0.031827392
wavelet-HHH	glcm	Autocorrelation	30.07233106
wavelet-HHH	glcm	SumEntropy	2.532490545
wavelet-HHH	glcm	SumSquares	1.109357301
wavelet-HHH	glcm	ClusterProminence	17.10377356
wavelet-HHH	glcm	Imc2	0.476944175
wavelet-HHH	glcm	Imc1	-0.064556007
wavelet-HHH	glcm	DifferenceAverage	1.106218555
wavelet-HHH	glcm	Id	0.593175238
wavelet-HHH	glcm	ClusterTendency	2.153268968
wavelet-HHH	firstorder	InterquartileRange	28.21414791
wavelet-HHH	firstorder	Skewness	-0.178513894
wavelet-HHH	firstorder	Uniformity	0.287395504
wavelet-HHH	firstorder	Median	-0.311042725

wavelet-HHH	firstorder	Energy	140474.9829
wavelet-HHH	firstorder	RobustMeanAbsoluteDeviation	11.83191053
wavelet-HHH	firstorder	MeanAbsoluteDeviation	18.64157779
wavelet-HHH	firstorder	TotalEnergy	3792824.537
wavelet-HHH	firstorder	Maximum	86.18951228
wavelet-HHH	firstorder	RootMeanSquared	25.15492383
wavelet-HHH	firstorder	90Percentile	29.4295541
wavelet-HHH	firstorder	Minimum	-107.1060294
wavelet-HHH	firstorder	Entropy	2.114209632
wavelet-HHH	firstorder	Range	193.2955417
wavelet-HHH	firstorder	Variance	632.5775375
wavelet-HHH	firstorder	10Percentile	-28.867702
wavelet-HHH	firstorder	Kurtosis	4.980662758
wavelet-HHH	firstorder	Mean	-0.438925389
wavelet-HHH	glrlm	ShortRunLowGrayLevelEmphasis	0.038580389
wavelet-HHH	glrlm	GrayLevelVariance	1.380378831
wavelet-HHH	glrlm	LowGrayLevelRunEmphasis	0.044208423
wavelet-HHH	glrlm	GrayLevelNonUniformityNormalized	0.260485959
wavelet-HHH	glrlm	RunVariance	0.319308443
wavelet-HHH	glrlm	GrayLevelNonUniformity	45.95500988
wavelet-HHH	glrlm	LongRunEmphasis	1.922898972
wavelet-HHH	glrlm	ShortRunHighGrayLevelEmphasis	26.25684782
wavelet-HHH	glrlm	RunLengthNonUniformity	116.470818
wavelet-HHH	glrlm	ShortRunEmphasis	0.836414383
wavelet-HHH	glrlm	LongRunHighGrayLevelEmphasis	59.59031563
wavelet-HHH	glrlm	RunPercentage	0.793832294
wavelet-HHH	glrlm	LongRunLowGrayLevelEmphasis	0.075902007
wavelet-HHH	glrlm	RunEntropy	3.065701199
wavelet-HHH	glrlm	HighGrayLevelRunEmphasis	31.29710922
wavelet-HHH	glrlm	RunLengthNonUniformityNormalized	0.657414947
wavelet-HHH	glszm	GrayLevelVariance	4.888888889
wavelet-HHH	glszm	ZoneVariance	391.5066667
wavelet-HHH	glszm	GrayLevelNonUniformityNormalized	0.18
wavelet-HHH	glszm	SizeZoneNonUniformityNormalized	0.504444444
wavelet-HHH	glszm	SizeZoneNonUniformity	15.13333333
wavelet-HHH	glszm	GrayLevelNonUniformity	5.4
wavelet-HHH	glszm	LargeAreaEmphasis	446.2666667
wavelet-HHH	glszm	SmallAreaHighGrayLevelEmphasis	26.04856048
wavelet-HHH	glszm	ZonePercentage	0.135135135
wavelet-HHH	glszm	LargeAreaLowGrayLevelEmphasis	15.24318302
wavelet-HHH	glszm	LargeAreaHighGrayLevelEmphasis	13654.6
wavelet-HHH	glszm	HighGrayLevelZoneEmphasis	33.33333333
wavelet-HHH	glszm	SmallAreaEmphasis	0.725183509
wavelet-HHH	glszm	LowGrayLevelZoneEmphasis	0.086779394
wavelet-HHH	glszm	ZoneEntropy	3.574418314
wavelet-HHH	glszm	SmallAreaLowGrayLevelEmphasis	0.073960976
wavelet-HHH	ngtdm	Coarseness	0.026017622
wavelet-HHH	ngtdm	Complexity	29.35523324
wavelet-HHH	ngtdm	Strength	0.743623485
wavelet-HHH	ngtdm	Contrast	0.028785953
wavelet-HHH	ngtdm	Busyness	0.609477026
wavelet-HLL	gldm	GrayLevelVariance	13.07426345
wavelet-HLL	gldm	HighGrayLevelEmphasis	102.045045
wavelet-HLL	gldm	DependenceEntropy	5.841814183
wavelet-HLL	gldm	DependenceNonUniformity	35.90990991
wavelet-HLL	gldm	GrayLevelNonUniformity	17.78378378

wavelet-HLL	gldm	SmallDependenceEmphasis	0.213460849
wavelet-HLL	gldm	SmallDependenceHighGrayLevelEmphasis	24.48917668
wavelet-HLL	gldm	DependenceNonUniformityNormalized	0.161756351
wavelet-HLL	gldm	LargeDependenceEmphasis	16.53153153
wavelet-HLL	gldm	LargeDependenceLowGrayLevelEmphasis	0.379693803
wavelet-HLL	gldm	DependenceVariance	3.610421232
wavelet-HLL	gldm	LargeDependenceHighGrayLevelEmphasis	1608.45045
wavelet-HLL	gldm	SmallDependenceLowGrayLevelEmphasis	0.006633766
wavelet-HLL	gldm	LowGrayLevelEmphasis	0.029313566
wavelet-HLL	glcm	JointAverage	9.05626698
wavelet-HLL	glcm	SumAverage	18.11253396
wavelet-HLL	glcm	JointEntropy	6.564810477
wavelet-HLL	glcm	ClusterShade	34.88091428
wavelet-HLL	glcm	MaximumProbability	0.033309027
wavelet-HLL	glcm	Idmn	0.967994053
wavelet-HLL	glcm	JointEnergy	0.012979407
wavelet-HLL	glcm	Contrast	11.71215593
wavelet-HLL	glcm	DifferenceEntropy	2.805356044
wavelet-HLL	glcm	InverseVariance	0.320857245
wavelet-HLL	glcm	DifferenceVariance	4.548167859
wavelet-HLL	glcm	Idn	0.883524872
wavelet-HLL	glcm	Idm	0.326352656
wavelet-HLL	glcm	Correlation	0.518948697
wavelet-HLL	glcm	Autocorrelation	88.36315767
wavelet-HLL	glcm	SumEntropy	4.44890672
wavelet-HLL	glcm	SumSquares	12.19500043
wavelet-HLL	glcm	ClusterProminence	3170.750203
wavelet-HLL	glcm	Imc2	0.885217137
wavelet-HLL	glcm	Imc1	-0.220165751
wavelet-HLL	glcm	DifferenceAverage	2.589372077
wavelet-HLL	glcm	Id	0.408361154
wavelet-HLL	glcm	ClusterTendency	37.0678458
wavelet-HLL	firstorder	InterquartileRange	142.2961679
wavelet-HLL	firstorder	Skewness	0.002563812
wavelet-HLL	firstorder	Uniformity	0.080107134
wavelet-HLL	firstorder	Median	-2.764589281
wavelet-HLL	firstorder	Energy	1822197.332
wavelet-HLL	firstorder	RobustMeanAbsoluteDeviation	60.24665553
wavelet-HLL	firstorder	MeanAbsoluteDeviation	77.59431648
wavelet-HLL	firstorder	TotalEnergy	49199327.95
wavelet-HLL	firstorder	Maximum	210.5216503
wavelet-HLL	firstorder	RootMeanSquared	90.59854352
wavelet-HLL	firstorder	90Percentile	118.3208846
wavelet-HLL	firstorder	Minimum	-221.7732082
wavelet-HLL	firstorder	Entropy	3.762434174
wavelet-HLL	firstorder	Range	432.2948585
wavelet-HLL	firstorder	Variance	8202.60386
wavelet-HLL	firstorder	10Percentile	-118.0333729
wavelet-HLL	firstorder	Kurtosis	2.124551553
wavelet-HLL	firstorder	Mean	-2.343550229
wavelet-HLL	glrlm	ShortRunLowGrayLevelEmphasis	0.027889242
wavelet-HLL	glrlm	GrayLevelVariance	13.02750702
wavelet-HLL	glrlm	LowGrayLevelRunEmphasis	0.029851593
wavelet-HLL	glrlm	GrayLevelNonUniformityNormalized	0.079295866
wavelet-HLL	glrlm	RunVariance	0.123458588
wavelet-HLL	glrlm	GrayLevelNonUniformity	15.84719695

wavelet-HLL	glrlm	LongRunEmphasis	1.368514799
wavelet-HLL	glrlm	ShortRunHighGrayLevelEmphasis	95.20829436
wavelet-HLL	glrlm	RunLengthNonUniformity	164.3290647
wavelet-HLL	glrlm	ShortRunEmphasis	0.921814929
wavelet-HLL	glrlm	LongRunHighGrayLevelEmphasis	138.2590004
wavelet-HLL	glrlm	RunPercentage	0.9002079
wavelet-HLL	glrlm	LongRunLowGrayLevelEmphasis	0.038770209
wavelet-HLL	glrlm	RunEntropy	4.189384711
wavelet-HLL	glrlm	HighGrayLevelRunEmphasis	102.719354
wavelet-HLL	glrlm	RunLengthNonUniformityNormalized	0.818384037
wavelet-HLL	glszm	GrayLevelVariance	14.76968557
wavelet-HLL	glszm	ZoneVariance	18.78796023
wavelet-HLL	glszm	GrayLevelNonUniformityNormalized	0.081429723
wavelet-HLL	glszm	SizeZoneNonUniformityNormalized	0.236764311
wavelet-HLL	glszm	SizeZoneNonUniformity	14.44262295
wavelet-HLL	glszm	GrayLevelNonUniformity	4.967213115
wavelet-HLL	glszm	LargeAreaEmphasis	32.03278689
wavelet-HLL	glszm	SmallAreaHighGrayLevelEmphasis	59.83303498
wavelet-HLL	glszm	ZonePercentage	0.274774775
wavelet-HLL	glszm	LargeAreaLowGrayLevelEmphasis	0.779631999
wavelet-HLL	glszm	LargeAreaHighGrayLevelEmphasis	2717.442623
wavelet-HLL	glszm	HighGrayLevelZoneEmphasis	117.4098361
wavelet-HLL	glszm	SmallAreaEmphasis	0.490761405
wavelet-HLL	glszm	LowGrayLevelZoneEmphasis	0.036253361
wavelet-HLL	glszm	ZoneEntropy	5.191074789
wavelet-HLL	glszm	SmallAreaLowGrayLevelEmphasis	0.014071789
wavelet-HLL	ngtdm	Coarseness	0.036436583
wavelet-HLL	ngtdm	Complexity	209.4770593
wavelet-HLL	ngtdm	Strength	3.966560947
wavelet-HLL	ngtdm	Contrast	0.139724408
wavelet-HLL	ngtdm	Busyness	0.184808837
wavelet-LHL	gldm	GrayLevelVariance	38.96771772
wavelet-LHL	gldm	HighGrayLevelEmphasis	406.3288288
wavelet-LHL	gldm	DependenceEntropy	6.092116344
wavelet-LHL	gldm	DependenceNonUniformity	44.34234234
wavelet-LHL	gldm	GrayLevelNonUniformity	15.14414414
wavelet-LHL	gldm	SmallDependenceEmphasis	0.387160968
wavelet-LHL	gldm	SmallDependenceHighGrayLevelEmphasis	181.0276627
wavelet-LHL	gldm	DependenceNonUniformityNormalized	0.199740281
wavelet-LHL	gldm	LargeDependenceEmphasis	11.69369369
wavelet-LHL	gldm	LargeDependenceLowGrayLevelEmphasis	0.043363862
wavelet-LHL	gldm	DependenceVariance	3.691421151
wavelet-LHL	gldm	LargeDependenceHighGrayLevelEmphasis	4165.463964
wavelet-LHL	gldm	SmallDependenceLowGrayLevelEmphasis	0.006082472
wavelet-LHL	gldm	LowGrayLevelEmphasis	0.008404514
wavelet-LHL	glcm	JointAverage	18.91560091
wavelet-LHL	glcm	SumAverage	37.83120181
wavelet-LHL	glcm	JointEntropy	7.113020946
wavelet-LHL	glcm	ClusterShade	1091.07432
wavelet-LHL	glcm	MaximumProbability	0.028956708
wavelet-LHL	glcm	Idmn	0.977064584
wavelet-LHL	glcm	JointEnergy	0.00949382
wavelet-LHL	glcm	Contrast	45.19519936
wavelet-LHL	glcm	DifferenceEntropy	3.610210389
wavelet-LHL	glcm	InverseVariance	0.233981168
wavelet-LHL	glcm	DifferenceVariance	21.62323101

wavelet-LHL	glcm	Idn	0.907109425
wavelet-LHL	glcm	Idm	0.238341021
wavelet-LHL	glcm	Correlation	0.318795822
wavelet-LHL	glcm	Autocorrelation	369.6833567
wavelet-LHL	glcm	SumEntropy	4.781178278
wavelet-LHL	glcm	SumSquares	34.44348713
wavelet-LHL	glcm	ClusterProminence	51295.4187
wavelet-LHL	glcm	Imc2	0.963775914
wavelet-LHL	glcm	Imc1	-0.316776367
wavelet-LHL	glcm	DifferenceAverage	4.725550765
wavelet-LHL	glcm	Id	0.323593079
wavelet-LHL	glcm	ClusterTendency	92.57874916
wavelet-LHL	firstorder	InterquartileRange	125.4847156
wavelet-LHL	firstorder	Skewness	0.964340419
wavelet-LHL	firstorder	Uniformity	0.068216866
wavelet-LHL	firstorder	Median	-20.59212745
wavelet-LHL	firstorder	Energy	5489614.944
wavelet-LHL	firstorder	RobustMeanAbsoluteDeviation	57.51270296
wavelet-LHL	firstorder	MeanAbsoluteDeviation	106.8407261
wavelet-LHL	firstorder	TotalEnergy	148219603.5
wavelet-LHL	firstorder	Maximum	571.9096872
wavelet-LHL	firstorder	RootMeanSquared	157.251376
wavelet-LHL	firstorder	90Percentile	202.578672
wavelet-LHL	firstorder	Minimum	-452.9873202
wavelet-LHL	firstorder	Entropy	4.326594087
wavelet-LHL	firstorder	Range	1024.897007
wavelet-LHL	firstorder	Variance	24652.01017
wavelet-LHL	firstorder	10Percentile	-168.172711
wavelet-LHL	firstorder	Kurtosis	5.450647355
wavelet-LHL	firstorder	Mean	-8.716941566
wavelet-LHL	glrlm	ShortRunLowGrayLevelEmphasis	0.008599744
wavelet-LHL	glrlm	GrayLevelVariance	40.5517056
wavelet-LHL	glrlm	LowGrayLevelRunEmphasis	0.008784988
wavelet-LHL	glrlm	GrayLevelNonUniformityNormalized	0.064694809
wavelet-LHL	glrlm	RunVariance	0.08804203
wavelet-LHL	glrlm	GrayLevelNonUniformity	13.35791392
wavelet-LHL	glrlm	LongRunEmphasis	1.248940397
wavelet-LHL	glrlm	ShortRunHighGrayLevelEmphasis	390.9838575
wavelet-LHL	glrlm	RunLengthNonUniformity	180.2366402
wavelet-LHL	glrlm	ShortRunEmphasis	0.946986306
wavelet-LHL	glrlm	LongRunHighGrayLevelEmphasis	498.8583419
wavelet-LHL	glrlm	RunPercentage	0.92966043
wavelet-LHL	glrlm	LongRunLowGrayLevelEmphasis	0.009635709
wavelet-LHL	glrlm	RunEntropy	4.662786073
wavelet-LHL	glrlm	HighGrayLevelRunEmphasis	410.0432601
wavelet-LHL	glrlm	RunLengthNonUniformityNormalized	0.871630031
wavelet-LHL	glszm	GrayLevelVariance	58.9043107
wavelet-LHL	glszm	ZoneVariance	11.95002082
wavelet-LHL	glszm	GrayLevelNonUniformityNormalized	0.042898792
wavelet-LHL	glszm	SizeZoneNonUniformityNormalized	0.475426905
wavelet-LHL	glszm	SizeZoneNonUniformity	46.59183673
wavelet-LHL	glszm	GrayLevelNonUniformity	4.204081633
wavelet-LHL	glszm	LargeAreaEmphasis	17.08163265
wavelet-LHL	glszm	SmallAreaHighGrayLevelEmphasis	352.6961647
wavelet-LHL	glszm	ZonePercentage	0.441441441
wavelet-LHL	glszm	LargeAreaLowGrayLevelEmphasis	0.067528937

wavelet-LHL	glszm	LargeAreaHighGrayLevelEmphasis	5854.214286
wavelet-LHL	glszm	HighGrayLevelZoneEmphasis	469.1734694
wavelet-LHL	glszm	SmallAreaEmphasis	0.716127439
wavelet-LHL	glszm	LowGrayLevelZoneEmphasis	0.014374707
wavelet-LHL	glszm	ZoneEntropy	5.518526967
wavelet-LHL	glszm	SmallAreaLowGrayLevelEmphasis	0.013114272
wavelet-LHL	ngtdm	Coarseness	0.031598439
wavelet-LHL	ngtdm	Complexity	1448.240866
wavelet-LHL	ngtdm	Strength	13.54608869
wavelet-LHL	ngtdm	Contrast	0.241695457
wavelet-LHL	ngtdm	Busyness	0.047601927
wavelet-LHH	gldm	GrayLevelVariance	7.793685577
wavelet-LHH	gldm	HighGrayLevelEmphasis	119.6576577
wavelet-LHH	gldm	DependenceEntropy	5.566816964
wavelet-LHH	gldm	DependenceNonUniformity	29.43243243
wavelet-LHH	gldm	GrayLevelNonUniformity	30.81981982
wavelet-LHH	gldm	SmallDependenceEmphasis	0.270796813
wavelet-LHH	gldm	SmallDependenceHighGrayLevelEmphasis	36.093039
wavelet-LHH	gldm	DependenceNonUniformityNormalized	0.132578524
wavelet-LHH	gldm	LargeDependenceEmphasis	23.28828829
wavelet-LHH	gldm	LargeDependenceLowGrayLevelEmphasis	0.265333142
wavelet-LHH	gldm	DependenceVariance	7.503774044
wavelet-LHH	gldm	LargeDependenceHighGrayLevelEmphasis	2519.36036
wavelet-LHH	gldm	SmallDependenceLowGrayLevelEmphasis	0.006298174
wavelet-LHH	gldm	LowGrayLevelEmphasis	0.020475422
wavelet-LHH	glcm	JointAverage	10.58120129
wavelet-LHH	glcm	SumAverage	21.16240257
wavelet-LHH	glcm	JointEntropy	5.864586533
wavelet-LHH	glcm	ClusterShade	-2.711743915
wavelet-LHH	glcm	MaximumProbability	0.080668539
wavelet-LHH	glcm	Idmn	0.964169733
wavelet-LHH	glcm	JointEnergy	0.028112266
wavelet-LHH	glcm	Contrast	15.81931333
wavelet-LHH	glcm	DifferenceEntropy	2.930455714
wavelet-LHH	glcm	InverseVariance	0.360900874
wavelet-LHH	glcm	DifferenceVariance	8.312574115
wavelet-LHH	glcm	Idn	0.889111044
wavelet-LHH	glcm	Idm	0.364835119
wavelet-LHH	glcm	Correlation	-0.043915489
wavelet-LHH	glcm	Autocorrelation	111.6886582
wavelet-LHH	glcm	SumEntropy	3.690142187
wavelet-LHH	glcm	SumSquares	7.633302157
wavelet-LHH	glcm	ClusterProminence	1257.327149
wavelet-LHH	glcm	Imc2	0.850671865
wavelet-LHH	glcm	Imc1	-0.200727135
wavelet-LHH	glcm	DifferenceAverage	2.692331696
wavelet-LHH	glcm	Id	0.433973493
wavelet-LHH	glcm	ClusterTendency	14.7138953
wavelet-LHH	firstorder	InterquartileRange	63.91506847
wavelet-LHH	firstorder	Skewness	-0.033061995
wavelet-LHH	firstorder	Uniformity	0.138828017
wavelet-LHH	firstorder	Median	-4.321356407
wavelet-LHH	firstorder	Energy	1062384.039
wavelet-LHH	firstorder	RobustMeanAbsoluteDeviation	27.37355061
wavelet-LHH	firstorder	MeanAbsoluteDeviation	48.68002098
wavelet-LHH	firstorder	TotalEnergy	28684369.04

wavelet-LHH	firstorder	Maximum	207.7075668
wavelet-LHH	firstorder	RootMeanSquared	69.17740735
wavelet-LHH	firstorder	90Percentile	84.5340895
wavelet-LHH	firstorder	Minimum	-238.1630429
wavelet-LHH	firstorder	Entropy	3.308957012
wavelet-LHH	firstorder	Range	445.8706097
wavelet-LHH	firstorder	Variance	4782.907552
wavelet-LHH	firstorder	10Percentile	-64.7685217
wavelet-LHH	firstorder	Kurtosis	4.821369406
wavelet-LHH	firstorder	Mean	1.614352917
wavelet-LHH	glrlm	ShortRunLowGrayLevelEmphasis	0.020211292
wavelet-LHH	glrlm	GrayLevelVariance	8.525190812
wavelet-LHH	glrlm	LowGrayLevelRunEmphasis	0.021400835
wavelet-LHH	glrlm	GrayLevelNonUniformityNormalized	0.126130304
wavelet-LHH	glrlm	RunVariance	0.184728247
wavelet-LHH	glrlm	GrayLevelNonUniformity	24.81688869
wavelet-LHH	glrlm	LongRunEmphasis	1.471362669
wavelet-LHH	glrlm	ShortRunHighGrayLevelEmphasis	111.8059342
wavelet-LHH	glrlm	RunLengthNonUniformity	158.8726563
wavelet-LHH	glrlm	ShortRunEmphasis	0.916413498
wavelet-LHH	glrlm	LongRunHighGrayLevelEmphasis	172.3494721
wavelet-LHH	glrlm	RunPercentage	0.885654886
wavelet-LHH	glrlm	LongRunLowGrayLevelEmphasis	0.027469051
wavelet-LHH	glrlm	RunEntropy	3.868222494
wavelet-LHH	glrlm	HighGrayLevelRunEmphasis	121.00188
wavelet-LHH	glrlm	RunLengthNonUniformityNormalized	0.805561075
wavelet-LHH	glszm	GrayLevelVariance	17.31120517
wavelet-LHH	glszm	ZoneVariance	60.96146135
wavelet-LHH	glszm	GrayLevelNonUniformityNormalized	0.082646469
wavelet-LHH	glszm	SizeZoneNonUniformityNormalized	0.436400089
wavelet-LHH	glszm	SizeZoneNonUniformity	29.23880597
wavelet-LHH	glszm	GrayLevelNonUniformity	5.537313433
wavelet-LHH	glszm	LargeAreaEmphasis	71.94029851
wavelet-LHH	glszm	SmallAreaHighGrayLevelEmphasis	93.03964891
wavelet-LHH	glszm	ZonePercentage	0.301801802
wavelet-LHH	glszm	LargeAreaLowGrayLevelEmphasis	0.768031374
wavelet-LHH	glszm	LargeAreaHighGrayLevelEmphasis	7604.537313
wavelet-LHH	glszm	HighGrayLevelZoneEmphasis	134.4029851
wavelet-LHH	glszm	SmallAreaEmphasis	0.684443694
wavelet-LHH	glszm	LowGrayLevelZoneEmphasis	0.029671294
wavelet-LHH	glszm	ZoneEntropy	4.814860923
wavelet-LHH	glszm	SmallAreaLowGrayLevelEmphasis	0.015049545
wavelet-LHH	ngtdm	Coarseness	0.032616423
wavelet-LHH	ngtdm	Complexity	240.7715747
wavelet-LHH	ngtdm	Strength	2.868470626
wavelet-LHH	ngtdm	Contrast	0.102877277
wavelet-LHH	ngtdm	Busyness	0.134790617
wavelet-LLH	gldm	GrayLevelVariance	14.75523496
wavelet-LLH	gldm	HighGrayLevelEmphasis	175.0855856
wavelet-LLH	gldm	DependenceEntropy	5.945924568
wavelet-LLH	gldm	DependenceNonUniformity	32.76576577
wavelet-LLH	gldm	GrayLevelNonUniformity	20.99099099
wavelet-LLH	gldm	SmallDependenceEmphasis	0.265167051
wavelet-LLH	gldm	SmallDependenceHighGrayLevelEmphasis	50.75034889
wavelet-LLH	gldm	DependenceNonUniformityNormalized	0.147593539
wavelet-LLH	gldm	LargeDependenceEmphasis	20.1981982

wavelet-LLH	gldm	LargeDependenceLowGrayLevelEmphasis	0.151727399
wavelet-LLH	gldm	DependenceVariance	6.220030842
wavelet-LLH	gldm	LargeDependenceHighGrayLevelEmphasis	3382.382883
wavelet-LLH	gldm	SmallDependenceLowGrayLevelEmphasis	0.007731957
wavelet-LLH	gldm	LowGrayLevelEmphasis	0.014353759
wavelet-LLH	glcm	JointAverage	12.55831291
wavelet-LLH	glcm	SumAverage	25.11662582
wavelet-LLH	glcm	JointEntropy	6.510946605
wavelet-LLH	glcm	ClusterShade	56.87326271
wavelet-LLH	glcm	MaximumProbability	0.054036847
wavelet-LLH	glcm	Idmn	0.964595907
wavelet-LLH	glcm	JointEnergy	0.016090271
wavelet-LLH	glcm	Contrast	25.25117164
wavelet-LLH	glcm	DifferenceEntropy	3.197725228
wavelet-LLH	glcm	InverseVariance	0.293134107
wavelet-LLH	glcm	DifferenceVariance	13.09003897
wavelet-LLH	glcm	Idn	0.89039446
wavelet-LLH	glcm	Idm	0.318854464
wavelet-LLH	glcm	Correlation	0.094744884
wavelet-LLH	glcm	Autocorrelation	159.366995
wavelet-LLH	glcm	SumEntropy	4.228979029
wavelet-LLH	glcm	SumSquares	14.2753544
wavelet-LLH	glcm	ClusterProminence	4997.655953
wavelet-LLH	glcm	Imc2	0.921416115
wavelet-LLH	glcm	Imc1	-0.26036386
wavelet-LLH	glcm	DifferenceAverage	3.364063451
wavelet-LLH	glcm	Id	0.396935534
wavelet-LLH	glcm	ClusterTendency	31.85024595
wavelet-LLH	firstorder	InterquartileRange	97.29180255
wavelet-LLH	firstorder	Skewness	0.010810447
wavelet-LLH	firstorder	Uniformity	0.094554013
wavelet-LLH	firstorder	Median	-39.23817446
wavelet-LLH	firstorder	Energy	2487782.524
wavelet-LLH	firstorder	RobustMeanAbsoluteDeviation	41.47144449
wavelet-LLH	firstorder	MeanAbsoluteDeviation	69.75304332
wavelet-LLH	firstorder	TotalEnergy	67170128.14
wavelet-LLH	firstorder	Maximum	236.1900649
wavelet-LLH	firstorder	RootMeanSquared	105.8594709
wavelet-LLH	firstorder	90Percentile	61.83908919
wavelet-LLH	firstorder	Minimum	-333.1357615
wavelet-LLH	firstorder	Entropy	3.807142336
wavelet-LLH	firstorder	Range	569.3258264
wavelet-LLH	firstorder	Variance	9094.480178
wavelet-LLH	firstorder	10Percentile	-147.6823456
wavelet-LLH	firstorder	Kurtosis	3.979232354
wavelet-LLH	firstorder	Mean	-45.9537529
wavelet-LLH	glrlm	ShortRunLowGrayLevelEmphasis	0.014559897
wavelet-LLH	glrlm	GrayLevelVariance	15.71288492
wavelet-LLH	glrlm	LowGrayLevelRunEmphasis	0.015155367
wavelet-LLH	glrlm	GrayLevelNonUniformityNormalized	0.088260144
wavelet-LLH	glrlm	RunVariance	0.150041929
wavelet-LLH	glrlm	GrayLevelNonUniformity	17.53534198
wavelet-LLH	glrlm	LongRunEmphasis	1.407250449
wavelet-LLH	glrlm	ShortRunHighGrayLevelEmphasis	163.0952462
wavelet-LLH	glrlm	RunLengthNonUniformity	163.1944343
wavelet-LLH	glrlm	ShortRunEmphasis	0.922636132

wavelet-LLH	glrlm	LongRunHighGrayLevelEmphasis	244.3544589
wavelet-LLH	glrlm	RunPercentage	0.894663895
wavelet-LLH	glrlm	LongRunLowGrayLevelEmphasis	0.01826381
wavelet-LLH	glrlm	RunEntropy	4.291474135
wavelet-LLH	glrlm	HighGrayLevelRunEmphasis	176.0102917
wavelet-LLH	glrlm	RunLengthNonUniformityNormalized	0.819352259
wavelet-LLH	glszm	GrayLevelVariance	26.68155376
wavelet-LLH	glszm	ZoneVariance	27.92981798
wavelet-LLH	glszm	GrayLevelNonUniformityNormalized	0.055732783
wavelet-LLH	glszm	SizeZoneNonUniformityNormalized	0.340589229
wavelet-LLH	glszm	SizeZoneNonUniformity	24.8630137
wavelet-LLH	glszm	GrayLevelNonUniformity	4.068493151
wavelet-LLH	glszm	LargeAreaEmphasis	37.17808219
wavelet-LLH	glszm	SmallAreaHighGrayLevelEmphasis	118.0572311
wavelet-LLH	glszm	ZonePercentage	0.328828829
wavelet-LLH	glszm	LargeAreaLowGrayLevelEmphasis	0.259171278
wavelet-LLH	glszm	LargeAreaHighGrayLevelEmphasis	6538.561644
wavelet-LLH	glszm	HighGrayLevelZoneEmphasis	187.5890411
wavelet-LLH	glszm	SmallAreaEmphasis	0.594282376
wavelet-LLH	glszm	LowGrayLevelZoneEmphasis	0.026299269
wavelet-LLH	glszm	ZoneEntropy	5.364281237
wavelet-LLH	glszm	SmallAreaLowGrayLevelEmphasis	0.021185851
wavelet-LLH	ngtdm	Coarseness	0.033612618
wavelet-LLH	ngtdm	Complexity	467.8201905
wavelet-LLH	ngtdm	Strength	5.154834131
wavelet-LLH	ngtdm	Contrast	0.140283728
wavelet-LLH	ngtdm	Busyness	0.090863178
wavelet-LLL	gldm	GrayLevelVariance	770.8091064
wavelet-LLL	gldm	HighGrayLevelEmphasis	5424.261261
wavelet-LLL	gldm	DependenceEntropy	6.741201733
wavelet-LLL	gldm	DependenceNonUniformity	96.48648649
wavelet-LLL	gldm	GrayLevelNonUniformity	4.027027027
wavelet-LLL	gldm	SmallDependenceEmphasis	0.679825999
wavelet-LLL	gldm	SmallDependenceHighGrayLevelEmphasis	3346.621815
wavelet-LLL	gldm	DependenceNonUniformityNormalized	0.434623813
wavelet-LLL	gldm	LargeDependenceEmphasis	4.099099099
wavelet-LLL	gldm	LargeDependenceLowGrayLevelEmphasis	0.012175477
wavelet-LLL	gldm	DependenceVariance	1.260936612
wavelet-LLL	gldm	LargeDependenceHighGrayLevelEmphasis	30141.38739
wavelet-LLL	gldm	SmallDependenceLowGrayLevelEmphasis	0.006145075
wavelet-LLL	gldm	LowGrayLevelEmphasis	0.007032788
wavelet-LLL	glcm	JointAverage	72.08494572
wavelet-LLL	glcm	SumAverage	144.1698914
wavelet-LLL	glcm	JointEntropy	8.047324936
wavelet-LLL	glcm	ClusterShade	-70038.31412
wavelet-LLL	glcm	MaximumProbability	0.013660497
wavelet-LLL	glcm	Idmn	0.975891891
wavelet-LLL	glcm	JointEnergy	0.004105493
wavelet-LLL	glcm	Contrast	296.961272
wavelet-LLL	glcm	DifferenceEntropy	4.807376866
wavelet-LLL	glcm	InverseVariance	0.092555203
wavelet-LLL	glcm	DifferenceVariance	110.2876353
wavelet-LLL	glcm	Idn	0.896433634
wavelet-LLL	glcm	Idm	0.095029013
wavelet-LLL	glcm	Correlation	0.773075045
wavelet-LLL	glcm	Autocorrelation	5710.649284

wavelet-LLL	glcm	SumEntropy	6.367001355
wavelet-LLL	glcm	SumSquares	661.6414942
wavelet-LLL	glcm	ClusterProminence	13668938.22
wavelet-LLL	glcm	Imc2	0.999771984
wavelet-LLL	glcm	Imc1	-0.647048651
wavelet-LLL	glcm	DifferenceAverage	13.29550187
wavelet-LLL	glcm	Id	0.167189115
wavelet-LLL	glcm	ClusterTendency	2349.604705
wavelet-LLL	firstorder	InterquartileRange	1231.423927
wavelet-LLL	firstorder	Skewness	-0.505845738
wavelet-LLL	firstorder	Uniformity	0.018139761
wavelet-LLL	firstorder	Median	-477.7361601
wavelet-LLL	firstorder	Energy	181960488.7
wavelet-LLL	firstorder	RobustMeanAbsoluteDeviation	472.9783028
wavelet-LLL	firstorder	MeanAbsoluteDeviation	597.7968556
wavelet-LLL	firstorder	TotalEnergy	4912933195
wavelet-LLL	firstorder	Maximum	399.9480934
wavelet-LLL	firstorder	RootMeanSquared	905.340732
wavelet-LLL	firstorder	90Percentile	213.4165551
wavelet-LLL	firstorder	Minimum	-2264.264984
wavelet-LLL	firstorder	Entropy	6.063576018
wavelet-LLL	firstorder	Range	2664.213078
wavelet-LLL	firstorder	Variance	481339.4997
wavelet-LLL	firstorder	10Percentile	-1555.845198
wavelet-LLL	firstorder	Kurtosis	2.184222459
wavelet-LLL	firstorder	Mean	-581.6376375
wavelet-LLL	glrlm	ShortRunLowGrayLevelEmphasis	0.007121746
wavelet-LLL	glrlm	GrayLevelVariance	763.9646011
wavelet-LLL	glrlm	LowGrayLevelRunEmphasis	0.007167188
wavelet-LLL	glrlm	GrayLevelNonUniformityNormalized	0.017570246
wavelet-LLL	glrlm	RunVariance	0.029296688
wavelet-LLL	glrlm	GrayLevelNonUniformity	3.799464041
wavelet-LLL	glrlm	LongRunEmphasis	1.086381279
wavelet-LLL	glrlm	ShortRunHighGrayLevelEmphasis	5223.722219
wavelet-LLL	glrlm	RunLengthNonUniformity	205.4382754
wavelet-LLL	glrlm	ShortRunEmphasis	0.979913761
wavelet-LLL	glrlm	LongRunHighGrayLevelEmphasis	6005.902093
wavelet-LLL	glrlm	RunPercentage	0.973665974
wavelet-LLL	glrlm	LongRunLowGrayLevelEmphasis	0.007350648
wavelet-LLL	glrlm	RunEntropy	6.159912388
wavelet-LLL	glrlm	HighGrayLevelRunEmphasis	5371.208525
wavelet-LLL	glrlm	RunLengthNonUniformityNormalized	0.949260471
wavelet-LLL	glszm	GrayLevelVariance	686.1804689
wavelet-LLL	glszm	ZoneVariance	1.030773697
wavelet-LLL	glszm	GrayLevelNonUniformityNormalized	0.015314269
wavelet-LLL	glszm	SizeZoneNonUniformityNormalized	0.677819713
wavelet-LLL	glszm	SizeZoneNonUniformity	112.5180723
wavelet-LLL	glszm	GrayLevelNonUniformity	2.542168675
wavelet-LLL	glszm	LargeAreaEmphasis	2.819277108
wavelet-LLL	glszm	SmallAreaHighGrayLevelEmphasis	4124.909696
wavelet-LLL	glszm	ZonePercentage	0.747747748
wavelet-LLL	glszm	LargeAreaLowGrayLevelEmphasis	0.012225268
wavelet-LLL	glszm	LargeAreaHighGrayLevelEmphasis	19207.71084
wavelet-LLL	glszm	HighGrayLevelZoneEmphasis	4989.066265
wavelet-LLL	glszm	SmallAreaEmphasis	0.848859554
wavelet-LLL	glszm	LowGrayLevelZoneEmphasis	0.008444507

wavelet-LLL	glszm	ZoneEntropy	6.502786494
wavelet-LLL	glszm	SmallAreaLowGrayLevelEmphasis	0.007982094
wavelet-LLL	ngtdm	Coarseness	0.039826961
wavelet-LLL	ngtdm	Complexity	21506.01486
wavelet-LLL	ngtdm	Strength	173.3865233
wavelet-LLL	ngtdm	Contrast	1.644248877
wavelet-LLL	ngtdm	Busyness	0.004521081
wavelet-HHL	gldm	GrayLevelVariance	3.175898872
wavelet-HHL	gldm	HighGrayLevelEmphasis	46.01351351
wavelet-HHL	gldm	DependenceEntropy	5.599921217
wavelet-HHL	gldm	DependenceNonUniformity	22.51351351
wavelet-HHL	gldm	GrayLevelNonUniformity	41.89189189
wavelet-HHL	gldm	SmallDependenceEmphasis	0.171768078
wavelet-HHL	gldm	SmallDependenceHighGrayLevelEmphasis	9.105472153
wavelet-HHL	gldm	DependenceNonUniformityNormalized	0.101412223
wavelet-HHL	gldm	LargeDependenceEmphasis	36.99099099
wavelet-HHL	gldm	LargeDependenceLowGrayLevelEmphasis	0.973048016
wavelet-HHL	gldm	DependenceVariance	10.99017937
wavelet-HHL	gldm	LargeDependenceHighGrayLevelEmphasis	1635.274775
wavelet-HHL	gldm	SmallDependenceLowGrayLevelEmphasis	0.01027895
wavelet-HHL	gldm	LowGrayLevelEmphasis	0.042784691
wavelet-HHL	glcm	JointAverage	6.560387236
wavelet-HHL	glcm	SumAverage	13.12077447
wavelet-HHL	glcm	JointEntropy	5.133988822
wavelet-HHL	glcm	ClusterShade	-4.716599156
wavelet-HHL	glcm	MaximumProbability	0.10367637
wavelet-HHL	glcm	Idmn	0.963220256
wavelet-HHL	glcm	JointEnergy	0.043758259
wavelet-HHL	glcm	Contrast	5.094600879
wavelet-HHL	glcm	DifferenceEntropy	2.350269921
wavelet-HHL	glcm	InverseVariance	0.402488936
wavelet-HHL	glcm	DifferenceVariance	2.220257365
wavelet-HHL	glcm	Idn	0.879195493
wavelet-HHL	glcm	Idm	0.447712297
wavelet-HHL	glcm	Correlation	0.087425124
wavelet-HHL	glcm	Autocorrelation	43.34047153
wavelet-HHL	glcm	SumEntropy	3.234530168
wavelet-HHL	glcm	SumSquares	2.847197048
wavelet-HHL	glcm	ClusterProminence	179.7547852
wavelet-HHL	glcm	Imc2	0.646234799
wavelet-HHL	glcm	Imc1	-0.105748205
wavelet-HHL	glcm	DifferenceAverage	1.67357537
wavelet-HHL	glcm	Id	0.508802961
wavelet-HHL	glcm	ClusterTendency	6.294187314
wavelet-HHL	firstorder	InterquartileRange	41.29840525
wavelet-HHL	firstorder	Skewness	-0.195845793
wavelet-HHL	firstorder	Uniformity	0.188702216
wavelet-HHL	firstorder	Median	0.422274179
wavelet-HHL	firstorder	Energy	419287.856
wavelet-HHL	firstorder	RobustMeanAbsoluteDeviation	18.13591288
wavelet-HHL	firstorder	MeanAbsoluteDeviation	31.10902541
wavelet-HHL	firstorder	TotalEnergy	11320772.11
wavelet-HHL	firstorder	Maximum	114.2581477
wavelet-HHL	firstorder	RootMeanSquared	43.45899258
wavelet-HHL	firstorder	90Percentile	57.77919694
wavelet-HHL	firstorder	Minimum	-146.8283797

wavelet-HHL	firstorder	Entropy	2.775182729
wavelet-HHL	firstorder	Range	261.0865275
wavelet-HHL	firstorder	Variance	1887.162807
wavelet-HHL	firstorder	10Percentile	-44.28561685
wavelet-HHL	firstorder	Kurtosis	4.430371208
wavelet-HHL	firstorder	Mean	1.233380968
wavelet-HHL	glrlm	ShortRunLowGrayLevelEmphasis	0.041611464
wavelet-HHL	glrlm	GrayLevelVariance	3.56883719
wavelet-HHL	glrlm	LowGrayLevelRunEmphasis	0.045280344
wavelet-HHL	glrlm	GrayLevelNonUniformityNormalized	0.171070888
wavelet-HHL	glrlm	RunVariance	0.254562791
wavelet-HHL	glrlm	GrayLevelNonUniformity	32.02942131
wavelet-HHL	glrlm	LongRunEmphasis	1.679982139
wavelet-HHL	glrlm	ShortRunHighGrayLevelEmphasis	41.34935913
wavelet-HHL	glrlm	RunLengthNonUniformity	139.0686871
wavelet-HHL	glrlm	ShortRunEmphasis	0.882510579
wavelet-HHL	glrlm	LongRunHighGrayLevelEmphasis	76.36410914
wavelet-HHL	glrlm	RunPercentage	0.842342342
wavelet-HHL	glrlm	LongRunLowGrayLevelEmphasis	0.064793384
wavelet-HHL	glrlm	RunEntropy	3.506369122
wavelet-HHL	glrlm	HighGrayLevelRunEmphasis	46.45101573
wavelet-HHL	glrlm	RunLengthNonUniformityNormalized	0.740031203
wavelet-HHL	glszm	GrayLevelVariance	8.151111111
wavelet-HHL	glszm	ZoneVariance	133.2622222
wavelet-HHL	glszm	GrayLevelNonUniformityNormalized	0.109135802
wavelet-HHL	glszm	SizeZoneNonUniformityNormalized	0.302716049
wavelet-HHL	glszm	SizeZoneNonUniformity	13.62222222
wavelet-HHL	glszm	GrayLevelNonUniformity	4.911111111
wavelet-HHL	glszm	LargeAreaEmphasis	157.6
wavelet-HHL	glszm	SmallAreaHighGrayLevelEmphasis	31.27921547
wavelet-HHL	glszm	ZonePercentage	0.202702703
wavelet-HHL	glszm	LargeAreaLowGrayLevelEmphasis	3.951975762
wavelet-HHL	glszm	LargeAreaHighGrayLevelEmphasis	6954.022222
wavelet-HHL	glszm	HighGrayLevelZoneEmphasis	53.48888889
wavelet-HHL	glszm	SmallAreaEmphasis	0.558889869
wavelet-HHL	glszm	LowGrayLevelZoneEmphasis	0.064596038
wavelet-HHL	glszm	ZoneEntropy	4.597082818
wavelet-HHL	glszm	SmallAreaLowGrayLevelEmphasis	0.031969184
wavelet-HHL	ngtdm	Coarseness	0.031548658
wavelet-HHL	ngtdm	Complexity	55.42619791
wavelet-HHL	ngtdm	Strength	1.077440222
wavelet-HHL	ngtdm	Contrast	0.071555651
wavelet-HHL	ngtdm	Busyness	0.401000083
wavelet-HLH	gldm	GrayLevelVariance	2.047094392
wavelet-HLH	gldm	HighGrayLevelEmphasis	45.83333333
wavelet-HLH	gldm	DependenceEntropy	5.271698289
wavelet-HLH	gldm	DependenceNonUniformity	20.01801802
wavelet-HLH	gldm	GrayLevelNonUniformity	51.61261261
wavelet-HLH	gldm	SmallDependenceEmphasis	0.16529223
wavelet-HLH	gldm	SmallDependenceHighGrayLevelEmphasis	9.449525076
wavelet-HLH	gldm	DependenceNonUniformityNormalized	0.090171252
wavelet-HLH	gldm	LargeDependenceEmphasis	45.99099099
wavelet-HLH	gldm	LargeDependenceLowGrayLevelEmphasis	1.090616992
wavelet-HLH	gldm	DependenceVariance	12.74693613
wavelet-HLH	gldm	LargeDependenceHighGrayLevelEmphasis	2037.382883
wavelet-HLH	gldm	SmallDependenceLowGrayLevelEmphasis	0.009437076

wavelet-HLH	gldm	LowGrayLevelEmphasis	0.03058336
wavelet-HLH	glcm	JointAverage	6.615126762
wavelet-HLH	glcm	SumAverage	13.23025352
wavelet-HLH	glcm	JointEntropy	4.571034056
wavelet-HLH	glcm	ClusterShade	2.202388765
wavelet-HLH	glcm	MaximumProbability	0.149850122
wavelet-HLH	glcm	Idmn	0.978522896
wavelet-HLH	glcm	JointEnergy	0.068400539
wavelet-HLH	glcm	Contrast	4.024232519
wavelet-HLH	glcm	DifferenceEntropy	2.178877981
wavelet-HLH	glcm	InverseVariance	0.447234393
wavelet-HLH	glcm	DifferenceVariance	1.944949993
wavelet-HLH	glcm	Idn	0.909399521
wavelet-HLH	glcm	Idm	0.504343207
wavelet-HLH	glcm	Correlation	0.001831784
wavelet-HLH	glcm	Autocorrelation	43.75885458
wavelet-HLH	glcm	SumEntropy	2.88555425
wavelet-HLH	glcm	SumSquares	2.010592661
wavelet-HLH	glcm	ClusterProminence	84.34595278
wavelet-HLH	glcm	Imc2	0.63117009
wavelet-HLH	glcm	Imc1	-0.110578873
wavelet-HLH	glcm	DifferenceAverage	1.418074687
wavelet-HLH	glcm	Id	0.552108034
wavelet-HLH	glcm	ClusterTendency	4.018138123
wavelet-HLH	firstorder	InterquartileRange	32.26287043
wavelet-HLH	firstorder	Skewness	0.216639184
wavelet-HLH	firstorder	Uniformity	0.232489246
wavelet-HLH	firstorder	Median	2.722666107
wavelet-HLH	firstorder	Energy	266739.3475
wavelet-HLH	firstorder	RobustMeanAbsoluteDeviation	14.45131631
wavelet-HLH	firstorder	MeanAbsoluteDeviation	24.30940338
wavelet-HLH	firstorder	TotalEnergy	7201962.383
wavelet-HLH	firstorder	Maximum	160.6539643
wavelet-HLH	firstorder	RootMeanSquared	34.66307246
wavelet-HLH	firstorder	90Percentile	41.42155377
wavelet-HLH	firstorder	Minimum	-131.5843607
wavelet-HLH	firstorder	Entropy	2.455236704
wavelet-HLH	firstorder	Range	292.238325
wavelet-HLH	firstorder	Variance	1189.716866
wavelet-HLH	firstorder	10Percentile	-35.83257431
wavelet-HLH	firstorder	Kurtosis	5.909801813
wavelet-HLH	firstorder	Mean	3.436819208
wavelet-HLH	glrlm	ShortRunLowGrayLevelEmphasis	0.028582253
wavelet-HLH	glrlm	GrayLevelVariance	2.382151981
wavelet-HLH	glrlm	LowGrayLevelRunEmphasis	0.032033159
wavelet-HLH	glrlm	GrayLevelNonUniformityNormalized	0.208315192
wavelet-HLH	glrlm	RunVariance	0.305626903
wavelet-HLH	glrlm	GrayLevelNonUniformity	37.83073297
wavelet-HLH	glrlm	LongRunEmphasis	1.82936582
wavelet-HLH	glrlm	ShortRunHighGrayLevelEmphasis	40.115658
wavelet-HLH	glrlm	RunLengthNonUniformity	127.170286
wavelet-HLH	glrlm	ShortRunEmphasis	0.858350996
wavelet-HLH	glrlm	LongRunHighGrayLevelEmphasis	82.49827408
wavelet-HLH	glrlm	RunPercentage	0.816701317
wavelet-HLH	glrlm	LongRunLowGrayLevelEmphasis	0.052172913
wavelet-HLH	glrlm	RunEntropy	3.297563641

wavelet-HLH	glrlm	HighGrayLevelRunEmphasis	46.32137738
wavelet-HLH	glrlm	RunLengthNonUniformityNormalized	0.696225658
wavelet-HLH	glszm	GrayLevelVariance	6.967784352
wavelet-HLH	glszm	ZoneVariance	213.6489152
wavelet-HLH	glszm	GrayLevelNonUniformityNormalized	0.130834977
wavelet-HLH	glszm	SizeZoneNonUniformityNormalized	0.418803419
wavelet-HLH	glszm	SizeZoneNonUniformity	16.33333333
wavelet-HLH	glszm	GrayLevelNonUniformity	5.102564103
wavelet-HLH	glszm	LargeAreaEmphasis	246.0512821
wavelet-HLH	glszm	SmallAreaHighGrayLevelEmphasis	40.86801903
wavelet-HLH	glszm	ZonePercentage	0.175675676
wavelet-HLH	glszm	LargeAreaLowGrayLevelEmphasis	5.957184659
wavelet-HLH	glszm	LargeAreaHighGrayLevelEmphasis	10662.4359
wavelet-HLH	glszm	HighGrayLevelZoneEmphasis	58.51282051
wavelet-HLH	glszm	SmallAreaEmphasis	0.651670462
wavelet-HLH	glszm	LowGrayLevelZoneEmphasis	0.054785856
wavelet-HLH	glszm	ZoneEntropy	4.278114718
wavelet-HLH	glszm	SmallAreaLowGrayLevelEmphasis	0.046104168
wavelet-HLH	ngtdm	Coarseness	0.027687124
wavelet-HLH	ngtdm	Complexity	57.26159193
wavelet-HLH	ngtdm	Strength	1.285404798
wavelet-HLH	ngtdm	Contrast	0.040728686
wavelet-HLH	ngtdm	Busyness	0.394905816

Image type	Feature Class	Feature Name	10: RTSTRUCT: C99-DNU_segment_Tumor
general	info	BoundingBox	(0, 1, 1, 6, 7, 5)
general	info	EnabledImageTypes	{u'Original': {}, u'Wavelet': {}}
general	info	GeneralSettings	{'distances': [1], 'additionalInfo': True, 'force2D': False, 'interpolator': 'sitkBSpline', 'resampledPixelSpacing': [3.0], 'label': 1, 'normalizeScale': 1, 'normalize': False, 'force2Ddimension': 0, 'removeOutliers': None, 'minimumROISize': None, u'binWidth': 25.0, 'minimumROIDimensions': 1, u'correctMask': True, 'preCrop': False, u'symmetricalGLCM': True, 'resegmentRange': None, 'padDistance': 5}
general	info	ImageHash	a70adbc7365e0a4e72f44c25f62bf144db3258dc
general	info	ImageSpacing	(0.9765625000000002, 0.9765625000000002, 2.0)
general	info	MaskHash	15bb1094c84bdffc91d44168820c2ae5f398452b
general	info	NumpyVersion	1.13.1
general	info	PyWaveletVersion	0.5.2
general	info	SimpleITKVersion	1.1.0
general	info	Version	1.3.0.post102+g1b0eb74
general	info	VolumeNum	1
general	info	VoxelNum	60
original	shape	Maximum3DDiameter	24.73863375
original	shape	Maximum2DDiameterSlice	15.29705854
original	shape	Sphericity	0.804880811
original	shape	MinorAxis	12.8521947
original	shape	Elongation	0.58710221
original	shape	SurfaceVolumeRatio	0.511580772
original	shape	Volume	1620
original	shape	MajorAxis	21.89089818
original	shape	SurfaceArea	828.76085
original	shape	Flatness	0.422271343
original	shape	LeastAxis	9.243898979
original	shape	Maximum2DDiameterColumn	15
original	shape	Maximum2DDiameterRow	17.49285568
original	gldm	GrayLevelVariance	315.6497222
original	gldm	HighGrayLevelEmphasis	822.65
original	gldm	DependenceEntropy	5.156564762
original	gldm	DependenceNonUniformity	36.76666667
original	gldm	GrayLevelNonUniformity	2.533333333
original	gldm	SmallDependenceEmphasis	0.803356481
original	gldm	SmallDependenceHighGrayLevelEmphasis	637.0978009
original	gldm	DependenceNonUniformityNormalized	0.612777778
original	gldm	LargeDependenceEmphasis	2.566666667
original	gldm	LargeDependenceLowGrayLevelEmphasis	0.081600354
original	gldm	DependenceVariance	0.606666667
original	gldm	LargeDependenceHighGrayLevelEmphasis	2422.416667
original	gldm	SmallDependenceLowGrayLevelEmphasis	0.036920109
original	gldm	LowGrayLevelEmphasis	0.044439172
original	glcm	JointAverage	25.24458555
original	glcm	SumAverage	50.4891711

original	glcm	JointEntropy	5.796134582
original	glcm	ClusterShade	4129.994632
original	glcm	MaximumProbability	0.033117147
original	glcm	Idmn	0.906571994
original	glcm	JointEnergy	0.018981632
original	glcm	Contrast	600.9406925
original	glcm	DifferenceEntropy	4.345036272
original	glcm	InverseVariance	0.075878718
original	glcm	DifferenceVariance	212.5114
original	glcm	Idn	0.806396878
original	glcm	Idm	0.073633434
original	glcm	Correlation	0.08427991
original	glcm	Autocorrelation	660.9896334
original	glcm	SumEntropy	4.614879324
original	glcm	SumSquares	322.944709
original	glcm	ClusterProminence	1054130.905
original	glcm	Imc2	0.999623638
original	glcm	Imc1	-0.769438221
original	glcm	DifferenceAverage	19.17537792
original	glcm	Id	0.13294796
original	glcm	ClusterTendency	690.8381436
original	firstorder	InterquartileRange	786
original	firstorder	Skewness	0.69397403
original	firstorder	Uniformity	0.042222222
original	firstorder	Median	-578.5
original	firstorder	Energy	25358766
original	firstorder	RobustMeanAbsoluteDeviation	292.4826389
original	firstorder	MeanAbsoluteDeviation	375.4944444
original	firstorder	TotalEnergy	684686682
original	firstorder	Maximum	706
original	firstorder	RootMeanSquared	650.1123749
original	firstorder	90Percentile	114.9
original	firstorder	Minimum	-1024
original	firstorder	Entropy	4.874954813
original	firstorder	Range	1730
original	firstorder	Variance	196609.2456
original	firstorder	10Percentile	-957.3
original	firstorder	Kurtosis	2.411044079
original	firstorder	Mean	-475.4333333
original	glrlm	ShortRunLowGrayLevelEmphasis	0.044354834
original	glrlm	GrayLevelVariance	315.2951056
original	glrlm	LowGrayLevelRunEmphasis	0.044693277
original	glrlm	GrayLevelNonUniformityNormalized	0.041499603
original	glrlm	RunVariance	0.015366022
original	glrlm	GrayLevelNonUniformity	2.452100444
original	glrlm	LongRunEmphasis	1.047617583
original	glrlm	ShortRunHighGrayLevelEmphasis	807.6786123
original	glrlm	RunLengthNonUniformity	57.2893187
original	glrlm	ShortRunEmphasis	0.988095604
original	glrlm	LongRunHighGrayLevelEmphasis	867.7405892
original	glrlm	RunPercentage	0.984615385
original	glrlm	LongRunLowGrayLevelEmphasis	0.046047048
original	glrlm	RunEntropy	4.926756758
original	glrlm	HighGrayLevelRunEmphasis	819.6910077
original	glrlm	RunLengthNonUniformityNormalized	0.969267956

original	glszm	GrayLevelVariance	314.2106882
original	glszm	ZoneVariance	0.341407151
original	glszm	GrayLevelNonUniformityNormalized	0.035755479
original	glszm	SizeZoneNonUniformityNormalized	0.816993464
original	glszm	SizeZoneNonUniformity	41.66666667
original	glszm	GrayLevelNonUniformity	1.823529412
original	glszm	LargeAreaEmphasis	1.725490196
original	glszm	SmallAreaHighGrayLevelEmphasis	729.7376089
original	glszm	ZonePercentage	0.85
original	glszm	LargeAreaLowGrayLevelEmphasis	0.067079699
original	glszm	LargeAreaHighGrayLevelEmphasis	1447.392157
original	glszm	HighGrayLevelZoneEmphasis	805.1372549
original	glszm	SmallAreaEmphasis	0.917347495
original	glszm	LowGrayLevelZoneEmphasis	0.046103431
original	glszm	ZoneEntropy	5.093802303
original	glszm	SmallAreaLowGrayLevelEmphasis	0.042007851
original	ngtdm	Coarseness	0.027879114
original	ngtdm	Complexity	12826.28612
original	ngtdm	Strength	58.31439952
original	ngtdm	Contrast	7.274612489
original	ngtdm	Busyness	0.058737683
wavelet-HHH	gldm	GrayLevelVariance	59.98305556
wavelet-HHH	gldm	HighGrayLevelEmphasis	433.1166667
wavelet-HHH	gldm	DependenceEntropy	5.048394346
wavelet-HHH	gldm	DependenceNonUniformity	27.4
wavelet-HHH	gldm	GrayLevelNonUniformity	3.533333333
wavelet-HHH	gldm	SmallDependenceEmphasis	0.693171296
wavelet-HHH	gldm	SmallDependenceHighGrayLevelEmphasis	280.0587963
wavelet-HHH	gldm	DependenceNonUniformityNormalized	0.456666667
wavelet-HHH	gldm	LargeDependenceEmphasis	2.933333333
wavelet-HHH	gldm	LargeDependenceLowGrayLevelEmphasis	0.034079523
wavelet-HHH	gldm	DependenceVariance	0.582222222
wavelet-HHH	gldm	LargeDependenceHighGrayLevelEmphasis	1261.95
wavelet-HHH	gldm	SmallDependenceLowGrayLevelEmphasis	0.023693843
wavelet-HHH	gldm	LowGrayLevelEmphasis	0.024848622
wavelet-HHH	glcm	JointAverage	19.23013541
wavelet-HHH	glcm	SumAverage	38.46027082
wavelet-HHH	glcm	JointEntropy	5.716394353
wavelet-HHH	glcm	ClusterShade	-257.6316111
wavelet-HHH	glcm	MaximumProbability	0.04065085
wavelet-HHH	glcm	Idmn	0.903004011
wavelet-HHH	glcm	JointEnergy	0.020356242
wavelet-HHH	glcm	Contrast	144.1991405
wavelet-HHH	glcm	DifferenceEntropy	3.893172613
wavelet-HHH	glcm	InverseVariance	0.099699973
wavelet-HHH	glcm	DifferenceVariance	43.02189811
wavelet-HHH	glcm	Idn	0.795501346
wavelet-HHH	glcm	Idm	0.10335829
wavelet-HHH	glcm	Correlation	-0.042929043
wavelet-HHH	glcm	Autocorrelation	367.2996005
wavelet-HHH	glcm	SumEntropy	4.279500213
wavelet-HHH	glcm	SumSquares	69.25174198
wavelet-HHH	glcm	ClusterProminence	46375.96555
wavelet-HHH	glcm	Imc2	0.998102175
wavelet-HHH	glcm	Imc1	-0.659440296

wavelet-HHH	glcm	DifferenceAverage	9.772809242
wavelet-HHH	glcm	Id	0.181732556
wavelet-HHH	glcm	ClusterTendency	132.8078274
wavelet-HHH	firstorder	InterquartileRange	288.9668201
wavelet-HHH	firstorder	Skewness	-0.206091219
wavelet-HHH	firstorder	Uniformity	0.058888889
wavelet-HHH	firstorder	Median	14.53870149
wavelet-HHH	firstorder	Energy	2268919.061
wavelet-HHH	firstorder	RobustMeanAbsoluteDeviation	118.5505481
wavelet-HHH	firstorder	MeanAbsoluteDeviation	160.2260448
wavelet-HHH	firstorder	TotalEnergy	61260814.64
wavelet-HHH	firstorder	Maximum	359.4888491
wavelet-HHH	firstorder	RootMeanSquared	194.4616098
wavelet-HHH	firstorder	90Percentile	221.3242662
wavelet-HHH	firstorder	Minimum	-472.7450038
wavelet-HHH	firstorder	Entropy	4.41064997
wavelet-HHH	firstorder	Range	832.233853
wavelet-HHH	firstorder	Variance	37801.94784
wavelet-HHH	firstorder	10Percentile	-242.7383943
wavelet-HHH	firstorder	Kurtosis	2.448126482
wavelet-HHH	firstorder	Mean	-3.65647879
wavelet-HHH	glrlm	ShortRunLowGrayLevelEmphasis	0.025220225
wavelet-HHH	glrlm	GrayLevelVariance	60.03841585
wavelet-HHH	glrlm	LowGrayLevelRunEmphasis	0.025281808
wavelet-HHH	glrlm	GrayLevelNonUniformityNormalized	0.057743773
wavelet-HHH	glrlm	RunVariance	0.023184572
wavelet-HHH	glrlm	GrayLevelNonUniformity	3.394035103
wavelet-HHH	glrlm	LongRunEmphasis	1.066238856
wavelet-HHH	glrlm	ShortRunHighGrayLevelEmphasis	425.5112623
wavelet-HHH	glrlm	RunLengthNonUniformity	56.5253977
wavelet-HHH	glrlm	ShortRunEmphasis	0.984939761
wavelet-HHH	glrlm	LongRunHighGrayLevelEmphasis	461.8299633
wavelet-HHH	glrlm	RunPercentage	0.979487179
wavelet-HHH	glrlm	LongRunLowGrayLevelEmphasis	0.025588117
wavelet-HHH	glrlm	RunEntropy	4.490010821
wavelet-HHH	glrlm	HighGrayLevelRunEmphasis	432.6550445
wavelet-HHH	glrlm	RunLengthNonUniformityNormalized	0.96144391
wavelet-HHH	glszm	GrayLevelVariance	60.60680529
wavelet-HHH	glszm	ZoneVariance	0.559546314
wavelet-HHH	glszm	GrayLevelNonUniformityNormalized	0.047258979
wavelet-HHH	glszm	SizeZoneNonUniformityNormalized	0.666351607
wavelet-HHH	glszm	SizeZoneNonUniformity	30.65217391
wavelet-HHH	glszm	GrayLevelNonUniformity	2.173913043
wavelet-HHH	glszm	LargeAreaEmphasis	2.260869565
wavelet-HHH	glszm	SmallAreaHighGrayLevelEmphasis	333.1008454
wavelet-HHH	glszm	ZonePercentage	0.766666667
wavelet-HHH	glszm	LargeAreaLowGrayLevelEmphasis	0.037683811
wavelet-HHH	glszm	LargeAreaHighGrayLevelEmphasis	938.0652174
wavelet-HHH	glszm	HighGrayLevelZoneEmphasis	419.9565217
wavelet-HHH	glszm	SmallAreaEmphasis	0.842657005
wavelet-HHH	glszm	LowGrayLevelZoneEmphasis	0.031148135
wavelet-HHH	glszm	ZoneEntropy	4.958344565
wavelet-HHH	glszm	SmallAreaLowGrayLevelEmphasis	0.030686338
wavelet-HHH	ngtdm	Coarseness	0.045394637
wavelet-HHH	ngtdm	Complexity	2319.811428

wavelet-HHH	ngtdm	Strength	18.56264129
wavelet-HHH	ngtdm	Contrast	1.177839614
wavelet-HHH	ngtdm	Busyness	0.04490223
wavelet-HLL	gldm	GrayLevelVariance	179.8388889
wavelet-HLL	gldm	HighGrayLevelEmphasis	2101.2
wavelet-HLL	gldm	DependenceEntropy	5.181727679
wavelet-HLL	gldm	DependenceNonUniformity	33.8
wavelet-HLL	gldm	GrayLevelNonUniformity	2.333333333
wavelet-HLL	gldm	SmallDependenceEmphasis	0.777430556
wavelet-HLL	gldm	SmallDependenceHighGrayLevelEmphasis	1562.130208
wavelet-HLL	gldm	DependenceNonUniformityNormalized	0.563333333
wavelet-HLL	gldm	LargeDependenceEmphasis	2.3
wavelet-HLL	gldm	LargeDependenceLowGrayLevelEmphasis	0.01810811
wavelet-HLL	gldm	DependenceVariance	0.432222222
wavelet-HLL	gldm	LargeDependenceHighGrayLevelEmphasis	5223
wavelet-HLL	gldm	SmallDependenceLowGrayLevelEmphasis	0.017378676
wavelet-HLL	gldm	LowGrayLevelEmphasis	0.017489276
wavelet-HLL	glcm	JointAverage	44.53137729
wavelet-HLL	glcm	SumAverage	89.06275458
wavelet-HLL	glcm	JointEntropy	5.812296372
wavelet-HLL	glcm	ClusterShade	-8547.7646
wavelet-HLL	glcm	MaximumProbability	0.031570813
wavelet-HLL	glcm	Idmn	0.938887445
wavelet-HLL	glcm	JointEnergy	0.01861818
wavelet-HLL	glcm	Contrast	390.6821485
wavelet-HLL	glcm	DifferenceEntropy	4.161289952
wavelet-HLL	glcm	InverseVariance	0.087163292
wavelet-HLL	glcm	DifferenceVariance	149.5926164
wavelet-HLL	glcm	Idn	0.843461729
wavelet-HLL	glcm	Idm	0.08238767
wavelet-HLL	glcm	Correlation	0.124458299
wavelet-HLL	glcm	Autocorrelation	2008.920064
wavelet-HLL	glcm	SumEntropy	4.579061512
wavelet-HLL	glcm	SumSquares	220.8770173
wavelet-HLL	glcm	ClusterProminence	820609.9151
wavelet-HLL	glcm	Imc2	0.999665401
wavelet-HLL	glcm	Imc1	-0.778047794
wavelet-HLL	glcm	DifferenceAverage	14.85651695
wavelet-HLL	glcm	Id	0.150982754
wavelet-HLL	glcm	ClusterTendency	492.8259209
wavelet-HLL	firstorder	InterquartileRange	411.055468
wavelet-HLL	firstorder	Skewness	-0.888789227
wavelet-HLL	firstorder	Uniformity	0.038888889
wavelet-HLL	firstorder	Median	75.75923996
wavelet-HLL	firstorder	Energy	6701533.592
wavelet-HLL	firstorder	RobustMeanAbsoluteDeviation	167.0828631
wavelet-HLL	firstorder	MeanAbsoluteDeviation	256.0358042
wavelet-HLL	firstorder	TotalEnergy	180941407
wavelet-HLL	firstorder	Maximum	675.5838135
wavelet-HLL	firstorder	RootMeanSquared	334.2038697
wavelet-HLL	firstorder	90Percentile	362.4919708
wavelet-HLL	firstorder	Minimum	-1050.64545
wavelet-HLL	firstorder	Entropy	4.883822629
wavelet-HLL	firstorder	Range	1726.229263
wavelet-HLL	firstorder	Variance	111608.6274

wavelet-HLL	firstorder	10Percentile	-416.4064052
wavelet-HLL	firstorder	Kurtosis	3.937506034
wavelet-HLL	firstorder	Mean	9.143257568
wavelet-HLL	glrlm	ShortRunLowGrayLevelEmphasis	0.017733248
wavelet-HLL	glrlm	GrayLevelVariance	181.0886406
wavelet-HLL	glrlm	LowGrayLevelRunEmphasis	0.017738579
wavelet-HLL	glrlm	GrayLevelNonUniformityNormalized	0.038557432
wavelet-HLL	glrlm	RunVariance	0.014077102
wavelet-HLL	glrlm	GrayLevelNonUniformity	2.281109531
wavelet-HLL	glrlm	LongRunEmphasis	1.043983086
wavelet-HLL	glrlm	ShortRunHighGrayLevelEmphasis	2070.148888
wavelet-HLL	glrlm	RunLengthNonUniformity	57.52855422
wavelet-HLL	glrlm	ShortRunEmphasis	0.989004228
wavelet-HLL	glrlm	LongRunHighGrayLevelEmphasis	2202.699315
wavelet-HLL	glrlm	RunPercentage	0.985897436
wavelet-HLL	glrlm	LongRunLowGrayLevelEmphasis	0.017759904
wavelet-HLL	glrlm	RunEntropy	4.917859333
wavelet-HLL	glrlm	HighGrayLevelRunEmphasis	2096.658973
wavelet-HLL	glrlm	RunLengthNonUniformityNormalized	0.971845796
wavelet-HLL	glszm	GrayLevelVariance	194.65
wavelet-HLL	glszm	ZoneVariance	0.32
wavelet-HLL	glszm	GrayLevelNonUniformityNormalized	0.0368
wavelet-HLL	glszm	SizeZoneNonUniformityNormalized	0.7504
wavelet-HLL	glszm	SizeZoneNonUniformity	37.52
wavelet-HLL	glszm	GrayLevelNonUniformity	1.84
wavelet-HLL	glszm	LargeAreaEmphasis	1.76
wavelet-HLL	glszm	SmallAreaHighGrayLevelEmphasis	1765.233472
wavelet-HLL	glszm	ZonePercentage	0.833333333
wavelet-HLL	glszm	LargeAreaLowGrayLevelEmphasis	0.021249517
wavelet-HLL	glszm	LargeAreaHighGrayLevelEmphasis	3880.28
wavelet-HLL	glszm	HighGrayLevelZoneEmphasis	2035.06
wavelet-HLL	glszm	SmallAreaEmphasis	0.888472222
wavelet-HLL	glszm	LowGrayLevelZoneEmphasis	0.020889713
wavelet-HLL	glszm	ZoneEntropy	5.05366069
wavelet-HLL	glszm	SmallAreaLowGrayLevelEmphasis	0.020832572
wavelet-HLL	ngtdm	Coarseness	0.056813061
wavelet-HLL	ngtdm	Complexity	6111.536152
wavelet-HLL	ngtdm	Strength	57.82909284
wavelet-HLL	ngtdm	Contrast	2.772451962
wavelet-HLL	ngtdm	Busyness	0.014694112
wavelet-LHL	gldm	GrayLevelVariance	94.79888889
wavelet-LHL	gldm	HighGrayLevelEmphasis	493.4666667
wavelet-LHL	gldm	DependenceEntropy	5.115061012
wavelet-LHL	gldm	DependenceNonUniformity	25.03333333
wavelet-LHL	gldm	GrayLevelNonUniformity	3
wavelet-LHL	gldm	SmallDependenceEmphasis	0.632986111
wavelet-LHL	gldm	SmallDependenceHighGrayLevelEmphasis	332.8898148
wavelet-LHL	gldm	DependenceNonUniformityNormalized	0.417222222
wavelet-LHL	gldm	LargeDependenceEmphasis	3.1
wavelet-LHL	gldm	LargeDependenceLowGrayLevelEmphasis	0.052616437
wavelet-LHL	gldm	DependenceVariance	0.54
wavelet-LHL	gldm	LargeDependenceHighGrayLevelEmphasis	1336.033333
wavelet-LHL	gldm	SmallDependenceLowGrayLevelEmphasis	0.042520452
wavelet-LHL	gldm	LowGrayLevelEmphasis	0.04397819
wavelet-LHL	glcm	JointAverage	18.52877596

wavelet-LHL	glcm	SumAverage	37.05755192
wavelet-LHL	glcm	JointEntropy	5.772677031
wavelet-LHL	glcm	ClusterShade	1447.064289
wavelet-LHL	glcm	MaximumProbability	0.037657745
wavelet-LHL	glcm	Idmn	0.928430792
wavelet-LHL	glcm	JointEnergy	0.019391184
wavelet-LHL	glcm	Contrast	154.0654865
wavelet-LHL	glcm	DifferenceEntropy	3.875152084
wavelet-LHL	glcm	InverseVariance	0.07746816
wavelet-LHL	glcm	DifferenceVariance	47.57411141
wavelet-LHL	glcm	Idn	0.822685278
wavelet-LHL	glcm	Idm	0.094846307
wavelet-LHL	glcm	Correlation	0.21126504
wavelet-LHL	glcm	Autocorrelation	363.4707844
wavelet-LHL	glcm	SumEntropy	4.461907366
wavelet-LHL	glcm	SumSquares	96.69370545
wavelet-LHL	glcm	ClusterProminence	149580.8128
wavelet-LHL	glcm	Imc2	0.998661223
wavelet-LHL	glcm	Imc1	-0.684688954
wavelet-LHL	glcm	DifferenceAverage	10.00176215
wavelet-LHL	glcm	Id	0.176922984
wavelet-LHL	glcm	ClusterTendency	232.7093354
wavelet-LHL	firstorder	InterquartileRange	330.1883382
wavelet-LHL	firstorder	Skewness	0.050529282
wavelet-LHL	firstorder	Uniformity	0.05
wavelet-LHL	firstorder	Median	-84.99782464
wavelet-LHL	firstorder	Energy	4018650.406
wavelet-LHL	firstorder	RobustMeanAbsoluteDeviation	136.7830458
wavelet-LHL	firstorder	MeanAbsoluteDeviation	195.5673325
wavelet-LHL	firstorder	TotalEnergy	108503561
wavelet-LHL	firstorder	Maximum	474.6046718
wavelet-LHL	firstorder	RootMeanSquared	258.800129
wavelet-LHL	firstorder	90Percentile	238.8642074
wavelet-LHL	firstorder	Minimum	-568.3721948
wavelet-LHL	firstorder	Entropy	4.525326379
wavelet-LHL	firstorder	Range	1042.976867
wavelet-LHL	firstorder	Variance	59421.81164
wavelet-LHL	firstorder	10Percentile	-375.1937731
wavelet-LHL	firstorder	Kurtosis	2.546420474
wavelet-LHL	firstorder	Mean	-86.92350156
wavelet-LHL	glrlm	ShortRunLowGrayLevelEmphasis	0.044864581
wavelet-LHL	glrlm	GrayLevelVariance	96.06783268
wavelet-LHL	glrlm	LowGrayLevelRunEmphasis	0.044938307
wavelet-LHL	glrlm	GrayLevelNonUniformityNormalized	0.049427932
wavelet-LHL	glrlm	RunVariance	0.023024192
wavelet-LHL	glrlm	GrayLevelNonUniformity	2.898143253
wavelet-LHL	glrlm	LongRunEmphasis	1.072200632
wavelet-LHL	glrlm	ShortRunHighGrayLevelEmphasis	487.9384107
wavelet-LHL	glrlm	RunLengthNonUniformity	55.96494834
wavelet-LHL	glrlm	ShortRunEmphasis	0.981949842
wavelet-LHL	glrlm	LongRunHighGrayLevelEmphasis	525.233677
wavelet-LHL	glrlm	RunPercentage	0.976923077
wavelet-LHL	glrlm	LongRunLowGrayLevelEmphasis	0.045233211
wavelet-LHL	glrlm	RunEntropy	4.586830224
wavelet-LHL	glrlm	HighGrayLevelRunEmphasis	495.397464

wavelet-LHL	glrlm	RunLengthNonUniformityNormalized	0.953951616
wavelet-LHL	glszm	GrayLevelVariance	114.9691725
wavelet-LHL	glszm	ZoneVariance	0.657652785
wavelet-LHL	glszm	GrayLevelNonUniformityNormalized	0.042725798
wavelet-LHL	glszm	SizeZoneNonUniformityNormalized	0.585722012
wavelet-LHL	glszm	SizeZoneNonUniformity	25.18604651
wavelet-LHL	glszm	GrayLevelNonUniformity	1.837209302
wavelet-LHL	glszm	LargeAreaEmphasis	2.604651163
wavelet-LHL	glszm	SmallAreaHighGrayLevelEmphasis	427.1059432
wavelet-LHL	glszm	ZonePercentage	0.716666667
wavelet-LHL	glszm	LargeAreaLowGrayLevelEmphasis	0.066477911
wavelet-LHL	glszm	LargeAreaHighGrayLevelEmphasis	1151.488372
wavelet-LHL	glszm	HighGrayLevelZoneEmphasis	524.3255814
wavelet-LHL	glszm	SmallAreaEmphasis	0.793565891
wavelet-LHL	glszm	LowGrayLevelZoneEmphasis	0.059717033
wavelet-LHL	glszm	ZoneEntropy	4.99010458
wavelet-LHL	glszm	SmallAreaLowGrayLevelEmphasis	0.058941809
wavelet-LHL	ngtdm	Coarseness	0.062916439
wavelet-LHL	ngtdm	Complexity	2643.512755
wavelet-LHL	ngtdm	Strength	33.12768733
wavelet-LHL	ngtdm	Contrast	1.770092461
wavelet-LHL	ngtdm	Busyness	0.037669689
wavelet-LHH	gldm	GrayLevelVariance	43.08305556
wavelet-LHH	gldm	HighGrayLevelEmphasis	207.35
wavelet-LHH	gldm	DependenceEntropy	4.802479554
wavelet-LHH	gldm	DependenceNonUniformity	28.63333333
wavelet-LHH	gldm	GrayLevelNonUniformity	3.566666667
wavelet-LHH	gldm	SmallDependenceEmphasis	0.700925926
wavelet-LHH	gldm	SmallDependenceHighGrayLevelEmphasis	169.3185185
wavelet-LHH	gldm	DependenceNonUniformityNormalized	0.477222222
wavelet-LHH	gldm	LargeDependenceEmphasis	2.566666667
wavelet-LHH	gldm	LargeDependenceLowGrayLevelEmphasis	0.179200262
wavelet-LHH	gldm	DependenceVariance	0.415555556
wavelet-LHH	gldm	LargeDependenceHighGrayLevelEmphasis	438.8833333
wavelet-LHH	gldm	SmallDependenceLowGrayLevelEmphasis	0.031886526
wavelet-LHH	gldm	LowGrayLevelEmphasis	0.060696302
wavelet-LHH	glcm	JointAverage	12.85607737
wavelet-LHH	glcm	SumAverage	25.71215473
wavelet-LHH	glcm	JointEntropy	5.768171808
wavelet-LHH	glcm	ClusterShade	296.7085442
wavelet-LHH	glcm	MaximumProbability	0.033498709
wavelet-LHH	glcm	Idmn	0.910578758
wavelet-LHH	glcm	JointEnergy	0.019221031
wavelet-LHH	glcm	Contrast	90.90788011
wavelet-LHH	glcm	DifferenceEntropy	3.678751535
wavelet-LHH	glcm	InverseVariance	0.133878216
wavelet-LHH	glcm	DifferenceVariance	29.36316124
wavelet-LHH	glcm	Idn	0.805314712
wavelet-LHH	glcm	Idm	0.119322756
wavelet-LHH	glcm	Correlation	-0.01440765
wavelet-LHH	glcm	Autocorrelation	164.6995657
wavelet-LHH	glcm	SumEntropy	4.170037764
wavelet-LHH	glcm	SumSquares	44.78705953
wavelet-LHH	glcm	ClusterProminence	23243.56633
wavelet-LHH	glcm	Imc2	0.99704153

wavelet-LHH	glcm	Imc1	-0.625294066
wavelet-LHH	glcm	DifferenceAverage	7.602698105
wavelet-LHH	glcm	Id	0.207208244
wavelet-LHH	glcm	ClusterTendency	88.240358
wavelet-LHH	firstorder	InterquartileRange	222.0105103
wavelet-LHH	firstorder	Skewness	0.406770914
wavelet-LHH	firstorder	Uniformity	0.059444444
wavelet-LHH	firstorder	Median	-0.667134991
wavelet-LHH	firstorder	Energy	1657397.581
wavelet-LHH	firstorder	RobustMeanAbsoluteDeviation	89.60618688
wavelet-LHH	firstorder	MeanAbsoluteDeviation	130.6243226
wavelet-LHH	firstorder	TotalEnergy	44749734.7
wavelet-LHH	firstorder	Maximum	397.4956646
wavelet-LHH	firstorder	RootMeanSquared	166.2025662
wavelet-LHH	firstorder	90Percentile	265.1432828
wavelet-LHH	firstorder	Minimum	-297.7435483
wavelet-LHH	firstorder	Entropy	4.24371643
wavelet-LHH	firstorder	Range	695.239213
wavelet-LHH	firstorder	Variance	27545.51193
wavelet-LHH	firstorder	10Percentile	-180.9813447
wavelet-LHH	firstorder	Kurtosis	2.732060599
wavelet-LHH	firstorder	Mean	8.819358879
wavelet-LHH	glrlm	ShortRunLowGrayLevelEmphasis	0.059067531
wavelet-LHH	glrlm	GrayLevelVariance	43.32222946
wavelet-LHH	glrlm	LowGrayLevelRunEmphasis	0.060248693
wavelet-LHH	glrlm	GrayLevelNonUniformityNormalized	0.059214906
wavelet-LHH	glrlm	RunVariance	0.017922151
wavelet-LHH	glrlm	GrayLevelNonUniformity	3.489497741
wavelet-LHH	glrlm	LongRunEmphasis	1.055717115
wavelet-LHH	glrlm	ShortRunHighGrayLevelEmphasis	206.6619402
wavelet-LHH	glrlm	RunLengthNonUniformity	56.84406922
wavelet-LHH	glrlm	ShortRunEmphasis	0.986070721
wavelet-LHH	glrlm	LongRunHighGrayLevelEmphasis	216.3884568
wavelet-LHH	glrlm	RunPercentage	0.982051282
wavelet-LHH	glrlm	LongRunLowGrayLevelEmphasis	0.064973342
wavelet-LHH	glrlm	RunEntropy	4.295967368
wavelet-LHH	glrlm	HighGrayLevelRunEmphasis	208.6072435
wavelet-LHH	glrlm	RunLengthNonUniformityNormalized	0.964155698
wavelet-LHH	glszm	GrayLevelVariance	46.07876867
wavelet-LHH	glszm	ZoneVariance	0.327750113
wavelet-LHH	glszm	GrayLevelNonUniformityNormalized	0.056586691
wavelet-LHH	glszm	SizeZoneNonUniformityNormalized	0.645993662
wavelet-LHH	glszm	SizeZoneNonUniformity	30.36170213
wavelet-LHH	glszm	GrayLevelNonUniformity	2.659574468
wavelet-LHH	glszm	LargeAreaEmphasis	1.957446809
wavelet-LHH	glszm	SmallAreaHighGrayLevelEmphasis	208.9816785
wavelet-LHH	glszm	ZonePercentage	0.783333333
wavelet-LHH	glszm	LargeAreaLowGrayLevelEmphasis	0.1287205
wavelet-LHH	glszm	LargeAreaHighGrayLevelEmphasis	356.7446809
wavelet-LHH	glszm	HighGrayLevelZoneEmphasis	229.1914894
wavelet-LHH	glszm	SmallAreaEmphasis	0.831560284
wavelet-LHH	glszm	LowGrayLevelZoneEmphasis	0.052657246
wavelet-LHH	glszm	ZoneEntropy	4.713955341
wavelet-LHH	glszm	SmallAreaLowGrayLevelEmphasis	0.034519805
wavelet-LHH	ngtdm	Coarseness	0.061817268

wavelet-LHH	ngtdm	Complexity	1229.972169
wavelet-LHH	ngtdm	Strength	13.30052981
wavelet-LHH	ngtdm	Contrast	1.028955771
wavelet-LHH	ngtdm	Busyness	0.078211328
wavelet-LLH	gldm	GrayLevelVariance	426.1163889
wavelet-LLH	gldm	HighGrayLevelEmphasis	3541.616667
wavelet-LLH	gldm	DependenceEntropy	5.294309137
wavelet-LLH	gldm	DependenceNonUniformity	40.8
wavelet-LLH	gldm	GrayLevelNonUniformity	1.833333333
wavelet-LLH	gldm	SmallDependenceEmphasis	0.85
wavelet-LLH	gldm	SmallDependenceHighGrayLevelEmphasis	2945.491667
wavelet-LLH	gldm	DependenceNonUniformityNormalized	0.68
wavelet-LLH	gldm	LargeDependenceEmphasis	1.6
wavelet-LLH	gldm	LargeDependenceLowGrayLevelEmphasis	0.017923095
wavelet-LLH	gldm	DependenceVariance	0.16
wavelet-LLH	gldm	LargeDependenceHighGrayLevelEmphasis	5926.116667
wavelet-LLH	gldm	SmallDependenceLowGrayLevelEmphasis	0.017504397
wavelet-LLH	gldm	LowGrayLevelEmphasis	0.017588136
wavelet-LLH	glcm	JointAverage	55.55315862
wavelet-LLH	glcm	SumAverage	111.1063172
wavelet-LLH	glcm	JointEntropy	5.836102307
wavelet-LLH	glcm	ClusterShade	-6473.161697
wavelet-LLH	glcm	MaximumProbability	0.027066733
wavelet-LLH	glcm	Idmn	0.92963439
wavelet-LLH	glcm	JointEnergy	0.018227903
wavelet-LLH	glcm	Contrast	793.4678547
wavelet-LLH	glcm	DifferenceEntropy	4.359030232
wavelet-LLH	glcm	InverseVariance	0.051800128
wavelet-LLH	glcm	DifferenceVariance	282.9408429
wavelet-LLH	glcm	Idn	0.830125515
wavelet-LLH	glcm	Idm	0.046582124
wavelet-LLH	glcm	Correlation	0.068501966
wavelet-LLH	glcm	Autocorrelation	3132.958205
wavelet-LLH	glcm	SumEntropy	4.649306369
wavelet-LLH	glcm	SumSquares	442.1713903
wavelet-LLH	glcm	ClusterProminence	2598345.962
wavelet-LLH	glcm	Imc2	0.999879057
wavelet-LLH	glcm	Imc1	-0.835717106
wavelet-LLH	glcm	DifferenceAverage	21.65821615
wavelet-LLH	glcm	Id	0.105793912
wavelet-LLH	glcm	ClusterTendency	975.2177064
wavelet-LLH	firstorder	InterquartileRange	634.7666806
wavelet-LLH	firstorder	Skewness	-0.490390857
wavelet-LLH	firstorder	Uniformity	0.030555556
wavelet-LLH	firstorder	Median	56.43036495
wavelet-LLH	firstorder	Energy	16324533.82
wavelet-LLH	firstorder	RobustMeanAbsoluteDeviation	297.5268635
wavelet-LLH	firstorder	MeanAbsoluteDeviation	417.6840463
wavelet-LLH	firstorder	TotalEnergy	440762413.1
wavelet-LLH	firstorder	Maximum	882.6873604
wavelet-LLH	firstorder	RootMeanSquared	521.6086307
wavelet-LLH	firstorder	90Percentile	540.5420055
wavelet-LLH	firstorder	Minimum	-1444.854471
wavelet-LLH	firstorder	Entropy	5.181727679
wavelet-LLH	firstorder	Range	2327.541831

wavelet-LLH	firstorder	Variance	267561.3498
wavelet-LLH	firstorder	10Percentile	-775.8732986
wavelet-LLH	firstorder	Kurtosis	2.823644565
wavelet-LLH	firstorder	Mean	-67.18789918
wavelet-LLH	glrlm	ShortRunLowGrayLevelEmphasis	0.017725991
wavelet-LLH	glrlm	GrayLevelVariance	424.9556502
wavelet-LLH	glrlm	LowGrayLevelRunEmphasis	0.01772945
wavelet-LLH	glrlm	GrayLevelNonUniformityNormalized	0.030482376
wavelet-LLH	glrlm	RunVariance	0.007638962
wavelet-LLH	glrlm	GrayLevelNonUniformity	1.815262139
wavelet-LLH	glrlm	LongRunEmphasis	1.024890364
wavelet-LLH	glrlm	ShortRunHighGrayLevelEmphasis	3512.853874
wavelet-LLH	glrlm	RunLengthNonUniformity	58.68792225
wavelet-LLH	glrlm	ShortRunEmphasis	0.993777409
wavelet-LLH	glrlm	LongRunHighGrayLevelEmphasis	3637.454235
wavelet-LLH	glrlm	RunPercentage	0.992307692
wavelet-LLH	glrlm	LongRunLowGrayLevelEmphasis	0.017743285
wavelet-LLH	glrlm	RunEntropy	5.190230709
wavelet-LLH	glrlm	HighGrayLevelRunEmphasis	3537.773946
wavelet-LLH	glrlm	RunLengthNonUniformityNormalized	0.984722076
wavelet-LLH	glszm	GrayLevelVariance	411.2496571
wavelet-LLH	glszm	ZoneVariance	0.098765432
wavelet-LLH	glszm	GrayLevelNonUniformityNormalized	0.029492455
wavelet-LLH	glszm	SizeZoneNonUniformityNormalized	0.802469136
wavelet-LLH	glszm	SizeZoneNonUniformity	43.33333333
wavelet-LLH	glszm	GrayLevelNonUniformity	1.592592593
wavelet-LLH	glszm	LargeAreaEmphasis	1.333333333
wavelet-LLH	glszm	SmallAreaHighGrayLevelEmphasis	3162.375
wavelet-LLH	glszm	ZonePercentage	0.9
wavelet-LLH	glszm	LargeAreaLowGrayLevelEmphasis	0.019666432
wavelet-LLH	glszm	LargeAreaHighGrayLevelEmphasis	4818.277778
wavelet-LLH	glszm	HighGrayLevelZoneEmphasis	3493.555556
wavelet-LLH	glszm	SmallAreaEmphasis	0.916666667
wavelet-LLH	glszm	LowGrayLevelZoneEmphasis	0.019480344
wavelet-LLH	glszm	ZoneEntropy	5.29646366
wavelet-LLH	glszm	SmallAreaLowGrayLevelEmphasis	0.019433822
wavelet-LLH	ngtdm	Coarseness	0.044246738
wavelet-LLH	ngtdm	Complexity	17046.63459
wavelet-LLH	ngtdm	Strength	90.22021569
wavelet-LLH	ngtdm	Contrast	7.518885139
wavelet-LLH	ngtdm	Busyness	0.01428515
wavelet-LLL	gldm	GrayLevelVariance	814.4941667
wavelet-LLL	gldm	HighGrayLevelEmphasis	2826.016667
wavelet-LLL	gldm	DependenceEntropy	5.548394346
wavelet-LLL	gldm	DependenceNonUniformity	49.2
wavelet-LLL	gldm	GrayLevelNonUniformity	1.533333333
wavelet-LLL	gldm	SmallDependenceEmphasis	0.925
wavelet-LLL	gldm	SmallDependenceHighGrayLevelEmphasis	2735.666667
wavelet-LLL	gldm	DependenceNonUniformityNormalized	0.82
wavelet-LLL	gldm	LargeDependenceEmphasis	1.3
wavelet-LLL	gldm	LargeDependenceLowGrayLevelEmphasis	0.021671288
wavelet-LLL	gldm	DependenceVariance	0.09
wavelet-LLL	gldm	LargeDependenceHighGrayLevelEmphasis	3187.416667
wavelet-LLL	gldm	SmallDependenceLowGrayLevelEmphasis	0.021189494
wavelet-LLL	gldm	LowGrayLevelEmphasis	0.021285853

wavelet-LLL	glcm	JointAverage	51.11402779
wavelet-LLL	glcm	SumAverage	102.2280556
wavelet-LLL	glcm	JointEntropy	5.865795495
wavelet-LLL	glcm	ClusterShade	23207.16963
wavelet-LLL	glcm	MaximumProbability	0.021655176
wavelet-LLL	glcm	Idmn	0.912063932
wavelet-LLL	glcm	JointEnergy	0.017661808
wavelet-LLL	glcm	Contrast	1395.49459
wavelet-LLL	glcm	DifferenceEntropy	4.510070585
wavelet-LLL	glcm	InverseVariance	0.029562599
wavelet-LLL	glcm	DifferenceVariance	415.9935179
wavelet-LLL	glcm	Idn	0.805014644
wavelet-LLL	glcm	Idm	0.025433066
wavelet-LLL	glcm	Correlation	0.240709956
wavelet-LLL	glcm	Autocorrelation	2810.745435
wavelet-LLL	glcm	SumEntropy	4.747217517
wavelet-LLL	glcm	SumSquares	893.8436415
wavelet-LLL	glcm	ClusterProminence	10706019.34
wavelet-LLL	glcm	Imc2	0.999940606
wavelet-LLL	glcm	Imc1	-0.873933256
wavelet-LLL	glcm	DifferenceAverage	30.23393013
wavelet-LLL	glcm	Id	0.073149473
wavelet-LLL	glcm	ClusterTendency	2179.879976
wavelet-LLL	firstorder	InterquartileRange	1075.311374
wavelet-LLL	firstorder	Skewness	0.572146026
wavelet-LLL	firstorder	Uniformity	0.025555556
wavelet-LLL	firstorder	Median	-1602.884109
wavelet-LLL	firstorder	Energy	155369442.7
wavelet-LLL	firstorder	RobustMeanAbsoluteDeviation	451.6997817
wavelet-LLL	firstorder	MeanAbsoluteDeviation	600.0622514
wavelet-LLL	firstorder	TotalEnergy	4194974952
wavelet-LLL	firstorder	Maximum	228.273253
wavelet-LLL	firstorder	RootMeanSquared	1609.189458
wavelet-LLL	firstorder	90Percentile	-363.3988027
wavelet-LLL	firstorder	Minimum	-2540.517178
wavelet-LLL	firstorder	Entropy	5.456564762
wavelet-LLL	firstorder	Range	2768.790431
wavelet-LLL	firstorder	Variance	510226.3644
wavelet-LLL	firstorder	10Percentile	-2178.302594
wavelet-LLL	firstorder	Kurtosis	2.329480468
wavelet-LLL	firstorder	Mean	-1441.965446
wavelet-LLL	glrlm	ShortRunLowGrayLevelEmphasis	0.021361174
wavelet-LLL	glrlm	GrayLevelVariance	816.5707879
wavelet-LLL	glrlm	LowGrayLevelRunEmphasis	0.021364985
wavelet-LLL	glrlm	GrayLevelNonUniformityNormalized	0.025467246
wavelet-LLL	glrlm	RunVariance	0.003842737
wavelet-LLL	glrlm	GrayLevelNonUniformity	1.522270677
wavelet-LLL	glrlm	LongRunEmphasis	1.011868903
wavelet-LLL	glrlm	ShortRunHighGrayLevelEmphasis	2828.837985
wavelet-LLL	glrlm	RunLengthNonUniformity	59.32090995
wavelet-LLL	glrlm	ShortRunEmphasis	0.997032774
wavelet-LLL	glrlm	LongRunHighGrayLevelEmphasis	2846.750671
wavelet-LLL	glrlm	RunPercentage	0.996153846
wavelet-LLL	glrlm	LongRunLowGrayLevelEmphasis	0.021380228
wavelet-LLL	glrlm	RunEntropy	5.464313733

wavelet-LLL	glrlm	HighGrayLevelRunEmphasis	2832.420523
wavelet-LLL	glrlm	RunLengthNonUniformityNormalized	0.992314527
wavelet-LLL	glszm	GrayLevelVariance	841.8990459
wavelet-LLL	glszm	ZoneVariance	0.049861496
wavelet-LLL	glszm	GrayLevelNonUniformityNormalized	0.024315174
wavelet-LLL	glszm	SizeZoneNonUniformityNormalized	0.900277008
wavelet-LLL	glszm	SizeZoneNonUniformity	51.31578947
wavelet-LLL	glszm	GrayLevelNonUniformity	1.385964912
wavelet-LLL	glszm	LargeAreaEmphasis	1.157894737
wavelet-LLL	glszm	SmallAreaHighGrayLevelEmphasis	2863.798246
wavelet-LLL	glszm	ZonePercentage	0.95
wavelet-LLL	glszm	LargeAreaLowGrayLevelEmphasis	0.022541401
wavelet-LLL	glszm	LargeAreaHighGrayLevelEmphasis	3101.561404
wavelet-LLL	glszm	HighGrayLevelZoneEmphasis	2911.350877
wavelet-LLL	glszm	SmallAreaEmphasis	0.960526316
wavelet-LLL	glszm	LowGrayLevelZoneEmphasis	0.022338541
wavelet-LLL	glszm	ZoneEntropy	5.560788698
wavelet-LLL	glszm	SmallAreaLowGrayLevelEmphasis	0.022287825
wavelet-LLL	ngtdm	Coarseness	0.034926679
wavelet-LLL	ngtdm	Complexity	36091.95121
wavelet-LLL	ngtdm	Strength	144.6619222
wavelet-LLL	ngtdm	Contrast	13.97025299
wavelet-LLL	ngtdm	Busyness	0.020119984
wavelet-HHL	gldm	GrayLevelVariance	63.86972222
wavelet-HHL	gldm	HighGrayLevelEmphasis	323.6166667
wavelet-HHL	gldm	DependenceEntropy	4.981727679
wavelet-HHL	gldm	DependenceNonUniformity	27.9
wavelet-HHL	gldm	GrayLevelNonUniformity	3.266666667
wavelet-HHL	gldm	SmallDependenceEmphasis	0.655555556
wavelet-HHL	gldm	SmallDependenceHighGrayLevelEmphasis	247.0509259
wavelet-HHL	gldm	DependenceNonUniformityNormalized	0.465
wavelet-HHL	gldm	LargeDependenceEmphasis	2.6
wavelet-HHL	gldm	LargeDependenceLowGrayLevelEmphasis	0.061477924
wavelet-HHL	gldm	DependenceVariance	0.35
wavelet-HHL	gldm	LargeDependenceHighGrayLevelEmphasis	671.5833333
wavelet-HHL	gldm	SmallDependenceLowGrayLevelEmphasis	0.039258671
wavelet-HHL	gldm	LowGrayLevelEmphasis	0.043283073
wavelet-HHL	glcm	JointAverage	16.47539801
wavelet-HHL	glcm	SumAverage	32.95079602
wavelet-HHL	glcm	JointEntropy	5.751684107
wavelet-HHL	glcm	ClusterShade	484.4321951
wavelet-HHL	glcm	MaximumProbability	0.035921634
wavelet-HHL	glcm	Idmn	0.928416701
wavelet-HHL	glcm	JointEnergy	0.019673067
wavelet-HHL	glcm	Contrast	136.9425462
wavelet-HHL	glcm	DifferenceEntropy	3.765084041
wavelet-HHL	glcm	InverseVariance	0.115774586
wavelet-HHL	glcm	DifferenceVariance	49.37715852
wavelet-HHL	glcm	Idn	0.826748053
wavelet-HHL	glcm	Idm	0.110557773
wavelet-HHL	glcm	Correlation	-0.025621758
wavelet-HHL	glcm	Autocorrelation	271.2049787
wavelet-HHL	glcm	SumEntropy	4.271814768
wavelet-HHL	glcm	SumSquares	68.05451539
wavelet-HHL	glcm	ClusterProminence	56229.38344

wavelet-HHL	glcm	Imc2	0.998407367
wavelet-HHL	glcm	Imc1	-0.673260279
wavelet-HHL	glcm	DifferenceAverage	9.148529344
wavelet-HHL	glcm	Id	0.192905516
wavelet-HHL	glcm	ClusterTendency	135.2755154
wavelet-HHL	firstorder	InterquartileRange	265.7352263
wavelet-HHL	firstorder	Skewness	0.299044399
wavelet-HHL	firstorder	Uniformity	0.054444444
wavelet-HHL	firstorder	Median	2.563085224
wavelet-HHL	firstorder	Energy	2381260.765
wavelet-HHL	firstorder	RobustMeanAbsoluteDeviation	105.8520106
wavelet-HHL	firstorder	MeanAbsoluteDeviation	154.0318734
wavelet-HHL	firstorder	TotalEnergy	64294040.64
wavelet-HHL	firstorder	Maximum	574.7795814
wavelet-HHL	firstorder	RootMeanSquared	199.2176684
wavelet-HHL	firstorder	90Percentile	204.5797632
wavelet-HHL	firstorder	Minimum	-392.0908715
wavelet-HHL	firstorder	Entropy	4.410383097
wavelet-HHL	firstorder	Range	966.870453
wavelet-HHL	firstorder	Variance	39638.52855
wavelet-HHL	firstorder	10Percentile	-279.6895526
wavelet-HHL	firstorder	Kurtosis	3.139816716
wavelet-HHL	firstorder	Mean	-7.010767681
wavelet-HHL	glrlm	ShortRunLowGrayLevelEmphasis	0.043744102
wavelet-HHL	glrlm	GrayLevelVariance	64.31141352
wavelet-HHL	glrlm	LowGrayLevelRunEmphasis	0.043916143
wavelet-HHL	glrlm	GrayLevelNonUniformityNormalized	0.054095576
wavelet-HHL	glrlm	RunVariance	0.019195929
wavelet-HHL	glrlm	GrayLevelNonUniformity	3.183994322
wavelet-HHL	glrlm	LongRunEmphasis	1.059778036
wavelet-HHL	glrlm	ShortRunHighGrayLevelEmphasis	322.42005
wavelet-HHL	glrlm	RunLengthNonUniformity	56.62189068
wavelet-HHL	glrlm	ShortRunEmphasis	0.985055491
wavelet-HHL	glrlm	LongRunHighGrayLevelEmphasis	338.8022063
wavelet-HHL	glrlm	RunPercentage	0.980769231
wavelet-HHL	glrlm	LongRunLowGrayLevelEmphasis	0.044604309
wavelet-HHL	glrlm	RunEntropy	4.465132489
wavelet-HHL	glrlm	HighGrayLevelRunEmphasis	325.6964812
wavelet-HHL	glrlm	RunLengthNonUniformityNormalized	0.961608142
wavelet-HHL	glszm	GrayLevelVariance	70.84246914
wavelet-HHL	glszm	ZoneVariance	0.355555556
wavelet-HHL	glszm	GrayLevelNonUniformityNormalized	0.047901235
wavelet-HHL	glszm	SizeZoneNonUniformityNormalized	0.582222222
wavelet-HHL	glszm	SizeZoneNonUniformity	26.2
wavelet-HHL	glszm	GrayLevelNonUniformity	2.155555556
wavelet-HHL	glszm	LargeAreaEmphasis	2.133333333
wavelet-HHL	glszm	SmallAreaHighGrayLevelEmphasis	311.1123457
wavelet-HHL	glszm	ZonePercentage	0.75
wavelet-HHL	glszm	LargeAreaLowGrayLevelEmphasis	0.066636263
wavelet-HHL	glszm	LargeAreaHighGrayLevelEmphasis	602.8222222
wavelet-HHL	glszm	HighGrayLevelZoneEmphasis	358.3333333
wavelet-HHL	glszm	SmallAreaEmphasis	0.790740741
wavelet-HHL	glszm	LowGrayLevelZoneEmphasis	0.053877188
wavelet-HHL	glszm	ZoneEntropy	4.924969207
wavelet-HHL	glszm	SmallAreaLowGrayLevelEmphasis	0.051386501

wavelet-HHL	ngtdm	Coarseness	0.058277649
wavelet-HHL	ngtdm	Complexity	1949.116576
wavelet-HHL	ngtdm	Strength	20.58526243
wavelet-HHL	ngtdm	Contrast	1.325130883
wavelet-HHL	ngtdm	Busyness	0.053533394
wavelet-HLH	gldm	GrayLevelVariance	230.7141667
wavelet-HLH	gldm	HighGrayLevelEmphasis	1188.616667
wavelet-HLH	gldm	DependenceEntropy	5.202479554
wavelet-HLH	gldm	DependenceNonUniformity	41.7
wavelet-HLH	gldm	GrayLevelNonUniformity	2.2
wavelet-HLH	gldm	SmallDependenceEmphasis	0.860185185
wavelet-HLH	gldm	SmallDependenceHighGrayLevelEmphasis	1069.607407
wavelet-HLH	gldm	DependenceNonUniformityNormalized	0.695
wavelet-HLH	gldm	LargeDependenceEmphasis	1.633333333
wavelet-HLH	gldm	LargeDependenceLowGrayLevelEmphasis	0.01980816
wavelet-HLH	gldm	DependenceVariance	0.193333333
wavelet-HLH	gldm	LargeDependenceHighGrayLevelEmphasis	1710.95
wavelet-HLH	gldm	SmallDependenceLowGrayLevelEmphasis	0.018778032
wavelet-HLH	gldm	LowGrayLevelEmphasis	0.018960354
wavelet-HLH	glcm	JointAverage	31.42324631
wavelet-HLH	glcm	SumAverage	62.84649262
wavelet-HLH	glcm	JointEntropy	5.844122431
wavelet-HLH	glcm	ClusterShade	5213.893335
wavelet-HLH	glcm	MaximumProbability	0.026185405
wavelet-HLH	glcm	Idmn	0.935235261
wavelet-HLH	glcm	JointEnergy	0.018094326
wavelet-HLH	glcm	Contrast	511.9785715
wavelet-HLH	glcm	DifferenceEntropy	4.249979355
wavelet-HLH	glcm	InverseVariance	0.053937314
wavelet-HLH	glcm	DifferenceVariance	181.1827973
wavelet-HLH	glcm	Idn	0.835726479
wavelet-HLH	glcm	Idm	0.050940966
wavelet-HLH	glcm	Correlation	-0.053269994
wavelet-HLH	glcm	Autocorrelation	981.9911672
wavelet-HLH	glcm	SumEntropy	4.485236909
wavelet-HLH	glcm	SumSquares	248.9394349
wavelet-HLH	glcm	ClusterProminence	758147.4392
wavelet-HLH	glcm	Imc2	0.999816397
wavelet-HLH	glcm	Imc1	-0.810372258
wavelet-HLH	glcm	DifferenceAverage	17.56463189
wavelet-HLH	glcm	Id	0.118940434
wavelet-HLH	glcm	ClusterTendency	483.7791682
wavelet-HLH	firstorder	InterquartileRange	495.9360372
wavelet-HLH	firstorder	Skewness	0.806885384
wavelet-HLH	firstorder	Uniformity	0.036666667
wavelet-HLH	firstorder	Median	-49.29247815
wavelet-HLH	firstorder	Energy	8732105.282
wavelet-HLH	firstorder	RobustMeanAbsoluteDeviation	202.3944271
wavelet-HLH	firstorder	MeanAbsoluteDeviation	297.9770323
wavelet-HLH	firstorder	TotalEnergy	235766842.6
wavelet-HLH	firstorder	Maximum	1254.197919
wavelet-HLH	firstorder	RootMeanSquared	381.4906133
wavelet-HLH	firstorder	90Percentile	506.1579766
wavelet-HLH	firstorder	Minimum	-717.2957899
wavelet-HLH	firstorder	Entropy	5.021567004

wavelet-HLH	firstorder	Range	1971.493709
wavelet-HLH	firstorder	Variance	144340.5338
wavelet-HLH	firstorder	10Percentile	-350.4198311
wavelet-HLH	firstorder	Kurtosis	3.788470052
wavelet-HLH	firstorder	Mean	34.56232372
wavelet-HLH	glrlm	ShortRunLowGrayLevelEmphasis	0.019091287
wavelet-HLH	glrlm	GrayLevelVariance	232.2921521
wavelet-HLH	glrlm	LowGrayLevelRunEmphasis	0.019099104
wavelet-HLH	glrlm	GrayLevelNonUniformityNormalized	0.036535401
wavelet-HLH	glrlm	RunVariance	0.007687786
wavelet-HLH	glrlm	GrayLevelNonUniformity	2.175363036
wavelet-HLH	glrlm	LongRunEmphasis	1.023602931
wavelet-HLH	glrlm	ShortRunHighGrayLevelEmphasis	1186.470544
wavelet-HLH	glrlm	RunLengthNonUniformity	58.63642494
wavelet-HLH	glrlm	ShortRunEmphasis	0.994099267
wavelet-HLH	glrlm	LongRunHighGrayLevelEmphasis	1211.108548
wavelet-HLH	glrlm	RunPercentage	0.992307692
wavelet-HLH	glrlm	LongRunLowGrayLevelEmphasis	0.019130373
wavelet-HLH	glrlm	RunEntropy	5.035963519
wavelet-HLH	glrlm	HighGrayLevelRunEmphasis	1191.398145
wavelet-HLH	glrlm	RunLengthNonUniformityNormalized	0.984624429
wavelet-HLH	glszm	GrayLevelVariance	252.9876543
wavelet-HLH	glszm	ZoneVariance	0.135802469
wavelet-HLH	glszm	GrayLevelNonUniformityNormalized	0.034979424
wavelet-HLH	glszm	SizeZoneNonUniformityNormalized	0.829218107
wavelet-HLH	glszm	SizeZoneNonUniformity	44.77777778
wavelet-HLH	glszm	GrayLevelNonUniformity	1.888888889
wavelet-HLH	glszm	LargeAreaEmphasis	1.37037037
wavelet-HLH	glszm	SmallAreaHighGrayLevelEmphasis	1165.235082
wavelet-HLH	glszm	ZonePercentage	0.9
wavelet-HLH	glszm	LargeAreaLowGrayLevelEmphasis	0.021420568
wavelet-HLH	glszm	LargeAreaHighGrayLevelEmphasis	1529.574074
wavelet-HLH	glszm	HighGrayLevelZoneEmphasis	1227.814815
wavelet-HLH	glszm	SmallAreaEmphasis	0.927983539
wavelet-HLH	glszm	LowGrayLevelZoneEmphasis	0.020919935
wavelet-HLH	glszm	ZoneEntropy	5.157393752
wavelet-HLH	glszm	SmallAreaLowGrayLevelEmphasis	0.020827699
wavelet-HLH	ngtdm	Coarseness	0.045467227
wavelet-HLH	ngtdm	Complexity	10646.15021
wavelet-HLH	ngtdm	Strength	57.70368743
wavelet-HLH	ngtdm	Contrast	3.986149017
wavelet-HLH	ngtdm	Busyness	0.02770357

Image type	Feature Class	Feature Name	16: RTSTRUCT: C99-DNU_B_segment_Tumor
general	info	BoundingBox	(1, 1, 1, 5, 8, 6)
general	info	EnabledImageTypes	{u'Original': {}, u'Wavelet': {}}
general	info	GeneralSettings	{'distances': [1], 'additionalInfo': True, 'force2D': False, 'interpolator': 'sitkBSpline', 'resampledPixelSpacing': [3.0], 'label': 1, 'normalizeScale': 1, 'normalize': False, 'force2Ddimension': 0, 'removeOutliers': None, 'minimumROISize': None, u'binWidth': 25.0, 'minimumROIDimensions': 1, u'correctMask': True, 'preCrop': False, u'symmetricalGLCM': True, 'resegmentRange': None, 'padDistance': 5}
general	info	ImageHash	8ee41fdaecc9482d953734bd399d19c868ee569e
general	info	ImageSpacing	(0.9765625000000002, 0.9765625000000002, 2.0)
general	info	MaskHash	5e666ca43e0f316bc2a2dcace68d277e9cbbb681
general	info	NumpyVersion	1.13.1
general	info	PyWaveletVersion	0.5.2
general	info	SimpleITKVersion	1.1.0
general	info	Version	1.3.0.post102+g1b0eb74
general	info	VolumeNum	1
general	info	VoxelNum	99
original	shape	Maximum3DDiameter	26.4952826
original	shape	Maximum2DDiameterSlice	16.97056275
original	shape	Sphericity	0.802388048
original	shape	MinorAxis	15.63024906
original	shape	Elongation	0.609399113
original	shape	SurfaceVolumeRatio	0.434276858
original	shape	Volume	2673
original	shape	MajorAxis	25.64862455
original	shape	SurfaceArea	1160.822042
original	shape	Flatness	0.386163754
original	shape	LeastAxis	9.904569144
original	shape	Maximum2DDiameterColumn	16.15549442
original	shape	Maximum2DDiameterRow	25.8069758
original	gldm	GrayLevelVariance	150.426079
original	gldm	HighGrayLevelEmphasis	866.3939394
original	gldm	DependenceEntropy	5.622803686
original	gldm	DependenceNonUniformity	48.83838384
original	gldm	GrayLevelNonUniformity	3.888888889
original	gldm	SmallDependenceEmphasis	0.727502806
original	gldm	SmallDependenceHighGrayLevelEmphasis	563.473931
original	gldm	DependenceNonUniformityNormalized	0.493317008
original	gldm	LargeDependenceEmphasis	4.050505051
original	gldm	LargeDependenceLowGrayLevelEmphasis	0.020303989
original	gldm	DependenceVariance	1.405774921
original	gldm	LargeDependenceHighGrayLevelEmphasis	4616.878788
original	gldm	SmallDependenceLowGrayLevelEmphasis	0.015764239
original	gldm	LowGrayLevelEmphasis	0.016366762
original	glcm	JointAverage	29.33855051

original	glcm	SumAverage	58.67710102
original	glcm	JointEntropy	6.597148813
original	glcm	ClusterShade	-2405.584576
original	glcm	MaximumProbability	0.027408226
original	glcm	Idmn	0.944838185
original	glcm	JointEnergy	0.01120329
original	glcm	Contrast	242.5218306
original	glcm	DifferenceEntropy	4.342963063
original	glcm	InverseVariance	0.128541789
original	glcm	DifferenceVariance	104.1540996
original	glcm	Idn	0.854483198
original	glcm	Idm	0.120223844
original	glcm	Correlation	0.11999913
original	glcm	Autocorrelation	878.8299179
original	glcm	SumEntropy	5.052429204
original	glcm	SumSquares	139.0540516
original	glcm	ClusterProminence	310915.2618
original	glcm	Imc2	0.998654164
original	glcm	Imc1	-0.621617569
original	glcm	DifferenceAverage	11.55144204
original	glcm	Id	0.193180874
original	glcm	ClusterTendency	313.6943757
original	firstorder	InterquartileRange	453
original	firstorder	Skewness	-0.262264386
original	firstorder	Uniformity	0.039281706
original	firstorder	Median	-156
original	firstorder	Energy	14025431
original	firstorder	RobustMeanAbsoluteDeviation	187.3581157
original	firstorder	MeanAbsoluteDeviation	251.0921335
original	firstorder	TotalEnergy	378686637
original	firstorder	Maximum	606
original	firstorder	RootMeanSquared	376.3921096
original	firstorder	90Percentile	59.2
original	firstorder	Minimum	-875
original	firstorder	Entropy	4.990609934
original	firstorder	Range	1481
original	firstorder	Variance	93648.06081
original	firstorder	10Percentile	-700.8
original	firstorder	Kurtosis	2.614118928
original	firstorder	Mean	-219.1414141
original	glrlm	ShortRunLowGrayLevelEmphasis	0.016702833
original	glrlm	GrayLevelVariance	151.7401869
original	glrlm	LowGrayLevelRunEmphasis	0.01673271
original	glrlm	GrayLevelNonUniformityNormalized	0.038009004
original	glrlm	RunVariance	0.024062801
original	glrlm	GrayLevelNonUniformity	3.672714402
original	glrlm	LongRunEmphasis	1.07437946
original	glrlm	ShortRunHighGrayLevelEmphasis	836.3579648
original	glrlm	RunLengthNonUniformity	91.98596743
original	glrlm	ShortRunEmphasis	0.981405135
original	glrlm	LongRunHighGrayLevelEmphasis	946.7507055
original	glrlm	RunPercentage	0.975912976
original	glrlm	LongRunLowGrayLevelEmphasis	0.016852218
original	glrlm	RunEntropy	5.09620068
original	glrlm	HighGrayLevelRunEmphasis	858.436513

original	glrlm	RunLengthNonUniformityNormalized	0.951874398
original	glszm	GrayLevelVariance	161.9421433
original	glszm	ZoneVariance	0.658284024
original	glszm	GrayLevelNonUniformityNormalized	0.032215648
original	glszm	SizeZoneNonUniformityNormalized	0.72748192
original	glszm	SizeZoneNonUniformity	56.74358974
original	glszm	GrayLevelNonUniformity	2.512820513
original	glszm	LargeAreaEmphasis	2.269230769
original	glszm	SmallAreaHighGrayLevelEmphasis	664.8968519
original	glszm	ZonePercentage	0.787878788
original	glszm	LargeAreaLowGrayLevelEmphasis	0.022226226
original	glszm	LargeAreaHighGrayLevelEmphasis	2298.320513
original	glszm	HighGrayLevelZoneEmphasis	795.9487179
original	glszm	SmallAreaEmphasis	0.875512821
original	glszm	LowGrayLevelZoneEmphasis	0.020230475
original	glszm	ZoneEntropy	5.437466764
original	glszm	SmallAreaLowGrayLevelEmphasis	0.01988754
original	ngtdm	Coarseness	0.045394094
original	ngtdm	Complexity	5866.883644
original	ngtdm	Strength	36.72264162
original	ngtdm	Contrast	1.536949873
original	ngtdm	Busyness	0.019783563
wavelet-HHH	gldm	GrayLevelVariance	42.69605142
wavelet-HHH	gldm	HighGrayLevelEmphasis	319.4646465
wavelet-HHH	gldm	DependenceEntropy	5.356234398
wavelet-HHH	gldm	DependenceNonUniformity	43.88888889
wavelet-HHH	gldm	GrayLevelNonUniformity	4.737373737
wavelet-HHH	gldm	SmallDependenceEmphasis	0.660634119
wavelet-HHH	gldm	SmallDependenceHighGrayLevelEmphasis	228.4287318
wavelet-HHH	gldm	DependenceNonUniformityNormalized	0.44332211
wavelet-HHH	gldm	LargeDependenceEmphasis	2.898989899
wavelet-HHH	gldm	LargeDependenceLowGrayLevelEmphasis	0.030264713
wavelet-HHH	gldm	DependenceVariance	0.510560147
wavelet-HHH	gldm	LargeDependenceHighGrayLevelEmphasis	837.7272727
wavelet-HHH	gldm	SmallDependenceLowGrayLevelEmphasis	0.018022565
wavelet-HHH	gldm	LowGrayLevelEmphasis	0.019970394
wavelet-HHH	glcm	JointAverage	16.54534394
wavelet-HHH	glcm	SumAverage	33.09068788
wavelet-HHH	glcm	JointEntropy	6.606591975
wavelet-HHH	glcm	ClusterShade	-60.27930501
wavelet-HHH	glcm	MaximumProbability	0.023076749
wavelet-HHH	glcm	Idmn	0.925969317
wavelet-HHH	glcm	JointEnergy	0.010968068
wavelet-HHH	glcm	Contrast	87.10449837
wavelet-HHH	glcm	DifferenceEntropy	3.904287732
wavelet-HHH	glcm	InverseVariance	0.121407655
wavelet-HHH	glcm	DifferenceVariance	26.54754229
wavelet-HHH	glcm	Idn	0.819839033
wavelet-HHH	glcm	Idm	0.114854629
wavelet-HHH	glcm	Correlation	-0.046849229
wavelet-HHH	glcm	Autocorrelation	271.9861082
wavelet-HHH	glcm	SumEntropy	4.610641583
wavelet-HHH	glcm	SumSquares	41.6887344
wavelet-HHH	glcm	ClusterProminence	17469.36596
wavelet-HHH	glcm	Imc2	0.995061098

wavelet-HHH	glcm	Imc1	-0.523180658
wavelet-HHH	glcm	DifferenceAverage	7.534050567
wavelet-HHH	glcm	Id	0.204910864
wavelet-HHH	glcm	ClusterTendency	79.65043923
wavelet-HHH	firstorder	InterquartileRange	224.329929
wavelet-HHH	firstorder	Skewness	-0.161407594
wavelet-HHH	firstorder	Uniformity	0.04785226
wavelet-HHH	firstorder	Median	3.963701704
wavelet-HHH	firstorder	Energy	2625745.701
wavelet-HHH	firstorder	RobustMeanAbsoluteDeviation	92.71242242
wavelet-HHH	firstorder	MeanAbsoluteDeviation	131.3419986
wavelet-HHH	firstorder	TotalEnergy	70895133.93
wavelet-HHH	firstorder	Maximum	359.0765791
wavelet-HHH	firstorder	RootMeanSquared	162.8578639
wavelet-HHH	firstorder	90Percentile	219.7136175
wavelet-HHH	firstorder	Minimum	-380.9262747
wavelet-HHH	firstorder	Entropy	4.548677107
wavelet-HHH	firstorder	Range	740.0028538
wavelet-HHH	firstorder	Variance	26513.02052
wavelet-HHH	firstorder	10Percentile	-219.7370182
wavelet-HHH	firstorder	Kurtosis	2.590551361
wavelet-HHH	firstorder	Mean	3.108589994
wavelet-HHH	glrlm	ShortRunLowGrayLevelEmphasis	0.020192347
wavelet-HHH	glrlm	GrayLevelVariance	43.08367999
wavelet-HHH	glrlm	LowGrayLevelRunEmphasis	0.020282702
wavelet-HHH	glrlm	GrayLevelNonUniformityNormalized	0.047708497
wavelet-HHH	glrlm	RunVariance	0.02095306
wavelet-HHH	glrlm	GrayLevelNonUniformity	4.624177964
wavelet-HHH	glrlm	LongRunEmphasis	1.065013344
wavelet-HHH	glrlm	ShortRunHighGrayLevelEmphasis	316.0821883
wavelet-HHH	glrlm	RunLengthNonUniformity	92.90626535
wavelet-HHH	glrlm	ShortRunEmphasis	0.983746664
wavelet-HHH	glrlm	LongRunHighGrayLevelEmphasis	338.1656451
wavelet-HHH	glrlm	RunPercentage	0.979020979
wavelet-HHH	glrlm	LongRunLowGrayLevelEmphasis	0.020644119
wavelet-HHH	glrlm	RunEntropy	4.619159648
wavelet-HHH	glrlm	HighGrayLevelRunEmphasis	320.4988797
wavelet-HHH	glrlm	RunLengthNonUniformityNormalized	0.958093881
wavelet-HHH	glszm	GrayLevelVariance	48.13221329
wavelet-HHH	glszm	ZoneVariance	0.439919649
wavelet-HHH	glszm	GrayLevelNonUniformityNormalized	0.045288532
wavelet-HHH	glszm	SizeZoneNonUniformityNormalized	0.603725347
wavelet-HHH	glszm	SizeZoneNonUniformity	44.67567568
wavelet-HHH	glszm	GrayLevelNonUniformity	3.351351351
wavelet-HHH	glszm	LargeAreaEmphasis	2.22972973
wavelet-HHH	glszm	SmallAreaHighGrayLevelEmphasis	284.1865616
wavelet-HHH	glszm	ZonePercentage	0.747474747
wavelet-HHH	glszm	LargeAreaLowGrayLevelEmphasis	0.031639406
wavelet-HHH	glszm	LargeAreaHighGrayLevelEmphasis	676.2567568
wavelet-HHH	glszm	HighGrayLevelZoneEmphasis	335.2972973
wavelet-HHH	glszm	SmallAreaEmphasis	0.805649399
wavelet-HHH	glszm	LowGrayLevelZoneEmphasis	0.02479501
wavelet-HHH	glszm	ZoneEntropy	5.154870933
wavelet-HHH	glszm	SmallAreaLowGrayLevelEmphasis	0.023655295
wavelet-HHH	ngtdm	Coarseness	0.046947412

wavelet-HHH	ngtdm	Complexity	1678.419397
wavelet-HHH	ngtdm	Strength	11.76616792
wavelet-HHH	ngtdm	Contrast	0.636337189
wavelet-HHH	ngtdm	Busyness	0.058709907
wavelet-HLL	gldm	GrayLevelVariance	148.107336
wavelet-HLL	gldm	HighGrayLevelEmphasis	968.7272727
wavelet-HLL	gldm	DependenceEntropy	5.591033514
wavelet-HLL	gldm	DependenceNonUniformity	46.15151515
wavelet-HLL	gldm	GrayLevelNonUniformity	3
wavelet-HLL	gldm	SmallDependenceEmphasis	0.685746352
wavelet-HLL	gldm	SmallDependenceHighGrayLevelEmphasis	634.4938272
wavelet-HLL	gldm	DependenceNonUniformityNormalized	0.466176921
wavelet-HLL	gldm	LargeDependenceEmphasis	2.616161616
wavelet-HLL	gldm	LargeDependenceLowGrayLevelEmphasis	0.017269479
wavelet-HLL	gldm	DependenceVariance	0.411386593
wavelet-HLL	gldm	LargeDependenceHighGrayLevelEmphasis	2726.222222
wavelet-HLL	gldm	SmallDependenceLowGrayLevelEmphasis	0.013527409
wavelet-HLL	gldm	LowGrayLevelEmphasis	0.014170671
wavelet-HLL	glcm	JointAverage	28.13678437
wavelet-HLL	glcm	SumAverage	56.27356874
wavelet-HLL	glcm	JointEntropy	6.696017053
wavelet-HLL	glcm	ClusterShade	-204.0655848
wavelet-HLL	glcm	MaximumProbability	0.018469844
wavelet-HLL	glcm	Idmn	0.927730517
wavelet-HLL	glcm	JointEnergy	0.010133459
wavelet-HLL	glcm	Contrast	216.09303
wavelet-HLL	glcm	DifferenceEntropy	4.291157288
wavelet-HLL	glcm	InverseVariance	0.095496911
wavelet-HLL	glcm	DifferenceVariance	67.99851039
wavelet-HLL	glcm	Idn	0.827577569
wavelet-HLL	glcm	Idm	0.091558779
wavelet-HLL	glcm	Correlation	0.259302051
wavelet-HLL	glcm	Autocorrelation	828.3070256
wavelet-HLL	glcm	SumEntropy	5.139071094
wavelet-HLL	glcm	SumSquares	143.9483074
wavelet-HLL	glcm	ClusterProminence	280351.1149
wavelet-HLL	glcm	Imc2	0.999542749
wavelet-HLL	glcm	Imc1	-0.694795624
wavelet-HLL	glcm	DifferenceAverage	11.46467348
wavelet-HLL	glcm	Id	0.168883998
wavelet-HLL	glcm	ClusterTendency	359.7001998
wavelet-HLL	firstorder	InterquartileRange	523.5405897
wavelet-HLL	firstorder	Skewness	-0.19812744
wavelet-HLL	firstorder	Uniformity	0.03030303
wavelet-HLL	firstorder	Median	-64.99480659
wavelet-HLL	firstorder	Energy	9699335.611
wavelet-HLL	firstorder	RobustMeanAbsoluteDeviation	206.4903397
wavelet-HLL	firstorder	MeanAbsoluteDeviation	262.7871434
wavelet-HLL	firstorder	TotalEnergy	261882061.5
wavelet-HLL	firstorder	Maximum	441.7338557
wavelet-HLL	firstorder	RootMeanSquared	313.0065287
wavelet-HLL	firstorder	90Percentile	318.2085209
wavelet-HLL	firstorder	Minimum	-750.5737667
wavelet-HLL	firstorder	Entropy	5.216089923
wavelet-HLL	firstorder	Range	1192.307622

wavelet-HLL	firstorder	Variance	92914.25026
wavelet-HLL	firstorder	10Percentile	-454.4114268
wavelet-HLL	firstorder	Kurtosis	2.007848112
wavelet-HLL	firstorder	Mean	-71.1254998
wavelet-HLL	glrlm	ShortRunLowGrayLevelEmphasis	0.014376664
wavelet-HLL	glrlm	GrayLevelVariance	148.5488256
wavelet-HLL	glrlm	LowGrayLevelRunEmphasis	0.014404992
wavelet-HLL	glrlm	GrayLevelNonUniformityNormalized	0.030218721
wavelet-HLL	glrlm	RunVariance	0.018627892
wavelet-HLL	glrlm	GrayLevelNonUniformity	2.935981255
wavelet-HLL	glrlm	LongRunEmphasis	1.057603112
wavelet-HLL	glrlm	ShortRunHighGrayLevelEmphasis	951.0517263
wavelet-HLL	glrlm	RunLengthNonUniformity	93.57103617
wavelet-HLL	glrlm	ShortRunEmphasis	0.985599222
wavelet-HLL	glrlm	LongRunHighGrayLevelEmphasis	1028.786259
wavelet-HLL	glrlm	RunPercentage	0.981351981
wavelet-HLL	glrlm	LongRunLowGrayLevelEmphasis	0.014518305
wavelet-HLL	glrlm	RunEntropy	5.261431967
wavelet-HLL	glrlm	HighGrayLevelRunEmphasis	966.5986327
wavelet-HLL	glrlm	RunLengthNonUniformityNormalized	0.962744216
wavelet-HLL	glszm	GrayLevelVariance	156.4415584
wavelet-HLL	glszm	ZoneVariance	0.307977737
wavelet-HLL	glszm	GrayLevelNonUniformityNormalized	0.029178614
wavelet-HLL	glszm	SizeZoneNonUniformityNormalized	0.622870636
wavelet-HLL	glszm	SizeZoneNonUniformity	47.96103896
wavelet-HLL	glszm	GrayLevelNonUniformity	2.246753247
wavelet-HLL	glszm	LargeAreaEmphasis	1.961038961
wavelet-HLL	glszm	SmallAreaHighGrayLevelEmphasis	748.1190476
wavelet-HLL	glszm	ZonePercentage	0.777777778
wavelet-HLL	glszm	LargeAreaLowGrayLevelEmphasis	0.019495957
wavelet-HLL	glszm	LargeAreaHighGrayLevelEmphasis	1975.012987
wavelet-HLL	glszm	HighGrayLevelZoneEmphasis	940.4415584
wavelet-HLL	glszm	SmallAreaEmphasis	0.817460317
wavelet-HLL	glszm	LowGrayLevelZoneEmphasis	0.017648491
wavelet-HLL	glszm	ZoneEntropy	5.532639463
wavelet-HLL	glszm	SmallAreaLowGrayLevelEmphasis	0.017261422
wavelet-HLL	ngtdm	Coarseness	0.04969368
wavelet-HLL	ngtdm	Complexity	5536.214119
wavelet-HLL	ngtdm	Strength	39.81846128
wavelet-HLL	ngtdm	Contrast	1.195302708
wavelet-HLL	ngtdm	Busyness	0.019084618
wavelet-LHL	gldm	GrayLevelVariance	81.64554637
wavelet-LHL	gldm	HighGrayLevelEmphasis	943.8686869
wavelet-LHL	gldm	DependenceEntropy	5.533100812
wavelet-LHL	gldm	DependenceNonUniformity	38.17171717
wavelet-LHL	gldm	GrayLevelNonUniformity	4.131313131
wavelet-LHL	gldm	SmallDependenceEmphasis	0.617213805
wavelet-LHL	gldm	SmallDependenceHighGrayLevelEmphasis	639.4501964
wavelet-LHL	gldm	DependenceNonUniformityNormalized	0.385572901
wavelet-LHL	gldm	LargeDependenceEmphasis	3.545454545
wavelet-LHL	gldm	LargeDependenceLowGrayLevelEmphasis	0.015687139
wavelet-LHL	gldm	DependenceVariance	0.699928579
wavelet-LHL	gldm	LargeDependenceHighGrayLevelEmphasis	2881.343434
wavelet-LHL	gldm	SmallDependenceLowGrayLevelEmphasis	0.010946561
wavelet-LHL	gldm	LowGrayLevelEmphasis	0.011599459

wavelet-LHL	glcm	JointAverage	28.72570077
wavelet-LHL	glcm	SumAverage	57.45140155
wavelet-LHL	glcm	JointEntropy	6.616020208
wavelet-LHL	glcm	ClusterShade	768.3058902
wavelet-LHL	glcm	MaximumProbability	0.021333109
wavelet-LHL	glcm	Idmn	0.954460163
wavelet-LHL	glcm	JointEnergy	0.010947923
wavelet-LHL	glcm	Contrast	146.2107511
wavelet-LHL	glcm	DifferenceEntropy	4.099879137
wavelet-LHL	glcm	InverseVariance	0.14620896
wavelet-LHL	glcm	DifferenceVariance	56.87483536
wavelet-LHL	glcm	Idn	0.865796048
wavelet-LHL	glcm	Idm	0.131908088
wavelet-LHL	glcm	Correlation	0.158280245
wavelet-LHL	glcm	Autocorrelation	838.1854478
wavelet-LHL	glcm	SumEntropy	4.901268924
wavelet-LHL	glcm	SumSquares	85.83935573
wavelet-LHL	glcm	ClusterProminence	137009.8968
wavelet-LHL	glcm	Imc2	0.99845625
wavelet-LHL	glcm	Imc1	-0.620839821
wavelet-LHL	glcm	DifferenceAverage	8.994734423
wavelet-LHL	glcm	Id	0.212211909
wavelet-LHL	glcm	ClusterTendency	197.1466718
wavelet-LHL	firstorder	InterquartileRange	277.7851018
wavelet-LHL	firstorder	Skewness	0.155315007
wavelet-LHL	firstorder	Uniformity	0.041730436
wavelet-LHL	firstorder	Median	-145.5285739
wavelet-LHL	firstorder	Energy	6623583.026
wavelet-LHL	firstorder	RobustMeanAbsoluteDeviation	111.7516696
wavelet-LHL	firstorder	MeanAbsoluteDeviation	171.5286331
wavelet-LHL	firstorder	TotalEnergy	178836741.7
wavelet-LHL	firstorder	Maximum	440.6515827
wavelet-LHL	firstorder	RootMeanSquared	258.6597747
wavelet-LHL	firstorder	90Percentile	150.1670047
wavelet-LHL	firstorder	Minimum	-827.6953619
wavelet-LHL	firstorder	Entropy	4.83434049
wavelet-LHL	firstorder	Range	1268.346945
wavelet-LHL	firstorder	Variance	50508.09855
wavelet-LHL	firstorder	10Percentile	-381.4434595
wavelet-LHL	firstorder	Kurtosis	3.456229264
wavelet-LHL	firstorder	Mean	-128.0499141
wavelet-LHL	glrlm	ShortRunLowGrayLevelEmphasis	0.011838492
wavelet-LHL	glrlm	GrayLevelVariance	82.62048772
wavelet-LHL	glrlm	LowGrayLevelRunEmphasis	0.011872266
wavelet-LHL	glrlm	GrayLevelNonUniformityNormalized	0.041146747
wavelet-LHL	glrlm	RunVariance	0.026374734
wavelet-LHL	glrlm	GrayLevelNonUniformity	3.966084127
wavelet-LHL	glrlm	LongRunEmphasis	1.082203302
wavelet-LHL	glrlm	ShortRunHighGrayLevelEmphasis	932.6726671
wavelet-LHL	glrlm	RunLengthNonUniformity	91.34849485
wavelet-LHL	glrlm	ShortRunEmphasis	0.979449175
wavelet-LHL	glrlm	LongRunHighGrayLevelEmphasis	1012.08494
wavelet-LHL	glrlm	RunPercentage	0.973581974
wavelet-LHL	glrlm	LongRunLowGrayLevelEmphasis	0.012007359
wavelet-LHL	glrlm	RunEntropy	4.927843116

wavelet-LHL	glrlm	HighGrayLevelRunEmphasis	948.5551217
wavelet-LHL	glrlm	RunLengthNonUniformityNormalized	0.947250531
wavelet-LHL	glszm	GrayLevelVariance	93.73387755
wavelet-LHL	glszm	ZoneVariance	0.785510204
wavelet-LHL	glszm	GrayLevelNonUniformityNormalized	0.035510204
wavelet-LHL	glszm	SizeZoneNonUniformityNormalized	0.584081633
wavelet-LHL	glszm	SizeZoneNonUniformity	40.88571429
wavelet-LHL	glszm	GrayLevelNonUniformity	2.485714286
wavelet-LHL	glszm	LargeAreaEmphasis	2.785714286
wavelet-LHL	glszm	SmallAreaHighGrayLevelEmphasis	839.3958333
wavelet-LHL	glszm	ZonePercentage	0.707070707
wavelet-LHL	glszm	LargeAreaLowGrayLevelEmphasis	0.018535083
wavelet-LHL	glszm	LargeAreaHighGrayLevelEmphasis	2390.214286
wavelet-LHL	glszm	HighGrayLevelZoneEmphasis	1009.228571
wavelet-LHL	glszm	SmallAreaEmphasis	0.79265873
wavelet-LHL	glszm	LowGrayLevelZoneEmphasis	0.01571317
wavelet-LHL	glszm	ZoneEntropy	5.422000517
wavelet-LHL	glszm	SmallAreaLowGrayLevelEmphasis	0.015342663
wavelet-LHL	ngtdm	Coarseness	0.056030875
wavelet-LHL	ngtdm	Complexity	2949.75307
wavelet-LHL	ngtdm	Strength	27.51690679
wavelet-LHL	ngtdm	Contrast	0.800491128
wavelet-LHL	ngtdm	Busyness	0.02336158
wavelet-LHH	gldm	GrayLevelVariance	29.78104275
wavelet-LHH	gldm	HighGrayLevelEmphasis	182.1414141
wavelet-LHH	gldm	DependenceEntropy	5.228666817
wavelet-LHH	gldm	DependenceNonUniformity	39.4040404
wavelet-LHH	gldm	GrayLevelNonUniformity	5.98989899
wavelet-LHH	gldm	SmallDependenceEmphasis	0.62191358
wavelet-LHH	gldm	SmallDependenceHighGrayLevelEmphasis	125.1820988
wavelet-LHH	gldm	DependenceNonUniformityNormalized	0.39802061
wavelet-LHH	gldm	LargeDependenceEmphasis	3.323232323
wavelet-LHH	gldm	LargeDependenceLowGrayLevelEmphasis	0.05072414
wavelet-LHH	gldm	DependenceVariance	0.612386491
wavelet-LHH	gldm	LargeDependenceHighGrayLevelEmphasis	511.5555556
wavelet-LHH	gldm	SmallDependenceLowGrayLevelEmphasis	0.018041661
wavelet-LHH	gldm	LowGrayLevelEmphasis	0.022779123
wavelet-LHH	glcm	JointAverage	12.51474469
wavelet-LHH	glcm	SumAverage	25.02948937
wavelet-LHH	glcm	JointEntropy	6.490563549
wavelet-LHH	glcm	ClusterShade	259.6084517
wavelet-LHH	glcm	MaximumProbability	0.022920661
wavelet-LHH	glcm	Idmn	0.943387851
wavelet-LHH	glcm	JointEnergy	0.012112669
wavelet-LHH	glcm	Contrast	69.56352217
wavelet-LHH	glcm	DifferenceEntropy	3.793656823
wavelet-LHH	glcm	InverseVariance	0.146788012
wavelet-LHH	glcm	DifferenceVariance	24.15328235
wavelet-LHH	glcm	Idn	0.842969115
wavelet-LHH	glcm	Idm	0.134177907
wavelet-LHH	glcm	Correlation	-0.023845552
wavelet-LHH	glcm	Autocorrelation	155.8038689
wavelet-LHH	glcm	SumEntropy	4.438482558
wavelet-LHH	glcm	SumSquares	33.96008121
wavelet-LHH	glcm	ClusterProminence	15533.25917

wavelet-LHH	glcm	Imc2	0.990413968
wavelet-LHH	glcm	Imc1	-0.478699253
wavelet-LHH	glcm	DifferenceAverage	6.568875609
wavelet-LHH	glcm	Id	0.226910933
wavelet-LHH	glcm	ClusterTendency	66.27680268
wavelet-LHH	firstorder	InterquartileRange	150.0101886
wavelet-LHH	firstorder	Skewness	0.568552325
wavelet-LHH	firstorder	Uniformity	0.06050403
wavelet-LHH	firstorder	Median	-7.459750291
wavelet-LHH	firstorder	Energy	1823290.949
wavelet-LHH	firstorder	RobustMeanAbsoluteDeviation	73.57270779
wavelet-LHH	firstorder	MeanAbsoluteDeviation	106.0228143
wavelet-LHH	firstorder	TotalEnergy	49228855.63
wavelet-LHH	firstorder	Maximum	497.0749039
wavelet-LHH	firstorder	RootMeanSquared	135.7095439
wavelet-LHH	firstorder	90Percentile	178.1289662
wavelet-LHH	firstorder	Minimum	-296.9991187
wavelet-LHH	firstorder	Entropy	4.230233142
wavelet-LHH	firstorder	Range	794.0740226
wavelet-LHH	firstorder	Variance	18397.91699
wavelet-LHH	firstorder	10Percentile	-179.6203419
wavelet-LHH	firstorder	Kurtosis	3.815130106
wavelet-LHH	firstorder	Mean	-4.377591441
wavelet-LHH	glrlm	ShortRunLowGrayLevelEmphasis	0.022816078
wavelet-LHH	glrlm	GrayLevelVariance	30.1667195
wavelet-LHH	glrlm	LowGrayLevelRunEmphasis	0.023052145
wavelet-LHH	glrlm	GrayLevelNonUniformityNormalized	0.060109399
wavelet-LHH	glrlm	RunVariance	0.024816733
wavelet-LHH	glrlm	GrayLevelNonUniformity	5.802980137
wavelet-LHH	glrlm	LongRunEmphasis	1.077409961
wavelet-LHH	glrlm	ShortRunHighGrayLevelEmphasis	180.2646741
wavelet-LHH	glrlm	RunLengthNonUniformity	91.80136515
wavelet-LHH	glrlm	ShortRunEmphasis	0.98064751
wavelet-LHH	glrlm	LongRunHighGrayLevelEmphasis	194.3573741
wavelet-LHH	glrlm	RunPercentage	0.975135975
wavelet-LHH	glrlm	LongRunLowGrayLevelEmphasis	0.023996411
wavelet-LHH	glrlm	RunEntropy	4.333208474
wavelet-LHH	glrlm	HighGrayLevelRunEmphasis	183.0832141
wavelet-LHH	glrlm	RunLengthNonUniformityNormalized	0.950366534
wavelet-LHH	glszm	GrayLevelVariance	34.87839714
wavelet-LHH	glszm	ZoneVariance	0.605038683
wavelet-LHH	glszm	GrayLevelNonUniformityNormalized	0.055346161
wavelet-LHH	glszm	SizeZoneNonUniformityNormalized	0.573497322
wavelet-LHH	glszm	SizeZoneNonUniformity	40.71830986
wavelet-LHH	glszm	GrayLevelNonUniformity	3.929577465
wavelet-LHH	glszm	LargeAreaEmphasis	2.549295775
wavelet-LHH	glszm	SmallAreaHighGrayLevelEmphasis	162.5511894
wavelet-LHH	glszm	ZonePercentage	0.717171717
wavelet-LHH	glszm	LargeAreaLowGrayLevelEmphasis	0.047969511
wavelet-LHH	glszm	LargeAreaHighGrayLevelEmphasis	404
wavelet-LHH	glszm	HighGrayLevelZoneEmphasis	196.6338028
wavelet-LHH	glszm	SmallAreaEmphasis	0.785872457
wavelet-LHH	glszm	LowGrayLevelZoneEmphasis	0.026687751
wavelet-LHH	glszm	ZoneEntropy	5.066735106
wavelet-LHH	glszm	SmallAreaLowGrayLevelEmphasis	0.024045343

wavelet-LHH	ngtdm	Coarseness	0.046358852
wavelet-LHH	ngtdm	Complexity	1015.624937
wavelet-LHH	ngtdm	Strength	9.099708813
wavelet-LHH	ngtdm	Contrast	0.542066467
wavelet-LHH	ngtdm	Busyness	0.107919673
wavelet-LLH	gldm	GrayLevelVariance	481.046832
wavelet-LLH	gldm	HighGrayLevelEmphasis	4542.212121
wavelet-LLH	gldm	DependenceEntropy	5.891785408
wavelet-LLH	gldm	DependenceNonUniformity	48.05050505
wavelet-LLH	gldm	GrayLevelNonUniformity	2.636363636
wavelet-LLH	gldm	SmallDependenceEmphasis	0.718434343
wavelet-LLH	gldm	SmallDependenceHighGrayLevelEmphasis	3423.976431
wavelet-LLH	gldm	DependenceNonUniformityNormalized	0.485358637
wavelet-LLH	gldm	LargeDependenceEmphasis	2.757575758
wavelet-LLH	gldm	LargeDependenceLowGrayLevelEmphasis	0.011148519
wavelet-LLH	gldm	DependenceVariance	0.552800735
wavelet-LLH	gldm	LargeDependenceHighGrayLevelEmphasis	11530.27273
wavelet-LLH	gldm	SmallDependenceLowGrayLevelEmphasis	0.010545234
wavelet-LLH	gldm	LowGrayLevelEmphasis	0.010633725
wavelet-LLH	glcm	JointAverage	66.07377087
wavelet-LLH	glcm	SumAverage	132.1475417
wavelet-LLH	glcm	JointEntropy	6.710778589
wavelet-LLH	glcm	ClusterShade	-5988.704931
wavelet-LLH	glcm	MaximumProbability	0.019698605
wavelet-LLH	glcm	Idmn	0.942155513
wavelet-LLH	glcm	JointEnergy	0.01003496
wavelet-LLH	glcm	Contrast	958.2486613
wavelet-LLH	glcm	DifferenceEntropy	4.585317608
wavelet-LLH	glcm	InverseVariance	0.095661262
wavelet-LLH	glcm	DifferenceVariance	484.459606
wavelet-LLH	glcm	Idn	0.868585861
wavelet-LLH	glcm	Idm	0.08968519
wavelet-LLH	glcm	Correlation	-0.137439885
wavelet-LLH	glcm	Autocorrelation	4314.36383
wavelet-LLH	glcm	SumEntropy	5.064852731
wavelet-LLH	glcm	SumSquares	427.0384327
wavelet-LLH	glcm	ClusterProminence	3686596.037
wavelet-LLH	glcm	Imc2	0.999743941
wavelet-LLH	glcm	Imc1	-0.723899727
wavelet-LLH	glcm	DifferenceAverage	20.01520467
wavelet-LLH	glcm	Id	0.157843167
wavelet-LLH	glcm	ClusterTendency	749.9050696
wavelet-LLH	firstorder	InterquartileRange	403.9551842
wavelet-LLH	firstorder	Skewness	-0.333605106
wavelet-LLH	firstorder	Uniformity	0.026629936
wavelet-LLH	firstorder	Median	-137.9492381
wavelet-LLH	firstorder	Energy	31900431.76
wavelet-LLH	firstorder	RobustMeanAbsoluteDeviation	218.0616052
wavelet-LLH	firstorder	MeanAbsoluteDeviation	384.601144
wavelet-LLH	firstorder	TotalEnergy	861311657.6
wavelet-LLH	firstorder	Maximum	1019.634471
wavelet-LLH	firstorder	RootMeanSquared	567.6500537
wavelet-LLH	firstorder	90Percentile	692.2916149
wavelet-LLH	firstorder	Minimum	-1719.426754
wavelet-LLH	firstorder	Entropy	5.486341312

wavelet-LLH	firstorder	Range	2739.061225
wavelet-LLH	firstorder	Variance	301378.8197
wavelet-LLH	firstorder	10Percentile	-996.2657972
wavelet-LLH	firstorder	Kurtosis	3.554816387
wavelet-LLH	firstorder	Mean	-144.3875473
wavelet-LLH	glrlm	ShortRunLowGrayLevelEmphasis	0.010828358
wavelet-LLH	glrlm	GrayLevelVariance	488.606226
wavelet-LLH	glrlm	LowGrayLevelRunEmphasis	0.010832464
wavelet-LLH	glrlm	GrayLevelNonUniformityNormalized	0.026235274
wavelet-LLH	glrlm	RunVariance	0.023536878
wavelet-LLH	glrlm	GrayLevelNonUniformity	2.549280484
wavelet-LLH	glrlm	LongRunEmphasis	1.0626102
wavelet-LLH	glrlm	ShortRunHighGrayLevelEmphasis	4500.675866
wavelet-LLH	glrlm	RunLengthNonUniformity	93.85567748
wavelet-LLH	glrlm	ShortRunEmphasis	0.986649005
wavelet-LLH	glrlm	LongRunHighGrayLevelEmphasis	4805.266564
wavelet-LLH	glrlm	RunPercentage	0.981351981
wavelet-LLH	glrlm	LongRunLowGrayLevelEmphasis	0.010850877
wavelet-LLH	glrlm	RunEntropy	5.544058522
wavelet-LLH	glrlm	HighGrayLevelRunEmphasis	4553.080094
wavelet-LLH	glrlm	RunLengthNonUniformityNormalized	0.965706131
wavelet-LLH	glszm	GrayLevelVariance	583.7523011
wavelet-LLH	glszm	ZoneVariance	0.427514793
wavelet-LLH	glszm	GrayLevelNonUniformityNormalized	0.022024984
wavelet-LLH	glszm	SizeZoneNonUniformityNormalized	0.68869165
wavelet-LLH	glszm	SizeZoneNonUniformity	53.71794872
wavelet-LLH	glszm	GrayLevelNonUniformity	1.717948718
wavelet-LLH	glszm	LargeAreaEmphasis	2.038461538
wavelet-LLH	glszm	SmallAreaHighGrayLevelEmphasis	4118.060897
wavelet-LLH	glszm	ZonePercentage	0.787878788
wavelet-LLH	glszm	LargeAreaLowGrayLevelEmphasis	0.013718038
wavelet-LLH	glszm	LargeAreaHighGrayLevelEmphasis	8883.269231
wavelet-LLH	glszm	HighGrayLevelZoneEmphasis	4687.961538
wavelet-LLH	glszm	SmallAreaEmphasis	0.855235043
wavelet-LLH	glszm	LowGrayLevelZoneEmphasis	0.01341422
wavelet-LLH	glszm	ZoneEntropy	5.846111674
wavelet-LLH	glszm	SmallAreaLowGrayLevelEmphasis	0.013366487
wavelet-LLH	ngtdm	Coarseness	0.033612617
wavelet-LLH	ngtdm	Complexity	32098.14878
wavelet-LLH	ngtdm	Strength	79.36130569
wavelet-LLH	ngtdm	Contrast	5.742219333
wavelet-LLH	ngtdm	Busyness	0.010680514
wavelet-LLL	gldm	GrayLevelVariance	618.2928273
wavelet-LLL	gldm	HighGrayLevelEmphasis	2563.191919
wavelet-LLL	gldm	DependenceEntropy	5.934862807
wavelet-LLL	gldm	DependenceNonUniformity	54.71717172
wavelet-LLL	gldm	GrayLevelNonUniformity	2.232323232
wavelet-LLL	gldm	SmallDependenceEmphasis	0.760942761
wavelet-LLL	gldm	SmallDependenceHighGrayLevelEmphasis	1958.464646
wavelet-LLL	gldm	DependenceNonUniformityNormalized	0.552698704
wavelet-LLL	gldm	LargeDependenceEmphasis	2.090909091
wavelet-LLL	gldm	LargeDependenceLowGrayLevelEmphasis	0.021179132
wavelet-LLL	gldm	DependenceVariance	0.286093256
wavelet-LLL	gldm	LargeDependenceHighGrayLevelEmphasis	5419.676768
wavelet-LLL	gldm	SmallDependenceLowGrayLevelEmphasis	0.016725048

wavelet-LLL	gldm	LowGrayLevelEmphasis	0.017607574
wavelet-LLL	glcm	JointAverage	49.73761955
wavelet-LLL	glcm	SumAverage	99.47523909
wavelet-LLL	glcm	JointEntropy	6.742334512
wavelet-LLL	glcm	ClusterShade	-6341.922756
wavelet-LLL	glcm	MaximumProbability	0.017715697
wavelet-LLL	glcm	Idmn	0.911643114
wavelet-LLL	glcm	JointEnergy	0.009743847
wavelet-LLL	glcm	Contrast	865.4352648
wavelet-LLL	glcm	DifferenceEntropy	4.873223272
wavelet-LLL	glcm	InverseVariance	0.036750996
wavelet-LLL	glcm	DifferenceVariance	248.000715
wavelet-LLL	glcm	Idn	0.80388546
wavelet-LLL	glcm	Idm	0.046383565
wavelet-LLL	glcm	Correlation	0.275165608
wavelet-LLL	glcm	Autocorrelation	2635.211237
wavelet-LLL	glcm	SumEntropy	5.428365582
wavelet-LLL	glcm	SumSquares	592.3710578
wavelet-LLL	glcm	ClusterProminence	4972396.689
wavelet-LLL	glcm	Imc2	0.999897909
wavelet-LLL	glcm	Imc1	-0.776310244
wavelet-LLL	glcm	DifferenceAverage	23.89274562
wavelet-LLL	glcm	Id	0.099143721
wavelet-LLL	glcm	ClusterTendency	1504.048966
wavelet-LLL	firstorder	InterquartileRange	980.4610068
wavelet-LLL	firstorder	Skewness	0.112262815
wavelet-LLL	firstorder	Uniformity	0.02254872
wavelet-LLL	firstorder	Median	-709.9931383
wavelet-LLL	firstorder	Energy	81511277.36
wavelet-LLL	firstorder	RobustMeanAbsoluteDeviation	411.409617
wavelet-LLL	firstorder	MeanAbsoluteDeviation	529.9300081
wavelet-LLL	firstorder	TotalEnergy	2200804489
wavelet-LLL	firstorder	Maximum	443.6527859
wavelet-LLL	firstorder	RootMeanSquared	907.3842824
wavelet-LLL	firstorder	90Percentile	255.0332238
wavelet-LLL	firstorder	Minimum	-1746.680186
wavelet-LLL	firstorder	Entropy	5.682401004
wavelet-LLL	firstorder	Range	2190.332972
wavelet-LLL	firstorder	Variance	386188.076
wavelet-LLL	firstorder	10Percentile	-1500.663
wavelet-LLL	firstorder	Kurtosis	1.923490012
wavelet-LLL	firstorder	Mean	-661.1793705
wavelet-LLL	glrlm	ShortRunLowGrayLevelEmphasis	0.017764782
wavelet-LLL	glrlm	GrayLevelVariance	618.2913403
wavelet-LLL	glrlm	LowGrayLevelRunEmphasis	0.017799834
wavelet-LLL	glrlm	GrayLevelNonUniformityNormalized	0.022331912
wavelet-LLL	glrlm	RunVariance	0.013197386
wavelet-LLL	glrlm	GrayLevelNonUniformity	2.181967856
wavelet-LLL	glrlm	LongRunEmphasis	1.040720297
wavelet-LLL	glrlm	ShortRunHighGrayLevelEmphasis	2536.644992
wavelet-LLL	glrlm	RunLengthNonUniformity	95.14907808
wavelet-LLL	glrlm	ShortRunEmphasis	0.989819926
wavelet-LLL	glrlm	LongRunHighGrayLevelEmphasis	2668.084077
wavelet-LLL	glrlm	RunPercentage	0.986790987
wavelet-LLL	glrlm	LongRunLowGrayLevelEmphasis	0.017940041

wavelet-LLL	glrlm	RunEntropy	5.716360349
wavelet-LLL	glrlm	HighGrayLevelRunEmphasis	2562.932809
wavelet-LLL	glrlm	RunLengthNonUniformityNormalized	0.973605227
wavelet-LLL	glszm	GrayLevelVariance	614.7339236
wavelet-LLL	glszm	ZoneVariance	0.179706779
wavelet-LLL	glszm	GrayLevelNonUniformityNormalized	0.019596458
wavelet-LLL	glszm	SizeZoneNonUniformityNormalized	0.699811293
wavelet-LLL	glszm	SizeZoneNonUniformity	58.08433735
wavelet-LLL	glszm	GrayLevelNonUniformity	1.626506024
wavelet-LLL	glszm	LargeAreaEmphasis	1.602409639
wavelet-LLL	glszm	SmallAreaHighGrayLevelEmphasis	2224.481928
wavelet-LLL	glszm	ZonePercentage	0.838383838
wavelet-LLL	glszm	LargeAreaLowGrayLevelEmphasis	0.022414406
wavelet-LLL	glszm	LargeAreaHighGrayLevelEmphasis	4114.722892
wavelet-LLL	glszm	HighGrayLevelZoneEmphasis	2567.73494
wavelet-LLL	glszm	SmallAreaEmphasis	0.862784471
wavelet-LLL	glszm	LowGrayLevelZoneEmphasis	0.020299213
wavelet-LLL	glszm	ZoneEntropy	5.989497263
wavelet-LLL	glszm	SmallAreaLowGrayLevelEmphasis	0.019774536
wavelet-LLL	ngtdm	Coarseness	0.029010832
wavelet-LLL	ngtdm	Complexity	32589.43888
wavelet-LLL	ngtdm	Strength	99.64534189
wavelet-LLL	ngtdm	Contrast	5.475823143
wavelet-LLL	ngtdm	Busyness	0.016454754
wavelet-HHL	gldm	GrayLevelVariance	79.86368738
wavelet-HHL	gldm	HighGrayLevelEmphasis	690.3030303
wavelet-HHL	gldm	DependenceEntropy	5.380379443
wavelet-HHL	gldm	DependenceNonUniformity	41.86868687
wavelet-HHL	gldm	GrayLevelNonUniformity	4.171717172
wavelet-HHL	gldm	SmallDependenceEmphasis	0.653198653
wavelet-HHL	gldm	SmallDependenceHighGrayLevelEmphasis	482.7631874
wavelet-HHL	gldm	DependenceNonUniformityNormalized	0.422916029
wavelet-HHL	gldm	LargeDependenceEmphasis	3.060606061
wavelet-HHL	gldm	LargeDependenceLowGrayLevelEmphasis	0.01691012
wavelet-HHL	gldm	DependenceVariance	0.545658606
wavelet-HHL	gldm	LargeDependenceHighGrayLevelEmphasis	1950.585859
wavelet-HHL	gldm	SmallDependenceLowGrayLevelEmphasis	0.011984011
wavelet-HHL	gldm	LowGrayLevelEmphasis	0.012731899
wavelet-HHL	glcm	JointAverage	25.11804444
wavelet-HHL	glcm	SumAverage	50.23608888
wavelet-HHL	glcm	JointEntropy	6.569593252
wavelet-HHL	glcm	ClusterShade	511.6622838
wavelet-HHL	glcm	MaximumProbability	0.02245027
wavelet-HHL	glcm	Idmn	0.946070745
wavelet-HHL	glcm	JointEnergy	0.011356946
wavelet-HHL	glcm	Contrast	152.7397285
wavelet-HHL	glcm	DifferenceEntropy	4.085628939
wavelet-HHL	glcm	InverseVariance	0.107539682
wavelet-HHL	glcm	DifferenceVariance	56.29898273
wavelet-HHL	glcm	Idn	0.850879713
wavelet-HHL	glcm	Idm	0.105986941
wavelet-HHL	glcm	Correlation	-0.023145712
wavelet-HHL	glcm	Autocorrelation	630.9658409
wavelet-HHL	glcm	SumEntropy	4.699362564
wavelet-HHL	glcm	SumSquares	75.97070434

wavelet-HHL	glcm	ClusterProminence	93590.39822
wavelet-HHL	glcm	Imc2	0.997893626
wavelet-HHL	glcm	Imc1	-0.590740835
wavelet-HHL	glcm	DifferenceAverage	9.378536085
wavelet-HHL	glcm	Id	0.19078703
wavelet-HHL	glcm	ClusterTendency	151.1430889
wavelet-HHL	firstorder	InterquartileRange	289.6833536
wavelet-HHL	firstorder	Skewness	0.297891945
wavelet-HHL	firstorder	Uniformity	0.042138557
wavelet-HHL	firstorder	Median	-9.70551159
wavelet-HHL	firstorder	Energy	5000437.672
wavelet-HHL	firstorder	RobustMeanAbsoluteDeviation	123.7281006
wavelet-HHL	firstorder	MeanAbsoluteDeviation	177.1814969
wavelet-HHL	firstorder	TotalEnergy	135011817.1
wavelet-HHL	firstorder	Maximum	613.7195534
wavelet-HHL	firstorder	RootMeanSquared	224.7431232
wavelet-HHL	firstorder	90Percentile	314.4399422
wavelet-HHL	firstorder	Minimum	-551.2401029
wavelet-HHL	firstorder	Entropy	4.790021141
wavelet-HHL	firstorder	Range	1164.959656
wavelet-HHL	firstorder	Variance	49597.29292
wavelet-HHL	firstorder	10Percentile	-231.0293773
wavelet-HHL	firstorder	Kurtosis	3.080897703
wavelet-HHL	firstorder	Mean	30.20229312
wavelet-HHL	glrlm	ShortRunLowGrayLevelEmphasis	0.012944642
wavelet-HHL	glrlm	GrayLevelVariance	81.02653539
wavelet-HHL	glrlm	LowGrayLevelRunEmphasis	0.012980386
wavelet-HHL	glrlm	GrayLevelNonUniformityNormalized	0.041730008
wavelet-HHL	glrlm	RunVariance	0.024122051
wavelet-HHL	glrlm	GrayLevelNonUniformity	4.038460084
wavelet-HHL	glrlm	LongRunEmphasis	1.071542827
wavelet-HHL	glrlm	ShortRunHighGrayLevelEmphasis	682.1532154
wavelet-HHL	glrlm	RunLengthNonUniformity	92.6003691
wavelet-HHL	glrlm	ShortRunEmphasis	0.983013978
wavelet-HHL	glrlm	LongRunHighGrayLevelEmphasis	736.4738937
wavelet-HHL	glrlm	RunPercentage	0.977466977
wavelet-HHL	glrlm	LongRunLowGrayLevelEmphasis	0.013126668
wavelet-HHL	glrlm	RunEntropy	4.864661149
wavelet-HHL	glrlm	HighGrayLevelRunEmphasis	692.2335454
wavelet-HHL	glrlm	RunLengthNonUniformityNormalized	0.956460778
wavelet-HHL	glszm	GrayLevelVariance	97.88580247
wavelet-HHL	glszm	ZoneVariance	0.651041667
wavelet-HHL	glszm	GrayLevelNonUniformityNormalized	0.037037037
wavelet-HHL	glszm	SizeZoneNonUniformityNormalized	0.624614198
wavelet-HHL	glszm	SizeZoneNonUniformity	44.97222222
wavelet-HHL	glszm	GrayLevelNonUniformity	2.666666667
wavelet-HHL	glszm	LargeAreaEmphasis	2.541666667
wavelet-HHL	glszm	SmallAreaHighGrayLevelEmphasis	612.8762539
wavelet-HHL	glszm	ZonePercentage	0.727272727
wavelet-HHL	glszm	LargeAreaLowGrayLevelEmphasis	0.019851807
wavelet-HHL	glszm	LargeAreaHighGrayLevelEmphasis	1665.388889
wavelet-HHL	glszm	HighGrayLevelZoneEmphasis	720.1111111
wavelet-HHL	glszm	SmallAreaEmphasis	0.816238426
wavelet-HHL	glszm	LowGrayLevelZoneEmphasis	0.016722145
wavelet-HHL	glszm	ZoneEntropy	5.223550481

wavelet-HHL	glszm	SmallAreaLowGrayLevelEmphasis	0.016305001
wavelet-HHL	ngtdm	Coarseness	0.043736486
wavelet-HHL	ngtdm	Complexity	3315.261825
wavelet-HHL	ngtdm	Strength	19.74000074
wavelet-HHL	ngtdm	Contrast	1.076042174
wavelet-HHL	ngtdm	Busyness	0.037902819
wavelet-HLH	gldm	GrayLevelVariance	51.67084991
wavelet-HLH	gldm	HighGrayLevelEmphasis	406.5555556
wavelet-HLH	gldm	DependenceEntropy	5.22739715
wavelet-HLH	gldm	DependenceNonUniformity	46.33333333
wavelet-HLH	gldm	GrayLevelNonUniformity	4.818181818
wavelet-HLH	gldm	SmallDependenceEmphasis	0.691130752
wavelet-HLH	gldm	SmallDependenceHighGrayLevelEmphasis	274.4752385
wavelet-HLH	gldm	DependenceNonUniformityNormalized	0.468013468
wavelet-HLH	gldm	LargeDependenceEmphasis	2.95959596
wavelet-HLH	gldm	LargeDependenceLowGrayLevelEmphasis	0.022602429
wavelet-HLH	gldm	DependenceVariance	0.633200694
wavelet-HLH	gldm	LargeDependenceHighGrayLevelEmphasis	1145.121212
wavelet-HLH	gldm	SmallDependenceLowGrayLevelEmphasis	0.013910011
wavelet-HLH	gldm	LowGrayLevelEmphasis	0.015088047
wavelet-HLH	glcm	JointAverage	18.52879522
wavelet-HLH	glcm	SumAverage	37.05759044
wavelet-HLH	glcm	JointEntropy	6.578974483
wavelet-HLH	glcm	ClusterShade	61.27047537
wavelet-HLH	glcm	MaximumProbability	0.024337472
wavelet-HLH	glcm	Idmn	0.934065161
wavelet-HLH	glcm	JointEnergy	0.011220709
wavelet-HLH	glcm	Contrast	109.9975733
wavelet-HLH	glcm	DifferenceEntropy	4.000519271
wavelet-HLH	glcm	InverseVariance	0.120794229
wavelet-HLH	glcm	DifferenceVariance	38.34773206
wavelet-HLH	glcm	Idn	0.831092379
wavelet-HLH	glcm	Idm	0.108190429
wavelet-HLH	glcm	Correlation	-0.036873137
wavelet-HLH	glcm	Autocorrelation	341.4825868
wavelet-HLH	glcm	SumEntropy	4.676542315
wavelet-HLH	glcm	SumSquares	53.11022283
wavelet-HLH	glcm	ClusterProminence	33355.41511
wavelet-HLH	glcm	Imc2	0.996297495
wavelet-HLH	glcm	Imc1	-0.547551067
wavelet-HLH	glcm	DifferenceAverage	8.325879781
wavelet-HLH	glcm	Id	0.194931789
wavelet-HLH	glcm	ClusterTendency	102.4433181
wavelet-HLH	firstorder	InterquartileRange	248.6053265
wavelet-HLH	firstorder	Skewness	0.156257867
wavelet-HLH	firstorder	Uniformity	0.048668503
wavelet-HLH	firstorder	Median	35.28418705
wavelet-HLH	firstorder	Energy	3330846.389
wavelet-HLH	firstorder	RobustMeanAbsoluteDeviation	102.7057545
wavelet-HLH	firstorder	MeanAbsoluteDeviation	145.4385518
wavelet-HLH	firstorder	TotalEnergy	89932852.49
wavelet-HLH	firstorder	Maximum	479.5494232
wavelet-HLH	firstorder	RootMeanSquared	183.4254972
wavelet-HLH	firstorder	90Percentile	278.0910007
wavelet-HLH	firstorder	Minimum	-423.4871531

wavelet-HLH	firstorder	Entropy	4.584552046
wavelet-HLH	firstorder	Range	903.0365763
wavelet-HLH	firstorder	Variance	32523.03206
wavelet-HLH	firstorder	10Percentile	-182.5808343
wavelet-HLH	firstorder	Kurtosis	2.677575307
wavelet-HLH	firstorder	Mean	33.49449148
wavelet-HLH	glrlm	ShortRunLowGrayLevelEmphasis	0.015266455
wavelet-HLH	glrlm	GrayLevelVariance	52.039436
wavelet-HLH	glrlm	LowGrayLevelRunEmphasis	0.015326241
wavelet-HLH	glrlm	GrayLevelNonUniformityNormalized	0.048064124
wavelet-HLH	glrlm	RunVariance	0.020145962
wavelet-HLH	glrlm	GrayLevelNonUniformity	4.662790355
wavelet-HLH	glrlm	LongRunEmphasis	1.0632107
wavelet-HLH	glrlm	ShortRunHighGrayLevelEmphasis	400.2890623
wavelet-HLH	glrlm	RunLengthNonUniformity	93.17190622
wavelet-HLH	glrlm	ShortRunEmphasis	0.984197325
wavelet-HLH	glrlm	LongRunHighGrayLevelEmphasis	432.0734488
wavelet-HLH	glrlm	RunPercentage	0.97979798
wavelet-HLH	glrlm	LongRunLowGrayLevelEmphasis	0.015565384
wavelet-HLH	glrlm	RunEntropy	4.661878931
wavelet-HLH	glrlm	HighGrayLevelRunEmphasis	406.6459396
wavelet-HLH	glrlm	RunLengthNonUniformityNormalized	0.959708076
wavelet-HLH	glszm	GrayLevelVariance	56.32271468
wavelet-HLH	glszm	ZoneVariance	0.500519391
wavelet-HLH	glszm	GrayLevelNonUniformityNormalized	0.043628809
wavelet-HLH	glszm	SizeZoneNonUniformityNormalized	0.649238227
wavelet-HLH	glszm	SizeZoneNonUniformity	49.34210526
wavelet-HLH	glszm	GrayLevelNonUniformity	3.315789474
wavelet-HLH	glszm	LargeAreaEmphasis	2.197368421
wavelet-HLH	glszm	SmallAreaHighGrayLevelEmphasis	327.8665936
wavelet-HLH	glszm	ZonePercentage	0.767676768
wavelet-HLH	glszm	LargeAreaLowGrayLevelEmphasis	0.022878055
wavelet-HLH	glszm	LargeAreaHighGrayLevelEmphasis	887.8815789
wavelet-HLH	glszm	HighGrayLevelZoneEmphasis	401.5
wavelet-HLH	glszm	SmallAreaEmphasis	0.833220029
wavelet-HLH	glszm	LowGrayLevelZoneEmphasis	0.018527914
wavelet-HLH	glszm	ZoneEntropy	5.073135244
wavelet-HLH	glszm	SmallAreaLowGrayLevelEmphasis	0.017873013
wavelet-HLH	ngtdm	Coarseness	0.043115007
wavelet-HLH	ngtdm	Complexity	2012.605488
wavelet-HLH	ngtdm	Strength	13.21399492
wavelet-HLH	ngtdm	Contrast	0.771261601
wavelet-HLH	ngtdm	Busyness	0.054334695

Image type	Feature Class	Feature Name	11: RTSTRUCT: C99-DNU_segment_Tumor
general	info	BoundingBox	(0, 0, 1, 9, 6, 7)
general	info	EnabledImageTypes	{u'Original': {}, u'Wavelet': {}}
general	info	GeneralSettings	{'distances': [1], 'additionalInfo': True, 'force2D': False, 'interpolator': 'sitkBSpline', 'resampledPixelSpacing': [3.0], 'label': 1, 'normalizeScale': 1, 'normalize': False, 'force2Ddimension': 0, 'removeOutliers': None, 'minimumROISize': None, u'binWidth': 25.0, 'minimumROIDimensions': 1, u'correctMask': True, 'preCrop': False, u'symmetricalGLCM': True, 'resegmentRange': None, 'padDistance': 5}
general	info	ImageHash	d1448d1054ef3f5958caa29f0aec3fa175e10e06
general	info	ImageSpacing	(0.9765625000000002, 0.9765625000000002, 2.0)
general	info	MaskHash	5428d4eb24a2ef46135486e49994c85f4ad1c773
general	info	NumpyVersion	1.13.1
general	info	PyWaveletVersion	0.5.2
general	info	SimpleITKVersion	1.1.0
general	info	Version	1.3.0.post102+g1b0eb74
general	info	VolumeNum	1
general	info	VoxelNum	150
original	shape	Maximum3DDiameter	27
original	shape	Maximum2DDiameterSlice	21.21320344
original	shape	Sphericity	0.838176628
original	shape	MinorAxis	15.87696363
original	shape	Elongation	0.626386244
original	shape	SurfaceVolumeRatio	0.361962493
original	shape	Volume	4050
original	shape	MajorAxis	25.34692257
original	shape	SurfaceArea	1465.948095
original	shape	Flatness	0.582919168
original	shape	LeastAxis	14.77520701
original	shape	Maximum2DDiameterColumn	26.83281573
original	shape	Maximum2DDiameterRow	20.1246118
original	gldm	GrayLevelVariance	116.0924889
original	gldm	HighGrayLevelEmphasis	641.1133333
original	gldm	DependenceEntropy	5.765071067
original	gldm	DependenceNonUniformity	48.37333333
original	gldm	GrayLevelNonUniformity	7.2
original	gldm	SmallDependenceEmphasis	0.581936608
original	gldm	SmallDependenceHighGrayLevelEmphasis	233.8262815
original	gldm	DependenceNonUniformityNormalized	0.322488889
original	gldm	LargeDependenceEmphasis	9.32
original	gldm	LargeDependenceLowGrayLevelEmphasis	0.037529055
original	gldm	DependenceVariance	3.9376
original	gldm	LargeDependenceHighGrayLevelEmphasis	10222.92667

original	gldm	SmallDependenceLowGrayLevelEmphasis	0.015094516
original	gldm	LowGrayLevelEmphasis	0.018466266
original	glcm	JointAverage	25.49598556
original	glcm	SumAverage	50.99197113
original	glcm	JointEntropy	6.901877295
original	glcm	ClusterShade	-2609.839479
original	glcm	MaximumProbability	0.044544394
original	glcm	Idmn	0.902941158
original	glcm	JointEnergy	0.011237481
original	glcm	Contrast	171.5436168
original	glcm	DifferenceEntropy	4.38353599
original	glcm	InverseVariance	0.163276232
original	glcm	DifferenceVariance	74.77633397
original	glcm	Idn	0.813522949
original	glcm	Idm	0.171310376
original	glcm	Correlation	0.170241989
original	glcm	Autocorrelation	667.6836284
original	glcm	SumEntropy	5.127303344
original	glcm	SumSquares	103.307913
original	glcm	ClusterProminence	158811.4548
original	glcm	Imc2	0.991343586
original	glcm	Imc1	-0.455941056
original	glcm	DifferenceAverage	9.74229526
original	glcm	Id	0.239876815
original	glcm	ClusterTendency	241.6880351
original	firstorder	InterquartileRange	492.75
original	firstorder	Skewness	-0.44388371
original	firstorder	Uniformity	0.048
original	firstorder	Median	-185.5
original	firstorder	Energy	21457803
original	firstorder	RobustMeanAbsoluteDeviation	200.2843322
original	firstorder	MeanAbsoluteDeviation	239.3032
original	firstorder	TotalEnergy	579360681
original	firstorder	Maximum	60
original	firstorder	RootMeanSquared	378.2221834
original	firstorder	90Percentile	33
original	firstorder	Minimum	-805
original	firstorder	Entropy	4.763949425
original	firstorder	Range	865
original	firstorder	Variance	72717.44396
original	firstorder	10Percentile	-686.2
original	firstorder	Kurtosis	1.799043769
original	firstorder	Mean	-265.2066667
original	glrlm	ShortRunLowGrayLevelEmphasis	0.019094118
original	glrlm	GrayLevelVariance	115.2479218
original	glrlm	LowGrayLevelRunEmphasis	0.019251573
original	glrlm	GrayLevelNonUniformityNormalized	0.04368768
original	glrlm	RunVariance	0.054984902
original	glrlm	GrayLevelNonUniformity	6.225202342
original	glrlm	LongRunEmphasis	1.166381085
original	glrlm	ShortRunHighGrayLevelEmphasis	573.7533079
original	glrlm	RunLengthNonUniformity	128.5921295
original	glrlm	ShortRunEmphasis	0.96087334

original	glrlm	LongRunHighGrayLevelEmphasis	799.2908514
original	glrlm	RunPercentage	0.949230769
original	glrlm	LongRunLowGrayLevelEmphasis	0.019889827
original	glrlm	RunEntropy	4.994971061
original	glrlm	HighGrayLevelRunEmphasis	616.5426661
original	glrlm	RunLengthNonUniformityNormalized	0.902348211
original	glszm	GrayLevelVariance	96.02295674
original	glszm	ZoneVariance	4.598363269
original	glszm	GrayLevelNonUniformityNormalized	0.033266022
original	glszm	SizeZoneNonUniformityNormalized	0.652247848
original	glszm	SizeZoneNonUniformity	63.26804124
original	glszm	GrayLevelNonUniformity	3.226804124
original	glszm	LargeAreaEmphasis	6.989690722
original	glszm	SmallAreaHighGrayLevelEmphasis	305.8606872
original	glszm	ZonePercentage	0.646666667
original	glszm	LargeAreaLowGrayLevelEmphasis	0.039360444
original	glszm	LargeAreaHighGrayLevelEmphasis	7296.381443
original	glszm	HighGrayLevelZoneEmphasis	437.7010309
original	glszm	SmallAreaEmphasis	0.833838525
original	glszm	LowGrayLevelZoneEmphasis	0.024979429
original	glszm	ZoneEntropy	5.44992444
original	glszm	SmallAreaLowGrayLevelEmphasis	0.022508314
original	ngtdm	Coarseness	0.018975175
original	ngtdm	Complexity	4319.069974
original	ngtdm	Strength	14.50942542
original	ngtdm	Contrast	1.550614164
original	ngtdm	Busyness	0.056405124
wavelet-HHH	gldm	GrayLevelVariance	10.75222222
wavelet-HHH	gldm	HighGrayLevelEmphasis	85.28666667
wavelet-HHH	gldm	DependenceEntropy	5.155485945
wavelet-HHH	gldm	DependenceNonUniformity	39.57333333
wavelet-HHH	gldm	GrayLevelNonUniformity	13.86666667
wavelet-HHH	gldm	SmallDependenceEmphasis	0.464606425
wavelet-HHH	gldm	SmallDependenceHighGrayLevelEmphasis	35.58794777
wavelet-HHH	gldm	DependenceNonUniformityNormalized	0.263822222
wavelet-HHH	gldm	LargeDependenceEmphasis	6.506666667
wavelet-HHH	gldm	LargeDependenceLowGrayLevelEmphasis	0.133925977
wavelet-HHH	gldm	DependenceVariance	1.548622222
wavelet-HHH	gldm	LargeDependenceHighGrayLevelEmphasis	574.98
wavelet-HHH	gldm	SmallDependenceLowGrayLevelEmphasis	0.04262138
wavelet-HHH	gldm	LowGrayLevelEmphasis	0.052149079
wavelet-HHH	glcm	JointAverage	8.542772345
wavelet-HHH	glcm	SumAverage	17.08554469
wavelet-HHH	glcm	JointEntropy	6.482787004
wavelet-HHH	glcm	ClusterShade	-15.21110469
wavelet-HHH	glcm	MaximumProbability	0.032015143
wavelet-HHH	glcm	Idmn	0.928907767
wavelet-HHH	glcm	JointEnergy	0.013446624
wavelet-HHH	glcm	Contrast	22.54594988
wavelet-HHH	glcm	DifferenceEntropy	3.233388182
wavelet-HHH	glcm	InverseVariance	0.247027034
wavelet-HHH	glcm	DifferenceVariance	8.192244342
wavelet-HHH	glcm	Idn	0.825558773

wavelet-HHH	glcm	Idm	0.222725751
wavelet-HHH	glcm	Correlation	-0.042246796
wavelet-HHH	glcm	Autocorrelation	72.53832767
wavelet-HHH	glcm	SumEntropy	3.97724548
wavelet-HHH	glcm	SumSquares	10.82679283
wavelet-HHH	glcm	ClusterProminence	1325.895583
wavelet-HHH	glcm	Imc2	0.896280835
wavelet-HHH	glcm	Imc1	-0.225131015
wavelet-HHH	glcm	DifferenceAverage	3.761576249
wavelet-HHH	glcm	Id	0.317223421
wavelet-HHH	glcm	ClusterTendency	20.76122146
wavelet-HHH	firstorder	InterquartileRange	93.09348955
wavelet-HHH	firstorder	Skewness	-0.258308227
wavelet-HHH	firstorder	Uniformity	0.092444444
wavelet-HHH	firstorder	Median	4.689432436
wavelet-HHH	firstorder	Energy	996359.9937
wavelet-HHH	firstorder	RobustMeanAbsoluteDeviation	42.85691436
wavelet-HHH	firstorder	MeanAbsoluteDeviation	63.65572925
wavelet-HHH	firstorder	TotalEnergy	26901719.83
wavelet-HHH	firstorder	Maximum	197.3406346
wavelet-HHH	firstorder	RootMeanSquared	81.50091998
wavelet-HHH	firstorder	90Percentile	100.6835545
wavelet-HHH	firstorder	Minimum	-198.329252
wavelet-HHH	firstorder	Entropy	3.662822942
wavelet-HHH	firstorder	Range	395.6698866
wavelet-HHH	firstorder	Variance	6629.932744
wavelet-HHH	firstorder	10Percentile	-108.48495
wavelet-HHH	firstorder	Kurtosis	2.933504973
wavelet-HHH	firstorder	Mean	3.530894223
wavelet-HHH	glrlm	ShortRunLowGrayLevelEmphasis	0.053383249
wavelet-HHH	glrlm	GrayLevelVariance	11.05155773
wavelet-HHH	glrlm	LowGrayLevelRunEmphasis	0.053955389
wavelet-HHH	glrlm	GrayLevelNonUniformityNormalized	0.090212726
wavelet-HHH	glrlm	RunVariance	0.053550318
wavelet-HHH	glrlm	GrayLevelNonUniformity	12.89433056
wavelet-HHH	glrlm	LongRunEmphasis	1.155973192
wavelet-HHH	glrlm	ShortRunHighGrayLevelEmphasis	81.88958306
wavelet-HHH	glrlm	RunLengthNonUniformity	130.3315708
wavelet-HHH	glrlm	ShortRunEmphasis	0.964636034
wavelet-HHH	glrlm	LongRunHighGrayLevelEmphasis	99.17089758
wavelet-HHH	glrlm	RunPercentage	0.952820513
wavelet-HHH	glrlm	LongRunLowGrayLevelEmphasis	0.056399747
wavelet-HHH	glrlm	RunEntropy	3.895840103
wavelet-HHH	glrlm	HighGrayLevelRunEmphasis	85.0500068
wavelet-HHH	glrlm	RunLengthNonUniformityNormalized	0.911424569
wavelet-HHH	glszm	GrayLevelVariance	15.64317867
wavelet-HHH	glszm	ZoneVariance	6.736149584
wavelet-HHH	glszm	GrayLevelNonUniformityNormalized	0.076523546
wavelet-HHH	glszm	SizeZoneNonUniformityNormalized	0.545360111
wavelet-HHH	glszm	SizeZoneNonUniformity	41.44736842
wavelet-HHH	glszm	GrayLevelNonUniformity	5.815789474
wavelet-HHH	glszm	LargeAreaEmphasis	10.63157895
wavelet-HHH	glszm	SmallAreaHighGrayLevelEmphasis	55.44285537

wavelet-HHH	glszm	ZonePercentage	0.506666667
wavelet-HHH	glszm	LargeAreaLowGrayLevelEmphasis	0.223449601
wavelet-HHH	glszm	LargeAreaHighGrayLevelEmphasis	909.0394737
wavelet-HHH	glszm	HighGrayLevelZoneEmphasis	79.01315789
wavelet-HHH	glszm	SmallAreaEmphasis	0.764747689
wavelet-HHH	glszm	LowGrayLevelZoneEmphasis	0.086947923
wavelet-HHH	glszm	ZoneEntropy	4.702426041
wavelet-HHH	glszm	SmallAreaLowGrayLevelEmphasis	0.081347303
wavelet-HHH	ngtdm	Coarseness	0.037838888
wavelet-HHH	ngtdm	Complexity	242.8783905
wavelet-HHH	ngtdm	Strength	2.486407124
wavelet-HHH	ngtdm	Contrast	0.246164142
wavelet-HHH	ngtdm	Busyness	0.206102511
wavelet-HLL	gldm	GrayLevelVariance	70.75115556
wavelet-HLL	gldm	HighGrayLevelEmphasis	482.3
wavelet-HLL	gldm	DependenceEntropy	5.790899754
wavelet-HLL	gldm	DependenceNonUniformity	54.16
wavelet-HLL	gldm	GrayLevelNonUniformity	5.813333333
wavelet-HLL	gldm	SmallDependenceEmphasis	0.588425926
wavelet-HLL	gldm	SmallDependenceHighGrayLevelEmphasis	295.8316204
wavelet-HLL	gldm	DependenceNonUniformityNormalized	0.361066667
wavelet-HLL	gldm	LargeDependenceEmphasis	3.866666667
wavelet-HLL	gldm	LargeDependenceLowGrayLevelEmphasis	0.024160246
wavelet-HLL	gldm	DependenceVariance	0.769066667
wavelet-HLL	gldm	LargeDependenceHighGrayLevelEmphasis	1682.446667
wavelet-HLL	gldm	SmallDependenceLowGrayLevelEmphasis	0.012166988
wavelet-HLL	gldm	LowGrayLevelEmphasis	0.01363776
wavelet-HLL	glcm	JointAverage	19.34786054
wavelet-HLL	glcm	SumAverage	38.69572107
wavelet-HLL	glcm	JointEntropy	7.303520542
wavelet-HLL	glcm	ClusterShade	1062.624705
wavelet-HLL	glcm	MaximumProbability	0.015876645
wavelet-HLL	glcm	Idmn	0.942196465
wavelet-HLL	glcm	JointEnergy	0.006809303
wavelet-HLL	glcm	Contrast	110.4322432
wavelet-HLL	glcm	DifferenceEntropy	4.156543426
wavelet-HLL	glcm	InverseVariance	0.115244779
wavelet-HLL	glcm	DifferenceVariance	37.5104669
wavelet-HLL	glcm	Idn	0.842065597
wavelet-HLL	glcm	Idm	0.117112542
wavelet-HLL	glcm	Correlation	0.169552621
wavelet-HLL	glcm	Autocorrelation	386.2186214
wavelet-HLL	glcm	SumEntropy	5.125770119
wavelet-HLL	glcm	SumSquares	67.07765262
wavelet-HLL	glcm	ClusterProminence	80208.52147
wavelet-HLL	glcm	Imc2	0.994486628
wavelet-HLL	glcm	Imc1	-0.474344614
wavelet-HLL	glcm	DifferenceAverage	8.28166361
wavelet-HLL	glcm	Id	0.203182296
wavelet-HLL	glcm	ClusterTendency	157.8783672
wavelet-HLL	firstorder	InterquartileRange	312.1099276
wavelet-HLL	firstorder	Skewness	0.143524687
wavelet-HLL	firstorder	Uniformity	0.038755556

wavelet-HLL	firstorder	Median	-59.37276412
wavelet-HLL	firstorder	Energy	7088908.09
wavelet-HLL	firstorder	RobustMeanAbsoluteDeviation	120.5973456
wavelet-HLL	firstorder	MeanAbsoluteDeviation	169.370029
wavelet-HLL	firstorder	TotalEnergy	191400518.4
wavelet-HLL	firstorder	Maximum	437.2539325
wavelet-HLL	firstorder	RootMeanSquared	217.3922429
wavelet-HLL	firstorder	90Percentile	211.3483056
wavelet-HLL	firstorder	Minimum	-542.3639256
wavelet-HLL	firstorder	Entropy	4.881156524
wavelet-HLL	firstorder	Range	979.6178581
wavelet-HLL	firstorder	Variance	44133.5096
wavelet-HLL	firstorder	10Percentile	-314.7035275
wavelet-HLL	firstorder	Kurtosis	2.599480022
wavelet-HLL	firstorder	Mean	-55.90954898
wavelet-HLL	glrlm	ShortRunLowGrayLevelEmphasis	0.013862249
wavelet-HLL	glrlm	GrayLevelVariance	71.49155411
wavelet-HLL	glrlm	LowGrayLevelRunEmphasis	0.013942807
wavelet-HLL	glrlm	GrayLevelNonUniformityNormalized	0.038424027
wavelet-HLL	glrlm	RunVariance	0.031306776
wavelet-HLL	glrlm	GrayLevelNonUniformity	5.595126822
wavelet-HLL	glrlm	LongRunEmphasis	1.093482122
wavelet-HLL	glrlm	ShortRunHighGrayLevelEmphasis	474.1433758
wavelet-HLL	glrlm	RunLengthNonUniformity	137.4865936
wavelet-HLL	glrlm	ShortRunEmphasis	0.977817069
wavelet-HLL	glrlm	LongRunHighGrayLevelEmphasis	524.439959
wavelet-HLL	glrlm	RunPercentage	0.970769231
wavelet-HLL	glrlm	LongRunLowGrayLevelEmphasis	0.014280235
wavelet-HLL	glrlm	RunEntropy	4.99346209
wavelet-HLL	glrlm	HighGrayLevelRunEmphasis	483.7580056
wavelet-HLL	glrlm	RunLengthNonUniformityNormalized	0.943571051
wavelet-HLL	glszm	GrayLevelVariance	81.2491
wavelet-HLL	glszm	ZoneVariance	1.33
wavelet-HLL	glszm	GrayLevelNonUniformityNormalized	0.0376
wavelet-HLL	glszm	SizeZoneNonUniformityNormalized	0.5744
wavelet-HLL	glszm	SizeZoneNonUniformity	57.44
wavelet-HLL	glszm	GrayLevelNonUniformity	3.76
wavelet-HLL	glszm	LargeAreaEmphasis	3.58
wavelet-HLL	glszm	SmallAreaHighGrayLevelEmphasis	398.8139592
wavelet-HLL	glszm	ZonePercentage	0.666666667
wavelet-HLL	glszm	LargeAreaLowGrayLevelEmphasis	0.029009321
wavelet-HLL	glszm	LargeAreaHighGrayLevelEmphasis	1502.11
wavelet-HLL	glszm	HighGrayLevelZoneEmphasis	502.73
wavelet-HLL	glszm	SmallAreaEmphasis	0.786176998
wavelet-HLL	glszm	LowGrayLevelZoneEmphasis	0.01861963
wavelet-HLL	glszm	ZoneEntropy	5.500818565
wavelet-HLL	glszm	SmallAreaLowGrayLevelEmphasis	0.017896607
wavelet-HLL	ngtdm	Coarseness	0.035910454
wavelet-HLL	ngtdm	Complexity	3106.651665
wavelet-HLL	ngtdm	Strength	17.06180483
wavelet-HLL	ngtdm	Contrast	0.619796385
wavelet-HLL	ngtdm	Busyness	0.044365034
wavelet-LHL	gldm	GrayLevelVariance	130.6824889

wavelet-LHL	gldm	HighGrayLevelEmphasis	1076.04
wavelet-LHL	gldm	DependenceEntropy	5.798534734
wavelet-LHL	gldm	DependenceNonUniformity	58.38666667
wavelet-LHL	gldm	GrayLevelNonUniformity	4.586666667
wavelet-LHL	gldm	SmallDependenceEmphasis	0.618877778
wavelet-LHL	gldm	SmallDependenceHighGrayLevelEmphasis	645.7116315
wavelet-LHL	gldm	DependenceNonUniformityNormalized	0.389244444
wavelet-LHL	gldm	LargeDependenceEmphasis	3.52
wavelet-LHL	gldm	LargeDependenceLowGrayLevelEmphasis	0.012038218
wavelet-LHL	gldm	DependenceVariance	0.6976
wavelet-LHL	gldm	LargeDependenceHighGrayLevelEmphasis	4189.426667
wavelet-LHL	gldm	SmallDependenceLowGrayLevelEmphasis	0.007895655
wavelet-LHL	gldm	LowGrayLevelEmphasis	0.008501693
wavelet-LHL	glcm	JointAverage	30.22501729
wavelet-LHL	glcm	SumAverage	60.45003458
wavelet-LHL	glcm	JointEntropy	7.345462821
wavelet-LHL	glcm	ClusterShade	1486.457273
wavelet-LHL	glcm	MaximumProbability	0.016402258
wavelet-LHL	glcm	Idmn	0.941618221
wavelet-LHL	glcm	JointEnergy	0.006586438
wavelet-LHL	glcm	Contrast	196.3057902
wavelet-LHL	glcm	DifferenceEntropy	4.378093831
wavelet-LHL	glcm	InverseVariance	0.124701284
wavelet-LHL	glcm	DifferenceVariance	77.40183139
wavelet-LHL	glcm	Idn	0.851593919
wavelet-LHL	glcm	Idm	0.116710104
wavelet-LHL	glcm	Correlation	0.282617341
wavelet-LHL	glcm	Autocorrelation	952.6542811
wavelet-LHL	glcm	SumEntropy	5.422213173
wavelet-LHL	glcm	SumSquares	137.1614249
wavelet-LHL	glcm	ClusterProminence	253284.6844
wavelet-LHL	glcm	Imc2	0.998237212
wavelet-LHL	glcm	Imc1	-0.557729785
wavelet-LHL	glcm	DifferenceAverage	10.3076958
wavelet-LHL	glcm	Id	0.196850243
wavelet-LHL	glcm	ClusterTendency	352.3399095
wavelet-LHL	firstorder	InterquartileRange	481.0168294
wavelet-LHL	firstorder	Skewness	-0.147165491
wavelet-LHL	firstorder	Uniformity	0.030577778
wavelet-LHL	firstorder	Median	-81.99498935
wavelet-LHL	firstorder	Energy	12583559.09
wavelet-LHL	firstorder	RobustMeanAbsoluteDeviation	198.7410922
wavelet-LHL	firstorder	MeanAbsoluteDeviation	248.3841502
wavelet-LHL	firstorder	TotalEnergy	339756095.4
wavelet-LHL	firstorder	Maximum	482.5178925
wavelet-LHL	firstorder	RootMeanSquared	289.6383848
wavelet-LHL	firstorder	90Percentile	310.6615645
wavelet-LHL	firstorder	Minimum	-785.7244801
wavelet-LHL	firstorder	Entropy	5.156989695
wavelet-LHL	firstorder	Range	1268.242373
wavelet-LHL	firstorder	Variance	81861.8289
wavelet-LHL	firstorder	10Percentile	-406.1436788
wavelet-LHL	firstorder	Kurtosis	1.993088021

wavelet-LHL	firstorder	Mean	-45.03959408
wavelet-LHL	glrlm	ShortRunLowGrayLevelEmphasis	0.008664686
wavelet-LHL	glrlm	GrayLevelVariance	131.0349651
wavelet-LHL	glrlm	LowGrayLevelRunEmphasis	0.008693958
wavelet-LHL	glrlm	GrayLevelNonUniformityNormalized	0.030393576
wavelet-LHL	glrlm	RunVariance	0.027180184
wavelet-LHL	glrlm	GrayLevelNonUniformity	4.440207643
wavelet-LHL	glrlm	LongRunEmphasis	1.083262409
wavelet-LHL	glrlm	ShortRunHighGrayLevelEmphasis	1048.440748
wavelet-LHL	glrlm	RunLengthNonUniformity	138.725142
wavelet-LHL	glrlm	ShortRunEmphasis	0.979794898
wavelet-LHL	glrlm	LongRunHighGrayLevelEmphasis	1171.161359
wavelet-LHL	glrlm	RunPercentage	0.973846154
wavelet-LHL	glrlm	LongRunLowGrayLevelEmphasis	0.008819494
wavelet-LHL	glrlm	RunEntropy	5.247801982
wavelet-LHL	glrlm	HighGrayLevelRunEmphasis	1072.843722
wavelet-LHL	glrlm	RunLengthNonUniformityNormalized	0.948724404
wavelet-LHL	glszm	GrayLevelVariance	135.6091136
wavelet-LHL	glszm	ZoneVariance	0.808828765
wavelet-LHL	glszm	GrayLevelNonUniformityNormalized	0.03008188
wavelet-LHL	glszm	SizeZoneNonUniformityNormalized	0.585617658
wavelet-LHL	glszm	SizeZoneNonUniformity	62.0754717
wavelet-LHL	glszm	GrayLevelNonUniformity	3.188679245
wavelet-LHL	glszm	LargeAreaEmphasis	2.811320755
wavelet-LHL	glszm	SmallAreaHighGrayLevelEmphasis	827.0670569
wavelet-LHL	glszm	ZonePercentage	0.706666667
wavelet-LHL	glszm	LargeAreaLowGrayLevelEmphasis	0.014003772
wavelet-LHL	glszm	LargeAreaHighGrayLevelEmphasis	3260.867925
wavelet-LHL	glszm	HighGrayLevelZoneEmphasis	1049.245283
wavelet-LHL	glszm	SmallAreaEmphasis	0.793022719
wavelet-LHL	glszm	LowGrayLevelZoneEmphasis	0.01137118
wavelet-LHL	glszm	ZoneEntropy	5.591095045
wavelet-LHL	glszm	SmallAreaLowGrayLevelEmphasis	0.011024877
wavelet-LHL	ngtdm	Coarseness	0.031347583
wavelet-LHL	ngtdm	Complexity	5298.975107
wavelet-LHL	ngtdm	Strength	22.31908527
wavelet-LHL	ngtdm	Contrast	1.211809201
wavelet-LHL	ngtdm	Busyness	0.034156539
wavelet-LHH	gldm	GrayLevelVariance	29.19222222
wavelet-LHH	gldm	HighGrayLevelEmphasis	406.8466667
wavelet-LHH	gldm	DependenceEntropy	5.603399885
wavelet-LHH	gldm	DependenceNonUniformity	48.22666667
wavelet-LHH	gldm	GrayLevelNonUniformity	9.24
wavelet-LHH	gldm	SmallDependenceEmphasis	0.532131481
wavelet-LHH	gldm	SmallDependenceHighGrayLevelEmphasis	223.3831148
wavelet-LHH	gldm	DependenceNonUniformityNormalized	0.321511111
wavelet-LHH	gldm	LargeDependenceEmphasis	4.826666667
wavelet-LHH	gldm	LargeDependenceLowGrayLevelEmphasis	0.021812263
wavelet-LHH	gldm	DependenceVariance	1.088888889
wavelet-LHH	gldm	LargeDependenceHighGrayLevelEmphasis	1861.553333
wavelet-LHH	gldm	SmallDependenceLowGrayLevelEmphasis	0.008551276
wavelet-LHH	gldm	LowGrayLevelEmphasis	0.010040987
wavelet-LHH	glcm	JointAverage	19.56625252

wavelet-LHH	glcm	SumAverage	39.13250503
wavelet-LHH	glcm	JointEntropy	6.956720478
wavelet-LHH	glcm	ClusterShade	85.39638537
wavelet-LHH	glcm	MaximumProbability	0.023319857
wavelet-LHH	glcm	Idmn	0.964877165
wavelet-LHH	glcm	JointEnergy	0.009193271
wavelet-LHH	glcm	Contrast	54.24930495
wavelet-LHH	glcm	DifferenceEntropy	3.76141172
wavelet-LHH	glcm	InverseVariance	0.166053698
wavelet-LHH	glcm	DifferenceVariance	20.54531306
wavelet-LHH	glcm	Idn	0.874938561
wavelet-LHH	glcm	Idm	0.159432315
wavelet-LHH	glcm	Correlation	-0.031788498
wavelet-LHH	glcm	Autocorrelation	381.985068
wavelet-LHH	glcm	SumEntropy	4.51835461
wavelet-LHH	glcm	SumSquares	26.25111095
wavelet-LHH	glcm	ClusterProminence	9061.775927
wavelet-LHH	glcm	Imc2	0.968419079
wavelet-LHH	glcm	Imc1	-0.336707413
wavelet-LHH	glcm	DifferenceAverage	5.737118697
wavelet-LHH	glcm	Id	0.253337596
wavelet-LHH	glcm	ClusterTendency	50.75513884
wavelet-LHH	firstorder	InterquartileRange	179.3585338
wavelet-LHH	firstorder	Skewness	0.072794105
wavelet-LHH	firstorder	Uniformity	0.0616
wavelet-LHH	firstorder	Median	-12.79474155
wavelet-LHH	firstorder	Energy	2769475.932
wavelet-LHH	firstorder	RobustMeanAbsoluteDeviation	73.58170008
wavelet-LHH	firstorder	MeanAbsoluteDeviation	106.5572572
wavelet-LHH	firstorder	TotalEnergy	74775850.18
wavelet-LHH	firstorder	Maximum	448.6144325
wavelet-LHH	firstorder	RootMeanSquared	135.8792585
wavelet-LHH	firstorder	90Percentile	162.5987887
wavelet-LHH	firstorder	Minimum	-474.5352574
wavelet-LHH	firstorder	Entropy	4.26905276
wavelet-LHH	firstorder	Range	923.1496899
wavelet-LHH	firstorder	Variance	18461.55526
wavelet-LHH	firstorder	10Percentile	-172.1847272
wavelet-LHH	firstorder	Kurtosis	3.84268661
wavelet-LHH	firstorder	Mean	-1.271856262
wavelet-LHH	glrlm	ShortRunLowGrayLevelEmphasis	0.010212845
wavelet-LHH	glrlm	GrayLevelVariance	29.66360016
wavelet-LHH	glrlm	LowGrayLevelRunEmphasis	0.010300463
wavelet-LHH	glrlm	GrayLevelNonUniformityNormalized	0.060894639
wavelet-LHH	glrlm	RunVariance	0.035818576
wavelet-LHH	glrlm	GrayLevelNonUniformity	8.806382595
wavelet-LHH	glrlm	LongRunEmphasis	1.112429475
wavelet-LHH	glrlm	ShortRunHighGrayLevelEmphasis	396.833498
wavelet-LHH	glrlm	RunLengthNonUniformity	134.3198706
wavelet-LHH	glrlm	ShortRunEmphasis	0.971892631
wavelet-LHH	glrlm	LongRunHighGrayLevelEmphasis	450.9628288
wavelet-LHH	glrlm	RunPercentage	0.964102564
wavelet-LHH	glrlm	LongRunLowGrayLevelEmphasis	0.010650937

wavelet-LHH	glrlm	RunEntropy	4.432366431
wavelet-LHH	glrlm	HighGrayLevelRunEmphasis	407.6593642
wavelet-LHH	glrlm	RunLengthNonUniformityNormalized	0.928362847
wavelet-LHH	glszm	GrayLevelVariance	35.17400856
wavelet-LHH	glszm	ZoneVariance	1.635102324
wavelet-LHH	glszm	GrayLevelNonUniformityNormalized	0.055382125
wavelet-LHH	glszm	SizeZoneNonUniformityNormalized	0.508613713
wavelet-LHH	glszm	SizeZoneNonUniformity	47.30107527
wavelet-LHH	glszm	GrayLevelNonUniformity	5.150537634
wavelet-LHH	glszm	LargeAreaEmphasis	4.23655914
wavelet-LHH	glszm	SmallAreaHighGrayLevelEmphasis	312.1020131
wavelet-LHH	glszm	ZonePercentage	0.62
wavelet-LHH	glszm	LargeAreaLowGrayLevelEmphasis	0.024327079
wavelet-LHH	glszm	LargeAreaHighGrayLevelEmphasis	1646.16129
wavelet-LHH	glszm	HighGrayLevelZoneEmphasis	414.3763441
wavelet-LHH	glszm	SmallAreaEmphasis	0.74103644
wavelet-LHH	glszm	LowGrayLevelZoneEmphasis	0.014267864
wavelet-LHH	glszm	ZoneEntropy	5.322926592
wavelet-LHH	glszm	SmallAreaLowGrayLevelEmphasis	0.013421932
wavelet-LHH	ngtdm	Coarseness	0.032532823
wavelet-LHH	ngtdm	Complexity	1232.404655
wavelet-LHH	ngtdm	Strength	8.202860327
wavelet-LHH	ngtdm	Contrast	0.398274478
wavelet-LHH	ngtdm	Busyness	0.067914686
wavelet-LLH	gldm	GrayLevelVariance	115.9264
wavelet-LLH	gldm	HighGrayLevelEmphasis	665.36
wavelet-LLH	gldm	DependenceEntropy	5.841137131
wavelet-LLH	gldm	DependenceNonUniformity	63.17333333
wavelet-LLH	gldm	GrayLevelNonUniformity	4.906666667
wavelet-LLH	gldm	SmallDependenceEmphasis	0.661157407
wavelet-LLH	gldm	SmallDependenceHighGrayLevelEmphasis	488.4278241
wavelet-LLH	gldm	DependenceNonUniformityNormalized	0.421155556
wavelet-LLH	gldm	LargeDependenceEmphasis	3.293333333
wavelet-LLH	gldm	LargeDependenceLowGrayLevelEmphasis	0.020577737
wavelet-LLH	gldm	DependenceVariance	0.690488889
wavelet-LLH	gldm	LargeDependenceHighGrayLevelEmphasis	1817.693333
wavelet-LLH	gldm	SmallDependenceLowGrayLevelEmphasis	0.011704278
wavelet-LLH	gldm	LowGrayLevelEmphasis	0.012936852
wavelet-LLH	glcm	JointAverage	22.83243953
wavelet-LLH	glcm	SumAverage	45.66487907
wavelet-LLH	glcm	JointEntropy	7.374318883
wavelet-LLH	glcm	ClusterShade	2378.659326
wavelet-LLH	glcm	MaximumProbability	0.013927461
wavelet-LLH	glcm	Idmn	0.947550661
wavelet-LLH	glcm	JointEnergy	0.006379796
wavelet-LLH	glcm	Contrast	183.1583878
wavelet-LLH	glcm	DifferenceEntropy	4.404127042
wavelet-LLH	glcm	InverseVariance	0.102464357
wavelet-LLH	glcm	DifferenceVariance	65.06065288
wavelet-LLH	glcm	Idn	0.851175461
wavelet-LLH	glcm	Idm	0.100415836
wavelet-LLH	glcm	Correlation	0.168870356
wavelet-LLH	glcm	Autocorrelation	539.2583635

wavelet-LLH	glcm	SumEntropy	5.276688254
wavelet-LLH	glcm	SumSquares	109.3800114
wavelet-LLH	glcm	ClusterProminence	238703.6164
wavelet-LLH	glcm	Imc2	0.997795134
wavelet-LLH	glcm	Imc1	-0.541512113
wavelet-LLH	glcm	DifferenceAverage	10.45191415
wavelet-LLH	glcm	Id	0.179832465
wavelet-LLH	glcm	ClusterTendency	254.361658
wavelet-LLH	firstorder	InterquartileRange	390.0546299
wavelet-LLH	firstorder	Skewness	0.387859028
wavelet-LLH	firstorder	Uniformity	0.032711111
wavelet-LLH	firstorder	Median	-89.96017087
wavelet-LLH	firstorder	Energy	11290103.82
wavelet-LLH	firstorder	RobustMeanAbsoluteDeviation	160.0048801
wavelet-LLH	firstorder	MeanAbsoluteDeviation	220.2816604
wavelet-LLH	firstorder	TotalEnergy	304832803.1
wavelet-LLH	firstorder	Maximum	707.3233694
wavelet-LLH	firstorder	RootMeanSquared	274.3489726
wavelet-LLH	firstorder	90Percentile	337.0680571
wavelet-LLH	firstorder	Minimum	-624.6565683
wavelet-LLH	firstorder	Entropy	5.133330528
wavelet-LLH	firstorder	Range	1331.979938
wavelet-LLH	firstorder	Variance	72592.43786
wavelet-LLH	firstorder	10Percentile	-389.7006874
wavelet-LLH	firstorder	Kurtosis	2.68909952
wavelet-LLH	firstorder	Mean	-51.71963769
wavelet-LLH	glrlm	ShortRunLowGrayLevelEmphasis	0.013108771
wavelet-LLH	glrlm	GrayLevelVariance	116.8731022
wavelet-LLH	glrlm	LowGrayLevelRunEmphasis	0.013168506
wavelet-LLH	glrlm	GrayLevelNonUniformityNormalized	0.032417068
wavelet-LLH	glrlm	RunVariance	0.025693647
wavelet-LLH	glrlm	GrayLevelNonUniformity	4.747844834
wavelet-LLH	glrlm	LongRunEmphasis	1.075338895
wavelet-LLH	glrlm	ShortRunHighGrayLevelEmphasis	659.901108
wavelet-LLH	glrlm	RunLengthNonUniformity	139.9099579
wavelet-LLH	glrlm	ShortRunEmphasis	0.982352361
wavelet-LLH	glrlm	LongRunHighGrayLevelEmphasis	707.0631781
wavelet-LLH	glrlm	RunPercentage	0.976410256
wavelet-LLH	glrlm	LongRunLowGrayLevelEmphasis	0.013431264
wavelet-LLH	glrlm	RunEntropy	5.219406137
wavelet-LLH	glrlm	HighGrayLevelRunEmphasis	669.1112998
wavelet-LLH	glrlm	RunLengthNonUniformityNormalized	0.9548449
wavelet-LLH	glszm	GrayLevelVariance	128.6215405
wavelet-LLH	glszm	ZoneVariance	0.714390066
wavelet-LLH	glszm	GrayLevelNonUniformityNormalized	0.030598166
wavelet-LLH	glszm	SizeZoneNonUniformityNormalized	0.637529421
wavelet-LLH	glszm	SizeZoneNonUniformity	70.76576577
wavelet-LLH	glszm	GrayLevelNonUniformity	3.396396396
wavelet-LLH	glszm	LargeAreaEmphasis	2.540540541
wavelet-LLH	glszm	SmallAreaHighGrayLevelEmphasis	625.8249249
wavelet-LLH	glszm	ZonePercentage	0.74
wavelet-LLH	glszm	LargeAreaLowGrayLevelEmphasis	0.021375085
wavelet-LLH	glszm	LargeAreaHighGrayLevelEmphasis	1456.036036

wavelet-LLH	glszm	HighGrayLevelZoneEmphasis	721.1711712
wavelet-LLH	glszm	SmallAreaEmphasis	0.825795796
wavelet-LLH	glszm	LowGrayLevelZoneEmphasis	0.016229524
wavelet-LLH	glszm	ZoneEntropy	5.698713984
wavelet-LLH	glszm	SmallAreaLowGrayLevelEmphasis	0.015560011
wavelet-LLH	ngtdm	Coarseness	0.034417391
wavelet-LLH	ngtdm	Complexity	5540.922869
wavelet-LLH	ngtdm	Strength	24.33941996
wavelet-LLH	ngtdm	Contrast	0.955827754
wavelet-LLH	ngtdm	Busyness	0.03926645
wavelet-LLL	gldm	GrayLevelVariance	592.3788889
wavelet-LLL	gldm	HighGrayLevelEmphasis	2686.966667
wavelet-LLL	gldm	DependenceEntropy	6.219996607
wavelet-LLL	gldm	DependenceNonUniformity	100.4133333
wavelet-LLL	gldm	GrayLevelNonUniformity	2.693333333
wavelet-LLL	gldm	SmallDependenceEmphasis	0.844074074
wavelet-LLL	gldm	SmallDependenceHighGrayLevelEmphasis	2080.540741
wavelet-LLL	gldm	DependenceNonUniformityNormalized	0.669422222
wavelet-LLL	gldm	LargeDependenceEmphasis	1.653333333
wavelet-LLL	gldm	LargeDependenceLowGrayLevelEmphasis	0.010759005
wavelet-LLL	gldm	DependenceVariance	0.181155556
wavelet-LLL	gldm	LargeDependenceHighGrayLevelEmphasis	5302.3
wavelet-LLL	gldm	SmallDependenceLowGrayLevelEmphasis	0.009633176
wavelet-LLL	gldm	LowGrayLevelEmphasis	0.009857416
wavelet-LLL	glcm	JointAverage	53.04817999
wavelet-LLL	glcm	SumAverage	106.09636
wavelet-LLL	glcm	JointEntropy	7.550434452
wavelet-LLL	glcm	ClusterShade	-7457.695187
wavelet-LLL	glcm	MaximumProbability	0.010382724
wavelet-LLL	glcm	Idmn	0.922417374
wavelet-LLL	glcm	JointEnergy	0.005435001
wavelet-LLL	glcm	Contrast	798.950636
wavelet-LLL	glcm	DifferenceEntropy	5.255795443
wavelet-LLL	glcm	InverseVariance	0.043725601
wavelet-LLL	glcm	DifferenceVariance	246.5530197
wavelet-LLL	glcm	Idn	0.815249098
wavelet-LLL	glcm	Idm	0.041087301
wavelet-LLL	glcm	Correlation	0.317358617
wavelet-LLL	glcm	Autocorrelation	2996.95585
wavelet-LLL	glcm	SumEntropy	6.022997917
wavelet-LLL	glcm	SumSquares	581.3290498
wavelet-LLL	glcm	ClusterProminence	5198879.454
wavelet-LLL	glcm	Imc2	0.999867576
wavelet-LLL	glcm	Imc1	-0.707431463
wavelet-LLL	glcm	DifferenceAverage	23.03635584
wavelet-LLL	glcm	Id	0.096446364
wavelet-LLL	glcm	ClusterTendency	1526.365563
wavelet-LLL	firstorder	InterquartileRange	998.6334
wavelet-LLL	firstorder	Skewness	0.150245887
wavelet-LLL	firstorder	Uniformity	0.017955556
wavelet-LLL	firstorder	Median	-822.0919506
wavelet-LLL	firstorder	Energy	162198470.3
wavelet-LLL	firstorder	RobustMeanAbsoluteDeviation	409.2484338

wavelet-LLL	firstorder	MeanAbsoluteDeviation	522.1791385
wavelet-LLL	firstorder	TotalEnergy	4379358697
wavelet-LLL	firstorder	Maximum	317.2817925
wavelet-LLL	firstorder	RootMeanSquared	1039.866883
wavelet-LLL	firstorder	90Percentile	3.385748608
wavelet-LLL	firstorder	Minimum	-1965.107996
wavelet-LLL	firstorder	Entropy	5.955005901
wavelet-LLL	firstorder	Range	2282.389788
wavelet-LLL	firstorder	Variance	369912.3154
wavelet-LLL	firstorder	10Percentile	-1592.968896
wavelet-LLL	firstorder	Kurtosis	1.943043666
wavelet-LLL	firstorder	Mean	-843.4517293
wavelet-LLL	glrlm	ShortRunLowGrayLevelEmphasis	0.009919089
wavelet-LLL	glrlm	GrayLevelVariance	590.154447
wavelet-LLL	glrlm	LowGrayLevelRunEmphasis	0.009927851
wavelet-LLL	glrlm	GrayLevelNonUniformityNormalized	0.0179539
wavelet-LLL	glrlm	RunVariance	0.008202903
wavelet-LLL	glrlm	GrayLevelNonUniformity	2.670992234
wavelet-LLL	glrlm	LongRunEmphasis	1.024991165
wavelet-LLL	glrlm	ShortRunHighGrayLevelEmphasis	2651.719635
wavelet-LLL	glrlm	RunLengthNonUniformity	146.3452704
wavelet-LLL	glrlm	ShortRunEmphasis	0.993752209
wavelet-LLL	glrlm	LongRunHighGrayLevelEmphasis	2775.21358
wavelet-LLL	glrlm	RunPercentage	0.991794872
wavelet-LLL	glrlm	LongRunLowGrayLevelEmphasis	0.009962895
wavelet-LLL	glrlm	RunEntropy	5.971268464
wavelet-LLL	glrlm	HighGrayLevelRunEmphasis	2676.418424
wavelet-LLL	glrlm	RunLengthNonUniformityNormalized	0.983594194
wavelet-LLL	glszm	GrayLevelVariance	559.5166518
wavelet-LLL	glszm	ZoneVariance	0.120071285
wavelet-LLL	glszm	GrayLevelNonUniformityNormalized	0.017821341
wavelet-LLL	glszm	SizeZoneNonUniformityNormalized	0.799621297
wavelet-LLL	glszm	SizeZoneNonUniformity	107.1492537
wavelet-LLL	glszm	GrayLevelNonUniformity	2.388059701
wavelet-LLL	glszm	LargeAreaEmphasis	1.373134328
wavelet-LLL	glszm	SmallAreaHighGrayLevelEmphasis	2210.959784
wavelet-LLL	glszm	ZonePercentage	0.893333333
wavelet-LLL	glszm	LargeAreaLowGrayLevelEmphasis	0.011372389
wavelet-LLL	glszm	LargeAreaHighGrayLevelEmphasis	4047.350746
wavelet-LLL	glszm	HighGrayLevelZoneEmphasis	2535.783582
wavelet-LLL	glszm	SmallAreaEmphasis	0.915008292
wavelet-LLL	glszm	LowGrayLevelZoneEmphasis	0.010866603
wavelet-LLL	glszm	ZoneEntropy	6.160691336
wavelet-LLL	glszm	SmallAreaLowGrayLevelEmphasis	0.010741452
wavelet-LLL	ngtdm	Coarseness	0.025571703
wavelet-LLL	ngtdm	Complexity	37728.88414
wavelet-LLL	ngtdm	Strength	77.68481911
wavelet-LLL	ngtdm	Contrast	3.686496993
wavelet-LLL	ngtdm	Busyness	0.014614374
wavelet-HHL	gldm	GrayLevelVariance	10.34333333
wavelet-HHL	gldm	HighGrayLevelEmphasis	100.5933333
wavelet-HHL	gldm	DependenceEntropy	5.330150818
wavelet-HHL	gldm	DependenceNonUniformity	36.16

wavelet-HHL	gldm	GrayLevelNonUniformity	13.4
wavelet-HHL	gldm	SmallDependenceEmphasis	0.447239645
wavelet-HHL	gldm	SmallDependenceHighGrayLevelEmphasis	52.10530737
wavelet-HHL	gldm	DependenceNonUniformityNormalized	0.241066667
wavelet-HHL	gldm	LargeDependenceEmphasis	7.866666667
wavelet-HHL	gldm	LargeDependenceLowGrayLevelEmphasis	0.159292099
wavelet-HHL	gldm	DependenceVariance	2.106666667
wavelet-HHL	gldm	LargeDependenceHighGrayLevelEmphasis	712.7533333
wavelet-HHL	gldm	SmallDependenceLowGrayLevelEmphasis	0.01109884
wavelet-HHL	gldm	LowGrayLevelEmphasis	0.029067737
wavelet-HHL	glcm	JointAverage	9.618138659
wavelet-HHL	glcm	SumAverage	19.23627732
wavelet-HHL	glcm	JointEntropy	6.436472917
wavelet-HHL	glcm	ClusterShade	-15.70206891
wavelet-HHL	glcm	MaximumProbability	0.031598491
wavelet-HHL	glcm	Idmn	0.939617902
wavelet-HHL	glcm	JointEnergy	0.013657299
wavelet-HHL	glcm	Contrast	20.92300513
wavelet-HHL	glcm	DifferenceEntropy	3.201525914
wavelet-HHL	glcm	InverseVariance	0.249550378
wavelet-HHL	glcm	DifferenceVariance	7.614512638
wavelet-HHL	glcm	Idn	0.83824208
wavelet-HHL	glcm	Idm	0.232932138
wavelet-HHL	glcm	Correlation	-0.005729928
wavelet-HHL	glcm	Autocorrelation	92.46993768
wavelet-HHL	glcm	SumEntropy	4.009192319
wavelet-HHL	glcm	SumSquares	10.40394315
wavelet-HHL	glcm	ClusterProminence	1393.982909
wavelet-HHL	glcm	Imc2	0.900121673
wavelet-HHL	glcm	Imc1	-0.230261638
wavelet-HHL	glcm	DifferenceAverage	3.622393851
wavelet-HHL	glcm	Id	0.326743596
wavelet-HHL	glcm	ClusterTendency	20.69276748
wavelet-HHL	firstorder	InterquartileRange	116.2854893
wavelet-HHL	firstorder	Skewness	0.04093656
wavelet-HHL	firstorder	Uniformity	0.089333333
wavelet-HHL	firstorder	Median	-5.566997623
wavelet-HHL	firstorder	Energy	957510.8705
wavelet-HHL	firstorder	RobustMeanAbsoluteDeviation	47.08506055
wavelet-HHL	firstorder	MeanAbsoluteDeviation	65.07232817
wavelet-HHL	firstorder	TotalEnergy	25852793.5
wavelet-HHL	firstorder	Maximum	192.9708385
wavelet-HHL	firstorder	RootMeanSquared	79.89621896
wavelet-HHL	firstorder	90Percentile	107.8880931
wavelet-HHL	firstorder	Minimum	-220.2024926
wavelet-HHL	firstorder	Entropy	3.660964071
wavelet-HHL	firstorder	Range	413.1733311
wavelet-HHL	firstorder	Variance	6383.138192
wavelet-HHL	firstorder	10Percentile	-97.16323078
wavelet-HHL	firstorder	Kurtosis	2.720205817
wavelet-HHL	firstorder	Mean	-0.517311972
wavelet-HHL	glrlm	ShortRunLowGrayLevelEmphasis	0.028443512
wavelet-HHL	glrlm	GrayLevelVariance	10.66242792

wavelet-HHL	glrlm	LowGrayLevelRunEmphasis	0.02941077
wavelet-HHL	glrlm	GrayLevelNonUniformityNormalized	0.087632532
wavelet-HHL	glrlm	RunVariance	0.059176858
wavelet-HHL	glrlm	GrayLevelNonUniformity	12.43767889
wavelet-HHL	glrlm	LongRunEmphasis	1.177179117
wavelet-HHL	glrlm	ShortRunHighGrayLevelEmphasis	97.64274705
wavelet-HHL	glrlm	RunLengthNonUniformity	127.383271
wavelet-HHL	glrlm	ShortRunEmphasis	0.958793052
wavelet-HHL	glrlm	LongRunHighGrayLevelEmphasis	116.9194134
wavelet-HHL	glrlm	RunPercentage	0.946153846
wavelet-HHL	glrlm	LongRunLowGrayLevelEmphasis	0.03344436
wavelet-HHL	glrlm	RunEntropy	3.922561108
wavelet-HHL	glrlm	HighGrayLevelRunEmphasis	101.2929878
wavelet-HHL	glrlm	RunLengthNonUniformityNormalized	0.89713929
wavelet-HHL	glszm	GrayLevelVariance	15.04462222
wavelet-HHL	glszm	ZoneVariance	8.373333333
wavelet-HHL	glszm	GrayLevelNonUniformityNormalized	0.082311111
wavelet-HHL	glszm	SizeZoneNonUniformityNormalized	0.525688889
wavelet-HHL	glszm	SizeZoneNonUniformity	39.42666667
wavelet-HHL	glszm	GrayLevelNonUniformity	6.173333333
wavelet-HHL	glszm	LargeAreaEmphasis	12.37333333
wavelet-HHL	glszm	SmallAreaHighGrayLevelEmphasis	91.50391276
wavelet-HHL	glszm	ZonePercentage	0.5
wavelet-HHL	glszm	LargeAreaLowGrayLevelEmphasis	0.209272994
wavelet-HHL	glszm	LargeAreaHighGrayLevelEmphasis	1141.213333
wavelet-HHL	glszm	HighGrayLevelZoneEmphasis	113.1866667
wavelet-HHL	glszm	SmallAreaEmphasis	0.750746091
wavelet-HHL	glszm	LowGrayLevelZoneEmphasis	0.030793764
wavelet-HHL	glszm	ZoneEntropy	4.725551112
wavelet-HHL	glszm	SmallAreaLowGrayLevelEmphasis	0.016732869
wavelet-HHL	ngtdm	Coarseness	0.036856118
wavelet-HHL	ngtdm	Complexity	246.1014868
wavelet-HHL	ngtdm	Strength	2.613372494
wavelet-HHL	ngtdm	Contrast	0.229332421
wavelet-HHL	ngtdm	Busyness	0.230275015
wavelet-HLH	gldm	GrayLevelVariance	21.46026667
wavelet-HLH	gldm	HighGrayLevelEmphasis	192.5466667
wavelet-HLH	gldm	DependenceEntropy	5.496687152
wavelet-HLH	gldm	DependenceNonUniformity	43.37333333
wavelet-HLH	gldm	GrayLevelNonUniformity	10.25333333
wavelet-HLH	gldm	SmallDependenceEmphasis	0.510494331
wavelet-HLH	gldm	SmallDependenceHighGrayLevelEmphasis	99.76985518
wavelet-HLH	gldm	DependenceNonUniformityNormalized	0.289155556
wavelet-HLH	gldm	LargeDependenceEmphasis	6.04
wavelet-HLH	gldm	LargeDependenceLowGrayLevelEmphasis	0.052828614
wavelet-HLH	gldm	DependenceVariance	1.601955556
wavelet-HLH	gldm	LargeDependenceHighGrayLevelEmphasis	1069.253333
wavelet-HLH	gldm	SmallDependenceLowGrayLevelEmphasis	0.011492854
wavelet-HLH	gldm	LowGrayLevelEmphasis	0.015234851
wavelet-HLH	glcm	JointAverage	13.08932251
wavelet-HLH	glcm	SumAverage	26.17864503
wavelet-HLH	glcm	JointEntropy	6.839526447
wavelet-HLH	glcm	ClusterShade	88.27295375

wavelet-HLH	glcm	MaximumProbability	0.024412948
wavelet-HLH	glcm	Idmn	0.945691604
wavelet-HLH	glcm	JointEnergy	0.010035332
wavelet-HLH	glcm	Contrast	43.43456975
wavelet-HLH	glcm	DifferenceEntropy	3.64756916
wavelet-HLH	glcm	InverseVariance	0.168733721
wavelet-HLH	glcm	DifferenceVariance	15.65749819
wavelet-HLH	glcm	Idn	0.8458652
wavelet-HLH	glcm	Idm	0.171421201
wavelet-HLH	glcm	Correlation	-0.041494108
wavelet-HLH	glcm	Autocorrelation	170.5846222
wavelet-HLH	glcm	SumEntropy	4.393346098
wavelet-HLH	glcm	SumSquares	20.90793482
wavelet-HLH	glcm	ClusterProminence	5393.211424
wavelet-HLH	glcm	Imc2	0.95557333
wavelet-HLH	glcm	Imc1	-0.305833896
wavelet-HLH	glcm	DifferenceAverage	5.207918324
wavelet-HLH	glcm	Id	0.267108963
wavelet-HLH	glcm	ClusterTendency	40.19716954
wavelet-HLH	firstorder	InterquartileRange	159.3250191
wavelet-HLH	firstorder	Skewness	0.390856327
wavelet-HLH	firstorder	Uniformity	0.068355556
wavelet-HLH	firstorder	Median	11.20945887
wavelet-HLH	firstorder	Energy	2041109.582
wavelet-HLH	firstorder	RobustMeanAbsoluteDeviation	64.13203278
wavelet-HLH	firstorder	MeanAbsoluteDeviation	92.1469088
wavelet-HLH	firstorder	TotalEnergy	55109958.72
wavelet-HLH	firstorder	Maximum	342.2988006
wavelet-HLH	firstorder	RootMeanSquared	116.6507489
wavelet-HLH	firstorder	90Percentile	158.1499967
wavelet-HLH	firstorder	Minimum	-277.3353521
wavelet-HLH	firstorder	Entropy	4.089020258
wavelet-HLH	firstorder	Range	619.6341527
wavelet-HLH	firstorder	Variance	13381.95063
wavelet-HLH	firstorder	10Percentile	-127.3192282
wavelet-HLH	firstorder	Kurtosis	2.908994798
wavelet-HLH	firstorder	Mean	15.01487873
wavelet-HLH	glrlm	ShortRunLowGrayLevelEmphasis	0.015332523
wavelet-HLH	glrlm	GrayLevelVariance	21.87905822
wavelet-HLH	glrlm	LowGrayLevelRunEmphasis	0.015577965
wavelet-HLH	glrlm	GrayLevelNonUniformityNormalized	0.067250325
wavelet-HLH	glrlm	RunVariance	0.04684098
wavelet-HLH	glrlm	GrayLevelNonUniformity	9.658019763
wavelet-HLH	glrlm	LongRunEmphasis	1.138794114
wavelet-HLH	glrlm	ShortRunHighGrayLevelEmphasis	187.3700909
wavelet-HLH	glrlm	RunLengthNonUniformity	132.010822
wavelet-HLH	glrlm	ShortRunEmphasis	0.967753014
wavelet-HLH	glrlm	LongRunHighGrayLevelEmphasis	217.9974233
wavelet-HLH	glrlm	RunPercentage	0.957435897
wavelet-HLH	glrlm	LongRunLowGrayLevelEmphasis	0.016625501
wavelet-HLH	glrlm	RunEntropy	4.286012651
wavelet-HLH	glrlm	HighGrayLevelRunEmphasis	193.1487669
wavelet-HLH	glrlm	RunLengthNonUniformityNormalized	0.918694929

wavelet-HLH	glszm	GrayLevelVariance	27.12177169
wavelet-HLH	glszm	ZoneVariance	2.639979339
wavelet-HLH	glszm	GrayLevelNonUniformityNormalized	0.06017562
wavelet-HLH	glszm	SizeZoneNonUniformityNormalized	0.52427686
wavelet-HLH	glszm	SizeZoneNonUniformity	46.13636364
wavelet-HLH	glszm	GrayLevelNonUniformity	5.295454545
wavelet-HLH	glszm	LargeAreaEmphasis	5.545454545
wavelet-HLH	glszm	SmallAreaHighGrayLevelEmphasis	146.9499974
wavelet-HLH	glszm	ZonePercentage	0.586666667
wavelet-HLH	glszm	LargeAreaLowGrayLevelEmphasis	0.05254178
wavelet-HLH	glszm	LargeAreaHighGrayLevelEmphasis	1016.340909
wavelet-HLH	glszm	HighGrayLevelZoneEmphasis	197.6022727
wavelet-HLH	glszm	SmallAreaEmphasis	0.751932438
wavelet-HLH	glszm	LowGrayLevelZoneEmphasis	0.020634843
wavelet-HLH	glszm	ZoneEntropy	5.142314257
wavelet-HLH	glszm	SmallAreaLowGrayLevelEmphasis	0.01868267
wavelet-HLH	ngtdm	Coarseness	0.034490746
wavelet-HLH	ngtdm	Complexity	726.5286976
wavelet-HLH	ngtdm	Strength	5.208054058
wavelet-HLH	ngtdm	Contrast	0.359888039
wavelet-HLH	ngtdm	Busyness	0.130224957

Image type	Feature Class	Feature Name	18: RTSTRUCT: C99-DNU_segment_Tumo_ RUL
general	info	BoundingBox	(1, 0, 1, 8, 11, 3)
general	info	EnabledImageTypes	{u'Original': {}, u'Wavelet': {}}
general	info	GeneralSettings	{'distances': [1], 'additionalInfo': True, 'force2D': False, 'interpolator': 'sitkBSpline', 'resampledPixelSpacing': [3.0], 'label': 1, 'normalizeScale': 1, 'normalize': False, 'force2Ddimension': 0, 'removeOutliers': None, 'minimumROISize': None, u'binWidth': 25.0, 'minimumROIDimensions': 1, u'correctMask': True, 'preCrop': False, u'symmetricalGLCM': True, 'resegmentRange': None, 'padDistance': 5}
general	info	ImageHash	1005effc4c41e5b03ca8944b795fe7c464f2fe6c
general	info	ImageSpacing	(0.9765624999999998, 0.9765624999999998, 1.999999999999999)
general	info	MaskHash	40c8f073805f24a244bec4bc0448c1896a5c73ad
general	info	NumpyVersion	1.13.1
general	info	PyWaveletVersion	0.5.2
general	info	SimpleITKVersion	1.1.0
general	info	Version	1.3.0.post102+g1b0eb74
general	info	VolumeNum	1
general	info	VoxelNum	162
original	shape	Maximum3DDiameter	33.67491648
original	shape	Maximum2DDiameterSlice	33.54101966
original	shape	Sphericity	0.738667979
original	shape	MinorAxis	20.5442125
original	shape	Elongation	0.63343432
original	shape	SurfaceVolumeRatio	0.400321202
original	shape	Volume	4374
original	shape	MajorAxis	32.43305872
original	shape	SurfaceArea	1751.004938
original	shape	Flatness	0.300004299
original	shape	LeastAxis	9.730057047
original	shape	Maximum2DDiameterColumn	21.84032967
original	shape	Maximum2DDiameterRow	27.65863337
original	gldm	GrayLevelVariance	80.39719555
original	gldm	HighGrayLevelEmphasis	191.2962963
original	gldm	DependenceEntropy	5.580289318
original	gldm	DependenceNonUniformity	45.90123457
original	gldm	GrayLevelNonUniformity	12.33333333
original	gldm	SmallDependenceEmphasis	0.508620813
original	gldm	SmallDependenceHighGrayLevelEmphasis	157.087018
original	gldm	DependenceNonUniformityNormalized	0.283340954
original	gldm	LargeDependenceEmphasis	6.728395062
original	gldm	LargeDependenceLowGrayLevelEmphasis	0.598171448
original	gldm	DependenceVariance	2.007163542
original	gldm	LargeDependenceHighGrayLevelEmphasis	383.8271605
original	gldm	SmallDependenceLowGrayLevelEmphasis	0.014446356
original	gldm	LowGrayLevelEmphasis	0.056702709
original	glcm	JointAverage	11.77121367
original	glcm	SumAverage	23.54242734

original	glcm	JointEntropy	6.979275388
original	glcm	ClusterShade	3564.13441
original	glcm	MaximumProbability	0.040213448
original	glcm	Idmn	0.939431741
original	glcm	JointEnergy	0.01006735
original	glcm	Contrast	120.5866215
original	glcm	DifferenceEntropy	4.170411828
original	glcm	InverseVariance	0.173861958
original	glcm	DifferenceVariance	54.59524025
original	glcm	Idn	0.850877092
original	glcm	Idm	0.174854154
original	glcm	Correlation	0.357178729
original	glcm	Autocorrelation	171.9181171
original	glcm	SumEntropy	5.17288971
original	glcm	SumSquares	93.385822
original	glcm	ClusterProminence	191552.3233
original	glcm	Imc2	0.991186221
original	glcm	Imc1	-0.459444724
original	glcm	DifferenceAverage	8.030834932
original	glcm	Id	0.251874892
original	glcm	ClusterTendency	252.9566665
original	firstorder	InterquartileRange	320.5
original	firstorder	Skewness	1.289371194
original	firstorder	Uniformity	0.076131687
original	firstorder	Median	-763.5
original	firstorder	Energy	81968408
original	firstorder	RobustMeanAbsoluteDeviation	126.7786865
original	firstorder	MeanAbsoluteDeviation	182.2130773
original	firstorder	TotalEnergy	2213147016
original	firstorder	Maximum	65
original	firstorder	RootMeanSquared	711.3211842
original	firstorder	90Percentile	-352.5
original	firstorder	Minimum	-905
original	firstorder	Entropy	4.340812044
original	firstorder	Range	970
original	firstorder	Variance	50002.75995
original	firstorder	10Percentile	-867.9
original	firstorder	Kurtosis	3.949116309
original	firstorder	Mean	-675.2592593
original	glrlm	ShortRunLowGrayLevelEmphasis	0.051995441
original	glrlm	GrayLevelVariance	81.85798146
original	glrlm	LowGrayLevelRunEmphasis	0.05508125
original	glrlm	GrayLevelNonUniformityNormalized	0.071591716
original	glrlm	RunVariance	0.051125715
original	glrlm	GrayLevelNonUniformity	11.08448948
original	glrlm	LongRunEmphasis	1.149781734
original	glrlm	ShortRunHighGrayLevelEmphasis	196.6223482
original	glrlm	RunLengthNonUniformity	141.5704581
original	glrlm	ShortRunEmphasis	0.965736659
original	glrlm	LongRunHighGrayLevelEmphasis	204.9084341
original	glrlm	RunPercentage	0.954890788
original	glrlm	LongRunLowGrayLevelEmphasis	0.068726456
original	glrlm	RunEntropy	4.573033489
original	glrlm	HighGrayLevelRunEmphasis	198.2534203
original	glrlm	RunLengthNonUniformityNormalized	0.914241658

original	glszm	GrayLevelVariance	93.98271468
original	glszm	ZoneVariance	3.344709141
original	glszm	GrayLevelNonUniformityNormalized	0.044875346
original	glszm	SizeZoneNonUniformityNormalized	0.532188366
original	glszm	SizeZoneNonUniformity	50.55789474
original	glszm	GrayLevelNonUniformity	4.263157895
original	glszm	LargeAreaEmphasis	6.252631579
original	glszm	SmallAreaHighGrayLevelEmphasis	258.2773077
original	glszm	ZonePercentage	0.586419753
original	glszm	LargeAreaLowGrayLevelEmphasis	0.518888288
original	glszm	LargeAreaHighGrayLevelEmphasis	458.8421053
original	glszm	HighGrayLevelZoneEmphasis	283.8421053
original	glszm	SmallAreaEmphasis	0.756591374
original	glszm	LowGrayLevelZoneEmphasis	0.036747106
original	glszm	ZoneEntropy	5.482925282
original	glszm	SmallAreaLowGrayLevelEmphasis	0.016053807
original	ngtdm	Coarseness	0.01831664
original	ngtdm	Complexity	3311.690187
original	ngtdm	Strength	23.06237141
original	ngtdm	Contrast	0.699512127
original	ngtdm	Busyness	0.234749354
wavelet-HHH	gldm	GrayLevelVariance	11.46578266
wavelet-HHH	gldm	HighGrayLevelEmphasis	195.8888889
wavelet-HHH	gldm	DependenceEntropy	5.355217408
wavelet-HHH	gldm	DependenceNonUniformity	39.38271605
wavelet-HHH	gldm	GrayLevelNonUniformity	15.08641975
wavelet-HHH	gldm	SmallDependenceEmphasis	0.382033608
wavelet-HHH	gldm	SmallDependenceHighGrayLevelEmphasis	78.05765089
wavelet-HHH	gldm	DependenceNonUniformityNormalized	0.243103185
wavelet-HHH	gldm	LargeDependenceEmphasis	7.308641975
wavelet-HHH	gldm	LargeDependenceLowGrayLevelEmphasis	0.052296738
wavelet-HHH	gldm	DependenceVariance	1.453436976
wavelet-HHH	gldm	LargeDependenceHighGrayLevelEmphasis	1387.493827
wavelet-HHH	gldm	SmallDependenceLowGrayLevelEmphasis	0.009016838
wavelet-HHH	gldm	LowGrayLevelEmphasis	0.013107661
wavelet-HHH	glcm	JointAverage	13.60623731
wavelet-HHH	glcm	SumAverage	27.21247461
wavelet-HHH	glcm	JointEntropy	6.503403525
wavelet-HHH	glcm	ClusterShade	-28.38598752
wavelet-HHH	glcm	MaximumProbability	0.033768645
wavelet-HHH	glcm	Idmn	0.958243283
wavelet-HHH	glcm	JointEnergy	0.013101299
wavelet-HHH	glcm	Contrast	23.12873098
wavelet-HHH	glcm	DifferenceEntropy	3.264563313
wavelet-HHH	glcm	InverseVariance	0.237871127
wavelet-HHH	glcm	DifferenceVariance	8.685844845
wavelet-HHH	glcm	Idn	0.864735204
wavelet-HHH	glcm	Idm	0.235021903
wavelet-HHH	glcm	Correlation	-0.020616123
wavelet-HHH	glcm	Autocorrelation	184.9086855
wavelet-HHH	glcm	SumEntropy	4.056995639
wavelet-HHH	glcm	SumSquares	11.33996515
wavelet-HHH	glcm	ClusterProminence	1677.655758
wavelet-HHH	glcm	Imc2	0.896858048
wavelet-HHH	glcm	Imc1	-0.225977045

wavelet-HHH	glcm	DifferenceAverage	3.757527565
wavelet-HHH	glcm	Id	0.327851006
wavelet-HHH	glcm	ClusterTendency	22.23112961
wavelet-HHH	firstorder	InterquartileRange	103.037198
wavelet-HHH	firstorder	Skewness	-0.54127584
wavelet-HHH	firstorder	Uniformity	0.093126048
wavelet-HHH	firstorder	Median	8.062463265
wavelet-HHH	firstorder	Energy	1162810.351
wavelet-HHH	firstorder	RobustMeanAbsoluteDeviation	43.29891987
wavelet-HHH	firstorder	MeanAbsoluteDeviation	65.27162819
wavelet-HHH	firstorder	TotalEnergy	31395879.49
wavelet-HHH	firstorder	Maximum	203.4103787
wavelet-HHH	firstorder	RootMeanSquared	84.72214395
wavelet-HHH	firstorder	90Percentile	97.62441397
wavelet-HHH	firstorder	Minimum	-318.833379
wavelet-HHH	firstorder	Entropy	3.665800825
wavelet-HHH	firstorder	Range	522.2437577
wavelet-HHH	firstorder	Variance	7175.791536
wavelet-HHH	firstorder	10Percentile	-113.970134
wavelet-HHH	firstorder	Kurtosis	3.826353942
wavelet-HHH	firstorder	Mean	1.431830832
wavelet-HHH	glrlm	ShortRunLowGrayLevelEmphasis	0.013230051
wavelet-HHH	glrlm	GrayLevelVariance	11.76897204
wavelet-HHH	glrlm	LowGrayLevelRunEmphasis	0.013501772
wavelet-HHH	glrlm	GrayLevelNonUniformityNormalized	0.091766247
wavelet-HHH	glrlm	RunVariance	0.059305576
wavelet-HHH	glrlm	GrayLevelNonUniformity	14.05496072
wavelet-HHH	glrlm	LongRunEmphasis	1.180679437
wavelet-HHH	glrlm	ShortRunHighGrayLevelEmphasis	188.2779044
wavelet-HHH	glrlm	RunLengthNonUniformity	137.3875519
wavelet-HHH	glrlm	ShortRunEmphasis	0.957629408
wavelet-HHH	glrlm	LongRunHighGrayLevelEmphasis	230.4592762
wavelet-HHH	glrlm	RunPercentage	0.945394112
wavelet-HHH	glrlm	LongRunLowGrayLevelEmphasis	0.014648695
wavelet-HHH	glrlm	RunEntropy	3.926714991
wavelet-HHH	glrlm	HighGrayLevelRunEmphasis	196.2871447
wavelet-HHH	glrlm	RunLengthNonUniformityNormalized	0.895702667
wavelet-HHH	glszm	GrayLevelVariance	17.4992284
wavelet-HHH	glszm	ZoneVariance	7.465277778
wavelet-HHH	glszm	GrayLevelNonUniformityNormalized	0.077160494
wavelet-HHH	glszm	SizeZoneNonUniformityNormalized	0.409722222
wavelet-HHH	glszm	SizeZoneNonUniformity	29.5
wavelet-HHH	glszm	GrayLevelNonUniformity	5.555555556
wavelet-HHH	glszm	LargeAreaEmphasis	12.52777778
wavelet-HHH	glszm	SmallAreaHighGrayLevelEmphasis	137.2467073
wavelet-HHH	glszm	ZonePercentage	0.444444444
wavelet-HHH	glszm	LargeAreaLowGrayLevelEmphasis	0.086151302
wavelet-HHH	glszm	LargeAreaHighGrayLevelEmphasis	2479.472222
wavelet-HHH	glszm	HighGrayLevelZoneEmphasis	200.5
wavelet-HHH	glszm	SmallAreaEmphasis	0.662849949
wavelet-HHH	glszm	LowGrayLevelZoneEmphasis	0.021535483
wavelet-HHH	glszm	ZoneEntropy	5.078211683
wavelet-HHH	glszm	SmallAreaLowGrayLevelEmphasis	0.018981454
wavelet-HHH	ngtdm	Coarseness	0.034220666
wavelet-HHH	ngtdm	Complexity	365.6275021

wavelet-HHH	ngtdm	Strength	3.76485935
wavelet-HHH	ngtdm	Contrast	0.207005356
wavelet-HHH	ngtdm	Busyness	0.120703251
wavelet-HLL	gldm	GrayLevelVariance	74.05746075
wavelet-HLL	gldm	HighGrayLevelEmphasis	785.8271605
wavelet-HLL	gldm	DependenceEntropy	5.928636253
wavelet-HLL	gldm	DependenceNonUniformity	58.16049383
wavelet-HLL	gldm	GrayLevelNonUniformity	7.061728395
wavelet-HLL	gldm	SmallDependenceEmphasis	0.587985254
wavelet-HLL	gldm	SmallDependenceHighGrayLevelEmphasis	442.8389438
wavelet-HLL	gldm	DependenceNonUniformityNormalized	0.359015394
wavelet-HLL	gldm	LargeDependenceEmphasis	4.012345679
wavelet-HLL	gldm	LargeDependenceLowGrayLevelEmphasis	0.015059099
wavelet-HLL	gldm	DependenceVariance	0.851851852
wavelet-HLL	gldm	LargeDependenceHighGrayLevelEmphasis	3478.481481
wavelet-HLL	gldm	SmallDependenceLowGrayLevelEmphasis	0.010332279
wavelet-HLL	gldm	LowGrayLevelEmphasis	0.010964784
wavelet-HLL	glcm	JointAverage	26.45233622
wavelet-HLL	glcm	SumAverage	52.90467243
wavelet-HLL	glcm	JointEntropy	7.263699533
wavelet-HLL	glcm	ClusterShade	-774.1813189
wavelet-HLL	glcm	MaximumProbability	0.015867018
wavelet-HLL	glcm	Idmn	0.952228833
wavelet-HLL	glcm	JointEnergy	0.007031553
wavelet-HLL	glcm	Contrast	135.0463512
wavelet-HLL	glcm	DifferenceEntropy	4.26258058
wavelet-HLL	glcm	InverseVariance	0.119694231
wavelet-HLL	glcm	DifferenceVariance	51.62630212
wavelet-HLL	glcm	Idn	0.859685338
wavelet-HLL	glcm	Idm	0.123387
wavelet-HLL	glcm	Correlation	0.115954093
wavelet-HLL	glcm	Autocorrelation	710.475493
wavelet-HLL	glcm	SumEntropy	5.202241341
wavelet-HLL	glcm	SumSquares	78.11654501
wavelet-HLL	glcm	ClusterProminence	113554.7356
wavelet-HLL	glcm	Imc2	0.995452073
wavelet-HLL	glcm	Imc1	-0.490536632
wavelet-HLL	glcm	DifferenceAverage	8.863726075
wavelet-HLL	glcm	Id	0.206874868
wavelet-HLL	glcm	ClusterTendency	177.4198289
wavelet-HLL	firstorder	InterquartileRange	220.7807819
wavelet-HLL	firstorder	Skewness	-0.531312065
wavelet-HLL	firstorder	Uniformity	0.043590916
wavelet-HLL	firstorder	Median	19.09627562
wavelet-HLL	firstorder	Energy	7506170.76
wavelet-HLL	firstorder	RobustMeanAbsoluteDeviation	101.0327754
wavelet-HLL	firstorder	MeanAbsoluteDeviation	160.8864607
wavelet-HLL	firstorder	TotalEnergy	202666610.5
wavelet-HLL	firstorder	Maximum	556.4938152
wavelet-HLL	firstorder	RootMeanSquared	215.254239
wavelet-HLL	firstorder	90Percentile	266.5191624
wavelet-HLL	firstorder	Minimum	-628.6384317
wavelet-HLL	firstorder	Entropy	4.822981919
wavelet-HLL	firstorder	Range	1185.132247
wavelet-HLL	firstorder	Variance	46317.82986

wavelet-HLL	firstorder	10Percentile	-263.2610526
wavelet-HLL	firstorder	Kurtosis	3.77316038
wavelet-HLL	firstorder	Mean	4.069097364
wavelet-HLL	glrlm	ShortRunLowGrayLevelEmphasis	0.011229726
wavelet-HLL	glrlm	GrayLevelVariance	75.14682617
wavelet-HLL	glrlm	LowGrayLevelRunEmphasis	0.011261127
wavelet-HLL	glrlm	GrayLevelNonUniformityNormalized	0.043057401
wavelet-HLL	glrlm	RunVariance	0.033845677
wavelet-HLL	glrlm	GrayLevelNonUniformity	6.76651024
wavelet-HLL	glrlm	LongRunEmphasis	1.097228266
wavelet-HLL	glrlm	ShortRunHighGrayLevelEmphasis	764.1926762
wavelet-HLL	glrlm	RunLengthNonUniformity	148.3829561
wavelet-HLL	glrlm	ShortRunEmphasis	0.977906188
wavelet-HLL	glrlm	LongRunHighGrayLevelEmphasis	867.3922255
wavelet-HLL	glrlm	RunPercentage	0.97008547
wavelet-HLL	glrlm	LongRunLowGrayLevelEmphasis	0.011400863
wavelet-HLL	glrlm	RunEntropy	4.95202976
wavelet-HLL	glrlm	HighGrayLevelRunEmphasis	783.3497796
wavelet-HLL	glrlm	RunLengthNonUniformityNormalized	0.943791432
wavelet-HLL	glszm	GrayLevelVariance	89.83975207
wavelet-HLL	glszm	ZoneVariance	0.885619835
wavelet-HLL	glszm	GrayLevelNonUniformityNormalized	0.036859504
wavelet-HLL	glszm	SizeZoneNonUniformityNormalized	0.558347107
wavelet-HLL	glszm	SizeZoneNonUniformity	61.41818182
wavelet-HLL	glszm	GrayLevelNonUniformity	4.054545455
wavelet-HLL	glszm	LargeAreaEmphasis	3.054545455
wavelet-HLL	glszm	SmallAreaHighGrayLevelEmphasis	577.4621959
wavelet-HLL	glszm	ZonePercentage	0.679012346
wavelet-HLL	glszm	LargeAreaLowGrayLevelEmphasis	0.0182599
wavelet-HLL	glszm	LargeAreaHighGrayLevelEmphasis	2611.436364
wavelet-HLL	glszm	HighGrayLevelZoneEmphasis	757.8272727
wavelet-HLL	glszm	SmallAreaEmphasis	0.774364822
wavelet-HLL	glszm	LowGrayLevelZoneEmphasis	0.015442523
wavelet-HLL	glszm	ZoneEntropy	5.7767825
wavelet-HLL	glszm	SmallAreaLowGrayLevelEmphasis	0.01507054
wavelet-HLL	ngtdm	Coarseness	0.038160739
wavelet-HLL	ngtdm	Complexity	3437.791315
wavelet-HLL	ngtdm	Strength	23.92993294
wavelet-HLL	ngtdm	Contrast	0.494014956
wavelet-HLL	ngtdm	Busyness	0.02312352
wavelet-LHL	gldm	GrayLevelVariance	65.29660113
wavelet-LHL	gldm	HighGrayLevelEmphasis	755.4938272
wavelet-LHL	gldm	DependenceEntropy	5.870390938
wavelet-LHL	gldm	DependenceNonUniformity	61.08641975
wavelet-LHL	gldm	GrayLevelNonUniformity	7.037037037
wavelet-LHL	gldm	SmallDependenceEmphasis	0.569819959
wavelet-LHL	gldm	SmallDependenceHighGrayLevelEmphasis	397.3616358
wavelet-LHL	gldm	DependenceNonUniformityNormalized	0.377076665
wavelet-LHL	gldm	LargeDependenceEmphasis	3.62962963
wavelet-LHL	gldm	LargeDependenceLowGrayLevelEmphasis	0.061559185
wavelet-LHL	gldm	DependenceVariance	0.64228014
wavelet-LHL	gldm	LargeDependenceHighGrayLevelEmphasis	2986.08642
wavelet-LHL	gldm	SmallDependenceLowGrayLevelEmphasis	0.011187208
wavelet-LHL	gldm	LowGrayLevelEmphasis	0.02104642
wavelet-LHL	glcm	JointAverage	26.12446637

wavelet-LHL	glcm	SumAverage	52.24893274
wavelet-LHL	glcm	JointEntropy	7.192731156
wavelet-LHL	glcm	ClusterShade	-886.0807458
wavelet-LHL	glcm	MaximumProbability	0.018041954
wavelet-LHL	glcm	Idmn	0.953422114
wavelet-LHL	glcm	JointEnergy	0.007585574
wavelet-LHL	glcm	Contrast	128.8189496
wavelet-LHL	glcm	DifferenceEntropy	4.153412258
wavelet-LHL	glcm	InverseVariance	0.148680111
wavelet-LHL	glcm	DifferenceVariance	58.84304852
wavelet-LHL	glcm	Idn	0.866874896
wavelet-LHL	glcm	Idm	0.137405714
wavelet-LHL	glcm	Correlation	0.040237998
wavelet-LHL	glcm	Autocorrelation	685.0886398
wavelet-LHL	glcm	SumEntropy	5.005715194
wavelet-LHL	glcm	SumSquares	66.98545283
wavelet-LHL	glcm	ClusterProminence	79067.33004
wavelet-LHL	glcm	Imc2	0.995220463
wavelet-LHL	glcm	Imc1	-0.498257844
wavelet-LHL	glcm	DifferenceAverage	8.148024102
wavelet-LHL	glcm	Id	0.223119881
wavelet-LHL	glcm	ClusterTendency	139.1228617
wavelet-LHL	firstorder	InterquartileRange	226.3670872
wavelet-LHL	firstorder	Skewness	-0.684074578
wavelet-LHL	firstorder	Uniformity	0.0434385
wavelet-LHL	firstorder	Median	19.70612177
wavelet-LHL	firstorder	Energy	6563572.221
wavelet-LHL	firstorder	RobustMeanAbsoluteDeviation	97.82020962
wavelet-LHL	firstorder	MeanAbsoluteDeviation	152.8216739
wavelet-LHL	firstorder	TotalEnergy	177216450
wavelet-LHL	firstorder	Maximum	512.9439167
wavelet-LHL	firstorder	RootMeanSquared	201.2855631
wavelet-LHL	firstorder	90Percentile	230.9925
wavelet-LHL	firstorder	Minimum	-632.8796208
wavelet-LHL	firstorder	Entropy	4.781109647
wavelet-LHL	firstorder	Range	1145.823538
wavelet-LHL	firstorder	Variance	40477.00644
wavelet-LHL	firstorder	10Percentile	-291.9723465
wavelet-LHL	firstorder	Kurtosis	3.884818464
wavelet-LHL	firstorder	Mean	-6.234698622
wavelet-LHL	glrlm	ShortRunLowGrayLevelEmphasis	0.020726241
wavelet-LHL	glrlm	GrayLevelVariance	65.98250385
wavelet-LHL	glrlm	LowGrayLevelRunEmphasis	0.021127226
wavelet-LHL	glrlm	GrayLevelNonUniformityNormalized	0.042904156
wavelet-LHL	glrlm	RunVariance	0.030932482
wavelet-LHL	glrlm	GrayLevelNonUniformity	6.755674503
wavelet-LHL	glrlm	LongRunEmphasis	1.090904835
wavelet-LHL	glrlm	ShortRunHighGrayLevelEmphasis	735.3661453
wavelet-LHL	glrlm	RunLengthNonUniformity	149.1989675
wavelet-LHL	glrlm	ShortRunEmphasis	0.978940083
wavelet-LHL	glrlm	LongRunHighGrayLevelEmphasis	830.1717569
wavelet-LHL	glrlm	RunPercentage	0.971984805
wavelet-LHL	glrlm	LongRunLowGrayLevelEmphasis	0.022737966
wavelet-LHL	glrlm	RunEntropy	4.905246865
wavelet-LHL	glrlm	HighGrayLevelRunEmphasis	752.9313288

wavelet-LHL	glrlm	RunLengthNonUniformityNormalized	0.946709292
wavelet-LHL	glszm	GrayLevelVariance	74.17719008
wavelet-LHL	glszm	ZoneVariance	0.612892562
wavelet-LHL	glszm	GrayLevelNonUniformityNormalized	0.037024793
wavelet-LHL	glszm	SizeZoneNonUniformityNormalized	0.506446281
wavelet-LHL	glszm	SizeZoneNonUniformity	55.70909091
wavelet-LHL	glszm	GrayLevelNonUniformity	4.072727273
wavelet-LHL	glszm	LargeAreaEmphasis	2.781818182
wavelet-LHL	glszm	SmallAreaHighGrayLevelEmphasis	499.1176288
wavelet-LHL	glszm	ZonePercentage	0.679012346
wavelet-LHL	glszm	LargeAreaLowGrayLevelEmphasis	0.050885935
wavelet-LHL	glszm	LargeAreaHighGrayLevelEmphasis	2240.363636
wavelet-LHL	glszm	HighGrayLevelZoneEmphasis	714.7272727
wavelet-LHL	glszm	SmallAreaEmphasis	0.737042929
wavelet-LHL	glszm	LowGrayLevelZoneEmphasis	0.021258204
wavelet-LHL	glszm	ZoneEntropy	5.766371164
wavelet-LHL	glszm	SmallAreaLowGrayLevelEmphasis	0.01406113
wavelet-LHL	ngtdm	Coarseness	0.035244684
wavelet-LHL	ngtdm	Complexity	3127.080933
wavelet-LHL	ngtdm	Strength	14.72003794
wavelet-LHL	ngtdm	Contrast	0.619943879
wavelet-LHL	ngtdm	Busyness	0.031299797
wavelet-LHH	gldm	GrayLevelVariance	29.35409998
wavelet-LHH	gldm	HighGrayLevelEmphasis	180.4012346
wavelet-LHH	gldm	DependenceEntropy	5.383810615
wavelet-LHH	gldm	DependenceNonUniformity	62.0617284
wavelet-LHH	gldm	GrayLevelNonUniformity	9.444444444
wavelet-LHH	gldm	SmallDependenceEmphasis	0.618355624
wavelet-LHH	gldm	SmallDependenceHighGrayLevelEmphasis	114.7709191
wavelet-LHH	gldm	DependenceNonUniformityNormalized	0.383097089
wavelet-LHH	gldm	LargeDependenceEmphasis	3.641975309
wavelet-LHH	gldm	LargeDependenceLowGrayLevelEmphasis	0.051011544
wavelet-LHH	gldm	DependenceVariance	0.739368999
wavelet-LHH	gldm	LargeDependenceHighGrayLevelEmphasis	625.7962963
wavelet-LHH	gldm	SmallDependenceLowGrayLevelEmphasis	0.024234458
wavelet-LHH	gldm	LowGrayLevelEmphasis	0.027947121
wavelet-LHH	glcm	JointAverage	12.35031742
wavelet-LHH	glcm	SumAverage	24.70063484
wavelet-LHH	glcm	JointEntropy	7.037994477
wavelet-LHH	glcm	ClusterShade	111.9742502
wavelet-LHH	glcm	MaximumProbability	0.023507739
wavelet-LHH	glcm	Idmn	0.938302004
wavelet-LHH	glcm	JointEnergy	0.008647171
wavelet-LHH	glcm	Contrast	62.84392165
wavelet-LHH	glcm	DifferenceEntropy	3.84376971
wavelet-LHH	glcm	InverseVariance	0.169630826
wavelet-LHH	glcm	DifferenceVariance	22.1534567
wavelet-LHH	glcm	Idn	0.837210034
wavelet-LHH	glcm	Idm	0.149032998
wavelet-LHH	glcm	Correlation	-0.025022009
wavelet-LHH	glcm	Autocorrelation	151.6965643
wavelet-LHH	glcm	SumEntropy	4.626168053
wavelet-LHH	glcm	SumSquares	30.58508533
wavelet-LHH	glcm	ClusterProminence	10696.7127
wavelet-LHH	glcm	Imc2	0.97912843

wavelet-LHH	glcm	Imc1	-0.378238953
wavelet-LHH	glcm	DifferenceAverage	6.244518269
wavelet-LHH	glcm	Id	0.239096796
wavelet-LHH	glcm	ClusterTendency	59.49641966
wavelet-LHH	firstorder	InterquartileRange	193.8170849
wavelet-LHH	firstorder	Skewness	0.323714127
wavelet-LHH	firstorder	Uniformity	0.05829904
wavelet-LHH	firstorder	Median	0.813262469
wavelet-LHH	firstorder	Energy	3003011.443
wavelet-LHH	firstorder	RobustMeanAbsoluteDeviation	74.41667473
wavelet-LHH	firstorder	MeanAbsoluteDeviation	107.608113
wavelet-LHH	firstorder	TotalEnergy	81081308.96
wavelet-LHH	firstorder	Maximum	421.9888827
wavelet-LHH	firstorder	RootMeanSquared	136.1510473
wavelet-LHH	firstorder	90Percentile	160.5650655
wavelet-LHH	firstorder	Minimum	-297.9466074
wavelet-LHH	firstorder	Entropy	4.346608562
wavelet-LHH	firstorder	Range	719.9354901
wavelet-LHH	firstorder	Variance	18504.06545
wavelet-LHH	firstorder	10Percentile	-174.8106268
wavelet-LHH	firstorder	Kurtosis	3.049332007
wavelet-LHH	firstorder	Mean	-5.74823622
wavelet-LHH	glrlm	ShortRunLowGrayLevelEmphasis	0.028291708
wavelet-LHH	glrlm	GrayLevelVariance	29.82592729
wavelet-LHH	glrlm	LowGrayLevelRunEmphasis	0.028475483
wavelet-LHH	glrlm	GrayLevelNonUniformityNormalized	0.057577819
wavelet-LHH	glrlm	RunVariance	0.030003214
wavelet-LHH	glrlm	GrayLevelNonUniformity	9.07573737
wavelet-LHH	glrlm	LongRunEmphasis	1.087043244
wavelet-LHH	glrlm	ShortRunHighGrayLevelEmphasis	177.2546813
wavelet-LHH	glrlm	RunLengthNonUniformity	149.5636067
wavelet-LHH	glrlm	ShortRunEmphasis	0.979893768
wavelet-LHH	glrlm	LongRunHighGrayLevelEmphasis	195.3932232
wavelet-LHH	glrlm	RunPercentage	0.972934473
wavelet-LHH	glrlm	LongRunLowGrayLevelEmphasis	0.029263655
wavelet-LHH	glrlm	RunEntropy	4.467923531
wavelet-LHH	glrlm	HighGrayLevelRunEmphasis	180.6967735
wavelet-LHH	glrlm	RunLengthNonUniformityNormalized	0.948578873
wavelet-LHH	glszm	GrayLevelVariance	36.54930606
wavelet-LHH	glszm	ZoneVariance	1.005113221
wavelet-LHH	glszm	GrayLevelNonUniformityNormalized	0.051538025
wavelet-LHH	glszm	SizeZoneNonUniformityNormalized	0.617401185
wavelet-LHH	glszm	SizeZoneNonUniformity	68.53153153
wavelet-LHH	glszm	GrayLevelNonUniformity	5.720720721
wavelet-LHH	glszm	LargeAreaEmphasis	3.135135135
wavelet-LHH	glszm	SmallAreaHighGrayLevelEmphasis	151.1585611
wavelet-LHH	glszm	ZonePercentage	0.685185185
wavelet-LHH	glszm	LargeAreaLowGrayLevelEmphasis	0.054700366
wavelet-LHH	glszm	LargeAreaHighGrayLevelEmphasis	556.963964
wavelet-LHH	glszm	HighGrayLevelZoneEmphasis	183.8108108
wavelet-LHH	glszm	SmallAreaEmphasis	0.809827327
wavelet-LHH	glszm	LowGrayLevelZoneEmphasis	0.036528467
wavelet-LHH	glszm	ZoneEntropy	5.078571358
wavelet-LHH	glszm	SmallAreaLowGrayLevelEmphasis	0.034470504
wavelet-LHH	ngtdm	Coarseness	0.032177829

wavelet-LHH	ngtdm	Complexity	1255.25932
wavelet-LHH	ngtdm	Strength	7.111548171
wavelet-LHH	ngtdm	Contrast	0.381418842
wavelet-LHH	ngtdm	Busyness	0.105377647
wavelet-LLH	gldm	GrayLevelVariance	73.09327846
wavelet-LLH	gldm	HighGrayLevelEmphasis	728.0740741
wavelet-LLH	gldm	DependenceEntropy	5.749701932
wavelet-LLH	gldm	DependenceNonUniformity	64.20987654
wavelet-LLH	gldm	GrayLevelNonUniformity	6.703703704
wavelet-LLH	gldm	SmallDependenceEmphasis	0.613340192
wavelet-LLH	gldm	SmallDependenceHighGrayLevelEmphasis	445.3160151
wavelet-LLH	gldm	DependenceNonUniformityNormalized	0.396357263
wavelet-LLH	gldm	LargeDependenceEmphasis	3.345679012
wavelet-LLH	gldm	LargeDependenceLowGrayLevelEmphasis	0.015474496
wavelet-LLH	gldm	DependenceVariance	0.608901082
wavelet-LLH	gldm	LargeDependenceHighGrayLevelEmphasis	2515.419753
wavelet-LLH	gldm	SmallDependenceLowGrayLevelEmphasis	0.009910796
wavelet-LLH	gldm	LowGrayLevelEmphasis	0.010789089
wavelet-LLH	glcm	JointAverage	24.69103749
wavelet-LLH	glcm	SumAverage	49.38207498
wavelet-LLH	glcm	JointEntropy	7.303050384
wavelet-LLH	glcm	ClusterShade	-1283.115644
wavelet-LLH	glcm	MaximumProbability	0.013118808
wavelet-LLH	glcm	Idmn	0.934382679
wavelet-LLH	glcm	JointEnergy	0.006813623
wavelet-LLH	glcm	Contrast	149.2572966
wavelet-LLH	glcm	DifferenceEntropy	4.318102609
wavelet-LLH	glcm	InverseVariance	0.11210614
wavelet-LLH	glcm	DifferenceVariance	52.61616685
wavelet-LLH	glcm	Idn	0.834850835
wavelet-LLH	glcm	Idm	0.111541991
wavelet-LLH	glcm	Correlation	0.112842848
wavelet-LLH	glcm	Autocorrelation	619.2525762
wavelet-LLH	glcm	SumEntropy	5.204731372
wavelet-LLH	glcm	SumSquares	84.17745379
wavelet-LLH	glcm	ClusterProminence	106833.1306
wavelet-LLH	glcm	Imc2	0.995596166
wavelet-LLH	glcm	Imc1	-0.504734489
wavelet-LLH	glcm	DifferenceAverage	9.505538792
wavelet-LLH	glcm	Id	0.192695946
wavelet-LLH	glcm	ClusterTendency	187.4525185
wavelet-LLH	firstorder	InterquartileRange	303.873957
wavelet-LLH	firstorder	Skewness	-0.651027682
wavelet-LLH	firstorder	Uniformity	0.041380887
wavelet-LLH	firstorder	Median	23.27475838
wavelet-LLH	firstorder	Energy	7430475.357
wavelet-LLH	firstorder	RobustMeanAbsoluteDeviation	122.4440551
wavelet-LLH	firstorder	MeanAbsoluteDeviation	174.3297778
wavelet-LLH	firstorder	TotalEnergy	200622834.6
wavelet-LLH	firstorder	Maximum	402.1019272
wavelet-LLH	firstorder	RootMeanSquared	214.1661314
wavelet-LLH	firstorder	90Percentile	224.3808897
wavelet-LLH	firstorder	Minimum	-627.8581262
wavelet-LLH	firstorder	Entropy	4.806400521
wavelet-LLH	firstorder	Range	1029.960053

wavelet-LLH	firstorder	Variance	45338.80226
wavelet-LLH	firstorder	10Percentile	-325.071932
wavelet-LLH	firstorder	Kurtosis	2.96892481
wavelet-LLH	firstorder	Mean	-22.98542087
wavelet-LLH	glrlm	ShortRunLowGrayLevelEmphasis	0.010973731
wavelet-LLH	glrlm	GrayLevelVariance	73.54040108
wavelet-LLH	glrlm	LowGrayLevelRunEmphasis	0.011014817
wavelet-LLH	glrlm	GrayLevelNonUniformityNormalized	0.041260108
wavelet-LLH	glrlm	RunVariance	0.025131507
wavelet-LLH	glrlm	GrayLevelNonUniformity	6.515788164
wavelet-LLH	glrlm	LongRunEmphasis	1.077998213
wavelet-LLH	glrlm	ShortRunHighGrayLevelEmphasis	712.8159383
wavelet-LLH	glrlm	RunLengthNonUniformity	150.0391917
wavelet-LLH	glrlm	ShortRunEmphasis	0.980500447
wavelet-LLH	glrlm	LongRunHighGrayLevelEmphasis	786.0751414
wavelet-LLH	glrlm	RunPercentage	0.974833808
wavelet-LLH	glrlm	LongRunLowGrayLevelEmphasis	0.011179163
wavelet-LLH	glrlm	RunEntropy	4.91036803
wavelet-LLH	glrlm	HighGrayLevelRunEmphasis	727.4677789
wavelet-LLH	glrlm	RunLengthNonUniformityNormalized	0.949736986
wavelet-LLH	glszm	GrayLevelVariance	79.57402278
wavelet-LLH	glszm	ZoneVariance	0.734995383
wavelet-LLH	glszm	GrayLevelNonUniformityNormalized	0.040320098
wavelet-LLH	glszm	SizeZoneNonUniformityNormalized	0.567251462
wavelet-LLH	glszm	SizeZoneNonUniformity	64.66666667
wavelet-LLH	glszm	GrayLevelNonUniformity	4.596491228
wavelet-LLH	glszm	LargeAreaEmphasis	2.754385965
wavelet-LLH	glszm	SmallAreaHighGrayLevelEmphasis	567.9029094
wavelet-LLH	glszm	ZonePercentage	0.703703704
wavelet-LLH	glszm	LargeAreaLowGrayLevelEmphasis	0.018047663
wavelet-LLH	glszm	LargeAreaHighGrayLevelEmphasis	2004.912281
wavelet-LLH	glszm	HighGrayLevelZoneEmphasis	724.9122807
wavelet-LLH	glszm	SmallAreaEmphasis	0.781661793
wavelet-LLH	glszm	LowGrayLevelZoneEmphasis	0.014408626
wavelet-LLH	glszm	ZoneEntropy	5.563869922
wavelet-LLH	glszm	SmallAreaLowGrayLevelEmphasis	0.013874911
wavelet-LLH	ngtdm	Coarseness	0.027745491
wavelet-LLH	ngtdm	Complexity	3566.237434
wavelet-LLH	ngtdm	Strength	15.29560657
wavelet-LLH	ngtdm	Contrast	0.739130559
wavelet-LLH	ngtdm	Busyness	0.039402274
wavelet-LLL	gldm	GrayLevelVariance	349.5655007
wavelet-LLL	gldm	HighGrayLevelEmphasis	937.1790123
wavelet-LLL	gldm	DependenceEntropy	6.060636735
wavelet-LLL	gldm	DependenceNonUniformity	78.55555556
wavelet-LLL	gldm	GrayLevelNonUniformity	4.098765432
wavelet-LLL	gldm	SmallDependenceEmphasis	0.715569273
wavelet-LLL	gldm	SmallDependenceHighGrayLevelEmphasis	736.1829218
wavelet-LLL	gldm	DependenceNonUniformityNormalized	0.484910837
wavelet-LLL	gldm	LargeDependenceEmphasis	3.061728395
wavelet-LLL	gldm	LargeDependenceLowGrayLevelEmphasis	0.321204075
wavelet-LLL	gldm	DependenceVariance	0.755829904
wavelet-LLL	gldm	LargeDependenceHighGrayLevelEmphasis	1862.993827
wavelet-LLL	gldm	SmallDependenceLowGrayLevelEmphasis	0.013321778
wavelet-LLL	gldm	LowGrayLevelEmphasis	0.038009713

wavelet-LLL	glcm	JointAverage	27.47022648
wavelet-LLL	glcm	SumAverage	54.94045297
wavelet-LLL	glcm	JointEntropy	7.442922984
wavelet-LLL	glcm	ClusterShade	34548.29769
wavelet-LLL	glcm	MaximumProbability	0.01398182
wavelet-LLL	glcm	Idmn	0.961659536
wavelet-LLL	glcm	JointEnergy	0.006087931
wavelet-LLL	glcm	Contrast	350.748915
wavelet-LLL	glcm	DifferenceEntropy	4.755200356
wavelet-LLL	glcm	InverseVariance	0.090422646
wavelet-LLL	glcm	DifferenceVariance	143.6281623
wavelet-LLL	glcm	Idn	0.87414541
wavelet-LLL	glcm	Idm	0.089244293
wavelet-LLL	glcm	Correlation	0.564261163
wavelet-LLL	glcm	Autocorrelation	980.9742278
wavelet-LLL	glcm	SumEntropy	5.887151375
wavelet-LLL	glcm	SumSquares	400.0276598
wavelet-LLL	glcm	ClusterProminence	4575015.117
wavelet-LLL	glcm	Imc2	0.999579407
wavelet-LLL	glcm	Imc1	-0.652550374
wavelet-LLL	glcm	DifferenceAverage	14.10978407
wavelet-LLL	glcm	Id	0.159177154
wavelet-LLL	glcm	ClusterTendency	1249.361724
wavelet-LLL	firstorder	InterquartileRange	606.1725372
wavelet-LLL	firstorder	Skewness	1.071854935
wavelet-LLL	firstorder	Uniformity	0.025301021
wavelet-LLL	firstorder	Median	-2028.505201
wavelet-LLL	firstorder	Energy	639791315.8
wavelet-LLL	firstorder	RobustMeanAbsoluteDeviation	256.7276312
wavelet-LLL	firstorder	MeanAbsoluteDeviation	370.5564455
wavelet-LLL	firstorder	TotalEnergy	17274365526
wavelet-LLL	firstorder	Maximum	-308.0614507
wavelet-LLL	firstorder	RootMeanSquared	1987.291904
wavelet-LLL	firstorder	90Percentile	-1238.843663
wavelet-LLL	firstorder	Minimum	-2524.700556
wavelet-LLL	firstorder	Entropy	5.531067075
wavelet-LLL	firstorder	Range	2216.639105
wavelet-LLL	firstorder	Variance	218244.3504
wavelet-LLL	firstorder	10Percentile	-2424.521876
wavelet-LLL	firstorder	Kurtosis	3.766637332
wavelet-LLL	firstorder	Mean	-1931.601605
wavelet-LLL	glrlm	ShortRunLowGrayLevelEmphasis	0.034698839
wavelet-LLL	glrlm	GrayLevelVariance	349.4545877
wavelet-LLL	glrlm	LowGrayLevelRunEmphasis	0.036450602
wavelet-LLL	glrlm	GrayLevelNonUniformityNormalized	0.025049743
wavelet-LLL	glrlm	RunVariance	0.019929544
wavelet-LLL	glrlm	GrayLevelNonUniformity	3.977261105
wavelet-LLL	glrlm	LongRunEmphasis	1.061292808
wavelet-LLL	glrlm	ShortRunHighGrayLevelEmphasis	936.3906349
wavelet-LLL	glrlm	RunLengthNonUniformity	152.4657771
wavelet-LLL	glrlm	ShortRunEmphasis	0.984676798
wavelet-LLL	glrlm	LongRunHighGrayLevelEmphasis	979.1102534
wavelet-LLL	glrlm	RunPercentage	0.98005698
wavelet-LLL	glrlm	LongRunLowGrayLevelEmphasis	0.043457657
wavelet-LLL	glrlm	RunEntropy	5.600278959

wavelet-LLL	glrlm	HighGrayLevelRunEmphasis	944.9345586
wavelet-LLL	glrlm	RunLengthNonUniformityNormalized	0.960140911
wavelet-LLL	glszm	GrayLevelVariance	350.5384091
wavelet-LLL	glszm	ZoneVariance	0.451608903
wavelet-LLL	glszm	GrayLevelNonUniformityNormalized	0.023622047
wavelet-LLL	glszm	SizeZoneNonUniformityNormalized	0.685907372
wavelet-LLL	glszm	SizeZoneNonUniformity	87.11023622
wavelet-LLL	glszm	GrayLevelNonUniformity	3
wavelet-LLL	glszm	LargeAreaEmphasis	2.078740157
wavelet-LLL	glszm	SmallAreaHighGrayLevelEmphasis	895.1240179
wavelet-LLL	glszm	ZonePercentage	0.783950617
wavelet-LLL	glszm	LargeAreaLowGrayLevelEmphasis	0.140687636
wavelet-LLL	glszm	LargeAreaHighGrayLevelEmphasis	1610.433071
wavelet-LLL	glszm	HighGrayLevelZoneEmphasis	1014.716535
wavelet-LLL	glszm	SmallAreaEmphasis	0.853388014
wavelet-LLL	glszm	LowGrayLevelZoneEmphasis	0.022552709
wavelet-LLL	glszm	ZoneEntropy	5.936535797
wavelet-LLL	glszm	SmallAreaLowGrayLevelEmphasis	0.012176558
wavelet-LLL	ngtdm	Coarseness	0.034547532
wavelet-LLL	ngtdm	Complexity	13063.78706
wavelet-LLL	ngtdm	Strength	81.70164571
wavelet-LLL	ngtdm	Contrast	1.690117476
wavelet-LLL	ngtdm	Busyness	0.030429538
wavelet-HHL	gldm	GrayLevelVariance	18.21189605
wavelet-HHL	gldm	HighGrayLevelEmphasis	170.4753086
wavelet-HHL	gldm	DependenceEntropy	5.447672383
wavelet-HHL	gldm	DependenceNonUniformity	45.49382716
wavelet-HHL	gldm	GrayLevelNonUniformity	12.67901235
wavelet-HHL	gldm	SmallDependenceEmphasis	0.462170782
wavelet-HHL	gldm	SmallDependenceHighGrayLevelEmphasis	90.73767147
wavelet-HHL	gldm	DependenceNonUniformityNormalized	0.280826094
wavelet-HHL	gldm	LargeDependenceEmphasis	5.555555556
wavelet-HHL	gldm	LargeDependenceLowGrayLevelEmphasis	0.052305271
wavelet-HHL	gldm	DependenceVariance	1.098765432
wavelet-HHL	gldm	LargeDependenceHighGrayLevelEmphasis	844.2283951
wavelet-HHL	gldm	SmallDependenceLowGrayLevelEmphasis	0.011478887
wavelet-HHL	gldm	LowGrayLevelEmphasis	0.016120675
wavelet-HHL	glcm	JointAverage	12.46440222
wavelet-HHL	glcm	SumAverage	24.92880444
wavelet-HHL	glcm	JointEntropy	6.800760902
wavelet-HHL	glcm	ClusterShade	59.46663862
wavelet-HHL	glcm	MaximumProbability	0.031048032
wavelet-HHL	glcm	Idmn	0.94581973
wavelet-HHL	glcm	JointEnergy	0.010726198
wavelet-HHL	glcm	Contrast	40.61329262
wavelet-HHL	glcm	DifferenceEntropy	3.583625815
wavelet-HHL	glcm	InverseVariance	0.210694858
wavelet-HHL	glcm	DifferenceVariance	15.96090731
wavelet-HHL	glcm	Idn	0.850080024
wavelet-HHL	glcm	Idm	0.195165768
[truncated: 391,131 more chars]
