# Supplementary material for: Radiomic feature stability across 4D respiratory phases and its impact on lung tumor prognosis prediction
Source: PLoS One. 2019 May 7;14(5):e0216480. doi: 10.1371/journal.pone.0216480 (PMC6504105; doi:10.1371/journal.pone.0216480)
Supplement: S2 File — (PDF) [file pone.0216480.s002.pdf]

Supplementary File 2: List of Radiomics features used with cutoffs of P-values, Q-values, and COV

1. Criterion: stable threshold is 5; Pval is 0.005; Qval is 0.04
Stable Features:
wavelet_HHL_glcm_Id
wavelet_HLL_glrlm_RunLengthNonUniformityNormalized
wavelet_HLH_gldm_DependenceNonUniformityNormalized
Full Features:
wavelet_HHL_glcm_Id
wavelet_LLL_glszm_LargeAreaHighGrayLevelEmphasis
original_glszm_LargeAreaHighGrayLevelEmphasis
wavelet_LLL_glszm_LargeAreaEmphasis
wavelet_HLL_glrlm_RunLengthNonUniformityNormalized
wavelet_HLL_glszm_LargeAreaEmphasis
wavelet_LHL_gldm_GrayLevelNonUniformity
wavelet_HLH_gldm_DependenceNonUniformityNormalized
2. Criterion: stable threshold is 10; Pval is 0.005; Qval is 0.04
Stable Features:
wavelet_HHL_glcm_Id
wavelet_HLL_glrlm_RunLengthNonUniformityNormalized
wavelet_LHL_gldm_GrayLevelNonUniformity
wavelet_HLH_gldm_DependenceNonUniformityNormalized
Full Features:
wavelet_HHL_glcm_Id
wavelet_LLL_glszm_LargeAreaHighGrayLevelEmphasis
original_glszm_LargeAreaHighGrayLevelEmphasis
wavelet_LLL_glszm_LargeAreaEmphasis
wavelet_HLL_glrlm_RunLengthNonUniformityNormalized
wavelet_HLL_glszm_LargeAreaEmphasis
wavelet_LHL_gldm_GrayLevelNonUniformity
wavelet_HLH_gldm_DependenceNonUniformityNormalized
3. Criterion: stable threshold is 15; Pval is 0.005; Qval is 0.04
Stable Features:
wavelet_HHL_glcm_Id
wavelet_HLL_glrlm_RunLengthNonUniformityNormalized
wavelet_LHL_gldm_GrayLevelNonUniformity
wavelet_HLH_gldm_DependenceNonUniformityNormalized
Full Features:
wavelet_HHL_glcm_Id
wavelet_LLL_glszm_LargeAreaHighGrayLevelEmphasis
original_glszm_LargeAreaHighGrayLevelEmphasis
wavelet_LLL_glszm_LargeAreaEmphasis
wavelet_HLL_glrlm_RunLengthNonUniformityNormalized
wavelet_HLL_glszm_LargeAreaEmphasis
wavelet_LHL_gldm_GrayLevelNonUniformity
wavelet_HLH_gldm_DependenceNonUniformityNormalized
4. Criterion: stable threshold is 20; Pval is 0.005; Qval is 0.04
Stable Features:

wavelet_HHL_glcm_Id
wavelet_LLL_glszm_LargeAreaEmphasis
wavelet_HLL_glrlm_RunLengthNonUniformityNormalized
wavelet_HLL_glszm_LargeAreaEmphasis
wavelet_LHL_gldm_GrayLevelNonUniformity
wavelet_HLH_gldm_DependenceNonUniformityNormalized
Full Features:
wavelet_HHL_glcm_Id
wavelet_LLL_glszm_LargeAreaHighGrayLevelEmphasis
original_glszm_LargeAreaHighGrayLevelEmphasis
wavelet_LLL_glszm_LargeAreaEmphasis
wavelet_HLL_glrlm_RunLengthNonUniformityNormalized
wavelet_HLL_glszm_LargeAreaEmphasis
wavelet_LHL_gldm_GrayLevelNonUniformity
wavelet_HLH_gldm_DependenceNonUniformityNormalized
5. Criterion: stable threshold is 25; Pval is 0.005; Qval is 0.04
Stable Features:
wavelet_HHL_glcm_Id
wavelet_LLL_glszm_LargeAreaHighGrayLevelEmphasis
original_glszm_LargeAreaHighGrayLevelEmphasis
wavelet_LLL_glszm_LargeAreaEmphasis
wavelet_HLL_glrlm_RunLengthNonUniformityNormalized
wavelet_HLL_glszm_LargeAreaEmphasis
wavelet_LHL_gldm_GrayLevelNonUniformity
wavelet_HLH_gldm_DependenceNonUniformityNormalized
Full Features:
wavelet_HHL_glcm_Id
wavelet_LLL_glszm_LargeAreaHighGrayLevelEmphasis
original_glszm_LargeAreaHighGrayLevelEmphasis
wavelet_LLL_glszm_LargeAreaEmphasis
wavelet_HLL_glrlm_RunLengthNonUniformityNormalized
wavelet_HLL_glszm_LargeAreaEmphasis
wavelet_LHL_gldm_GrayLevelNonUniformity
wavelet_HLH_gldm_DependenceNonUniformityNormalized
6. Criterion: stable threshold is 5; Pval is 0.007; Qval is 0.045
Stable Features:
wavelet_HHL_glcm_Id
wavelet_HLL_gldm_DependenceNonUniformityNormalized
Full Features:
wavelet_HHL_glcm_Id
wavelet_LLL_glszm_LargeAreaHighGrayLevelEmphasis
original_glszm_LargeAreaHighGrayLevelEmphasis
wavelet_HLL_gldm_DependenceNonUniformityNormalized
wavelet_LLL_glszm_LargeAreaEmphasis
wavelet_HLL_glszm_LargeAreaEmphasis
wavelet_LHL_gldm_GrayLevelNonUniformity
7. Criterion: stable threshold is 10; Pval is 0.007; Qval is 0.045
Stable Features:
wavelet_HHL_glcm_Id
wavelet_HLL_gldm_DependenceNonUniformityNormalized
wavelet_LHL_gldm_GrayLevelNonUniformity

Full Features:
wavelet_HHL_glcm_Id
wavelet_LLL_glszm_LargeAreaHighGrayLevelEmphasis
original_glszm_LargeAreaHighGrayLevelEmphasis
wavelet_HLL_gldm_DependenceNonUniformityNormalized
wavelet_LLL_glszm_LargeAreaEmphasis
wavelet_HLL_glszm_LargeAreaEmphasis
wavelet_LHL_gldm_GrayLevelNonUniformity
8. Criterion: stable threshold is 15; Pval is 0.007; Qval is 0.045
Stable Features:
wavelet_HHL_glcm_Id
wavelet_HLL_gldm_DependenceNonUniformityNormalized
wavelet_LHL_gldm_GrayLevelNonUniformity
Full Features:
wavelet_HHL_glcm_Id
wavelet_LLL_glszm_LargeAreaHighGrayLevelEmphasis
original_glszm_LargeAreaHighGrayLevelEmphasis
wavelet_HLL_gldm_DependenceNonUniformityNormalized
wavelet_LLL_glszm_LargeAreaEmphasis
wavelet_HLL_glszm_LargeAreaEmphasis
wavelet_LHL_gldm_GrayLevelNonUniformity
9. Criterion: stable threshold is 20; Pval is 0.007; Qval is 0.045
Stable Features:
wavelet_HHL_glcm_Id
wavelet_HLL_gldm_DependenceNonUniformityNormalized
wavelet_LLL_glszm_LargeAreaEmphasis
wavelet_HLL_glszm_LargeAreaEmphasis
wavelet_LHL_gldm_GrayLevelNonUniformity
Full Features:
wavelet_HHL_glcm_Id
wavelet_LLL_glszm_LargeAreaHighGrayLevelEmphasis
original_glszm_LargeAreaHighGrayLevelEmphasis
wavelet_HLL_gldm_DependenceNonUniformityNormalized
wavelet_LLL_glszm_LargeAreaEmphasis
wavelet_HLL_glszm_LargeAreaEmphasis
wavelet_LHL_gldm_GrayLevelNonUniformity
10. Criterion: stable threshold is 25; Pval is 0.007; Qval is 0.045
Stable Features:
wavelet_HHL_glcm_Id
wavelet_LLL_glszm_LargeAreaHighGrayLevelEmphasis
original_glszm_LargeAreaHighGrayLevelEmphasis
wavelet_HLL_gldm_DependenceNonUniformityNormalized
wavelet_LLL_glszm_LargeAreaEmphasis
wavelet_HLL_glszm_LargeAreaEmphasis
wavelet_LHL_gldm_GrayLevelNonUniformity
Full Features:
wavelet_HHL_glcm_Id
wavelet_LLL_glszm_LargeAreaHighGrayLevelEmphasis
original_glszm_LargeAreaHighGrayLevelEmphasis
wavelet_HLL_gldm_DependenceNonUniformityNormalized
wavelet_LLL_glszm_LargeAreaEmphasis

wavelet_HLL_glszm_LargeAreaEmphasis
wavelet_LHL_gldm_GrayLevelNonUniformity

11. Criterion: stable threshold is 5; Pval is 0.01; Qval is 0.05

Stable Features:

wavelet_HHL_glcm_Id
wavelet_HHH_glcm_DifferenceAverage
wavelet_HLL_gldm_DependenceNonUniformityNormalized

Full Features:

wavelet_HHL_glcm_Id
wavelet_HHH_glcm_DifferenceAverage
wavelet_LLL_glszm_LargeAreaHighGrayLevelEmphasis
original_glszm_LargeAreaHighGrayLevelEmphasis
wavelet_HLL_gldm_DependenceNonUniformityNormalized
wavelet_LLL_glszm_LargeAreaEmphasis
wavelet_HLL_glszm_LargeAreaEmphasis
wavelet_HLH_gldm_SmallDependenceEmphasis
wavelet_LHL_gldm_GrayLevelNonUniformity

12. Criterion: stable threshold is 10; Pval is 0.01; Qval is 0.05

Stable Features:

wavelet_HHL_glcm_Id
wavelet_HHH_glcm_DifferenceAverage
wavelet_HLL_gldm_DependenceNonUniformityNormalized
wavelet_HLH_gldm_SmallDependenceEmphasis
wavelet_LHL_gldm_GrayLevelNonUniformity

Full Features:

wavelet_HHL_glcm_Id
wavelet_HHH_glcm_DifferenceAverage
wavelet_LLL_glszm_LargeAreaHighGrayLevelEmphasis
original_glszm_LargeAreaHighGrayLevelEmphasis
wavelet_HLL_gldm_DependenceNonUniformityNormalized
wavelet_LLL_glszm_LargeAreaEmphasis
wavelet_HLL_glszm_LargeAreaEmphasis
wavelet_HLH_gldm_SmallDependenceEmphasis
wavelet_LHL_gldm_GrayLevelNonUniformity

13. Criterion: stable threshold is 15; Pval is 0.01; Qval is 0.05

Stable Features:

wavelet_HHL_glcm_Id
wavelet_HHH_glcm_DifferenceAverage
wavelet_HLL_gldm_DependenceNonUniformityNormalized
wavelet_HLH_gldm_SmallDependenceEmphasis
wavelet_LHL_gldm_GrayLevelNonUniformity

Full Features:

wavelet_HHL_glcm_Id
wavelet_HHH_glcm_DifferenceAverage
wavelet_LLL_glszm_LargeAreaHighGrayLevelEmphasis
original_glszm_LargeAreaHighGrayLevelEmphasis
wavelet_HLL_gldm_DependenceNonUniformityNormalized
wavelet_LLL_glszm_LargeAreaEmphasis
wavelet_HLL_glszm_LargeAreaEmphasis
wavelet_HLH_gldm_SmallDependenceEmphasis
wavelet_LHL_gldm_GrayLevelNonUniformity

14. Criterion: stable threshold is 20; Pval is 0.01; Qval is 0.05
Stable Features:
wavelet_HHL_glcm_Id
wavelet_HHH_glcm_DifferenceAverage
wavelet_HLL_gldm_DependenceNonUniformityNormalized
wavelet_LLL_glszm_LargeAreaEmphasis
wavelet_HLL_glszm_LargeAreaEmphasis
wavelet_HLH_gldm_SmallDependenceEmphasis
wavelet_LHL_gldm_GrayLevelNonUniformity
Full Features:
wavelet_HHL_glcm_Id
wavelet_HHH_glcm_DifferenceAverage
wavelet_LLL_glszm_LargeAreaHighGrayLevelEmphasis
original_glszm_LargeAreaHighGrayLevelEmphasis
wavelet_HLL_gldm_DependenceNonUniformityNormalized
wavelet_LLL_glszm_LargeAreaEmphasis
wavelet_HLL_glszm_LargeAreaEmphasis
wavelet_HLH_gldm_SmallDependenceEmphasis
wavelet_LHL_gldm_GrayLevelNonUniformity
15. Criterion: stable threshold is 25; Pval is 0.01; Qval is 0.05
Stable Features:
wavelet_HHL_glcm_Id
wavelet_HHH_glcm_DifferenceAverage
wavelet_LLL_glszm_LargeAreaHighGrayLevelEmphasis
original_glszm_LargeAreaHighGrayLevelEmphasis
wavelet_HLL_gldm_DependenceNonUniformityNormalized
wavelet_LLL_glszm_LargeAreaEmphasis
wavelet_HLL_glszm_LargeAreaEmphasis
wavelet_HLH_gldm_SmallDependenceEmphasis
wavelet_LHL_gldm_GrayLevelNonUniformity
Full Features:
wavelet_HHL_glcm_Id
wavelet_HHH_glcm_DifferenceAverage
wavelet_LLL_glszm_LargeAreaHighGrayLevelEmphasis
original_glszm_LargeAreaHighGrayLevelEmphasis
wavelet_HLL_gldm_DependenceNonUniformityNormalized
wavelet_LLL_glszm_LargeAreaEmphasis
wavelet_HLL_glszm_LargeAreaEmphasis
wavelet_HLH_gldm_SmallDependenceEmphasis
wavelet_LHL_gldm_GrayLevelNonUniformity
16. Criterion: stable threshold is 5; Pval is 0.03; Qval is 0.07
Stable Features:
wavelet_HHL_glcm_Id
wavelet_HHH_glcm_DifferenceAverage
wavelet_HLL_glcm_DifferenceEntropy
original_glrlm_LongRunEmphasis
wavelet_LLH_firstorder_Entropy
wavelet_LLH_glcm_Idmn
wavelet_LLH_glszm_SmallAreaEmphasis
original_glcm_Imc2

wavelet_HLH_gldm_DependenceNonUniformityNormalized
original_firstorder_10Percentile
Full Features:
wavelet_HHL_glcm_Id
wavelet_HHH_glrlm_GrayLevelVariance
wavelet_LLH_glrlm_RunVariance
wavelet_HLL_glcm_DifferenceEntropy
wavelet_LLL_glszm_LargeAreaHighGrayLevelEmphasis
original_glszm_LargeAreaHighGrayLevelEmphasis
wavelet_LLH_firstorder_Entropy
wavelet_LLH_glcm_Idmn
wavelet_LLL_glszm_LargeAreaEmphasis
wavelet_HLH_glszm_ZonePercentage
wavelet_LLH_glszm_SmallAreaEmphasis
wavelet_HHH_firstorder_Median
wavelet_HLL_glszm_LargeAreaEmphasis
wavelet_LLH_glszm_ZoneVariance
wavelet_HHL_glszm_ZoneVariance
original_glcm_Imc2
wavelet_HLH_glszm_LargeAreaHighGrayLevelEmphasis
original_firstorder_10Percentile
wavelet_HLL_firstorder_Kurtosis
wavelet_LLH_glszm_LargeAreaHighGrayLevelEmphasis
17. Criterion: stable threshold is 10; Pval is 0.03; Qval is 0.07
Stable Features:
wavelet_HHL_glcm_Id
wavelet_HHH_glrlm_GrayLevelVariance
wavelet_LLH_glrlm_RunVariance
wavelet_HLL_glcm_DifferenceEntropy
wavelet_LLH_firstorder_Entropy
wavelet_LLH_glcm_Idmn
wavelet_HLH_glszm_ZonePercentage
wavelet_LLH_glszm_SmallAreaEmphasis
original_glcm_Imc2
wavelet_LHL_gldm_GrayLevelNonUniformity
original_firstorder_10Percentile
wavelet_HLL_firstorder_Kurtosis
Full Features:
wavelet_HHL_glcm_Id
wavelet_HHH_glrlm_GrayLevelVariance
wavelet_LLH_glrlm_RunVariance
wavelet_HLL_glcm_DifferenceEntropy
wavelet_LLL_glszm_LargeAreaHighGrayLevelEmphasis
original_glszm_LargeAreaHighGrayLevelEmphasis
wavelet_LLH_firstorder_Entropy
wavelet_LLH_glcm_Idmn
wavelet_LLL_glszm_LargeAreaEmphasis
wavelet_HLH_glszm_ZonePercentage
wavelet_LLH_glszm_SmallAreaEmphasis
wavelet_HHH_firstorder_Median
wavelet_HLL_glszm_LargeAreaEmphasis

wavelet_LLH_glszm_ZoneVariance
wavelet_HHL_glszm_ZoneVariance
original_glcm_Imc2
wavelet_HLH_glszm_LargeAreaHighGrayLevelEmphasis
original_firstorder_10Percentile
wavelet_HLL_firstorder_Kurtosis
wavelet_LLH_glszm_LargeAreaHighGrayLevelEmphasis

18. Criterion: stable threshold is 15; Pval is 0.03; Qval is 0.07

Stable Features:

wavelet_HHL_glcm_Id
wavelet_HHH_glrlm_GrayLevelVariance
wavelet_LLH_glrlm_RunVariance
wavelet_HLL_glcm_DifferenceEntropy
wavelet_LLH_firstorder_Entropy
wavelet_LLH_glcm_Idmn
wavelet_HLH_glszm_ZonePercentage
wavelet_LLH_glszm_SmallAreaEmphasis
original_glcm_Imc2
wavelet_LHL_gldm_GrayLevelNonUniformity
original_firstorder_10Percentile
wavelet_HLL_firstorder_Kurtosis

Full Features:

wavelet_HHL_glcm_Id
wavelet_HHH_glrlm_GrayLevelVariance
wavelet_LLH_glrlm_RunVariance
wavelet_HLL_glcm_DifferenceEntropy
wavelet_LLL_glszm_LargeAreaHighGrayLevelEmphasis
original_glszm_LargeAreaHighGrayLevelEmphasis
wavelet_LLH_firstorder_Entropy
wavelet_LLH_glcm_Idmn
wavelet_LLL_glszm_LargeAreaEmphasis
wavelet_HLH_glszm_ZonePercentage
wavelet_LLH_glszm_SmallAreaEmphasis
wavelet_HHH_firstorder_Median
wavelet_HLL_glszm_LargeAreaEmphasis
wavelet_LLH_glszm_ZoneVariance
wavelet_HHL_glszm_ZoneVariance
original_glcm_Imc2
wavelet_HLH_glszm_LargeAreaHighGrayLevelEmphasis
original_firstorder_10Percentile
wavelet_HLL_firstorder_Kurtosis
wavelet_LLH_glszm_LargeAreaHighGrayLevelEmphasis

19. Criterion: stable threshold is 20; Pval is 0.03; Qval is 0.07

Stable Features:

wavelet_HHL_glcm_Id
wavelet_HHH_glrlm_GrayLevelVariance
wavelet_LLH_glrlm_RunVariance
wavelet_HLL_glcm_DifferenceEntropy
wavelet_LLH_firstorder_Entropy
wavelet_LLH_glcm_Idmn
wavelet_LLL_glszm_LargeAreaEmphasis

wavelet_HLH_glszm_ZonePercentage
wavelet_LLH_glszm_SmallAreaEmphasis
wavelet_HLL_glszm_LargeAreaEmphasis
wavelet_LLH_glszm_ZoneVariance
wavelet_HHL_glszm_ZoneVariance
original_glcm_Imc2
original_firstorder_10Percentile
wavelet_HLL_firstorder_Kurtosis
Full Features:
wavelet_HHL_glcm_Id
wavelet_HHH_glrlm_GrayLevelVariance
wavelet_LLH_glrlm_RunVariance
wavelet_HLL_glcm_DifferenceEntropy
wavelet_LLL_glszm_LargeAreaHighGrayLevelEmphasis
original_glszm_LargeAreaHighGrayLevelEmphasis
wavelet_LLH_firstorder_Entropy
wavelet_LLH_glcm_Idmn
wavelet_LLL_glszm_LargeAreaEmphasis
wavelet_HLH_glszm_ZonePercentage
wavelet_LLH_glszm_SmallAreaEmphasis
wavelet_HHH_firstorder_Median
wavelet_HLL_glszm_LargeAreaEmphasis
wavelet_LLH_glszm_ZoneVariance
wavelet_HHL_glszm_ZoneVariance
original_glcm_Imc2
wavelet_HLH_glszm_LargeAreaHighGrayLevelEmphasis
original_firstorder_10Percentile
wavelet_HLL_firstorder_Kurtosis
wavelet_LLH_glszm_LargeAreaHighGrayLevelEmphasis
20. Criterion: stable threshold is 25; Pval is 0.03; Qval is 0.07
Stable Features:
wavelet_HHL_glcm_Id
wavelet_HHH_glrlm_GrayLevelVariance
wavelet_LLH_glrlm_RunVariance
wavelet_HLL_glcm_DifferenceEntropy
wavelet_LLL_glszm_LargeAreaHighGrayLevelEmphasis
original_glszm_LargeAreaHighGrayLevelEmphasis
wavelet_LLH_firstorder_Entropy
wavelet_LLH_glcm_Idmn
wavelet_LLL_glszm_LargeAreaEmphasis
wavelet_HLH_glszm_ZonePercentage
wavelet_LLH_glszm_SmallAreaEmphasis
wavelet_HLL_glszm_LargeAreaEmphasis
wavelet_LLH_glszm_ZoneVariance
wavelet_HHL_glszm_ZoneVariance
original_glcm_Imc2
original_firstorder_10Percentile
wavelet_HLL_firstorder_Kurtosis
wavelet_LLH_glszm_LargeAreaHighGrayLevelEmphasis
Full Features:
wavelet_HHL_glcm_Id

wavelet_HHH_glrlm_GrayLevelVariance
wavelet_LLH_glrlm_RunVariance
wavelet_HLL_glcm_DifferenceEntropy
wavelet_LLL_glszm_LargeAreaHighGrayLevelEmphasis
original_glszm_LargeAreaHighGrayLevelEmphasis
wavelet_LLH_firstorder_Entropy
wavelet_LLH_glcm_Idmn
wavelet_LLL_glszm_LargeAreaEmphasis
wavelet_HLH_glszm_ZonePercentage
wavelet_LLH_glszm_SmallAreaEmphasis
wavelet_HHH_firstorder_Median
wavelet_HLL_glszm_LargeAreaEmphasis
wavelet_LLH_glszm_ZoneVariance
wavelet_HHL_glszm_ZoneVariance
original_glcm_Imc2
wavelet_HLH_glszm_LargeAreaHighGrayLevelEmphasis
original_firstorder_10Percentile
wavelet_HLL_firstorder_Kurtosis
wavelet_LLH_glszm_LargeAreaHighGrayLevelEmphasis
21. Criterion: stable threshold is 5; Pval is 0.05; Qval is 0.09
Stable Features:
wavelet_HHL_glcm_Id
wavelet_HHH_gldm_DependenceEntropy
wavelet_HLL_glcm_DifferenceEntropy
wavelet_LLH_firstorder_RootMeanSquared
original_glrlm_LongRunEmphasis
wavelet_LLH_glcm_Idmn
wavelet_LLH_glszm_SmallAreaEmphasis
original_glcm_Imc2
wavelet_HLH_gldm_DependenceNonUniformityNormalized
wavelet_HLL_glcm_Idn
wavelet_LLL_firstorder_Entropy
Full Features:
wavelet_HHL_glcm_Id
wavelet_HHH_glrlm_GrayLevelVariance
wavelet_LLH_glrlm_RunVariance
wavelet_HLL_glcm_DifferenceEntropy
wavelet_LLL_glszm_LargeAreaHighGrayLevelEmphasis
wavelet_LLL_firstorder_Skewness
original_glszm_LargeAreaHighGrayLevelEmphasis
wavelet_LLH_firstorder_RootMeanSquared
wavelet_LLH_glcm_Idmn
wavelet_LLL_glszm_LargeAreaEmphasis
wavelet_HLH_glszm_ZonePercentage
wavelet_HLH_glszm_SizeZoneNonUniformityNormalized
wavelet_LLH_glszm_SmallAreaEmphasis
wavelet_HLH_glcm_JointAverage
wavelet_HHL_glcm_ClusterProminence
wavelet_HHL_ngtdm_Contrast
wavelet_HHH_firstorder_Median
wavelet_HLL_glszm_LargeAreaEmphasis

wavelet_HLL_ngtdm_Contrast
wavelet_LLH_glszm_ZoneVariance
original_firstorder_MeanAbsoluteDeviation
wavelet_HHL_glszm_ZoneVariance
original_glcm_Imc2
original_firstorder_Maximum
wavelet_HLH_glszm_LargeAreaHighGrayLevelEmphasis
wavelet_HLL_firstorder_Kurtosis
wavelet_LLH_glszm_LargeAreaHighGrayLevelEmphasis

22. Criterion: stable threshold is 10; Pval is 0.05; Qval is 0.09

Stable Features:

wavelet_HHL_glcm_Id
wavelet_HHH_glrlm_GrayLevelVariance
wavelet_LLH_glrlm_RunVariance
wavelet_HLL_glcm_DifferenceEntropy
wavelet_LLH_firstorder_RootMeanSquared
wavelet_LLH_glcm_Idmn
wavelet_HLH_glszm_ZonePercentage
wavelet_HLH_glszm_SizeZoneNonUniformityNormalized
wavelet_LLH_glszm_SmallAreaEmphasis
original_firstorder_MeanAbsoluteDeviation
wavelet_LLL_gldm_GrayLevelNonUniformity
original_glcm_Imc2
wavelet_LHL_gldm_GrayLevelNonUniformity
wavelet_HLL_glcm_Idn

Full Features:

wavelet_HHL_glcm_Id
wavelet_HHH_glrlm_GrayLevelVariance
wavelet_LLH_glrlm_RunVariance
wavelet_HLL_glcm_DifferenceEntropy
wavelet_LLL_glszm_LargeAreaHighGrayLevelEmphasis
wavelet_LLL_firstorder_Skewness
original_glszm_LargeAreaHighGrayLevelEmphasis
wavelet_LLH_firstorder_RootMeanSquared
wavelet_LLH_glcm_Idmn
wavelet_LLL_glszm_LargeAreaEmphasis
wavelet_HLH_glszm_ZonePercentage
wavelet_HLH_glszm_SizeZoneNonUniformityNormalized
wavelet_LLH_glszm_SmallAreaEmphasis
wavelet_HLH_glcm_JointAverage
wavelet_HHL_glcm_ClusterProminence
wavelet_HHL_ngtdm_Contrast
wavelet_HHH_firstorder_Median
wavelet_HLL_glszm_LargeAreaEmphasis
wavelet_HLL_ngtdm_Contrast
wavelet_LLH_glszm_ZoneVariance
original_firstorder_MeanAbsoluteDeviation
wavelet_HHL_glszm_ZoneVariance
original_glcm_Imc2
original_firstorder_Maximum
wavelet_HLH_glszm_LargeAreaHighGrayLevelEmphasis

wavelet_HLL_firstorder_Kurtosis
wavelet_LLH_glszm_LargeAreaHighGrayLevelEmphasis
23. Criterion: stable threshold is 15; Pval is 0.05; Qval is 0.09
Stable Features:
wavelet_HHL_glcm_Id
wavelet_HHH_glrlm_GrayLevelVariance
wavelet_LLH_glrlm_RunVariance
wavelet_HLL_glcm_DifferenceEntropy
wavelet_LLH_firstorder_RootMeanSquared
wavelet_LLH_glcm_Idmn
wavelet_HLH_glszm_ZonePercentage
wavelet_HLH_glszm_SizeZoneNonUniformityNormalized
wavelet_LLH_glszm_SmallAreaEmphasis
wavelet_HLH_glcm_JointAverage
wavelet_HHL_ngtdm_Contrast
wavelet_HLL_ngtdm_Contrast
original_firstorder_MeanAbsoluteDeviation
wavelet_LLL_gldm_GrayLevelNonUniformity
original_glcm_Imc2
wavelet_LHL_gldm_GrayLevelNonUniformity
wavelet_HLL_firstorder_Kurtosis
Full Features:
wavelet_HHL_glcm_Id
wavelet_HHH_glrlm_GrayLevelVariance
wavelet_LLH_glrlm_RunVariance
wavelet_HLL_glcm_DifferenceEntropy
wavelet_LLL_glszm_LargeAreaHighGrayLevelEmphasis
wavelet_LLL_firstorder_Skewness
original_glszm_LargeAreaHighGrayLevelEmphasis
wavelet_LLH_firstorder_RootMeanSquared
wavelet_LLH_glcm_Idmn
wavelet_LLL_glszm_LargeAreaEmphasis
wavelet_HLH_glszm_ZonePercentage
wavelet_HLH_glszm_SizeZoneNonUniformityNormalized
wavelet_LLH_glszm_SmallAreaEmphasis
wavelet_HLH_glcm_JointAverage
wavelet_HHL_glcm_ClusterProminence
wavelet_HHL_ngtdm_Contrast
wavelet_HHH_firstorder_Median
wavelet_HLL_glszm_LargeAreaEmphasis
wavelet_HLL_ngtdm_Contrast
wavelet_LLH_glszm_ZoneVariance
original_firstorder_MeanAbsoluteDeviation
wavelet_HHL_glszm_ZoneVariance
original_glcm_Imc2
original_firstorder_Maximum
wavelet_HLH_glszm_LargeAreaHighGrayLevelEmphasis
wavelet_HLL_firstorder_Kurtosis
wavelet_LLH_glszm_LargeAreaHighGrayLevelEmphasis
24. Criterion: stable threshold is 20; Pval is 0.05; Qval is 0.09

Stable Features:
wavelet_HHL_glcm_Id
wavelet_HHH_glrlm_GrayLevelVariance
wavelet_LLH_glrlm_RunVariance
wavelet_HLL_glcm_DifferenceEntropy
wavelet_LLH_firstorder_RootMeanSquared
wavelet_LLH_glcm_Idmn
wavelet_LLL_glszm_LargeAreaEmphasis
wavelet_HLH_glszm_ZonePercentage
wavelet_HLH_glszm_SizeZoneNonUniformityNormalized
wavelet_LLH_glszm_SmallAreaEmphasis
wavelet_HLH_glcm_JointAverage
wavelet_HHL_glcm_ClusterProminence
wavelet_HHL_ngtdm_Contrast
wavelet_HLL_glszm_LargeAreaEmphasis
wavelet_HLL_ngtdm_Contrast
wavelet_LLH_glszm_ZoneVariance
original_firstorder_MeanAbsoluteDeviation
wavelet_HHL_glszm_ZoneVariance
original_glcm_Imc2
wavelet_HLL_firstorder_Kurtosis
Full Features:
wavelet_HHL_glcm_Id
wavelet_HHH_glrlm_GrayLevelVariance
wavelet_LLH_glrlm_RunVariance
wavelet_HLL_glcm_DifferenceEntropy
wavelet_LLL_glszm_LargeAreaHighGrayLevelEmphasis
wavelet_LLL_firstorder_Skewness
original_glszm_LargeAreaHighGrayLevelEmphasis
wavelet_LLH_firstorder_RootMeanSquared
wavelet_LLH_glcm_Idmn
wavelet_LLL_glszm_LargeAreaEmphasis
wavelet_HLH_glszm_ZonePercentage
wavelet_HLH_glszm_SizeZoneNonUniformityNormalized
wavelet_LLH_glszm_SmallAreaEmphasis
wavelet_HLH_glcm_JointAverage
wavelet_HHL_glcm_ClusterProminence
wavelet_HHL_ngtdm_Contrast
wavelet_HHH_firstorder_Median
wavelet_HLL_glszm_LargeAreaEmphasis
wavelet_HLL_ngtdm_Contrast
wavelet_LLH_glszm_ZoneVariance
original_firstorder_MeanAbsoluteDeviation
wavelet_HHL_glszm_ZoneVariance
original_glcm_Imc2
original_firstorder_Maximum
wavelet_HLH_glszm_LargeAreaHighGrayLevelEmphasis
wavelet_HLL_firstorder_Kurtosis
wavelet_LLH_glszm_LargeAreaHighGrayLevelEmphasis
25. Criterion: stable threshold is 25; Pval is 0.05; Qval is 0.09

Stable Features:
wavelet_HHL_glcm_Id
wavelet_HHH_glrlm_GrayLevelVariance
wavelet_LLH_glrlm_RunVariance
wavelet_HLL_glcm_DifferenceEntropy
wavelet_LLL_glszm_LargeAreaHighGrayLevelEmphasis
wavelet_LLL_firstorder_Skewness
original_glszm_LargeAreaHighGrayLevelEmphasis
wavelet_LLH_firstorder_RootMeanSquared
wavelet_LLH_glcm_Idmn
wavelet_LLL_glszm_LargeAreaEmphasis
wavelet_HLH_glszm_ZonePercentage
wavelet_HLH_glszm_SizeZoneNonUniformityNormalized
wavelet_LLH_glszm_SmallAreaEmphasis
wavelet_HLH_glcm_JointAverage
wavelet_HHL_glcm_ClusterProminence
wavelet_HHL_ngtdm_Contrast
wavelet_HLL_glszm_LargeAreaEmphasis
wavelet_HLL_ngtdm_Contrast
wavelet_LLH_glszm_ZoneVariance
original_firstorder_MeanAbsoluteDeviation
wavelet_HHL_glszm_ZoneVariance
original_glcm_Imc2
wavelet_HLL_firstorder_Kurtosis
wavelet_LLH_glszm_LargeAreaHighGrayLevelEmphasis
Full Features:
wavelet_HHL_glcm_Id
wavelet_HHH_glrlm_GrayLevelVariance
wavelet_LLH_glrlm_RunVariance
wavelet_HLL_glcm_DifferenceEntropy
wavelet_LLL_glszm_LargeAreaHighGrayLevelEmphasis
wavelet_LLL_firstorder_Skewness
original_glszm_LargeAreaHighGrayLevelEmphasis
wavelet_LLH_firstorder_RootMeanSquared
wavelet_LLH_glcm_Idmn
wavelet_LLL_glszm_LargeAreaEmphasis
wavelet_HLH_glszm_ZonePercentage
wavelet_HLH_glszm_SizeZoneNonUniformityNormalized
wavelet_LLH_glszm_SmallAreaEmphasis
wavelet_HLH_glcm_JointAverage
wavelet_HHL_glcm_ClusterProminence
wavelet_HHL_ngtdm_Contrast
wavelet_HHH_firstorder_Median
wavelet_HLL_glszm_LargeAreaEmphasis
wavelet_HLL_ngtdm_Contrast
wavelet_LLH_glszm_ZoneVariance
original_firstorder_MeanAbsoluteDeviation
wavelet_HHL_glszm_ZoneVariance
original_glcm_Imc2
original_firstorder_Maximum
wavelet_HLH_glszm_LargeAreaHighGrayLevelEmphasis

wavelet_HLL_firstorder_Kurtosis
wavelet_LLH_glszm_LargeAreaHighGrayLevelEmphasis
